# NF-κB drives acquired resistance to a novel mutant-selective EGFR inhibitor

**DOI:** 10.18632/oncotarget.3956

**Published:** 2015-04-29

**Authors:** Elena Galvani, Jing Sun, Leticia G. Leon, Rocco Sciarrillo, Ravi S. Narayan, Robert Tjin Tham Sjin, Kwangho Lee, Kadoaki Ohashi, Daniëlle A.M. Heideman, Roberta R. Alfieri, Guus J. Heynen, René Bernards, Egbert F. Smit, William Pao, Godefridus J. Peters, Elisa Giovannetti

**Affiliations:** ^1^ Department Medical Oncology, VU University Medical Center, Amsterdam, The Netherlands; ^2^ Division of Hematology/Oncology, Department of Medicine, Vanderbilt University School of Medicine and Vanderbilt-Ingram Cancer Center, Nashville, TN, USA; ^3^ Instituto de Tecnologias Biomedicas, Center for Biomedical Research of the Canary Islands, University of La Laguna, Tenerife, Spain; ^4^ Department Hematology, VU University Medical Center, Amsterdam, The Netherlands; ^5^ Department Radiation Oncology, VU University Medical Center, Amsterdam, The Netherlands; ^6^ Celgene Avilomics Research, Bedford, MA, USA; ^7^ Department of Pathology, VU University Medical Center, Amsterdam, The Netherlands; ^8^ Department of Clinical and Experimental Medicine, University of Parma, Parma, Italy; ^9^ Division of Molecular Carcinogenesis, The Netherlands Cancer Institute, Amsterdam, The Netherlands; ^10^ Department of Pulmonary Diseases, VU University Medical Center, Amsterdam, The Netherlands; ^11^ Cancer Pharmacology Lab, AIRC Start-Up Unit, DIPINT, University of Pisa, Pisa, Italy

**Keywords:** drug-resistance, EGFR-T790M, NSCLC, NF-κB, EMT

## Abstract

The clinical efficacy of EGFR tyrosine kinase inhibitors (TKIs) in non-small cell lung cancer (NSCLC) harbouring activating *EGFR* mutations is limited by the emergence of acquired resistance, mostly ascribed to the secondary EGFR-T790M mutation. Selective EGFR-T790M inhibitors have been proposed as a new, extremely relevant therapeutic approach. Here, we demonstrate that the novel irreversible EGFR-TKI CNX-2006, a structural analog of CO-1686, currently tested in a phase-1/2 trial, is active against *in vitro* and *in vivo* NSCLC models expressing mutant EGFR, with minimal effect on the wild-type receptor. By integration of genetic and functional analyses in isogenic cell pairs we provide evidence of the crucial role played by NF-κB1 in driving CNX-2006 acquired resistance and show that NF-κB activation may replace the oncogenic EGFR signaling in NSCLC when effective and persistent inhibition of the target is achieved in the presence of the T790M mutation. In this context, we demonstrate that the sole, either genetic or pharmacologic, inhibition of NF-κB is sufficient to reduce the viability of cells that adapted to EGFR-TKIs. Overall, our findings support the rational inhibition of members of the NF-κB pathway as a promising therapeutic option for patients who progress after treatment with novel mutant-selective EGFR-TKIs.

## INTRODUCTION

Activating mutations of the epidermal growth factor receptor (EGFR) have been correlated with clinical activity of the small molecule inhibitors erlotinib, gefitinib and afatinib in non–small cell lung cancer (NSCLC) patients [1-3]. Despite initial efficacy, the success of EGFR tyrosine kinase inhibitors (TKIs) has been limited by the emergence of drug resistance, often ascribed to additional alterations within the target oncogene [4, 5].

The T790M mutation, which occurs within EGFR catalytic pocket, plays the most important role among the resistance mechanisms, arising in ∼60% of the cases [3, 6, 7]. Tumors harboring this mutation retain their dependency on EGFR signaling for growth, thus prompting the development of novel inhibitors of the receptor [8]. Second-generation TKIs, such as afatinib (BIBW2992) and dacomitinib (PF00299804), have shown antitumor activity against T790M-NSCLC. However, the lack of selectivity upon mutant EGFR as compared to the wild-type (WT) receptor, and evidence of acquired resistance to second-generation TKIs driven by the same T790M mutation, emphasized the need to develop more potent and specific agents against this mutation [9-11]. Selective EGFR-T790M inhibitors, including CO-1686 [12] and AZD9291 [13], have been proposed as alternative salvage therapies and are currently undergoing clinical development in patients who developed the T790M mutation (NCT01526928, NCT01802632).

In this study, we demonstrated the *in vitro* and *in vivo* selectivity and efficacy of the novel irreversible EGFR-TKI CNX-2006, a structural analog of CO-1686, in preclinical NSCLC models harboring activating mutations and the T790M. A comparable activity was observed *in vitro* for CO-1686. Furthermore, we developed isogenic pairs of CNX-2006-sensitive and -resistant cancer cells to address the mechanisms of resistance that may emerge upon constant and selective inhibition of the EGFR-T790M oncogene. By integrating genetic and functional studies we demonstrated the key role of NF-κB1 in driving adaptive resistance to CNX-2006 both through overexpression and constitutive activation. Finally, we showed that the inhibition of members of the NF-κB pathway effectively reduced CNX-2006-resistant cells proliferation and survival, thus supporting innovative therapeutic strategies for patients who progress after treatment with novel mutant-selective EGFR-TKIs.

## RESULTS

### CNX-2006 selectively inhibits mutant EGFR *in vitro*

**Figure 1:**
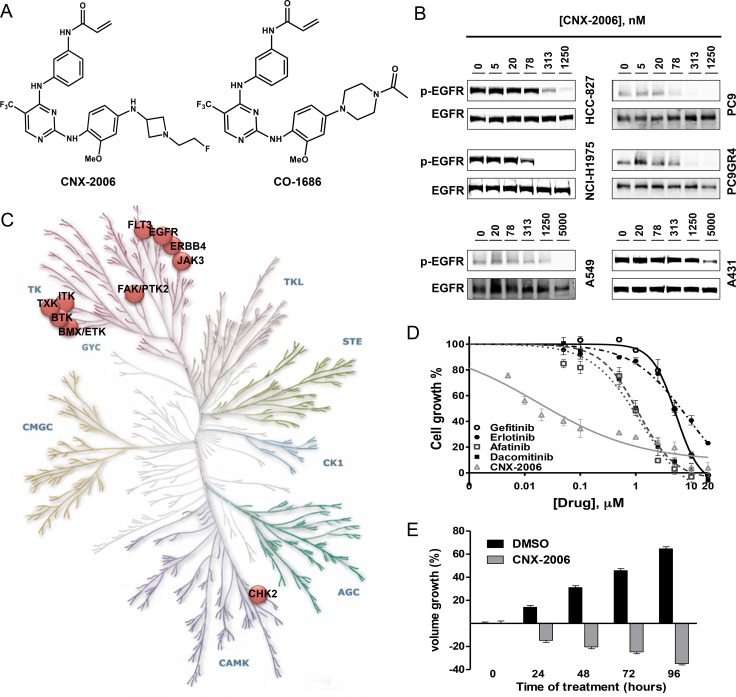
*In vitro* activity of CNX-2006 **A.** Molecular structure of CNX-2006 and CO-1686; **B.** EGFR phosphorylation inhibition evaluated after 2 hours treatment with 0.1% DMSO or the indicated concentrations of CNX-2006; **C.** kinase inhibition profile of 1 μM CNX-2006 in the presence of 100 μM ATP. The dots indicate enzymes that were inhibited >50% by the inhibitor relative to DMSO. Adjusted from www.cellsignal.com/reference/kinase/index.html; **D.** anti-proliferative effect of erlotinib (●), gefitinib (○), afatinib (■), dacomitinib (□) and CNX-2006 (Δ) in PC9DR1 cells. Data plotted as mean ± SEM; **E.** effect of 0.1% DMSO or 1 μM CNX-2006 in NCI-H1975-derived tumor spheres. The bar graph shows the mean ± SEM of the percentage of spheroids volume growth normalized to the volume at the time 0 treatment.

CNX-2006 is structurally related to CO-1686 (Figure 1A), and acts as a potent mutant-selective EGFR inhibitor through covalent interaction with the highly conserved Cys797 residue in the kinase domain of EGFR (Supplementary Figure 1A-1C).

The efficacy of CNX-2006 against cells expressing WT or mutant EGFR was evaluated in surrogate kinase assays and tumor cell lines. Similar to erlotinib and afatinib, CNX-2006 readily inhibited EGFR phosphorylation in 293H cells harbouring either the exon 19 delE746-A750 or the L858R variant (Supplementary Figure 2A). In NSCLC cells expressing the above mentioned activating mutations (PC9 and HCC-827 cells), CNX-2006 concentrations ranging between 55 and 104 nM were sufficient to reduce to 50% (IC_50_) the phosphorylation of EGFR after 2 hours treatment (Figure 1B). In cells expressing either EGFR-T790M alone or the T790M mutation in *cis* with activating mutations, CNX-2006 effectively inhibited the phosphorylation of the receptor at low nanomolar concentrations while no effect was observed after treatment with erlotinib (Figure 1B and Supplementary Figure 2A). Particularly, IC_50_s of about 46 and 61 nM were obtained after 2 hours treatment with CNX-2006 in the NSCLC cell lines NCI-H1975 and PC9GR4, respectively (Figure 1B). Importantly, while both erlotinib and afatinib inhibited the activity of the WT-receptor at low nanomolar concentrations, CNX-2006 affected the WT-EGFR only at concentrations which are over 10-fold higher than the ones necessary to inhibit mutated receptor (Figure 1B and Supplementary Figure 2A).

The efficacy of CNX-2006 was also tested against rare EGFR mutations, including EGFR-G719S, -ex19ins (I744-K745insKIPVAI), -L861Q, -ex20ins (H773-V774HVdup), and -T854A. CNX-2006 was as active as erlotinib against the former three variants of the receptor. Partial sensitivity to CNX-2006 was also observed in EGFR-T854A cells, while no effect was detected in cells transfected with the ex20ins variant of the receptor (Supplementary Figure 2B).

The selectivity of the inhibitor on the target was tested in a panel of 62 recombinant protein kinases using the radiometric assay HotSpot [14]. Eleven kinases, including EGFR-L858R/T790M and WT-EGFR, showed inhibition >50% after treatment with 1 μM CNX-2006 (Figure 1C and Supplementary Table 1). The most effective inhibition, about 95.96%, was observed against mutant EGFR, and high levels of inhibition were also observed for EGFR-sequence-related kinases. The only exception to this cluster was the cell cycle checkpoint Chk2, member of the calcium and calmodulin-regulated kinases. When tested in NCI-H1975 cells, CNX-2006 showed a strong profile of inhibition of EGFR downstream signaling pathways relative to DMSO treated cells. One μM CNX-2006 reduced the phosphorylation of several kinase substrates in a peptides based array, including different members of the MAPK, PI3K, Src and CDK families (Supplementary Table 2 and 3). In the same conditions, no evidence of inhibition of either EGFR or downstream signaling pathway was achieved by 1 μM gefitinib in NCI-H1975 (Supplementary Table 2).

### CNX-2006 inhibits mutant-EGFR cell proliferation by inducing apoptosis *in vitro*

In a panel of 23 NSCLC cell lines, the range of variation in sensitivity to CNX-2006, expressed as 50% growth inhibition concentration (GI_50_), was between 3 and 8000 nM (Table 1). Despite CO-1686 and CNX-2006 displayed comparable activity in cells expressing mutated EGFR, a higher selectivity was observed with the latter compound due to its more limited activity in cells harbouring the wild-type receptor (Table 1). Importantly, CNX-2006 was up to 290-fold more active than gefitinib and erlotinib, and 55-fold more effective than second-generation EGFR-TKIs, in PC9DR1 cells carrying a focal amplification of EGFR-T790M (Figure 1D) [9]. Interestingly, resistance to gefitinib driven by *MET* amplification resulted in resistance to both CNX-2006 and CO-1686, with over 1000-fold drop in drug activity in HCC-827GR5 cells compared to parental cells [15]. The exceptional activity of the inhibitor in EGFR-T790M cells was further confirmed in three-dimensional tumor spheroids derived from NCI-H1975 cells. After 96 hours treatment with 1 μM CNX-2006, the initial spheroids volume was reduced of about 40%, suggesting the ability of the inhibitor to penetrate multicellular structures (Figure 1E). Furthermore, increase of cellular fragments in Sub-G1 phase, chromatin condensation and nuclear fragmentation were observed after treatment with the EGFR-TKI (Supplementary Figure 3A). Moreover, reduction of the mitochondrial membrane potential, as measured by DiOC6 staining, and loss of membrane integrity by AnnexinV, showed that 24 hours treatment with CNX-2006 effectively induced ∼16% apoptosis in NCI-H1975 cells (Supplementary Figure 3B-3C).

**Table 1:** Anti-proliferative effect of gefitinib, CO-1686 or CNX-2006 in NSCLC cells

**Cell line**	**EGFR** **status**	**KRas** **status**	**Gefitinib** **GI_50_ (μM)**	**CO-1686** **GI_50_ (μM)**	**CNX-2006** **GI_50_ (μM)**
**A549**	WT	Mut (G12S)	8.0±0.2	1.2±0.2	2.7±0.1
**Calu-1**	WT	Mut (G12C)	19.0±0.8	2.0±0.3	8.0±0.3
**Calu-6**	WT	Mut (Q61K)	15.3±0.3	nd	3.2±0.3
**NCI-H1299**	WT	WT	7.9±0.6	4.1±0.2	4.5±0.5
**NCI-H1703**	WT	WT	8.2±0.4	nd	4.3±0.3
**NCI-H23**	WT	Mut (G12C)	11.2±0.7	nd	2.4±0.3
**NCI-H292**	WT	WT	0.10±0.02	nd	0.34±0.04
**NCI-H460**	WT	Mut (Q61H)	13.0±0.8	2.3±0.2	5.0±0.6
**NCI-H520**	WT	WT	9.2±0.5	1.6±0.1	5.2±0.4
**NCI-H522**	WT	WT	13.7±0.6	0.6±0.1	1.9±0.4
**NCI-H596**	WT	WT	15.1±0.7	2.0±0.1	4.2±0.3
**SKMES-1**	WT	WT	8.1±0.4	nd	1.4±0.1
**SW1573**	WT	Mut (G12C)	5.2±0.2	1.1±0.1	1.9±0.2
**NCI-H3255**	L858R	WT	0.020±0.004	0.005±0.002	0.006±0.002
**HCC-827**	delE746-A750	WT	0.011±0.003	0.002±0.001	0.003±0.002
**PC9**	delE746-A750	WT	0.040±0.004	0.004±0.002	0.009±0.001
**HCC-827GR5** **(Engelman et al., 2007)**	delE746-A750 (*MET ampl*)	WT	8.7±0.7	1.2±0.2	3.6±0.3
**NCI-H1975**	L858R/T790M	WT	10.0±0.9	0.170±0.009	0.072±0.002
**PC9GR4** **(Ercan et al., 2010)**	delE746-A750/T790M	WT	2.1±0.2	0.029±0.001	0.008±0.002
**PC9DR1** **(Ercan et al., 2010)**	delE746-A750/T790M (*ampl*)	WT	5.3±0.8	0.059±0.004	0.018±0.003
**Cell line**	**EGFR** **status**	**KRas** **status**	**Erlotinib** **GI_50_ (μM)**	**CO-1686** **GI_50_ (μM)**	**CNX-2006** **GI_50_ (μM)**
**PC9/ER** **(Ohashi et al., 2012)**	delE746-A750/T790M	WT	>5.0	0.10±0.03	0.04±0.01
**HCC-827/R1** **(Ohashi et al., 2012)**	delE746-A750/T790M	WT	>5.0	nd	0.092±0.007
**H3255/XLR** **(Ohashi et al., 2012)**	L858R/T790M	WT	>5.0	0.049±0.001	0.020±0.001

**WT, wild-type; Mut, mutant; del, deletion; ampl, amplification; nd, not determined**

### CNX-2006 inhibits EGFR-T790M tumor growth *in vivo*

**Figure 2:**
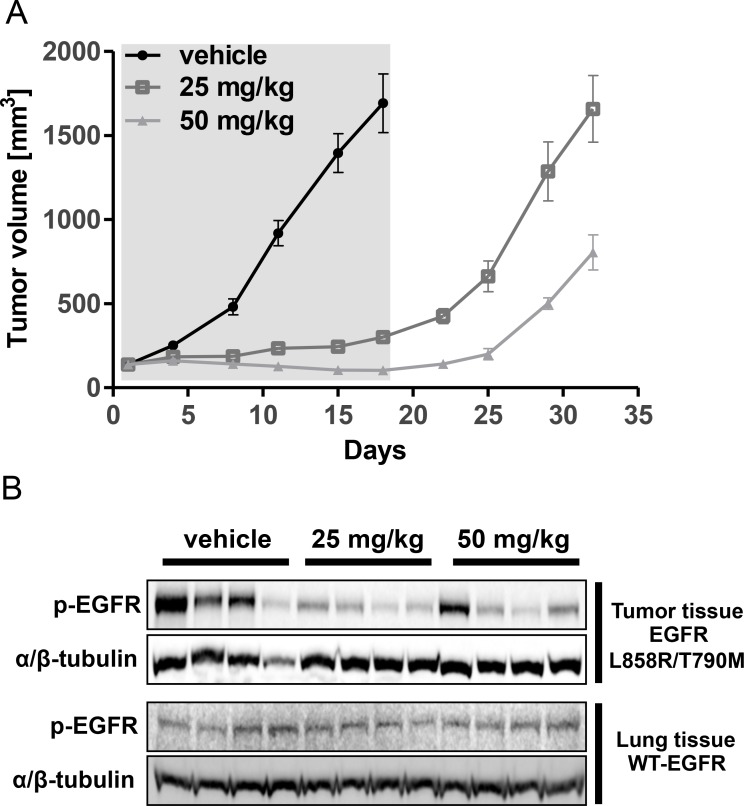
*In vivo* activity of CNX-2006 **A.** Tumor volume growth in xenografts during (day 0-17, grey background) and after (day 18-32) IP administration of vehicle or CNX-2006 at 25 or 50 mg/kg; **B.** inhibition of EGFR phosphorylation 1 hour after IP administration of vehicle or CNX-2006 at 25 or 50 mg/kg as determined by immunoblot. Lysates were obtained from tumor and normal lung tissue of 4 mice per group.

Consistent with its *in vitro* anti-proliferative effect in cells carrying EGFR-T790M, CNX-2006 daily dosed at either 25 or 50 mg/kg inhibited the growth of subcutaneous NCI-H1975-derived tumors (Figure 2A). In the groups treated with the inhibitor, tumor growth was observed only after drug withdrawal (over day 18^th^ in Figure 2A). Reduction of target phosphorylation was observed 1 hour after the administration of both the doses of the drug in tumor tissue expressing mutant EGFR. Remarkably, as observed *in vitro*, CNX-2006 did not affect the activation of the wild-type receptor expressed in normal lung tissue (Figure 2B and Supplementary Figure 4A). Of note, CNX-2006 accumulation was observed 1 hour after the treatment in lungs, with concentrations up to ∼13 and ∼7-fold higher than in plasma and liver, respectively (Supplementary Figure 4B). A similar behavior has previously been reported for gefitinib that reached up to 10-fold higher concentrations in lung tissue as compared to plasma 2 hours after either intravenous or oral administration [16, 17]. This accumulation has been more recently associated with active uptake of the first-generation EGFR-TKI in NSCLC cells by various members of the solute carrier (SLC) superfamily of transporters [18, 19]. The tissue specific distribution of various SLCs having CNX-2006 as a substrate may explain its preferential accumulation in lungs as compared to liver or plasma.

### Chromosomal instability in CNX-2006-resistant cells affects cell differentiation and proliferation

**Figure 3:**
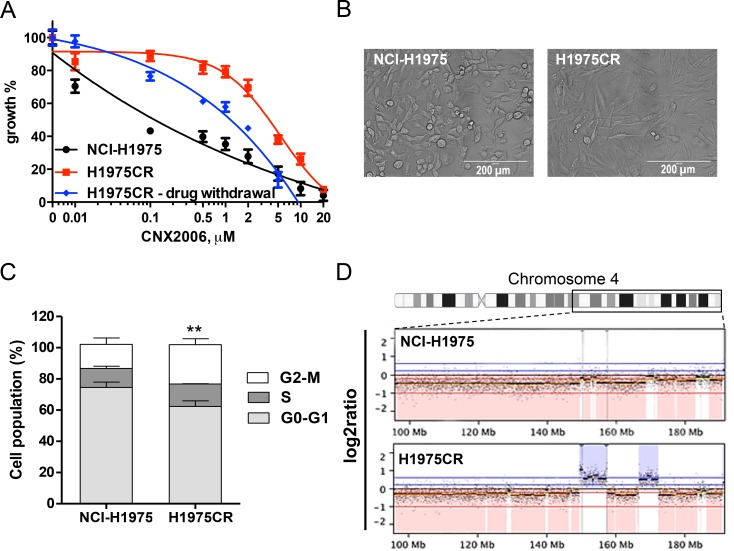
NCI-H1975-derived CNX-2006-resistant cells **A.** Anti-proliferative effect of 72 hours treatment with 0.01-20 μM CNX-2006 in NCI-H1975 and H1975CR cells continuously cultured in the presence of the drug or after 3 months of drug withdrawal. The mean ± SEM is plotted for all the tested concentrations; **B.** representative picture of H1975CR and NCI-H1975 cells in standard culture conditions; **C.** cell cycle distribution of NCI-H1975 and H1975CR cells 24 hours after plating. *P < 0.05 relative to G2-M phase in NCI-H1975 cells; **D.** differential copy number variation in chromosome 4 in parental and resistant cells as determined by array CGH. *Red*, loss; *blue*, gain *vs.* XX/XY control.

*In vitro* acquired resistance to CNX-2006 was achieved by applying escalating concentrations (up to 1 μM) in NCI-H1975 cells. After 8-10 months, we isolated a pool of CNX-2006-resistant (CR) cells which was over 60-fold less sensitive to the drug (GI_50_=4.6 μM) compared to the parental cell line (Figure 3A). Cross-resistance to the quinazoline-derivatives afatinib and dacomitinib as well as CO-1686 (GI_50_s>1 μM) was also observed (data not shown). The obtained resistant cells showed increased dimensions and acquired spindle-like morphology, a shorter doubling time and a different cell cycle distribution compared to parental cells. A significant increase (*P < 0.01*) in the number of cells in G2-M phase was observed in H1975CR compared to NCI-1975 cells (Figure 3B-3C). After over 3 months of drug withdrawal, H1975CR sensitivity to CNX-2006 was partially restored, with GI_50_ at 72 hours treatment of 940 nM (Figure 3A). EGFR-independent growth and proliferation were confirmed in H1975CR cells by transient EGFR knockdown (Supplementary Figure 5A-5B).

Genome-wide high-resolution analysis of DNA copy number variations was performed by array-CGH in H1975CR cells and compared to the parental cells. Chromosomal imbalances in the resistant cells mostly overlapped the NCI-H1975 cells (Supplementary Figure 6A), including common DNA aberrations in lung cancer, such as amplifications in locus 7p11.2, containing the *EGFR* gene, and gains at 5p15.33-p15.31, 1q21-q25, 8q24.21 and 11q13 loci [http://AtlasGeneticsOncology.org, 20]. Large deletions were observed in 9p24.1-p23 and 13q21 loci which were previously associated with lung adenocarcinomas [21]. Three large regions emerged in H1975CR cells that were differentially altered compared to the parental cells, i.e. amplification in chromosome 4, and deletions in chromosomes 2 and 8. Multiple genes involved in cell survival and motility regulation, such as *MYCN*, *NRBP1*, *EPCAM, PKP4, RHOB, SNAI2, ASPH, GDAP1* and *IL7*, were included in the large deletions in 2p24.3-p21 and 8q12.3-q13.3 loci (Supplementary Figure 6B). Similarly, the 4q31.2 locus has previously been related to lung cancer development (Figure 3D) [22]. Several genes important for cell differentiation and proliferation are included in this locus, such as the *Hedgehog-interacting protein*, *Glycophorin A*, *IL15*, *GRB2-associated binding protein-1 and SH3D19*. Overexpression of these genes in H1975CR cells was confirmed by RNA-seq (Supplementary Dataset 1).

### Expression of altered cell adhesion patterns reduced cell motility in H1975CR

**Figure 4:**
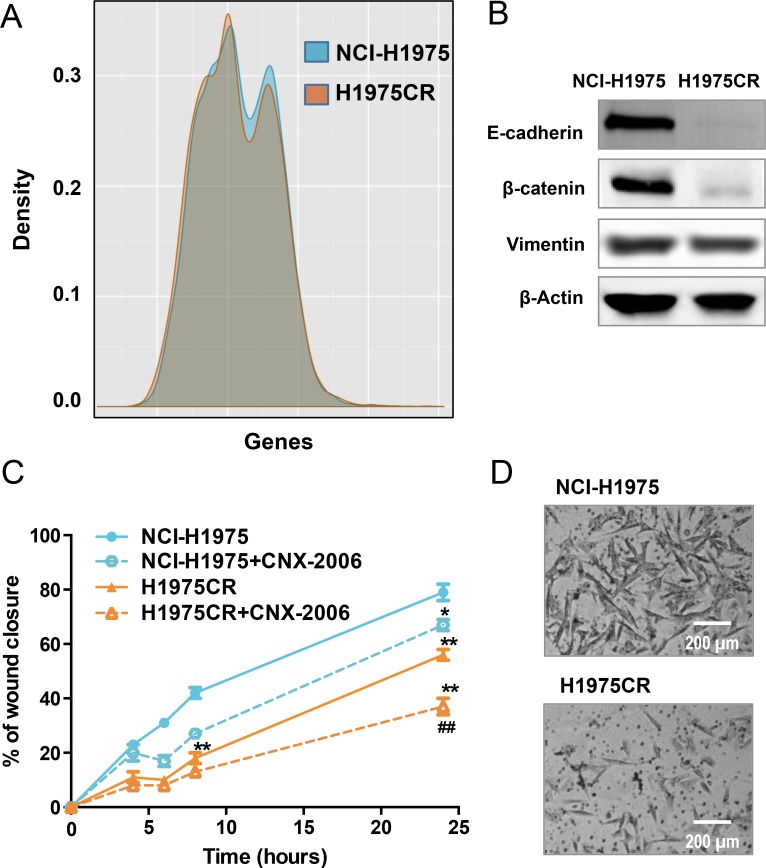
Migration and invasion **A.** Graphical representation of whole transcriptome comparing NCI-H1975 and H1975CR cells; **B.** E-cadherin, β-catenin and vimentin protein expression in NCI-H1975 vs H1975CR cells as detected by immunoblot. β-actin was included as a loading control; **C.** cell migration as measured by wound healing assay. The percentage of migration is relative to the treatment starting point (*t* = 0) with 0.1% DMSO or 1 μM CNX-2006 and is plotted as mean ± SEM of four replicates in 3 independent experiments. **P < 0.01 vs NCI-H1975 DMSO-treated cells, ^##^*P < 0.01* vs H1975CR DMSO-treated cells; **D.** representative images of cellular invasiveness through the matrigel in NCI-H1975 vs H1975CR cells.

Further comparison of NCI-H1975 and H1975CR cells transcriptomes showed similar expression patterns, with only few exceptions (Figure 4A). The analysis of RNA-seq using beta-binomial distribution [23] followed by KEGG pathway analysis (Kanehisa Laboratories; Kyoto University), and TopHat2 coupled Cufflinks analysis, demonstrated a differential expression of genes involved in cell-cell and cell-extracellular matrix (ECM) interaction. Specifically, decreased expression of *CDH1* (epithelial cadherin: E-cadherin) was observed in CNX-2006-resistant cells, further confirmed by immunoblot analysis (Figure 4B). A concomitant reduction of β-catenin was also observed in H1975CR cells (Figure 4B). Given the role of cell adhesion in cancer aggressiveness, we investigated the migratory and invasive behavior of H1975CR cells. Surprisingly, the H1975CR cells displayed a significantly reduced motility (*P < 0.01*) in a wound-healing assay compared to parental cells, as well as reduced invasiveness through the ECM (Figure 4C-4D). Both behaviors were further reduced by the treatment with 1 μM CNX-2006.

**Figure 5:**
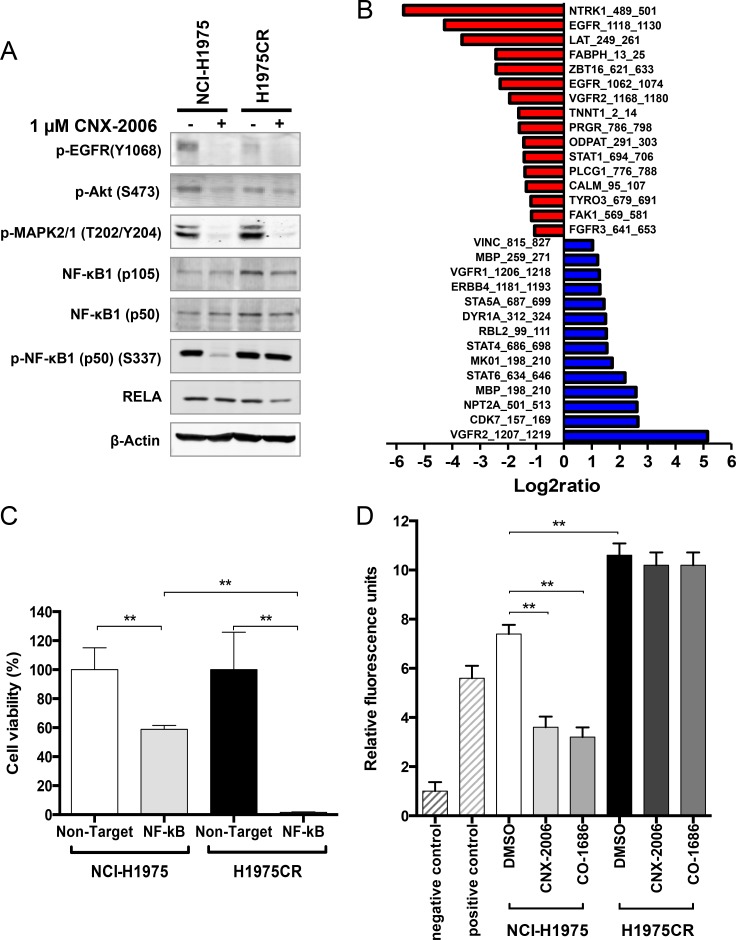
NF-κB-mediated resistance to CNX-2006 **A.** Modulation of key signaling proteins in NCI-H1975 and H1975CR cells after 2 hours treatment with 0.1% DMSO or 1 μM CNX-2006; **B.** phospho-peptides differentially modulated in H1975CR cells compared to NCI-H1975 cells. A fold-change >2 was chosen as cutoff. *Red*, reduction; *blue*, enhancement; **C.** effect of NF-κB silencing on cell viability in NCI-H1975 and H1975CR as assessed by cell count after DAPI-staining. Cell viability was determined 72 hours post-siRNA transfection and Non-Target siRNA transfected cells were used as control. Data plotted as mean ± SEM; **D.** NF-kB activity expressed in relative fluorescence units as compared to the negative control. Cells were treated with 0.1% DMSO, 1 μM CNX-2006 or 1 μM CO-1686 for 6 hours. Data plotted as mean ± SEM. ** *P < 0.01*.

To explore the mechanisms underlying these results, we evaluated the effect of 1 μM CNX-2006 on EGFR phosphorylation, as well as on the activation of its major effectors. After 2 hours treatment, EGFR phosphorylation was inhibited in both NCI-H1975 and H1975CR cells with concomitant reduction of Akt and ERK1/2 activation (Figure 5A). Despite their proliferation profile was unaltered following EGFR knockdown, CNX-resistant cells conserved a certain level of dependency on the oncogene signaling for cellular motility as demonstrated by the retained effect of the drug in this process.

### Overexpression and constitutive activation of NF-κB1 drive adaptive resistance to CNX-2006

Genetic characterization was performed for recurrent *EGFR* (exons 18-21), *KRAS* (exons 2-3), *BRAF* (exon 15) and *PIK3CA* (exons 9 and 20) mutations in H1975CR cells [24]. After CNX-2006 resistance development, EGFR-L858R/T790M was the only detected target alteration, and no additional mutations emerged in any of the other candidate genes. Furthermore, differently from previously reported results describing resistance to third generation EGFR-TKIs [25, 26], neither reduced IGFBP3 gene expression nor MAPK1 amplification was observed in CNX-2006-resistant cells by RNA-seq and aCGH.

The reduced expression of CDH1 observed in H1975CR cells, and previous results on the involvement of EMT in NSCLC resistance to EGFR-TKI treatment [12, 27, 28], led us to investigate the role of this process in resistance to CNX-2006. Gene expression analysis of the 76 genes included in the EMT signature developed by Byers and colleagues was performed [29]. A broad reduction in the expression of epithelial markers was observed in H1975CR cells compared to parental cells, while there was only slight or no increase in mesenchymal markers (Supplementary Figure 7A). Results were validated by qRT-PCR or immunoblot for various EMT markers, including CLDN4, CLDN7, EPCAM, ZEB1, FN1, AXL, MMP2 and vimentin (Figure 4B and Supplementary Figure 7B). A key role for EMT in resistance to CNX-2006 was excluded on the basis of these results.

Activated kinases were investigated in parental and H1975CR cells by high-throughput array to unravel diversely triggered pathways that may drive the resistance to CNX-2006. A pool of 30 differentially phosphorylated (>2-fold) peptides was identified in CNX-2006-resistant cells compared to parental cells (Figure 5B and Supplementary Table 2). The phosphorylation sites on the peptides were associated with 24 kinases, 13 of which were downregulated in H1975CR cells, including FGFR3, FAK1 and EGFR. Conversely, 11 kinases were upregulated in the resistant cells, including ERBB4, PKC, MAPK1, and JAK. About 65% reduction in EGFR autophosphorylation and ∼40% enhancement in MAPK1 (p42) activation was confirmed by immunoblot in H1975CR cells compared to NCI-H1975 cells (Figure 5A). Interestingly, in line with recent results showing the involvement of NF-κB signaling in modulating lung cancer dependence upon EGFR oncogenic signaling [30], 17 of the modulated kinases could directly or indirectly be linked to NF-κB pathway. Immunoblot analysis revealed ∼30% overexpression of NF-κB1 (p50) and ∼74% of its precursor (p105), but not RELA (p65), in CNX-2006-resistant compared to the parental cells (Figure 5A). In line with these results, about 3-fold increase in ATM phosphorylation was also found in resistant cells compared to NCI-H1975 (Supplementary Figure 8A). NF-κB1 knockdown inhibited H1975CR cells viability of ∼99%, while ∼60% of the NCI-H1975 cells survived after down-regulation of the transcription factor (Figure 5C and Supplementary Figure 8B). Data on its activation further supported NF-κB1 as key player in CNX-2006-resistance development. The treatment with 1 μM CNX-2006 did not affect the phosphorylation of the transcription factor in H1975CR cells, while its complete inhibition was achieved under the same conditions in parental cells (Figure 5A). Basal activity of NF-κB as monitored by fluorescence was 1.45-fold higher in H1975CR than in parental cells (Figure 5D). Furthermore, the treatment with either 1 μM CNX-2006 or CO-1686 caused ∼50% inhibition of NF-κB in NCI-H1975, while no effect was observed in the resistant cells under the same conditions.

### Pharmacological inhibition of NF-κB pathway reduces cell viability in CNX-2006-resistant cells

**Figure 6:**
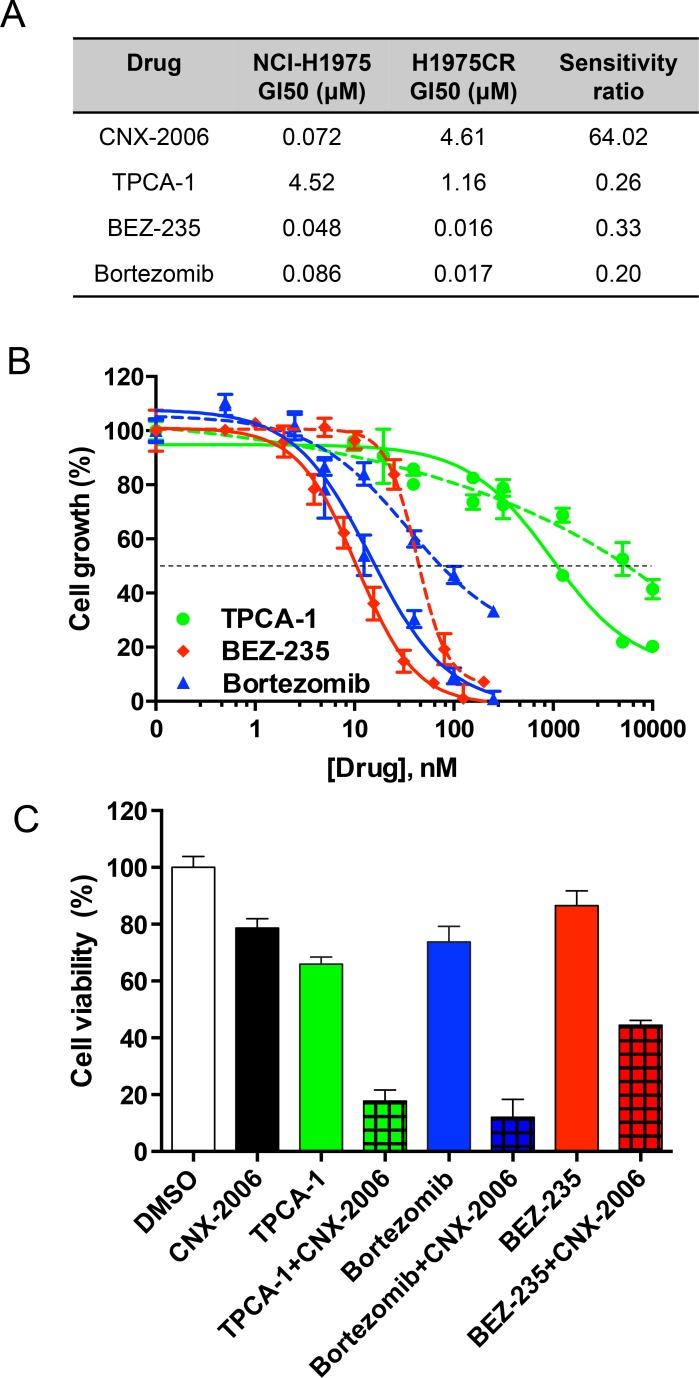
Targeting NF-κB pathway **A.** Growth Inhibition 50 (GI_50_s) of different drugs in NCI-H1975 and H1975CR cells after 72 hours treatment. The ratio of H1975CR *vs* NCI-H1975 cells GI_50_s is indicated for each drug; **B.** anti-proliferative effect of the tested inhibitors affecting the NF-kB pathway at different levels in H1975CR (continuous lines) and NCI-H1975 cells (dashed lines). The mean ± SEM is plotted for each drug at the tested concentrations. The dashed black line indicates the GI_50_; **C.** antiproliferative effect of 72 hours treatment with the indicated drugs at their GI_25_, either as single agent or in combination with CNX-2006 in H1975CR cells. Data plotted as mean ± SEM. ** *P < 0.01*.

The canonical activation of NF-κB relies on phosphorylation-induced degradation of IκB family members mediated by the IκB kinase (IKK) complex and consequent nuclear translocation of NF-κB dimers [31]. Thus, we tested the effect of agents targeting the NF-κB pathway at different levels. These drugs displayed a greater efficacy in reducing cell viability of NF-κB-dependent H1975CR cells than parental cells (Figure 6A-6B). Particularly, the small molecule TPCA-1 reduced both IKKβ and IkBα phosphorylation of ∼70% in resistant cells (Supplementary Figure 9A-9B) and had 4-fold increased anti-proliferative effect in H1975CR compared to NCI-H1975 cells. Prolonged inhibition of the proteasomes by bortezomib also altered the pathway by causing IkBα accumulation in H1975CR cells (Supplementary Figure 9C). The inhibitor effectively reduced cell viability in both resistant and parental cells, with GI_50_ ∼16.8 and 85.8 nM, respectively (Figure 6A-6B). Furthermore, the PI3K/mTOR inhibitor BEZ-235 was also tested as it has been shown to interfere with the ‘atypical’ NF-κB activation pathway, which includes ATM, ATR and DNA-PK [32, 33]. The compound reduced ATM phosphorylation by ∼20% in CNX-2006-resistant cells where it showed over 3-fold increased anti-proliferative effect over NCI-H1975 parental cells (Figure 6A and Supplementary Figure 9D). Despite the greater activity in CNX-2006 resistant cells compared to parental cells, single agent TPCA-1 was unable to completely inhibit the pathway, as shown by the residual phosphorylation of both IKKβ and IkBα (Supplementary Figure 9A-9B). To achieve a greater inhibition of the pro-survival signaling cascade, the NF-κB pathway inhibitors TPCA-1, bortezomib or BEZ-235, were combined with CNX-2006 at their respective GI_25_. After 72 hours treatment, ∼82%, ∼88% and ∼55% inhibition of H1975CR cell viability was observed for TPCA-1, bortezomib or BEZ-235, in combination with CNX-2006, respectively (Figure 6C).

## DISCUSSION

In this study we evaluated the mechanisms of resistance that may emerge by targeting EGFR-T790M in NSCLC and identified NF-κB as key player in adaptive and reversible resistance to the novel mutant selective EGFR inhibitor CNX-2006. For the first time, we demonstrate that NF-κB pathway activation may replace the oncogene signaling in lung cancer when effective and persistent inhibition of EGFR is achieved in the presence of the T790M mutation. In this context, we show that the sole inhibition of NF-κB activity is sufficient to reduce the viability of cells that developed resistance to third-generation EGFR-TKI by becoming independent from the signaling of the receptor.

Recent preclinical studies reported promising results in terms of greater efficacy and selectivity against EGFR-T790M by either reversible indolocarbazole-based or irreversible pyrimidine-based TKIs, such as WZ4002, AZD9291 and CO-1686 [12, 13, 34, 35]. Our results demonstrate that CNX-2006 abrogates mutant-EGFR activity, both *in vitro* and *in vivo*. Conversely, the weak inhibition of WT-EGFR suggests a more limited toxicity of CNX-2006 compared to first- and second-generation TKIs [36, 37]. CNX-2006 also showed good stability and favorable pharmacokinetics, and potently inhibited the growth of tumor spheres and subcutaneous xenografts.

Given the clinical challenge represented by the emergence of drug resistance against novel TKIs, acquired resistance to CNX-2006 was investigated as an important scenario to develop and test alternative treatment strategies. To address this aim we developed isogenic pairs of drug-sensitive and -resistant cancer cells, a strategy which has already been successfully applied to model EGFR-TKIs resistance in NSCLC [15, 27, 28, 38, 39].

In contrast to recent findings reporting the acquisition of additional EGFR mutations driving the resistance to WZ4002 [26, 40], we did not identify any additional EGFR mutation that may explain the secondary resistance to CNX-2006. Similarly, no evidence of promoter-methylation-driven repression of IGFBP3 expression and consequent activation of the IGF1R pathway, a mechanism previously associated with WZ4002-resistance in PC9 cells [25], was observed in H1975CR. Despite H1975CR displayed increased MAPK1 phosphorylation compared to NCI-H1975 cells, neither genomic amplification nor enhanced expression of MAPK1 was observed in CNX-2006-resistant cells, all of which have been described to drive WZ4002 resistance in PC9GR cells [26]. Furthermore, unlike previous findings in H1975 WZR cells [26], MAPK1 phosphorylation was completely abrogated by CNX-2006 treatment in H1975CR cells and no effect on cell viability was observed after treatment with the MEK1/2 inhibitor MEK162 (data not shown).

Our studies on CNX-2006-resistance demonstrated RNA-based enrichment of deregulated cell-cell and cell-ECM adhesion patterns. In particular, E-cadherin and β-catenin degradation after cell junctions disruption has been previously associated with tumor dedifferentiation, invasiveness and metastases formation [41]. Furthermore, a relevant role for EMT in resistance to EGFR and PI3K/Akt inhibitors has recently been demonstrated in NSCLC cell lines and patient samples, leading to the development of a 76-gene signature defining the transformation process [29]. A similar signature has been observed to drive acquired resistance to CO-1686 in both NCI-H1975 and HCC-827 cells [12]. However, while confirming a predictive role of altered E-cadherin/β-catenin expression in response to EGFR-TKIs in NSCLC [42], our results showed a reduction in the invasive behavior of CNX-2006-resistant cells. A possible explanation for this discrepancy is the down-regulation of FAK1 activity observed in H1975CR cells, which is critical for growth factor and integrin-induced cell migration [43]. Interestingly, H1975CR cells motility was further inhibited by CNX-2006 treatment, thus supporting the use of the inhibitor as maintenance treatment after the emergence of drug-resistance, as reported previously for other EGFR-TKIs [44].

In the last decade, a critical role for NF-κB pathway in lung cancer has been described in chemical-induced lung tumor models [45] as well as in models harboring commonly mutated genes such as *KRAS* and Trp53 [46]. Although the EGF-EGFR axis is known to activate NF-κB signalling pathway through different mediators [47, 48], erlotinib treatment did not affect NF-κB activation in a variety of cancer cells, thus highlighting the complexity of a pathway for which constitutive activation has been associated to treatment resistance in different cancer types [49-51]. Recently, Bivona and colleagues provided evidence of the involvement of NF-κB in modulating lung cancer dependence upon EGFR oncogenic signaling and consequently in resistance to erlotinib [30]. The study showed how inhibition of NF-κB signaling enhances erlotinib-induced apoptosis in lung cancer cells harboring activating mutations of EGFR. While the study by Bivona *et al.* limited the relevance of the pathway to non-T790M cells, we observed a lower expression of NF-κB1 in PC9 as compared to PC9GR4 cells, where resistance to gefitinib is driven by the acquisition of the T790M mutation (data not shown).

In the present study, we demonstrate that persistent NF-κB activation can intervene at different anti-EGFR treatment stages and drive drug resistance in NSCLCs carrying the T790M mutation. Despite its hyperactivation in H1975CR cells, NF-κB signaling remains tightly regulated by IKK and IκB. Indeed, we showed for the first time that the sole inhibition of carefully selected members of the NF-κB pathway, including IKKβ, is sufficient to successfully antagonize tumor growth and overcame resistance to CNX-2006. However, a possible explanation for NF-κB activation in H1975CR cells may reside in a cytokine-mediated upregulation of IKK family members. Several cytokines were differentially modulated in CNX-2006-resistant compared to NCI-H1975 cells at gene expression level, including some members of the TNF family as well as interleukins which have previously been shown to promote IKK activation (Supplementary Dataset 1).

In conclusion, this study demonstrates the excellent efficacy of the novel mutant selective EGFR inhibitor CNX-2006 in several representative preclinical models of NSCLC. Furthermore, we provide evidence of the key role of NF-κB signaling in driving lung cancer resistance when effective and persistent inhibition of EGFR is achieved in the presence of the T790M mutation. Whether NF-κB upregulation existed prior to CNX-2006 treatment in a subset of NCI-H1975 cells that expanded under the pressure of drug selection, or emerged as adaptive response to compensate the inhibition of EGFR is still under investigation. However, regardless bortezomib limited efficacy in unselected NSCLC patients [52], we demonstrated that proteasome inhibition might represent a valid therapeutic strategy to bypass NF-κB-mediated EGFR-TKI resistance in selected patients. Furthermore, here we demonstrated that NF-κB inhibition is also a valid strategy to enhance the effect of third-generation EGFR-TKIs in NSCLC expressing EGFR-T790M. Similarly, previous studies reported a role for NF-κB inhibition in sensitizing cells to erlotinib-induced cell death [26, 44]. This strategy is currently evaluated in a phase 1/2 clinical trial by combining erlotinib and quinacrine in NSCLCs (NCT01839955). Our results should pave the way for molecular profiling on multiple levels in patients treated with mutant-selective EGFR inhibitors and support the use of novel treatment strategies to either overcome or delay the escape from oncogene addiction in EGFR-mutant NSCLC.

## MATERIALS AND METHODS

Note that additional materials and methods are included in the Supplemental Methods file.

### Drugs and chemicals

The synthesis of CNX-2006 is described in the Supplemental Methods. CO-1686 and gefitinib were a generous gift from Clovis Oncology (San Francisco, CA) and AstraZeneca (Macclesfield, UK), respectively. Additional drugs and chemicals are described in the Supplemental Methods.

### Cell culture

The human NSCLC cell lines A549, Calu-1, Calu-6, NCI-H460, NCI-H292, NCI-H1299, NCI-H1730, NCI-H23, NCI-H3255, NCI-H520, NCI-H522, NCI-H596, HCC-827, SKMES-1, SW1573 and NCI-H1975 and the human epidermoid carcinoma A431 cells were purchased from ATCC (Manassas, VA), and cultured as recommended. 293H cells were purchased from Invitrogen (Carlsbad, CA). PC9, PC9GR4, PC9DR1 and HCC-827 GR5 were a kind gift of Dr. Pasi A. Jänne, Harvard University, Boston, MA [13]. PC9/ER, HCC-827/R1 and H3255/XLR cells are isogenic erlotinib- or XL-647-resistant lines derived from PC9, HCC-827 and NCI-H3255 cells, respectively, as previously described [53]. Additional information on cell cultures procedures is reported in the Supplemental Methods.

### Transfections and siRNA

Transfections with mutant *EGFR* constructs were performed in 293H cells using pcDNA3.1(−) vectors and Lipofectamine 2000 (Invitrogen; Carlsbad, CA). Two μg DNA was used per sample as previously described [54]. *EGFR* mutations G719S, ex19ins, ex20ins, L861Q, T854A, T790M, and L858R/T790M were generated by site-directed mutagenesis using a WT-EGFR cDNA template as previously described [54].

Transfections with siRNA were performed on 1×10^5^ cells/well plated into 6-well plates using Lipofectamine 2000. For all procedures, standard protocols were used according to the manufacturers' manuals. Synthetic siRNA targeting either EGFR (ID#103549) or NF-kB1 (ID#107297) was purchased from Applied Biosystems/Ambion (Foster City, CA). Non-Target siRNA was used as control.

### Tumor spheres

Spheroids from NCI-H1975 cells of about 300 μm in diameter were generated and treated with 0.1% DMSO or CNX-2006 at the NCI-H1975 GI_50_. Measurement and analysis were performed as previously described [55].

### Generation of CNX-2006-resistant cells

NCI-H1975 were cultured with increasing concentrations of CNX-2006 in step-wise fashion until cells emerged that were growing in the presence of 1 μM CNX-2006. Cells were initially treated with a drug concentration at which 30% of the cells were growth inhibited or killed (GI_30_) and when cells resumed normal growth patterns, the drug concentration was increased.

### RNA-sequencing

Quantification and quality assessment for RNA extracted from NCI-H1975 and H1975CR cells were performed with a Bioanalyzer (Agilent, Santa Clara, CA). Sequencing libraries were constructed with a TruSeq mRNA Library Preparation Kit using poly-A-enriched RNA (Illumina, San Diego, CA). Captured libraries were sequenced on an Illumina HiSeq2000 platform with a single-end 50-base protocol. Sequences were aligned to the human genome (Hg19) with TopHat2 [56]. HTSeq was used to assess the number of uniquely assigned reads for each gene; expression values were then normalized to 10^7^ total reads and log_2_ transformed (results are provided as Supplementary Dataset 1). TopHat2 [http://tophat.cbcb.umd.edu/index.shtml] and Cufflinks [http://cufflinks.cbcb.umd.edu/] were used to compare the two-condition transcriptomes [56, 57]. The results were visualized by CummeRbund [http://compbio.mit.edu/cummeRbund/].

### Kinase inhibition profile & peptide substrate array

Kinase inhibition profile of CNX-2006 was performed by Reaction Biology Corporation (RBC, Malvern, PA). Differential kinase activity in NCI-H1975 and H1975CR cells was analyzed by a kinase peptide substrate array (PamChip®, PamGene International, ‘s-Hertogenbosch, The Netherlands) as previously described [55].

### Monitoring of NF-kB activation

The Cignal NF-kB Pathway Reporter Assay Kit (GFP) (Qiagen, Hilden, Germany) was used to assess NF-kB activity in NCI-H1975 and H1975CR according to manufacturer's specifications. A mixture of a constitutively expressing GFP construct was used as positive control to assess transfection efficiency. A negative control, in which GFP expression was controlled by a basal promoter, was used to identify pathway-specific effects and determine background reporter activity. Expression of the GFP reporter was monitored via FACS at 488 nm for excitation and 530/30 nm for emission.

### ELISA assay

Phosphorylation and expression levels of intracellular proteins were determined by ELISA assays according to manufacturers' protocols. The following kits were used: PathScan® Phospho-IKKβ (Ser177/181) Sandwich ELISA Kit #7080 (Cell Signaling Technology, Danvers, MA); Human Phospho-ATM (S1981) DuoSet IC (R&D Systems, Minneapolis, MN); phospho-IκBα * [pS32] #KHO0221 and total IκBα #KHO0221 (Invitrogen).

### Statistical analysis

All experiments were performed in triplicate and repeated at least twice. Data are expressed as mean values ± SEM and analyzed using the two-tailed Student's t-test or ANOVA followed by the Bonferroni's multiple comparison test by using Prism (GraphPad Software). RNA-seq data were analyzed by beta-binomial model as previously reported [23].

## SUPPLEMENTAL MATERIAL AND METHODS FIGURES AND TABLES




