# Supplementary material for: NF-κB drives acquired resistance to a novel mutant-selective EGFR inhibitor
Source: Oncotarget. 2015 Apr 29;6(40):42717–32. doi: 10.18632/oncotarget.3956 (PMC4767465; doi:10.18632/oncotarget.3956)
Supplement: Supplementary file 1 [file oncotarget-06-42717-s001.pdf]

NF-κB drives acquired resistance to a novel mutant-selective EGFR inhibitor

Supplemental Material and Methods

Synthesis of CNX-2006

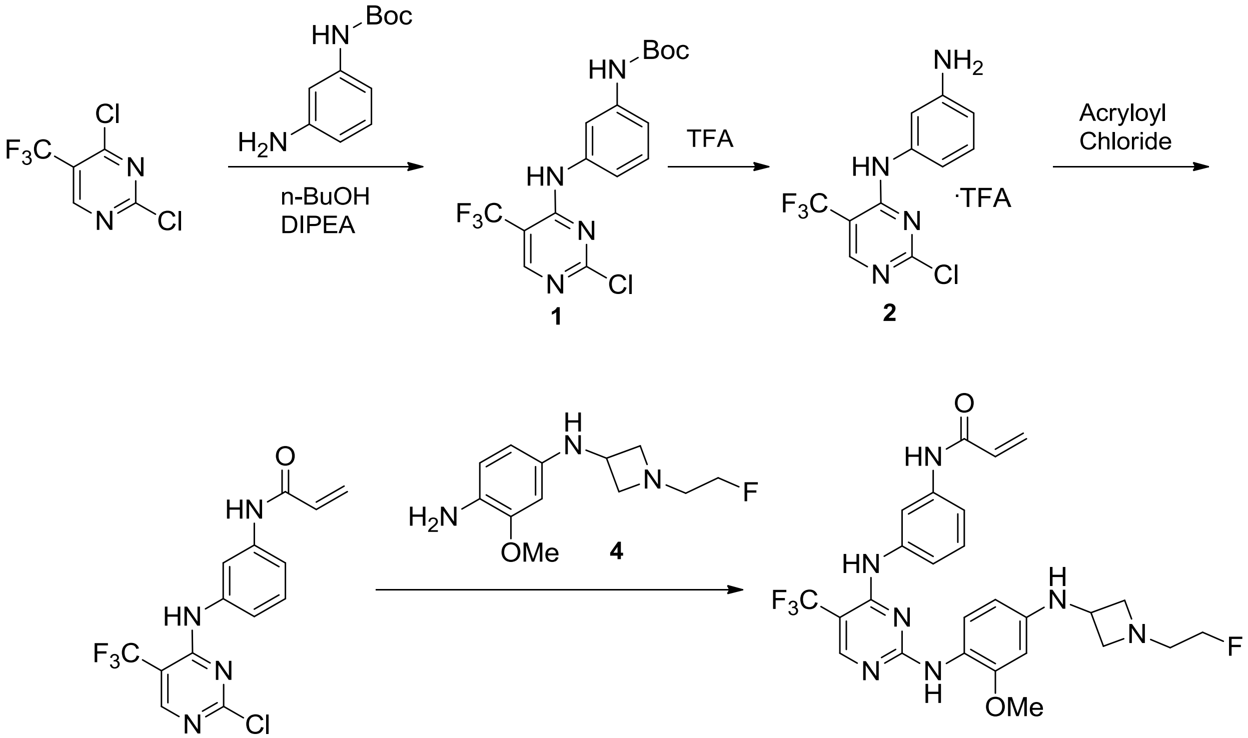

Compound 1: In a 25 mL 3-neck round bottom flask equipped with a magnetic stirrer and $CaCl_2$ drying tube, N-Boc-1,3-diaminobenzene (0.96 g) and 1-butanol (9.0 mL) were charged. The reaction mixture was cooled to 0 °C. 2,4-Dichloro-5-trifluoromethylpyrimidine (1.0 g) was added drop wise to the reaction mixture at 0 °C. The DIPEA (0.96 mL) was drop wise added to the above reaction mixture at 0 °C and the reaction mixture was stirred for 1 hr at 0 °C to 5 °C. The reaction mixture was then allowed to warm to room temperature. Reaction mixture was stirred for another 4 hrs at room temperature and was monitored by TLC using Hexane: Ethyl acetate (7: 3). The solid precipitated out was filtered off and washed with 1-butanol (2 mL). The solid was dried under reduced pressure at 40 °C for 1 hr to yield 0.7 g of compound 1. ^{1}H-

NMR (DMSO-d6, 400 MHz) δ 1.48 (S, 9 H), 7.02 (m, 1 H), 7.26 (m, 2 H), 7.58 (S, 1 H), 8.57 (S, 1 H), 9.48 (S, 1 H), 9.55 (S, 1 H). LC/MS: m/z 389.2 ($M+ H^+$).

Compound 2: To the above solid (0.7 g) in DCM (5 mL) was added TFA (2.5 mL) slowly at 0 °C. The reaction mixture was allowed to warm to room temperature. Reaction mixture was stirred for another 10 minutes at room temperature. The crude was concentrated under reduced pressure and used in next step (0.52 g).

Compound 3: The concentrated crude was dissolved in DCM (25 mL) and cooled to -30 °C. To the reaction mixture was slowly added acryloyl chloride (0.2 g) at -30 °C. The reaction was warmed to room temperature stirred at room temperature for 1.0 hr. The reaction was monitored on TLC using Hexane: Ethyl acetate (7:3) as mobile phase. Reaction was completed after 1 hr. The reaction was worked up and purified by flash column chromatography using Hexane and ethyl acetate (0.5 g). ^{1}H-NMR (DMSO-d6, 400 MHz) δ 5.76 (dd, J = 2.0, 10.0 Hz, 1 H), 6.24 (dd, J = 2.0, 17.2 Hz, 1 H), 6.48 (m, 1 H), 7.14 (d, J = 8.8 Hz, 1 H), 7.37 (t, J = 8.0 Hz, 1 H), 7.94 (S, 1 H), 8.59 (S, 1 H), 9.60 (S, 1 H), 10.26 (S, 1 H). LC/MS: m/z 343.2 ($M+ H^+$).

CNX-2006: Compound 3 (16 mg, 0.05 mmol) and compound 4 (20 mg, 0.08 mmol) were dissolved in dioxane (1.0 mL) with catalytic trifluoroacetic acid. The mixture was stirred overnight at 50 °C. The crude was concentrated under reduced pressure and purified using HPLC (TFA as modifier) to give CNX-2006 (10 mg) as a TFA salt. ^{1}H-NMR (DMSO-d6, 400 MHz) δ 10.13 (s, 1 H), 8.53 (br, 1 H), 8.24 (s, 1 H), 8.07 (s, 1 H), 7.74 (br, 1 H), 7.24 (m, 3 H), 6.45 (dd, J = 10.4, 17.2 Hz, 1 H), 6.26 (dd, J = 2.0, 17.2 Hz, 1 H), 6.18 (s, 1 H), 5.92 (br, 1 H), 5.76 (dd, J = 2.0, 10.4 Hz, 1 H), 4.48 (t, J = 4.8 Hz, 1 H), 4.36 (t, J = 4.8 Hz, 1 H), 3.93 (br, 1 H), 3.69 (s, 3 H), 2.6-2.9 (m, 4 H). LC/MS: m/z 546.2 ($M+ H^+$).

Drugs and chemicals

Erlotinib, afatinib, dacomitinib, TPCA-1, bortezomib, MEK162 and BEZ-235 were purchased from Selleck Chemicals (Munich, Germany). The drugs were dissolved in DMSO at the stock concentration of 10 mM and diluted in culture medium before use. RPMI-1640 medium (2mM L-glutamine), fetal bovine serum (FBS), penicillin (50 IU/ml) and streptomycin (50 µg/ml) were

from Gibco (Gaithersburg, MD). All other chemicals were purchased from Sigma-Aldrich (Zwijndrecht, The Netherlands).

Cell culture

Cells were maintained as monolayers in RPMI supplemented with 10% heat-inactivated FBS and 1% streptomycin and penicillin in a 37 ˚C water-saturated atmosphere of 5% CO2 in air in 75 cm2 tissue culture flasks (Costar, Cambridge, MA). HCC-827GR5 and PC9GR4 were cultured in the presence of 1 µM gefitinib. PC9/ER and HCC827/R1 were cultured in the presence of 1 µM erlotinib. H3255/XLR and PC9DR1 were cultured in the presence of 1 µM XL-647 or PF00299804, respectively. The cell lines were tested for their authentication by PCR profiling using short tandem repeats (STR), which was performed by BaseClear (Leiden, The Netherlands) in March 2013.

EGFR-L858R/T790M Mass Spectrometry

The EGFR-L858R/T790M protein was obtained from BPS Bioscience (San Diego, CA). Intact EGFR-L858R/T790M protein (5 µl of a 2.27 µM solution in phosphate buffered saline solution [pH 7.4]) was incubated at RT for 1 hour in a 10-fold excess of CNX-2006 (10 mM stock diluted 1:88 in 50% DMSO and then diluted again 1:5 in the protein solution for a final concentration of 22.7 µM) at 37 ˚C. At the end of the incubation, 5 µL aliquots of the samples were diluted with 15 µL of 0.2% TFA and then purified/concentrated with C4 ZipTips® and directly spotted onto a MALDI target plate using Sinapinic acid as the desorption matrix (10 mg/ml in 0.1% TFA in acetonitrile:water 50:50 v/v). Finally, each spot was analyzed on an ABSciex 4800 MALDI TOF-TOF mass spectrometer fitted with a HM2 detector (CovalX Saugus, MA).

Pepsin Digestion

CNX-2006 was incubated with EGFR-L858R/T790M protein as described in the MS experiment for 30 min. Sample was boiled for 5 min at 95 ˚C followed by pepsin (Princeton Separations, Freehold Township, NJ) digestion for 2 hours at RT. Digestion was terminated by freezing the sample. The peptide solution was transferred to HPLC-vial, where 1.3 µg of protein was injected into the Shimadzu AccuSpot via Agilent 1260 series capillary pumps. A gradient of 2% to 95%

organic over 10 min was used to separate the peptides off of a C18 column. The eluent was spotted every 15 seconds at a 10 µl/min total flow. A 10 mg/ml solution of alpha-Cyano-4-hydroxycinnamic acid was used as the desorption matrix. Data was collected and analyzed on an AB Sciex 4800 MALDI TOF-TOF. Sample was analyzed on an ABSciex 4800 MALDI TOF-TOF mass spectrometer fitted with a HM2 detector.

Immunoblot

Cells incubated with different concentrations of the inhibitors, were lysed by RIPA buffer supplemented with phosphatase and protease inhibitor cocktails. Procedures for protein extraction, solubilization, and protein analysis by 1-D PAGE are described elsewhere (Galvani et al., 2013b). Fifty µg of proteins were resolved by 8% SDS-PAGE and transferred to PVDF membranes. Fluorescent proteins were detected by an Odyssey Infrared Imager (LI-COR Biosciences, Lincoln, NE), at 84 µm resolution, 0 mm offset, using high quality settings.
The following antibodies from Bioké (Leiden, The Netherlands) were used: anti-phospho-EGFR (Y1068) (#3777), anti-EGFR (#2232S), anti-phospho-p44/42 (T202/Y204) (#4370), anti-phospho-Akt (S473) (#4060), anti-E-cadherin (#3195S), anti-α/β-tubulin (#2148) and anti-β-actin (#3700). Monoclonal anti-vimentin antibody (V6630) was purchased from Sigma-Aldrich, anti-β-catenin (ab32572) was from Abcam (Cambridge, UK), anti-phospho-EGFR (Y1173) (sc-12351), anti-NF-κB p50 (sc-8414), anti-phospho-NF-κB p50 (S337) (sc-33022) and anti-RELA (sc-372) were from Santa Cruz Biotechnology (Heidelberg, Germany). As secondary antibodies, goat-anti-mouse-InfraRedDye (#926-32210 and #926-32220, Westburg, Leusden, The Netherlands) or goat-anti-rabbit-InfraRedDye (#926-32211 and #926-32221) were used.

Inhibition of cell proliferation and cell cycle distribution

Absorbance: Cell growth inhibition for CNX-2006, TPCA-1, bortezomib and BEZ-235 was assessed by sulforhodamine B (SRB) assay according to the NCI protocol with slight modifications as previously described (Galvani et al., 2013b).

Fluorescence: Three days after treatment with either EGFR or NF-kB1 siRNA, NCI-H1975 and H1975CR cells were fixed with 3.7% formaldehyde and DNA was stained with DAPI diluted in

PBS (0.3 µg/ml). Cell number was assessed by counting the number of DAPI-stained cells using the Acumen Ex3 laser scanning cytometer (TTP LabTech, Royston, UK).

Distribution of the cells in the cell cycle was determined by propidium iodide (PI) staining and flow cytometry analysis (Galvani et al., 2013b).

Apoptosis

Fluorescence microscopy. 2x105 NCI-H1975 cells were treated with GI50 CNX-2006 for 24 hours and visualized by fluorescence microscopy (UV-2A filter; excitation, 300-380 nm; dichroic mirror, 400 nm; absorption, 420 nm; LeicaDMI300B, Wetzlar, Germany) after staining with 50 µg/ml Hoechst 33342 and 50 µg/ml PI.

Mitochondrial membrane potential. NCI-H1975 cells treated as described above, were incubated with 40 nM DiOC6 and analyzed as described (Galvani et al., 2013b).

AnnexinV/PI staining. Performed according to the manufacturer`s protocol (AnnexinV-FITC/PI apoptosis detection Kit I, Becton Dickinson, San José, CA), with minor modifications and analyzed as described (Galvani et al., 2013b).

Xenograft studies

All the procedures related to animal handling, care, and the treatment in this manuscript were performed according to the guidelines approved by Institutional Animal Care and Use Committees (IACUC) following the guidance of the Association for Assessment and Accreditation of Laboratory Animal Care (AAALAC). NCI-H1975 xenograft studies were performed by Charles River Laboratories (Wilmington, MA). Briefly, NCr nu/nu mice were sub-cutaneously implanted with $1x10^7$ tumor cells in 50% Matrigel (injection volume of 0.2 mL/mouse). Once tumors reached 100-150 mm^3, animals were dosed with vehicle (5% DMSO:15% Solutol HS15 in PBS) or the outlined doses of CNX-2006 (N=10 animals/group). Animals were dosed daily (qd) for 17 days via IP injection and tumor size was measured twice per week. Tumor growth was monitored over time to determine tumor growth inhibition of the

experimental agent vs. vehicle. The endpoint of the experiment was a mean tumor volume (MTV) in control group of 2000 mm^3. Percent TGI was defined as the difference between the MTV of the designated control group and the MTV of the drug-treated group, expressed as a percentage of the MTV of the designated control group. Data is presented as mean ± standard error of the mean (SEM).

Pharmacodynamics

Frozen tumor tissues, which were removed at 1 hour post-last dose, were homogenized using a CryoPrep™ system (Covaris, Inc; Woburn, MA), lysed in Lysis buffer with protease inhibitors and total protein concentration determined as described above. Seventy-five µg proteins were loaded into NuPAGE® Novex® 4-12% Bis-Tris Midi Gels and immuniblot was performed as described above. Signal intensities were analyzed via the Odyssey Fc imaging system and Image Studio software (LI-COR; Lincoln, NE) and plotted as tubulin-normalized percent phosphorylation relative to vehicle using Prism (GraphPad Software Inc., San Diego, CA) Statistical significance was determined using an unpaired Student's t-test.

Wound healing assay

LeicaDMI300B migration station (Leica Microsystems) integrated with the Scratch Assay 6.1 software was used to compare cell migration of NCI-H1975 and H1975CR treated with 0.1% DMSO or 1µM CNX-2006 as previously described (Galvani et al., 2013b).

Invasion assay

Transwell chambers coated with MatrigelTM with 6.5 mm diameter polycarbonate filters (8µm pore size, BD Biosciences) were used. One hundred thousand NCI-H1975 or H1975CR cells were loaded in the upper chamber in serum free RPMI medium with 0.1% DMSO or 1µM CNX-2006 for 24 hours. Ten% FBS was used as chemoattractant in the lower chamber. Non-invasive cells were removed with a cotton swab while the remaining cells were fixed with 100% methanol and stained with Giemsa.

Comparative Genomic Hybridization (array-CGH) and mutation analyses

Genomic DNA from NCI-H1975 and H1975CR was extracted using Ambion®-RecoverAll kit (Life Technologies, Breda, The Netherlands). The quantity and purity of extracted DNA were assessed at 260-280nm with the NanoDrop®-1000-Detector (NanoDrop-Technologies, Wilmington, DE). DNA labeling was carried out using Enzo's CGH Labeling Kit for Oligo Arrays (Enzo Life Sciences, Farmingdale, NY). DNA from blood of eighteen healthy males or females (XY-mix and XX-mix) was used as a reference. DNA was hybridized using Agilent SurePrint G3 Human CGH Microarray Kit for 4x180K platform (Agilent Technologies, Santa Clara, CA). The slides were scanned on Agilent Microarray Scanner, followed by data extraction and normalization by Feature Extraction v10.5 software (Agilent Technologies). Nexus Copy Number™ software was used to analyse the DNA copy number variations (BioDiscovery, Hawthorne, CA).

EGFR (exons 18 to 21), KRAS (exons 2 and 3), BRAF (exon 15) and PIK3CA (exons 9 and 20) mutational status was determined by high resolution melting (HRM) followed by Sanger sequencing as previously described (Heideman et al., 2012).

Quantitative real-time RT-PCR.

Total RNA was isolated using the TRI REAGENT LS (Invitrogen, Carlsbad, CA). One μg RNA was retro-transcribed using the DyNAmo cDNA Synthesis Kit (Thermo Scientific, Vantaa, Finland), according to the manufacturers' instructions. Primers and probes to specifically amplify CLDN4 (Hs00976831_s1), CLDN7 (Hs00600772_m1), EPCAM (Hs00901885_m1), ZEB1 (Hs00232783_m1), FN1 (Hs00365052_m1), AXL (Hs01064444_m1), MMP2 (Hs01548727_m1), E-cadherin (Hs01023894_m1), NF-kB1 (Hs00765730_m1) and EGFR (Hs01076078_m1) were obtained from Applied Biosystems Assay-on-Demand Gene expression products. The quantitative real-time PCR was performed in a 25 µl reaction volume containing TaqMan Universal master mix (Applied Biosystems). All reactions were performed in triplicate using the ABI-PRISM 7500 sequence detection system instrument (Applied Biosystems). Amplifications were normalized to beta-actin (TaqMan® B-actin Detection Reagents part number 401846). The fold change was calculated by the ΔΔCT method and results were plotted as $2^{-\Delta\Delta CT}$.

A

B

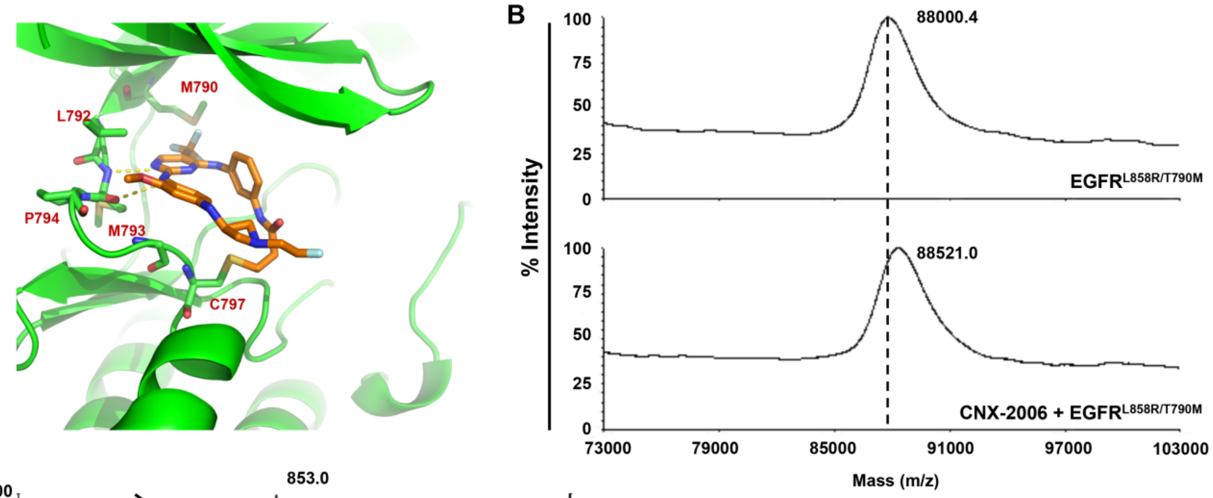

C

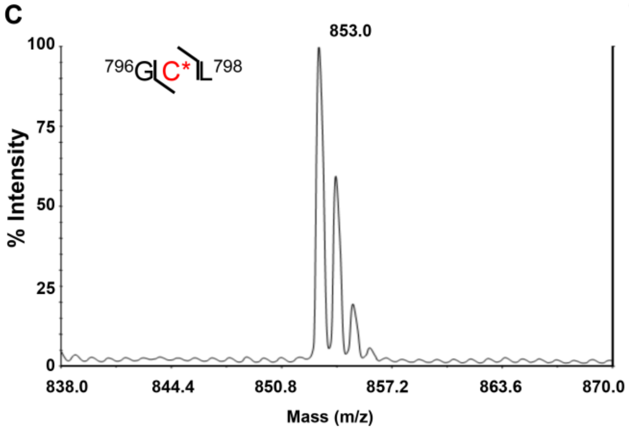

Supplementary Figure 1. (A) Structural modeling of CNX-2006 binding to EGFR-T790M. The receptor is shown in a ribbon representation (green) with the bound CNX-2006 in orange; **(B)** Top panel: mass spectrum of the intact EGFR L858R/T790M protein (m/z 88000.4 Da). Bottom panel: mass spectrum of EGFR L858R/T790M protein following 1 hour incubation with CNX-2006 (MW=545.54). The positive shift of 521 Da of the centroid mass indicates the complete stoichiometric modification of EGFR L858R/T790M protein by CNX-2006; **(C)** Pepsin digest analysis of CNX-2006 and EGFR L858R/T790M by MS/MS confirming a single CNX-2006 modification on Cys797 (oxidized peptide m/z=853).

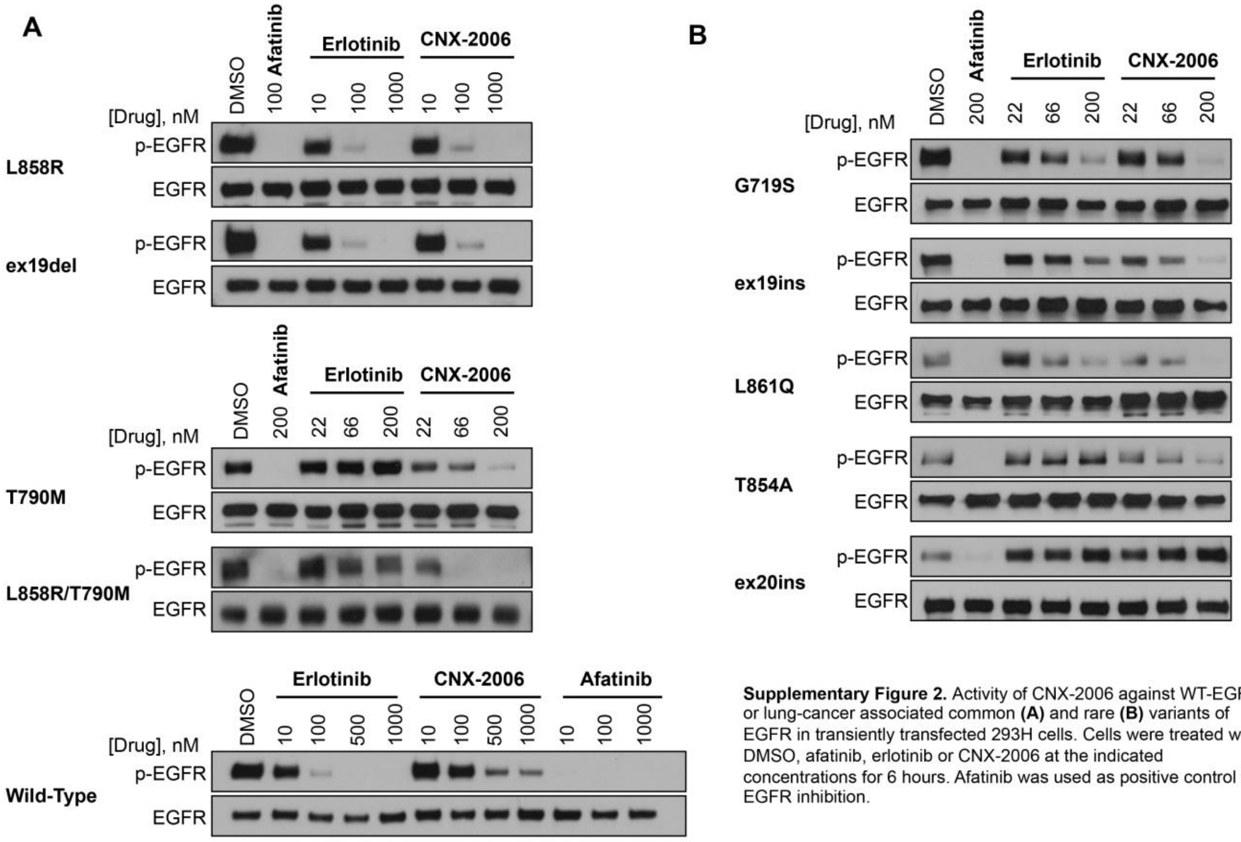

Supplementary Figure 2. Activity of CNX-2006 against WT-EGFR or lung-cancer associated common **(A)** and rare **(B)** variants of EGFR in transiently transfected 293H cells. Cells were treated with DMSO, afatinib, erlotinib or CNX-2006 at the indicated concentrations for 6 hours. Afatinib was used as positive control for EGFR inhibition.

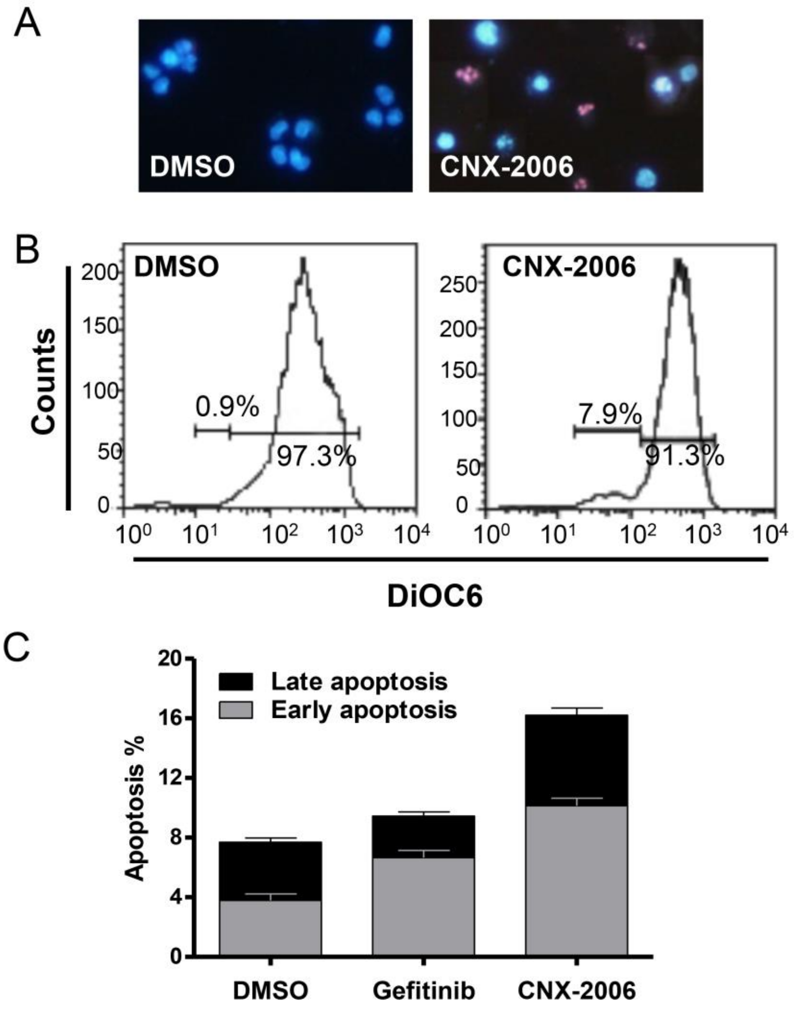

Supplementary Figure 3. (A) Chromatin condensation and nuclear fragmentation as detected by Hoechst-PI staining using fluorescence microscopy following treatment with either 0.1% DMSO or 1 μM CNX-2006 for 24 hours in NCI-H1975 cells; **(B)** mitochondrial membrane potential as measured by DiOC6-lebeling using flow cytometry after treatment with either 0.1% DMSO or 1 μM CNX-2006 for 24 hours in NCI-H1975 cells. Results are plotted as staining intensity *vs.* number of cells **(C)** Annexin V–FITC and PI staining analyzed by flow cytometry in NCI-H1975 cells treated for 24 hours with 0.1% DMSO, 1 μM gefitinib, or 1 μM CNX-2006. *Columns*, average; *bars*, SD.

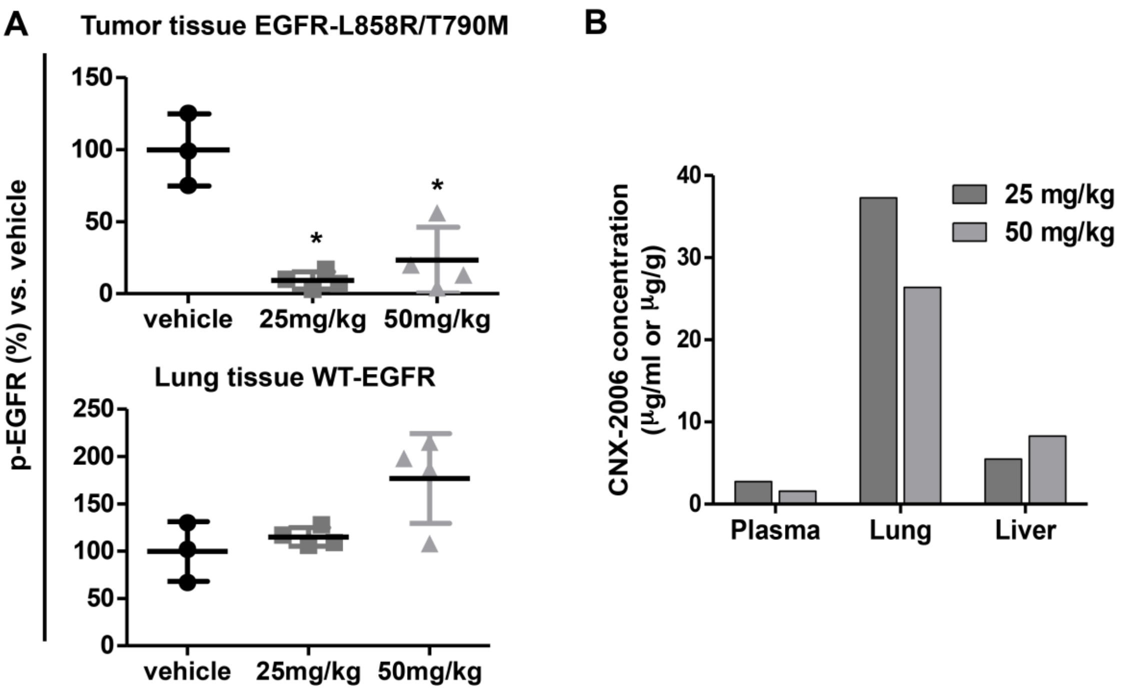

Supplementary Figure 4. (A) Quantification of phospho-EGFR in NCI-H1975 (upper plot) tumors and in normal lung tissue (bottom plot) following vehicle, 25 or 50 mg/kg IP CNX-2006 administration (N=4 animals/gp). Established (300-500 mm^3) NCI-H1975 tumors following three days of drug administration were harvested at 1 hour post-last dose for EGFR signaling analysis. Phospho-EGFR levels from Fig. 2B were quantified by densitometric analysis and plotted relative to the vehicle treated group (vehicle = 100%). Data plotted as mean ± SEM. **P<0.05* comparing vehicle to treated groups; **(B)** CNX-2006 concentration in plasma (µg/ml), lung and liver tissue (µg/g) 1 hour after IP administration of either 25 or 50 mg/kg CNX-2006

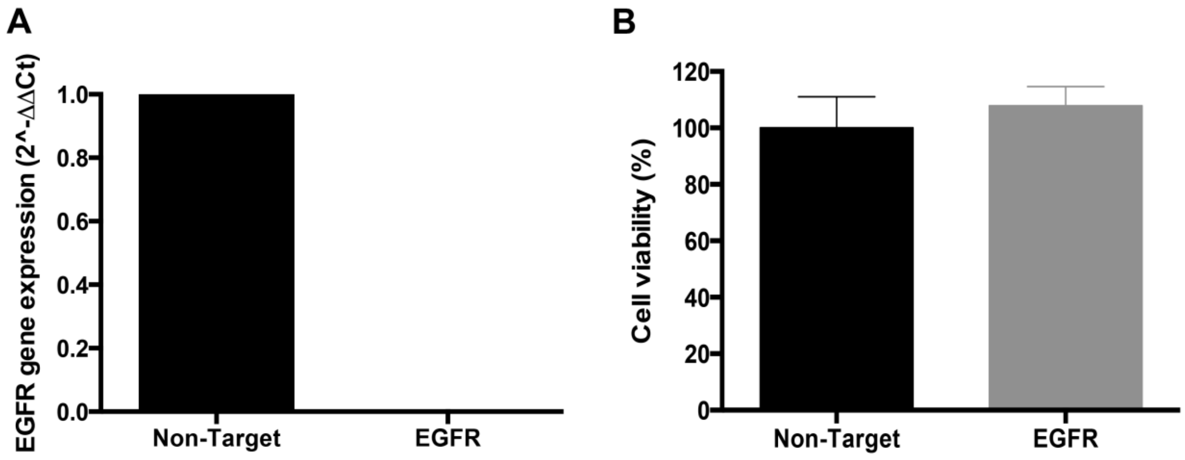

Supplementary Figure 5. (A) EGFR gene expression as determined by qRT-PCR in H1975CR 72-hours post-transfection with Non-Target or EGFR specific siRNA; data analyzed by the 2^-ΔΔCt method normalized to beta-actin; **(B)** cell viability assessed 72-hours post-transfection with Non-Target or EGFR specific siRNA. Non-Target siRNA treated cells were used as control representing 100% cell viability. Data plotted as mean ± SEM.

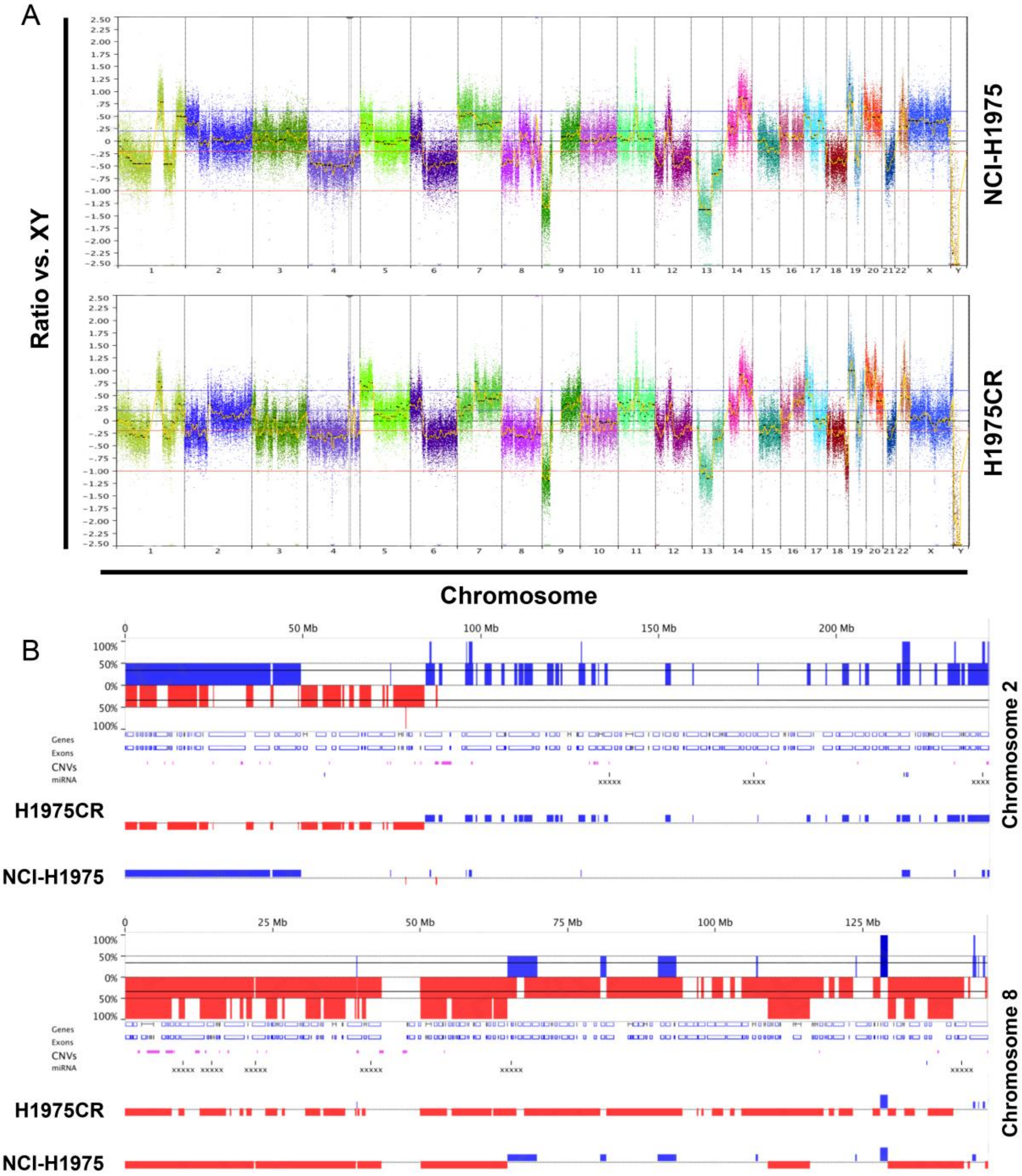

Supplementary Figure 6. (A) Whole genome copy number variation relative to XX/XY controls; **(B)** differential copy number variations in chromosome 2 and 8 in parental and resistant cells *vs*. XX/XY controls. *Red*, loss; *blue*, gain. http://www.ncbi.nlm.nih.gov/geo/info/linking.html

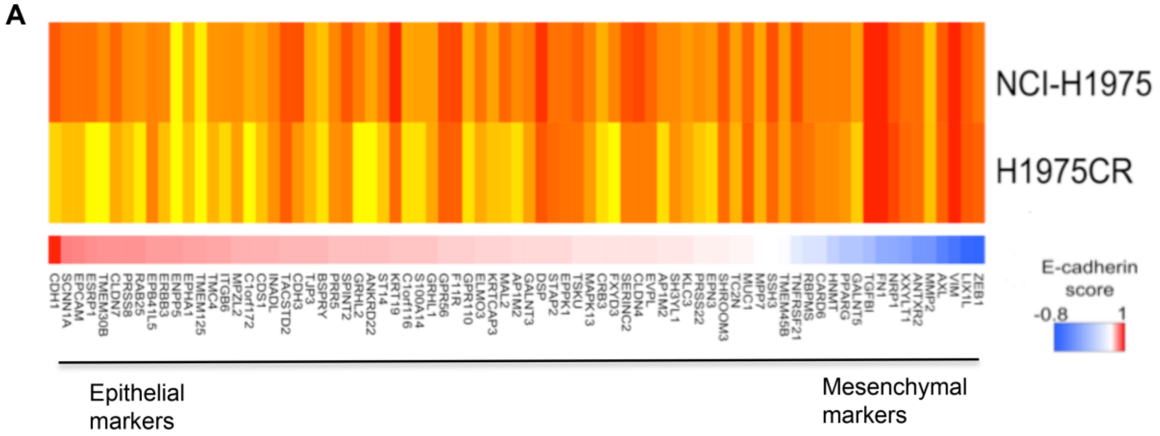

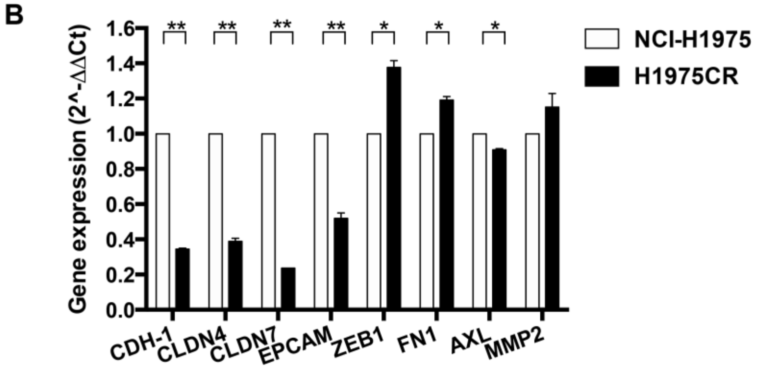

Supplementary Figure 7. (A) Gene expression analysis of the 76 genes included in the EMT signature by Byers et al. in NCI-H1975 and H1975CR cells. Log2 normalized counts of the genes ranked according to E-cadherin score; **(B)** gene expression as determined by qRT-PCR in NCI-H1975 and H1975CR of the indicated EMT markers; data analyzed by the 2^-ΔΔCt method are normalised to beta-actin. Data plotted as mean ± SEM. **P<0.05; **P<0.01*.

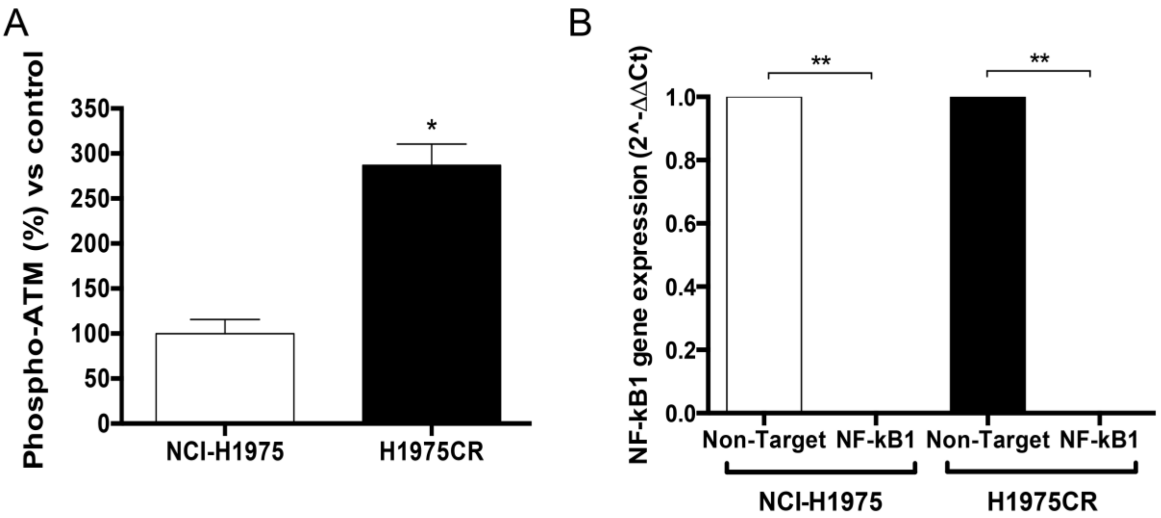

Supplementary Figure 8: (A) ATM basal phosphorylation in NCI-H1975 and H1975CR cells as determined by ELISA; **(B)** NF-kB1 gene expression as determined by qRT-PCR in NCI-H1975 and H1975CR following siRNA knock-down 72-hours post-transfection; data analyzed by the 2^-ΔΔCt method normalized to beta-actin. Data plotted as mean ± SD. **P<0.05; **P<0.01*.

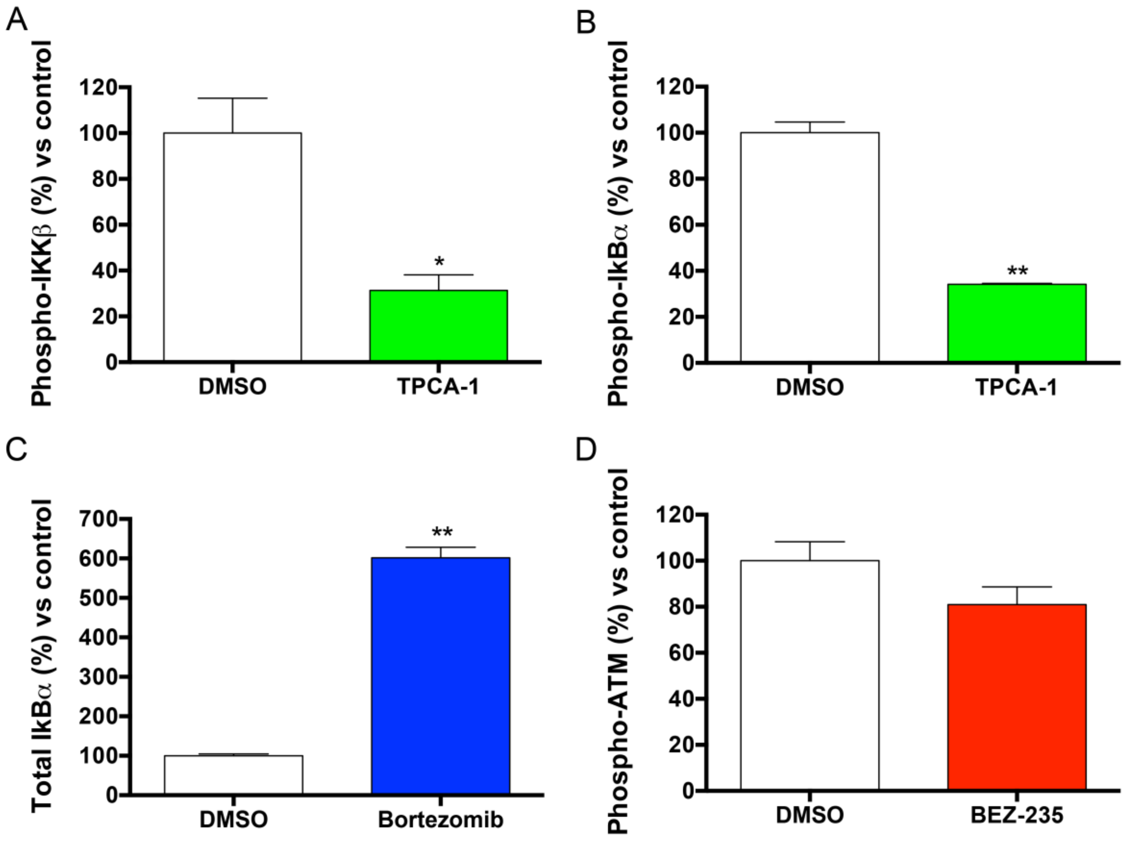

Supplementary Figure 9. (A) Levels of phospho-IKKβ after 2-hour treatment with 1 μM TPCA-1; **(B)** levels of phospho-IkBα after 2-hour treatment with 1 μM TPCA-1; **(C)** levels of IkBα after 72 hours treatment with IC_{50} Bortezomib; **(D)** levels of phospho-ATM after 2-hour treatment with 1 μM BEZ-235. Data plotted as mean ± SD. **P<0.05; **P<0.01*.

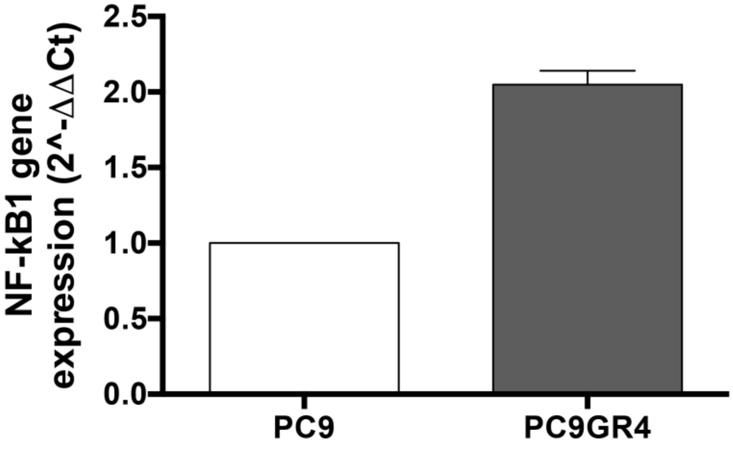

Supplementary Figure 10. Basal gene expression levels of NF-kB1 in PC9 and PC9GR4 cells as determined by qRT-PCR; data analyzed by the 2^-ΔΔCt method normalized to beta-actin.

Supplementary Table 1. Kinase inhibition profile of CNX-2006 in a panel of recombinant kinases

Kinases	% Enzyme Inhibition		
	Data 1	Data 2	AVE
EGFR-L858R/T790M	95.76	96.17	95.96
TXK	94.09	93.69	93.89
FLT3	86.56	86.92	86.74
FAK/PTK2	86.50	84.50	85.50
WT-EGFR	81.99	83.41	82.70
CHK2	81.79	83.60	82.70
ERBB4/HER4	79.90	84.18	82.04
JAK3	74.17	73.45	73.81
BMX/ETK	72.26	73.65	72.95
ITK	64.73	63.26	63.99
BTK	58.77	60.49	59.63
BLK	44.31	46.44	45.37
IGF1R	44.22	41.40	42.81
Aurora A	42.10	39.23	40.66
IR	39.96	40.33	40.14
PLK1	37.44	41.53	39.48
DAPK1	21.36	26.44	23.90
DYRK2	22.02	24.48	23.25
TEC	19.24	15.83	17.54
IKKa/CHUK	17.95	3.84	10.90
ROCK1	12.25	6.11	9.18
FMS	10.75	6.81	8.78
TAK1	7.23	9.06	8.14
c-Kit	7.41	5.43	6.42
c-MET	10.43	1.55	5.99
JNK1	6.84	4.69	5.77
NEK2	5.05	5.71	5.38
FGFR3	5.78	4.75	5.26
ERK1	5.26	4.70	4.98
PKCa	2.75	4.76	3.76
TRKA	5.72	1.05	3.39
YES/YES1	5.38	0.83	3.11
FLT1/VEGFR1	1.35	3.93	2.64
WNK2	3.69	0.58	2.13
LYN	0.23	2.74	1.48
PAK2	2.34	0.14	1.24
RET	-1.38	2.82	0.72

100%

0%

Percentage of enzyme inhibition of 1 μM CNX-2006 in the presence of 100 μM ATP. DMSO and staurosporine were used as negative and positive control, respectively.
AVE, average.

Supplementary Table 2. Cell signaling profile in NCI-H1975 cells treated with either CNX-2006 or gefitinib

Substrate ID	Substrate Phosphorylation inhibition (%)	
	CNX-2006	Gefitinib
MK01_180_192	98.78	60.99
PRGR_545_557	97.75	83.91
LAT_249_261	74.43	22.38
PGFRB_1002_1014	71.61	14.25
CD3Z_146_158	71.44	38.38
RAF1_332_344	70.70	53.97
K2C8_425_437	68.66	6.39
FAK1_569_581	66.59	8.46
VGFR3_1061_1073	66.09	69.70
CALM_95_107	64.52	69.60
EGFR_1165_1177	63.72	-2.24
PDPK1_2_14	62.20	45.01
PAXI_24_36	61.30	35.37
NTRK1_489_501	59.94	75.70
VGFR2_1168_1180	59.19	89.27
P85A_600_612	57.78	40.16
PGFRB_1014_1028	57.26	31.36
KSYK_518_530	57.02	84.13
CRK_214_226	56.75	-3.18
VGFR1_1320_1332	56.24	32.37
SRC8_CHICK_470_482	55.04	-25.95
EGFR_908_920	53.13	-94.96
RET_1022_1034	52.68	51.61
PGFRB_572_584	52.58	31.20
SRC8_CHICK_476_488	52.46	38.24
MK12_178_190	52.39	9.98
SRC8_CHICK_492_504	51.84	41.00
ENOG_37_49	51.34	24.71

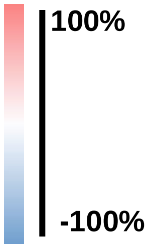

Substrate phosphorylation inhibition profile of 1 μM drug, in cell lysate from NCI-H1975 cells in the presence of 100 μM ATP. DMSO was used as control. Fifty % inhibition was chosen as cutoff for CNX-2006 activity.

Supplementary Table 3. Phospho-peptides and associated kinases in PamChip array			
**Substrate ID**	**Phospho-site**	**Description**	**Associated kinase**
**CALM_95_107**	Y99/S101	Calmodulin	**IGFR/EGFR/c-Src/JAK2**
**CD3Z_146_158**	Y153	T-cell surface glycoprotein CD3 zeta chain	**ZAP70/Syk**
**CDK7_157_169**	Y169/S164	Cell division protein kinase 7	**CDK1/CDK2**
**CRK_214_226**	Y221#	Proto-oncogene C-crk (p38)	**PTK**
**DYR1A_312_324**	Y319/Y321	Dual specificity Y-phosphorylation-regulated kinase 1A	**DYR1A**
**EGFR_1062_1074**	Y1068#	Epidermal growth factor receptor	**EGFR**
**EGFR_1118_1130**	Y1125	Epidermal growth factor receptor	**EGFR**
**EGFR_1165_1177**	Y1172	Epidermal growth factor receptor	**EGFR**
**EGFR_908_920**	Y915	Epidermal growth factor receptor	**EGFR**
**ENOG_37_49**	Y44	Gamma-enolase	**c-Src**
**ERBB4_1181_1193**	Y1188	Receptor tyrosine-protein kinase erbB-4	**ERBB-4**
**FABH_13_25**	Y19	Fatty acid-binding protein, heart	**PKC/IGFR**
**FAK1_569_581**	Y576/Y577	Focal adhesion kinase 1	**Rho/FAK1**
**FGFR3_641_653**	Y643	Fibroblast growth factor receptor 3	**FGFR3**
**K2C8_425_437**	Y427/Y437	Keratin, type II cytoskeletal 8	**p38**
**KSYK_518_530**	Y525/Y526	Tyrosine-protein kinase SYK	**Syk**
**LAT_249_261**	Y255	Linker for activation of T-cells family member 1	**ZAP70/Syk**
**MBP_198_210**	Y203*	Myelin basic protein	**MAPK/PKA/PKC**
**MBP_259_271**	Y268*	Myelin basic protein	**MAPK/PKA/PKC**
**MK01_180_192**	Y187	Mitogen-activated protein kinase 1 (ERK2)	**MAP2K1/MAP2K3**
**MK01_198_210**	Y205	Mitogen-activated protein kinase 1 (ERK2)	**MAP2K1/MAP2K3**
**MK12_178_190**	Y185	Mitogen-activated protein kinase 12	**MAP2K**
**NPT2A_501_513**	Y511/T508	Renal sodium-dependent phosphate transport protein 2	**PKC**
**NTRK1_489_501**	Y496	High affinity nerve growth factor receptor	**NTRK1**
**ODPAT_291_303**	Y299	Pyruvate dehydrogenase E1 subunit alpha	**PDK**
**P85A_600_612**	Y607#	Phosphatidylinositol 3-kinase regulatory subunit alpha	**RTK**
**PAXI_24_36**	Y31/Y33	Paxillin	**Rho**
**PDPK1_369_381**	Y373/Y376	3-phosphoinositide-dependent protein kinase 1	**PI3K/Src**
**PGFRB_1002_1014**	Y1009	Beta-type platelet-derived growth factor receptor	**PDGFRB**
**PGFRB_1014_1028**	Y1021	Beta-type platelet-derived growth factor receptor	**PDGFRB**
**PGFRB_572_584**	Y579/Y581	Beta-type platelet-derived growth factor receptor	**PDGFRB**
**PLCG1_776_788**	Y783	1-phosphatidylinositol-4,5-bisphosphate phosphodiesterase gamma 1	**ITK/SYK/TXK**
**PRGR_545_557**	Y557	Progesteron receptor	**CDK2**
**PRGR_786_798**	Y795†	Progesteron receptor	**CDK2**
**RAF1_332_344**	Y340/Y341	RAF proto-oncogene serine/threonine-protein kinase	**PAK1/Raf**
**RBL2_99_111**	Y111/S103	Retinoblastoma-like protein 2	**CDK1/CDK2**
**RET_1022_1034**	Y1029	Proto-oncogene tyrosine-protein kinase receptor ret	**RET**
**SRC8_CHICK_470_482**	Y477	Src substrate protein p85 (p80)	**RTK**
**SRC8_CHICK_476_488**	Y477/Y483	Src substrate protein p85 (p80)	**RTK**
**SRC8_CHICK_492_504**	Y492/Y499/502	Src substrate protein p85 (p80)	**RTK**
**STA5A_687_699**	Y694	Signal transducer and activator of transcription 5A	**JAK2**
**STAT1_694_706**	Y701	Signal transducer and activator of transcription 1-alpha/beta	**JAK1/JAK2**
**STAT4_686_698**	Y693	Signal transducer and activator of transcription 4	**JAK1/JAK2**
**STAT6_634_646**	Y641	Signal transducer and activator of transcription 6	**JAK1/JAK3**
**TNNT1_2_14**	Y9*	Troponin T	**PKC**
**TYRO3_679_691**	Y686	Tyrosine-protein kinase receptor TYRO3	**TYRO3**
**VGFR1_1206_1218**	Y1213	Vascular endothelial growth factor receptor 1	**VEGFR1**
**VGFR1_1320_1332**	Y1327	Vascular endothelial growth factor receptor 1	**VEGFR1**
**VGFR2_1168_1180**	Y1175	Vascular endothelial growth factor receptor 2	**VEGFR2**
**VGFR2_1207_1219**	Y1214	Vascular endothelial growth factor receptor 2	**VEGFR2**
**VGFR3_1061_1073**	Y1063/Y1068	Vascular endothelial growth factor receptor 3	**VEGFR3**
**VINC_815_827**	Y821	Vinculin	**PKC**
**ZBT16_621_633**	Y630/S628	Zinc finger and BTB domain-containing protein 16	**PDPK1**

*** not indicated by Uniprot (http://www.uniprot.org/uniprot/)/ Scansite (http://scansite3.mit.edu/); # indicated as SH2 domain by Scansite; † indicated as ATM_K domain by Scansite.**
