# Supplementary material for: NF-κB drives acquired resistance to a novel mutant-selective EGFR inhibitor
Source: Oncotarget. 2015 Apr 29;6(40):42717–32. doi: 10.18632/oncotarget.3956 (PMC4767465; doi:10.18632/oncotarget.3956)
Supplement: Supplementary file 2 [file oncotarget-06-42717-s002.pdf]

ensembl_gene_id	NCI-H1975	Test1 (H1975CR)	gene_biotype	chromc	start_position	end_position	gene_id
ENSG00000000003	7.593829938	8.211532164	protein_coding	X	99883667	99894988	TSPAN6
ENSG00000000419	8.318496529	8.598211347	protein_coding	20	49551404	49575092	DPM1
ENSG00000000457	5.472368541	5.726093467	protein_coding	1	169821804	169863408	SCYL3
ENSG00000000460	6.372135143	6.807590479	protein_coding	1	169631245	169823221	C1orf112
ENSG00000000938	0.869158192	1.353254395	protein_coding	1	27938575	27961788	FGR
ENSG00000000971	5.239686603	1.353254395	protein_coding	1	196621008	196716634	CFH
ENSG00000001036	9.807018378	9.873397164	protein_coding	6	143816614	143832827	FUCA2
ENSG00000001084	8.241782151	8.080102494	protein_coding	6	53362139	53481768	GCLC
ENSG00000001167	9.389186877	9.785641795	protein_coding	6	41040684	41067715	NFYA
ENSG00000001460	6.640654398	6.149127429	protein_coding	1	24683489	24743424	C1orf201
ENSG00000001461	8.421213901	8.35545848	protein_coding	1	24742284	24799466	NIPAL3
ENSG00000001497	10.35389236	10.56493943	protein_coding	X	64732462	64754655	LAS1L
ENSG00000001561	1.407729925	2.971122874	protein_coding	6	46097701	46114435	ENPP4
ENSG00000001617	8.357195795	7.728957399	protein_coding	3	50192478	50226508	SEMA3F
ENSG00000001629	9.92763636	9.321350625	protein_coding	7	91875548	92030698	ANKIB1
ENSG00000001630	5.899377978	5.137803333	protein_coding	7	91741465	91772266	CYP51A1
ENSG00000001631	8.207901394	7.73528765	protein_coding	7	91828283	91875480	KRIT1
ENSG00000002016	7.537174746	7.582164879	protein_coding	12	1021243	1100356	RAD52
ENSG00000002079	5.806547963	3.493416095	pseudogene	7	98836417	98908753	MYH16
ENSG00000002330	7.269909329	7.635032283	protein_coding	11	64037302	64052176	BAD
ENSG00000002549	9.093128531	9.551614431	protein_coding	4	17578815	17609595	LAP3
ENSG00000002586	9.703551077	10.31565348	protein_coding	X	2609220	2659350	CD99
ENSG00000002587	5.889353623	2.039052734	protein_coding	4	11394774	11431389	HS3ST1
ENSG00000002726	1.799000381	0.390640832	protein_coding	7	150521715	150558592	ABP1
ENSG00000002745	2.671945279	2.144285137	protein_coding	7	120965421	120981158	WNT16
ENSG00000002746	6.188122351	5.248158938	protein_coding	7	43152198	43602938	HECW1
ENSG00000002822	8.911889356	8.83570377	protein_coding	7	1855383	2272878	MAD1L1
ENSG00000002834	12.10252306	11.39239264	protein_coding	17	37026256	37078023	LASP1
ENSG00000002919	7.423680977	7.145279744	protein_coding	17	46184911	46200105	SNX11
ENSG00000003056	9.856739382	9.927841127	protein_coding	12	9092959	9102551	M6PR
ENSG00000003096	3.698846687	4.127774132	protein_coding	X	117031776	117251303	KLHL13
ENSG00000003137	5.958104008	5.004391715	protein_coding	2	72356367	72375167	CYP26B1
ENSG00000003147	3.448790144	3.279072565	protein_coding	7	8152814	8302317	ICA1
ENSG00000003249	7.0218028	6.50081754	protein_coding	16	90071273	90086536	DBNDD1
ENSG00000003393	8.888175056	8.644911378	protein_coding	2	202565277	202645912	ALS2
ENSG00000003400	7.575190993	6.980975423	protein_coding	2	202047604	202094129	CASP10
ENSG00000003402	7.916116295	8.326402026	protein_coding	2	201980827	202029033	CFLAR
ENSG00000003436	9.809013881	9.51467792	protein_coding	2	188328957	188430487	TFPI
ENSG00000003509	7.633406721	7.955508102	protein_coding	2	37458774	37480546	C2orf56
ENSG00000003756	8.874265427	9.124010896	protein_coding	3	50126341	50156454	RBM5
ENSG00000003987	3.211941663	3.813624741	protein_coding	8	17155539	17271037	MTMR7
ENSG00000003989	6.788216822	6.167968749	protein_coding	8	17354597	17428082	SLC7A2
ENSG00000004059	9.697818752	9.370979666	protein_coding	7	127228399	127231759	ARF5
ENSG00000004139	5.741167503	6.235021771	protein_coding	17	26691378	26728046	SARM1
ENSG00000004142	11.15660165	10.77947409	processed_transc	17	26673659	26684545	POLDIP2
ENSG00000004399	8.723027268	7.563312496	protein_coding	3	129274018	129325661	PLXND1
ENSG00000004455	10.3557137	10.5557289	protein_coding	1	33473585	33546597	AK2
ENSG00000004468	0	0.390640832	protein_coding	4	15779898	15851069	CD38
ENSG00000004478	11.31757708	10.91320863	protein_coding	12	2904119	2914576	FKBP4
ENSG00000004487	10.36885012	10.51369144	protein_coding	1	23345941	23410182	KDM1A
ENSG00000004534	9.662202373	10.39075874	protein_coding	3	49977440	50137478	RBM6
ENSG00000004660	6.579622637	5.700439663	protein_coding	17	3763609	3796338	CAMKK1
ENSG00000004700	8.942510063	9.018963395	protein_coding	12	21621845	21654603	RECQL
ENSG00000004766	8.074911866	8.177844832	protein_coding	7	92861653	92988338	CCDC132
ENSG00000004776	10.2752422	9.81083605	protein_coding	19	36245470	36247930	HSPB6
ENSG00000004777	7.31554162	7.195117037	protein_coding	19	36266417	36279724	ARHGAP33
ENSG00000004779	8.708847212	9.315729406	protein_coding	16	23592323	23607677	NDUFAB1
ENSG00000004799	5.158541613	1.16600992	protein_coding	7	95212811	95225828	PDK4
ENSG00000004838	2.238690726	3.532712221	protein_coding	3	50378541	50384283	ZMYND10
ENSG00000004848	0.499066092	1.667587519	protein_coding	X	25021811	25034065	ARX
ENSG00000004864	9.646664866	9.15109385	protein_coding	7	95749532	95951459	SLC25A13
ENSG00000004866	7.757079669	7.655203404	protein_coding	7	116593292	116870157	ST7

ENSG00000004897	9.693504511	9.721192935	protein_coding	17	45195069	45266788	CDC27
ENSG00000004939	1.16343121	0.390640832	protein_coding	17	42325753	42345509	SLC4A1
ENSG00000004948	0.869158192	0.950786998	protein_coding	7	93053799	93204042	CALCR
ENSG00000004961	8.610066544	8.953504395	protein_coding	X	11129421	11141198	HCCS
ENSG00000004975	9.747220804	10.11989792	protein_coding	17	7128660	7137868	DVL2
ENSG00000005001	7.030950614	2.912743273	protein_coding	16	2902728	2908171	PRSS22
ENSG00000005007	11.19703742	11.03109593	protein_coding	19	18942744	18979038	UPF1
ENSG00000005020	9.012297947	8.265014588	protein_coding	7	26706681	27034858	SKAP2
ENSG00000005022	11.99195722	12.25254279	protein_coding	X	118602363	118605282	SLC25A5
ENSG00000005059	7.751556534	8.282410365	protein_coding	4	110481361	110609874	CCDC109B
ENSG00000005073	0.499066092	0	protein_coding	7	27221129	27224842	HOXA11
ENSG00000005075	7.754320745	7.782918269	protein_coding	7	102113565	102119354	POLR2J
ENSG00000005100	10.39166588	10.39509645	protein_coding	17	5344232	5372236	DHX33
ENSG00000005102	7.759833327	7.129345795	protein_coding	17	41717756	41739322	MEOX1
ENSG00000005108	0.499066092	0	protein_coding	7	11410064	11871824	THSD7A
ENSG00000005156	8.674235392	8.458531597	protein_coding	17	33307542	33332083	LIG3
ENSG00000005175	7.578314254	7.699043321	protein_coding	12	48057070	48099844	RPAP3
ENSG00000005187	5.036678094	4.794098771	protein_coding	16	20621565	20808903	ACSM3
ENSG00000005189	6.84118134	7.492830145	protein_coding	16	20817751	20860990	
ENSG00000005194	9.46032044	9.885305703	protein_coding	16	57462081	57481440	CIAPIN1
ENSG00000005206	9.507778518	9.772364814	protein_coding	19	2328629	2355099	
ENSG00000005238	7.337827682	8.049948078	protein_coding	9	35104109	35115999	FAM214B
ENSG00000005243	4.165528823	6.1803943	protein_coding	17	46103535	46115139	COPZ2
ENSG00000005249	4.517578978	6.123616685	protein_coding	7	106685094	106802256	PRKAR2B
ENSG00000005302	7.624369505	8.618900187	protein_coding	X	11776278	11793870	MSL3
ENSG00000005339	10.38410065	10.28289766	protein_coding	16	3775055	3930722	CREBBP
ENSG00000005379	4.261916566	5.816437314	protein_coding	17	56378598	56406152	BZRAP1
ENSG00000005436	7.461385447	7.505230566	protein_coding	2	75879126	75938115	GCFC2
ENSG00000005448	7.444369108	8.120964787	protein_coding	2	74648805	74652882	WDR54
ENSG00000005469	6.335686705	6.057793599	protein_coding	7	86974997	87029111	CROT
ENSG00000005471	0.499066092	0.697730409	protein_coding	7	87031013	87109751	ABCB4
ENSG00000005483	9.837316871	9.25681271	protein_coding	7	104654626	104754808	MLL5
ENSG00000005486	9.543374069	8.700968226	protein_coding	7	75471920	75518244	RHBDD2
ENSG00000005700	9.628700366	9.500501806	protein_coding	6	82879700	82957471	IBTK
ENSG00000005801	8.006999229	8.260632671	protein_coding	11	3360491	3400448	ZNF195
ENSG00000005810	9.195324263	9.143180916	protein_coding	13	77618792	77901185	MYCBP2
ENSG00000005812	7.740446424	7.933654619	protein_coding	13	77566740	77601330	FBXL3
ENSG00000005844	0	0.697730409	protein_coding	16	30483979	30534289	ITGAL
ENSG00000005882	6.777386522	6.915587884	protein_coding	17	48172101	48189516	PDK2
ENSG00000005884	12.62804711	10.90551078	protein_coding	17	48133332	48167848	ITGA3
ENSG00000005889	8.753741308	8.936206949	protein_coding	X	24167290	24234372	ZFX
ENSG00000005893	11.08402209	11.27798894	protein_coding	X	119561682	119603220	LAMP2
ENSG00000005955	8.964161368	9.0050596	protein_coding	17	34900773	34946278	GGNBP2
ENSG00000005961	5.191549282	4.566889334	protein_coding	17	42449550	42466873	ITGA2B
ENSG00000006007	9.776749803	9.779529137	protein_coding	16	19513015	19533467	GDE1
ENSG00000006015	7.46813618	8.129000175	protein_coding	19	18699495	18703146	C19orf60
ENSG00000006016	1.960915222	1.353254395	protein_coding	19	18704037	18717660	CRLF1
ENSG00000006025	6.979904289	6.677762549	protein_coding	17	45884733	45899200	OSBPL7
ENSG00000006042	0.499066092	0	protein_coding	17	31254928	31268667	TMEM98
ENSG00000006047	7.22677265	6.387491945	protein_coding	17	7191571	7197876	YBX2
ENSG00000006062	8.307246212	7.373448694	protein_coding	17	43340488	43394414	MAP3K14
ENSG00000006075	1.799000381	1.353254395	protein_coding	17	34415602	34417515	CCL3
ENSG00000006114	8.150302734	8.401295742	protein_coding	17	35874900	35969488	SYNRG
ENSG00000006116	0.499066092	0	protein_coding	16	24266874	24374122	CACNG3
ENSG00000006118	11.28329831	11.69509293	protein_coding	11	60691935	60704631	TMEM132A
ENSG00000006125	11.91081554	11.38570126	protein_coding	17	33914282	34053434	AP2B1
ENSG00000006194	8.243750574	8.423679567	protein_coding	16	3313800	3341460	ZNF263
ENSG00000006210	10.60538231	10.62099642	protein_coding	16	57406370	57418960	CX3CL1
ENSG00000006282	9.016909338	9.515904068	protein_coding	17	48620419	48633213	SPATA20
ENSG00000006283	2.35979773	1.802319292	protein_coding	17	48638429	48704835	CACNA1G
ENSG00000006327	10.38944493	10.69431005	protein_coding	16	3070313	3072384	TNFRSF12A
ENSG00000006432	9.189208632	8.141764426	protein_coding	14	71189243	71276251	MAP3K9
ENSG00000006451	9.194306789	9.535986486	protein_coding	7	39663082	39747723	RALA

ENSG00000006453	9.3522601	8.222087975	protein_coding	7	97920963	98030380	
ENSG00000006459	7.572060955	7.529716161	protein_coding	7	139784546	139876835	JHDM1D
ENSG00000006468	6.975172889	6.720932357	protein_coding	7	13930853	14031050	ETV1
ENSG00000006530	8.98901727	8.670461892	protein_coding	7	141250989	141355044	AGK
ENSG00000006534	10.11026574	9.6159211	processed_transc	11	67776048	67796744	ALDH3B1
ENSG00000006555	5.848544806	2.652276565	protein_coding	1	55245385	55266940	TTC22
ENSG00000006576	8.225937332	8.301022059	protein_coding	7	77428109	77586818	PHTF2
ENSG00000006606	3.004694206	1.16600992	protein_coding	7	75398851	75419214	CCL26
ENSG00000006607	7.819120959	7.444711851	protein_coding	2	242295658	242434256	FARP2
ENSG00000006611	2.847891871	1.802319292	protein_coding	11	17515442	17565963	USH1C
ENSG00000006625	9.081068723	8.84060518	protein_coding	7	30536237	30544460	GGCT
ENSG00000006634	8.441937187	8.099860884	protein_coding	7	87505531	87538856	DBF4
ENSG00000006638	5.649112675	6.057793599	protein_coding	19	3594504	3606831	TBXA2R
ENSG00000006652	9.271590235	9.368945519	protein_coding	7	112063023	112121072	IFRD1
ENSG00000006695	8.815923273	8.973277306	protein_coding	17	13972846	14111994	COX10
ENSG00000006704	8.782461599	8.483815721	protein_coding	7	73868120	74016931	GTF2IRD1
ENSG00000006712	8.018542337	8.053329862	protein_coding	19	39876275	39881757	PAF1
ENSG00000006715	9.514316103	9.545023257	protein_coding	7	38762563	38971994	VPS41
ENSG00000006740	5.4452544	5.923798455	protein_coding	17	12692829	12894960	ARHGAP44
ENSG00000006744	10.98276711	11.44016012	protein_coding	17	12894930	12921381	ELAC2
ENSG00000006747	5.879259127	0.697730409	protein_coding	7	12610203	12693228	SCIN
ENSG00000006756	7.392081667	7.962719647	protein_coding	X	2822011	2847392	ARSD
ENSG00000006757	7.281453427	7.664078611	protein_coding	X	7866288	7895780	PNPLA4
ENSG00000006788	0.499066092	0.390640832	protein_coding	17	10204183	10276322	MYH13
ENSG00000006831	10.5732602	10.00540199	protein_coding	12	1797740	1897844	ADIPOR2
ENSG00000006837	2.928422289	3.993387124	protein_coding	5	133541305	133706738	CDKL3
ENSG00000007001	0	0.390640832	protein_coding	2	158851691	158992478	UPP2
ENSG00000007038	8.292108003	8.683615101	protein_coding	16	2867164	2871720	PRSS21
ENSG00000007047	7.572060955	7.210344614	protein_coding	19	45754550	45808541	MARK4
ENSG00000007062	0.499066092	0.390640832	protein_coding	4	15964699	16086001	PROM1
ENSG00000007080	10.76910987	11.13549418	protein_coding	19	18043824	18054793	CCDC124
ENSG00000007129	0.869158192	0	protein_coding	19	42041702	42093197	CEACAM21
ENSG00000007168	10.73032559	11.17095165	protein_coding	17	2496923	2588909	PAFAH1B1
ENSG00000007171	0.869158192	0	protein_coding	17	26083792	26127555	NOS2
ENSG00000007174	0.869158192	1.16600992	protein_coding	17	11501748	11873485	DNAH9
ENSG00000007202	11.59550853	11.29701405	protein_coding	17	26941459	26972472	KIAA0100
ENSG00000007237	2.471521042	3.411459265	protein_coding	17	9813926	10101868	GAS7
ENSG00000007255	6.592037289	6.601272494	protein_coding	19	45666187	45681485	TRAPPC6A
ENSG00000007264	2.10647801	2.579085888	protein_coding	19	3777968	3801810	MATK
ENSG00000007306	0.869158192	0	protein_coding	19	42177235	42192296	CEACAM7
ENSG00000007314	5.141750194	3.570966319	protein_coding	17	62015914	62050278	SCN4A
ENSG00000007341	6.407685389	6.651226688	protein_coding	1	113066140	113163447	ST7L
ENSG00000007350	0	0.390640832	protein_coding	X	153524024	153558713	TKTL1
ENSG00000007372	2.57521082	2.420525079	protein_coding	11	31806340	31839509	PAX6
ENSG00000007376	9.195324263	9.282254961	protein_coding	16	834974	838397	RPUSD1
ENSG00000007384	8.700271793	8.943515377	protein_coding	16	108058	126354	RHBDF1
ENSG00000007392	8.539709228	8.945336714	protein_coding	16	238968	279462	LUC7L
ENSG00000007402	4.026095388	3.493416095	protein_coding	3	50400233	50541675	CACNA2D2
ENSG00000007516	5.774228067	4.65855978	protein_coding	16	1383602	1399439	BAIAP3
ENSG00000007520	9.320904342	9.397831074	protein_coding	16	1399241	1401912	C16orf42
ENSG00000007541	9.548160668	9.917672197	protein_coding	16	616995	634136	PIGQ
ENSG00000007545	8.072698871	7.984140487	protein_coding	16	1662326	1727909	CRAMP1L
ENSG00000007866	8.70742151	8.861011175	protein_coding	6	35441374	35464861	TEAD3
ENSG00000007923	9.029515185	9.059047571	protein_coding	1	6694228	6761984	DNAJC11
ENSG00000007944	7.773523328	7.688207601	protein_coding	6	16129356	16148479	MYLIP
ENSG00000007952	2.10647801	1.518964905	protein_coding	X	100098313	100129334	NOX1
ENSG00000007968	7.654275977	7.711939664	protein_coding	1	23832922	23857712	E2F2
ENSG00000008018	10.54320643	10.64808893	protein_coding	6	170844205	170862429	PSMB1
ENSG00000008056	3.274897671	4.489113623	protein_coding	X	47431303	47479252	SYN1
ENSG00000008083	9.30119149	8.780659733	protein_coding	6	15245734	15522252	JARID2
ENSG00000008086	3.871730003	4.468996429	protein_coding	X	18443703	18671749	CDKL5
ENSG00000008118	1.960915222	1.16600992	protein_coding	1	209757062	209787283	CAMK1G
ENSG00000008128	8.241782151	8.003500716	protein_coding	1	1634169	1655966	CDK11A

ENSG00000008130	9.922115648	10.03777287	protein_coding	1	1682671	1711896	NADK
ENSG00000008226	2.238690726	1.667587519	protein_coding	3	38080696	38165516	DLEC1
ENSG00000008256	9.331123617	8.511125832	protein_coding	7	6201407	6312275	CYTH3
ENSG00000008277	1.799000381	1.518964905	protein_coding	7	87563458	87832204	ADAM22
ENSG00000008282	9.881603942	9.521408873	protein_coding	7	105730949	105753022	SYPL1
ENSG00000008283	10.47697524	9.761034214	protein_coding	17	61509668	61523722	CYB561
ENSG00000008294	10.40095672	10.18146283	protein_coding	17	49039535	49198226	SPAG9
ENSG00000008300	8.732871027	8.549948921	protein_coding	3	48673902	48700348	CELSR3
ENSG00000008311	6.244259325	6.811590016	protein_coding	7	121715701	121784334	AASS
ENSG00000008323	5.472368541	2.721932731	protein_coding	12	6419602	6437672	PLEKHG6
ENSG00000008324	6.335686705	6.642271819	protein_coding	3	42623332	42636606	SS18L2
ENSG00000008382	7.530740159	6.847095496	protein_coding	19	4343524	4360082	MPND
ENSG00000008394	11.02515609	10.64416949	protein_coding	12	16500076	16762193	MGST1
ENSG00000008405	7.896231123	7.204272857	protein_coding	12	107385142	107487607	CRY1
ENSG00000008438	3.004694206	1.802319292	protein_coding	19	46522415	46526556	PGLYRP1
ENSG00000008441	12.27220068	12.12937721	protein_coding	19	13106584	13209610	NFIX
ENSG00000008513	9.370391259	8.578395649	protein_coding	8	134467091	134584183	ST3GAL1
ENSG00000008516	5.763291843	6.465715653	protein_coding	16	3096682	3110703	MMP25
ENSG00000008517	10.6854095	11.90805612	protein_coding	16	3115298	3131908	IL32
ENSG00000008710	10.19815945	10.44293712	protein_coding	16	2138711	2185899	PKD1
ENSG00000008735	7.46813618	5.32851069	protein_coding	22	51039114	51052409	MAPK8IP2
ENSG00000008838	10.09126063	9.89993707	protein_coding	17	38175350	38210661	MED24
ENSG00000008853	7.657232808	7.318294738	protein_coding	8	22844930	22877712	RHOBTB2
ENSG00000008869	8.027710784	8.151264044	protein_coding	2	37195526	37311485	HEATR5B
ENSG00000008952	9.632461462	9.568257332	protein_coding	3	169684423	169716161	SEC62
ENSG00000008988	11.22943395	11.75823332	protein_coding	8	56979854	56987069	RPS20
ENSG00000009307	11.88361595	11.93374232	protein_coding	1	115259534	115301297	CSDE1
ENSG00000009335	10.80254523	10.39542958	protein_coding	7	156931607	157062066	UBE3C
ENSG00000009413	8.059349131	8.347216179	protein_coding	6	111620234	111804918	REV3L
ENSG00000009694	0	0.697730409	protein_coding	X	123509753	124097666	ODZ1
ENSG00000009724	4.19837882	3.935919592	protein_coding	1	11086580	11107290	MASP2
ENSG00000009780	5.672682881	5.339632724	protein_coding	1	28052490	28088475	FAM76A
ENSG00000009790	4.821592551	4.566889334	protein_coding	1	209929377	209955668	TRAF3IP3
ENSG00000009830	9.648894781	10.10123101	protein_coding	14	77741299	77787227	POMT2
ENSG00000009844	8.797280438	8.81990641	protein_coding	6	142468367	142542085	VTA1
ENSG00000009950	6.320845526	3.411459265	protein_coding	7	73007524	73038873	MLXIPL
ENSG00000009954	11.15764605	10.73119995	protein_coding	7	72854728	72936608	BAZ1B
ENSG00000010017	8.809292739	9.068270303	protein_coding	6	13621730	13711953	RANBP9
ENSG00000010030	4.981125677	5.22435463	protein_coding	6	36322419	36356164	ETV7
ENSG00000010072	7.633406721	7.694718794	protein_coding	1	231472850	231490769	C1orf124
ENSG00000010165	8.8704485	9.190020311	protein_coding	1	171750788	171783163	METTL13
ENSG00000010219	6.26766507	6.835357159	protein_coding	12	4671370	4723325	DYRK4
ENSG00000010244	10.77354482	10.64836849	protein_coding	17	30677150	30714780	ZNF207
ENSG00000010256	11.10257995	11.09402129	protein_coding	3	48636435	48648409	UQCRC1
ENSG00000010270	8.299696963	8.17939326	protein_coding	7	38217824	38270272	STARD3NL
ENSG00000010278	9.70569484	9.362144241	protein_coding	12	6308881	6347427	CD9
ENSG00000010282	0.499066092	0.390640832	protein_coding	3	42734155	42744319	HHATL
ENSG00000010292	11.74213696	11.08702611	protein_coding	12	6602522	6641121	NCAPD2
ENSG00000010295	7.461385447	7.515074729	protein_coding	12	6647541	6665239	IFFO1
ENSG00000010310	5.485736881	4.274079748	protein_coding	19	46171502	46185704	GIPR
ENSG00000010318	5.472368541	5.840125386	protein_coding	3	52443510	52457657	PHF7
ENSG00000010319	2.471521042	2.242360793	protein_coding	3	52467069	52479101	SEMA3G
ENSG00000010322	9.151960225	9.305130221	protein_coding	3	52489134	52527087	NISCH
ENSG00000010327	0.499066092	0.950786998	protein_coding	3	52529354	52558511	STAB1
ENSG00000010361	5.472368541	5.674321415	protein_coding	19	50310126	50320633	FUZ
ENSG00000010379	0.499066092	1.667587519	protein_coding	12	329789	372039	SLC6A13
ENSG00000010404	9.536164262	9.661506506	protein_coding	X	148558521	148615470	IDS
ENSG00000010438	6.0776925	2.971122874	protein_coding	9	33750515	33799230	PRSS3
ENSG00000010539	7.478203418	7.683850422	protein_coding	16	3272325	3285456	ZNF200
ENSG00000010610	0.869158192	1.518964905	protein_coding	12	6896024	6929974	CD4
ENSG00000010626	6.129712275	5.901455548	protein_coding	12	6982733	7023407	LRRC23
ENSG00000010671	0.869158192	1.16600992	protein_coding	X	100604435	100641183	BTK
ENSG00000010704	7.543580762	7.167295693	protein_coding	6	26087509	26098571	HFE

ENSG00000010803	8.549290825	8.630267069	protein_coding	1	41492872	41707826	SCMH1
ENSG00000010810	6.252103507	8.117738058	protein_coding	6	111981535	112194655	FYN
ENSG00000010818	8.890689734	8.93895195	protein_coding	6	143072604	143266338	HIVEP2
ENSG00000010932	0.499066092	1.16600992	protein_coding	1	171217638	171255117	FMO1
ENSG00000011007	9.375786469	9.429406685	protein_coding	1	24069645	24088549	TCEB3
ENSG00000011009	9.388297379	9.20378064	protein_coding	1	24117460	24122029	LYPLA2
ENSG00000011021	8.032273249	7.741590246	protein_coding	1	11866207	11903201	CLCN6
ENSG00000011028	8.408986408	8.190185975	protein_coding	17	60704762	60770956	MRC2
ENSG00000011052	7.416718478	7.439552137	protein_coding	17	49230920	49249108	NME2
ENSG00000011083	0.869158192	0.390640832	protein_coding	5	149569520	149602351	SLC6A7
ENSG00000011105	9.192269688	8.531880603	protein_coding	12	3186521	3395730	TSPAN9
ENSG00000011114	9.0577617	8.398639372	protein_coding	14	93703896	93799438	BTBD7
ENSG00000011132	8.646233023	9.112709611	protein_coding	19	3750771	3761673	APBA3
ENSG00000011143	7.709444959	7.83885796	protein_coding	17	56282799	56296966	MKS1
ENSG00000011198	7.292905885	7.929979971	protein_coding	3	43731605	43775863	ABHD5
ENSG00000011201	1.16343121	1.16600992	protein_coding	X	8496915	8700227	KAL1
ENSG00000011243	9.41997878	9.353254373	protein_coding	19	15490859	15529833	AKAP8L
ENSG00000011258	7.666067113	7.728957399	protein_coding	17	49254786	49337524	MBTD1
ENSG00000011260	8.695964893	8.628000846	protein_coding	17	49337889	49375297	UTP18
ENSG00000011275	10.16062356	10.08397376	protein_coding	7	5659678	5821370	RNF216
ENSG00000011295	8.663981856	9.286571792	protein_coding	17	15902782	15948336	TTC19
ENSG00000011304	12.54148129	12.35046418	protein_coding	19	797392	812327	PTBP1
ENSG00000011332	5.472368541	5.317302248	protein_coding	19	38701646	38720354	DPF1
ENSG00000011347	8.267164507	6.480864016	protein_coding	11	61282785	61348620	SYT7
ENSG00000011376	8.749591335	8.457255683	protein_coding	3	45429998	45590913	LARS2
ENSG00000011405	9.801015254	10.0097603	protein_coding	11	17099277	17229530	PIK3C2A
ENSG00000011422	8.986668406	8.398639372	protein_coding	19	44150271	44174502	PLAUR
ENSG00000011426	11.80147326	11.84396407	protein_coding	7	36429415	36493400	ANLN
ENSG00000011451	10.76842635	10.8620608	protein_coding	19	15532319	15560762	WIZ
ENSG00000011454	9.076658223	9.00068704	protein_coding	9	125703112	125867145	RABGAP1
ENSG00000011465	0	0.390640832	protein_coding	12	91539025	91576900	DCN
ENSG00000011478	8.129161475	8.602834575	protein_coding	19	46195741	46207240	QPCTL
ENSG00000011485	9.576551475	9.752217392	protein_coding	19	46850251	46896238	PPP5C
ENSG00000011523	7.726437377	7.65742733	protein_coding	2	65283500	65314138	CEP68
ENSG00000011566	8.261346552	8.362291274	protein_coding	2	39476407	39664453	MAP4K3
ENSG00000011590	1.960915222	2.334191469	protein_coding	19	36203830	36207940	ZBTB32
ENSG00000011638	8.659565077	8.53672099	protein_coding	16	21169698	21191937	TMEM159
ENSG00000012048	9.008829705	8.920552509	protein_coding	17	41196312	41322290	BRCA1
ENSG00000012061	6.872051157	7.373448694	protein_coding	19	45910591	45982086	ERCC1
ENSG00000012124	1.960915222	2.144285137	protein_coding	19	35820072	35838258	CD22
ENSG00000012171	7.530740159	8.132201841	polymorphic_pse	3	50304990	50314977	SEMA3B
ENSG00000012174	8.795939543	9.243551304	protein_coding	X	21857754	21903542	MBTPS2
ENSG00000012211	8.965354743	8.820898829	protein_coding	X	49031151	49042845	PRICKLE3
ENSG00000012223	5.40360309	6.99159092	protein_coding	3	46477136	46526724	LTF
ENSG00000012232	9.77334709	9.379087693	protein_coding	8	28457986	28613116	EXTL3
ENSG00000012660	10.68396043	10.36445494	protein_coding	6	53132196	53213947	ELOVL5
ENSG00000012779	0.869158192	1.16600992	protein_coding	10	45869661	45941561	ALOX5
ENSG00000012822	8.731468881	9.471724717	protein_coding	12	54104902	54121529	CALCOCO1
ENSG00000012963	9.013452178	8.975061478	protein_coding	14	93673401	93695561	UBR7
ENSG00000012983	8.954578658	9.124010896	protein_coding	14	50885219	51027844	MAP4K5
ENSG00000013016	1.960915222	0.697730409	protein_coding	2	31456880	31492313	EHD3
ENSG00000013275	9.623418305	9.790209352	protein_coding	19	40477073	40487351	PSMC4
ENSG00000013288	8.623735441	8.929781562	protein_coding	4	6576902	6625089	MAN2B2
ENSG00000013297	8.908170811	7.989446368	protein_coding	3	170136653	170578169	CLDN11
ENSG00000013306	11.10610075	10.69241345	protein_coding	17	42396993	42402238	SLC25A39
ENSG00000013364	11.79896536	11.61268024	protein_coding	16	29828560	29859355	MVP
ENSG00000013374	9.072234198	9.012896966	protein_coding	7	151038785	151075535	NUB1
ENSG00000013375	9.24628631	9.575331647	protein_coding	6	83870869	83903655	PGM3
ENSG00000013392	5.485736881	6.136428444	protein_coding	6	83903098	83906382	RWDD2A
ENSG00000013441	8.077121473	8.220584723	protein_coding	2	201717732	201729467	CLK1
ENSG00000013503	7.609180529	7.321103092	protein_coding	12	106751436	106903976	POLR3B
ENSG00000013523	9.004192374	9.132828786	protein_coding	14	77253588	77292589	ANGEL1
ENSG00000013561	8.593182305	8.788797839	protein_coding	5	141337893	141369856	RNF14

ENSG00000013563	7.007971391	6.525377513	protein_coding	X	153629579	153640449	DNASE1L1
ENSG00000013573	9.338510612	9.397831074	protein_coding	12	31226779	31257725	DDX11
ENSG00000013583	8.398422578	7.922602475	protein_coding	12	13127798	13153207	HEBP1
ENSG00000013588	11.83106916	10.82073834	protein_coding	12	13043716	13070871	GPRC5A
ENSG00000013619	7.348842929	7.167295693	protein_coding	X	149529689	149682448	MAMLD1
ENSG00000013725	2.10647801	1.518964905	protein_coding	11	60739115	60787849	CD6
ENSG00000013810	10.06756346	9.874353473	protein_coding	4	1723227	1746898	TACC3
ENSG00000014123	7.770795701	8.188649093	protein_coding	6	96969471	97003152	UFL1
ENSG00000014138	9.373990307	9.789702559	protein_coding	11	65029233	65073060	POLA2
ENSG00000014164	8.655134734	8.165397208	protein_coding	8	144519825	144623623	ZC3H3
ENSG00000014216	11.37213372	10.55214769	protein_coding	11	64948037	64979477	CAPN1
ENSG00000014257	5.054730169	1.802319292	protein_coding	3	132036211	132087142	ACPP
ENSG00000014641	9.392739397	9.851710903	protein_coding	2	63815743	63834331	MDH1
ENSG00000014824	9.520824196	9.737577069	protein_coding	4	41992489	42089551	SLC30A9
ENSG00000014914	7.194570056	7.84667518	protein_coding	1	149900543	149908791	MTMR11
ENSG00000014919	9.195324263	9.240587696	protein_coding	10	101471601	101491866	COX15
ENSG00000015133	9.260938843	7.198175438	protein_coding	14	91737667	91884188	CCDC88C
ENSG00000015153	7.437505974	7.852510423	protein_coding	12	42550906	42632151	YAF2
ENSG00000015171	8.47750173	8.508664347	protein_coding	10	180405	300577	ZMYND11
ENSG00000015285	3.78787645	3.453019579	protein_coding	X	48534985	48549818	WAS
ENSG00000015413	0.499066092	0.390640832	protein_coding	16	89679716	89704839	DPEP1
ENSG00000015475	9.450127191	9.440426033	protein_coding	22	18216906	18257536	BID
ENSG00000015479	11.41774464	11.41852429	protein_coding	5	138609441	138667360	MATR3
ENSG00000015520	2.35979773	2.420525079	protein_coding	7	44552134	44580914	NPC1L1
ENSG00000015532	8.839544001	8.486319932	protein_coding	17	48423453	48440499	XYLT2
ENSG00000015568	2.57521082	2.334191469	protein_coding	2	110550335	110615272	RGPD5
ENSG00000015592	0	0.697730409	protein_coding	8	27092840	27115937	STMN4
ENSG00000015676	10.54639963	10.22297151	protein_coding	7	44421969	44530479	NUDCD3
ENSG00000016391	5.036678094	3.368666104	protein_coding	3	53850387	53880417	CHDH
ENSG00000016402	0.869158192	0.390640832	protein_coding	6	137321108	137366298	IL20RA
ENSG00000016864	8.552470601	8.902852973	protein_coding	3	52728505	52740048	GLT8D1
ENSG00000017260	10.52146546	10.55632491	protein_coding	3	130569439	130735556	ATP2C1
ENSG00000017373	6.0776925	5.9163892	protein_coding	17	36686259	36762183	SRCIN1
ENSG00000017427	0.499066092	0.390640832	protein_coding	12	102789645	102874423	IGF1
ENSG00000017483	3.211941663	3.748356452	protein_coding	X	48316920	48328644	SLC38A5
ENSG00000017621	4.6658819	4.917990497	protein_coding	X	49019061	49024822	MAGIX
ENSG00000017797	10.20625762	9.85268169	protein_coding	18	9475007	9538112	RALBP1
ENSG00000018189	7.206730419	7.077141316	protein_coding	4	71569921	71673032	RUFY3
ENSG00000018236	6.489593565	5.32851069	protein_coding	12	41086244	41465772	CNTN1
ENSG00000018280	2.847891871	2.912743273	protein_coding	2	219246752	219261617	SLC11A1
ENSG00000018408	9.691342543	9.31220499	protein_coding	3	149235022	149454501	WWTR1
ENSG00000018510	9.933745809	9.568257332	protein_coding	2	178257372	178408564	AGPS
ENSG00000018610	7.993024472	8.327799018	protein_coding	X	118672112	118699397	CXorf56
ENSG00000018625	1.407729925	0.390640832	protein_coding	1	160085549	160113381	ATP1A2
ENSG00000018699	8.844740944	9.052302903	protein_coding	2	32853099	33046118	TTC27
ENSG00000018869	5.695874189	6.246885341	protein_coding	19	56894648	56904889	ZNF582
ENSG00000019102	1.960915222	0.390640832	protein_coding	11	124617368	124622134	VSIG2
ENSG00000019144	9.697818752	9.506065582	protein_coding	11	118477155	118528741	PHLDB1
ENSG00000019169	1.960915222	1.667587519	protein_coding	2	119699742	119752236	MARCO
ENSG00000019186	5.763291843	2.334191469	protein_coding	20	52769988	52790512	CYP24A1
ENSG00000019485	4.517578978	4.342020395	protein_coding	11	45115564	45247734	PRDM11
ENSG00000019505	0.499066092	0	protein_coding	11	45261852	45307870	SYT13
ENSG00000019549	7.19863491	6.044260771	protein_coding	8	49830249	49834299	SNAI2
ENSG00000019582	8.485841807	4.761389014	protein_coding	5	149781200	149792492	CD74
ENSG00000019995	8.97959878	9.227175607	protein_coding	10	126630692	126676758	ZRANB1
ENSG00000020129	9.417365074	9.080752488	protein_coding	1	36023074	36032875	NCDN
ENSG00000020181	5.223818625	4.489113623	protein_coding	8	37641709	37702414	GPR124
ENSG00000020219	0	0.390640832	pseudogene	7	152142562	152144235	CCT8L1P
ENSG00000020256	8.541310587	8.472492669	protein_coding	20	50668202	50820847	ZFP64
ENSG00000020426	7.990682129	8.196317182	protein_coding	14	61201460	61436671	MNAT1
ENSG00000020577	9.993458163	10.76639755	protein_coding	14	55033815	55260033	SAMD4A
ENSG00000020633	1.407729925	1.353254395	protein_coding	1	25226002	25291612	RUNX3
ENSG00000020922	8.640267888	9.139208113	protein_coding	11	94152895	94227074	MRE11A

ENSG00000021300	4.999881834	5.57429348	protein_coding	11	73357223	73373864	PLEKHB1
ENSG00000021355	9.431250437	8.565427152	protein_coding	6	2832566	2842240	SERPINB1
ENSG00000021461	0	0.390640832	protein_coding	7	99425636	99463718	CYP3A43
ENSG00000021488	1.16343121	1.16600992	protein_coding	19	33321421	33360683	SLC7A9
ENSG00000021574	7.413224588	7.933654619	protein_coding	2	32288680	32382706	SPAST
ENSG00000021645	0	2.144285137	protein_coding	14	78708734	80330762	NRXN3
ENSG00000021762	8.177331204	7.890820294	protein_coding	11	3108346	3187969	OSBPL5
ENSG00000021776	9.221529789	8.861975727	protein_coding	15	35147732	35262040	AQR
ENSG00000021826	6.733233509	8.124184315	protein_coding	2	211342406	211543831	CPS1
ENSG00000022267	8.344411036	7.728957399	protein_coding	X	135229559	135293518	FHL1
ENSG00000022277	9.71423818	9.745961151	protein_coding	20	55043647	55093943	C20orf43
ENSG00000022556	2.471521042	1.925536307	protein_coding	19	55476438	55512510	NLRP2
ENSG00000022567	6.535310252	6.343971006	protein_coding	8	142217265	142318404	SLC45A4
ENSG00000022840	10.46900434	10.03178518	protein_coding	12	120971283	121015397	RNF10
ENSG00000022976	8.092494681	7.365308824	protein_coding	14	102783714	102809044	ZNF839
ENSG00000023041	8.825155121	9.189251987	protein_coding	10	114190058	114206672	ZDHHC6
ENSG00000023171	3.871730003	3.183544561	protein_coding	11	123396344	123498482	GRAMD1B
ENSG00000023191	10.82835312	10.89518266	protein_coding	11	494512	507300	RNH1
ENSG00000023228	10.13062028	9.940680592	protein_coding	2	206986149	207024327	NDUFS1
ENSG00000023287	9.282163564	9.824260193	protein_coding	8	53535016	53658403	RB1CC1
ENSG00000023318	9.188186835	9.564114571	protein_coding	9	102741461	102861322	ERP44
ENSG00000023330	9.489645889	9.576507334	protein_coding	3	52232102	52248343	ALAS1
ENSG00000023445	10.57443379	11.09278931	protein_coding	11	102188215	102210134	BIRC3
ENSG00000023516	8.878072281	8.660517685	protein_coding	13	42846289	42897396	AKAP11
ENSG00000023572	6.244259325	7.073814954	protein_coding	1	193065598	193075244	GLRX2
ENSG00000023608	7.813830732	8.060069742	protein_coding	14	62229075	62263146	SNAPC1
ENSG00000023697	8.382430368	8.301022059	protein_coding	12	16064106	16190220	DERA
ENSG00000023734	10.84951254	10.6393958	protein_coding	12	16035325	16056412	STRAP
ENSG00000023839	5.223818625	5.072639111	protein_coding	10	101542489	101611949	ABCC2
ENSG00000023892	8.137655191	7.41347308	protein_coding	6	35265595	35289548	DEF6
ENSG00000023902	6.970425921	7.737391576	protein_coding	1	150121373	150132260	PLEKHO1
ENSG00000023909	8.227927477	7.549009758	protein_coding	1	94350761	94374966	GCLM
ENSG00000024048	8.922987828	9.23613084	protein_coding	6	42531800	42661242	UBR2
ENSG00000024422	10.68758037	10.4179039	protein_coding	19	48216601	48246390	EHD2
ENSG00000024526	8.249639777	8.512355001	protein_coding	1	68939835	68962904	DEPDC1
ENSG00000024862	6.449220685	6.742042315	protein_coding	6	139094657	139114456	CCDC28A
ENSG00000025039	3.502389126	4.888000647	protein_coding	6	90074355	90121989	RRAGD
ENSG00000025156	6.956090577	7.201227369	protein_coding	6	122720691	122754264	HSF2
ENSG00000025293	9.435562348	9.362144241	protein_coding	20	34359896	34538303	PHF20
ENSG00000025423	5.795854912	5.832272499	protein_coding	12	57145945	57181574	HSD17B6
ENSG00000025434	7.706593329	7.797107849	protein_coding	11	47269851	47290396	NR1H3
ENSG00000025708	9.955528053	9.761034214	protein_coding	22	50964181	50968485	TYMP
ENSG00000025770	9.575770339	9.578855838	protein_coding	22	50946645	50963063	NCAPH2
ENSG00000025772	9.865075275	9.89664616	protein_coding	20	43570771	43589127	TOMM34
ENSG00000025796	9.849001727	9.940224002	protein_coding	6	108188960	108279482	SEC63
ENSG00000025800	9.766517491	9.472356449	protein_coding	1	32573639	32642169	KPNA6
ENSG00000026025	14.36107066	14.03041213	protein_coding	10	17270258	17279592	VIM
ENSG00000026036	6.560798025	7.026422172	protein_coding	20	62290653	62330037	RTEL1
ENSG00000026103	6.86695184	6.535085617	protein_coding	10	90729553	90775542	FAS
ENSG00000026297	6.640654398	7.164171052	protein_coding	6	167342992	167370679	RNASET2
ENSG00000026508	12.97388146	13.12193712	protein_coding	11	35160417	35253949	CD44
ENSG00000026559	2.10647801	3.027231696	protein_coding	20	49620193	49639666	KCNG1
ENSG00000026652	7.800519667	7.672899552	protein_coding	6	161551011	161695093	AGPAT4
ENSG00000026751	0.869158192	0.950786998	protein_coding	1	160709037	160724611	SLAMF7
ENSG00000026950	8.78516728	8.948064416	protein_coding	6	26402465	26415444	BTN3A1
ENSG00000027001	8.385999544	8.312357201	protein_coding	13	24304328	24463558	MIPEP
ENSG00000027075	6.435508026	2.912743273	protein_coding	14	61654277	62017694	PRKCH
ENSG00000027644	0.869158192	0.950786998	protein_coding	1	156809855	156828810	INSRR
ENSG00000027697	10.20170799	10.54495849	protein_coding	6	137518621	137540586	IFNGR1
ENSG00000027847	7.928406768	8.391976969	protein_coding	5	177027101	177037348	B4GALT7
ENSG00000027869	7.084651883	5.674321415	protein_coding	1	156776035	156786654	SH2D2A
ENSG00000028116	7.341508784	7.470237199	protein_coding	2	58134786	58387055	VRK2
ENSG00000028137	6.305850085	4.468996429	protein_coding	1	12227060	12269285	TNFRSF1B

ENSG00000028203	8.961771651	9.207579776	protein_coding	12	95611522	95696566	VEZT
ENSG00000028277	4.322773689	4.888000647	protein_coding	19	42592650	42700737	POU2F2
ENSG00000028310	9.645176339	9.162882465	protein_coding	5	850406	892939	BRD9
ENSG00000028528	9.145657575	9.328346488	protein_coding	15	64386322	64438289	SNX1
ENSG00000028839	7.35977471	7.243290054	protein_coding	6	134273308	134308637	TBPL1
ENSG00000029153	9.626438993	8.942603846	protein_coding	12	27485787	27576241	ARNTL2
ENSG00000029363	10.45207816	10.67110946	protein_coding	6	136578001	136610989	BCLAF1
ENSG00000029364	11.02114496	10.67495649	protein_coding	14	69864732	69929105	SLC39A9
ENSG00000029534	6.244259325	4.42790041	protein_coding	8	41510739	41754280	ANK1
ENSG00000029639	6.670228606	6.746227492	protein_coding	6	155578643	155635627	TFB1M
ENSG00000029725	10.28292016	10.99153907	protein_coding	17	5185558	5289129	RABEP1
ENSG00000029993	9.217529022	8.726612716	protein_coding	X	150148982	150159248	HMGB3
ENSG00000030066	10.08961991	10.12793923	protein_coding	11	47799639	47870107	NUP160
ENSG00000030110	8.215945216	8.14493792	protein_coding	6	33540324	33548070	BAK1
ENSG00000030304	0.869158192	0	protein_coding	9	113431051	113563859	MUSK
ENSG00000030419	5.485736881	5.995870484	protein_coding	2	213864429	214017151	IKZF2
ENSG00000030582	11.76218811	12.78306555	protein_coding	17	42422491	42430470	GRN
ENSG00000031003	7.334137164	8.024328814	protein_coding	5	137273649	137387650	FAM13B
ENSG00000031081	8.422952257	8.643790155	protein_coding	3	119013220	119139561	ARHGAP31
ENSG00000031544	3.652182994	4.319727133	processed_transc	15	72084977	72110600	NR2E3
ENSG00000031691	6.112579918	6.854868327	protein_coding	6	49431096	49460820	CENPQ
ENSG00000031698	10.77558713	11.09525222	protein_coding	1	109756540	109780791	SARS
ENSG00000031823	10.03667449	10.27603922	protein_coding	19	5916154	5978320	RANBP3
ENSG00000032219	8.070482475	7.973469823	protein_coding	14	58765103	58840605	ARID4A
ENSG00000032389	8.607011344	7.991210667	protein_coding	2	3192696	3381653	TSSC1
ENSG00000032444	11.36106887	11.44483493	protein_coding	19	7599038	7626653	PNPLA6
ENSG00000032742	6.442380648	6.450406542	protein_coding	13	21140585	21265572	IFT88
ENSG00000033011	7.549958459	7.805153958	protein_coding	16	5083703	5135395	ALG1
ENSG00000033030	8.518728184	8.516036237	protein_coding	12	122957417	122985518	ZCCHC8
ENSG00000033050	10.65946972	10.29687419	protein_coding	7	150904923	150924316	ABCF2
ENSG00000033100	10.27908628	9.789702559	protein_coding	7	150929575	150935908	CHPF2
ENSG00000033122	1.16343121	1.518964905	protein_coding	1	70034081	70589164	LRRC7
ENSG00000033170	10.19968132	10.74250451	protein_coding	14	65877310	66210839	FUT8
ENSG00000033178	10.40492027	10.57085065	protein_coding	4	68481479	68566897	UBA6
ENSG00000033327	5.977159882	6.673373662	protein_coding	11	77926343	78129394	GAB2
ENSG00000033627	8.906929163	8.595894166	protein_coding	17	40610862	40674596	ATP6V0A1
ENSG00000033800	7.698004462	7.764467022	protein_coding	15	68346517	68483096	PIAS1
ENSG00000033867	8.981959173	8.686884751	protein_coding	3	27414214	27525911	SLC4A7
ENSG00000034063	11.07603452	10.84411243	protein_coding	19	4909510	4962162	UHRF1
ENSG00000034152	10.36749671	10.04925084	protein_coding	17	21187984	21218552	MAP2K3
ENSG00000034510	13.07484232	13.72817777	protein_coding	2	85132749	85133795	TMSB10
ENSG00000034533	5.986594255	6.044260771	protein_coding	3	130732719	130746493	ASTE1
ENSG00000034677	7.821758815	7.987679908	protein_coding	8	101269288	101348446	RNF19A
ENSG00000034693	7.599989788	8.229580833	protein_coding	6	143771918	143811753	PEX3
ENSG00000034713	8.799958495	8.725553248	protein_coding	16	75600249	75611779	GABARAPL2
ENSG00000035115	7.127924785	6.787424764	protein_coding	2	217730	266398	SH3YL1
ENSG00000035141	8.429884808	8.724493	protein_coding	2	70523107	70529222	FAM136A
ENSG00000035403	11.55941449	11.03430824	protein_coding	10	75757872	75879918	VCL
ENSG00000035499	7.530740159	7.741590246	protein_coding	5	59892739	59996017	DEPDC1B
ENSG00000035664	4.883502971	4.794098771	protein_coding	15	64199235	64364232	DAPK2
ENSG00000035681	8.842144812	9.116755954	protein_coding	8	59496063	59572403	NSMAF
ENSG00000035687	9.56636357	9.915351046	protein_coding	1	244571796	244615436	ADSS
ENSG00000035720	0	0.697730409	protein_coding	4	68424446	68473055	STAP1
ENSG00000035862	10.62061112	10.37834106	protein_coding	17	76849059	76921469	TIMP2
ENSG00000035928	10.08797732	10.1744918	protein_coding	4	39289076	39367995	RFC1
ENSG00000036054	9.094219895	9.232406246	protein_coding	3	99979844	100044095	TBC1D23
ENSG00000036257	9.856739382	9.548022979	protein_coding	2	225334867	225450110	CUL3
ENSG00000036473	0	0.390640832	protein_coding	X	38211798	38280703	OTC
ENSG00000036530	2.35979773	2.144285137	protein_coding	14	100150641	100193638	CYP46A1
ENSG00000036549	8.89695732	9.038718229	protein_coding	1	78028101	78149104	ZZZ3
ENSG00000036672	6.313367288	5.871116342	protein_coding	11	119225925	119252436	USP2
ENSG00000037042	7.049074049	7.507697912	protein_coding	17	40811266	40819021	TUBG2
ENSG00000037241	6.766474304	7.600774083	protein_coding	5	172385732	172396774	RPL26L1

ENSG00000037280	0.869158192	0.950786998	protein_coding	5	180028506	180076624	FLT4
ENSG00000037474	11.39112882	11.02852092	protein_coding	5	6599352	6633404	NSUN2
ENSG00000037637	8.072698871	8.213044874	protein_coding	1	16573334	16678949	FBXO42
ENSG00000037749	8.599344916	8.461080043	protein_coding	5	153418466	153600038	MFAP3
ENSG00000037757	8.107705799	8.11126287	protein_coding	19	13875337	13885096	MRI1
ENSG00000037897	8.177331204	8.25035614	protein_coding	12	58162254	58166576	METTL1
ENSG00000037965	4.409500985	4.710890357	protein_coding	12	54402832	54407570	HOXC8
ENSG00000038002	7.250461488	8.432796436	protein_coding	4	178351924	178363657	AGA
ENSG00000038210	8.513842764	8.810943783	protein_coding	4	25162263	25280714	PI4K2B
ENSG00000038219	8.990190269	9.198444985	protein_coding	4	13570362	13629347	BOD1L
ENSG00000038274	8.546104025	9.031020259	protein_coding	5	162930120	162946342	MAT2B
ENSG00000038295	0.869158192	0.390640832	protein_coding	4	166794410	167025047	TLL1
ENSG00000038358	10.33139226	10.29151462	protein_coding	16	67906975	67918406	EDC4
ENSG00000038382	12.38774818	12.04600302	protein_coding	5	14143811	14532235	TRIO
ENSG00000038427	12.13456961	11.83711343	protein_coding	5	82767284	82878122	VCAN
ENSG00000038532	9.044272373	8.864865519	protein_coding	16	11038345	11276046	CLEC16A
ENSG00000039068	9.750685625	2.242360793	protein_coding	16	68771128	68869445	CDH1
ENSG00000039123	9.479658457	9.377739515	protein_coding	5	54603588	54721409	SKIV2L2
ENSG00000039139	8.045874708	7.373448694	protein_coding	5	13690440	13944652	DNAH5
ENSG00000039319	8.605481314	8.84060518	protein_coding	5	79703832	79775169	ZFYVE16
ENSG00000039523	10.58456506	10.23902354	protein_coding	16	67552321	67580689	FAM65A
ENSG00000039560	10.97627036	10.09795979	protein_coding	5	34656342	34832732	RAI14
ENSG00000039600	0.499066092	1.16600992	protein_coding	5	157052687	157098488	SOX30
ENSG00000039650	8.255505039	8.240005883	protein_coding	19	50364462	50370818	PNKP
ENSG00000039987	1.16343121	1.802319292	protein_coding	19	12862516	12869272	BEST2
ENSG00000040199	7.686472517	7.641787375	protein_coding	16	71671738	71758604	PHLPP2
ENSG00000040275	8.623735441	8.957119685	protein_coding	5	169010638	169031782	CCDC99
ENSG00000040341	7.250461488	7.03670965	protein_coding	8	74332604	74659943	STAU2
ENSG00000040487	7.035502862	7.505230566	protein_coding	1	19638820	19655794	PQLC2
ENSG00000040531	8.554057865	9.303001007	protein_coding	17	3539762	3564836	CTNS
ENSG00000040608	7.106450572	6.762847836	protein_coding	22	20228938	20270769	RTN4R
ENSG00000040633	9.561637084	10.24421147	protein_coding	17	7138350	7142825	PHF23
ENSG00000040933	8.507303029	8.241489045	protein_coding	2	99061317	99207496	INPP4A
ENSG00000041353	7.952677993	7.41347308	protein_coding	18	52495708	52562747	RAB27B
ENSG00000041357	9.847708073	10.34423495	protein_coding	15	78832747	78841604	PSMA4
ENSG00000041515	0.499066092	0	protein_coding	13	109248500	109860355	MYO16
ENSG00000041802	9.354083505	9.295880847	protein_coding	3	194361517	194393206	LSG1
ENSG00000041880	7.169937448	7.84667518	protein_coding	3	51976361	51982883	PARP3
ENSG00000041982	11.8309055	13.1220881	protein_coding	9	117782806	117880536	TNC
ENSG00000041988	6.343050432	6.988061092	protein_coding	1	6684926	6695646	THAP3
ENSG00000042062	7.803191723	7.472765069	protein_coding	20	49202645	49308065	FAM65C
ENSG00000042088	8.991362316	8.576046394	protein_coding	14	90421283	90511106	TDP1
ENSG00000042286	8.649206367	7.975253757	protein_coding	10	71857979	71892690	AIFM2
ENSG00000042317	6.646617938	6.360445592	protein_coding	14	88851268	88936694	SPATA7
ENSG00000042429	8.278730502	8.75700367	protein_coding	11	93517393	93547861	MED17
ENSG00000042445	9.53214319	9.684588223	protein_coding	2	85569211	85581743	RETSAT
ENSG00000042493	9.767886	9.702468294	protein_coding	2	85621346	85645555	CAPG
ENSG00000042753	9.878440025	10.46849361	protein_coding	19	47341423	47354203	AP2S1
ENSG00000042781	1.16343121	3.368666104	protein_coding	1	215796236	216596738	USH2A
ENSG00000042832	0	0.390640832	protein_coding	8	133879203	134147147	TG
ENSG00000042980	2.35979773	3.232099092	protein_coding	8	24151553	24216531	ADAM28
ENSG00000043039	5.175139844	3.714580548	protein_coding	11	129245835	129322171	BARX2
ENSG00000043093	8.876804446	8.617758557	protein_coding	3	182655862	182703741	DCUN1D1
ENSG00000043143	9.092036341	7.500283181	protein_coding	5	133860003	133918918	PHF15
ENSG00000043355	8.016241092	7.416102302	protein_coding	13	100634026	100639018	ZIC2
ENSG00000043514	7.471499737	7.515074729	protein_coding	1	40306708	40349183	TRIT1
ENSG00000043591	3.211941663	1.925536307	protein_coding	10	115803806	115806667	ADRB1
ENSG00000044090	9.456930686	10.21582146	protein_coding	6	43005355	43021683	CUL7
ENSG00000044115	11.72444875	11.59480528	protein_coding	5	137946656	138270723	CTNNA1
ENSG00000044446	8.53006357	8.581912372	protein_coding	X	18910418	19002716	PHKA2
ENSG00000044459	5.60078458	5.832272499	protein_coding	9	17134980	17503921	CNTLN
ENSG00000044574	13.18950907	13.24270751	protein_coding	9	127997132	128003622	HSPA5
ENSG00000046604	11.10826314	10.47039222	protein_coding	18	29078027	29128813	DSG2

ENSG00000046647	6.204385613	6.568558581	protein_coding	X	14026398	14048012	GEMIN8
ENSG00000046651	7.60612345	8.049948078	protein_coding	X	13752832	13787480	OFD1
ENSG00000046653	3.004694206	3.133298822	protein_coding	X	13789150	13956757	GPM6B
ENSG00000046774	0	0.390640832	protein_coding	X	141290131	141293076	MAGEC2
ENSG00000047056	7.537174746	7.154756319	protein_coding	10	1095478	1178237	WDR37
ENSG00000047188	9.086562954	8.869669013	protein_coding	5	112849380	112930982	YTHDC2
ENSG00000047230	7.933293789	7.683850422	protein_coding	X	16606126	16731059	CTPS2
ENSG00000047249	8.45218826	8.439273392	protein_coding	8	54628117	54756118	ATP6V1H
ENSG00000047315	10.34199724	10.72882263	protein_coding	4	57843888	57897334	POLR2B
ENSG00000047346	6.658471572	7.0637896	protein_coding	15	52873514	53002014	FAM214A
ENSG00000047365	5.346128795	5.004391715	protein_coding	4	36067620	36246131	ARAP2
ENSG00000047410	10.32350339	10.66421414	protein_coding	1	186282954	186344825	TPR
ENSG00000047457	5.191549282	3.32456471	protein_coding	3	148880197	148939842	CP
ENSG00000047578	8.693086466	8.265014588	protein_coding	16	27561454	27791690	KIAA0556
ENSG00000047579	6.721980839	7.309836726	protein_coding	6	15523032	15663289	DTNBP1
ENSG00000047621	7.765324926	7.992972811	protein_coding	12	4596894	4647674	C12orf4
ENSG00000047634	8.431612753	8.98660512	protein_coding	X	17755588	17773105	SCML1
ENSG00000047644	9.837316871	9.618206363	protein_coding	X	9983602	10112518	WWC3
ENSG00000047648	0.499066092	1.802319292	protein_coding	X	11136239	11683821	ARHGAP6
ENSG00000047662	5.977159882	5.855704078	protein_coding	4	17630929	17783135	FAM184B
ENSG00000047849	12.41802582	12.22628546	protein_coding	3	47892182	48130769	MAP4
ENSG00000047932	8.652173596	8.689060408	protein_coding	6	117639374	117923691	GOPC
ENSG00000047936	5.588445295	4.810179682	protein_coding	6	117609463	117747018	ROS1
ENSG00000048028	9.123378905	9.222677059	protein_coding	11	113668596	113746292	USP28
ENSG00000048052	8.748205354	9.112709611	protein_coding	7	18126572	19036993	HDAC9
ENSG00000048140	9.332973914	9.291591823	protein_coding	5	176074388	176086058	TSPAN17
ENSG00000048162	8.105542576	8.542748725	protein_coding	5	175810949	175815976	NOP16
ENSG00000048342	7.182306324	6.926693689	protein_coding	4	15471489	15603180	CC2D2A
ENSG00000048392	7.584540566	7.811159227	protein_coding	8	103216730	103251346	RRM2B
ENSG00000048405	7.80585884	7.551403417	protein_coding	7	126986844	127071978	ZNF800
ENSG00000048462	0	0.697730409	protein_coding	16	12058964	12061925	TNFRSF17
ENSG00000048471	8.114176072	7.989446368	protein_coding	16	12070594	12668146	SNX29
ENSG00000048540	2.471521042	2.579085888	protein_coding	12	16701307	16763528	LMO3
ENSG00000048544	9.225519491	9.069105817	protein_coding	6	42174539	42185603	MRPS10
ENSG00000048545	0	0.390640832	protein_coding	6	42123144	42147794	GUCA1A
ENSG00000048649	9.142495894	9.329044212	protein_coding	11	77371041	77532063	RSF1
ENSG00000048707	8.909411391	8.729786462	protein_coding	1	12290124	12572099	VPS13D
ENSG00000048740	0.869158192	0.950786998	protein_coding	10	11047259	11378666	CELF2
ENSG00000048828	12.18127881	11.74548469	protein_coding	9	96214173	96328397	FAM120A
ENSG00000048991	10.1300883	9.659843595	protein_coding	2	136289025	136482840	R3HDM1
ENSG00000049089	4.261916566	4.319727133	protein_coding	1	40766159	40783488	COL9A2
ENSG00000049130	8.114176072	6.729413443	protein_coding	12	88885885	88974628	KITLG
ENSG00000049167	6.693459083	6.592001111	protein_coding	5	60169658	60240900	ERCC8
ENSG00000049192	6.994006093	7.318294738	protein_coding	5	64444563	64777747	ADAMTS6
ENSG00000049239	10.16634253	10.20521953	protein_coding	1	9294863	9331396	H6PD
ENSG00000049245	9.790280964	9.582371442	protein_coding	1	7831329	7841492	VAMP3
ENSG00000049246	6.793601635	6.110690132	protein_coding	1	7844380	7905237	PER3
ENSG00000049249	8.185546801	8.022604578	protein_coding	1	7979907	8000926	TNFRSF9
ENSG00000049283	6.379315619	1.802319292	protein_coding	17	48609904	48621111	EPN3
ENSG00000049319	1.616589159	0.390640832	processed_transc	2	31747550	31806136	SRD5A2
ENSG00000049323	8.12275821	9.099684516	protein_coding	2	33172039	33624576	LTBP1
ENSG00000049449	11.15346388	11.28087678	protein_coding	11	31833939	32127301	RCN1
ENSG00000049540	1.616589159	2.788380093	protein_coding	7	73442119	73484237	ELN
ENSG00000049541	8.993703557	8.810943783	protein_coding	7	73645829	73668774	RFC2
ENSG00000049618	9.490475063	9.528108569	protein_coding	6	157099063	157530401	ARID1B
ENSG00000049656	11.56572272	11.22236782	protein_coding	5	1317859	1345214	CLPTM1L
ENSG00000049759	10.52105974	10.59918141	protein_coding	18	55711619	56068772	NEDD4L
ENSG00000049768	3.652182994	3.714580548	protein_coding	X	49106897	49121288	FOXP3
ENSG00000049769	7.169937448	7.807158493	protein_coding	X	49126306	49157929	PPP1R3F
ENSG00000049860	10.02241241	10.41100059	protein_coding	5	73935848	74018472	HEXB
ENSG00000049883	6.228441838	6.051043053	protein_coding	5	71616194	71656052	PTCD2
ENSG00000050130	9.237422823	9.484933332	protein_coding	14	59951161	59971429	JKAMP
ENSG00000050165	1.616589159	0.697730409	protein_coding	11	11984653	12031316	DKK3

ENSG00000050327	6.877132514	5.66550919	protein_coding	7	144052381	144077725	ARHGEF5
ENSG00000050344	11.12971059	10.40505707	protein_coding	7	26191860	26226745	NFE2L3
ENSG00000050393	8.061582684	8.303864205	protein_coding	6	13791020	13814800	CCDC90A
ENSG00000050405	10.94787535	10.80377176	protein_coding	12	50569571	50677353	LIMA1
ENSG00000050426	9.920270706	9.950689189	protein_coding	12	51441745	51454207	LETMD1
ENSG00000050438	2.35979773	3.027231696	protein_coding	12	51785101	51902980	SLC4A8
ENSG00000050555	0.869158192	0.390640832	protein_coding	9	133884469	133969860	LAMC3
ENSG00000050628	0	1.16600992	protein_coding	1	71318036	71513491	PTGER3
ENSG00000050730	7.066972635	7.477807561	protein_coding	4	122052563	122148621	TNIP3
ENSG00000050748	8.728660495	8.480051236	protein_coding	5	179660143	179719099	MAPK9
ENSG00000050767	0.499066092	1.16600992	protein_coding	5	177664619	178017556	COL23A1
ENSG00000050820	10.60958628	10.2319528	protein_coding	16	75262928	75301951	BCAR1
ENSG00000051009	8.525216449	8.715982854	protein_coding	11	6232565	6255941	FAM160A2
ENSG00000051108	9.915339294	10.26003019	protein_coding	16	56965960	56977798	HERPUD1
ENSG00000051128	9.843820138	9.799804744	protein_coding	19	19040010	19052041	HOMER3
ENSG00000051180	7.945439403	8.0397549	protein_coding	15	40986972	41024356	RAD51
ENSG00000051341	7.923503136	8.027771115	protein_coding	3	121150274	121265488	POLQ
ENSG00000051382	8.8704485	8.490068116	protein_coding	3	138372860	138553780	PIK3CB
ENSG00000051523	10.605765	10.77051277	protein_coding	16	88709691	88717560	CYBA
ENSG00000051596	6.023726639	6.270323763	protein_coding	5	175344876	175461683	THOC3
ENSG00000051620	7.983632192	8.458531597	protein_coding	6	138724668	138734310	HEBP2
ENSG00000051825	8.011627561	8.130601896	protein_coding	12	123636867	123728561	MPHOSPH9
ENSG00000052126	8.124895791	7.475288518	protein_coding	12	19282648	19529334	PLEKHA5
ENSG00000052344	6.846372398	3.232099092	protein_coding	16	31142756	31147083	PRSS8
ENSG00000052723	7.959880444	8.013952351	protein_coding	1	115312100	115323306	SIKE1
ENSG00000052749	10.34612589	10.46119237	protein_coding	10	99116115	99161127	RRP12
ENSG00000052795	6.699208722	7.801136513	protein_coding	4	159690290	159829201	FNIP2
ENSG00000052802	9.143550557	7.784953917	protein_coding	4	166248775	166264312	MSMO1
ENSG00000052841	9.309672858	9.906496457	protein_coding	11	43380482	43516483	TTC17
ENSG00000052850	1.16343121	2.039052734	protein_coding	11	44281994	44331716	ALX4
ENSG00000053108	0.869158192	0	protein_coding	5	132532147	132948255	FSTL4
ENSG00000053254	9.106170722	8.913126522	protein_coding	14	89591215	90085493	FOXN3
ENSG00000053371	8.185546801	8.591248608	protein_coding	1	19630459	19638640	AKR7A2
ENSG00000053372	9.38294884	9.369623887	protein_coding	1	19578033	19586622	MRTO4
ENSG00000053438	3.274897671	3.532712221	protein_coding	20	36149617	36152092	NNAT
ENSG00000053501	8.3480754	8.715982854	protein_coding	19	17326155	17330638	USE1
ENSG00000053524	5.207774174	5.112089758	protein_coding	3	182895831	183146566	MCF2L2
ENSG00000053702	4.352265886	3.906303962	protein_coding	12	2934514	2944710	NRIP2
ENSG00000053747	8.893200037	5.393992	protein_coding	18	21269562	21535030	LAMA3
ENSG00000053770	8.864064429	9.173021923	protein_coding	14	57735627	57756797	MUDENG
ENSG00000053900	7.754320745	7.962719647	protein_coding	4	25378835	25420120	ANAPC4
ENSG00000053918	4.19837882	3.411459265	protein_coding	11	2465914	2870221	KCNQ1
ENSG00000054116	8.797280438	8.782698567	protein_coding	1	36602173	36615098	TRAPPC3
ENSG00000054118	10.82474139	10.76742745	protein_coding	1	36690017	36770958	THRAP3
ENSG00000054148	9.088754802	9.392500624	protein_coding	9	139743176	139745488	PHPT1
ENSG00000054179	3.448790144	1.518964905	protein_coding	9	139942550	139948497	ENTPD2
ENSG00000054219	0.499066092	0.697730409	protein_coding	2	160628362	160761260	LY75
ENSG00000054267	8.640267888	8.683615101	protein_coding	1	235294949	235491534	ARID4B
ENSG00000054277	6.861834435	7.04352744	protein_coding	1	241753404	241840678	OPN3
ENSG00000054282	7.33043718	7.67728988	protein_coding	1	243419320	243663394	SDCCAG8
ENSG00000054356	1.16343121	2.242360793	protein_coding	2	220154345	220174370	PTPRN
ENSG00000054392	3.652182994	3.570966319	protein_coding	1	210501596	210849638	HHAT
ENSG00000054523	9.94104316	9.834615514	protein_coding	1	10270764	10441661	KIF1B
ENSG00000054598	8.048129203	8.522150834	protein_coding	6	1610679	1614127	FOXC1
ENSG00000054611	8.984315711	9.275755399	protein_coding	22	47158518	47571336	TBC1D22A
ENSG00000054654	9.030655728	9.616492755	protein_coding	14	64319683	64693165	SYNE2
ENSG00000054690	8.546104025	8.711708882	protein_coding	14	68000018	68056329	PLEKHH1
ENSG00000054793	9.276405902	8.797898671	protein_coding	20	50213053	50385173	ATP9A
ENSG00000054938	2.238690726	2.144285137	protein_coding	11	74407474	74442430	CHRDL2
ENSG00000054965	9.643686274	9.583541409	protein_coding	11	73111532	73309234	FAM168A
ENSG00000054967	7.80585884	8.090015513	protein_coding	11	73087309	73108519	RELT
ENSG00000054983	9.013452178	9.056522014	protein_coding	14	88304164	88460009	GALC
ENSG00000055044	10.36839913	10.37631728	protein_coding	2	203130439	203168389	NOP58

ENSG00000055070	10.15174033	10.00671086	protein_coding	1	16679070	16724640	C1orf144
ENSG00000055118	3.274897671	2.501982735	protein_coding	7	150642049	150675403	KCNH2
ENSG00000055130	10.22630637	10.1024558	protein_coding	7	148395006	148498201	CUL1
ENSG00000055147	7.190493718	7.378849871	protein_coding	5	153369688	153418496	FAM114A2
ENSG00000055163	1.407729925	0.950786998	protein_coding	5	156693089	156822606	CYFIP2
ENSG00000055208	9.796328827	9.610765985	protein_coding	6	149539777	149732749	TAB2
ENSG00000055211	7.792473675	7.946442679	protein_coding	6	149887410	149912884	C6orf72
ENSG00000055332	10.19714397	10.78736503	protein_coding	2	37326353	37384208	EIF2AK2
ENSG00000055483	9.427791605	9.081580806	protein_coding	17	76792965	76836969	USP36
ENSG00000055609	9.545769354	9.093948849	protein_coding	7	151832010	152133090	MLL3
ENSG00000055732	5.090170488	4.363974406	protein_coding	1	85483765	85514182	MCOLN3
ENSG00000055813	0	0.390640832	protein_coding	2	56411258	56613308	CCDC85A
ENSG00000055917	10.21431058	9.91813598	protein_coding	2	20448452	20551995	PUM2
ENSG00000055950	8.846037259	9.246508835	protein_coding	10	102729215	102747272	MRPL43
ENSG00000055955	1.16343121	0.697730409	protein_coding	3	52846991	52865495	ITIH4
ENSG00000056050	7.04004079	7.703354924	protein_coding	4	170650616	170679104	C4orf27
ENSG00000056097	10.41587333	10.59599647	protein_coding	5	32354456	32444867	ZFR
ENSG00000056277	7.345180517	7.195117037	protein_coding	X	129336682	129402873	ZNF280C
ENSG00000056487	0.499066092	0	protein_coding	22	45277042	45405880	PHF21B
ENSG00000056558	9.532948302	10.69512212	protein_coding	9	123664671	123691451	TRAF1
ENSG00000056586	9.818289907	9.87244022	protein_coding	9	125606835	125667562	RC3H2
ENSG00000056661	7.660183591	7.348889996	protein_coding	17	36890150	36904558	PCGF2
ENSG00000056736	4.292665995	4.17820932	protein_coding	3	53880607	53899827	IL17RB
ENSG00000056972	8.325948295	8.262094789	protein_coding	6	111877657	111927481	TRAF3IP2
ENSG00000056998	0	0.390640832	protein_coding	X	2746829	2800859	GYG2
ENSG00000057019	11.49083588	11.25541	protein_coding	3	98514785	98620533	DCBLD2
ENSG00000057149	4.352265886	2.039052734	protein_coding	18	61322431	61329197	SERPINB3
ENSG00000057252	9.848355045	9.931061721	protein_coding	1	179262925	179327815	SOAT1
ENSG00000057294	6.96088487	6.2290532	protein_coding	12	32943679	33049774	PKP2
ENSG00000057468	0.499066092	0.950786998	protein_coding	1	76262567	76378923	MSH4
ENSG00000057593	0	0.390640832	protein_coding	13	113760105	113774995	F7
ENSG00000057608	11.2523464	10.99747635	protein_coding	10	5807186	5884095	GDI2
ENSG00000057657	3.989024711	2.334191469	protein_coding	6	106534195	106557814	PRDM1
ENSG00000057663	8.045874708	8.200898549	protein_coding	6	106632351	106773666	ATG5
ENSG00000057704	6.809636522	5.751299057	protein_coding	12	94960900	95044338	TMCC3
ENSG00000057757	8.457286611	8.563056668	protein_coding	1	24104895	24114722	PITHD1
ENSG00000057935	8.096857104	8.143352046	protein_coding	2	42721709	42984087	MTA3
ENSG00000058056	8.635777795	8.426290271	protein_coding	3	179370543	179507189	USP13
ENSG00000058063	9.895443335	9.870524428	protein_coding	3	182511288	182639423	ATP11B
ENSG00000058085	10.17410476	8.436686098	protein_coding	1	183155373	183214035	LAMC2
ENSG00000058091	6.970425921	7.449853178	protein_coding	7	90095738	90839905	CDK14
ENSG00000058262	12.40048713	12.27670419	protein_coding	3	127770484	127790526	SEC61A1
ENSG00000058272	8.900704842	8.935290787	protein_coding	12	80167343	80329240	PPP1R12A
ENSG00000058335	3.335220907	2.579085888	protein_coding	15	79252289	79383215	RASGRF1
ENSG00000058404	1.799000381	2.420525079	protein_coding	7	44256749	44374176	CAMK2B
ENSG00000058453	7.91858278	7.856387513	protein_coding	1	17222232	17299459	CROCC
ENSG00000058600	9.498740691	9.707843036	protein_coding	16	22308730	22346424	POLR3E
ENSG00000058668	10.5736515	9.61477711	protein_coding	1	203595689	203713209	ATP2B4
ENSG00000058673	10.09562678	10.18532111	protein_coding	1	203764782	203823252	ZC3H11A
ENSG00000058729	7.726437377	7.778838337	protein_coding	5	96496571	96518964	RIOK2
ENSG00000058799	7.612231145	7.696882678	protein_coding	1	54317392	54356407	YIPF1
ENSG00000058804	9.327415887	9.244291255	protein_coding	1	54231133	54304533	TMEM48
ENSG00000058866	6.721980839	6.360445592	protein_coding	3	185823457	186080026	DGKG
ENSG00000059122	9.71423818	9.410412513	protein_coding	16	2961980	3001208	FLYWCH1
ENSG00000059145	8.215945216	8.18248514	protein_coding	16	1413206	1464752	UNKL
ENSG00000059377	4.19837882	4.202780776	protein_coding	7	139476850	139720125	TBXAS1
ENSG00000059378	9.465390183	9.460942741	protein_coding	7	139723544	139763521	PARP12
ENSG00000059573	10.73313074	10.84849884	protein_coding	10	97365696	97416463	ALDH18A1
ENSG00000059588	8.646233023	9.108651887	protein_coding	1	234527059	234614849	TARBP1
ENSG00000059691	8.688758029	9.472987904	protein_coding	4	152591656	152682175	PET112
ENSG00000059728	7.82964357	7.577474828	protein_coding	2	70124820	70170077	MXD1
ENSG00000059758	8.12703021	7.91704449	protein_coding	12	96672039	96794338	CDK17
ENSG00000059769	7.549958459	7.922602475	protein_coding	9	114393632	114432508	DNAJC25

ENSG00000059804	8.973680846	8.46235258	protein_coding	12	8071826	8088871	SLC2A3
ENSG00000059915	3.830412367	3.532712221	protein_coding	10	104162376	104181296	PSD
ENSG00000060069	8.364450825	8.366375472	protein_coding	18	77439801	77514510	CTDP1
ENSG00000060138	12.01172959	11.44853179	protein_coding	12	10851683	10875911	CSDA
ENSG00000060140	5.286269488	4.021284656	protein_coding	12	10771538	10826917	STYK1
ENSG00000060237	11.50198055	11.0422015	protein_coding	12	861759	1020618	WNK1
ENSG00000060303	0.499066092	0.390640832	pseudogene	6	50824968	50825375	RPS17P5
ENSG00000060339	9.364975797	9.526284462	protein_coding	10	70480769	70552134	CCAR1
ENSG00000060491	9.901690348	10.00234331	protein_coding	20	61436187	61445352	OGFR
ENSG00000060558	9.737474801	9.071609459	protein_coding	19	3136191	3163766	GNA15
ENSG00000060566	5.806547963	2.652276565	protein_coding	19	4153629	4173050	CREB3L3
ENSG00000060642	7.23866569	7.365308824	protein_coding	1	27113963	27124889	PIGV
ENSG00000060656	8.280649185	7.016060809	protein_coding	1	29563028	29653325	PTPRU
ENSG00000060688	9.037480115	9.066597822	protein_coding	1	31732417	31769662	SNRNP40
ENSG00000060709	0.499066092	0.697730409	protein_coding	12	130880682	131200826	RIMBP2
ENSG00000060718	0.499066092	0	protein_coding	1	103342023	103574052	COL11A1
ENSG00000060749	9.985245743	10.12150977	protein_coding	11	32914724	33014862	QSER1
ENSG00000060762	7.37062428	8.202422444	protein_coding	6	166778407	166796486	BRP44L
ENSG00000060971	7.990682129	8.099860884	protein_coding	3	38164201	38178733	ACAA1
ENSG00000060982	11.18760954	10.92871239	protein_coding	12	24964295	25102393	BCAT1
ENSG00000061273	9.801683503	10.0214624	protein_coding	12	48176505	48226915	HDAC7
ENSG00000061337	1.960915222	1.16600992	protein_coding	8	20103676	20161474	LZTS1
ENSG00000061455	0.869158192	1.353254395	protein_coding	5	122424816	122523745	PRDM6
ENSG00000061656	7.596913151	8.443145654	protein_coding	20	34203814	34208971	SPAG4
ENSG00000061676	11.18120429	10.99901167	protein_coding	2	183789605	183903586	NCKAP1
ENSG00000061794	9.538571537	9.562335454	protein_coding	12	27863706	27909228	MRPS35
ENSG00000061918	3.950976309	6.934050347	protein_coding	4	156680144	156728743	GUCY1B3
ENSG00000061936	9.258993749	9.063247032	protein_coding	12	132195626	132284282	SFSWAP
ENSG00000061938	9.018059887	8.565427152	protein_coding	3	195590235	195638816	TNK2
ENSG00000061987	8.674235392	8.899098923	protein_coding	12	62860597	62991363	MON2
ENSG00000062038	10.21380859	6.668971382	protein_coding	16	68670092	68756519	CDH3
ENSG00000062194	9.441577455	9.290875744	protein_coding	5	56469775	56560505	GPBP1
ENSG00000062282	8.059349131	8.552341023	protein_coding	11	75470557	75512579	DGAT2
ENSG00000062370	6.798966424	7.329495501	protein_coding	19	44830706	44905774	ZFP112
ENSG00000062485	11.86103218	11.72932276	protein_coding	12	56665483	56694176	CS
ENSG00000062524	1.407729925	0.950786998	protein_coding	15	41795783	41806085	LTK
ENSG00000062582	11.16441632	11.37998908	protein_coding	7	43906157	43909492	MRPS24
ENSG00000062598	8.744039402	8.702045897	protein_coding	20	44994688	45061704	ELMO2
ENSG00000062650	9.791627126	9.848794614	protein_coding	10	88195013	88281572	WAPAL
ENSG00000062716	10.28483328	10.7926861	protein_coding	17	57784863	57917950	VMP1
ENSG00000062725	8.382430368	8.622319665	protein_coding	17	58520520	58603580	APPBP2
ENSG00000062822	9.842521827	9.943872684	protein_coding	19	50887593	50921270	POLD1
ENSG00000063015	1.616589159	2.334191469	protein_coding	17	27281919	27333458	SEZ6
ENSG00000063046	11.51058949	11.52176793	protein_coding	12	53399942	53435993	EIF4B
ENSG00000063127	5.158541613	4.976162367	protein_coding	19	49792897	49828474	SLC6A16
ENSG00000063169	8.362640485	7.95731437	protein_coding	19	48111453	48206534	GLTSCR1
ENSG00000063176	7.978913023	8.044860491	protein_coding	19	49122548	49133973	SPHK2
ENSG00000063177	12.04738372	12.46725444	protein_coding	19	49118585	49122793	RPL18
ENSG00000063180	5.331395195	6.673373662	protein_coding	19	49141272	49149451	CA11
ENSG00000063241	9.733978068	9.411071665	protein_coding	19	55964345	55973049	ISOC2
ENSG00000063244	11.77840425	11.66127838	protein_coding	19	56165416	56186081	U2AF2
ENSG00000063245	11.89195533	11.39605971	protein_coding	19	56186561	56207132	EPN1
ENSG00000063322	7.04004079	6.977419495	protein_coding	19	39881963	39891201	MED29
ENSG00000063438	7.08025234	7.724721747	protein_coding	5	304291	438406	AHRR
ENSG00000063587	8.265227794	8.450859136	protein_coding	X	152599613	152618384	ZNF275
ENSG00000063601	9.142495894	8.977733605	protein_coding	X	149861435	149933576	MTMR1
ENSG00000063660	11.24007313	9.43525094	protein_coding	2	241375088	241407493	GPC1
ENSG00000063761	7.781675404	7.971683681	protein_coding	14	78266426	78401355	ADCK1
ENSG00000063854	7.865879816	8.480051236	protein_coding	16	1845621	1877195	HAGH
ENSG00000063978	9.577332188	9.570029165	protein_coding	4	2463947	2627047	RNF4
ENSG00000064012	8.774313977	8.689060408	protein_coding	2	202098166	202152434	CASP8
ENSG00000064042	4.6658819	5.42565083	protein_coding	4	41361624	41702061	LIMCH1
ENSG00000064102	9.530531618	9.373010948	protein_coding	12	27058114	27091259	ASUN

ENSG00000064115	9.05552222	8.797898671	protein_coding	12	27126128	27167367	TM7SF3
ENSG00000064195	6.320845526	3.368666104	protein_coding	17	48067369	48072588	DLX3
ENSG00000064199	5.538004898	5.759604001	protein_coding	11	124543694	124567414	SPA17
ENSG00000064201	2.928422289	3.32456471	protein_coding	11	2323227	2339430	TSPAN32
ENSG00000064205	6.163378456	6.24096575	protein_coding	20	43343485	43357150	WISP2
ENSG00000064225	2.762599152	4.048652948	protein_coding	3	98451080	98540045	ST3GAL6
ENSG00000064270	4.883502971	3.027231696	protein_coding	16	84402133	84497793	ATP2C2
ENSG00000064300	3.830412367	0.697730409	protein_coding	17	47572655	47592379	NGFR
ENSG00000064309	6.469548347	7.351639476	protein_coding	11	125825691	125933230	CDON
ENSG00000064313	9.021506037	8.591248608	protein_coding	8	120743015	120845103	TAF2
ENSG00000064393	10.4228399	9.757928543	protein_coding	7	139246316	139477577	HIPK2
ENSG00000064419	10.04854664	9.570029165	protein_coding	7	128594948	128695198	TNPO3
ENSG00000064490	8.954578658	9.111898978	protein_coding	19	19303008	19312678	RFXANK
ENSG00000064545	9.799008649	10.06399506	protein_coding	19	19229978	19249275	TMEM161A
ENSG00000064547	8.257454839	6.637773454	protein_coding	19	19734468	19739739	LPAR2
ENSG00000064601	11.24548624	11.83195402	protein_coding	20	44519591	44527459	CTSA
ENSG00000064607	10.37918427	10.53924152	protein_coding	19	19101697	19144380	SUGP2
ENSG00000064651	7.797842652	7.786986696	protein_coding	5	127419458	127525380	SLC12A2
ENSG00000064652	7.388527525	7.560938532	protein_coding	5	122179134	122365049	SNX24
ENSG00000064666	11.56336037	11.24620466	protein_coding	19	1026580	1039063	CNN2
ENSG00000064687	9.230491154	9.139208113	protein_coding	19	1040102	1065571	ABCA7
ENSG00000064692	0	0.697730409	protein_coding	5	121647049	121799914	SNCAIP
ENSG00000064703	7.832262274	8.24888207	protein_coding	1	112297867	112310638	DDX20
ENSG00000064726	9.402463961	9.537194665	protein_coding	15	83685174	83736106	BTBD1
ENSG00000064763	1.799000381	2.242360793	protein_coding	12	29302036	29493913	FAR2
ENSG00000064835	0	0.390640832	protein_coding	3	87308554	87325737	POU1F1
ENSG00000064886	6.421663777	7.237355663	protein_coding	1	111743393	111786062	CHI3L2
ENSG00000064932	11.85557193	11.55748408	protein_coding	19	1107634	1174282	SBNO2
ENSG00000064933	8.041355121	8.269383236	protein_coding	2	190648811	190742355	PMS1
ENSG00000064961	10.65983832	10.9641779	protein_coding	19	3572775	3579086	HMG20B
ENSG00000064989	2.35979773	1.802319292	protein_coding	2	188207856	188313187	CALCRL
ENSG00000064995	8.063812784	8.440565301	protein_coding	6	34845555	34855866	TAF11
ENSG00000064999	9.413872751	8.930701228	protein_coding	6	34857038	35085317	ANKS1A
ENSG00000065000	12.34792747	12.13357684	protein_coding	19	2100993	2151556	AP3D1
ENSG00000065029	8.708847212	8.890616425	protein_coding	6	35226686	35263762	ZNF76
ENSG00000065054	8.779750834	7.76240224	protein_coding	16	2075357	2089027	SLC9A3R2
ENSG00000065057	7.985986001	7.950075687	protein_coding	16	2089816	2097867	NTHL1
ENSG00000065060	9.687008864	9.815822531	protein_coding	6	34759857	34850915	UHRF1BP1
ENSG00000065135	9.469601403	9.695451059	protein_coding	1	110091233	110136975	GNAI3
ENSG00000065150	11.75978288	11.60981212	protein_coding	13	98605742	98676551	IPO5
ENSG00000065154	10.82079098	11.00163984	protein_coding	10	126085872	126107545	OAT
ENSG00000065183	9.536164262	9.516516751	protein_coding	1	118472343	118503049	WDR3
ENSG00000065243	9.122309386	9.031020259	protein_coding	1	89149905	89301938	PKN2
ENSG00000065268	9.95852752	9.839520617	protein_coding	19	984328	994569	WDR18
ENSG00000065308	9.842521827	10.16201509	protein_coding	6	52362200	52441713	TRAM2
ENSG00000065320	9.381161578	9.317839931	protein_coding	17	8924859	9147317	NTN1
ENSG00000065325	5.512107582	6.544728831	protein_coding	17	9728868	9795419	GLP2R
ENSG00000065328	9.24628631	9.028445114	protein_coding	10	13203554	13253104	MCM10
ENSG00000065357	8.169068555	6.941369681	protein_coding	12	56321103	56347811	DGKA
ENSG00000065361	7.115078652	3.532712221	protein_coding	12	56473641	56497289	ERBB3
ENSG00000065413	3.911897206	4.250702764	protein_coding	2	197831741	198175897	ANKRD44
ENSG00000065427	11.61369821	11.52298807	protein_coding	16	75661622	75682541	KARS
ENSG00000065457	8.092494681	7.722599249	protein_coding	16	75630879	75657198	ADAT1
ENSG00000065485	9.019209518	8.875412173	protein_coding	3	122785909	122944074	PDIA5
ENSG00000065491	8.116326397	8.257703981	protein_coding	6	37225548	37300746	TBC1D22B
ENSG00000065518	8.701704574	9.102951831	protein_coding	3	120315156	120321347	NDUFB4
ENSG00000065526	10.49278612	10.11302738	protein_coding	1	16174359	16266955	SPEN
ENSG00000065534	8.243750574	9.291591823	protein_coding	3	123328896	123603178	MYLK
ENSG00000065548	9.843171129	9.771851712	protein_coding	2	187350883	187374090	ZC3H15
ENSG00000065559	8.992533411	9.459031647	protein_coding	17	11924135	12047050	MAP2K4
ENSG00000065600	7.773523328	7.948260326	protein_coding	1	212537273	212588243	TMEM206
ENSG00000065613	9.968084123	10.05600976	protein_coding	10	105726959	105788991	SLK
ENSG00000065615	7.454602977	7.119700263	protein_coding	6	84569362	84670146	CYB5R4

ENSG00000065618	10.39299681	6.403479675	protein_coding	10	105791044	105845760	COL17A1
ENSG00000065621	7.587643674	6.445267191	protein_coding	10	106028631	106059616	GSTO2
ENSG00000065665	6.887241849	6.959507185	protein_coding	10	12171636	12211960	SEC61A2
ENSG00000065675	0.499066092	0	protein_coding	10	6469105	6622263	PRKCQ
ENSG00000065717	6.509364082	6.223059835	protein_coding	19	2997637	3045786	TLE2
ENSG00000065802	8.851210906	8.535512415	protein_coding	2	239335383	239360891	ASB1
ENSG00000065809	9.818950204	9.145559356	protein_coding	10	14560556	14816896	FAM107B
ENSG00000065833	9.25411951	8.715982854	protein_coding	6	83920108	84140797	ME1
ENSG00000065882	9.710684602	9.378413761	protein_coding	4	37892708	38140796	TBC1D1
ENSG00000065883	9.486324422	9.353254373	protein_coding	7	39989636	40136733	CDK13
ENSG00000065911	10.55197078	10.58172296	protein_coding	2	74425689	74444692	MTHFD2
ENSG00000065923	7.976547636	6.799557986	protein_coding	X	46464753	46618490	SLC9A7
ENSG00000065970	8.913126744	8.488819803	protein_coding	12	8185299	8208099	FOXJ2
ENSG00000065978	11.95908793	11.93443023	protein_coding	1	43148098	43168023	YBX1
ENSG00000065989	10.18592632	9.076603748	protein_coding	19	10527449	10580305	PDE4A
ENSG00000066027	7.997697782	7.819127564	protein_coding	1	212458782	212535200	PPP2R5A
ENSG00000066032	0	0.697730409	protein_coding	2	79412357	80875905	CTNNA2
ENSG00000066044	10.6929933	10.52621885	protein_coding	19	8023458	8070529	ELAVL1
ENSG00000066056	1.407729925	1.16600992	protein_coding	1	43766664	43788779	TIE1
ENSG00000066084	10.37739234	9.789195588	protein_coding	12	50898768	51142450	DIP2B
ENSG00000066117	10.09726068	9.80733523	protein_coding	12	50478755	50494495	SMARCD1
ENSG00000066135	8.600881464	8.70742221	protein_coding	1	44115829	44171186	KDM4A
ENSG00000066136	8.299696963	8.411872581	protein_coding	1	41157320	41237275	NFYC
ENSG00000066185	2.238690726	2.501982735	protein_coding	1	42896000	42921938	ZMYND12
ENSG00000066230	6.129712275	7.321103092	protein_coding	5	473425	524447	SLC9A3
ENSG00000066248	6.616550626	5.456629825	protein_coding	2	233743396	233877982	NGEF
ENSG00000066279	9.490475063	9.637486317	protein_coding	1	197053258	197115824	ASPM
ENSG00000066322	10.25830282	10.35352335	protein_coding	1	43829068	43833697	ELOVL1
ENSG00000066336	0.499066092	1.16600992	protein_coding	11	47376411	47400127	SPI1
ENSG00000066379	6.328285199	6.862599506	protein_coding	6	30026676	30032686	ZNRD1
ENSG00000066405	1.407729925	1.667587519	protein_coding	3	137717577	137752494	CLDN18
ENSG00000066422	8.717371959	8.381252713	protein_coding	3	101367733	101396339	ZBTB11
ENSG00000066427	7.521034145	6.57327766	protein_coding	14	92524896	92572965	ATXN3
ENSG00000066455	9.606690509	8.775550005	protein_coding	14	93260576	93306308	GOLGA5
ENSG00000066468	3.004694206	2.334191469	protein_coding	10	123237848	123357972	FGFR2
ENSG00000066557	7.549958459	7.799123587	protein_coding	1	70610488	70671303	LRRC40
ENSG00000066583	7.55947245	7.563312496	protein_coding	5	128430386	128449721	ISOC1
ENSG00000066629	8.320363082	7.465168131	protein_coding	14	100204030	100408397	EML1
ENSG00000066651	6.941611359	7.138927266	protein_coding	6	126307576	126360937	TRMT11
ENSG00000066654	9.25411951	9.373010948	protein_coding	16	20744986	20753406	THUMPD1
ENSG00000066697	8.730065371	9.02758571	protein_coding	9	103189438	103213511	C9orf30
ENSG00000066735	3.077135474	3.679994897	protein_coding	14	104605060	104647231	KIF26A
ENSG00000066739	9.285033801	8.543951256	protein_coding	14	96747595	96830207	ATG2B
ENSG00000066777	9.487986111	8.7455513	protein_coding	8	68085747	68255912	ARFGEF1
ENSG00000066827	7.246540262	7.093659051	protein_coding	8	135490031	135725292	ZFAT
ENSG00000066855	9.10941293	8.679244009	protein_coding	8	66556124	66683496	MTFR1
ENSG00000066923	5.512107582	5.306006045	protein_coding	7	99775186	99819111	STAG3
ENSG00000066926	7.993024472	7.9428005	protein_coding	18	55215515	55254004	FECH
ENSG00000066933	8.227927477	7.964516916	protein_coding	15	72114632	72410918	MYO9A
ENSG00000067048	0.499066092	0.697730409	protein_coding	Y	15016019	15032390	DDX3Y
ENSG00000067057	12.40324164	11.86948799	protein_coding	10	3109712	3179904	PFKP
ENSG00000067064	9.06334524	7.720473624	protein_coding	10	1085848	1095110	IDI1
ENSG00000067066	9.450127191	9.568848185	protein_coding	2	231280657	231408805	SP100
ENSG00000067082	12.23594806	10.83284173	protein_coding	10	3818188	3827473	KLF6
ENSG00000067113	6.155035276	6.136428444	protein_coding	5	54720682	54830878	PPAP2A
ENSG00000067141	8.741255403	8.987489278	protein_coding	15	73344051	73597547	NEO1
ENSG00000067167	9.803686393	9.759999733	protein_coding	8	71485677	71520622	TRAM1
ENSG00000067177	9.24628631	9.29015931	protein_coding	X	71798664	71934167	PHKA1
ENSG00000067182	10.47781173	9.881981209	protein_coding	12	6437923	6451280	TNFRSF1A
ENSG00000067191	7.491518048	7.198175438	protein_coding	17	37329709	37353902	CACNB1
ENSG00000067208	8.577659618	8.657187659	protein_coding	1	92974253	93257961	EVI5
ENSG00000067221	6.777386522	7.607691112	protein_coding	15	74275547	74286963	STOML1
ENSG00000067225	14.78403844	14.72602368	protein_coding	15	72491370	72524164	PKM2

ENSG00000067248	9.200400892	9.014632836	protein_coding	5	54552073	54603550	DHX29
ENSG00000067334	9.478823042	9.644229954	protein_coding	1	94333373	94345474	DNTTIP2
ENSG00000067365	7.803191723	7.909600377	protein_coding	16	8715540	8740081	METTL22
ENSG00000067369	9.372191905	9.126421125	protein_coding	15	43699407	43802926	TP53BP1
ENSG00000067445	0.499066092	0.697730409	protein_coding	X	54946895	54957864	TRO
ENSG00000067533	8.223944437	8.259169069	protein_coding	1	218458629	218511325	RRP15
ENSG00000067560	12.02180189	12.02720851	protein_coding	3	49396578	49450431	RHOA
ENSG00000067596	9.235445742	8.954409067	protein_coding	17	41561331	41604154	DHX8
ENSG00000067601	3.603959378	3.570966319	pseudogene	7	66752528	66767894	PMS2P4
ENSG00000067606	6.428602508	5.361622823	protein_coding	1	1981909	2116834	PRKCZ
ENSG00000067646	0.869158192	0	protein_coding	Y	2803112	2850547	ZFY
ENSG00000067704	11.08264807	11.20954246	protein_coding	1	220267444	220321380	IARS2
ENSG00000067715	5.869093502	5.059245468	protein_coding	12	79257773	79845788	SYT1
ENSG00000067798	8.422952257	8.534302826	protein_coding	12	78224685	78606790	NAV3
ENSG00000067829	9.962118669	10.14745308	protein_coding	X	153051221	153059978	IDH3G
ENSG00000067836	7.066972635	7.408200217	protein_coding	16	4846969	4852951	ROGDI
ENSG00000067840	0.869158192	0.390640832	protein_coding	X	153067621	153096020	PDZD4
ENSG00000067842	0	0.390640832	protein_coding	X	152783134	152848397	ATP2B3
ENSG00000067900	9.349520666	9.398495997	protein_coding	18	18529701	18691812	ROCK1
ENSG00000067955	9.449274494	9.303711094	protein_coding	16	67063019	67134961	CBFB
ENSG00000067992	6.922076877	7.41347308	protein_coding	X	24483338	24557954	PDK3
ENSG00000068001	8.173205795	8.076782959	protein_coding	3	50355221	50360337	HYAL2
ENSG00000068024	8.274885464	7.911465009	protein_coding	2	239969864	240323348	HDAC4
ENSG00000068028	7.933293789	8.394645624	protein_coding	3	50367219	50378411	RASSF1
ENSG00000068078	5.525114348	4.841813558	protein_coding	4	1795034	1810599	FGFR3
ENSG00000068079	8.364450825	9.444295206	protein_coding	17	41158742	41166473	IFI35
ENSG00000068097	8.753741308	8.950786969	protein_coding	17	58120562	58156292	HEATR6
ENSG00000068120	10.74499215	10.25600002	protein_coding	17	40714092	40718295	COASY
ENSG00000068137	8.414239457	7.644032061	protein_coding	17	40819935	40829048	PLEKHH3
ENSG00000068305	8.000028772	8.116121984	protein_coding	15	100017370	100256671	MEF2A
ENSG00000068308	9.836012692	9.893819392	protein_coding	X	48779305	48815648	OTUD5
ENSG00000068323	10.43581223	10.7182091	protein_coding	X	48886242	48901012	TFE3
ENSG00000068354	8.858936753	8.833738534	protein_coding	X	48397845	48420997	TBC1D25
ENSG00000068366	11.08895774	11.02206322	protein_coding	X	108872579	108976632	ACSL4
ENSG00000068383	7.64536895	7.307006333	protein_coding	10	134351324	134596984	INPP5A
ENSG00000068394	8.646233023	8.793860943	protein_coding	X	48970334	48980151	GPKOW
ENSG00000068400	8.880604615	9.178452256	protein_coding	X	48830134	48858675	GRIPAP1
ENSG00000068438	10.3350898	10.36275233	protein_coding	X	48334541	48344752	FTSJ1
ENSG00000068489	9.575770339	8.883034329	protein_coding	17	57233101	57279924	PRR11
ENSG00000068650	9.2686931	8.554729165	protein_coding	13	113344643	113541482	ATP11A
ENSG00000068654	10.89186426	10.7764079	protein_coding	2	86247339	86333278	POLR1A
ENSG00000068697	10.1782276	10.42640534	protein_coding	2	20232411	20251789	LAPTM4A
ENSG00000068724	9.479658457	8.859080135	protein_coding	2	47143296	47303276	TTC7A
ENSG00000068745	8.360827871	8.414504719	protein_coding	3	48725436	48777786	IP6K2
ENSG00000068784	7.194570056	7.655203404	protein_coding	2	45615819	45839304	SRBD1
ENSG00000068793	11.0436345	10.74093244	protein_coding	15	22892005	23006016	CYFIP1
ENSG00000068796	8.875535495	8.898158883	protein_coding	5	61601989	61833076	KIF2A
ENSG00000068831	1.799000381	1.353254395	protein_coding	11	64494383	64512928	RASGRP2
ENSG00000068878	10.46268027	10.28217725	protein_coding	2	54091204	54197977	PSME4
ENSG00000068885	6.814942123	7.012590419	protein_coding	3	159974774	160117668	IFT80
ENSG00000068903	7.985986001	8.492561507	protein_coding	19	39369197	39390502	SIRT2
ENSG00000068912	8.626755466	8.894392583	protein_coding	2	54014181	54045956	ERLEC1
ENSG00000068971	7.938164312	8.608592853	protein_coding	11	64685025	64701947	PPP2R5B
ENSG00000068976	3.274897671	2.652276565	protein_coding	11	64513861	64527769	PYGM
ENSG00000069011	9.342189974	8.564242397	protein_coding	5	134362615	134370503	PITX1
ENSG00000069020	7.416718478	6.948652068	protein_coding	5	65892176	66465423	MAST4
ENSG00000069122	2.238690726	0	protein_coding	6	46820249	46922680	GPR116
ENSG00000069188	1.616589159	1.518964905	protein_coding	17	71330523	71640227	SDK2
ENSG00000069248	9.565576897	9.748571216	protein_coding	1	229577044	229644103	NUP133
ENSG00000069275	12.01519088	11.80675339	protein_coding	1	205681947	205719404	NUCKS1
ENSG00000069329	10.45760098	10.90644604	protein_coding	16	46690819	46723430	VPS35
ENSG00000069345	9.544172939	9.8994674	protein_coding	16	46989299	47007699	DNAJA2
ENSG00000069399	10.48739661	10.38808288	protein_coding	19	45250962	45263301	BCL3

ENSG00000069424	8.697401955	8.799913305	protein_coding	1	6051526	6161253	KCNAB2
ENSG00000069431	2.671945279	2.721932731	protein_coding	12	21950335	22094336	ABCC9
ENSG00000069493	5.360713447	4.917990497	protein_coding	12	9809270	9848413	CLEC2D
ENSG00000069509	6.820228284	7.129345795	protein_coding	X	44382885	44402247	FUNDC1
ENSG00000069535	1.16343121	0.390640832	protein_coding	X	43625858	43741693	MAOB
ENSG00000069667	1.407729925	2.242360793	protein_coding	15	60780483	61521518	RORA
ENSG00000069696	3.78787645	2.721932731	protein_coding	11	637293	640706	DRD4
ENSG00000069702	7.049074049	6.287656023	protein_coding	1	92145902	92371892	TGFBR3
ENSG00000069712	4.734682531	4.990346085	protein_coding	1	92632542	92650280	KIAA1107
ENSG00000069812	5.91922012	4.127774132	protein_coding	1	6472478	6484730	HES2
ENSG00000069849	10.31557114	10.65784121	protein_coding	3	141594966	141645356	ATP1B3
ENSG00000069869	8.114176072	8.289597152	protein_coding	15	56119120	56285944	NEDD4
ENSG00000069943	6.733233509	6.495854971	protein_coding	15	55611158	55647846	PIGB
ENSG00000069956	9.104005195	9.040423325	protein_coding	15	52244303	52358462	MAPK6
ENSG00000069966	7.630400601	7.237355663	protein_coding	15	52413117	52483566	GNB5
ENSG00000069974	8.474152153	8.53672099	protein_coding	15	55495164	55611311	RAB27A
ENSG00000069998	8.560389515	8.38394126	protein_coding	22	17618401	17646177	CECR5
ENSG00000070010	9.956728588	10.27856981	protein_coding	22	19437464	19466738	UFD1L
ENSG00000070018	9.712107084	9.663720744	protein_coding	12	12268959	12419946	LRP6
ENSG00000070019	0.869158192	0.697730409	protein_coding	12	14765576	14849519	GUCY2C
ENSG00000070031	1.799000381	0.390640832	protein_coding	11	626431	627143	SCT
ENSG00000070047	9.622662143	9.329741599	protein_coding	11	576486	612222	PHRF1
ENSG00000070061	9.866992122	9.909298518	protein_coding	9	111629797	111696396	IKBKAP
ENSG00000070081	9.27062517	9.812832711	protein_coding	11	17229700	17371521	NUCB2
ENSG00000070087	8.646233023	8.765275934	protein_coding	3	149682691	149768575	PFN2
ENSG00000070159	8.179389493	7.225413142	protein_coding	9	112137974	112260593	PTPN3
ENSG00000070182	7.666067113	7.452416987	protein_coding	14	65213002	65346601	SPTB
ENSG00000070190	5.270908378	5.792353798	protein_coding	4	100737990	100791311	DAPP1
ENSG00000070214	10.25000281	9.496780665	protein_coding	9	108006903	108201452	SLC44A1
ENSG00000070269	7.30049066	8.026050991	protein_coding	14	56955072	57117324	C14orf101
ENSG00000070366	9.664408419	10.21242219	protein_coding	17	1963133	2207069	SMG6
ENSG00000070367	10.07973621	10.26696498	protein_coding	14	57670518	57735726	EXOC5
ENSG00000070371	7.68357507	7.95731437	protein_coding	22	19166986	19279239	CLTCL1
ENSG00000070388	0.869158192	1.16600992	protein_coding	19	639926	643703	FGF22
ENSG00000070404	10.23721557	10.44519147	protein_coding	19	676389	683392	FSTL3
ENSG00000070413	10.09997975	10.12553154	protein_coding	22	19023795	19109967	DGCR2
ENSG00000070423	9.834707333	9.87244022	protein_coding	19	647530	663233	RNF126
ENSG00000070444	10.05079697	9.636359306	protein_coding	17	2287354	2304412	MNT
ENSG00000070476	8.016241092	8.449576416	protein_coding	3	126156444	126194762	ZXDC
ENSG00000070495	8.507303029	8.578395649	protein_coding	17	74708919	74722866	JMJD6
ENSG00000070501	6.771940731	6.624193585	protein_coding	8	42195972	42229326	POLB
ENSG00000070540	7.395627075	7.825075067	protein_coding	17	66417423	66453653	WIPI1
ENSG00000070601	0.499066092	0.390640832	protein_coding	9	37650997	37746901	FRMPD1
ENSG00000070610	8.433338631	8.68797299	protein_coding	9	35736863	35749983	GBA2
ENSG00000070614	10.72081773	10.49142535	protein_coding	5	149865381	149937773	NDST1
ENSG00000070669	10.6572561	10.55751618	protein_coding	7	97481430	97501854	ASNS
ENSG00000070718	8.311006074	8.886830358	protein_coding	8	42010464	42029191	AP3M2
ENSG00000070729	3.603959378	3.453019579	protein_coding	16	57917503	58005020	CNGB1
ENSG00000070731	5.795854912	4.385599336	protein_coding	17	74561461	74582145	ST6GALNAC2
ENSG00000070756	13.22593694	13.21984726	protein_coding	8	101698044	101735037	PABPC1
ENSG00000070759	4.734682531	5.404622322	protein_coding	1	45809555	45956872	TESK2
ENSG00000070761	8.118473522	8.832754911	protein_coding	16	58147496	58163354	C16orf80
ENSG00000070770	10.12635891	10.028782	protein_coding	16	58191811	58231824	CSNK2A2
ENSG00000070778	8.068262669	8.18248514	protein_coding	14	88932122	89021077	PTPN21
ENSG00000070785	8.251597515	8.34031144	protein_coding	1	45316450	45452282	EIF2B3
ENSG00000070808	2.35979773	1.518964905	protein_coding	5	149599054	149669854	CAMK2A
ENSG00000070814	10.84335951	10.53410706	protein_coding	5	149737202	149779870	TCOF1
ENSG00000070831	10.71266873	10.59309496	protein_coding	1	22379120	22419437	CDC42
ENSG00000070882	9.30213633	8.749726325	protein_coding	7	24836158	25021253	OSBPL3
ENSG00000070886	1.960915222	1.16600992	protein_coding	1	22890057	22930087	EPHA8
ENSG00000070950	8.267164507	8.151264044	protein_coding	3	8817088	9005457	RAD18
ENSG00000070961	11.15738502	10.93101216	protein_coding	12	89981828	90103077	ATP2B1
ENSG00000071051	7.945439403	7.093659051	protein_coding	2	106361354	106510730	NCK2

ENSG00000071054	12.04808789	11.31191436	protein_coding	2	102313312	102508449	MAP4K4
ENSG00000071073	5.695874189	4.676215056	protein_coding	2	99235569	99347589	MGAT4A
ENSG00000071082	11.45177601	11.74313081	protein_coding	2	101618177	101640494	RPL31
ENSG00000071127	11.85573283	11.80813028	protein_coding	4	10075963	10118573	WDR1
ENSG00000071189	8.558809206	8.639296529	protein_coding	7	17832466	17980130	SNX13
ENSG00000071205	7.821758815	7.913327235	protein_coding	4	148653214	148993931	ARHGAP10
ENSG00000071242	9.561637084	9.774927587	protein_coding	6	166822852	167319939	RPS6KA2
ENSG00000071243	6.9512803	7.012590419	protein_coding	7	120590803	120617270	ING3
ENSG00000071246	5.512107582	6.450406542	protein_coding	14	77228532	77249354	VASH1
ENSG00000071282	6.738827102	6.59664425	protein_coding	3	8543393	8609805	LMCD1
ENSG00000071462	9.821588372	9.321350625	protein_coding	7	73097355	73119491	WBSCR22
ENSG00000071537	11.00440756	10.53531681	protein_coding	14	81937893	82000205	SEL1L
ENSG00000071539	10.33046639	10.05600976	protein_coding	5	892758	919472	TRIP13
ENSG00000071553	10.60116605	11.09340543	protein_coding	X	153656978	153664862	ATP6AP1
ENSG00000071564	11.54392161	11.25669407	protein_coding	19	1609293	1652326	TCF3
ENSG00000071575	8.011627561	6.995112131	protein_coding	2	12857015	12882860	TRIB2
ENSG00000071626	11.08895774	10.94609939	protein_coding	19	1407584	1435683	DAZAP1
ENSG00000071655	11.18274414	11.36288594	protein_coding	19	1576678	1592710	MBD3
ENSG00000071794	9.77334709	9.412389065	protein_coding	3	148747914	148804341	HLTF
ENSG00000071859	10.41761813	10.59164201	protein_coding	X	153672473	153679002	FAM50A
ENSG00000071889	9.135091581	9.402479116	protein_coding	X	153733350	153744566	FAM3A
ENSG00000071894	10.10323589	9.995767095	protein_coding	8	145618444	145634753	CPSF1
ENSG00000071909	0.869158192	1.353254395	protein_coding	2	171034655	171511662	MYO3B
ENSG00000071967	5.498982484	4.075511708	protein_coding	2	172378757	172414643	CYBRD1
ENSG00000071994	8.799958495	9.188483253	protein_coding	6	170884383	170893780	PDCD2
ENSG00000072042	10.49567985	10.33696907	protein_coding	14	68143518	68162531	RDH11
ENSG00000072062	10.82210899	10.54795834	protein_coding	19	14202509	14228574	PRKACA
ENSG00000072071	10.52753755	10.16475361	protein_coding	19	14258547	14316972	LPHN1
ENSG00000072110	13.77550477	14.1956816	protein_coding	14	69340860	69446157	ACTN1
ENSG00000072121	9.543374069	10.2319528	protein_coding	14	68194091	68283307	ZFYVE26
ENSG00000072133	2.57521082	0	protein_coding	X	83318984	83442933	RPS6KA6
ENSG00000072134	9.304024157	8.85617872	protein_coding	17	19140690	19240028	EPN2
ENSG00000072135	9.320904342	8.989255971	protein_coding	2	131113580	131132982	PTPN18
ENSG00000072163	2.928422289	2.144285137	protein_coding	2	128395956	128439360	LIMS2
ENSG00000072182	3.744048221	2.788380093	protein_coding	2	220378892	220403494	ACCN4
ENSG00000072195	7.990682129	9.738102504	protein_coding	2	220299568	220363009	SPEG
ENSG00000072201	2.762599152	1.353254395	protein_coding	4	54325468	54567572	LNX1
ENSG00000072210	9.634713422	9.130429247	protein_coding	17	19551459	19580909	ALDH3A2
ENSG00000072274	12.84577052	12.57962489	protein_coding	3	195754054	195809060	TFRC
ENSG00000072310	11.76972141	11.47556657	protein_coding	17	17714653	17740325	SREBF1
ENSG00000072315	0	0.390640832	protein_coding	X	111017543	111326004	TRPC5
ENSG00000072364	10.07808233	9.937023818	protein_coding	5	132211071	132299326	AFF4
ENSG00000072401	8.103376106	7.91704449	protein_coding	10	60094735	60129403	UBE2D1
ENSG00000072415	10.10918645	10.30150311	protein_coding	14	67707826	67802536	MPP5
ENSG00000072422	5.550781289	5.886365696	protein_coding	10	62629196	62761198	RHOBTB1
ENSG00000072501	11.23042696	11.45956588	protein_coding	X	53401070	53449677	SMC1A
ENSG00000072506	9.761030438	10.20027059	protein_coding	X	53458206	53461320	HSD17B10
ENSG00000072518	10.00742874	9.67034314	protein_coding	11	63606400	63678491	MARK2
ENSG00000072571	8.508940744	8.529454305	protein_coding	5	162887175	162918947	HMMR
ENSG00000072609	8.096857104	8.171634445	protein_coding	12	133416937	133485772	CHFR
ENSG00000072682	9.373091386	9.737577069	protein_coding	5	131527531	131631008	P4HA2
ENSG00000072736	8.700271793	8.68797299	protein_coding	16	68111243	68263162	NFATC3
ENSG00000072756	7.621344476	7.898361689	protein_coding	3	3168600	3192563	TRNT1
ENSG00000072778	12.22253612	12.95924873	protein_coding	17	7120444	7128587	ACADVL
ENSG00000072786	9.815645694	9.611339686	protein_coding	5	171469077	171615390	STK10
ENSG00000072803	8.999540089	8.887777807	protein_coding	5	171288553	171433877	FBXW11
ENSG00000072818	5.141750194	6.037446454	protein_coding	17	7239848	7254797	ACAP1
ENSG00000072832	6.236372259	6.737844962	protein_coding	4	5749811	5894785	CRMP1
ENSG00000072840	8.407231133	9.10783897	protein_coding	4	5712924	5830772	EVC
ENSG00000072849	8.331511968	9.218164439	protein_coding	17	5374571	5389537	DERL2
ENSG00000072858	1.960915222	0.950786998	protein_coding	3	113251143	113348425	SIDT1
ENSG00000072864	8.555643385	8.176294739	protein_coding	16	15737124	15820210	NDE1
ENSG00000072952	0.869158192	2.144285137	protein_coding	11	10594638	10715535	MRVI1

ENSG00000072954	6.103936836	5.187895122	protein_coding	19	16771938	16799814	TMEM38A
ENSG00000072958	10.66609026	10.73594288	protein_coding	19	16308665	16346156	AP1M1
ENSG00000073008	11.53811874	11.29219179	protein_coding	19	45147098	45169429	PVR
ENSG00000073009	7.757079669	7.789016615	protein_coding	X	153769414	153796782	IKBKG
ENSG00000073050	8.428154791	8.036341107	protein_coding	19	44047464	44079730	XRCC1
ENSG00000073060	11.15398732	11.36458839	protein_coding	12	125261606	125367214	SCARB1
ENSG00000073067	4.568725997	3.081239798	protein_coding	7	1022835	1029276	CYP2W1
ENSG00000073111	11.32944272	11.14543121	protein_coding	3	127317066	127341276	MCM2
ENSG00000073146	1.407729925	1.802319292	protein_coding	22	50528308	50600119	MOV10L1
ENSG00000073150	7.367016815	6.064512706	protein_coding	22	50609160	50618723	PANX2
ENSG00000073169	9.27736711	9.381780274	protein_coding	22	50639408	50656045	
ENSG00000073282	8.362640485	5.620622115	protein_coding	3	189349205	189615068	TP63
ENSG00000073331	7.35977471	7.756190105	protein_coding	4	113206665	113363776	ALPK1
ENSG00000073350	7.08903805	4.17820932	protein_coding	17	73521783	73571289	LLGL2
ENSG00000073417	8.875535495	9.082408649	protein_coding	15	85523671	85682376	PDE8A
ENSG00000073464	7.22677265	7.743685008	protein_coding	X	10125024	10205700	CLCN4
ENSG00000073536	8.360827871	8.282410365	protein_coding	17	33458366	33469322	NLE1
ENSG00000073578	11.36627314	11.33745188	protein_coding	5	218356	256815	SDHA
ENSG00000073584	9.308732943	9.120790981	protein_coding	17	38781214	38821393	SMARCE1
ENSG00000073598	3.830412367	3.081239798	protein_coding	17	33448650	33457751	FNDC8
ENSG00000073605	7.72361917	7.646273261	protein_coding	17	38060848	38077313	GSDMB
ENSG00000073614	9.364975797	9.077434451	protein_coding	12	389223	498620	KDM5A
ENSG00000073670	4.261916566	3.64455972	protein_coding	17	42836568	42859214	ADAM11
ENSG00000073711	7.811178326	7.836897019	protein_coding	3	135684515	135866733	PPP2R3A
ENSG00000073712	9.966893004	10.59193272	protein_coding	14	53323986	53419153	FERMT2
ENSG00000073737	1.799000381	0.390640832	protein_coding	2	169921299	169952677	DHRS9
ENSG00000073754	1.960915222	0.950786998	protein_coding	1	157800704	157868046	CD5L
ENSG00000073756	3.830412367	4.810179682	protein_coding	1	186640923	186649559	PTGS2
ENSG00000073792	10.05304379	9.414362912	protein_coding	3	185361527	185542844	IGF2BP2
ENSG00000073803	8.137655191	7.600774083	protein_coding	3	185000729	185206885	MAP3K13
ENSG00000073849	8.646233023	8.365015357	protein_coding	3	186648274	186796341	ST6GAL1
ENSG00000073905	2.928422289	2.721932731	pseudogene	X	80184999	80185845	VDAC1P1
ENSG00000073910	0	2.501982735	protein_coding	13	32605437	32870794	FRY
ENSG00000073921	11.30160288	11.34333464	protein_coding	11	85668727	85780924	PICALM
ENSG00000073969	8.694526397	8.720244202	protein_coding	17	44668075	44834830	NSF
ENSG00000074047	5.929039784	5.709041716	protein_coding	2	121493199	121750229	GLI2
ENSG00000074054	9.681936396	9.271406033	protein_coding	2	122095354	122407163	CLASP1
ENSG00000074071	9.940436455	10.1004139	protein_coding	16	1821891	1823156	MRPS34
ENSG00000074181	6.25990527	2.851901313	protein_coding	19	15270445	15311792	NOTCH3
ENSG00000074201	10.03098657	10.36207072	protein_coding	11	77225981	77348850	CLNS1A
ENSG00000074211	2.57521082	2.501982735	protein_coding	4	6322305	6565327	PPP2R2C
ENSG00000074219	9.012297947	9.008548131	protein_coding	19	49843857	49865714	TEAD2
ENSG00000074266	8.391336806	8.635917101	protein_coding	11	85955586	85989781	EED
ENSG00000074276	2.847891871	3.570966319	protein_coding	5	175969512	176022975	CDHR2
ENSG00000074319	8.984315711	9.338084052	protein_coding	11	18489883	18548779	TSG101
ENSG00000074356	8.455589162	9.01636662	protein_coding	17	3714460	3749540	C17orf85
ENSG00000074370	6.84118134	6.799557986	protein_coding	17	3822869	3867736	ATP2A3
ENSG00000074410	9.237422823	8.992782879	protein_coding	15	63613577	63674360	CA12
ENSG00000074416	10.05584741	9.172244489	protein_coding	3	127407909	127558019	MGLL
ENSG00000074527	8.094677541	6.682138126	protein_coding	12	96051583	96184930	NTN4
ENSG00000074582	8.426422697	8.666050705	protein_coding	2	219523487	219528166	BCS1L
ENSG00000074590	6.456028446	6.843193319	protein_coding	12	106457118	106533811	NUAK1
ENSG00000074603	8.393111514	8.245929405	protein_coding	15	65734801	65810042	DPP8
ENSG00000074621	6.897280837	7.255086103	protein_coding	15	65903704	65953333	SLC24A1
ENSG00000074657	8.910650905	8.797898671	protein_coding	18	56529832	56653712	ZNF532
ENSG00000074660	3.502389126	3.608232228	protein_coding	17	1537152	1549042	SCARF1
ENSG00000074695	11.34029198	11.25962481	protein_coding	18	56995055	57026503	LMAN1
ENSG00000074696	10.25830282	10.29008203	protein_coding	15	65822756	65870687	PTPLAD1
ENSG00000074706	0.869158192	0.697730409	protein_coding	6	154475631	154677926	IPCEF1
ENSG00000074755	10.02927581	10.24716765	protein_coding	17	3907739	4046314	ZZEF1
ENSG00000074771	0.499066092	0	protein_coding	6	155716504	155777037	NOX3
ENSG00000074800	14.91898157	14.85875419	protein_coding	1	8921061	8939308	ENO1
ENSG00000074803	0	0.697730409	protein_coding	15	48483861	48596275	SLC12A1

ENSG00000074842	10.56580525	10.83308768	protein_coding	19	4657566	4670415	C19orf10
ENSG00000074855	8.794597401	8.601680156	protein_coding	19	17434032	17445638	ANO8
ENSG00000074935	6.846372398	7.454976247	protein_coding	6	112391861	112408716	TUBE1
ENSG00000074964	7.840089987	8.138583935	protein_coding	1	17866330	18024369	ARHGEF10L
ENSG00000074966	3.004694206	3.532712221	protein_coding	4	48068410	48136273	TXK
ENSG00000075035	3.146112541	0	protein_coding	12	108523248	108644314	WSCD2
ENSG00000075043	5.81716234	7.129345795	protein_coding	20	62037542	62103993	KCNQ2
ENSG00000075073	3.652182994	3.133298822	protein_coding	10	71163659	71176623	TACR2
ENSG00000075089	6.970425921	7.472765069	protein_coding	12	100592900	100635643	ACTR6
ENSG00000075131	7.1740722	7.188980716	protein_coding	15	66628544	66679084	TIPIN
ENSG00000075142	9.548160668	9.55995988	protein_coding	7	87834433	87856308	SRI
ENSG00000075151	9.040880241	9.075772566	protein_coding	1	21132972	21503377	EIF4G3
ENSG00000075188	7.888702948	7.758263791	protein_coding	12	102467967	102513902	NUP37
ENSG00000075213	8.888175056	8.635917101	protein_coding	7	83587659	84122040	SEMA3A
ENSG00000075218	9.168633912	9.248723012	protein_coding	22	46692638	46726707	GTSE1
ENSG00000075223	9.941649609	9.113519788	protein_coding	7	80371854	80551675	SEMA3C
ENSG00000075234	8.133414584	8.481307156	protein_coding	22	46663858	46689905	TTC38
ENSG00000075239	10.21431058	10.59512663	protein_coding	11	107992243	108018503	ACAT1
ENSG00000075240	9.100750797	9.097229165	protein_coding	22	46971909	47075688	GRAMD4
ENSG00000075275	10.28817515	9.174575533	protein_coding	22	46756731	46933067	CELSR1
ENSG00000075290	0.499066092	1.518964905	protein_coding	10	102222798	102243501	WNT8B
ENSG00000075292	9.624929439	9.94751216	protein_coding	2	71503691	71662199	ZNF638
ENSG00000075303	7.494827594	7.173524745	protein_coding	7	87462883	87505672	SLC25A40
ENSG00000075336	7.64536895	8.424985509	protein_coding	18	71815746	71826197	TIMM21
ENSG00000075391	9.144604451	9.267043514	protein_coding	1	178062864	178448644	RASAL2
ENSG00000075399	7.281453427	7.087074634	protein_coding	16	89773542	89787394	C16orf7
ENSG00000075407	8.497437504	9.351881826	protein_coding	10	38383264	38412276	ZNF37A
ENSG00000075413	10.61377804	9.974965329	protein_coding	14	103851701	103970168	MARK3
ENSG00000075415	11.7274412	11.69333284	protein_coding	12	98987369	98995946	SLC25A3
ENSG00000075420	11.17063773	11.04729845	protein_coding	3	171757418	172119455	FNDC3B
ENSG00000075426	11.38489919	11.35125567	protein_coding	2	28615315	28640179	FOSL2
ENSG00000075461	2.238690726	0.950786998	protein_coding	17	64961013	65029518	CACNG4
ENSG00000075539	9.308732943	9.257545892	protein_coding	4	48499378	48782339	FRYL
ENSG00000075568	9.687008864	9.412389065	protein_coding	2	98372799	98612388	TMEM131
ENSG00000075618	12.94710098	10.81527095	protein_coding	7	5632439	5646286	FSCN1
ENSG00000075624	16.54663365	16.10672433	protein_coding	7	5566782	5603415	ACTB
ENSG00000075643	9.03179537	8.706348549	protein_coding	18	33767482	33852120	MOCOS
ENSG00000075651	8.528449672	8.540340653	protein_coding	3	171318195	171528740	PLD1
ENSG00000075702	9.023798905	8.802929991	protein_coding	19	36545783	36596012	WDR62
ENSG00000075711	10.02126533	9.489309466	protein_coding	3	196769431	197026171	DLG1
ENSG00000075785	11.40416813	10.98047916	protein_coding	3	128444965	128533639	RAB7A
ENSG00000075790	8.799958495	8.658298522	protein_coding	7	107220422	107269615	BCAP29
ENSG00000075826	5.707331524	6.695185789	protein_coding	10	102246399	102289628	SEC31B
ENSG00000075856	9.361354163	9.340162149	protein_coding	12	108916357	108955176	SART3
ENSG00000075884	0	0.697730409	protein_coding	2	143848931	144525921	ARHGAP15
ENSG00000075886	3.211941663	2.652276565	protein_coding	2	132233666	132240507	TUBA3D
ENSG00000075891	1.799000381	1.518964905	protein_coding	10	102495360	102589698	PAX2
ENSG00000075914	8.245716314	8.120964787	protein_coding	3	45016733	45077558	EXOSC7
ENSG00000075945	6.979904289	7.472765069	protein_coding	1	169890467	170054349	KIFAP3
ENSG00000075975	8.447071828	8.256237404	protein_coding	3	12598513	12625212	MKRN2
ENSG00000076003	10.59809193	10.47355103	protein_coding	2	136597196	136633996	MCM6
ENSG00000076043	9.003030709	9.422231406	protein_coding	11	114310108	114321001	REXO2
ENSG00000076053	7.959880444	8.251828705	protein_coding	11	114270752	114284925	RBM7
ENSG00000076067	8.227927477	7.807158493	protein_coding	12	56915713	56984745	RBMS2
ENSG00000076108	11.08704033	10.61728821	protein_coding	12	56989380	57030600	BAZ2A
ENSG00000076201	10.25147099	10.1498245	protein_coding	3	47422501	47454931	PTPN23
ENSG00000076242	9.161362874	9.380434611	protein_coding	3	37034823	37107380	MLH1
ENSG00000076248	9.600559263	9.330438649	protein_coding	12	109535379	109548797	UNG
ENSG00000076258	2.671945279	2.579085888	protein_coding	1	171283347	171311223	FMO4
ENSG00000076321	6.882196036	7.20731193	protein_coding	1	173684080	173755840	KLHL20
ENSG00000076344	5.207774174	4.342020395	protein_coding	16	318300	325980	RGS11
ENSG00000076351	7.266040677	6.376734172	protein_coding	17	26721661	26733228	SLC46A1
ENSG00000076356	9.131906634	8.159132889	protein_coding	1	208195587	208417665	PLXNA2

ENSG00000076382	9.777429384	9.450079579	protein_coding	17	26904608	26926297	SPAG5
ENSG00000076513	9.822247161	9.719065235	protein_coding	12	110436991	110477568	ANKRD13A
ENSG00000076554	9.971651591	10.12673589	protein_coding	8	80870571	81143467	TPD52
ENSG00000076555	5.588445295	5.784235782	protein_coding	12	109554400	109706031	ACACB
ENSG00000076604	9.671006391	9.116755954	protein_coding	17	27071036	27077974	TRAF4
ENSG00000076641	6.442380648	7.012590419	protein_coding	8	81882526	82024303	PAG1
ENSG00000076650	7.119373418	6.966698819	protein_coding	19	33571786	33621318	GPATCH1
ENSG00000076662	7.530740159	7.813155441	protein_coding	19	10444454	10450345	ICAM3
ENSG00000076685	9.767201908	9.870045082	protein_coding	10	104845940	104953056	NT5C2
ENSG00000076706	9.613557158	10.69620416	protein_coding	11	119179241	119191799	MCAM
ENSG00000076716	5.672682881	3.081239798	protein_coding	X	132434131	132549518	GPC4
ENSG00000076770	5.538004898	2.420525079	protein_coding	X	131503345	131623996	MBNL3
ENSG00000076826	7.618313091	5.871116342	protein_coding	19	7660788	7683194	CAMSAP3
ENSG00000076864	7.590740122	6.699508919	protein_coding	1	21922708	21995856	RAP1GAP
ENSG00000076924	9.578112479	9.695451059	protein_coding	19	7684412	7694451	XAB2
ENSG00000076928	8.911889356	9.108651887	protein_coding	19	42387267	42411597	ARHGEF1
ENSG00000076944	10.79685863	10.46151059	protein_coding	19	7701991	7712759	STXBP2
ENSG00000076984	9.786910053	9.820791836	protein_coding	19	7968728	7979363	MAP2K7
ENSG00000077009	2.238690726	2.039052734	protein_coding	19	3933101	3942412	ITGB1BP3
ENSG00000077044	10.57560642	9.770824962	protein_coding	2	234263153	234380750	DGKD
ENSG00000077080	1.799000381	1.353254395	protein_coding	7	100240720	100254084	ACTL6B
ENSG00000077092	1.16343121	0.390640832	protein_coding	3	25215823	25639423	RARB
ENSG00000077097	10.77932387	10.88833484	protein_coding	3	25639475	25706398	TOP2B
ENSG00000077147	10.98659239	11.17270185	protein_coding	10	98277866	98347209	TM9SF3
ENSG00000077150	10.90746982	11.32596348	protein_coding	10	104154229	104162281	NFKB2
ENSG00000077152	7.933293789	8.409235632	protein_coding	1	202300785	202311108	UBE2T
ENSG00000077157	6.956090577	7.026422172	protein_coding	1	202317827	202557697	PPP1R12B
ENSG00000077232	10.6991035	10.74668835	protein_coding	2	183580999	183653542	DNAJC10
ENSG00000077235	10.90031222	10.89376853	protein_coding	16	27470876	27561251	GTF3C1
ENSG00000077238	10.18797239	9.792234745	protein_coding	16	27324989	27376099	IL4R
ENSG00000077254	8.632776617	8.76011133	protein_coding	1	78161672	78225537	USP33
ENSG00000077312	10.01378699	9.775439595	protein_coding	19	41256759	41271296	SNRPA
ENSG00000077348	8.158673248	8.598211347	protein_coding	19	41892281	41903256	EXOSC5
ENSG00000077380	9.454383134	9.329741599	protein_coding	2	172543919	172604930	DYNC1I2
ENSG00000077420	0	0.950786998	protein_coding	10	26726706	26856732	APBB1IP
ENSG00000077454	8.971306859	8.601680156	protein_coding	7	100169855	100183776	LRCH4
ENSG00000077458	7.37062428	7.803146634	protein_coding	11	95502106	95523573	FAM76B
ENSG00000077463	8.768856552	8.864865519	protein_coding	19	4174106	4182596	SIRT6
ENSG00000077514	8.318496529	8.736113084	protein_coding	11	74204896	74380162	POLD3
ENSG00000077522	0.499066092	0.950786998	protein_coding	1	236849754	236927931	ACTN2
ENSG00000077549	10.93909487	10.87788257	protein_coding	1	19665267	19812066	CAPZB
ENSG00000077616	3.146112541	2.652276565	protein_coding	11	89864683	89926062	NAALAD2
ENSG00000077684	8.849919232	8.924251218	protein_coding	4	129730779	129796379	PHF17
ENSG00000077713	8.462367008	8.282410365	protein_coding	X	118533023	118588441	SLC25A43
ENSG00000077721	9.500388149	10.04925084	protein_coding	X	118708501	118718381	UBE2A
ENSG00000077782	8.66984994	8.467431533	protein_coding	8	38268656	38326352	FGFR1
ENSG00000077800	1.16343121	1.925536307	protein_coding	7	72742167	72772634	FKBP6
ENSG00000077809	10.74533954	10.44744231	protein_coding	7	74071994	74175026	GTF2I
ENSG00000077935	7.916116295	7.848622884	protein_coding	22	45739944	45809500	SMC1B
ENSG00000077942	6.892270075	6.799557986	protein_coding	22	45898118	45997015	FBLN1
ENSG00000077984	1.616589159	1.518964905	protein_coding	20	24929866	24940564	CST7
ENSG00000078018	2.847891871	3.411459265	protein_coding	2	210288782	210598842	MAP2
ENSG00000078043	7.666067113	7.994732806	protein_coding	18	44392060	44497468	PIAS2
ENSG00000078053	1.407729925	2.420525079	protein_coding	7	38423305	38671167	AMPH
ENSG00000078061	10.37155313	10.35283736	protein_coding	X	47420516	47431307	ARAF
ENSG00000078070	7.868433623	8.587754596	protein_coding	3	182733006	182833863	MCCC1
ENSG00000078081	5.346128795	5.767861411	protein_coding	3	182840001	182881627	LAMP3
ENSG00000078098	0.499066092	0.697730409	protein_coding	2	163027194	163101661	FAP
ENSG00000078114	4.999881834	3.64455972	protein_coding	10	21068902	21463116	NEBL
ENSG00000078124	8.375265417	8.282410365	protein_coding	11	76571911	76737841	ACER3
ENSG00000078140	9.827506672	9.768769266	protein_coding	4	39699664	39784412	UBE2K
ENSG00000078142	8.074911866	8.8288137	protein_coding	18	39535171	39667794	PIK3C3
ENSG00000078177	6.449220685	6.660126317	protein_coding	4	40058446	40159872	N4BP2

ENSG00000078237	7.498129565	7.421346415	protein_coding	12	4430371	4462338	C12orf5
ENSG00000078246	8.668385155	8.352716275	protein_coding	12	2986389	3050306	TULP3
ENSG00000078269	8.965354743	8.862939634	protein_coding	6	158402888	158520208	SYNJ2
ENSG00000078295	0.499066092	0	protein_coding	5	7396321	7830194	ADCY2
ENSG00000078304	10.45037457	9.4622154	protein_coding	14	102228135	102394326	PPP2R5C
ENSG00000078319	5.090170488	4.810179682	pseudogene	7	99918615	99939531	PMS2P1
ENSG00000078369	11.85541103	11.66570416	protein_coding	1	1716725	1822502	GNB1
ENSG00000078399	1.16343121	2.039052734	protein_coding	7	27202057	27209269	HOXA9
ENSG00000078401	6.738827102	7.281279771	protein_coding	6	12290596	12297427	EDN1
ENSG00000078403	8.851210906	8.38394126	protein_coding	10	21823094	22032559	MLLT10
ENSG00000078487	4.77879189	6.110690132	protein_coding	7	99998449	100026615	ZCWPW1
ENSG00000078618	10.85789018	10.86374712	protein_coding	1	52254863	52344477	NRD1
ENSG00000078668	9.310612162	9.874831389	protein_coding	8	42249142	42263415	VDAC3
ENSG00000078674	9.354994343	9.804327751	protein_coding	8	17780349	17887453	PCM1
ENSG00000078687	6.393570237	6.963107483	protein_coding	17	76000249	76104916	TNRC6C
ENSG00000078699	8.620709081	8.801925129	protein_coding	20	32077881	32237842	CBFA2T2
ENSG00000078747	10.25635417	9.804829433	protein_coding	20	32951041	33099198	ITCH
ENSG00000078795	1.960915222	0.697730409	protein_coding	5	137223657	137278436	PKD2L2
ENSG00000078804	8.655134734	8.454700467	protein_coding	20	33292094	33301243	TP53INP2
ENSG00000078808	10.63230386	11.10098272	protein_coding	1	1152288	1167411	SDF4
ENSG00000078814	4.543379137	5.361622823	protein_coding	20	33563206	33590240	MYH7B
ENSG00000078900	2.671945279	3.32456471	protein_coding	1	3569084	3652765	TP73
ENSG00000078902	9.168633912	9.630144961	protein_coding	11	1295601	1330884	TOLLIP
ENSG00000078967	6.554468172	6.554308016	protein_coding	7	43966037	43993166	UBE2D4
ENSG00000079101	0.869158192	0	protein_coding	18	596998	650334	CLUL1
ENSG00000079102	0.499066092	0	protein_coding	8	92967203	93115514	RUNX1T1
ENSG00000079112	0	0.950786998	protein_coding	8	95139399	95229531	CDH17
ENSG00000079134	7.824391858	7.978815021	protein_coding	18	214527	268059	THOC1
ENSG00000079150	5.346128795	5.583678991	protein_coding	2	179328391	179343327	FKBP7
ENSG00000079156	7.584540566	7.460081189	protein_coding	2	179059208	179264160	OSBPL6
ENSG00000079215	0.869158192	2.652276565	protein_coding	5	36606457	36688436	SLC1A3
ENSG00000079246	11.87014001	11.8484726	protein_coding	2	216972187	217071026	XRCC5
ENSG00000079257	5.054730169	5.361622823	protein_coding	3	158363611	158390482	LXN
ENSG00000079263	3.950976309	4.710890357	protein_coding	2	231090444	231223762	SP140
ENSG00000079277	8.027710784	7.830998152	protein_coding	1	47023090	47082515	MKNK1
ENSG00000079308	6.998676229	7.167295693	protein_coding	2	218664512	218867718	TNS1
ENSG00000079313	10.068119	9.881505655	protein_coding	19	1815245	1848452	REXO1
ENSG00000079332	10.25098176	9.906963845	protein_coding	10	71909960	71930279	SAR1A
ENSG00000079335	5.40360309	5.150490275	protein_coding	1	100810584	100985833	CDC14A
ENSG00000079337	4.543379137	4.127774132	protein_coding	12	48128455	48164823	RAPGEF3
ENSG00000079385	3.502389126	2.501982735	protein_coding	19	43011458	43032661	CEACAM1
ENSG00000079387	8.043616684	8.098224647	protein_coding	12	48436681	48500091	SENP1
ENSG00000079393	1.16343121	0.697730409	protein_coding	10	76854190	76868976	DUSP13
ENSG00000079432	9.106170722	8.796890297	protein_coding	19	42788817	42799949	CIC
ENSG00000079435	4.999881834	5.717592784	protein_coding	19	42905666	42931578	LIPE
ENSG00000079459	10.70732926	9.744915802	protein_coding	8	11653082	11696818	FDFT1
ENSG00000079462	6.9512803	7.113233895	protein_coding	19	42801185	42806929	PAFAH1B3
ENSG00000079482	6.47626108	6.977419495	protein_coding	X	67262186	67653755	OPHN1
ENSG00000079616	10.07310926	10.32931774	protein_coding	16	29801499	29816706	KIF22
ENSG00000079689	0.499066092	0	protein_coding	6	25652464	25702011	SCGN
ENSG00000079691	9.159278686	8.449576416	protein_coding	6	25279306	25620758	LRRC16A
ENSG00000079739	10.08303831	9.961079008	protein_coding	1	64058916	64125916	PGM1
ENSG00000079785	10.47822979	10.12512987	protein_coding	2	15731302	15771225	DDX1
ENSG00000079805	12.12191655	12.10636339	protein_coding	19	10824143	10942586	DNM2
ENSG00000079819	10.97715801	11.12347869	protein_coding	6	131160487	131384462	EPB41L2
ENSG00000079841	0.499066092	0	protein_coding	6	72596406	73112845	RIMS1
ENSG00000079931	1.16343121	2.334191469	protein_coding	6	132617194	132722684	MOXD1
ENSG00000079950	7.947856304	8.41318925	protein_coding	6	132779054	132834337	STX7
ENSG00000079974	7.440941622	7.73528765	protein_coding	22	51205929	51222091	RABL2B
ENSG00000079999	10.94303755	11.29540842	protein_coding	19	10596796	10614054	KEAP1
ENSG00000080007	1.799000381	1.16600992	protein_coding	6	74104471	74127292	DDX43
ENSG00000080031	6.766474304	6.376734172	protein_coding	19	55692618	55720874	PTPRH
ENSG00000080166	0.499066092	0.390640832	protein_coding	13	95090830	95131936	DCT

ENSG00000080189	9.396283191	9.478029618	protein_coding	20	44978167	44993043	SLC35C2
ENSG00000080200	7.026383958	6.926693689	protein_coding	3	97595819	97663810	CRYBG3
ENSG00000080224	2.10647801	3.232099092	protein_coding	3	96533425	97471304	EPHA6
ENSG00000080298	4.165528823	3.813624741	protein_coding	9	3218297	3526004	RFX3
ENSG00000080345	10.00858687	9.738627748	protein_coding	2	152266397	152364527	RIF1
ENSG00000080371	8.585441838	8.119352325	protein_coding	12	72148654	72184699	RAB21
ENSG00000080493	2.57521082	1.16600992	protein_coding	4	72053003	72437804	SLC4A4
ENSG00000080503	7.618313091	4.42790041	protein_coding	9	2015342	2193624	SMARCA2
ENSG00000080511	0.499066092	0	protein_coding	19	10123925	10132954	RDH8
ENSG00000080546	5.270908378	5.593003837	protein_coding	6	109307640	109416022	SESN1
ENSG00000080561	6.204385613	6.970281236	protein_coding	X	107068985	107170423	MID2
ENSG00000080572	0	0.390640832	protein_coding	X	106449862	106487473	CXorf41
ENSG00000080573	2.671945279	1.353254395	protein_coding	19	10070237	10121147	COL5A3
ENSG00000080603	11.5901033	11.66293964	protein_coding	16	30709530	30755602	SRCAP
ENSG00000080608	8.12275821	8.058387721	protein_coding	9	2720469	2844241	KIAA0020
ENSG00000080618	0	0.697730409	protein_coding	13	46627321	46679161	CPB2
ENSG00000080644	2.847891871	1.925536307	protein_coding	15	78885394	78913637	CHRNA3
ENSG00000080802	8.536501169	8.17939326	protein_coding	7	135046547	135194875	CNOT4
ENSG00000080815	10.26558692	10.29972451	protein_coding	14	73603126	73690399	PSEN1
ENSG00000080819	8.426422697	8.667154767	protein_coding	3	98239976	98312567	CPOX
ENSG00000080822	8.485841807	8.827826712	protein_coding	3	98216756	98241910	CLDND1
ENSG00000080823	7.878603882	6.815578497	protein_coding	14	102690837	102771537	MOK
ENSG00000080824	13.92513574	13.14955249	protein_coding	14	102547106	102606036	HSP90AA1
ENSG00000080839	8.838241835	9.107025596	protein_coding	20	35624752	35724398	RBL1
ENSG00000080845	11.53068103	11.74417745	protein_coding	20	34894258	35157040	DLGAP4
ENSG00000080854	4.437288965	4.932754837	protein_coding	11	133785185	133826880	IGSF9B
ENSG00000080947	4.352265886	4.42790041	pseudogene	1	16793931	16819196	CROCCP3
ENSG00000080986	8.002356002	8.475016594	protein_coding	18	2571510	2616634	NDC80
ENSG00000081014	7.6689999	7.869875863	protein_coding	15	51200869	51298097	AP4E1
ENSG00000081019	7.277615649	7.442134301	protein_coding	1	114304454	114355098	RSBN1
ENSG00000081026	7.1740722	7.292770473	protein_coding	1	113933371	114228545	MAGI3
ENSG00000081041	9.447567586	8.847439299	protein_coding	4	74962793	74965010	CXCL2
ENSG00000081051	0	0.697730409	protein_coding	4	74296855	74321891	AFP
ENSG00000081052	1.407729925	0.950786998	protein_coding	2	227867427	228028829	COL4A4
ENSG00000081059	7.053569556	5.283144778	protein_coding	5	133450402	133487556	TCF7
ENSG00000081087	8.199812472	8.501254594	protein_coding	6	108362613	108487058	OSTM1
ENSG00000081148	1.407729925	2.144285137	protein_coding	3	100945570	101039404	IMPG2
ENSG00000081154	9.963910896	9.935650135	protein_coding	3	101292939	101313281	PCNP
ENSG00000081177	9.077762113	9.454562557	protein_coding	14	69658228	69709075	EXD2
ENSG00000081181	7.494827594	7.609989438	protein_coding	14	68086515	68118437	ARG2
ENSG00000081189	5.741167503	4.448594745	protein_coding	5	88013975	88199922	MEF2C
ENSG00000081237	0	0.390640832	protein_coding	1	198607801	198726545	PTPRC
ENSG00000081248	0	0.390640832	protein_coding	1	201008642	201081694	CACNA1S
ENSG00000081277	0	0.697730409	protein_coding	1	201252580	201302121	PKP1
ENSG00000081307	8.66103884	8.76630664	protein_coding	3	132373290	132396941	UBA5
ENSG00000081320	8.574534937	7.681666888	protein_coding	2	196998290	197041227	STK17B
ENSG00000081377	8.166995477	7.896480033	protein_coding	9	99252523	99382112	CDC14B
ENSG00000081386	7.457998197	7.544210495	protein_coding	9	99518147	99540411	ZNF510
ENSG00000081479	0.499066092	0	protein_coding	2	169983619	170219195	LRP2
ENSG00000081665	8.112022537	8.216065544	protein_coding	19	19902620	19932560	ZNF506
ENSG00000081692	6.138202758	6.963107483	protein_coding	1	227918126	227923112	JMJD4
ENSG00000081721	7.813830732	8.285289377	protein_coding	1	161719548	161727028	DUSP12
ENSG00000081760	9.151960225	8.930701228	protein_coding	12	125549925	125627873	AACS
ENSG00000081791	8.75650134	8.749726325	protein_coding	5	141303373	141321612	KIAA0141
ENSG00000081803	7.214780754	7.090370599	protein_coding	7	121958481	122526813	CADPS2
ENSG00000081818	2.35979773	2.334191469	protein_coding	5	140501581	140505201	PCDHB4
ENSG00000081842	3.911897206	2.788380093	protein_coding	5	140207563	140391929	PCDHA6
ENSG00000081853	5.986594255	5.43605125	protein_coding	5	140718539	140892546	PCDHGA2
ENSG00000081870	6.887241849	7.460081189	protein_coding	1	54387234	54411975	HSPB11
ENSG00000081913	8.041355121	7.815148897	protein_coding	18	60382672	60647666	PHLPP1
ENSG00000081923	8.641761486	8.435390709	protein_coding	18	55313658	55470327	ATP8B1
ENSG00000081985	2.928422289	1.925536307	protein_coding	1	67773047	67862583	IL12RB2
ENSG00000082014	6.640654398	6.937714655	protein_coding	7	150935850	150974982	SMARCD3

ENSG00000082068	8.718787866	8.675956977	protein_coding	5	37379314	37753537	WDR70
ENSG00000082074	6.585843317	2.039052734	protein_coding	5	39105338	39274630	FYB
ENSG00000082126	0.499066092	1.353254395	protein_coding	2	202509593	202563417	MPP4
ENSG00000082146	6.922076877	6.904395923	protein_coding	2	202252581	202345574	STRADB
ENSG00000082153	11.01424267	10.44100201	protein_coding	2	201675317	201688569	BZW1
ENSG00000082196	3.950976309	4.363974406	protein_coding	5	33987279	34124633	C1QTNF3
ENSG00000082212	9.499564655	9.823765224	protein_coding	18	48405419	48476160	ME2
ENSG00000082213	9.635463295	9.066597822	protein_coding	5	31532373	31555165	C5orf22
ENSG00000082258	8.580777545	8.501254594	protein_coding	2	135675805	135716912	CCNT2
ENSG00000082269	7.062518763	6.440109466	protein_coding	6	71122644	71270877	FAM135A
ENSG00000082293	0.499066092	1.353254395	protein_coding	6	70576463	70919679	COL19A1
ENSG00000082438	6.112579918	7.080460027	protein_coding	2	165510134	165700189	COBLL1
ENSG00000082458	8.380642463	7.659647832	protein_coding	X	69664711	69725337	DLG3
ENSG00000082497	6.777386522	6.450406542	protein_coding	1	210406144	210419976	SERTAD4
ENSG00000082512	6.846372398	7.670699368	protein_coding	1	211499957	211548288	TRAF5
ENSG00000082515	7.553136766	8.116121984	protein_coding	5	154320630	154348971	MRPL22
ENSG00000082516	9.31342641	9.227175607	protein_coding	5	154266976	154317769	GEMIN5
ENSG00000082556	0.499066092	0	protein_coding	8	54138284	54164257	OPRK1
ENSG00000082641	11.78467101	12.03823726	protein_coding	17	46125686	46138906	NFE2L1
ENSG00000082684	3.448790144	5.163066621	protein_coding	3	122628041	122747452	SEMA5B
ENSG00000082701	10.06978434	9.761034214	protein_coding	3	119540170	119813264	GSK3B
ENSG00000082781	10.43710307	10.72538179	protein_coding	3	124480795	124606674	ITGB5
ENSG00000082805	10.37514929	10.06273716	protein_coding	12	1099675	1605090	ERC1
ENSG00000082898	11.26790536	11.31032524	protein_coding	2	61704984	61765761	XPO1
ENSG00000082996	8.331511968	8.202422444	protein_coding	3	149530495	149679926	RNF13
ENSG00000083093	8.257454839	8.663840043	protein_coding	16	23614488	23652631	PALB2
ENSG00000083097	6.393570237	6.915587884	protein_coding	6	83777385	83881069	DOPEY1
ENSG00000083099	7.881135284	8.106387334	protein_coding	6	90277863	90348472	LYRM2
ENSG00000083123	6.820228284	6.952279523	protein_coding	6	80816364	81055987	BCKDHB
ENSG00000083168	8.976050934	8.811942385	protein_coding	8	41786997	41909508	KAT6A
ENSG00000083223	8.53810609	8.633659743	protein_coding	9	88902648	88969369	ZCCHC6
ENSG00000083290	7.440941622	8.606292299	protein_coding	17	19674143	19771249	ULK2
ENSG00000083307	7.76258174	0	protein_coding	8	102504660	102681954	GRHL2
ENSG00000083312	10.60193356	10.4020761	protein_coding	5	72112139	72212560	TNPO1
ENSG00000083444	11.57728171	12.27453258	protein_coding	1	11994262	12035599	PLOD1
ENSG00000083454	7.715131365	7.093659051	protein_coding	17	3575748	3599698	P2RX5
ENSG00000083457	7.269909329	8.554729165	protein_coding	17	3617920	3704537	ITGAE
ENSG00000083520	8.70742151	8.159132889	protein_coding	13	73329540	73356234	DIS3
ENSG00000083535	6.47626108	6.044260771	protein_coding	13	73356197	73590592	PIBF1
ENSG00000083544	6.343050432	6.316088276	protein_coding	13	60970591	61148012	TDRD3
ENSG00000083635	6.970425921	6.293387477	protein_coding	13	45513384	45563618	NUFIP1
ENSG00000083642	8.445362311	7.894595919	protein_coding	13	33160564	33352157	PDS5B
ENSG00000083720	10.30146577	10.46786018	protein_coding	5	41730167	41870621	OXCT1
ENSG00000083750	6.350376764	6.881748114	protein_coding	X	55744172	55785207	RRAGB
ENSG00000083799	9.310612162	10.30469907	protein_coding	16	50775961	50835846	CYLD
ENSG00000083807	7.457998197	7.243290054	protein_coding	19	59009704	59023432	SLC27A5
ENSG00000083812	7.498129565	7.281279771	protein_coding	19	58978459	58984781	ZNF324
ENSG00000083814	6.738827102	6.729413443	protein_coding	19	58231120	58238995	ZNF671
ENSG00000083817	6.788216822	6.414040573	protein_coding	19	58082935	58090243	ZNF416
ENSG00000083828	7.593829938	7.439552137	protein_coding	19	58280997	58320617	ZNF586
ENSG00000083838	6.998676229	7.312661576	protein_coding	19	58987795	59000899	ZNF446
ENSG00000083842	7.46813618	7.532142019	protein_coding	19	58790318	58807254	ZNF8
ENSG00000083844	9.171738888	8.815929893	protein_coding	19	57702868	57734212	ZNF264
ENSG00000083845	13.10844823	13.55231539	protein_coding	19	58898636	58906170	RPS5
ENSG00000083857	10.90249438	11.3952271	protein_coding	4	187508937	187647876	FAT1
ENSG00000083896	9.30213633	9.469827862	protein_coding	4	69176105	69215807	YTHDC1
ENSG00000083937	8.443650765	8.269383236	protein_coding	3	87276421	87304698	CHMP2B
ENSG00000084070	8.18759341	7.992972811	protein_coding	1	40810522	40888998	SMAP2
ENSG00000084072	8.574534937	8.861975727	protein_coding	1	40157854	40229586	PPIE
ENSG00000084073	8.98077946	9.09886653	protein_coding	1	40723779	40759856	ZMPSTE24
ENSG00000084090	10.99333545	10.44196989	protein_coding	2	96850597	96874563	STARD7
ENSG00000084092	8.640267888	8.66273344	protein_coding	4	57829536	57844989	NOA1
ENSG00000084093	8.718787866	8.66273344	protein_coding	4	57774042	57813577	REST

ENSG00000084110	2.671945279	2.501982735	protein_coding	12	96366440	96390143	HAL
ENSG00000084112	9.802351442	9.398495997	protein_coding	12	109176466	109251366	SSH1
ENSG00000084207	12.58648018	13.19077694	protein_coding	11	67351066	67354131	GSTP1
ENSG00000084234	13.08523984	13.22660304	protein_coding	11	129939732	130014699	APLP2
ENSG00000084444	7.484876093	6.510691757	protein_coding	12	13197218	13295455	KIAA1467
ENSG00000084463	10.56541181	10.20141416	protein_coding	12	14939410	14956474	WBP11
ENSG00000084623	10.97597436	10.84728173	protein_coding	1	32687529	32697205	EIF3I
ENSG00000084628	0.499066092	0.950786998	protein_coding	1	31652592	31712401	NKAIN1
ENSG00000084636	9.088754802	10.07443515	protein_coding	1	32117848	32169920	COL16A1
ENSG00000084652	10.18899434	10.07941968	protein_coding	1	32645287	32663886	TXLNA
ENSG00000084676	7.712290964	7.460081189	protein_coding	2	24714783	24993571	NCOA1
ENSG00000084693	8.805299741	8.739266022	protein_coding	2	27265232	27293490	AGBL5
ENSG00000084710	2.471521042	2.652276565	protein_coding	2	25264999	25378243	EFR3B
ENSG00000084731	9.526494794	9.682951719	protein_coding	2	26149471	26205618	KIF3C
ENSG00000084733	10.59809193	10.86061382	protein_coding	2	26256976	26360323	RAB10
ENSG00000084734	2.671945279	1.925536307	protein_coding	2	27719709	27746554	GCKR
ENSG00000084754	10.80654585	10.8996516	protein_coding	2	26413504	26467594	HADHA
ENSG00000084764	7.940593421	8.026050991	protein_coding	2	27193480	27250064	MAPRE3
ENSG00000084774	11.46259163	11.34712838	protein_coding	2	27440258	27466660	CAD
ENSG00000085063	10.36659374	11.25669407	protein_coding	11	33719807	33757991	CD59
ENSG00000085117	8.650690744	8.534302826	protein_coding	11	44585977	44641913	CD82
ENSG00000085185	8.502378699	7.996490656	protein_coding	X	129115083	129192058	BCORL1
ENSG00000085224	9.567149814	9.608468897	protein_coding	X	76760378	77041702	ATRX
ENSG00000085231	8.965354743	9.003312166	protein_coding	5	68646811	68665840	TAF9
ENSG00000085274	7.67776266	7.842771865	protein_coding	3	169490853	169507504	MYNN
ENSG00000085276	8.309127368	7.928139131	protein_coding	3	168801287	169381406	MECOM
ENSG00000085365	8.839544001	8.449576416	processed_transc	5	77656339	77776562	SCAMP1
ENSG00000085377	8.978417134	8.951693347	protein_coding	6	105725440	105850959	PREP
ENSG00000085382	6.560798025	6.750400563	protein_coding	6	105175968	105307794	HACE1
ENSG00000085415	9.616598542	9.469827862	protein_coding	18	12947983	12987533	SEH1L
ENSG00000085433	7.202688343	7.087074634	protein_coding	1	109512836	109584850	WDR47
ENSG00000085449	9.466233411	9.390496617	protein_coding	2	224720433	224810104	WDFY1
ENSG00000085465	4.6658819	5.085909553	protein_coding	1	111956936	111970399	OVGP1
ENSG00000085491	8.635777795	8.605140645	protein_coding	1	108676658	108743471	SLC25A24
ENSG00000085511	8.528449672	8.317991549	protein_coding	6	161412759	161551917	MAP3K4
ENSG00000085514	3.950976309	3.081239798	protein_coding	7	99965153	99997719	PILRA
ENSG00000085552	6.598204782	5.717592784	protein_coding	1	159896829	159915394	IGSF9
ENSG00000085644	8.380642463	8.343077302	protein_coding	16	3185057	3192804	ZNF213
ENSG00000085662	14.02987148	13.5303007	protein_coding	7	134127127	134144036	AKR1B1
ENSG00000085719	9.857382318	9.650942216	protein_coding	8	87497059	87573726	CPNE3
ENSG00000085721	9.191250057	9.353940157	protein_coding	16	15153879	15188174	RRN3
ENSG00000085733	12.4773665	12.08463011	protein_coding	11	70244510	70282690	CTTN
ENSG00000085741	1.799000381	0.697730409	protein_coding	11	75897369	75921803	WNT11
ENSG00000085760	8.593182305	8.870627795	protein_coding	2	55463731	55496483	MTIF2
ENSG00000085788	8.908170811	8.675956977	protein_coding	8	38082736	38133076	DDHD2
ENSG00000085831	5.175139844	3.027231696	protein_coding	1	51752930	51810788	TTC39A
ENSG00000085832	9.200400892	8.965221116	protein_coding	1	51819935	51985000	EPS15
ENSG00000085840	8.156585168	7.985911283	protein_coding	1	52838501	52870131	ORC1
ENSG00000085871	5.538004898	5.456629825	protein_coding	4	140586922	140661899	MGST2
ENSG00000085872	10.62891894	10.36138879	protein_coding	19	16628700	16653341	CHERP
ENSG00000085978	8.585441838	8.317991549	protein_coding	2	234118697	234204320	ATG16L1
ENSG00000085982	9.457778871	9.224178134	protein_coding	2	234384166	234475428	USP40
ENSG00000085998	9.122309386	9.478029618	protein_coding	1	46654354	46685977	POMGNT1
ENSG00000085999	8.137655191	8.220584723	protein_coding	1	46713360	46744145	RAD54L
ENSG00000086015	10.22978644	10.66670026	protein_coding	1	46252659	46501896	MAST2
ENSG00000086061	10.23770949	10.54735887	protein_coding	9	33025209	33039905	DNAJA1
ENSG00000086062	11.62733291	11.32281433	protein_coding	9	33104080	33167354	B4GALT1
ENSG00000086065	8.549290825	9.080752488	protein_coding	9	33264940	33281977	CHMP5
ENSG00000086102	8.971306859	8.989255971	protein_coding	9	33290509	33371155	NFX1
ENSG00000086159	1.16343121	1.518964905	protein_coding	12	50360977	50370922	AQP6
ENSG00000086189	8.53489446	8.65940853	protein_coding	5	61683081	61699766	DIMT1
ENSG00000086200	8.735671237	8.612036819	protein_coding	5	61699799	61924409	IPO11
ENSG00000086232	11.29259848	11.01101834	protein_coding	7	6061881	6098861	EIF2AK1

ENSG00000086289	9.74652684	9.568257332	protein_coding	7	37723446	37991543	EPDR1
ENSG00000086300	7.119373418	7.307006333	protein_coding	7	26331541	26413949	SNX10
ENSG00000086475	10.00336794	9.594609026	protein_coding	10	13359424	13390297	SEPHS1
ENSG00000086504	10.04685659	10.13074314	protein_coding	16	416927	420568	MRPL28
ENSG00000086506	0.499066092	0.950786998	protein_coding	16	230452	231180	HBQ1
ENSG00000086544	6.585843317	6.24096575	protein_coding	19	41223008	41246763	ITPKC
ENSG00000086570	6.610461229	2.652276565	protein_coding	5	150883654	150948505	FAT2
ENSG00000086589	8.849919232	8.770422115	protein_coding	5	150070356	150080669	RBM22
ENSG00000086598	10.62778887	10.60293639	protein_coding	12	124069078	124083116	TMED2
ENSG00000086619	3.448790144	3.935919592	protein_coding	1	236378855	236445319	ERO1LB
ENSG00000086666	8.635777795	8.641545092	protein_coding	15	80351910	80430735	ZFAND6
ENSG00000086712	8.805299741	9.055679178	protein_coding	X	16804550	16862642	TXLNG
ENSG00000086717	1.407729925	2.788380093	protein_coding	X	18694029	18846039	PPEF1
ENSG00000086730	5.613019223	5.163066621	protein_coding	7	73613982	73644161	LAT2
ENSG00000086758	13.83036287	13.67167652	protein_coding	X	53559057	53713673	HUWE1
ENSG00000086827	8.665451116	8.89250574	protein_coding	11	113603909	113644533	ZW10
ENSG00000086848	5.588445295	5.94580061	protein_coding	11	111652919	111750145	ALG9
ENSG00000086967	0.499066092	0.390640832	protein_coding	19	50936160	50969583	MYBPC2
ENSG00000087008	7.132181536	7.593823731	protein_coding	4	8368439	8442450	ACOX3
ENSG00000087053	9.792299736	9.833140726	protein_coding	11	95566046	95658479	MTMR2
ENSG00000087074	9.953725374	9.996206443	protein_coding	19	49375649	49379318	PPP1R15A
ENSG00000087076	4.19837882	5.84793576	protein_coding	19	49316279	49339934	HSD17B14
ENSG00000087077	10.39609755	9.728615271	protein_coding	7	100464760	100471076	TRIP6
ENSG00000087085	2.35979773	2.420525079	protein_coding	7	100487615	100494594	ACHE
ENSG00000087086	13.31165094	13.59300384	protein_coding	19	49468566	49470135	FTL
ENSG00000087087	10.65910102	10.62384241	protein_coding	7	100472733	100486285	SRRT
ENSG00000087088	9.65186265	9.592867142	protein_coding	19	49458072	49465055	BAX
ENSG00000087095	7.787084643	7.126137778	protein_coding	17	26369182	26523407	NLK
ENSG00000087111	10.00336794	9.469827862	protein_coding	17	26880405	26898546	PIGS
ENSG00000087116	4.75690578	7.005624493	protein_coding	5	178537852	178772431	ADAMTS2
ENSG00000087152	9.528110879	8.967911514	protein_coding	17	42269174	42277453	ATXN7L3
ENSG00000087157	7.464764762	7.577474828	protein_coding	17	76374735	76420635	PGS1
ENSG00000087191	10.88683462	11.08475568	protein_coding	17	61904810	61909386	PSMC5
ENSG00000087206	8.243750574	8.333373496	protein_coding	5	176332006	176449634	UIMC1
ENSG00000087237	2.35979773	3.532712221	protein_coding	16	56995762	57017757	CETP
ENSG00000087245	3.146112541	3.411459265	protein_coding	16	55512742	55540603	MMP2
ENSG00000087253	7.663128351	8.234057939	protein_coding	16	55542910	55620578	LPCAT2
ENSG00000087258	9.414746625	9.818309323	protein_coding	16	56225302	56391356	GNAO1
ENSG00000087263	9.12764907	9.381107599	protein_coding	16	56485402	56513012	OGFOD1
ENSG00000087266	10.15121608	10.3589995	protein_coding	4	2794750	2842825	SH3BP2
ENSG00000087269	9.487155506	9.562335454	protein_coding	4	2939660	2965112	NOP14
ENSG00000087274	10.39609755	10.39709408	protein_coding	4	2845584	2931803	ADD1
ENSG00000087299	7.161632221	7.392265003	protein_coding	14	50704281	50779266	L2HGDH
ENSG00000087301	6.738827102	7.315480906	protein_coding	14	52897308	53019240	TXNDC16
ENSG00000087302	10.05416589	10.4939164	protein_coding	14	52456193	52471420	C14orf166
ENSG00000087303	3.911897206	3.493416095	protein_coding	14	52471521	52536545	NID2
ENSG00000087338	7.599989788	7.827052132	protein_coding	2	70056774	70108528	GMCL1
ENSG00000087365	11.92222955	11.84628102	protein_coding	11	65818200	65836779	SF3B2
ENSG00000087448	8.407231133	8.295320915	protein_coding	12	27932953	27955973	KLHDC5
ENSG00000087460	13.13533723	13.13501313	protein_coding	20	57414773	57486247	GNAS
ENSG00000087470	10.22531053	10.28900665	protein_coding	12	32832134	32905700	DNM1L
ENSG00000087494	4.923348194	3.813624741	protein_coding	12	28111017	28125638	PTHLH
ENSG00000087495	0.869158192	2.579085888	protein_coding	20	58152564	58422766	PHACTR3
ENSG00000087502	8.735671237	8.706348549	protein_coding	12	29490285	29534122	ERGIC2
ENSG00000087510	8.766120079	8.481307156	protein_coding	20	55204358	55214339	TFAP2C
ENSG00000087586	9.546566899	9.439133999	protein_coding	20	54944445	54967393	AURKA
ENSG00000087589	2.10647801	2.039052734	protein_coding	20	54987168	55034396	CASS4
ENSG00000087842	7.549958459	7.053694091	protein_coding	X	15402921	15511687	PIR
ENSG00000087884	5.255381949	5.995870484	protein_coding	11	77532155	77629478	C11orf67
ENSG00000087903	7.981274537	7.999999944	protein_coding	19	5993175	6110664	RFX2
ENSG00000087916	3.989024711	4.319727133	protein_coding	X	115567790	115592625	SLC6A14
ENSG00000087995	7.281453427	7.421346415	protein_coding	17	60501246	60527454	METTL2A
ENSG00000088002	2.10647801	2.334191469	protein_coding	19	49055429	49102683	SULT2B1

ENSG00000088035	7.04004079	7.41347308	protein_coding	1	63833261	63904233	ALG6
ENSG00000088038	10.22032095	9.804327751	protein_coding	19	54641444	54659419	CNOT3
ENSG00000088053	5.525114348	3.748356452	protein_coding	19	55525073	55549632	GP6
ENSG00000088179	7.96466218	7.813155441	protein_coding	2	120517207	120741394	PTPN4
ENSG00000088205	10.33370433	10.13673322	protein_coding	2	118572226	118589955	DDX18
ENSG00000088247	12.18320327	11.99712347	protein_coding	19	6413121	6424822	KHSRP
ENSG00000088256	9.347691482	9.364868601	protein_coding	19	3094408	3121452	GNA11
ENSG00000088280	7.660183591	7.20731193	protein_coding	1	23750352	23810750	ASAP3
ENSG00000088298	9.632461462	10.10937675	protein_coding	20	33703160	33865928	EDEM2
ENSG00000088305	8.096857104	8.066778281	protein_coding	20	31350191	31397162	DNMT3B
ENSG00000088325	11.71206111	11.71591486	protein_coding	20	30327074	30389608	TPX2
ENSG00000088340	9.520824196	8.578395649	pseudogene	20	34146507	34195451	FER1L4
ENSG00000088356	7.680671792	8.398639372	protein_coding	20	30532758	30539895	PDRG1
ENSG00000088367	8.515473076	7.315480906	protein_coding	20	34679426	34820721	EPB41L1
ENSG00000088387	8.369868254	8.129000175	protein_coding	13	99445741	99738879	DOCK9
ENSG00000088448	9.125515568	9.342237258	protein_coding	13	111530887	111567416	ANKRD10
ENSG00000088451	6.35766608	6.450406542	protein_coding	13	95226308	95248511	TGDS
ENSG00000088538	6.26766507	7.373448694	protein_coding	3	50712672	51421629	DOCK3
ENSG00000088543	5.899377978	6.217041467	protein_coding	3	50595462	50608458	C3orf18
ENSG00000088682	8.495786671	9.212881815	protein_coding	16	57481337	57495187	COQ9
ENSG00000088726	6.196276899	5.901455548	protein_coding	3	12775024	12810956	TMEM40
ENSG00000088727	5.625150983	5.545764946	protein_coding	3	47269516	47324941	KIF9
ENSG00000088766	8.839544001	9.540813141	protein_coding	20	5986736	6020699	CRLS1
ENSG00000088808	8.500733514	7.568048738	protein_coding	14	104200089	104313927	PPP1R13B
ENSG00000088812	10.25440288	10.85238672	protein_coding	20	3451687	3631769	ATRN
ENSG00000088826	8.78921631	8.031205224	protein_coding	20	4101627	4168394	SMOX
ENSG00000088827	2.928422289	3.183544561	protein_coding	20	3667617	3687775	SIGLEC1
ENSG00000088832	11.935382	12.20629775	protein_coding	20	1349622	1373806	FKBP1A
ENSG00000088833	9.963910896	10.17099362	protein_coding	20	1422807	1448417	NSFL1C
ENSG00000088836	6.96088487	6.559073862	protein_coding	20	3208063	3219887	SLC4A11
ENSG00000088854	7.703736051	7.991210667	protein_coding	20	3229951	3388272	C20orf194
ENSG00000088876	8.006999229	8.727671407	protein_coding	20	2462463	2505348	ZNF343
ENSG00000088881	4.883502971	5.137803333	protein_coding	20	2673480	2740754	EBF4
ENSG00000088882	1.407729925	1.667587519	protein_coding	20	2774715	2781283	CPXM1
ENSG00000088888	10.57638764	10.78533279	protein_coding	20	3827487	3849280	MAVS
ENSG00000088899	7.01259661	8.125791388	protein_coding	20	3143263	3154192	
ENSG00000088926	0.499066092	0.390640832	protein_coding	4	187187099	187210835	F11
ENSG00000088930	10.72258318	11.05933204	protein_coding	20	21283942	21370463	XRN2
ENSG00000088970	7.430610036	7.929979971	processed_transc	20	21106624	21227260	PLK1S1
ENSG00000088986	10.14438343	10.63995824	protein_coding	12	120907653	120936296	DYNLL1
ENSG00000088992	0.869158192	0	protein_coding	12	117476728	117537284	TESC
ENSG00000089006	10.56777082	10.39809186	protein_coding	20	17922241	17949623	SNX5
ENSG00000089009	10.49939184	10.82840737	protein_coding	12	112842994	112856642	RPL6
ENSG00000089022	8.009315251	8.022604578	protein_coding	12	112279782	112334343	MAPKAPK5
ENSG00000089041	3.744048221	3.368666104	protein_coding	12	121570622	121623876	P2RX7
ENSG00000089048	9.265790136	9.305839261	protein_coding	20	13694969	13765532	ESF1
ENSG00000089050	9.030655728	9.622766057	protein_coding	20	18467184	18477887	RBBP9
ENSG00000089053	10.77218167	10.9795907	protein_coding	12	121746048	121837699	ANAPC5
ENSG00000089057	9.934355333	9.922303325	protein_coding	20	4833002	4990939	SLC23A2
ENSG00000089060	8.857651982	8.683615101	protein_coding	12	113736564	113797298	SLC24A6
ENSG00000089063	10.35252484	10.73962107	protein_coding	20	5080486	5093749	C20orf30
ENSG00000089091	4.981125677	5.361622823	protein_coding	20	18364011	18447829	DZANK1
ENSG00000089094	8.303476525	8.231074746	protein_coding	12	121866902	122018920	KDM2B
ENSG00000089101	1.616589159	0.390640832	protein_coding	20	20033158	20341346	C20orf26
ENSG00000089123	7.384964606	7.362585296	protein_coding	20	13246709	13619587	TASP1
ENSG00000089127	7.923503136	8.600524813	protein_coding	12	113344582	113369991	OAS1
ENSG00000089154	11.74578763	11.5975586	protein_coding	12	120565007	120632513	GCN1L1
ENSG00000089157	12.52265571	12.59935153	protein_coding	12	120634489	120639038	RPLP0
ENSG00000089159	11.46491382	10.61357045	protein_coding	12	120648250	120703574	PXN
ENSG00000089163	3.871730003	4.274079748	protein_coding	12	120740119	120751052	SIRT4
ENSG00000089177	8.701704574	8.754928172	protein_coding	20	16252749	16554078	KIF16B
ENSG00000089195	8.823839897	9.055679178	protein_coding	20	5917881	5931182	TRMT6
ENSG00000089199	0.869158192	1.353254395	protein_coding	20	5892076	5906007	CHGB

ENSG00000089220	10.50924406	10.78533279	protein_coding	12	118573663	118583389	PEBP1
ENSG00000089234	7.720795447	7.864110577	protein_coding	12	112080797	112123790	BRAP
ENSG00000089248	10.17719799	10.42575316	protein_coding	12	112451120	112461255	ERP29
ENSG00000089280	12.21264596	12.25364568	protein_coding	16	31191431	31203127	FUS
ENSG00000089289	8.550881589	8.740315472	protein_coding	X	69353299	69386174	IGBP1
ENSG00000089327	10.66755738	10.91390641	protein_coding	19	35645633	35660787	FXYD5
ENSG00000089335	6.96088487	7.444711851	protein_coding	19	35168544	35177302	ZNF302
ENSG00000089336	1.616589159	2.334191469	processed_transc	19	36103665	36116251	
ENSG00000089351	9.459473748	9.658178764	protein_coding	19	35485688	35517375	GRAMD1A
ENSG00000089356	7.420203928	0	protein_coding	19	35606732	35615227	FXYD3
ENSG00000089472	1.16343121	0	protein_coding	X	65382391	65488709	HEPH
ENSG00000089486	8.15239992	8.34031144	protein_coding	16	4560720	4588829	C16orf5
ENSG00000089505	4.230497448	4.872768657	protein_coding	16	66600294	66613040	CMTM1
ENSG00000089558	1.799000381	2.420525079	protein_coding	17	40308910	40333296	KCNH4
ENSG00000089597	12.8201102	12.95631633	protein_coding	11	62392298	62414104	GANAB
ENSG00000089639	8.964161368	8.337540265	protein_coding	19	19740288	19754455	GMIP
ENSG00000089682	7.395627075	7.605389119	protein_coding	X	106307650	106362057	RBM41
ENSG00000089685	10.17668292	9.814826612	protein_coding	17	76210277	76221715	BIRC5
ENSG00000089692	4.962122459	4.903073497	protein_coding	12	6881678	6887621	LAG3
ENSG00000089693	11.43076267	11.05364443	protein_coding	12	6857170	6876641	MLF2
ENSG00000089723	7.234712227	6.174194902	protein_coding	14	94492675	94515276	OTUB2
ENSG00000089737	11.27874564	10.8467946	protein_coding	14	94517266	94547591	DDX24
ENSG00000089775	7.153278907	7.607691112	protein_coding	14	64915824	64971931	ZBTB25
ENSG00000089818	8.085926212	7.933654619	protein_coding	12	7926148	8250367	NECAP1
ENSG00000089820	7.67776266	7.609989438	protein_coding	X	153172821	153200452	ARHGAP4
ENSG00000089847	5.316509574	5.923798455	protein_coding	19	4186267	4224811	ANKRD24
ENSG00000089876	9.108333003	9.330438649	protein_coding	10	127524906	127585005	DHX32
ENSG00000089902	9.86443576	9.045526555	protein_coding	14	103058998	103196891	RCOR1
ENSG00000089916	8.904442656	9.323452948	protein_coding	14	76618259	76720685	C14orf118
ENSG00000090006	9.285033801	9.11108789	protein_coding	19	41099072	41135725	LTBP4
ENSG00000090013	8.261346552	8.190185975	protein_coding	19	40953693	40971725	BLVRB
ENSG00000090020	8.936437694	8.285289377	protein_coding	1	27425306	27493472	SLC9A1
ENSG00000090054	9.282163564	9.354625616	protein_coding	9	94793427	94877690	SPTLC1
ENSG00000090060	11.84992942	11.22086486	protein_coding	14	96967770	97033448	PAPOLA
ENSG00000090061	10.24853313	9.064085459	protein_coding	14	99947506	100001381	CCNK
ENSG00000090097	8.460675529	8.843538053	protein_coding	3	51991470	52008032	PCBP4
ENSG00000090238	4.821592551	5.953060722	protein_coding	16	30103635	30108236	YPEL3
ENSG00000090263	7.91858278	8.278081046	protein_coding	7	140702196	140715028	MRPS33
ENSG00000090266	9.008829705	9.385138948	protein_coding	7	140390577	140422590	NDUFB2
ENSG00000090273	10.26219225	10.31706091	protein_coding	1	27226729	27273353	NUDC
ENSG00000090316	9.512684482	9.738102504	protein_coding	4	1283639	1333935	MAEA
ENSG00000090339	12.79717209	13.42066788	protein_coding	19	10381517	10397291	ICAM1
ENSG00000090372	10.19052591	10.04075742	protein_coding	19	47222770	47250251	STRN4
ENSG00000090432	8.385999544	8.529454305	protein_coding	1	20825943	20834654	MUL1
ENSG00000090447	8.925442603	8.276635048	protein_coding	16	4307187	4323001	TFAP4
ENSG00000090470	7.590740122	7.258020107	protein_coding	15	65409717	65426174	PDCD7
ENSG00000090487	9.672468519	9.570619293	protein_coding	15	65255362	65282648	SPG21
ENSG00000090512	0.499066092	0.390640832	protein_coding	3	186353758	186370930	FETUB
ENSG00000090520	9.76309053	9.67034314	protein_coding	3	186285192	186315061	DNAJB11
ENSG00000090530	11.00759685	10.55453615	protein_coding	3	189674517	189840226	LEPREL1
ENSG00000090534	0	0.390640832	protein_coding	3	184089723	184095932	THPO
ENSG00000090539	1.16343121	1.518964905	protein_coding	3	184097861	184108524	CHRD
ENSG00000090554	5.81716234	6.210997888	protein_coding	19	49977486	49989487	FLT3LG
ENSG00000090565	9.05552222	9.335308591	protein_coding	16	475619	573011	RAB11FIP3
ENSG00000090581	8.301587982	9.15109385	protein_coding	16	1401924	1413352	GNPTG
ENSG00000090612	7.773523328	7.774746834	protein_coding	12	133707570	133783698	ZNF268
ENSG00000090615	10.24460665	10.074019	protein_coding	12	133345495	133405444	GOLGA3
ENSG00000090621	11.12090211	11.32088646	protein_coding	1	40026488	40042462	PABPC4
ENSG00000090659	0.499066092	0	protein_coding	19	7804879	7812464	CD209
ENSG00000090661	8.263288478	7.630511253	protein_coding	19	8271620	8327305	CERS4
ENSG00000090674	8.185546801	8.989255971	protein_coding	19	7587496	7598894	MCOLN1
ENSG00000090686	9.351347533	9.281534232	protein_coding	1	22004791	22110099	USP48
ENSG00000090776	9.850294221	9.709451562	protein_coding	X	68048840	68061990	EFNB1

ENSG00000090857	8.393111514	8.423679567	protein_coding	16	70147529	70195203	PDPR
ENSG00000090861	12.15236149	12.50030747	protein_coding	16	70286293	70323446	AARS
ENSG00000090863	11.69345794	11.92914782	protein_coding	16	74485856	74641012	GLG1
ENSG00000090889	9.506139483	9.694368449	protein_coding	X	69509879	69640682	KIF4A
ENSG00000090905	9.86443576	9.822279296	protein_coding	16	24741016	24838953	TNRC6A
ENSG00000090920	3.146112541	2.501982735	protein_coding	19	40353964	40440533	FCGBP
ENSG00000090924	7.119373418	6.505763096	protein_coding	19	39903225	39919054	PLEKHG2
ENSG00000090932	4.230497448	4.777836593	protein_coding	19	39989557	39999118	DLL3
ENSG00000090971	8.171138658	8.597053222	protein_coding	19	55996594	55998935	NAT14
ENSG00000090975	8.774313977	8.273738696	protein_coding	12	123468027	123634562	PITPNM2
ENSG00000090989	8.443650765	8.634788863	protein_coding	4	56719782	56771200	EXOC1
ENSG00000091009	9.295509417	8.847439299	protein_coding	5	145583113	145718814	RBM27
ENSG00000091010	0.499066092	0	protein_coding	5	145718587	145720083	POU4F3
ENSG00000091039	9.144604451	9.222677059	protein_coding	12	76745577	76953589	OSBPL8
ENSG00000091073	8.197783135	7.591499486	protein_coding	7	76090993	76135312	
ENSG00000091106	1.799000381	3.027231696	protein_coding	2	32449522	32490923	NLRC4
ENSG00000091127	9.432113851	9.021555505	protein_coding	7	105080108	105162714	PUS7
ENSG00000091128	0.499066092	0.697730409	protein_coding	7	107663993	107770801	LAMB4
ENSG00000091129	1.407729925	1.353254395	protein_coding	7	107788082	108097161	NRCAM
ENSG00000091136	7.33043718	6.766973175	protein_coding	7	107564244	107643700	LAMB1
ENSG00000091137	1.16343121	2.851901313	protein_coding	7	107301080	107358254	SLC26A4
ENSG00000091138	0.869158192	0.390640832	protein_coding	7	107405912	107443670	SLC26A3
ENSG00000091140	10.73207945	10.4439037	protein_coding	7	107531415	107572175	DLD
ENSG00000091157	7.319279953	7.53698153	protein_coding	18	54318616	54698828	WDR7
ENSG00000091164	8.903197794	9.115138778	protein_coding	18	54264439	54318505	TXNL1
ENSG00000091262	2.471521042	2.721932731	protein_coding	16	16243422	16317351	ABCC6
ENSG00000091317	9.792299736	9.679125977	protein_coding	3	32522804	32544900	CMTM6
ENSG00000091409	11.14427291	8.200898549	protein_coding	2	173292082	173371181	ITGA6
ENSG00000091428	2.35979773	3.845183986	protein_coding	2	173600002	173917621	RAPGEF4
ENSG00000091436	10.34245657	9.561148156	protein_coding	2	173940163	174132738	
ENSG00000091482	2.35979773	3.368666104	protein_coding	X	21724090	21776281	SMPX
ENSG00000091483	9.771983754	10.39775935	protein_coding	1	241660903	241683061	FH
ENSG00000091490	9.940436455	9.014632836	protein_coding	4	25749055	25865382	SEL1L3
ENSG00000091513	0.499066092	0.950786998	protein_coding	3	133464800	133497850	TF
ENSG00000091527	11.21144195	10.97000497	protein_coding	3	133292574	133309105	CDV3
ENSG00000091536	4.292665995	3.87606762	protein_coding	17	18012020	18083116	MYO15A
ENSG00000091542	11.09578492	11.50689105	protein_coding	17	18086392	18113268	ALKBH5
ENSG00000091583	0.499066092	0.390640832	protein_coding	17	64208148	64225556	APOH
ENSG00000091592	8.539709228	9.037011115	protein_coding	17	5402748	5487832	NLRP1
ENSG00000091622	7.575190993	7.699043321	protein_coding	17	6354583	6459877	PITPNM3
ENSG00000091640	7.995363019	8.926097022	protein_coding	17	4862523	4871132	SPAG7
ENSG00000091651	8.116326397	8.594734178	protein_coding	16	46723555	46732306	ORC6
ENSG00000091656	1.960915222	2.242360793	protein_coding	8	77593454	77779521	ZFHX4
ENSG00000091704	0.499066092	0	protein_coding	7	130020180	130027955	CPA1
ENSG00000091732	8.299696963	8.001751392	protein_coding	7	129658126	129691291	ZC3HC1
ENSG00000091831	0.869158192	0.697730409	protein_coding	6	151977826	152450754	ESR1
ENSG00000091844	4.842525946	3.453019579	protein_coding	6	153331857	153452384	RGS17
ENSG00000091879	0.869158192	1.518964905	protein_coding	8	6357172	6420930	ANGPT2
ENSG00000091947	8.124895791	7.892709342	protein_coding	17	42088559	42092417	TMEM101
ENSG00000091986	6.068837212	7.32390599	protein_coding	3	112323407	112368377	CCDC80
ENSG00000092010	10.62212519	11.22948558	protein_coding	14	24605367	24608176	PSME1
ENSG00000092020	7.434062125	7.844724842	protein_coding	14	35554673	35591723	PPP2R3C
ENSG00000092036	7.440941622	8.085067518	protein_coding	14	23415437	23426370	HAUS4
ENSG00000092051	1.407729925	0.697730409	protein_coding	14	24037244	24048009	JPH4
ENSG00000092054	0	0.390640832	protein_coding	14	23881947	23904927	MYH7
ENSG00000092067	0	0.390640832	protein_coding	14	23586513	23588825	CEBPE
ENSG00000092068	4.842525946	3.368666104	protein_coding	14	23594504	23652883	SLC7A8
ENSG00000092094	8.235860718	9.069940847	protein_coding	14	20914570	20923264	OSGEP
ENSG00000092098	8.094677541	8.076782959	protein_coding	14	24615892	24629870	RNF31
ENSG00000092108	9.154055005	9.635231414	protein_coding	14	31091318	31205018	SCFD1
ENSG00000092140	8.448779323	8.898158883	protein_coding	14	31028329	31089269	G2E3
ENSG00000092148	10.60078215	10.69132855	protein_coding	14	31569318	31677010	HECTD1
ENSG00000092199	11.85750144	11.99261667	protein_coding	14	21677295	21737653	HNRNPC

ENSG00000092200	3.448790144	3.183544561	protein_coding	14	21756098	21819460	RPGRIP1
ENSG00000092201	11.38579079	11.68490889	protein_coding	14	21819631	21852425	SUPT16H
ENSG00000092203	9.452682263	9.469195022	protein_coding	14	21944756	21967319	TOX4
ENSG00000092208	6.93675248	7.389591938	protein_coding	14	39583427	39606177	GEMIN2
ENSG00000092295	5.158541613	5.163066621	protein_coding	14	24718320	24733638	TGM1
ENSG00000092330	7.967047117	8.585420545	protein_coding	14	24708849	24711880	TINF2
ENSG00000092421	6.204385613	4.976162367	protein_coding	5	115779312	115910630	SEMA6A
ENSG00000092439	8.966547133	9.075772566	protein_coding	15	50844670	50979012	TRPM7
ENSG00000092445	8.717371959	7.91704449	protein_coding	15	41849873	41871536	TYRO3
ENSG00000092470	8.207901394	8.120964787	protein_coding	15	44119161	44160617	WDR76
ENSG00000092529	1.16343121	2.242360793	protein_coding	15	42640301	42704516	CAPN3
ENSG00000092531	8.171138658	8.696649474	protein_coding	15	42783431	42837547	SNAP23
ENSG00000092607	2.10647801	2.579085888	protein_coding	1	119425666	119532179	TBX15
ENSG00000092621	10.48406996	10.60062679	protein_coding	1	120202421	120286838	PHGDH
ENSG00000092758	3.502389126	2.242360793	protein_coding	20	61447596	61472511	COL9A3
ENSG00000092820	12.043434	11.33363257	protein_coding	6	159186773	159240444	EZR
ENSG00000092841	11.56414825	12.6902668	protein_coding	12	56551945	56557280	MYL6
ENSG00000092847	9.084367772	9.03187762	protein_coding	1	36335409	36395211	EIF2C1
ENSG00000092850	0	0.390640832	protein_coding	1	36549676	36553876	TEKT2
ENSG00000092853	7.73205733	8.0397549	protein_coding	1	36185819	36235568	CLSPN
ENSG00000092871	8.8704485	8.127396674	protein_coding	17	33333009	33416334	RFFL
ENSG00000092929	9.650379479	9.346378541	protein_coding	17	73823310	73840798	UNC13D
ENSG00000092931	6.449220685	6.403479675	protein_coding	17	74733783	74775336	MFSD11
ENSG00000092964	10.93422749	10.67988765	protein_coding	8	26371547	26515694	DPYSL2
ENSG00000092969	9.447567586	9.863317461	protein_coding	1	218519577	218617961	TGFB2
ENSG00000092978	7.363400307	7.53698153	protein_coding	1	217600334	217804424	GPATCH2
ENSG00000093000	9.69925397	9.791222404	protein_coding	22	45559722	45583892	NUP50
ENSG00000093009	8.98901727	9.395168311	protein_coding	22	19466982	19508135	CDC45
ENSG00000093010	9.959126666	10.3244275	protein_coding	22	19929130	19957498	COMT
ENSG00000093072	2.35979773	1.667587519	protein_coding	22	17660194	17739125	CECR1
ENSG00000093100	10.54799358	10.67000842	protein_coding	22	18270418	18507325	
ENSG00000093134	0.499066092	2.144285137	protein_coding	6	133043923	133055904	VNN3
ENSG00000093144	8.728660495	8.75700367	protein_coding	6	127609855	127664754	ECHDC1
ENSG00000093167	8.731468881	8.727671407	protein_coding	3	37094117	37225180	LRRFIP2
ENSG00000093183	8.930339658	8.866788836	protein_coding	3	42589461	42642572	SEC22C
ENSG00000093217	6.965663283	6.926693689	protein_coding	3	38388251	38462839	XYLB
ENSG00000094631	9.376683712	9.814328395	protein_coding	X	48659784	48683392	HDAC6
ENSG00000094755	0.499066092	0	protein_coding	5	170190354	170241051	GABRP
ENSG00000094804	9.637710578	9.678031042	protein_coding	17	38443885	38459171	CDC6
ENSG00000094841	6.804311337	7.309836726	protein_coding	X	74493920	74524435	UPRT
ENSG00000094880	8.956980309	9.003312166	protein_coding	5	137523339	137549032	CDC23
ENSG00000094914	9.022652927	9.385809746	protein_coding	12	53701240	53718648	AAAS
ENSG00000094916	11.52624036	11.57376546	protein_coding	12	54624724	54673955	CBX5
ENSG00000094963	0	0.390640832	protein_coding	1	171154347	171181822	FMO2
ENSG00000094975	8.197783135	8.379906558	protein_coding	1	172501489	172580971	C1orf9
ENSG00000095002	9.168633912	9.310086199	protein_coding	2	47630108	47789450	MSH2
ENSG00000095015	7.680671792	7.490337219	protein_coding	5	56111401	56191979	MAP3K1
ENSG00000095059	9.79766936	10.03691901	protein_coding	19	12786534	12792682	DHPS
ENSG00000095066	9.545769354	9.212881815	protein_coding	19	12873817	12886434	HOOK2
ENSG00000095110	0	0.697730409	protein_coding	11	114392437	114430617	FAM55A
ENSG00000095139	11.50936279	11.37712449	protein_coding	11	118443105	118473730	ARCN1
ENSG00000095203	7.461385447	6.310446475	protein_coding	9	111934254	112083244	EPB41L4B
ENSG00000095209	8.295907473	8.359562039	protein_coding	9	108456825	108538893	TMEM38B
ENSG00000095261	9.507778518	9.117563863	protein_coding	9	123577774	123605229	PSMD5
ENSG00000095303	4.923348194	2.242360793	protein_coding	9	125132824	125157982	PTGS1
ENSG00000095319	11.45708792	11.42555489	protein_coding	9	131709977	131769375	NUP188
ENSG00000095321	9.854808855	9.824260193	protein_coding	9	131857073	131873468	CRAT
ENSG00000095370	3.950976309	2.788380093	protein_coding	9	130500596	130541020	SH2D3C
ENSG00000095380	9.105088364	9.225677649	protein_coding	9	100819021	100845357	NANS
ENSG00000095383	8.637276046	6.664555628	protein_coding	9	100961311	101017915	TBC1D2
ENSG00000095397	7.2307479	7.472765069	protein_coding	9	117164360	117267730	DFNB31
ENSG00000095464	2.238690726	1.802319292	protein_coding	10	95372345	95425767	PDE6C
ENSG00000095485	8.382430368	8.605140645	protein_coding	10	101992050	102027437	CWF19L1

ENSG00000095539	5.649112675	5.317302248	protein_coding	10	102729275	102745628	SEMA4G
ENSG00000095564	9.319038489	9.624472234	protein_coding	10	93683822	93790082	BTAF1
ENSG00000095574	7.91858278	8.053329862	protein_coding	10	124750322	124768333	IKZF5
ENSG00000095585	1.16343121	0	protein_coding	10	97951458	98031333	BLNK
ENSG00000095587	3.950976309	1.667587519	protein_coding	10	98124363	98273675	TLL2
ENSG00000095596	0	0.390640832	protein_coding	10	94833232	94837647	CYP26A1
ENSG00000095637	7.102117104	6.530239731	protein_coding	10	97071528	97321171	SORBS1
ENSG00000095713	0.499066092	0.390640832	protein_coding	10	99624757	99790585	CRTAC1
ENSG00000095739	7.671926738	8.847439299	protein_coding	10	28966271	28971868	BAMBI
ENSG00000095752	6.502803968	6.434933235	protein_coding	19	55875757	55881814	IL11
ENSG00000095777	0	0.390640832	protein_coding	10	26223196	26501456	MYO3A
ENSG00000095787	10.47404372	11.22311871	protein_coding	10	28821422	28909928	WAC
ENSG00000095794	6.998676229	8.677053487	protein_coding	10	35415719	35501886	CREM
ENSG00000095906	8.783815073	9.249460316	protein_coding	16	1832902	1839192	NUBP2
ENSG00000095917	1.616589159	2.788380093	protein_coding	16	1306060	1308532	TPSD1
ENSG00000095932	1.616589159	1.16600992	protein_coding	19	3474405	3480540	C19orf77
ENSG00000095951	8.895705979	9.090661058	protein_coding	6	12008995	12165232	HIVEP1
ENSG00000096060	9.677574326	9.623903733	protein_coding	6	35541362	35696360	FKBP5
ENSG00000096063	10.86462139	10.85795724	protein_coding	6	35800743	35889119	SRPK1
ENSG00000096070	8.741255403	9.090661058	protein_coding	6	36164521	36200567	BRPF3
ENSG00000096080	6.897280837	7.490337219	protein_coding	6	43639040	43655545	MRPS18A
ENSG00000096088	0.499066092	0.390640832	protein_coding	6	41704449	41721847	PGC
ENSG00000096092	7.222786417	7.632773539	protein_coding	6	52535907	52551386	TMEM14A
ENSG00000096093	7.149084042	7.560938532	protein_coding	6	52285106	52387892	EFHC1
ENSG00000096384	13.55762252	13.27139536	protein_coding	6	44214824	44221620	HSP90AB1
ENSG00000096395	0.869158192	1.925536307	protein_coding	6	33762450	33771788	MLN
ENSG00000096401	9.022652927	9.212125573	protein_coding	6	44355262	44418163	CDC5L
ENSG00000096433	12.14303164	11.80950586	protein_coding	6	33588522	33664351	ITPR3
ENSG00000096654	6.652556929	6.720932357	protein_coding	6	27418522	27440897	ZNF184
ENSG00000096696	11.66986148	9.881981209	protein_coding	6	7541808	7586950	DSP
ENSG00000096717	7.82964357	7.551403417	protein_coding	10	69644427	69678147	SIRT1
ENSG00000096746	10.36207036	10.30221393	protein_coding	10	70090931	70102948	HNRNPH3
ENSG00000096872	5.60078458	6.568558581	protein_coding	9	26947037	27062928	IFT74
ENSG00000096968	4.593635215	5.09905904	protein_coding	9	4985033	5128183	JAK2
ENSG00000096996	2.35979773	2.039052734	protein_coding	19	18170371	18197697	IL12RB1
ENSG00000097007	11.11499978	11.25962481	protein_coding	9	133589268	133763062	ABL1
ENSG00000097021	9.89419068	10.11626465	protein_coding	1	6324333	6454451	ACOT7
ENSG00000097033	9.610509348	9.66813905	protein_coding	1	87170259	87213867	SH3GLB1
ENSG00000097046	7.35977471	7.848622884	protein_coding	1	91966404	91991321	CDC7
ENSG00000097096	3.698846687	3.453019579	protein_coding	1	85622556	85666729	SYDE2
ENSG00000099139	5.525114348	3.87606762	protein_coding	9	78505560	78977255	PCSK5
ENSG00000099194	12.92985885	11.54222083	protein_coding	10	102106877	102124591	SCD
ENSG00000099203	9.722731226	10.39942117	protein_coding	19	10943115	10946983	TMED1
ENSG00000099204	10.93696741	10.21280028	protein_coding	10	116190872	116444414	ABLIM1
ENSG00000099219	8.061582684	7.198175438	protein_coding	9	5765076	5833117	ERMP1
ENSG00000099246	9.095310434	10.25746684	protein_coding	10	27793197	27831143	RAB18
ENSG00000099250	9.985245743	10.73304628	protein_coding	10	33466420	33625190	NRP1
ENSG00000099251	3.830412367	3.714580548	pseudogene	10	38645305	38667433	HSD17B7P2
ENSG00000099256	7.671926738	6.651226688	protein_coding	10	25137554	25241533	PRTFDC1
ENSG00000099260	1.616589159	2.242360793	protein_coding	1	100111499	100160097	PALMD
ENSG00000099282	9.256071182	8.056703738	protein_coding	10	71211229	71267425	TSPAN15
ENSG00000099284	1.616589159	1.667587519	protein_coding	10	71812552	71872032	H2AFY2
ENSG00000099290	4.800350936	5.018301917	protein_coding	10	51827648	51893269	FAM21A
ENSG00000099308	8.749591335	8.453421159	protein_coding	19	18208603	18262499	MAST3
ENSG00000099326	8.708847212	9.165228673	protein_coding	19	59073285	59084942	MZF1
ENSG00000099330	7.136425764	7.201227369	protein_coding	19	17337055	17340027	OCEL1
ENSG00000099331	11.05964614	10.87716715	protein_coding	19	17212466	17324103	MYO9B
ENSG00000099337	2.847891871	2.652276565	protein_coding	19	38810482	38819640	KCNK6
ENSG00000099338	3.274897671	2.579085888	protein_coding	19	38826415	38861589	CATSPERG
ENSG00000099341	10.39698226	10.72060396	protein_coding	19	38865176	38874464	PSMD8
ENSG00000099364	9.338510612	9.010289238	protein_coding	16	30934376	30960104	FBXL19
ENSG00000099365	5.331395195	5.516660875	protein_coding	16	31000577	31021949	STX1B
ENSG00000099377	8.15239992	8.704198827	protein_coding	16	30996519	31000473	HSD3B7

ENSG00000099381	10.05640748	9.982080506	protein_coding	16	30968615	30996437	SETD1A
ENSG00000099385	7.68357507	8.073455769	protein_coding	16	30844947	30905696	BCL7C
ENSG00000099399	0	5.42565083	protein_coding	X	30233677	30238206	MAGEB2
ENSG00000099617	1.16343121	1.353254395	protein_coding	19	1286168	1299944	EFNA2
ENSG00000099622	10.59037783	10.94700871	protein_coding	19	1269267	1274806	CIRBP
ENSG00000099624	9.961520764	9.87244022	protein_coding	19	1241749	1244823	ATP5D
ENSG00000099625	4.642199401	3.532712221	protein_coding	19	1229178	1237990	C19orf26
ENSG00000099769	1.960915222	2.039052734	protein_coding	16	1840414	1844972	IGFALS
ENSG00000099783	12.29717648	12.31348272	protein_coding	19	8509651	8553885	HNRNPM
ENSG00000099785	8.239811039	8.140175057	protein_coding	19	8478187	8503895	2-Mar
ENSG00000099795	9.552931438	10.08480023	protein_coding	19	14676892	14682886	NDUFB7
ENSG00000099797	10.3836544	10.20901488	protein_coding	19	14640382	14676791	TECR
ENSG00000099800	9.611271904	9.927841127	protein_coding	19	2425630	2427875	TIMM13
ENSG00000099804	9.645920794	10.01193453	protein_coding	19	531733	542084	CDC34
ENSG00000099810	8.278730502	7.815148897	protein_coding	9	21802635	22032985	MTAP
ENSG00000099812	8.619193517	7.093659051	protein_coding	19	751146	764318	C19orf21
ENSG00000099814	11.13661291	9.844897037	protein_coding	14	105331617	105363089	KIAA0284
ENSG00000099817	11.44494811	11.43157717	protein_coding	19	1086578	1095391	POLR2E
ENSG00000099821	10.9786362	10.91553323	protein_coding	19	617223	633568	POLRMT
ENSG00000099822	4.999881834	4.604259349	protein_coding	19	589893	617157	HCN2
ENSG00000099834	4.593635215	3.368666104	protein_coding	11	616565	626078	CDHR5
ENSG00000099840	4.19837882	6.064512706	protein_coding	19	2096380	2099592	IZUMO4
ENSG00000099849	7.186405829	6.440109466	protein_coding	11	560404	564021	RASSF7
ENSG00000099860	9.256071182	9.20378064	protein_coding	19	2476123	2478257	GADD45B
ENSG00000099864	6.456028446	7.318294738	protein_coding	19	708953	748328	PALM
ENSG00000099866	2.762599152	2.851901313	protein_coding	19	496454	505340	MADCAM1
ENSG00000099869	0.499066092	1.353254395	protein_coding	11	2161731	2169894	IGF2-AS
ENSG00000099875	11.59454479	11.36101092	protein_coding	19	2037481	2051243	MKNK2
ENSG00000099882	0.499066092	0	pseudogene	22	51112843	51171726	
ENSG00000099889	6.861834435	8.001751392	protein_coding	22	19957419	20004331	ARVCF
ENSG00000099899	9.074447906	9.349820553	protein_coding	22	20099389	20104915	TRMT2A
ENSG00000099901	10.28052518	10.24679846	protein_coding	22	20103461	20114878	RANBP1
ENSG00000099904	10.0355387	9.760517066	protein_coding	22	20116979	20135530	ZDHHC8
ENSG00000099910	7.663128351	8.518485187	protein_coding	22	20783528	20850170	KLHL22
ENSG00000099917	11.01510727	10.98977517	protein_coding	22	20850200	20941919	MED15
ENSG00000099937	1.16343121	1.518964905	protein_coding	22	21128167	21142008	SERPIND1
ENSG00000099940	8.263288478	8.143352046	protein_coding	22	21213271	21245502	SNAP29
ENSG00000099942	10.64836751	10.53107824	protein_coding	22	21271714	21308037	CRKL
ENSG00000099949	9.870181217	10.25453171	protein_coding	22	21322090	21353327	LZTR1
ENSG00000099953	5.4452544	6.136428444	protein_coding	22	24110413	24126503	MMP11
ENSG00000099954	7.246540262	7.119700263	protein_coding	22	17840837	18033845	CECR2
ENSG00000099956	10.00336794	10.35009017	protein_coding	22	24129150	24176703	SMARCB1
ENSG00000099957	7.144876945	7.354383726	protein_coding	22	21369316	21383119	P2RX6
ENSG00000099958	5.575999561	5.734544355	protein_coding	22	24176690	24181315	DERL3
ENSG00000099960	3.004694206	1.518964905	protein_coding	22	21383007	21387129	SLC7A4
ENSG00000099968	10.14121895	10.08314681	protein_coding	22	18111621	18213388	BCL2L13
ENSG00000099974	4.409500985	5.137803333	protein_coding	22	24309089	24314721	DDTL
ENSG00000099977	4.409500985	5.09905904	protein_coding	22	24313554	24322660	DDT
ENSG00000099984	4.352265886	5.271576845	protein_coding	22	24322339	24326106	GSTT2
ENSG00000099985	1.616589159	1.518964905	protein_coding	22	30658818	30662829	OSM
ENSG00000099991	10.38231481	10.08438705	protein_coding	22	24407642	24574596	CABIN1
ENSG00000099992	8.369868254	8.414504719	protein_coding	22	30687979	30723035	TBC1D10A
ENSG00000099994	5.072559152	3.411459265	protein_coding	22	24577227	24585078	SUSD2
ENSG00000099995	11.21646228	11.09791564	protein_coding	22	30727979	30752936	SF3A1
ENSG00000099998	3.448790144	1.518964905	protein_coding	22	24615622	24641110	GGT5
ENSG00000099999	8.054871625	8.660517685	protein_coding	22	30773808	30817760	RNF215
ENSG00000100003	9.167597433	8.675956977	protein_coding	22	30792846	30820603	SEC14L2
ENSG00000100012	0.869158192	0	protein_coding	22	30843946	30868036	SEC14L3
ENSG00000100014	8.647720461	9.249460316	protein_coding	22	24666786	24813707	SPECC1L
ENSG00000100023	9.740963079	9.771851712	protein_coding	22	22006559	22052565	PPIL2
ENSG00000100024	3.274897671	1.353254395	protein_coding	22	24863206	24924358	UPB1
ENSG00000100027	3.871730003	4.903073497	protein_coding	22	22051833	22090123	YPEL1
ENSG00000100028	6.298293508	6.485878328	protein_coding	22	24951471	24978020	SNRPD3

ENSG00000100029	11.80531025	12.05235468	protein_coding	22	30972615	31003070	PES1
ENSG00000100030	10.899376	10.83603586	protein_coding	22	22108789	22221970	MAPK1
ENSG00000100031	4.903563137	3.493416095	protein_coding	22	24979718	25024972	GGT1
ENSG00000100033	1.799000381	1.925536307	protein_coding	22	18900294	18924066	PRODH
ENSG00000100034	9.904803739	9.905561226	protein_coding	22	22273793	22307209	PPM1F
ENSG00000100036	7.537174746	7.688207601	protein_coding	22	31031639	31065003	SLC35E4
ENSG00000100038	8.539709228	8.920552509	protein_coding	22	22311397	22337213	TOP3B
ENSG00000100053	3.448790144	2.144285137	protein_coding	22	25595817	25603330	CRYBB3
ENSG00000100055	5.054730169	4.7279204	protein_coding	22	37678068	37711389	CYTH4
ENSG00000100056	7.997697782	8.280968702	protein_coding	22	19117792	19132197	DGCR14
ENSG00000100058	6.171673665	6.445267191	pseudogene	22	25844072	25916821	CRYBB2P1
ENSG00000100060	6.965663283	6.995112131	protein_coding	22	37865101	37882439	MFNG
ENSG00000100065	9.702835781	9.569438796	protein_coding	22	37886400	37915549	CARD10
ENSG00000100068	6.25990527	6.252780742	protein_coding	22	25747385	25801344	LRP5L
ENSG00000100075	10.12368915	9.617635386	protein_coding	22	19163095	19166343	SLC25A1
ENSG00000100077	6.749949642	5.901455548	protein_coding	22	25960816	26118985	ADRBK2
ENSG00000100078	0.499066092	0.697730409	protein_coding	22	31530795	31536593	PLA2G3
ENSG00000100079	4.642199401	3.027231696	protein_coding	22	37966255	37978623	LGALS2
ENSG00000100083	10.01378699	10.07734489	protein_coding	22	38004481	38029571	GGA1
ENSG00000100084	8.185546801	8.14810445	protein_coding	22	19318221	19435224	HIRA
ENSG00000100092	8.177331204	6.142791909	protein_coding	22	38030661	38062939	SH3BP1
ENSG00000100097	12.99762442	13.3537158	protein_coding	22	38071615	38075813	LGALS1
ENSG00000100099	9.252165193	9.293022915	protein_coding	22	26839389	26879820	HPS4
ENSG00000100100	6.782811835	7.329495501	protein_coding	22	31677579	31688520	PIK3IP1
ENSG00000100104	7.37781226	7.621426466	protein_coding	22	26879843	26887899	SRRD
ENSG00000100105	9.078865158	9.189251987	protein_coding	22	31721790	31742218	PATZ1
ENSG00000100106	8.025424129	7.512619979	protein_coding	22	38093011	38172563	TRIOBP
ENSG00000100109	9.473800365	9.433305485	protein_coding	22	26887191	26908471	TFIP11
ENSG00000100116	8.549290825	8.940779054	protein_coding	22	38203912	38213183	GCAT
ENSG00000100124	8.101206377	8.298174303	protein_coding	22	38226862	38240438	ANKRD54
ENSG00000100129	11.52745281	11.46195347	protein_coding	22	38244875	38285414	EIF3L
ENSG00000100138	10.86558043	11.08702611	protein_coding	22	42069934	42086508	NHP2L1
ENSG00000100139	10.49154418	9.92368977	protein_coding	22	38301664	38338829	MICALL1
ENSG00000100142	8.949763328	9.409093306	protein_coding	22	38348614	38437113	POLR2F
ENSG00000100146	1.16343121	1.925536307	protein_coding	22	38366693	38383429	SOX10
ENSG00000100147	6.146643566	5.995870484	protein_coding	22	42196683	42222303	CCDC134
ENSG00000100150	7.824391858	7.905863867	protein_coding	22	32149944	32303001	DEPDC5
ENSG00000100151	7.845284967	7.980592361	protein_coding	22	38452318	38471708	PICK1
ENSG00000100154	8.344411036	8.17939326	protein_coding	22	28374004	29075853	TTC28
ENSG00000100156	2.928422289	3.133298822	protein_coding	22	38474141	38480100	SLC16A8
ENSG00000100162	8.118473522	8.601680156	protein_coding	22	42334725	42343168	CENPM
ENSG00000100167	2.671945279	1.667587519	protein_coding	22	42372276	42394225	3-sep
ENSG00000100170	1.16343121	0	protein_coding	22	32439019	32509016	SLC5A1
ENSG00000100181	0	0.697730409	processed_transc	22	17082777	17179521	TPTEP1
ENSG00000100191	5.684325135	5.816437314	protein_coding	22	32614465	32651328	SLC5A4
ENSG00000100196	8.771587845	8.418443943	protein_coding	22	38864067	38879452	KDELR3
ENSG00000100197	4.491309013	4.342020395	protein_coding	22	42522501	42540472	CYP2D6
ENSG00000100201	11.99444363	12.19390275	protein_coding	22	38879445	38903665	DDX17
ENSG00000100206	4.464551814	4.794098771	protein_coding	22	38914954	38966291	DMC1
ENSG00000100207	10.4075566	10.03990532	protein_coding	22	42556019	42611448	TCF20
ENSG00000100209	6.640654398	6.82352253	protein_coding	22	29138019	29153503	HSCB
ENSG00000100211	7.337827682	7.741590246	protein_coding	22	39052641	39069859	CBY1
ENSG00000100216	10.38632984	10.16318938	protein_coding	22	39077953	39080818	TOMM22
ENSG00000100218	0.499066092	1.353254395	protein_coding	22	23401593	23487208	RTDR1
ENSG00000100219	11.25478858	11.50009186	protein_coding	22	29190543	29196585	XBP1
ENSG00000100220	11.0197097	11.15333184	protein_coding	22	32783569	32808242	C22orf28
ENSG00000100221	9.96986896	10.05516663	protein_coding	22	39081548	39097561	JOSD1
ENSG00000100225	10.92505666	11.05385548	protein_coding	22	32870663	32894818	FBXO7
ENSG00000100226	9.532948302	9.350507971	protein_coding	22	39101728	39134304	GTPBP1
ENSG00000100227	10.22231885	10.25526605	protein_coding	22	42979727	43010968	POLDIP3
ENSG00000100228	4.593635215	5.072639111	protein_coding	22	23487513	23506537	RAB36
ENSG00000100234	0	0.950786998	protein_coding	22	33197687	33259030	TIMP3
ENSG00000100239	9.867630505	10.00365497	protein_coding	22	50781733	50883514	PPP6R2

ENSG00000100241	11.42361729	11.3450603	protein_coding	22	50885184	50913464	SBF1
ENSG00000100242	10.61453887	10.58610742	protein_coding	22	39130730	39190148	SUN2
ENSG00000100243	11.81063176	11.86131148	protein_coding	22	43013846	43045574	CYB5R3
ENSG00000100246	7.402691859	7.219404604	protein_coding	22	39174513	39190203	DNAL4
ENSG00000100253	3.004694206	2.971122874	protein_coding	22	50925213	50929077	MIOX
ENSG00000100258	10.11188316	10.38238014	protein_coding	22	50941376	50946135	LMF2
ENSG00000100263	9.065572619	9.365548888	protein_coding	22	29655841	29664198	RHBDD3
ENSG00000100266	10.23919023	10.14586997	protein_coding	22	43231418	43411151	PACSIN2
ENSG00000100271	6.023726639	6.966698819	protein_coding	22	43414609	43485434	TTLL1
ENSG00000100276	2.10647801	2.971122874	protein_coding	22	29708922	29711745	RASL10A
ENSG00000100280	12.11373306	12.07571958	protein_coding	22	29723669	29819168	AP1B1
ENSG00000100281	8.844740944	8.983949389	protein_coding	22	35653445	35691800	HMGXB4
ENSG00000100284	9.490475063	9.795772355	protein_coding	22	35695268	35743985	TOM1
ENSG00000100285	1.16343121	2.242360793	protein_coding	22	29876181	29887379	NEFH
ENSG00000100288	4.464551814	4.841813558	protein_coding	22	51017378	51039884	CHKB
ENSG00000100290	7.186405829	4.547834947	protein_coding	22	43506754	43525718	BIK
ENSG00000100292	9.323698603	8.983063058	protein_coding	22	35776354	35790207	HMOX1
ENSG00000100294	8.382430368	8.395978102	protein_coding	22	43528886	43539400	MCAT
ENSG00000100296	10.9652776	11.30855749	protein_coding	22	29901868	29951205	THOC5
ENSG00000100297	10.73907363	10.73278266	protein_coding	22	35796056	35820495	MCM5
ENSG00000100298	1.616589159	0.390640832	protein_coding	22	39493229	39500072	APOBEC3H
ENSG00000100299	8.668385155	9.079923694	protein_coding	22	51063446	51066607	ARSA
ENSG00000100300	10.99654928	11.3529719	protein_coding	22	43547520	43559248	TSPO
ENSG00000100302	8.577659618	7.778838337	protein_coding	22	35936915	35950048	RASD2
ENSG00000100304	11.5651325	11.26619728	protein_coding	22	43562628	43583139	TTLL12
ENSG00000100307	6.846372398	7.077141316	protein_coding	22	39516172	39548679	CBX7
ENSG00000100311	7.250461488	6.505763096	protein_coding	22	39619364	39640756	PDGFB
ENSG00000100312	2.762599152	1.353254395	protein_coding	22	51176624	51183762	ACR
ENSG00000100314	2.671945279	2.420525079	protein_coding	22	30116073	30127828	CABP7
ENSG00000100316	13.32652848	13.41279083	protein_coding	22	39708887	39716394	RPL3
ENSG00000100319	7.657232808	8.001751392	protein_coding	22	30126945	30163000	ZMAT5
ENSG00000100320	10.83195583	10.67961414	protein_coding	22	36134783	36424473	RBFOX2
ENSG00000100321	5.239686603	5.80042639	protein_coding	22	39745930	39781593	SYNGR1
ENSG00000100324	8.643253539	8.83570377	protein_coding	22	39795746	39913596	TAB1
ENSG00000100325	10.70411607	10.89306095	protein_coding	22	30184597	30234271	ASCC2
ENSG00000100330	10.27427957	10.4845527	protein_coding	22	30279144	31521442	MTMR3
ENSG00000100335	10.15174033	9.83707015	protein_coding	22	39895437	39914137	SMCR7L
ENSG00000100336	0.499066092	0.390640832	protein_coding	22	36585172	36600886	APOL4
ENSG00000100342	9.357723414	9.938853364	protein_coding	22	36649056	36663576	APOL1
ENSG00000100344	6.414691513	4.903073497	protein_coding	22	44319619	44360368	PNPLA3
ENSG00000100345	15.03514795	15.40942747	protein_coding	22	36677323	36784063	MYH9
ENSG00000100346	3.502389126	2.721932731	protein_coding	22	39966758	40085742	CACNA1I
ENSG00000100347	9.355904607	9.445582628	protein_coding	22	44351301	44406411	SAMM50
ENSG00000100348	9.520012288	9.840499639	protein_coding	22	36863083	36878077	TXN2
ENSG00000100350	10.13274625	10.6252633	protein_coding	22	36883237	36903148	FOXRED2
ENSG00000100351	0.869158192	1.667587519	protein_coding	22	40297086	40369725	GRAP2
ENSG00000100353	11.79796098	11.72389497	protein_coding	22	36906897	36925483	EIF3D
ENSG00000100354	9.123378905	9.491180897	protein_coding	22	40440821	40731811	TNRC6B
ENSG00000100359	9.776749803	9.775951422	protein_coding	22	40766595	40806122	SGSM3
ENSG00000100360	8.120617457	8.576046394	protein_coding	22	37154246	37172300	IFT27
ENSG00000100364	10.89468575	11.00709988	protein_coding	22	45588114	45636650	KIAA0930
ENSG00000100365	0.499066092	1.16600992	protein_coding	22	37257030	37274057	NCF4
ENSG00000100372	8.303476525	8.46235258	protein_coding	22	41165634	41215403	SLC25A17
ENSG00000100373	0.499066092	0	protein_coding	22	45680863	45691755	UPK3A
ENSG00000100376	8.018542337	7.931818465	protein_coding	22	45704849	45737836	FAM118A
ENSG00000100379	8.735671237	8.951693347	protein_coding	22	37447779	37459430	KCTD17
ENSG00000100380	9.787584866	9.934733619	protein_coding	22	41220539	41253026	ST13
ENSG00000100385	3.393122761	3.96493948	protein_coding	22	37515126	37571094	IL2RB
ENSG00000100387	5.948480845	6.695185789	protein_coding	22	41347351	41369313	RBX1
ENSG00000100393	10.48947188	10.27603922	protein_coding	22	41487790	41576081	EP300
ENSG00000100395	9.160321156	9.20301961	protein_coding	22	41601209	41627275	L3MBTL2
ENSG00000100399	2.35979773	2.912743273	protein_coding	22	41625517	41636938	CHADL
ENSG00000100401	12.14868931	11.96460276	protein_coding	22	41641615	41698963	RANGAP1

ENSG00000100403	11.55664594	11.74522334	protein_coding	22	41697526	41756151	ZC3H7B
ENSG00000100410	8.131289597	8.259169069	protein_coding	22	41855721	41864729	PHF5A
ENSG00000100412	9.751377591	9.418302523	protein_coding	22	41865117	41924993	ACO2
ENSG00000100413	9.305909516	9.243551304	protein_coding	22	41921808	41940610	POLR3H
ENSG00000100416	8.961771651	9.41961334	protein_coding	22	46726772	46753237	TRMU
ENSG00000100417	7.67484765	8.120964787	protein_coding	22	41972898	41985894	PMM1
ENSG00000100418	10.27764595	10.4464781	protein_coding	22	41994032	42017100	PPPDE2
ENSG00000100422	8.539709228	8.625731057	protein_coding	22	47080308	47134158	CERK
ENSG00000100425	8.94855698	8.724493	protein_coding	22	50166931	50221160	BRD1
ENSG00000100426	9.194306789	9.11432951	protein_coding	22	50247490	50282090	ZBED4
ENSG00000100427	1.407729925	1.353254395	protein_coding	22	50497820	50524331	MLC1
ENSG00000100429	6.138202758	6.016808232	protein_coding	22	50683612	50689834	HDAC10
ENSG00000100433	2.762599152	0	protein_coding	14	88649113	88793251	KCNK10
ENSG00000100439	9.311550855	9.409753061	protein_coding	14	23067146	23081265	ABHD4
ENSG00000100441	8.967738537	9.411071665	protein_coding	14	24898492	24910540	KHNYN
ENSG00000100442	8.554057865	8.907531839	protein_coding	14	45584803	45604522	FKBP3
ENSG00000100445	5.977159882	6.729413443	protein_coding	14	24908972	24912111	SDR39U1
ENSG00000100461	9.786910053	10.07609858	protein_coding	14	23369854	23388396	RBM23
ENSG00000100462	10.01263302	10.26441394	protein_coding	14	23389720	23398794	PRMT5
ENSG00000100473	2.57521082	1.802319292	protein_coding	14	31343720	31364271	COCH
ENSG00000100478	4.821592551	4.7279204	protein_coding	14	31494312	31562818	AP4S1
ENSG00000100479	7.416718478	7.982367515	protein_coding	14	50110273	50155140	POLE2
ENSG00000100483	6.112579918	6.349483465	protein_coding	14	50575350	50583318	METTL21D
ENSG00000100485	8.316627558	8.458531597	protein_coding	14	50583847	50698276	SOS2
ENSG00000100490	6.482942724	6.198834244	protein_coding	14	50796310	50883179	CDKL1
ENSG00000100503	9.377580398	9.599243793	protein_coding	14	51186481	51297839	NIN
ENSG00000100504	11.60281194	11.06206254	protein_coding	14	51324609	51411454	PYGL
ENSG00000100505	6.965663283	6.787424764	protein_coding	14	51441980	51562779	TRIM9
ENSG00000100519	9.089849478	9.215148166	protein_coding	14	53173890	53195305	PSMC6
ENSG00000100522	8.994872754	9.025865365	protein_coding	14	53241912	53258386	GNPNAT1
ENSG00000100523	7.981274537	8.481307156	protein_coding	14	53510686	53620000	DDHD1
ENSG00000100526	8.507303029	8.608592853	protein_coding	14	54863567	54886936	CDKN3
ENSG00000100528	9.078865158	9.551614431	protein_coding	14	54893654	54908149	CNIH
ENSG00000100532	6.204385613	6.673373662	protein_coding	14	54976530	55005567	CGRRF1
ENSG00000100554	9.177928864	9.982524041	protein_coding	14	67761088	67826982	ATP6V1D
ENSG00000100557	0	0.390640832	protein_coding	14	57936019	57960585	C14orf105
ENSG00000100558	9.165522239	8.551145468	protein_coding	14	67853700	67878917	PLEK2
ENSG00000100564	7.698004462	7.883239271	protein_coding	14	68048672	68067004	PIGH
ENSG00000100567	10.70483073	11.25761057	protein_coding	14	58711549	58738730	PSMA3
ENSG00000100568	9.592086026	9.732839523	protein_coding	14	68113792	68141548	VTI1B
ENSG00000100575	8.359012976	8.599368544	protein_coding	14	58875212	58894332	TIMM9
ENSG00000100577	8.35173048	9.084889332	protein_coding	14	77787227	77797940	GSTZ1
ENSG00000100578	9.223526019	9.590541354	protein_coding	14	58894103	59015216	KIAA0586
ENSG00000100580	10.02241241	10.11302738	protein_coding	14	77801364	77843452	TMED8
ENSG00000100583	4.352265886	4.888000647	protein_coding	14	77843032	77857840	SAMD15
ENSG00000100591	11.38177426	11.7133786	protein_coding	14	77924213	77935817	AHSA1
ENSG00000100592	8.977234519	9.078264676	protein_coding	14	59655364	59838123	DAAM1
ENSG00000100593	1.16343121	0.390640832	protein_coding	14	77940740	77965210	ISM2
ENSG00000100596	10.04234008	10.12673589	protein_coding	14	77972340	78083116	SPTLC2
ENSG00000100599	9.267726094	8.614328238	protein_coding	14	92980118	93155339	RIN3
ENSG00000100600	10.53398641	10.05516663	protein_coding	14	93170152	93215047	LGMN
ENSG00000100601	7.348842929	7.862183683	protein_coding	14	78138747	78174363	ALKBH1
ENSG00000100603	10.27427957	10.55065289	protein_coding	14	78183942	78227550	SNW1
ENSG00000100604	2.10647801	1.16600992	protein_coding	14	93389425	93401638	CHGA
ENSG00000100605	10.64091829	9.147933883	protein_coding	14	93403259	93582665	ITPK1
ENSG00000100612	9.70569484	9.863799047	protein_coding	14	60610838	60636574	DHRS7
ENSG00000100614	9.742356032	10.25893217	protein_coding	14	60712470	60765805	PPM1A
ENSG00000100625	8.925442603	8.755966294	protein_coding	14	61176246	61191066	SIX4
ENSG00000100626	2.35979773	3.608232228	protein_coding	14	69725994	69821183	GALNTL1
ENSG00000100628	3.554067925	2.334191469	protein_coding	14	94400499	94443137	ASB2
ENSG00000100629	8.068262669	8.220584723	protein_coding	14	80943330	81425861	CEP128
ENSG00000100632	10.24608033	10.92733077	protein_coding	14	69846848	69865344	ERH
ENSG00000100644	12.43868722	11.4940384	protein_coding	14	62162231	62214976	HIF1A

ENSG00000100647	9.430386506	9.510378177	protein_coding	14	70078313	70181859	KIAA0247
ENSG00000100650	11.1505816	11.3196583	protein_coding	14	70193617	70238722	SRSF5
ENSG00000100652	2.671945279	1.353254395	protein_coding	14	70242134	70264006	SLC10A1
ENSG00000100664	11.56690245	10.68343859	protein_coding	14	103799881	103811362	EIF5
ENSG00000100665	0.499066092	0.390640832	protein_coding	14	95027428	95036250	SERPINA4
ENSG00000100697	10.18541435	9.494294562	protein_coding	14	95552565	95624347	DICER1
ENSG00000100711	8.415986232	7.946442679	protein_coding	14	104182067	104200005	ZFYVE21
ENSG00000100714	11.68967225	12.00238191	protein_coding	14	64854749	64926725	MTHFD1
ENSG00000100722	10.18848346	9.865723786	protein_coding	14	89029253	89079853	ZC3H14
ENSG00000100726	9.849648119	9.931061721	protein_coding	16	1543345	1560458	TELO2
ENSG00000100731	10.73207945	10.96911003	protein_coding	14	71374122	71582099	PCNX
ENSG00000100739	7.517784241	5.886365696	protein_coding	14	96722161	96735304	BDKRB1
ENSG00000100744	8.271030151	7.410839058	protein_coding	14	96829814	96853625	C14orf129
ENSG00000100749	8.487504052	8.044860491	protein_coding	14	97263641	97398059	VRK1
ENSG00000100764	8.715954662	8.305283181	protein_coding	14	90722839	90738968	PSMC1
ENSG00000100767	7.840089987	7.955508102	protein_coding	14	73704205	73741348	PAPLN
ENSG00000100784	5.538004898	5.059245468	protein_coding	14	91336799	91526980	RPS6KA5
ENSG00000100796	9.547364003	8.393311914	protein_coding	14	91923955	91976898	SMEK1
ENSG00000100802	6.400645076	6.89311646	protein_coding	14	23456110	23479375	C14orf93
ENSG00000100804	10.29531035	10.80276749	protein_coding	14	23485752	23504439	PSMB5
ENSG00000100811	10.82769711	10.00496543	protein_coding	14	100704635	100749129	YY1
ENSG00000100813	10.89625086	10.99791518	protein_coding	14	23527773	23564823	ACIN1
ENSG00000100814	9.219530792	9.500501806	protein_coding	14	20779527	20801471	CCNB1IP1
ENSG00000100815	8.834328271	7.95731437	protein_coding	14	92432335	92507241	TRIP11
ENSG00000100823	10.86941024	11.34074226	protein_coding	14	20923350	20925927	APEX1
ENSG00000100836	9.865714507	10.02059883	protein_coding	14	23790498	23795394	PABPN1
ENSG00000100852	10.2292898	10.2059794	protein_coding	14	32545320	32628934	ARHGAP5
ENSG00000100865	8.251597515	7.41347308	protein_coding	14	102808956	102829253	CINP
ENSG00000100867	12.09040513	12.4476313	protein_coding	14	24099324	24114848	DHRS2
ENSG00000100883	9.401582613	9.763100953	protein_coding	14	35451163	35498773	SRP54
ENSG00000100884	0	0.390640832	protein_coding	14	24540046	24547309	CPNE6
ENSG00000100888	10.44482396	10.30647154	protein_coding	14	21853353	21924285	CHD8
ENSG00000100889	8.936437694	9.890987074	protein_coding	14	24563262	24579807	PCK2
ENSG00000100890	3.554067925	3.679994897	protein_coding	14	35591052	35743271	
ENSG00000100897	8.766120079	8.900038352	protein_coding	14	24583404	24594451	DCAF11
ENSG00000100902	7.348842929	7.955508102	protein_coding	14	35747839	35786699	PSMA6
ENSG00000100906	10.4388224	10.94632677	protein_coding	14	35870717	35873955	NFKBIA
ENSG00000100908	6.771940731	7.318294738	protein_coding	14	24608174	24610797	FAM158A
ENSG00000100911	10.03610671	10.82494899	protein_coding	14	24612574	24616779	PSME2
ENSG00000100916	6.664362065	7.359856617	protein_coding	14	36295524	36401531	BRMS1L
ENSG00000100918	6.907250452	7.289906366	protein_coding	14	24641062	24649463	REC8
ENSG00000100926	6.535310252	6.605885938	protein_coding	14	24658349	24682679	TM9SF1
ENSG00000100934	10.59037783	10.41659153	protein_coding	14	39501123	39578850	SEC23A
ENSG00000100938	9.316235179	9.570029165	protein_coding	14	24701628	24708448	GMPR2
ENSG00000100941	10.3122922	10.69809579	protein_coding	14	39644387	39652422	PNN
ENSG00000100949	8.195750939	8.605140645	protein_coding	14	24734744	24740945	RABGGTA
ENSG00000100968	6.032862302	8.312357201	protein_coding	14	24834879	24848810	NFATC4
ENSG00000100979	11.27658409	10.63517057	protein_coding	20	44527399	44540794	PLTP
ENSG00000100982	8.697401955	8.352716275	protein_coding	20	44563267	44576662	PCIF1
ENSG00000100983	10.13857657	10.38271622	protein_coding	20	33516236	33543620	GSS
ENSG00000100985	7.250461488	8.056703738	protein_coding	20	44637547	44645200	MMP9
ENSG00000100987	1.16343121	0.950786998	protein_coding	20	25051521	25062996	VSX1
ENSG00000100991	10.91706117	11.52314052	protein_coding	20	33590207	33680674	TRPC4AP
ENSG00000100994	11.85105969	11.57655918	protein_coding	20	25228705	25278650	PYGB
ENSG00000100997	9.200400892	9.59692827	protein_coding	20	25275379	25371619	ABHD12
ENSG00000101000	10.11618743	10.73383684	protein_coding	20	33758727	33765165	PROCR
ENSG00000101003	8.728660495	9.028445114	protein_coding	20	25388363	25433264	GINS1
ENSG00000101004	8.292108003	8.32500368	protein_coding	20	25433341	25566153	NINL
ENSG00000101017	8.500733514	9.109464345	protein_coding	20	44746911	44758502	CD40
ENSG00000101019	9.015757872	9.44751161	protein_coding	20	33890369	33999944	UQCC
ENSG00000101040	9.945282964	9.942505509	protein_coding	20	45837859	45985567	ZMYND8
ENSG00000101049	1.799000381	2.039052734	protein_coding	20	42187608	42216877	SGK2
ENSG00000101052	7.491518048	7.93548844	protein_coding	20	42219571	42275936	IFT52

ENSG00000101057	12.16799437	11.80662815	protein_coding	20	42295754	42345136	MYBL2
ENSG00000101076	0	0.697730409	protein_coding	20	42984340	43060030	HNF4A
ENSG00000101079	9.392739397	9.368945519	protein_coding	20	35280169	35374481	NDRG3
ENSG00000101082	0.499066092	1.16600992	protein_coding	20	35240721	35274619	SLA2
ENSG00000101084	6.897280837	7.462626902	protein_coding	20	35234137	35240960	C20orf24
ENSG00000101096	3.554067925	5.41517489	protein_coding	20	50003494	50179339	NFATC2
ENSG00000101098	0	0.390640832	protein_coding	20	43380449	43438979	RIMS4
ENSG00000101104	8.681514984	8.743459249	protein_coding	20	43538703	43587676	PABPC1L
ENSG00000101109	9.014605485	8.975061478	protein_coding	20	43595115	43708600	STK4
ENSG00000101115	5.375152132	4.406904905	protein_coding	20	50400581	50419014	SALL4
ENSG00000101126	10.76431847	10.60149333	protein_coding	20	49505585	49547958	ADNP
ENSG00000101132	7.498129565	8.244450803	protein_coding	20	52824386	52844591	PFDN4
ENSG00000101138	8.753741308	8.919626348	protein_coding	20	54967427	54979518	CSTF1
ENSG00000101144	2.57521082	4.961837817	protein_coding	20	55743804	55841685	BMP7
ENSG00000101146	9.145657575	9.320649169	protein_coding	20	55926066	55954267	RAE1
ENSG00000101150	10.95720266	10.81576885	protein_coding	20	62496596	62522898	TPD52L2
ENSG00000101152	10.77558713	10.2975873	protein_coding	20	62526518	62567384	DNAJC5
ENSG00000101158	10.4529292	10.53319908	protein_coding	20	57556296	57570188	TH1L
ENSG00000101160	10.22879299	10.13673322	protein_coding	20	57570240	57582302	CTSZ
ENSG00000101161	10.8798901	10.62639901	protein_coding	20	62612488	62664453	PRPF6
ENSG00000101162	1.407729925	2.334191469	protein_coding	20	57594309	57601709	TUBB1
ENSG00000101166	9.88413009	9.797285822	protein_coding	20	57608200	57617964	SLMO2
ENSG00000101180	0.499066092	0.390640832	protein_coding	20	60790026	60795323	HRH3
ENSG00000101181	9.221529789	9.189251987	protein_coding	20	60758085	60778624	GTPBP5
ENSG00000101182	11.05740958	11.4514184	protein_coding	20	60711791	60718496	PSMA7
ENSG00000101187	10.57482477	10.57320837	protein_coding	20	61273797	61317137	SLCO4A1
ENSG00000101188	1.16343121	1.518964905	protein_coding	20	61340189	61394123	NTSR1
ENSG00000101189	9.156146748	9.150304507	protein_coding	20	61427805	61431945	C20orf20
ENSG00000101190	7.858191159	8.053329862	protein_coding	20	61472467	61493115	TCFL5
ENSG00000101191	10.17719799	10.1024558	protein_coding	20	61509090	61569304	DIDO1
ENSG00000101193	9.330197577	8.89344947	protein_coding	20	61569471	61578019	C20orf11
ENSG00000101194	7.792473675	7.304170377	protein_coding	20	61584052	61599949	SLC17A9
ENSG00000101197	1.407729925	0.697730409	protein_coding	20	61867235	61871859	BIRC7
ENSG00000101198	1.16343121	1.16600992	protein_coding	20	61872136	61904046	NKAIN4
ENSG00000101199	10.71231338	11.05132081	protein_coding	20	61904137	61921142	ARFGAP1
ENSG00000101203	3.211941663	3.493416095	protein_coding	20	61924538	61966203	COL20A1
ENSG00000101210	11.50936279	11.64973533	protein_coding	20	62119366	62130505	EEF1A2
ENSG00000101213	7.671926738	7.490337219	protein_coding	20	62159778	62168723	PTK6
ENSG00000101216	8.848626402	8.511125832	protein_coding	20	62218955	62258394	GMEB2
ENSG00000101220	10.67341097	10.84411243	protein_coding	20	3734155	3749034	C20orf27
ENSG00000101222	2.928422289	3.027231696	protein_coding	20	3758151	3762095	SPEF1
ENSG00000101224	11.62261323	11.55941784	protein_coding	20	3767578	3786762	CDC25B
ENSG00000101230	5.498982484	5.112089758	protein_coding	20	13202418	13281298	ISM1
ENSG00000101236	8.750975986	9.132029383	protein_coding	20	3912068	3996229	RNF24
ENSG00000101246	8.798620088	9.045526555	protein_coding	20	62329996	62339355	ARFRP1
ENSG00000101247	7.621344476	8.032919217	protein_coding	20	13765596	13799067	C20orf7
ENSG00000101255	10.80321277	10.65060297	protein_coding	20	361261	378203	TRIB3
ENSG00000101265	4.322773689	4.622587519	protein_coding	20	4760669	4804291	RASSF2
ENSG00000101266	11.00150208	11.1390794	protein_coding	20	459116	524482	CSNK2A1
ENSG00000101276	6.652556929	5.150490275	protein_coding	20	740724	749131	C20orf54
ENSG00000101278	2.10647801	2.334191469	pseudogene	20	820090	820620	
ENSG00000101280	2.847891871	3.081239798	protein_coding	20	853296	896977	ANGPT4
ENSG00000101282	0.869158192	2.579085888	protein_coding	20	939095	982907	RSPO4
ENSG00000101290	9.921500929	9.720129478	protein_coding	20	5107432	5178533	CDS2
ENSG00000101294	11.20741307	11.52375014	protein_coding	20	30102231	30157370	HM13
ENSG00000101298	7.916116295	8.391976969	protein_coding	20	1246960	1289972	SNPH
ENSG00000101306	5.346128795	5.717592784	protein_coding	20	30407111	30422492	MYLK2
ENSG00000101307	4.77879189	4.710890357	protein_coding	20	1544167	1600707	SIRPB1
ENSG00000101310	10.26655536	10.22634605	protein_coding	20	18488137	18542059	SEC23B
ENSG00000101311	10.11457483	9.422885181	protein_coding	20	6055492	6104191	FERMT1
ENSG00000101323	0.499066092	0.950786998	protein_coding	20	7863628	7921121	HAO1
ENSG00000101331	0	0.697730409	protein_coding	20	30598245	30619984	C20orf160
ENSG00000101333	1.407729925	2.721932731	protein_coding	20	9049410	9461889	PLCB4

ENSG00000101335	8.179389493	10.87525763	protein_coding	20	35169887	35178228	MYL9
ENSG00000101337	11.03456701	11.07626155	protein_coding	20	30697309	30755061	TM9SF4
ENSG00000101342	3.211941663	4.363974406	protein_coding	20	35504534	35522638	C20orf118
ENSG00000101343	9.074447906	9.294452589	protein_coding	20	20015012	20036690	CRNKL1
ENSG00000101346	10.20978632	10.50352879	protein_coding	20	30795683	30826470	POFUT1
ENSG00000101347	9.169669647	10.17487997	protein_coding	20	35518632	35580246	SAMHD1
ENSG00000101350	10.5685563	10.36547554	protein_coding	20	30865467	30922814	KIF3B
ENSG00000101353	3.077135474	4.048652948	protein_coding	20	35729629	35807991	C20orf132
ENSG00000101361	11.72990091	12.33535115	protein_coding	20	2632791	2639039	NOP56
ENSG00000101363	10.02183898	10.24495109	protein_coding	20	35918041	35945663	MANBAL
ENSG00000101365	10.56462462	11.02766156	protein_coding	20	2639041	2644865	IDH3B
ENSG00000101367	10.91211876	11.11197052	protein_coding	20	31407699	31438211	MAPRE1
ENSG00000101384	10.71834247	10.05811545	protein_coding	20	10618332	10654608	JAG1
ENSG00000101391	9.4269256	9.414362912	protein_coding	20	31946645	31989367	CDK5RAP1
ENSG00000101400	8.312882337	8.124184315	protein_coding	20	31995761	32031698	SNTA1
ENSG00000101405	0.499066092	0.697730409	protein_coding	20	3052266	3053163	OXT
ENSG00000101407	10.16374581	10.25966428	protein_coding	20	36611409	36661870	TTI1
ENSG00000101412	9.707835423	9.922303325	protein_coding	20	32263489	32274210	E2F1
ENSG00000101413	9.237422823	9.275031414	protein_coding	20	36661948	36720768	RPRD1B
ENSG00000101417	6.830742851	6.923001245	protein_coding	20	32294512	32308125	PXMP4
ENSG00000101421	10.9164443	10.77896351	protein_coding	20	32399110	32442172	CHMP4B
ENSG00000101439	9.896694904	10.74040803	protein_coding	20	23608534	23619110	CST3
ENSG00000101440	3.211941663	2.420525079	protein_coding	20	32782375	32857150	ASIP
ENSG00000101441	0.499066092	0	protein_coding	20	23666277	23669677	CST4
ENSG00000101442	7.609180529	7.764467022	protein_coding	20	37377085	37400834	ACTR5
ENSG00000101443	8.646233023	7.655203404	protein_coding	20	44098346	44110172	WFDC2
ENSG00000101444	12.29219201	12.22009251	protein_coding	20	32868074	32899608	AHCY
ENSG00000101445	0	0.390640832	protein_coding	20	37434348	37551667	PPP1R16B
ENSG00000101447	10.15697238	10.49857555	protein_coding	20	37554955	37581703	FAM83D
ENSG00000101448	0.869158192	1.353254395	protein_coding	20	44165625	44176391	SPINLW1
ENSG00000101452	8.278730502	8.419754632	protein_coding	20	37590942	37668366	DHX35
ENSG00000101457	9.045401313	9.394501851	protein_coding	20	44420576	44440066	DNTTIP1
ENSG00000101460	4.981125677	3.96493948	protein_coding	20	33134658	33148149	MAP1LC3A
ENSG00000101464	8.558809206	8.524589435	protein_coding	20	33148346	33264910	PIGU
ENSG00000101470	0.869158192	1.16600992	protein_coding	20	44451853	44462384	TNNC2
ENSG00000101473	7.524276744	7.661864922	protein_coding	20	44470360	44486045	ACOT8
ENSG00000101474	10.64241121	11.29165499	protein_coding	20	24943561	24973615	C20orf3
ENSG00000101493	8.35173048	8.051639961	protein_coding	18	74069644	74207146	ZNF516
ENSG00000101542	0	0.390640832	protein_coding	18	59000988	59223006	CDH20
ENSG00000101544	9.081068723	8.948972504	protein_coding	18	77866915	77905406	ADNP2
ENSG00000101546	7.440941622	7.309836726	protein_coding	18	77794346	77810651	RBFA
ENSG00000101557	9.947700127	9.715334193	protein_coding	18	158483	213739	USP14
ENSG00000101558	10.27572327	10.07817516	protein_coding	18	9913999	9960018	VAPA
ENSG00000101574	7.062518763	7.485338407	protein_coding	18	2537524	2571508	METTL4
ENSG00000101577	8.718787866	8.825850709	protein_coding	18	2916992	3013313	LPIN2
ENSG00000101596	9.378476527	9.537194665	protein_coding	18	2655886	2805015	SMCHD1
ENSG00000101605	2.847891871	3.993387124	protein_coding	18	3066805	3220106	MYOM1
ENSG00000101608	10.58572949	10.77436015	protein_coding	18	3247528	3256234	MYL12A
ENSG00000101624	6.393570237	6.642271819	protein_coding	18	12672631	12702773	CEP76
ENSG00000101638	1.616589159	2.334191469	protein_coding	18	44259081	44337132	ST8SIA5
ENSG00000101639	8.45218826	8.614328238	protein_coding	18	12991361	13125051	CEP192
ENSG00000101654	9.478823042	10.01106523	protein_coding	18	13726660	13764557	RNMT
ENSG00000101665	7.850461307	7.881337775	protein_coding	18	46446223	46477081	SMAD7
ENSG00000101670	10.08303831	8.262094789	protein_coding	18	47088427	47119278	LIPG
ENSG00000101680	0.499066092	0	protein_coding	18	6941743	7117813	LAMA1
ENSG00000101695	6.820228284	6.655683365	protein_coding	18	29598335	29653176	RNF125
ENSG00000101745	8.072698871	7.699043321	protein_coding	18	9136758	9285206	ANKRD12
ENSG00000101746	0	0.390640832	protein_coding	18	31431064	31803515	NOL4
ENSG00000101751	6.846372398	7.18590274	protein_coding	18	51795889	51821101	POLI
ENSG00000101752	9.900443107	9.841477998	protein_coding	18	19321281	19450918	MIB1
ENSG00000101773	8.920528869	9.021555505	protein_coding	18	20513290	20606449	RBBP8
ENSG00000101782	9.251187042	8.996301187	protein_coding	18	21032787	21063104	RIOK3
ENSG00000101811	9.143550557	9.267771518	protein_coding	X	100075384	100096485	CSTF2

ENSG00000101842	1.799000381	2.144285137	protein_coding	X	107288200	107322414	VSIG1
ENSG00000101843	8.674235392	9.176127473	protein_coding	X	107327437	107334848	PSMD10
ENSG00000101844	7.46813618	7.586839732	protein_coding	X	107334898	107397901	ATG4A
ENSG00000101846	7.842689815	8.740315472	protein_coding	X	7137497	7272851	STS
ENSG00000101849	9.674658935	9.352568262	protein_coding	X	9431335	9687780	TBL1X
ENSG00000101850	0.499066092	1.925536307	protein_coding	X	9693386	9754337	GPR143
ENSG00000101856	9.503677432	9.812333804	protein_coding	X	118370216	118378429	PGRMC1
ENSG00000101868	8.759256101	9.091483708	protein_coding	X	24712036	25015103	POLA1
ENSG00000101871	8.814599602	8.728729322	protein_coding	X	10413350	10851773	MID1
ENSG00000101882	8.241782151	8.667154767	protein_coding	X	119059014	119077735	NKAP
ENSG00000101888	7.33043718	7.55379311	protein_coding	X	108779010	108787919	NXT2
ENSG00000101890	0.499066092	0	protein_coding	X	108616135	108725301	GUCY2F
ENSG00000101892	0.869158192	0	protein_coding	X	119495967	119516226	ATP1B4
ENSG00000101898	5.072559152	5.072639111	pseudogene	20	30135307	30135852	
ENSG00000101901	8.261346552	8.635917101	protein_coding	X	110909043	111003877	ALG13
ENSG00000101911	10.44268341	10.24864348	protein_coding	X	12809474	12842341	PRPS2
ENSG00000101928	7.743231983	8.524589435	protein_coding	X	134021656	134049297	MOSPD1
ENSG00000101935	8.846037259	8.576046394	protein_coding	X	109437414	109683461	AMMECR1
ENSG00000101940	9.974025012	10.66310783	protein_coding	X	48448430	48463581	WDR13
ENSG00000101945	8.33889698	8.519708104	protein_coding	X	48553945	48567403	SUV39H1
ENSG00000101958	0.499066092	0	protein_coding	X	14547420	14749934	GLRA2
ENSG00000101966	9.485492858	9.503595441	protein_coding	X	122993574	123047829	XIAP
ENSG00000101972	10.67960449	10.68643641	protein_coding	X	123094062	123556514	STAG2
ENSG00000101974	9.203438318	9.307964293	protein_coding	X	138808505	139027435	ATP11C
ENSG00000101977	0	0.697730409	protein_coding	X	138663929	138790386	MCF2
ENSG00000101986	8.831713317	8.973277306	protein_coding	X	152990323	153010216	ABCD1
ENSG00000101997	8.790563466	9.185404214	protein_coding	X	49091927	49106987	CCDC22
ENSG00000102001	1.16343121	1.353254395	protein_coding	X	49061523	49089833	CACNA1F
ENSG00000102003	3.830412367	5.112089758	protein_coding	X	49044269	49056718	SYP
ENSG00000102007	10.57247728	10.99087786	protein_coding	X	49028273	49031588	PLP2
ENSG00000102021	0.499066092	0	protein_coding	X	114524293	114542121	LUZP4
ENSG00000102024	11.41839834	11.93156176	protein_coding	X	114795501	114885181	PLS3
ENSG00000102030	8.616157604	9.245030827	protein_coding	X	153194695	153200676	NAA10
ENSG00000102032	1.616589159	4.7279204	protein_coding	X	153200716	153210232	RENBP
ENSG00000102034	9.940436455	9.470460424	protein_coding	X	129198849	129244691	ELF4
ENSG00000102038	8.53006357	8.66273344	protein_coding	X	128580480	128657477	SMARCA1
ENSG00000102043	2.847891871	2.579085888	protein_coding	X	63444187	63615333	MTMR8
ENSG00000102048	4.543379137	4.872768657	protein_coding	X	15252900	15288589	ASB9
ENSG00000102053	4.543379137	4.101879561	protein_coding	X	64708615	64727767	ZC3H12B
ENSG00000102054	10.50514714	10.86663337	protein_coding	X	16857406	16888537	RBBP7
ENSG00000102057	5.223818625	4.42790041	protein_coding	X	48818639	48827976	KCND1
ENSG00000102069	0.869158192	1.16600992	protein_coding	X	142967173	142968355	UBE2NL
ENSG00000102078	6.171673665	6.803579823	protein_coding	X	129473874	129507335	SLC25A14
ENSG00000102081	9.652603664	10.03606464	protein_coding	X	146993469	147032645	FMR1
ENSG00000102096	8.547698305	8.229580833	protein_coding	X	48770459	48776301	PIM2
ENSG00000102098	6.350376764	6.505763096	protein_coding	X	18257434	18372847	SCML2
ENSG00000102100	8.970118399	9.18617459	protein_coding	X	48760459	48769235	SLC35A2
ENSG00000102103	9.893563944	10.43388431	protein_coding	X	48755195	48760420	PQBP1
ENSG00000102104	0	0.697730409	protein_coding	X	18658030	18690229	RS1
ENSG00000102109	3.950976309	7.126137778	protein_coding	X	48689504	48694035	PCSK1N
ENSG00000102119	9.99813007	9.870524428	protein_coding	X	153607557	153609883	EMD
ENSG00000102125	8.520352988	8.598211347	protein_coding	X	153639854	153650065	TAZ
ENSG00000102128	0.869158192	0	protein_coding	X	102192200	102193228	RAB40AL
ENSG00000102144	13.22867268	13.64876991	protein_coding	X	77320685	77384793	PGK1
ENSG00000102145	0.499066092	0.950786998	protein_coding	X	48644962	48652718	GATA1
ENSG00000102158	10.18592632	10.34388978	protein_coding	X	77081861	77151090	MAGT1
ENSG00000102172	10.93605468	11.1380844	protein_coding	X	21958691	22025798	SMS
ENSG00000102174	3.78787645	3.679994897	protein_coding	X	22050559	22269427	PHEX
ENSG00000102178	9.770619129	9.749613921	protein_coding	X	153712056	153715009	UBL4A
ENSG00000102181	9.585892264	9.833632489	protein_coding	X	149934810	150067289	CD99L2
ENSG00000102189	8.829093615	9.189251987	protein_coding	12	93164413	93323107	EEA1
ENSG00000102195	0.499066092	0	protein_coding	X	150345056	150349937	GPR50
ENSG00000102218	7.46813618	7.48283249	protein_coding	X	46696375	46741793	RP2

ENSG00000102221	8.255505039	8.567793748	protein_coding	X	46771711	46920641	PHF16
ENSG00000102225	11.22669965	11.22799002	protein_coding	X	47077259	47089396	CDK16
ENSG00000102226	10.16166506	10.15021936	protein_coding	X	47092089	47107727	USP11
ENSG00000102230	0	0.390640832	protein_coding	X	24576204	24690794	PCYT1B
ENSG00000102241	9.974025012	10.25636687	protein_coding	X	135579238	135594505	HTATSF1
ENSG00000102265	10.25537886	11.04028549	protein_coding	X	47441712	47446188	TIMP1
ENSG00000102271	7.186405829	7.164171052	protein_coding	X	86772752	86925050	KLHL4
ENSG00000102302	9.168633912	9.304420832	protein_coding	X	54471887	54522599	FGD1
ENSG00000102309	6.830742851	7.332282157	protein_coding	X	71401203	71522776	PIN4
ENSG00000102312	8.237837231	8.229580833	protein_coding	X	48367350	48379202	PORCN
ENSG00000102316	10.08742937	10.66116973	protein_coding	X	54834032	54842445	MAGED2
ENSG00000102317	10.79920289	10.54135041	protein_coding	X	48432837	48437454	RBM3
ENSG00000102349	3.004694206	2.242360793	protein_coding	X	56258854	56314322	KLF8
ENSG00000102359	7.093410923	8.226588358	protein_coding	X	99899215	99926296	SRPX2
ENSG00000102362	8.523597116	8.590084877	protein_coding	X	99929488	99987110	SYTL4
ENSG00000102384	7.698004462	8.024328814	protein_coding	X	100353178	100418670	CENPI
ENSG00000102385	3.211941663	3.96493948	protein_coding	X	100474758	100519486	DRP2
ENSG00000102387	1.407729925	2.851901313	protein_coding	X	100523241	100548059	TAF7L
ENSG00000102390	8.133414584	8.528239625	protein_coding	X	75392771	75398039	CXorf26
ENSG00000102393	8.767488965	8.98660512	protein_coding	X	100652791	100662913	GLA
ENSG00000102401	9.03179537	9.545623701	protein_coding	X	100877787	100882833	ARMCX3
ENSG00000102409	0	0.950786998	protein_coding	X	102470020	102472174	BEX4
ENSG00000102445	0.499066092	0	protein_coding	13	46916139	47012325	KIAA0226L
ENSG00000102452	4.642199401	5.187895122	protein_coding	13	101706130	102068843	NALCN
ENSG00000102471	7.908691438	7.641787375	protein_coding	13	80055287	80130210	NDFIP2
ENSG00000102524	0.869158192	0.697730409	protein_coding	13	108903588	108959385	TNFSF13B
ENSG00000102531	8.710271506	8.418443943	protein_coding	13	49550048	49783888	FNDC3A
ENSG00000102539	0	0.390640832	protein_coding	13	49794474	49796513	MLNR
ENSG00000102543	5.538004898	4.917990497	protein_coding	13	49822047	49867618	CDADC1
ENSG00000102547	5.239686603	4.857374129	protein_coding	13	49882786	50018262	CAB39L
ENSG00000102554	7.923503136	6.167968749	protein_coding	13	73629114	73651676	KLF5
ENSG00000102572	3.698846687	3.32456471	protein_coding	13	99102455	99230194	STK24
ENSG00000102575	5.223818625	3.64455972	protein_coding	19	11685477	11689801	ACP5
ENSG00000102580	7.19863491	7.083771121	protein_coding	13	96329411	96443301	DNAJC3
ENSG00000102595	7.54038131	7.716212954	protein_coding	13	96453834	96705736	UGGT2
ENSG00000102606	8.836938493	8.612036819	protein_coding	13	111766906	111958084	ARHGEF7
ENSG00000102683	0.499066092	0.390640832	protein_coding	13	23755091	23899304	SGCG
ENSG00000102699	9.833400793	9.814826612	protein_coding	13	24995064	25086948	PARP4
ENSG00000102710	8.80263159	8.385283657	protein_coding	13	37583449	37633850	FAM48A
ENSG00000102738	6.93675248	6.708116507	protein_coding	13	41303432	41345309	MRPS31
ENSG00000102743	7.572060955	6.82352253	protein_coding	13	41363548	41386596	SLC25A15
ENSG00000102753	9.143550557	8.516036237	protein_coding	13	50273447	50367057	KPNA3
ENSG00000102760	2.671945279	0	protein_coding	13	42031695	42045018	C13orf15
ENSG00000102763	7.712290964	7.029859489	protein_coding	13	42140961	42535256	KIAA0564
ENSG00000102780	5.649112675	5.09905904	protein_coding	13	42614176	42830714	DGKH
ENSG00000102781	7.427149666	7.077141316	protein_coding	13	30776767	30881621	KATNAL1
ENSG00000102786	7.603059879	6.963107483	protein_coding	13	51928213	52028400	INTS6
ENSG00000102794	0.869158192	0.697730409	protein_coding	13	77522632	77532777	IRG1
ENSG00000102796	3.502389126	3.87606762	protein_coding	13	52342129	52378293	DHRS12
ENSG00000102802	0.499066092	0	protein_coding	13	31480328	31499709	C13orf33
ENSG00000102804	10.2924605	9.033590816	protein_coding	13	45007655	45151283	TSC22D1
ENSG00000102805	6.917151646	7.332282157	protein_coding	13	77564795	77576652	CLN5
ENSG00000102854	3.004694206	3.411459265	protein_coding	16	693262	818865	MSLN
ENSG00000102858	10.07973621	10.11059465	protein_coding	16	4666494	4740975	MGRN1
ENSG00000102870	7.90122817	7.646273261	protein_coding	16	30789778	30798523	ZNF629
ENSG00000102871	8.15239992	8.080102494	protein_coding	16	67188083	67194201	TRADD
ENSG00000102878	7.097770581	7.524852175	protein_coding	16	67197288	67203848	HSF4
ENSG00000102879	5.806547963	5.072639111	protein_coding	16	30194148	30200397	CORO1A
ENSG00000102882	9.316235179	8.810943783	protein_coding	16	30125426	30134827	MAPK3
ENSG00000102886	4.800350936	3.96493948	protein_coding	16	30116131	30125177	GDPD3
ENSG00000102890	8.205883411	5.004391715	protein_coding	16	67233014	67237932	ELMO3
ENSG00000102893	9.520824196	9.418302523	protein_coding	16	47495034	47735434	PHKB
ENSG00000102897	8.663981856	8.621180739	protein_coding	16	20911190	20936328	LYRM1

ENSG00000102898	10.68214706	10.75164095	protein_coding	16	67880635	67906470	NUTF2
ENSG00000102900	10.40887296	10.77972931	protein_coding	16	56764017	56880792	NUP93
ENSG00000102901	8.872994239	9.654843329	protein_coding	16	67862060	67881714	CENPT
ENSG00000102904	4.131913373	5.32851069	protein_coding	16	67840668	67861971	TSNAXIP1
ENSG00000102908	9.674658935	9.788181112	protein_coding	16	69598997	69738569	NFAT5
ENSG00000102910	9.756212088	9.871003614	protein_coding	16	48278207	48395747	LONP2
ENSG00000102921	9.228504544	9.712663243	protein_coding	16	48572637	48654059	N4BP1
ENSG00000102931	8.775675113	9.115947593	protein_coding	16	57279010	57287516	ARL2BP
ENSG00000102934	5.575999561	4.048652948	protein_coding	16	57290004	57318599	PLLP
ENSG00000102935	5.158541613	4.250702764	protein_coding	16	49521435	49891830	ZNF423
ENSG00000102962	1.616589159	0.950786998	protein_coding	16	57392684	57400102	CCL22
ENSG00000102967	7.524276744	7.32390599	protein_coding	16	72042643	72058954	DHODH
ENSG00000102970	1.16343121	0.390640832	protein_coding	16	57438679	57449974	CCL17
ENSG00000102974	9.465390183	9.478029618	protein_coding	16	67596310	67673086	CTCF
ENSG00000102977	9.419108071	9.396500307	protein_coding	16	67691433	67694713	ACD
ENSG00000102978	9.358631958	9.661506506	protein_coding	16	57496299	57505922	POLR2C
ENSG00000102981	3.077135474	4.566889334	protein_coding	16	67694851	67696680	PARD6A
ENSG00000102984	4.942865586	5.175534283	protein_coding	16	71893583	71929239	ZNF821
ENSG00000102996	9.345859976	9.045526555	protein_coding	16	58059470	58080805	MMP15
ENSG00000103005	10.73383117	10.43031225	protein_coding	16	58033450	58055522	C16orf57
ENSG00000103018	9.96986896	9.561148156	protein_coding	16	69458428	69500169	CYB5B
ENSG00000103021	7.842689815	7.436965343	protein_coding	16	58265061	58317740	CCDC113
ENSG00000103023	0	0.390640832	protein_coding	16	58313901	58328951	PRSS54
ENSG00000103024	8.497437504	8.581912372	protein_coding	16	1820287	1821731	NME3
ENSG00000103034	6.364918751	6.223059835	protein_coding	16	58496750	58547532	NDRG4
ENSG00000103035	10.1252916	10.16982567	protein_coding	16	74330673	74340186	PSMD7
ENSG00000103037	7.803191723	8.613182984	protein_coding	16	58549383	58554431	SETD6
ENSG00000103042	8.599344916	9.011159004	protein_coding	16	58699013	58719008	SLC38A7
ENSG00000103043	10.09399102	10.32722395	protein_coding	16	70721342	70835064	VAC14
ENSG00000103044	8.362640485	5.486957566	protein_coding	16	69139467	69152622	HAS3
ENSG00000103047	8.731468881	8.492561507	protein_coding	16	68877507	69119083	TMCO7
ENSG00000103051	9.441577455	9.709451562	protein_coding	16	70514471	70557468	COG4
ENSG00000103056	4.437288965	5.085909553	protein_coding	16	68392230	68482591	SMPD3
ENSG00000103061	6.0776925	6.002883551	protein_coding	16	68318406	68344849	SLC7A6OS
ENSG00000103064	8.866621448	8.807943825	protein_coding	16	68298433	68335722	SLC7A6
ENSG00000103066	7.726437377	7.799123587	protein_coding	16	68269105	68294961	PLA2G15
ENSG00000103067	8.760631512	7.774746834	protein_coding	16	68259871	68272005	ESRP2
ENSG00000103089	3.077135474	2.851901313	protein_coding	16	74746853	74808729	FA2H
ENSG00000103091	9.510233586	9.329741599	protein_coding	16	74907468	75034071	WDR59
ENSG00000103111	9.3522601	9.405128432	protein_coding	16	77224732	77236302	MON1B
ENSG00000103121	8.054871625	8.24888207	protein_coding	16	81009698	81053875	C16orf61
ENSG00000103126	9.467076146	9.53960799	protein_coding	16	337440	402673	AXIN1
ENSG00000103145	8.555643385	9.324153043	protein_coding	16	3072626	3074287	HCFC1R1
ENSG00000103148	10.3836544	10.07276981	protein_coding	16	134273	188859	NPRL3
ENSG00000103150	6.535310252	6.577981353	protein_coding	16	83932731	83949787	MLYCD
ENSG00000103152	7.296703257	8.058387721	protein_coding	16	127006	135852	MPG
ENSG00000103154	4.821592551	4.075511708	protein_coding	16	84002237	84036381	NECAB2
ENSG00000103160	8.549290825	8.501254594	protein_coding	16	84155886	84178797	HSDL1
ENSG00000103168	9.639954365	9.80533094	protein_coding	16	84211458	84220669	TAF1C
ENSG00000103174	8.500733514	8.847439299	protein_coding	16	5074845	5084142	NAGPA
ENSG00000103175	0.499066092	0	protein_coding	16	84328252	84363450	WFDC1
ENSG00000103184	0.499066092	0.950786998	protein_coding	16	5008318	5069159	SEC14L5
ENSG00000103187	13.47430901	12.8074285	protein_coding	16	84599200	84651683	COTL1
ENSG00000103194	10.45165245	10.32407756	protein_coding	16	84733584	84813528	USP10
ENSG00000103196	6.548110424	5.564846512	protein_coding	16	84853590	84954374	CRISPLD2
ENSG00000103197	10.32629262	10.49826541	protein_coding	16	2097466	2138716	TSC2
ENSG00000103199	7.434062125	7.532142019	protein_coding	16	4798240	4817219	ZNF500
ENSG00000103202	10.32164089	10.26951153	protein_coding	16	446725	460367	NME4
ENSG00000103222	10.24706195	10.01973474	protein_coding	16	16043434	16236931	ABCC1
ENSG00000103226	5.588445295	6.037446454	protein_coding	16	16326452	16388668	NOMO3
ENSG00000103227	7.935731106	7.782918269	protein_coding	16	903634	1031318	LMF1
ENSG00000103241	5.018397273	3.608232228	protein_coding	16	86544133	86548076	FOXF1
ENSG00000103245	8.316627558	8.464894292	protein_coding	16	779753	791329	NARFL

ENSG00000103248	7.990682129	8.159132889	protein_coding	16	86563782	86588841	MTHFSD
ENSG00000103249	10.41150208	10.58201568	protein_coding	16	1494935	1525581	CLCN7
ENSG00000103253	7.430610036	7.260948155	protein_coding	16	776936	779733	HAGHL
ENSG00000103254	7.311793575	7.705505903	protein_coding	16	770581	772601	FAM173A
ENSG00000103257	13.59216843	13.15261038	protein_coding	16	87863629	87903094	SLC7A5
ENSG00000103260	9.74513791	9.759482214	protein_coding	16	765115	769499	METRN
ENSG00000103264	8.805299741	8.949880022	protein_coding	16	87362942	87425748	FBXO31
ENSG00000103266	9.154055005	9.128827334	protein_coding	16	730224	732799	STUB1
ENSG00000103269	4.491309013	4.15321211	protein_coding	16	725666	728268	RHBDL1
ENSG00000103274	7.071412799	7.563312496	protein_coding	16	10837678	10863208	NUBP1
ENSG00000103275	9.960324212	10.13593598	protein_coding	16	1355548	1377019	UBE2I
ENSG00000103310	0.869158192	0	protein_coding	16	21208771	21222868	ZP2
ENSG00000103313	7.08025234	5.271576845	protein_coding	16	3292027	3306627	MEFV
ENSG00000103316	1.960915222	2.851901313	protein_coding	16	21269839	21314372	CRYM
ENSG00000103319	9.462857538	9.064085459	protein_coding	16	22217603	22298554	EEF2K
ENSG00000103326	10.62023235	10.52500144	protein_coding	16	577717	604636	SOLH
ENSG00000103335	11.89383682	12.0755117	protein_coding	16	88781751	88851619	PIEZO1
ENSG00000103342	11.85299524	11.74522334	protein_coding	16	11961985	12009939	GSPT1
ENSG00000103343	7.190493718	7.301328834	protein_coding	16	3451190	3459370	ZNF174
ENSG00000103351	7.30049066	7.53698153	protein_coding	16	3550963	3586581	CLUAP1
ENSG00000103353	9.687732048	9.722786654	protein_coding	16	23568392	23585710	UBFD1
ENSG00000103355	0.499066092	0.390640832	protein_coding	16	2833954	2836708	PRSS33
ENSG00000103356	9.156146748	9.001562612	protein_coding	16	23533335	23569052	EARS2
ENSG00000103363	9.924571906	10.5962863	protein_coding	16	2821415	2827278	TCEB2
ENSG00000103365	9.968084123	10.05221177	protein_coding	16	23474863	23533316	GGA2
ENSG00000103375	1.16343121	0.950786998	protein_coding	16	25227052	25240261	AQP8
ENSG00000103381	9.348606364	9.733893656	protein_coding	16	12756919	12897874	CPPED1
ENSG00000103404	9.492959727	9.644229954	protein_coding	16	23072727	23160591	USP31
ENSG00000103415	9.390075828	9.563521776	protein_coding	16	4524719	4560348	HMOX2
ENSG00000103423	10.03837651	10.22971271	protein_coding	16	4475806	4506776	DNAJA3
ENSG00000103426	9.132969064	8.14493792	protein_coding	16	4404543	4466639	CORO7
ENSG00000103429	9.74513791	9.844409099	protein_coding	16	14726672	14763093	BFAR
ENSG00000103472	0.869158192	0.390640832	pseudogene	16	29086163	29128039	RRN3P2
ENSG00000103479	8.622223055	8.945336714	protein_coding	16	53467889	53525561	RBL2
ENSG00000103485	9.048782844	8.76630664	protein_coding	16	29674600	29710020	QPRT
ENSG00000103490	4.543379137	1.667587519	protein_coding	16	31212806	31214771	PYCARD
ENSG00000103494	6.782811835	7.485338407	protein_coding	16	53631595	53737850	RPGRIP1L
ENSG00000103495	9.187164314	9.520187397	protein_coding	16	29817427	29823649	MAZ
ENSG00000103496	9.272554655	9.552212138	protein_coding	16	31044210	31054296	STX4
ENSG00000103502	9.87399885	9.91813598	protein_coding	16	29869678	29875057	CDIPT
ENSG00000103507	10.1097262	10.4040641	protein_coding	16	31117428	31124110	BCKDK
ENSG00000103510	8.572970055	8.663840043	protein_coding	16	31127075	31142714	KAT8
ENSG00000103512	8.53328596	9.069105817	protein_coding	16	14927538	14990017	NOMO1
ENSG00000103522	3.871730003	2.144285137	protein_coding	16	27413483	27462115	IL21R
ENSG00000103528	6.188122351	2.579085888	protein_coding	16	19179293	19279652	SYT17
ENSG00000103534	8.557227164	6.530239731	protein_coding	16	19421818	19510435	TMC5
ENSG00000103540	8.54291017	8.409235632	protein_coding	16	19535133	19564730	CCP110
ENSG00000103544	8.607011344	8.925174415	protein_coding	16	19566562	19718116	C16orf62
ENSG00000103546	0	0.390640832	protein_coding	16	55689516	55740104	SLC6A2
ENSG00000103549	10.04064274	9.665931589	protein_coding	16	30773066	30787628	RNF40
ENSG00000103550	7.893726093	8.060069742	protein_coding	16	19716722	19729557	C16orf88
ENSG00000103591	9.412998348	9.382452634	protein_coding	15	67493371	67547533	AAGAB
ENSG00000103599	4.292665995	4.226940739	protein_coding	15	67547138	67794598	IQCH
ENSG00000103642	8.009315251	8.774525884	protein_coding	15	63413999	63434260	LACTB
ENSG00000103647	3.744048221	2.721932731	protein_coding	15	68871308	69020145	CORO2B
ENSG00000103653	9.553725034	9.452002561	protein_coding	15	75074398	75095539	CSK
ENSG00000103657	9.161362874	9.155820862	protein_coding	15	63900817	64126147	HERC1
ENSG00000103671	7.556308086	7.752033772	protein_coding	15	64679947	64747502	TRIP4
ENSG00000103707	7.003331296	6.771086752	protein_coding	15	65294845	65321977	MTFMT
ENSG00000103723	1.616589159	1.802319292	protein_coding	15	83328033	83378666	AP3B2
ENSG00000103740	2.35979773	2.788380093	protein_coding	15	78459810	78538030	ACSBG1
ENSG00000103742	2.57521082	2.501982735	protein_coding	15	65673802	65715410	IGDCC4
ENSG00000103769	9.689899429	9.830679389	protein_coding	15	66018392	66184329	RAB11A

ENSG00000103811	7.993024472	6.520498852	protein_coding	15	79213400	79241916	CTSH
ENSG00000103832	0.499066092	0.697730409	pseudogene	15	31083776	31094062	
ENSG00000103852	7.402691859	7.551403417	protein_coding	15	99676528	99791428	TTC23
ENSG00000103855	10.47236588	10.22933902	protein_coding	15	73976307	74006859	CD276
ENSG00000103876	8.447071828	7.711939664	protein_coding	15	80444832	80479288	FAH
ENSG00000103888	7.481543613	8.283850589	protein_coding	15	81071684	81244117	KIAA1199
ENSG00000103932	9.630205981	8.988372895	protein_coding	15	41809374	41836467	RPAP1
ENSG00000103942	8.721615518	8.649387576	protein_coding	15	83509838	83654661	HOMER2
ENSG00000103966	10.24165476	9.652615414	protein_coding	15	42190950	42264776	EHD4
ENSG00000103978	8.619193517	8.439273392	protein_coding	15	42502730	42565861	TMEM87A
ENSG00000103994	9.728365606	9.41961334	protein_coding	15	42705021	42783336	ZFP106
ENSG00000103995	8.063812784	7.752033772	protein_coding	15	49005125	49103343	CEP152
ENSG00000104043	0.499066092	0.697730409	protein_coding	15	50150435	50475014	ATP8B4
ENSG00000104047	6.699208722	7.026422172	protein_coding	15	49913177	49937333	DTWD1
ENSG00000104055	1.407729925	1.353254395	protein_coding	15	43524793	43559055	TGM5
ENSG00000104059	1.407729925	0	protein_coding	15	29412457	29862927	FAM189A1
ENSG00000104064	8.261346552	8.226588358	protein_coding	15	50569389	50647605	GABPB1
ENSG00000104067	10.95329854	10.36785414	protein_coding	15	29991571	30261068	TJP1
ENSG00000104081	7.218789138	7.512619979	protein_coding	15	40380091	40401093	BMF
ENSG00000104093	8.324088959	8.309531754	protein_coding	15	51739908	51915030	DMXL2
ENSG00000104112	0.499066092	0	protein_coding	15	51973550	52013223	SCG3
ENSG00000104129	6.956090577	6.673373662	protein_coding	15	41060067	41099675	DNAJC17
ENSG00000104131	10.19511088	9.920915547	protein_coding	15	44829255	44855227	EIF3J
ENSG00000104133	9.211506993	8.882083758	protein_coding	15	44854894	44955876	SPG11
ENSG00000104140	6.699208722	5.150490275	protein_coding	15	41164412	41166487	RHOV
ENSG00000104142	8.886916071	8.984835176	protein_coding	15	41186628	41196173	VPS18
ENSG00000104147	6.032862302	6.775188634	protein_coding	15	41601466	41624819	OIP5
ENSG00000104154	5.550781289	6.515603638	protein_coding	15	45771809	45815005	SLC30A4
ENSG00000104164	8.617676359	8.275187599	protein_coding	15	45879321	45908197	PLDN
ENSG00000104177	1.799000381	0	protein_coding	15	48431625	48470714	MYEF2
ENSG00000104205	6.798966424	5.742946029	protein_coding	8	67579831	67774257	SGK3
ENSG00000104213	4.883502971	6.084484284	protein_coding	8	17433942	17501580	PDGFRL
ENSG00000104218	7.68357507	7.532142019	protein_coding	8	67974661	68108498	CSPP1
ENSG00000104219	8.600881464	9.133627746	protein_coding	8	17013538	17082308	ZDHHC2
ENSG00000104221	5.763291843	5.683080141	protein_coding	8	37700786	37707459	BRF2
ENSG00000104228	8.325948295	8.116121984	protein_coding	8	27142404	27168836	TRIM35
ENSG00000104231	8.48251156	7.803146634	protein_coding	8	82613569	82645138	ZFAND1
ENSG00000104237	3.78787645	4.29708397	protein_coding	8	55471729	55682531	RP1
ENSG00000104267	2.57521082	1.353254395	protein_coding	8	86376081	86393693	CA2
ENSG00000104290	7.524276744	7.699043321	protein_coding	8	28351729	28431775	FZD3
ENSG00000104299	8.166995477	8.157562549	protein_coding	8	28625178	28747759	INTS9
ENSG00000104312	8.523597116	8.513583123	protein_coding	8	90769975	90803291	RIPK2
ENSG00000104313	0	0.697730409	protein_coding	8	72109668	72274467	EYA1
ENSG00000104320	9.293610409	9.434602747	protein_coding	8	90945564	91015456	NBN
ENSG00000104321	0.869158192	1.925536307	protein_coding	8	72932152	72987852	TRPA1
ENSG00000104324	4.568725997	6.539915281	protein_coding	8	97657455	98161882	
ENSG00000104325	7.876068031	8.476276902	protein_coding	8	91013633	91064320	DECR1
ENSG00000104327	2.238690726	0	protein_coding	8	91070836	91107703	CALB1
ENSG00000104331	9.925798469	10.19070543	protein_coding	8	57870492	57906403	IMPAD1
ENSG00000104341	11.18837626	10.9630546	protein_coding	8	98787285	98865241	LAPTM4B
ENSG00000104343	7.624369505	7.373448694	protein_coding	8	74692332	74791145	UBE2W
ENSG00000104356	9.296457985	9.003312166	protein_coding	8	99129525	99172062	POP1
ENSG00000104361	6.220467583	6.167968749	protein_coding	8	99202061	99306621	NIPAL2
ENSG00000104365	8.629769183	9.113519788	protein_coding	8	42128820	42189973	IKBKB
ENSG00000104368	8.950968668	9.096409785	protein_coding	8	42032236	42065242	PLAT
ENSG00000104369	6.155035276	0.950786998	protein_coding	8	75146935	75233563	JPH1
ENSG00000104375	7.289098491	6.89311646	protein_coding	8	99413631	99955055	STK3
ENSG00000104381	6.622614428	6.142791909	protein_coding	8	75233365	75401107	GDAP1
ENSG00000104388	9.728365606	9.806333433	protein_coding	8	61429416	61536186	RAB2A
ENSG00000104408	10.75261554	11.26528623	protein_coding	8	109213445	109447562	EIF3E
ENSG00000104412	7.565780433	7.635032283	protein_coding	8	109455830	109499145	TTC35
ENSG00000104413	8.508940744	0	protein_coding	8	95653302	95719694	ESRP1
ENSG00000104419	10.78034131	12.19361545	protein_coding	8	134249414	134314265	NDRG1

ENSG00000104427	4.981125677	5.018301917	protein_coding	8	79578282	79629935	FAM164A
ENSG00000104432	5.107569429	5.187895122	protein_coding	8	79587978	79717758	IL7
ENSG00000104435	0.499066092	0	protein_coding	8	80523049	80578397	STMN2
ENSG00000104442	9.144604451	8.788797839	protein_coding	8	66514694	66546442	ARMC1
ENSG00000104447	6.121171528	6.716673044	protein_coding	8	116420724	116821899	TRPS1
ENSG00000104450	6.26766507	6.091080508	protein_coding	8	101170134	101271506	SPAG1
ENSG00000104472	8.020839917	7.752033772	protein_coding	8	141521397	141527236	CHRAC1
ENSG00000104490	0.869158192	0.390640832	protein_coding	8	102698771	103137135	NCALD
ENSG00000104497	6.0776925	5.629711952	protein_coding	8	82711816	82755101	SNX16
ENSG00000104517	10.09726068	9.827720232	protein_coding	8	103264501	103425069	UBR5
ENSG00000104518	9.1498624	9.317839931	protein_coding	8	144635377	144645232	GSDMD
ENSG00000104522	9.219530792	9.679673133	protein_coding	8	144694788	144700218	TSTA3
ENSG00000104524	7.893726093	8.240005883	protein_coding	8	144686083	144691943	PYCRL
ENSG00000104529	11.22918559	11.32613823	protein_coding	8	144661867	144681711	EEF1D
ENSG00000104537	0.869158192	0	protein_coding	8	124693034	124749647	ANXA13
ENSG00000104549	9.832093067	9.111898978	protein_coding	8	126010739	126034525	SQLE
ENSG00000104611	8.894453552	8.721307575	protein_coding	8	19171128	19253729	SH2D4A
ENSG00000104613	9.215524471	9.436546455	protein_coding	8	19674651	19709594	INTS10
ENSG00000104626	8.380642463	8.272288337	protein_coding	8	8859657	8974256	ERI1
ENSG00000104635	11.67898277	11.79796044	protein_coding	8	22224762	22291642	SLC39A14
ENSG00000104643	7.250461488	7.298481685	protein_coding	8	11141925	11185646	MTMR9
ENSG00000104660	8.255505039	8.440565301	protein_coding	8	29952914	30034724	LEPROTL1
ENSG00000104671	6.560798025	7.18590274	protein_coding	8	30013813	30041156	DCTN6
ENSG00000104679	7.161632221	7.389591938	protein_coding	8	23127633	23153792	R3HCC1
ENSG00000104687	9.326487463	7.76240224	protein_coding	8	30535583	30585443	GSR
ENSG00000104689	8.276809264	7.922602475	protein_coding	8	23047965	23082639	TNFRSF10A
ENSG00000104691	5.986594255	6.803579823	processed_transc	8	30589764	30624522	UBXN8
ENSG00000104695	9.106170722	9.14238723	protein_coding	8	30631973	30671830	PPP2CB
ENSG00000104714	7.686472517	7.688207601	protein_coding	8	564744	688106	ERICH1
ENSG00000104723	9.340351466	9.711058296	protein_coding	8	15274724	15624158	TUSC3
ENSG00000104728	9.086562954	8.909399145	protein_coding	8	1772142	1906807	ARHGEF10
ENSG00000104731	8.213938461	8.238521194	protein_coding	16	87730091	87799598	KLHDC4
ENSG00000104738	11.16207636	11.21313751	protein_coding	8	48872745	48890720	MCM4
ENSG00000104756	8.440221571	8.220584723	protein_coding	8	25285366	25315992	KCTD9
ENSG00000104763	8.344411036	8.932538804	protein_coding	8	17913934	17942494	ASAH1
ENSG00000104765	10.00452934	10.23828088	protein_coding	8	26240414	26363152	BNIP3L
ENSG00000104774	10.77524694	11.32805909	protein_coding	19	12757325	12777556	MAN2B1
ENSG00000104783	8.15239992	6.847095496	protein_coding	19	44270685	44285409	KCNN4
ENSG00000104804	3.652182994	3.993387124	protein_coding	19	49384222	49401990	TULP2
ENSG00000104805	10.54759526	10.84971493	protein_coding	19	49403307	49426528	NUCB1
ENSG00000104808	1.407729925	0.697730409	protein_coding	19	49436939	49448226	DHDH
ENSG00000104812	10.06867432	10.15416199	protein_coding	19	49471382	49496567	GYS1
ENSG00000104814	2.762599152	3.232099092	protein_coding	19	39078281	39108643	MAP4K1
ENSG00000104818	2.238690726	3.748356452	protein_coding	19	49535130	49536495	CGB2
ENSG00000104823	6.892270075	6.349483465	protein_coding	19	39306068	39322497	ECH1
ENSG00000104824	11.41009619	11.27636199	protein_coding	19	39327029	39342987	HNRNPL
ENSG00000104825	7.847875458	8.16070152	protein_coding	19	39390604	39399533	NFKBIB
ENSG00000104826	3.211941663	2.144285137	protein_coding	19	49519237	49520347	LHB
ENSG00000104827	0.869158192	0.697730409	protein_coding	19	49526128	49527632	CGB
ENSG00000104833	2.671945279	2.039052734	protein_coding	19	6494331	6502330	TUBB4A
ENSG00000104835	8.576098123	8.573693309	protein_coding	19	39405904	39466380	FBXO17
ENSG00000104848	4.999881834	3.570966319	protein_coding	19	49570675	49576198	KCNA7
ENSG00000104852	11.15294026	11.15962135	protein_coding	19	49588465	49611869	SNRNP70
ENSG00000104853	10.36071057	10.69620416	protein_coding	19	45457848	45496598	CLPTM1
ENSG00000104856	9.582785382	9.832156695	protein_coding	19	45504695	45541452	RELB
ENSG00000104859	9.100750797	9.252405772	protein_coding	19	45542298	45574214	CLASRP
ENSG00000104863	3.393122761	3.781359661	protein_coding	19	49617581	49621717	LIN7B
ENSG00000104866	8.766120079	9.026725794	protein_coding	19	45595050	45651335	PPP1R37
ENSG00000104870	5.707331524	6.002883551	protein_coding	19	50015536	50029685	FCGRT
ENSG00000104872	9.41997878	9.844897037	protein_coding	19	49949550	49955115	PIH1D1
ENSG00000104879	1.16343121	0.390640832	protein_coding	19	45809672	45826233	CKM
ENSG00000104880	10.21932096	9.942961378	protein_coding	19	7459999	7537370	ARHGEF18
ENSG00000104881	8.866621448	8.500015927	protein_coding	19	45882892	45909607	PPP1R13L

ENSG00000104883	5.239686603	4.932754837	protein_coding	19	7541761	7553905	PEX11G
ENSG00000104884	8.625246244	8.444434101	protein_coding	19	45854246	45873876	ERCC2
ENSG00000104885	10.87291199	10.93834688	protein_coding	19	2164148	2232577	DOT1L
ENSG00000104886	8.953376332	8.871585941	protein_coding	19	2233155	2236319	PLEKHJ1
ENSG00000104888	3.077135474	2.851901313	protein_coding	19	49932656	49945617	SLC17A7
ENSG00000104889	10.20978632	10.66863094	protein_coding	19	12917428	12924462	RNASEH2A
ENSG00000104892	6.171673665	5.018301917	protein_coding	19	45843998	45854778	KLC3
ENSG00000104894	4.6658819	4.075511708	protein_coding	19	49838428	49843859	CD37
ENSG00000104897	10.92505666	10.88478007	protein_coding	19	2236816	2248677	SF3A2
ENSG00000104899	8.576098123	9.336696989	protein_coding	19	2249113	2252072	AMH
ENSG00000104901	2.35979773	3.368666104	protein_coding	19	49867042	49878371	DKKL1
ENSG00000104903	5.637181576	5.784235782	protein_coding	19	13209848	13213681	LYL1
ENSG00000104904	12.91171191	13.24215198	protein_coding	19	2269520	2273487	OAZ1
ENSG00000104907	10.74464467	11.25063056	protein_coding	19	13215716	13227563	TRMT1
ENSG00000104915	10.28722112	9.841477998	protein_coding	19	13254903	13261052	STX10
ENSG00000104936	8.727254249	8.535512415	protein_coding	19	46272978	46285815	DMPK
ENSG00000104938	0.869158192	0	protein_coding	19	7828035	7834490	CLEC4M
ENSG00000104941	0.499066092	0	protein_coding	19	46298968	46318605	RSPH6A
ENSG00000104946	7.692250012	7.664078611	protein_coding	19	50380682	50392006	TBC1D17
ENSG00000104951	3.744048221	4.932754837	protein_coding	19	50392916	50432796	IL4I1
ENSG00000104953	3.554067925	3.608232228	protein_coding	19	2977444	2995177	TLE6
ENSG00000104957	8.070482475	8.390640789	protein_coding	19	13842574	13874106	CCDC130
ENSG00000104960	9.657041774	9.549819822	protein_coding	19	50354138	50363990	PTOV1
ENSG00000104964	11.43550666	11.5870971	protein_coding	19	3052908	3062964	AES
ENSG00000104967	1.16343121	0.697730409	protein_coding	19	46442771	46476657	NOVA2
ENSG00000104969	11.27007994	11.37222466	protein_coding	19	2754712	2783354	SGTA
ENSG00000104972	0	0.390640832	protein_coding	19	55085346	55148979	LILRB1
ENSG00000104973	9.429522058	9.29944532	protein_coding	19	50321536	50342036	MED25
ENSG00000104976	8.368064704	8.060069742	protein_coding	19	7985194	7998843	SNAPC2
ENSG00000104979	9.98230137	10.42379484	protein_coding	19	13885257	13889582	C19orf53
ENSG00000104980	9.915339294	10.33835587	protein_coding	19	7991604	8008708	TIMM44
ENSG00000104983	4.842525946	4.932754837	protein_coding	19	46498339	46521874	CCDC61
ENSG00000104998	9.164483521	8.962525691	protein_coding	19	14142262	14164026	IL27RA
ENSG00000105011	9.886021803	10.04288547	protein_coding	19	14230321	14247768	ASF1B
ENSG00000105048	10.09126063	10.17332668	protein_coding	19	55644162	55660606	TNNT1
ENSG00000105053	7.484876093	7.465168131	protein_coding	19	50479726	50528805	VRK3
ENSG00000105058	9.852875742	10.23157969	protein_coding	19	16296235	16302855	FAM32A
ENSG00000105063	11.48606281	11.07563806	protein_coding	19	55741148	55770363	PPP6R1
ENSG00000105072	7.511262389	7.575124073	protein_coding	19	16607205	16632163	C19orf44
ENSG00000105085	7.348842929	7.329495501	protein_coding	19	16685718	16739015	MED26
ENSG00000105088	5.239686603	3.96493948	protein_coding	19	9964395	10047070	OLFM2
ENSG00000105122	0	0.390640832	protein_coding	19	15562438	15575382	RASAL3
ENSG00000105127	9.4441477	9.323452948	protein_coding	19	15464341	15490603	AKAP8
ENSG00000105131	5.417620896	4.075511708	protein_coding	19	15337730	15343858	EPHX3
ENSG00000105132	3.274897671	4.048652948	processed_transc	19	58053208	58071231	ZNF550
ENSG00000105135	9.665877245	10.04033143	protein_coding	19	15225795	15236596	ILVBL
ENSG00000105136	6.9512803	7.266786485	protein_coding	19	57986988	58006048	ZNF419
ENSG00000105137	8.889432943	9.787165921	protein_coding	19	15218214	15225789	SYDE1
ENSG00000105141	0	0.390640832	protein_coding	19	15160291	15166900	CASP14
ENSG00000105146	3.989024711	3.368666104	protein_coding	19	57742377	57746915	AURKC
ENSG00000105171	8.016241092	8.165397208	protein_coding	19	30097170	30108162	POP4
ENSG00000105173	7.491518048	7.495318771	protein_coding	19	30302901	30315216	CCNE1
ENSG00000105176	9.559268022	9.577682064	protein_coding	19	30414551	30506611	URI1
ENSG00000105185	8.981959173	9.830186618	protein_coding	19	33072096	33078322	PDCD5
ENSG00000105186	9.27062517	8.765275934	protein_coding	19	33087907	33166102	ANKRD27
ENSG00000105193	10.80354643	11.2101107	protein_coding	19	39923852	39926618	RPS16
ENSG00000105197	8.625246244	8.887777807	protein_coding	19	39971052	39981528	TIMM50
ENSG00000105202	10.27042264	10.22522208	protein_coding	19	40325094	40337054	FBL
ENSG00000105204	5.977159882	6.568558581	protein_coding	19	40315993	40324841	DYRK1B
ENSG00000105219	2.847891871	1.353254395	protein_coding	19	40728115	40732597	CNTD2
ENSG00000105220	12.6832119	12.20230485	protein_coding	19	34855645	34893318	GPI
ENSG00000105221	9.549752678	9.416334062	protein_coding	19	40736224	40791302	AKT2
ENSG00000105223	9.633212506	10.2541644	protein_coding	19	40854491	40886346	PLD3

ENSG00000105227	5.054730169	4.777836593	protein_coding	19	40899672	40919273	PRX
ENSG00000105229	9.255095676	9.160532436	protein_coding	19	4007748	4039383	PIAS4
ENSG00000105245	7.700873103	6.827478203	protein_coding	19	41171810	41196556	NUMBL
ENSG00000105246	7.402691859	8.508664347	protein_coding	19	4229540	4237515	EBI3
ENSG00000105248	8.830404061	8.935290787	protein_coding	19	4247087	4269083	CCDC94
ENSG00000105251	2.847891871	2.144285137	protein_coding	19	4278598	4290721	SHD
ENSG00000105254	9.02723139	9.242070261	protein_coding	19	36605888	36616847	TBCB
ENSG00000105255	2.57521082	2.144285137	protein_coding	19	4304591	4323843	FSD1
ENSG00000105258	7.639400234	8.071789292	protein_coding	19	36604612	36606206	POLR2I
ENSG00000105261	1.407729925	2.039052734	protein_coding	19	36602105	36604613	OVOL3
ENSG00000105270	4.883502971	5.137803333	protein_coding	19	36505563	36523775	CLIP3
ENSG00000105278	5.316509574	4.048652948	protein_coding	19	3804022	3869027	ZFR2
ENSG00000105281	11.95893816	11.96033084	protein_coding	19	47278140	47291851	SLC1A5
ENSG00000105287	9.068907253	8.423679567	protein_coding	19	47177574	47220384	PRKD2
ENSG00000105289	5.223818625	3.96493948	protein_coding	19	3708382	3750810	TJP3
ENSG00000105290	6.984620223	7.625976011	protein_coding	19	36359138	36370699	APLP1
ENSG00000105298	8.620709081	8.633659743	protein_coding	19	3610626	3626813	C19orf29
ENSG00000105321	7.876068031	7.351639476	protein_coding	19	47759731	47775208	CCDC9
ENSG00000105323	11.03683924	10.73040795	protein_coding	19	41768391	41813811	HNRNPUL1
ENSG00000105325	10.03155637	10.06189795	protein_coding	19	3506295	3536755	FZR1
ENSG00000105327	6.882196036	7.260948155	protein_coding	19	47724079	47736023	BBC3
ENSG00000105329	9.437283511	9.227924003	protein_coding	19	41836813	41859831	TGFB1
ENSG00000105339	6.62865285	4.94736961	protein_coding	8	142127377	142205907	DENND3
ENSG00000105341	7.454602977	7.546612122	protein_coding	19	41937224	42006550	ATP5SL
ENSG00000105355	11.11419304	10.57085065	protein_coding	19	4838346	4867780	PLIN3
ENSG00000105357	8.929116952	8.924251218	protein_coding	19	50706885	50813802	MYH14
ENSG00000105364	11.18479473	11.2674718	protein_coding	19	10362640	10370736	MRPL4
ENSG00000105369	1.799000381	0.950786998	protein_coding	19	42381190	42385439	CD79A
ENSG00000105370	0	0.390640832	protein_coding	19	51883164	51891210	LIM2
ENSG00000105371	2.57521082	1.925536307	protein_coding	19	10397650	10399198	ICAM4
ENSG00000105372	11.33799045	11.80762974	protein_coding	19	42363988	42375482	RPS19
ENSG00000105373	10.59231022	10.47764716	protein_coding	19	48248793	48260323	GLTSCR2
ENSG00000105374	0	0.390640832	protein_coding	19	51874874	51875960	NKG7
ENSG00000105376	7.434062125	6.3217081	protein_coding	19	10400655	10407453	ICAM5
ENSG00000105379	9.646664866	9.969605823	protein_coding	19	51848410	51869672	ETFB
ENSG00000105388	0.499066092	0	protein_coding	19	42212504	42234339	CEACAM5
ENSG00000105393	10.23721557	10.41626325	protein_coding	19	17378232	17390162	BABAM1
ENSG00000105397	11.4374429	11.56401869	protein_coding	19	10461209	10491352	TYK2
ENSG00000105401	12.48954875	12.65223146	protein_coding	19	10501809	10514271	CDC37
ENSG00000105402	9.409495425	9.982524041	protein_coding	19	47990891	48018497	NAPA
ENSG00000105404	8.243750574	8.737164829	protein_coding	19	42460838	42463528	RABAC1
ENSG00000105419	6.892270075	7.00911166	protein_coding	19	47906316	47922780	MEIS3
ENSG00000105426	12.11292561	11.41868818	protein_coding	19	5173977	5340814	PTPRS
ENSG00000105427	3.502389126	3.845183986	protein_coding	19	42891173	42894444	CNFN
ENSG00000105429	8.368064704	8.347216179	protein_coding	19	42829761	42882921	MEGF8
ENSG00000105438	10.81782106	10.67961414	protein_coding	19	48885827	48894810	KDELR1
ENSG00000105443	9.208486525	9.494294562	protein_coding	19	48972289	48985571	CYTH2
ENSG00000105447	9.281205548	9.290875744	protein_coding	19	48949030	48957164	GRWD1
ENSG00000105464	8.500733514	8.088368066	protein_coding	19	48898132	48948187	GRIN2D
ENSG00000105467	1.407729925	0	protein_coding	19	48867657	48879627	SYNGR4
ENSG00000105472	4.261916566	5.751299057	protein_coding	19	51226605	51228979	CLEC11A
ENSG00000105479	3.652182994	4.319727133	protein_coding	19	48799714	48825130	CCDC114
ENSG00000105483	7.67776266	8.232567113	protein_coding	19	48706403	48759203	CARD8
ENSG00000105486	9.202426553	9.242810972	protein_coding	19	48618703	48673601	LIG1
ENSG00000105497	6.283060568	6.651226688	protein_coding	19	52074551	52092991	ZNF175
ENSG00000105499	6.616550626	7.563312496	protein_coding	19	48551100	48614109	PLA2G4C
ENSG00000105501	0.499066092	0.697730409	protein_coding	19	52114781	52150151	SIGLEC5
ENSG00000105509	0.869158192	1.802319292	protein_coding	19	52216365	52227237	HAS1
ENSG00000105514	10.47026584	9.132029383	protein_coding	19	11432723	11450344	RAB3D
ENSG00000105516	4.842525946	4.604259349	protein_coding	19	49133820	49140639	DBP
ENSG00000105518	10.11618743	10.51798133	protein_coding	19	11453452	11456981	TMEM205
ENSG00000105519	8.53328596	6.387491945	protein_coding	19	5911386	5916219	CAPS
ENSG00000105520	10.60767691	10.84922862	protein_coding	19	11466062	11476370	

ENSG00000105523	1.960915222	0.697730409	protein_coding	19	49103857	49116694	FAM83E
ENSG00000105538	3.871730003	4.448594745	protein_coding	19	49223843	49243970	RASIP1
ENSG00000105549	0.499066092	1.353254395	protein_coding	19	361750	376013	THEG
ENSG00000105550	2.10647801	1.353254395	protein_coding	19	49259344	49261580	FGF21
ENSG00000105552	9.126582713	9.245770021	protein_coding	19	49298322	49314320	BCAT2
ENSG00000105556	9.090943324	9.029304007	protein_coding	19	305575	344791	MIER2
ENSG00000105559	7.511262389	7.100213554	protein_coding	19	49340355	49371884	PLEKHA4
ENSG00000105568	12.54607477	12.53753354	protein_coding	19	52693274	52730687	PPP2R1A
ENSG00000105576	11.81229071	11.96370446	protein_coding	19	12810008	12834810	TNPO2
ENSG00000105583	9.834054211	10.39176091	protein_coding	19	12778881	12780465	WDR83OS
ENSG00000105605	0	0.697730409	protein_coding	19	54412589	54447195	CACNG7
ENSG00000105607	9.506139483	9.925997554	protein_coding	19	13001974	13010783	GCDH
ENSG00000105609	0.499066092	2.242360793	protein_coding	19	54754263	54761167	LILRB5
ENSG00000105610	2.238690726	3.64455972	protein_coding	19	12995236	12998017	KLF1
ENSG00000105612	10.28004571	10.38774804	protein_coding	19	12986023	12992335	DNASE2
ENSG00000105613	7.420203928	6.299096252	protein_coding	19	12949259	12985765	MAST1
ENSG00000105617	6.931877181	6.862599506	protein_coding	19	54658899	54663620	LENG1
ENSG00000105618	10.00742874	10.054323	protein_coding	19	54618837	54635140	PRPF31
ENSG00000105619	7.242608348	7.596144237	protein_coding	19	54610320	54619055	TFPT
ENSG00000105639	7.206730419	6.343971006	protein_coding	19	17935589	17958880	JAK3
ENSG00000105640	10.01263302	10.31000998	protein_coding	19	17970730	17974962	RPL18A
ENSG00000105641	4.292665995	3.133298822	protein_coding	19	17982782	18005982	SLC5A5
ENSG00000105642	3.077135474	2.501982735	protein_coding	19	18062111	18110893	KCNN1
ENSG00000105643	8.286389971	8.804937616	protein_coding	19	18111944	18124903	ARRDC2
ENSG00000105647	11.0234384	10.86807433	protein_coding	19	18263928	18281343	PIK3R2
ENSG00000105649	3.603959378	3.081239798	protein_coding	19	18307594	18314839	RAB3A
ENSG00000105650	2.847891871	3.453019579	protein_coding	19	18318771	18359010	PDE4C
ENSG00000105655	10.4375331	10.06021806	protein_coding	19	18545198	18549111	ISYNA1
ENSG00000105656	8.580777545	8.900977169	protein_coding	19	18553475	18632937	ELL
ENSG00000105662	8.724437638	8.754928172	protein_coding	19	18794425	18893142	CRTC1
ENSG00000105663	9.875269153	9.570619293	protein_coding	19	36208921	36229779	
ENSG00000105664	0.499066092	0	protein_coding	19	18893583	18902123	COMP
ENSG00000105668	1.616589159	2.721932731	protein_coding	19	36157715	36169367	UPK1A
ENSG00000105669	11.15451056	11.54657683	protein_coding	19	19010323	19030199	COPE
ENSG00000105671	10.12475765	10.46246481	protein_coding	19	19030484	19039442	DDX49
ENSG00000105672	2.671945279	2.579085888	protein_coding	19	36132647	36135773	ETV2
ENSG00000105675	0.499066092	0	protein_coding	19	36041096	36054560	ATP4A
ENSG00000105676	10.12849117	10.13513829	protein_coding	19	19144384	19170563	ARMC6
ENSG00000105677	9.364975797	9.635231414	protein_coding	19	36036532	36038428	TMEM147
ENSG00000105679	4.437288965	5.004391715	protein_coding	19	36024314	36036221	GAPDHS
ENSG00000105694	0.499066092	0.950786998	protein_coding	19	2538108	2538786	
ENSG00000105695	0	0.697730409	protein_coding	19	35783028	35804707	MAG
ENSG00000105696	2.847891871	2.652276565	protein_coding	19	18723682	18731841	TMEM59L
ENSG00000105698	9.13084342	9.388489822	protein_coding	19	35759896	35770718	USF2
ENSG00000105699	9.863155878	8.935290787	protein_coding	19	35739559	35758865	LSR
ENSG00000105700	10.38097398	10.58610742	protein_coding	19	18668572	18680197	KXD1
ENSG00000105701	11.89022848	11.88499174	protein_coding	19	18642568	18654383	FKBP8
ENSG00000105705	8.577659618	8.89250574	protein_coding	19	19387323	19431318	SUGP1
ENSG00000105707	2.671945279	3.813624741	protein_coding	19	35531410	35557475	HPN
ENSG00000105708	7.304268146	7.240325909	protein_coding	19	19821282	19843921	ZNF14
ENSG00000105711	6.171673665	6.582669759	protein_coding	19	35521592	35531352	SCN1B
ENSG00000105717	6.777386522	6.554308016	protein_coding	19	19672522	19729725	PBX4
ENSG00000105722	8.380642463	8.036341107	protein_coding	19	42751717	42759309	ERF
ENSG00000105723	8.626755466	8.461080043	protein_coding	19	42734338	42746777	GSK3A
ENSG00000105726	10.86909148	11.2104894	protein_coding	19	19756007	19774502	ATP13A1
ENSG00000105732	6.979904289	7.167295693	protein_coding	19	42572864	42585717	ZNF574
ENSG00000105737	2.847891871	2.579085888	protein_coding	19	42502477	42569957	GRIK5
ENSG00000105738	9.9657009	9.548022979	protein_coding	19	38397861	38699012	SIPA1L3
ENSG00000105750	7.434062125	7.784953917	protein_coding	19	21106059	21133503	ZNF85
ENSG00000105755	7.234712227	6.646756201	protein_coding	19	44010872	44031396	ETHE1
ENSG00000105767	5.512107582	5.871116342	protein_coding	19	44126522	44143991	CADM4
ENSG00000105771	8.197783135	8.276635048	protein_coding	19	44235301	44259142	SMG9
ENSG00000105778	9.675388335	9.031020259	protein_coding	7	32535038	33078516	AVL9

ENSG00000105784	1.16343121	0.390640832	protein_coding	7	87256864	87461611	RUNDC3B
ENSG00000105792	5.054730169	4.250702764	protein_coding	7	89874488	89940377	C7orf63
ENSG00000105793	7.484876093	7.418726742	protein_coding	7	89964537	90020769	GTPBP10
ENSG00000105808	4.734682531	4.448594745	protein_coding	7	102220093	102283316	RASA4
ENSG00000105810	10.38499274	10.27205358	protein_coding	7	92234235	92465908	CDK6
ENSG00000105819	9.551342933	9.514064455	protein_coding	7	102937869	102969958	PMPCB
ENSG00000105821	9.111570361	8.983063058	protein_coding	7	102952921	102985320	DNAJC2
ENSG00000105825	7.819120959	8.814934048	protein_coding	7	93514709	93520303	TFPI2
ENSG00000105829	7.883662252	8.034631177	protein_coding	7	93592074	93633694	BET1
ENSG00000105835	12.01144078	12.05541466	protein_coding	7	105888731	105926772	NAMPT
ENSG00000105849	8.908170811	8.674859633	protein_coding	7	19735085	19748710	TWISTNB
ENSG00000105851	0	0.390640832	protein_coding	7	106505723	106547590	PIK3CG
ENSG00000105852	0.869158192	0.390640832	protein_coding	7	94989256	95025680	PON3
ENSG00000105854	8.894453552	8.93712253	protein_coding	7	95034175	95064510	PON2
ENSG00000105855	3.448790144	3.081239798	protein_coding	7	20370325	20450419	ITGB8
ENSG00000105856	8.299696963	8.681431211	protein_coding	7	106809406	106842974	HBP1
ENSG00000105865	7.071412799	6.480864016	protein_coding	7	107203929	107218906	DUS4L
ENSG00000105866	7.246540262	7.002128877	protein_coding	7	21467652	21554440	SP4
ENSG00000105875	6.872051157	6.465715653	protein_coding	7	134868590	134896316	WDR91
ENSG00000105877	8.036821332	8.401295742	protein_coding	7	21582833	21941457	DNAH11
ENSG00000105879	8.574534937	8.046558348	protein_coding	7	107384142	107401142	CBLL1
ENSG00000105887	10.24853313	9.564114571	protein_coding	7	135611509	135662101	MTPN
ENSG00000105889	3.077135474	3.133298822	protein_coding	7	22459063	22672544	STEAP1B
ENSG00000105894	2.57521082	2.652276565	protein_coding	7	136912088	137028611	PTN
ENSG00000105926	8.793254009	8.486319932	protein_coding	7	24612887	24729159	MPP6
ENSG00000105928	5.613019223	5.466810074	protein_coding	7	24737972	24809244	DFNA5
ENSG00000105929	0.499066092	1.16600992	protein_coding	7	138391040	138484305	ATP6V0A4
ENSG00000105939	10.7796631	9.881029944	protein_coding	7	138728266	138794465	ZC3HAV1
ENSG00000105948	7.759833327	7.487839978	protein_coding	7	138818490	138876732	TTC26
ENSG00000105953	11.29639666	10.4179039	protein_coding	7	44646171	44748665	OGDH
ENSG00000105963	7.813830732	6.787424764	protein_coding	7	937540	995043	ADAP1
ENSG00000105968	10.67268056	10.6147154	protein_coding	7	44866390	44887682	H2AFV
ENSG00000105971	10.30004761	8.823871996	protein_coding	7	115927434	116148595	CAV2
ENSG00000105974	12.39573703	11.60521124	protein_coding	7	116164839	116201233	CAV1
ENSG00000105976	11.16207636	10.81227999	protein_coding	7	116312248	116438440	MET
ENSG00000105982	5.107569429	5.236305879	protein_coding	7	156432975	156469824	RNF32
ENSG00000105983	9.4441477	9.072443042	protein_coding	7	156473571	156685924	LMBR1
ENSG00000105988	0.869158192	0.950786998	pseudogene	10	93976089	93976550	NHP2P1
ENSG00000105991	1.407729925	0.697730409	protein_coding	7	27132612	27135615	HOXA1
ENSG00000105993	10.51251323	10.07151953	protein_coding	7	157128075	157210133	DNAJB6
ENSG00000105996	1.16343121	0.697730409	protein_coding	7	27139721	27142430	HOXA2
ENSG00000105997	3.004694206	2.420525079	protein_coding	7	27145816	27192200	HOXA3
ENSG00000106003	7.988335976	7.18590274	protein_coding	7	2552163	2568811	LFNG
ENSG00000106004	3.502389126	2.912743273	protein_coding	7	27180671	27183287	HOXA5
ENSG00000106006	1.407729925	2.039052734	protein_coding	7	27185015	27192217	HOXA6
ENSG00000106009	10.1086465	9.632407821	protein_coding	7	2577511	2595361	BRAT1
ENSG00000106012	9.189208632	8.605140645	protein_coding	7	2598632	2654368	IQCE
ENSG00000106018	0.869158192	0.390640832	protein_coding	7	158820866	158937649	VIPR2
ENSG00000106025	5.718698586	6.403479675	protein_coding	7	120427376	120498456	TSPAN12
ENSG00000106028	9.95852752	9.987393954	protein_coding	7	141438121	141487722	SSBP1
ENSG00000106031	2.928422289	0.950786998	protein_coding	7	27233122	27239725	HOXA13
ENSG00000106034	6.290697143	4.250702764	protein_coding	7	120628731	120937498	C7orf58
ENSG00000106038	1.799000381	1.353254395	protein_coding	7	27282164	27287047	EVX1
ENSG00000106049	10.32582812	10.02103068	protein_coding	7	27565061	27702614	HIBADH
ENSG00000106052	10.63680476	10.62355806	protein_coding	7	27778950	27880938	TAX1BP1
ENSG00000106069	2.35979773	1.518964905	protein_coding	7	29186185	29553944	CHN2
ENSG00000106070	10.50760669	10.14309534	protein_coding	7	50657760	50861159	GRB10
ENSG00000106077	7.319279953	6.440109466	protein_coding	7	73150424	73153197	ABHD11
ENSG00000106078	7.190493718	5.112089758	protein_coding	7	51083909	51384515	COBL
ENSG00000106080	9.224523099	8.735060572	protein_coding	7	30050203	30066300	FKBP14
ENSG00000106086	7.254372086	7.237355663	protein_coding	7	30067020	30170096	PLEKHA8
ENSG00000106089	7.819120959	7.307006333	protein_coding	7	73113535	73134017	STX1A
ENSG00000106100	7.837485466	7.337839373	protein_coding	7	30464143	30518400	NOD1

ENSG00000106105	12.28073386	12.14054918	protein_coding	7	30634297	30673649	GARS
ENSG00000106113	2.762599152	2.912743273	protein_coding	7	30692200	30739745	CRHR2
ENSG00000106123	3.274897671	0.390640832	protein_coding	7	142552792	142568847	EPHB6
ENSG00000106125	3.911897206	4.406904905	protein_coding	7	30811033	30932002	FAM188B
ENSG00000106133	2.35979773	1.925536307	pseudogene	7	72418832	72425329	NSUN5P2
ENSG00000106144	9.581229429	8.762179391	protein_coding	7	142985308	143004789	CASP2
ENSG00000106153	11.08895774	11.47099045	protein_coding	7	56169262	56174269	CHCHD2
ENSG00000106178	1.616589159	0	protein_coding	7	75440983	75452674	CCL24
ENSG00000106211	12.30650561	12.14590491	protein_coding	7	75931861	75933612	HSPB1
ENSG00000106236	0.869158192	0	protein_coding	7	98246609	98259180	NPTX2
ENSG00000106244	10.71834247	10.27169071	protein_coding	7	98989671	99006452	PDAP1
ENSG00000106245	8.605481314	8.463623996	protein_coding	7	99006264	99017239	BUD31
ENSG00000106246	6.25990527	5.893930351	protein_coding	7	99014362	99063820	PTCD1
ENSG00000106258	1.799000381	1.518964905	protein_coding	7	99245817	99277621	CYP3A5
ENSG00000106261	9.49130376	9.189251987	protein_coding	7	99613204	99639312	ZKSCAN1
ENSG00000106263	12.84884518	12.40789592	protein_coding	7	2393721	2420380	EIF3B
ENSG00000106266	9.843820138	9.576507334	protein_coding	7	2291405	2393953	SNX8
ENSG00000106268	8.324088959	7.985911283	protein_coding	7	2281857	2290781	NUDT1
ENSG00000106278	0	0.390640832	protein_coding	7	121513143	121702090	PTPRZ1
ENSG00000106290	9.258020218	8.639296529	protein_coding	7	99704693	99717464	TAF6
ENSG00000106299	9.453532949	9.015499988	protein_coding	7	123321989	123389121	WASL
ENSG00000106302	0.869158192	0.390640832	protein_coding	7	123469037	123517532	HYAL4
ENSG00000106305	8.741255403	8.792849743	protein_coding	7	6048876	6063465	AIMP2
ENSG00000106327	7.507990374	7.216390925	protein_coding	7	100218039	100240402	TFR2
ENSG00000106328	0.869158192	0	protein_coding	7	127231463	127242198	FSCN3
ENSG00000106330	7.420203928	7.057067116	protein_coding	7	100209725	100213007	MOSPD3
ENSG00000106333	5.301468762	4.29708397	protein_coding	7	100199800	100205798	PCOLCE
ENSG00000106336	2.928422289	2.652276565	protein_coding	7	100181605	100198740	FBXO24
ENSG00000106344	9.395398058	9.253876247	protein_coding	7	127950437	127983962	RBM28
ENSG00000106346	8.60394966	8.112884393	protein_coding	7	6144515	6201195	USP42
ENSG00000106348	10.83228291	10.10734462	protein_coding	7	128032331	128050306	IMPDH1
ENSG00000106351	8.730065371	7.623703032	protein_coding	7	100136834	100165842	AGFG2
ENSG00000106355	8.387780826	8.664945797	protein_coding	7	32524951	32534895	LSM5
ENSG00000106366	12.57880104	12.38131702	protein_coding	7	100770370	100782547	SERPINE1
ENSG00000106367	9.819610198	9.535382018	protein_coding	7	100797678	100804877	AP1S1
ENSG00000106384	0.499066092	0.390640832	protein_coding	7	100838288	100844302	MOGAT3
ENSG00000106392	9.235445742	9.264127823	protein_coding	7	7196565	7288251	C1GALT1
ENSG00000106397	11.04080703	11.14364759	protein_coding	7	100849258	100861701	PLOD3
ENSG00000106399	8.109865782	8.25035614	protein_coding	7	7676149	7758238	RPA3
ENSG00000106400	9.336667406	9.231660172	protein_coding	7	100860949	100867471	ZNHIT1
ENSG00000106404	7.367016815	7.612284109	protein_coding	7	100875373	100882101	CLDN15
ENSG00000106415	6.931877181	6.398170093	protein_coding	7	8008425	8133902	GLCCI1
ENSG00000106436	1.16343121	0.697730409	protein_coding	7	101256605	101272576	MYL10
ENSG00000106443	9.047656547	8.906597279	protein_coding	7	11013499	11209250	PHF14
ENSG00000106459	7.73205733	7.711939664	protein_coding	7	129251555	129396922	NRF1
ENSG00000106460	8.781106853	9.089014348	protein_coding	7	12250867	12276886	TMEM106B
ENSG00000106462	9.049908262	9.424844729	protein_coding	7	148504475	148581413	EZH2
ENSG00000106477	7.997697782	7.795089291	protein_coding	7	130036375	130082274	CEP41
ENSG00000106479	8.013936171	7.933654619	protein_coding	7	149535456	149564568	ZNF862
ENSG00000106483	0.869158192	0.697730409	protein_coding	7	37945543	38065297	SFRP4
ENSG00000106484	6.220467583	3.845183986	protein_coding	7	130126046	130146133	MEST
ENSG00000106524	7.140657543	6.766973175	protein_coding	7	16639401	16685442	ANKMY2
ENSG00000106526	5.458875168	5.317302248	protein_coding	7	149941005	150020814	ACTR3C
ENSG00000106537	8.890689734	6.919299326	protein_coding	7	16793160	16824161	TSPAN13
ENSG00000106538	5.512107582	3.87606762	protein_coding	7	150035408	150038763	RARRES2
ENSG00000106541	3.603959378	0	protein_coding	7	16831435	16873057	AGR2
ENSG00000106546	11.21971615	11.17134077	protein_coding	7	17338246	17385776	AHR
ENSG00000106554	9.185117095	9.208338404	protein_coding	7	132469629	132766848	CHCHD3
ENSG00000106560	4.962122459	5.283144778	protein_coding	7	150382785	150390729	GIMAP2
ENSG00000106565	0	0.697730409	protein_coding	7	150488373	150498450	TMEM176B
ENSG00000106571	8.593182305	8.540340653	protein_coding	7	42000548	42277469	GLI3
ENSG00000106588	6.343050432	6.720932357	protein_coding	7	42956460	42971822	PSMA2
ENSG00000106591	8.732871027	9.269953325	protein_coding	7	42971799	42988557	MRPL32

ENSG00000106603	9.099664364	8.995422414	protein_coding	7	43648083	43769316	C7orf44
ENSG00000106605	8.831713317	8.874456566	protein_coding	7	43798279	43846939	BLVRA
ENSG00000106608	10.34841449	10.09303901	protein_coding	7	43915493	43966010	URGCP
ENSG00000106609	10.94455111	10.23083319	protein_coding	7	66386212	66423538	C7orf42
ENSG00000106610	6.804311337	6.628734426	protein_coding	7	66767608	66786513	STAG3L4
ENSG00000106615	9.59671396	9.45966896	protein_coding	7	151163098	151217206	RHEB
ENSG00000106617	8.382430368	8.034631177	protein_coding	7	151253210	151574210	PRKAG2
ENSG00000106624	3.554067925	3.232099092	protein_coding	7	44143960	44154161	AEBP1
ENSG00000106628	11.47479401	11.16744486	protein_coding	7	44154286	44163957	POLD2
ENSG00000106631	0.499066092	1.802319292	protein_coding	7	44178463	44180931	MYL7
ENSG00000106633	5.649112675	5.200150955	protein_coding	7	44183872	44237769	GCK
ENSG00000106635	8.915598341	8.624594823	protein_coding	7	72950686	72972332	BCL7B
ENSG00000106636	11.07769075	10.58844036	protein_coding	7	44240567	44253893	YKT6
ENSG00000106638	9.747220804	9.591123153	protein_coding	7	72983990	72993121	TBL2
ENSG00000106665	9.358631958	8.35545848	protein_coding	7	73703803	73820273	CLIP2
ENSG00000106682	12.47254858	11.97076348	protein_coding	7	73588575	73611431	EIF4H
ENSG00000106683	10.5575205	9.737577069	protein_coding	7	73497263	73536855	LIMK1
ENSG00000106686	1.616589159	1.16600992	protein_coding	9	4553386	4666674	C9orf68
ENSG00000106689	4.923348194	3.845183986	protein_coding	9	126763949	126795580	LHX2
ENSG00000106692	8.833021386	8.729786462	protein_coding	9	108320411	108403399	FKTN
ENSG00000106701	6.658471572	7.005624493	protein_coding	9	108210077	108314714	FSD1L
ENSG00000106714	4.292665995	3.133298822	protein_coding	9	39072764	39288456	CNTNAP3
ENSG00000106723	10.28244148	10.10937675	protein_coding	9	91003334	91093609	SPIN1
ENSG00000106733	7.356139979	7.551403417	protein_coding	9	77675489	77703133	C9orf95
ENSG00000106771	10.10648469	9.823270084	protein_coding	9	111777417	111882225	C9orf5
ENSG00000106772	3.911897206	2.420525079	protein_coding	9	79226292	79521003	PRUNE2
ENSG00000106780	7.242608348	6.582669759	protein_coding	9	123363091	123476748	MEGF9
ENSG00000106785	10.59578202	10.05263426	protein_coding	9	100831557	100881494	TRIM14
ENSG00000106789	8.447071828	6.690849666	protein_coding	9	100883257	100954922	CORO2A
ENSG00000106799	10.24509804	10.44325939	protein_coding	9	101866320	101916474	TGFBR1
ENSG00000106803	9.651121255	10.00190583	protein_coding	9	101984346	101992897	SEC61B
ENSG00000106804	5.785082014	5.691786012	protein_coding	9	123714616	123837452	C5
ENSG00000106809	1.960915222	2.039052734	protein_coding	9	95146249	95166978	OGN
ENSG00000106819	0.869158192	1.667587519	protein_coding	9	95218487	95244788	ASPN
ENSG00000106823	5.360713447	5.516660875	protein_coding	9	95256365	95298937	ECM2
ENSG00000106829	7.430610036	8.635917101	protein_coding	9	82186688	82341658	TLE4
ENSG00000106852	5.375152132	3.781359661	protein_coding	9	124964856	124991905	LHX6
ENSG00000106853	9.72977077	9.025865365	protein_coding	9	114312002	114362135	PTGR1
ENSG00000106868	7.517784241	7.405556541	protein_coding	9	114803065	114937688	SUSD1
ENSG00000106927	0.499066092	0.697730409	protein_coding	9	116822407	116840752	AMBP
ENSG00000106948	8.544507981	7.926295939	protein_coding	9	117096436	117156685	AKNA
ENSG00000106952	0.869158192	0.697730409	protein_coding	9	117656003	117692697	TNFSF8
ENSG00000106976	8.329659793	5.09905904	protein_coding	9	130965658	131017527	DNM1
ENSG00000106991	10.79383898	10.43615284	protein_coding	9	130577291	130617035	ENG
ENSG00000106992	6.005279909	5.03207928	protein_coding	9	130628759	130640022	AK1
ENSG00000106993	5.60078458	6.1803943	protein_coding	9	4679559	4708398	CDC37L1
ENSG00000107014	0.869158192	0.950786998	protein_coding	9	5299868	5304969	RLN2
ENSG00000107020	5.938793061	6.252780742	protein_coding	9	5357973	5437878	C9orf46
ENSG00000107021	9.371291863	9.181546152	protein_coding	9	131549483	131572711	TBC1D13
ENSG00000107036	7.621344476	7.807158493	protein_coding	9	5629025	5776557	KIAA1432
ENSG00000107077	6.721980839	6.934050347	protein_coding	9	6720863	7175648	KDM4C
ENSG00000107104	1.960915222	0.390640832	protein_coding	9	470291	746105	KANK1
ENSG00000107130	10.75157835	10.32302723	protein_coding	9	132934857	132999583	NCS1
ENSG00000107140	7.440941622	7.507697912	protein_coding	9	35605281	35610038	TESK1
ENSG00000107147	4.842525946	4.961837817	protein_coding	9	138594031	138684992	KCNT1
ENSG00000107159	7.858191159	8.537928553	protein_coding	9	35673853	35681156	CA9
ENSG00000107164	10.20676225	9.929682347	protein_coding	9	133454352	133513739	FUBP3
ENSG00000107165	0	0.697730409	protein_coding	9	12685439	12710274	TYRP1
ENSG00000107175	8.382430368	8.948064416	protein_coding	9	35732332	35737001	CREB3
ENSG00000107185	8.742648075	8.457255683	protein_coding	9	35749203	35758572	RGP1
ENSG00000107186	0	0.950786998	protein_coding	9	13105703	13279589	MPDZ
ENSG00000107187	2.10647801	2.652276565	protein_coding	9	139088096	139096955	LHX3
ENSG00000107201	9.06334524	9.637486317	protein_coding	9	32455300	32526322	DDX58

ENSG00000107223	10.5575205	11.1885511	protein_coding	9	139756571	139760738	EDF1
ENSG00000107249	6.84118134	7.096940025	protein_coding	9	3824127	4348392	GLIS3
ENSG00000107262	7.663128351	8.405271149	protein_coding	9	33247818	33264761	BAG1
ENSG00000107263	9.863155878	9.681313358	protein_coding	9	134452157	134615461	RAPGEF1
ENSG00000107281	8.284478912	8.945336714	protein_coding	9	139933922	139940655	NPDC1
ENSG00000107282	3.335220907	1.802319292	protein_coding	9	72045204	72287222	APBA1
ENSG00000107290	9.528918244	9.420922967	protein_coding	9	135136743	135230372	SETX
ENSG00000107295	0.499066092	0.390640832	protein_coding	9	17578953	17797127	SH3GL2
ENSG00000107317	2.57521082	2.501982735	protein_coding	9	139871956	139879887	PTGDS
ENSG00000107331	9.697100607	9.022418507	protein_coding	9	139901686	139923374	ABCA2
ENSG00000107338	7.824391858	7.720473624	protein_coding	9	37919131	38069210	SHB
ENSG00000107341	9.704980607	9.232406246	protein_coding	9	33817565	33920402	UBE2R2
ENSG00000107362	7.575190993	7.724721747	protein_coding	9	74477368	74525847	FAM108B1
ENSG00000107371	7.250461488	7.575124073	protein_coding	9	37766975	37801434	EXOSC3
ENSG00000107372	10.88305084	10.34802633	protein_coding	9	74966341	74980163	ZFAND5
ENSG00000107404	10.53599578	10.13513829	protein_coding	1	1270656	1284760	DVL1
ENSG00000107438	11.23488705	11.91782908	protein_coding	10	96997329	97050781	PDLIM1
ENSG00000107443	7.277615649	7.985911283	protein_coding	10	97803151	97820627	CCNJ
ENSG00000107485	0.499066092	0	protein_coding	10	8095567	8117161	GATA3
ENSG00000107518	0.869158192	2.242360793	protein_coding	10	116853124	117708503	ATRNL1
ENSG00000107521	8.904442656	9.280091692	protein_coding	10	100175955	100206709	HPS1
ENSG00000107537	7.666067113	7.832967122	protein_coding	10	13319796	13344412	PHYH
ENSG00000107551	7.140657543	7.598461017	protein_coding	10	45454855	45490172	RASSF4
ENSG00000107554	8.193715877	8.116121984	protein_coding	10	101635334	101769676	DNMBP
ENSG00000107560	7.572060955	7.752033772	protein_coding	10	119764427	119806114	RAB11FIP2
ENSG00000107562	0.499066092	0.950786998	protein_coding	10	44793038	44881941	CXCL12
ENSG00000107566	9.519199923	9.935650135	protein_coding	10	101909851	101948091	ERLIN1
ENSG00000107581	11.97798309	12.21358951	protein_coding	10	120794356	120840396	EIF3A
ENSG00000107593	0	0.390640832	protein_coding	10	102047903	102090243	PKD2L1
ENSG00000107611	3.652182994	2.912743273	protein_coding	10	16865963	17171830	CUBN
ENSG00000107614	6.059927234	4.872768657	protein_coding	10	17184253	17244053	TRDMT1
ENSG00000107618	0.499066092	0	protein_coding	10	48381487	48390991	RBP3
ENSG00000107625	9.147761519	8.972384393	protein_coding	10	70661034	70706603	DDX50
ENSG00000107643	8.171138658	8.058387721	protein_coding	10	49514698	49647403	MAPK8
ENSG00000107651	9.799008649	9.8994674	protein_coding	10	121652223	121702014	SEC23IP
ENSG00000107669	8.154494062	8.299598884	protein_coding	10	123499939	123688316	ATE1
ENSG00000107672	7.430610036	8.48506837	protein_coding	10	123716603	123734732	NSMCE4A
ENSG00000107679	8.150302734	8.875412173	protein_coding	10	124134173	124191867	PLEKHA1
ENSG00000107719	3.004694206	3.183544561	protein_coding	10	72238564	72328205	KIAA1274
ENSG00000107731	8.191677941	7.911465009	protein_coding	10	72972327	73062621	UNC5B
ENSG00000107736	3.448790144	3.96493948	protein_coding	10	73156691	73575702	CDH23
ENSG00000107738	7.615275323	7.106738413	protein_coding	10	73507316	73533337	C10orf54
ENSG00000107742	4.821592551	5.339632724	protein_coding	10	73818793	73848790	SPOCK2
ENSG00000107745	8.991362316	8.955313173	protein_coding	10	74127098	74385899	MICU1
ENSG00000107758	8.457286611	8.234057939	protein_coding	10	75196186	75255782	PPP3CB
ENSG00000107771	9.024943975	9.247985331	protein_coding	10	86088342	86278273	FAM190B
ENSG00000107779	7.751556534	8.199373043	protein_coding	10	88516396	88684945	BMPR1A
ENSG00000107789	7.68357507	8.599368544	protein_coding	10	89264632	89313217	MINPP1
ENSG00000107796	4.962122459	5.393992	protein_coding	10	90694831	90751147	ACTA2
ENSG00000107798	10.37335233	10.58025851	protein_coding	10	90973326	91174382	LIPA
ENSG00000107807	0.499066092	0.697730409	protein_coding	10	102889257	102897545	TLX1
ENSG00000107815	9.261910407	9.381107599	protein_coding	10	102747124	102754158	C10orf2
ENSG00000107816	9.487155506	10.05009744	protein_coding	10	102756375	102767593	LZTS2
ENSG00000107819	10.09726068	10.16005582	protein_coding	10	102790991	102800998	SFXN3
ENSG00000107821	7.8607586	8.445721399	protein_coding	10	102820999	102827888	KAZALD1
ENSG00000107829	8.05038018	8.851330023	protein_coding	10	103370423	103455110	FBXW4
ENSG00000107831	0.499066092	1.518964905	protein_coding	10	103530081	103535827	FGF8
ENSG00000107833	9.21351713	9.512836742	protein_coding	10	103541082	103543170	NPM3
ENSG00000107854	9.273518431	9.343619007	protein_coding	10	93558069	93625033	TNKS2
ENSG00000107859	2.35979773	3.232099092	protein_coding	10	103989943	104001231	PITX3
ENSG00000107862	10.78406577	10.77102634	protein_coding	10	104005289	104142656	GBF1
ENSG00000107863	10.24509804	9.70408282	protein_coding	10	24872538	25012597	ARHGAP21
ENSG00000107864	5.431503808	4.94736961	protein_coding	10	93808399	94050844	CPEB3

ENSG00000107872	6.721980839	7.507697912	protein_coding	10	104178946	104182893	FBXL15
ENSG00000107874	8.746818039	9.326251289	protein_coding	10	104183002	104192418	CUEDC2
ENSG00000107882	8.371669553	8.360927302	protein_coding	10	104263744	104393292	SUFU
ENSG00000107890	7.511262389	8.790825214	protein_coding	10	27280843	27389421	ANKRD26
ENSG00000107897	8.016241092	9.418302523	protein_coding	10	27484144	27531068	ACBD5
ENSG00000107902	8.394884042	8.83570377	protein_coding	10	126150403	126306457	LHPP
ENSG00000107929	9.991116523	9.282254961	protein_coding	10	855484	977564	LARP4B
ENSG00000107937	10.14332937	9.83559787	protein_coding	10	1034338	1065876	GTPBP4
ENSG00000107938	7.454602977	7.953699568	protein_coding	10	127408084	127452712	C10orf137
ENSG00000107949	9.621905585	10.05094354	protein_coding	10	127512115	127542264	BCCIP
ENSG00000107951	8.693086466	9.314320673	protein_coding	10	30598730	30663377	MTPAP
ENSG00000107954	3.448790144	3.87606762	protein_coding	10	105253736	105352309	NEURL
ENSG00000107957	7.392081667	6.408769788	protein_coding	10	105348285	105615301	SH3PXD2A
ENSG00000107959	10.65688683	10.56612361	protein_coding	10	3179920	3215003	PITRM1
ENSG00000107960	7.250461488	6.89311646	protein_coding	10	105642300	105677963	OBFC1
ENSG00000107968	8.219950371	10.13991779	protein_coding	10	30722866	30750762	MAP3K8
ENSG00000107984	8.417730894	7.287036562	protein_coding	10	54074056	54077802	DKK1
ENSG00000108001	0.499066092	0	protein_coding	10	131633547	131762538	EBF3
ENSG00000108010	9.904803739	10.07276981	protein_coding	10	131934663	131982785	GLRX3
ENSG00000108021	10.71905012	10.39942117	protein_coding	10	5726801	5805703	FAM208B
ENSG00000108039	8.778393539	9.420922967	protein_coding	10	111624524	111683311	XPNPEP1
ENSG00000108055	10.02984629	10.37665477	protein_coding	10	112327449	112364394	SMC3
ENSG00000108061	8.555643385	8.955313173	protein_coding	10	112679301	112773425	SHOC2
ENSG00000108064	9.555310919	9.239845842	protein_coding	10	60144782	60158981	TFAM
ENSG00000108091	10.12902374	9.724908876	protein_coding	10	61548521	61666414	CCDC6
ENSG00000108094	9.30119149	10.26258899	protein_coding	10	35297479	35379570	CUL2
ENSG00000108100	9.662202373	10.33835587	protein_coding	10	35535953	35860852	CCNY
ENSG00000108106	10.20069501	10.05643114	protein_coding	19	55912652	55919325	UBE2S
ENSG00000108107	13.11592303	13.43228164	protein_coding	19	55896713	55914612	RPL28
ENSG00000108175	10.74360174	10.71046517	protein_coding	10	80828792	81076285	ZMIZ1
ENSG00000108176	1.616589159	1.667587519	protein_coding	10	69556427	69597924	DNAJC12
ENSG00000108179	10.91520975	10.68997129	protein_coding	10	81107225	81115093	PPIF
ENSG00000108187	4.352265886	4.547834947	protein_coding	10	70042417	70092684	PBLD
ENSG00000108219	9.761717462	9.794762494	protein_coding	10	82213922	82282387	TSPAN14
ENSG00000108239	7.030950614	7.758263791	protein_coding	10	96162261	96295687	TBC1D12
ENSG00000108242	0.869158192	1.16600992	protein_coding	10	96442710	96495947	CYP2C18
ENSG00000108256	10.69659067	10.15887898	protein_coding	17	27582855	27621166	NUFIP2
ENSG00000108262	10.18848346	9.651500164	protein_coding	17	27900487	27916645	GIT1
ENSG00000108264	7.161632221	6.95589788	protein_coding	17	35766965	35838372	TADA2A
ENSG00000108270	10.16166506	9.969158297	protein_coding	17	35306175	35414170	AATF
ENSG00000108272	6.567100227	6.281901708	protein_coding	17	34948329	34957235	DHRS11
ENSG00000108278	7.413224588	7.747865429	protein_coding	17	34842479	34851667	ZNHIT3
ENSG00000108292	10.74046841	10.44261478	protein_coding	17	36861795	36886056	MLLT6
ENSG00000108294	9.76309053	9.955215698	protein_coding	17	36909003	36920483	PSMB3
ENSG00000108296	7.795160661	7.789016615	protein_coding	17	36957447	36981589	CWC25
ENSG00000108298	12.44019009	12.83249443	protein_coding	17	37356536	37360980	RPL19
ENSG00000108306	7.911170638	7.705505903	protein_coding	17	37408897	37557898	FBXL20
ENSG00000108309	1.799000381	2.144285137	protein_coding	17	42385943	42396039	RUNDC3A
ENSG00000108312	10.3787365	10.39642851	protein_coding	17	42282401	42298994	UBTF
ENSG00000108342	7.488200893	6.009862691	protein_coding	17	38171614	38174066	CSF3
ENSG00000108344	11.23958002	10.83628127	protein_coding	17	38137050	38154213	PSMD3
ENSG00000108349	9.795658092	9.666483771	protein_coding	17	38296507	38328431	CASC3
ENSG00000108352	7.46813618	6.866449614	protein_coding	17	38333263	38351906	RAPGEFL1
ENSG00000108370	3.448790144	4.226940739	protein_coding	17	63133456	63223819	RGS9
ENSG00000108375	7.262161623	7.167295693	protein_coding	17	56429861	56494480	RNF43
ENSG00000108379	4.517578978	4.744751758	protein_coding	17	44841694	44896082	WNT3
ENSG00000108381	0.499066092	0	protein_coding	17	3377404	3406713	ASPA
ENSG00000108384	7.593829938	8.166959047	protein_coding	17	56769934	56811703	RAD51C
ENSG00000108387	3.077135474	2.912743273	protein_coding	17	56597616	56618062	4-sep
ENSG00000108389	9.599791022	9.638612448	protein_coding	17	56566893	56595251	MTMR4
ENSG00000108395	9.544971367	9.768254884	protein_coding	17	57060002	57184266	TRIM37
ENSG00000108405	0.499066092	0.390640832	protein_coding	17	3799891	3819960	P2RX1
ENSG00000108406	9.524067267	9.793751926	protein_coding	17	57642886	57685712	DHX40

ENSG00000108423	7.471499737	7.549009758	protein_coding	17	57936851	57970296	TUBD1
ENSG00000108424	12.24690755	11.97967783	protein_coding	17	45726842	45760998	KPNB1
ENSG00000108433	7.962273293	8.060069742	protein_coding	17	45000486	45105003	GOSR2
ENSG00000108439	8.801295662	8.576046394	protein_coding	17	46018889	46026673	PNPO
ENSG00000108442	0	0.390640832	pseudogene	2	74855455	74856069	FAM18B4P
ENSG00000108443	8.787867896	8.803934152	protein_coding	17	57970447	58027925	RPS6KB1
ENSG00000108448	8.337054268	7.960920138	protein_coding	17	18601311	18639432	TRIM16L
ENSG00000108452	0	0.950786998	pseudogene	17	15578790	15578957	ZNF29P
ENSG00000108465	9.28407769	9.57591961	protein_coding	17	46047900	46059147	CDK5RAP3
ENSG00000108468	9.765147683	9.843432726	protein_coding	17	46147414	46178883	CBX1
ENSG00000108469	8.525216449	8.652735634	protein_coding	17	73622925	73663269	RECQL5
ENSG00000108474	7.153278907	7.971683681	protein_coding	17	16120524	16229573	PIGL
ENSG00000108479	8.101206377	8.371803145	protein_coding	17	73754018	73761307	GALK1
ENSG00000108506	8.068262669	8.202422444	protein_coding	17	59942728	60005377	INTS2
ENSG00000108509	9.87399885	10.354209	protein_coding	17	4871287	4890960	CAMTA2
ENSG00000108510	9.828818559	9.902283133	protein_coding	17	60019967	60142643	MED13
ENSG00000108511	0.869158192	0.697730409	protein_coding	17	46671639	46682354	HOXB6
ENSG00000108515	6.129712275	7.444711851	protein_coding	17	4851387	4860426	ENO3
ENSG00000108518	13.00880848	13.78229465	protein_coding	17	4848947	4852309	PFN1
ENSG00000108523	10.33877788	10.93330827	protein_coding	17	4843423	4848518	RNF167
ENSG00000108528	9.47296155	9.933357752	protein_coding	17	4840425	4843463	SLC25A11
ENSG00000108551	6.96088487	5.32851069	protein_coding	17	17397751	17399707	RASD1
ENSG00000108556	3.211941663	3.813624741	protein_coding	17	4801069	4806369	CHRNE
ENSG00000108557	10.42890818	10.70321774	protein_coding	17	17584787	17714767	RAI1
ENSG00000108559	10.1653044	10.4939164	protein_coding	17	5289346	5323000	NUP88
ENSG00000108561	10.94757346	11.63019492	protein_coding	17	5336099	5342471	C1QBP
ENSG00000108576	1.16343121	0.950786998	protein_coding	17	28521337	28562954	SLC6A4
ENSG00000108578	9.308732943	9.028445114	protein_coding	17	28575223	28619074	BLMH
ENSG00000108582	11.03513541	10.84703819	protein_coding	17	28705942	28796730	CPD
ENSG00000108587	8.472474444	8.286726732	protein_coding	17	28804426	28853837	GOSR1
ENSG00000108588	10.39963312	10.66725214	protein_coding	17	61822611	61851088	CCDC47
ENSG00000108590	5.239686603	6.27612435	protein_coding	17	6546635	6554954	MED31
ENSG00000108591	9.608219258	10.02361908	protein_coding	17	17991239	18011285	DRG2
ENSG00000108592	10.15069164	10.21544416	protein_coding	17	61896795	61905031	FTSJ3
ENSG00000108599	8.528449672	9.002437654	protein_coding	17	19807788	19881150	AKAP10
ENSG00000108602	0	0.390640832	protein_coding	17	19641297	19651746	ALDH3A1
ENSG00000108604	10.33647394	10.28505673	protein_coding	17	61909441	61920351	SMARCD2
ENSG00000108622	5.660945913	3.570966319	protein_coding	17	62079956	62097994	ICAM2
ENSG00000108639	9.450127191	9.453283127	protein_coding	17	76164671	76169009	SYNGR2
ENSG00000108641	7.549958459	8.387964709	protein_coding	17	19244550	19281469	B9D1
ENSG00000108651	9.371291863	9.317839931	protein_coding	17	30187923	30228731	UTP6
ENSG00000108654	12.34070362	12.44054631	protein_coding	17	62494374	62502484	DDX5
ENSG00000108666	7.093410923	6.97385478	protein_coding	17	30658392	30669189	C17orf75
ENSG00000108669	8.189637121	8.171634445	protein_coding	17	76670130	76778379	CYTH1
ENSG00000108671	11.09032576	10.56789807	protein_coding	17	30771502	30809398	PSMD11
ENSG00000108679	12.5445785	12.94924282	protein_coding	17	76967338	76976061	LGALS3BP
ENSG00000108688	0	0.390640832	protein_coding	17	32597240	32599261	CCL7
ENSG00000108691	10.47320504	11.60434694	protein_coding	17	32582313	32584222	CCL2
ENSG00000108700	0	0.390640832	protein_coding	17	32646055	32648421	CCL8
ENSG00000108733	5.752272083	5.953060722	protein_coding	17	33897849	33905656	PEX12
ENSG00000108753	0	0.390640832	protein_coding	17	36046434	36105237	HNF1B
ENSG00000108759	1.407729925	0.697730409	protein_coding	17	39616063	39623681	KRT32
ENSG00000108771	7.210761201	7.996490656	protein_coding	17	40253422	40264751	DHX58
ENSG00000108773	9.886651823	10.33071191	protein_coding	17	40265126	40273376	KAT2A
ENSG00000108774	10.52995925	10.5557289	protein_coding	17	40276994	40307035	RAB5C
ENSG00000108784	8.448779323	8.771449152	protein_coding	17	40687951	40696464	NAGLU
ENSG00000108785	2.471521042	1.518964905	protein_coding	17	40698771	40699464	HSD17B1P1
ENSG00000108786	7.04456449	6.568558581	protein_coding	17	40703984	40707857	HSD17B1
ENSG00000108788	8.422952257	8.117738058	protein_coding	17	40719078	40725220	MLX
ENSG00000108797	9.290757201	9.80181672	protein_coding	17	40834632	40852010	CNTNAP1
ENSG00000108799	8.150302734	8.678149164	protein_coding	17	40852294	40897071	EZH1
ENSG00000108813	5.141750194	4.676215056	protein_coding	17	48046334	48052321	DLX4
ENSG00000108819	10.12154978	9.554003775	processed_transc	17	48211104	48227991	PPP1R9B

ENSG00000108821	12.2631316	10.99242021	protein_coding	17	48260650	48278993	COL1A1
ENSG00000108823	1.407729925	0.390640832	protein_coding	17	48241575	48253292	SGCA
ENSG00000108825	8.362640485	8.634788863	protein_coding	17	41102543	41132545	AARSD1
ENSG00000108826	7.96466218	8.291030223	protein_coding	17	48445218	48450575	MRPL27
ENSG00000108828	11.61521956	11.29647904	protein_coding	17	41166622	41174459	VAT1
ENSG00000108829	11.82894007	11.32071108	protein_coding	17	48458599	48474914	LRRC59
ENSG00000108830	3.274897671	2.721932731	protein_coding	17	41177258	41184057	RND2
ENSG00000108839	5.637181576	6.866449614	protein_coding	17	6899384	6914055	ALOX12
ENSG00000108840	8.53006357	7.834933409	protein_coding	17	42154121	42201014	HDAC5
ENSG00000108846	9.876538338	9.400488931	protein_coding	17	48712138	48769063	ABCC3
ENSG00000108848	9.4441477	9.657623394	protein_coding	17	48796905	48834076	LUC7L3
ENSG00000108852	6.820228284	6.398170093	protein_coding	17	41952725	41987068	MPP2
ENSG00000108854	9.009986712	9.304420832	protein_coding	17	62540735	62658386	SMURF2
ENSG00000108861	9.761717462	9.906496457	protein_coding	17	41843490	41856368	DUSP3
ENSG00000108878	0	0.390640832	protein_coding	17	65040706	65052913	CACNG1
ENSG00000108883	11.21244741	11.08310221	protein_coding	17	42927655	42976993	EFTUD2
ENSG00000108924	1.799000381	1.16600992	protein_coding	17	53342321	53402426	HLF
ENSG00000108932	5.018397273	5.593003837	protein_coding	17	66263167	66287405	SLC16A6
ENSG00000108946	10.54320643	10.64669035	protein_coding	17	66508110	66528908	PRKAR1A
ENSG00000108947	5.107569429	6.117167886	protein_coding	17	7608520	7614696	EFNB3
ENSG00000108950	2.57521082	3.493416095	protein_coding	17	66531258	66597095	FAM20A
ENSG00000108953	13.07836245	13.28314478	protein_coding	17	1247566	1303505	YWHAE
ENSG00000108958	1.16343121	1.353254395	pseudogene	17	1761333	1761740	
ENSG00000108960	8.403714162	7.718344862	protein_coding	17	53469974	53499341	MMD
ENSG00000108961	5.107569429	5.808454063	protein_coding	17	8191969	8193409	RANGRF
ENSG00000108963	9.034071956	9.591123153	protein_coding	17	1933431	1946724	DPH1
ENSG00000109016	7.757079669	8.714915547	protein_coding	17	21030258	21094836	DHRS7B
ENSG00000109046	9.283120944	9.120790981	protein_coding	17	25621106	25640657	WSB1
ENSG00000109061	0	0.390640832	protein_coding	17	10395624	10421860	MYH1
ENSG00000109062	10.07642654	9.674741242	protein_coding	17	72744763	72765498	SLC9A3R1
ENSG00000109063	5.827699195	6.850987146	protein_coding	17	10531844	10559465	MYH3
ENSG00000109065	8.324088959	8.770422115	protein_coding	17	72766688	72772470	NAT9
ENSG00000109066	8.391336806	8.570156468	protein_coding	17	72772622	72835918	TMEM104
ENSG00000109079	10.04403543	9.800308001	protein_coding	17	26662730	26674035	TNFAIP1
ENSG00000109083	6.922076877	7.234379288	protein_coding	17	26655353	26662495	IFT20
ENSG00000109084	9.633963159	8.875412173	protein_coding	17	26646161	26655711	TMEM97
ENSG00000109089	9.493786999	9.50483104	protein_coding	17	72983727	73001895	CDR2L
ENSG00000109099	8.641761486	9.722786654	protein_coding	17	15133095	15168643	PMP22
ENSG00000109103	8.116326397	8.398639372	protein_coding	17	26873725	26879659	UNC119
ENSG00000109107	9.556895063	8.814934048	protein_coding	17	26900133	26903952	ALDOC
ENSG00000109111	10.98541646	10.65283399	protein_coding	17	26989302	27029248	SUPT6H
ENSG00000109113	10.53479049	10.50291056	protein_coding	17	27041299	27045447	RAB34
ENSG00000109118	9.43383913	8.76733661	protein_coding	17	27232268	27278789	PHF12
ENSG00000109133	9.540174158	9.267043514	protein_coding	4	41937137	41962589	TMEM33
ENSG00000109163	1.16343121	1.518964905	protein_coding	4	68605046	68620078	GNRHR
ENSG00000109171	9.263851576	9.169909673	protein_coding	4	48343339	48428076	SLAIN2
ENSG00000109180	9.252165193	9.480543884	protein_coding	4	48807229	48863834	OCIAD1
ENSG00000109184	8.458982065	8.724493	protein_coding	4	52709166	52783003	DCUN1D4
ENSG00000109189	6.989320791	7.340609974	protein_coding	4	53460340	53525502	USP46
ENSG00000109193	0	0.390640832	protein_coding	4	70676498	70725870	SULT1E1
ENSG00000109205	0	0.950786998	protein_coding	4	71062213	71070293	ODAM
ENSG00000109220	6.541724534	6.866449614	protein_coding	4	54875956	54930857	CHIC2
ENSG00000109255	3.211941663	1.16600992	protein_coding	4	56461396	56502865	NMU
ENSG00000109265	4.261916566	6.002883551	protein_coding	4	57036361	57194791	KIAA1211
ENSG00000109270	7.413224588	7.881337775	protein_coding	4	100799493	100815647	LAMTOR3
ENSG00000109272	0.499066092	1.16600992	protein_coding	4	74718906	74719872	PF4V1
ENSG00000109320	10.20221422	10.70885777	protein_coding	4	103422486	103538459	NFKB1
ENSG00000109321	3.554067925	0	protein_coding	4	75310851	75320726	AREG
ENSG00000109323	7.546773135	8.385283657	protein_coding	4	103552660	103682151	MANBA
ENSG00000109332	10.63605558	10.88478007	protein_coding	4	103715540	103790053	UBE2D3
ENSG00000109339	2.10647801	2.039052734	protein_coding	4	86936276	87515284	MAPK10
ENSG00000109381	7.883662252	7.953699568	protein_coding	4	139949266	140098372	ELF2
ENSG00000109390	7.537174746	7.971683681	protein_coding	4	140188034	140223705	NDUFC1

ENSG00000109436	9.150911694	9.212125573	protein_coding	4	141541919	141677274	TBC1D9
ENSG00000109445	8.16075831	8.405271149	protein_coding	4	142142041	142155851	ZNF330
ENSG00000109452	7.734859118	7.340609974	protein_coding	4	142944313	143768585	INPP4B
ENSG00000109458	6.290697143	6.980975423	protein_coding	4	144257915	144395721	GAB1
ENSG00000109466	7.066972635	6.82352253	protein_coding	4	166128770	166244308	KLHL2
ENSG00000109472	7.273767634	9.042126408	protein_coding	4	166282346	166419472	CPE
ENSG00000109475	10.04403543	10.96933381	protein_coding	4	109541722	109551568	RPL34
ENSG00000109501	9.58976648	9.843920995	protein_coding	4	6271576	6304992	WFS1
ENSG00000109511	0.869158192	1.353254395	protein_coding	4	169013666	169108893	ANXA10
ENSG00000109519	9.379372099	9.403141901	protein_coding	4	7060633	7069924	GRPEL1
ENSG00000109534	7.981274537	8.088368066	protein_coding	4	110736666	110745893	GAR1
ENSG00000109536	7.277615649	8.112884393	protein_coding	4	190861943	190884359	FRG1
ENSG00000109572	9.789607412	10.35523688	protein_coding	4	170533784	170644824	CLCN3
ENSG00000109576	6.050961887	6.815578497	protein_coding	4	170981373	171012850	AADAT
ENSG00000109586	9.124447632	9.432656416	protein_coding	4	174089904	174245118	GALNT7
ENSG00000109606	10.70661584	10.64697018	protein_coding	4	24519064	24586173	DHX15
ENSG00000109610	3.448790144	2.971122874	protein_coding	4	24797085	24802464	SOD3
ENSG00000109618	6.573375019	6.926693689	protein_coding	4	25121627	25162204	SEPSECS
ENSG00000109625	3.077135474	1.925536307	protein_coding	4	8594387	8621488	CPZ
ENSG00000109654	7.488200893	7.996490656	protein_coding	4	154073494	154260472	TRIM2
ENSG00000109667	2.847891871	1.16600992	protein_coding	4	9772777	10056560	SLC2A9
ENSG00000109670	7.698004462	7.907733332	protein_coding	4	153242410	153456172	FBXW7
ENSG00000109674	7.017207048	7.4317778	protein_coding	4	178230990	178284097	NEIL3
ENSG00000109680	4.923348194	5.339632724	protein_coding	4	26578059	26756973	TBC1D19
ENSG00000109684	0	0.390640832	protein_coding	4	10488019	10686489	CLNK
ENSG00000109685	11.20842134	10.91715822	protein_coding	4	1873151	1983934	WHSC1
ENSG00000109686	9.816968406	10.29044031	protein_coding	4	152041431	152149182	SH3D19
ENSG00000109689	7.581430769	7.832967122	protein_coding	4	26859300	27027003	STIM2
ENSG00000109705	2.238690726	1.802319292	protein_coding	4	13542454	13546674	NKX3-2
ENSG00000109736	9.365879787	9.376390077	protein_coding	4	2932288	2936586	MFSD10
ENSG00000109738	1.407729925	5.759604001	protein_coding	4	157997209	158093242	GLRB
ENSG00000109743	2.671945279	5.57429348	protein_coding	4	15704573	15739936	BST1
ENSG00000109756	7.765324926	8.211532164	protein_coding	4	160025330	160281321	RAPGEF2
ENSG00000109758	2.35979773	3.027231696	protein_coding	4	3443614	3451211	HGFAC
ENSG00000109762	6.922076877	7.560938532	protein_coding	4	186125391	186291339	SNX25
ENSG00000109771	4.381167248	5.306006045	protein_coding	4	186285032	186317053	LRP2BP
ENSG00000109775	7.556308086	7.85445027	protein_coding	4	186320694	186347139	UFSP2
ENSG00000109787	9.029515185	8.159132889	protein_coding	4	38665817	38702663	KLF3
ENSG00000109790	9.887911038	10.1101888	protein_coding	4	39046451	39128477	KLHL5
ENSG00000109794	1.407729925	1.518964905	protein_coding	4	187025573	187093821	FAM149A
ENSG00000109805	9.102921211	9.568257332	protein_coding	4	17812525	17846485	NCAPG
ENSG00000109814	9.604394344	9.103767504	protein_coding	4	39500375	39529931	UGDH
ENSG00000109819	0.869158192	0.697730409	protein_coding	4	23756664	23905712	PPARGC1A
ENSG00000109832	0.499066092	0.390640832	protein_coding	11	125773271	125793158	DDX25
ENSG00000109846	1.407729925	2.242360793	protein_coding	11	111779289	111794446	CRYAB
ENSG00000109854	9.671006391	9.275755399	protein_coding	11	20385231	20405329	HTATIP2
ENSG00000109861	10.96706591	10.9490526	protein_coding	11	88026773	88070955	CTSC
ENSG00000109881	7.374222747	7.714077892	protein_coding	11	27352374	27385415	CCDC34
ENSG00000109911	7.292905885	7.326703453	protein_coding	11	31531297	31805546	ELP4
ENSG00000109917	9.388297379	9.483680566	protein_coding	11	116648436	116658766	ZNF259
ENSG00000109919	10.27668493	10.73883367	protein_coding	11	47638867	47664175	MTCH2
ENSG00000109920	9.692063559	10.13234293	protein_coding	11	47738072	47788995	FNBP4
ENSG00000109927	3.448790144	3.532712221	protein_coding	11	120971882	121062202	TECTA
ENSG00000109929	8.528449672	8.439273392	protein_coding	11	121163162	121179403	SC5DL
ENSG00000109944	5.124761037	4.903073497	protein_coding	11	122753391	122830506	C11orf63
ENSG00000109956	1.16343121	0.950786998	protein_coding	11	134248398	134281812	B3GAT1
ENSG00000109971	13.63994066	13.70259499	protein_coding	11	122928197	122933938	HSPA8
ENSG00000109991	1.799000381	1.518964905	protein_coding	11	57105848	57138073	P2RX3
ENSG00000110002	5.795854912	7.148445524	protein_coding	11	123986069	124018428	VWA5A
ENSG00000110011	7.356139979	7.402908012	protein_coding	11	63997750	64001758	DNAJC4
ENSG00000110013	7.921045056	8.439273392	protein_coding	11	124503009	124565603	SIAE
ENSG00000110025	8.039090007	8.312357201	protein_coding	11	64781654	64808044	SNX15
ENSG00000110031	5.458875168	5.776071827	protein_coding	11	58294344	58345693	LPXN

ENSG00000110042	8.143992822	5.878761167	protein_coding	11	58938903	58976060	DTX4
ENSG00000110046	10.06311141	10.27748581	protein_coding	11	64662007	64684722	ATG2A
ENSG00000110047	10.63155234	10.19874442	protein_coding	11	64619114	64655768	EHD1
ENSG00000110048	10.38543857	10.30114757	protein_coding	11	59341871	59383617	OSBP
ENSG00000110057	9.27062517	8.804937616	protein_coding	11	67758575	67772452	UNC93B1
ENSG00000110060	7.132181536	7.173524745	protein_coding	11	125763381	125773116	PUS3
ENSG00000110063	8.357195795	8.440565301	protein_coding	11	126173647	126215644	DCPS
ENSG00000110066	8.996041004	9.049765501	protein_coding	11	67922330	67981295	SUV420H1
ENSG00000110074	9.304967144	9.616492755	protein_coding	11	126138950	126148026	FOXRED1
ENSG00000110075	10.89562502	10.70885777	protein_coding	11	68228186	68382802	PPP6R3
ENSG00000110076	0.869158192	1.802319292	protein_coding	11	64373646	64490660	NRXN2
ENSG00000110080	8.088119026	8.093304774	protein_coding	11	126225535	126310239	ST3GAL4
ENSG00000110090	9.794315688	8.166959047	protein_coding	11	68522088	68611878	CPT1A
ENSG00000110092	11.93355396	11.1042471	protein_coding	11	69455855	69469242	CCND1
ENSG00000110104	9.544172939	9.378413761	protein_coding	11	60609544	60618554	CCDC86
ENSG00000110107	11.31219382	11.33119682	protein_coding	11	60658202	60674060	PRPF19
ENSG00000110108	11.0257282	11.40353163	protein_coding	11	60681346	60690915	TMEM109
ENSG00000110169	1.960915222	3.133298822	protein_coding	11	6452279	6463847	HPX
ENSG00000110171	7.106450572	7.541804862	protein_coding	11	6469843	6495689	TRIM3
ENSG00000110172	9.001868107	9.247985331	protein_coding	11	89934328	89956532	CHORDC1
ENSG00000110195	0.869158192	0.390640832	protein_coding	11	71900602	71907345	FOLR1
ENSG00000110200	6.469548347	6.628734426	protein_coding	11	71817424	71823826	C11orf51
ENSG00000110203	0	0.390640832	protein_coding	11	71825915	71850936	FOLR3
ENSG00000110218	9.095310434	9.261937181	protein_coding	11	93862094	93915138	PANX1
ENSG00000110237	9.335744919	9.511607983	protein_coding	11	73019334	73080136	ARHGEF17
ENSG00000110274	9.576551475	9.566483319	protein_coding	11	117185273	117283984	CEP164
ENSG00000110315	7.855619141	8.308116953	protein_coding	11	10533225	10562777	RNF141
ENSG00000110318	6.112579918	5.953060722	protein_coding	11	101785746	101871789	KIAA1377
ENSG00000110321	13.18778473	12.88088067	protein_coding	11	10818597	10830657	EIF4G2
ENSG00000110324	1.960915222	3.368666104	protein_coding	11	117857063	117872196	IL10RA
ENSG00000110328	8.946141254	8.857146507	protein_coding	11	11292423	11643552	GALNTL4
ENSG00000110330	9.62945337	10.37361447	protein_coding	11	102217942	102249401	BIRC2
ENSG00000110344	9.185117095	9.081580806	protein_coding	11	118230300	118269926	UBE4A
ENSG00000110347	0	0.390640832	processed_transc	11	102733467	102745764	MMP12
ENSG00000110367	9.92641136	9.703544845	protein_coding	11	118620034	118661858	DDX6
ENSG00000110375	3.004694206	3.027231696	protein_coding	11	118795873	118829269	UPK2
ENSG00000110395	9.574206797	9.215902826	protein_coding	11	119076752	119177651	CBL
ENSG00000110400	11.32967441	9.678031042	protein_coding	11	119494120	119599794	PVRL1
ENSG00000110422	8.953376332	8.784734524	protein_coding	11	33278218	33378569	HIPK3
ENSG00000110427	3.448790144	1.518964905	protein_coding	11	33563618	33695648	C11orf41
ENSG00000110429	8.154494062	8.247406493	protein_coding	11	33762485	33796089	FBXO3
ENSG00000110435	8.611591722	9.127223641	protein_coding	11	34937376	35042138	PDHX
ENSG00000110442	9.095310434	9.385809746	protein_coding	11	36295051	36310999	COMMD9
ENSG00000110446	5.286269488	5.09905904	protein_coding	11	60704556	60720002	SLC15A3
ENSG00000110448	2.762599152	2.652276565	protein_coding	11	60869867	60895324	CD5
ENSG00000110455	5.977159882	6.695185789	protein_coding	11	44087475	44105772	ACCS
ENSG00000110492	8.217949183	6.530239731	protein_coding	11	46402306	46405375	MDK
ENSG00000110497	9.144604451	9.009418947	protein_coding	11	46417964	46615675	AMBRA1
ENSG00000110514	9.424324461	9.387150406	protein_coding	11	47290712	47357945	MADD
ENSG00000110536	6.400645076	6.839280559	protein_coding	11	47586982	47595013	PTPMT1
ENSG00000110583	8.497437504	8.591248608	protein_coding	11	63706431	63724800	NAA40
ENSG00000110619	10.34932891	10.65728571	protein_coding	11	3022152	3078843	CARS
ENSG00000110628	6.414691513	6.136428444	protein_coding	11	2920951	2946476	SLC22A18
ENSG00000110651	11.88503548	12.37246026	protein_coding	11	2397407	2418627	CD81
ENSG00000110660	10.09126063	9.653172715	protein_coding	11	107661717	107799019	SLC35F2
ENSG00000110665	0.869158192	0.697730409	protein_coding	11	2316875	2324279	C11orf21
ENSG00000110675	0.869158192	0.950786998	protein_coding	11	107461817	107537505	ELMOD1
ENSG00000110693	0.869158192	1.518964905	protein_coding	11	15987995	16761138	SOX6
ENSG00000110696	10.07863383	10.51369144	protein_coding	11	16634679	16778649	C11orf58
ENSG00000110697	11.37213372	10.80602883	protein_coding	11	67259239	67273734	PITPNM1
ENSG00000110700	11.08923145	11.82418014	protein_coding	11	17095936	17099334	RPS13
ENSG00000110711	10.21028972	10.60264789	protein_coding	11	67250512	67258574	AIP
ENSG00000110713	11.06243696	11.01340773	protein_coding	11	3692313	3819022	NUP98

ENSG00000110717	10.70839874	11.18527956	protein_coding	11	67798084	67804111	NDUFS8
ENSG00000110719	10.22481234	10.49547112	protein_coding	11	67806483	67818362	TCIRG1
ENSG00000110721	9.30213633	8.891561392	protein_coding	11	67820326	67888736	CHKA
ENSG00000110723	7.190493718	6.3217081	protein_coding	11	108376158	108464465	EXPH5
ENSG00000110756	8.49908645	8.891561392	protein_coding	11	18300223	18343745	HPS5
ENSG00000110768	9.314363274	9.728615271	protein_coding	11	18343842	18388591	GTF2H1
ENSG00000110786	0	0.390640832	protein_coding	11	18749475	18814268	PTPN5
ENSG00000110799	1.407729925	0.390640832	protein_coding	12	6058040	6233936	VWF
ENSG00000110801	6.005279909	6.174194902	protein_coding	12	122326637	122356203	PSMD9
ENSG00000110811	9.200400892	9.343619007	protein_coding	12	6937204	6949018	LEPREL2
ENSG00000110841	9.587443199	9.218164439	protein_coding	12	27676364	27848497	PPFIBP1
ENSG00000110844	7.07583934	7.192052139	protein_coding	12	49962001	50038449	PRPF40B
ENSG00000110851	8.734271811	8.697730375	protein_coding	12	108126643	108155049	PRDM4
ENSG00000110852	1.616589159	1.925536307	protein_coding	12	10005583	10022735	CLEC2B
ENSG00000110871	7.933293789	8.001751392	protein_coding	12	120941077	120972237	COQ5
ENSG00000110876	5.141750194	5.792353798	protein_coding	12	109015686	109027735	SELPLG
ENSG00000110880	11.5189442	11.37137818	protein_coding	12	109038885	109125372	CORO1C
ENSG00000110881	5.795854912	5.317302248	protein_coding	12	50451331	50477394	ACCN2
ENSG00000110888	8.60394966	8.487570409	protein_coding	12	30862486	30907885	CAPRIN2
ENSG00000110900	2.10647801	2.788380093	protein_coding	12	31079362	31149534	TSPAN11
ENSG00000110906	9.342189974	8.852301067	protein_coding	12	109886461	109915349	KCTD10
ENSG00000110911	10.14332937	10.44904791	protein_coding	12	51373184	51422349	SLC11A2
ENSG00000110917	11.32549834	11.19870325	protein_coding	12	121124672	121139667	MLEC
ENSG00000110921	8.398422578	7.080460027	protein_coding	12	110011060	110035067	MVK
ENSG00000110925	7.976547636	8.184028599	protein_coding	12	51454990	51477447	CSRNP2
ENSG00000110931	9.627946969	9.369623887	protein_coding	12	121675497	121736111	CAMKK2
ENSG00000110944	2.847891871	3.183544561	protein_coding	12	56732663	56734193	IL23A
ENSG00000110955	13.33555496	13.36290097	protein_coding	12	57031959	57039798	ATP5B
ENSG00000110958	12.24199224	12.14253511	protein_coding	12	57057127	57082159	PTGES3
ENSG00000110987	7.751556534	7.743685008	protein_coding	12	122457328	122499948	BCL7A
ENSG00000111011	9.397167781	9.456479577	protein_coding	12	122989240	123011547	RSRC2
ENSG00000111012	5.575999561	4.794098771	protein_coding	12	58156117	58161034	CYP27B1
ENSG00000111057	11.92007705	11.71070402	protein_coding	12	53342655	53346685	KRT18
ENSG00000111077	8.414239457	8.993663261	protein_coding	12	53440753	53458156	TENC1
ENSG00000111087	4.381167248	3.570966319	protein_coding	12	57853918	57866045	GLI1
ENSG00000111110	6.283060568	7.690381265	protein_coding	12	63038367	63328817	PPM1H
ENSG00000111142	9.580450822	9.721192935	protein_coding	12	95867296	95909615	METAP2
ENSG00000111144	9.869543962	9.607318979	protein_coding	12	96394606	96437298	LTA4H
ENSG00000111145	9.482995289	8.934374042	protein_coding	12	96588160	96663613	ELK3
ENSG00000111181	4.999881834	3.32456471	protein_coding	12	299243	323736	SLC6A12
ENSG00000111186	0	0.697730409	protein_coding	12	1639057	1756409	WNT5B
ENSG00000111196	7.440941622	7.405556541	protein_coding	12	10758612	10766222	MAGOHB
ENSG00000111199	6.350376764	3.532712221	protein_coding	12	110220890	110271212	TRPV4
ENSG00000111203	6.793601635	7.210344614	protein_coding	12	2921788	2968957	ITFG2
ENSG00000111206	10.62778887	10.07360272	protein_coding	12	2966847	2986206	FOXM1
ENSG00000111215	4.543379137	4.547834947	protein_coding	12	10977559	11324212	PRR4
ENSG00000111218	0	0.697730409	protein_coding	12	3490515	3703139	PRMT8
ENSG00000111224	7.367016815	6.839280559	protein_coding	12	3900213	3982608	PARP11
ENSG00000111229	9.437283511	9.730728943	protein_coding	12	110872630	110888227	ARPC3
ENSG00000111231	7.945439403	8.162268448	protein_coding	12	110890289	110907019	GPN3
ENSG00000111237	8.817245731	8.992782879	protein_coding	12	110928902	110939922	VPS29
ENSG00000111245	1.407729925	0	protein_coding	12	111348638	111358526	MYL2
ENSG00000111247	7.639400234	8.210017866	protein_coding	12	4647950	4669214	RAD51AP1
ENSG00000111249	1.616589159	0.390640832	protein_coding	12	111471828	111788358	CUX2
ENSG00000111252	9.527303063	8.955313173	protein_coding	12	111843752	111889427	SH2B3
ENSG00000111254	3.004694206	3.232099092	protein_coding	12	4724677	4758213	AKAP3
ENSG00000111261	4.593635215	3.183544561	protein_coding	12	12482198	12503475	MANSC1
ENSG00000111266	8.377059993	8.251828705	protein_coding	12	12628829	12715317	DUSP16
ENSG00000111269	8.417730894	8.524589435	protein_coding	12	12764761	12798042	CREBL2
ENSG00000111271	7.903720218	7.694718794	protein_coding	12	112123857	112194903	ACAD10
ENSG00000111275	5.389447744	5.564846512	protein_coding	12	112204691	112247782	ALDH2
ENSG00000111276	8.774313977	8.638170933	protein_coding	12	12867992	12875305	CDKN1B
ENSG00000111291	4.19837882	3.64455972	protein_coding	12	13093709	13105081	GPRC5D

ENSG00000111300	9.143550557	8.528239625	protein_coding	12	112464500	112546826	NAA25
ENSG00000111305	0.869158192	0.390640832	protein_coding	12	13236494	13256619	GSG1
ENSG00000111319	8.472474444	3.453019579	protein_coding	12	6456009	6486896	SCNN1A
ENSG00000111321	10.52753755	9.87769557	protein_coding	12	6484211	6500733	LTBR
ENSG00000111325	3.698846687	3.714580548	protein_coding	12	123459127	123464590	OGFOD2
ENSG00000111328	8.041355121	7.764467022	protein_coding	12	123745528	123756881	CDK2AP1
ENSG00000111331	11.48043941	11.55793056	protein_coding	12	113376157	113411054	OAS3
ENSG00000111335	9.41124795	10.16005582	protein_coding	12	113416200	113449528	OAS2
ENSG00000111339	1.616589159	1.353254395	protein_coding	12	14978503	14996429	ART4
ENSG00000111341	7.250461488	7.799123587	protein_coding	12	15034115	15038860	MGP
ENSG00000111344	5.090170488	1.16600992	protein_coding	12	113536624	113574044	RASAL1
ENSG00000111348	6.171673665	4.990346085	protein_coding	12	15094951	15114662	ARHGDIB
ENSG00000111358	8.666918882	8.441856054	protein_coding	12	124118375	124146479	GTF2H3
ENSG00000111361	9.100750797	9.33252778	protein_coding	12	124104953	124118313	EIF2B1
ENSG00000111364	8.247679379	8.122575449	protein_coding	12	124086624	124105482	DDX55
ENSG00000111371	9.477150759	9.407772892	protein_coding	12	46581584	46663800	SLC38A1
ENSG00000111405	0.499066092	0	protein_coding	12	48103517	48119350	ENDOU
ENSG00000111412	7.514527001	7.394933125	protein_coding	12	117153593	117175875	C12orf49
ENSG00000111424	9.203438318	9.110276346	protein_coding	12	48235320	48336831	VDR
ENSG00000111445	8.502378699	8.671562585	protein_coding	12	118451393	118470935	RFC5
ENSG00000111450	8.307246212	8.377210474	protein_coding	12	131274145	131323811	STX2
ENSG00000111452	6.646617938	7.225413142	protein_coding	12	131438452	131626014	GPR133
ENSG00000111481	11.054609	10.84874214	protein_coding	12	54694986	54745633	COPZ1
ENSG00000111490	6.335686705	4.585695337	protein_coding	12	65174589	65274812	TBC1D30
ENSG00000111530	10.40887296	10.67797198	protein_coding	12	67663061	67713731	CAND1
ENSG00000111540	7.615275323	7.752033772	protein_coding	12	56367697	56388490	RAB5B
ENSG00000111554	5.785082014	6.568558581	protein_coding	12	68666223	68726161	MDM1
ENSG00000111581	9.211506993	9.316433258	protein_coding	12	69080514	69136785	NUP107
ENSG00000111596	9.229498191	9.209096633	protein_coding	12	70636774	70748773	CNOT2
ENSG00000111602	10.54000614	10.93697446	protein_coding	12	56810903	56843187	TIMELESS
ENSG00000111605	10.068119	10.06231762	protein_coding	12	69633317	69668138	CPSF6
ENSG00000111615	7.484876093	7.502758994	protein_coding	12	75890684	75905416	KRR1
ENSG00000111639	9.541775001	9.988277629	protein_coding	12	6601150	6603007	MRPL51
ENSG00000111640	15.33814457	15.0266238	protein_coding	12	6643093	6647537	GAPDH
ENSG00000111641	10.43839276	10.09427078	protein_coding	12	6666029	6677857	NOP2
ENSG00000111642	11.67205586	11.52633815	protein_coding	12	6679249	6716642	CHD4
ENSG00000111644	1.407729925	2.242360793	protein_coding	12	6747241	6756626	ACRBP
ENSG00000111647	8.074911866	8.036341107	protein_coding	12	100422233	100536626	UHRF1BP1L
ENSG00000111652	9.738871124	9.57120918	protein_coding	12	6832907	6841041	COPS7A
ENSG00000111653	6.693459083	7.077141316	protein_coding	12	6759446	6772314	ING4
ENSG00000111664	6.343050432	6.25865215	protein_coding	12	6949118	6956557	GNB3
ENSG00000111665	6.872051157	7.176629214	protein_coding	12	6957967	6961230	CDCA3
ENSG00000111666	8.096857104	8.044860491	protein_coding	12	102090725	102137918	CHPT1
ENSG00000111667	10.66938918	10.58435524	protein_coding	12	6961292	6975796	USP5
ENSG00000111669	12.53546772	12.62692064	protein_coding	12	6976283	6980112	TPI1
ENSG00000111670	8.617676359	9.137615924	protein_coding	12	102139275	102224716	GNPTAB
ENSG00000111671	6.212449006	6.246885341	protein_coding	12	6980099	6998522	SPSB2
ENSG00000111674	8.613115289	10.027493	protein_coding	12	7022909	7032861	ENO2
ENSG00000111676	11.76201644	11.23433547	protein_coding	12	7033626	7051484	ATN1
ENSG00000111678	7.33043718	8.511125832	protein_coding	12	7052141	7055166	C12orf57
ENSG00000111679	7.740446424	6.941369681	protein_coding	12	7055631	7070479	PTPN6
ENSG00000111684	9.772665583	8.635917101	protein_coding	12	7085348	7125814	LPCAT3
ENSG00000111696	8.229914881	7.737391576	protein_coding	12	104164231	104234975	NT5DC3
ENSG00000111704	0	0.950786998	protein_coding	12	7940390	7948655	NANOG
ENSG00000111707	8.93886971	8.387964709	protein_coding	12	118814185	118855840	SUDS3
ENSG00000111711	9.275444054	9.008548131	protein_coding	12	21654715	21671091	GOLT1B
ENSG00000111716	12.91086048	12.57492656	protein_coding	12	21788276	21910791	LDHB
ENSG00000111725	7.326727684	7.462626902	protein_coding	12	120105558	120119435	PRKAB1
ENSG00000111726	8.359012976	8.508664347	protein_coding	12	22199108	22218608	CMAS
ENSG00000111727	6.188122351	6.682138126	protein_coding	12	104458235	104498639	HCFC2
ENSG00000111728	3.211941663	4.761389014	protein_coding	12	22216707	22589975	ST8SIA1
ENSG00000111729	2.671945279	1.353254395	protein_coding	12	8276228	8291203	CLEC4A
ENSG00000111731	8.475827914	8.424985509	protein_coding	12	22601517	22697480	KIAA0528

ENSG00000111732	1.616589159	1.16600992	protein_coding	12	8754762	8765467	AICDA
ENSG00000111737	9.643686274	9.378413761	protein_coding	12	120532899	120555306	RAB35
ENSG00000111752	5.938793061	5.767861411	protein_coding	12	9066492	9094063	PHC1
ENSG00000111775	7.54038131	7.903991977	protein_coding	12	120875893	120878545	COX6A1
ENSG00000111785	6.554468172	7.135740508	protein_coding	12	107168373	107283090	RIC8B
ENSG00000111786	10.56619858	10.44840588	protein_coding	12	120899471	120907596	SRSF9
ENSG00000111788	5.827699195	6.281901708	pseudogene	12	9429831	9466684	
ENSG00000111790	7.959880444	7.962719647	protein_coding	12	27091316	27119583	FGFR1OP2
ENSG00000111796	0.499066092	1.518964905	protein_coding	12	9747147	9760482	KLRB1
ENSG00000111799	11.19525848	11.96336745	protein_coding	6	75794042	75915767	COL12A1
ENSG00000111801	8.043616684	8.534302826	protein_coding	6	26440700	26453643	BTN3A3
ENSG00000111802	8.320363082	8.417132063	protein_coding	6	24650205	24667261	TDP2
ENSG00000111816	4.942865586	3.027231696	protein_coding	6	116262693	116381921	FRK
ENSG00000111817	10.25684158	10.25819969	protein_coding	6	116575336	116759442	DSE
ENSG00000111832	7.995363019	8.519708104	protein_coding	6	116892530	116918838	RWDD1
ENSG00000111834	0.499066092	1.518964905	protein_coding	6	116937642	116954148	RSPH4A
ENSG00000111837	3.448790144	3.714580548	protein_coding	6	10762956	10838764	MAK
ENSG00000111843	9.432113851	10.29508988	protein_coding	6	10723148	10731362	TMEM14C
ENSG00000111845	8.49908645	8.930701228	protein_coding	6	10694928	10710015	PAK1IP1
ENSG00000111846	8.80263159	8.759076187	protein_coding	6	10492456	10629601	GCNT2
ENSG00000111850	4.689181911	4.990346085	protein_coding	6	88032306	88052043	C6orf162
ENSG00000111859	9.932525987	10.43485697	protein_coding	6	11183531	11382581	NEDD9
ENSG00000111860	5.774228067	6.037446454	protein_coding	6	118781935	119031238	CEP85L
ENSG00000111863	4.517578978	4.676215056	protein_coding	6	11712287	11807279	C6orf105
ENSG00000111875	7.488200893	7.600774083	protein_coding	6	119215384	119230332	ASF1A
ENSG00000111877	6.313367288	6.064512706	protein_coding	6	119134618	119256327	MCM9
ENSG00000111879	0.499066092	0.950786998	protein_coding	6	119280928	119470552	FAM184A
ENSG00000111880	7.778963161	7.766528853	protein_coding	6	89319985	89673348	RNGTT
ENSG00000111885	9.451831075	9.222677059	protein_coding	6	119498374	119670926	MAN1A1
ENSG00000111886	1.407729925	0.697730409	protein_coding	6	89967239	90025018	GABRR2
ENSG00000111897	9.450979385	9.503595441	protein_coding	6	122764493	122907269	SERINC1
ENSG00000111906	8.114176072	8.437980325	protein_coding	6	125596496	125623282	HDDC2
ENSG00000111907	7.319279953	5.938503778	protein_coding	6	125440195	125585553	TPD52L1
ENSG00000111911	7.44778847	7.335063441	protein_coding	6	126277927	126301387	HINT3
ENSG00000111912	10.37110298	9.638612448	protein_coding	6	126102307	126252266	NCOA7
ENSG00000111913	2.238690726	2.579085888	protein_coding	6	24797601	25042238	FAM65B
ENSG00000111961	4.903563137	1.353254395	protein_coding	6	148593440	148873186	SASH1
ENSG00000111962	7.19863491	7.201227369	protein_coding	6	149068464	149398126	UST
ENSG00000111981	6.616550626	6.926693689	protein_coding	6	150285143	150294844	ULBP1
ENSG00000112029	7.797842652	8.316585024	protein_coding	6	153291664	153304714	FBXO5
ENSG00000112031	7.2307479	7.249200135	protein_coding	6	153308497	153323820	MTRF1L
ENSG00000112033	9.212512411	9.349132808	protein_coding	6	35310335	35395968	PPARD
ENSG00000112038	0	0.390640832	protein_coding	6	154331631	154568001	OPRM1
ENSG00000112039	7.706593329	7.648510984	protein_coding	6	35420138	35434880	FANCE
ENSG00000112041	1.16343121	1.353254395	protein_coding	6	35465651	35480715	TULP1
ENSG00000112053	1.16343121	2.144285137	protein_coding	6	35911291	35992645	SLC26A8
ENSG00000112062	9.620391278	9.860907117	protein_coding	6	35995488	36079013	MAPK14
ENSG00000112078	9.95492741	9.96826283	protein_coding	6	36410544	36458920	KCTD20
ENSG00000112079	9.323698603	9.624472234	protein_coding	6	36461669	36515247	STK38
ENSG00000112081	11.4283848	11.62693594	protein_coding	6	36562145	36571209	SRSF3
ENSG00000112096	14.09432195	14.437099	protein_coding	6	160100096	160183561	SOD2
ENSG00000112110	8.708847212	8.954409067	protein_coding	6	160210844	160219468	MRPL18
ENSG00000112118	10.70661584	10.90714709	protein_coding	6	52128807	52149582	MCM3
ENSG00000112130	8.164919416	8.530667964	protein_coding	6	37321748	37362514	RNF8
ENSG00000112137	2.847891871	3.279072565	protein_coding	6	12717893	13288645	PHACTR1
ENSG00000112139	5.207774174	6.174194902	protein_coding	6	37598455	37667082	MDGA1
ENSG00000112144	8.048129203	7.977035488	protein_coding	6	52866077	52926600	ICK
ENSG00000112146	8.727254249	9.119984878	protein_coding	6	52916789	52965671	FBXO9
ENSG00000112149	7.686472517	8.073455769	protein_coding	6	14117872	14137149	CD83
ENSG00000112159	9.928248471	9.720129478	protein_coding	6	90352218	90529442	MDN1
ENSG00000112167	6.907250452	7.389591938	protein_coding	6	39071840	39082965	SAYSD1
ENSG00000112175	4.062237333	2.501982735	protein_coding	6	55618443	55740362	BMP5
ENSG00000112182	4.517578978	4.363974406	protein_coding	6	90636248	91006627	BACH2

ENSG00000112183	5.175139844	4.075511708	protein_coding	6	17281577	17294106	RBM24
ENSG00000112186	8.299696963	8.140175057	protein_coding	6	17393447	17558023	CAP2
ENSG00000112200	8.571403474	8.78167951	protein_coding	6	56951642	57035105	ZNF451
ENSG00000112208	8.150302734	8.377210474	protein_coding	6	57037124	57049735	BAG2
ENSG00000112210	7.030950614	7.348889996	protein_coding	6	57053581	57087078	RAB23
ENSG00000112212	1.616589159	1.667587519	protein_coding	6	41010293	41012076	TSPO2
ENSG00000112218	1.799000381	1.353254395	protein_coding	6	97245888	97285353	GPR63
ENSG00000112234	7.161632221	7.243290054	protein_coding	6	99321334	99395849	FBXL4
ENSG00000112237	9.191250057	9.028445114	protein_coding	6	99990256	100016849	CCNC
ENSG00000112242	8.978417134	9.138412238	protein_coding	6	20402137	20493945	E2F3
ENSG00000112245	10.4075566	9.744392843	protein_coding	6	64231666	64293492	PTP4A1
ENSG00000112249	9.755522437	9.569438796	protein_coding	6	100956070	101329248	ASCC3
ENSG00000112273	0	0.390640832	protein_coding	6	22569678	22571892	HDGFL1
ENSG00000112282	7.878603882	8.135396417	protein_coding	6	131895106	131949369	MED23
ENSG00000112290	8.269098624	8.576046394	protein_coding	6	110421022	110501207	WASF1
ENSG00000112293	1.16343121	2.144285137	protein_coding	6	24424793	24495433	GPLD1
ENSG00000112294	4.097495944	5.674321415	protein_coding	6	24495080	24537435	ALDH5A1
ENSG00000112297	10.22481234	9.026725794	protein_coding	6	106808784	107018335	AIM1
ENSG00000112299	7.928406768	9.110276346	protein_coding	6	133002729	133035188	VNN1
ENSG00000112303	3.274897671	2.912743273	protein_coding	6	133065009	133084598	VNN2
ENSG00000112304	7.759833327	7.996490656	protein_coding	6	24667263	24705293	ACOT13
ENSG00000112305	6.121171528	6.615068802	protein_coding	6	71377479	71571718	SMAP1
ENSG00000112306	11.42491909	12.11326566	protein_coding	6	133135580	133138703	RPS12
ENSG00000112308	11.02658594	11.21540346	protein_coding	6	24705089	24721064	C6orf62
ENSG00000112309	2.671945279	2.242360793	protein_coding	6	71566382	71666741	B3GAT2
ENSG00000112312	8.295907473	8.787783083	protein_coding	6	24775159	24786327	GMNN
ENSG00000112319	0.499066092	0	protein_coding	6	133561736	133853258	EYA4
ENSG00000112320	2.928422289	1.802319292	protein_coding	6	107811162	107981357	SOBP
ENSG00000112335	9.731875952	10.03691901	protein_coding	6	108532426	108582464	SNX3
ENSG00000112339	9.168633912	9.215148166	protein_coding	6	135281516	135424194	HBS1L
ENSG00000112343	7.596913151	7.786986696	protein_coding	6	25963030	25985348	TRIM38
ENSG00000112357	5.346128795	5.6022688	protein_coding	6	137143717	137235059	PEX7
ENSG00000112365	7.491518048	7.591499486	protein_coding	6	109783797	109804440	ZBTB24
ENSG00000112367	6.62865285	7.176629214	protein_coding	6	110012499	110146631	FIG4
ENSG00000112378	10.14332937	9.967814887	protein_coding	6	138411923	138428648	PERP
ENSG00000112379	1.616589159	4.202780776	protein_coding	6	138483058	138665800	KIAA1244
ENSG00000112406	7.440941622	7.582164879	protein_coding	6	139456249	139501939	HECA
ENSG00000112414	9.561637084	9.233897238	protein_coding	6	142622991	142767403	GPR126
ENSG00000112419	8.393111514	8.497535398	protein_coding	6	143857982	144152322	PHACTR2
ENSG00000112425	4.821592551	5.516660875	protein_coding	6	145822719	146057160	EPM2A
ENSG00000112473	11.21144195	11.37476113	protein_coding	6	33168222	33172216	SLC39A7
ENSG00000112486	0.499066092	0.390640832	protein_coding	6	167525295	167553184	CCR6
ENSG00000112494	1.407729925	0.697730409	protein_coding	6	167684657	167729507	UNC93A
ENSG00000112511	8.380642463	8.374509343	protein_coding	6	33378176	33384230	PHF1
ENSG00000112514	9.632461462	10.12191245	protein_coding	6	33384219	33386094	CUTA
ENSG00000112531	10.31930939	10.42998708	protein_coding	6	163835032	163999628	QKI
ENSG00000112541	2.671945279	1.667587519	protein_coding	6	165740776	166075588	PDE10A
ENSG00000112559	7.599989788	6.559073862	protein_coding	6	41604620	41621984	MDFI
ENSG00000112561	5.995967334	6.382123086	protein_coding	6	41651716	41703997	TFEB
ENSG00000112576	9.419108071	9.115138778	protein_coding	6	41902671	42018095	CCND3
ENSG00000112578	9.037480115	8.918699593	protein_coding	6	41888926	41900784	BYSL
ENSG00000112584	7.916116295	8.185570409	protein_coding	6	170599791	170716153	FAM120B
ENSG00000112592	7.457998197	7.591499486	protein_coding	6	170863390	170881957	TBP
ENSG00000112599	3.830412367	3.133298822	protein_coding	6	42152139	42162694	GUCA1B
ENSG00000112619	0.499066092	0	protein_coding	6	42664340	42690312	PRPH2
ENSG00000112624	7.76258174	7.819127564	protein_coding	6	42714696	42836296	KIAA0240
ENSG00000112640	9.813659349	9.754296791	protein_coding	6	42952237	42980080	PPP2R5D
ENSG00000112651	9.071126069	9.317839931	protein_coding	6	43021767	43027544	MRPL2
ENSG00000112655	9.718490953	9.678031042	protein_coding	6	43044006	43129457	PTK7
ENSG00000112658	10.02642002	9.801313989	protein_coding	6	43139037	43149243	SRF
ENSG00000112659	8.762005612	9.009418947	protein_coding	6	43149913	43192325	CUL9
ENSG00000112664	8.953376332	9.055679178	protein_coding	6	34255997	34360451	NUDT3
ENSG00000112667	8.947349623	9.53356709	protein_coding	6	43193367	43197222	C6orf108

ENSG00000112679	7.514527001	8.14493792	protein_coding	6	292097	351355	DUSP22
ENSG00000112685	9.085465781	8.859080135	protein_coding	6	485133	693117	EXOC2
ENSG00000112695	8.666918882	9.167571071	protein_coding	6	75947391	75960039	COX7A2
ENSG00000112697	10.45335453	10.60004881	protein_coding	6	75962640	75994684	TMEM30A
ENSG00000112699	8.470794781	8.151264044	protein_coding	6	1624041	2245926	GMDS
ENSG00000112701	9.022652927	9.232406246	protein_coding	6	76311225	76427997	SENP6
ENSG00000112706	0.869158192	0.390640832	protein_coding	6	76630832	76782395	IMPG1
ENSG00000112715	11.05544975	12.05108658	protein_coding	6	43737921	43754224	VEGFA
ENSG00000112739	9.886021803	10.29187254	protein_coding	6	4021501	4065217	PRPF4B
ENSG00000112742	8.665451116	8.791837833	protein_coding	6	80713604	80752244	TTK
ENSG00000112759	9.99696351	9.697613846	protein_coding	6	44187242	44201888	SLC29A1
ENSG00000112761	1.616589159	1.518964905	protein_coding	6	112375275	112392171	WISP3
ENSG00000112763	7.757079669	8.24888207	protein_coding	6	26458150	26476849	BTN2A1
ENSG00000112769	0.869158192	0	protein_coding	6	112429963	112576141	LAMA4
ENSG00000112773	7.461385447	7.701200733	protein_coding	6	82201156	82462491	FAM46A
ENSG00000112782	0	0.390640832	protein_coding	6	45848750	46048132	CLIC5
ENSG00000112787	10.19612778	9.641985576	protein_coding	12	133066137	133161774	FBRSL1
ENSG00000112796	0	0.390640832	protein_coding	6	46128152	46138708	ENPP5
ENSG00000112812	6.103936836	6.198834244	protein_coding	6	27215480	27224403	PRSS16
ENSG00000112851	10.08358792	9.870524428	protein_coding	5	65222303	65378377	ERBB2IP
ENSG00000112852	6.032862302	7.272601283	protein_coding	5	140474227	140476962	PCDHB2
ENSG00000112855	8.18144485	8.302443832	protein_coding	5	140071011	140078889	HARS2
ENSG00000112874	6.343050432	6.408769788	protein_coding	5	102884556	102898494	NUDT12
ENSG00000112877	8.039090007	7.598461017	protein_coding	5	612387	667283	CEP72
ENSG00000112893	9.437283511	9.518964886	protein_coding	5	109025067	109205326	MAN2A1
ENSG00000112902	5.331395195	5.248158938	protein_coding	5	9035138	9546187	SEMA5A
ENSG00000112936	0	0.697730409	protein_coding	5	40909354	40983041	C7
ENSG00000112941	9.740266097	9.554600493	protein_coding	5	6714718	6757161	PAPD7
ENSG00000112972	10.83522323	9.050611798	protein_coding	5	43289497	43313614	HMGCS1
ENSG00000112977	11.76681548	11.38419141	protein_coding	5	10679342	10761384	DAP
ENSG00000112981	2.10647801	0.390640832	protein_coding	5	137450866	137475132	NME5
ENSG00000112983	8.521975964	9.066597822	protein_coding	5	137475455	137514675	BRD8
ENSG00000112984	9.612034058	9.296594447	protein_coding	5	137514408	137523404	KIF20A
ENSG00000112992	7.609180529	7.519971735	protein_coding	5	43602791	43707507	NNT
ENSG00000112996	9.882867569	9.70837941	protein_coding	5	44809027	44820530	MRPS30
ENSG00000113013	11.92483898	11.82356136	protein_coding	5	137890571	137911133	HSPA9
ENSG00000113048	9.860592703	9.623335007	protein_coding	5	71515236	71616473	MRPS27
ENSG00000113068	8.713115882	8.926097022	protein_coding	5	139624624	139682706	PFDN1
ENSG00000113070	8.422952257	8.163833676	protein_coding	5	139712428	139726216	HBEGF
ENSG00000113073	2.35979773	2.039052734	protein_coding	5	139739787	139754722	SLC4A9
ENSG00000113083	10.35161244	10.31600547	protein_coding	5	121398890	121414206	LOX
ENSG00000113108	6.622614428	7.012590419	protein_coding	5	139937853	139973337	APBB3
ENSG00000113119	7.136425764	7.204272857	protein_coding	5	140019012	140024993	TMCO6
ENSG00000113140	11.41730868	12.48692692	protein_coding	5	151040657	151066726	SPARC
ENSG00000113141	9.578112479	9.370301936	protein_coding	5	140026643	140042064	IK
ENSG00000113161	9.332049062	8.675956977	protein_coding	5	74632154	74657929	HMGCR
ENSG00000113163	8.417730894	8.696649474	protein_coding	5	74664311	74807963	COL4A3BP
ENSG00000113194	9.439002622	9.439780161	protein_coding	5	175874629	175937075	FAF2
ENSG00000113205	3.698846687	3.781359661	protein_coding	5	140480234	140483406	PCDHB3
ENSG00000113209	6.188122351	6.941369681	protein_coding	5	140514800	140517703	PCDHB5
ENSG00000113211	3.077135474	3.411459265	protein_coding	5	140529683	140532868	PCDHB6
ENSG00000113212	2.847891871	3.133298822	protein_coding	5	140552243	140555957	PCDHB7
ENSG00000113231	4.19837882	3.532712221	protein_coding	5	76506274	76725632	PDE8B
ENSG00000113240	6.687686437	7.195117037	protein_coding	5	178029665	178057616	CLK4
ENSG00000113248	5.4452544	5.516660875	protein_coding	5	140625147	140627799	PCDHB15
ENSG00000113249	0	0.697730409	protein_coding	5	156456424	156486130	HAVCR1
ENSG00000113262	2.10647801	2.652276565	protein_coding	5	178392096	178423207	GRM6
ENSG00000113269	8.333361768	9.093127603	protein_coding	5	179345911	179499118	RNF130
ENSG00000113272	6.252103507	6.843193319	protein_coding	5	157158205	157168456	THG1L
ENSG00000113273	7.773523328	7.760334499	protein_coding	5	78073032	78281910	ARSB
ENSG00000113282	9.646664866	9.9763021	protein_coding	5	157212751	157286183	CLINT1
ENSG00000113296	3.004694206	3.748356452	protein_coding	5	79287134	79379110	THBS4
ENSG00000113300	9.194306789	9.043827484	protein_coding	5	179921412	180005405	CNOT6

ENSG00000113312	8.284478912	8.558303982	protein_coding	5	159436120	159492518	TTC1
ENSG00000113318	7.46813618	7.480322213	protein_coding	5	79950467	80172631	MSH3
ENSG00000113319	2.471521042	2.912743273	protein_coding	5	80256491	80525975	RASGRF2
ENSG00000113328	9.31529953	9.110276346	protein_coding	5	162864575	162875197	CCNG1
ENSG00000113356	8.245716314	7.940975956	protein_coding	5	89767565	89810370	POLR3G
ENSG00000113360	10.68287268	10.33558093	protein_coding	5	31400604	31532303	DROSHA
ENSG00000113368	10.09562678	9.615349218	protein_coding	5	126112315	126172712	LMNB1
ENSG00000113369	9.385625588	9.827226448	protein_coding	5	90664541	90679176	ARRDC3
ENSG00000113384	10.79215866	10.29936853	protein_coding	5	32124810	32174456	GOLPH3
ENSG00000113387	10.48615002	10.60091569	protein_coding	5	32531739	32604185	SUB1
ENSG00000113389	4.999881834	2.242360793	protein_coding	5	32689176	32791819	NPR3
ENSG00000113391	6.400645076	6.530239731	protein_coding	5	92953775	93447404	FAM172A
ENSG00000113407	12.23446399	12.07415977	protein_coding	5	33440802	33469644	TARS
ENSG00000113441	8.971306859	9.115138778	protein_coding	5	96271098	96373219	LNPEP
ENSG00000113448	7.194570056	6.95589788	protein_coding	5	58264865	59817947	PDE4D
ENSG00000113456	8.46911316	8.693401904	protein_coding	5	34905369	34919094	RAD1
ENSG00000113460	9.380267116	9.326950027	protein_coding	5	34915481	34926101	BRIX1
ENSG00000113494	3.004694206	1.16600992	protein_coding	5	35048861	35230794	PRLR
ENSG00000113504	10.54639963	10.47134059	protein_coding	5	1050499	1112172	SLC12A7
ENSG00000113520	0	0.950786998	protein_coding	5	132009678	132018368	IL4
ENSG00000113522	8.983137923	8.878275203	protein_coding	5	131891711	131980313	RAD50
ENSG00000113525	0	0.390640832	protein_coding	5	131877136	131892530	IL5
ENSG00000113552	9.578112479	9.31220499	protein_coding	5	141371314	141392606	GNPDA1
ENSG00000113555	4.165528823	4.406904905	protein_coding	5	141323150	141349304	PCDH12
ENSG00000113558	9.947096215	10.51123433	protein_coding	5	133492082	133561762	SKP1
ENSG00000113569	11.05768934	10.68480201	protein_coding	5	37288239	37371283	NUP155
ENSG00000113575	9.931915689	9.818806167	protein_coding	5	133530025	133561833	PPP2CA
ENSG00000113578	3.950976309	4.7279204	protein_coding	5	141971743	142077617	FGF1
ENSG00000113580	10.05472661	9.922303325	protein_coding	5	142657496	142815077	NR3C1
ENSG00000113583	9.155101256	9.522629316	protein_coding	5	133291201	133304478	C5orf15
ENSG00000113593	8.118473522	8.283850589	protein_coding	5	64859063	64883376	PPWD1
ENSG00000113594	9.143550557	8.464894292	protein_coding	5	38475065	38608456	LIFR
ENSG00000113595	6.727618145	6.923001245	protein_coding	5	64885507	64921802	TRIM23
ENSG00000113597	7.356139979	7.475288518	protein_coding	5	64920543	64962060	C5orf44
ENSG00000113615	9.43383913	9.250933796	protein_coding	5	133984479	134063513	SEC24A
ENSG00000113621	8.314756163	8.865827498	protein_coding	5	134209460	134237323	TXNDC15
ENSG00000113638	7.935731106	8.090015513	protein_coding	5	40714577	40756077	TTC33
ENSG00000113643	9.791627126	9.954311532	protein_coding	5	167913450	167946304	RARS
ENSG00000113645	10.33647394	10.12753823	protein_coding	5	167718656	167899308	WWC1
ENSG00000113648	10.2188207	10.021894	protein_coding	5	134669590	134735604	H2AFY
ENSG00000113649	10.14227455	9.834615514	protein_coding	5	145826874	145891524	TCERG1
ENSG00000113657	9.507778518	9.900875954	protein_coding	5	146770374	146889619	DPYSL3
ENSG00000113658	9.393626162	9.062408117	protein_coding	5	135468534	135524435	SMAD5
ENSG00000113712	9.723436728	9.355995556	protein_coding	5	148871760	148931007	CSNK1A1
ENSG00000113716	9.512684482	9.370301936	protein_coding	5	149379884	149432386	HMGXB3
ENSG00000113719	10.68396043	10.36615554	protein_coding	5	172261278	172379688	ERGIC1
ENSG00000113721	5.036678094	6.35497494	protein_coding	5	149493400	149535423	PDGFRB
ENSG00000113732	9.829474056	10.28361771	protein_coding	5	172410760	172462448	ATP6V0E1
ENSG00000113734	6.541724534	6.525377513	protein_coding	5	172571445	172591390	BNIP1
ENSG00000113739	10.81749069	11.3533149	protein_coding	5	172741716	172756506	STC2
ENSG00000113742	7.334137164	7.426571535	protein_coding	5	173315283	173388979	CPEB4
ENSG00000113749	0	0.390640832	protein_coding	5	175085033	175113245	HRH2
ENSG00000113758	10.10215133	10.23269872	protein_coding	5	176883609	176901402	DBN1
ENSG00000113761	7.308035767	7.309836726	protein_coding	5	176449697	176508190	ZNF346
ENSG00000113763	5.036678094	2.501982735	protein_coding	5	176237478	176307897	UNC5A
ENSG00000113790	6.592037289	6.246885341	protein_coding	3	184907503	184999778	EHHADH
ENSG00000113810	10.43150107	10.32232659	protein_coding	3	160117062	160152750	SMC4
ENSG00000113811	7.474855471	7.575124073	protein_coding	3	53918437	53926015	
ENSG00000113812	7.781675404	7.924450389	protein_coding	3	53901093	53916229	ACTR8
ENSG00000113838	6.882196036	6.729413443	protein_coding	3	186263862	186288332	TBCCD1
ENSG00000113845	9.385625588	9.68785567	protein_coding	3	119217379	119243937	TIMMDC1
ENSG00000113851	7.01259661	7.551403417	protein_coding	3	3190676	3221394	CRBN
ENSG00000113916	7.89873181	8.443145654	protein_coding	3	187439165	187463515	BCL6

ENSG00000113924	0.869158192	0.950786998	protein_coding	3	120347020	120401418	HGD
ENSG00000113946	3.698846687	3.608232228	protein_coding	3	190040330	190129932	CLDN16
ENSG00000113966	5.360713447	5.526427614	protein_coding	3	97483365	97519953	ARL6
ENSG00000113971	6.92698535	7.210344614	protein_coding	3	132276986	132441303	NPHP3
ENSG00000114019	10.30806544	9.622766057	protein_coding	3	134074716	134094321	AMOTL2
ENSG00000114021	8.470794781	8.914056863	protein_coding	3	100053545	100074449	NIT2
ENSG00000114023	8.274885464	8.896276962	protein_coding	3	122103023	122131181	FAM162A
ENSG00000114026	7.461385447	7.635032283	protein_coding	3	9791628	9829903	OGG1
ENSG00000114030	10.2292898	10.06525187	protein_coding	3	122140796	122233792	KPNA1
ENSG00000114054	9.440719688	9.764649066	protein_coding	3	135969148	136056738	PCCB
ENSG00000114062	9.38294884	9.253876247	protein_coding	15	25582381	25684128	UBE3A
ENSG00000114098	8.201838958	8.196317182	protein_coding	3	137906109	138017231	ARMC8
ENSG00000114107	7.127924785	6.762847836	protein_coding	3	138213186	138313380	CEP70
ENSG00000114113	0.499066092	0	protein_coding	3	139171726	139199589	RBP2
ENSG00000114115	1.16343121	0.697730409	protein_coding	3	139236276	139258671	RBP1
ENSG00000114120	9.587443199	9.592867142	protein_coding	3	140660672	140698775	SLC25A36
ENSG00000114125	9.188186835	9.01636662	protein_coding	3	141457046	141466402	RNF7
ENSG00000114126	9.396283191	9.378413761	protein_coding	3	141663277	141868386	TFDP2
ENSG00000114127	8.426422697	8.402622096	protein_coding	3	142025449	142166904	XRN1
ENSG00000114166	5.60078458	6.104183162	protein_coding	3	20081515	20195896	KAT2B
ENSG00000114200	0.869158192	0	protein_coding	3	165490692	165555260	BCHE
ENSG00000114209	8.231899551	8.394645624	protein_coding	3	167401086	167452727	PDCD10
ENSG00000114251	5.660945913	5.150490275	protein_coding	3	55499743	55523973	WNT5A
ENSG00000114268	8.112022537	8.565427152	protein_coding	3	48555117	48599448	PFKFB4
ENSG00000114270	11.93477291	12.15989435	protein_coding	3	48601506	48632700	COL7A1
ENSG00000114279	0	0.390640832	protein_coding	3	191859684	192485553	FGF12
ENSG00000114302	9.741659723	9.510378177	protein_coding	3	48782030	48885279	PRKAR2A
ENSG00000114315	11.0376904	9.073276144	protein_coding	3	193853934	193856521	HES1
ENSG00000114316	9.249228746	9.386480232	protein_coding	3	49315264	49378145	USP4
ENSG00000114331	8.962967004	8.68797299	protein_coding	3	194995465	195163807	ACAP2
ENSG00000114346	10.00974407	9.881029944	protein_coding	3	172468472	172539264	ECT2
ENSG00000114349	0.499066092	0.390640832	protein_coding	3	50229045	50233949	GNAT1
ENSG00000114353	11.61502948	11.56120056	protein_coding	3	50263724	50296787	GNAI2
ENSG00000114354	10.54320643	10.45161314	protein_coding	3	100428205	100467810	TFG
ENSG00000114374	0.499066092	0	protein_coding	Y	14813160	14972764	USP9Y
ENSG00000114378	4.942865586	3.96493948	protein_coding	3	50337320	50349812	HYAL1
ENSG00000114383	7.639400234	7.918899532	protein_coding	3	50357458	50365682	TUSC2
ENSG00000114388	7.488200893	7.815148897	protein_coding	3	50384761	50388522	NPRL2
ENSG00000114391	10.70554504	11.17987491	protein_coding	3	101399935	101405626	RPL24
ENSG00000114395	7.31554162	7.844724842	protein_coding	3	50388126	50395891	CYB561D2
ENSG00000114405	6.386460534	6.091080508	protein_coding	3	62304648	62321888	C3orf14
ENSG00000114416	10.47739354	10.30185857	protein_coding	3	180585929	180694950	FXR1
ENSG00000114423	7.206730419	7.487839978	protein_coding	3	105374305	105588396	CBLB
ENSG00000114439	10.46394731	10.46119237	protein_coding	3	107241783	107530171	BBX
ENSG00000114446	9.034071956	8.775550005	protein_coding	3	107879659	107941417	IFT57
ENSG00000114450	8.613115289	8.349968848	protein_coding	3	179116990	179169378	GNB4
ENSG00000114455	0	0.950786998	protein_coding	3	108015376	108097132	HHLA2
ENSG00000114473	6.35766608	6.424524724	protein_coding	3	197615946	197687013	IQCG
ENSG00000114480	9.527303063	9.660952415	protein_coding	3	81538850	81811312	GBE1
ENSG00000114491	8.890689734	9.023280994	protein_coding	3	124449213	124464040	UMPS
ENSG00000114503	9.839921696	9.535986486	protein_coding	3	196662273	196669468	NCBP2
ENSG00000114520	7.76258174	7.998246367	protein_coding	3	125165495	125239041	SNX4
ENSG00000114529	6.704935538	6.210997888	protein_coding	3	111805182	111849851	C3orf52
ENSG00000114541	7.384964606	6.24096575	protein_coding	3	69219141	69591734	FRMD4B
ENSG00000114544	8.027710784	7.890820294	protein_coding	3	125725198	125820404	SLC41A3
ENSG00000114547	0.869158192	0.390640832	protein_coding	3	125687987	125702297	ROPN1B
ENSG00000114554	11.47772401	11.03302417	protein_coding	3	126707437	126756235	PLXNA1
ENSG00000114573	9.535360944	9.119984878	protein_coding	3	113465664	113530903	ATP6V1A
ENSG00000114626	6.979904289	7.718344862	protein_coding	3	127391778	127399768	ABTB1
ENSG00000114631	9.812996626	10.30576281	protein_coding	3	127348024	127391652	PODXL2
ENSG00000114646	1.616589159	1.353254395	protein_coding	3	47603729	47622282	CSPG5
ENSG00000114648	8.453889713	8.492561507	protein_coding	3	47324407	47388306	KLHL18
ENSG00000114650	10.11296043	9.975856648	protein_coding	3	47455184	47518616	SCAP

ENSG00000114654	0	1.353254395	protein_coding	3	128720472	128759585	CCDC48
ENSG00000114656	1.616589159	0.950786998	protein_coding	3	128628709	128721533	KIAA1257
ENSG00000114670	5.072559152	5.072639111	protein_coding	3	130745694	131069309	NEK11
ENSG00000114686	10.3876657	10.21242219	protein_coding	3	131181068	131221827	MRPL3
ENSG00000114698	3.698846687	4.528525526	protein_coding	3	145910126	145968966	PLSCR4
ENSG00000114735	7.35977471	7.862183683	protein_coding	3	50606583	50622366	HEMK1
ENSG00000114737	4.800350936	4.342020395	protein_coding	3	50643921	50649262	CISH
ENSG00000114738	9.689899429	9.29015931	protein_coding	3	50648951	50686720	MAPKAPK3
ENSG00000114739	5.239686603	4.794098771	protein_coding	3	38495342	38524948	ACVR2B
ENSG00000114742	8.207901394	8.741364159	protein_coding	3	39093489	39138155	WDR48
ENSG00000114744	7.988335976	7.683850422	protein_coding	3	149456257	149470286	COMMD2
ENSG00000114745	8.342575358	8.398639372	protein_coding	3	39138150	39149854	GORASP1
ENSG00000114757	1.616589159	2.039052734	protein_coding	3	179512746	179754841	PEX5L
ENSG00000114767	9.100750797	8.736113084	protein_coding	3	51967446	51975957	RRP9
ENSG00000114770	9.175868488	9.395168311	protein_coding	3	183637722	183735803	ABCC5
ENSG00000114771	3.393122761	0	protein_coding	3	151531825	151546272	AADAC
ENSG00000114779	8.53489446	8.32500368	protein_coding	3	52002526	52017425	ABHD14B
ENSG00000114784	7.308035767	7.519971735	protein_coding	3	40351175	40353915	EIF1B
ENSG00000114786	0.869158192	1.925536307	protein_coding	3	52009066	52023199	
ENSG00000114790	3.077135474	2.579085888	protein_coding	3	153838792	153975616	ARHGEF26
ENSG00000114796	7.182306324	7.791043682	protein_coding	3	183353356	183398733	KLHL24
ENSG00000114805	0.499066092	0.697730409	protein_coding	3	155093369	155462856	PLCH1
ENSG00000114812	2.238690726	1.925536307	protein_coding	3	42530791	42579059	VIPR1
ENSG00000114841	7.15746161	7.41347308	protein_coding	3	52350335	52434507	DNAH1
ENSG00000114850	10.9593005	10.88880815	protein_coding	3	156257929	156272973	SSR3
ENSG00000114853	7.680671792	8.206984494	protein_coding	3	42695176	42709072	ZBTB47
ENSG00000114854	0.869158192	1.16600992	protein_coding	3	52485118	52488086	TNNC1
ENSG00000114857	9.497916256	9.791222404	protein_coding	3	42642106	42690227	NKTR
ENSG00000114859	6.835971536	6.084484284	protein_coding	3	184063973	184079439	CLCN2
ENSG00000114861	9.24628631	8.963424725	protein_coding	3	71003844	71633140	FOXP1
ENSG00000114867	13.3821508	12.99210928	protein_coding	3	184032283	184053146	EIF4G1
ENSG00000114902	9.001868107	9.161316205	protein_coding	3	52738971	52742182	SPCS1
ENSG00000114904	7.437505974	7.572769481	protein_coding	3	52744800	52804965	NEK4
ENSG00000114923	6.35766608	4.872768657	protein_coding	2	220492049	220506702	SLC4A3
ENSG00000114933	7.72925009	7.641787375	protein_coding	2	206858445	206951027	INO80D
ENSG00000114942	9.981711773	10.26660082	protein_coding	2	207024309	207027652	EEF1B2
ENSG00000114956	8.251597515	8.278081046	protein_coding	2	74153953	74186088	DGUOK
ENSG00000114978	8.954578658	8.753889302	protein_coding	2	74382165	74406025	MOB1A
ENSG00000114982	9.420848964	9.401152631	protein_coding	2	97258907	97308524	KANSL3
ENSG00000114988	8.675694252	8.83570377	protein_coding	2	97371666	97405801	LMAN2L
ENSG00000114993	8.698837588	8.595894166	protein_coding	2	74652963	74669549	RTKN
ENSG00000114999	10.08688122	9.730200815	protein_coding	2	113239731	113290227	TTL
ENSG00000115008	5.695874189	4.319727133	protein_coding	2	113531492	113542167	IL1A
ENSG00000115009	6.290697143	5.456629825	protein_coding	2	228678558	228682272	CCL20
ENSG00000115020	8.783815073	8.401295742	protein_coding	2	209130991	209223475	PIKFYVE
ENSG00000115041	6.579622637	5.47691899	protein_coding	2	95963052	96051825	KCNIP3
ENSG00000115042	7.789781675	7.716212954	protein_coding	2	96068469	96082364	FAHD2A
ENSG00000115053	13.74686114	13.27940439	protein_coding	2	232318242	232348352	NCL
ENSG00000115073	9.53937307	9.396500307	protein_coding	2	98272431	98280570	ACTR1B
ENSG00000115084	8.66984994	8.417132063	protein_coding	2	114462588	114514400	SLC35F5
ENSG00000115085	0.869158192	1.802319292	protein_coding	2	98330023	98356325	ZAP70
ENSG00000115091	11.28114357	11.20194453	protein_coding	2	114647537	114720173	ACTR3
ENSG00000115107	10.19307491	9.760517066	protein_coding	2	119981384	120023228	STEAP3
ENSG00000115109	7.481543613	7.48283249	protein_coding	2	120770581	120936695	EPB41L5
ENSG00000115112	7.824391858	6.624193585	protein_coding	2	121974163	122042783	TFCP2L1
ENSG00000115128	8.959377969	8.896276962	protein_coding	2	24290454	24299313	
ENSG00000115129	7.058051099	7.405556541	protein_coding	2	24300303	24308731	TP53I3
ENSG00000115137	5.346128795	5.404622322	protein_coding	2	25166505	25194963	DNAJC27
ENSG00000115145	8.154494062	7.817139602	protein_coding	2	152973315	153032506	STAM2
ENSG00000115155	0	0.697730409	protein_coding	2	26680071	26781566	OTOF
ENSG00000115159	9.111570361	8.605140645	protein_coding	2	157291802	157470247	GPD2
ENSG00000115163	7.813830732	8.235547225	protein_coding	2	26987157	27023935	CENPA
ENSG00000115165	0	1.353254395	protein_coding	2	158271131	158345473	CYTIP

ENSG00000115170	7.888702948	7.950075687	protein_coding	2	158592958	158732374	ACVR1
ENSG00000115183	9.478823042	9.219670215	protein_coding	2	159825146	160089170	TANC1
ENSG00000115194	3.077135474	2.652276565	protein_coding	2	27476552	27498685	SLC30A3
ENSG00000115204	9.024943975	9.493672366	protein_coding	2	27532360	27548547	MPV17
ENSG00000115207	9.859309406	9.716934393	protein_coding	2	27548716	27579868	GTF3C2
ENSG00000115211	8.004679484	8.541545191	protein_coding	2	27587219	27593353	EIF2B4
ENSG00000115216	10.47906555	11.19066403	protein_coding	2	27650657	27665126	NRBP1
ENSG00000115221	6.693459083	1.925536307	protein_coding	2	160956177	161128399	ITGB6
ENSG00000115226	4.19837882	2.334191469	protein_coding	2	27714750	27718112	FNDC4
ENSG00000115233	10.25342625	10.21468926	protein_coding	2	162164549	162268228	PSMD14
ENSG00000115234	10.55355859	10.49547112	protein_coding	2	27593389	27599995	SNX17
ENSG00000115239	6.681890601	6.610484676	protein_coding	2	53759810	54087297	ASB3
ENSG00000115241	11.44943256	11.50411343	protein_coding	2	27604061	27632554	PPM1G
ENSG00000115252	0	0.390640832	protein_coding	2	183004763	183387919	PDE1A
ENSG00000115255	8.467429577	8.106387334	protein_coding	19	1491023	1497926	REEP6
ENSG00000115257	6.163378456	6.827478203	protein_coding	19	1481427	1490407	PCSK4
ENSG00000115266	7.527512071	7.492830145	protein_coding	19	1450148	1473243	APC2
ENSG00000115267	8.088119026	8.595894166	protein_coding	2	163123589	163175213	IFIH1
ENSG00000115268	11.48148243	11.70305416	protein_coding	19	1438363	1440492	RPS15
ENSG00000115271	6.975172889	6.737844962	protein_coding	2	163175350	163228105	GCA
ENSG00000115274	7.726437377	7.987679908	protein_coding	2	74682150	74688011	INO80B
ENSG00000115275	9.931305133	10.2460598	protein_coding	2	74688184	74692537	MOGS
ENSG00000115282	7.842689815	8.262094789	protein_coding	2	74710200	74722013	TTC31
ENSG00000115286	8.505663452	8.854241196	protein_coding	19	1383526	1395587	NDUFS7
ENSG00000115289	7.102117104	7.343375265	protein_coding	2	74732170	74735707	PCGF1
ENSG00000115290	3.652182994	2.912743273	protein_coding	2	165349322	165478358	GRB14
ENSG00000115295	8.889432943	9.089837938	protein_coding	2	29320560	29412509	CLIP4
ENSG00000115297	0.869158192	2.420525079	protein_coding	2	74740590	74744769	TLX2
ENSG00000115306	12.29788714	12.3953757	protein_coding	2	54683422	54896812	SPTBN1
ENSG00000115307	9.660729799	10.10775128	protein_coding	2	74753772	74757066	AUP1
ENSG00000115310	11.3772983	11.30235327	protein_coding	2	55199325	55339757	RTN4
ENSG00000115317	7.537174746	7.679480043	protein_coding	2	74756504	74760472	HTRA2
ENSG00000115318	4.517578978	4.406904905	protein_coding	2	74759541	74782817	LOXL3
ENSG00000115325	6.129712275	5.734544355	protein_coding	2	74776153	74784681	DOK1
ENSG00000115339	7.997697782	5.792353798	protein_coding	2	166604101	166651192	GALNT3
ENSG00000115350	7.765324926	8.345837872	protein_coding	2	75185619	75197255	POLE4
ENSG00000115353	0	0.390640832	protein_coding	2	75276231	75426826	TACR1
ENSG00000115355	9.337589303	9.169909673	protein_coding	2	55514978	55647057	CCDC88A
ENSG00000115363	5.141750194	3.87606762	protein_coding	2	75696428	75796848	FAM176A
ENSG00000115364	8.436784206	8.744505654	protein_coding	2	75873909	75917977	MRPL19
ENSG00000115365	8.98077946	8.770422115	protein_coding	2	211295973	211342376	LANCL1
ENSG00000115368	9.242353704	9.273582355	protein_coding	2	190306159	190340291	WDR75
ENSG00000115380	9.510233586	10.07318632	protein_coding	2	56093102	56151274	EFEMP1
ENSG00000115392	6.528867325	7.070480904	protein_coding	2	58386378	58468507	FANCL
ENSG00000115414	12.83707757	13.25762674	protein_coding	2	216225163	216300895	FN1
ENSG00000115415	10.49526681	10.83234969	protein_coding	2	191829084	191885686	STAT1
ENSG00000115419	10.96676801	10.42966183	protein_coding	2	191745553	191830278	GLS
ENSG00000115421	7.202688343	7.148445524	protein_coding	2	60983365	61029220	PAPOLG
ENSG00000115423	0.869158192	2.144285137	protein_coding	2	84743579	85046713	DNAH6
ENSG00000115425	5.637181576	5.004391715	protein_coding	2	216861052	216947678	PECR
ENSG00000115446	7.68357507	7.652976045	protein_coding	2	99225042	99234978	UNC50
ENSG00000115457	3.603959378	2.501982735	protein_coding	2	217497551	217529159	IGFBP2
ENSG00000115459	7.726437377	8.027771115	protein_coding	2	85581517	85618875	ELMOD3
ENSG00000115461	2.57521082	2.788380093	protein_coding	2	217536828	217560248	IGFBP5
ENSG00000115464	9.990530519	10.27023828	protein_coding	2	61414591	61697904	USP34
ENSG00000115468	2.57521082	1.353254395	protein_coding	2	233470767	233547491	EFHD1
ENSG00000115474	1.407729925	0.697730409	protein_coding	2	233631174	233641278	KCNJ13
ENSG00000115484	11.02829989	10.99528021	protein_coding	2	62095224	62115939	CCT4
ENSG00000115486	8.901951857	9.104582717	protein_coding	2	85774743	85788670	GGCX
ENSG00000115504	8.324088959	8.282410365	protein_coding	2	62900986	63273622	EHBP1
ENSG00000115507	5.695874189	4.640685763	protein_coding	2	63277192	63284971	OTX1
ENSG00000115514	7.967047117	7.827052132	protein_coding	2	99921090	99957165	TXNDC9
ENSG00000115520	7.878603882	7.266786485	protein_coding	2	198318147	198340032	COQ10B

ENSG00000115524	11.43378338	11.3074958	protein_coding	2	198256698	198299815	SF3B1
ENSG00000115525	5.191549282	4.710890357	protein_coding	2	86066267	86116137	ST3GAL5
ENSG00000115539	6.92698535	6.923001245	protein_coding	2	101179152	101193197	PDCL3
ENSG00000115540	8.292108003	7.852510423	protein_coding	2	198380295	198418423	MOB4
ENSG00000115541	9.305909516	9.379087693	protein_coding	2	198364718	198368181	HSPE1
ENSG00000115548	9.207478296	9.693285025	protein_coding	2	86667770	86719839	KDM3A
ENSG00000115556	6.998676229	6.742042315	protein_coding	2	219472488	219501907	PLCD4
ENSG00000115561	10.08248848	10.13434017	protein_coding	2	86730554	86948245	CHMP3
ENSG00000115568	8.986668406	8.786767612	protein_coding	2	219502639	219524378	ZNF142
ENSG00000115590	0.869158192	0	protein_coding	2	102608306	102645006	IL1R2
ENSG00000115592	0.869158192	0.697730409	protein_coding	2	219687106	219696809	PRKAG3
ENSG00000115594	8.150302734	7.10998981	protein_coding	2	102681004	102796334	IL1R1
ENSG00000115596	0.499066092	1.16600992	protein_coding	2	219724544	219738955	WNT6
ENSG00000115598	3.004694206	1.667587519	protein_coding	2	102803433	102856462	IL1RL2
ENSG00000115602	3.211941663	0.390640832	protein_coding	2	102927962	102968497	IL1RL1
ENSG00000115604	5.60078458	5.09905904	protein_coding	2	102927989	103015218	IL18R1
ENSG00000115607	0	0.390640832	protein_coding	2	103035149	103069025	IL18RAP
ENSG00000115616	1.616589159	1.518964905	protein_coding	2	103236166	103327777	SLC9A2
ENSG00000115641	7.834876234	4.319727133	protein_coding	2	105974169	106054960	FHL2
ENSG00000115648	8.175269974	7.284161038	protein_coding	2	238394071	238463961	MLPH
ENSG00000115649	8.940084182	9.012896966	protein_coding	2	220036619	220042828	CNPPD1
ENSG00000115652	9.694224448	9.528108569	protein_coding	2	106709759	106810795	UXS1
ENSG00000115657	5.729976786	5.871116342	protein_coding	2	220074490	220083712	ABCB6
ENSG00000115661	7.876068031	7.86795666	protein_coding	2	220110177	220115059	STK16
ENSG00000115677	13.5663983	13.09629528	protein_coding	2	242166679	242256476	HDLBP
ENSG00000115685	9.052156467	8.899098923	protein_coding	2	242088991	242123067	PPP1R7
ENSG00000115687	8.105542576	7.772696723	protein_coding	2	242045514	242089679	PASK
ENSG00000115694	10.51984191	10.26696498	protein_coding	2	242432089	242449145	STK25
ENSG00000115705	0.499066092	0.390640832	protein_coding	2	1377995	1547483	TPO
ENSG00000115718	0.499066092	1.353254395	protein_coding	2	128176003	128186822	PROC
ENSG00000115738	6.005279909	5.018301917	protein_coding	2	8818975	8824583	ID2
ENSG00000115750	7.292905885	7.090370599	protein_coding	2	9983483	10074537	TAF1B
ENSG00000115756	10.84692499	9.901814226	protein_coding	2	10443015	10567743	HPCAL1
ENSG00000115758	10.95269697	10.6241267	protein_coding	2	10580094	10588630	ODC1
ENSG00000115760	9.988771077	10.12834013	protein_coding	2	32582096	32843966	BIRC6
ENSG00000115761	8.75235931	8.554729165	protein_coding	2	10710892	10830101	NOL10
ENSG00000115762	10.50103856	10.16748693	protein_coding	2	131862420	132111282	PLEKHB2
ENSG00000115806	10.36794799	10.314597	protein_coding	2	171784974	171823639	GORASP2
ENSG00000115808	8.646233023	8.560682282	protein_coding	2	37070783	37193615	STRN
ENSG00000115816	9.223526019	9.509147322	protein_coding	2	37428755	37458856	CEBPZ
ENSG00000115825	9.222528249	9.570029165	protein_coding	2	37477645	37551951	PRKD3
ENSG00000115827	7.969428119	7.768587741	protein_coding	2	172290727	172341562	DCAF17
ENSG00000115828	1.616589159	1.667587519	protein_coding	2	37571717	37600465	QPCT
ENSG00000115839	9.488816239	9.380434611	protein_coding	2	135809835	135933964	RAB3GAP1
ENSG00000115840	8.261346552	8.015686953	protein_coding	2	172640880	172864766	SLC25A12
ENSG00000115841	4.568725997	4.604259349	protein_coding	2	38150330	38294285	FAM82A1
ENSG00000115844	6.138202758	5.200150955	protein_coding	2	172964167	172967628	DLX2
ENSG00000115850	1.407729925	0.950786998	protein_coding	2	136545410	136594750	LCT
ENSG00000115866	9.950715896	9.821287826	protein_coding	2	136664247	136743670	DARS
ENSG00000115875	10.48073561	10.84386835	protein_coding	2	38970741	38978636	SRSF7
ENSG00000115884	10.89718911	9.875309148	protein_coding	2	20400558	20425194	SDC1
ENSG00000115896	0	1.16600992	protein_coding	2	198669426	199437305	PLCL1
ENSG00000115902	7.633406721	8.657187659	protein_coding	2	65215611	65250999	SLC1A4
ENSG00000115904	8.950968668	9.176127473	protein_coding	2	39208537	39351486	SOS1
ENSG00000115919	9.362260424	9.321350625	protein_coding	2	143635067	143799890	KYNU
ENSG00000115934	0.499066092	0	lincRNA	12	23334268	23404433	
ENSG00000115935	2.847891871	2.039052734	protein_coding	2	175424300	175547644	WIPF1
ENSG00000115942	8.748205354	8.553535588	protein_coding	2	201773696	201828403	ORC2
ENSG00000115944	9.167597433	9.602710123	protein_coding	2	42560686	42596150	COX7A2L
ENSG00000115946	7.969428119	8.051639961	protein_coding	2	68384976	68403370	PNO1
ENSG00000115947	8.016241092	7.915187059	protein_coding	2	148691732	148779147	ORC4
ENSG00000115956	0	0.390640832	protein_coding	2	68592305	68624585	PLEK
ENSG00000115963	9.965104479	9.806834418	protein_coding	2	151324709	151395525	RND3

ENSG00000115966	9.232475031	8.990138507	protein_coding	2	175936978	176033110	ATF2
ENSG00000115970	8.894453552	8.867749534	protein_coding	2	43393800	43823185	THADA
ENSG00000115977	6.598204782	6.615068802	protein_coding	2	69688532	69901481	AAK1
ENSG00000115993	9.392739397	9.250933796	protein_coding	2	202241930	202316334	TRAK2
ENSG00000115998	6.023726639	6.515603638	protein_coding	2	70377012	70475747	C2orf42
ENSG00000116001	8.579219424	8.985720419	protein_coding	2	70436576	70475792	TIA1
ENSG00000116005	8.97959878	8.76733661	protein_coding	2	70484518	70508323	PCYOX1
ENSG00000116016	11.0365554	11.09463689	protein_coding	2	46520806	46613836	EPAS1
ENSG00000116017	4.097495944	4.127774132	protein_coding	19	926037	972781	ARID3A
ENSG00000116030	9.834054211	9.764133213	protein_coding	2	203070903	203103331	SUMO1
ENSG00000116031	0.499066092	1.353254395	protein_coding	2	71057347	71062952	CD207
ENSG00000116032	2.762599152	2.242360793	protein_coding	19	1000418	1009731	GRIN3B
ENSG00000116035	4.131913373	4.489113623	protein_coding	2	71127720	71160576	VAX2
ENSG00000116039	4.261916566	4.448594745	protein_coding	2	71163012	71192555	ATP6V1B1
ENSG00000116044	10.34015849	10.08190551	protein_coding	2	178092323	178257425	NFE2L2
ENSG00000116062	10.43020521	10.67220966	protein_coding	2	48010221	48034092	MSH6
ENSG00000116095	7.262161623	7.258020107	protein_coding	2	179345195	179370093	PLEKHA3
ENSG00000116096	8.006999229	8.015686953	protein_coding	2	73114489	73119287	SPR
ENSG00000116106	3.335220907	3.027231696	protein_coding	2	222282747	222438922	EPHA4
ENSG00000116117	3.393122761	5.22435463	protein_coding	2	205410516	206484886	PARD3B
ENSG00000116120	9.930694319	9.628445484	protein_coding	2	223435255	223521056	FARSB
ENSG00000116127	8.380642463	8.736113084	protein_coding	2	73612886	73837920	ALMS1
ENSG00000116128	9.3522601	8.976843446	protein_coding	1	147013182	147098017	BCL9
ENSG00000116132	0.869158192	1.518964905	protein_coding	1	170631869	170708560	PRRX1
ENSG00000116133	11.69705415	10.81601773	protein_coding	1	55315300	55352921	DHCR24
ENSG00000116138	7.68357507	7.795089291	protein_coding	1	15853308	15918874	DNAJC16
ENSG00000116141	0.499066092	0.390640832	protein_coding	1	220701568	220837803	MARK1
ENSG00000116151	5.575999561	5.953060722	protein_coding	1	2252692	2323146	MORN1
ENSG00000116157	0	0.390640832	protein_coding	1	53068044	53074723	GPX7
ENSG00000116161	8.552470601	9.162882465	protein_coding	1	174968300	174980851	CACYBP
ENSG00000116171	8.611591722	8.861975727	protein_coding	1	53392901	53517375	SCP2
ENSG00000116176	5.458875168	7.449853178	protein_coding	16	1271651	1275257	TPSG1
ENSG00000116183	2.471521042	5.339632724	protein_coding	1	176432307	176814735	PAPPA2
ENSG00000116191	8.090308512	7.836897019	protein_coding	1	178694300	179065553	RALGPS2
ENSG00000116194	1.16343121	0.950786998	protein_coding	1	178818840	178840187	ANGPTL1
ENSG00000116198	8.713115882	8.714915547	protein_coding	1	3728645	3773778	CEP104
ENSG00000116199	9.294560225	9.406451268	protein_coding	1	178994939	179045697	FAM20B
ENSG00000116205	6.236372259	6.419292172	protein_coding	1	54519260	54578192	TCEANC2
ENSG00000116209	9.015757872	9.652615414	protein_coding	1	54497347	54519177	TMEM59
ENSG00000116212	8.489164384	8.423679567	protein_coding	1	54411750	54433841	LRRC42
ENSG00000116213	8.023133844	8.237034976	protein_coding	1	3547331	3569325	WRAP73
ENSG00000116221	10.15121608	9.991366237	protein_coding	1	54649714	54686603	MRPL37
ENSG00000116237	10.14648923	10.1891691	protein_coding	1	6281252	6296032	ICMT
ENSG00000116251	9.9233443	10.2911566	protein_coding	1	6241329	6269449	RPL22
ENSG00000116254	3.393122761	5.271576845	protein_coding	1	6161853	6240183	CHD5
ENSG00000116260	11.1012235	11.71244306	protein_coding	1	180123969	180173165	QSOX1
ENSG00000116266	7.91858278	8.349968848	protein_coding	1	109289296	109352148	STXBP3
ENSG00000116273	7.89873181	7.817139602	protein_coding	1	6673745	6684093	PHF13
ENSG00000116285	11.00004714	10.55304383	protein_coding	1	8064464	8086368	ERRFI1
ENSG00000116288	10.12208492	10.67165966	protein_coding	1	8014351	8045565	PARK7
ENSG00000116299	3.911897206	4.15321211	protein_coding	1	109656301	109749401	KIAA1324
ENSG00000116337	9.600559263	9.580614711	protein_coding	1	110158726	110174673	AMPD2
ENSG00000116350	9.832093067	9.942049496	protein_coding	1	29474255	29508499	SRSF4
ENSG00000116353	8.237837231	8.279525596	protein_coding	1	29519385	29557454	MECR
ENSG00000116396	5.729976786	5.709041716	protein_coding	1	110753965	110825722	KCNC4
ENSG00000116406	8.893200037	9.272857279	protein_coding	1	184659637	184724047	EDEM3
ENSG00000116455	8.135536446	8.308116953	protein_coding	1	111982512	111991998	WDR77
ENSG00000116459	9.794987046	10.08768916	protein_coding	1	111991486	112005395	ATP5F1
ENSG00000116473	8.579219424	8.305283181	protein_coding	1	112084840	112259313	RAP1A
ENSG00000116478	10.27283443	10.24384153	protein_coding	1	32757687	32799236	HDAC1
ENSG00000116489	10.627035	10.45481329	protein_coding	1	113161795	113214241	CAPZA1
ENSG00000116497	7.491518048	7.924450389	protein_coding	1	33282368	33324476	S100PBP
ENSG00000116514	8.14188337	7.70765368	protein_coding	1	33402046	33430286	RNF19B

ENSG00000116521	10.30947573	10.60322483	protein_coding	1	155225770	155232221	SCAMP3
ENSG00000116525	6.005279909	5.383282767	protein_coding	1	33611003	33647671	TRIM62
ENSG00000116539	9.709260716	9.811335474	protein_coding	1	155305059	155532598	ASH1L
ENSG00000116544	0.499066092	0.950786998	protein_coding	1	35331037	35395186	DLGAP3
ENSG00000116560	11.87157285	11.63231638	protein_coding	1	35641979	35658749	SFPQ
ENSG00000116574	6.738827102	7.625976011	protein_coding	1	228870824	228882416	RHOU
ENSG00000116580	9.108333003	9.145559356	protein_coding	1	155719508	155829191	GON4L
ENSG00000116584	10.92597637	11.50349546	protein_coding	1	155916645	155966129	ARHGEF2
ENSG00000116586	8.146099194	8.310945169	protein_coding	1	156024543	156028301	LAMTOR2
ENSG00000116604	10.07310926	9.921840881	protein_coding	1	156433519	156470620	MEF2D
ENSG00000116641	8.678607554	9.151882762	protein_coding	1	62920399	63153969	DOCK7
ENSG00000116649	10.76466124	10.77128306	protein_coding	1	11114641	11120081	SRM
ENSG00000116652	0.869158192	0.697730409	protein_coding	1	64014635	64015911	DLEU2L
ENSG00000116661	8.373468605	7.805153958	protein_coding	1	11708424	11715842	FBXO2
ENSG00000116663	6.014532757	5.506827567	protein_coding	1	11724181	11734411	FBXO6
ENSG00000116667	6.005279909	6.130036786	protein_coding	1	184356192	184598154	C1orf21
ENSG00000116668	5.207774174	5.526427614	protein_coding	1	185126212	185260897	SWT1
ENSG00000116670	8.879339004	8.638170933	protein_coding	1	11734538	11751707	MAD2L2
ENSG00000116675	6.305850085	6.382123086	protein_coding	1	65713902	65881552	DNAJC6
ENSG00000116678	5.124761037	4.468996429	protein_coding	1	65886248	66107242	LEPR
ENSG00000116679	9.003030709	9.435898843	protein_coding	1	185265520	185286461	IVNS1ABP
ENSG00000116685	9.199386995	9.141593107	protein_coding	1	11979648	11986485	KIAA2013
ENSG00000116688	10.55593704	10.60926879	protein_coding	1	12040238	12073571	MFN2
ENSG00000116690	1.16343121	1.667587519	protein_coding	1	186265405	186283694	PRG4
ENSG00000116691	8.247679379	8.322202916	protein_coding	1	12079523	12092102	MIIP
ENSG00000116698	10.33877788	10.17759419	protein_coding	1	183441351	183567381	SMG7
ENSG00000116701	4.999881834	4.604259349	protein_coding	1	183524698	183560011	NCF2
ENSG00000116703	0.499066092	0.390640832	protein_coding	1	186412698	186430254	PDC
ENSG00000116704	7.246540262	7.621426466	protein_coding	1	67465015	67519782	SLC35D1
ENSG00000116711	2.35979773	3.993387124	protein_coding	1	186798085	186958113	PLA2G4A
ENSG00000116717	9.75690141	9.395834462	protein_coding	1	68150744	68154021	GADD45A
ENSG00000116729	8.915598341	9.089837938	protein_coding	1	68564142	68698803	WLS
ENSG00000116731	8.322227223	8.293892101	protein_coding	1	14026693	14151560	PRDM2
ENSG00000116741	4.75690578	3.570966319	protein_coding	1	192778169	192781403	RGS2
ENSG00000116745	3.335220907	0	protein_coding	1	68894505	68915642	RPE65
ENSG00000116747	8.508940744	8.624594823	protein_coding	1	193028552	193060907	TROVE2
ENSG00000116748	0	0.390640832	protein_coding	1	115215719	115238239	AMPD1
ENSG00000116750	8.659565077	8.806942451	protein_coding	1	192984889	193029237	UCHL5
ENSG00000116752	7.940593421	8.38394126	protein_coding	1	115110178	115124260	BCAS2
ENSG00000116754	10.57012597	10.96012994	protein_coding	1	70671365	70718735	SRSF11
ENSG00000116761	6.462804234	6.716673044	protein_coding	1	70876901	70905534	CTH
ENSG00000116771	5.316509574	4.777836593	protein_coding	1	15898848	15911605	AGMAT
ENSG00000116774	5.431503808	6.287656023	protein_coding	1	114522063	114524876	OLFML3
ENSG00000116785	0.869158192	0	protein_coding	1	196743925	196764536	CFHR3
ENSG00000116786	9.886021803	10.00452874	protein_coding	1	16010827	16061264	PLEKHM2
ENSG00000116791	6.716321419	6.480864016	protein_coding	1	75171170	75199092	CRYZ
ENSG00000116793	8.018542337	8.496293532	protein_coding	1	114234854	114302111	PHTF1
ENSG00000116809	8.135536446	8.503728743	protein_coding	1	16268364	16302627	ZBTB17
ENSG00000116815	6.554468172	7.188980716	protein_coding	1	117057157	117113661	CD58
ENSG00000116819	4.165528823	4.048652948	protein_coding	1	36038971	36060929	TFAP2E
ENSG00000116830	8.929116952	8.695567763	protein_coding	1	117602925	117645492	TTF2
ENSG00000116833	4.593635215	4.42790041	protein_coding	1	199996730	200146552	NR5A2
ENSG00000116852	8.337054268	7.724721747	protein_coding	1	200938520	200992828	KIF21B
ENSG00000116857	9.088754802	8.913126522	protein_coding	1	201103900	201140702	TMEM9
ENSG00000116863	7.974178365	8.393311914	protein_coding	1	36554476	36559533	ADPRHL2
ENSG00000116871	10.65577847	10.75553892	protein_coding	1	36621180	36646450	MAP7D1
ENSG00000116874	7.281453427	7.575124073	protein_coding	1	119573839	119683294	WARS2
ENSG00000116882	0	0.390640832	protein_coding	1	119911402	119936753	HAO2
ENSG00000116883	5.223818625	5.339632724	protein_coding	1	36789335	36794818	
ENSG00000116885	4.962122459	5.66550919	protein_coding	1	36881428	36916086	OSCP1
ENSG00000116898	9.36407124	9.50483104	protein_coding	1	36921319	36930038	MRPS15
ENSG00000116903	7.95748362	7.628245414	protein_coding	1	231468480	231473598	EXOC8
ENSG00000116906	9.193288598	9.245770021	protein_coding	1	231376953	231413719	GNPAT

ENSG00000116918	8.417730894	8.262094789	protein_coding	1	231664287	231702270	TSNAX
ENSG00000116922	6.771940731	7.057067116	protein_coding	1	38147242	38157914	C1orf109
ENSG00000116954	8.105542576	8.331981894	protein_coding	1	39303870	39325495	RRAGC
ENSG00000116957	7.363400307	7.67728988	protein_coding	1	235530675	235612283	TBCE
ENSG00000116962	1.16343121	0.697730409	protein_coding	1	236139130	236228462	NID1
ENSG00000116977	8.239811039	8.385283657	protein_coding	1	236681300	236716281	LGALS8
ENSG00000116983	4.491309013	3.608232228	protein_coding	1	40144320	40157361	HPCAL4
ENSG00000116984	9.545769354	9.829200569	protein_coding	1	236958581	237067281	MTR
ENSG00000116985	6.26766507	5.393992	protein_coding	1	40222854	40254533	BMP8B
ENSG00000116990	3.652182994	2.912743273	protein_coding	1	40361098	40367928	MYCL1
ENSG00000116991	7.026383958	7.321103092	protein_coding	1	232533711	232697304	SIPA1L2
ENSG00000117000	8.199812472	8.512355001	protein_coding	1	40627045	40706593	RLF
ENSG00000117009	1.407729925	2.334191469	protein_coding	1	241695434	241758944	KMO
ENSG00000117010	4.352265886	4.841813558	protein_coding	1	40997233	41013841	ZNF684
ENSG00000117013	5.018397273	4.810179682	protein_coding	1	41249684	41306124	KCNQ4
ENSG00000117016	6.400645076	5.6022688	protein_coding	1	41086351	41131329	RIMS3
ENSG00000117020	7.913645586	8.954409067	protein_coding	1	243651535	244014381	AKT3
ENSG00000117036	8.650690744	8.579568843	protein_coding	1	157090983	157108266	ETV3
ENSG00000117054	8.235860718	8.500015927	protein_coding	1	76190036	76253260	ACADM
ENSG00000117069	0.869158192	0.697730409	protein_coding	1	77333126	77531396	ST6GALNAC5
ENSG00000117114	0.499066092	1.353254395	protein_coding	1	81771845	82458107	LPHN2
ENSG00000117115	5.785082014	4.508954154	protein_coding	1	17393256	17445948	PADI2
ENSG00000117118	9.448421292	9.66482659	protein_coding	1	17345217	17380665	SDHB
ENSG00000117122	5.090170488	3.813624741	protein_coding	1	17300997	17307330	MFAP2
ENSG00000117133	7.881135284	8.234057939	protein_coding	1	84944942	84963473	RPF1
ENSG00000117139	9.441577455	9.650384052	protein_coding	1	202696526	202778598	KDM5B
ENSG00000117143	10.71160242	10.85602211	protein_coding	1	162531321	162569627	UAP1
ENSG00000117148	3.603959378	6.136428444	protein_coding	1	18081808	18153558	ACTL8
ENSG00000117151	6.509364082	6.525377513	protein_coding	1	85018804	85040163	CTBS
ENSG00000117152	3.502389126	3.183544561	protein_coding	1	163038565	163046592	RGS4
ENSG00000117153	8.333361768	8.334763756	protein_coding	1	202860228	202897764	KLHL12
ENSG00000117155	7.481543613	7.648510984	protein_coding	1	85109390	85156486	SSX2IP
ENSG00000117174	8.219950371	8.197845922	protein_coding	1	86115106	86174116	ZNHIT6
ENSG00000117222	7.950269163	7.582164879	protein_coding	1	205055270	205091143	RBBP5
ENSG00000117226	7.416718478	7.58917149	protein_coding	1	89472349	89488577	GBP3
ENSG00000117228	8.978417134	9.875786748	protein_coding	1	89518002	89531043	GBP1
ENSG00000117242	6.121171528	5.974624386	processed_transc	1	20969150	20978686	PINK1-AS1
ENSG00000117245	4.230497448	4.489113623	protein_coding	1	20990507	21044510	KIF17
ENSG00000117262	5.191549282	5.526427614	protein_coding	1	145764411	145827103	GPR89A
ENSG00000117266	8.280649185	7.928139131	protein_coding	1	205473723	205501921	CDK18
ENSG00000117280	8.092494681	7.982367515	protein_coding	1	205737114	205744588	RAB7L1
ENSG00000117281	3.830412367	3.570966319	protein_coding	1	145695798	145715614	CD160
ENSG00000117289	11.24990009	11.91550818	protein_coding	1	145438469	145442635	TXNIP
ENSG00000117298	11.9539871	12.12657065	protein_coding	1	21543740	21671997	ECE1
ENSG00000117305	7.578314254	7.994732806	protein_coding	1	24128375	24165110	HMGCL
ENSG00000117308	8.787867896	9.040423325	protein_coding	1	24122089	24127271	GALE
ENSG00000117318	9.226515195	9.884831244	protein_coding	1	23884409	23886285	ID3
ENSG00000117335	10.42977299	10.16592567	protein_coding	1	207925402	207968858	CD46
ENSG00000117360	9.445858656	9.741251102	protein_coding	1	150293925	150325671	PRPF3
ENSG00000117362	11.05208383	11.01665963	protein_coding	1	150237804	150241980	APH1A
ENSG00000117385	9.46791839	10.0752671	protein_coding	1	43212006	43232755	LEPRE1
ENSG00000117394	11.07022268	11.5307423	protein_coding	1	43391519	43424539	SLC2A1
ENSG00000117395	10.51455271	11.06938831	protein_coding	1	43629846	43727589	EBNA1BP2
ENSG00000117399	11.12811304	10.94154414	protein_coding	1	43824599	43828874	CDC20
ENSG00000117400	1.407729925	1.925536307	protein_coding	1	43803475	43818443	MPL
ENSG00000117407	7.161632221	6.677762549	protein_coding	1	44398992	44402913	ARTN
ENSG00000117408	9.207478296	9.426149618	protein_coding	1	44412611	44433694	IPO13
ENSG00000117410	8.860220382	9.124010896	protein_coding	1	44440159	44443967	ATP6V0B
ENSG00000117411	9.930083245	9.893819392	protein_coding	1	44444615	44456840	B4GALT2
ENSG00000117419	9.255095676	9.546823838	protein_coding	1	44686742	44820932	ERI3
ENSG00000117425	3.502389126	3.570966319	protein_coding	1	45285516	45308735	PTCH2
ENSG00000117448	9.205459721	9.317839931	protein_coding	1	46016215	46035721	AKR1A1
ENSG00000117450	11.70296837	11.92465406	protein_coding	1	45976708	45988719	PRDX1

ENSG00000117461	8.353554553	7.73528765	protein_coding	1	46505812	46642160	PIK3R3
ENSG00000117472	6.0776925	3.232099092	protein_coding	1	46640745	46651630	TSPAN1
ENSG00000117475	7.406211319	7.696882678	protein_coding	1	169337208	169365778	BLZF1
ENSG00000117477	1.407729925	0.950786998	protein_coding	1	169364108	169429907	C1orf114
ENSG00000117479	7.186405829	7.359856617	protein_coding	1	169433147	169455241	SLC19A2
ENSG00000117480	6.428602508	6.365895578	protein_coding	1	46859937	46879520	FAAH
ENSG00000117481	8.490822808	8.571336379	protein_coding	1	46805849	46830824	NSUN4
ENSG00000117500	9.563214301	9.565299431	protein_coding	1	93615299	93646285	TMED5
ENSG00000117505	9.079967361	9.148724524	protein_coding	1	93811445	93828149	DR1
ENSG00000117507	0	0.390640832	protein_coding	1	171106879	171130707	FMO6P
ENSG00000117519	10.18182545	10.35249425	protein_coding	1	95362507	95392834	CNN3
ENSG00000117523	11.17837695	11.21294852	protein_coding	1	171454651	171562650	PRRC2C
ENSG00000117525	10.33370433	9.839030856	protein_coding	1	94994781	95007356	F3
ENSG00000117528	8.655134734	8.993663261	protein_coding	1	94883933	94984222	ABCD3
ENSG00000117533	6.421663777	6.725179132	protein_coding	1	171669300	171711387	VAMP4
ENSG00000117543	7.319279953	7.825075067	protein_coding	1	101455179	101491644	DPH5
ENSG00000117569	7.348842929	7.210344614	protein_coding	1	97187221	97280349	PTBP2
ENSG00000117586	2.238690726	3.133298822	protein_coding	1	173152873	173176452	TNFSF4
ENSG00000117592	10.97804511	11.18527956	protein_coding	1	173446405	173457946	PRDX6
ENSG00000117593	9.503677432	9.493672366	protein_coding	1	173793641	173827684	DARS2
ENSG00000117594	0.869158192	1.518964905	protein_coding	1	209859510	209908295	HSD11B1
ENSG00000117595	8.741255403	4.385599336	protein_coding	1	209959036	209979465	IRF6
ENSG00000117597	8.14188337	8.520929986	protein_coding	1	210001352	210025558	DIEXF
ENSG00000117602	6.35766608	6.104183162	protein_coding	1	24829387	24863506	RCAN3
ENSG00000117614	7.971805197	8.293892101	protein_coding	1	25549170	25558993	SYF2
ENSG00000117616	7.751556534	8.593573257	protein_coding	1	25568728	25664704	C1orf63
ENSG00000117620	7.95748362	7.852510423	protein_coding	1	100435345	100492535	SLC35A3
ENSG00000117625	7.695130106	7.924450389	protein_coding	1	211431719	211489727	RCOR3
ENSG00000117632	10.37514929	11.16295153	protein_coding	1	26210672	26233482	STMN1
ENSG00000117640	7.430610036	7.84081624	protein_coding	1	26145131	26159432	FAM54B
ENSG00000117643	2.57521082	3.679994897	protein_coding	1	25943959	26112698	MAN1C1
ENSG00000117650	7.759833327	7.797107849	protein_coding	1	211836114	211848960	NEK2
ENSG00000117676	9.675388335	9.003312166	protein_coding	1	26856252	26901521	RPS6KA1
ENSG00000117682	8.994872754	8.69448524	protein_coding	1	26758773	26797785	DHDDS
ENSG00000117691	8.475827914	9.066597822	protein_coding	1	212606229	212619714	NENF
ENSG00000117697	7.789781675	8.036341107	protein_coding	1	212899495	212965124	NSL1
ENSG00000117713	10.26316297	10.10815782	protein_coding	1	27022524	27108595	ARID1A
ENSG00000117724	11.21520883	11.4509377	protein_coding	1	214776538	214837931	CENPF
ENSG00000117748	8.605481314	9.127223641	protein_coding	1	28218035	28241257	RPA2
ENSG00000117751	8.455589162	8.836685385	protein_coding	1	28157289	28178187	PPP1R8
ENSG00000117758	7.985986001	8.34031144	protein_coding	1	28099694	28150963	STX12
ENSG00000117791	5.684325135	5.700439663	protein_coding	1	220921567	220958150	2-Mar
ENSG00000117834	0.499066092	0.697730409	protein_coding	1	48688357	48714316	SLC5A9
ENSG00000117859	9.569505979	9.603863718	protein_coding	1	52042851	52254889	OSBPL9
ENSG00000117862	8.967738537	9.543220426	protein_coding	1	52485803	52521843	TXNDC12
ENSG00000117868	10.54040657	10.13034291	protein_coding	7	158523686	158622944	ESYT2
ENSG00000117877	5.774228067	5.175534283	protein_coding	19	45909467	45914024	CD3EAP
ENSG00000117899	8.918065711	9.08571528	protein_coding	15	81239667	81282219	MESDC2
ENSG00000117906	8.687312327	9.506682457	protein_coding	15	77223960	77242601	RCN2
ENSG00000117971	2.671945279	1.667587519	protein_coding	15	78916461	79012628	CHRNB4
ENSG00000117983	6.522395495	4.489113623	protein_coding	11	1244296	1284402	MUC5B
ENSG00000117984	12.68203286	12.31779513	protein_coding	11	1773982	1785222	CTSD
ENSG00000118004	2.847891871	2.788380093	protein_coding	2	3642426	3692048	COLEC11
ENSG00000118007	7.943018447	7.86795666	protein_coding	3	136055077	136471220	STAG1
ENSG00000118017	0.869158192	0.950786998	protein_coding	3	137842560	137851229	A4GNT
ENSG00000118046	10.35662351	10.16553509	protein_coding	19	1205798	1228434	STK11
ENSG00000118058	10.3656902	10.43744755	protein_coding	11	118307205	118397539	MLL
ENSG00000118094	1.407729925	1.518964905	protein_coding	11	118528026	118550399	TREH
ENSG00000118096	6.414691513	7.522414019	protein_coding	11	118415243	118443685	IFT46
ENSG00000118137	0	0.390640832	protein_coding	11	116706467	116708666	APOA1
ENSG00000118156	4.77879189	6.475832214	protein_coding	19	48023942	48059113	ZNF541
ENSG00000118160	3.448790144	2.851901313	protein_coding	19	47931279	47975307	SLC8A2
ENSG00000118162	7.084651883	7.354383726	protein_coding	19	47978400	47987521	KPTN

ENSG00000118181	10.80820951	11.53043899	protein_coding	11	118886422	118889401	RPS25
ENSG00000118193	8.549290825	8.323603978	protein_coding	1	200520628	200589862	KIF14
ENSG00000118197	6.970425921	7.20731193	protein_coding	1	200593024	200639126	DDX59
ENSG00000118200	9.303080552	9.247247272	protein_coding	1	200708686	200829832	CAMSAP2
ENSG00000118217	9.819610198	10.38405976	protein_coding	1	161736084	161933860	ATF6
ENSG00000118242	6.502803968	6.246885341	protein_coding	2	216807557	216898819	MREG
ENSG00000118246	8.941297632	8.876367148	protein_coding	2	207630081	207657233	FASTKD2
ENSG00000118257	8.312882337	5.776071827	protein_coding	2	206546562	206662857	NRP2
ENSG00000118260	8.45218826	8.471229048	protein_coding	2	208394461	208468155	CREB1
ENSG00000118263	8.893200037	8.69123279	protein_coding	2	207938861	208031991	KLF7
ENSG00000118276	7.289098491	7.603083446	protein_coding	18	29202209	29264686	B4GALT6
ENSG00000118292	2.238690726	2.788380093	protein_coding	1	150240600	150253327	C1orf54
ENSG00000118298	3.077135474	2.420525079	protein_coding	1	150230169	150237478	CA14
ENSG00000118307	1.16343121	1.518964905	protein_coding	12	25261354	25348096	CASC1
ENSG00000118308	1.16343121	0.950786998	protein_coding	12	25173936	25261268	LRMP
ENSG00000118322	2.35979773	2.420525079	protein_coding	5	159990127	160279221	ATP10B
ENSG00000118363	7.246540262	7.544210495	protein_coding	11	74660292	74690076	SPCS2
ENSG00000118369	6.798966424	6.819555981	protein_coding	11	77899858	77925757	USP35
ENSG00000118407	0	1.353254395	protein_coding	6	76005626	76203496	FILIP1
ENSG00000118412	7.91858278	8.293892101	processed_transc	6	90539613	90584155	CASP8AP2
ENSG00000118418	7.985986001	7.951888765	protein_coding	6	79910962	79944406	HMGN3
ENSG00000118420	5.301468762	5.656642807	protein_coding	6	83602117	83775560	UBE2CBP
ENSG00000118454	7.748787017	7.774746834	protein_coding	1	70726271	70820417	ANKRD13C
ENSG00000118473	0	0.390640832	protein_coding	1	66999066	67213982	SGIP1
ENSG00000118482	9.342189974	9.6159211	protein_coding	6	64345725	64489229	PHF3
ENSG00000118491	0	0.697730409	protein_coding	6	144185573	144259483	C6orf94
ENSG00000118495	0	1.353254395	protein_coding	6	144261437	144385735	PLAGL1
ENSG00000118496	8.002356002	7.780879746	protein_coding	6	146119271	146135889	FBXO30
ENSG00000118503	10.27620418	10.48955424	protein_coding	6	138188351	138204449	TNFAIP3
ENSG00000118507	5.270908378	5.404622322	protein_coding	6	131456806	131604675	AKAP7
ENSG00000118508	9.399818301	10.01670633	protein_coding	6	146864829	146876101	RAB32
ENSG00000118513	4.062237333	4.021284656	protein_coding	6	135502453	135540311	MYB
ENSG00000118514	0	1.16600992	protein_coding	6	135238528	135271260	ALDH8A1
ENSG00000118515	10.31837574	9.870045082	protein_coding	6	134490384	134639250	SGK1
ENSG00000118518	7.266040677	7.848622884	protein_coding	6	127587755	127609712	RNF146
ENSG00000118520	0.869158192	0.697730409	protein_coding	6	131894284	131905472	ARG1
ENSG00000118523	11.10203752	10.36717494	protein_coding	6	132269316	132272513	CTGF
ENSG00000118526	0	0.390640832	protein_coding	6	134210276	134216691	TCF21
ENSG00000118557	3.911897206	4.42790041	protein_coding	16	72146056	72210777	PMFBP1
ENSG00000118564	8.650690744	9.032734472	protein_coding	4	15606162	15683302	FBXL5
ENSG00000118579	7.913645586	7.929979971	protein_coding	4	17616254	17627477	MED28
ENSG00000118596	4.381167248	7.029859489	protein_coding	12	59989848	60176395	SLC16A7
ENSG00000118600	7.654275977	8.101495266	protein_coding	12	64173583	64203338	TMEM5
ENSG00000118620	7.413224588	6.952279523	protein_coding	19	21203426	21242852	ZNF430
ENSG00000118640	8.398422578	8.517261232	protein_coding	2	85788685	85809154	VAMP8
ENSG00000118655	7.886184801	8.0397549	protein_coding	1	114447763	114456708	DCLRE1B
ENSG00000118680	10.35616868	10.62242012	protein_coding	18	3261907	3278282	MYL12B
ENSG00000118689	9.807018378	9.630144961	protein_coding	6	108881038	109005977	FOXO3
ENSG00000118690	4.165528823	4.810179682	protein_coding	6	109169619	109295186	ARMC2
ENSG00000118705	12.10184497	12.86190128	protein_coding	20	35806813	35870022	RPN2
ENSG00000118707	9.44329146	9.469827862	protein_coding	20	35201891	35222560	TGIF2
ENSG00000118762	7.692250012	8.349968848	protein_coding	4	88928820	88998929	PKD2
ENSG00000118777	7.115078652	6.563824016	protein_coding	4	89011416	89152474	ABCG2
ENSG00000118785	0	1.16600992	protein_coding	4	88896802	88904562	SPP1
ENSG00000118804	3.603959378	4.385599336	protein_coding	4	77172886	77232752	STBD1
ENSG00000118816	11.3060841	11.0698058	protein_coding	4	77968311	77997158	CCNI
ENSG00000118849	4.800350936	4.489113623	protein_coding	3	158414681	158450485	RARRES1
ENSG00000118855	9.086562954	9.712663243	protein_coding	3	158449987	158547508	MFSD1
ENSG00000118873	9.164483521	9.479287299	protein_coding	1	220321635	220445796	RAB3GAP2
ENSG00000118894	7.67484765	7.465168131	protein_coding	16	5134117	5147809	FAM86A
ENSG00000118898	8.006999229	5.35066967	protein_coding	16	4932508	5010742	PPL
ENSG00000118900	9.836012692	9.700851959	protein_coding	16	4896666	4932361	UBN1
ENSG00000118922	6.912209543	6.293387477	protein_coding	13	74260226	74708394	KLF12

ENSG00000118939	5.485736881	5.456629825	protein_coding	13	76123619	76180085	UCHL3
ENSG00000118960	8.241782151	7.733180651	protein_coding	2	20760208	20850849	HS1BP3
ENSG00000118961	6.820228284	6.310446475	protein_coding	2	20883803	21022882	C2orf43
ENSG00000118965	8.650690744	8.270836518	protein_coding	2	20110021	20189892	WDR35
ENSG00000118971	0.869158192	0	protein_coding	12	4382938	4414516	CCND2
ENSG00000118976	0	0.390640832	pseudogene	12	3948625	3951691	
ENSG00000118985	9.3522601	8.948064416	protein_coding	5	95220802	95297775	ELL2
ENSG00000118997	1.407729925	1.802319292	protein_coding	2	196602427	196935730	DNAH7
ENSG00000119004	6.528867325	6.908136232	protein_coding	2	204103663	204163009	CYP20A1
ENSG00000119013	7.695130106	7.939149102	protein_coding	2	201936156	201950473	NDUFB3
ENSG00000119041	8.760631512	8.467431533	protein_coding	2	197627756	197664449	GTF3C3
ENSG00000119042	8.023133844	7.953699568	protein_coding	2	200134223	200335989	SATB2
ENSG00000119048	8.227927477	8.320800493	protein_coding	5	133706870	133727683	UBE2B
ENSG00000119121	1.799000381	2.144285137	protein_coding	9	77337411	77503010	TRPM6
ENSG00000119138	7.178195136	6.582669759	protein_coding	9	72999503	73029540	KLF9
ENSG00000119139	11.00527807	11.11844254	protein_coding	9	71736224	71870124	TJP2
ENSG00000119185	8.904442656	8.846464976	protein_coding	2	9543604	9563676	ITGB1BP1
ENSG00000119203	9.441577455	8.975061478	protein_coding	2	9563697	9613230	CPSF3
ENSG00000119227	5.958104008	6.037446454	protein_coding	3	196673214	196695931	PIGZ
ENSG00000119231	8.861502869	8.69448524	protein_coding	3	196594727	196661585	SENP5
ENSG00000119242	8.215945216	8.685795691	protein_coding	12	124403207	124457378	CCDC92
ENSG00000119280	8.687312327	8.245929405	protein_coding	1	230972865	231005335	C1orf198
ENSG00000119283	0	0.697730409	protein_coding	1	231297858	231357302	TRIM67
ENSG00000119285	10.16841654	10.28181691	protein_coding	1	236712305	236767804	HEATR1
ENSG00000119314	11.25527652	10.46786018	protein_coding	9	114980715	115095947	PTBP3
ENSG00000119318	11.73007644	11.37948398	protein_coding	9	110045418	110094475	RAD23B
ENSG00000119321	9.410371954	9.682951719	protein_coding	9	115923286	115983641	FKBP15
ENSG00000119326	10.67268056	10.70724858	protein_coding	9	111704851	111775809	CTNNAL1
ENSG00000119328	7.832262274	7.500283181	protein_coding	9	111696461	111713024	FAM206A
ENSG00000119333	10.90560604	10.80176251	protein_coding	9	131395940	131419066	WDR34
ENSG00000119335	12.53807668	12.2859871	protein_coding	9	131445703	131458679	SET
ENSG00000119383	11.69237733	11.53920901	protein_coding	9	131873229	131911225	PPP2R4
ENSG00000119392	10.26024885	10.276401	protein_coding	9	131266979	131304567	GLE1
ENSG00000119396	10.21681795	10.01019541	protein_coding	9	123940415	123985292	RAB14
ENSG00000119397	8.243750574	7.760334499	protein_coding	9	123837141	123939888	CNTRL
ENSG00000119401	8.638772743	8.553535588	protein_coding	9	119449581	119463579	TRIM32
ENSG00000119402	8.782461599	8.732953241	protein_coding	9	123514256	123555690	FBXW2
ENSG00000119403	9.506959233	9.431357402	protein_coding	9	123617976	123639606	PHF19
ENSG00000119408	9.959126666	9.657067809	protein_coding	9	127019885	127115586	NEK6
ENSG00000119411	3.335220907	1.353254395	protein_coding	9	116111821	116133513	BSPRY
ENSG00000119414	9.788259363	9.499882282	protein_coding	9	127908852	127952218	PPP6C
ENSG00000119421	8.819887015	9.250933796	protein_coding	9	124894745	124922098	NDUFA8
ENSG00000119431	7.89873181	7.984140487	protein_coding	9	116135699	116139279	HDHD3
ENSG00000119446	8.12703021	8.122575449	protein_coding	9	125001834	125027118	RBM18
ENSG00000119457	1.960915222	2.721932731	protein_coding	9	115641200	115653193	SLC46A2
ENSG00000119471	9.557686483	9.100502039	protein_coding	9	115142217	115234690	HSDL2
ENSG00000119487	10.55395527	10.70429374	protein_coding	9	128199672	128469513	MAPKAP1
ENSG00000119508	3.652182994	2.420525079	protein_coding	9	102584137	102629173	NR4A3
ENSG00000119509	8.148202495	8.315177126	protein_coding	9	102861538	103063282	INVS
ENSG00000119514	6.220467583	3.368666104	protein_coding	9	101569981	101612363	GALNT12
ENSG00000119522	9.581229429	9.320649169	protein_coding	9	126141933	126692431	DENND1A
ENSG00000119523	8.962967004	9.256079154	protein_coding	9	101978708	101984238	ALG2
ENSG00000119537	9.197357059	9.11108789	protein_coding	18	60994971	61034743	KDSR
ENSG00000119541	8.462367008	8.310945169	protein_coding	18	61056423	61089693	VPS4B
ENSG00000119547	7.026383958	6.815578497	protein_coding	18	55102917	55158530	ONECUT2
ENSG00000119559	8.518728184	8.872543451	protein_coding	19	1473203	1479228	C19orf25
ENSG00000119574	8.243750574	8.278081046	protein_coding	19	59024899	59030921	ZBTB45
ENSG00000119596	10.40183845	10.18647659	protein_coding	14	75230069	75304012	YLPM1
ENSG00000119599	9.27736711	9.079094423	protein_coding	14	73393040	73426411	DCAF4
ENSG00000119608	2.928422289	4.202780776	protein_coding	14	75319736	75330537	PROX2
ENSG00000119616	9.247267789	9.328346488	protein_coding	14	75179847	75203394	FCF1
ENSG00000119630	6.528867325	6.475832214	protein_coding	14	75408537	75422487	PGF
ENSG00000119632	7.04456449	7.0637896	protein_coding	14	94594116	94596590	IFI27L2

ENSG00000119636	4.131913373	4.528525526	protein_coding	14	74486056	74549566	C14orf45
ENSG00000119638	10.39166588	10.43777104	protein_coding	14	75548822	75594047	NEK9
ENSG00000119640	6.616550626	7.029859489	protein_coding	14	75519924	75536186	ACYP1
ENSG00000119650	7.222786417	7.683850422	protein_coding	14	76368479	76550928	IFT43
ENSG00000119655	9.823563838	10.17914289	protein_coding	14	74942895	74960880	NPC2
ENSG00000119660	0	0.390640832	pseudogene	14	75758809	75761016	
ENSG00000119661	6.095241663	6.198834244	protein_coding	14	74111578	74165604	DNAL1
ENSG00000119669	10.41500014	10.00714689	protein_coding	14	77490888	77495034	IRF2BPL
ENSG00000119673	7.395627075	8.109639521	protein_coding	14	74034324	74042357	ACOT2
ENSG00000119681	8.718787866	8.639296529	protein_coding	14	74964873	75079306	LTBP2
ENSG00000119682	9.686285316	9.901345166	protein_coding	14	75119880	75179818	KIAA0317
ENSG00000119684	7.55947245	7.892709342	protein_coding	14	75480467	75518235	MLH3
ENSG00000119685	8.950968668	8.747640323	protein_coding	14	76099968	76421421	TTLL5
ENSG00000119686	4.19837882	5.004391715	protein_coding	14	76044960	76129557	
ENSG00000119688	8.572970055	9.067434305	protein_coding	14	74752126	74769759	ABCD4
ENSG00000119689	10.33969843	10.54495849	protein_coding	14	75348594	75370448	DLST
ENSG00000119698	8.344411036	7.741590246	protein_coding	14	94612465	94746072	PPP4R4
ENSG00000119699	6.407685389	7.36802722	protein_coding	14	76424442	76449334	TGFB3
ENSG00000119703	3.448790144	5.47691899	protein_coding	14	75530873	75545126	FAM164C
ENSG00000119705	9.404225042	10.20293751	protein_coding	14	78174414	78227447	SLIRP
ENSG00000119707	10.84724869	11.33032591	protein_coding	14	73525144	73588122	RBM25
ENSG00000119711	7.409722215	7.714077892	protein_coding	14	74523553	74551196	ALDH6A1
ENSG00000119714	5.018397273	4.640685763	protein_coding	14	91698876	91720269	GPR68
ENSG00000119715	2.471521042	1.16600992	protein_coding	14	76776957	76968178	ESRRB
ENSG00000119718	8.445362311	8.874456566	protein_coding	14	75469614	75476292	EIF2B2
ENSG00000119720	8.359012976	8.034631177	protein_coding	14	90742580	90798481	C14orf102
ENSG00000119723	6.496213887	7.022976646	protein_coding	14	74416629	74430373	COQ6
ENSG00000119725	3.211941663	3.64455972	protein_coding	14	74353320	74399214	ZNF410
ENSG00000119729	8.109865782	8.448292555	protein_coding	2	46768945	46810260	RHOQ
ENSG00000119737	4.165528823	4.547834947	protein_coding	2	54080050	54087126	GPR75
ENSG00000119760	8.875535495	9.209096633	protein_coding	2	27873679	27886676	SUPT7L
ENSG00000119771	8.687312327	8.058387721	protein_coding	2	23608088	23931481	KLHL29
ENSG00000119772	7.292905885	6.615068802	protein_coding	2	25455845	25565459	DNMT3A
ENSG00000119777	10.72575555	10.84874214	protein_coding	2	27255778	27264563	TMEM214
ENSG00000119778	6.9512803	6.712401119	protein_coding	2	23971534	24149984	ATAD2B
ENSG00000119782	2.35979773	2.144285137	protein_coding	2	24272571	24286551	FKBP1B
ENSG00000119787	9.154055005	9.25681271	protein_coding	2	38522022	38604427	ATL2
ENSG00000119801	8.764749894	9.423538659	protein_coding	2	30369807	30383399	YPEL5
ENSG00000119812	9.843171129	9.94569357	protein_coding	2	33808725	33824449	FAM98A
ENSG00000119820	8.759256101	9.048918708	protein_coding	2	32502979	32537002	YIPF4
ENSG00000119844	8.203862602	8.259169069	protein_coding	2	64751465	64820139	AFTPH
ENSG00000119862	6.509364082	5.988823159	protein_coding	2	64681103	64688515	LGALSL
ENSG00000119865	0	0.390640832	protein_coding	2	68511303	68547183	CNRIP1
ENSG00000119866	0.499066092	0	protein_coding	2	60678302	60780702	BCL11A
ENSG00000119878	7.292905885	7.572769481	protein_coding	2	46843555	46852881	CRIPT
ENSG00000119888	8.295907473	3.493416095	protein_coding	2	47572297	47614740	EPCAM
ENSG00000119899	7.190493718	7.434373903	protein_coding	6	74303102	74363878	SLC17A5
ENSG00000119900	7.285281023	7.56568256	protein_coding	6	71998506	72011973	OGFRL1
ENSG00000119906	9.068907253	9.566483319	protein_coding	10	102672720	102724893	FAM178A
ENSG00000119912	9.652603664	9.911163541	protein_coding	10	94211441	94333833	IDE
ENSG00000119915	4.642199401	5.018301917	protein_coding	10	103986085	103989346	ELOVL3
ENSG00000119917	9.441577455	10.40968193	protein_coding	10	91087651	91100728	IFIT3
ENSG00000119922	8.862784218	9.581200526	protein_coding	10	91061712	91069033	IFIT2
ENSG00000119927	7.842689815	8.235547225	protein_coding	10	113909624	113975135	GPAM
ENSG00000119929	8.146099194	8.750768196	protein_coding	10	101462315	101515891	CUTC
ENSG00000119938	6.25990527	7.100213554	protein_coding	10	93388199	93392828	PPP1R3C
ENSG00000119943	5.827699195	6.264499759	protein_coding	10	100143322	100174941	PYROXD2
ENSG00000119950	8.626755466	9.045526555	protein_coding	10	111967363	112047123	MXI1
ENSG00000119953	8.684416567	8.784734524	protein_coding	10	112052798	112064709	SMNDC1
ENSG00000119965	6.681890601	6.881748114	protein_coding	10	124690419	124713919	C10orf88
ENSG00000119969	9.328343713	9.402479116	protein_coding	10	96305547	96373662	HELLS
ENSG00000119973	0	0.697730409	protein_coding	10	120352916	120355160	PRLHR
ENSG00000119977	9.218530254	9.414362912	protein_coding	10	97423153	97454712	TCTN3

ENSG00000119979	7.778963161	8.127396674	protein_coding	10	120863598	120897496	FAM45A
ENSG00000119986	6.721980839	6.577981353	protein_coding	10	99437181	99447080	AVPI1
ENSG00000120008	9.154055005	9.691658363	protein_coding	10	122610695	122669036	WDR11
ENSG00000120029	8.282565319	8.758040301	protein_coding	10	103605356	103815950	C10orf76
ENSG00000120049	3.146112541	4.448594745	protein_coding	10	103585731	103603677	KCNIP2
ENSG00000120051	4.491309013	5.045726316	protein_coding	10	106113522	106214848	CCDC147
ENSG00000120053	9.194306789	9.221925935	protein_coding	10	101156627	101190393	GOT1
ENSG00000120055	6.872051157	6.637773454	protein_coding	10	104209574	104216054	C10orf95
ENSG00000120057	0.869158192	1.518964905	protein_coding	10	99526508	99531709	SFRP5
ENSG00000120063	9.98347984	10.10978283	protein_coding	17	63005407	63052920	GNA13
ENSG00000120068	0.869158192	0.390640832	protein_coding	17	46688446	46692653	HOXB8
ENSG00000120071	8.650690744	8.42106413	protein_coding	17	44107334	44270166	KIAA1267
ENSG00000120075	0.499066092	0	protein_coding	17	46668619	46671323	HOXB5
ENSG00000120087	0	0.390640832	protein_coding	17	46684590	46710934	HOXB7
ENSG00000120088	0	0.697730409	protein_coding	17	43699267	43913193	CRHR1
ENSG00000120093	1.16343121	1.925536307	protein_coding	17	46626232	46667634	HOXB3
ENSG00000120129	10.77830572	8.724493	protein_coding	5	172195093	172198198	DUSP1
ENSG00000120137	9.228504544	9.188483253	protein_coding	5	167975500	168006605	PANK3
ENSG00000120149	5.649112675	5.339632724	protein_coding	5	174151536	174158144	MSX2
ENSG00000120156	0	0.950786998	protein_coding	9	27109139	27230173	TEK
ENSG00000120158	6.442380648	6.064512706	protein_coding	9	4792869	4861064	RCL1
ENSG00000120159	6.670228606	6.984522608	protein_coding	9	26840683	26892802	C9orf82
ENSG00000120162	2.928422289	2.334191469	protein_coding	9	27325207	27529779	MOB3B
ENSG00000120215	1.960915222	1.353254395	protein_coding	9	5890802	5910606	MLANA
ENSG00000120217	7.0218028	7.315480906	protein_coding	9	5450503	5470566	CD274
ENSG00000120251	0	0.390640832	protein_coding	4	158125334	158287227	GRIA2
ENSG00000120253	8.999540089	9.029304007	protein_coding	6	150045451	150070801	NUP43
ENSG00000120254	9.648151859	9.487435606	protein_coding	6	151186685	151423023	MTHFD1L
ENSG00000120256	9.326487463	9.023280994	protein_coding	6	150139934	150186199	LRP11
ENSG00000120262	1.407729925	0.390640832	protein_coding	6	151815165	151942328	C6orf97
ENSG00000120265	9.524876896	9.637486317	protein_coding	6	150070579	150132556	PCMT1
ENSG00000120278	5.072559152	3.411459265	protein_coding	6	150920999	151164799	PLEKHG1
ENSG00000120279	0	0.390640832	protein_coding	6	153019030	153045702	MYCT1
ENSG00000120306	7.72925009	7.389591938	protein_coding	5	139554227	139661637	C5orf32
ENSG00000120314	8.779750834	8.296748314	protein_coding	5	140044261	140053709	WDR55
ENSG00000120318	8.15239992	7.809160246	protein_coding	5	141013251	141061788	ARAP3
ENSG00000120322	6.469548347	6.619638408	protein_coding	5	140557371	140560081	PCDHB8
ENSG00000120324	4.19837882	4.710890357	protein_coding	5	140571942	140575215	PCDHB10
ENSG00000120327	6.489593565	5.953060722	protein_coding	5	140602931	140605858	PCDHB14
ENSG00000120328	3.871730003	3.608232228	protein_coding	5	140588269	140591696	PCDHB12
ENSG00000120329	1.16343121	0.390640832	protein_coding	5	140682196	140683612	SLC25A2
ENSG00000120333	7.246540262	7.641787375	protein_coding	1	174979925	174992561	MRPS14
ENSG00000120334	6.96088487	6.988061092	protein_coding	1	173768688	173793858	CENPL
ENSG00000120337	3.830412367	5.150490275	protein_coding	1	173009100	173020103	TNFSF18
ENSG00000120370	5.889353623	6.934050347	protein_coding	1	170501270	170522974	GORAB
ENSG00000120436	0	0.390640832	protein_coding	6	167569759	167571817	GPR31
ENSG00000120437	8.154494062	8.151264044	protein_coding	6	160181360	160200144	ACAT2
ENSG00000120438	11.30160288	11.15057154	protein_coding	6	160199530	160210781	TCP1
ENSG00000120440	1.960915222	3.232099092	protein_coding	6	167738574	167772991	TTLL2
ENSG00000120451	9.859951197	9.843432726	protein_coding	11	130745331	130786404	SNX19
ENSG00000120457	4.568725997	3.081239798	protein_coding	11	128760140	128790930	KCNJ5
ENSG00000120458	7.689364157	8.041458772	protein_coding	11	124636394	124670569	C11orf61
ENSG00000120471	1.407729925	1.802319292	protein_coding	11	128804626	128813040	TP53AIP1
ENSG00000120498	0	0.390640832	protein_coding	X	69748790	70128581	TEX11
ENSG00000120500	3.146112541	3.279072565	protein_coding	X	69488155	69501690	ARR3
ENSG00000120509	8.120617457	8.520929986	protein_coding	X	69506445	69510364	PDZD11
ENSG00000120519	6.585843317	6.246885341	protein_coding	4	147175127	147443123	SLC10A7
ENSG00000120526	8.135536446	8.379906558	protein_coding	8	110253148	110346614	NUDCD1
ENSG00000120533	7.800519667	8.673761454	protein_coding	8	110346553	110358182	ENY2
ENSG00000120539	8.591637531	9.712663243	protein_coding	10	27443753	27475848	MASTL
ENSG00000120549	10.27572327	9.160532436	protein_coding	10	23983675	24836772	KIAA1217
ENSG00000120555	2.928422289	3.232099092	protein_coding	10	38671951	38691844	SEPT7L
ENSG00000120594	0.499066092	0.390640832	protein_coding	10	20105168	20569286	PLXDC2

ENSG00000120616	7.281453427	8.780659733	protein_coding	10	32556679	32667726	EPC1
ENSG00000120645	3.004694206	0.390640832	protein_coding	12	175931	287626	IQSEC3
ENSG00000120647	7.654275977	7.892709342	protein_coding	12	498439	551811	CCDC77
ENSG00000120656	7.234712227	7.470237199	protein_coding	1	28915835	28969597	TAF12
ENSG00000120658	0	0.697730409	protein_coding	13	43787654	44361044	ENOX1
ENSG00000120662	6.204385613	5.683080141	protein_coding	13	41790825	41837742	MTRF1
ENSG00000120664	1.16343121	1.353254395	protein_coding	13	36920568	36943738	SPG20OS
ENSG00000120675	1.407729925	1.518964905	protein_coding	13	43597339	43683045	DNAJC15
ENSG00000120685	9.476313889	8.605140645	protein_coding	13	39584002	39612252	PROSER1
ENSG00000120686	9.009986712	8.475016594	protein_coding	13	38923986	38937140	UFM1
ENSG00000120688	6.820228284	6.495854971	protein_coding	13	41635410	41658137	WBP4
ENSG00000120690	8.536501169	8.117738058	protein_coding	13	41506056	41635576	ELF1
ENSG00000120693	3.603959378	2.971122874	protein_coding	13	37418968	37494902	SMAD9
ENSG00000120694	10.12475765	9.70408282	protein_coding	13	31710762	31736525	HSPH1
ENSG00000120696	6.252103507	5.700439663	protein_coding	13	41763969	41768702	KBTBD7
ENSG00000120697	6.435508026	6.592001111	protein_coding	13	37523912	37574398	ALG5
ENSG00000120699	7.615275323	7.637287497	protein_coding	13	37572953	37583750	EXOSC8
ENSG00000120705	10.45462978	10.27277906	protein_coding	5	137841784	137878989	ETF1
ENSG00000120708	12.73547073	13.63261147	protein_coding	5	135364584	135399507	TGFBI
ENSG00000120709	9.479658457	9.411071665	protein_coding	5	137667624	137685416	FAM53C
ENSG00000120725	8.755121984	9.157393097	protein_coding	5	138282409	138629246	SIL1
ENSG00000120727	8.241782151	8.129000175	protein_coding	5	138677276	138705406	PAIP2
ENSG00000120729	2.57521082	2.501982735	protein_coding	5	137203480	137223540	MYOT
ENSG00000120733	10.11833477	9.735999615	protein_coding	5	137688285	137772717	KDM3B
ENSG00000120738	12.83258876	6.252780742	protein_coding	5	137801169	137805004	EGR1
ENSG00000120742	10.56777082	10.55364094	protein_coding	3	150259781	150321015	SERP1
ENSG00000120756	8.455589162	7.948260326	protein_coding	3	142315229	142432506	PLS1
ENSG00000120784	6.535310252	6.787424764	protein_coding	19	38123389	38147162	ZFP30
ENSG00000120798	6.998676229	7.429177016	protein_coding	12	95415669	95467479	NR2C1
ENSG00000120800	9.187164314	8.911264037	protein_coding	12	101673887	101780394	UTP20
ENSG00000120802	10.23869682	10.5201215	protein_coding	12	98909290	98944157	TMPO
ENSG00000120805	8.628263112	8.897218229	protein_coding	12	101786898	101801598	ARL1
ENSG00000120820	3.335220907	3.64455972	protein_coding	12	104382762	104457961	GLT8D2
ENSG00000120832	5.977159882	6.2290532	protein_coding	12	107371069	107380944	MTERFD3
ENSG00000120833	6.335686705	5.555337277	protein_coding	12	93963590	93977263	SOCS2
ENSG00000120837	7.642387679	8.194786822	protein_coding	12	104510855	104532067	NFYB
ENSG00000120860	6.393570237	7.106738413	protein_coding	12	102406705	102455927	CCDC53
ENSG00000120868	7.341508784	7.026422172	protein_coding	12	99038919	99129204	APAF1
ENSG00000120875	9.119096064	8.768365845	protein_coding	8	29190581	29208185	DUSP4
ENSG00000120885	8.129161475	9.462851308	protein_coding	8	27454434	27472548	CLU
ENSG00000120889	10.77354482	10.83136512	protein_coding	8	22877646	22926692	TNFRSF10B
ENSG00000120896	9.816307202	9.684588223	protein_coding	8	22402499	22433301	SORBS3
ENSG00000120899	9.362260424	8.202422444	protein_coding	8	27168999	27316903	PTK2B
ENSG00000120903	0.869158192	0.390640832	protein_coding	8	27317279	27337400	CHRNA2
ENSG00000120910	7.593829938	8.401295742	protein_coding	8	22298332	22398652	PPP3CC
ENSG00000120913	5.588445295	6.071200665	protein_coding	8	22435792	22455538	PDLIM2
ENSG00000120915	5.191549282	5.22435463	protein_coding	8	27348296	27403081	EPHX2
ENSG00000120925	6.283060568	6.525377513	protein_coding	8	42704780	42752433	RNF170
ENSG00000120937	0.499066092	2.912743273	protein_coding	1	11917521	11918988	NPPB
ENSG00000120942	7.911170638	7.999999944	protein_coding	1	11333263	11356106	UBIAD1
ENSG00000120948	10.54719682	10.76613996	protein_coding	1	11072414	11085796	TARDBP
ENSG00000120949	2.35979773	1.802319292	protein_coding	1	12123434	12204264	TNFRSF8
ENSG00000120963	8.830404061	8.993663261	protein_coding	8	102190106	102218421	ZNF706
ENSG00000120992	9.559268022	9.535986486	protein_coding	8	54958938	55014577	LYPLA1
ENSG00000121022	8.944931872	8.81990641	protein_coding	8	67955314	67996018	COPS5
ENSG00000121039	7.444369108	7.083771121	protein_coding	8	74206847	74237516	RDH10
ENSG00000121053	0.869158192	2.039052734	protein_coding	17	56270089	56282534	EPX
ENSG00000121057	9.901066862	10.09098372	protein_coding	17	55162551	55198710	AKAP1
ENSG00000121058	7.870982917	8.163833676	protein_coding	17	55015563	55038411	COIL
ENSG00000121060	10.99157943	11.15293784	protein_coding	17	54965270	54991409	TRIM25
ENSG00000121064	8.337054268	9.040423325	protein_coding	17	55055468	55084127	SCPEP1
ENSG00000121067	8.583888749	8.754928172	protein_coding	17	47676246	47755596	SPOP
ENSG00000121068	0.499066092	0.390640832	protein_coding	17	59477257	59486827	TBX2

ENSG00000121073	8.810621286	8.793860943	protein_coding	17	47778305	47786376	SLC35B1
ENSG00000121075	1.616589159	0.950786998	protein_coding	17	59529779	59562469	TBX4
ENSG00000121083	9.340351466	8.886830358	protein_coding	17	56160780	56167618	DYNLL2
ENSG00000121089	5.660945913	6.24096575	pseudogene	4	165864442	165865089	NACA3P
ENSG00000121101	3.274897671	3.232099092	protein_coding	17	56634042	56769416	TEX14
ENSG00000121104	6.693459083	5.96747225	protein_coding	17	47787694	47866542	FAM117A
ENSG00000121152	9.047656547	8.89533508	protein_coding	2	97001525	97039583	NCAPH
ENSG00000121210	8.810621286	8.946246521	protein_coding	4	154387498	154557863	KIAA0922
ENSG00000121211	6.941611359	7.987679908	protein_coding	4	154265801	154336270	MND1
ENSG00000121236	7.420203928	7.195117037	protein_coding	11	5617339	5634188	TRIM6
ENSG00000121270	1.616589159	2.334191469	protein_coding	16	48200821	48281479	ABCC11
ENSG00000121274	8.191677941	8.46235258	protein_coding	16	50186829	50269221	PAPD5
ENSG00000121281	9.911010438	10.14031537	protein_coding	16	50280048	50352046	ADCY7
ENSG00000121289	6.771940731	6.948652068	protein_coding	19	33369908	33462869	CEP89
ENSG00000121310	8.314756163	8.669360359	protein_coding	1	53361656	53392884	ECHDC2
ENSG00000121316	0.869158192	1.667587519	protein_coding	12	14656595	14721283	PLBD1
ENSG00000121318	0.869158192	0	protein_coding	12	10977916	10978957	TAS2R10
ENSG00000121335	0	1.16600992	protein_coding	12	11544476	11653975	PRB2
ENSG00000121350	5.795854912	5.555337277	protein_coding	12	21590549	21623300	PYROXD1
ENSG00000121361	1.960915222	1.16600992	protein_coding	12	21917889	21928515	KCNJ8
ENSG00000121380	1.960915222	2.334191469	protein_coding	12	12202797	12363947	BCL2L14
ENSG00000121390	8.566693498	8.68797299	protein_coding	13	20248896	20357142	PSPC1
ENSG00000121406	6.956090577	6.811590016	protein_coding	19	58038693	58052124	ZNF549
ENSG00000121410	6.163378456	6.624193585	protein_coding	19	58858172	58864865	A1BG
ENSG00000121413	8.891945431	9.040423325	protein_coding	19	58595212	58629793	ZSCAN18
ENSG00000121417	7.395627075	7.510161044	protein_coding	19	58141761	58154147	ZNF211
ENSG00000121454	3.989024711	4.29708397	protein_coding	1	180199421	180249380	LHX4
ENSG00000121481	7.04004079	6.908136232	protein_coding	1	185014496	185071740	RNF2
ENSG00000121486	7.402691859	8.053329862	protein_coding	1	185087220	185126204	TRMT1L
ENSG00000121542	6.738827102	6.877938671	protein_coding	3	122920774	122992977	SEC22A
ENSG00000121552	5.141750194	4.406904905	protein_coding	3	122044091	122060819	CSTA
ENSG00000121577	1.16343121	2.144285137	protein_coding	3	119355304	119384171	POPDC2
ENSG00000121578	7.285281023	7.57041104	protein_coding	3	118930579	118959950	B4GALT4
ENSG00000121579	10.82868101	10.58756595	protein_coding	3	113437841	113465147	NAA50
ENSG00000121594	1.16343121	0.697730409	protein_coding	3	119243140	119278449	CD80
ENSG00000121621	8.368064704	8.728729322	protein_coding	11	28042167	28129693	KIF18A
ENSG00000121634	0	0.390640832	protein_coding	1	147374946	147381393	GJA8
ENSG00000121644	9.665877245	9.778507838	protein_coding	1	244816237	244872335	PPPDE1
ENSG00000121653	7.186405829	7.679480043	protein_coding	11	45907202	45928016	MAPK8IP1
ENSG00000121671	6.872051157	6.673373662	protein_coding	11	45868669	45904798	CRY2
ENSG00000121680	8.002356002	8.417132063	protein_coding	11	45931220	45940363	PEX16
ENSG00000121690	6.892270075	7.022976646	protein_coding	11	33037410	33055128	DEPDC7
ENSG00000121691	9.116949866	8.878275203	protein_coding	11	34460472	34493609	CAT
ENSG00000121716	9.136151669	9.062408117	protein_coding	7	99933702	99965454	PILRB
ENSG00000121741	9.564789794	9.250933796	protein_coding	13	20532810	20665968	ZMYM2
ENSG00000121742	2.10647801	0	protein_coding	13	20796110	20806534	GJB6
ENSG00000121743	6.005279909	3.027231696	protein_coding	13	20712394	20735188	GJA3
ENSG00000121749	8.094677541	8.669360359	protein_coding	12	72233487	72320629	TBC1D15
ENSG00000121753	7.726437377	8.019149911	protein_coding	1	32192718	32230494	BAI2
ENSG00000121764	0.499066092	0.950786998	protein_coding	1	32083287	32098119	HCRTR1
ENSG00000121766	7.639400234	7.951888765	protein_coding	1	31769842	31837783	ZCCHC17
ENSG00000121769	2.35979773	1.353254395	protein_coding	1	31838472	31849697	FABP3
ENSG00000121774	10.97774947	10.85408437	protein_coding	1	32479430	32526451	KHDRBS1
ENSG00000121775	7.845284967	7.714077892	protein_coding	1	32537632	32568467	TMEM39B
ENSG00000121797	5.625150983	5.112089758	protein_coding	3	46448654	46451014	CCRL2
ENSG00000121807	0.499066092	0.390640832	protein_coding	3	46395225	46402419	CCR2
ENSG00000121848	8.508940744	8.83962623	protein_coding	1	145611036	145690661	RNF115
ENSG00000121851	7.440941622	7.648510984	protein_coding	1	145456236	145470388	POLR3GL
ENSG00000121858	3.698846687	3.96493948	protein_coding	3	172223298	172241297	TNFSF10
ENSG00000121864	8.389559911	8.315177126	protein_coding	3	179040779	179053323	ZNF639
ENSG00000121871	0	0.390640832	protein_coding	3	164904508	164914897	SLITRK3
ENSG00000121879	8.385999544	8.363653959	protein_coding	3	178865902	178957881	PIK3CA
ENSG00000121892	10.63155234	10.65144	protein_coding	4	39824483	39979576	PDS5A

ENSG00000121895	2.762599152	2.242360793	protein_coding	4	38968365	39034542	TMEM156
ENSG00000121897	5.879259127	5.808454063	protein_coding	4	39460620	39479273	LIAS
ENSG00000121898	0.869158192	1.16600992	protein_coding	10	125465729	125699783	CPXM2
ENSG00000121900	7.593829938	6.984522608	protein_coding	1	33360195	33367039	TMEM54
ENSG00000121903	5.472368541	5.259915406	protein_coding	1	33938232	33961995	ZSCAN20
ENSG00000121904	0.499066092	0.697730409	protein_coding	1	33979609	34631443	CSMD2
ENSG00000121905	1.616589159	2.039052734	protein_coding	1	33351595	33364042	HPCA
ENSG00000121931	8.183497283	8.253299769	protein_coding	1	111489807	111506701	LRIF1
ENSG00000121940	8.504022011	8.802929991	protein_coding	1	109472130	109506111	CLCC1
ENSG00000121957	8.032273249	7.883239271	protein_coding	1	109417972	109473044	GPSM2
ENSG00000121964	7.119373418	6.811590016	protein_coding	2	144703321	145090135	GTDC1
ENSG00000121966	0	0.390640832	protein_coding	2	136871919	136875735	CXCR4
ENSG00000121988	7.049074049	6.815578497	protein_coding	2	135894486	136288806	ZRANB3
ENSG00000121989	6.502803968	6.167968749	protein_coding	2	148602086	148688393	ACVR2A
ENSG00000122008	7.757079669	7.568048738	protein_coding	5	74807581	74896969	POLK
ENSG00000122026	6.350376764	5.709041716	protein_coding	13	27825446	27830828	RPL21
ENSG00000122033	6.94645393	6.549526374	protein_coding	13	28009776	28024739	MTIF3
ENSG00000122034	9.221529789	8.598211347	protein_coding	13	27998681	28009958	GTF3A
ENSG00000122035	3.652182994	3.279072565	protein_coding	13	27844464	27847827	RASL11A
ENSG00000122042	7.575190993	7.234379288	protein_coding	13	30338508	30424821	UBL3
ENSG00000122068	9.185117095	9.162882465	protein_coding	3	197464050	197511317	FYTTD1
ENSG00000122085	7.734859118	7.575124073	protein_coding	2	242014469	242041747	MTERFD2
ENSG00000122122	1.16343121	0.390640832	protein_coding	X	128913894	128929177	SASH3
ENSG00000122126	9.336667406	9.640299997	protein_coding	X	128673826	128726538	OCRL
ENSG00000122133	4.642199401	4.528525526	protein_coding	9	138453602	138458801	PAEP
ENSG00000122140	10.19307491	10.0814915	protein_coding	9	138391830	138396519	MRPS2
ENSG00000122188	0.499066092	0	protein_coding	1	203734304	203745361	LAX1
ENSG00000122194	0.499066092	0	protein_coding	6	161123270	161174338	PLG
ENSG00000122203	9.502033728	9.239845842	protein_coding	5	175773064	175788971	KIAA1191
ENSG00000122218	12.04653826	11.92084068	protein_coding	1	160259063	160313190	COPA
ENSG00000122224	0.499066092	0	protein_coding	1	160765896	160798045	LY9
ENSG00000122257	9.728365606	9.779529137	protein_coding	16	24549014	24584184	RBBP6
ENSG00000122299	9.290757201	9.406451268	protein_coding	16	11844442	11891099	ZC3H7A
ENSG00000122335	5.977159882	6.737844962	protein_coding	6	158530536	158589312	SERAC1
ENSG00000122359	10.59501123	10.18686154	protein_coding	10	81910645	81965433	ANXA11
ENSG00000122367	3.077135474	4.319727133	protein_coding	10	88426549	88495825	LDB3
ENSG00000122376	7.81647827	8.223589663	protein_coding	10	88854953	88951225	FAM35A
ENSG00000122378	7.003331296	7.237355663	protein_coding	10	82167585	82192753	FAM213A
ENSG00000122386	7.842689815	8.043160634	protein_coding	16	3162561	3170518	ZNF205
ENSG00000122390	9.507778518	9.511607983	protein_coding	16	3493682	3536960	NAA60
ENSG00000122406	11.89117065	12.24248605	protein_coding	1	93297597	93307481	RPL5
ENSG00000122417	7.90122817	8.265014588	protein_coding	1	86786611	86862025	ODF2L
ENSG00000122420	1.16343121	1.925536307	protein_coding	1	78769568	79005434	PTGFR
ENSG00000122432	0	1.925536307	pseudogene	1	84971974	85031877	SPATA1
ENSG00000122435	6.050961887	6.414040573	protein_coding	1	100598706	100616053	CCDC76
ENSG00000122477	2.10647801	1.667587519	protein_coding	1	100614409	100643771	LRRC39
ENSG00000122481	5.718698586	6.445267191	protein_coding	1	95699711	95712781	RWDD3
ENSG00000122482	8.074911866	8.673761454	protein_coding	1	91380859	91487829	ZNF644
ENSG00000122483	6.733233509	7.019522872	protein_coding	1	93645476	93744287	CCDC18
ENSG00000122484	6.975172889	7.182818183	protein_coding	1	92764522	92853730	RPAP2
ENSG00000122490	8.750975986	8.323603978	protein_coding	18	77662420	77711664	PQLC1
ENSG00000122497	5.91922012	6.264499759	protein_coding	1	148003642	148025863	NBPF14
ENSG00000122507	7.374222747	6.995112131	protein_coding	7	33168856	33645680	BBS9
ENSG00000122512	7.030950614	7.033288635	protein_coding	7	6012870	6048756	PMS2
ENSG00000122515	11.87839906	11.15313485	protein_coding	7	44788180	44809477	ZMIZ2
ENSG00000122543	0.499066092	1.802319292	protein_coding	7	5919458	5925993	OCM
ENSG00000122545	10.30806544	10.414292	protein_coding	7	35840542	35944917	7-sep
ENSG00000122547	6.793601635	5.700439663	protein_coding	7	36192758	36341152	EEPD1
ENSG00000122548	0	0.390640832	protein_coding	7	26572740	26578407	KIAA0087
ENSG00000122550	8.417730894	8.282410365	protein_coding	7	23145353	23215040	KLHL7
ENSG00000122557	8.35173048	7.83885796	protein_coding	7	35672269	35734745	HERPUD2
ENSG00000122565	10.94273465	10.90012121	protein_coding	7	26240782	26252976	CBX3
ENSG00000122566	13.61577736	13.42479433	protein_coding	7	26229547	26241149	HNRNPA2B1

ENSG00000122574	4.491309013	1.518964905	protein_coding	7	29846102	29956682	WIPF3
ENSG00000122591	9.942255804	9.572977394	protein_coding	7	22980878	23053749	FAM126A
ENSG00000122592	0.499066092	0.950786998	protein_coding	7	27193335	27197555	HOXA7
ENSG00000122641	8.967738537	9.832648794	protein_coding	7	41724712	41742706	INHBA
ENSG00000122642	10.02413132	9.797285822	protein_coding	7	32997017	33046543	FKBP9
ENSG00000122643	8.325948295	8.269383236	protein_coding	7	33053742	33102409	NT5C3
ENSG00000122644	8.316627558	8.135396417	protein_coding	7	12726481	12730559	ARL4A
ENSG00000122674	6.548110424	6.037446454	protein_coding	7	5938356	5965605	CCZ1
ENSG00000122678	7.692250012	7.668495823	protein_coding	7	44111846	44122139	POLM
ENSG00000122687	8.973680846	8.714915547	protein_coding	7	2273866	2281840	FTSJ2
ENSG00000122692	9.195324263	9.065760854	protein_coding	9	33041762	33076665	SMU1
ENSG00000122694	6.095241663	6.365895578	protein_coding	9	36136730	36163910	GLIPR2
ENSG00000122696	7.062518763	6.877938671	protein_coding	9	37879400	37904350	MCART1
ENSG00000122705	9.731875952	10.09303901	protein_coding	9	36190853	36304778	CLTA
ENSG00000122707	6.320845526	6.535085617	protein_coding	9	36036430	36124448	RECK
ENSG00000122711	0	0.950786998	protein_coding	9	33218363	33248565	SPINK4
ENSG00000122729	10.56147155	10.66697623	protein_coding	9	32384601	32450834	ACO1
ENSG00000122733	0.499066092	1.518964905	protein_coding	9	34957484	34984679	KIAA1045
ENSG00000122735	2.57521082	3.570966319	protein_coding	9	34457412	34520982	DNAI1
ENSG00000122741	8.325948295	8.305283181	protein_coding	9	37800499	37867663	DCAF10
ENSG00000122756	0	0.697730409	protein_coding	9	34551430	34590121	CNTFR
ENSG00000122778	8.059349131	7.953699568	protein_coding	7	138516126	138666064	KIAA1549
ENSG00000122779	10.60805898	10.15416199	protein_coding	7	138144953	138274738	TRIM24
ENSG00000122783	8.199812472	8.253299769	protein_coding	7	134777115	134855547	C7orf49
ENSG00000122786	11.13343133	11.31050189	protein_coding	7	134429003	134655479	CALD1
ENSG00000122787	1.16343121	0.950786998	protein_coding	7	137687070	137802732	AKR1D1
ENSG00000122852	0.499066092	0	protein_coding	10	81370695	81375197	SFTPA1
ENSG00000122861	11.01712264	8.983949389	protein_coding	10	75668935	75677255	PLAU
ENSG00000122862	6.25990527	4.048652948	protein_coding	10	70847862	70864567	SRGN
ENSG00000122863	9.672468519	9.214393111	protein_coding	10	73724123	73773322	CHST3
ENSG00000122870	7.464764762	7.832967122	protein_coding	10	60272900	60591195	BICC1
ENSG00000122873	9.526494794	9.472987904	protein_coding	10	60028818	60049346	CISD1
ENSG00000122877	5.948480845	3.279072565	protein_coding	10	64571756	64679660	EGR2
ENSG00000122882	8.495786671	8.241489045	protein_coding	10	74889913	74927853	ECD
ENSG00000122884	9.812996626	9.747005744	protein_coding	10	74766975	74856732	P4HA1
ENSG00000122912	6.428602508	6.142791909	protein_coding	10	70242521	70287231	SLC25A16
ENSG00000122952	9.597483841	9.617635386	protein_coding	10	58116989	58121036	ZWINT
ENSG00000122958	9.715657165	9.622766057	protein_coding	10	70883268	70932617	VPS26A
ENSG00000122965	8.915598341	8.873500326	protein_coding	12	114254543	114404176	RBM19
ENSG00000122966	9.151960225	8.910331892	protein_coding	12	120123595	120315095	CIT
ENSG00000122970	6.313367288	6.577981353	protein_coding	12	110562140	110656602	IFT81
ENSG00000122971	6.92698535	7.138927266	protein_coding	12	121163538	121177811	ACADS
ENSG00000122986	1.616589159	1.802319292	protein_coding	12	111065646	111142755	HVCN1
ENSG00000123009	3.603959378	3.679994897	pseudogene	12	120719940	120720632	NME2P1
ENSG00000123064	10.37335233	9.975411057	protein_coding	12	113594979	113623284	DDX54
ENSG00000123066	9.494613796	8.958022094	protein_coding	12	116395711	116715143	MED13L
ENSG00000123080	7.621344476	8.351343215	protein_coding	1	51426417	51440305	CDKN2C
ENSG00000123091	8.914363072	9.077434451	protein_coding	1	51701943	51739127	RNF11
ENSG00000123094	7.903720218	8.232567113	protein_coding	12	26111962	26232825	RASSF8
ENSG00000123095	7.633406721	7.354383726	protein_coding	12	26272959	26278060	BHLHE41
ENSG00000123096	4.593635215	2.242360793	protein_coding	12	26274924	26452223	SSPN
ENSG00000123104	8.894453552	9.177677745	protein_coding	12	26490342	26986131	ITPR2
ENSG00000123106	7.501423995	7.225413142	protein_coding	12	28286182	28732883	CCDC91
ENSG00000123119	1.16343121	4.342020395	protein_coding	8	91803778	91971636	NECAB1
ENSG00000123124	8.596266901	7.980592361	protein_coding	8	87354967	87490649	WWP1
ENSG00000123130	9.489645889	9.931061721	protein_coding	X	23720370	23784592	ACOT9
ENSG00000123131	10.0326953	10.95403662	protein_coding	X	23682379	23704516	PRDX4
ENSG00000123136	10.99771618	11.1476082	protein_coding	19	14519633	14530171	DDX39A
ENSG00000123143	11.56375436	11.89327141	protein_coding	19	14544169	14582678	PKN1
ENSG00000123144	10.72540341	11.43141474	protein_coding	19	12841455	12845529	C19orf43
ENSG00000123146	11.24130516	11.33779859	protein_coding	19	14492213	14519531	CD97
ENSG00000123154	8.070482475	8.119352325	protein_coding	19	12777614	12786646	WDR83
ENSG00000123159	10.7728634	10.58989654	protein_coding	19	14588572	14606944	GIPC1

ENSG00000123178	5.986594255	5.393992	protein_coding	13	50486842	50510626	SPRYD7
ENSG00000123179	7.64536895	7.170413581	protein_coding	13	50234859	50265623	EBPL
ENSG00000123191	5.637181576	5.137803333	protein_coding	13	52506809	52585630	ATP7B
ENSG00000123200	9.551342933	8.903789961	protein_coding	13	46528600	46626894	ZC3H13
ENSG00000123201	0.499066092	0.950786998	pseudogene	13	51568651	51654998	GUCY1B2
ENSG00000123213	9.766517491	8.961626095	protein_coding	5	65018023	65167553	NLN
ENSG00000123219	7.356139979	7.252146121	protein_coding	5	64813593	64858998	CENPK
ENSG00000123240	10.73488119	10.57468	protein_coding	10	13141449	13180291	OPTN
ENSG00000123243	0	1.16600992	protein_coding	10	7601232	7708961	ITIH5
ENSG00000123268	8.3053626	8.049948078	protein_coding	12	51157493	51214905	ATF1
ENSG00000123297	8.407231133	9.01636662	protein_coding	12	58176372	58201854	TSFM
ENSG00000123329	0.869158192	1.518964905	protein_coding	12	57866038	57882597	ARHGAP9
ENSG00000123338	0.869158192	0.950786998	protein_coding	12	54891495	54937726	NCKAP1L
ENSG00000123342	5.360713447	5.96747225	protein_coding	12	56229217	56236750	MMP19
ENSG00000123349	9.68845487	10.39676134	protein_coding	12	53689075	53693234	PFDN5
ENSG00000123352	8.919297816	8.861011175	protein_coding	12	49760367	49921207	SPATS2
ENSG00000123353	7.254372086	7.219404604	protein_coding	12	56211703	56215663	ORMDL2
ENSG00000123358	10.12208492	3.993387124	protein_coding	12	52416616	52453291	NR4A1
ENSG00000123360	0	0.697730409	protein_coding	12	54943134	54973023	PDE1B
ENSG00000123364	6.095241663	6.642271819	protein_coding	12	54332535	54340328	HOXC13
ENSG00000123374	9.548956892	9.562928737	protein_coding	12	56360553	56366568	CDK2
ENSG00000123384	10.3925533	10.85868225	protein_coding	12	57522276	57607134	LRP1
ENSG00000123388	1.616589159	1.667587519	protein_coding	12	54366910	54371427	HOXC11
ENSG00000123395	8.312882337	8.187110572	protein_coding	12	52463030	52471278	C12orf44
ENSG00000123405	5.588445295	4.468996429	protein_coding	12	54685895	54694905	NFE2
ENSG00000123411	6.897280837	6.877938671	protein_coding	12	56401443	56432219	IKZF4
ENSG00000123415	7.657232808	7.926295939	protein_coding	12	54558529	54582778	SMUG1
ENSG00000123416	10.86781572	11.17192425	protein_coding	12	49521565	49525180	TUBA1B
ENSG00000123427	7.334137164	7.32390599	protein_coding	12	58165275	58176324	METTL21B
ENSG00000123444	6.086493765	6.424524724	protein_coding	11	47593749	47600567	KBTBD4
ENSG00000123453	7.90122817	7.918899532	protein_coding	9	136528682	136605077	SARDH
ENSG00000123454	4.883502971	4.903073497	protein_coding	9	136501482	136524466	DBH
ENSG00000123472	8.278730502	8.579568843	protein_coding	1	47098409	47139539	ATPAF1
ENSG00000123473	8.38421606	8.628000846	protein_coding	1	47715811	47779819	STIL
ENSG00000123485	9.108333003	9.304420832	protein_coding	2	234742062	234763212	HJURP
ENSG00000123496	1.407729925	2.652276565	protein_coding	X	114238538	114254540	IL13RA2
ENSG00000123500	0.869158192	0.390640832	protein_coding	6	116440086	116479910	COL10A1
ENSG00000123505	10.06867432	10.31353975	protein_coding	6	111195973	111216916	AMD1
ENSG00000123545	6.456028446	7.033288635	protein_coding	6	97337189	97345757	NDUFAF4
ENSG00000123552	6.830742851	7.116470702	protein_coding	6	99880190	99969604	USP45
ENSG00000123560	1.16343121	1.518964905	protein_coding	X	103028647	103047548	PLP1
ENSG00000123562	11.85428416	12.18323438	protein_coding	X	102930424	102943086	MORF4L2
ENSG00000123570	1.799000381	3.532712221	protein_coding	X	103077252	103087158	RAB9B
ENSG00000123572	0	0.697730409	protein_coding	X	105066536	105202602	NRK
ENSG00000123575	9.717783028	9.747527757	protein_coding	X	103411301	103440583	FAM199X
ENSG00000123595	7.639400234	8.652735634	protein_coding	X	13707244	13728625	RAB9A
ENSG00000123600	7.870982917	7.724721747	protein_coding	2	172179761	172291312	METTL8
ENSG00000123607	7.440941622	7.743685008	protein_coding	2	166713985	166810353	TTC21B
ENSG00000123609	8.112022537	8.125791388	protein_coding	2	152126979	152146571	NMI
ENSG00000123610	0.869158192	1.925536307	protein_coding	2	152214106	152236560	TNFAIP6
ENSG00000123612	0.869158192	0.697730409	protein_coding	2	158388888	158485517	ACVR1C
ENSG00000123636	7.319279953	7.10998981	protein_coding	2	160175490	160473203	BAZ2B
ENSG00000123643	7.703736051	7.621426466	protein_coding	5	150816607	150871942	SLC36A1
ENSG00000123684	9.064459359	8.913126522	protein_coding	1	211916799	212004114	LPGAT1
ENSG00000123685	4.712111592	4.7279204	protein_coding	1	212859760	212873327	BATF3
ENSG00000123689	9.000704568	9.075772566	protein_coding	1	209848765	209849733	G0S2
ENSG00000123728	8.946141254	8.750768196	protein_coding	X	131337053	131353471	RAP2C
ENSG00000123737	8.385999544	8.512355001	protein_coding	4	122722472	122738176	EXOSC9
ENSG00000123739	7.071412799	6.984522608	protein_coding	4	110631145	110651233	PLA2G12A
ENSG00000123810	5.286269488	5.923798455	protein_coding	19	41860322	41870078	B9D2
ENSG00000123815	6.305850085	6.408769788	protein_coding	19	41197434	41222790	ADCK4
ENSG00000123836	7.292905885	6.690849666	protein_coding	1	207222801	207254369	PFKFB2
ENSG00000123870	5.718698586	5.832272499	pseudogene	19	53095291	53100660	ZNF137P

ENSG00000123892	4.381167248	1.802319292	protein_coding	11	87846431	87908635	RAB38
ENSG00000123901	1.799000381	2.851901313	protein_coding	11	94110477	94134585	GPR83
ENSG00000123908	8.489164384	8.363653959	protein_coding	8	141541264	141645718	EIF2C2
ENSG00000123933	9.191250057	9.495538149	protein_coding	4	2249159	2264021	MXD4
ENSG00000123965	4.062237333	4.226940739	pseudogene	7	74306894	74366314	PMS2P5
ENSG00000123975	9.830784156	10.0823194	protein_coding	9	91926113	91931618	CKS2
ENSG00000123977	3.950976309	3.608232228	protein_coding	2	228735770	228789060	WDR69
ENSG00000123983	10.68287268	9.842944292	protein_coding	2	223725652	223809357	ACSL3
ENSG00000123989	11.23290649	11.12689325	protein_coding	2	220403669	220408509	CHPF
ENSG00000123992	9.850294221	9.674741242	protein_coding	2	220238268	220586393	DNPEP
ENSG00000123999	3.393122761	4.342020395	protein_coding	2	220433884	220440435	INHA
ENSG00000124006	9.255095676	9.496780665	protein_coding	2	220415451	220436581	OBSL1
ENSG00000124067	9.260938843	9.36078013	protein_coding	16	67977377	68002597	SLC12A4
ENSG00000124074	7.765324926	8.202422444	protein_coding	16	67696848	67700667	C16orf48
ENSG00000124091	1.799000381	2.501982735	protein_coding	20	55066548	55100981	GCNT7
ENSG00000124092	0.499066092	0.697730409	protein_coding	20	56071021	56100708	CTCFL
ENSG00000124097	0.869158192	0.390640832	pseudogene	20	56063448	56064083	HMGB1P1
ENSG00000124098	8.492479328	8.652735634	protein_coding	20	54933971	54943719	FAM210B
ENSG00000124102	4.903563137	5.283144778	protein_coding	20	43803517	43805185	PI3
ENSG00000124103	2.471521042	0.697730409	protein_coding	20	55092243	55101198	FAM209A
ENSG00000124104	7.07583934	7.522414019	protein_coding	20	44462449	44471914	SNX21
ENSG00000124107	8.54291017	8.141764426	protein_coding	20	43880880	43883205	SLPI
ENSG00000124116	5.107569429	6.970281236	protein_coding	20	44376583	44420571	WFDC3
ENSG00000124120	8.883132512	8.791837833	protein_coding	20	43104526	43123244	TTPAL
ENSG00000124126	7.102117104	3.679994897	protein_coding	20	47240790	47444420	PREX1
ENSG00000124134	3.78787645	4.29708397	protein_coding	20	43720951	43729753	KCNS1
ENSG00000124140	3.393122761	3.813624741	protein_coding	20	44650329	44688789	SLC12A5
ENSG00000124143	7.886184801	8.99190196	protein_coding	20	37230577	37279678	ARHGAP40
ENSG00000124145	12.2990708	12.03972983	protein_coding	20	43953928	43977064	SDC4
ENSG00000124151	10.14069086	9.794762494	protein_coding	20	46130601	46285621	NCOA3
ENSG00000124155	10.46605656	11.10016547	protein_coding	20	44044707	44054884	PIGT
ENSG00000124159	2.35979773	2.334191469	protein_coding	20	43922085	43937169	MATN4
ENSG00000124160	9.020358234	9.178452256	protein_coding	20	44689624	44718591	NCOA5
ENSG00000124164	9.756212088	9.483680566	protein_coding	20	56964178	57026157	VAPB
ENSG00000124171	8.092494681	7.246248121	protein_coding	20	49348081	49373332	PARD6B
ENSG00000124172	9.772665583	10.38338814	protein_coding	20	57600522	57607437	ATP5E
ENSG00000124177	8.954578658	9.149514732	protein_coding	20	40030741	40247133	CHD6
ENSG00000124181	9.205459721	9.137615924	protein_coding	20	39765600	39804361	PLCG1
ENSG00000124191	9.998712996	9.277202279	protein_coding	20	42543492	42698256	TOX2
ENSG00000124193	10.91829414	11.00709988	protein_coding	20	42086568	42092245	SRSF6
ENSG00000124194	0.499066092	2.334191469	protein_coding	20	42875887	42909013	GDAP1L1
ENSG00000124198	9.877806407	10.03862623	protein_coding	20	47538275	47653230	ARFGEF2
ENSG00000124201	10.41062624	10.49826541	protein_coding	20	47826982	47894963	ZNFX1
ENSG00000124207	11.86631214	11.80248922	protein_coding	20	47662838	47713489	CSE1L
ENSG00000124209	8.966547133	8.857146507	protein_coding	20	56884752	56942563	RAB22A
ENSG00000124212	1.16343121	0.390640832	protein_coding	20	48123992	48184683	PTGIS
ENSG00000124214	10.99362791	10.80176251	protein_coding	20	47729878	47804904	STAU1
ENSG00000124215	3.950976309	4.29708397	protein_coding	20	58533471	58609066	CDH26
ENSG00000124216	8.929116952	8.458531597	protein_coding	20	48599536	48605423	SNAI1
ENSG00000124217	7.437505974	7.258020107	protein_coding	20	49575363	49577820	MOCS3
ENSG00000124222	10.09453648	9.698154036	protein_coding	20	57226328	57254582	STX16
ENSG00000124224	7.132181536	7.016060809	protein_coding	20	56806188	56884495	PPP4R1L
ENSG00000124225	10.04007652	8.660517685	protein_coding	20	56223448	56286592	PMEPA1
ENSG00000124226	9.527303063	9.639175184	protein_coding	20	48552945	48570429	RNF114
ENSG00000124227	0	0.390640832	protein_coding	20	56793551	56803709	ANKRD60
ENSG00000124228	9.94104316	9.807835867	protein_coding	20	47835832	47860614	DDX27
ENSG00000124232	0	0.390640832	protein_coding	20	43935491	43945803	RBPJL
ENSG00000124233	0	0.390640832	protein_coding	20	43835638	43838413	SEMG1
ENSG00000124243	6.820228284	6.870289474	protein_coding	20	49411431	49493714	BCAS4
ENSG00000124249	1.616589159	1.16600992	protein_coding	20	43374421	43379675	KCNK15
ENSG00000124251	3.554067925	3.32456471	protein_coding	20	44002526	44036529	TP53TG5
ENSG00000124253	0.499066092	0	protein_coding	20	56131260	56141513	PCK1
ENSG00000124256	0.499066092	0.390640832	protein_coding	20	56178902	56195632	ZBP1

ENSG00000124257	4.261916566	4.604259349	protein_coding	20	44517264	44519926	NEURL2
ENSG00000124275	9.782177506	9.63410264	protein_coding	5	7851299	7906138	MTRR
ENSG00000124279	7.734859118	7.556178852	protein_coding	5	7859272	7869150	FASTKD3
ENSG00000124299	9.396283191	9.589959321	protein_coding	19	33877855	34012799	PEPD
ENSG00000124313	8.497437504	8.715982854	protein_coding	X	53262058	53350522	IQSEC2
ENSG00000124333	9.290757201	9.504213373	protein_coding	X	155110956	155173433	VAMP7
ENSG00000124334	3.603959378	3.570966319	protein_coding	X	155227246	155251689	IL9R
ENSG00000124356	8.860220382	8.899098923	protein_coding	2	74056086	74100786	STAMBP
ENSG00000124357	8.148202495	8.983949389	protein_coding	2	71291474	71306935	NAGK
ENSG00000124370	5.158541613	5.496926774	protein_coding	2	71336814	71357369	MCEE
ENSG00000124374	5.223818625	5.536128679	protein_coding	2	71409869	71454213	PAIP2B
ENSG00000124380	6.793601635	7.119700263	protein_coding	2	70120692	70132707	SNRNP27
ENSG00000124383	8.628263112	8.520929986	protein_coding	2	71357444	71377231	MPHOSPH10
ENSG00000124391	1.407729925	0.390640832	protein_coding	16	88704999	88706881	IL17C
ENSG00000124406	4.903563137	1.518964905	protein_coding	4	42410390	42659122	ATP8A1
ENSG00000124422	12.21189204	12.30393206	protein_coding	17	20902906	20947073	USP22
ENSG00000124429	1.616589159	0	protein_coding	X	84532402	84634748	POF1B
ENSG00000124440	1.407729925	3.081239798	protein_coding	19	46800303	46846690	HIF3A
ENSG00000124444	6.050961887	6.403479675	protein_coding	19	44100544	44105309	ZNF576
ENSG00000124455	0.499066092	1.925536307	protein_coding	19	44080952	44083238	
ENSG00000124459	7.997697782	8.206984494	protein_coding	19	44416776	44439411	ZNF45
ENSG00000124466	7.748787017	4.693656882	protein_coding	19	43964946	43969831	LYPD3
ENSG00000124467	2.10647801	3.279072565	protein_coding	19	43256838	43269848	PSG8
ENSG00000124469	0	0.390640832	protein_coding	19	43084393	43099082	CEACAM8
ENSG00000124479	0	0.390640832	protein_coding	X	43808022	43832750	NDP
ENSG00000124486	11.73638147	11.68259048	protein_coding	X	40944888	41092185	USP9X
ENSG00000124491	0	0.390640832	protein_coding	6	6144318	6321246	F13A1
ENSG00000124493	0.499066092	1.16600992	protein_coding	6	33989628	34123399	GRM4
ENSG00000124496	8.465744027	7.436965343	protein_coding	6	42192669	42419789	TRERF1
ENSG00000124507	0	0.390640832	protein_coding	6	34433916	34503006	PACSIN1
ENSG00000124508	7.186405829	8.101495266	protein_coding	6	26383324	26395102	BTN2A2
ENSG00000124523	7.049074049	7.951888765	protein_coding	6	13574826	13612524	SIRT5
ENSG00000124529	0.869158192	1.353254395	protein_coding	6	26027124	26027480	HIST1H4B
ENSG00000124532	8.3352092	8.548751381	protein_coding	6	24403153	24425810	MRS2
ENSG00000124535	9.420848964	9.484307085	protein_coding	6	2765648	2786927	WRNIP1
ENSG00000124541	9.387407331	9.422231406	protein_coding	6	42989383	42997335	RRP36
ENSG00000124549	4.962122459	5.466810074	pseudogene	6	26421619	26431928	BTN2A3P
ENSG00000124562	9.582785382	10.27531537	protein_coding	6	34725183	34741571	SNRPC
ENSG00000124570	10.22779886	10.13074314	protein_coding	6	2948393	2972399	SERPINB6
ENSG00000124571	10.44096868	10.30469907	protein_coding	6	43490072	43543812	XPO5
ENSG00000124574	9.232475031	9.587045627	protein_coding	6	43395104	43418168	ABCC10
ENSG00000124575	3.077135474	2.721932731	protein_coding	6	26234440	26235216	HIST1H1D
ENSG00000124587	7.603059879	7.243290054	protein_coding	6	42931608	42946958	PEX6
ENSG00000124588	8.787867896	8.896276962	protein_coding	6	2988221	3019996	NQO2
ENSG00000124593	4.097495944	4.976162367	protein_coding	6	41748087	41757879	PRICKLE4
ENSG00000124596	7.102117104	7.298481685	protein_coding	6	41001366	41065526	C6orf130
ENSG00000124602	5.316509574	5.988823159	protein_coding	6	40994772	41006928	UNC5CL
ENSG00000124608	7.740446424	7.747865429	protein_coding	6	44267391	44281063	AARS2
ENSG00000124610	2.10647801	1.667587519	protein_coding	6	26017260	26018040	HIST1H1A
ENSG00000124613	7.30049066	7.309836726	protein_coding	6	27342394	27371683	ZNF391
ENSG00000124614	9.953725374	10.68070787	protein_coding	6	34385231	34393902	RPS10
ENSG00000124615	0.499066092	0.390640832	protein_coding	6	39867354	39902290	MOCS1
ENSG00000124635	4.942865586	4.826083323	protein_coding	6	27094058	27100541	HIST1H2BJ
ENSG00000124641	8.3053626	8.486319932	protein_coding	6	41873092	41888877	MED20
ENSG00000124657	0.499066092	2.242360793	protein_coding	6	27925019	27925960	OR2B6
ENSG00000124659	7.621344476	8.330588949	protein_coding	6	42712219	42714558	TBCC
ENSG00000124664	2.671945279	0.950786998	protein_coding	6	34505579	34524110	SPDEF
ENSG00000124678	0.499066092	0.950786998	protein_coding	6	35085848	35116387	TCP11
ENSG00000124688	7.35977471	7.582164879	protein_coding	6	43597277	43608689	MAD2L1BP
ENSG00000124693	2.10647801	1.802319292	protein_coding	6	26031817	26032288	HIST1H3B
ENSG00000124701	1.407729925	2.652276565	protein_coding	6	41021043	41032465	APOBEC2
ENSG00000124702	10.14438343	10.53863841	protein_coding	6	42981951	42989036	KLHDC3
ENSG00000124713	1.616589159	2.039052734	protein_coding	6	42928496	42931618	GNMT

ENSG00000124731	0.499066092	0.697730409	protein_coding	6	41243712	41254457	TREM1
ENSG00000124733	8.329659793	9.112709611	protein_coding	6	42979832	42981706	MEA1
ENSG00000124743	2.471521042	3.232099092	protein_coding	6	53512699	53530506	KLHL31
ENSG00000124749	1.16343121	0	protein_coding	6	55921388	56258892	COL21A1
ENSG00000124762	9.9349646	9.12320659	protein_coding	6	36644305	36655116	CDKN1A
ENSG00000124766	8.357195795	8.595894166	protein_coding	6	21593972	21598847	SOX4
ENSG00000124767	11.2306751	11.33953089	protein_coding	6	38643719	38670917	GLO1
ENSG00000124772	4.821592551	4.547834947	protein_coding	6	36708552	36807778	CPNE5
ENSG00000124782	9.51676008	8.948064416	protein_coding	6	7107830	7252213	RREB1
ENSG00000124783	11.27104537	11.24343157	protein_coding	6	7268539	7347679	SSR1
ENSG00000124784	8.380642463	8.581912372	protein_coding	6	7389729	7418270	RIOK1
ENSG00000124785	1.616589159	1.518964905	protein_coding	6	5998232	6007200	NRN1
ENSG00000124786	7.488200893	8.106387334	protein_coding	6	8413301	8435794	SLC35B3
ENSG00000124787	7.190493718	7.556178852	protein_coding	6	4994966	5004297	RPP40
ENSG00000124788	8.737069306	8.941691738	protein_coding	6	16299343	16761722	ATXN1
ENSG00000124789	10.68830327	10.75268144	protein_coding	6	17615269	17706818	NUP153
ENSG00000124795	10.93331302	11.33363257	protein_coding	6	18224099	18265054	DEK
ENSG00000124802	7.947856304	8.492561507	protein_coding	6	8015959	8102811	EEF1E1
ENSG00000124813	9.203438318	8.880180737	protein_coding	6	45295894	45518818	RUNX2
ENSG00000124827	0	0.390640832	protein_coding	6	10873456	10882174	GCM2
ENSG00000124831	10.65133646	10.20635918	protein_coding	2	238536219	238722325	LRRFIP1
ENSG00000124835	0	0.697730409	antisense	2	237957242	237964810	
ENSG00000124839	8.12275821	7.18590274	protein_coding	2	238482965	238510257	RAB17
ENSG00000124875	8.674235392	10.3431992	protein_coding	4	74702214	74714781	CXCL6
ENSG00000124882	9.079967361	4.075511708	protein_coding	4	75230860	75254468	EREG
ENSG00000124915	0.499066092	0.390640832	lincRNA	11	61513965	61525127	
ENSG00000124920	7.827020104	7.346135265	protein_coding	11	61520114	61555990	C11orf9
ENSG00000124942	13.1218216	12.29895485	protein_coding	11	62201016	62323719	AHNAK
ENSG00000125037	8.197783135	8.603988071	protein_coding	3	10004221	10052800	TMEM111
ENSG00000125046	2.762599152	2.652276565	protein_coding	3	8661086	8786726	C3orf32
ENSG00000125084	0.499066092	0	protein_coding	12	49372398	49375459	WNT1
ENSG00000125089	8.382430368	7.931818465	protein_coding	4	8183799	8242830	SH3TC1
ENSG00000125107	11.45560255	11.44933421	protein_coding	16	58553855	58663790	CNOT1
ENSG00000125122	4.131913373	4.7279204	protein_coding	16	67241042	67260951	LRRC29
ENSG00000125124	8.53328596	9.388489822	protein_coding	16	56500748	56554195	BBS2
ENSG00000125144	0.499066092	0.390640832	protein_coding	16	56700643	56701977	MT1G
ENSG00000125148	11.36038867	12.73346486	protein_coding	16	56642111	56643409	MT2A
ENSG00000125149	8.225937332	8.468698482	protein_coding	16	67143861	67182442	C16orf70
ENSG00000125166	11.22196453	11.47887128	protein_coding	16	58741035	58768261	GOT2
ENSG00000125170	6.386460534	6.708116507	protein_coding	16	57505863	57521239	DOK4
ENSG00000125245	0	1.16600992	protein_coding	13	99906968	99913998	GPR18
ENSG00000125246	5.967663407	5.855704078	protein_coding	13	100258919	100549387	CLYBL
ENSG00000125247	7.093410923	7.32390599	protein_coding	13	101256181	101327347	TMTC4
ENSG00000125249	8.197783135	8.083414408	protein_coding	13	98086476	98120244	RAP2A
ENSG00000125257	8.613115289	8.422372441	protein_coding	13	95672083	95953687	ABCC4
ENSG00000125266	9.485492858	8.557113361	protein_coding	13	107142093	107187462	EFNB2
ENSG00000125285	2.928422289	0	protein_coding	13	95361886	95364389	SOX21
ENSG00000125304	10.48656567	10.60380154	protein_coding	13	100153671	100215645	TM9SF2
ENSG00000125319	7.132181536	7.119700263	protein_coding	17	42219274	42239844	C17orf53
ENSG00000125337	5.967663407	5.294620694	protein_coding	6	168396921	168445769	KIF25
ENSG00000125347	9.405104778	9.681313358	protein_coding	5	131817301	131826490	IRF1
ENSG00000125351	7.715131365	8.044860491	protein_coding	X	118967985	118986961	UPF3B
ENSG00000125352	7.281453427	7.900240894	protein_coding	X	119004497	119005791	RNF113A
ENSG00000125354	9.985245743	9.862835714	protein_coding	X	118749687	118827333	6-sep
ENSG00000125355	3.652182994	3.081239798	protein_coding	X	119392505	119445411	FAM70A
ENSG00000125356	9.021506037	9.906963845	protein_coding	X	119005450	119010625	NDUFA1
ENSG00000125375	5.741167503	5.759604001	protein_coding	14	50779044	50802276	ATP5S
ENSG00000125378	5.649112675	6.023720496	protein_coding	14	54416454	54425479	BMP4
ENSG00000125384	0.499066092	0.390640832	protein_coding	14	52781023	52795324	PTGER2
ENSG00000125386	8.276809264	8.375860541	protein_coding	4	2626988	2734292	FAM193A
ENSG00000125388	5.346128795	5.886365696	protein_coding	4	2965335	3042474	GRK4
ENSG00000125398	10.38231481	9.730728943	protein_coding	17	70117161	70122561	SOX9
ENSG00000125409	2.238690726	2.652276565	protein_coding	17	15207128	15244958	TEKT3

ENSG00000125414	0	0.390640832	protein_coding	17	10368772	10453274	MYH2
ENSG00000125430	7.058051099	6.827478203	protein_coding	17	14204400	14252721	HS3ST3B1
ENSG00000125434	6.897280837	6.762847836	protein_coding	17	8191082	8198661	SLC25A35
ENSG00000125445	9.245304163	9.55579319	protein_coding	17	73257749	73262456	MRPS7
ENSG00000125447	9.142495894	9.344309385	protein_coding	17	73232695	73258444	GGA3
ENSG00000125449	6.585843317	6.223059835	protein_coding	17	73106082	73126360	ARMC7
ENSG00000125450	9.419108071	9.52932336	protein_coding	17	73201597	73231854	NUP85
ENSG00000125454	8.215945216	8.377210474	protein_coding	17	73269062	73285530	SLC25A19
ENSG00000125457	6.634666104	6.686500471	protein_coding	17	73262313	73267303	MIF4GD
ENSG00000125458	7.974178365	8.418443943	protein_coding	17	73126324	73127877	NT5C
ENSG00000125459	7.250461488	7.922602475	protein_coding	1	155579979	155718153	MSTO1
ENSG00000125462	7.776245808	7.977035488	protein_coding	1	156374044	156400493	C1orf61
ENSG00000125482	7.481543613	7.201227369	protein_coding	9	135251008	135282213	TTF1
ENSG00000125484	10.35252484	10.05811545	protein_coding	9	135545422	135570342	GTF3C4
ENSG00000125485	8.327805238	8.351343215	protein_coding	9	135468384	135545788	DDX31
ENSG00000125492	0.499066092	0.390640832	protein_coding	9	135457572	135465653	BARHL1
ENSG00000125503	9.902936511	10.4325864	protein_coding	19	55602283	55628927	PPP1R12C
ENSG00000125505	11.71738313	12.05013478	protein_coding	19	54677107	54693733	MBOAT7
ENSG00000125508	2.57521082	1.667587519	protein_coding	20	62172163	62178857	SRMS
ENSG00000125510	4.6658819	3.453019579	protein_coding	20	62711526	62731996	OPRL1
ENSG00000125520	10.01724335	9.51467792	protein_coding	20	62369623	62374858	SLC2A4RG
ENSG00000125531	3.502389126	1.802319292	protein_coding	20	62184373	62188061	C20orf195
ENSG00000125534	10.82342579	10.31846698	protein_coding	20	62152077	62153559	PPDPF
ENSG00000125538	5.286269488	3.64455972	protein_coding	2	113587328	113594480	IL1B
ENSG00000125551	0.869158192	0.950786998	protein_coding	2	88045917	88058312	PLGLB2
ENSG00000125571	0.499066092	0.950786998	protein_coding	2	113670548	113676459	IL37
ENSG00000125611	7.581430769	7.485338407	protein_coding	2	113341817	113346852	CHCHD5
ENSG00000125618	7.182306324	7.182818183	protein_coding	2	113973574	114036527	PAX8
ENSG00000125629	8.077121473	8.122575449	protein_coding	2	118846028	118868573	INSIG2
ENSG00000125630	9.784207642	9.489933546	protein_coding	2	113299492	113334673	POLR1B
ENSG00000125631	1.407729925	0.697730409	pseudogene	2	118617003	118692518	HTR5BP
ENSG00000125633	9.170704638	9.233151934	protein_coding	2	118673054	118771709	CCDC93
ENSG00000125637	8.009315251	6.963107483	protein_coding	2	113914902	113960814	PSD4
ENSG00000125648	11.46406982	10.89612464	protein_coding	19	6436093	6459781	SLC25A23
ENSG00000125650	4.322773689	3.748356452	protein_coding	19	6375159	6377338	PSPN
ENSG00000125651	10.67742157	10.64697018	protein_coding	19	6379580	6393992	GTF2F1
ENSG00000125652	8.373468605	8.848412964	protein_coding	19	6372444	6375261	ALKBH7
ENSG00000125656	10.3009932	10.44132471	protein_coding	19	6361463	6368915	CLPP
ENSG00000125657	9.12871464	8.591248608	protein_coding	19	6531010	6535939	TNFSF9
ENSG00000125675	0.499066092	0	protein_coding	X		122318006	122624766	GRIA3
ENSG00000125676	9.911010438	9.930142285	protein_coding	X		122734412	122866906	THOC2
ENSG00000125686	9.588992469	9.379087693	protein_coding	17	37560538	37607521	MED1
ENSG00000125691	11.19194892	11.78885999	protein_coding	17	37004118	37010064	RPL23
ENSG00000125695	8.229914881	8.774525884	protein_coding	17	61780196	61819330	STRADA
ENSG00000125703	6.676071387	6.746227492	protein_coding	1	63249806	63339980	ATG4C
ENSG00000125726	9.228504544	9.479915728	protein_coding	19	6583135	6591163	CD70
ENSG00000125730	14.2767362	14.582944	protein_coding	19	6677846	6730573	C3
ENSG00000125731	9.533752964	8.11126287	protein_coding	19	6752211	6767523	SH2D3A
ENSG00000125733	10.92198671	11.08599454	protein_coding	19	6739691	6751537	TRIP10
ENSG00000125734	9.80834902	10.22372209	protein_coding	19	6730066	6737614	GPR108
ENSG00000125735	6.283060568	6.104183162	protein_coding	19	6663148	6670599	TNFSF14
ENSG00000125740	9.051032802	2.420525079	protein_coding	19	45971253	45978414	FOSB
ENSG00000125741	7.943018447	7.541804862	protein_coding	19	46030685	46105470	OPA3
ENSG00000125743	9.998712996	10.80853254	protein_coding	19	46190713	46195443	SNRPD2
ENSG00000125744	6.212449006	5.611474642	protein_coding	19	45988550	46000313	RTN2
ENSG00000125746	7.648344073	7.04352744	protein_coding	19	46112660	46148726	EML2
ENSG00000125753	9.173805166	8.817919521	protein_coding	19	46010688	46030236	VASP
ENSG00000125755	10.24608033	10.04713217	protein_coding	19	46318693	46366548	SYMPK
ENSG00000125772	7.663128351	8.032919217	protein_coding	20	5525081	5591672	GPCPD1
ENSG00000125775	3.950976309	4.622587519	protein_coding	20	1290619	1309883	SDCBP2
ENSG00000125779	9.021506037	9.317136766	protein_coding	20	3869486	3907605	PANK2
ENSG00000125780	1.407729925	1.518964905	protein_coding	20	2276647	2321724	TGM3
ENSG00000125787	1.407729925	2.334191469	protein_coding	20	3024268	3026393	GNRH2

ENSG00000125798	6.103936836	0.697730409	protein_coding	20	22561643	22566093	FOXA2
ENSG00000125804	5.286269488	5.496926774	protein_coding	20	26035291	26073683	FAM182A
ENSG00000125810	0	0.390640832	protein_coding	20	23059986	23066977	CD93
ENSG00000125812	8.164919416	8.177844832	protein_coding	20	23342787	23353700	GZF1
ENSG00000125814	7.850461307	8.431497548	protein_coding	20	23355159	23402125	NAPB
ENSG00000125817	11.10230876	11.13329882	protein_coding	20	3764498	3767337	CENPB
ENSG00000125818	11.21320105	11.16744486	protein_coding	20	1093906	1149022	PSMF1
ENSG00000125821	9.323698603	9.445582628	protein_coding	20	18568537	18744561	DTD1
ENSG00000125826	10.8899802	11.25210284	protein_coding	20	388142	411610	RBCK1
ENSG00000125827	8.803966282	9.708915586	protein_coding	20	7957995	8000476	TMX4
ENSG00000125834	10.1124219	9.869085912	protein_coding	20	2082257	2157684	STK35
ENSG00000125835	11.43787282	11.96538831	protein_coding	20	2442280	2451499	SNRPB
ENSG00000125841	9.751377591	9.890987074	protein_coding	20	327527	335512	NRSN2
ENSG00000125843	8.057112115	8.093304774	protein_coding	20	3801178	3805949	C20orf29
ENSG00000125844	12.12164897	12.19810991	protein_coding	20	17594323	17662940	RRBP1
ENSG00000125845	8.901951857	8.755966294	protein_coding	20	6748311	6760910	BMP2
ENSG00000125846	7.858191159	8.349968848	protein_coding	20	18269121	18297640	ZNF133
ENSG00000125848	3.652182994	0.697730409	protein_coding	20	14303634	14318262	FLRT3
ENSG00000125850	4.409500985	0	protein_coding	20	17937623	18039832	OVOL2
ENSG00000125863	9.749300695	10.11059465	protein_coding	20	10385832	10414870	MKKS
ENSG00000125864	4.712111592	5.112089758	protein_coding	20	17474550	17549865	BFSP1
ENSG00000125868	11.20816934	11.53210637	protein_coding	20	17550508	17588887	DSTN
ENSG00000125870	8.965354743	9.434602747	protein_coding	20	16710606	16722421	SNRPB2
ENSG00000125871	8.772951555	8.848412964	protein_coding	20	17949556	17971765	C20orf72
ENSG00000125872	1.407729925	1.353254395	protein_coding	20	6021424	6034695	LRRN4
ENSG00000125875	9.559268022	10.12874091	protein_coding	20	416124	443197	TBC1D20
ENSG00000125877	9.367686069	9.786150016	protein_coding	20	3189514	3204516	ITPA
ENSG00000125885	9.429522058	9.565299431	protein_coding	20	5931298	5975852	MCM8
ENSG00000125895	3.274897671	3.279072565	protein_coding	20	1161205	1166059	TMEM74B
ENSG00000125898	7.773523328	7.340609974	protein_coding	20	814358	826922	FAM110A
ENSG00000125900	0.499066092	0.390640832	protein_coding	20	1514897	1539489	SIRPD
ENSG00000125901	9.553725034	10.03307035	protein_coding	20	3026591	3028900	MRPS26
ENSG00000125910	3.335220907	3.081239798	protein_coding	19	3178736	3180329	S1PR4
ENSG00000125912	11.51140672	11.37965236	protein_coding	19	3185875	3209572	NCLN
ENSG00000125931	0.499066092	0.950786998	protein_coding	X	71521488	71527037	CITED1
ENSG00000125944	10.9613953	10.76845662	protein_coding	1	23630264	23670829	HNRNPR
ENSG00000125945	6.856698814	7.219404604	protein_coding	1	23685941	23695935	ZNF436
ENSG00000125952	9.807683852	9.965124308	protein_coding	14	65472892	65569413	MAX
ENSG00000125962	6.032862302	4.961837817	protein_coding	X	101854096	101969594	ARMCX5
ENSG00000125965	2.35979773	2.788380093	protein_coding	20	34021145	34042568	GDF5
ENSG00000125966	9.121239074	9.902751889	protein_coding	20	33814457	33864804	MMP24
ENSG00000125967	8.724437638	8.480051236	protein_coding	20	32244893	32262269	NECAB3
ENSG00000125968	9.817629308	9.087365757	protein_coding	20	30193086	30194318	ID1
ENSG00000125970	11.2591741	11.21936033	protein_coding	20	32581452	32696114	RALY
ENSG00000125971	10.08083775	10.44583493	protein_coding	20	33104214	33128762	DYNLRB1
ENSG00000125977	10.22730153	10.43680034	protein_coding	20	32676104	32700138	EIF2S2
ENSG00000125991	11.00556812	11.74103524	protein_coding	20	34129770	34145405	ERGIC3
ENSG00000125995	8.931561329	10.01453929	protein_coding	20	34287194	34288906	ROMO1
ENSG00000125997	1.407729925	1.16600992	pseudogene	20	31935526	31942250	BPIFB9P
ENSG00000125998	0.869158192	0.390640832	protein_coding	20	33873534	33880204	FAM83C
ENSG00000126001	10.51373727	10.96440245	protein_coding	20	34042985	34099804	CEP250
ENSG00000126003	9.0577617	9.068270303	protein_coding	20	30780306	30795594	PLAGL2
ENSG00000126005	8.631273684	9.457118017	antisense	20	33804634	33865934	MT1P3
ENSG00000126010	1.16343121	1.667587519	protein_coding	X	16141679	16171144	GRPR
ENSG00000126012	10.93818348	11.22518166	protein_coding	X	53220503	53254604	KDM5C
ENSG00000126016	3.989024711	3.493416095	protein_coding	X	112017731	112084043	AMOT
ENSG00000126062	8.523597116	8.709567138	protein_coding	3	50392180	50397041	TMEM115
ENSG00000126067	10.53800235	10.49080191	protein_coding	1	36067185	36107445	PSMB2
ENSG00000126070	7.04004079	7.373448694	protein_coding	1	36396319	36522063	EIF2C3
ENSG00000126088	9.354083505	10.28325773	protein_coding	1	45477819	45481247	UROD
ENSG00000126091	7.04004079	7.405556541	protein_coding	1	44171495	44396831	ST3GAL3
ENSG00000126106	5.695874189	5.404622322	protein_coding	1	45100910	45140227	TMEM53
ENSG00000126107	9.349520666	9.379761309	protein_coding	1	45468212	45477001	HECTD3

ENSG00000126214	8.695964893	7.977035488	protein_coding	14	104028233	104167888	KLC1
ENSG00000126215	9.818950204	9.053992028	protein_coding	14	104163946	104181841	XRCC3
ENSG00000126216	8.342575358	8.031205224	protein_coding	13	113139325	113242481	TUBGCP3
ENSG00000126217	5.255381949	1.16600992	protein_coding	13	113548692	113754053	MCF2L
ENSG00000126218	0.869158192	1.518964905	protein_coding	13	113777128	113803843	F10
ENSG00000126226	8.705994399	8.46235258	protein_coding	13	113831891	113863029	PCID2
ENSG00000126231	2.57521082	2.334191469	protein_coding	13	113812968	113826694	PROZ
ENSG00000126233	0	0.390640832	protein_coding	8	143822362	143823829	SLURP1
ENSG00000126243	8.146099194	7.801136513	protein_coding	19	36428022	36436095	LRFN3
ENSG00000126246	4.6658819	5.125003832	protein_coding	19	36230153	36233351	IGFLR1
ENSG00000126247	11.27056274	11.2337767	protein_coding	19	36630918	36641254	CAPNS1
ENSG00000126249	7.31554162	6.984522608	protein_coding	19	34895303	34917070	PDCD2L
ENSG00000126254	9.863155878	9.55340681	protein_coding	19	36119980	36128586	RBM42
ENSG00000126259	2.10647801	0.390640832	protein_coding	19	36347810	36358048	KIRREL2
ENSG00000126261	9.940436455	9.908365102	protein_coding	19	34919264	34960795	UBA2
ENSG00000126262	0.869158192	1.16600992	protein_coding	19	35940617	35942667	FFAR2
ENSG00000126264	1.16343121	1.353254395	protein_coding	19	36393382	36395173	HCST
ENSG00000126267	9.421718623	9.680766824	protein_coding	19	36139125	36149686	COX6B1
ENSG00000126351	8.494133947	7.616862529	protein_coding	17	38218446	38250120	THRA
ENSG00000126353	0.869158192	0.390640832	protein_coding	17	38710021	38721724	CCR7
ENSG00000126368	7.285281023	6.762847836	protein_coding	17	38249040	38256978	NR1D1
ENSG00000126391	9.032934112	8.696649474	protein_coding	11	65154070	65180996	FRMD8
ENSG00000126432	10.57638764	10.97402535	protein_coding	11	64085560	64089283	PRDX5
ENSG00000126453	8.38421606	8.457255683	protein_coding	19	50168399	50177172	BCL2L12
ENSG00000126456	9.147761519	8.929781562	protein_coding	19	50162829	50169132	IRF3
ENSG00000126457	11.64531434	11.52785834	protein_coding	19	50179043	50192286	PRMT1
ENSG00000126458	8.774313977	8.919626348	protein_coding	19	50138552	50143400	RRAS
ENSG00000126460	4.517578978	4.202780776	protein_coding	19	50084587	50094265	PRRG2
ENSG00000126461	10.38410065	10.10571686	protein_coding	19	50145382	50161905	SCAF1
ENSG00000126464	9.532948302	9.375039375	protein_coding	19	50094912	50129695	PRR12
ENSG00000126467	3.554067925	3.748356452	protein_coding	19	50243018	50266543	TSKS
ENSG00000126500	5.417620896	5.137803333	protein_coding	11	63870660	63886645	FLRT1
ENSG00000126522	8.853790788	8.386624806	protein_coding	7	65540785	65558545	ASL
ENSG00000126524	9.577332188	9.49491649	protein_coding	7	66452664	66460635	SBDS
ENSG00000126550	0	0.390640832	protein_coding	4	70916119	70924562	HTN1
ENSG00000126561	7.784382559	8.508664347	protein_coding	17	40439565	40463961	STAT5A
ENSG00000126562	6.956090577	7.295628905	protein_coding	17	40932649	40949061	WNK4
ENSG00000126581	8.331511968	8.344458247	protein_coding	17	40962150	40976310	BECN1
ENSG00000126583	4.026095388	3.96493948	protein_coding	19	54382444	54410906	PRKCG
ENSG00000126602	11.52522921	11.57052385	protein_coding	16	3708038	3767565	TRAP1
ENSG00000126603	8.349904097	7.650745242	protein_coding	16	4364762	4389598	GLIS2
ENSG00000126653	8.541310587	8.048254208	protein_coding	17	28443799	28513493	NSRP1
ENSG00000126698	9.637710578	9.858492738	protein_coding	1	28527068	28559536	DNAJC8
ENSG00000126705	9.564002263	9.317839931	protein_coding	1	27860546	27930942	AHDC1
ENSG00000126709	9.766517491	11.44917376	protein_coding	1	27992572	27998729	IFI6
ENSG00000126746	9.083268927	8.906597279	protein_coding	12	6775643	6798738	ZNF384
ENSG00000126749	6.94645393	7.012590419	processed_transc	12	7079944	7095921	EMG1
ENSG00000126752	0	0.390640832	protein_coding	X	48114752	48126879	SSX1
ENSG00000126756	8.842144812	9.5858785	protein_coding	X	47511197	47518560	UXT
ENSG00000126759	4.409500985	4.127774132	protein_coding	X	47483612	47489704	CFP
ENSG00000126767	9.704266019	9.782079226	protein_coding	X	47494920	47510003	ELK1
ENSG00000126768	8.682966505	8.882083758	protein_coding	X	48750730	48755426	TIMM17B
ENSG00000126773	10.01436362	10.78939442	protein_coding	14	60558629	60635851	C14orf135
ENSG00000126775	8.219950371	9.065760854	protein_coding	14	55833110	55878576	ATG14
ENSG00000126777	11.26887224	11.63992791	protein_coding	14	56025790	56168244	KTN1
ENSG00000126778	7.23866569	7.307006333	protein_coding	14	61110132	61124977	SIX1
ENSG00000126785	0.499066092	0	protein_coding	14	63670832	63759937	RHOJ
ENSG00000126787	9.228504544	9.488060496	protein_coding	14	55614830	55658396	DLGAP5
ENSG00000126790	8.337054268	8.877321491	protein_coding	14	59927081	59951148	C14orf149
ENSG00000126803	8.672775055	9.004186147	protein_coding	14	65002623	65009955	HSPA2
ENSG00000126804	9.004192374	9.322752514	protein_coding	14	64970430	65000408	ZBTB1
ENSG00000126814	8.650690744	9.121596633	protein_coding	14	61438169	61448076	TRMT5
ENSG00000126821	9.119096064	9.162099548	protein_coding	14	64150932	64194757	SGPP1

ENSG00000126822	9.972245312	9.764649066	protein_coding	14	65170820	65213610	PLEKHG3
ENSG00000126838	0	0.697730409	protein_coding	12	9301436	9360966	PZP
ENSG00000126856	1.799000381	1.16600992	protein_coding	16	90122974	90158480	PRDM7
ENSG00000126858	8.245716314	7.953699568	protein_coding	17	30469473	30552746	RHOT1
ENSG00000126860	4.734682531	6.25865215	protein_coding	17	29643428	29648767	EVI2A
ENSG00000126861	2.671945279	2.721932731	protein_coding	17	29621665	29624349	OMG
ENSG00000126870	8.33889698	8.601680156	protein_coding	7	158649269	158749438	WDR60
ENSG00000126878	5.255381949	1.518964905	protein_coding	9	133971863	133998539	AIF1L
ENSG00000126882	5.563445526	5.717592784	protein_coding	9	134133463	134151934	FAM78A
ENSG00000126883	10.13539932	9.860907117	protein_coding	9	134000948	134110057	NUP214
ENSG00000126890	0.499066092	0	protein_coding	X	153880246	153881853	CTAG2
ENSG00000126895	3.211941663	2.242360793	protein_coding	X	153167985	153172620	AVPR2
ENSG00000126903	9.120167967	9.319947372	protein_coding	X	153715645	153719016	SLC10A3
ENSG00000126934	12.01245138	12.01739401	protein_coding	19	4090319	4124126	MAP2K2
ENSG00000126945	10.07808233	10.17138273	protein_coding	X	100663207	100669121	HNRNPH2
ENSG00000126947	5.899377978	6.771086752	protein_coding	X	100805514	100809683	ARMCX1
ENSG00000126953	7.258272112	7.871792517	protein_coding	X	100600649	100604184	TIMM8A
ENSG00000127022	12.635844	12.66803498	protein_coding	5	179105629	179157926	CANX
ENSG00000127054	10.23028291	10.22409723	protein_coding	1	1246965	1260071	CPSF3L
ENSG00000127074	0.499066092	0	protein_coding	1	192605275	192629390	RGS13
ENSG00000127080	6.664362065	6.695185789	protein_coding	9	95375466	95432547	IPPK
ENSG00000127081	6.305850085	6.217041467	protein_coding	9	95607874	95640304	ZNF484
ENSG00000127083	1.16343121	1.16600992	protein_coding	9	95176527	95186743	OMD
ENSG00000127084	5.175139844	2.652276565	protein_coding	9	95709733	95798518	FGD3
ENSG00000127124	7.906207969	7.679480043	protein_coding	1	41972036	42501596	HIVEP3
ENSG00000127125	7.556308086	7.568048738	protein_coding	1	42921788	42948411	PPCS
ENSG00000127129	3.603959378	6.554308016	protein_coding	1	41944446	41950344	EDN2
ENSG00000127152	0.499066092	0	protein_coding	14	99635624	99737861	BCL11B
ENSG00000127184	9.76309053	10.11747676	protein_coding	5	85913721	85916779	COX7C
ENSG00000127191	9.417365074	9.474249985	protein_coding	9	139776364	139821059	TRAF2
ENSG00000127220	7.311793575	7.365308824	protein_coding	19	17402941	17414282	ABHD8
ENSG00000127241	1.16343121	0.390640832	protein_coding	3	186935942	187009810	MASP1
ENSG00000127249	0	0.697730409	protein_coding	3	193119866	193310900	ATP13A4
ENSG00000127311	5.869093502	5.995870484	protein_coding	12	66696325	66737423	HELB
ENSG00000127314	7.399163791	7.519971735	protein_coding	12	69004619	69054372	RAP1B
ENSG00000127325	1.16343121	1.353254395	protein_coding	12	70037140	70093256	BEST3
ENSG00000127328	6.912209543	7.659647832	protein_coding	12	70132461	70216984	RAB3IP
ENSG00000127329	3.78787645	4.385599336	protein_coding	12	70910630	71031220	PTPRB
ENSG00000127334	8.458982065	8.301022059	protein_coding	12	68042118	68059186	DYRK2
ENSG00000127337	7.621344476	8.056703738	protein_coding	12	69753483	69784576	YEATS4
ENSG00000127362	0.499066092	1.667587519	protein_coding	7	141463897	141464997	TAS2R3
ENSG00000127364	1.616589159	1.518964905	protein_coding	7	141478242	141479235	TAS2R4
ENSG00000127366	2.671945279	3.748356452	protein_coding	7	141490017	141491166	TAS2R5
ENSG00000127377	1.16343121	0.390640832	protein_coding	7	151125921	151137899	CRYGN
ENSG00000127399	9.881603942	8.591248608	protein_coding	7	150019728	150035239	LRRC61
ENSG00000127412	0.869158192	0.390640832	protein_coding	7	142605267	142630905	TRPV5
ENSG00000127415	5.958104008	6.577981353	protein_coding	4	980785	998316	IDUA
ENSG00000127418	9.533752964	8.865827498	protein_coding	4	1003724	1020685	FGFRL1
ENSG00000127419	7.726437377	8.029489191	protein_coding	4	926175	952444	TMEM175
ENSG00000127423	6.646617938	6.725179132	protein_coding	1	26158414	26185903	C1orf135
ENSG00000127445	9.670274772	9.979416425	protein_coding	19	9945999	9960365	PIN1
ENSG00000127452	8.678607554	8.397309351	protein_coding	19	9920947	9929731	FBXL12
ENSG00000127463	10.11349877	10.46881022	protein_coding	1	19542158	19578046	KIAA0090
ENSG00000127481	11.61806776	11.72680998	protein_coding	1	19401000	19536770	UBR4
ENSG00000127483	10.25684158	10.42542696	protein_coding	1	21069154	21113816	HP1BP3
ENSG00000127507	7.123655438	6.408769788	protein_coding	19	14843205	14889353	EMR2
ENSG00000127511	10.05192082	10.13792826	protein_coding	19	16940218	16991164	SIN3B
ENSG00000127526	10.38900032	10.32127499	protein_coding	19	16661661	16683193	SLC35E1
ENSG00000127527	9.264821181	9.493672366	protein_coding	19	16466220	16582781	EPS15L1
ENSG00000127528	7.943018447	7.176629214	protein_coding	19	16435651	16438337	KLF2
ENSG00000127533	5.879259127	3.411459265	protein_coding	19	16999826	17002830	F2RL3
ENSG00000127540	9.818950204	10.62980078	protein_coding	19	1597154	1605483	UQCR11
ENSG00000127554	7.434062125	7.764467022	protein_coding	16	2034208	2037750	GFER

ENSG00000127561	8.009315251	7.782918269	protein_coding	16	2039661	2044276	SYNGR3
ENSG00000127564	9.832747078	9.898527599	protein_coding	16	3018103	3030505	PKMYT1
ENSG00000127578	2.57521082	2.334191469	protein_coding	16	680932	684116	WFIKKN1
ENSG00000127580	8.54291017	8.590084877	protein_coding	16	734622	740444	WDR24
ENSG00000127585	7.700873103	7.480322213	protein_coding	16	742500	755829	FBXL16
ENSG00000127586	10.1242235	10.77410398	protein_coding	16	838046	850737	CHTF18
ENSG00000127588	2.10647801	2.144285137	protein_coding	16	848041	850733	GNG13
ENSG00000127589	5.806547963	5.855704078	pseudogene	8	30209389	30211034	TUBBP1
ENSG00000127603	11.09087261	11.24472634	protein_coding	1	39546988	39952849	MACF1
ENSG00000127616	13.04220317	12.99755058	protein_coding	19	11071598	11172958	SMARCA4
ENSG00000127663	9.442434712	9.699773395	protein_coding	19	4969124	5153606	KDM4B
ENSG00000127666	9.432113851	9.242070261	protein_coding	19	4815939	4831737	TICAM1
ENSG00000127688	6.449220685	6.619638408	protein_coding	16	81348557	81413940	GAN
ENSG00000127720	4.322773689	4.7279204	protein_coding	12	82752276	82873015	C12orf26
ENSG00000127774	5.360713447	6.392840898	protein_coding	17	3572090	3572962	TMEM93
ENSG00000127804	9.482995289	10.07941968	protein_coding	17	2319352	2415200	METTL16
ENSG00000127824	10.52955592	10.44551324	protein_coding	2	220114433	220142892	TUBA4A
ENSG00000127831	0	0.697730409	protein_coding	2	219283815	219318018	VIL1
ENSG00000127837	10.43796299	10.32931774	protein_coding	2	219128850	219134980	AAMP
ENSG00000127838	9.86187486	9.393168008	protein_coding	2	219135115	219211516	PNKD
ENSG00000127863	4.261916566	4.489113623	protein_coding	13	24144509	24250232	TNFRSF19
ENSG00000127870	8.681514984	8.755966294	protein_coding	13	26706253	26796791	RNF6
ENSG00000127884	10.36614204	10.71794276	protein_coding	10	135175984	135187193	ECHS1
ENSG00000127914	8.725846631	8.747640323	protein_coding	7	91570181	91739987	AKAP9
ENSG00000127920	7.847875458	8.439273392	protein_coding	7	93551011	93555831	GNG11
ENSG00000127922	9.740266097	9.665379195	protein_coding	7	96110938	96339203	SHFM1
ENSG00000127928	1.16343121	0.950786998	protein_coding	7	93220885	93540577	GNGT1
ENSG00000127946	10.00452934	9.537194665	protein_coding	7	75162621	75368280	HIP1
ENSG00000127947	10.51414505	10.2319528	protein_coding	7	77166592	77269388	PTPN12
ENSG00000127948	9.96986896	9.522019224	protein_coding	7	75528518	75616173	POR
ENSG00000127952	8.890689734	8.806942451	protein_coding	7	75625656	75677322	STYXL1
ENSG00000127954	2.847891871	3.493416095	protein_coding	7	87900207	87936206	STEAP4
ENSG00000127955	7.770795701	7.284161038	protein_coding	7	79763271	79848718	GNAI1
ENSG00000127957	4.842525946	4.777836593	pseudogene	7	75132254	75157478	PMS2P3
ENSG00000127980	7.671926738	7.490337219	protein_coding	7	92116334	92157845	PEX1
ENSG00000127989	6.917151646	6.79148052	protein_coding	7	91500243	91510034	MTERF
ENSG00000127990	8.431612753	7.858322159	protein_coding	7	94214542	94285521	SGCE
ENSG00000127993	7.140657543	7.408200217	protein_coding	7	92158087	92167319	RBM48
ENSG00000127995	6.965663283	7.029859489	protein_coding	7	94138531	94186331	CASD1
ENSG00000128000	5.107569429	5.383282767	protein_coding	19	40534167	40562116	ZNF780B
ENSG00000128011	4.689181911	4.250702764	protein_coding	19	39797208	39805976	LRFN1
ENSG00000128016	7.757079669	5.018301917	protein_coding	19	39897487	39900045	ZFP36
ENSG00000128039	7.22677265	7.507697912	protein_coding	4	56212276	56239263	SRD5A3
ENSG00000128040	0	0.390640832	protein_coding	4	57676026	57687908	SPINK2
ENSG00000128045	2.238690726	1.925536307	protein_coding	4	53728457	53733000	RASL11B
ENSG00000128050	11.35014693	11.24620466	protein_coding	4	57301907	57327534	PAICS
ENSG00000128052	1.16343121	1.667587519	protein_coding	4	55944644	55991756	KDR
ENSG00000128059	8.738466022	8.540340653	protein_coding	4	57259528	57301781	PPAT
ENSG00000128159	9.538571537	9.497401522	protein_coding	22	50656118	50683421	TUBGCP6
ENSG00000128165	8.251597515	8.288162655	protein_coding	22	50919985	50924869	ADM2
ENSG00000128185	7.437505974	8.041458772	protein_coding	22	20301799	20307603	DGCR6L
ENSG00000128191	8.764749894	8.9643232	protein_coding	22	20067755	20099400	DGCR8
ENSG00000128203	5.60078458	6.051043053	protein_coding	22	26825239	26840981	ASPHD2
ENSG00000128218	3.502389126	2.579085888	protein_coding	22	24094930	24096655	VPREB3
ENSG00000128228	8.602416377	8.697730375	protein_coding	22	21996550	21998587	SDF2L1
ENSG00000128242	2.928422289	3.32456471	protein_coding	22	30950622	30970574	GAL3ST1
ENSG00000128245	11.78060919	11.85829373	protein_coding	22	32340447	32353590	YWHAH
ENSG00000128253	0	1.518964905	protein_coding	22	32586422	32600718	RFPL2
ENSG00000128254	1.616589159	2.242360793	protein_coding	22	32329507	32341504	C22orf24
ENSG00000128262	1.799000381	1.16600992	pseudogene	22	24647796	24661493	POM121L9P
ENSG00000128266	5.660945913	6.510691757	protein_coding	22	23412540	23467224	GNAZ
ENSG00000128268	1.16343121	2.334191469	protein_coding	22	39853349	39888199	MGAT3
ENSG00000128271	4.409500985	3.87606762	protein_coding	22	24813847	24838328	ADORA2A

ENSG00000128272	11.44366427	11.6521071	protein_coding	22	39915700	39918691	ATF4
ENSG00000128274	8.565120083	7.8660349	protein_coding	22	43088127	43117304	A4GALT
ENSG00000128276	1.799000381	2.334191469	protein_coding	22	32750872	32757148	RFPL3
ENSG00000128283	11.54292278	11.4769838	protein_coding	22	37956454	37965412	CDC42EP1
ENSG00000128284	7.842689815	7.225413142	protein_coding	22	36536372	36562225	APOL3
ENSG00000128285	2.762599152	2.039052734	protein_coding	22	41074754	41078818	MCHR1
ENSG00000128294	8.565120083	9.124010896	protein_coding	22	26921458	26992681	TPST2
ENSG00000128298	3.911897206	3.993387124	protein_coding	22	38480896	38506677	BAIAP2L2
ENSG00000128309	9.866353456	10.0752671	protein_coding	22	37415676	37425863	MPST
ENSG00000128310	0	0.390640832	protein_coding	22	38219389	38221502	GALR3
ENSG00000128311	8.685865174	8.887777807	protein_coding	22	37406900	37415681	TST
ENSG00000128335	9.64070152	9.774415396	protein_coding	22	36622256	36636000	APOL2
ENSG00000128340	10.28435524	9.957022333	protein_coding	22	37621301	37640488	RAC2
ENSG00000128342	11.46005406	11.71951137	protein_coding	22	30636436	30642840	LIF
ENSG00000128346	6.94645393	6.1803943	protein_coding	22	38339528	38349676	C22orf23
ENSG00000128383	0.499066092	0	protein_coding	22	39348746	39359188	APOBEC3A
ENSG00000128394	7.792473675	7.213370937	protein_coding	22	39436609	39449915	APOBEC3F
ENSG00000128408	6.320845526	6.495854971	protein_coding	22	45809572	45828376	RIBC2
ENSG00000128422	7.781675404	9.226426823	protein_coding	17	39775689	39781094	KRT17
ENSG00000128438	0	0.390640832	pseudogene	17	16826229	16838153	TBC1D27
ENSG00000128463	9.072234198	9.129628513	protein_coding	15	34517200	34522357	TMEM85
ENSG00000128482	4.999881834	5.506827567	protein_coding	17	19314507	19320589	RNF112
ENSG00000128487	10.13168366	10.68371137	protein_coding	17	19912614	20222339	SPECC1
ENSG00000128510	9.820929282	10.26039601	protein_coding	7	129932974	129964020	CPA4
ENSG00000128512	8.484177644	8.66273344	protein_coding	7	111366168	111846466	DOCK4
ENSG00000128513	8.408986408	8.323603978	protein_coding	7	124462440	124570037	POT1
ENSG00000128524	9.077762113	9.281534232	protein_coding	7	128502880	128505898	ATP6V1F
ENSG00000128534	8.099033379	8.129000175	protein_coding	7	117824086	117832878	NAA38
ENSG00000128536	3.554067925	2.039052734	protein_coding	7	105517242	105676877	CDHR3
ENSG00000128563	8.101206377	7.964516916	protein_coding	7	102004319	102067123	PRKRIP1
ENSG00000128564	4.642199401	4.990346085	protein_coding	7	100805790	100808874	VGF
ENSG00000128567	11.32410363	10.6583965	protein_coding	7	131185021	131242976	PODXL
ENSG00000128573	0.499066092	0.697730409	protein_coding	7	113726382	114333827	FOXP2
ENSG00000128578	8.941297632	8.397309351	protein_coding	7	129074274	129128240	FAM40B
ENSG00000128581	7.928406768	7.556178852	protein_coding	7	100956975	100965104	RABL5
ENSG00000128585	9.332973914	9.196916881	protein_coding	7	130794855	131181395	MKLN1
ENSG00000128590	7.792473675	8.034631177	protein_coding	7	108210012	108215294	DNAJB9
ENSG00000128591	5.613019223	4.693656882	protein_coding	7	128470431	128499328	FLNC
ENSG00000128594	2.762599152	2.788380093	protein_coding	7	127667124	127672160	LRRC4
ENSG00000128595	12.00013163	11.8880726	protein_coding	7	128379346	128411861	CALU
ENSG00000128596	5.485736881	3.993387124	protein_coding	7	128430811	128462186	CCDC136
ENSG00000128604	5.485736881	1.802319292	protein_coding	7	128577666	128590089	IRF5
ENSG00000128606	1.799000381	1.518964905	protein_coding	7	102553438	102585396	LRRC17
ENSG00000128607	8.580777545	8.273738696	protein_coding	7	129710350	129773596	KLHDC10
ENSG00000128609	8.528449672	8.228085371	protein_coding	7	123177051	123198309	NDUFA5
ENSG00000128617	4.097495944	3.813624741	protein_coding	7	128412545	128415844	OPN1SW
ENSG00000128626	8.400188597	8.761145731	protein_coding	19	39421348	39423657	MRPS12
ENSG00000128641	11.18658661	10.61500149	protein_coding	2	192109911	192290115	MYO1B
ENSG00000128645	0	1.16600992	protein_coding	2	177053307	177055688	HOXD1
ENSG00000128652	3.393122761	3.935919592	protein_coding	2	177001340	177039577	HOXD3
ENSG00000128654	8.205883411	7.985911283	protein_coding	2	177134123	177202753	MTX2
ENSG00000128655	1.799000381	2.039052734	protein_coding	2	178492797	178973081	PDE11A
ENSG00000128656	6.343050432	4.857374129	protein_coding	2	175664091	175870097	CHN1
ENSG00000128683	6.320845526	7.690381265	protein_coding	2	171669723	171717661	GAD1
ENSG00000128692	2.762599152	3.133298822	pseudogene	2	171608221	171609562	EIF2S2P4
ENSG00000128694	7.07583934	6.387491945	protein_coding	2	190611386	190627953	OSGEPL1
ENSG00000128699	8.247679379	8.344458247	protein_coding	2	190635049	190649097	ORMDL1
ENSG00000128708	9.319971717	9.328346488	protein_coding	2	172778958	172848599	HAT1
ENSG00000128710	0.499066092	0	protein_coding	2	176973518	176984670	HOXD10
ENSG00000128713	0.499066092	0	protein_coding	2	176968944	176974722	HOXD11
ENSG00000128731	9.870818191	9.870045082	protein_coding	15	28356186	28567298	HERC2
ENSG00000128739	4.097495944	4.202780776	protein_coding	15	25068794	25223870	SNRPN
ENSG00000128789	8.094677541	8.234057939	protein_coding	18	12702425	12725739	PSMG2

ENSG00000128791	9.349520666	9.140003549	protein_coding	18	9334765	9402418	TWSG1
ENSG00000128805	8.272959095	7.881337775	protein_coding	10	49654077	49864310	ARHGAP22
ENSG00000128829	9.487986111	9.138412238	protein_coding	15	40226347	40327797	EIF2AK4
ENSG00000128833	5.785082014	4.17820932	protein_coding	15	52484519	52587995	MYO5C
ENSG00000128849	7.165790811	7.472765069	protein_coding	15	57668165	57842925	CGNL1
ENSG00000128872	5.672682881	6.664555628	protein_coding	15	52043758	52108565	TMOD2
ENSG00000128881	7.819120959	7.858322159	protein_coding	15	43030932	43213007	TTBK2
ENSG00000128886	1.407729925	1.518964905	protein_coding	15	44064798	44069741	ELL3
ENSG00000128891	6.825495146	6.45552765	protein_coding	15	40820882	40857256	C15orf57
ENSG00000128908	9.633963159	9.351881826	protein_coding	15	41267889	41408552	INO80
ENSG00000128915	8.730065371	8.89250574	protein_coding	15	60711808	60771359	NARG2
ENSG00000128917	1.16343121	1.518964905	protein_coding	15	41221538	41231237	DLL4
ENSG00000128918	2.35979773	2.334191469	protein_coding	15	58245622	58790065	ALDH1A2
ENSG00000128923	7.356139979	7.161039629	protein_coding	15	59063391	59154099	FAM63B
ENSG00000128928	9.807683852	9.351195062	protein_coding	15	40697686	40728146	IVD
ENSG00000128944	8.742648075	8.684705808	protein_coding	15	40674922	40686446	C15orf23
ENSG00000128951	9.294560225	9.504213373	protein_coding	15	48623208	48635570	DUT
ENSG00000128965	6.96088487	7.652976045	protein_coding	15	41245160	41248710	CHAC1
ENSG00000128973	9.286944125	9.224928086	protein_coding	15	68499330	68549549	CLN6
ENSG00000128989	10.26413304	10.18532111	protein_coding	15	52839242	52862080	ARPP19
ENSG00000129003	9.029515185	9.280091692	protein_coding	15	62144588	62352672	VPS13C
ENSG00000129007	7.053569556	6.50081754	protein_coding	15	68483043	68498417	CALML4
ENSG00000129009	3.448790144	2.652276565	protein_coding	15	74466012	74469213	ISLR
ENSG00000129028	5.331395195	5.683080141	protein_coding	15	71173681	71185124	THAP10
ENSG00000129038	4.230497448	4.101879561	protein_coding	15	74218330	74244478	LOXL1
ENSG00000129048	1.616589159	0.697730409	protein_coding	3	132316081	132337811	CCRL1
ENSG00000129055	8.245716314	8.176294739	protein_coding	3	134196548	134205558	ANAPC13
ENSG00000129071	8.550881589	8.524589435	protein_coding	3	129149787	129158878	MBD4
ENSG00000129083	10.85724747	11.01188767	protein_coding	11	14464986	14521573	COPB1
ENSG00000129084	8.492479328	8.939865791	protein_coding	11	14515329	14665181	PSMA1
ENSG00000129103	11.23216308	10.68425679	protein_coding	7	56131695	56148363	SUMF2
ENSG00000129116	11.2999484	12.09564019	protein_coding	4	169418217	169849608	PALLD
ENSG00000129128	10.14227455	10.32862015	protein_coding	4	177241115	177253396	SPCS3
ENSG00000129158	6.428602508	6.948652068	protein_coding	11	17809595	18034709	SERGEF
ENSG00000129159	0.869158192	1.16600992	protein_coding	11	17756359	17804602	KCNC1
ENSG00000129167	2.35979773	3.411459265	protein_coding	11	18039111	18063973	TPH1
ENSG00000129170	1.616589159	2.242360793	protein_coding	11	19203578	19232120	CSRP3
ENSG00000129173	7.222786417	7.454976247	protein_coding	11	19245610	19263389	E2F8
ENSG00000129187	9.238410348	9.442361917	protein_coding	4	183811213	183839089	DCTD
ENSG00000129194	4.097495944	4.15321211	protein_coding	17	7491498	7493488	SOX15
ENSG00000129195	8.550881589	9.517741338	protein_coding	17	6347761	6354382	FAM64A
ENSG00000129197	7.855619141	8.75700367	protein_coding	17	5322961	5336340	RPAIN
ENSG00000129204	4.026095388	4.761389014	protein_coding	17	5019733	5078329	USP6
ENSG00000129214	1.16343121	2.501982735	protein_coding	17	7531287	7536701	SHBG
ENSG00000129219	9.56636357	9.614204774	protein_coding	17	4710391	4726727	PLD2
ENSG00000129221	0	0.697730409	protein_coding	17	6327057	6338519	AIPL1
ENSG00000129226	10.26413304	10.88074071	protein_coding	17	7482922	7485431	CD68
ENSG00000129235	8.426422697	9.446868902	protein_coding	17	6544078	6547920	TXNDC17
ENSG00000129244	1.16343121	1.353254395	protein_coding	17	7554254	7561087	ATP1B2
ENSG00000129245	10.07532163	10.62951761	protein_coding	17	7494548	7518215	FXR2
ENSG00000129250	11.78331834	12.13157854	protein_coding	17	4901243	4931696	KIF1C
ENSG00000129255	9.536164262	10.54765864	protein_coding	17	7486974	7496107	MPDU1
ENSG00000129270	7.049074049	0.950786998	protein_coding	17	34092876	34122640	MMP28
ENSG00000129277	0.499066092	0	protein_coding	17	34431220	34433014	CCL4
ENSG00000129282	6.171673665	5.974624386	protein_coding	17	34958001	34965397	MRM1
ENSG00000129292	7.222786417	7.527286218	protein_coding	8	133787618	133861052	PHF20L1
ENSG00000129295	1.407729925	0.390640832	protein_coding	8	133584320	133687838	LRRC6
ENSG00000129315	8.883132512	8.868709593	protein_coding	12	49082247	49110681	CCNT1
ENSG00000129317	7.578314254	7.737391576	protein_coding	12	44122410	44152620	PUS7L
ENSG00000129347	10.37963191	10.58844036	protein_coding	19	10663761	10676713	KRI1
ENSG00000129351	13.15645415	13.1238483	protein_coding	19	10764937	10803093	ILF3
ENSG00000129353	10.80054076	10.34250829	protein_coding	19	10713121	10755235	SLC44A2
ENSG00000129354	7.084651883	1.518964905	protein_coding	19	10683348	10697992	AP1M2

ENSG00000129355	6.290697143	6.490875274	protein_coding	19	10677139	10679655	CDKN2D
ENSG00000129422	8.981959173	9.350507971	protein_coding	8	17501304	17658426	MTUS1
ENSG00000129437	3.393122761	3.081239798	protein_coding	19	51580574	51587502	KLK14
ENSG00000129451	5.512107582	0.950786998	protein_coding	19	51516001	51523431	KLK10
ENSG00000129455	4.131913373	0	protein_coding	19	51499264	51504958	KLK8
ENSG00000129460	7.865879816	8.46616347	protein_coding	14	23938897	23979071	NGDN
ENSG00000129465	0.869158192	0.950786998	protein_coding	14	24805227	24809251	RIPK3
ENSG00000129467	1.16343121	1.353254395	protein_coding	14	24787555	24804299	ADCY4
ENSG00000129472	7.593829938	7.485338407	protein_coding	14	21927179	21945132	RAB2B
ENSG00000129473	8.649206367	9.190020311	protein_coding	14	23767999	23780968	BCL2L2
ENSG00000129474	11.10555965	11.30731878	protein_coding	14	23440383	23451851	AJUBA
ENSG00000129480	7.136425764	7.225413142	protein_coding	14	31915242	31926716	C14orf126
ENSG00000129484	8.950968668	9.336002957	protein_coding	14	20811741	20826064	PARP2
ENSG00000129493	7.842689815	8.234057939	protein_coding	14	31760994	31889788	HEATR5A
ENSG00000129514	2.10647801	1.353254395	protein_coding	14	38059189	38069245	FOXA1
ENSG00000129515	9.830784156	10.08768916	protein_coding	14	35030300	35099389	SNX6
ENSG00000129518	7.680671792	8.329194658	protein_coding	14	34985135	35008916	EAPP
ENSG00000129521	8.872994239	10.53319908	protein_coding	14	34393437	34931980	EGLN3
ENSG00000129534	8.398422578	8.976843446	protein_coding	14	45672393	45722743	MIS18BP1
ENSG00000129535	2.10647801	3.411459265	protein_coding	14	24549316	24584223	NRL
ENSG00000129538	5.375152132	0.950786998	protein_coding	14	21269387	21271437	RNASE1
ENSG00000129559	8.303476525	8.942603846	protein_coding	14	24686058	24701660	NEDD8
ENSG00000129562	10.04910955	10.48830548	protein_coding	14	23033805	23058175	DAD1
ENSG00000129566	8.944931872	9.379087693	protein_coding	14	20833826	20881588	TEP1
ENSG00000129595	1.407729925	1.353254395	protein_coding	5	111478138	111755013	EPB41L4A
ENSG00000129596	0.499066092	0.390640832	protein_coding	5	115140430	115152651	CDO1
ENSG00000129625	9.448421292	9.188483253	protein_coding	5	112212084	112258236	REEP5
ENSG00000129636	8.429884808	9.147933883	protein_coding	16	47188298	47498060	ITFG1
ENSG00000129646	4.883502971	5.383282767	protein_coding	17	74270130	74303761	QRICH2
ENSG00000129654	7.169937448	0.697730409	protein_coding	17	74132425	74137380	FOXJ1
ENSG00000129657	10.1782276	9.817812307	protein_coding	17	75084831	75213183	SEC14L1
ENSG00000129667	10.09071393	9.742822827	protein_coding	17	74466976	74497508	RHBDF2
ENSG00000129673	4.464551814	3.081239798	protein_coding	17	74449433	74466198	AANAT
ENSG00000129675	2.928422289	3.279072565	protein_coding	X	135747706	135864247	ARHGEF6
ENSG00000129680	9.107252267	9.242070261	protein_coding	X	135295381	135338641	MAP7D3
ENSG00000129682	4.292665995	3.64455972	protein_coding	X	137713735	138304939	FGF13
ENSG00000129691	8.257454839	8.652735634	protein_coding	8	37962760	38001594	ASH2L
ENSG00000129696	6.407685389	6.485878328	protein_coding	8	33330904	33371119	TTI2
ENSG00000129749	3.554067925	3.845183986	protein_coding	11	3686817	3692614	CHRNA10
ENSG00000129757	7.0218028	6.25865215	protein_coding	11	2904443	2907111	CDKN1C
ENSG00000129810	6.393570237	6.544728831	protein_coding	3	20202085	20227784	SGOL1
ENSG00000129910	3.393122761	1.802319292	protein_coding	16	89238175	89261900	CDH15
ENSG00000129911	9.752069226	9.489933546	protein_coding	19	1852399	1863564	KLF16
ENSG00000129925	9.933136027	9.582956544	protein_coding	16	420773	437113	TMEM8A
ENSG00000129932	8.389559911	8.449576416	protein_coding	19	3490820	3500938	DOHH
ENSG00000129933	10.31697412	10.49733458	protein_coding	19	19431614	19469564	MAU2
ENSG00000129946	4.381167248	4.917990497	protein_coding	19	416587	460996	SHC2
ENSG00000129951	4.062237333	3.183544561	protein_coding	19	812518	821967	
ENSG00000129968	7.883662252	8.279525596	protein_coding	19	1876809	1885500	FAM108A1
ENSG00000129988	1.16343121	2.971122874	protein_coding	20	36974759	37005665	LBP
ENSG00000129990	6.059927234	6.204928885	protein_coding	19	55684464	55691720	SYT5
ENSG00000129991	6.687686437	6.660126317	protein_coding	19	55663138	55669100	TNNI3
ENSG00000129993	1.16343121	0.950786998	protein_coding	16	88941266	89043612	CBFA2T3
ENSG00000130005	6.35766608	7.204272857	protein_coding	19	1397089	1401552	GAMT
ENSG00000130021	8.105542576	8.032919217	protein_coding	X	6966961	7066231	HDHD1
ENSG00000130023	7.2307479	7.113233895	protein_coding	6	170151721	170181680	C6orf70
ENSG00000130024	8.02999382	8.486319932	protein_coding	6	170104001	170124151	PHF10
ENSG00000130032	0.499066092	0.950786998	protein_coding	X	150863596	150874396	PRRG3
ENSG00000130038	2.57521082	1.925536307	protein_coding	12	3715799	3873985	EFCAB4B
ENSG00000130045	1.960915222	2.039052734	protein_coding	9	91150016	91199189	NXNL2
ENSG00000130052	1.616589159	4.406904905	protein_coding	X	67867508	67945684	STARD8
ENSG00000130055	1.16343121	0.697730409	protein_coding	X	69642881	69653240	GDPD2
ENSG00000130066	9.550548025	8.870627795	protein_coding	X	23801290	23804343	SAT1

ENSG00000130119	9.369490093	9.340854184	protein_coding	X	54556644	54587504	GNL3L
ENSG00000130147	10.33969843	9.944783414	protein_coding	2	235860617	235964358	SH3BP4
ENSG00000130150	7.913645586	8.013952351	protein_coding	X	14891563	14940449	MOSPD2
ENSG00000130158	10.23869682	9.648708264	protein_coding	19	11309973	11373157	DOCK6
ENSG00000130159	10.72187726	10.54615918	protein_coding	19	11616745	11639987	ECSIT
ENSG00000130164	12.88200393	11.57744029	protein_coding	19	11200038	11244492	LDLR
ENSG00000130165	10.15801652	10.17254941	protein_coding	19	11663858	11670051	ELOF1
ENSG00000130173	1.407729925	2.039052734	protein_coding	19	11348126	11352618	C19orf80
ENSG00000130175	12.48227259	12.39487596	protein_coding	19	11546269	11561783	PRKCSH
ENSG00000130176	1.616589159	4.250702764	protein_coding	19	11649579	11661138	CNN1
ENSG00000130177	8.867898261	8.948972504	protein_coding	13	115000362	115038198	CDC16
ENSG00000130182	0.869158192	0.390640832	protein_coding	16	3138895	3142861	ZSCAN10
ENSG00000130193	7.700873103	8.205465413	protein_coding	8	143808621	143818345	C8orf55
ENSG00000130201	0.869158192	0.390640832	protein_coding	19	45715879	45737469	EXOC3L2
ENSG00000130202	9.636212778	9.673093524	protein_coding	19	45349393	45392485	PVRL2
ENSG00000130203	4.593635215	3.748356452	protein_coding	19	45408956	45412650	APOE
ENSG00000130204	9.990530519	9.995327612	protein_coding	19	45394477	45406935	TOMM40
ENSG00000130208	5.158541613	5.175534283	protein_coding	19	45417921	45422606	APOC1
ENSG00000130222	2.57521082	1.667587519	protein_coding	9	92219928	92221470	GADD45G
ENSG00000130224	0	0.697730409	protein_coding	X	114345185	114468635	LRCH2
ENSG00000130226	0.499066092	0	protein_coding	7	153584182	154685995	DPP6
ENSG00000130227	10.25342625	10.12271748	protein_coding	8	21777180	21864096	XPO7
ENSG00000130244	6.328285199	6.338437404	protein_coding	19	38893775	38899728	FAM98C
ENSG00000130254	9.31342641	9.381780274	protein_coding	19	5587011	5622938	SAFB2
ENSG00000130255	11.91761312	12.6905383	protein_coding	19	5690272	5691674	RPL36
ENSG00000130270	8.193715877	8.122575449	protein_coding	19	1782074	1812275	ATP8B3
ENSG00000130287	2.35979773	2.579085888	protein_coding	19	19322782	19363061	NCAN
ENSG00000130294	0.499066092	0.390640832	protein_coding	2	241653181	241759725	KIF1A
ENSG00000130299	9.338510612	8.913126522	protein_coding	19	17445809	17453539	GTPBP3
ENSG00000130300	2.57521082	2.579085888	protein_coding	19	17462264	17488148	PLVAP
ENSG00000130303	11.11983076	12.20078083	protein_coding	19	17502082	17516457	BST2
ENSG00000130304	7.296703257	6.628734426	protein_coding	19	17579578	17616977	SLC27A1
ENSG00000130305	8.872994239	8.435390709	protein_coding	7	72716514	72722864	NSUN5
ENSG00000130307	0.869158192	0.697730409	protein_coding	19	17360838	17375573	USHBP1
ENSG00000130309	13.07601665	12.7937829	protein_coding	19	17666460	17693965	GLT25D1
ENSG00000130311	9.480493389	9.701929717	protein_coding	19	17420337	17434094	DDA1
ENSG00000130312	9.215524471	9.830679389	protein_coding	19	17416477	17417652	MRPL34
ENSG00000130313	10.32907648	10.45481329	protein_coding	19	17622432	17632095	PGLS
ENSG00000130332	9.4441477	9.940224002	protein_coding	19	2321522	2328614	LSM7
ENSG00000130338	8.611591722	8.366375472	protein_coding	6	158733692	158932860	TULP4
ENSG00000130340	9.106170722	8.948064416	protein_coding	6	158244281	158366109	SNX9
ENSG00000130347	7.071412799	6.733635362	protein_coding	6	107018903	107077373	RTN4IP1
ENSG00000130348	7.491518048	7.307006333	protein_coding	6	107077453	107116292	QRSL1
ENSG00000130349	6.086493765	6.758710666	protein_coding	6	107349407	107372546	C6orf203
ENSG00000130363	6.664362065	6.59664425	protein_coding	6	159397312	159421219	RSPH3
ENSG00000130377	3.502389126	3.493416095	protein_coding	19	6135710	6193112	ACSBG2
ENSG00000130382	11.80897089	11.64245619	protein_coding	19	6210393	6279959	MLLT1
ENSG00000130383	0.869158192	0.390640832	protein_coding	19	5866182	5903798	FUT5
ENSG00000130396	10.46605656	10.4017445	protein_coding	6	168227602	168372703	MLLT4
ENSG00000130402	12.60056532	12.45595985	protein_coding	19	39138310	39222223	ACTN4
ENSG00000130413	1.616589159	0.950786998	protein_coding	11	8413418	8615836	STK33
ENSG00000130414	9.399818301	9.303711094	protein_coding	2	240831867	240964819	NDUFA10
ENSG00000130427	1.407729925	0.950786998	protein_coding	7	100318423	100321323	EPO
ENSG00000130429	10.52955592	9.681859685	protein_coding	7	98971872	98992424	ARPC1B
ENSG00000130433	4.261916566	4.075511708	protein_coding	19	54495542	54515923	CACNG6
ENSG00000130449	8.164919416	7.825075067	protein_coding	5	60628100	60841997	ZSWIM6
ENSG00000130475	9.239397198	8.441856054	protein_coding	19	17858527	17899377	FCHO1
ENSG00000130477	6.421663777	6.465715653	protein_coding	19	17712137	17799401	UNC13A
ENSG00000130479	10.18131202	10.00496543	protein_coding	19	17830291	17845322	MAP1S
ENSG00000130487	9.058880138	7.699043321	protein_coding	22	50986462	50989451	KLHDC7B
ENSG00000130489	8.653654925	8.908465794	protein_coding	22	50961997	50964868	SCO2
ENSG00000130508	9.029515185	0.950786998	protein_coding	2	1635659	1748624	PXDN
ENSG00000130511	9.968679314	9.987835859	protein_coding	19	18530221	18545371	SSBP4

ENSG00000130513	8.655134734	9.209096633	protein_coding	19	18496968	18499986	GDF15
ENSG00000130517	8.417730894	8.975061478	protein_coding	19	18451408	18480763	PGPEP1
ENSG00000130518	4.75690578	5.187895122	protein_coding	19	18367909	18385319	KIAA1683
ENSG00000130520	11.09278494	11.29075987	protein_coding	19	18417720	18434001	LSM4
ENSG00000130522	11.08841017	11.06750809	protein_coding	19	18390563	18392432	JUND
ENSG00000130528	0.869158192	0.697730409	protein_coding	19	49654458	49658681	HRC
ENSG00000130529	8.267164507	7.452416987	protein_coding	19	49661052	49715091	TRPM4
ENSG00000130544	7.55947245	7.495318771	protein_coding	19	7069471	7087979	ZNF557
ENSG00000130545	4.543379137	1.518964905	protein_coding	19	6464260	6467225	CRB3
ENSG00000130558	9.073341477	8.940779054	protein_coding	9	137967268	138013025	OLFM1
ENSG00000130559	10.09617162	9.751176567	protein_coding	9	138700333	138799074	CAMSAP1
ENSG00000130560	9.245304163	8.868709593	protein_coding	9	138824815	138853226	UBAC1
ENSG00000130561	0.499066092	0.697730409	protein_coding	2	234216309	234255701	SAG
ENSG00000130584	6.917151646	6.615068802	protein_coding	20	62375019	62462597	ZBTB46
ENSG00000130589	10.91119017	11.13229982	protein_coding	20	62189439	62205592	
ENSG00000130590	5.948480845	6.3217081	protein_coding	20	62605466	62611361	SAMD10
ENSG00000130592	5.563445526	5.085909553	protein_coding	11	1874200	1913497	LSP1
ENSG00000130595	2.762599152	0.390640832	protein_coding	11	1940792	1959936	TNNT3
ENSG00000130598	3.448790144	1.518964905	protein_coding	11	1860219	1862910	TNNI2
ENSG00000130600	7.35977471	5.9163892	processed_transc	11	2016406	2022700	H19
ENSG00000130612	0.499066092	0.390640832	protein_coding	19	41396731	41406413	CYP2G1P
ENSG00000130635	12.49709786	12.5303468	protein_coding	9	137533620	137736686	COL5A1
ENSG00000130638	10.30052049	10.24679846	protein_coding	22	46067678	46241187	ATXN10
ENSG00000130640	10.48406996	10.83529938	protein_coding	10	135093135	135125841	TUBGCP2
ENSG00000130643	1.16343121	1.518964905	protein_coding	10	135138927	135150475	CALY
ENSG00000130649	6.877132514	7.801136513	protein_coding	10	135333910	135374724	CYP2E1
ENSG00000130653	5.458875168	5.893930351	protein_coding	9	140354404	140444986	PNPLA7
ENSG00000130669	8.858936753	8.001751392	protein_coding	19	39616420	39670046	PAK4
ENSG00000130675	4.097495944	4.342020395	protein_coding	7	156786745	156803370	MNX1
ENSG00000130684	5.741167503	6.210997888	protein_coding	20	25654851	25677477	ZNF337
ENSG00000130695	8.972494341	8.979512278	protein_coding	1	26560691	26605299	CEP85
ENSG00000130699	8.847332411	8.428896259	protein_coding	20	60528525	60640866	TAF4
ENSG00000130701	4.131913373	0	protein_coding	20	60985293	61002589	C20orf151
ENSG00000130702	11.55882167	10.35043385	protein_coding	20	60883011	60942368	LAMA5
ENSG00000130703	9.11480047	8.613182984	protein_coding	20	60813580	60871268	OSBPL2
ENSG00000130706	10.27956608	10.23045979	protein_coding	20	60877149	60883918	ADRM1
ENSG00000130707	12.2161591	12.12997791	protein_coding	9	133320094	133376661	ASS1
ENSG00000130711	2.471521042	2.501982735	protein_coding	9	133539981	133558368	PRDM12
ENSG00000130713	9.754142144	9.706232715	protein_coding	9	133569108	133580248	EXOSC2
ENSG00000130714	7.824391858	8.094946597	protein_coding	9	134378289	134399193	POMT1
ENSG00000130717	8.02999382	8.282410365	protein_coding	9	134399188	134406655	UCK1
ENSG00000130720	6.965663283	6.142791909	protein_coding	9	133777825	133814673	FIBCD1
ENSG00000130723	11.41272309	10.80075684	protein_coding	9	134269480	134375584	PRRC2B
ENSG00000130724	10.07421587	10.22597149	protein_coding	19	59062934	59066486	CHMP2A
ENSG00000130725	10.40448041	10.45704918	protein_coding	19	59067080	59070343	UBE2M
ENSG00000130726	13.03205925	12.88266396	protein_coding	19	59055836	59062082	TRIM28
ENSG00000130731	9.120167967	9.753257466	protein_coding	16	684429	686358	C16orf13
ENSG00000130733	10.22431399	10.28649432	protein_coding	19	11033446	11039357	YIPF2
ENSG00000130734	8.400188597	7.994732806	protein_coding	19	10654593	10664093	ATG4D
ENSG00000130741	11.56000706	11.43157717	protein_coding	X	24072833	24096088	EIF2S3
ENSG00000130748	6.489593565	7.083771121	protein_coding	19	47549168	47551882	TMEM160
ENSG00000130749	10.08633286	9.727557272	protein_coding	19	47567449	47617009	ZC3H4
ENSG00000130751	2.238690726	2.579085888	protein_coding	19	47524143	47549016	NPAS1
ENSG00000130758	6.984620223	6.896886088	protein_coding	19	40697651	40721481	MAP3K10
ENSG00000130762	8.770222845	8.26792849	protein_coding	1	3370990	3397677	ARHGEF16
ENSG00000130764	9.336667406	9.241329169	protein_coding	1	3696784	3713068	LRRC47
ENSG00000130766	8.269098624	8.117738058	protein_coding	1	28586038	28609002	SESN2
ENSG00000130768	5.613019223	4.489113623	protein_coding	1	28261504	28285668	SMPDL3B
ENSG00000130770	8.602416377	8.869669013	protein_coding	1	28562620	28573417	ATPIF1
ENSG00000130772	6.567100227	7.138927266	protein_coding	1	28655513	28662476	MED18
ENSG00000130775	6.400645076	6.963107483	protein_coding	1	28199055	28213196	C1orf38
ENSG00000130779	10.08633286	9.937938881	protein_coding	12	122755979	122907179	CLIP1
ENSG00000130783	2.762599152	2.420525079	protein_coding	12	123258874	123312075	CCDC62

ENSG00000130787	9.860592703	9.381780274	protein_coding	12	123319000	123347507	HIP1R
ENSG00000130803	10.28674387	10.2541644	protein_coding	19	9251056	9274090	ZNF317
ENSG00000130810	7.464764762	7.495318771	protein_coding	19	10216965	10225414	PPAN
ENSG00000130811	11.51263169	11.72628042	protein_coding	19	10225690	10230599	EIF3G
ENSG00000130812	3.393122761	4.021284656	protein_coding	19	10203013	10213425	ANGPTL6
ENSG00000130813	9.05999771	9.640299997	protein_coding	19	10196806	10203927	C19orf66
ENSG00000130816	12.50614595	12.65835159	protein_coding	19	10244023	10305755	DNMT1
ENSG00000130818	7.990682129	7.909600377	protein_coding	19	9638683	9649303	ZNF426
ENSG00000130821	7.642387679	7.883239271	protein_coding	X	152953554	152962048	SLC6A8
ENSG00000130822	2.238690726	2.242360793	protein_coding	X	152935185	152954465	PNCK
ENSG00000130826	11.18300063	11.26127074	protein_coding	X	153991031	154005964	DKC1
ENSG00000130827	9.813659349	9.428104741	protein_coding	X	153686621	153701989	PLXNA3
ENSG00000130829	8.107705799	7.462626902	protein_coding	X	152907946	152916781	DUSP9
ENSG00000130830	8.771587845	8.579568843	protein_coding	X	154006959	154049282	MPP1
ENSG00000130844	8.947349623	9.20225818	protein_coding	19	54024235	54083523	ZNF331
ENSG00000130856	7.071412799	7.23139676	protein_coding	18	74536116	74682680	ZNF236
ENSG00000130876	0.499066092	0.950786998	protein_coding	19	33699570	33716756	SLC7A10
ENSG00000130881	9.012297947	9.509147322	protein_coding	19	33685166	33699765	LRP3
ENSG00000130921	7.748787017	7.444711851	protein_coding	12	123717463	123742506	C12orf65
ENSG00000130935	9.524067267	9.756373197	protein_coding	17	65714061	65740318	NOL11
ENSG00000130939	9.752069226	9.675838676	protein_coding	1	10092890	10241297	UBE4B
ENSG00000130940	8.169068555	8.309531754	protein_coding	1	10696661	10856707	CASZ1
ENSG00000130943	1.16343121	1.518964905	protein_coding	22	46651560	46659219	PKDREJ
ENSG00000130948	1.407729925	2.144285137	protein_coding	9	98997588	99064434	HSD17B3
ENSG00000130950	0	0.390640832	protein_coding	9	97077605	97090926	FAM22F
ENSG00000130956	7.642387679	7.389591938	protein_coding	9	99212483	99253618	HABP4
ENSG00000130957	1.16343121	1.925536307	protein_coding	9	97321002	97356075	FBP2
ENSG00000130958	7.514527001	6.831423059	protein_coding	9	99082988	99145992	SLC35D2
ENSG00000130962	6.771940731	6.930376707	protein_coding	X	37208528	37316548	PRRG1
ENSG00000130985	12.78640906	12.8300936	protein_coding	X	47050260	47074527	UBA1
ENSG00000130988	4.292665995	4.319727133	protein_coding	X	46937775	46952712	RGN
ENSG00000130997	3.78787645	4.489113623	protein_coding	4	2073645	2243848	POLN
ENSG00000131013	7.660183591	7.926295939	protein_coding	6	149825869	149867174	PPIL4
ENSG00000131015	5.869093502	6.037446454	protein_coding	6	150263136	150270371	ULBP2
ENSG00000131016	7.444369108	8.17474298	protein_coding	6	151561134	151679692	AKAP12
ENSG00000131018	4.026095388	4.857374129	protein_coding	6	152442819	152958936	SYNE1
ENSG00000131019	6.023726639	5.259915406	protein_coding	6	150383341	150390257	ULBP3
ENSG00000131023	8.550881589	8.704198827	protein_coding	6	149979289	150039392	LATS1
ENSG00000131037	7.190493718	6.615068802	protein_coding	19	55583407	55599290	EPS8L1
ENSG00000131042	0.499066092	0	protein_coding	19	54777675	54785039	LILRB2
ENSG00000131043	9.599791022	9.737051443	protein_coding	20	34824381	34858840	C20orf4
ENSG00000131044	0.869158192	2.144285137	protein_coding	20	30458505	30532764	TTLL9
ENSG00000131051	11.19830674	11.91178696	protein_coding	20	34291531	34330234	RBM39
ENSG00000131061	7.50471092	6.99159092	protein_coding	20	32319463	32380075	ZNF341
ENSG00000131067	8.074911866	8.46235258	protein_coding	20	33432523	33460663	GGT7
ENSG00000131069	10.10702545	9.423538659	protein_coding	20	33459949	33515769	ACSS2
ENSG00000131080	4.543379137	1.16600992	protein_coding	X	65815479	65859108	EDA2R
ENSG00000131089	7.663128351	8.104758487	protein_coding	X	62854847	63005426	ARHGEF9
ENSG00000131094	4.618121645	4.872768657	protein_coding	17	43037061	43045644	C1QL1
ENSG00000131095	3.698846687	2.144285137	protein_coding	17	42982993	42992914	GFAP
ENSG00000131096	1.16343121	0.697730409	protein_coding	17	42030111	42081837	PYY
ENSG00000131097	2.35979773	1.802319292	protein_coding	17	42925279	42927840	HIGD1B
ENSG00000131100	9.712817799	9.925536292	protein_coding	22	18074902	18111584	ATP6V1E1
ENSG00000131115	7.768062907	7.860254213	protein_coding	19	44716691	44741420	ZNF227
ENSG00000131116	6.386460534	7.210344614	protein_coding	19	44111377	44124014	ZNF428
ENSG00000131126	0	0.950786998	protein_coding	19	43910657	43922763	TEX101
ENSG00000131127	5.827699195	5.674321415	protein_coding	4	331603	378653	ZNF141
ENSG00000131142	0.499066092	0.697730409	protein_coding	19	8117651	8127534	CCL25
ENSG00000131143	11.13210359	11.39289324	protein_coding	16	85832239	85840650	COX4I1
ENSG00000131148	8.844740944	8.857146507	protein_coding	16	85812230	85833148	COX4NB
ENSG00000131149	10.35206871	10.17410353	protein_coding	16	85645015	85709810	KIAA0182
ENSG00000131153	8.410739551	8.765275934	protein_coding	16	85709804	85722605	GINS2
ENSG00000131165	10.71408926	10.39442996	protein_coding	16	89710839	89724253	CHMP1A

ENSG00000131171	7.993024472	8.721307575	protein_coding	X	80457442	80554046	SH3BGRL
ENSG00000131174	9.543374069	10.24790575	protein_coding	X	77154935	77162870	COX7B
ENSG00000131183	0	0.950786998	protein_coding	5	176806236	176825849	SLC34A1
ENSG00000131187	5.472368541	4.710890357	protein_coding	5	176829141	176836577	F12
ENSG00000131188	6.535310252	6.505763096	protein_coding	5	176873446	176883283	PRR7
ENSG00000131196	7.186405829	7.301328834	protein_coding	18	77155772	77289325	NFATC1
ENSG00000131203	2.35979773	1.925536307	protein_coding	8	39759794	39785963	IDO1
ENSG00000131236	11.5844836	11.57258753	protein_coding	1	40505905	40538321	CAP1
ENSG00000131238	10.09289948	10.28361771	protein_coding	1	40538379	40563375	PPT1
ENSG00000131242	6.17992145	3.32456471	protein_coding	17	29718642	29865236	RAB11FIP4
ENSG00000131263	10.13698882	10.14547392	protein_coding	X	73805052	73834452	RLIM
ENSG00000131269	8.875535495	8.899098923	protein_coding	X	74273109	74376567	ABCB7
ENSG00000131323	10.32210674	9.571798825	protein_coding	14	103243813	103377837	TRAF3
ENSG00000131351	8.114176072	8.480051236	protein_coding	19	17160573	17186343	HAUS8
ENSG00000131368	8.878072281	9.290875744	protein_coding	3	15083967	15106842	MRPS25
ENSG00000131370	4.734682531	4.826083323	protein_coding	3	15296360	15382875	SH3BP5
ENSG00000131373	8.079327701	8.38394126	protein_coding	3	15602211	15643338	HACL1
ENSG00000131374	9.168633912	8.660517685	protein_coding	3	17199899	18486309	TBC1D5
ENSG00000131375	7.698004462	8.367734306	protein_coding	3	15247659	15294425	CAPN7
ENSG00000131378	9.826850281	7.033288635	protein_coding	3	16357352	16555533	RFTN1
ENSG00000131379	0.499066092	0	protein_coding	3	14716606	14814541	C3orf20
ENSG00000131381	8.541310587	8.610889744	protein_coding	3	15111580	15140670	ZFYVE20
ENSG00000131386	0.499066092	0	protein_coding	3	16216156	16273499	GALNTL2
ENSG00000131389	10.40843431	9.644790504	protein_coding	3	14444076	14530857	SLC6A6
ENSG00000131398	6.96088487	7.284161038	protein_coding	19	50815194	50836772	KCNC3
ENSG00000131400	2.10647801	2.420525079	protein_coding	19	50861735	50868931	NAPSA
ENSG00000131401	2.762599152	3.32456471	pseudogene	19	50837053	50848024	NAPSB
ENSG00000131408	9.462857538	9.574743444	protein_coding	19	50832949	50886266	NR1H2
ENSG00000131409	4.883502971	4.048652948	protein_coding	19	51020149	51071302	LRRC4B
ENSG00000131435	9.278327677	10.00671086	protein_coding	5	131593364	131609147	PDLIM4
ENSG00000131437	7.865879816	8.093304774	protein_coding	5	132028368	132073330	KIF3A
ENSG00000131446	10.46605656	10.59280449	protein_coding	5	180217541	180242652	MGAT1
ENSG00000131459	10.16945243	10.22297151	protein_coding	5	179727690	179780387	GFPT2
ENSG00000131462	9.894817144	9.830679389	protein_coding	17	40761358	40767254	TUBG1
ENSG00000131467	10.58106613	10.28613505	protein_coding	17	40976443	40995775	PSME3
ENSG00000131469	11.76972141	12.35740611	protein_coding	17	41150446	41154956	RPL27
ENSG00000131470	6.236372259	6.515603638	protein_coding	17	40724329	40729747	PSMC3IP
ENSG00000131471	7.08025234	6.771086752	protein_coding	17	41003201	41010138	AOC3
ENSG00000131473	11.91421833	11.23414924	protein_coding	17	40023161	40075272	ACLY
ENSG00000131475	8.53489446	9.065760854	protein_coding	17	40925454	40931616	VPS25
ENSG00000131480	7.026383958	7.122922611	protein_coding	17	40996609	41002722	AOC2
ENSG00000131495	7.0218028	7.546612122	protein_coding	5	140018325	140027370	NDUFA2
ENSG00000131503	4.863159938	4.489113623	protein_coding	5	139781399	139929163	ANKHD1
ENSG00000131504	11.63184932	11.0366594	protein_coding	5	140894583	140998622	DIAPH1
ENSG00000131507	9.697818752	9.514064455	protein_coding	5	141488070	141534008	NDFIP1
ENSG00000131508	9.552931438	9.461579211	protein_coding	5	138906016	139008018	UBE2D2
ENSG00000131558	9.578892348	9.138412238	protein_coding	7	132937829	133751342	EXOC4
ENSG00000131584	9.053279257	9.098048079	protein_coding	1	1227756	1244989	ACAP3
ENSG00000131591	8.373468605	8.507432028	protein_coding	1	1017198	1051741	C1orf159
ENSG00000131620	1.16343121	0.697730409	protein_coding	11	69924408	70035634	ANO1
ENSG00000131626	10.13327726	10.47921951	protein_coding	11	70116806	70230509	PPFIA1
ENSG00000131634	1.960915222	2.420525079	protein_coding	16	1578689	1605581	TMEM204
ENSG00000131650	6.560798025	4.566889334	protein_coding	16	3014160	3018381	KREMEN2
ENSG00000131652	9.615078651	10.1446815	protein_coding	16	3074032	3077756	THOC6
ENSG00000131653	11.06994534	10.85117288	protein_coding	16	2205699	2228130	TRAF7
ENSG00000131668	0.869158192	1.667587519	protein_coding	9	96713628	96717654	BARX1
ENSG00000131669	9.388297379	9.800811083	protein_coding	9	95883771	95896570	NINJ1
ENSG00000131686	0.869158192	0.950786998	protein_coding	1	9005922	9035151	CA6
ENSG00000131697	8.13977083	8.205465413	protein_coding	1	5922871	6052533	NPHP4
ENSG00000131711	9.99696351	9.867165657	protein_coding	5	71403061	71505395	MAP1B
ENSG00000131724	10.39432652	10.2460598	protein_coding	X	117861535	117928502	IL13RA1
ENSG00000131725	8.259402007	8.591248608	protein_coding	X	117480036	117590949	WDR44
ENSG00000131730	2.762599152	2.971122874	protein_coding	5	80529104	80562216	CKMT2

ENSG00000131732	7.384964606	7.655203404	protein_coding	5	80597409	80609116	ZCCHC9
ENSG00000131737	1.16343121	0	protein_coding	17	39533902	39538655	KRT34
ENSG00000131746	8.883132512	0	protein_coding	17	38632080	38657854	TNS4
ENSG00000131747	11.26814714	11.40485593	protein_coding	17	38544768	38574408	TOP2A
ENSG00000131748	9.0577617	9.019827949	protein_coding	17	37793318	37820454	STARD3
ENSG00000131759	8.925442603	8.129000175	protein_coding	17	38465436	38513895	RARA
ENSG00000131773	4.409500985	3.714580548	protein_coding	8	136469700	136668965	KHDRBS3
ENSG00000131778	9.778108644	10.44615655	protein_coding	1	146714291	146767443	CHD1L
ENSG00000131779	8.092494681	8.301022059	protein_coding	1	145516252	145523730	PEX11B
ENSG00000131781	4.097495944	4.528525526	protein_coding	1	146646930	146714700	FMO5
ENSG00000131788	9.800346696	9.522019224	protein_coding	1	145575233	145586546	PIAS3
ENSG00000131791	9.036344956	9.309379244	protein_coding	1	146626685	146644129	PRKAB2
ENSG00000131795	9.341271012	9.909298518	protein_coding	1	145507598	145511444	RBM8A
ENSG00000131797	6.252103507	6.930376707	pseudogene	16	31711911	31721097	KIAA0664L3
ENSG00000131828	10.2337534	10.48705564	protein_coding	X	19362011	19379823	PDHA1
ENSG00000131844	10.36207036	10.15966365	protein_coding	5	70883115	70954531	MCCC2
ENSG00000131845	7.584540566	7.770643695	protein_coding	19	57862645	57871266	ZNF304
ENSG00000131848	6.693459083	6.970281236	protein_coding	19	56732681	56826294	ZSCAN5A
ENSG00000131849	5.752272083	5.938503778	protein_coding	19	58944182	58951589	ZNF132
ENSG00000131871	7.940593421	7.991210667	protein_coding	15	101811022	101817705	
ENSG00000131873	8.927893208	9.043827484	protein_coding	15	101715928	101792137	CHSY1
ENSG00000131876	8.061582684	8.306700762	protein_coding	15	101821715	101835487	SNRPA1
ENSG00000131885	2.238690726	1.518964905	protein_coding	17	16744057	16749197	KRT17P1
ENSG00000131899	10.85048168	11.306079	protein_coding	17	18128901	18148189	LLGL1
ENSG00000131910	0.499066092	1.16600992	protein_coding	1	27237980	27240457	NR0B2
ENSG00000131914	0.499066092	1.353254395	protein_coding	1	26737269	26756213	LIN28A
ENSG00000131931	6.610461229	6.835357159	protein_coding	8	42691817	42698468	THAP1
ENSG00000131941	7.311793575	6.712401119	protein_coding	19	33469499	33555824	RHPN2
ENSG00000131943	7.50471092	7.546612122	protein_coding	19	30190483	30206364	C19orf12
ENSG00000131944	4.883502971	4.710890357	protein_coding	19	33463148	33467946	C19orf40
ENSG00000131966	9.34494335	9.952048633	protein_coding	14	58666798	58701750	ACTR10
ENSG00000131969	3.448790144	2.851901313	protein_coding	14	51338878	51371688	ABHD12B
ENSG00000131979	6.290697143	6.293387477	protein_coding	14	55308726	55369570	GCH1
ENSG00000131981	9.325558442	9.407112231	protein_coding	14	55590828	55612126	LGALS3
ENSG00000131982	0.499066092	0.390640832	pseudogene	12	20602589	20603017	UBE2L2
ENSG00000132000	4.19837882	3.813624741	protein_coding	19	14042000	14064204	PODNL1
ENSG00000132002	10.61757819	10.72908697	protein_coding	19	14625582	14640049	DNAJB1
ENSG00000132003	9.722731226	9.924613326	protein_coding	19	13906274	13943044	ZSWIM4
ENSG00000132004	9.151960225	9.20378064	protein_coding	19	12799121	12807457	FBXW9
ENSG00000132005	8.610066544	8.379906558	protein_coding	19	14072343	14117134	RFX1
ENSG00000132010	4.026095388	4.15321211	protein_coding	19	12241300	12251222	ZNF20
ENSG00000132016	6.522395495	7.896480033	protein_coding	19	13993168	14016909	C19orf57
ENSG00000132017	9.167597433	9.195387157	protein_coding	19	14063319	14072254	DCAF15
ENSG00000132024	9.931915689	10.03606464	protein_coding	19	14016956	14041693	CC2D1A
ENSG00000132026	5.090170488	4.319727133	protein_coding	19	12936293	12946242	RTBDN
ENSG00000132031	4.593635215	3.993387124	protein_coding	2	20191872	20212455	MATN3
ENSG00000132109	7.746012174	8.280968702	protein_coding	11	4406127	4414926	TRIM21
ENSG00000132122	5.375152132	5.647721597	protein_coding	1	48761044	48937845	SPATA6
ENSG00000132128	9.527303063	9.820295675	protein_coding	1	46726868	46769280	LRRC41
ENSG00000132130	6.17992145	2.912743273	protein_coding	17	35294772	35301917	LHX1
ENSG00000132139	0.499066092	0.390640832	protein_coding	17	34071530	34079897	GAS2L2
ENSG00000132141	3.335220907	4.319727133	protein_coding	17	33254878	33288506	CCT6B
ENSG00000132142	10.84465707	10.04628383	protein_coding	17	35441923	35766902	ACACA
ENSG00000132153	10.26752315	10.23157969	protein_coding	3	47844399	47891685	DHX30
ENSG00000132155	10.18541435	10.02921141	protein_coding	3	12625100	12705725	RAF1
ENSG00000132170	7.748787017	6.408769788	protein_coding	3	12328867	12475855	PPARG
ENSG00000132182	10.25147099	9.237617989	protein_coding	3	13357737	13461809	NUP210
ENSG00000132185	0.869158192	0.697730409	protein_coding	1	161676762	161684142	FCRLA
ENSG00000132196	6.129712275	5.42565083	protein_coding	1	162760492	162782607	HSD17B7
ENSG00000132199	8.45218826	8.616616024	protein_coding	18	672546	712676	ENOSF1
ENSG00000132204	0	0.697730409	protein_coding	18	1268312	1359630	LINC00470
ENSG00000132205	4.593635215	4.566889334	protein_coding	18	2847028	2914090	EMILIN2
ENSG00000132207	0.869158192	1.16600992	protein_coding	16	30205208	30208882	SLX1A

ENSG00000132254	9.948303785	9.795267513	protein_coding	11	6496910	6502666	ARFIP2
ENSG00000132256	8.698837588	8.638170933	protein_coding	11	5684425	5959849	TRIM5
ENSG00000132259	0.499066092	2.501982735	protein_coding	11	6255995	6265659	CNGA4
ENSG00000132274	5.431503808	7.318294738	protein_coding	11	5710919	5758319	TRIM22
ENSG00000132275	7.845284967	8.159132889	protein_coding	11	6616305	6624850	RRP8
ENSG00000132286	7.950269163	8.008735998	protein_coding	11	6502677	6530208	FXC1
ENSG00000132294	9.186141067	9.284414991	protein_coding	8	132916335	133025889	EFR3A
ENSG00000132300	9.423456371	9.735473414	protein_coding	2	86333305	86369280	PTCD3
ENSG00000132305	10.66057525	10.90854817	protein_coding	2	86371055	86422893	IMMT
ENSG00000132313	8.641761486	9.046375341	protein_coding	2	86426580	86440917	MRPL35
ENSG00000132323	8.158673248	8.124184315	protein_coding	2	239079042	239112370	ILKAP
ENSG00000132326	6.699208722	4.990346085	protein_coding	2	239152679	239198743	PER2
ENSG00000132329	8.558809206	8.283850589	protein_coding	2	238767536	238820756	RAMP1
ENSG00000132330	6.984620223	6.624193585	protein_coding	2	238969530	239008054	SCLY
ENSG00000132334	8.777034966	8.978623216	protein_coding	10	129705325	129884119	PTPRE
ENSG00000132341	11.35789184	11.40054751	protein_coding	12	131356424	131362223	RAN
ENSG00000132356	10.27812622	10.3400875	protein_coding	5	40759481	40798476	PRKAA1
ENSG00000132357	7.72925009	7.157901395	protein_coding	5	40841286	40860275	CARD6
ENSG00000132359	10.71940382	10.87477986	protein_coding	17	2699732	2941034	RAP1GAP2
ENSG00000132361	12.10712564	12.35740611	protein_coding	17	2592680	2614927	KIAA0664
ENSG00000132376	8.851210906	9.748049581	protein_coding	17	1397872	1420182	INPP5K
ENSG00000132382	11.67077621	11.76030407	protein_coding	17	4442192	4458681	MYBBP1A
ENSG00000132383	10.99829927	11.47304361	protein_coding	17	1733266	1802848	RPA1
ENSG00000132386	4.800350936	4.932754837	protein_coding	17	1665253	1680859	SERPINF1
ENSG00000132388	10.36975169	10.39342964	protein_coding	17	4172512	4269969	UBE2G1
ENSG00000132394	8.45218826	8.094946597	protein_coding	3	127872297	128127485	EEFSEC
ENSG00000132405	8.203862602	8.104758487	protein_coding	4	6910969	7034845	TBC1D14
ENSG00000132406	6.970425921	7.00911166	protein_coding	4	4237269	4249950	TMEM128
ENSG00000132423	6.830742851	6.919299326	protein_coding	6	99817276	99842080	COQ3
ENSG00000132424	9.186141067	9.516516751	protein_coding	6	99845927	99873207	PNISR
ENSG00000132429	0.499066092	0	protein_coding	6	105606155	105627870	POPDC3
ENSG00000132432	9.262881318	9.601555605	protein_coding	7	54819943	54827667	SEC61G
ENSG00000132434	8.096857104	8.385283657	protein_coding	7	55433141	55501435	LANCL2
ENSG00000132436	8.637276046	8.860045978	protein_coding	7	50511831	50518088	FIGNL1
ENSG00000132437	0	0.697730409	protein_coding	7	50526134	50633154	DDC
ENSG00000132463	10.23721557	10.42705723	protein_coding	4	71681499	71705662	GRSF1
ENSG00000132464	0	0.390640832	protein_coding	4	71494461	71552533	ENAM
ENSG00000132465	0	0.390640832	protein_coding	4	71521258	71547534	IGJ
ENSG00000132466	10.82704081	10.81576885	protein_coding	4	73939093	74124515	ANKRD17
ENSG00000132467	8.753741308	8.790825214	protein_coding	4	71554196	71556267	UTP3
ENSG00000132470	12.22216178	5.80042639	protein_coding	17	73713474	73753898	ITGB4
ENSG00000132471	10.84562947	10.68343859	protein_coding	17	73841780	73851501	WBP2
ENSG00000132475	11.38912937	11.42326962	protein_coding	17	73772517	73775860	H3F3B
ENSG00000132478	8.162840364	8.477536111	protein_coding	17	73780681	73821886	UNK
ENSG00000132481	9.394512382	9.565299431	protein_coding	17	73870247	73874656	TRIM47
ENSG00000132485	9.169669647	9.405790002	protein_coding	1	71528974	71546980	ZRANB2
ENSG00000132498	0.869158192	0	protein_coding	9	43089972	43133544	ANKRD20A3
ENSG00000132507	13.57565834	13.82177636	protein_coding	17	7210318	7215782	EIF5A
ENSG00000132510	9.841222348	9.652615414	protein_coding	17	7743222	7758106	KDM6B
ENSG00000132517	3.146112541	2.144285137	protein_coding	17	4935895	4955304	GPR172B
ENSG00000132518	1.16343121	0.697730409	protein_coding	17	7905988	7923658	GUCY2D
ENSG00000132522	9.355904607	9.930602076	protein_coding	17	7214643	7222394	GPS2
ENSG00000132530	9.847060811	11.24213563	protein_coding	17	6659156	6678962	XAF1
ENSG00000132535	8.513842764	8.982176182	protein_coding	17	7093209	7123369	DLG4
ENSG00000132541	6.738827102	6.945015469	protein_coding	8	99114572	99129469	HRSP12
ENSG00000132549	7.959880444	7.832967122	protein_coding	8	100025494	100889808	VPS13B
ENSG00000132561	5.538004898	6.434933235	protein_coding	8	98881068	99048944	MATN2
ENSG00000132563	6.275383355	5.317302248	protein_coding	5	137774706	137782658	REEP2
ENSG00000132570	6.283060568	6.460630645	protein_coding	5	134240596	134343649	PCBD2
ENSG00000132581	8.286389971	8.213044874	protein_coding	17	26975374	26989203	SDF2
ENSG00000132589	10.08193845	9.494294562	protein_coding	17	27206353	27224712	FLOT2
ENSG00000132591	9.722731226	9.493672366	protein_coding	17	27182020	27188085	ERAL1
ENSG00000132600	8.971306859	9.210611898	protein_coding	16	68344877	68392466	PRMT7

ENSG00000132603	8.690202285	8.668257984	protein_coding	16	69373343	69377014	NIP7
ENSG00000132604	8.373468605	8.108014344	protein_coding	16	69389464	69442474	TERF2
ENSG00000132612	9.572641559	9.608468897	protein_coding	16	69333562	69358949	VPS4A
ENSG00000132613	8.150302734	7.784953917	protein_coding	16	70695108	70719954	MTSS1L
ENSG00000132622	5.512107582	4.385599336	protein_coding	20	3713314	3733758	HSPA12B
ENSG00000132623	8.025424129	7.586839732	protein_coding	20	10015689	10037410	ANKRD5
ENSG00000132635	8.936437694	9.397831074	protein_coding	20	2815960	2821836	FAM113A
ENSG00000132639	0.499066092	0	protein_coding	20	10199478	10288066	SNAP25
ENSG00000132640	8.472474444	9.05483585	protein_coding	20	11871371	11907257	BTBD3
ENSG00000132646	11.18325706	11.69725626	protein_coding	20	5095599	5107272	PCNA
ENSG00000132661	8.440221571	8.640421249	protein_coding	20	23331373	23335414	NXT1
ENSG00000132664	7.23866569	7.758263791	protein_coding	20	18447771	18465287	POLR3F
ENSG00000132669	9.762404159	9.348444734	protein_coding	20	19867165	19983101	RIN2
ENSG00000132670	10.19511088	10.42412141	protein_coding	20	2844830	3019722	PTPRA
ENSG00000132676	10.77047592	10.94609939	protein_coding	1	155657751	155708803	DAP3
ENSG00000132677	3.077135474	1.518964905	protein_coding	1	156339003	156355011	RHBG
ENSG00000132680	8.711694395	9.315729406	protein_coding	1	155882834	155904191	KIAA0907
ENSG00000132681	0.869158192	0.390640832	protein_coding	1	160121360	160156767	ATP1A4
ENSG00000132688	10.38677526	9.449438015	protein_coding	1	156638555	156647189	NES
ENSG00000132692	5.550781289	6.470782801	protein_coding	1	156611182	156629324	BCAN
ENSG00000132694	9.612795809	9.868126104	protein_coding	1	156904632	157015162	ARHGEF11
ENSG00000132698	6.482942724	0.950786998	protein_coding	1	156030951	156040295	RAB25
ENSG00000132702	2.762599152	2.039052734	protein_coding	1	156589086	156595517	HAPLN2
ENSG00000132716	9.290757201	9.823765224	protein_coding	1	160185505	160254920	DCAF8
ENSG00000132718	2.847891871	4.274079748	protein_coding	1	155829300	155854990	SYT11
ENSG00000132740	8.793254009	8.847439299	protein_coding	11	68671310	68708070	IGHMBP2
ENSG00000132744	3.744048221	3.279072565	protein_coding	11	67410026	67418130	ACY3
ENSG00000132746	4.543379137	3.081239798	protein_coding	11	67429633	67448671	ALDH3B2
ENSG00000132749	8.08373006	8.017419471	protein_coding	11	68474908	68519032	MTL5
ENSG00000132763	7.127924785	7.246248121	protein_coding	1	45965725	45976739	MMACHC
ENSG00000132768	9.044272373	9.113519788	protein_coding	1	44435672	44439041	DPH2
ENSG00000132773	7.178195136	7.793067905	protein_coding	1	45805342	45809647	TOE1
ENSG00000132780	10.74290603	10.73172771	protein_coding	1	46049518	46084566	NASP
ENSG00000132781	7.062518763	7.73528765	protein_coding	1	45794835	45806142	MUTYH
ENSG00000132792	10.1653044	10.27132774	protein_coding	20	36322408	36500531	CTNNBL1
ENSG00000132793	7.974178365	7.959118381	protein_coding	20	39969560	39989222	LPIN3
ENSG00000132801	5.958104008	6.582669759	protein_coding	20	44486256	44507761	ZSWIM3
ENSG00000132819	8.931561329	8.941691738	protein_coding	20	55966463	55984389	RBM38
ENSG00000132821	7.737655476	8.653849929	protein_coding	20	36531499	36573752	VSTM2L
ENSG00000132823	9.005353105	9.18617459	protein_coding	20	42825136	42839431	C20orf111
ENSG00000132824	10.58534145	10.54555896	protein_coding	20	43124862	43150750	SERINC3
ENSG00000132825	6.721980839	6.592001111	protein_coding	20	58511894	58515352	PPP1R3D
ENSG00000132832	2.10647801	2.652276565	antisense	20	43285092	43324737	
ENSG00000132837	2.57521082	2.420525079	protein_coding	5	78293438	78531861	DMGDH
ENSG00000132840	3.004694206	0.390640832	protein_coding	5	78365540	78385289	BHMT2
ENSG00000132842	9.481327838	9.206061322	protein_coding	5	77296349	77590579	AP3B1
ENSG00000132846	7.423680977	6.904395923	protein_coding	5	76367897	76383148	ZBED3
ENSG00000132849	7.454602977	6.729413443	protein_coding	1	62208149	62629592	INADL
ENSG00000132855	0	0.950786998	protein_coding	1	63063158	63071830	ANGPTL3
ENSG00000132874	0.869158192	0	protein_coding	18	43194766	43263072	SLC14A2
ENSG00000132879	7.549958459	7.51752531	protein_coding	1	11714432	11723384	FBXO44
ENSG00000132881	5.072559152	5.294620694	protein_coding	1	16555170	16563495	RSG1
ENSG00000132906	6.17992145	6.376734172	protein_coding	1	15817327	15853029	CASP9
ENSG00000132912	9.030655728	9.069940847	protein_coding	5	150088002	150138671	DCTN4
ENSG00000132915	1.960915222	1.925536307	protein_coding	5	149237519	149324356	PDE6A
ENSG00000132932	1.407729925	1.518964905	protein_coding	13	25946209	26599989	ATP8A2
ENSG00000132938	1.16343121	0.390640832	protein_coding	13	29598748	30077892	MTUS2
ENSG00000132950	6.788216822	6.387491945	protein_coding	13	20397622	20437776	ZMYM5
ENSG00000132952	7.494827594	6.926693689	protein_coding	13	31191830	31233686	USPL1
ENSG00000132953	9.415619969	9.130429247	protein_coding	13	21351469	21477187	XPO4
ENSG00000132958	0.499066092	0	protein_coding	13	19997017	20110903	TPTE2
ENSG00000132963	9.122309386	8.908465794	protein_coding	13	29233241	29253062	POMP
ENSG00000132964	8.419473449	8.265014588	protein_coding	13	26828276	26979375	CDK8

ENSG00000132965	1.960915222	1.802319292	protein_coding	13	31309645	31338556	ALOX5AP
ENSG00000132967	1.407729925	0.390640832	pseudogene	3	22423310	22424420	HMGB1P5
ENSG00000132970	0.499066092	0	protein_coding	13	27131840	27263085	WASF3
ENSG00000132972	0.499066092	0	protein_coding	13	25338290	25454059	RNF17
ENSG00000132975	0	0.390640832	protein_coding	13	27329341	27334922	GPR12
ENSG00000133026	8.807962967	8.306700762	protein_coding	17	8377523	8534079	MYH10
ENSG00000133027	9.019209518	9.744392843	protein_coding	17	17408877	17495022	PEMT
ENSG00000133028	9.242353704	9.76979748	protein_coding	17	10583654	10600885	SCO1
ENSG00000133030	11.23809968	11.10730076	protein_coding	17	16946074	17088874	MPRIP
ENSG00000133048	4.712111592	5.187895122	protein_coding	1	203148059	203155877	CHI3L1
ENSG00000133055	0.499066092	0.697730409	protein_coding	1	203136939	203144941	MYBPH
ENSG00000133056	7.795160661	6.520498852	protein_coding	1	204391756	204463852	PIK3C2B
ENSG00000133059	7.81647827	7.88892877	protein_coding	1	205111632	205180727	DSTYK
ENSG00000133063	0	0.390640832	protein_coding	1	203181955	203242769	CHIT1
ENSG00000133065	9.05999771	9.003312166	protein_coding	1	205758221	205782876	SLC41A1
ENSG00000133067	0.869158192	1.667587519	protein_coding	1	202162938	202288909	LGR6
ENSG00000133069	5.316509574	5.878761167	protein_coding	1	205197304	205242471	TMCC2
ENSG00000133083	3.744048221	2.851901313	protein_coding	13	36343122	36705467	DCLK1
ENSG00000133101	4.409500985	3.813624741	protein_coding	13	37005967	37017019	CCNA1
ENSG00000133103	7.37062428	7.002128877	protein_coding	13	40229764	40365802	COG6
ENSG00000133104	8.830404061	8.68797299	protein_coding	13	36875775	36944317	SPG20
ENSG00000133106	3.502389126	0.950786998	protein_coding	13	43460524	43566407	EPSTI1
ENSG00000133107	1.407729925	0.390640832	protein_coding	13	38210773	38444562	TRPC4
ENSG00000133110	2.57521082	5.393992	protein_coding	13	38136720	38183563	POSTN
ENSG00000133111	5.498982484	5.43605125	protein_coding	13	37393361	37403241	RFXAP
ENSG00000133112	12.75768193	12.49262104	protein_coding	13	45911008	45915505	TPT1
ENSG00000133114	6.782811835	6.343971006	protein_coding	13	45563687	45607746	KIAA1704
ENSG00000133119	8.199812472	7.84667518	protein_coding	13	34392186	34540695	RFC3
ENSG00000133121	6.84118134	6.915587884	protein_coding	13	33677307	33924767	STARD13
ENSG00000133131	8.546104025	8.772475459	protein_coding	X	106183964	106243474	MORC4
ENSG00000133134	0.499066092	0	protein_coding	X	102564274	102565974	BEX2
ENSG00000133138	7.15746161	7.659647832	protein_coding	X	106045910	106119375	TBC1D8B
ENSG00000133142	9.533752964	9.713197829	protein_coding	X	102831159	102842657	TCEAL4
ENSG00000133149	0	0.390640832	protein_coding	X	104463611	104465358	TEX13A
ENSG00000133193	8.460675529	8.454700467	protein_coding	17	71203492	71228510	FAM104A
ENSG00000133195	8.162840364	7.688207601	protein_coding	17	70642086	71088853	SLC39A11
ENSG00000133216	9.242353704	8.824861692	protein_coding	1	23037332	23241818	EPHB2
ENSG00000133226	10.2733163	10.02663303	protein_coding	1	24958207	24999758	SRRM1
ENSG00000133243	11.35584576	11.03559117	protein_coding	19	1985447	2015702	BTBD2
ENSG00000133246	3.744048221	3.183544561	protein_coding	19	8554941	8567522	PRAM1
ENSG00000133247	8.417730894	8.703122764	protein_coding	19	55851221	55859488	SUV420H2
ENSG00000133250	7.748787017	7.688207601	protein_coding	19	8575463	8579048	ZNF414
ENSG00000133256	1.16343121	0.697730409	protein_coding	4	619373	664571	PDE6B
ENSG00000133265	10.40931148	10.22071743	protein_coding	19	55773592	55791751	HSPBP1
ENSG00000133275	11.09333086	11.10750411	protein_coding	19	1941161	1981309	CSNK1G2
ENSG00000133302	7.44778847	7.343375265	protein_coding	5	93954052	94075141	ANKRD32
ENSG00000133313	10.4819869	10.65199776	protein_coding	18	72163443	72190687	CNDP2
ENSG00000133315	9.023798905	9.198444985	protein_coding	11	63766030	63933578	MACROD1
ENSG00000133316	9.054401174	9.250197244	protein_coding	11	62599814	62609281	WDR74
ENSG00000133317	1.407729925	0.390640832	protein_coding	11	63273556	63284246	LGALS12
ENSG00000133318	10.76910987	10.75916755	protein_coding	11	63448918	63527363	RTN3
ENSG00000133321	5.255381949	4.640685763	protein_coding	11	63304281	63313934	RARRES3
ENSG00000133328	1.16343121	1.16600992	protein_coding	11	63320242	63330855	HRASLS2
ENSG00000133392	4.517578978	4.903073497	protein_coding	16	15796992	15950890	MYH11
ENSG00000133393	8.295907473	8.163833676	protein_coding	16	15959576	15982472	FOPNL
ENSG00000133398	8.753741308	8.506198656	protein_coding	5	6371994	6378707	MED10
ENSG00000133401	4.517578978	3.232099092	protein_coding	5	31639517	32111037	PDZD2
ENSG00000133422	10.67231523	10.4342086	protein_coding	22	31322596	31364284	MORC2
ENSG00000133424	7.262161623	7.343375265	protein_coding	22	33561991	34318829	LARGE
ENSG00000133433	3.146112541	3.608232228	protein_coding	22	24299601	24303373	GSTT2B
ENSG00000133454	1.16343121	1.353254395	protein_coding	22	26138111	26427007	MYO18B
ENSG00000133460	6.640654398	6.858739095	protein_coding	22	24198890	24231170	SLC2A11
ENSG00000133466	9.834054211	10.12713712	protein_coding	22	37576207	37595425	C1QTNF6

ENSG00000133477	6.305850085	3.027231696	protein_coding	22	40390953	40426043	FAM83F
ENSG00000133488	7.8607586	8.075120324	protein_coding	22	30884900	30901698	SEC14L4
ENSG00000133519	2.471521042	2.652276565	pseudogene	22	23732793	23744913	ZDHHC8P1
ENSG00000133561	1.407729925	0.697730409	protein_coding	7	150322463	150329473	GIMAP6
ENSG00000133574	0.499066092	0.950786998	protein_coding	7	150264365	150271041	GIMAP4
ENSG00000133597	7.686472517	7.392265003	protein_coding	7	140372953	140396061	ADCK2
ENSG00000133606	10.05696733	9.827226448	protein_coding	7	140152840	140179369	MKRN1
ENSG00000133612	10.73523103	10.64136335	protein_coding	7	150782918	150841523	AGAP3
ENSG00000133619	8.004679484	8.5924114	protein_coding	7	149411872	149431664	KRBA1
ENSG00000133624	6.095241663	6.758710666	pseudogene	7	149244245	149321843	ZNF767
ENSG00000133627	7.242608348	6.628734426	protein_coding	7	152456834	152552463	ACTR3B
ENSG00000133639	9.101836412	9.122401836	protein_coding	12	92536286	92539673	BTG1
ENSG00000133640	2.671945279	2.851901313	protein_coding	12	85430092	85657002	LRRIQ1
ENSG00000133641	7.568924112	7.922602475	protein_coding	12	88427623	88443937	C12orf29
ENSG00000133657	11.49373351	11.44435203	protein_coding	3	194123401	194219093	ATP13A3
ENSG00000133661	0.499066092	0.390640832	protein_coding	10	81697496	81742370	SFTPD
ENSG00000133665	1.16343121	2.144285137	protein_coding	10	82104501	82127829	DYDC2
ENSG00000133678	7.084651883	7.836897019	protein_coding	10	81838402	81852313	C10orf57
ENSG00000133687	0.499066092	0.697730409	protein_coding	12	29653757	29937692	TMTC1
ENSG00000133703	8.75650134	8.487570409	protein_coding	12	25357723	25403870	KRAS
ENSG00000133704	9.806352596	9.305130221	protein_coding	12	30781922	30848920	IPO8
ENSG00000133706	10.86366171	10.62554732	protein_coding	5	145492601	145562223	LARS
ENSG00000133710	5.054730169	5.306006045	protein_coding	5	147405246	147516852	SPINK5
ENSG00000133731	7.140657543	7.073814954	protein_coding	8	82570196	82598928	IMPA1
ENSG00000133739	6.931877181	6.110690132	protein_coding	8	86019323	86058315	LRRCC1
ENSG00000133740	6.400645076	6.398170093	protein_coding	8	86089460	86129387	E2F5
ENSG00000133742	0.499066092	0.390640832	protein_coding	8	86239837	86291243	CA1
ENSG00000133773	7.517784241	7.782918269	protein_coding	12	82617460	82752584	CCDC59
ENSG00000133789	9.222528249	9.197681135	protein_coding	11	9685624	9774538	SWAP70
ENSG00000133794	7.210761201	7.519971735	protein_coding	11	13298199	13408813	ARNTL
ENSG00000133800	0	0.390640832	protein_coding	11	10578513	10633236	LYVE1
ENSG00000133805	7.67484765	8.210017866	protein_coding	11	10329860	10529126	AMPD3
ENSG00000133808	5.316509574	4.048652948	protein_coding	11	12297627	12380691	MICALCL
ENSG00000133812	8.807962967	8.685795691	protein_coding	11	9800214	10315754	SBF2
ENSG00000133816	11.05488931	11.11965282	protein_coding	11	12115543	12285334	MICAL2
ENSG00000133818	9.692784215	9.847334256	protein_coding	11	14299472	14386052	RRAS2
ENSG00000133835	9.959126666	9.572977394	protein_coding	5	118788138	118878028	HSD17B4
ENSG00000133858	8.605481314	8.354088029	protein_coding	12	72003252	72061505	ZFC3H1
ENSG00000133863	8.448779323	8.588920207	protein_coding	8	30689060	30748122	TEX15
ENSG00000133872	9.661466274	10.04075742	protein_coding	8	29920528	29940723	TMEM66
ENSG00000133874	5.81716234	5.555337277	protein_coding	8	33405273	33424643	RNF122
ENSG00000133884	9.290757201	9.450079579	protein_coding	11	65101225	65120720	DPF2
ENSG00000133895	9.850940035	9.862353807	protein_coding	11	64570988	64578766	MEN1
ENSG00000133935	8.610066544	8.32500368	protein_coding	14	76116134	76127532	C14orf1
ENSG00000133943	7.743231983	6.866449614	protein_coding	14	91526677	91691703	C14orf159
ENSG00000133958	0.869158192	0.950786998	protein_coding	14	93799565	94174222	UNC79
ENSG00000133961	10.13274625	10.15219202	protein_coding	14	73741815	73930348	NUMB
ENSG00000133962	0.869158192	0.390640832	protein_coding	14	92047040	92247051	CATSPERB
ENSG00000133980	0.499066092	0.390640832	protein_coding	14	74769772	74826711	VRTN
ENSG00000133983	7.511262389	8.203944731	protein_coding	14	70792102	70826448	COX16
ENSG00000133985	4.842525946	5.317302248	protein_coding	14	71108504	71142077	TTC9
ENSG00000133997	8.066039442	7.926295939	protein_coding	14	71047974	71067384	MED6
ENSG00000134001	11.0328605	11.31085514	protein_coding	14	67826714	67853233	EIF2S1
ENSG00000134007	1.16343121	2.242360793	protein_coding	14	70989078	71001732	ADAM20
ENSG00000134013	12.07763287	12.8499187	protein_coding	8	23154702	23282841	LOXL2
ENSG00000134014	8.385999544	8.517261232	protein_coding	8	27947190	28048673	ELP3
ENSG00000134030	8.959377969	8.93803753	protein_coding	18	46065417	46389588	CTIF
ENSG00000134046	9.477150759	9.453922984	protein_coding	18	51677971	51751158	MBD2
ENSG00000134049	8.278730502	8.935290787	protein_coding	18	44681413	44702745	IER3IP1
ENSG00000134056	6.502803968	6.628734426	protein_coding	5	68513587	68525956	MRPS36
ENSG00000134057	10.74255805	10.58347835	protein_coding	5	68462837	68474072	CCNB1
ENSG00000134058	8.438503912	8.496293532	protein_coding	5	68530668	68573250	CDK7
ENSG00000134070	9.749993326	9.806834418	protein_coding	3	10206549	10285427	IRAK2

ENSG00000134072	7.169937448	7.782918269	protein_coding	3	9799026	9811676	CAMK1
ENSG00000134077	8.617676359	8.362291274	protein_coding	3	9404526	9428475	THUMPD3
ENSG00000134086	9.681936396	9.542017285	protein_coding	3	10182692	10193904	VHL
ENSG00000134107	11.54132321	10.4653237	protein_coding	3	5020801	5027008	BHLHE40
ENSG00000134108	9.528110879	9.678578614	protein_coding	3	5163905	5222596	ARL8B
ENSG00000134109	9.402463961	8.906597279	protein_coding	3	5229331	5261642	EDEM1
ENSG00000134138	7.413224588	7.287036562	protein_coding	15	37181409	37393504	MEIS2
ENSG00000134146	6.704935538	5.792353798	protein_coding	15	35509546	35838394	ATPBD4
ENSG00000134152	7.654275977	7.644032061	protein_coding	15	34432875	34502297	C15orf29
ENSG00000134153	8.05038018	8.552341023	protein_coding	15	34376218	34394149	C15orf24
ENSG00000134183	3.077135474	3.493416095	protein_coding	1	110145889	110155679	GNAT2
ENSG00000134186	9.345859976	9.389159064	protein_coding	1	109234945	109244425	PRPF38B
ENSG00000134198	2.928422289	2.971122874	protein_coding	1	115590632	115632121	TSPAN2
ENSG00000134201	0	0.950786998	protein_coding	1	110254877	110260888	GSTM5
ENSG00000134202	7.37062428	7.397596322	protein_coding	1	110276554	110284384	GSTM3
ENSG00000134207	0	0.390640832	protein_coding	1	114631914	114696541	SYT6
ENSG00000134215	4.883502971	4.508954154	protein_coding	1	108113782	108507766	VAV3
ENSG00000134222	7.800519667	7.973469823	protein_coding	1	109822178	109825808	PSRC1
ENSG00000134242	4.491309013	4.226940739	protein_coding	1	114356433	114414381	PTPN22
ENSG00000134243	6.236372259	8.117738058	protein_coding	1	109852192	109940573	SORT1
ENSG00000134245	3.652182994	3.935919592	protein_coding	1	113009163	113065288	WNT2B
ENSG00000134247	9.046529371	7.714077892	protein_coding	1	117452679	117532980	PTGFRN
ENSG00000134248	7.746012174	8.443145654	protein_coding	1	110943871	110950564	HBXIP
ENSG00000134250	11.15267838	11.16724979	protein_coding	1	120454176	120612240	NOTCH2
ENSG00000134253	5.239686603	5.893930351	protein_coding	1	117653682	117665209	TRIM45
ENSG00000134255	7.562629889	7.733180651	protein_coding	1	111682249	111727724	CEPT1
ENSG00000134256	1.407729925	0.390640832	protein_coding	1	117544382	117579167	CD101
ENSG00000134258	0	1.16600992	protein_coding	1	117686209	117753556	VTCN1
ENSG00000134259	3.744048221	5.496926774	protein_coding	1	115828539	115880857	NGF
ENSG00000134262	6.989320791	7.423961341	protein_coding	1	114437370	114447823	AP4B1
ENSG00000134265	8.393111514	8.286726732	protein_coding	18	10525873	10552762	NAPG
ENSG00000134278	9.680483837	9.339469783	protein_coding	18	12446511	12658133	SPIRE1
ENSG00000134283	9.280246895	9.358048031	protein_coding	12	42632249	42853517	PPHLN1
ENSG00000134285	8.565120083	8.306700762	protein_coding	12	49315301	49320257	FKBP11
ENSG00000134287	10.56423086	10.06063822	protein_coding	12	49297286	49351334	ARF3
ENSG00000134291	8.833021386	9.443006635	protein_coding	12	48357352	48362661	TMEM106C
ENSG00000134294	12.20584638	11.88961056	protein_coding	12	46751972	46766650	SLC38A2
ENSG00000134297	5.613019223	5.57429348	pseudogene	12	45566847	45609824	PLEKHA8P1
ENSG00000134308	12.09518276	11.54282244	protein_coding	2	9724101	9771143	YWHAQ
ENSG00000134313	8.981959173	8.547552846	protein_coding	2	8865408	8977760	KIDINS220
ENSG00000134317	5.707331524	5.187895122	protein_coding	2	10085341	10142411	GRHL1
ENSG00000134318	9.529725156	8.891561392	protein_coding	2	11319887	11488456	ROCK2
ENSG00000134321	7.222786417	6.984522608	protein_coding	2	7005937	7038370	RSAD2
ENSG00000134324	9.708548245	8.540340653	protein_coding	2	11817721	11967535	LPIN1
ENSG00000134326	6.670228606	6.25865215	protein_coding	2	6980701	7006766	CMPK2
ENSG00000134330	7.925957035	7.568048738	protein_coding	2	9613787	9636672	IAH1
ENSG00000134333	14.27775723	14.66120029	protein_coding	11	18415935	18429972	LDHA
ENSG00000134339	7.178195136	6.530239731	protein_coding	11	18260770	18270190	SAA2
ENSG00000134352	10.81682972	10.81651536	protein_coding	5	55230923	55290772	IL6ST
ENSG00000134363	4.230497448	1.16600992	protein_coding	5	52776239	52782964	FST
ENSG00000134369	9.38294884	8.69123279	protein_coding	1	201592411	201796102	NAV1
ENSG00000134371	8.646233023	8.820898829	protein_coding	1	193091147	193223031	CDC73
ENSG00000134375	9.019209518	9.057364358	protein_coding	1	201924619	201939789	TIMM17A
ENSG00000134376	0	0.697730409	protein_coding	1	197170592	197447585	CRB1
ENSG00000134398	1.407729925	1.16600992	protein_coding	16	23701647	23724821	ERN2
ENSG00000134419	9.12764907	9.750655873	protein_coding	16	18636822	18801705	RPS15A
ENSG00000134440	10.73032559	11.01210492	protein_coding	18	55267896	55289177	NARS
ENSG00000134443	0	0.390640832	protein_coding	18	56887400	56898006	GRP
ENSG00000134444	8.267164507	8.180940029	protein_coding	18	59854507	59974355	KIAA1468
ENSG00000134452	9.43383913	9.08571528	protein_coding	10	5931535	5979556	FBXO18
ENSG00000134453	9.847060811	9.673093524	protein_coding	10	6130949	6159420	RBM17
ENSG00000134460	0	0.697730409	protein_coding	10	6052652	6104333	IL2RA
ENSG00000134461	5.929039784	5.32851069	protein_coding	10	5903689	5931869	ANKRD16

ENSG00000134463	7.627388205	7.019522872	protein_coding	10	11784365	11806069	ECHDC3
ENSG00000134470	9.000704568	8.405271149	protein_coding	10	5991038	6020150	IL15RA
ENSG00000134480	8.421213901	8.322202916	protein_coding	5	86687311	86708836	CCNH
ENSG00000134489	1.407729925	0.950786998	protein_coding	18	22040593	22059921	HRH4
ENSG00000134490	7.420203928	6.525377513	protein_coding	18	20777719	21017925	TMEM241
ENSG00000134504	7.712290964	7.819127564	protein_coding	18	24034874	24209206	KCTD1
ENSG00000134508	7.89873181	6.95589788	protein_coding	18	20714528	20840434	CABLES1
ENSG00000134516	1.407729925	2.420525079	protein_coding	5	169064251	169510386	DOCK2
ENSG00000134531	9.06334524	7.985911283	protein_coding	12	13349650	13369708	EMP1
ENSG00000134533	6.138202758	6.16171561	protein_coding	12	15260717	15501609	RERG
ENSG00000134539	0.499066092	0.390640832	protein_coding	12	10378657	10469850	KLRD1
ENSG00000134545	3.004694206	1.925536307	protein_coding	12	10594863	10607284	KLRC1
ENSG00000134548	2.471521042	2.144285137	protein_coding	12	21679241	21690311	C12orf39
ENSG00000134551	0.499066092	0.697730409	protein_coding	12	11081835	11086368	PRH2
ENSG00000134569	5.389447744	5.059245468	protein_coding	11	46878419	46940193	LRP4
ENSG00000134571	0	0.390640832	protein_coding	11	47352957	47374253	MYBPC3
ENSG00000134574	8.219950371	8.149685112	protein_coding	11	47236493	47260767	DDB2
ENSG00000134575	8.64474405	9.002437654	protein_coding	11	47260853	47270457	ACP2
ENSG00000134590	9.882235894	10.34112548	protein_coding	X	134166333	134167576	FAM127A
ENSG00000134594	2.10647801	1.802319292	protein_coding	X	129305623	129318844	RAB33A
ENSG00000134597	7.527512071	7.507697912	protein_coding	X	129535943	129547317	RBMX2
ENSG00000134627	3.78787645	4.250702764	protein_coding	11	94277006	94354587	PIWIL4
ENSG00000134644	9.957928125	9.913491433	protein_coding	1	31404353	31538838	PUM1
ENSG00000134668	7.356139979	7.510161044	protein_coding	1	32256023	32281652	SPOCD1
ENSG00000134684	11.50115796	11.47257006	protein_coding	1	33240840	33283754	YARS
ENSG00000134686	9.96986896	10.01627318	protein_coding	1	33789224	33896653	PHC2
ENSG00000134690	9.243337861	9.240587696	protein_coding	1	38158090	38175391	CDCA8
ENSG00000134697	9.49130376	9.540210691	protein_coding	1	38032417	38061540	GNL2
ENSG00000134698	7.003331296	7.053694091	protein_coding	1	36273773	36323491	EIF2C4
ENSG00000134709	4.593635215	0.390640832	protein_coding	1	60280458	60342050	HOOK1
ENSG00000134716	2.35979773	1.518964905	protein_coding	1	60358980	60392462	CYP2J2
ENSG00000134717	7.911170638	8.073455769	protein_coding	1	52521797	52556388	BTF3L4
ENSG00000134744	8.741255403	9.097229165	protein_coding	1	52873954	53019159	ZCCHC11
ENSG00000134748	8.292108003	8.363653959	protein_coding	1	52870236	52883992	PRPF38A
ENSG00000134755	6.704935538	4.841813558	protein_coding	18	28645940	28682378	DSC2
ENSG00000134758	8.209916558	8.260632671	protein_coding	18	29671828	29711524	RNF138
ENSG00000134759	9.172772397	9.229419632	protein_coding	18	33709407	33757909	ELP2
ENSG00000134762	0.499066092	0	protein_coding	18	28570052	28622781	DSC3
ENSG00000134765	0	0.390640832	protein_coding	18	28709199	28742819	DSC1
ENSG00000134769	4.352265886	7.786986696	protein_coding	18	32073254	32471808	DTNA
ENSG00000134775	8.723027268	7.720473624	protein_coding	18	33877702	34360018	FHOD3
ENSG00000134779	9.462857538	9.501740056	protein_coding	18	34376035	34409158	TPGS2
ENSG00000134780	7.413224588	7.512619979	protein_coding	11	61447905	61514473	DAGLA
ENSG00000134802	9.562425908	10.52500144	protein_coding	11	57174427	57195053	SLC43A3
ENSG00000134809	8.101206377	8.734007291	protein_coding	11	57295936	57298276	TIMM10
ENSG00000134812	0	0.697730409	protein_coding	11	59596741	59612974	GIF
ENSG00000134815	9.348606364	9.356680039	protein_coding	19	47852538	47889887	DHX34
ENSG00000134824	11.02200542	9.679673133	protein_coding	11	61583728	61634826	FADS2
ENSG00000134825	5.827699195	6.403479675	protein_coding	11	61556435	61560274	C11orf10
ENSG00000134827	3.393122761	1.667587519	protein_coding	11	59620273	59634048	TCN1
ENSG00000134830	0.499066092	0	protein_coding	19	47844040	47845272	GPR77
ENSG00000134851	9.248248601	9.627311386	protein_coding	4	56262124	56292339	TMEM165
ENSG00000134852	8.741255403	9.136021975	protein_coding	4	56294070	56413305	CLOCK
ENSG00000134864	3.871730003	4.406904905	protein_coding	13	101183801	101241782	A2LD1
ENSG00000134871	1.16343121	0.950786998	protein_coding	13	110958159	111165374	COL4A2
ENSG00000134873	1.16343121	0.697730409	protein_coding	13	96085858	96231906	CLDN10
ENSG00000134874	0.499066092	0.950786998	protein_coding	13	96230457	96296957	DZIP1
ENSG00000134882	9.259966624	9.041275118	protein_coding	13	99853028	100038688	UBAC2
ENSG00000134884	9.416492786	9.639175184	protein_coding	13	107194021	107220512	ARGLU1
ENSG00000134897	7.451199747	7.596144237	protein_coding	13	103451399	103493885	BIVM
ENSG00000134899	8.614637248	8.48506837	protein_coding	13	103459705	103528345	ERCC5
ENSG00000134900	9.035208904	8.924251218	protein_coding	13	103249353	103331521	TPP2
ENSG00000134901	7.084651883	7.797107849	protein_coding	13	103436631	103451357	KDELC1

ENSG00000134905	9.287898339	9.474880612	protein_coding	13	111290133	111365950	CARS2
ENSG00000134909	8.895705979	8.764244491	protein_coding	11	128834989	129149219	ARHGAP32
ENSG00000134910	11.87665589	12.07561565	protein_coding	11	125461607	125495110	STT3A
ENSG00000134917	0.499066092	1.353254395	protein_coding	11	130274820	130298888	ADAMTS8
ENSG00000134940	3.335220907	4.226940739	protein_coding	11	125541417	125551018	ACRV1
ENSG00000134954	11.04335201	10.79015471	protein_coding	11	128328656	128457437	ETS1
ENSG00000134955	7.837485466	6.866449614	protein_coding	11	124932963	124959131	SLC37A2
ENSG00000134962	2.35979773	1.667587519	protein_coding	4	39408473	39453156	KLB
ENSG00000134970	10.30052049	9.746483542	protein_coding	5	114949205	114968689	TMED7
ENSG00000134982	9.150911694	8.84060518	protein_coding	5	112043195	112181936	APC
ENSG00000134986	8.02999382	9.042977197	protein_coding	5	110998318	111333161	C5orf13
ENSG00000134987	9.761717462	9.416334062	protein_coding	5	110427414	110466200	WDR36
ENSG00000134996	8.371669553	8.191721221	protein_coding	9	77703459	77762181	OSTF1
ENSG00000135002	8.479173607	8.437980325	protein_coding	9	79000433	79009433	RFK
ENSG00000135018	11.2368649	10.9758086	protein_coding	9	86274878	86323118	UBQLN1
ENSG00000135040	8.647720461	8.794871435	protein_coding	9	88556061	88637213	NAA35
ENSG00000135045	7.326727684	8.159132889	protein_coding	9	77561497	77567802	C9orf40
ENSG00000135046	12.97410379	12.35226715	protein_coding	9	75766673	75785309	ANXA1
ENSG00000135047	10.18182545	10.15652241	protein_coding	9	90340434	90346308	CTSL1
ENSG00000135048	8.66103884	8.844514354	protein_coding	9	74298282	74431606	TMEM2
ENSG00000135049	8.472474444	8.792849743	protein_coding	9	88161455	88356944	AGTPBP1
ENSG00000135052	10.68178411	10.97179319	protein_coding	9	88641061	88715088	GOLM1
ENSG00000135063	5.83815965	6.811590016	protein_coding	9	71939488	72007371	FAM189A2
ENSG00000135069	11.16051428	11.22649291	protein_coding	9	80912043	80945009	PSAT1
ENSG00000135070	8.032273249	8.43019749	protein_coding	9	88879461	88897676	ISCA1
ENSG00000135074	2.847891871	1.16600992	protein_coding	5	156822542	157002783	ADAM19
ENSG00000135077	1.616589159	0	protein_coding	5	156512843	156569880	HAVCR2
ENSG00000135083	6.35766608	6.136428444	protein_coding	5	159678659	159772403	CCNJL
ENSG00000135090	8.764749894	8.005247922	protein_coding	12	118587606	118810750	TAOK3
ENSG00000135093	6.716321419	6.646756201	protein_coding	12	109460894	109525831	USP30
ENSG00000135094	2.671945279	2.144285137	protein_coding	12	113830250	113841693	SDS
ENSG00000135097	0	0.697730409	protein_coding	12	120779133	120806983	MSI1
ENSG00000135100	3.652182994	3.279072565	protein_coding	12	121416346	121442296	HNF1A
ENSG00000135108	8.649206367	8.458531597	protein_coding	12	117581146	117628336	FBXO21
ENSG00000135114	8.830404061	8.191721221	protein_coding	12	121458121	121477045	OASL
ENSG00000135116	2.35979773	3.32456471	protein_coding	12	117298932	117319232	HRK
ENSG00000135119	4.821592551	5.150490275	protein_coding	12	117176096	117291436	RNFT2
ENSG00000135124	7.663128351	8.125791388	protein_coding	12	121647660	121671909	P2RX4
ENSG00000135127	5.785082014	2.501982735	protein_coding	12	120427648	120532298	CCDC64
ENSG00000135144	2.238690726	1.925536307	protein_coding	12	113494514	113535833	DTX1
ENSG00000135148	9.31529953	8.245929405	protein_coding	12	112563305	112591407	TRAFD1
ENSG00000135164	9.230491154	9.189251987	protein_coding	7	86781677	86825646	DMTF1
ENSG00000135185	7.603059879	7.03670965	protein_coding	7	86825478	86849903	C7orf23
ENSG00000135205	3.211941663	3.532712221	protein_coding	7	76751751	76958850	CCDC146
ENSG00000135211	7.2307479	6.930376707	protein_coding	7	77423045	77427897	TMEM60
ENSG00000135213	9.034071956	8.120964787	protein_coding	7	75046066	75115548	POM121C
ENSG00000135218	0.499066092	0.697730409	protein_coding	7	79998891	80308593	CD36
ENSG00000135241	8.631273684	8.5924114	protein_coding	7	108110866	108210110	PNPLA8
ENSG00000135245	7.246540262	7.192052139	protein_coding	7	128095903	128098472	HILPDA
ENSG00000135248	1.16343121	0.950786998	protein_coding	7	128349115	128371677	FAM71F1
ENSG00000135249	7.813830732	7.714077892	protein_coding	7	105172532	105208124	RINT1
ENSG00000135250	9.165522239	9.049765501	protein_coding	7	104751151	105039755	SRPK2
ENSG00000135253	2.762599152	1.16600992	processed_transc	7	128502505	128550773	KCP
ENSG00000135269	10.61757819	9.800811083	protein_coding	7	115850547	115898837	TES
ENSG00000135272	7.062518763	7.161039629	protein_coding	7	114562209	114659256	MDFIC
ENSG00000135297	7.64536895	7.709798264	protein_coding	6	74171301	74218959	MTO1
ENSG00000135299	3.652182994	4.622587519	protein_coding	6	90142889	90343553	ANKRD6
ENSG00000135314	5.301468762	5.545764946	protein_coding	6	73951037	74020088	KHDC1
ENSG00000135315	6.313367288	6.668971382	protein_coding	6	84833960	84937353	KIAA1009
ENSG00000135316	11.13316588	10.94791747	protein_coding	6	86267696	86353510	SYNCRIP
ENSG00000135317	8.701704574	8.908465794	protein_coding	6	86215214	86303874	SNX14
ENSG00000135318	8.936437694	8.385283657	protein_coding	6	86159302	86205500	NT5E
ENSG00000135324	0.499066092	0.390640832	protein_coding	6	84743475	84800600	MRAP2

ENSG00000135334	8.5900911	8.407915348	protein_coding	6	88384790	88411927	AKIRIN2
ENSG00000135336	8.081530559	8.341695034	protein_coding	6	88299839	88377169	ORC3
ENSG00000135338	4.923348194	4.932754837	protein_coding	6	80194708	80247175	LCA5
ENSG00000135341	8.935220147	9.236874606	protein_coding	6	91223292	91296786	MAP3K7
ENSG00000135346	0	0.950786998	protein_coding	6	87795216	87804824	CGA
ENSG00000135362	6.861834435	6.99159092	protein_coding	11	36317838	36486754	PRR5L
ENSG00000135363	2.847891871	2.579085888	protein_coding	11	33880122	33913836	LMO2
ENSG00000135365	9.092036341	9.496159541	protein_coding	11	45950871	46142985	PHF21A
ENSG00000135372	10.56973371	10.48830548	protein_coding	11	34127149	34169217	NAT10
ENSG00000135373	7.434062125	3.133298822	protein_coding	11	34642640	34682604	EHF
ENSG00000135374	0.869158192	0	protein_coding	11	34500340	34535352	ELF5
ENSG00000135378	6.482942724	5.776071827	protein_coding	11	32851489	32879669	PRRG4
ENSG00000135387	11.76630206	11.76340464	protein_coding	11	34073230	34122703	CAPRIN1
ENSG00000135390	10.66425427	11.21275951	protein_coding	12	54026510	54071192	ATP5G2
ENSG00000135392	7.983632192	7.902117654	protein_coding	12	56214744	56224608	DNAJC14
ENSG00000135404	11.20942892	11.84323163	protein_coding	12	56119107	56123491	CD63
ENSG00000135406	2.35979773	2.912743273	protein_coding	12	49687035	49692465	PRPH
ENSG00000135407	5.763291843	5.726093467	protein_coding	12	58191159	58212487	AVIL
ENSG00000135409	0	0.390640832	protein_coding	12	53817639	53825318	AMHR2
ENSG00000135414	7.706593329	7.436965343	protein_coding	12	56137064	56150911	GDF11
ENSG00000135423	5.498982484	4.676215056	protein_coding	12	56864736	56882198	GLS2
ENSG00000135424	4.026095388	4.363974406	protein_coding	12	56078352	56109827	ITGA7
ENSG00000135436	3.989024711	3.96493948	protein_coding	12	49976668	49999422	FAM186B
ENSG00000135437	2.671945279	3.081239798	protein_coding	12	56114151	56118489	RDH5
ENSG00000135439	2.471521042	1.802319292	protein_coding	12	58118981	58135940	AGAP2
ENSG00000135441	5.316509574	6.376734172	protein_coding	12	56109820	56113871	BLOC1S1
ENSG00000135443	0.499066092	0.390640832	protein_coding	12	52753790	52761265	KRT85
ENSG00000135446	10.2558666	10.57409153	protein_coding	12	58142034	58149796	CDK4
ENSG00000135451	9.287898339	9.55995988	protein_coding	12	49717019	49725514	TROAP
ENSG00000135452	7.609180529	7.397596322	protein_coding	12	58131796	58143994	TSPAN31
ENSG00000135454	8.66103884	8.351343215	protein_coding	12	58017193	58027138	B4GALNT1
ENSG00000135457	9.31342641	8.953504395	protein_coding	12	51487446	51566926	TFCP2
ENSG00000135469	5.977159882	6.881748114	protein_coding	12	56660642	56664750	COQ10A
ENSG00000135472	0.499066092	0.390640832	protein_coding	12	50260679	50298000	FAIM2
ENSG00000135473	9.332049062	9.852196378	protein_coding	12	56710007	56727837	PAN2
ENSG00000135476	9.887281568	9.952048633	protein_coding	12	53662083	53687427	ESPL1
ENSG00000135477	5.774228067	4.274079748	pseudogene	12	52644250	52652651	KRT121P
ENSG00000135480	12.78412844	12.61959647	protein_coding	12	52626304	52662180	KRT7
ENSG00000135482	5.346128795	5.893930351	protein_coding	12	56511943	56516278	ZC3H10
ENSG00000135486	10.68033139	10.89353271	protein_coding	12	54673977	54680872	HNRNPA1
ENSG00000135502	3.146112541	3.813624741	protein_coding	12	58013310	58019934	SLC26A10
ENSG00000135503	8.544507981	8.358195482	protein_coding	12	52345451	52390862	ACVR1B
ENSG00000135506	10.84238558	11.32875696	protein_coding	12	58087738	58115340	OS9
ENSG00000135517	0	0.697730409	protein_coding	12	56843286	56862950	MIP
ENSG00000135519	3.004694206	3.081239798	protein_coding	12	49932940	49952077	KCNH3
ENSG00000135521	8.05038018	8.234057939	protein_coding	6	144164481	144184949	LTV1
ENSG00000135525	6.62865285	2.788380093	protein_coding	6	136663875	136871792	MAP7
ENSG00000135535	10.86781572	11.08516875	protein_coding	6	109687717	109703762	CD164
ENSG00000135537	5.550781289	6.142791909	protein_coding	6	108616098	108847999	LACE1
ENSG00000135540	7.153278907	6.398170093	protein_coding	6	138743180	139013708	NHSL1
ENSG00000135541	7.144876945	7.666288907	protein_coding	6	135604670	135830219	AHI1
ENSG00000135547	1.16343121	1.16600992	protein_coding	6	126068810	126082415	HEY2
ENSG00000135549	3.004694206	2.851901313	protein_coding	6	122793062	123047518	PKIB
ENSG00000135577	0.499066092	0.950786998	protein_coding	6	142379467	142409936	NMBR
ENSG00000135587	6.877132514	6.799557986	protein_coding	6	109761966	109765122	SMPD2
ENSG00000135596	8.435062447	8.696649474	protein_coding	6	109765265	109787171	MICAL1
ENSG00000135597	8.78516728	8.331981894	protein_coding	6	139224630	139309398	REPS1
ENSG00000135604	5.525114348	6.445267191	protein_coding	6	144471663	144509507	STX11
ENSG00000135605	5.795854912	6.198834244	protein_coding	4	48137800	48271881	TEC
ENSG00000135617	6.489593565	7.18590274	protein_coding	2	73455134	73460366	PRADC1
ENSG00000135622	7.093410923	7.568048738	protein_coding	2	74881355	74909186	SEMA4F
ENSG00000135624	11.43421439	11.68108834	protein_coding	2	73460548	73480149	CCT7
ENSG00000135625	3.077135474	0.697730409	protein_coding	2	73518058	73520833	EGR4

ENSG00000135631	9.072234198	9.138412238	protein_coding	2	73300510	73383849	RAB11FIP5
ENSG00000135632	9.189208632	8.825850709	protein_coding	2	73441350	73454365	SMYD5
ENSG00000135636	9.045401313	8.914056863	protein_coding	2	71680852	71913898	DYSF
ENSG00000135637	6.305850085	6.16171561	protein_coding	2	74699113	74710535	CCDC142
ENSG00000135638	1.799000381	1.667587519	protein_coding	2	73143176	73162020	EMX1
ENSG00000135643	5.83815965	4.101879561	protein_coding	12	70760056	70828072	KCNMB4
ENSG00000135655	9.291708897	9.484933332	protein_coding	12	62654119	62811211	USP15
ENSG00000135677	10.38454676	10.87501876	protein_coding	12	65107225	65153227	GNS
ENSG00000135678	7.186405829	5.496926774	protein_coding	12	69235977	69365350	CPM
ENSG00000135679	8.721615518	8.531880603	protein_coding	12	69201956	69239214	MDM2
ENSG00000135686	9.309672858	8.825850709	protein_coding	16	84682131	84701292	KLHL36
ENSG00000135697	0.499066092	0.390640832	protein_coding	16	81272053	81324747	BCMO1
ENSG00000135698	7.952677993	8.053329862	protein_coding	16	82181403	82203831	MPHOSPH6
ENSG00000135702	2.671945279	2.721932731	protein_coding	16	75562433	75569145	CHST5
ENSG00000135709	7.609180529	7.122922611	protein_coding	16	85061375	85127836	KIAA0513
ENSG00000135720	10.12689227	10.46310061	protein_coding	16	66754796	66785701	DYNC1LI2
ENSG00000135722	5.741167503	5.9163892	protein_coding	16	67193834	67199718	FBXL8
ENSG00000135723	9.92641136	9.729672494	protein_coding	16	67263290	67281561	FHOD1
ENSG00000135736	6.830742851	7.632773539	protein_coding	16	57546090	57570511	CCDC102A
ENSG00000135740	6.407685389	6.510691757	protein_coding	16	67271586	67306093	SLC9A5
ENSG00000135744	3.989024711	5.47691899	protein_coding	1	230838269	230850043	AGT
ENSG00000135747	5.909333162	6.252780742	protein_coding	1	247108849	247242113	ZNF670
ENSG00000135749	8.251597515	8.616616024	protein_coding	1	233119881	233431459	PCNXL2
ENSG00000135750	0.499066092	0	protein_coding	1	233749750	233808258	KCNK1
ENSG00000135763	8.903197794	8.78167951	protein_coding	1	229761981	229795946	URB2
ENSG00000135766	9.59285838	9.987393954	protein_coding	1	231499497	231560790	EGLN1
ENSG00000135773	0.869158192	0.697730409	protein_coding	1	230883130	230937749	CAPN9
ENSG00000135775	8.344411036	8.410554709	protein_coding	1	230778235	230829728	COG2
ENSG00000135776	8.18144485	8.581912372	protein_coding	1	229652329	229694442	ABCB10
ENSG00000135778	8.484177644	8.702045897	protein_coding	1	233086351	233114548	NTPCR
ENSG00000135801	8.510576602	8.511125832	protein_coding	1	229728858	229761794	TAF5L
ENSG00000135821	8.455589162	8.013952351	protein_coding	1	182350839	182361341	GLUL
ENSG00000135823	8.223944437	8.133800013	protein_coding	1	180941861	180992047	STX6
ENSG00000135828	5.191549282	5.57429348	protein_coding	1	182542769	182558391	RNASEL
ENSG00000135829	11.41403475	11.59567532	protein_coding	1	182808439	182856886	DHX9
ENSG00000135835	4.464551814	4.17820932	protein_coding	1	180882290	180920750	KIAA1614
ENSG00000135837	8.3352092	8.779639234	protein_coding	1	179923873	180084015	CEP350
ENSG00000135838	2.928422289	4.903073497	protein_coding	1	182758428	182799519	NPL
ENSG00000135842	9.019209518	9.197681135	protein_coding	1	184759858	184943682	FAM129A
ENSG00000135845	7.289098491	7.728957399	protein_coding	1	172339329	172413230	PIGC
ENSG00000135847	8.027710784	8.058387721	protein_coding	1	180244515	180472089	ACBD6
ENSG00000135862	11.77193965	11.85841456	protein_coding	1	182992597	183114727	LAMC1
ENSG00000135870	8.164919416	8.159132889	protein_coding	1	173900352	173991435	RC3H1
ENSG00000135898	0	0.697730409	protein_coding	2	231772033	231825781	GPR55
ENSG00000135899	7.821758815	8.101495266	protein_coding	2	231032009	231090444	SP110
ENSG00000135900	8.223944437	7.955508102	protein_coding	2	224822121	224832431	MRPL44
ENSG00000135902	2.847891871	2.144285137	protein_coding	2	233390703	233401377	CHRND
ENSG00000135905	8.105542576	8.260632671	protein_coding	2	225629807	225907162	DOCK10
ENSG00000135912	9.237422823	8.962525691	protein_coding	2	219575568	219620139	TTLL4
ENSG00000135913	7.326727684	6.787424764	protein_coding	2	219314974	219433084	USP37
ENSG00000135914	0.869158192	0.697730409	protein_coding	2	231972944	231989832	HTR2B
ENSG00000135916	9.861233924	9.788181112	protein_coding	2	231729354	231743963	ITM2C
ENSG00000135917	5.538004898	4.94736961	protein_coding	2	228549926	228582728	SLC19A3
ENSG00000135919	0.499066092	0.950786998	protein_coding	2	224839829	224904036	SERPINE2
ENSG00000135924	8.750975986	9.01636662	protein_coding	2	220143989	220151622	DNAJB2
ENSG00000135925	5.91922012	6.495854971	protein_coding	2	219745085	219764303	WNT10A
ENSG00000135926	10.67996799	10.37834106	protein_coding	2	219138915	219157309	TMBIM1
ENSG00000135929	5.806547963	5.824376633	protein_coding	2	219646472	219680016	CYP27A1
ENSG00000135930	9.644431499	9.365548888	protein_coding	2	233414762	233448354	EIF4E2
ENSG00000135931	8.059349131	8.044860491	protein_coding	2	232063260	232239548	ARMC9
ENSG00000135932	10.6991035	10.13633465	protein_coding	2	231577560	231685790	CAB39
ENSG00000135940	9.645920794	9.769283465	protein_coding	2	98262503	98264846	COX5B
ENSG00000135945	8.322227223	7.991210667	protein_coding	2	100016938	100106497	REV1

ENSG00000135951	4.062237333	3.714580548	protein_coding	2	99613724	99771427	TSGA10
ENSG00000135953	7.132181536	6.495854971	protein_coding	2	103332299	103353347	MFSD9
ENSG00000135956	9.633963159	8.866788836	protein_coding	2	96916312	96931732	TMEM127
ENSG00000135960	0.869158192	0	protein_coding	2	109510927	109605828	EDAR
ENSG00000135966	8.507303029	7.996490656	protein_coding	2	105880871	105946491	TGFBRAP1
ENSG00000135968	8.265227794	8.124184315	protein_coding	2	109065017	109125871	GCC2
ENSG00000135972	8.249639777	8.012215662	protein_coding	2	105654441	105716418	MRPS9
ENSG00000135973	0	1.518964905	protein_coding	2	105858200	105859924	GPR45
ENSG00000135974	7.461385447	7.198175438	protein_coding	2	105953816	105965668	C2orf49
ENSG00000135976	5.958104008	5.840125386	protein_coding	2	97779233	97930258	ANKRD36
ENSG00000135999	7.262161623	7.295628905	protein_coding	2	149402009	149545130	EPC2
ENSG00000136002	8.209916558	6.505763096	protein_coding	2	131594489	131804836	ARHGEF4
ENSG00000136003	8.774313977	9.267771518	protein_coding	12	108956358	108963160	ISCU
ENSG00000136010	8.586993258	8.98128876	protein_coding	12	105413568	105478355	ALDH1L2
ENSG00000136011	1.960915222	2.039052734	protein_coding	12	103981051	104160505	STAB2
ENSG00000136014	0.499066092	0.697730409	protein_coding	12	95910336	95945266	USP44
ENSG00000136021	9.001868107	8.955313173	protein_coding	12	100660918	100735502	SCYL2
ENSG00000136026	11.25600812	11.43660358	protein_coding	12	106631655	106698057	CKAP4
ENSG00000136040	4.517578978	4.794098771	protein_coding	12	94542499	94701451	PLXNC1
ENSG00000136044	7.50471092	7.733180651	protein_coding	12	105567074	105630016	APPL2
ENSG00000136045	9.44329146	9.53356709	protein_coding	12	108079509	108106944	PWP1
ENSG00000136048	8.231899551	8.565427152	protein_coding	12	102271129	102405908	DRAM1
ENSG00000136051	8.879339004	9.10783897	protein_coding	12	105501102	105562912	KIAA1033
ENSG00000136052	8.235860718	8.44700755	protein_coding	12	105196331	105352522	SLC41A2
ENSG00000136059	4.261916566	4.101879561	protein_coding	3	38029550	38048679	VILL
ENSG00000136068	13.12449481	12.82527993	protein_coding	3	57994127	58157982	FLNB
ENSG00000136098	5.550781289	4.693656882	protein_coding	13	52706775	52733996	NEK3
ENSG00000136100	7.273767634	7.258020107	protein_coding	13	52986839	53024763	VPS36
ENSG00000136104	6.998676229	6.712401119	protein_coding	13	51483814	51544592	RNASEH2B
ENSG00000136108	9.043142548	8.735060572	protein_coding	13	53029495	53050485	CKAP2
ENSG00000136110	0.499066092	0	protein_coding	13	53277399	53313947	LECT1
ENSG00000136111	9.421718623	8.508664347	protein_coding	13	75858808	76056250	TBC1D4
ENSG00000136114	3.871730003	3.411459265	protein_coding	13	52951305	52980629	THSD1
ENSG00000136122	6.738827102	6.310446475	protein_coding	13	73302061	73330318	BORA
ENSG00000136141	6.970425921	6.434933235	protein_coding	13	47127303	47325710	LRCH1
ENSG00000136143	8.032273249	7.524852175	protein_coding	13	48510622	48612125	SUCLA2
ENSG00000136144	7.50471092	6.866449614	protein_coding	13	50106082	50159719	RCBTB1
ENSG00000136146	6.877132514	6.460630645	protein_coding	13	48627459	48669267	MED4
ENSG00000136147	6.897280837	6.95589788	protein_coding	13	50069746	50103123	PHF11
ENSG00000136149	1.407729925	2.039052734	pseudogene	13	55014839	55015450	RPL13AP25
ENSG00000136152	7.590740122	6.881748114	protein_coding	13	46039060	46110765	COG3
ENSG00000136153	7.136425764	5.908941696	protein_coding	13	76194570	76434004	LMO7
ENSG00000136155	6.469548347	0	protein_coding	13	78109809	78219398	SCEL
ENSG00000136156	9.622662143	9.432007055	protein_coding	13	48807294	48837063	ITM2B
ENSG00000136158	7.878603882	7.423961341	protein_coding	13	80910111	80915086	SPRY2
ENSG00000136159	7.140657543	6.577981353	protein_coding	13	48611703	48621282	NUDT15
ENSG00000136161	4.261916566	4.932754837	protein_coding	13	49063095	49109872	RCBTB2
ENSG00000136167	7.444369108	8.440565301	protein_coding	13	46700061	46786006	LCP1
ENSG00000136169	6.146643566	5.611474642	protein_coding	13	50018429	50069138	SETDB2
ENSG00000136193	11.30467048	11.04305224	protein_coding	7	29959719	30029905	SCRN1
ENSG00000136197	1.16343121	1.353254395	protein_coding	7	42948872	42951904	C7orf25
ENSG00000136205	11.32107712	10.56907983	protein_coding	7	47314752	47622156	TNS3
ENSG00000136206	2.471521042	2.334191469	protein_coding	7	44040488	44049721	SPDYE1
ENSG00000136213	7.624369505	7.65742733	protein_coding	7	2443223	2474242	CHST12
ENSG00000136231	8.723027268	8.027771115	protein_coding	7	23349828	23510086	IGF2BP3
ENSG00000136235	2.57521082	4.622587519	protein_coding	7	23275586	23314727	GPNMB
ENSG00000136237	5.785082014	2.039052734	protein_coding	7	22157909	22396763	RAPGEF5
ENSG00000136238	11.81345084	11.25412478	protein_coding	7	6414154	6443608	RAC1
ENSG00000136240	11.77211014	11.32000931	protein_coding	7	6485584	6523873	KDELR2
ENSG00000136243	8.508940744	8.507432028	protein_coding	7	23221446	23240630	NUPL2
ENSG00000136244	11.27682442	9.513450729	protein_coding	7	22765503	22771621	IL6
ENSG00000136247	8.721615518	8.739266022	protein_coding	7	6617065	6629005	ZDHHC4
ENSG00000136250	7.214780754	6.89311646	protein_coding	7	36552456	36764154	AOAH

ENSG00000136261	10.89718911	10.43971049	protein_coding	7	16685756	16746148	BZW2
ENSG00000136270	10.80087503	10.66200066	protein_coding	7	45139699	45151646	TBRG4
ENSG00000136271	10.45505461	10.2490122	protein_coding	7	44605016	44614650	DDX56
ENSG00000136273	7.624369505	7.41347308	protein_coding	7	47735328	48019178	HUS1
ENSG00000136274	3.146112541	1.925536307	protein_coding	7	45120037	45128513	NACAD
ENSG00000136275	0.869158192	0	protein_coding	7	47834889	47859428	C7orf69
ENSG00000136279	10.63792779	10.5837707	protein_coding	7	44084232	44109055	DBNL
ENSG00000136280	9.602094519	9.22117442	protein_coding	7	45039074	45116068	CCM2
ENSG00000136286	2.671945279	2.144285137	protein_coding	7	45002261	45018697	MYO1G
ENSG00000136295	12.02595418	12.07675852	protein_coding	7	2671585	2704436	TTYH3
ENSG00000136315	0.499066092	0	lincRNA	14	21387500	21388458	
ENSG00000136319	6.502803968	6.815578497	protein_coding	14	20754387	20774153	TTC5
ENSG00000136327	4.409500985	3.96493948	protein_coding	14	37049216	37051812	NKX2-8
ENSG00000136352	4.903563137	3.453019579	protein_coding	14	36985602	36990354	NKX2-1
ENSG00000136367	5.613019223	6.803579823	protein_coding	14	23990069	24025401	ZFHX2
ENSG00000136371	4.292665995	4.508954154	protein_coding	15	80125927	80189721	MTHFS
ENSG00000136378	4.097495944	4.826083323	protein_coding	15	79051545	79103805	ADAMTS7
ENSG00000136379	8.094677541	7.619146302	protein_coding	15	80972025	81047962	FAM108C1
ENSG00000136381	9.154055005	9.138412238	protein_coding	15	78729773	78793798	IREB2
ENSG00000136383	5.763291843	5.112089758	protein_coding	15	85359911	85416713	ALPK3
ENSG00000136404	3.78787645	3.279072565	protein_coding	15	83776159	83813606	TM6SF1
ENSG00000136425	3.146112541	2.501982735	protein_coding	15	78396948	78423886	CIB2
ENSG00000136436	9.458626558	9.23613084	protein_coding	17	46908350	46943884	CALCOCO2
ENSG00000136444	8.179389493	8.530667964	protein_coding	17	48556161	48563336	RSAD1
ENSG00000136448	10.60461664	10.34147131	protein_coding	17	43138680	43186377	NMT1
ENSG00000136449	2.471521042	1.16600992	protein_coding	17	48585745	48608862	MYCBPAP
ENSG00000136450	11.56355738	11.74456975	protein_coding	17	56078284	56084707	SRSF1
ENSG00000136451	9.669542781	9.763100953	protein_coding	17	56048910	56065615	VEZF1
ENSG00000136457	2.671945279	1.925536307	protein_coding	17	48541857	48546327	CHAD
ENSG00000136463	8.597806729	8.886830358	protein_coding	17	61678231	61685724	TACO1
ENSG00000136478	9.016909338	9.742822827	protein_coding	17	62224796	62340653	TEX2
ENSG00000136485	10.69443332	10.730936	protein_coding	17	61627822	61671639	DCAF7
ENSG00000136490	6.616550626	5.98174124	protein_coding	17	61773250	61777519	LIMD2
ENSG00000136492	7.913645586	8.800919568	protein_coding	17	59759985	59940755	BRIP1
ENSG00000136504	8.646233023	8.487570409	protein_coding	17	47865917	47906458	KAT7
ENSG00000136514	3.004694206	3.183544561	protein_coding	3	187086120	187089864	RTP4
ENSG00000136518	9.721319186	9.705158168	protein_coding	3	179280668	179306196	ACTL6A
ENSG00000136521	8.465744027	8.571336379	protein_coding	3	179322478	179345435	NDUFB5
ENSG00000136522	8.435062447	8.678149164	protein_coding	3	179306073	179322442	MRPL47
ENSG00000136527	10.89186426	10.76356151	protein_coding	3	185633694	185655924	TRA2B
ENSG00000136531	0.499066092	0	protein_coding	2	166095912	166248818	SCN2A
ENSG00000136536	9.11480047	8.899098923	protein_coding	2	160569000	160625359	7-Mar
ENSG00000136542	5.827699195	2.501982735	protein_coding	2	158114110	158170723	GALNT5
ENSG00000136560	7.824391858	8.076782959	protein_coding	2	161993419	162092732	TANK
ENSG00000136573	1.407729925	0.390640832	protein_coding	8	11351510	11422113	BLK
ENSG00000136603	8.576098123	8.382597612	protein_coding	3	170075466	170114623	SKIL
ENSG00000136628	11.87602149	12.15577074	protein_coding	1	220141943	220220000	EPRS
ENSG00000136630	4.292665995	4.566889334	protein_coding	1	221051699	221059124	HLX
ENSG00000136631	8.658089807	9.010289238	protein_coding	1	150039369	150117505	VPS45
ENSG00000136636	10.02584819	10.03478213	protein_coding	1	215740735	215795073	KCTD3
ENSG00000136643	8.495786671	8.625731057	protein_coding	1	213224589	213448116	RPS6KC1
ENSG00000136653	6.646617938	5.784235782	protein_coding	1	206680879	206762616	RASSF5
ENSG00000136682	5.649112675	5.840125386	protein_coding	2	114195268	114253766	CBWD2
ENSG00000136688	0.499066092	0.950786998	protein_coding	2	113730780	113743220	IL36G
ENSG00000136689	2.10647801	2.501982735	protein_coding	2	113864791	113891593	IL1RN
ENSG00000136695	0.499066092	0	protein_coding	2	113816215	113822325	IL36RN
ENSG00000136696	1.407729925	2.334191469	protein_coding	2	113779668	113810444	IL36B
ENSG00000136699	10.35753275	10.08645176	protein_coding	2	130908981	130940323	SMPD4
ENSG00000136709	10.02011733	9.622766057	protein_coding	2	128458596	128568761	WDR33
ENSG00000136710	7.304268146	7.295628905	protein_coding	2	131095814	131099922	CCDC115
ENSG00000136715	9.520012288	8.990138507	protein_coding	2	128698791	128785694	SAP130
ENSG00000136717	8.970118399	8.452140716	protein_coding	2	127805603	127864931	BIN1
ENSG00000136718	10.37245301	10.00452874	protein_coding	2	131099798	131105383	IMP4

ENSG00000136720	8.871721931	8.426290271	protein_coding	2	128994290	129076151	HS6ST1
ENSG00000136731	10.67596446	10.33523369	protein_coding	2	128848774	128953251	UGGT1
ENSG00000136732	1.407729925	0	protein_coding	2	127413509	127454246	GYPC
ENSG00000136738	9.003030709	8.958022094	protein_coding	10	17686124	17757913	STAM
ENSG00000136754	9.10941293	9.802319276	protein_coding	10	27035522	27150016	ABI1
ENSG00000136758	10.94636528	11.66942788	protein_coding	10	27399383	27444195	YME1L1
ENSG00000136770	8.484177644	8.866788836	protein_coding	10	22045466	22292698	DNAJC1
ENSG00000136783	6.640654398	7.258020107	protein_coding	9	107509969	107522403	NIPSNAP3A
ENSG00000136802	10.0287051	9.89711675	protein_coding	9	131644391	131680318	LRRC8A
ENSG00000136807	9.769253212	9.618206363	protein_coding	9	130547958	130553066	CDK9
ENSG00000136810	10.77456633	11.21219232	protein_coding	9	113006091	113018920	TXN
ENSG00000136811	9.990530519	9.892875904	protein_coding	9	131217466	131263239	ODF2
ENSG00000136813	10.92566986	11.05785962	protein_coding	9	114122972	114247025	KIAA0368
ENSG00000136816	8.886916071	8.931620308	protein_coding	9	132565432	132573560	TOR1B
ENSG00000136819	9.139327269	8.900977169	protein_coding	9	132589569	132598142	C9orf78
ENSG00000136824	10.09399102	10.25269422	protein_coding	9	106856541	106903698	SMC2
ENSG00000136826	9.498740691	8.327799018	protein_coding	9	110247133	110252763	KLF4
ENSG00000136827	8.772951555	9.032734472	protein_coding	9	132575222	132586413	TOR1A
ENSG00000136828	6.236372259	6.110690132	protein_coding	9	129677053	129985445	RALGPS1
ENSG00000136830	12.67274826	12.05941448	protein_coding	9	130267618	130341268	FAM129B
ENSG00000136840	9.955528053	9.492427169	protein_coding	9	130670114	130679317	ST6GALNAC4
ENSG00000136842	2.471521042	2.912743273	protein_coding	9	100263462	100364023	TMOD1
ENSG00000136848	9.64070152	9.37436355	protein_coding	9	124329336	124547809	DAB2IP
ENSG00000136854	9.006512903	8.663840043	protein_coding	9	130374540	130457460	STXBP1
ENSG00000136856	8.05038018	8.020878279	protein_coding	9	130159421	130170703	SLC2A8
ENSG00000136859	2.762599152	2.501982735	protein_coding	9	129849611	129885162	ANGPTL2
ENSG00000136861	9.475476535	9.474249985	protein_coding	9	123151147	123342448	CDK5RAP2
ENSG00000136866	6.146643566	5.9163892	protein_coding	9	115800660	115819039	ZFP37
ENSG00000136867	3.146112541	3.748356452	protein_coding	9	115913222	115926417	SLC31A2
ENSG00000136868	9.311550855	8.761145731	protein_coding	9	115983842	116029217	SLC31A1
ENSG00000136869	5.360713447	6.123616685	protein_coding	9	120466610	120479149	TLR4
ENSG00000136870	6.9512803	7.281279771	protein_coding	9	104161155	104172942	ZNF189
ENSG00000136874	7.402691859	7.309836726	protein_coding	9	102668915	102732618	STX17
ENSG00000136875	9.820929282	9.510993211	protein_coding	9	116037623	116055466	PRPF4
ENSG00000136877	10.17616765	9.700851959	protein_coding	9	130556876	130576606	FPGS
ENSG00000136878	8.962967004	8.96970233	protein_coding	9	132596977	132644107	USP20
ENSG00000136881	1.16343121	0.950786998	protein_coding	9	104122699	104145801	BAAT
ENSG00000136883	1.960915222	2.579085888	protein_coding	9	116848406	116861571	KIF12
ENSG00000136888	9.150911694	9.483053775	protein_coding	9	117350026	117360653	ATP6V1G1
ENSG00000136891	9.346776019	9.599822093	protein_coding	9	103064359	103115221	TEX10
ENSG00000136895	6.138202758	6.235021771	protein_coding	9	129986544	130155939	GARNL3
ENSG00000136897	8.301587982	8.344458247	protein_coding	9	104149915	104160896	MRPL50
ENSG00000136908	9.788933545	9.779018578	protein_coding	9	130697378	130700763	DPM2
ENSG00000136918	1.799000381	1.518964905	protein_coding	9	127615755	127620160	WDR38
ENSG00000136925	7.326727684	7.683850422	protein_coding	9	100362362	100395962	TSTD2
ENSG00000136928	0.499066092	0	protein_coding	9	101050391	101471479	GABBR2
ENSG00000136930	11.0713315	11.33380639	protein_coding	9	127115745	127177723	PSMB7
ENSG00000136931	0.869158192	0.697730409	protein_coding	9	127243516	127269709	NR5A1
ENSG00000136932	6.585843317	6.827478203	protein_coding	9	100666771	100684852	C9orf156
ENSG00000136933	8.565120083	8.563056668	protein_coding	9	127962821	127996437	RABEPK
ENSG00000136935	8.227927477	8.464894292	protein_coding	9	127640646	127710292	GOLGA1
ENSG00000136936	6.489593565	6.610484676	protein_coding	9	100437191	100459639	XPA
ENSG00000136937	9.984657349	10.02577254	protein_coding	9	100395908	100436030	NCBP1
ENSG00000136938	11.59338746	11.50981718	protein_coding	9	100745638	100778225	ANP32B
ENSG00000136939	0.499066092	0	protein_coding	9	125486269	125487204	OR1L4
ENSG00000136940	8.357195795	8.52337065	protein_coding	9	125560668	125590910	PDCL
ENSG00000136942	11.57650097	12.1284757	protein_coding	9	127620159	127624260	RPL35
ENSG00000136943	6.014532757	6.287656023	protein_coding	9	99794940	99801925	CTSL2
ENSG00000136944	1.616589159	1.518964905	protein_coding	9	129376722	129463311	LMX1B
ENSG00000136950	9.424324461	9.556984901	protein_coding	9	127624409	127640003	ARPC5L
ENSG00000136960	2.57521082	2.652276565	protein_coding	8	120569326	120685693	ENPP2
ENSG00000136982	7.2307479	7.495318771	protein_coding	8	120846216	120868250	DSCC1
ENSG00000136986	9.405104778	9.118371319	protein_coding	8	124025458	124054663	DERL1

ENSG00000136997	14.9449048	13.80212345	protein_coding	8	128747680	128753674	MYC
ENSG00000136999	5.827699195	7.532142019	protein_coding	8	120428546	120436593	NOV
ENSG00000137033	0.499066092	0.390640832	protein_coding	9	6215805	6257983	IL33
ENSG00000137038	6.693459083	7.57041104	protein_coding	9	7796490	7888380	C9orf123
ENSG00000137040	7.161632221	7.447284805	protein_coding	9	6011043	6015618	RANBP6
ENSG00000137054	8.872994239	8.827826712	protein_coding	9	37485932	37503694	POLR1E
ENSG00000137055	8.013936171	8.108014344	protein_coding	9	26904081	26947461	PLAA
ENSG00000137070	4.981125677	5.112089758	protein_coding	9	34650699	34661884	IL11RA
ENSG00000137073	9.841222348	9.52750079	protein_coding	9	33921691	34048947	UBAP2
ENSG00000137074	7.985986001	8.254769334	protein_coding	9	32972604	33025166	APTX
ENSG00000137075	7.46813618	7.348889996	protein_coding	9	36336393	36487545	RNF38
ENSG00000137076	12.33621351	12.51831426	protein_coding	9	35696945	35732392	TLN1
ENSG00000137077	0.869158192	0.697730409	protein_coding	9	34709002	34710121	CCL21
ENSG00000137094	7.15746161	7.679480043	protein_coding	9	34989638	34998897	DNAJB5
ENSG00000137098	1.960915222	3.183544561	protein_coding	9	35808042	35812269	SPAG8
ENSG00000137100	8.274885464	8.612036819	protein_coding	9	34613548	34620515	DCTN3
ENSG00000137101	1.799000381	1.925536307	protein_coding	9	35609530	35646807	CD72
ENSG00000137103	7.026383958	7.022976646	protein_coding	9	35814448	35854844	TMEM8B
ENSG00000137106	9.43383913	9.631842439	protein_coding	9	37422663	37436987	GRHPR
ENSG00000137124	10.37784053	10.11181153	protein_coding	9	38392661	38398658	ALDH1B1
ENSG00000137133	6.704935538	7.173524745	protein_coding	9	35812957	35815351	HINT2
ENSG00000137135	6.835971536	6.668971382	protein_coding	9	35658872	35675863	C9orf100
ENSG00000137142	3.698846687	5.816437314	protein_coding	9	38408991	38424444	IGFBPL1
ENSG00000137145	7.420203928	7.38691391	protein_coding	9	19288622	19374275	DENND4C
ENSG00000137154	11.85926789	12.39229118	protein_coding	9	19375713	19380252	RPS6
ENSG00000137161	8.922987828	9.448154031	protein_coding	6	42896938	42907025	CNPY3
ENSG00000137166	10.49112996	10.37259961	protein_coding	6	41514164	41570122	FOXP4
ENSG00000137168	9.045401313	9.321350625	protein_coding	6	36822603	36842800	PPIL1
ENSG00000137171	7.985986001	8.450859136	protein_coding	6	43008515	43042837	KLC4
ENSG00000137177	9.415619969	9.498022112	protein_coding	6	17759414	17987854	KIF13A
ENSG00000137185	6.171673665	6.779278886	protein_coding	6	28192664	28201260	ZNF193
ENSG00000137193	9.27062517	9.073276144	protein_coding	6	37137979	37143202	PIM1
ENSG00000137198	5.207774174	5.953060722	protein_coding	6	16238811	16295780	GMPR
ENSG00000137200	9.419108071	9.81731512	protein_coding	6	37400995	37450603	FTSJD2
ENSG00000137203	9.875269153	9.271406033	protein_coding	6	10393419	10419892	TFAP2A
ENSG00000137207	9.815645694	10.26404913	protein_coding	6	43479565	43484728	YIPF3
ENSG00000137210	8.457286611	9.119178324	protein_coding	6	10747973	10930656	TMEM14B
ENSG00000137216	8.565120083	8.634788863	protein_coding	6	44094651	44123256	TMEM63B
ENSG00000137218	4.230497448	4.622587519	protein_coding	6	41737914	41754280	FRS3
ENSG00000137221	9.185117095	9.242810972	protein_coding	6	43445261	43474294	TJAP1
ENSG00000137225	1.16343121	0	protein_coding	6	44126548	44152139	CAPN11
ENSG00000137259	0.869158192	0.697730409	protein_coding	6	26033320	26033796	HIST1H2AB
ENSG00000137261	0.869158192	3.027231696	protein_coding	6	24544332	24646383	KIAA0319
ENSG00000137265	1.616589159	2.039052734	protein_coding	6	391739	411447	IRF4
ENSG00000137266	7.706593329	7.815148897	protein_coding	6	3269196	3457256	SLC22A23
ENSG00000137267	7.30049066	7.492830145	protein_coding	6	3153903	3157809	TUBB2A
ENSG00000137269	7.003331296	6.505763096	protein_coding	6	53659295	53788919	LRRC1
ENSG00000137270	0.869158192	0.390640832	protein_coding	6	52991762	53013627	GCM1
ENSG00000137274	6.749949642	7.073814954	protein_coding	6	3118608	3153812	BPHL
ENSG00000137275	8.447071828	8.690147008	protein_coding	6	3064092	3115421	RIPK1
ENSG00000137285	1.799000381	0.390640832	protein_coding	6	3224517	3231964	TUBB2B
ENSG00000137288	8.443650765	9.405128432	protein_coding	6	33665345	33679504	C6orf125
ENSG00000137309	13.47331321	12.89520344	protein_coding	6	34204577	34214008	HMGA1
ENSG00000137310	8.133414584	8.501254594	protein_coding	6	31126319	31134936	TCF19
ENSG00000137312	9.540974801	9.801313989	protein_coding	6	30695486	30710510	FLOT1
ENSG00000137331	12.19253252	12.01652799	protein_coding	6	30710976	30712331	IER3
ENSG00000137337	10.52915247	10.44261478	protein_coding	6	30667584	30685666	MDC1
ENSG00000137338	3.004694206	3.906303962	protein_coding	6	28249314	28270326	PGBD1
ENSG00000137343	6.749949642	7.326703453	protein_coding	6	30594619	30614600	ATAT1
ENSG00000137364	7.845284967	7.907733332	protein_coding	6	18128542	18155305	TPMT
ENSG00000137393	8.311006074	8.672662438	protein_coding	6	18368779	18469105	RNF144B
ENSG00000137404	8.487504052	8.391976969	protein_coding	6	30655824	30659197	NRM
ENSG00000137409	11.2978184	11.58403148	protein_coding	6	36935917	36954074	MTCH1

ENSG00000137411	8.675694252	9.038718229	protein_coding	6	30876019	30894236	VARS2
ENSG00000137413	8.346244381	8.40394723	protein_coding	6	42018251	42055199	TAF8
ENSG00000137414	7.642387679	7.632773539	protein_coding	6	17600583	17611950	FAM8A1
ENSG00000137434	1.407729925	3.453019579	protein_coding	6	10671651	10695030	C6orf52
ENSG00000137440	6.163378456	1.353254395	protein_coding	4	15937192	15940363	FGFBP1
ENSG00000137449	7.08025234	7.070480904	protein_coding	4	15004298	15071777	CPEB2
ENSG00000137460	6.129712275	6.338437404	protein_coding	4	153857504	153900848	FHDC1
ENSG00000137462	7.876068031	8.754928172	protein_coding	4	154622652	154626851	TLR2
ENSG00000137463	0.499066092	0.390640832	protein_coding	4	140187317	140201492	C4orf49
ENSG00000137473	0	0.390640832	protein_coding	4	147627790	147867034	TTC29
ENSG00000137474	6.212449006	4.841813558	protein_coding	11	76839310	76926284	MYO7A
ENSG00000137478	7.651313074	7.903991977	protein_coding	11	72547790	72853306	FCHSD2
ENSG00000137486	8.60394966	8.558303982	protein_coding	11	74975226	75062873	ARRB1
ENSG00000137491	4.292665995	2.788380093	protein_coding	11	74811608	74917594	SLCO2B1
ENSG00000137492	8.223944437	8.651620478	protein_coding	11	76061000	76092015	PRKRIR
ENSG00000137494	6.975172889	7.195117037	protein_coding	11	82904754	82971736	ANKRD42
ENSG00000137496	6.489593565	6.783357574	protein_coding	11	71709587	71716761	IL18BP
ENSG00000137497	10.93696741	10.89400432	protein_coding	11	71713910	71791739	NUMA1
ENSG00000137500	8.016241092	8.417132063	protein_coding	11	82970139	82997450	CCDC90B
ENSG00000137501	8.133414584	8.282410365	protein_coding	11	85405267	85522184	SYTL2
ENSG00000137502	7.285281023	7.598461017	protein_coding	11	82684175	82782965	RAB30
ENSG00000137504	9.415619969	9.626176395	protein_coding	11	85370752	85393951	CREBZF
ENSG00000137507	2.847891871	3.493416095	protein_coding	11	76368568	76381791	LRRC32
ENSG00000137509	9.75276053	10.4845527	protein_coding	11	82534544	82681626	PRCP
ENSG00000137513	8.316627558	8.594734178	protein_coding	11	78147007	78285919	NARS2
ENSG00000137522	7.789781675	8.309531754	protein_coding	11	71639747	71708643	RNF121
ENSG00000137547	9.016909338	9.472987904	protein_coding	8	55047770	55060461	MRPL15
ENSG00000137558	0	0.390640832	protein_coding	8	75736772	75767264	PI15
ENSG00000137563	8.251597515	8.962525691	protein_coding	8	63927638	63951730	GGH
ENSG00000137571	5.301468762	5.893930351	protein_coding	8	70584568	70747299	SLCO5A1
ENSG00000137573	0.499066092	0	protein_coding	8	70378859	70573150	SULF1
ENSG00000137574	7.940593421	7.909600377	protein_coding	8	56685701	56738007	TGS1
ENSG00000137575	10.58844284	10.78736503	protein_coding	8	59465483	59495419	SDCBP
ENSG00000137601	7.797842652	8.449576416	protein_coding	4	170314426	170533780	NEK1
ENSG00000137628	8.867898261	9.489309466	protein_coding	4	169137444	169239958	DDX60
ENSG00000137634	0	0.390640832	protein_coding	11	114441313	114466484	FAM55D
ENSG00000137642	8.032273249	7.103479673	protein_coding	11	121322912	121504387	SORL1
ENSG00000137648	7.33043718	0.390640832	protein_coding	11	117947753	117992605	TMPRSS4
ENSG00000137656	8.211928911	8.305283181	protein_coding	11	116618886	116643704	BUD13
ENSG00000137673	1.799000381	0.950786998	protein_coding	11	102391239	102401484	MMP7
ENSG00000137691	3.950976309	4.406904905	protein_coding	11	101918174	101955291	C11orf70
ENSG00000137692	9.465390183	9.673093524	protein_coding	11	102932805	102962944	DCUN1D5
ENSG00000137693	10.40887296	10.29258813	protein_coding	11	101981192	102104154	YAP1
ENSG00000137699	5.91922012	4.021284656	protein_coding	11	119981983	120056237	TRIM29
ENSG00000137700	8.195750939	8.509895614	processed_transc	11	118894824	118901616	SLC37A4
ENSG00000137707	0.499066092	0.390640832	protein_coding	11	111338251	111383079	BTG4
ENSG00000137709	2.471521042	1.518964905	protein_coding	11	120107349	120190653	POU2F3
ENSG00000137710	10.01896842	10.02835246	protein_coding	11	110045605	110167447	RDX
ENSG00000137713	8.885655987	8.965221116	protein_coding	11	111597632	111637169	PPP2R1B
ENSG00000137714	7.416718478	7.795089291	protein_coding	11	110300607	110335605	FDX1
ENSG00000137720	6.220467583	6.592001111	protein_coding	11	111749659	111756699	C11orf1
ENSG00000137727	1.407729925	1.16600992	protein_coding	11	110447766	110583912	ARHGAP20
ENSG00000137745	5.637181576	2.652276565	protein_coding	11	102813724	102826463	MMP13
ENSG00000137747	2.471521042	1.667587519	protein_coding	11	117771358	117800174	TMPRSS13
ENSG00000137752	6.47626108	6.642271819	protein_coding	11	104896170	104972158	CASP1
ENSG00000137757	0.499066092	0	protein_coding	11	104864962	104893895	CASP5
ENSG00000137760	7.734859118	7.996490656	protein_coding	11	107373452	107436472	ALKBH8
ENSG00000137764	7.15746161	7.36802722	protein_coding	15	67835021	68116181	MAP2K5
ENSG00000137767	7.214780754	7.022976646	protein_coding	15	45923346	45983492	SQRDL
ENSG00000137770	8.602416377	8.618900187	protein_coding	15	44719432	44821236	CTDSPL2
ENSG00000137776	9.790280964	9.788181112	protein_coding	15	59171244	59225852	SLTM
ENSG00000137801	12.55818714	12.33621903	protein_coding	15	39873280	39891667	THBS1
ENSG00000137802	8.171138658	7.596144237	protein_coding	15	42066632	42120053	MAPKBP1

ENSG00000137804	9.889797802	10.14150744	protein_coding	15	41624892	41673248	NUSAP1
ENSG00000137806	7.53396104	7.534563804	protein_coding	15	41679551	41694717	NDUFAF1
ENSG00000137807	9.674658935	9.83559787	protein_coding	15	69706585	69740764	KIF23
ENSG00000137808	2.671945279	1.518964905	protein_coding	15	69222843	69355083	
ENSG00000137809	5.072559152	6.304782526	protein_coding	15	68594050	68724502	ITGA11
ENSG00000137812	8.790563466	8.826839049	protein_coding	15	40886218	40956540	CASC5
ENSG00000137814	8.462367008	8.326402026	protein_coding	15	42841008	42862192	HAUS2
ENSG00000137815	9.415619969	9.281534232	protein_coding	15	41700606	41775761	RTF1
ENSG00000137817	7.630400601	8.397309351	protein_coding	15	72533522	72565340	PARP6
ENSG00000137818	12.25205067	12.6868007	protein_coding	15	69745123	69748255	RPLP1
ENSG00000137819	7.68357507	6.582669759	protein_coding	15	69591286	69700119	PAQR5
ENSG00000137821	6.670228606	7.087074634	protein_coding	15	71145578	71342418	LRRC49
ENSG00000137822	9.074447906	8.309531754	protein_coding	15	43661419	43699293	TUBGCP4
ENSG00000137824	8.3480754	8.347216179	protein_coding	15	41028082	41048049	FAM82A2
ENSG00000137825	3.211941663	4.075511708	protein_coding	15	41785591	41795747	ITPKA
ENSG00000137831	9.331123617	9.590541354	protein_coding	15	70946893	71055932	UACA
ENSG00000137834	6.112579918	6.002883551	protein_coding	15	66994670	67074338	SMAD6
ENSG00000137841	3.830412367	3.813624741	protein_coding	15	40570377	40600136	PLCB2
ENSG00000137842	6.17992145	6.460630645	protein_coding	15	43415477	43477344	TMEM62
ENSG00000137843	8.114176072	3.183544561	protein_coding	15	40509629	40569688	PAK6
ENSG00000137845	10.92628281	10.6782458	protein_coding	15	58887403	59042177	ADAM10
ENSG00000137857	3.78787645	3.748356452	protein_coding	15	45422131	45457774	DUOX1
ENSG00000137860	0	0.697730409	protein_coding	15	45544428	45568149	SLC28A2
ENSG00000137868	8.98901727	7.955508102	protein_coding	15	74471807	74502046	STRA6
ENSG00000137871	7.1740722	7.551403417	protein_coding	15	56922379	57210769	ZNF280D
ENSG00000137875	2.471521042	2.039052734	protein_coding	15	52401460	52404972	BCL2L10
ENSG00000137876	9.483828293	9.861871738	protein_coding	15	55473004	55489265	RSL24D1
ENSG00000137877	3.077135474	2.912743273	protein_coding	15	42140345	42186275	SPTBN5
ENSG00000137878	4.437288965	3.183544561	protein_coding	15	57884106	58006943	GCOM1
ENSG00000137880	5.286269488	4.640685763	protein_coding	15	41056218	41059906	GCHFR
ENSG00000137936	8.814599602	8.774525884	protein_coding	1	94027347	94312706	BCAR3
ENSG00000137941	5.270908378	6.610484676	protein_coding	1	84330711	84464833	TTLL7
ENSG00000137942	6.861834435	7.100213554	protein_coding	1	93913658	94020218	FNBP1L
ENSG00000137944	6.541724534	7.307006333	protein_coding	1	89401456	89458636	CCBL2
ENSG00000137947	7.08025234	7.060432274	protein_coding	1	89318615	89357627	GTF2B
ENSG00000137955	9.771301603	9.682405806	protein_coding	1	76251879	76261100	RABGGTB
ENSG00000137959	7.464764762	9.272131838	protein_coding	1	79085607	79108484	IFI44L
ENSG00000137960	0.869158192	0.697730409	protein_coding	1	78445226	78604133	GIPC2
ENSG00000137962	10.00626968	9.505448443	protein_coding	1	94614544	94740624	ARHGAP29
ENSG00000137965	8.713115882	9.302290571	protein_coding	1	79115481	79129763	IFI44
ENSG00000137970	1.407729925	1.802319292	pseudogene	1	97144430	97145176	
ENSG00000137975	1.960915222	1.353254395	protein_coding	1	86889769	86922241	CLCA2
ENSG00000137992	7.132181536	6.870289474	protein_coding	1	100659634	100715390	DBT
ENSG00000137996	8.327805238	8.18248514	protein_coding	1	100731763	100758325	RTCD1
ENSG00000138002	8.391336806	8.88967084	protein_coding	2	27667238	27712656	IFT172
ENSG00000138018	9.834054211	9.85268169	protein_coding	2	26531415	26618759	EPT1
ENSG00000138028	0	0.390640832	protein_coding	2	27321757	27341995	CGREF1
ENSG00000138029	9.515945881	10.037346	protein_coding	2	26466038	26513336	HADHB
ENSG00000138030	4.165528823	5.283144778	protein_coding	2	27309615	27323640	KHK
ENSG00000138031	9.43986141	8.599368544	protein_coding	2	25042038	25142708	ADCY3
ENSG00000138032	8.269098624	8.621180739	protein_coding	2	44395108	44471523	PPM1B
ENSG00000138035	8.840844993	9.322752514	protein_coding	2	55861400	55921045	PNPT1
ENSG00000138036	6.146643566	6.699508919	protein_coding	2	44001178	44037149	DYNC2LI1
ENSG00000138041	9.514316103	9.599243793	protein_coding	2	55774428	55846015	SMEK2
ENSG00000138050	6.749949642	6.97385478	protein_coding	2	39963200	40006407	THUMPD2
ENSG00000138061	12.04865098	11.59480528	protein_coding	2	38294116	38337044	CYP1B1
ENSG00000138069	10.2173189	10.17991662	protein_coding	2	65297835	65357240	RAB1A
ENSG00000138071	11.21545961	11.49900721	protein_coding	2	65454887	65498387	ACTR2
ENSG00000138073	9.61887538	9.849767366	protein_coding	2	27353624	27357543	PREB
ENSG00000138074	9.510233586	9.730200815	protein_coding	2	27422455	27435826	SLC5A6
ENSG00000138075	0.499066092	1.16600992	protein_coding	2	44039611	44066004	ABCG5
ENSG00000138078	8.910650905	9.178452256	protein_coding	2	44543420	44589001	PREPL
ENSG00000138079	0.499066092	1.353254395	protein_coding	2	44502599	44548633	SLC3A1

ENSG00000138080	3.448790144	3.64455972	protein_coding	2	27301435	27309271	EMILIN1
ENSG00000138081	9.253142682	9.472356449	protein_coding	2	48016455	48132932	FBXO11
ENSG00000138083	2.10647801	2.579085888	protein_coding	2	45168902	45173216	SIX3
ENSG00000138085	8.888175056	9.478658596	protein_coding	2	27434895	27440046	C2orf28
ENSG00000138092	8.043616684	7.55379311	protein_coding	2	25016005	25045245	CENPO
ENSG00000138095	11.32967441	11.44885281	protein_coding	2	44113363	44223144	LRPPRC
ENSG00000138100	2.928422289	2.971122874	protein_coding	2	27505260	27600344	TRIM54
ENSG00000138101	7.01259661	6.293387477	protein_coding	2	25600067	25896503	DTNB
ENSG00000138107	10.92137194	11.11379372	protein_coding	10	104238986	104262482	ACTR1A
ENSG00000138109	2.928422289	0	protein_coding	10	96698415	96749447	CYP2C9
ENSG00000138111	7.478203418	7.359856617	protein_coding	10	104221149	104239484	TMEM180
ENSG00000138115	1.407729925	3.493416095	protein_coding	10	96796530	96829254	CYP2C8
ENSG00000138119	12.27099476	12.24664642	protein_coding	10	95066186	95242074	MYOF
ENSG00000138131	5.995967334	5.372493443	protein_coding	10	100007447	100028007	LOXL4
ENSG00000138134	7.593829938	7.641787375	protein_coding	10	90639491	90734910	STAMBPL1
ENSG00000138135	1.960915222	2.721932731	protein_coding	10	90965694	90967071	CH25H
ENSG00000138138	8.687312327	9.11108789	protein_coding	10	89511269	89601100	ATAD1
ENSG00000138152	0.869158192	0.390640832	protein_coding	10	124030821	124097677	BTBD16
ENSG00000138160	9.722025379	9.873875397	protein_coding	10	94353043	94415150	KIF11
ENSG00000138161	2.762599152	4.528525526	protein_coding	10	124591665	124639146	CUZD1
ENSG00000138162	8.810621286	8.85617872	protein_coding	10	123748689	124014060	TACC2
ENSG00000138166	10.10215133	8.42106413	protein_coding	10	112257596	112271302	DUSP5
ENSG00000138172	9.089849478	9.439780161	protein_coding	10	105206543	105212660	CALHM2
ENSG00000138175	7.345180517	8.005247922	protein_coding	10	104436315	104474164	ARL3
ENSG00000138180	9.704266019	10.05853621	protein_coding	10	95256389	95288849	CEP55
ENSG00000138182	9.070017088	9.355310748	protein_coding	10	91461367	91534700	KIF20B
ENSG00000138185	7.514527001	7.999999944	protein_coding	10	97454774	97637023	ENTPD1
ENSG00000138190	7.071412799	7.593823731	protein_coding	10	94590935	94819250	EXOC6
ENSG00000138193	7.15746161	7.903991977	protein_coding	10	95753746	96088149	PLCE1
ENSG00000138231	7.778963161	7.752033772	protein_coding	3	137879854	137893791	DBR1
ENSG00000138246	10.02984629	9.951142479	protein_coding	3	132136370	132257876	DNAJC13
ENSG00000138271	6.522395495	1.518964905	protein_coding	3	151011891	151034740	GPR87
ENSG00000138279	10.42110141	9.960628829	protein_coding	10	75135203	75173834	ANXA7
ENSG00000138286	7.413224588	7.142107001	protein_coding	10	74927924	75004262	FAM149B1
ENSG00000138293	10.8217796	10.45225374	protein_coding	10	51565108	51590734	NCOA4
ENSG00000138297	8.517101548	8.231074746	protein_coding	10	51592080	51623365	TIMM23
ENSG00000138303	8.398422578	7.95731437	protein_coding	10	73856278	73980083	ASCC1
ENSG00000138311	2.671945279	2.579085888	protein_coding	10	64133951	64431771	ZNF365
ENSG00000138316	3.146112541	4.250702764	protein_coding	10	72432559	72522197	ADAMTS14
ENSG00000138326	12.09108862	12.83218685	protein_coding	10	79793518	79816570	RPS24
ENSG00000138336	3.989024711	3.133298822	protein_coding	10	70320413	70454239	TET1
ENSG00000138346	7.913645586	7.58917149	protein_coding	10	70173821	70231879	DNA2
ENSG00000138347	3.502389126	2.579085888	protein_coding	10	69865912	69971774	MYPN
ENSG00000138356	6.567100227	7.815148897	protein_coding	2	201450591	201541787	AOX1
ENSG00000138363	10.69946212	10.29616073	protein_coding	2	216176540	216214487	ATIC
ENSG00000138375	8.844740944	8.635917101	protein_coding	2	217277137	217347776	SMARCAL1
ENSG00000138376	7.726437377	7.53698153	protein_coding	2	215590370	215674428	BARD1
ENSG00000138378	2.847891871	1.518964905	protein_coding	2	191894302	192016322	STAT4
ENSG00000138380	4.568725997	5.03207928	protein_coding	2	203776937	203851060	ALS2CR8
ENSG00000138381	9.081068723	9.212881815	protein_coding	2	190526146	190535557	ASNSD1
ENSG00000138382	7.923503136	8.122575449	protein_coding	2	170666591	170681441	METTL5
ENSG00000138385	10.02298561	10.12231502	protein_coding	2	170648443	170668574	SSB
ENSG00000138386	8.057112115	7.457530976	protein_coding	2	191511472	191557492	NAB1
ENSG00000138395	1.407729925	1.16600992	protein_coding	2	202655184	202760273	CDK15
ENSG00000138398	9.505319266	9.409093306	protein_coding	2	170440850	170504696	PPIG
ENSG00000138399	8.12703021	7.992972811	protein_coding	2	170386259	170430385	FASTKD1
ENSG00000138400	2.35979773	3.081239798	protein_coding	2	207602487	207630271	MDH1B
ENSG00000138411	6.699208722	6.371325053	protein_coding	2	197063977	197458416	HECW2
ENSG00000138413	9.564002263	8.539135107	protein_coding	2	209100951	209130798	IDH1
ENSG00000138430	9.915339294	9.919526433	protein_coding	2	174937175	175113426	OLA1
ENSG00000138433	7.671926738	7.776794036	protein_coding	2	175212749	175260443	CIR1
ENSG00000138434	9.733978068	9.606168145	protein_coding	2	182756560	182795465	SSFA2
ENSG00000138439	7.553136766	7.033288635	protein_coding	2	203499901	203634480	FAM117B

ENSG00000138442	8.978417134	8.820898829	protein_coding	2	203745323	203879521	WDR12
ENSG00000138443	9.327415887	8.418443943	protein_coding	2	204192942	204301606	ABI2
ENSG00000138448	10.74394947	10.76123697	protein_coding	2	187454790	187545628	ITGAV
ENSG00000138449	0.869158192	1.353254395	protein_coding	2	190425305	190448484	SLC40A1
ENSG00000138459	7.234712227	7.768587741	protein_coding	3	112280556	112304424	SLC35A5
ENSG00000138463	7.30049066	7.766528853	protein_coding	3	122513642	122599986	DIRC2
ENSG00000138468	6.727618145	6.995112131	protein_coding	3	101043049	101232085	SENP7
ENSG00000138483	0.499066092	0.697730409	protein_coding	3	107096188	107097481	CCDC54
ENSG00000138495	6.236372259	6.520498852	protein_coding	3	119373360	119396301	COX17
ENSG00000138496	8.779750834	8.966118473	protein_coding	3	122246806	122283424	PARP9
ENSG00000138587	5.899377978	6.24096575	protein_coding	15	56713742	56757335	MNS1
ENSG00000138592	8.47750173	8.431497548	protein_coding	15	50716577	50793280	USP8
ENSG00000138593	7.930852348	8.25035614	protein_coding	15	49280673	49338760	SECISBP2L
ENSG00000138594	9.4269256	9.094769627	protein_coding	15	52121825	52239492	TMOD3
ENSG00000138600	7.778963161	8.379906558	protein_coding	15	50999506	51058005	
ENSG00000138604	8.375265417	8.570156468	protein_coding	15	69452923	69564544	GLCE
ENSG00000138606	1.960915222	3.368666104	protein_coding	15	45459411	45493373	SHF
ENSG00000138613	6.032862302	6.779278886	protein_coding	15	63568217	63601325	APH1B
ENSG00000138614	8.876804446	8.940779054	protein_coding	15	65871091	65903627	C15orf44
ENSG00000138615	1.16343121	1.925536307	protein_coding	15	65488337	65503826	CILP
ENSG00000138617	6.92698535	6.835357159	protein_coding	15	65526798	65592956	PARP16
ENSG00000138621	6.407685389	6.771086752	protein_coding	15	75315896	75409803	PPCDC
ENSG00000138622	6.103936836	7.012590419	protein_coding	15	73612200	73661605	HCN4
ENSG00000138623	8.848626402	6.742042315	protein_coding	15	74701630	74726808	SEMA7A
ENSG00000138629	9.056642394	9.204541268	protein_coding	15	74738318	74753523	UBL7
ENSG00000138639	6.887241849	7.272601283	protein_coding	4	86396267	86923823	ARHGAP24
ENSG00000138640	7.916116295	8.053329862	protein_coding	4	89647106	90032549	FAM13A
ENSG00000138641	6.350376764	6.044260771	protein_coding	4	89444961	89629693	HERC3
ENSG00000138642	8.156585168	9.010289238	protein_coding	4	89299891	89364263	HERC6
ENSG00000138646	8.133414584	8.965221116	protein_coding	4	89378268	89427314	HERC5
ENSG00000138658	7.017207048	7.718344862	protein_coding	4	113460492	113558151	C4orf21
ENSG00000138660	7.921045056	8.168519197	protein_coding	4	113152893	113191203	AP1AR
ENSG00000138663	8.150302734	8.341695034	protein_coding	4	83955600	83996971	COPS4
ENSG00000138668	11.79913269	11.44853179	protein_coding	4	83273651	83295656	HNRNPD
ENSG00000138670	0.869158192	0	protein_coding	4	82347547	82393082	RASGEF1B
ENSG00000138674	10.90622757	10.89306095	protein_coding	4	83739814	83822319	SEC31A
ENSG00000138678	7.254372086	4.932754837	protein_coding	4	84457067	84527028	AGPAT9
ENSG00000138685	1.799000381	1.353254395	protein_coding	4	123747863	123819391	FGF2
ENSG00000138686	6.965663283	7.359856617	protein_coding	4	122745595	122791652	BBS7
ENSG00000138688	8.375265417	8.506198656	protein_coding	4	123073488	123283913	KIAA1109
ENSG00000138696	5.83815965	6.016808232	protein_coding	4	95679119	96079599	BMPR1B
ENSG00000138698	9.043142548	9.120790981	protein_coding	4	99182535	99365012	RAP1GDS1
ENSG00000138709	7.943018447	7.705505903	protein_coding	4	128982423	129144086	LARP1B
ENSG00000138735	6.733233509	6.712401119	protein_coding	4	120415550	120550146	PDE5A
ENSG00000138744	7.22677265	6.847095496	protein_coding	4	76831809	76862204	NAAA
ENSG00000138750	8.497437504	8.621180739	protein_coding	4	77035812	77069668	NUP54
ENSG00000138755	0.869158192	0.950786998	protein_coding	4	76922428	76928641	CXCL9
ENSG00000138756	7.384964606	7.741590246	protein_coding	4	79697496	79837526	BMP2K
ENSG00000138757	10.71089111	10.72432142	protein_coding	4	76567966	76649709	G3BP2
ENSG00000138758	10.41062624	10.16826693	protein_coding	4	77870856	77961537	11-sep
ENSG00000138759	7.498129565	8.008735998	protein_coding	4	78978724	79465423	FRAS1
ENSG00000138760	10.28960502	10.71874163	protein_coding	4	77079890	77135046	SCARB2
ENSG00000138764	7.615275323	8.104758487	protein_coding	4	78078304	78354542	CCNG2
ENSG00000138767	8.33889698	8.615472585	protein_coding	4	78634541	78740769	CNOT6L
ENSG00000138768	10.09943635	10.22522208	protein_coding	4	76649762	76735382	USO1
ENSG00000138769	0.499066092	2.242360793	protein_coding	4	76503215	76555900	CDKL2
ENSG00000138771	9.664408419	8.648269828	protein_coding	4	77356253	77704406	SHROOM3
ENSG00000138772	10.14174685	10.48986626	protein_coding	4	79472673	79531597	ANXA3
ENSG00000138777	8.048129203	8.686884751	protein_coding	4	106290234	106395238	PPA2
ENSG00000138778	8.853790788	9.040423325	protein_coding	4	104026963	104119566	CENPE
ENSG00000138780	7.23866569	7.36802722	protein_coding	4	106629935	106768885	GSTCD
ENSG00000138785	7.136425764	7.148445524	protein_coding	4	106603784	106817143	INTS12
ENSG00000138792	3.448790144	3.570966319	protein_coding	4	111286889	111486441	ENPEP

ENSG00000138794	5.869093502	5.42565083	protein_coding	4	110609785	110624739	CASP6
ENSG00000138795	0	0.697730409	protein_coding	4	108968701	109090112	LEF1
ENSG00000138796	8.593182305	8.89533508	protein_coding	4	108910870	108956331	HADH
ENSG00000138798	0.869158192	2.851901313	protein_coding	4	110834040	110933422	EGF
ENSG00000138801	8.16075831	8.377210474	protein_coding	4	108511433	108641608	PAPSS1
ENSG00000138802	8.925442603	8.784734524	protein_coding	4	110354928	110461612	SEC24B
ENSG00000138814	9.735377778	9.448796166	protein_coding	4	101944566	102269435	PPP3CA
ENSG00000138821	8.682966505	9.379087693	protein_coding	4	103172198	103352415	SLC39A8
ENSG00000138823	0.499066092	1.16600992	protein_coding	4	100484918	100545156	MTTP
ENSG00000138829	0.499066092	1.353254395	protein_coding	5	127593601	127994878	FBN2
ENSG00000138834	9.837968518	10.11221693	protein_coding	16	1756184	1820318	MAPK8IP3
ENSG00000138835	9.016909338	8.948972504	protein_coding	9	116207011	116360018	RGS3
ENSG00000138867	10.10269371	9.86620457	protein_coding	22	24936406	24951903	C22orf13
ENSG00000138942	9.086562954	9.391832931	protein_coding	22	31556168	31602999	RNF185
ENSG00000138944	5.417620896	6.835357159	protein_coding	22	44639547	44708731	KIAA1644
ENSG00000138964	0	0.390640832	protein_coding	22	44568836	44615413	PARVG
ENSG00000139044	6.502803968	4.15321211	protein_coding	12	569530	672675	B4GALNT3
ENSG00000139055	2.10647801	0	protein_coding	12	15066969	15092016	ERP27
ENSG00000139083	7.876068031	7.449853178	protein_coding	12	11802788	12044558	ETV6
ENSG00000139112	8.766120079	8.593573257	protein_coding	12	10365057	10375727	GABARAPL1
ENSG00000139116	9.113724571	9.305839261	protein_coding	12	39687030	39837192	KIF21A
ENSG00000139117	7.925957035	8.240005883	protein_coding	12	39040624	39301232	CPNE8
ENSG00000139131	8.531675665	8.695567763	protein_coding	12	32880424	32908836	YARS2
ENSG00000139132	6.228441838	5.339632724	protein_coding	12	32552463	32798984	FGD4
ENSG00000139133	4.999881834	5.638744877	protein_coding	12	34175216	34182629	ALG10
ENSG00000139144	1.16343121	0.697730409	protein_coding	12	18400548	18801348	PIK3C2G
ENSG00000139146	10.09344535	9.545623701	protein_coding	12	31433518	31479992	FAM60A
ENSG00000139154	8.883132512	8.205465413	protein_coding	12	19556979	19873735	AEBP2
ENSG00000139160	3.78787645	3.87606762	protein_coding	12	31800094	31826048	METTL20
ENSG00000139163	7.123655438	7.033288635	protein_coding	12	22778009	22838646	ETNK1
ENSG00000139168	8.583888749	8.825850709	protein_coding	12	42705880	42719920	ZCRB1
ENSG00000139173	4.062237333	2.652276565	protein_coding	12	44229770	44783545	TMEM117
ENSG00000139174	6.931877181	6.729413443	protein_coding	12	42852140	42984157	PRICKLE1
ENSG00000139178	9.05552222	8.915915748	protein_coding	12	7242183	7261869	C1RL
ENSG00000139180	8.325948295	8.37044814	protein_coding	12	4714110	4798454	NDUFA9
ENSG00000139182	8.299696963	7.778838337	protein_coding	12	7282294	7311541	CLSTN3
ENSG00000139187	3.211941663	3.748356452	protein_coding	12	9102640	9163356	KLRG1
ENSG00000139190	5.613019223	5.776071827	protein_coding	12	6571403	6580153	VAMP1
ENSG00000139192	7.837485466	7.977035488	protein_coding	12	6560856	6575683	TAPBPL
ENSG00000139193	1.960915222	1.925536307	protein_coding	12	6554033	6560884	CD27
ENSG00000139194	4.352265886	4.202780776	protein_coding	12	7276280	7281538	RBP5
ENSG00000139197	9.509415694	9.423538659	protein_coding	12	7341281	7371170	PEX5
ENSG00000139200	1.407729925	0.950786998	protein_coding	12	6802958	6809981	C12orf53
ENSG00000139209	1.16343121	1.353254395	protein_coding	12	47158544	47226191	SLC38A4
ENSG00000139211	8.632776617	8.628000846	protein_coding	12	47469490	47473734	AMIGO2
ENSG00000139218	9.591313258	9.733893656	protein_coding	12	46312914	46385903	SCAF11
ENSG00000139219	0	1.353254395	protein_coding	12	48366748	48398269	COL2A1
ENSG00000139233	7.250461488	6.945015469	protein_coding	12	66516842	66524548	LLPH
ENSG00000139239	4.026095388	3.081239798	pseudogene	12	63359095	63359764	RPL14P1
ENSG00000139263	8.408986408	8.289597152	protein_coding	12	59265931	59314303	LRIG3
ENSG00000139266	5.346128795	5.09905904	protein_coding	12	58148881	58154190	9-Mar
ENSG00000139269	7.296703257	7.666288907	protein_coding	12	57846106	57853063	INHBE
ENSG00000139278	8.794597401	9.644790504	protein_coding	12	75874460	75897633	GLIPR1
ENSG00000139287	1.16343121	0.697730409	protein_coding	12	72332626	72580398	TPH2
ENSG00000139289	11.15712394	9.540210691	protein_coding	12	76419227	76427712	PHLDA1
ENSG00000139291	6.798966424	6.858739095	protein_coding	12	72079867	72097836	TMEM19
ENSG00000139318	7.855619141	7.741590246	protein_coding	12	89741050	89747048	DUSP6
ENSG00000139323	7.311793575	7.426571535	protein_coding	12	89813495	89919801	POC1B
ENSG00000139324	8.631273684	8.568975592	protein_coding	12	88536073	88593664	TMTC3
ENSG00000139329	1.407729925	3.679994897	protein_coding	12	91496406	91505608	LUM
ENSG00000139343	8.915598341	8.852301067	protein_coding	12	96252706	96297606	SNRPF
ENSG00000139344	2.928422289	2.971122874	protein_coding	12	96337071	96362370	AMDHD1
ENSG00000139350	8.251597515	8.568975592	protein_coding	12	97301001	97347129	NEDD1

ENSG00000139351	1.407729925	1.925536307	protein_coding	12	102122426	102133250	SYCP3
ENSG00000139352	0.499066092	0	protein_coding	12	103351464	103354294	ASCL1
ENSG00000139354	7.64536895	7.616862529	protein_coding	12	100967461	101022064	GAS2L3
ENSG00000139364	0.499066092	0.697730409	protein_coding	12	125671382	126146917	TMEM132B
ENSG00000139370	8.077121473	8.288162655	protein_coding	12	129277739	129308528	SLC15A4
ENSG00000139372	8.009315251	8.506198656	protein_coding	12	104359582	104382652	TDG
ENSG00000139405	8.991362316	8.804937616	protein_coding	12	113623331	113630173	C12orf52
ENSG00000139410	7.136425764	7.799123587	protein_coding	12	113860042	113876081	SDSL
ENSG00000139428	7.893726093	7.63953919	protein_coding	12	109993255	110011679	MMAB
ENSG00000139433	8.282565319	7.679480043	protein_coding	12	110288959	110318293	GLTP
ENSG00000139436	7.612231145	7.699043321	protein_coding	12	110367607	110434194	GIT2
ENSG00000139437	7.478203418	7.485338407	protein_coding	12	110338069	110421646	TCHP
ENSG00000139438	5.485736881	4.826083323	protein_coding	12	110152033	110208312	C12orf34
ENSG00000139496	9.811006628	9.354625616	protein_coding	13	25875662	25923938	NUPL1
ENSG00000139505	8.582333986	8.418443943	protein_coding	13	25802307	25862147	MTMR6
ENSG00000139508	0.499066092	0	protein_coding	13	29274201	29293107	SLC46A3
ENSG00000139514	10.70232786	9.925997554	protein_coding	13	30083547	30169721	SLC7A1
ENSG00000139517	6.62865285	6.077857763	protein_coding	13	28120050	28194541	LNX2
ENSG00000139531	7.768062907	7.842771865	protein_coding	12	56390964	56400425	SUOX
ENSG00000139537	5.223818625	4.917990497	protein_coding	12	49297893	49325623	CCDC65
ENSG00000139540	2.671945279	2.420525079	protein_coding	12	56623833	56631630	SLC39A5
ENSG00000139546	8.536501169	8.863902898	protein_coding	12	53894705	53900215	TARBP2
ENSG00000139547	2.762599152	3.781359661	protein_coding	12	57345219	57353158	RDH16
ENSG00000139549	0.499066092	0.390640832	protein_coding	12	49483204	49488602	DHH
ENSG00000139567	4.19837882	3.570966319	protein_coding	12	52300692	52317145	ACVRL1
ENSG00000139572	1.960915222	0.950786998	protein_coding	12	54756229	54758271	GPR84
ENSG00000139574	4.568725997	4.903073497	protein_coding	12	53900472	53902004	NPFF
ENSG00000139579	9.223526019	8.93803753	protein_coding	12	56615799	56623638	OBFC2B
ENSG00000139597	3.335220907	3.411459265	protein_coding	13	32974861	33007091	N4BP2L1
ENSG00000139613	10.09943635	9.693826839	protein_coding	12	56556767	56583351	SMARCC2
ENSG00000139617	0.499066092	0.697730409	protein_coding	13	33006554	33054775	
ENSG00000139618	7.19863491	6.779278886	protein_coding	13	32889611	32973805	BRCA2
ENSG00000139620	7.530740159	7.591499486	protein_coding	12	49047184	49076021	KANSL2
ENSG00000139624	8.54291017	8.600524813	protein_coding	12	50523575	50561288	CERS5
ENSG00000139625	7.30049066	7.909600377	protein_coding	12	53874274	53893847	MAP3K12
ENSG00000139626	4.491309013	4.274079748	protein_coding	12	53585107	53601091	ITGB7
ENSG00000139629	8.748205354	6.136428444	protein_coding	12	51745682	51785436	GALNT6
ENSG00000139631	7.058051099	7.635032283	protein_coding	12	53551448	53575135	CSAD
ENSG00000139636	7.795160661	7.780879746	protein_coding	12	49490919	49504683	LMBR1L
ENSG00000139637	8.766120079	8.639296529	protein_coding	12	53693470	53700961	C12orf10
ENSG00000139641	10.91026098	10.70751691	protein_coding	12	56512034	56538457	ESYT1
ENSG00000139644	12.55630746	12.30100114	protein_coding	12	50101508	50158717	TMBIM6
ENSG00000139645	11.41206681	11.42098072	protein_coding	12	56631591	56652175	ANKRD52
ENSG00000139651	8.364450825	8.276635048	protein_coding	12	53574484	53583208	ZNF740
ENSG00000139656	0	0.697730409	protein_coding	13	44717679	44735393	C13orf44
ENSG00000139668	6.023726639	6.130036786	protein_coding	13	52158644	52334135	WDFY2
ENSG00000139675	4.230497448	4.048652948	protein_coding	13	53191605	53217919	HNRNPA1L2
ENSG00000139679	0.499066092	0	protein_coding	13	48963707	49018840	LPAR6
ENSG00000139684	8.33889698	8.073455769	protein_coding	13	47345391	47371367	ESD
ENSG00000139687	7.89873181	8.18248514	protein_coding	13	48877883	49056122	RB1
ENSG00000139697	9.804353406	9.534777296	protein_coding	12	123773656	123834988	SBNO1
ENSG00000139714	2.928422289	2.501982735	protein_coding	12	122089024	122110537	MORN3
ENSG00000139718	9.513500523	9.029304007	protein_coding	12	122242086	122270562	SETD1B
ENSG00000139719	6.335686705	6.50081754	protein_coding	12	122715292	122751068	VPS33A
ENSG00000139722	9.105088364	8.317991549	protein_coding	12	123349882	123380991	VPS37B
ENSG00000139725	5.575999561	4.17820932	protein_coding	12	122215664	122232261	RHOF
ENSG00000139726	9.69925397	9.621627484	protein_coding	12	123237321	123255611	DENR
ENSG00000139734	8.18144485	7.860254213	protein_coding	13	60239717	60738119	DIAPH3
ENSG00000139737	0.499066092	0	protein_coding	13	78272023	78338377	SLAIN1
ENSG00000139746	9.298353251	9.316433258	protein_coding	13	79885962	79980612	RBM26
ENSG00000139793	9.967488686	9.659288865	protein_coding	13	97873688	98046374	MBNL2
ENSG00000139797	6.489593565	6.544728831	protein_coding	13	98828039	98829519	RNF113B
ENSG00000139800	5.929039784	5.816437314	protein_coding	13	100615275	100624163	ZIC5

ENSG00000139826	6.782811835	7.016060809	protein_coding	13	108870727	108886603	ABHD13
ENSG00000139832	6.171673665	4.976162367	protein_coding	13	111175417	111214080	RAB20
ENSG00000139835	4.999881834	3.133298822	protein_coding	13	113978544	114018446	GRTP1
ENSG00000139842	9.729068359	9.595189186	protein_coding	13	113862552	113919399	CUL4A
ENSG00000139865	0	0.390640832	protein_coding	14	38065052	38510647	TTC6
ENSG00000139880	8.614637248	8.69123279	protein_coding	14	23516271	23526747	CDH24
ENSG00000139890	2.762599152	2.242360793	protein_coding	14	23352374	23356895	REM2
ENSG00000139899	4.923348194	6.535085617	protein_coding	14	24895738	24900160	CBLN3
ENSG00000139908	4.689181911	4.508954154	protein_coding	14	24674903	24677568	TSSK4
ENSG00000139910	4.712111592	6.192713747	protein_coding	14	26912299	27066960	NOVA1
ENSG00000139914	0.869158192	1.802319292	protein_coding	14	24600484	24602058	FITM1
ENSG00000139921	9.413872751	9.661506506	protein_coding	14	51706880	51722759	TMX1
ENSG00000139926	9.463702247	9.865723786	protein_coding	14	51955818	52197445	FRMD6
ENSG00000139946	0	0.390640832	protein_coding	14	56584532	56768244	PELI2
ENSG00000139970	0	0.390640832	protein_coding	14	60062694	60337684	RTN1
ENSG00000139971	2.928422289	4.65855978	protein_coding	14	58466453	58764857	C14orf37
ENSG00000139973	1.16343121	0	protein_coding	14	62453803	62568431	SYT16
ENSG00000139974	6.897280837	7.18590274	protein_coding	14	61447832	61550451	SLC38A6
ENSG00000139977	9.511051014	9.565299431	protein_coding	14	57857262	57882635	NAA30
ENSG00000139985	3.830412367	4.17820932	protein_coding	14	70924217	70926622	ADAM21
ENSG00000139988	2.35979773	2.652276565	protein_coding	14	68168603	68201169	RDH12
ENSG00000139990	9.832093067	10.09877829	protein_coding	14	69517598	69619867	DCAF5
ENSG00000139998	6.793601635	7.080460027	protein_coding	14	65412532	65439494	RAB15
ENSG00000140006	8.150302734	8.608592853	protein_coding	14	64063757	64108579	WDR89
ENSG00000140009	2.471521042	3.232099092	protein_coding	14	64550950	64804830	ESR2
ENSG00000140022	7.663128351	6.24096575	protein_coding	14	81727000	81902809	STON2
ENSG00000140025	6.573375019	6.45552765	protein_coding	14	90261013	90421121	EFCAB11
ENSG00000140030	0.499066092	0	protein_coding	14	88471468	88478419	GPR65
ENSG00000140043	6.188122351	6.475832214	protein_coding	14	74318547	74353530	PTGR2
ENSG00000140044	6.941611359	6.82352253	protein_coding	14	75894419	75940814	JDP2
ENSG00000140057	4.821592551	2.334191469	protein_coding	14	96858448	96955764	AK7
ENSG00000140090	7.04456449	6.911866869	protein_coding	14	92788925	92962596	SLC24A4
ENSG00000140092	1.616589159	2.501982735	protein_coding	14	92335756	92414331	FBLN5
ENSG00000140093	2.928422289	2.652276565	protein_coding	14	94749650	94759608	SERPINA10
ENSG00000140104	7.64536895	7.289906366	protein_coding	14	105452112	105476819	C14orf79
ENSG00000140105	11.72392003	11.09320008	protein_coding	14	100800125	100843142	WARS
ENSG00000140107	1.407729925	0.697730409	protein_coding	14	100789674	100796715	SLC25A47
ENSG00000140153	8.05038018	7.255086103	protein_coding	14	102605840	102691184	WDR20
ENSG00000140157	9.514316103	9.345689152	protein_coding	15	23004684	23034427	NIPA2
ENSG00000140181	7.842689815	8.724493	pseudogene	15	23282281	23378228	HERC2P2
ENSG00000140199	6.554468172	6.332882496	protein_coding	15	34525460	34630261	SLC12A6
ENSG00000140254	2.847891871	2.652276565	protein_coding	15	45409569	45422136	DUOXA1
ENSG00000140259	8.910650905	9.060728822	protein_coding	15	44096690	44117000	MFAP1
ENSG00000140262	9.2686931	9.259011141	protein_coding	15	57210323	57591479	TCF12
ENSG00000140263	8.900704842	8.099860884	protein_coding	15	45315302	45369383	SORD
ENSG00000140264	10.3009932	10.88145436	protein_coding	15	44069285	44094787	SERF2
ENSG00000140265	7.928406768	7.541804862	protein_coding	15	43650370	43663223	ZSCAN29
ENSG00000140279	1.960915222	1.925536307	protein_coding	15	45384848	45406542	DUOX2
ENSG00000140280	5.660945913	5.317302248	protein_coding	15	52015208	52043782	LYSMD2
ENSG00000140284	4.618121645	4.15321211	protein_coding	15	50474393	50528592	SLC27A2
ENSG00000140285	0.869158192	1.802319292	protein_coding	15	49715293	50149346	FGF7
ENSG00000140297	0.499066092	1.802319292	protein_coding	15	59887074	59932438	GCNT3
ENSG00000140299	8.760631512	8.669360359	protein_coding	15	59951345	59981733	BNIP2
ENSG00000140307	7.787084643	8.194786822	protein_coding	15	59930261	59949740	GTF2A2
ENSG00000140319	10.19714397	10.53319908	protein_coding	15	40327940	40331389	SRP14
ENSG00000140320	8.629769183	8.184028599	protein_coding	15	40731920	40760441	BAHD1
ENSG00000140323	5.60078458	5.212303549	protein_coding	15	40650436	40663257	DISP2
ENSG00000140326	7.792473675	7.664078611	protein_coding	15	43015757	43029417	CDAN1
ENSG00000140332	9.67684603	8.791837833	protein_coding	15	70340129	70390515	TLE3
ENSG00000140350	10.64203813	10.30434431	protein_coding	15	69039580	69113236	ANP32A
ENSG00000140365	8.631273684	9.350507971	protein_coding	15	75628232	75634268	COMMD4
ENSG00000140367	7.60612345	7.726841127	protein_coding	15	76135622	76193419	UBE2Q2
ENSG00000140368	1.799000381	2.420525079	protein_coding	15	77285700	77329673	PSTPIP1

ENSG00000140374	9.790954202	9.935650135	protein_coding	15	76507696	76603813	ETFA
ENSG00000140379	1.616589159	1.16600992	protein_coding	15	80253231	80263788	BCL2A1
ENSG00000140382	8.705994399	8.598211347	protein_coding	15	77712754	77777949	HMG20A
ENSG00000140386	6.567100227	6.646756201	protein_coding	15	76640526	77197785	SCAPER
ENSG00000140391	10.38097398	10.60984308	protein_coding	15	77336359	77376326	TSPAN3
ENSG00000140395	8.502378699	8.667154767	protein_coding	15	78570177	78592136	WDR61
ENSG00000140396	8.272959095	8.154416734	protein_coding	8	71021997	71316040	NCOA2
ENSG00000140398	6.086493765	5.855704078	protein_coding	15	75639296	75647592	NEIL1
ENSG00000140400	9.392739397	9.669792433	protein_coding	15	75648133	75660971	MAN2C1
ENSG00000140403	7.296703257	7.512619979	protein_coding	15	78556428	78574538	DNAJA4
ENSG00000140406	7.974178365	7.591499486	protein_coding	15	81293295	81296342	MESDC1
ENSG00000140416	10.17048758	11.59943942	protein_coding	15	63334831	63364114	TPM1
ENSG00000140443	9.91224858	9.722786654	protein_coding	15	99192200	99507759	IGF1R
ENSG00000140450	6.400645076	7.41347308	protein_coding	15	98462784	98517068	ARRDC4
ENSG00000140451	7.663128351	7.971683681	protein_coding	15	65107831	65117867	PIF1
ENSG00000140455	8.389559911	8.46235258	protein_coding	15	63796793	63886839	USP3
ENSG00000140459	2.471521042	1.16600992	protein_coding	15	74630100	74660081	CYP11A1
ENSG00000140463	6.640654398	6.854868327	protein_coding	15	72978527	73030817	BBS4
ENSG00000140464	10.81550688	10.94359578	protein_coding	15	74287014	74340153	PML
ENSG00000140465	4.165528823	6.155435249	protein_coding	15	75011883	75017951	CYP1A1
ENSG00000140470	1.16343121	0.950786998	protein_coding	15	100511794	100882210	ADAMTS17
ENSG00000140471	6.482942724	6.642271819	protein_coding	15	101099574	101143435	LINS
ENSG00000140474	8.023133844	8.66273344	protein_coding	15	75128457	75135687	ULK3
ENSG00000140479	1.407729925	0.697730409	protein_coding	15	101840818	102065405	PCSK6
ENSG00000140481	4.409500985	1.802319292	protein_coding	15	74509613	74628813	CCDC33
ENSG00000140497	9.263851576	9.075772566	protein_coding	15	75136071	75165706	SCAMP2
ENSG00000140505	0.499066092	0.697730409	protein_coding	15	75041185	75048543	CYP1A2
ENSG00000140506	0.499066092	0	protein_coding	15	75105057	75118099	LMAN1L
ENSG00000140511	6.634666104	6.831423059	protein_coding	15	89420519	89438857	HAPLN3
ENSG00000140519	0.499066092	0	protein_coding	15	89998680	90039844	RHCG
ENSG00000140521	9.615838797	9.697613846	protein_coding	15	89859534	89878092	POLG
ENSG00000140522	0	0.390640832	protein_coding	15	89753100	89764982	RLBP1
ENSG00000140525	9.609746388	9.84538481	protein_coding	15	89787180	89860492	FANCI
ENSG00000140526	9.158235462	8.647151213	protein_coding	15	89630690	89745591	ABHD2
ENSG00000140527	2.238690726	2.334191469	protein_coding	15	90234028	90286869	WDR93
ENSG00000140534	8.433338631	8.557113361	protein_coding	15	90118713	90174287	C15orf42
ENSG00000140538	1.616589159	0.390640832	protein_coding	15	88402982	88799999	NTRK3
ENSG00000140543	5.512107582	5.466810074	protein_coding	15	89054790	89089906	DET1
ENSG00000140545	7.834876234	8.434094156	protein_coding	15	89441916	89456642	MFGE8
ENSG00000140548	7.341508784	7.219404604	protein_coding	15	90544624	90625438	ZNF710
ENSG00000140553	9.972838789	9.972288065	protein_coding	15	91471728	91497323	UNC45A
ENSG00000140563	2.238690726	0	protein_coding	15	94774767	95023632	MCTP2
ENSG00000140564	10.44952202	10.54165143	protein_coding	15	91411822	91426688	FURIN
ENSG00000140575	11.4092195	11.47099045	protein_coding	15	90931450	91045475	IQGAP1
ENSG00000140577	8.614637248	8.565427152	protein_coding	15	91073157	91188577	CRTC3
ENSG00000140598	7.494827594	7.969895325	protein_coding	15	82422571	82555104	EFTUD1
ENSG00000140612	9.557686483	9.94751216	protein_coding	15	85212775	85259947	SEC11A
ENSG00000140623	0	0.390640832	protein_coding	16	4827670	4838522		12-sep
ENSG00000140632	9.532948302	9.253876247	protein_coding	16	4853204	4897343	GLYR1
ENSG00000140650	7.808521034	7.637287497	protein_coding	16	8882680	8943188	PMM2
ENSG00000140675	0.869158192	0	protein_coding	16	31494323	31502181	SLC5A2
ENSG00000140678	3.911897206	3.87606762	protein_coding	16	31366455	31394318	ITGAX
ENSG00000140682	8.18144485	8.705274089	protein_coding	16	31482906	31489281	TGFB1I1
ENSG00000140688	8.830404061	8.849385973	protein_coding	16	31500792	31520630	C16orf58
ENSG00000140691	7.754320745	7.885138265	protein_coding	16	31469401	31478487	ARMC5
ENSG00000140694	9.185117095	9.370301936	protein_coding	16	14529558	14726585	PARN
ENSG00000140718	8.510576602	8.727671407	protein_coding	16	53737875	54155853	FTO
ENSG00000140740	10.54040657	10.64976544	protein_coding	16	21963981	21994981	UQCRC2
ENSG00000140743	9.237422823	9.120790981	protein_coding	16	22357257	22448486	CDR2
ENSG00000140749	2.35979773	1.925536307	protein_coding	16	21652609	21663981	IGSF6
ENSG00000140750	9.592086026	9.581200526	protein_coding	16	24930710	25026675	ARHGAP17
ENSG00000140795	1.16343121	2.144285137	protein_coding	16	46740891	46824319	MYLK3
ENSG00000140798	0	0.390640832	protein_coding	16	48116884	48189929	ABCC12

ENSG00000140807	4.230497448	4.226940739	protein_coding	16	50582241	50670647	NKD1
ENSG00000140829	10.21581552	10.28721257	protein_coding	16	72127461	72146811	DHX38
ENSG00000140830	7.123655438	7.002128877	protein_coding	16	72078188	72128330	TXNL4B
ENSG00000140832	4.593635215	1.518964905	protein_coding	16	71660064	71676017	MARVELD3
ENSG00000140835	4.6658819	5.045726316	protein_coding	16	71560036	71574146	CHST4
ENSG00000140836	9.698536539	9.638612448	protein_coding	16	72816784	73093597	ZFHX3
ENSG00000140839	0.869158192	0	protein_coding	16	74442529	74455649	CLEC18B
ENSG00000140848	9.68845487	9.816320232	protein_coding	16	57126449	57181878	CPNE2
ENSG00000140853	9.199386995	10.2975873	protein_coding	16	57023397	57117443	NLRC5
ENSG00000140854	9.258020218	9.856074312	protein_coding	16	57769642	57791162	KATNB1
ENSG00000140859	10.21932096	10.67906696	protein_coding	16	57792129	57896957	KIFC3
ENSG00000140873	0.869158192	0.950786998	protein_coding	16	77281710	77469011	ADAMTS18
ENSG00000140876	4.131913373	4.448594745	protein_coding	16	77756411	77776157	NUDT7
ENSG00000140905	7.73205733	7.88892877	protein_coding	16	81115566	81130008	GCSH
ENSG00000140931	8.616157604	9.093127603	protein_coding	16	66637777	66647795	CMTM3
ENSG00000140932	0.499066092	1.16600992	protein_coding	16	66613351	66622178	CMTM2
ENSG00000140937	7.834876234	8.390640789	protein_coding	16	64977656	65160015	CDH11
ENSG00000140939	7.943018447	8.262094789	protein_coding	16	67204057	67209643	NOL3
ENSG00000140941	10.14648923	10.25489893	protein_coding	16	87417601	87438385	MAP1LC3B
ENSG00000140943	11.32944272	11.58957402	protein_coding	16	84087368	84150511	MBTPS1
ENSG00000140945	0.499066092	0.390640832	protein_coding	16	82660408	83830204	CDH13
ENSG00000140948	9.221529789	9.124814753	protein_coding	16	87439852	87525651	ZCCHC14
ENSG00000140950	9.250208226	8.749726325	protein_coding	16	84511681	84587639	KIAA1609
ENSG00000140961	7.23866569	6.79148052	protein_coding	16	83981887	83999937	OSGIN1
ENSG00000140968	3.077135474	2.334191469	protein_coding	16	85932409	85956197	IRF8
ENSG00000140983	10.20474267	10.276401	protein_coding	16	718086	724174	RHOT2
ENSG00000140986	3.004694206	2.420525079	protein_coding	16	1993975	2007607	RPL3L
ENSG00000140987	7.891216707	7.881337775	protein_coding	16	3432085	3451030	ZNF434
ENSG00000140988	12.38963843	12.49464367	protein_coding	16	2012053	2014861	RPS2
ENSG00000140990	9.435562348	9.881029944	protein_coding	16	2009509	2011976	NDUFB10
ENSG00000140992	8.891945431	8.725553248	protein_coding	16	2587965	2653189	PDPK1
ENSG00000140993	7.615275323	8.130601896	protein_coding	16	3340527	3355645	TIGD7
ENSG00000140995	8.457286611	9.208338404	protein_coding	16	90014333	90034468	DEF8
ENSG00000141002	10.14121895	10.58989654	protein_coding	16	89940000	89984641	TCF25
ENSG00000141012	9.409495425	9.53960799	protein_coding	16	88880142	88923378	GALNS
ENSG00000141013	7.402691859	7.426571535	protein_coding	16	90086037	90111383	GAS8
ENSG00000141026	7.787084643	8.17474298	protein_coding	17	17380300	17396535	MED9
ENSG00000141027	10.8250701	11.23303133	protein_coding	17	15934718	16119010	NCOR1
ENSG00000141028	0.499066092	1.353254395	lincRNA	17	13927802	13928991	
ENSG00000141030	9.93557361	10.62951761	protein_coding	17	17150134	17184607	COPS3
ENSG00000141034	8.164919416	8.634788863	protein_coding	17	17942736	17971718	C17orf39
ENSG00000141040	6.846372398	7.541804862	protein_coding	17	16454701	16472520	ZNF287
ENSG00000141048	0	0.390640832	protein_coding	17	10346607	10372876	MYH4
ENSG00000141068	8.915598341	8.35682763	protein_coding	17	25783670	25953461	KSR1
ENSG00000141076	10.21781967	10.28289766	protein_coding	16	69165194	69202941	CIRH1A
ENSG00000141084	8.724437638	8.89344947	protein_coding	16	67757005	67840555	RANBP10
ENSG00000141086	4.962122459	4.406904905	protein_coding	16	67963473	67965770	CTRL
ENSG00000141098	7.72361917	8.013952351	protein_coding	16	67708436	67753324	GFOD2
ENSG00000141101	9.733277703	9.768254884	protein_coding	16	69775770	69788843	NOB1
ENSG00000141127	8.582333986	9.132828786	protein_coding	17	18743398	18834581	PRPSAP2
ENSG00000141140	10.08248848	9.790209352	protein_coding	17	34851482	34891305	MYO19
ENSG00000141141	8.833021386	8.787783083	protein_coding	17	35969826	36003493	DDX52
ENSG00000141150	0.869158192	0	protein_coding	17	34058679	34070540	RASL10B
ENSG00000141179	7.680671792	7.460081189	protein_coding	17	53828356	53854748	PCTP
ENSG00000141194	0	0.390640832	protein_coding	17	56232494	56233517	OR4D1
ENSG00000141198	8.16075831	7.756190105	protein_coding	17	52978052	53039328	TOM1L1
ENSG00000141200	0	0.390640832	protein_coding	17	51900239	51902573	KIF2B
ENSG00000141219	7.575190993	8.196317182	protein_coding	17	71228372	71245091	C17orf80
ENSG00000141232	8.307246212	7.694718794	protein_coding	17	48939584	48945339	TOB1
ENSG00000141252	9.600559263	9.580028658	protein_coding	17	411908	618096	VPS53
ENSG00000141258	9.645920794	10.0955015	protein_coding	17	2240806	2284344	SGSM2
ENSG00000141279	10.11080508	9.791222404	protein_coding	17	45600306	45700642	NPEPPS
ENSG00000141294	3.989024711	4.29708397	protein_coding	17	45908993	45915077	LRRC46

ENSG00000141295	7.657232808	7.151604372	protein_coding	17	45915049	45918699	SCRN2
ENSG00000141298	8.536501169	8.21757351	protein_coding	17	27952956	28257170	SSH2
ENSG00000141314	0	0.697730409	protein_coding	17	30593195	30651680	RHBDL3
ENSG00000141337	6.283060568	6.89311646	protein_coding	17	66255323	66418872	ARSG
ENSG00000141349	9.267726094	9.643108201	protein_coding	17	42148098	42153711	G6PC3
ENSG00000141367	12.60784325	12.43001577	protein_coding	17	57697219	57773671	CLTC
ENSG00000141371	0.869158192	1.353254395	protein_coding	17	58469790	58508782	C17orf64
ENSG00000141376	7.106450572	7.616862529	protein_coding	17	58755172	59470198	BCAS3
ENSG00000141378	8.041355121	8.432796436	protein_coding	17	57774667	57784987	PTRH2
ENSG00000141380	9.560058142	9.718000208	protein_coding	18	23596219	23671164	SS18
ENSG00000141384	7.847875458	7.752033772	protein_coding	18	23806409	23971642	TAF4B
ENSG00000141385	9.868268606	9.764649066	protein_coding	18	12328943	12377253	AFG3L2
ENSG00000141391	4.689181911	4.777836593	protein_coding	18	12407895	12432234	SLMO1
ENSG00000141401	9.92702399	8.748683701	protein_coding	18	11981427	12030876	IMPA2
ENSG00000141404	4.131913373	3.845183986	protein_coding	18	11689184	11885684	GNAL
ENSG00000141424	9.657780134	9.917208266	protein_coding	18	33688495	33709357	SLC39A6
ENSG00000141425	9.12764907	8.915915748	protein_coding	18	33569787	33647539	RPRD1A
ENSG00000141428	6.522395495	7.0637896	protein_coding	18	33552046	33559241	C18orf21
ENSG00000141429	9.845117281	9.862353807	protein_coding	18	33161003	33291798	GALNT1
ENSG00000141431	0.499066092	0	protein_coding	18	31158541	31327377	ASXL3
ENSG00000141434	1.16343121	0.390640832	protein_coding	18	29769987	29800364	MEP1B
ENSG00000141437	0	0.390640832	protein_coding	18	29339525	29340843	MCART2
ENSG00000141441	7.50471092	6.881748114	protein_coding	18	29843506	30050447	FAM59A
ENSG00000141446	8.002356002	8.114504097	protein_coding	18	19109264	19180845	ESCO1
ENSG00000141447	8.815923273	8.524589435	protein_coding	18	21742008	21977823	OSBPL1A
ENSG00000141448	8.649206367	8.40394723	protein_coding	18	19749404	19782491	GATA6
ENSG00000141449	6.592037289	6.795524906	protein_coding	18	18822203	19102791	GREB1L
ENSG00000141452	7.959880444	7.926295939	protein_coding	18	21083462	21111771	C18orf8
ENSG00000141456	11.15842886	11.33189317	protein_coding	17	4574680	4607632	
ENSG00000141458	10.04007652	9.611339686	protein_coding	18	21111401	21166451	NPC1
ENSG00000141469	0.499066092	0	protein_coding	18	43304092	43332485	SLC14A1
ENSG00000141480	9.355904607	9.822279296	protein_coding	17	4613784	4624795	ARRB2
ENSG00000141497	2.10647801	1.353254395	protein_coding	17	4643319	4649411	ZMYND15
ENSG00000141499	9.151960225	9.546823838	protein_coding	17	7589389	7606820	WRAP53
ENSG00000141503	11.34740348	11.53119713	protein_coding	17	4736635	4801355	MINK1
ENSG00000141504	8.489164384	9.823270084	protein_coding	17	7529552	7531194	SAT2
ENSG00000141505	3.274897671	4.021284656	protein_coding	17	7076750	7082883	ASGR1
ENSG00000141506	3.004694206	4.448594745	protein_coding	17	8782228	8869024	PIK3R5
ENSG00000141510	12.28061402	12.51003135	protein_coding	17	7565097	7590856	TP53
ENSG00000141519	5.207774174	5.32851069	protein_coding	17	78010435	78074412	CCDC40
ENSG00000141522	11.93172359	11.58738872	protein_coding	17	79825598	79829282	ARHGDIA
ENSG00000141524	7.161632221	3.453019579	protein_coding	17	76108999	76128488	TMC6
ENSG00000141526	11.44921933	11.46972551	protein_coding	17	80186293	80197369	SLC16A3
ENSG00000141527	5.729976786	5.536128679	protein_coding	17	78152274	78182855	CARD14
ENSG00000141540	7.923503136	8.457255683	protein_coding	17	72209653	72258155	TTYH2
ENSG00000141542	5.707331524	5.776071827	protein_coding	17	80614944	80656598	RAB40B
ENSG00000141543	9.878440025	9.829693678	protein_coding	17	78109013	78120982	EIF4A3
ENSG00000141551	9.335744919	9.130429247	protein_coding	17	80202246	80231576	CSNK1D
ENSG00000141552	8.826469148	9.150304507	protein_coding	17	79849599	79858363	ANAPC11
ENSG00000141556	9.832747078	9.651500164	protein_coding	17	80709940	80914691	TBCD
ENSG00000141560	8.12275821	8.176294739	protein_coding	17	80674582	80685892	FN3KRP
ENSG00000141562	8.12703021	8.605140645	protein_coding	17	80416060	80446141	NARF
ENSG00000141564	9.683387493	9.467928508	protein_coding	17	78518619	78940173	RPTOR
ENSG00000141568	9.249228746	8.936206949	protein_coding	17	80477589	80562483	FOXK2
ENSG00000141569	9.116949866	9.326251289	protein_coding	17	73885041	73893084	TRIM65
ENSG00000141570	6.328285199	6.077857763	protein_coding	17	77765931	77775482	CBX8
ENSG00000141574	5.40360309	5.792353798	protein_coding	17	80278900	80291921	SECTM1
ENSG00000141576	5.360713447	5.112089758	protein_coding	17	74138534	74236390	RNF157
ENSG00000141577	7.993024472	7.632773539	protein_coding	17	79163393	79196799	AZI1
ENSG00000141579	2.671945279	1.667587519	protein_coding	17	80787311	80797931	ZNF750
ENSG00000141580	9.524067267	9.606743677	protein_coding	17	80572438	80606411	WDR45L
ENSG00000141582	8.447071828	7.892709342	protein_coding	17	77806955	77813228	CBX4
ENSG00000141622	4.77879189	2.721932731	protein_coding	18	43914187	44040783	RNF165

ENSG00000141627	8.70742151	8.891561392	protein_coding	18	46570172	46987079	DYM
ENSG00000141642	5.175139844	4.932754837	protein_coding	18	48494386	48514491	ELAC1
ENSG00000141644	9.179986301	9.379761309	protein_coding	18	47793887	47808144	MBD1
ENSG00000141646	9.342189974	9.334613891	protein_coding	18	48494410	48611415	SMAD4
ENSG00000141655	7.161632221	7.495318771	protein_coding	18	59992520	60054899	TNFRSF11A
ENSG00000141664	8.135536446	8.288162655	protein_coding	18	60190658	60245793	ZCCHC2
ENSG00000141665	2.471521042	3.781359661	protein_coding	18	71740588	71815100	FBXO15
ENSG00000141682	9.125515568	8.865827498	protein_coding	18	57567180	57571538	PMAIP1
ENSG00000141696	9.021506037	8.980400792	protein_coding	17	39958199	39968855	LEPREL4
ENSG00000141698	9.285033801	9.43525094	protein_coding	17	39981335	39992523	NT5C3L
ENSG00000141699	9.407740769	8.935290787	protein_coding	17	40731528	40761445	FAM134C
ENSG00000141720	9.475476535	9.209096633	protein_coding	17	36921942	36956158	PIP4K2B
ENSG00000141736	9.940436455	9.632972982	protein_coding	17	37844393	37884915	ERBB2
ENSG00000141738	8.217949183	6.733635362	protein_coding	17	37894187	37903545	GRB7
ENSG00000141741	7.96466218	8.104758487	protein_coding	17	37885409	37887040	MIEN1
ENSG00000141744	0	0.697730409	protein_coding	17	37824234	37826728	PNMT
ENSG00000141748	1.407729925	1.16600992	protein_coding	17	37313147	37323318	ARL5C
ENSG00000141750	0.499066092	1.16600992	protein_coding	17	37366789	37382040	STAC2
ENSG00000141753	7.182306324	5.700439663	protein_coding	17	38599702	38613983	IGFBP4
ENSG00000141756	10.68033139	11.536644	protein_coding	17	39968962	39979465	FKBP10
ENSG00000141759	8.738466022	8.966118473	protein_coding	18	77732869	77748532	TXNL4A
ENSG00000141837	6.228441838	6.775188634	protein_coding	19	13317256	13617274	CACNA1A
ENSG00000141854	3.393122761	3.64455972	protein_coding	19	14183348	14185873	
ENSG00000141858	9.622662143	9.509147322	protein_coding	19	14198652	14201848	SAMD1
ENSG00000141867	11.93477291	11.74887797	protein_coding	19	15348301	15443356	BRD4
ENSG00000141873	9.639206824	9.751176567	protein_coding	19	2732202	2740074	SLC39A3
ENSG00000141905	10.27620418	10.06147816	protein_coding	19	3359616	3463603	NFIC
ENSG00000141933	7.292905885	7.877527247	protein_coding	19	507497	519653	TPGS1
ENSG00000141934	10.03440201	9.858975937	protein_coding	19	281048	291435	PPAP2C
ENSG00000141956	7.666067113	7.887034762	protein_coding	21	43218385	43299591	PRDM15
ENSG00000141959	11.12384425	11.67547565	protein_coding	21	45719934	45747259	PFKL
ENSG00000141965	9.569505979	9.464757353	protein_coding	19	4791728	4795571	FEM1A
ENSG00000141968	7.850461307	7.946442679	protein_coding	19	6772722	6857371	VAV1
ENSG00000141971	9.461166635	9.522629316	protein_coding	19	17516503	17544533	FAM125A
ENSG00000141977	2.762599152	3.183544561	protein_coding	19	16272179	16284286	CIB3
ENSG00000141979	0.869158192	0.950786998	protein_coding	19	16589875	16739015	CALR3
ENSG00000141985	11.28664373	11.58271565	protein_coding	19	4360370	4400522	SH3GL1
ENSG00000141994	9.379372099	9.757410281	protein_coding	19	5785155	5791249	DUS3L
ENSG00000142002	11.49062868	10.89329685	protein_coding	19	4675238	4723875	DPP9
ENSG00000142039	7.627388205	7.370740503	protein_coding	19	41816094	41830785	CCDC97
ENSG00000142046	4.097495944	4.528525526	protein_coding	19	41856816	41889988	TMEM91
ENSG00000142065	5.255381949	5.496926774	protein_coding	19	36827162	36870078	ZFP14
ENSG00000142082	7.813830732	7.699043321	protein_coding	11	215458	236931	SIRT3
ENSG00000142089	11.96252829	12.66465318	protein_coding	11	319669	321340	IFITM3
ENSG00000142102	9.288851923	9.790715967	protein_coding	11	289135	296107	ATHL1
ENSG00000142156	10.51821653	11.39655904	protein_coding	21	47401651	47424964	COL6A1
ENSG00000142166	8.813274715	9.05483585	protein_coding	21	34696734	34732168	IFNAR1
ENSG00000142168	9.941649609	10.35626402	protein_coding	21	33031935	33041244	SOD1
ENSG00000142173	8.918065711	9.564114571	protein_coding	21	47518011	47552763	COL6A2
ENSG00000142178	10.87830713	9.177677745	protein_coding	21	44834395	44847008	SIK1
ENSG00000142182	1.616589159	2.039052734	protein_coding	21	45666222	45682099	DNMT3L
ENSG00000142185	7.214780754	9.305839261	protein_coding	21	45770046	45862964	TRPM2
ENSG00000142186	10.33000324	10.37564205	protein_coding	11	65292548	65306175	SCYL1
ENSG00000142188	6.509364082	7.249200135	protein_coding	21	34804792	34853499	TMEM50B
ENSG00000142192	12.53526683	12.89119321	protein_coding	21	27252861	27543446	APP
ENSG00000142197	7.115078652	6.408769788	protein_coding	21	37529080	37711195	DOPEY2
ENSG00000142207	9.561637084	9.078264676	protein_coding	21	33683329	33765335	URB1
ENSG00000142208	11.21068739	10.4017445	protein_coding	14	105235686	105262088	AKT1
ENSG00000142227	9.296457985	9.908831886	protein_coding	19	48828629	48833810	EMP3
ENSG00000142230	10.68721878	10.81128162	protein_coding	19	47634080	47713892	SAE1
ENSG00000142233	4.543379137	3.87606762	protein_coding	19	49164666	49176264	NTN5
ENSG00000142235	7.153278907	5.212303549	protein_coding	19	48988528	49016446	LMTK3
ENSG00000142252	7.527512071	7.948260326	protein_coding	19	45582530	45594782	GEMIN7

ENSG00000142273	7.08903805	0.697730409	protein_coding	19	45281126	45303891	CBLC
ENSG00000142279	3.004694206	3.027231696	protein_coding	19	34971874	34992085	WTIP
ENSG00000142303	5.575999561	6.766973175	protein_coding	19	8645126	8673183	ADAMTS10
ENSG00000142319	0.499066092	0.950786998	protein_coding	5	1392909	1445545	SLC6A3
ENSG00000142327	8.803966282	8.222087975	protein_coding	2	241505221	241518149	RNPEPL1
ENSG00000142330	8.191677941	7.844724842	protein_coding	2	241526133	241557122	CAPN10
ENSG00000142347	5.625150983	6.210997888	protein_coding	19	8585689	8642461	MYO1F
ENSG00000142396	8.803966282	9.208338404	processed_transc	19	58816715	58827023	
ENSG00000142405	1.960915222	0.697730409	protein_coding	19	54296857	54327648	NLRP12
ENSG00000142408	7.737655476	8.821890565	protein_coding	19	54466294	54493469	CACNG8
ENSG00000142409	9.707122248	9.459031647	protein_coding	19	56598732	56632649	ZNF787
ENSG00000142444	8.53489446	8.85617872	protein_coding	19	11039424	11040914	C19orf52
ENSG00000142449	4.381167248	3.96493948	protein_coding	19	8130287	8212650	FBN3
ENSG00000142453	11.88661109	11.72230247	protein_coding	19	10982253	11033448	CARM1
ENSG00000142459	9.513500523	9.359414727	protein_coding	19	7895161	7929861	EVI5L
ENSG00000142494	0.869158192	0.697730409	protein_coding	17	19437141	19482347	SLC47A1
ENSG00000142507	10.65133646	11.05174357	protein_coding	17	4699439	4701798	PSMB6
ENSG00000142511	0.499066092	0	protein_coding	19	51273858	51274928	GPR32
ENSG00000142512	3.554067925	3.906303962	protein_coding	19	51913275	51921057	SIGLEC10
ENSG00000142513	3.274897671	1.925536307	protein_coding	19	51293672	51298481	ACPT
ENSG00000142528	7.706593329	7.856387513	protein_coding	19	50529212	50552029	ZNF473
ENSG00000142530	4.292665995	3.748356452	protein_coding	19	50970047	50980003	FAM71E1
ENSG00000142534	11.69435783	12.41776886	protein_coding	19	49999634	50002944	RPS11
ENSG00000142539	0	0.390640832	protein_coding	19	50922195	50932246	SPIB
ENSG00000142541	11.39998188	12.14253511	protein_coding	19	49990811	49995565	RPL13A
ENSG00000142544	5.869093502	5.9163892	protein_coding	19	51600863	51611647	CTU1
ENSG00000142546	8.219950371	8.457255683	protein_coding	19	50058970	50083813	NOSIP
ENSG00000142549	2.471521042	2.579085888	protein_coding	19	51815102	51833608	IGLON5
ENSG00000142552	8.884394801	10.29401823	protein_coding	19	50030875	50046889	RCN3
ENSG00000142556	8.183497283	8.379906558	protein_coding	19	52516021	52531680	ZNF614
ENSG00000142583	3.448790144	3.493416095	protein_coding	1	9095166	9148537	SLC2A5
ENSG00000142599	9.676117367	9.787165921	protein_coding	1	8412457	8877702	RERE
ENSG00000142606	0.869158192	2.420525079	protein_coding	1	2522078	2564481	MMEL1
ENSG00000142609	1.407729925	2.039052734	protein_coding	1	1853396	1935276	C1orf222
ENSG00000142611	6.616550626	5.832272499	protein_coding	1	2985732	3355185	PRDM16
ENSG00000142619	6.068837212	3.081239798	protein_coding	1	17575593	17610728	PADI3
ENSG00000142621	4.322773689	4.17820932	protein_coding	1	15573768	15726858	FHAD1
ENSG00000142623	3.448790144	2.971122874	protein_coding	1	17531621	17572500	PADI1
ENSG00000142627	10.81682972	10.30256921	protein_coding	1	16450832	16482582	EPHA2
ENSG00000142632	6.979904289	7.029859489	protein_coding	1	16524349	16539140	ARHGEF19
ENSG00000142634	10.87291199	9.925997554	protein_coding	1	15736391	15756839	EFHD2
ENSG00000142655	7.481543613	7.724721747	protein_coding	1	10532345	10690815	PEX14
ENSG00000142657	10.01147814	9.840010211	protein_coding	1	10458649	10480233	PGD
ENSG00000142661	3.004694206	2.788380093	protein_coding	1	24382525	24438665	MYOM3
ENSG00000142669	10.35115602	10.40671051	protein_coding	1	26605667	26608007	SH3BGRL3
ENSG00000142675	8.011627561	7.405556541	protein_coding	1	26503894	26516377	CNKSR1
ENSG00000142676	12.43125725	12.70854633	protein_coding	1	24018269	24022915	RPL11
ENSG00000142677	4.491309013	3.493416095	protein_coding	1	24446261	24469611	IL22RA1
ENSG00000142684	1.616589159	2.144285137	protein_coding	1	26496362	26498551	ZNF593
ENSG00000142686	7.15746161	7.405556541	protein_coding	1	36179476	36185073	C1orf216
ENSG00000142687	9.049908262	8.972384393	protein_coding	1	35899091	36023551	KIAA0319L
ENSG00000142694	5.255381949	5.98174124	protein_coding	1	36787632	36789755	FAM176B
ENSG00000142700	0	0.697730409	protein_coding	1	50883222	50889172	DMRTA2
ENSG00000142731	8.269098624	8.393311914	protein_coding	4	128802016	128820350	PLK4
ENSG00000142733	8.588543011	8.677053487	protein_coding	1	27681670	27693383	MAP3K6
ENSG00000142748	0.869158192	0.390640832	protein_coding	1	27695603	27701315	FCN3
ENSG00000142751	7.784382559	7.703354924	protein_coding	1	27205773	27216788	GPN2
ENSG00000142765	7.296703257	4.489113623	protein_coding	1	27668513	27680421	SYTL1
ENSG00000142784	9.183066967	9.263397979	protein_coding	1	27561007	27635110	WDTC1
ENSG00000142794	8.011627561	8.229580833	protein_coding	1	21766621	21811498	NBPF3
ENSG00000142798	11.61388847	11.49978204	protein_coding	1	22148738	22263790	HSPG2
ENSG00000142856	6.469548347	7.070480904	protein_coding	1	63906441	64059392	ITGB3BP
ENSG00000142864	11.48710177	11.46131717	protein_coding	1	67873493	67896098	SERBP1

ENSG00000142867	8.329659793	7.596144237	protein_coding	1	85731931	85743771	BCL10
ENSG00000142871	12.7627528	12.28229911	protein_coding	1	86046444	86049645	CYR61
ENSG00000142875	7.017207048	7.033288635	protein_coding	1	84543745	84704181	PRKACB
ENSG00000142892	8.081530559	8.386624806	protein_coding	1	77554675	77685115	PIGK
ENSG00000142910	10.22779886	8.590084877	protein_coding	1	32042116	32053288	TINAGL1
ENSG00000142920	4.962122459	5.248158938	protein_coding	1	33546705	33586131	ADC
ENSG00000142937	11.72021353	12.24535336	protein_coding	1	45240923	45244451	RPS8
ENSG00000142945	9.322767784	9.502977244	protein_coding	1	45205490	45233439	KIF2C
ENSG00000142949	12.89552653	12.57639643	protein_coding	1	43990858	44089343	PTPRF
ENSG00000142959	1.616589159	0.390640832	protein_coding	1	45249257	45253377	BEST4
ENSG00000142961	6.646617938	6.549526374	protein_coding	1	47073387	47082563	MOB3C
ENSG00000143001	1.960915222	0.697730409	protein_coding	1	55446465	55457966	TMEM61
ENSG00000143013	7.819120959	8.492561507	protein_coding	1	87794151	87812788	LMO4
ENSG00000143028	0.869158192	3.748356452	protein_coding	1	110009180	110024759	SYPL2
ENSG00000143033	7.720795447	7.966311947	protein_coding	1	93544792	93604638	MTF2
ENSG00000143036	3.950976309	3.935919592	protein_coding	1	95285898	95360802	SLC44A3
ENSG00000143061	8.694526397	8.693401904	protein_coding	1	117117031	117210375	IGSF3
ENSG00000143067	7.621344476	7.558560655	protein_coding	1	120162045	120190396	ZNF697
ENSG00000143079	7.269909329	7.426571535	protein_coding	1	112938803	113003786	CTTNBP2NL
ENSG00000143093	4.962122459	4.794098771	protein_coding	1	110574199	110597263	FAM40A
ENSG00000143105	0	0.390640832	protein_coding	1	111059839	111061797	KCNA10
ENSG00000143106	9.553725034	9.934275143	protein_coding	1	109941653	109969062	PSMA5
ENSG00000143107	0.499066092	0	protein_coding	1	109255279	109285365	FNDC7
ENSG00000143110	1.960915222	2.420525079	protein_coding	1	112016414	112021134	C1orf162
ENSG00000143119	1.799000381	2.242360793	protein_coding	1	111415772	111442550	CD53
ENSG00000143126	6.528867325	5.446377228	protein_coding	1	109792641	109818377	CELSR2
ENSG00000143127	3.871730003	3.993387124	protein_coding	1	145524891	145543868	ITGA10
ENSG00000143140	0.499066092	0	protein_coding	1	147228332	147245484	GJA5
ENSG00000143147	5.036678094	4.810179682	protein_coding	1	168053997	168106821	GPR161
ENSG00000143149	9.85609616	9.989160763	protein_coding	1	165631453	165668100	ALDH9A1
ENSG00000143153	9.135091581	8.825850709	protein_coding	1	169074935	169101960	ATP1B1
ENSG00000143155	8.054871625	8.46616347	protein_coding	1	168148171	168169950	TIPRL
ENSG00000143156	7.384964606	7.648510984	protein_coding	1	169101769	169337205	NME7
ENSG00000143157	9.551342933	9.452002561	protein_coding	1	166808681	166825581	POGK
ENSG00000143158	7.311793575	8.205465413	protein_coding	1	167885967	167906278	BRP44
ENSG00000143162	8.205883411	8.476276902	protein_coding	1	167498914	167523004	CREG1
ENSG00000143164	8.983137923	9.33252778	protein_coding	1	167905021	168045081	DCAF6
ENSG00000143167	0	0.697730409	protein_coding	1	167022073	167059868	GPA33
ENSG00000143178	4.75690578	4.903073497	protein_coding	1	168250278	168283664	TBX19
ENSG00000143179	10.93879114	10.93926111	protein_coding	1	165796768	165880855	UCK2
ENSG00000143183	9.326487463	9.903220491	protein_coding	1	165696032	165738417	TMCO1
ENSG00000143184	0.499066092	0.390640832	protein_coding	1	168545711	168551315	XCL1
ENSG00000143190	8.786518219	8.726612716	protein_coding	1	167190066	167396582	POU2F1
ENSG00000143194	0.499066092	0	protein_coding	1	166958346	166991451	MAEL
ENSG00000143198	8.568265199	9.52750079	protein_coding	1	165600098	165631033	MGST3
ENSG00000143199	1.616589159	1.353254395	protein_coding	1	167778625	167883453	ADCY10
ENSG00000143207	8.154494062	8.343077302	protein_coding	1	175911248	176176629	RFWD2
ENSG00000143217	4.6658819	1.667587519	protein_coding	1	161040785	161059389	PVRL4
ENSG00000143222	9.147761519	9.880078052	protein_coding	1	161122566	161128646	UFC1
ENSG00000143224	7.621344476	8.405271149	protein_coding	1	161136200	161147803	PPOX
ENSG00000143226	2.928422289	1.667587519	protein_coding	1	161475220	161493803	FCGR2A
ENSG00000143228	8.561968095	9.124010896	protein_coding	1	163236366	163325554	NUF2
ENSG00000143248	3.950976309	4.65855978	protein_coding	1	163080911	163187426	RGS5
ENSG00000143252	9.820929282	10.09016079	protein_coding	1	161284047	161332984	SDHC
ENSG00000143256	10.16789832	10.71314019	protein_coding	1	161070346	161087901	PFDN2
ENSG00000143257	3.911897206	3.679994897	protein_coding	1	161199456	161208092	NR1I3
ENSG00000143258	8.986668406	9.170688365	protein_coding	1	161129240	161135513	USP21
ENSG00000143278	0.499066092	0	protein_coding	1	197008321	197036397	F13B
ENSG00000143294	10.06143835	10.02448085	protein_coding	1	156720402	156770607	PRCC
ENSG00000143303	7.974178365	8.078443681	protein_coding	1	156698234	156706752	RRNAD1
ENSG00000143314	9.49130376	9.990925412	protein_coding	1	156707095	156711382	MRPL24
ENSG00000143315	7.165790811	7.447284805	protein_coding	1	159997462	160001783	PIGM
ENSG00000143318	1.799000381	1.667587519	protein_coding	1	160160285	160171676	CASQ1

ENSG00000143319	9.868906425	9.658178764	protein_coding	1	156691683	156698591	ISG20L2
ENSG00000143320	8.162840364	5.072639111	protein_coding	1	156669398	156675608	CRABP2
ENSG00000143321	13.57712274	13.4851589	protein_coding	1	156711899	156736717	HDGF
ENSG00000143322	9.14038425	9.275031414	protein_coding	1	179068462	179198819	ABL2
ENSG00000143324	8.715954662	8.349968848	protein_coding	1	180601140	180859387	XPR1
ENSG00000143333	5.948480845	6.779278886	protein_coding	1	182567758	182573543	RGS16
ENSG00000143337	8.547698305	8.661625988	protein_coding	1	179851177	179888412	TOR1AIP1
ENSG00000143341	1.407729925	3.368666104	protein_coding	1	185703683	186160085	HMCN1
ENSG00000143344	5.431503808	6.445267191	protein_coding	1	183605208	183897666	RGL1
ENSG00000143353	6.907250452	7.222412001	protein_coding	1	219347186	219386207	LYPLAL1
ENSG00000143355	0.499066092	1.16600992	protein_coding	1	197881618	197904608	LHX9
ENSG00000143363	8.613115289	8.593573257	protein_coding	1	150980896	151008189	PRUNE
ENSG00000143365	3.554067925	2.851901313	protein_coding	1	151778547	151804348	RORC
ENSG00000143367	9.241368875	9.570619293	protein_coding	1	151512781	151556059	TUFT1
ENSG00000143368	10.61263604	10.42183387	protein_coding	1	149895209	149900236	SF3B4
ENSG00000143369	6.129712275	8.232567113	protein_coding	1	150480538	150486265	ECM1
ENSG00000143373	9.34494335	9.159748241	protein_coding	1	151254094	151264381	ZNF687
ENSG00000143374	9.457778871	9.427453327	protein_coding	1	150459887	150480078	TARS2
ENSG00000143375	8.103376106	6.729413443	protein_coding	1	151482986	151511168	CGN
ENSG00000143376	9.930694319	9.688943177	protein_coding	1	151584541	151671567	SNX27
ENSG00000143379	8.821205846	9.095589939	protein_coding	1	150898739	150937213	SETDB1
ENSG00000143382	6.902274257	7.23139676	protein_coding	1	150521884	150533413	ADAMTSL4
ENSG00000143384	12.10874659	11.83441318	protein_coding	1	150547032	150552073	MCL1
ENSG00000143387	5.889353623	5.555337277	protein_coding	1	150768684	150780799	CTSK
ENSG00000143390	9.937399098	10.15730836	protein_coding	1	151313116	151319833	RFX5
ENSG00000143393	10.36161724	10.49919564	protein_coding	1	151264273	151300191	PI4KB
ENSG00000143398	10.26945679	10.21921274	protein_coding	1	151170425	151222012	PIP5K1A
ENSG00000143401	10.97390063	11.16588355	protein_coding	1	150190717	150208504	ANP32E
ENSG00000143409	7.123655438	7.475288518	protein_coding	1	150969025	150980851	FAM63A
ENSG00000143412	4.863159938	4.604259349	protein_coding	1	150954493	150968110	ANXA9
ENSG00000143416	7.997697782	7.053694091	protein_coding	1	151336778	151345209	SELENBP1
ENSG00000143418	10.35115602	10.69485148	protein_coding	1	150933059	150947479	CERS2
ENSG00000143420	10.30004761	10.66587203	protein_coding	1	150573327	150602088	ENSA
ENSG00000143429	5.718698586	6.559073862	pseudogene	2	91823266	91847975	
ENSG00000143434	6.146643566	6.885547525	protein_coding	1	151104161	151119104	SEMA6C
ENSG00000143436	9.4441477	9.640862076	protein_coding	1	151732119	151736040	MRPL9
ENSG00000143437	9.292659966	9.291591823	protein_coding	1	150782181	150849244	ARNT
ENSG00000143442	10.22580854	10.37361447	protein_coding	1	151375200	151431941	POGZ
ENSG00000143443	5.498982484	5.125003832	protein_coding	1	151020216	151024462	C1orf56
ENSG00000143450	3.393122761	2.721932731	protein_coding	1	151735445	151743808	OAZ3
ENSG00000143452	6.998676229	6.651226688	protein_coding	1	150670536	150693364	HORMAD1
ENSG00000143457	8.742648075	9.037011115	protein_coding	1	150618701	150669672	GOLPH3L
ENSG00000143458	8.023133844	8.122575449	protein_coding	1	151043054	151098018	GABPB2
ENSG00000143466	8.899456749	9.362144241	protein_coding	1	206643791	206670223	IKBKE
ENSG00000143469	2.10647801	2.788380093	protein_coding	1	210111538	210337636	SYT14
ENSG00000143473	0.499066092	0	protein_coding	1	210856555	211307457	KCNH1
ENSG00000143476	8.899456749	9.13522434	protein_coding	1	212208919	212280742	DTL
ENSG00000143479	6.670228606	7.03670965	protein_coding	1	206808881	206857764	DYRK3
ENSG00000143486	8.583888749	9.012896966	protein_coding	1	206744620	206785904	EIF2D
ENSG00000143493	8.33889698	8.354088029	protein_coding	1	212113741	212208884	INTS7
ENSG00000143494	1.960915222	2.144285137	protein_coding	1	213123862	213165379	VASH2
ENSG00000143498	6.882196036	6.637773454	protein_coding	1	222731244	222763275	TAF1A
ENSG00000143499	8.457286611	8.371803145	protein_coding	1	214454445	214510474	SMYD2
ENSG00000143502	5.301468762	4.826083323	protein_coding	1	223394161	223537544	SUSD4
ENSG00000143507	7.850461307	7.512619979	protein_coding	1	221874766	221915518	DUSP10
ENSG00000143512	4.322773689	3.781359661	protein_coding	1	222695602	222721445	HHIPL2
ENSG00000143514	9.30213633	9.805832274	protein_coding	1	223967601	224033674	TP53BP2
ENSG00000143515	10.03837651	10.43226174	protein_coding	1	154298029	154323783	ATP8B2
ENSG00000143520	1.616589159	2.721932731	protein_coding	1	152321213	152332482	FLG2
ENSG00000143537	11.49683168	12.2544723	protein_coding	1	155023042	155035252	ADAM15
ENSG00000143543	10.47739354	10.83848808	protein_coding	1	153946745	153950164	JTB
ENSG00000143545	9.361354163	10.06231762	protein_coding	1	153954127	153958834	RAB13
ENSG00000143546	1.407729925	1.16600992	protein_coding	1	153362508	153363664	S100A8

ENSG00000143549	12.30273399	12.34400654	protein_coding	1	154127784	154167124	TPM3
ENSG00000143552	2.471521042	1.802319292	protein_coding	1	153965161	154127592	NUP210L
ENSG00000143553	8.120617457	8.7455513	protein_coding	1	153631130	153634306	SNAPIN
ENSG00000143554	6.922076877	6.097646709	protein_coding	1	153746830	153752633	SLC27A3
ENSG00000143569	11.57650097	11.76559685	protein_coding	1	154192655	154243986	UBAP2L
ENSG00000143570	11.4460171	11.53754982	protein_coding	1	153931575	153940188	SLC39A1
ENSG00000143575	9.979350975	10.41494939	protein_coding	1	154244987	154248351	HAX1
ENSG00000143578	6.92698535	7.416102302	protein_coding	1	153940010	153946839	CREB3L4
ENSG00000143590	5.695874189	7.106738413	protein_coding	1	155036224	155060014	EFNA3
ENSG00000143603	5.286269488	6.475832214	protein_coding	1	154679902	154842756	KCNN3
ENSG00000143612	10.899376	11.06771712	protein_coding	1	154179196	154193104	C1orf43
ENSG00000143614	10.06199625	9.784116057	protein_coding	1	153777201	153895451	GATAD2B
ENSG00000143621	11.54132321	11.88226087	protein_coding	1	153634512	153643524	ILF2
ENSG00000143622	8.2535526	8.053329862	protein_coding	1	155867599	155881195	RIT1
ENSG00000143624	11.03342956	11.21067871	protein_coding	1	153700543	153746555	INTS3
ENSG00000143627	1.16343121	1.16600992	protein_coding	1	155259086	155271225	PKLR
ENSG00000143630	5.948480845	6.59664425	protein_coding	1	155247374	155259639	HCN3
ENSG00000143631	5.625150983	6.186567173	protein_coding	1	152274651	152297679	FLG
ENSG00000143632	1.616589159	1.925536307	protein_coding	1	229566992	229569845	ACTA1
ENSG00000143633	6.907250452	7.652976045	protein_coding	1	231359509	231376933	C1orf131
ENSG00000143641	11.24352018	11.35708252	protein_coding	1	230193536	230417870	GALNT2
ENSG00000143643	7.67776266	8.060069742	protein_coding	1	231041989	231114621	TTC13
ENSG00000143653	8.307246212	9.099684516	protein_coding	1	246887349	246931439	SCCPDH
ENSG00000143669	8.271030151	8.608592853	protein_coding	1	235824341	236046940	LYST
ENSG00000143674	7.795160661	7.213370937	protein_coding	1	233463514	233520894	
ENSG00000143702	8.56983519	8.616616024	protein_coding	1	243287730	243418708	CEP170
ENSG00000143727	9.368588363	9.076603748	protein_coding	2	264140	278283	ACP1
ENSG00000143740	8.629769183	8.587754596	protein_coding	1	227916240	227968927	SNAP47
ENSG00000143742	9.810342685	10.19530463	protein_coding	1	225965531	225978168	SRP9
ENSG00000143748	8.020839917	8.559493622	protein_coding	1	224415036	224518089	NVL
ENSG00000143751	8.257454839	8.071789292	protein_coding	1	226170403	226187066	C1orf55
ENSG00000143753	9.549752678	9.403804382	protein_coding	1	224363458	224381143	DEGS1
ENSG00000143756	8.930339658	9.019827949	protein_coding	1	224301789	224349749	FBXO28
ENSG00000143761	12.33390546	12.48802068	protein_coding	1	228270361	228286912	ARF1
ENSG00000143771	8.325948295	8.885882287	protein_coding	1	224544552	224567161	CNIH4
ENSG00000143772	7.587643674	7.410839058	protein_coding	1	226819391	226927024	ITPKB
ENSG00000143774	10.48864213	11.0040448	protein_coding	1	228327663	228336685	GUK1
ENSG00000143776	9.30119149	9.445582628	protein_coding	1	227177566	227506175	CDC42BPA
ENSG00000143786	5.301468762	4.857374129	protein_coding	1	224622362	224928251	CNIH3
ENSG00000143793	7.680671792	7.86795666	protein_coding	1	228288427	228293112	C1orf35
ENSG00000143797	8.66103884	7.852510423	protein_coding	2	8992820	9143942	MBOAT2
ENSG00000143799	11.90024677	12.16067846	protein_coding	1	224102741	226595780	PARP1
ENSG00000143801	7.976547636	8.066778281	protein_coding	1	227057885	227083806	PSEN2
ENSG00000143811	8.632776617	8.865827498	protein_coding	1	226107578	226111978	PYCR2
ENSG00000143815	9.723436728	9.474880612	protein_coding	1	225589204	225616627	LBR
ENSG00000143816	8.746818039	5.855704078	protein_coding	1	228106357	228135631	WNT9A
ENSG00000143819	8.649206367	8.308116953	protein_coding	1	225997794	226033260	EPHX1
ENSG00000143839	0.499066092	0.697730409	protein_coding	1	204123944	204135465	REN
ENSG00000143842	8.625246244	7.8660349	protein_coding	1	204042243	204096863	SOX13
ENSG00000143845	5.107569429	3.714580548	protein_coding	1	204100190	204121307	ETNK2
ENSG00000143847	5.858855738	7.243290054	protein_coding	1	202995626	203047864	PPFIA4
ENSG00000143850	5.538004898	4.777836593	protein_coding	1	204190349	204346793	PLEKHA6
ENSG00000143851	0.869158192	1.16600992	protein_coding	1	202116141	202130716	PTPN7
ENSG00000143858	3.274897671	2.851901313	protein_coding	1	202559724	202679545	SYT2
ENSG00000143862	7.323008624	7.389591938	protein_coding	1	202102532	202113869	ARL8A
ENSG00000143867	3.077135474	1.802319292	protein_coding	2	19551246	19558414	OSR1
ENSG00000143869	0.499066092	0	protein_coding	2	20866424	20873418	GDF7
ENSG00000143870	11.17580182	10.91646202	protein_coding	2	10923517	10978103	PDIA6
ENSG00000143878	10.31182317	9.223427792	protein_coding	2	20646835	20649200	RHOB
ENSG00000143882	4.026095388	3.845183986	protein_coding	2	10861775	10925236	ATP6V1C2
ENSG00000143889	8.628263112	8.78167951	protein_coding	2	38789120	38830728	HNRPLL
ENSG00000143891	6.771940731	6.293387477	protein_coding	2	38893052	38968379	GALM
ENSG00000143919	5.879259127	6.136428444	protein_coding	2	44589089	44999731	CAMKMT

ENSG00000143924	9.616598542	9.569438796	protein_coding	2	42396490	42559688	EML4
ENSG00000143933	11.25649564	11.92684508	protein_coding	2	47272677	47403740	CALM2
ENSG00000143942	5.967663407	6.376734172	protein_coding	2	53994929	54002333	CHAC2
ENSG00000143947	10.01954299	10.62242012	protein_coding	2	55459039	55462989	RPS27A
ENSG00000143951	5.158541613	5.784235782	protein_coding	2	63348518	64054977	WDPCP
ENSG00000143952	7.952677993	7.799123587	protein_coding	2	64120035	64246567	VPS54
ENSG00000143970	9.233465948	9.369623887	protein_coding	2	25960557	26101385	ASXL2
ENSG00000143971	6.96088487	7.050313162	protein_coding	2	67624451	67637677	ETAA1
ENSG00000143977	8.15239992	9.029304007	protein_coding	2	70508494	70520903	SNRPG
ENSG00000143994	1.616589159	2.144285137	protein_coding	2	27346682	27353680	ABHD1
ENSG00000143995	5.672682881	5.593003837	protein_coding	2	66660584	66799890	MEIS1
ENSG00000144021	9.9233443	9.415020261	protein_coding	2	96931870	96939087	CIAO1
ENSG00000144026	7.434062125	6.995112131	protein_coding	2	95813075	95831158	ZNF514
ENSG00000144028	12.25266173	11.82318997	protein_coding	2	96940074	96971297	SNRNP200
ENSG00000144029	9.546566899	8.753889302	protein_coding	2	95752952	95815179	MRPS5
ENSG00000144031	1.799000381	1.925536307	protein_coding	2	71205510	71212626	ANKRD53
ENSG00000144034	7.0218028	7.534563804	protein_coding	2	73956231	73964527	TPRKB
ENSG00000144036	8.205883411	7.915187059	protein_coding	2	72403113	73053177	EXOC6B
ENSG00000144040	7.102117104	7.23139676	protein_coding	2	73169165	73302747	SFXN5
ENSG00000144043	9.713528165	9.675290063	protein_coding	2	71213068	71222075	TEX261
ENSG00000144045	1.616589159	3.232099092	protein_coding	2	74745258	74753463	DQX1
ENSG00000144048	7.621344476	7.598461017	protein_coding	2	73989311	74007284	DUSP11
ENSG00000144061	6.163378456	6.142791909	protein_coding	2	110879888	110962643	NPHP1
ENSG00000144063	7.827020104	3.081239798	protein_coding	2	110841447	110874143	MALL
ENSG00000144115	7.913645586	8.226588358	protein_coding	2	88469835	88486146	THNSL2
ENSG00000144118	9.546566899	9.00680492	protein_coding	2	120997640	121052285	RALB
ENSG00000144120	7.587643674	7.711939664	protein_coding	2	120436743	120444083	TMEM177
ENSG00000144130	1.799000381	0.697730409	protein_coding	2	113479063	113500456	NT5DC4
ENSG00000144134	5.763291843	5.840125386	protein_coding	2	114384806	114400973	RABL2A
ENSG00000144136	10.87386554	9.83707015	protein_coding	2	113403434	113421404	SLC20A1
ENSG00000144152	4.381167248	4.048652948	protein_coding	2	112895962	112945791	FBLN7
ENSG00000144161	6.846372398	6.803579823	protein_coding	2	112973254	113012713	ZC3H8
ENSG00000144182	5.270908378	4.826083323	protein_coding	2	99771418	99779620	LIPT1
ENSG00000144199	6.922076877	6.097646709	protein_coding	2	97747779	97760619	FAHD2B
ENSG00000144214	3.603959378	3.32456471	protein_coding	2	99900701	99921205	LYG1
ENSG00000144218	0.499066092	1.667587519	protein_coding	2	100163718	100759201	AFF3
ENSG00000144224	10.19917421	10.24679846	protein_coding	2	136499189	136542633	UBXN4
ENSG00000144227	0	0.390640832	protein_coding	2	139428342	139537918	NXPH2
ENSG00000144228	8.158673248	7.944622739	protein_coding	2	139259371	139331117	SPOPL
ENSG00000144230	0	1.16600992	protein_coding	2	128403439	128413754	GPR17
ENSG00000144231	8.614637248	8.171634445	protein_coding	2	128603896	128615731	POLR2D
ENSG00000144233	8.952173002	8.709567138	protein_coding	2	128619204	128643496	AMMECR1L
ENSG00000144283	10.02241241	9.580614711	protein_coding	2	159313476	159539391	PKP4
ENSG00000144306	6.998676229	6.977419495	protein_coding	2	175260458	175294303	SCRN3
ENSG00000144320	8.954578658	8.899098923	protein_coding	2	176788620	176867567	KIAA1715
ENSG00000144331	2.10647801	0.950786998	protein_coding	2	180306709	180726232	ZNF385B
ENSG00000144354	9.436423186	8.870627795	protein_coding	2	174219548	174233725	CDCA7
ENSG00000144355	2.762599152	3.368666104	protein_coding	2	172949468	172954405	DLX1
ENSG00000144357	9.12764907	8.910331892	protein_coding	2	170683968	170940641	UBR3
ENSG00000144362	4.568725997	4.710890357	protein_coding	2	170550975	170558218	PHOSPHO2
ENSG00000144366	7.136425764	7.664078611	protein_coding	2	189156396	189460653	GULP1
ENSG00000144369	5.036678094	4.961837817	protein_coding	2	187558698	187630685	FAM171B
ENSG00000144381	12.39750633	11.94402674	protein_coding	2	198351305	198381461	HSPD1
ENSG00000144395	6.236372259	6.775188634	protein_coding	2	197504278	197628214	CCDC150
ENSG00000144401	7.471499737	7.249200135	protein_coding	2	208445355	208490652	METTL21A
ENSG00000144406	0	0.390640832	protein_coding	2	210636717	210864024	UNC80
ENSG00000144410	0.499066092	1.518964905	protein_coding	2	207804278	207834198	CPO
ENSG00000144426	6.760987087	6.655683365	protein_coding	2	203879602	204091101	NBEAL1
ENSG00000144445	6.766474304	7.182818183	protein_coding	2	210885435	211036107	C2orf67
ENSG00000144451	6.421663777	6.142791909	protein_coding	2	214149113	215275225	SPAG16
ENSG00000144452	1.960915222	1.16600992	protein_coding	2	215796266	216003151	ABCA12
ENSG00000144455	8.229914881	8.401295742	protein_coding	3	3742498	4508965	SUMF1
ENSG00000144460	0.499066092	1.667587519	protein_coding	2	226265364	226518734	NYAP2

ENSG00000144468	8.625246244	8.061749803	protein_coding	2	227700297	227863931	RHBDD1
ENSG00000144476	2.10647801	0.390640832	protein_coding	2	237476430	237491001	CXCR7
ENSG00000144481	0.869158192	1.518964905	protein_coding	2	234826043	234928166	TRPM8
ENSG00000144485	5.575999561	4.65855978	protein_coding	2	239146908	239149303	HES6
ENSG00000144488	6.814942123	7.022976646	protein_coding	2	239008798	239041928	ESPNL
ENSG00000144504	7.440941622	7.219404604	protein_coding	2	241418839	241508626	ANKMY1
ENSG00000144524	9.05999771	8.669360359	protein_coding	2	232646381	232674093	COPS7B
ENSG00000144535	8.099033379	7.612284109	protein_coding	2	232825955	233209060	DIS3L2
ENSG00000144550	1.616589159	1.667587519	protein_coding	3	9745491	9771592	CPNE9
ENSG00000144554	9.558477469	9.110276346	protein_coding	3	10068098	10143614	FANCD2
ENSG00000144559	7.093410923	7.216390925	protein_coding	3	11831916	11888393	TAMM41
ENSG00000144560	7.925957035	7.903991977	protein_coding	3	11597544	11762220	VGLL4
ENSG00000144566	8.725846631	8.549948921	protein_coding	3	19988571	20026667	RAB5A
ENSG00000144567	9.966893004	9.814826612	protein_coding	2	220040947	220050201	FAM134A
ENSG00000144579	9.839270931	9.552809598	protein_coding	2	219262979	219270664	CTDSP1
ENSG00000144580	10.1097262	9.804327751	protein_coding	2	219433303	219461803	RQCD1
ENSG00000144583	8.032273249	6.117167886	protein_coding	2	217122588	217236750	4-Mar
ENSG00000144589	8.583888749	8.237034976	protein_coding	2	220462582	220481173	STK11IP
ENSG00000144591	10.09344535	10.3254768	protein_coding	2	220363589	220371710	GMPPA
ENSG00000144596	2.57521082	2.721932731	processed_transc	3	14530619	14583588	GRIP2
ENSG00000144597	8.143992822	7.648510984	protein_coding	3	15468862	15484120	EAF1
ENSG00000144619	0.499066092	0	protein_coding	3	2140497	3099645	CNTN4
ENSG00000144635	8.528449672	8.717049372	protein_coding	3	32567463	32612366	DYNC1LI1
ENSG00000144642	6.664362065	6.733635362	protein_coding	3	29322473	30051886	RBMS3
ENSG00000144645	7.913645586	6.762847836	protein_coding	3	31699382	32119072	OSBPL10
ENSG00000144647	8.109865782	8.419754632	protein_coding	3	43120724	43147568	C3orf39
ENSG00000144648	1.616589159	2.334191469	protein_coding	3	42846244	42949597	CCBP2
ENSG00000144649	2.471521042	3.64455972	protein_coding	3	43020759	43101703	FAM198A
ENSG00000144655	9.196341019	7.497803111	protein_coding	3	39183346	39196053	CSRNP1
ENSG00000144659	7.925957035	8.213044874	protein_coding	3	39424839	39438679	SLC25A38
ENSG00000144668	1.960915222	2.039052734	protein_coding	3	37493606	37865005	ITGA9
ENSG00000144671	2.238690726	2.242360793	protein_coding	3	38323785	38360066	SLC22A14
ENSG00000144674	9.735377778	9.787673606	protein_coding	3	37284668	37408242	GOLGA4
ENSG00000144677	8.720202385	7.793067905	protein_coding	3	37903451	38025960	CTDSPL
ENSG00000144681	7.91858278	7.551403417	protein_coding	3	36421836	36589499	STAC
ENSG00000144711	9.486324422	8.871585941	protein_coding	3	12938719	13114617	IQSEC1
ENSG00000144712	2.762599152	2.721932731	protein_coding	3	12837971	12913415	CAND2
ENSG00000144713	11.73725499	12.08690074	protein_coding	3	12875984	12883087	RPL32
ENSG00000144724	8.735671237	8.5772215	protein_coding	3	61547243	62283288	PTPRG
ENSG00000144730	7.568924112	7.182818183	protein_coding	3	57124010	57204334	IL17RD
ENSG00000144736	8.092494681	8.103127799	protein_coding	3	72798428	72911065	SHQ1
ENSG00000144741	7.507990374	7.591499486	protein_coding	3	66119285	66438530	SLC25A26
ENSG00000144744	8.585441838	8.880180737	protein_coding	3	69103881	69129559	UBA3
ENSG00000144746	9.466233411	9.662060384	protein_coding	3	69134095	69155217	ARL6IP5
ENSG00000144747	8.346244381	8.673761454	protein_coding	3	69068978	69101484	TMF1
ENSG00000144749	9.293610409	9.696532858	protein_coding	3	66429221	66551687	LRIG1
ENSG00000144791	8.708847212	8.673761454	protein_coding	3	45596886	45727830	LIMD1
ENSG00000144792	1.960915222	1.353254395	protein_coding	3	44619715	44641186	ZNF660
ENSG00000144802	9.631710027	9.692743008	protein_coding	3	101546835	101579866	NFKBIZ
ENSG00000144810	10.34749948	9.981193025	protein_coding	3	99357319	99518070	COL8A1
ENSG00000144815	7.035502862	6.815578497	protein_coding	3	101498046	101547073	FAM55C
ENSG00000144820	0	0.390640832	protein_coding	3	100328433	100414323	GPR128
ENSG00000144821	7.262161623	6.50081754	protein_coding	3	108099216	108248169	MYH15
ENSG00000144824	11.15921124	11.30039785	protein_coding	3	111451327	111695364	PHLDB2
ENSG00000144827	8.414239457	8.228085371	protein_coding	3	111697857	111712210	ABHD10
ENSG00000144834	0.499066092	0.390640832	protein_coding	3	111717511	111732734	TAGLN3
ENSG00000144837	0.499066092	0.390640832	protein_coding	3	119316689	119348658	PLA1A
ENSG00000144840	7.402691859	7.154756319	protein_coding	3	120405528	120461840	RABL3
ENSG00000144843	5.81716234	5.536128679	protein_coding	3	119298115	119308792	ADPRH
ENSG00000144847	0	0.390640832	protein_coding	3	118619404	118864915	IGSF11
ENSG00000144848	9.167597433	9.132828786	protein_coding	3	112251356	112280893	ATG3
ENSG00000144852	0.499066092	0.390640832	protein_coding	3	119499331	119537332	NR1I2
ENSG00000144857	0.499066092	0.697730409	protein_coding	3	112929850	113006303	BOC

ENSG00000144867	9.382055486	9.552809598	protein_coding	3	133502877	133544616	SRPRB
ENSG00000144893	1.16343121	4.406904905	protein_coding	3	150803484	151154860	MED12L
ENSG00000144895	10.08468652	9.853651825	protein_coding	3	150264465	150302029	EIF2A
ENSG00000144908	4.689181911	3.532712221	protein_coding	3	125822412	125916837	ALDH1L1
ENSG00000144909	8.000028772	8.130601896	protein_coding	3	125247702	125313934	OSBPL11
ENSG00000144935	6.84118134	7.373448694	protein_coding	3	142442916	142526730	TRPC1
ENSG00000144959	8.885655987	8.640421249	protein_coding	3	172348039	172429008	NCEH1
ENSG00000145002	1.616589159	1.518964905	protein_coding	8	12282913	12293915	FAM86B2
ENSG00000145012	9.467076146	9.311499072	protein_coding	3	187871072	188608460	LPP
ENSG00000145014	8.474152153	8.960725939	protein_coding	3	194308402	194354418	TMEM44
ENSG00000145016	9.036344956	8.34859317	protein_coding	3	197398264	197476598	KIAA0226
ENSG00000145020	6.005279909	6.45552765	protein_coding	3	49454211	49460186	AMT
ENSG00000145022	7.190493718	7.470237199	protein_coding	3	49449639	49453908	TCTA
ENSG00000145029	5.637181576	6.1803943	protein_coding	3	49460379	49466759	NICN1
ENSG00000145040	6.567100227	7.252146121	protein_coding	3	48599160	48601206	UCN2
ENSG00000145041	8.920528869	8.686884751	protein_coding	3	51433298	51534010	VPRBP
ENSG00000145050	8.720202385	8.897218229	protein_coding	3	51422478	51426828	MANF
ENSG00000145075	3.652182994	4.15321211	protein_coding	3	180320646	180588793	CCDC39
ENSG00000145087	1.799000381	1.353254395	protein_coding	3	120626919	121143608	STXBP5L
ENSG00000145088	3.274897671	4.29708397	protein_coding	3	121554030	121605373	EAF2
ENSG00000145107	4.292665995	4.448594745	protein_coding	3	196042953	196065374	TM4SF19
ENSG00000145113	8.610066544	2.334191469	protein_coding	3	195473636	195539148	MUC4
ENSG00000145147	3.871730003	5.187895122	protein_coding	4	20254883	20622184	SLIT2
ENSG00000145191	9.356814297	9.337390687	protein_coding	3	183852826	184402546	EIF2B5
ENSG00000145194	8.465744027	8.754928172	protein_coding	3	183967438	184010819	ECE2
ENSG00000145198	2.471521042	2.144285137	protein_coding	3	183948217	183960117	VWA5B2
ENSG00000145214	8.162840364	7.992972811	protein_coding	4	952675	980683	DGKQ
ENSG00000145216	8.635777795	8.398639372	protein_coding	4	54243810	55161439	FIP1L1
ENSG00000145217	4.981125677	4.761389014	protein_coding	4	972861	987228	SLC26A1
ENSG00000145220	8.771587845	8.584252102	protein_coding	4	4269428	4291896	LYAR
ENSG00000145241	6.687686437	7.122922611	protein_coding	4	68337521	68411324	CENPC1
ENSG00000145244	1.616589159	0.950786998	protein_coding	4	47596015	47840123	CORIN
ENSG00000145246	8.485841807	8.437980325	protein_coding	4	47487305	47595503	ATP10D
ENSG00000145247	8.561968095	8.801925129	protein_coding	4	48887036	48908954	OCIAD2
ENSG00000145248	0.869158192	0.390640832	protein_coding	4	48485360	48491213	SLC10A4
ENSG00000145283	0	0.390640832	protein_coding	4	87744621	87770416	SLC10A6
ENSG00000145284	3.211941663	6.117167886	protein_coding	4	83550692	83720010	SCD5
ENSG00000145287	5.360713447	3.532712221	protein_coding	4	84011201	84058228	PLAC8
ENSG00000145293	8.774313977	8.623457692	protein_coding	4	83351715	83382244	ENOPH1
ENSG00000145331	5.107569429	5.496926774	protein_coding	4	100467866	100485189	RG9MTD2
ENSG00000145332	7.639400234	7.475288518	protein_coding	4	88081255	88141760	KLHL8
ENSG00000145337	8.29020451	9.074940905	protein_coding	4	89442136	89444964	PIGY
ENSG00000145348	6.887241849	7.556178852	protein_coding	4	106965474	107242652	TBCK
ENSG00000145349	8.103376106	8.165397208	protein_coding	4	114372188	114683083	CAMK2D
ENSG00000145354	7.202688343	7.852510423	protein_coding	4	103790135	103810399	CISD2
ENSG00000145362	6.979904289	7.809160246	protein_coding	4	113739265	114309884	ANK2
ENSG00000145365	7.587643674	8.108014344	protein_coding	4	113196783	113207059	TIFA
ENSG00000145375	7.363400307	7.033288635	protein_coding	4	123844229	124235809	SPATA5
ENSG00000145384	0	0.390640832	protein_coding	4	120238405	120243545	FABP2
ENSG00000145386	9.293610409	9.463486936	protein_coding	4	122737599	122745087	CCNA2
ENSG00000145388	7.106450572	7.278392738	protein_coding	4	119606523	119632201	METTL14
ENSG00000145390	9.088754802	9.375714884	protein_coding	4	120133742	120216672	USP53
ENSG00000145391	10.0056898	10.21053023	protein_coding	4	140417243	140477928	SETD7
ENSG00000145414	7.277615649	7.204272857	protein_coding	4	164031225	164088073	NAF1
ENSG00000145416	4.322773689	5.163066621	protein_coding	4	164445450	165305202	1-Mar
ENSG00000145423	2.471521042	2.501982735	protein_coding	4	154701744	154710272	SFRP2
ENSG00000145425	8.974866377	9.89993707	protein_coding	4	152020725	152025804	RPS3A
ENSG00000145428	1.799000381	2.788380093	protein_coding	4	154631277	154681387	RNF175
ENSG00000145431	8.617676359	8.790825214	protein_coding	4	157681606	157892546	PDGFC
ENSG00000145439	7.813830732	8.283850589	protein_coding	4	169784921	169931426	CBR4
ENSG00000145451	0.869158192	0.950786998	protein_coding	4	175558065	175750465	GLRA3
ENSG00000145476	7.119373418	7.607691112	protein_coding	4	187112674	187134616	CYP4V2
ENSG00000145491	3.698846687	3.133298822	protein_coding	5	10441636	10472141	ROPN1L

ENSG00000145494	10.23523821	10.1891691	protein_coding	5	1801514	1816719	NDUFS6
ENSG00000145495	10.86909148	10.27277906	protein_coding	5	10353815	10435491	6-Mar
ENSG00000145506	6.005279909	1.802319292	protein_coding	5	1008944	1039058	NKD2
ENSG00000145526	1.16343121	0.390640832	protein_coding	5	19473060	20575982	CDH18
ENSG00000145536	6.738827102	7.258020107	protein_coding	5	5140443	5320417	ADAMTS16
ENSG00000145545	8.66103884	8.501254594	protein_coding	5	6633456	6669675	SRD5A1
ENSG00000145555	11.64140001	10.3997533	protein_coding	5	16665395	16936372	MYO10
ENSG00000145569	4.942865586	4.29708397	protein_coding	5	14581884	14615073	FAM105A
ENSG00000145592	12.92204367	13.31835675	protein_coding	5	40825364	40835437	RPL37
ENSG00000145604	9.839921696	9.393168008	protein_coding	5	36152091	36184421	SKP2
ENSG00000145623	11.91437282	11.79695211	protein_coding	5	38845960	38945698	OSMR
ENSG00000145632	10.65946972	10.03307035	protein_coding	5	57749809	57756087	PLK2
ENSG00000145675	7.106450572	6.850987146	protein_coding	5	67511548	67597649	PIK3R1
ENSG00000145685	9.388297379	9.292307546	protein_coding	5	77781040	78065844	LHFPL2
ENSG00000145687	6.196276899	6.440109466	protein_coding	5	80708840	81047616	SSBP2
ENSG00000145700	2.928422289	3.027231696	protein_coding	5	74364100	74532703	ANKRD31
ENSG00000145703	1.16343121	0.950786998	protein_coding	5	75699074	76003957	IQGAP2
ENSG00000145715	9.192269688	8.754928172	protein_coding	5	86563705	86688145	RASA1
ENSG00000145723	5.637181576	5.545764946	protein_coding	5	102421704	102455855	GIN1
ENSG00000145725	8.605481314	8.65607594	protein_coding	5	102455853	102538937	PPIP5K2
ENSG00000145730	10.52187106	11.06917952	protein_coding	5	102089685	102366809	PAM
ENSG00000145734	8.783815073	8.5772215	protein_coding	5	70751442	70863649	BDP1
ENSG00000145736	3.871730003	4.226940739	protein_coding	5	70330784	70363516	GTF2H2
ENSG00000145740	8.629769183	8.537928553	protein_coding	5	68389473	68426896	SLC30A5
ENSG00000145741	11.47185804	11.68040503	protein_coding	5	72794233	72801460	BTF3
ENSG00000145743	6.502803968	6.815578497	protein_coding	5	107194736	107717799	FBXL17
ENSG00000145757	0.499066092	0.390640832	protein_coding	5	94987885	95034415	SPATA9
ENSG00000145777	1.16343121	0	protein_coding	5	110405760	110413722	TSLP
ENSG00000145779	8.011627561	8.104758487	protein_coding	5	118604387	118730294	TNFAIP8
ENSG00000145780	8.857651982	8.269383236	protein_coding	5	114856608	114880591	FEM1C
ENSG00000145781	6.887241849	6.304782526	protein_coding	5	115420688	115748459	COMMD10
ENSG00000145782	9.769936332	8.588920207	protein_coding	5	115163893	115177555	ATG12
ENSG00000145794	1.16343121	1.667587519	protein_coding	5	126626523	126801429	MEGF10
ENSG00000145817	8.876804446	8.697730375	protein_coding	5	143537723	143550278	YIPF5
ENSG00000145819	8.531675665	7.502758994	protein_coding	5	142149949	142608576	ARHGAP26
ENSG00000145824	0.499066092	0.697730409	protein_coding	5	134906373	134914969	CXCL14
ENSG00000145833	10.33785675	10.4503311	protein_coding	5	134094469	134190823	DDX46
ENSG00000145860	9.868906425	10.74407487	protein_coding	5	158584417	158637061	RNF145
ENSG00000145861	0	0.950786998	protein_coding	5	159774758	159797648	C1QTNF2
ENSG00000145868	7.842689815	7.832967122	protein_coding	5	147763498	147822399	FBXO38
ENSG00000145882	7.474855471	7.722599249	protein_coding	5	148737570	148749216	PCYOX1L
ENSG00000145888	0.499066092	0.390640832	protein_coding	5	151202074	151304403	GLRA1
ENSG00000145901	11.93309658	11.97210415	protein_coding	5	150409506	150473138	TNIP1
ENSG00000145907	11.4781421	11.31226726	protein_coding	5	151150606	151192346	G3BP1
ENSG00000145908	7.058051099	7.357122767	protein_coding	5	150273954	150284545	ZNF300
ENSG00000145911	5.91922012	5.855704078	protein_coding	5	177540444	177553088	N4BP3
ENSG00000145912	8.596266901	9.05820621	protein_coding	5	177576461	177580968	NHP2
ENSG00000145916	7.740446424	7.93548844	protein_coding	5	177557997	177577566	RMND5B
ENSG00000145919	9.135091581	9.339469783	protein_coding	5	173034517	173043663	BOD1
ENSG00000145920	0	1.518964905	protein_coding	5	175223313	175311023	CPLX2
ENSG00000145934	4.75690578	4.42790041	protein_coding	5	166711804	167691162	ODZ2
ENSG00000145945	7.938164312	8.242970684	protein_coding	6	3849620	3851551	FAM50B
ENSG00000145949	3.698846687	4.710890357	protein_coding	6	2663863	2751200	MYLK4
ENSG00000145965	0.869158192	2.420525079	protein_coding	6	3982909	3984372	
ENSG00000145975	1.407729925	1.925536307	protein_coding	6	4049668	4087578	C6orf146
ENSG00000145979	7.049074049	7.551403417	protein_coding	6	13266774	13328776	TBC1D7
ENSG00000145982	7.916116295	8.034631177	protein_coding	6	5261584	5771816	FARS2
ENSG00000145990	6.825495146	6.440109466	protein_coding	6	13358062	13487787	GFOD1
ENSG00000145996	7.765324926	7.623703032	protein_coding	6	20534688	21232635	CDKAL1
ENSG00000146001	2.928422289	2.144285137	pseudogene	5	140613938	140617101	PCDHB18
ENSG00000146005	2.762599152	2.501982735	protein_coding	5	139175406	139224051	PSD2
ENSG00000146006	0.869158192	0.390640832	protein_coding	5	138208179	138211057	LRRTM2
ENSG00000146007	9.183066967	9.097229165	protein_coding	5	140079747	140086266	ZMAT2

ENSG00000146013	1.407729925	1.16600992	protein_coding	5	137588068	137610360	GFRA3
ENSG00000146021	4.712111592	3.608232228	protein_coding	5	136953189	137071779	KLHL3
ENSG00000146054	5.649112675	5.871116342	protein_coding	5	180620924	180632293	TRIM7
ENSG00000146063	8.787867896	8.890616425	protein_coding	5	180649499	180662809	TRIM41
ENSG00000146066	7.792473675	8.253299769	protein_coding	5	175815748	175816772	HIGD2A
ENSG00000146067	8.401952457	8.732953241	protein_coding	5	176946789	176981542	FAM193B
ENSG00000146072	10.44567929	8.911264037	protein_coding	6	47199268	47277641	TNFRSF21
ENSG00000146083	9.74513791	9.537194665	protein_coding	5	175953698	175965026	RNF44
ENSG00000146085	7.556308086	7.933654619	protein_coding	6	49398073	49430904	MUT
ENSG00000146090	5.4452544	5.988823159	protein_coding	5	179527795	179636153	RASGEF1C
ENSG00000146094	4.618121645	5.901455548	protein_coding	5	176928908	176938275	DOK3
ENSG00000146109	8.3053626	8.605140645	protein_coding	6	26597180	26600278	ABT1
ENSG00000146112	10.28196264	10.54195238	protein_coding	6	30644166	30655672	PPP1R18
ENSG00000146143	7.985986001	7.805153958	processed_transc	6	57179603	57513375	PRIM2
ENSG00000146147	0	0.390640832	protein_coding	6	53794780	54131078	MLIP
ENSG00000146192	0.869158192	0.390640832	protein_coding	6	36973422	36996846	FGD2
ENSG00000146197	3.274897671	4.048652948	protein_coding	6	35182190	35220856	SCUBE3
ENSG00000146205	5.858855738	5.43605125	protein_coding	2	242127924	242164792	ANO7
ENSG00000146215	0.499066092	1.667587519	protein_coding	6	43267448	43276535	CRIP3
ENSG00000146216	1.16343121	2.242360793	protein_coding	6	43211222	43255997	TTBK1
ENSG00000146221	0.869158192	0.390640832	protein_coding	6	44246480	44265458	TCTE1
ENSG00000146223	10.89687643	11.06415941	protein_coding	6	42847356	42857663	RPL7L1
ENSG00000146232	7.993024472	8.256237404	protein_coding	6	44225919	44233500	NFKBIE
ENSG00000146233	3.335220907	4.202780776	protein_coding	6	46517541	46620523	CYP39A1
ENSG00000146242	9.824221725	10.21015154	protein_coding	6	83072923	83080545	TPBG
ENSG00000146243	3.652182994	3.453019579	protein_coding	6	79577189	79608320	IRAK1BP1
ENSG00000146247	7.795160661	8.210017866	protein_coding	6	79650263	79787953	PHIP
ENSG00000146250	0	0.950786998	protein_coding	6	84222194	84235423	PRSS35
ENSG00000146263	8.680062001	8.501254594	protein_coding	6	97590037	97731093	MMS22L
ENSG00000146267	2.671945279	2.788380093	protein_coding	6	99728536	99797938	C6orf168
ENSG00000146276	0	0.390640832	protein_coding	6	89887220	89940997	GABRR1
ENSG00000146278	9.157191483	9.300868647	protein_coding	6	89790470	89794879	PNRC1
ENSG00000146281	8.913126744	8.925174415	protein_coding	6	89855769	89875284	PM20D2
ENSG00000146282	8.177331204	8.467431533	protein_coding	6	88224096	88299735	RARS2
ENSG00000146285	0.869158192	0	protein_coding	6	108025308	108145521	SCML4
ENSG00000146350	5.613019223	5.953060722	protein_coding	6	121400640	121655891	C6orf170
ENSG00000146374	0	0.390640832	protein_coding	6	127439749	127518910	RSPO3
ENSG00000146376	8.000028772	7.896480033	protein_coding	6	129897277	130031370	ARHGAP18
ENSG00000146386	8.12275821	8.15284125	protein_coding	6	139349819	139364439	C6orf115
ENSG00000146399	0.869158192	0.950786998	protein_coding	6	132966123	132967142	TAAR1
ENSG00000146409	7.737655476	7.902117654	protein_coding	6	133090507	133119747	C6orf192
ENSG00000146410	7.071412799	7.053694091	protein_coding	6	136552162	136571473	FAM54A
ENSG00000146411	5.054730169	5.125003832	protein_coding	6	134309835	134373774	SLC2A12
ENSG00000146414	7.709444959	7.93548844	protein_coding	6	146185381	146285559	SHPRH
ENSG00000146416	7.945439403	7.281279771	protein_coding	6	143381633	143661441	AIG1
ENSG00000146425	8.855079001	9.138412238	protein_coding	6	159057506	159065771	DYNLT1
ENSG00000146426	6.407685389	6.587342979	protein_coding	6	155153831	155578857	TIAM2
ENSG00000146433	9.044272373	9.11432951	protein_coding	6	158957466	159056460	TMEM181
ENSG00000146453	1.16343121	2.039052734	protein_coding	6	160221301	160241736	PNLDC1
ENSG00000146457	9.886651823	9.945238564	protein_coding	6	160146617	160177351	WTAP
ENSG00000146463	8.941297632	9.145559356	protein_coding	1	35734568	35887659	ZMYM4
ENSG00000146476	7.388527525	7.467704891	protein_coding	6	151773422	151791236	C6orf211
ENSG00000146477	5.649112675	5.271576845	protein_coding	6	160769300	160876014	SLC22A3
ENSG00000146521	2.238690726	1.353254395	protein_coding	6	168185217	168197539	C6orf123
ENSG00000146530	0.869158192	0	protein_coding	7	12370511	12443567	VWDE
ENSG00000146535	11.36988247	10.69485148	protein_coding	7	2767746	2883958	GNA12
ENSG00000146540	9.899819083	9.740202333	protein_coding	7	1036623	1177896	C7orf50
ENSG00000146555	4.712111592	6.037446454	protein_coding	7	3341080	4308632	SDK1
ENSG00000146556	5.158541613	4.917990497	pseudogene	2	114341664	114356655	WASH2P
ENSG00000146574	5.795854912	5.66550919	protein_coding	7	6836440	6866401	CCZ1B
ENSG00000146576	8.33889698	8.041458772	protein_coding	7	6629648	6648357	C7orf26
ENSG00000146587	8.577659618	8.162268448	protein_coding	7	5023349	5112854	RBAK
ENSG00000146592	9.079967361	8.93712253	protein_coding	7	28338940	28865511	CREB5

ENSG00000146648	11.50074649	10.79874338	protein_coding	7	55086714	55324313	EGFR
ENSG00000146666	2.10647801	0.950786998	antisense	7	47801074	47806370	LINC00525
ENSG00000146670	9.906668554	10.18493575	protein_coding	11	64833772	64851636	CDCA5
ENSG00000146674	6.528867325	6.795524906	protein_coding	7	45951850	45961473	IGFBP3
ENSG00000146676	10.38588427	10.21770649	protein_coding	7	44915896	44924960	PURB
ENSG00000146677	3.78787645	4.202780776	pseudogene	7	44507441	44507939	
ENSG00000146700	3.78787645	3.845183986	protein_coding	7	76018651	76039012	SRCRB4D
ENSG00000146701	12.0587492	11.76495242	protein_coding	7	75677369	75696826	MDH2
ENSG00000146707	5.239686603	5.175534283	protein_coding	7	76239303	76256578	POMZP3
ENSG00000146722	3.989024711	2.971122874	pseudogene	7	75021229	75024708	
ENSG00000146729	8.75650134	8.542748725	protein_coding	7	56019486	56067874	GBAS
ENSG00000146731	11.81725615	11.69495762	protein_coding	7	56119323	56131682	CCT6A
ENSG00000146733	9.094219895	8.927940468	protein_coding	7	56078744	56119297	PSPH
ENSG00000146755	1.407729925	0	protein_coding	7	72726535	72742085	TRIM50
ENSG00000146757	7.356139979	6.45552765	protein_coding	7	64838712	64866038	ZNF92
ENSG00000146776	6.275383355	5.824376633	protein_coding	7	105245514	105517050	ATXN7L1
ENSG00000146802	7.35977471	7.304170377	protein_coding	7	112405787	112430647	TMEM168
ENSG00000146826	7.648344073	7.381542895	protein_coding	7	99752043	99756338	C7orf43
ENSG00000146828	8.855079001	7.743685008	protein_coding	7	100424442	100464631	SLC12A9
ENSG00000146830	10.62363768	10.30930298	protein_coding	7	100277130	100287071	GIGYF1
ENSG00000146833	8.742648075	8.38394126	protein_coding	7	99474581	99517223	TRIM4
ENSG00000146834	10.1172615	10.12954213	protein_coding	7	100026413	100031741	MEPCE
ENSG00000146839	0.869158192	0	polymorphic_pse	7	100331249	100395419	ZAN
ENSG00000146842	8.620709081	8.269383236	protein_coding	7	129804555	129847610	TMEM209
ENSG00000146856	3.146112541	3.845183986	protein_coding	7	134671259	134832715	AGBL3
ENSG00000146858	6.548110424	6.16171561	protein_coding	7	138707126	138720775	ZC3HAV1L
ENSG00000146859	5.938793061	5.583678991	protein_coding	7	134832824	134850650	TMEM140
ENSG00000146872	7.821758815	7.973469823	protein_coding	17	60556386	60692839	TLK2
ENSG00000146904	5.498982484	3.368666104	protein_coding	7	143087382	143105985	EPHA1
ENSG00000146909	9.124447632	8.916844293	protein_coding	7	156742417	156765876	NOM1
ENSG00000146918	9.877172512	9.507299068	protein_coding	7	158424003	158497520	NCAPG2
ENSG00000146950	4.381167248	4.640685763	protein_coding	X	9754496	9917483	SHROOM2
ENSG00000146955	1.616589159	0	protein_coding	7	140103843	140126050	RAB19
ENSG00000146963	10.09453648	9.88340693	protein_coding	7	139025105	139108198	LUC7L2
ENSG00000146966	7.549958459	7.522414019	protein_coding	7	140218220	140373793	DENND2A
ENSG00000147003	0.869158192	0.950786998	protein_coding	X	15645441	15683154	TMEM27
ENSG00000147010	10.65946972	10.76046128	protein_coding	X	19552083	19905719	SH3KBP1
ENSG00000147027	6.716321419	8.519708104	protein_coding	X	34645181	34675405	TMEM47
ENSG00000147036	0	0.390640832	protein_coding	X	37430822	37543716	LANCL3
ENSG00000147041	0.869158192	0.950786998	protein_coding	X	37865835	37988072	SYTL5
ENSG00000147044	9.274481564	9.007676788	protein_coding	X	41374187	41782716	CASK
ENSG00000147050	8.577659618	8.826839049	protein_coding	X	44732423	44971847	KDM6A
ENSG00000147059	1.407729925	2.501982735	protein_coding	X	57160963	57164058	SPIN2A
ENSG00000147065	13.63376177	13.78025525	protein_coding	X	64887537	64961791	MSN
ENSG00000147082	3.554067925	4.17820932	protein_coding	X	49967364	50094909	CCNB3
ENSG00000147099	7.878603882	7.883239271	protein_coding	X	71549366	71792953	HDAC8
ENSG00000147100	9.324628822	9.522019224	protein_coding	X	73640638	73753752	SLC16A2
ENSG00000147117	1.407729925	1.16600992	protein_coding	X	47229982	47273704	ZNF157
ENSG00000147118	6.882196036	7.429177016	protein_coding	X	47834250	47863377	ZNF182
ENSG00000147119	6.462804234	6.686500471	protein_coding	X	46433219	46457843	CHST7
ENSG00000147121	6.676071387	7.429177016	protein_coding	X	46306292	46356857	ZNF673
ENSG00000147123	9.369490093	10.24753675	protein_coding	X	47001615	47004903	NDUFB11
ENSG00000147124	7.165790811	7.69255166	protein_coding	X	47305278	47342345	ZNF41
ENSG00000147127	2.238690726	2.039052734	protein_coding	X	69501943	69504852	RAB41
ENSG00000147130	9.824879313	9.937938881	protein_coding	X	70459474	70474996	ZMYM3
ENSG00000147133	9.011142793	9.106211763	protein_coding	X	70586114	70752224	TAF1
ENSG00000147140	12.07127337	12.18390949	protein_coding	X	70503042	70521018	NONO
ENSG00000147144	6.320845526	4.075511708	protein_coding	X	48916514	48927509	CCDC120
ENSG00000147145	0.499066092	0.390640832	protein_coding	X	78003206	78012591	LPAR4
ENSG00000147155	9.991116523	9.228672011	protein_coding	X	48379546	48387104	EBP
ENSG00000147160	1.960915222	2.039052734	protein_coding	X	69260392	69269788	AWAT2
ENSG00000147162	11.7230384	12.04579081	protein_coding	X	70752933	70795747	OGT
ENSG00000147164	9.40334477	9.336696989	protein_coding	X	70279094	70288273	SNX12

ENSG00000147166	3.211941663	3.781359661	protein_coding	X	70521584	70525221	ITGB1BP2
ENSG00000147168	0.499066092	0	protein_coding	X	70327254	70331958	IL2RG
ENSG00000147174	4.75690578	6.002883551	protein_coding	X	70798261	70833433	ACRC
ENSG00000147202	7.903720218	7.894595919	protein_coding	X	95939662	96859996	DIAPH2
ENSG00000147206	1.16343121	2.912743273	protein_coding	X	102330738	102348157	NXF3
ENSG00000147223	0	0.697730409	protein_coding	X	106143293	106146565	RIPPLY1
ENSG00000147224	10.11779823	10.03862623	protein_coding	X	106871737	106894256	PRPS1
ENSG00000147231	3.146112541	6.1803943	protein_coding	X	105855160	105922672	CXorf57
ENSG00000147234	2.762599152	1.667587519	protein_coding	X	106765680	106848481	FRMPD3
ENSG00000147251	5.81716234	7.161039629	protein_coding	X	117629861	117820126	DOCK11
ENSG00000147255	5.301468762	5.372493443	protein_coding	X	130407480	130533677	IGSF1
ENSG00000147257	1.16343121	0.697730409	protein_coding	X	132669773	133119922	GPC3
ENSG00000147274	10.55514466	10.39576264	protein_coding	X	135930163	135962923	RBMX
ENSG00000147316	7.30049066	7.167295693	protein_coding	8	6264113	6501144	MCPH1
ENSG00000147324	8.045874708	7.694718794	protein_coding	8	8640864	8751155	MFHAS1
ENSG00000147364	7.035502862	7.607691112	protein_coding	8	356428	421225	FBXO25
ENSG00000147382	7.80585884	7.860254213	protein_coding	X	152853377	152864707	FAM58A
ENSG00000147383	8.972494341	8.628000846	protein_coding	X	151999511	152038273	NSDHL
ENSG00000147394	7.210761201	5.294620694	protein_coding	X	152082986	152142025	ZNF185
ENSG00000147400	8.810621286	9.167571071	protein_coding	X	151995517	151999321	CETN2
ENSG00000147403	12.26773372	12.51831426	protein_coding	X	153618315	153637504	RPL10
ENSG00000147408	7.017207048	6.210997888	protein_coding	8	19261672	19615540	CSGALNACT1
ENSG00000147416	9.519199923	10.00409192	protein_coding	8	20054878	20084330	ATP6V1B2
ENSG00000147419	8.576098123	8.695567763	protein_coding	8	27590835	27630170	CCDC25
ENSG00000147421	6.414691513	6.877938671	protein_coding	8	28747911	28922281	HMBOX1
ENSG00000147437	3.393122761	3.608232228	protein_coding	8	25276776	25282170	GNRH1
ENSG00000147439	7.234712227	7.281279771	protein_coding	8	22477931	22526661	BIN3
ENSG00000147443	0	0.390640832	protein_coding	8	21766384	21771371	DOK2
ENSG00000147454	10.85402962	11.37019227	protein_coding	8	23386318	23432976	SLC25A37
ENSG00000147457	9.145657575	9.231660172	protein_coding	8	23101150	23119512	CHMP7
ENSG00000147459	9.862515511	9.534777296	protein_coding	8	25042238	25275598	DOCK5
ENSG00000147465	1.16343121	1.16600992	protein_coding	8	38001167	38008783	STAR
ENSG00000147471	8.020839917	7.862183683	protein_coding	8	37620111	37637283	PROSC
ENSG00000147475	9.093128531	9.025004422	protein_coding	8	37594117	37616619	ERLIN2
ENSG00000147481	0	0.390640832	protein_coding	8	50822349	51706678	SNTG1
ENSG00000147485	0	0.697730409	protein_coding	8	52232138	52722005	PXDNL
ENSG00000147509	6.496213887	6.365895578	protein_coding	8	54764368	54871863	RGS20
ENSG00000147526	8.826469148	9.071609459	protein_coding	8	38585704	38710546	TACC1
ENSG00000147533	8.237837231	8.824861692	protein_coding	8	41347915	41368499	GOLGA7
ENSG00000147535	7.258272112	7.679480043	protein_coding	8	38120648	38126761	PPAPDC1B
ENSG00000147536	8.032273249	8.40394723	protein_coding	8	41386725	41402565	GINS4
ENSG00000147548	8.771587845	8.769394346	protein_coding	8	38132544	38239790	WHSC1L1
ENSG00000147570	0.499066092	0	protein_coding	8	66933795	67012751	DNAJC5B
ENSG00000147571	0.499066092	0	protein_coding	8	67088620	67090960	CRH
ENSG00000147573	6.014532757	5.647721597	protein_coding	8	67039131	67087720	TRIM55
ENSG00000147576	3.004694206	3.368666104	protein_coding	8	67342420	67383836	ADHFE1
ENSG00000147586	7.206730419	7.103479673	protein_coding	8	80830952	80942524	MRPS28
ENSG00000147592	6.861834435	6.900645892	protein_coding	8	71547553	71581409	LACTB2
ENSG00000147601	7.23866569	7.116470702	protein_coding	8	73921099	73960357	TERF1
ENSG00000147604	7.44778847	7.57041104	protein_coding	8	74202506	74208024	RPL7
ENSG00000147606	1.799000381	2.039052734	protein_coding	8	92221722	92410378	SLC26A7
ENSG00000147614	1.407729925	1.518964905	protein_coding	8	86999552	87166457	ATP6V0D2
ENSG00000147642	5.090170488	2.851901313	protein_coding	8	110586207	110704020	SYBU
ENSG00000147647	0.499066092	1.353254395	protein_coding	8	105391652	105479281	DPYS
ENSG00000147649	10.79920289	10.41067104	protein_coding	8	98656407	98740998	MTDH
ENSG00000147650	8.325948295	8.542748725	protein_coding	8	105501459	105601417	LRP12
ENSG00000147654	6.469548347	6.703819132	protein_coding	8	110551940	110578225	EBAG9
ENSG00000147655	0	0.390640832	protein_coding	8	108911544	109095913	RSPO2
ENSG00000147669	7.60612345	8.022604578	protein_coding	8	101162812	101166230	POLR2K
ENSG00000147676	8.965354743	3.081239798	processed_transc	8	120177273	120257913	MAL2
ENSG00000147677	10.65355917	10.5464592	protein_coding	8	117654369	117779164	EIF3H
ENSG00000147679	7.110771062	6.746227492	protein_coding	8	117778742	117861702	UTP23
ENSG00000147684	9.353172091	9.636359306	protein_coding	8	125551344	125580751	NDUFB9

ENSG00000147687	7.23866569	7.167295693	protein_coding	8	125500726	125551699	TATDN1
ENSG00000147689	10.80488028	9.423538659	protein_coding	8	124191200	124222314	FAM83A
ENSG00000147697	2.238690726	1.518964905	protein_coding	8	130760442	130799134	GSDMC
ENSG00000147789	7.07583934	7.37615181	protein_coding	8	146052849	146072894	ZNF7
ENSG00000147799	7.935731106	7.63953919	protein_coding	8	145754563	145911194	ARHGAP39
ENSG00000147804	8.593182305	7.745776733	protein_coding	8	145635126	145642279	SLC39A4
ENSG00000147813	7.37781226	7.346135265	protein_coding	8	144656955	144660819	NAPRT1
ENSG00000147852	4.409500985	3.027231696	protein_coding	9	2621834	2654480	VLDLR
ENSG00000147853	7.409722215	7.56568256	protein_coding	9	4711155	4742043	AK3
ENSG00000147854	7.811178326	8.051639961	protein_coding	9	6413151	6507054	UHRF2
ENSG00000147862	8.364450825	8.170077662	protein_coding	9	14081842	14398982	NFIB
ENSG00000147869	0.499066092	1.518964905	protein_coding	9	14719722	14722715	CER1
ENSG00000147872	9.374888667	9.842944292	protein_coding	9	19108373	19149288	PLIN2
ENSG00000147874	8.02999382	7.989446368	protein_coding	9	19053141	19103117	HAUS6
ENSG00000147883	8.53810609	7.756190105	protein_coding	9	22002902	22009280	CDKN2B
ENSG00000147889	7.827020104	7.93548844	protein_coding	9	21967751	21995300	CDKN2A
ENSG00000147894	5.239686603	5.236305879	protein_coding	9	27546544	27573864	C9orf72
ENSG00000147905	8.116326397	8.219079902	protein_coding	9	37120536	37358146	ZCCHC7
ENSG00000147912	5.054730169	5.35066967	protein_coding	9	37510889	37588871	FBXO10
ENSG00000147955	10.59578202	10.35386622	protein_coding	9	34634719	34637806	SIGMAR1
ENSG00000147996	5.175139844	5.393992	protein_coding	9	70432004	70497240	CBWD5
ENSG00000148019	8.944931872	9.42875586	protein_coding	9	80850978	80894606	CEP78
ENSG00000148053	0.499066092	2.039052734	protein_coding	9	87283466	87638505	NTRK2
ENSG00000148057	4.689181911	5.35066967	protein_coding	9	86237964	86259045	C9orf103
ENSG00000148082	4.642199401	3.368666104	protein_coding	9	91628060	91793682	SHC3
ENSG00000148090	6.050961887	6.408769788	protein_coding	9	93976097	94124195	AUH
ENSG00000148110	9.849648119	9.782588703	protein_coding	9	97136833	97223324	HIATL1
ENSG00000148120	8.366258896	8.103127799	protein_coding	9	97488983	97849441	C9orf3
ENSG00000148143	9.743747641	9.84538481	protein_coding	9	109625378	109775915	ZNF462
ENSG00000148153	6.984620223	6.862599506	protein_coding	9	115448786	115480516	C9orf80
ENSG00000148154	11.18351346	11.26492165	protein_coding	9	114659046	114697649	UGCG
ENSG00000148156	0	0.697730409	protein_coding	9	111616871	111619239	ACTL7B
ENSG00000148158	9.122309386	8.705274089	protein_coding	9	115513118	115637267	SNX30
ENSG00000148175	10.38454676	9.76722557	protein_coding	9	124101355	124132531	STOM
ENSG00000148180	8.876804446	8.048254208	protein_coding	9	123970075	124095121	GSN
ENSG00000148187	8.680062001	8.680338024	protein_coding	9	125026882	125085743	MRRF
ENSG00000148200	7.165790811	5.446377228	protein_coding	9	127279888	127533589	NR6A1
ENSG00000148204	2.57521082	2.420525079	protein_coding	9	126118449	126142603	CRB2
ENSG00000148215	0.499066092	0	protein_coding	9	125551150	125552237	OR5C1
ENSG00000148218	8.650690744	8.507432028	protein_coding	9	116148597	116163613	ALAD
ENSG00000148219	5.512107582	5.84793576	protein_coding	9	119187504	120177348	ASTN2
ENSG00000148225	3.603959378	3.845183986	protein_coding	9	116077951	116102578	WDR31
ENSG00000148229	10.20170799	10.45704918	protein_coding	9	116169515	116172952	POLE3
ENSG00000148248	12.17716469	12.14659772	protein_coding	9	136228325	136242970	SURF4
ENSG00000148288	2.762599152	0.697730409	protein_coding	9	136028340	136039332	GBGT1
ENSG00000148290	7.8607586	8.029489191	protein_coding	9	136218610	136223552	SURF1
ENSG00000148291	7.430610036	7.228408053	protein_coding	9	136223428	136228045	SURF2
ENSG00000148296	9.409495425	9.547423533	protein_coding	9	136197552	136203235	SURF6
ENSG00000148297	9.260938843	9.456479577	protein_coding	9	136205160	136214986	MED22
ENSG00000148300	9.014605485	8.884933592	protein_coding	9	136271186	136283164	REXO4
ENSG00000148303	11.92499232	12.23905629	protein_coding	9	136215069	136218281	RPL7A
ENSG00000148308	10.25000281	10.19759874	protein_coding	9	135906076	135933888	GTF3C5
ENSG00000148331	8.596266901	8.254769334	protein_coding	9	132399171	132404444	ASB6
ENSG00000148334	10.82210899	10.72670616	protein_coding	9	130882972	130890741	PTGES2
ENSG00000148335	9.571074621	9.477400367	protein_coding	9	132371163	132398209	METTL11A
ENSG00000148337	10.49567985	10.54705904	protein_coding	9	130928343	130966662	CIZ1
ENSG00000148339	8.415986232	7.992972811	protein_coding	9	130830480	130871524	SLC25A25
ENSG00000148341	10.30052049	10.6377072	protein_coding	9	131769315	131790582	SH3GLB2
ENSG00000148343	8.684416567	8.899098923	protein_coding	9	131798900	131834361	FAM73B
ENSG00000148344	8.006999229	6.149127429	protein_coding	9	132500610	132515326	PTGES
ENSG00000148346	10.5335842	9.424844729	protein_coding	9	130911350	130915734	LCN2
ENSG00000148356	9.27062517	9.22117442	protein_coding	9	130213765	130265780	LRSAM1
ENSG00000148357	1.616589159	1.667587519	protein_coding	9	133028269	133309510	HMCN2

ENSG00000148358	10.69263307	10.46023731	protein_coding	9	132815705	132902448	GPR107
ENSG00000148362	9.036344956	9.141593107	protein_coding	9	139886870	139888436	C9orf142
ENSG00000148377	1.16343121	0.697730409	protein_coding	10	1064847	1071799	IDI2
ENSG00000148384	7.821758815	7.850567963	protein_coding	9	139323071	139334274	INPP5E
ENSG00000148396	11.18812073	10.9243327	protein_coding	9	139334549	139372141	SEC16A
ENSG00000148399	8.576098123	8.791837833	protein_coding	9	140449356	140473387	WDR85
ENSG00000148400	10.38677526	9.85994185	protein_coding	9	139388896	139440314	NOTCH1
ENSG00000148408	5.827699195	5.908941696	protein_coding	9	140772241	141019076	CACNA1B
ENSG00000148411	9.817629308	9.550418273	protein_coding	9	138898383	138987131	NACC2
ENSG00000148426	9.541775001	8.253299769	protein_coding	10	11865338	11914276	C10orf47
ENSG00000148429	8.237837231	7.940975956	protein_coding	10	11502509	11653753	USP6NL
ENSG00000148444	6.798966424	7.439552137	protein_coding	10	22604903	22609235	COMMD3
ENSG00000148450	7.127924785	7.309836726	protein_coding	10	23384435	23410942	MSRB2
ENSG00000148459	6.727618145	7.91704449	protein_coding	10	26986588	27035727	PDSS1
ENSG00000148468	9.232475031	8.114504097	protein_coding	10	15253642	15413061	FAM171A1
ENSG00000148481	7.784382559	7.607691112	protein_coding	10	15820169	15902519	FAM188A
ENSG00000148483	1.16343121	0.950786998	protein_coding	10	17794251	17842867	TMEM236
ENSG00000148484	9.778108644	9.595769114	protein_coding	10	16632610	16859527	RSU1
ENSG00000148488	0	0.697730409	protein_coding	10	17360382	17496386	ST8SIA6
ENSG00000148498	9.664408419	10.51767533	protein_coding	10	34398488	35104253	PARD3
ENSG00000148516	8.233881494	9.282254961	protein_coding	10	31607424	31821599	ZEB1
ENSG00000148572	7.695130106	7.605389119	protein_coding	10	64893050	64914783	NRBF2
ENSG00000148606	9.404225042	9.546823838	protein_coding	10	79729008	79789303	POLR3A
ENSG00000148634	9.439002622	9.11432951	protein_coding	10	69681665	69835105	HERC4
ENSG00000148655	0	0.697730409	protein_coding	10	77191211	78318978	C10orf11
ENSG00000148660	8.671313239	8.38394126	protein_coding	10	75572259	75634343	CAMK2G
ENSG00000148671	1.960915222	1.667587519	protein_coding	10	88727949	88730672	C10orf116
ENSG00000148672	10.37155313	10.35865784	protein_coding	10	88810243	88854627	GLUD1
ENSG00000148677	10.36297617	11.26583293	protein_coding	10	92671853	92681033	ANKRD1
ENSG00000148680	5.795854912	3.679994897	protein_coding	10	92500580	92617455	HTR7
ENSG00000148688	7.568924112	7.070480904	protein_coding	10	92631473	92668312	RPP30
ENSG00000148690	7.921045056	8.315177126	protein_coding	10	95427640	95462329	FRA10AC1
ENSG00000148700	7.990682129	6.371325053	protein_coding	10	111756126	111895323	ADD3
ENSG00000148719	9.105088364	8.820898829	protein_coding	10	74092588	74114988	DNAJB12
ENSG00000148730	10.23770949	9.700312778	protein_coding	10	72164135	72188374	EIF4EBP2
ENSG00000148735	2.57521082	1.353254395	protein_coding	10	115511213	115543188	C10orf81
ENSG00000148737	8.47750173	8.163833676	protein_coding	10	114710009	114927437	TCF7L2
ENSG00000148773	12.22066345	12.21434498	protein_coding	10	129894923	129924649	MKI67
ENSG00000148795	0.869158192	1.353254395	protein_coding	10	104590288	104597290	CYP17A1
ENSG00000148798	2.57521082	5.638744877	protein_coding	10	105036920	105050108	INA
ENSG00000148803	6.414691513	6.79148052	protein_coding	10	135168658	135171529	C10orf125
ENSG00000148814	6.146643566	7.070480904	protein_coding	10	134145614	134195010	LRRC27
ENSG00000148824	5.389447744	6.149127429	protein_coding	10	135207598	135234811	
ENSG00000148832	5.538004898	6.044260771	protein_coding	10	135192695	135205198	PAOX
ENSG00000148834	9.358631958	10.16553509	protein_coding	10	105995114	106027217	GSTO1
ENSG00000148835	6.956090577	7.803146634	protein_coding	10	105127724	105148822	TAF5
ENSG00000148840	10.84238558	10.54555896	protein_coding	10	103892787	103910082	PPRC1
ENSG00000148841	9.328343713	9.158178572	protein_coding	10	106071894	106098162	ITPRIP
ENSG00000148842	7.04456449	7.359856617	protein_coding	10	104678071	104838344	CNNM2
ENSG00000148843	10.65651747	10.89211697	protein_coding	10	105156405	105206049	PDCD11
ENSG00000148848	3.502389126	4.202780776	protein_coding	10	127700950	128077024	ADAM12
ENSG00000148908	8.271030151	8.957119685	protein_coding	10	121259340	121302220	RGS10
ENSG00000148925	8.410739551	8.83962623	protein_coding	11	13409548	13484844	BTBD10
ENSG00000148926	8.072698871	10.06189795	protein_coding	11	10326227	10328944	ADM
ENSG00000148935	0.499066092	1.353254395	protein_coding	11	22647188	22834601	GAS2
ENSG00000148943	8.445362311	8.414504719	protein_coding	11	27516123	27528320	LIN7C
ENSG00000148948	0	2.912743273	protein_coding	11	40135753	41481323	LRRC4C
ENSG00000148950	5.869093502	6.030599799	protein_coding	11	31453948	31531192	IMMP1L
ENSG00000148965	3.077135474	3.183544561	protein_coding	11	18252896	18258440	SAA4
ENSG00000148985	8.48251156	8.371803145	protein_coding	11	3818954	3847601	PGAP2
ENSG00000149016	7.64536895	7.805153958	protein_coding	11	62342517	62359649	TUT1
ENSG00000149021	0	0.390640832	protein_coding	11	62172575	62190667	SCGB1A1
ENSG00000149043	4.77879189	2.971122874	protein_coding	11	1848709	1858751	SYT8

ENSG00000149050	1.616589159	0.697730409	protein_coding	11	7020549	7041599	ZNF214
ENSG00000149084	9.999878142	10.06650758	protein_coding	11	43577986	43878167	HSD17B12
ENSG00000149089	7.1740722	7.635032283	protein_coding	11	34874641	34938046	APIP
ENSG00000149090	2.35979773	1.518964905	protein_coding	11	35453370	35551848	PAMR1
ENSG00000149091	9.807683852	9.577682064	protein_coding	11	46354455	46402104	DGKZ
ENSG00000149100	10.81285756	11.20251577	protein_coding	11	32605344	32627808	EIF3M
ENSG00000149115	11.73060292	11.56431502	protein_coding	11	57067112	57092426	TNKS1BP1
ENSG00000149131	2.928422289	4.363974406	protein_coding	11	57364860	57382326	SERPING1
ENSG00000149136	11.86007011	11.92638409	protein_coding	11	57093459	57103351	SSRP1
ENSG00000149150	7.395627075	7.022976646	protein_coding	11	57252007	57283259	SLC43A1
ENSG00000149177	9.241368875	9.225677649	protein_coding	11	48002110	48192393	PTPRJ
ENSG00000149179	8.497437504	8.387964709	protein_coding	11	46958240	47185936	C11orf49
ENSG00000149182	9.766517491	9.871482642	protein_coding	11	47185848	47198676	ARFGAP2
ENSG00000149187	10.93300807	10.60553028	protein_coding	11	47487496	47587121	CELF1
ENSG00000149196	8.35173048	8.806942451	protein_coding	11	86013253	86056969	C11orf73
ENSG00000149201	1.960915222	1.925536307	protein_coding	11	86085778	86134151	CCDC81
ENSG00000149212	1.799000381	1.802319292	protein_coding	11	94898704	94965705	SESN3
ENSG00000149218	8.879339004	8.696649474	protein_coding	11	94822974	94865809	ENDOD1
ENSG00000149231	7.31554162	7.873706627	protein_coding	11	96085933	96123087	CCDC82
ENSG00000149243	6.766474304	7.815148897	protein_coding	11	75133438	75141674	KLHL35
ENSG00000149256	0	0.697730409	protein_coding	11	78363876	79151992	ODZ4
ENSG00000149257	11.88314247	12.23198571	protein_coding	11	75273101	75283828	SERPINH1
ENSG00000149260	7.698004462	8.005247922	protein_coding	11	76777979	76837201	CAPN5
ENSG00000149262	8.179389493	8.124184315	protein_coding	11	77589766	77705724	INTS4
ENSG00000149269	9.855452651	9.618206363	protein_coding	11	77032752	77185680	PAK1
ENSG00000149273	13.25660174	13.69103991	protein_coding	11	75110530	75133324	RPS3
ENSG00000149289	8.462367008	8.838646614	protein_coding	11	109964087	110042566	ZC3H12C
ENSG00000149292	6.482942724	7.050313162	protein_coding	11	113185251	113254266	TTC12
ENSG00000149294	0	0.390640832	protein_coding	11	112831997	113149158	NCAM1
ENSG00000149300	0	0.390640832	protein_coding	11	111788756	111797596	C11orf52
ENSG00000149308	8.458982065	8.89250574	protein_coding	11	108027942	108093369	NPAT
ENSG00000149311	9.582007615	10.01149995	protein_coding	11	108093211	108239829	ATM
ENSG00000149313	8.753741308	9.014632836	protein_coding	11	105946228	105969437	AASDHPPT
ENSG00000149328	8.594725428	8.710638407	protein_coding	11	134201768	134248235	GLB1L2
ENSG00000149346	5.672682881	5.22435463	protein_coding	20	10415951	10617477	C20orf94
ENSG00000149357	9.648151859	10.12834013	protein_coding	11	71796941	71814433	LAMTOR1
ENSG00000149380	4.75690578	5.372493443	protein_coding	11	73946846	74022702	P4HA3
ENSG00000149403	2.10647801	0.950786998	protein_coding	11	120382468	120859101	GRIK4
ENSG00000149418	7.903720218	2.721932731	protein_coding	11	130029457	130080356	ST14
ENSG00000149428	11.98417782	12.19332809	protein_coding	11	118914899	118927940	HYOU1
ENSG00000149435	0.499066092	0	protein_coding	20	23965690	23969416	GGTLC1
ENSG00000149451	0	0.390640832	protein_coding	20	3648612	3662893	ADAM33
ENSG00000149474	7.546773135	8.088368066	protein_coding	20	18118499	18169031	CSRP2BP
ENSG00000149476	8.369868254	8.066778281	protein_coding	11	61100682	61116714	DAK
ENSG00000149480	10.90373985	10.87477986	protein_coding	11	62360686	62369312	MTA2
ENSG00000149483	8.331511968	8.730842828	protein_coding	11	61129473	61136683	TMEM138
ENSG00000149485	10.9821777	10.05937738	protein_coding	11	61567098	61647626	FADS1
ENSG00000149488	1.407729925	2.334191469	protein_coding	20	2517253	2622430	TMC2
ENSG00000149489	4.352265886	5.187895122	protein_coding	11	62379194	62382592	ROM1
ENSG00000149499	9.445858656	9.481799375	protein_coding	11	62369690	62380237	EML3
ENSG00000149503	10.15226439	10.08107738	protein_coding	11	61891445	61920635	INCENP
ENSG00000149506	1.16343121	1.925536307	protein_coding	11	60635035	60643166	ZP1
ENSG00000149527	6.998676229	4.127774132	protein_coding	1	2398898	2436969	PLCH2
ENSG00000149531	7.007971391	7.378849871	protein_coding	20	29611857	29634010	FRG1B
ENSG00000149532	10.37245301	10.30398946	protein_coding	11	61170121	61197503	CPSF7
ENSG00000149541	8.586993258	8.690147008	protein_coding	11	62382768	62389647	B3GAT3
ENSG00000149547	11.11553735	11.15333184	protein_coding	11	125439112	125454575	EI24
ENSG00000149548	6.364918751	7.116470702	protein_coding	11	124824017	124911385	CCDC15
ENSG00000149554	8.735671237	8.907531839	protein_coding	11	125495036	125546150	CHEK1
ENSG00000149557	1.407729925	1.518964905	protein_coding	11	125315646	125366213	FEZ1
ENSG00000149564	3.554067925	3.679994897	protein_coding	11	124622026	124632186	ESAM
ENSG00000149571	2.57521082	2.851901313	protein_coding	11	126293254	126873355	KIRREL3
ENSG00000149573	6.74439909	5.80042639	protein_coding	11	118124118	118135251	MPZL2

ENSG00000149577	8.539709228	8.714915547	protein_coding	11	117049449	117068160	SIDT2
ENSG00000149582	4.642199401	2.851901313	protein_coding	11	118401756	118417995	TMEM25
ENSG00000149591	7.218789138	11.64526021	protein_coding	11	117070037	117075503	TAGLN
ENSG00000149596	7.689364157	8.452140716	protein_coding	20	42740335	42816218	JPH2
ENSG00000149599	0.869158192	1.16600992	protein_coding	20	30435440	30458550	DUSP15
ENSG00000149600	7.933293789	8.455978641	protein_coding	20	31290493	31331814	COMMD7
ENSG00000149609	0.869158192	0.950786998	protein_coding	20	32250090	32251720	C20orf144
ENSG00000149633	4.437288965	4.622587519	protein_coding	20	36838890	36889174	KIAA1755
ENSG00000149634	3.211941663	2.971122874	protein_coding	20	44515128	44516274	SPATA25
ENSG00000149635	0.869158192	0.697730409	protein_coding	20	45169585	45179213	C20orf123
ENSG00000149636	8.795939543	9.401816026	protein_coding	20	35380194	35402230	DSN1
ENSG00000149639	10.922294	11.07355782	protein_coding	20	35405845	35492089	KIAA0889
ENSG00000149646	1.407729925	2.652276565	protein_coding	20	34556512	34618622	C20orf152
ENSG00000149651	0	1.16600992	protein_coding	20	44350990	44354469	SPINT4
ENSG00000149657	9.195324263	9.13522434	protein_coding	20	60697517	60710434	LSM14B
ENSG00000149658	10.02584819	9.69599206	protein_coding	20	61826781	61847586	YTHDF1
ENSG00000149679	8.166995477	8.254769334	protein_coding	20	60963686	60982341	CABLES2
ENSG00000149716	8.830404061	9.017232731	protein_coding	11	69467844	69490184	ORAOV1
ENSG00000149735	1.16343121	1.802319292	protein_coding	11	64701943	64703360	GPHA2
ENSG00000149743	7.524276744	7.928139131	protein_coding	11	63991271	63993726	TRPT1
ENSG00000149761	7.765324926	7.743685008	protein_coding	11	63993413	63998158	NUDT22
ENSG00000149781	4.75690578	5.317302248	protein_coding	11	63974150	63991354	FERMT3
ENSG00000149782	10.29483576	9.961978945	protein_coding	11	64018995	64036622	PLCB3
ENSG00000149792	9.453532949	9.401816026	protein_coding	11	64889655	64894843	MRPL49
ENSG00000149798	9.297405929	9.280091692	protein_coding	11	65082289	65089900	CDC42EP2
ENSG00000149806	10.48240375	11.16919932	protein_coding	11	64888100	64889945	FAU
ENSG00000149809	5.223818625	5.545764946	protein_coding	11	64879317	64883856	TM7SF2
ENSG00000149823	10.0016241	10.15770118	protein_coding	11	64856796	64879332	C11orf2
ENSG00000149922	4.464551814	4.342020395	protein_coding	16	30097114	30103208	TBX6
ENSG00000149923	10.67377603	10.67385839	protein_coding	16	30087299	30096698	PPP4C
ENSG00000149925	14.59712286	14.51531629	protein_coding	16	30064411	30081778	ALDOA
ENSG00000149926	4.322773689	4.448594745	protein_coding	16	30035748	30064299	FAM57B
ENSG00000149927	4.75690578	4.622587519	protein_coding	16	30016830	30034591	DOC2A
ENSG00000149929	7.058051099	7.340609974	protein_coding	16	30003645	30007757	HIRIP3
ENSG00000149930	9.803686393	9.924613326	protein_coding	16	29984962	30003582	TAOK2
ENSG00000149932	8.671313239	8.91219558	protein_coding	16	29952206	29984373	TMEM219
ENSG00000149948	9.139327269	7.902117654	protein_coding	12	66217911	66360075	HMGA2
ENSG00000149968	1.16343121	4.202780776	protein_coding	11	102706532	102714534	MMP3
ENSG00000150051	3.146112541	3.32456471	protein_coding	10	27961803	28034989	MKX
ENSG00000150054	5.40360309	4.406904905	protein_coding	10	28339922	28591995	MPP7
ENSG00000150076	0.499066092	2.501982735	protein_coding	10	32856651	33171802	C10orf68
ENSG00000150093	13.10851574	14.694746	protein_coding	10	33189247	33294720	ITGB1
ENSG00000150165	2.671945279	1.667587519	protein_coding	10	47157983	47174093	ANXA8L1
ENSG00000150201	0.499066092	0	protein_coding	10	43867090	43871783	FXYD4
ENSG00000150275	0	0.390640832	protein_coding	10	55562531	57387702	PCDH15
ENSG00000150281	5.806547963	6.712401119	protein_coding	16	30907928	30914881	CTF1
ENSG00000150316	8.429884808	8.844514354	processed_transc	11	94695787	94706776	CWC15
ENSG00000150337	0	0.390640832	protein_coding	1	149754227	149764074	FCGR1A
ENSG00000150347	8.935220147	8.337540265	protein_coding	10	63661059	63856703	ARID5B
ENSG00000150401	6.716321419	6.677762549	protein_coding	13	114110134	114145267	DCUN1D2
ENSG00000150403	9.419108071	9.233897238	protein_coding	13	114145310	114204542	TMCO3
ENSG00000150433	7.521034145	7.756190105	protein_coding	11	124966398	124981659	TMEM218
ENSG00000150455	5.672682881	5.629711952	protein_coding	11	126152960	126168740	TIRAP
ENSG00000150456	5.752272083	6.002883551	protein_coding	13	21296543	21348097	N6AMT2
ENSG00000150457	8.485841807	8.276635048	protein_coding	13	21547171	21635718	LATS2
ENSG00000150459	9.354994343	9.44751161	protein_coding	13	21714653	21723221	SAP18
ENSG00000150471	0	0.697730409	protein_coding	4	62066976	62938184	LPHN3
ENSG00000150477	4.6658819	5.004391715	protein_coding	18	34409069	34811481	KIAA1328
ENSG00000150510	0.869158192	0.390640832	protein_coding	13	51796503	51857930	FAM124A
ENSG00000150527	4.322773689	4.7279204	protein_coding	14	39734488	39856156	CTAGE5
ENSG00000150540	7.609180529	7.126137778	protein_coding	2	138721590	138773930	HNMT
ENSG00000150551	0	1.353254395	protein_coding	2	133402337	133429152	LYPD1
ENSG00000150556	1.616589159	0	protein_coding	2	149894621	150071776	LYPD6B

ENSG00000150593	8.899456749	9.225677649	protein_coding	10	112631565	112659764	PDCD4
ENSG00000150594	0	0.390640832	protein_coding	10	112836790	112840658	ADRA2A
ENSG00000150625	0.869158192	0.390640832	protein_coding	4	176554085	176923815	GPM6A
ENSG00000150628	0.499066092	0.950786998	protein_coding	4	177105789	177116822	SPATA4
ENSG00000150636	1.616589159	3.133298822	protein_coding	18	66401969	66722426	CCDC102B
ENSG00000150637	1.407729925	1.802319292	protein_coding	18	67528097	67624160	CD226
ENSG00000150656	0	2.039052734	protein_coding	18	72201675	72252261	CNDP1
ENSG00000150667	2.57521082	2.501982735	protein_coding	15	39892232	40075031	FSIP1
ENSG00000150672	2.762599152	2.652276565	protein_coding	11	83166055	85338966	DLG2
ENSG00000150687	11.32270757	10.82766699	protein_coding	11	86502101	86663886	PRSS23
ENSG00000150712	9.847708073	9.314320673	protein_coding	5	32227100	32313115	MTMR12
ENSG00000150722	4.712111592	4.448594745	protein_coding	2	182818968	182996125	PPP1R1C
ENSG00000150753	12.89396063	12.50100421	protein_coding	5	10250033	10266524	CCT5
ENSG00000150756	6.887241849	6.819555981	protein_coding	5	10226442	10250009	FAM173B
ENSG00000150760	9.335744919	9.339469783	protein_coding	10	128593978	129250781	DOCK1
ENSG00000150764	6.681890601	6.27612435	protein_coding	11	111797868	111893308	DIXDC1
ENSG00000150768	9.752069226	9.586462181	protein_coding	11	111895538	111935114	DLAT
ENSG00000150773	4.381167248	4.744751758	protein_coding	11	111934734	111944998	PIH1D2
ENSG00000150776	8.782461599	9.144766978	protein_coding	11	111944810	111955874	C11orf57
ENSG00000150779	7.575190993	8.690147008	protein_coding	11	111955524	111957522	TIMM8B
ENSG00000150782	7.457998197	6.633261019	protein_coding	11	112013974	112034840	IL18
ENSG00000150787	7.273767634	7.672899552	protein_coding	11	112097088	112140678	PTS
ENSG00000150867	9.619633528	8.606292299	protein_coding	10	22823778	23003503	PIP4K2A
ENSG00000150873	1.407729925	0.950786998	protein_coding	2	11273179	11286916	C2orf50
ENSG00000150907	7.498129565	7.129345795	protein_coding	13	41129817	41240734	FOXO1
ENSG00000150938	10.93575031	11.2410239	protein_coding	2	36583069	36778278	CRIM1
ENSG00000150961	10.12154978	9.924613326	protein_coding	4	119643978	119759838	SEC24D
ENSG00000150967	7.517784241	6.803579823	protein_coding	12	123405498	123466196	ABCB9
ENSG00000150977	7.1740722	6.941369681	protein_coding	12	123899936	123921264	RILPL2
ENSG00000150990	9.483828293	9.457756176	protein_coding	12	125431371	125473668	DHX37
ENSG00000150991	12.09450121	11.78263365	protein_coding	12	125396150	125401914	UBC
ENSG00000150995	7.030950614	7.080460027	protein_coding	3	4535032	4889524	ITPR1
ENSG00000151006	6.298293508	6.779278886	protein_coding	16	31093907	31100949	PRSS53
ENSG00000151012	9.480493389	8.734007291	protein_coding	4	139085251	139163503	SLC7A11
ENSG00000151014	7.990682129	8.010476879	protein_coding	4	139936943	139966906	CCRN4L
ENSG00000151023	2.847891871	2.579085888	protein_coding	10	25270908	25305085	ENKUR
ENSG00000151025	1.616589159	0.390640832	protein_coding	10	25463991	25891155	GPR158
ENSG00000151062	1.407729925	1.518964905	protein_coding	12	1901123	2028002	CACNA2D4
ENSG00000151065	7.053569556	7.157901395	protein_coding	12	2055220	2113701	DCP1B
ENSG00000151067	0	0.390640832	protein_coding	12	2079952	2807115	CACNA1C
ENSG00000151090	6.442380648	5.767861411	protein_coding	3	24158651	24536773	THRB
ENSG00000151092	7.572060955	8.173189549	protein_coding	3	25760435	25831530	NGLY1
ENSG00000151093	6.664362065	6.877938671	protein_coding	3	25824408	25836025	OXSM
ENSG00000151116	7.737655476	7.635032283	protein_coding	11	18552748	18610294	UEVLD
ENSG00000151117	3.502389126	5.759604001	protein_coding	11	18714669	18726332	TMEM86A
ENSG00000151131	6.449220685	6.746227492	protein_coding	12	105380088	105443515	C12orf45
ENSG00000151135	9.100750797	9.084889332	protein_coding	12	107349497	107372556	C12orf23
ENSG00000151136	0.499066092	0	protein_coding	12	107712190	108053419	BTBD11
ENSG00000151148	9.085465781	8.768365845	protein_coding	12	109915207	109974507	UBE3B
ENSG00000151150	6.0776925	0.950786998	protein_coding	10	61788159	62493248	ANK3
ENSG00000151151	8.694526397	8.418443943	protein_coding	10	59951278	60027694	IPMK
ENSG00000151164	2.847891871	3.64455972	protein_coding	12	110939460	110969891	RAD9B
ENSG00000151176	9.459473748	9.018963395	protein_coding	12	113796371	113827203	PLBD2
ENSG00000151208	9.843171129	9.643669187	protein_coding	10	79550549	79686378	DLG5
ENSG00000151224	0.499066092	0	protein_coding	10	82031576	82049440	MAT1A
ENSG00000151229	6.634666104	6.592001111	protein_coding	12	40148823	40499891	SLC2A13
ENSG00000151233	8.041355121	8.375860541	protein_coding	12	42475647	42538681	GXYLT1
ENSG00000151239	10.06645173	10.02232546	protein_coding	12	44187526	44200178	TWF1
ENSG00000151240	7.140657543	7.51752531	protein_coding	10	320130	735683	DIP2C
ENSG00000151247	7.754320745	8.101495266	protein_coding	4	99792835	99851788	EIF4E
ENSG00000151276	6.965663283	7.192052139	protein_coding	3	65342053	66024509	MAGI1
ENSG00000151287	6.835971536	7.161039629	protein_coding	13	103418340	103426161	C13orf27
ENSG00000151292	8.940084182	8.831770617	protein_coding	5	122847793	122952739	CSNK1G3

ENSG00000151303	4.292665995	3.906303962	processed_transc	10	88728247	88784489	AGAP11
ENSG00000151304	7.08025234	7.260948155	protein_coding	5	121297656	121411265	SRFBP1
ENSG00000151320	5.672682881	5.6022688	protein_coding	14	32798479	33300567	AKAP6
ENSG00000151322	0.869158192	1.353254395	protein_coding	14	33404139	34273382	NPAS3
ENSG00000151327	8.632776617	8.765275934	protein_coding	14	35514113	35582336	FAM177A1
ENSG00000151332	7.097770581	7.532142019	protein_coding	14	36767770	36789882	MBIP
ENSG00000151338	6.892270075	7.135740508	protein_coding	14	37667118	38021566	MIPOL1
ENSG00000151348	10.39653997	11.17697121	protein_coding	11	44117099	44266979	EXT2
ENSG00000151353	7.521034145	7.655203404	protein_coding	2	667335	677439	TMEM18
ENSG00000151360	0.869158192	0.950786998	protein_coding	2	3705785	3750261	ALLC
ENSG00000151364	7.053569556	6.2290532	protein_coding	11	77726761	77757237	KCTD14
ENSG00000151366	6.62865285	7.37615181	protein_coding	11	77779350	77791265	NDUFC2
ENSG00000151376	7.562629889	7.670699368	protein_coding	11	86152150	86383678	ME3
ENSG00000151388	1.16343121	0.390640832	protein_coding	5	33523640	33892297	ADAMTS12
ENSG00000151413	7.102117104	6.984522608	protein_coding	14	31959162	32330430	NUBPL
ENSG00000151414	8.12703021	8.900977169	protein_coding	1	198126093	198291550	NEK7
ENSG00000151418	0	0.390640832	protein_coding	1	198492352	198510075	ATP6V1G3
ENSG00000151422	8.05038018	7.973469823	protein_coding	5	108083523	108532542	FER
ENSG00000151445	8.847332411	9.218917524	protein_coding	14	77893018	77924295	C14orf133
ENSG00000151458	8.487504052	8.586588042	protein_coding	4	125585207	125633887	ANKRD50
ENSG00000151461	9.040880241	8.749726325	protein_coding	10	11962021	12085169	UPF2
ENSG00000151465	9.804353406	9.441071616	protein_coding	10	12237964	12292588	CDC123
ENSG00000151466	7.140657543	7.195117037	protein_coding	4	129786076	130014764	SCLT1
ENSG00000151468	9.456082002	7.743685008	protein_coding	10	12938627	13141652	CCDC3
ENSG00000151470	5.660945913	5.792353798	protein_coding	4	130014472	130034487	C4orf33
ENSG00000151474	8.269098624	7.394933125	protein_coding	10	13685706	14504141	FRMD4A
ENSG00000151491	9.414746625	9.273582355	protein_coding	12	15773092	16035263	EPS8
ENSG00000151498	8.422952257	8.720244202	protein_coding	11	134123389	134135749	ACAD8
ENSG00000151500	7.599989788	8.094946597	protein_coding	11	134118173	134123285	THYN1
ENSG00000151502	9.756212088	10.04925084	protein_coding	11	134094539	134117686	VPS26B
ENSG00000151503	10.1124219	10.39908895	protein_coding	11	134020014	134095348	NCAPD3
ENSG00000151532	8.154494062	8.437980325	protein_coding	10	114206756	114578503	VTI1A
ENSG00000151552	7.908691438	8.171634445	protein_coding	4	17461884	17513857	QDPR
ENSG00000151553	8.297803463	8.762179391	protein_coding	10	116581503	116659591	FAM160B1
ENSG00000151575	4.230497448	4.710890357	protein_coding	15	56536207	56738195	TEX9
ENSG00000151576	8.668385155	8.545152786	protein_coding	3	113724680	113807269	QTRTD1
ENSG00000151611	4.593635215	5.085909553	protein_coding	4	146539415	146581187	MMAA
ENSG00000151612	8.085926212	8.409235632	protein_coding	4	146678779	146859787	ZNF827
ENSG00000151617	4.689181911	4.547834947	protein_coding	4	148402069	148466106	EDNRA
ENSG00000151623	6.877132514	7.04352744	protein_coding	4	148999913	149365850	NR3C2
ENSG00000151631	1.407729925	0.390640832	pseudogene	10	4914093	4934167	
ENSG00000151632	1.799000381	1.16600992	protein_coding	10	5029967	5060207	AKR1C2
ENSG00000151640	5.538004898	6.35497494	protein_coding	10	134000404	134019266	DPYSL4
ENSG00000151650	0.869158192	0.390640832	protein_coding	10	135051175	135055433	VENTX
ENSG00000151651	8.3352092	9.149514732	protein_coding	10	135075907	135090372	ADAM8
ENSG00000151655	0.869158192	0.697730409	protein_coding	10	7745232	7791483	ITIH2
ENSG00000151657	6.710639711	6.544728831	protein_coding	10	7795188	7829990	KIN
ENSG00000151665	5.752272083	6.316088276	protein_coding	2	46808076	46844258	PIGF
ENSG00000151687	4.062237333	3.411459265	protein_coding	2	190539016	190625919	ANKAR
ENSG00000151689	5.141750194	4.640685763	protein_coding	2	191208196	191236391	INPP1
ENSG00000151690	6.26766507	6.287656023	protein_coding	2	191273081	191373931	MFSD6
ENSG00000151692	5.948480845	4.42790041	protein_coding	2	7057523	7208417	RNF144A
ENSG00000151693	9.203438318	8.26792849	protein_coding	2	9346894	9545812	ASAP2
ENSG00000151694	9.273518431	8.614328238	protein_coding	2	9628615	9695921	ADAM17
ENSG00000151704	1.616589159	1.667587519	protein_coding	11	128706210	128737268	KCNJ1
ENSG00000151715	6.196276899	5.767861411	protein_coding	11	129685714	129729898	TMEM45B
ENSG00000151718	9.325558442	9.38782027	protein_coding	4	184020446	184241930	WWC2
ENSG00000151725	7.870982917	8.846464976	protein_coding	4	185615772	185655287	MLF1IP
ENSG00000151726	9.662202373	9.742822827	protein_coding	4	185676749	185747972	ACSL1
ENSG00000151729	7.993024472	8.692317755	protein_coding	4	186064395	186068434	SLC25A4
ENSG00000151743	5.752272083	5.988823159	protein_coding	12	31824071	31882108	AMN1
ENSG00000151746	8.092494681	7.743685008	protein_coding	12	32259769	32536567	BICD1
ENSG00000151748	9.008829705	8.637044457	protein_coding	14	51098776	51135056	SAV1

ENSG00000151773	4.593635215	4.7279204	protein_coding	13	44398045	44453864	CCDC122
ENSG00000151778	0.499066092	0	protein_coding	13	44947801	44971850	SERP2
ENSG00000151779	8.883132512	8.581912372	protein_coding	2	15307032	15701454	NBAS
ENSG00000151789	0.499066092	1.353254395	protein_coding	3	21459915	22414812	ZNF385D
ENSG00000151790	2.847891871	4.448594745	protein_coding	4	156775890	156841558	TDO2
ENSG00000151806	8.717371959	8.973277306	protein_coding	4	44680444	44702943	GUF1
ENSG00000151812	1.16343121	1.16600992	protein_coding	14	58030640	58448912	SLC35F4
ENSG00000151835	10.07421587	10.18184912	protein_coding	13	23902965	24007841	SACS
ENSG00000151838	3.077135474	2.579085888	protein_coding	14	59971801	60043549	C14orf38
ENSG00000151846	3.077135474	2.579085888	protein_coding	13	25670006	25673392	PABPC3
ENSG00000151849	8.177331204	8.202422444	protein_coding	13	25457171	25497018	CENPJ
ENSG00000151876	7.423680977	6.854868327	protein_coding	5	41925356	41941845	FBXO4
ENSG00000151881	8.054871625	8.196317182	protein_coding	5	43444354	43483995	C5orf28
ENSG00000151882	7.556308086	7.161039629	protein_coding	5	43376747	43412493	CCL28
ENSG00000151883	8.023133844	8.22508979	protein_coding	5	49961733	50142356	PARP8
ENSG00000151892	0	0.390640832	protein_coding	10	117816444	118033126	GFRA1
ENSG00000151893	9.804353406	9.775439595	protein_coding	10	120433679	120514761	C10orf46
ENSG00000151914	10.90093603	10.75864974	protein_coding	6	56322785	56819426	DST
ENSG00000151917	4.322773689	4.508954154	protein_coding	6	56819773	56892140	BEND6
ENSG00000151923	9.809013881	10.16748693	protein_coding	10	121334199	121356541	TIAL1
ENSG00000151929	10.08523551	10.053901	protein_coding	10	121410882	121437331	BAG3
ENSG00000151963	0.499066092	0.390640832	pseudogene	10	38172522	38173037	
ENSG00000151967	4.097495944	3.813624741	protein_coding	3	159557650	159615149	SCHIP1
ENSG00000152022	9.694224448	9.826732496	protein_coding	1	145477085	145501669	LIX1L
ENSG00000152034	0.499066092	0	protein_coding	6	100367786	100442114	MCHR2
ENSG00000152049	1.407729925	0.950786998	protein_coding	2	223916532	224063117	KCNE4
ENSG00000152056	7.026383958	5.57429348	protein_coding	2	224616403	224702744	AP1S3
ENSG00000152061	6.956090577	7.093659051	protein_coding	1	174128548	174964445	RABGAP1L
ENSG00000152076	4.517578978	4.385599336	protein_coding	2	130896860	130902707	CCDC74B
ENSG00000152078	6.554468172	6.716673044	protein_coding	1	95582894	95663163	TMEM56
ENSG00000152082	8.205883411	8.228085371	protein_coding	2	130939310	130948302	MZT2B
ENSG00000152086	0.869158192	0	protein_coding	2	130949318	130956034	TUBA3E
ENSG00000152102	10.88494397	10.20103307	protein_coding	2	131805449	131851033	FAM168B
ENSG00000152104	7.881135284	7.549009758	protein_coding	1	214530851	214725792	PTPN14
ENSG00000152117	7.289098491	7.632773539	pseudogene	2	132250386	132279145	
ENSG00000152127	9.850294221	9.947057728	protein_coding	2	134877554	135212192	MGAT5
ENSG00000152128	1.799000381	0.697730409	protein_coding	2	135213330	135476570	TMEM163
ENSG00000152133	7.202688343	7.213370937	protein_coding	2	37311594	37326387	CCDC75
ENSG00000152137	8.426422697	8.998934298	protein_coding	12	119616447	119658936	HSPB8
ENSG00000152147	6.922076877	7.558560655	protein_coding	2	38978676	39009598	GEMIN6
ENSG00000152154	3.448790144	3.027231696	protein_coding	2	39892122	39945103	TMEM178
ENSG00000152193	7.084651883	6.750400563	protein_coding	13	79188426	79233314	RNF219
ENSG00000152217	0.499066092	0.390640832	protein_coding	18	42260138	42648475	SETBP1
ENSG00000152219	7.430610036	7.774746834	protein_coding	11	30344598	30359774	C11orf46
ENSG00000152223	9.678302254	9.864280473	protein_coding	18	43427574	43547240	EPG5
ENSG00000152229	7.084651883	6.023720496	protein_coding	18	43563502	43652238	PSTPIP2
ENSG00000152234	11.79812843	11.72548571	protein_coding	18	43664110	43684300	ATP5A1
ENSG00000152240	7.165790811	7.724721747	protein_coding	18	43684298	43708299	HAUS1
ENSG00000152242	8.245716314	8.135396417	protein_coding	18	43753988	43846954	C18orf25
ENSG00000152253	6.922076877	7.219404604	protein_coding	2	169690642	169769881	SPC25
ENSG00000152256	7.95748362	8.162268448	protein_coding	2	173420101	173489823	PDK1
ENSG00000152268	4.026095388	4.710890357	polymorphic_pse	11	13983914	14289646	SPON1
ENSG00000152284	7.556308086	7.198175438	protein_coding	2	85360533	85537511	TCF7L1
ENSG00000152291	11.52563376	11.51917171	protein_coding	2	85545147	85555548	TGOLN2
ENSG00000152292	2.238690726	2.039052734	protein_coding	2	85645844	85664152	SH2D6
ENSG00000152332	10.49856778	10.77102634	protein_coding	1	162467041	162499419	UHMK1
ENSG00000152348	5.986594255	6.167968749	protein_coding	5	81267844	81572241	ATG10
ENSG00000152359	7.058051099	7.240325909	protein_coding	5	74969949	75013313	POC5
ENSG00000152377	8.685865174	9.727557272	protein_coding	5	136310987	136934068	SPOCK1
ENSG00000152380	4.568725997	4.528525526	protein_coding	5	79783788	79838382	FAM151B
ENSG00000152382	7.194570056	7.512619979	protein_coding	1	166825747	166845564	TADA1
ENSG00000152402	0	0.697730409	protein_coding	11	106555148	106889250	GUCY1A2
ENSG00000152404	7.54038131	7.752033772	protein_coding	11	107197071	107328572	CWF19L2

ENSG00000152409	8.14188337	8.644911378	protein_coding	5	78531925	78623038	JMY
ENSG00000152413	7.639400234	7.219404604	protein_coding	5	78668459	78810040	HOMER1
ENSG00000152422	6.856698814	6.539915281	protein_coding	5	82373317	82652548	XRCC4
ENSG00000152433	4.923348194	5.767861411	protein_coding	19	57874891	57890923	ZNF547
ENSG00000152439	6.350376764	6.392840898	protein_coding	19	58011309	58024436	ZNF773
ENSG00000152443	7.590740122	7.57982176	protein_coding	19	58258164	58269527	ZNF776
ENSG00000152454	7.165790811	7.485338407	protein_coding	19	58452206	58459077	ZNF256
ENSG00000152455	8.045874708	7.776794036	protein_coding	10	14920819	14946314	SUV39H2
ENSG00000152457	7.773523328	7.362585296	protein_coding	10	14939358	14996431	DCLRE1C
ENSG00000152463	2.471521042	2.334191469	protein_coding	10	15074226	15115851	OLAH
ENSG00000152464	6.814942123	6.729413443	protein_coding	10	15139179	15148129	RPP38
ENSG00000152465	9.367686069	8.597053222	protein_coding	10	15144583	15210692	NMT2
ENSG00000152467	3.989024711	4.101879561	protein_coding	19	58545400	58565999	ZSCAN1
ENSG00000152475	6.103936836	6.559073862	protein_coding	19	58878993	58892389	ZNF837
ENSG00000152484	8.263288478	7.760334499	protein_coding	13	27640293	27746033	USP12
ENSG00000152487	0.869158192	1.16600992	processed_transc	10	18940195	18948196	
ENSG00000152492	8.984315711	9.551016476	protein_coding	3	191046866	191116459	CCDC50
ENSG00000152495	0.499066092	1.667587519	protein_coding	5	110559351	110830584	CAMK4
ENSG00000152503	6.535310252	6.450406542	protein_coding	5	114460459	114516243	TRIM36
ENSG00000152518	9.492131981	9.082408649	protein_coding	2	43449541	43453748	ZFP36L2
ENSG00000152520	8.400188597	7.69255166	protein_coding	13	28712643	28869475	PAN3
ENSG00000152527	5.346128795	4.048652948	protein_coding	2	43864412	43995126	PLEKHH2
ENSG00000152556	10.2752422	10.07026818	protein_coding	12	48498922	48540187	PFKM
ENSG00000152558	11.93019652	12.4517215	protein_coding	11	102267063	102341115	TMEM123
ENSG00000152578	0.499066092	0.390640832	protein_coding	11	105480721	105852819	GRIA4
ENSG00000152580	1.799000381	0.697730409	protein_coding	3	151143172	151176497	IGSF10
ENSG00000152582	5.763291843	6.198834244	protein_coding	5	35617946	35814713	SPEF2
ENSG00000152583	1.616589159	2.039052734	protein_coding	4	88394487	88452213	SPARCL1
ENSG00000152601	10.49609276	10.21619866	protein_coding	3	151961617	152183569	MBNL1
ENSG00000152611	2.10647801	1.16600992	protein_coding	5	35904397	35938881	CAPSL
ENSG00000152620	9.726959073	9.615349218	protein_coding	5	36192694	36242381	NADKD1
ENSG00000152642	7.292905885	7.090370599	protein_coding	3	32147181	32210205	GPD1L
ENSG00000152661	9.036344956	9.281534232	protein_coding	6	121756791	121770873	GJA1
ENSG00000152669	4.883502971	3.183544561	protein_coding	5	54526980	54529508	CCNO
ENSG00000152670	0	0.697730409	protein_coding	5	55033845	55112985	DDX4
ENSG00000152672	0.499066092	0.390640832	protein_coding	2	71035775	71047732	CLEC4F
ENSG00000152683	8.314756163	8.65940853	protein_coding	2	32390933	32449448	SLC30A6
ENSG00000152684	8.860220382	8.853271458	protein_coding	5	52083774	52099880	PELO
ENSG00000152689	3.989024711	4.888000647	protein_coding	2	33661391	33789817	RASGRP3
ENSG00000152700	8.579219424	8.52337065	protein_coding	5	133934233	133984961	SAR1B
ENSG00000152705	2.238690726	3.027231696	protein_coding	5	134303596	134347392	CATSPER3
ENSG00000152749	7.855619141	7.795089291	protein_coding	13	95254157	95286899	GPR180
ENSG00000152763	2.471521042	3.279072565	protein_coding	1	67278568	67390570	WDR78
ENSG00000152766	5.286269488	0	protein_coding	10	90581889	90611575	ANKRD22
ENSG00000152767	0	0.390640832	protein_coding	13	98795434	99102023	FARP1
ENSG00000152778	8.092494681	8.790825214	protein_coding	10	91174343	91180758	IFIT5
ENSG00000152782	6.196276899	6.077857763	protein_coding	10	91342745	91405215	PANK1
ENSG00000152784	6.068837212	6.023720496	protein_coding	4	81105033	81125483	PRDM8
ENSG00000152785	0.869158192	0	protein_coding	4	81952119	81978685	BMP3
ENSG00000152795	11.09932233	11.14642116	protein_coding	4	83343717	83351294	HNRPDL
ENSG00000152804	5.054730169	5.995870484	protein_coding	10	94447945	94455403	HHEX
ENSG00000152818	9.615078651	9.496780665	protein_coding	6	144606837	145174170	UTRN
ENSG00000152822	1.16343121	1.16600992	protein_coding	6	146348782	146758734	GRM1
ENSG00000152894	10.11994317	10.04203463	protein_coding	6	128289924	128841870	PTPRK
ENSG00000152904	8.685865174	8.700968226	protein_coding	1	235490665	235507847	GGPS1
ENSG00000152926	6.616550626	6.299096252	protein_coding	7	64437285	64467062	ZNF117
ENSG00000152931	0.499066092	0.697730409	lincRNA	5	59783540	59843484	PART1
ENSG00000152936	0.869158192	1.353254395	protein_coding	12	25562241	25801513	IFLTD1
ENSG00000152939	7.186405829	6.235021771	protein_coding	5	68710939	68740157	MARVELD2
ENSG00000152942	7.686472517	7.799123587	protein_coding	5	68665120	68710628	RAD17
ENSG00000152944	7.837485466	7.37615181	protein_coding	12	27175479	27219276	MED21
ENSG00000152952	11.34625883	12.05993995	protein_coding	3	145787227	145881440	PLOD2
ENSG00000152969	4.464551814	5.259915406	protein_coding	4	6027926	6202318	JAKMIP1

ENSG00000152990	9.198372384	9.375714884	protein_coding	4	22346694	22517677	GPR125
ENSG00000153006	6.9512803	6.771086752	protein_coding	5	64016338	64064512	SREK1IP1
ENSG00000153015	7.842689815	7.67728988	protein_coding	5	64064757	64314418	CWC27
ENSG00000153029	7.474855471	6.646756201	protein_coding	1	181003067	181031074	MR1
ENSG00000153037	6.676071387	6.633261019	protein_coding	5	112196885	112228776	SRP19
ENSG00000153044	7.734859118	7.644032061	protein_coding	5	68485375	68506184	CENPH
ENSG00000153046	8.867898261	8.865827498	protein_coding	6	4706345	4955785	CDYL
ENSG00000153048	9.464546462	10.04585947	protein_coding	16	8946799	8962866	CARHSP1
ENSG00000153060	1.407729925	0.950786998	protein_coding	16	10721358	10788802	TEKT5
ENSG00000153064	1.960915222	2.788380093	protein_coding	4	102332443	102995969	BANK1
ENSG00000153066	9.144604451	8.922403049	protein_coding	16	11772943	11836704	TXNDC11
ENSG00000153071	0.499066092	0.697730409	protein_coding	5	39371780	39462402	DAB2
ENSG00000153086	0.869158192	1.16600992	protein_coding	2	135596117	135659604	ACMSD
ENSG00000153093	3.830412367	0	protein_coding	2	111490150	111875799	ACOXL
ENSG00000153094	7.67484765	7.249200135	protein_coding	2	111876955	111924587	BCL2L11
ENSG00000153107	8.901951857	8.471229048	protein_coding	2	112523848	112642267	ANAPC1
ENSG00000153113	10.77456633	10.55304383	protein_coding	5	95865525	96115299	CAST
ENSG00000153130	8.203862602	8.734007291	protein_coding	4	141178440	141303710	SCOC
ENSG00000153132	0.869158192	2.039052734	protein_coding	4	141309609	141349122	CLGN
ENSG00000153140	6.579622637	6.766973175	protein_coding	5	89688078	89705603	CETN3
ENSG00000153147	10.40095672	10.26149292	protein_coding	4	144434616	144478639	SMARCA5
ENSG00000153157	0	1.353254395	protein_coding	6	10748027	10979553	SYCP2L
ENSG00000153165	0.499066092	0.697730409	protein_coding	2	107007564	107084832	RGPD3
ENSG00000153179	9.332049062	9.580028658	protein_coding	12	65004293	65091347	RASSF3
ENSG00000153187	12.63715499	12.47712421	protein_coding	1	245014468	245027844	HNRNPU
ENSG00000153201	10.3061829	10.09959632	protein_coding	2	109335937	109402267	RANBP2
ENSG00000153207	9.492131981	9.540813141	protein_coding	1	247002400	247095280	AHCTF1
ENSG00000153208	5.375152132	2.039052734	protein_coding	2	112656056	112787138	MERTK
ENSG00000153214	8.479173607	7.825075067	protein_coding	2	112812800	112876895	TMEM87B
ENSG00000153233	4.75690578	5.41517489	protein_coding	12	71031853	71314623	PTPRR
ENSG00000153234	8.394884042	4.406904905	protein_coding	2	157180944	157198860	NR4A2
ENSG00000153237	1.799000381	2.334191469	protein_coding	2	159027593	159313265	CCDC148
ENSG00000153246	3.744048221	3.748356452	protein_coding	2	160788519	160919121	PLA2R1
ENSG00000153250	9.438143322	8.948064416	protein_coding	2	161128662	161350305	RBMS1
ENSG00000153291	6.421663777	6.815578497	protein_coding	6	46620678	46649356	SLC25A27
ENSG00000153292	4.689181911	1.353254395	protein_coding	6	46963107	47010099	GPR110
ENSG00000153294	4.999881834	3.532712221	protein_coding	6	47653600	47689757	GPR115
ENSG00000153303	0.499066092	1.518964905	protein_coding	6	168456425	168482237	FRMD1
ENSG00000153310	8.691645096	8.234057939	protein_coding	8	130853716	131029375	FAM49B
ENSG00000153317	9.360447332	8.813937516	protein_coding	8	131064353	131455906	ASAP1
ENSG00000153339	8.613115289	8.458531597	protein_coding	18	29409136	29523063	TRAPPC8
ENSG00000153347	0	0.697730409	protein_coding	5	94727048	94786158	FAM81B
ENSG00000153363	5.806547963	5.620622115	processed_transc	1	211556145	211605878	LINC00467
ENSG00000153391	7.07583934	6.610484676	protein_coding	18	33034786	33077955	INO80C
ENSG00000153395	11.40240698	11.37323979	protein_coding	5	1456595	1524092	LPCAT1
ENSG00000153404	3.448790144	1.925536307	protein_coding	5	140373	190085	PLEKHG4B
ENSG00000153406	9.054401174	9.391832931	protein_coding	16	4511695	4524896	NMRAL1
ENSG00000153443	9.273518431	9.53356709	protein_coding	16	4656111	4665028	FAM100A
ENSG00000153446	0	1.925536307	protein_coding	16	5094123	5116111	C16orf89
ENSG00000153485	6.393570237	6.382123086	protein_coding	14	93651296	93653434	C14orf109
ENSG00000153487	7.097770581	7.216390925	protein_coding	13	111365083	111373421	ING1
ENSG00000153495	1.616589159	1.667587519	protein_coding	13	111968531	111996596	C13orf16
ENSG00000153531	5.417620896	5.248158938	protein_coding	13	114076260	114107839	ADPRHL1
ENSG00000153551	8.690202285	8.401295742	protein_coding	3	32433163	32524559	CMTM7
ENSG00000153558	5.498982484	5.767861411	protein_coding	3	33318517	33445154	FBXL2
ENSG00000153560	10.11994317	10.13034291	protein_coding	3	33429828	33482863	UBP1
ENSG00000153561	9.811006628	9.498022112	protein_coding	2	86947296	87005164	RMND5A
ENSG00000153563	1.16343121	0	protein_coding	2	87011729	87035519	CD8A
ENSG00000153574	8.398422578	8.046558348	protein_coding	2	88991162	89050445	RPIA
ENSG00000153575	8.092494681	8.026050991	protein_coding	15	22833395	22873892	TUBGCP5
ENSG00000153666	5.795854912	6.530239731	protein_coding	15	23255242	23268823	GOLGA8IP
ENSG00000153707	0.869158192	0.697730409	protein_coding	9	8314246	10612723	PTPRD
ENSG00000153714	6.35766608	6.831423059	protein_coding	9	12775020	12822130	C9orf150

ENSG00000153721	7.692250012	7.612284109	protein_coding	6	154726311	154831793	CNKSR3
ENSG00000153767	7.988335976	7.668495823	protein_coding	3	120461484	120501916	GTF2E1
ENSG00000153774	8.698837588	8.684705808	protein_coding	16	75327596	75467383	CFDP1
ENSG00000153786	10.14227455	9.995767095	protein_coding	16	85007787	85045141	ZDHHC7
ENSG00000153789	0.499066092	0.697730409	protein_coding	16	85131965	85146114	FAM92B
ENSG00000153790	6.129712275	5.42565083	protein_coding	7	25174316	25219975	C7orf31
ENSG00000153802	0	0.697730409	protein_coding	4	68686594	68749750	TMPRSS11D
ENSG00000153814	6.343050432	5.901455548	protein_coding	7	27870192	28220362	JAZF1
ENSG00000153815	10.74255805	10.06483306	protein_coding	16	81478775	81745173	CMIP
ENSG00000153823	6.462804234	6.896886088	protein_coding	2	229715242	230136001	PID1
ENSG00000153827	11.13104053	10.91390641	protein_coding	2	230631930	230787955	TRIP12
ENSG00000153832	5.948480845	5.125003832	protein_coding	2	230787018	230877825	FBXO36
ENSG00000153879	8.749591335	9.132029383	protein_coding	19	33864609	33873591	CEBPG
ENSG00000153885	7.337827682	7.915187059	protein_coding	19	34287751	34306668	KCTD15
ENSG00000153896	5.072559152	4.917990497	protein_coding	19	35248979	35270385	ZNF599
ENSG00000153898	4.491309013	3.081239798	protein_coding	1	85391268	85462796	MCOLN2
ENSG00000153902	3.950976309	3.453019579	protein_coding	19	35615417	35633355	LGI4
ENSG00000153904	8.732871027	8.168519197	protein_coding	1	85784168	86044046	DDAH1
ENSG00000153914	9.633963159	9.795772355	protein_coding	5	65435799	65479443	SREK1
ENSG00000153922	9.319038489	9.231660172	protein_coding	5	98190908	98262240	CHD1
ENSG00000153930	1.616589159	0.950786998	protein_coding	17	54230836	54589020	ANKFN1
ENSG00000153933	7.254372086	7.240325909	protein_coding	17	54911460	54946034	DGKE
ENSG00000153936	9.348606364	9.150304507	protein_coding	1	87380331	87634887	HS2ST1
ENSG00000153944	8.227927477	7.490337219	protein_coding	17	55333212	55757658	MSI2
ENSG00000153975	6.664362065	6.923001245	protein_coding	6	116956781	116989957	ZUFSP
ENSG00000153976	1.799000381	1.353254395	protein_coding	17	13399006	13505244	HS3ST3A1
ENSG00000153982	5.525114348	5.361622823	protein_coding	17	57297828	57353322	GDPD1
ENSG00000153989	8.965354743	8.823871996	protein_coding	6	117996665	118031803	NUS1
ENSG00000153993	0	1.16600992	protein_coding	7	84624869	84816171	SEMA3D
ENSG00000154001	10.41150208	10.35386622	protein_coding	14	63838075	64010092	PPP2R5E
ENSG00000154016	2.762599152	1.802319292	protein_coding	17	18923997	18950439	GRAP
ENSG00000154025	1.407729925	2.579085888	protein_coding	17	18853989	18924004	SLC5A10
ENSG00000154035	6.456028446	8.176294739	protein_coding	17	21142183	21156722	C17orf103
ENSG00000154040	5.40360309	4.566889334	protein_coding	18	21718942	21741564	CABYR
ENSG00000154059	8.421213901	7.8660349	protein_coding	18	22006580	22033499	IMPACT
ENSG00000154065	5.575999561	4.566889334	protein_coding	18	21178890	21242849	ANKRD29
ENSG00000154079	5.948480845	5.96747225	protein_coding	6	71276620	71299272	C6orf57
ENSG00000154096	1.616589159	1.667587519	protein_coding	11	119288090	119295695	THY1
ENSG00000154099	3.871730003	3.279072565	protein_coding	16	84178865	84212373	DNAAF1
ENSG00000154102	7.326727684	5.709041716	protein_coding	16	85741123	85784689	C16orf74
ENSG00000154114	7.797842652	8.44700755	protein_coding	11	120894781	120961484	TBCEL
ENSG00000154118	3.211941663	3.368666104	protein_coding	16	87635441	87731762	JPH3
ENSG00000154122	9.68845487	8.887777807	protein_coding	5	14704910	14871887	ANKH
ENSG00000154124	9.237422823	8.717049372	protein_coding	5	14664773	14699820	FAM105B
ENSG00000154127	9.362260424	9.430707456	protein_coding	11	122526383	122685181	UBASH3B
ENSG00000154133	8.552470601	10.47418196	protein_coding	11	124753587	124768396	ROBO4
ENSG00000154134	8.29020451	9.263397979	protein_coding	11	124735282	124751366	ROBO3
ENSG00000154144	8.635777795	8.786767612	protein_coding	11	124492732	124505287	TBRG1
ENSG00000154146	6.738827102	6.716673044	protein_coding	11	124609829	124617869	NRGN
ENSG00000154153	2.471521042	2.039052734	protein_coding	5	16473147	16617167	FAM134B
ENSG00000154162	0.499066092	0	protein_coding	5	21750777	22853731	CDH12
ENSG00000154174	10.23721557	10.01063039	protein_coding	3	100082275	100120283	TOMM70A
ENSG00000154175	6.509364082	7.926295939	protein_coding	3	100468000	100712359	ABI3BP
ENSG00000154217	6.560798025	6.59664425	protein_coding	17	65373924	65689645	PITPNC1
ENSG00000154222	8.453889713	8.942603846	protein_coding	1	52811395	52831865	CC2D1B
ENSG00000154229	8.523597116	8.338926518	protein_coding	17	64298944	64801114	PRKCA
ENSG00000154237	8.261346552	8.371803145	protein_coding	15	101459420	101610317	LRRK1
ENSG00000154240	5.806547963	6.429738366	protein_coding	17	63631656	64188194	CEP112
ENSG00000154252	3.871730003	3.714580548	protein_coding	2	242716240	242743623	GAL3ST2
ENSG00000154258	0	1.16600992	protein_coding	17	66970629	67057136	ABCA9
ENSG00000154262	0	0.390640832	protein_coding	17	67074847	67138015	ABCA6
ENSG00000154263	3.335220907	4.15321211	protein_coding	17	67143355	67240987	ABCA10
ENSG00000154265	5.785082014	7.73528765	protein_coding	17	67240576	67323323	ABCA5

ENSG00000154274	2.762599152	2.912743273	protein_coding	4	37455563	37625117	C4orf19
ENSG00000154277	4.999881834	5.137803333	protein_coding	4	41258430	41270472	UCHL1
ENSG00000154305	9.608983025	9.771851712	protein_coding	1	222791428	222841354	MIA3
ENSG00000154309	5.191549282	5.656642807	protein_coding	1	222988406	223179337	DISP1
ENSG00000154310	7.925957035	7.69255166	protein_coding	3	170779128	171178197	TNIK
ENSG00000154316	0.869158192	1.518964905	pseudogene	8	11197146	11225961	TDH
ENSG00000154319	7.202688343	6.930376707	protein_coding	8	11278972	11332224	FAM167A
ENSG00000154328	8.223944437	7.825075067	protein_coding	8	11627144	11644855	NEIL2
ENSG00000154330	0.869158192	0	protein_coding	9	70971815	71145977	PGM5
ENSG00000154342	1.407729925	0.390640832	protein_coding	1	228194752	228248961	WNT3A
ENSG00000154358	9.812996626	9.691658363	protein_coding	1	228395831	228566575	OBSCN
ENSG00000154359	6.793601635	7.210344614	protein_coding	8	12579403	12613582	LONRF1
ENSG00000154370	8.900704842	8.574870331	protein_coding	1	228581377	228594541	TRIM11
ENSG00000154380	11.6731518	11.24454145	protein_coding	1	225677502	225840844	ENAH
ENSG00000154415	0	0.390640832	protein_coding	7	113516832	113715975	PPP1R3A
ENSG00000154429	7.811178326	8.313767852	protein_coding	1	229456758	229479041	C1orf96
ENSG00000154447	8.431612753	8.434094156	protein_coding	4	170015407	170192256	SH3RF1
ENSG00000154451	8.077121473	8.682523569	protein_coding	1	89724633	89738544	GBP5
ENSG00000154473	10.57560642	11.09176185	protein_coding	10	124913793	124924886	BUB3
ENSG00000154479	1.799000381	2.144285137	protein_coding	2	170501935	170550943	C2orf77
ENSG00000154485	0.869158192	1.518964905	protein_coding	10	127455022	127464390	MMP21
ENSG00000154493	3.603959378	2.912743273	protein_coding	10	128113566	128359079	C10orf90
ENSG00000154511	7.285281023	8.17474298	protein_coding	1	93307724	93427079	FAM69A
ENSG00000154518	10.2915093	10.24050771	protein_coding	2	176040986	176049335	ATP5G3
ENSG00000154529	0.869158192	0	protein_coding	9	43684902	43924049	CNTNAP3B
ENSG00000154548	1.799000381	2.039052734	protein_coding	6	89805678	89827800	SRSF12
ENSG00000154553	2.35979773	2.652276565	protein_coding	4	186422903	186456766	PDLIM3
ENSG00000154556	2.35979773	2.652276565	protein_coding	4	186506598	186877806	SORBS2
ENSG00000154582	8.81194861	8.5924114	protein_coding	8	74857354	74884522	TCEB1
ENSG00000154589	3.004694206	4.075511708	protein_coding	8	74903587	74941322	LY96
ENSG00000154608	2.238690726	2.334191469	pseudogene	4	119388744	119477279	CEP170P1
ENSG00000154620	0	0.697730409	protein_coding	Y	15815447	15817904	TMSB4Y
ENSG00000154639	7.886184801	8.091661081	protein_coding	21	18884700	18965897	CXADR
ENSG00000154640	7.556308086	8.390640789	protein_coding	21	18965971	18985265	BTG3
ENSG00000154642	6.96088487	7.688207601	protein_coding	21	19161284	19191703	C21orf91
ENSG00000154645	0.869158192	0.697730409	protein_coding	21	19165801	19639690	CHODL
ENSG00000154646	1.16343121	6.577981353	protein_coding	21	19641433	19858197	TMPRSS15
ENSG00000154655	2.762599152	0.950786998	protein_coding	18	5954705	6414910	L3MBTL4
ENSG00000154710	3.744048221	3.081239798	protein_coding	7	66205720	66276423	RABGEF1
ENSG00000154719	7.587643674	8.058387721	protein_coding	21	26957968	26979829	MRPL39
ENSG00000154721	1.16343121	1.802319292	protein_coding	21	27011584	27089874	JAM2
ENSG00000154723	8.391336806	8.853271458	protein_coding	21	27088815	27107984	ATP5J
ENSG00000154727	7.680671792	7.743685008	protein_coding	21	27106881	27144771	GABPA
ENSG00000154734	1.616589159	0	protein_coding	21	28208606	28217728	ADAMTS1
ENSG00000154736	0.869158192	0	protein_coding	21	28290231	28338832	ADAMTS5
ENSG00000154743	7.530740159	7.621426466	protein_coding	3	12525931	12581122	TSEN2
ENSG00000154760	8.705994399	7.252146121	protein_coding	17	33762115	33775856	SLFN13
ENSG00000154767	8.223944437	8.282410365	protein_coding	3	14186647	14220283	XPC
ENSG00000154781	6.820228284	6.963107483	protein_coding	3	14693271	14714166	C3orf19
ENSG00000154783	0	0.950786998	protein_coding	3	14860469	14975895	FGD5
ENSG00000154803	9.563214301	10.68152763	protein_coding	17	17115526	17140502	FLCN
ENSG00000154813	7.440941622	7.774746834	protein_coding	3	16299485	16306479	DPH3
ENSG00000154814	6.851544845	7.138927266	protein_coding	3	16306706	16378822	OXNAD1
ENSG00000154822	0.869158192	0	protein_coding	3	16844159	17132094	PLCL2
ENSG00000154832	8.978417134	9.29944532	protein_coding	18	47808713	47814692	CXXC1
ENSG00000154839	7.985986001	8.013952351	protein_coding	18	47901365	47920543	SKA1
ENSG00000154845	9.877806407	9.528108569	protein_coding	18	9546789	9614567	PPP4R1
ENSG00000154864	0.499066092	0.697730409	protein_coding	18	10670238	11148761	PIEZO2
ENSG00000154874	2.928422289	3.935919592	processed_transc	17	18431900	18528930	CCDC144B
ENSG00000154889	5.986594255	5.863430791	protein_coding	18	11883470	11908779	MPPE1
ENSG00000154898	1.799000381	1.353254395	protein_coding	17	20224477	20305504	CCDC144C
ENSG00000154914	7.930852348	7.795089291	protein_coding	17	9548950	9633001	USP43
ENSG00000154917	5.858855738	6.2290532	protein_coding	3	133543083	133614680	RAB6B

ENSG00000154920	6.687686437	6.923001245	protein_coding	17	48450581	48458820	EME1
ENSG00000154928	0.499066092	0	protein_coding	3	134316643	134979309	EPHB1
ENSG00000154930	3.744048221	3.935919592	protein_coding	20	24986868	25039616	ACSS1
ENSG00000154945	9.141440458	8.625731057	protein_coding	17	48770553	48785285	ANKRD40
ENSG00000154957	6.236372259	6.815578497	protein_coding	17	11880762	11900785	ZNF18
ENSG00000154978	10.02699163	10.01193453	protein_coding	7	55503749	55640681	VOPP1
ENSG00000154999	0	0.950786998	pseudogene	1	45457506	45457895	
ENSG00000155008	7.618313091	7.830998152	protein_coding	X	84258832	84344972	APOOL
ENSG00000155011	0.499066092	0	protein_coding	4	107842959	108204963	DKK2
ENSG00000155016	4.517578978	4.810179682	protein_coding	4	108852525	108874613	CYP2U1
ENSG00000155026	0	1.16600992	protein_coding	7	5965177	6010314	RSPH10B
ENSG00000155034	8.360827871	7.745776733	protein_coding	7	5470966	5553429	FBXL18
ENSG00000155066	9.806352596	5.496926774	protein_coding	2	95940201	95957056	PROM2
ENSG00000155070	1.960915222	1.667587519	pseudogene	7	6895116	6899461	UNC93B2
ENSG00000155085	2.762599152	3.081239798	protein_coding	6	109814059	110012420	AKD1
ENSG00000155090	9.557686483	8.948064416	protein_coding	8	103661007	103668130	KLF10
ENSG00000155093	2.238690726	1.802319292	protein_coding	7	157331750	158380480	PTPRN2
ENSG00000155096	11.13130637	10.73410026	protein_coding	8	103838585	103906092	AZIN1
ENSG00000155097	9.178957949	9.230913712	protein_coding	8	104033291	104085279	ATP6V1C1
ENSG00000155099	5.977159882	6.002883551	protein_coding	8	92006024	92053292	TMEM55A
ENSG00000155100	7.246540262	6.716673044	protein_coding	8	92082424	92099323	OTUD6B
ENSG00000155111	7.556308086	7.929979971	protein_coding	6	110931181	111137161	CDK19
ENSG00000155115	7.808521034	7.999999944	protein_coding	6	111279763	111289093	GTF3C6
ENSG00000155130	11.675888	11.69333284	protein_coding	6	114178541	114184648	MARCKS
ENSG00000155158	3.989024711	3.608232228	protein_coding	9	15170843	15307358	TTC39B
ENSG00000155189	8.75235931	8.869669013	protein_coding	8	6565878	6617184	AGPAT5
ENSG00000155229	10.47906555	10.52195341	protein_coding	10	99218081	99258551	MMS19
ENSG00000155252	8.884394801	9.084062912	protein_coding	10	99344131	99436191	PI4K2A
ENSG00000155254	9.354994343	9.555196965	protein_coding	10	99472930	99484694	MARVELD1
ENSG00000155256	7.947856304	8.254769334	protein_coding	10	99496878	99520663	ZFYVE27
ENSG00000155265	5.625150983	5.339632724	protein_coding	10	99609996	99627782	GOLGA7B
ENSG00000155269	3.077135474	0.950786998	protein_coding	4	8560452	8621486	GPR78
ENSG00000155275	6.897280837	6.815578497	protein_coding	4	8437867	8495258	METTL19
ENSG00000155287	8.239811039	8.478794222	protein_coding	10	101370282	101380535	SLC25A28
ENSG00000155304	9.24628631	9.572977394	protein_coding	21	15743436	15755805	HSPA13
ENSG00000155307	0.499066092	0	protein_coding	21	15857549	15955723	SAMSN1
ENSG00000155313	8.688758029	8.669360359	protein_coding	21	17102344	17252377	USP25
ENSG00000155324	7.911170638	7.309836726	protein_coding	5	125695824	125832186	GRAMD3
ENSG00000155329	6.350376764	6.677762549	protein_coding	5	132332677	132362296	ZCCHC10
ENSG00000155330	6.872051157	7.18590274	protein_coding	16	46830519	46865323	C16orf87
ENSG00000155363	9.656303037	9.746483542	protein_coding	1	113215763	113243368	MOV10
ENSG00000155366	10.32629262	10.75631725	protein_coding	1	113243728	113250056	RHOC
ENSG00000155367	5.525114348	4.226940739	protein_coding	1	113245236	113258099	PPM1J
ENSG00000155368	9.631710027	9.291591823	protein_coding	2	120124497	120130126	DBI
ENSG00000155380	11.04391695	11.12066061	protein_coding	1	113454469	113499635	SLC16A1
ENSG00000155393	8.513842764	8.995422414	protein_coding	16	50099852	50140298	HEATR3
ENSG00000155428	3.077135474	2.579085888	protein_coding	7	72430016	72439997	TRIM74
ENSG00000155438	8.78516728	8.993663261	protein_coding	2	122484521	122494499	MKI67IP
ENSG00000155463	10.43063729	10.59076954	protein_coding	14	23235731	23241007	OXA1L
ENSG00000155465	7.066972635	6.952279523	protein_coding	14	23242431	23299029	SLC7A7
ENSG00000155506	11.83841494	11.4896766	protein_coding	5	154092462	154197167	LARP1
ENSG00000155508	8.803966282	9.042126408	protein_coding	5	154237113	154256353	CNOT8
ENSG00000155530	3.502389126	2.912743273	protein_coding	7	133812052	133949343	LRGUK
ENSG00000155542	6.023726639	5.908941696	protein_coding	5	56205087	56221359	C5orf35
ENSG00000155545	7.46813618	7.287036562	protein_coding	5	56215429	56267502	MIER3
ENSG00000155561	10.89060849	10.53319908	protein_coding	7	135242667	135333505	NUP205
ENSG00000155592	5.785082014	5.931169853	protein_coding	16	25247322	25269252	ZKSCAN2
ENSG00000155621	6.687686437	6.505763096	protein_coding	9	74526426	74600970	C9orf85
ENSG00000155629	3.211941663	0.950786998	protein_coding	10	98353069	98480271	PIK3AP1
ENSG00000155636	5.672682881	5.908941696	protein_coding	2	178977151	179001531	RBM45
ENSG00000155640	6.92698535	7.005624493	protein_coding	10	98741041	98745582	C10orf12
ENSG00000155657	6.62865285	6.819555981	protein_coding	2	179390716	179695529	TTN
ENSG00000155660	12.27147725	11.96673398	protein_coding	7	148700154	148725733	PDIA4

ENSG00000155666	4.491309013	5.555337277	protein_coding	16	27214807	27233089	JMJD5
ENSG00000155714	0.499066092	0.697730409	protein_coding	16	21995186	22012433	PDZD9
ENSG00000155729	7.058051099	6.941369681	protein_coding	2	201353675	201384507	KCTD18
ENSG00000155744	5.550781289	5.456629825	protein_coding	2	201843216	201936394	FAM126B
ENSG00000155749	2.671945279	2.721932731	protein_coding	2	202152994	202222121	ALS2CR12
ENSG00000155754	4.491309013	4.528525526	protein_coding	2	202352148	202483901	ALS2CR11
ENSG00000155755	7.517784241	7.681666888	protein_coding	2	202484907	202508293	TMEM237
ENSG00000155760	6.887241849	6.485878328	protein_coding	2	202899310	202903160	FZD7
ENSG00000155761	3.744048221	4.508954154	protein_coding	1	118496484	118727846	SPAG17
ENSG00000155792	6.155035276	7.343375265	protein_coding	8	120885957	121063152	DEPTOR
ENSG00000155827	9.020358234	9.214393111	protein_coding	9	104296133	104325622	RNF20
ENSG00000155846	7.990682129	6.99159092	protein_coding	5	149109861	149234585	PPARGC1B
ENSG00000155849	0	0.390640832	protein_coding	7	36893961	37488852	ELMO1
ENSG00000155850	7.993024472	8.899098923	protein_coding	5	149340300	149373018	SLC26A2
ENSG00000155858	6.610461229	6.699508919	protein_coding	5	157170703	157187717	LSM11
ENSG00000155868	5.729976786	6.445267191	protein_coding	5	156564423	156586030	MED7
ENSG00000155875	0	0.697730409	protein_coding	9	18927656	19033251	FAM154A
ENSG00000155876	8.448779323	8.917772241	protein_coding	9	19049372	19051019	RRAGA
ENSG00000155893	5.107569429	5.855704078	protein_coding	3	140947568	141013748	ACPL2
ENSG00000155903	7.660183591	7.892709342	protein_coding	3	141205889	141334184	RASA2
ENSG00000155906	7.049074049	7.315480906	protein_coding	6	151725989	151773259	RMND1
ENSG00000155918	4.19837882	4.202780776	protein_coding	6	150339470	150346668	RAET1L
ENSG00000155926	1.16343121	0.390640832	protein_coding	8	134048973	134115298	SLA
ENSG00000155957	5.036678094	5.383282767	protein_coding	12	66517709	66563852	TMBIM4
ENSG00000155959	9.061114416	9.33252778	protein_coding	X	154425284	154468098	VBP1
ENSG00000155962	0	0.697730409	protein_coding	X	154505500	154563966	CLIC2
ENSG00000155974	4.734682531	5.638744877	protein_coding	12	66741211	67197966	GRIP1
ENSG00000155975	7.990682129	8.527023921	protein_coding	8	17104080	17159936	VPS37A
ENSG00000155980	2.928422289	3.32456471	protein_coding	12	57943781	57980415	KIF5A
ENSG00000155984	6.496213887	6.365895578	protein_coding	X	148678216	148713568	TMEM185A
ENSG00000156006	0.499066092	0.697730409	protein_coding	8	18248755	18258728	NAT2
ENSG00000156009	1.16343121	3.64455972	protein_coding	X	149009941	149014609	MAGEA8
ENSG00000156011	8.803966282	8.176294739	protein_coding	8	18384811	18942240	PSD3
ENSG00000156017	8.101206377	8.053329862	protein_coding	9	77595936	77643339	C9orf41
ENSG00000156026	9.144604451	8.555921755	protein_coding	10	74451889	74647452	MCU
ENSG00000156030	10.74360174	10.4234682	protein_coding	14	74181825	74256988	C14orf43
ENSG00000156042	2.762599152	3.532712221	protein_coding	10	75013517	75119452	TTC18
ENSG00000156049	0.499066092	0.697730409	protein_coding	9	80037995	80263223	GNA14
ENSG00000156050	5.018397273	5.593003837	protein_coding	14	74398204	74417117	FAM161B
ENSG00000156052	9.611271904	9.557580388	protein_coding	9	80331003	80646374	GNAQ
ENSG00000156097	0.499066092	0.950786998	protein_coding	1	110082494	110091028	GPR61
ENSG00000156103	0.869158192	0	protein_coding	8	89044237	89340254	MMP16
ENSG00000156110	9.410371954	9.245030827	protein_coding	10	75910960	76469061	ADK
ENSG00000156113	0.869158192	0.697730409	protein_coding	10	78637355	79398353	KCNMA1
ENSG00000156127	0.869158192	0	protein_coding	14	75988768	76013358	BATF
ENSG00000156136	7.292905885	7.558560655	protein_coding	4	71858255	71896631	DCK
ENSG00000156140	0.869158192	1.353254395	protein_coding	4	73146686	73434516	ADAMTS3
ENSG00000156150	0	0.390640832	protein_coding	1	110602616	110613234	ALX3
ENSG00000156162	7.68357507	7.637287497	protein_coding	8	95731931	95806064	DPY19L4
ENSG00000156170	6.509364082	6.988061092	protein_coding	8	95907995	96128683	C8orf38
ENSG00000156171	7.222786417	7.485338407	protein_coding	1	111659955	111682838	DRAM2
ENSG00000156172	4.821592551	5.163066621	protein_coding	8	96257147	96281429	C8orf37
ENSG00000156194	0	0.390640832	protein_coding	4	76781020	76823724	PPEF2
ENSG00000156206	2.35979773	2.242360793	protein_coding	15	81299374	81441516	C15orf26
ENSG00000156219	0	0.697730409	protein_coding	4	76932337	77033955	ART3
ENSG00000156222	3.502389126	2.971122874	protein_coding	15	85427885	85518876	SLC28A1
ENSG00000156232	7.277615649	7.164171052	protein_coding	15	83477973	83503611	WHAMM
ENSG00000156239	5.613019223	5.988823159	protein_coding	21	30244513	30257693	N6AMT1
ENSG00000156253	6.567100227	6.729413443	protein_coding	21	30376705	30391699	RWDD2B
ENSG00000156256	8.80263159	8.979512278	protein_coding	21	30396950	30426809	USP16
ENSG00000156261	10.69227275	10.90504292	protein_coding	21	30428126	30446118	CCT8
ENSG00000156265	6.068837212	6.775188634	protein_coding	21	30449792	30548210	C21orf7
ENSG00000156273	8.731468881	8.956216712	protein_coding	21	30566392	31009660	BACH1

ENSG00000156298	0	1.16600992	protein_coding	X		38420623	38548169	TSPAN7
ENSG00000156299	7.945439403	7.572769481	protein_coding		21	32361860	32932290	TIAM1
ENSG00000156304	9.25411951	9.15109385	protein_coding		21	33043313	33104431	SCAF4
ENSG00000156313	6.716321419	7.260948155	protein_coding	X		38128424	38186817	RPGR
ENSG00000156345	7.04004079	7.436965343	protein_coding		9	90581356	90589695	CDK20
ENSG00000156374	7.478203418	8.078443681	protein_coding		10	105062553	105110891	PCGF6
ENSG00000156381	7.007971391	5.759604001	protein_coding		14	102973179	102976136	ANKRD9
ENSG00000156384	6.379315619	6.587342979	protein_coding		10	105881816	105886143	SFR1
ENSG00000156398	6.023726639	6.831423059	protein_coding		10	104474297	104498951	SFXN2
ENSG00000156411	9.406862641	9.13522434	protein_coding		14	104378625	104394606	C14orf2
ENSG00000156413	1.616589159	0.697730409	protein_coding		19	5830621	5839742	FUT6
ENSG00000156414	10.52995925	10.074019	protein_coding		14	104394799	104519004	TDRD9
ENSG00000156427	1.407729925	2.039052734	protein_coding		5	170846660	170884627	FGF18
ENSG00000156453	9.985833897	4.489113623	protein_coding		5	141232938	141258811	PCDH1
ENSG00000156463	9.099664364	9.008548131	protein_coding		5	145316142	145461354	SH3RF2
ENSG00000156467	8.833021386	9.15896362	protein_coding		8	97238148	97247862	UQCRB
ENSG00000156469	7.491518048	7.584504199	protein_coding		8	97251626	97273838	MTERFD1
ENSG00000156471	9.873363279	9.731256878	protein_coding		8	97273943	97349223	PTDSS1
ENSG00000156482	11.14900701	11.30625618	protein_coding		8	99037079	99058697	RPL30
ENSG00000156486	0.869158192	0.697730409	protein_coding		8	99439250	99445076	KCNS2
ENSG00000156500	6.068837212	5.647721597	protein_coding	X		133930819	133988640	FAM122C
ENSG00000156502	9.043142548	9.038718229	protein_coding		10	70939988	70968855	SUPV3L1
ENSG00000156504	9.468760142	9.511607983	protein_coding	X		133903596	133931262	FAM122B
ENSG00000156508	12.02923891	12.3668652	protein_coding		6	74225473	74233520	EEF1A1
ENSG00000156509	3.950976309	4.94736961	protein_coding		8	101145588	101158028	FBXO43
ENSG00000156510	9.427791605	9.397831074	protein_coding		10	70980059	71027315	HKDC1
ENSG00000156515	11.69183672	11.29362229	protein_coding		10	71029740	71161638	HK1
ENSG00000156521	8.855079001	8.166959047	protein_coding		10	71897737	71906432	TYSND1
ENSG00000156531	9.459473748	9.6397377	protein_coding	X		133507283	133562820	PHF6
ENSG00000156535	9.6309582	9.389159064	protein_coding		6	74405508	74538040	CD109
ENSG00000156574	3.004694206	2.039052734	protein_coding		10	72192071	72207707	NODAL
ENSG00000156587	9.690621167	10.31036334	protein_coding		11	57319129	57335757	UBE2L6
ENSG00000156599	10.68069471	10.69295559	protein_coding		11	57435219	57468659	ZDHHC5
ENSG00000156603	7.1740722	7.586839732	protein_coding		11	57471186	57479693	MED19
ENSG00000156639	9.976394534	9.89664616	protein_coding		6	37787275	38122400	ZFAND3
ENSG00000156642	9.784207642	10.0814915	protein_coding		15	73852355	73926475	NPTN
ENSG00000156650	8.000028772	7.586839732	protein_coding		10	76585340	76792380	KAT6B
ENSG00000156671	8.314756163	7.619146302	protein_coding		10	76859344	76941881	SAMD8
ENSG00000156675	9.290757201	8.381252713	protein_coding		8	37716136	37756985	RAB11FIP1
ENSG00000156697	9.14670993	9.338084052	protein_coding	X		129040097	129063737	UTP14A
ENSG00000156709	10.12368915	10.49888563	protein_coding	X		129263337	129299861	AIFM1
ENSG00000156711	7.734859118	5.96747225	protein_coding		6	36095586	36107842	MAPK13
ENSG00000156735	7.481543613	7.454976247	protein_coding		8	38034051	38070819	BAG4
ENSG00000156750	2.762599152	0.390640832	pseudogene		9	42858072	42876505	AQP7P3
ENSG00000156755	0	0.390640832	pseudogene		9	69777099	69777956	
ENSG00000156787	7.868433623	7.477807561	protein_coding		8	124054208	124164393	WDR67
ENSG00000156795	6.442380648	5.638744877	protein_coding		8	124428965	124479470	WDYHV1
ENSG00000156802	9.46791839	9.576507334	protein_coding		8	124332090	124428590	ATAD2
ENSG00000156804	9.649637321	8.103127799	protein_coding		8	124510129	124553446	FBXO32
ENSG00000156831	6.710639711	6.858739095	protein_coding		8	126103921	126379362	NSMCE2
ENSG00000156853	7.381392865	7.714077892	protein_coding		16	30613879	30635333	ZNF689
ENSG00000156858	9.062230259	9.421577335	protein_coding		16	30662038	30667761	PRR14
ENSG00000156860	10.43667292	10.32617591	protein_coding		16	30669752	30682131	FBRS
ENSG00000156869	6.400645076	5.545764946	protein_coding		1	100174259	100232187	FRRS1
ENSG00000156873	8.398422578	8.454700467	protein_coding		16	30759591	30772490	PHKG2
ENSG00000156875	8.662511098	8.607443034	protein_coding		1	100503653	100548933	HIAT1
ENSG00000156876	7.071412799	7.41347308	protein_coding		1	100549119	100598511	SASS6
ENSG00000156886	1.799000381	2.242360793	protein_coding		16	31404597	31437826	ITGAD
ENSG00000156920	0.499066092	0	protein_coding	X		135383122	135519215	GPR112
ENSG00000156928	8.288298501	8.26792849	protein_coding		7	23338358	23351348	C7orf30
ENSG00000156931	7.873527714	8.162268448	protein_coding		3	184529931	184770402	VPS8
ENSG00000156958	6.782811835	6.563824016	protein_coding		15	49447853	49660066	GALK2
ENSG00000156959	0.869158192	0	protein_coding		3	9543481	9595486	LHFPL4

ENSG00000156966	7.246540262	4.604259349	protein_coding	2	232260254	232265875	B3GNT7
ENSG00000156968	0.499066092	0.697730409	protein_coding	16	15489611	15503458	MPV17L
ENSG00000156970	9.386516735	9.280091692	protein_coding	15	40453224	40513337	BUB1B
ENSG00000156973	7.876068031	7.968104748	protein_coding	2	232597135	232650982	PDE6D
ENSG00000156976	10.89217803	11.38066227	protein_coding	3	186500994	186507689	EIF4A2
ENSG00000156983	8.602416377	8.149685112	protein_coding	3	9773413	9789702	BRPF1
ENSG00000156990	8.320363082	8.43019749	protein_coding	3	9879533	9886286	RPUSD3
ENSG00000157014	9.272554655	8.916844293	protein_coding	3	10289707	10327480	TATDN2
ENSG00000157017	3.603959378	3.570966319	protein_coding	3	10327359	10334631	GHRL
ENSG00000157020	10.22231885	10.29187254	protein_coding	3	10334815	10362862	SEC13
ENSG00000157021	0.499066092	0.390640832	pseudogene	15	41455322	41456695	FAM92A1P1
ENSG00000157036	6.313367288	6.695185789	protein_coding	3	38537618	38583652	EXOG
ENSG00000157045	7.978913023	8.006993014	protein_coding	16	15131710	15149921	NTAN1
ENSG00000157060	2.238690726	1.667587519	protein_coding	1	182869000	182922660	SHCBP1L
ENSG00000157064	6.041940478	4.363974406	protein_coding	1	183217372	183387737	NMNAT2
ENSG00000157077	7.348842929	7.612284109	protein_coding	1	52608046	52812358	ZFYVE9
ENSG00000157087	0.499066092	0	protein_coding	3	10365707	10749716	ATP2B2
ENSG00000157106	10.78034131	10.93353768	protein_coding	16	18816175	18937776	SMG1
ENSG00000157107	7.406211319	7.266786485	protein_coding	5	72251808	72386349	FCHO2
ENSG00000157110	7.64536895	8.226588358	protein_coding	8	30241944	30429778	RBPMS
ENSG00000157111	5.375152132	4.202780776	protein_coding	5	72416119	72427644	TMEM171
ENSG00000157150	5.795854912	3.935919592	protein_coding	3	12194551	12200851	TIMP4
ENSG00000157152	2.671945279	1.802319292	polymorphic_pse	3	12045862	12232907	SYN2
ENSG00000157168	8.327805238	7.819127564	protein_coding	8	31496902	32622548	NRG1
ENSG00000157181	7.416718478	7.825075067	protein_coding	1	186344890	186390507	C1orf27
ENSG00000157184	7.430610036	7.164171052	protein_coding	1	53662101	53679869	CPT2
ENSG00000157191	9.045401313	9.223427792	protein_coding	1	16767167	16786573	NECAP2
ENSG00000157193	8.620709081	8.338926518	protein_coding	1	53711217	53793742	LRP8
ENSG00000157211	0.499066092	0.390640832	protein_coding	1	54598747	54619443	CDCP2
ENSG00000157212	8.546104025	8.409235632	protein_coding	7	154735397	154794794	PAXIP1
ENSG00000157214	7.913645586	7.116470702	protein_coding	7	89796904	89870091	STEAP2
ENSG00000157216	7.409722215	7.541804862	protein_coding	1	54692190	54879152	SSBP3
ENSG00000157224	8.847332411	8.737164829	protein_coding	7	90013035	90142716	CLDN12
ENSG00000157227	12.72863472	12.68052698	protein_coding	14	23305766	23318236	MMP14
ENSG00000157240	6.917151646	6.45552765	protein_coding	7	90893783	90898123	FZD1
ENSG00000157259	8.368064704	8.088368066	protein_coding	7	92076767	92088150	GATAD1
ENSG00000157303	6.897280837	5.372493443	protein_coding	9	95820989	95847420	SUSD3
ENSG00000157306	2.471521042	3.993387124	processed_transc	14	23980969	24029987	
ENSG00000157315	2.928422289	2.420525079	protein_coding	16	69377151	69385712	TMED6
ENSG00000157322	1.16343121	0.950786998	protein_coding	16	69984810	69998141	CLEC18A
ENSG00000157326	6.204385613	6.733635362	protein_coding	14	24422795	24438488	DHRS4
ENSG00000157335	1.16343121	0.390640832	protein_coding	16	70207225	70221264	CLEC18C
ENSG00000157343	2.238690726	3.027231696	protein_coding	6	35704809	35716856	ARMC12
ENSG00000157349	7.071412799	7.033288635	protein_coding	16	70323566	70369186	DDX19B
ENSG00000157350	9.902313564	9.177677745	protein_coding	16	70413338	70473140	ST3GAL2
ENSG00000157353	7.863321481	7.862183683	protein_coding	16	70488498	70514177	FUK
ENSG00000157368	4.062237333	4.976162367	protein_coding	16	70613798	70694585	IL34
ENSG00000157379	6.442380648	7.281279771	protein_coding	14	24759804	24769039	DHRS1
ENSG00000157388	0.499066092	0	protein_coding	3	53528683	53846490	CACNA1D
ENSG00000157423	4.491309013	4.448594745	protein_coding	16	70841281	71264625	HYDIN
ENSG00000157426	6.882196036	7.170413581	protein_coding	4	57204456	57253666	AASDH
ENSG00000157429	3.004694206	4.710890357	protein_coding	16	71507493	71598992	ZNF19
ENSG00000157445	0.499066092	0.390640832	protein_coding	3	54156574	55108584	CACNA2D3
ENSG00000157450	8.440221571	8.374509343	protein_coding	15	59157374	59389618	RNF111
ENSG00000157456	9.090943324	9.079923694	protein_coding	15	59397277	59417244	CCNB2
ENSG00000157470	6.835971536	6.535085617	protein_coding	15	59664892	59815748	FAM81A
ENSG00000157483	10.1470152	9.776974532	protein_coding	15	59427113	59665099	MYO1E
ENSG00000157500	8.894453552	8.852301067	protein_coding	3	57261765	57312980	APPL1
ENSG00000157510	6.155035276	6.270323763	protein_coding	5	148651434	148721365	AFAP1L1
ENSG00000157514	8.561968095	7.572769481	protein_coding	X	106956451	107020572	TSC22D3
ENSG00000157538	8.295907473	8.253299769	protein_coding	21	38591910	38640262	DSCR3
ENSG00000157540	8.596266901	8.454700467	protein_coding	21	38739236	38887680	DYRK1A
ENSG00000157551	0.499066092	0.390640832	protein_coding	21	39529128	39673748	KCNJ15

ENSG00000157557	9.969274259	9.445582628	protein_coding	21	40177231	40196879	ETS2
ENSG00000157570	2.671945279	2.144285137	protein_coding	11	44748015	44953972	TSPAN18
ENSG00000157578	3.950976309	4.547834947	protein_coding	21	40777770	40817731	LCA5L
ENSG00000157593	9.471282455	9.547423533	protein_coding	6	44221833	44225291	SLC35B2
ENSG00000157600	9.953725374	9.780549713	protein_coding	X	109245859	109425962	TMEM164
ENSG00000157601	9.145657575	10.20787732	protein_coding	21	42792231	42831141	MX1
ENSG00000157613	10.72328876	10.44261478	protein_coding	11	46299212	46342972	CREB3L1
ENSG00000157617	8.500733514	8.631398847	protein_coding	21	43305221	43373999	C2CD2
ENSG00000157625	10.16010253	9.762067953	protein_coding	X	30845559	30993201	TAB3
ENSG00000157637	10.02011733	10.28325773	protein_coding	17	79218800	79269347	SLC38A10
ENSG00000157653	3.78787645	4.021284656	protein_coding	9	116172334	116191964	C9orf43
ENSG00000157654	1.407729925	1.353254395	protein_coding	9	112542589	112934792	PALM2-AKAP2
ENSG00000157657	8.349904097	7.497803111	protein_coding	9	116638562	116818871	ZNF618
ENSG00000157680	4.19837882	3.64455972	protein_coding	7	137073563	137531838	DGKI
ENSG00000157693	8.179389493	8.242970684	protein_coding	9	117373486	117408702	C9orf91
ENSG00000157734	3.830412367	4.604259349	protein_coding	15	64443914	64449680	SNX22
ENSG00000157741	8.484177644	8.352716275	protein_coding	7	138915102	138992981	UBN2
ENSG00000157764	7.289098491	6.827478203	protein_coding	7	140424943	140624564	BRAF
ENSG00000157765	6.121171528	5.22435463	protein_coding	4	25656923	25680370	SLC34A2
ENSG00000157766	0	0.390640832	protein_coding	15	89346674	89418585	ACAN
ENSG00000157778	8.677151638	8.752849683	protein_coding	7	1606966	1610641	PSMG3
ENSG00000157782	0.499066092	0.390640832	protein_coding	12	121078355	121105127	CABP1
ENSG00000157796	7.615275323	8.386624806	protein_coding	4	39184024	39287430	WDR19
ENSG00000157800	8.996041004	8.375860541	protein_coding	7	139993493	140104233	SLC37A3
ENSG00000157823	7.035502862	6.729413443	protein_coding	15	90373831	90437574	AP3S2
ENSG00000157827	10.08413733	10.02921141	protein_coding	2	153191751	153506348	FMNL2
ENSG00000157833	6.693459083	6.051043053	protein_coding	2	26395960	26412513	FAM59B
ENSG00000157837	8.697401955	8.454700467	protein_coding	12	121200313	121342174	
ENSG00000157856	0	0.390640832	protein_coding	2	26624784	26679579	CCDC164
ENSG00000157869	6.220467583	6.646756201	protein_coding	4	13362978	13485989	RAB28
ENSG00000157870	8.146099194	8.031205224	protein_coding	1	2517930	2522908	FAM213B
ENSG00000157873	6.830742851	7.57041104	protein_coding	1	2487078	2497061	TNFRSF14
ENSG00000157881	7.575190993	7.915187059	protein_coding	1	2439972	2458039	PANK4
ENSG00000157890	3.077135474	3.748356452	protein_coding	15	66187417	66546085	MEGF11
ENSG00000157895	5.301468762	4.640685763	protein_coding	12	121440243	121454305	C12orf43
ENSG00000157911	8.441937187	8.602834575	protein_coding	1	2336236	2345236	PEX10
ENSG00000157916	9.382055486	9.591123153	protein_coding	1	2323267	2335190	RER1
ENSG00000157927	7.759833327	7.292770473	protein_coding	7	4836686	4923350	RADIL
ENSG00000157933	10.22531053	9.84538481	protein_coding	1	2160134	2241558	SKI
ENSG00000157954	10.150167	10.12271748	protein_coding	7	5229819	5273457	WIPI2
ENSG00000157978	8.705994399	8.461080043	protein_coding	1	25870071	25895377	LDLRAP1
ENSG00000157985	8.717371959	8.379906558	protein_coding	2	236402733	237035198	AGAP1
ENSG00000157992	5.60078458	3.183544561	protein_coding	2	27665233	27669348	KRTCAP3
ENSG00000157999	4.261916566	3.608232228	protein_coding	7	6071007	6076017	ANKRD61
ENSG00000158006	6.979904289	7.10998981	protein_coding	1	26286258	26324648	PAFAH2
ENSG00000158008	1.616589159	1.667587519	protein_coding	1	26346375	26362955	EXTL1
ENSG00000158014	0.499066092	0	protein_coding	1	26363743	26372624	SLC30A2
ENSG00000158019	8.474152153	8.323603978	protein_coding	2	28112808	28561768	BRE
ENSG00000158023	7.587643674	7.20731193	protein_coding	12	122355768	122441833	WDR66
ENSG00000158042	9.129779423	10.06273716	protein_coding	11	6702013	6704632	MRPL17
ENSG00000158050	8.405473719	6.633261019	protein_coding	2	96808905	96811179	DUSP2
ENSG00000158055	0.499066092	0	protein_coding	1	24645812	24690972	GRHL3
ENSG00000158062	7.945439403	8.38930337	protein_coding	1	26607819	26644854	UBXN11
ENSG00000158079	7.440941622	7.607691112	protein_coding	9	96793076	96872138	PTPDC1
ENSG00000158089	0.499066092	0	protein_coding	2	31133333	31378068	GALNT14
ENSG00000158092	7.654275977	8.068450553	protein_coding	3	136581050	136668665	NCK1
ENSG00000158104	4.437288965	3.133298822	protein_coding	12	122277433	122326517	HPD
ENSG00000158106	7.603059879	7.749951106	protein_coding	8	144451057	144466390	RHPN1
ENSG00000158109	7.886184801	7.641787375	protein_coding	1	3541566	3546691	TPRG1L
ENSG00000158113	0.869158192	1.802319292	protein_coding	12	122652285	122688018	LRRC43
ENSG00000158122	7.660183591	7.012590419	protein_coding	9	99401859	99417599	C9orf21
ENSG00000158125	7.062518763	4.585695337	protein_coding	2	31557187	31637581	XDH
ENSG00000158156	6.598204782	6.673373662	protein_coding	1	28285973	28294607	XKR8

ENSG00000158158	7.886184801	6.977419495	protein_coding	2	97426639	97477628	CNNM4
ENSG00000158161	8.495786671	8.286726732	protein_coding	1	28296855	28415207	EYA3
ENSG00000158163	6.095241663	6.304782526	protein_coding	3	137780832	137834660	DZIP1L
ENSG00000158169	8.610066544	8.519708104	protein_coding	9	97861336	98079991	FANCC
ENSG00000158186	8.233881494	8.641545092	protein_coding	3	138066539	138124375	MRAS
ENSG00000158195	10.81252605	10.43388431	protein_coding	1	27730730	27816669	WASF2
ENSG00000158201	7.498129565	6.811590016	protein_coding	18	19230858	19284766	ABHD3
ENSG00000158220	5.707331524	6.123616685	protein_coding	3	138153428	138200528	ESYT3
ENSG00000158234	7.0218028	7.572769481	protein_coding	3	138327448	138352218	FAIM
ENSG00000158246	7.35977471	7.400254612	protein_coding	1	27331511	27339327	FAM46B
ENSG00000158258	0.499066092	1.353254395	protein_coding	3	139654027	140286919	CLSTN2
ENSG00000158270	0	0.390640832	protein_coding	18	319355	500729	COLEC12
ENSG00000158286	8.462367008	8.513583123	protein_coding	1	6265535	6281359	RNF207
ENSG00000158290	10.38988939	10.52408771	protein_coding	X	119658464	119709649	CUL4B
ENSG00000158292	7.627388205	5.9163892	protein_coding	1	6307406	6321035	GPR153
ENSG00000158296	8.510576602	8.428896259	protein_coding	20	45186463	45304714	SLC13A3
ENSG00000158301	5.485736881	5.236305879	protein_coding	X	101967257	101973607	GPRASP2
ENSG00000158315	5.090170488	4.508954154	protein_coding	1	39351479	39407471	RHBDL2
ENSG00000158321	4.464551814	0	protein_coding	7	69063905	70258054	AUTS2
ENSG00000158352	5.107569429	2.242360793	protein_coding	X	50334647	50557302	SHROOM4
ENSG00000158373	6.738827102	7.329495501	protein_coding	6	26158349	26171577	HIST1H2BD
ENSG00000158402	6.861834435	7.0637896	protein_coding	5	137620954	137674044	CDC25C
ENSG00000158406	5.346128795	5.717592784	protein_coding	6	26281283	26285762	HIST1H4H
ENSG00000158411	7.461385447	7.549009758	protein_coding	2	99777890	99797521	MITD1
ENSG00000158417	11.15215447	10.90105998	protein_coding	2	99953816	100016728	EIF5B
ENSG00000158423	3.211941663	4.101879561	protein_coding	X	53449639	53458059	RIBC1
ENSG00000158427	0.499066092	0	protein_coding	X	103173479	103229570	TMSB15B
ENSG00000158428	1.16343121	0.697730409	protein_coding	2	219221579	219232822	C2orf62
ENSG00000158435	9.183066967	8.764244491	protein_coding	2	101869264	101886778	C2orf29
ENSG00000158445	2.762599152	2.912743273	protein_coding	20	47984946	48099184	KCNB1
ENSG00000158457	2.671945279	2.334191469	protein_coding	7	128784712	128808671	TSPAN33
ENSG00000158467	7.578314254	7.243290054	protein_coding	7	128864864	129070052	AHCYL2
ENSG00000158470	10.77490668	10.9365167	protein_coding	20	48249482	48330415	B4GALT5
ENSG00000158473	0.869158192	1.16600992	protein_coding	1	158149737	158154686	CD1D
ENSG00000158480	8.249639777	7.749951106	protein_coding	20	48519928	48532080	SPATA2
ENSG00000158482	1.16343121	1.16600992	pseudogene	16	21391313	21403412	SNX29P1
ENSG00000158483	5.986594255	5.611474642	protein_coding	11	71498556	71512282	FAM86C1
ENSG00000158486	4.842525946	4.65855978	protein_coding	16	20943804	21170762	DNAH3
ENSG00000158516	0.869158192	0	protein_coding	7	129906667	129929638	CPA2
ENSG00000158517	0.869158192	0.390640832	protein_coding	7	74188309	74203659	NCF1
ENSG00000158525	0	1.925536307	protein_coding	7	129984630	130008571	CPA5
ENSG00000158526	8.768856552	8.917772241	protein_coding	X	54466834	54471920	TSR2
ENSG00000158528	3.698846687	0.390640832	protein_coding	7	94536514	94925727	PPP1R9A
ENSG00000158545	10.25147099	10.06692591	protein_coding	16	88636789	88698374	ZC3H18
ENSG00000158552	7.514527001	7.614575135	protein_coding	2	220059959	220074373	ZFAND2B
ENSG00000158553	1.407729925	0.697730409	protein_coding	6	27253682	27279949	POM121L2
ENSG00000158555	8.513842764	8.40394723	protein_coding	11	75145685	75236948	GDPD5
ENSG00000158560	0.499066092	0	protein_coding	7	95401866	95739634	DYNC1I1
ENSG00000158571	2.10647801	2.420525079	protein_coding	X	54959394	55024967	PFKFB1
ENSG00000158578	0.869158192	0.950786998	protein_coding	X	55035488	55057497	ALAS2
ENSG00000158604	9.995211901	9.536590702	protein_coding	7	44617493	44621886	TMED4
ENSG00000158615	9.722025379	9.162882465	protein_coding	1	204372515	204380945	PPP1R15B
ENSG00000158623	8.396654395	7.987679908	protein_coding	7	130146089	130353598	COPG2
ENSG00000158636	8.695964893	8.944426333	protein_coding	11	76155967	76264069	C11orf30
ENSG00000158669	9.684112495	9.657067809	protein_coding	8	41434706	41482520	AGPAT6
ENSG00000158683	0.499066092	2.039052734	protein_coding	7	47814250	47988088	PKD1L1
ENSG00000158691	6.204385613	6.799557986	protein_coding	6	28346732	28367511	ZSCAN12
ENSG00000158710	13.36541129	13.9992169	protein_coding	1	159887897	159895522	TAGLN2
ENSG00000158711	9.155101256	9.204541268	protein_coding	1	205566707	205601090	ELK4
ENSG00000158714	2.10647801	3.081239798	protein_coding	1	159796540	159807039	SLAMF8
ENSG00000158715	7.406211319	6.930376707	protein_coding	1	205626979	205649587	SLC45A3
ENSG00000158716	7.413224588	7.532142019	protein_coding	1	159750722	159752333	DUSP23
ENSG00000158717	8.02999382	7.931818465	protein_coding	16	88762903	88772829	RNF166

ENSG00000158731	0.499066092	0	pseudogene	1	159568088	159569017	OR10J6P
ENSG00000158747	9.24628631	9.248723012	protein_coding	1	19967048	19984945	NBL1
ENSG00000158764	0.499066092	0	protein_coding	1	160914815	160924589	ITLN2
ENSG00000158769	10.70447344	9.335308591	protein_coding	1	160965001	161008784	F11R
ENSG00000158773	9.042011838	9.566483319	protein_coding	1	161009041	161015767	USF1
ENSG00000158792	6.74439909	6.490875274	protein_coding	16	89762751	89768113	SPATA2L
ENSG00000158793	7.797842652	8.391976969	protein_coding	1	161087876	161095235	NIT1
ENSG00000158796	8.37885234	8.369091861	protein_coding	1	161090764	161102478	DEDD
ENSG00000158805	7.959880444	7.73528765	protein_coding	16	89786808	89807311	ZNF276
ENSG00000158806	2.471521042	0.697730409	protein_coding	8	21881636	21894408	NPM2
ENSG00000158813	4.734682531	3.32456471	protein_coding	X	68835911	69259319	EDA
ENSG00000158815	0.499066092	1.16600992	protein_coding	8	21899909	21906320	FGF17
ENSG00000158825	9.204449373	8.439273392	protein_coding	1	20915441	20945401	CDA
ENSG00000158828	8.836938493	9.008548131	protein_coding	1	20959948	20978004	PINK1
ENSG00000158850	9.334821842	8.944426333	protein_coding	1	161141100	161147309	B4GALT3
ENSG00000158856	6.482942724	7.745776733	protein_coding	8	21906506	21940038	EPB49
ENSG00000158859	3.077135474	3.368666104	protein_coding	1	161159538	161168846	ADAMTS4
ENSG00000158863	9.086562954	9.011159004	protein_coding	8	21946670	21962232	FAM160B2
ENSG00000158864	10.74011984	11.04071149	protein_coding	1	161166894	161184185	NDUFS2
ENSG00000158865	3.698846687	2.652276565	protein_coding	16	24857162	24922949	SLC5A11
ENSG00000158869	2.35979773	1.802319292	protein_coding	1	161185024	161190489	FCER1G
ENSG00000158882	7.73205733	7.940975956	protein_coding	1	161195793	161200408	TOMM40L
ENSG00000158887	2.10647801	2.334191469	protein_coding	1	161274525	161279762	MPZ
ENSG00000158941	10.35161244	10.20939387	protein_coding	8	22462145	22479027	KIAA1967
ENSG00000158966	5.431503808	6.117167886	protein_coding	1	64936428	65158741	CACHD1
ENSG00000158985	8.791909364	8.673761454	protein_coding	5	130581186	130734140	CDC42SE2
ENSG00000158987	6.386460534	6.642271819	protein_coding	5	130759614	130970929	RAPGEF6
ENSG00000159023	8.494133947	8.377210474	protein_coding	1	29213603	29446553	EPB41
ENSG00000159055	7.981274537	8.071789292	protein_coding	21	33640530	33651380	MIS18A
ENSG00000159063	9.019209518	9.184633427	protein_coding	11	77811982	77850706	ALG8
ENSG00000159069	10.32070875	10.38238014	protein_coding	9	139834887	139839148	FBXW5
ENSG00000159079	8.541310587	8.855210283	protein_coding	21	33951132	33985176	C21orf59
ENSG00000159082	6.912209543	7.00911166	protein_coding	21	33997269	34100359	SYNJ1
ENSG00000159086	8.561968095	8.70742221	protein_coding	21	34106210	34144169	GCFC1
ENSG00000159110	7.119373418	7.679480043	protein_coding	21	34602206	34637969	IFNAR2
ENSG00000159111	8.655134734	8.541545191	protein_coding	17	45900638	45908900	MRPL10
ENSG00000159128	9.407740769	9.20301961	protein_coding	21	34757299	34851655	IFNGR2
ENSG00000159131	10.67158427	10.53531681	protein_coding	21	34870940	34915797	GART
ENSG00000159140	11.17708996	11.07480633	protein_coding	21	34914924	34949812	SON
ENSG00000159147	7.630400601	7.920752191	protein_coding	21	34947783	34961014	DONSON
ENSG00000159164	3.077135474	2.420525079	protein_coding	1	149874870	149889434	SV2A
ENSG00000159166	9.120167967	1.802319292	protein_coding	1	201342372	201368736	LAD1
ENSG00000159167	9.023798905	9.946603152	protein_coding	8	23699428	23712320	STC1
ENSG00000159173	0.869158192	0.390640832	protein_coding	1	201372896	201398994	TNNI1
ENSG00000159176	9.05999771	8.369091861	protein_coding	1	201452658	201478584	CSRP1
ENSG00000159197	0.499066092	1.667587519	protein_coding	21	35736323	35743440	KCNE2
ENSG00000159199	9.262881318	9.430057217	protein_coding	17	46970127	46973233	ATP5G1
ENSG00000159200	8.120617457	7.999999944	protein_coding	21	35885440	35987441	RCAN1
ENSG00000159202	10.03155637	10.15455566	protein_coding	17	46985731	47006418	UBE2Z
ENSG00000159208	6.220467583	6.365895578	protein_coding	1	150254953	150259505	C1orf51
ENSG00000159210	8.774313977	8.725553248	protein_coding	17	47006678	47022479	SNF8
ENSG00000159214	6.155035276	5.832272499	protein_coding	1	44457031	44462200	CCDC24
ENSG00000159216	9.740266097	9.452002561	protein_coding	21	36160098	37357047	RUNX1
ENSG00000159217	0.499066092	0.697730409	protein_coding	17	47074774	47133012	IGF2BP1
ENSG00000159224	0.499066092	0.697730409	protein_coding	17	47035916	47045958	GIP
ENSG00000159228	8.688758029	8.374509343	protein_coding	21	37442239	37445464	CBR1
ENSG00000159231	5.869093502	6.123616685	protein_coding	21	37507210	37518864	CBR3
ENSG00000159239	4.261916566	4.250702764	protein_coding	2	74641304	74648718	C2orf81
ENSG00000159247	4.568725997	2.039052734	pseudogene	9	141044565	141071716	TUBBP5
ENSG00000159256	7.624369505	8.278081046	protein_coding	21	37692487	37758446	MORC3
ENSG00000159259	8.02999382	8.026050991	protein_coding	21	37757676	37789125	CHAF1B
ENSG00000159261	1.407729925	2.579085888	protein_coding	21	37832919	37948867	CLDN14
ENSG00000159263	1.407729925	1.353254395	protein_coding	21	38071433	38131815	SIM2

ENSG00000159267	7.832262274	7.871792517	protein_coding	21	38123493	38362536	HLCS
ENSG00000159289	1.16343121	0.950786998	protein_coding	15	74362198	74374891	GOLGA6A
ENSG00000159307	0	0.390640832	protein_coding	22	43599229	43739394	SCUBE1
ENSG00000159314	7.803191723	7.019522872	protein_coding	17	43471275	43511787	ARHGAP27
ENSG00000159322	9.319971717	9.288725373	protein_coding	15	73043710	73078187	ADPGK
ENSG00000159335	11.83564426	11.78974728	protein_coding	12	6874682	6880118	PTMS
ENSG00000159337	2.35979773	2.039052734	protein_coding	15	42359207	42386752	PLA2G4D
ENSG00000159339	3.603959378	1.16600992	protein_coding	1	17634690	17690499	PADI4
ENSG00000159346	9.651121255	9.619917937	protein_coding	1	202909951	202927700	ADIPOR1
ENSG00000159348	7.748787017	8.426290271	protein_coding	1	202930997	202936408	CYB5R1
ENSG00000159352	10.56028737	10.93284934	protein_coding	1	151227179	151239955	PSMD4
ENSG00000159363	8.81194861	8.296748314	protein_coding	1	17312453	17338423	ATP13A2
ENSG00000159374	0.499066092	0.390640832	protein_coding	2	74785010	74875465	C2orf65
ENSG00000159377	11.53691523	12.06717193	protein_coding	1	151372010	151374420	PSMB4
ENSG00000159387	1.16343121	0.390640832	protein_coding	16	55357672	55367395	IRX6
ENSG00000159388	5.967663407	5.96747225	protein_coding	1	203274619	203278730	BTG2
ENSG00000159399	10.25293768	10.58464742	protein_coding	2	75061108	75120486	HK2
ENSG00000159403	9.034071956	9.503595441	protein_coding	12	7187513	7245203	C1R
ENSG00000159409	0.499066092	0.390640832	protein_coding	1	151674880	151689290	CELF3
ENSG00000159423	7.04456449	7.758263791	protein_coding	1	19197926	19229275	ALDH4A1
ENSG00000159433	7.106450572	7.32390599	protein_coding	15	42867857	43013179	STARD9
ENSG00000159445	7.952677993	8.265014588	protein_coding	1	151846060	151882284	THEM4
ENSG00000159450	3.911897206	3.453019579	protein_coding	1	152078793	152086556	TCHH
ENSG00000159459	8.271030151	8.075120324	protein_coding	15	43235095	43398311	UBR1
ENSG00000159461	9.791627126	10.17914289	protein_coding	16	56395364	56459450	AMFR
ENSG00000159479	8.357195795	8.744505654	protein_coding	1	43849588	43855479	MED8
ENSG00000159496	3.554067925	3.532712221	protein_coding	22	24030323	24041363	RGL4
ENSG00000159556	0.869158192	0.390640832	protein_coding	15	76629065	76634817	ISL2
ENSG00000159579	8.655134734	9.023280994	protein_coding	16	57220049	57274387	RSPRY1
ENSG00000159588	3.652182994	4.021284656	protein_coding	1	46085716	46089729	CCDC17
ENSG00000159592	9.900443107	9.793751926	protein_coding	1	46092976	46153785	GPBP1L1
ENSG00000159593	9.684837132	10.21958906	protein_coding	16	66836778	66907159	NAE1
ENSG00000159596	8.401952457	8.291030223	protein_coding	1	46152886	46160115	TMEM69
ENSG00000159618	1.616589159	1.925536307	protein_coding	16	57576333	57625593	GPR114
ENSG00000159625	0	1.802319292	protein_coding	16	57728705	57765717	CCDC135
ENSG00000159640	5.141750194	3.781359661	protein_coding	17	61554422	61599209	ACE
ENSG00000159648	3.335220907	3.781359661	protein_coding	16	58010339	58022020	TEPP
ENSG00000159650	1.407729925	1.925536307	protein_coding	3	126200124	126236616	UROC1
ENSG00000159658	9.724141885	9.399160615	protein_coding	1	47124366	47184824	KIAA0494
ENSG00000159674	3.744048221	4.101879561	protein_coding	4	1160720	1202750	SPON2
ENSG00000159685	7.437505974	7.885138265	protein_coding	3	126423063	126679249	CHCHD6
ENSG00000159692	10.3341663	10.15100875	protein_coding	4	1205236	1243741	CTBP1
ENSG00000159708	1.16343121	0.697730409	protein_coding	16	67360701	67419106	LRRC36
ENSG00000159712	2.238690726	2.579085888	processed_transc	9	99918175	99983538	ANKRD18CP
ENSG00000159713	4.19837882	4.363974406	protein_coding	16	67423712	67427438	TPPP3
ENSG00000159714	6.733233509	7.083771121	protein_coding	16	67428322	67450736	ZDHHC1
ENSG00000159720	10.13433868	10.44519147	protein_coding	16	67471917	67515140	ATP6V0D1
ENSG00000159723	0	0.390640832	protein_coding	16	67516474	67517716	AGRP
ENSG00000159733	4.543379137	5.593003837	protein_coding	4	2271309	2420390	ZFYVE28
ENSG00000159753	3.830412367	3.781359661	protein_coding	16	67679030	67691456	RLTPR
ENSG00000159761	4.352265886	4.777836593	protein_coding	16	67700719	67702660	C16orf86
ENSG00000159784	1.616589159	1.667587519	protein_coding	7	143050493	143059863	FAM131B
ENSG00000159788	7.420203928	7.4317778	protein_coding	4	3294755	3441640	RGS12
ENSG00000159792	9.012297947	8.987489278	protein_coding	16	67927175	67963581	PSKH1
ENSG00000159840	11.68840814	11.37729315	protein_coding	7	143078173	143088204	ZYX
ENSG00000159842	11.05067904	11.42865055	protein_coding	17	906758	1090616	ABR
ENSG00000159860	0.499066092	0.390640832	pseudogene	7	143333745	143515397	FAM115D
ENSG00000159871	5.286269488	3.027231696	protein_coding	19	44300080	44324808	LYPD5
ENSG00000159873	9.233465948	9.61477711	protein_coding	22	29168662	29185283	CCDC117
ENSG00000159882	5.967663407	6.252780742	protein_coding	19	44507077	44518072	ZNF230
ENSG00000159884	4.062237333	5.187895122	protein_coding	9	35658301	35661508	CCDC107
ENSG00000159885	5.91922012	5.776071827	protein_coding	19	44529494	44537260	ZNF222
ENSG00000159899	5.550781289	3.570966319	protein_coding	9	35792151	35809729	NPR2

ENSG00000159904	2.238690726	2.788380093	pseudogene	7	5160870	5184177	ZNF890P
ENSG00000159905	5.958104008	5.886365696	protein_coding	19	44455380	44471752	ZNF221
ENSG00000159915	3.871730003	4.342020395	protein_coding	19	44754318	44815771	ZNF233
ENSG00000159917	5.316509574	5.294620694	protein_coding	19	44782947	44813601	ZNF235
ENSG00000159921	9.072234198	8.330588949	protein_coding	9	36214438	36277053	GNE
ENSG00000159958	3.146112541	2.579085888	protein_coding	22	42321045	42322822	TNFRSF13C
ENSG00000160007	10.27716552	9.753257466	protein_coding	19	47421933	47508323	ARHGAP35
ENSG00000160013	1.16343121	0.697730409	protein_coding	19	47123726	47128354	PTGIR
ENSG00000160014	10.97478974	10.78609522	protein_coding	19	47104566	47114039	CALM3
ENSG00000160049	10.07033902	9.496780665	protein_coding	1	10516579	10532583	DFFA
ENSG00000160050	5.741167503	5.80042639	protein_coding	1	32665987	32670988	CCDC28B
ENSG00000160051	4.77879189	5.085909553	protein_coding	1	32671236	32674288	IQCC
ENSG00000160055	5.472368541	6.360445592	protein_coding	1	32680073	32687972	TMEM234
ENSG00000160058	8.879339004	9.242070261	protein_coding	1	32830704	32860332	BSDC1
ENSG00000160062	2.671945279	2.721932731	protein_coding	1	33005028	33066591	ZBTB8A
ENSG00000160072	8.772951555	8.811942385	protein_coding	1	1407143	1433228	ATAD3B
ENSG00000160075	10.06756346	10.0885135	protein_coding	1	1477053	1510249	SSU72
ENSG00000160087	8.949763328	8.900038352	protein_coding	1	1189289	1209265	UBE2J2
ENSG00000160094	6.733233509	6.270323763	protein_coding	1	33722146	33766320	ZNF362
ENSG00000160097	3.004694206	1.925536307	protein_coding	1	33327869	33338083	FNDC5
ENSG00000160111	3.830412367	3.32456471	protein_coding	19	17003758	17137625	CPAMD8
ENSG00000160113	10.07532163	9.887675661	protein_coding	19	17342694	17356151	NR2F6
ENSG00000160117	6.068837212	5.112089758	protein_coding	19	17392454	17398455	ANKLE1
ENSG00000160124	7.64536895	7.593823731	protein_coding	3	122078438	122102078	CCDC58
ENSG00000160131	8.904442656	9.057364358	protein_coding	X	150564987	150577836	VMA21
ENSG00000160145	4.999881834	3.906303962	protein_coding	3	123798870	124440036	KALRN
ENSG00000160161	10.73907363	10.10978283	protein_coding	19	19649074	19657468	CILP2
ENSG00000160172	6.386460534	5.536128679	pseudogene	11	67559119	67572807	FAM86C2P
ENSG00000160179	1.616589159	2.334191469	protein_coding	21	43619799	43720714	ABCG1
ENSG00000160181	0.499066092	0	protein_coding	21	43766466	43771237	TFF2
ENSG00000160182	2.928422289	2.652276565	protein_coding	21	43782391	43786703	TFF1
ENSG00000160183	8.109865782	8.027771115	protein_coding	21	38437942	43816955	TMPRSS3
ENSG00000160185	3.652182994	4.048652948	protein_coding	21	43824008	43867791	UBASH3A
ENSG00000160188	3.871730003	3.935919592	protein_coding	21	43892596	43916464	RSPH1
ENSG00000160190	7.31554162	6.677762549	protein_coding	21	43916128	44001550	SLC37A1
ENSG00000160191	5.018397273	2.039052734	protein_coding	21	44073746	44195619	PDE9A
ENSG00000160193	9.354994343	9.113519788	protein_coding	21	44263204	44299678	WDR4
ENSG00000160194	8.353554553	8.587754596	protein_coding	21	44299754	44329783	NDUFV3
ENSG00000160199	7.19863491	7.524852175	protein_coding	21	44394620	44454172	PKNOX1
ENSG00000160200	10.38677526	10.07776009	protein_coding	21	44473301	44497053	CBS
ENSG00000160201	9.858024966	9.917208266	protein_coding	21	44513066	44527697	U2AF1
ENSG00000160202	1.799000381	1.925536307	protein_coding	21	44589118	44592915	CRYAA
ENSG00000160207	4.097495944	5.004391715	protein_coding	21	44949072	45079374	HSF2BP
ENSG00000160208	10.08413733	10.02792279	protein_coding	21	45079429	45115958	RRP1B
ENSG00000160209	10.60882284	10.02103068	protein_coding	21	45138975	45194151	PDXK
ENSG00000160211	11.34236022	10.24384153	protein_coding	X	153759606	153775787	G6PD
ENSG00000160213	7.89873181	7.784953917	protein_coding	21	45192393	45196326	CSTB
ENSG00000160214	9.036344956	8.941691738	protein_coding	21	45209394	45225174	RRP1
ENSG00000160216	9.611271904	9.511607983	protein_coding	21	45285067	45406417	AGPAT3
ENSG00000160218	9.212512411	9.272131838	protein_coding	21	45432200	45526433	TRAPPC10
ENSG00000160219	4.381167248	4.676215056	protein_coding	X	153903529	153979858	GAB3
ENSG00000160221	8.195750939	8.844514354	protein_coding	21	45553487	45565605	C21orf33
ENSG00000160223	9.49130376	9.53114363	protein_coding	21	45642874	45660849	ICOSLG
ENSG00000160224	0.499066092	0.950786998	protein_coding	21	45705721	45718531	AIRE
ENSG00000160226	6.646617938	7.002128877	protein_coding	21	45748827	45759285	C21orf2
ENSG00000160229	4.6658819	4.65855978	pseudogene	19	20959110	20991922	ZNF66P
ENSG00000160233	5.763291843	5.41517489	protein_coding	21	45875369	45878739	LRRC3
ENSG00000160255	7.72361917	6.59664425	protein_coding	21	46305868	46351904	ITGB2
ENSG00000160256	7.451199747	7.418726742	protein_coding	21	46359925	46396904	FAM207A
ENSG00000160271	9.145657575	9.197681135	protein_coding	9	135973107	136039301	RALGDS
ENSG00000160282	6.851544845	8.301022059	protein_coding	21	47556176	47575481	FTCD
ENSG00000160284	6.809636522	7.070480904	protein_coding	21	47581062	47604390	C21orf56
ENSG00000160285	10.44225492	10.14903446	protein_coding	21	47608360	47648738	LSS

ENSG00000160293	9.936790859	9.221925935	protein_coding	9	136627016	136857726	VAV2
ENSG00000160294	9.941649609	9.762067953	protein_coding	21	47655047	47706211	MCM3AP
ENSG00000160298	6.573375019	6.854868327	protein_coding	21	47720095	47743789	C21orf58
ENSG00000160299	9.089849478	9.464757353	protein_coding	21	47744036	47865682	PCNT
ENSG00000160305	8.999540089	8.89533508	protein_coding	21	47878812	47989926	DIP2A
ENSG00000160310	8.848626402	9.403804382	protein_coding	21	48055079	48085036	PRMT2
ENSG00000160318	2.35979773	2.788380093	protein_coding	19	51870352	51872257	CLDND2
ENSG00000160321	2.847891871	3.608232228	protein_coding	19	22148897	22193745	ZNF208
ENSG00000160323	7.37781226	7.823095289	protein_coding	9	136279478	136324508	ADAMTS13
ENSG00000160325	6.313367288	6.480864016	protein_coding	9	136325089	136335970	C9orf7
ENSG00000160326	10.6371792	10.80176251	protein_coding	9	136336217	136344259	SLC2A6
ENSG00000160336	8.487504052	8.415818989	processed_transc	19	53935227	53961515	ZNF761
ENSG00000160345	5.695874189	6.192713747	protein_coding	9	138387027	138393580	C9orf116
ENSG00000160352	6.956090577	6.889336956	protein_coding	19	21264971	21301990	ZNF714
ENSG00000160360	9.205459721	9.046375341	protein_coding	9	139221932	139254057	GPSM1
ENSG00000160392	6.35766608	6.3217081	protein_coding	19	40826973	40854421	C19orf47
ENSG00000160396	0.869158192	1.667587519	protein_coding	19	40885179	40896094	HIPK4
ENSG00000160401	5.718698586	5.871116342	protein_coding	9	130469268	130478281	C9orf117
ENSG00000160404	7.751556534	7.749951106	protein_coding	9	130493803	130497604	TOR2A
ENSG00000160408	8.64474405	8.500015927	protein_coding	9	130647600	130667687	ST6GALNAC6
ENSG00000160410	9.319971717	8.867749534	protein_coding	19	41082757	41097301	SHKBP1
ENSG00000160439	8.460675529	8.135396417	protein_coding	19	55555711	55580914	RDH13
ENSG00000160445	9.477987143	9.461579211	protein_coding	9	131492065	131534693	ZER1
ENSG00000160446	9.05552222	8.965221116	protein_coding	9	131483148	131486406	ZDHHC12
ENSG00000160447	7.873527714	7.747865429	protein_coding	9	131464802	131483197	PKN3
ENSG00000160460	2.762599152	3.133298822	protein_coding	19	40972148	41082365	SPTBN4
ENSG00000160469	5.806547963	6.419292172	protein_coding	19	55795534	55823901	BRSK1
ENSG00000160471	5.255381949	2.334191469	protein_coding	19	55861076	55866182	COX6B2
ENSG00000160472	3.78787645	2.721932731	protein_coding	19	55888204	55889612	TMEM190
ENSG00000160539	0.499066092	0.697730409	protein_coding	9	134165081	134184649	PPAPDC3
ENSG00000160551	7.757079669	7.556178852	protein_coding	17	27717943	27871502	TAOK1
ENSG00000160563	7.488200893	7.475288518	protein_coding	9	134735494	134955295	MED27
ENSG00000160570	7.110771062	7.026422172	protein_coding	19	42702752	42721813	DEDD2
ENSG00000160584	8.741255403	8.717049372	protein_coding	11	116714118	116969153	SIK3
ENSG00000160588	7.144876945	6.619638408	protein_coding	11	118097409	118123065	MPZL3
ENSG00000160593	0.869158192	0	protein_coding	11	118064455	118095809	AMICA1
ENSG00000160602	6.716321419	6.16171561	protein_coding	17	27055822	27069784	NEK8
ENSG00000160606	7.348842929	7.903991977	protein_coding	17	27051366	27053950	TLCD1
ENSG00000160613	9.318104658	9.705158168	protein_coding	11	117075053	117103241	PCSK7
ENSG00000160633	10.64539243	10.51645069	protein_coding	19	5623150	5668488	SAFB
ENSG00000160678	3.146112541	3.411459265	protein_coding	1	153600402	153604513	S100A1
ENSG00000160679	9.743052004	9.904157241	protein_coding	1	153606525	153618782	CHTOP
ENSG00000160685	9.283120944	9.131229537	protein_coding	1	154975127	154990998	ZBTB7B
ENSG00000160688	9.369490093	9.697613846	protein_coding	1	154955814	154965587	FLAD1
ENSG00000160691	11.29923875	11.48326581	protein_coding	1	154934774	154946871	SHC1
ENSG00000160695	8.711694395	9.143974165	processed_transc	11	118938403	118952688	VPS11
ENSG00000160703	8.766120079	9.229419632	protein_coding	11	119037277	119054725	NLRX1
ENSG00000160710	12.90029205	13.05982354	protein_coding	1	154554538	154600475	ADAR
ENSG00000160712	7.049074049	6.690849666	protein_coding	1	154377669	154441926	IL6R
ENSG00000160714	9.70212013	9.76979748	protein_coding	1	154521053	154531504	UBE2Q1
ENSG00000160716	2.928422289	3.133298822	protein_coding	1	154540257	154552502	CHRNB2
ENSG00000160741	9.72977077	9.791728663	protein_coding	1	153920145	153931101	CRTC2
ENSG00000160746	8.286389971	8.180940029	protein_coding	3	43396351	43733086	ANO10
ENSG00000160752	9.588218042	9.554003775	protein_coding	1	155278539	155290457	FDPS
ENSG00000160753	9.30119149	9.391832931	protein_coding	1	155290687	155300905	RUSC1
ENSG00000160766	6.610461229	7.591499486	pseudogene	1	155183616	155197214	GBAP1
ENSG00000160767	10.45505461	10.15573604	protein_coding	1	155216996	155225274	FAM189B
ENSG00000160781	7.484876093	7.690381265	protein_coding	1	156213206	156217881	PAQR6
ENSG00000160783	7.222786417	7.827052132	protein_coding	1	156182784	156212874	PMF1
ENSG00000160785	9.40334477	9.226426823	protein_coding	1	156163880	156182587	SLC25A44
ENSG00000160789	12.92519015	12.58554936	protein_coding	1	156052364	156109880	LMNA
ENSG00000160796	9.426059074	9.253876247	protein_coding	3	47021173	47051193	NBEAL2
ENSG00000160799	7.50471092	7.572769481	protein_coding	3	46963216	47023500	CCDC12

ENSG00000160801	1.16343121	2.652276565	protein_coding	3	46919236	46945287	PTH1R
ENSG00000160803	10.62477101	10.68752499	protein_coding	1	156005092	156023585	UBQLN4
ENSG00000160813	6.84118134	6.637773454	protein_coding	7	100032905	100034188	PPP1R35
ENSG00000160818	9.214521149	9.865723786	protein_coding	1	156564279	156571288	GPATCH4
ENSG00000160828	5.525114348	5.66550919	protein_coding	7	74112305	74306729	STAG3L2
ENSG00000160838	1.407729925	1.16600992	protein_coding	1	156890442	156902886	LRRC71
ENSG00000160862	5.472368541	4.888000647	protein_coding	7	99564343	99573780	AZGP1
ENSG00000160867	7.797842652	7.237355663	protein_coding	5	176513887	176525145	FGFR4
ENSG00000160868	0	0.390640832	protein_coding	7	99354604	99381888	CYP3A4
ENSG00000160870	0.499066092	0.390640832	protein_coding	7	99293368	99332819	CYP3A7
ENSG00000160877	12.53235086	12.18120713	protein_coding	19	13229109	13251955	NACC1
ENSG00000160886	5.909333162	6.637773454	protein_coding	8	143781529	143786545	LY6K
ENSG00000160888	12.50152617	11.05068645	protein_coding	19	13261282	13265716	IER2
ENSG00000160908	7.612231145	7.083771121	protein_coding	7	99084142	99097947	ZNF394
ENSG00000160917	8.622223055	8.070120888	protein_coding	7	99036545	99054994	CPSF4
ENSG00000160932	11.2048893	11.1476082	protein_coding	8	144099399	144105249	LY6E
ENSG00000160948	8.54291017	8.999810935	protein_coding	8	145649000	145653931	VPS28
ENSG00000160949	9.144604451	8.975061478	protein_coding	8	145654165	145669827	TONSL
ENSG00000160951	3.603959378	2.912743273	protein_coding	19	14583278	14586174	PTGER1
ENSG00000160953	8.935220147	9.292307546	protein_coding	19	1285890	1378430	MUM1
ENSG00000160957	9.505319266	9.647030526	processed_transc	8	145736667	145743229	RECQL4
ENSG00000160959	7.850461307	7.76240224	protein_coding	8	145743376	145750557	LRRC14
ENSG00000160961	6.535310252	7.284161038	protein_coding	19	14800613	14844557	ZNF333
ENSG00000160963	2.10647801	1.353254395	polymorphic_pse	7	101006101	101202304	EMID2
ENSG00000160972	7.967047117	8.363653959	protein_coding	8	145703352	145727504	PPP1R16A
ENSG00000160973	0.869158192	1.667587519	protein_coding	8	145698795	145701718	FOXH1
ENSG00000160991	8.890689734	8.073455769	protein_coding	7	102073553	102097268	ORAI2
ENSG00000160993	7.035502862	6.264499759	protein_coding	7	102096685	102105323	ALKBH4
ENSG00000160999	6.704935538	5.9163892	protein_coding	7	101928405	101962178	SH2B2
ENSG00000161010	7.575190993	7.733180651	protein_coding	5	179261436	179289173	C5orf45
ENSG00000161011	11.04504617	11.29040167	protein_coding	5	179233388	179265078	SQSTM1
ENSG00000161013	11.27946544	11.33658474	protein_coding	5	179224597	179233952	MGAT4B
ENSG00000161016	13.61025698	13.64124012	protein_coding	8	146015150	146017972	RPL8
ENSG00000161021	9.037480115	8.89533508	protein_coding	5	179159851	179223512	MAML1
ENSG00000161031	0.499066092	0	protein_coding	19	15579456	15590663	PGLYRP2
ENSG00000161036	8.825155121	8.395978102	protein_coding	7	102105376	102113615	LRWD1
ENSG00000161040	7.423680977	7.675096386	protein_coding	7	102453308	102715286	FBXL13
ENSG00000161048	7.049074049	6.889336956	protein_coding	7	102740223	102790007	NAPEPLD
ENSG00000161057	10.40404042	10.25085438	protein_coding	7	102984701	103009842	PSMC2
ENSG00000161082	4.543379137	3.411459265	protein_coding	19	3224701	3297074	CELF5
ENSG00000161091	11.07105438	11.27961406	protein_coding	19	3538263	3557571	MFSD12
ENSG00000161103	1.16343121	2.334191469	processed_transc	22	18837631	18848562	
ENSG00000161132	1.616589159	1.518964905	pseudogene	22	20287283	20300739	
ENSG00000161133	0	0.697730409	protein_coding	22	20704868	20745048	USP41
ENSG00000161149	1.616589159	2.420525079	pseudogene	22	21357184	21368581	
ENSG00000161179	9.472122247	9.55579319	protein_coding	22	21982378	21984353	YDJC
ENSG00000161180	2.671945279	2.851901313	protein_coding	22	21987007	21991616	CCDC116
ENSG00000161202	10.24116219	9.928301652	protein_coding	3	183873176	183891398	DVL3
ENSG00000161203	12.37758747	12.33143922	protein_coding	3	183892477	183901879	AP2M1
ENSG00000161204	9.500388149	9.397831074	protein_coding	3	183903811	183911800	ABCF3
ENSG00000161217	9.394512382	8.988372895	protein_coding	3	195941093	196014828	PCYT1A
ENSG00000161243	6.825495146	5.700439663	protein_coding	19	39514665	39523198	FBXO27
ENSG00000161249	9.514316103	7.593823731	protein_coding	19	35988122	36004560	DMKN
ENSG00000161265	5.91922012	6.633261019	protein_coding	19	36233430	36236336	U2AF1L4
ENSG00000161267	4.821592551	7.644032061	protein_coding	3	197236654	197300194	BDH1
ENSG00000161270	1.616589159	1.925536307	protein_coding	19	36316283	36342895	NPHS1
ENSG00000161277	4.863159938	5.66550919	protein_coding	19	36525887	36545664	THAP8
ENSG00000161281	2.35979773	2.652276565	protein_coding	19	36641824	36643771	COX7A1
ENSG00000161298	5.967663407	5.98174124	protein_coding	19	37095719	37119499	ZNF382
ENSG00000161326	8.150302734	7.962719647	protein_coding	17	35849937	35873603	DUSP14
ENSG00000161328	6.393570237	6.382123086	protein_coding	11	537527	554916	LRRC56
ENSG00000161381	3.652182994	3.183544561	protein_coding	17	37219556	37309485	PLXDC1
ENSG00000161395	5.575999561	5.988823159	protein_coding	17	37827376	37844310	PGAP3

ENSG00000161405	6.835971536	6.57327766	protein_coding	17	37913968	38020441	IKZF3
ENSG00000161509	3.830412367	2.242360793	protein_coding	17	72838162	72856966	GRIN2C
ENSG00000161513	7.399163791	7.426571535	protein_coding	17	72858620	72869156	FDXR
ENSG00000161526	8.947349623	9.233897238	protein_coding	17	73663399	73704139	SAP30BP
ENSG00000161533	8.690202285	8.703122764	protein_coding	17	73937596	73975515	ACOX1
ENSG00000161542	7.96466218	8.088368066	protein_coding	17	74307014	74380602	PRPSAP1
ENSG00000161544	3.830412367	2.579085888	protein_coding	17	74523440	74533782	CYGB
ENSG00000161547	11.18505085	11.39788976	protein_coding	17	74730201	74733493	SRSF2
ENSG00000161551	7.15746161	7.964516916	protein_coding	19	52359055	52394203	ZNF577
ENSG00000161558	6.548110424	7.148445524	protein_coding	19	48835613	48867197	TMEM143
ENSG00000161570	7.149084042	6.750400563	protein_coding	17	34198495	34207797	CCL5
ENSG00000161572	0.499066092	0.390640832	protein_coding	17	34261548	34270674	LYZL6
ENSG00000161594	0	1.16600992	protein_coding	17	39991937	40004636	KLHL10
ENSG00000161609	4.962122459	5.42565083	protein_coding	19	49891475	49921256	CCDC155
ENSG00000161610	0	0.697730409	protein_coding	17	40336078	40337470	HCRT
ENSG00000161618	8.826469148	9.663720744	protein_coding	19	49956473	49974304	ALDH16A1
ENSG00000161638	12.39695366	13.11603677	protein_coding	12	54789045	54813244	ITGA5
ENSG00000161640	0	0.390640832	protein_coding	19	50412758	50464429	SIGLEC11
ENSG00000161642	9.979350975	9.398495997	protein_coding	12	54762917	54785082	ZNF385A
ENSG00000161643	0.869158192	0.697730409	protein_coding	19	50472857	50478191	SIGLEC16
ENSG00000161647	6.670228606	6.376734172	protein_coding	17	41878167	41910538	MPP3
ENSG00000161649	0	0.390640832	protein_coding	17	41924516	41940994	CD300LG
ENSG00000161653	6.567100227	5.456629825	protein_coding	17	42082031	42086433	NAGS
ENSG00000161654	9.16240384	8.657187659	protein_coding	17	42112004	42144103	LSM12
ENSG00000161664	5.018397273	4.903073497	protein_coding	17	42248074	42256451	ASB16
ENSG00000161671	8.74542939	9.261937181	protein_coding	19	50979736	50986607	C19orf63
ENSG00000161677	7.296703257	7.900240894	protein_coding	19	51009255	51014435	JOSD2
ENSG00000161681	4.642199401	5.700439663	protein_coding	19	51165084	51222707	SHANK1
ENSG00000161682	6.414691513	5.960284481	protein_coding	17	42430585	42441243	FAM171A2
ENSG00000161692	8.303476525	8.343077302	protein_coding	17	42785976	42829632	DBF4B
ENSG00000161714	8.888175056	8.419754632	protein_coding	17	43186335	43210580	PLCD3
ENSG00000161791	9.012297947	9.705695541	protein_coding	12	50031724	50101948	FMNL3
ENSG00000161800	10.33555133	10.43128732	protein_coding	12	50370706	50426919	RACGAP1
ENSG00000161813	10.1252916	10.00452874	protein_coding	12	50786166	50873787	LARP4
ENSG00000161835	3.393122761	3.493416095	protein_coding	12	52400724	52409673	GRASP
ENSG00000161847	11.93675152	11.91143761	protein_coding	19	10426895	10444314	RAVER1
ENSG00000161860	6.733233509	7.883239271	protein_coding	19	13009600	13030076	SYCE2
ENSG00000161888	8.316627558	8.390640789	protein_coding	19	11257444	11266484	SPC24
ENSG00000161896	4.618121645	5.187895122	protein_coding	6	33689444	33714762	IP6K3
ENSG00000161904	8.997208309	9.048071417	protein_coding	6	33738979	33756913	LEMD2
ENSG00000161905	1.16343121	2.039052734	protein_coding	17	4534214	4545589	ALOX15
ENSG00000161912	5.91922012	7.51752531	pseudogene	6	41068761	41108571	
ENSG00000161914	7.169937448	7.359856617	protein_coding	19	11594245	11616738	ZNF653
ENSG00000161920	7.01259661	7.850567963	protein_coding	17	4634723	4636888	MED11
ENSG00000161921	6.220467583	5.700439663	protein_coding	17	4636828	4643223	CXCL16
ENSG00000161929	1.616589159	2.788380093	protein_coding	17	5113705	5138137	SCIMP
ENSG00000161939	0.499066092	0	protein_coding	17	6915954	6920839	C17orf49
ENSG00000161940	6.068837212	6.677762549	protein_coding	17	6926369	6933219	BCL6B
ENSG00000161955	4.962122459	6.030599799	protein_coding	17	7461609	7464925	TNFSF13
ENSG00000161956	10.37918427	10.87358473	protein_coding	17	7465272	7475287	SENP3
ENSG00000161958	7.68357507	9.543220426	protein_coding	17	7342689	7348256	FGF11
ENSG00000161960	13.53157464	13.86023647	protein_coding	17	7476024	7482324	EIF4A1
ENSG00000161970	12.12872304	13.15497334	protein_coding	17	8280838	8286568	RPL26
ENSG00000161980	7.952677993	8.112884393	protein_coding	16	96407	103628	POLR3K
ENSG00000161981	8.526833966	8.904726341	protein_coding	16	103010	107669	SNRNP25
ENSG00000161992	0.869158192	1.518964905	protein_coding	16	610422	615528	C16orf11
ENSG00000161996	10.13539932	10.42117962	protein_coding	16	699311	717833	WDR90
ENSG00000161999	8.066039442	8.190185975	protein_coding	16	731671	734529	JMJD8
ENSG00000162004	6.670228606	6.293387477	protein_coding	16	772582	776954	CCDC78
ENSG00000162006	0.869158192	0	protein_coding	16	819428	833370	MSLNL
ENSG00000162009	1.616589159	0.390640832	protein_coding	16	1122756	1131454	SSTR5
ENSG00000162032	1.407729925	1.802319292	protein_coding	16	1826713	1832821	SPSB3
ENSG00000162039	7.813830732	8.488819803	protein_coding	16	1883984	1934295	C16orf73

ENSG00000162062	7.717966186	8.012215662	protein_coding	16	2510081	2514964	C16orf59
ENSG00000162063	9.642940664	9.287290009	protein_coding	16	2479395	2508855	CCNF
ENSG00000162065	8.340737341	8.410554709	protein_coding	16	2525147	2555735	TBC1D24
ENSG00000162066	7.22677265	7.598461017	protein_coding	16	2570358	2581423	AMDHD2
ENSG00000162068	1.799000381	2.721932731	protein_coding	16	2521500	2524146	NTN3
ENSG00000162069	2.762599152	0.697730409	protein_coding	16	3077868	3085542	CCDC64B
ENSG00000162073	8.734271811	7.939149102	protein_coding	16	3019246	3023490	PAQR4
ENSG00000162076	7.955082807	8.053329862	protein_coding	16	2933187	2949383	FLYWCH2
ENSG00000162078	2.847891871	3.027231696	protein_coding	16	2880170	2882285	ZG16B
ENSG00000162086	7.850461307	8.076782959	protein_coding	16	3355486	3368574	ZNF75A
ENSG00000162104	9.38473389	9.104582717	protein_coding	16	4012657	4166186	ADCY9
ENSG00000162105	5.563445526	1.802319292	protein_coding	11	70313961	70963623	SHANK2
ENSG00000162129	9.356814297	9.337390687	protein_coding	11	72003469	72145692	CLPB
ENSG00000162139	7.049074049	7.012590419	protein_coding	11	74699179	74729938	NEU3
ENSG00000162144	8.893200037	9.155820862	protein_coding	11	61116226	61129771	CYBASC3
ENSG00000162148	3.830412367	3.993387124	protein_coding	11	61248592	61258403	PPP1R32
ENSG00000162174	6.220467583	6.831423059	protein_coding	11	62104920	62160882	ASRGL1
ENSG00000162188	0	0.950786998	protein_coding	11	62475130	62476673	GNG3
ENSG00000162191	9.874634141	10.12432619	protein_coding	11	62443970	62446567	UBXN1
ENSG00000162194	8.46911316	8.794871435	protein_coding	11	62430287	62439727	C11orf48
ENSG00000162222	7.633406721	7.844724842	protein_coding	11	62495541	62507756	TTC9C
ENSG00000162227	8.066039442	8.458531597	protein_coding	11	62538775	62554814	TAF6L
ENSG00000162231	9.142495894	9.474880612	protein_coding	11	62559598	62573774	NXF1
ENSG00000162236	8.955779984	9.258278703	protein_coding	11	62574369	62599560	STX5
ENSG00000162241	6.26766507	6.510691757	protein_coding	11	65142663	65151172	SLC25A45
ENSG00000162244	11.2393334	11.63443472	protein_coding	3	52027644	52029958	RPL29
ENSG00000162267	0	0.390640832	protein_coding	3	52828784	52843025	ITIH3
ENSG00000162290	8.072698871	7.887034762	processed_transc	3	53317448	53381637	DCP1A
ENSG00000162298	9.528110879	9.798797703	protein_coding	11	64889252	64902004	SYVN1
ENSG00000162300	5.827699195	6.35497494	protein_coding	11	64851695	64855870	ZFPL1
ENSG00000162302	10.32024245	9.684042928	protein_coding	11	64126620	64139687	RPS6KA4
ENSG00000162337	11.28353752	10.86735403	protein_coding	11	68080077	68216743	LRP5
ENSG00000162341	7.837485466	7.860254213	protein_coding	11	68816350	68858072	TPCN2
ENSG00000162344	2.10647801	2.242360793	protein_coding	11	69513000	69519410	FGF19
ENSG00000162365	0.499066092	0.697730409	protein_coding	1	47603107	47615413	CYP4A22
ENSG00000162366	7.35977471	7.909600377	protein_coding	1	47649265	47656716	PDZK1IP1
ENSG00000162367	3.211941663	0	protein_coding	1	47681963	47697892	TAL1
ENSG00000162368	10.13539932	10.40307044	protein_coding	1	47799469	47844511	CMPK1
ENSG00000162377	7.873527714	7.905863867	protein_coding	1	53152508	53164038	SELRC1
ENSG00000162378	8.322227223	8.525807191	protein_coding	1	53192126	53293014	ZYG11B
ENSG00000162384	7.304268146	7.926295939	protein_coding	1	53679771	53686289	C1orf123
ENSG00000162385	7.612231145	8.055017786	protein_coding	1	53692564	53704282	MAGOH
ENSG00000162390	4.734682531	3.232099092	protein_coding	1	55007930	55104865	ACOT11
ENSG00000162391	2.10647801	0.390640832	protein_coding	1	55074855	55089229	FAM151A
ENSG00000162396	6.220467583	6.419292172	protein_coding	1	55222571	55230187	PARS2
ENSG00000162398	1.799000381	0	protein_coding	1	55271736	55307925	C1orf177
ENSG00000162402	9.769253212	9.67638708	protein_coding	1	55532032	55681039	USP24
ENSG00000162407	8.677151638	8.119352325	protein_coding	1	56960419	57110974	PPAP2B
ENSG00000162408	8.738466022	8.487570409	protein_coding	1	6581407	6614595	NOL9
ENSG00000162413	9.306851272	9.420268302	protein_coding	1	6650784	6674667	KLHL21
ENSG00000162415	3.146112541	4.17820932	protein_coding	1	45482071	45771881	ZSWIM5
ENSG00000162419	7.82964357	7.778838337	protein_coding	1	28995244	29045865	GMEB1
ENSG00000162426	4.097495944	2.971122874	protein_coding	1	8377886	8404227	SLC45A1
ENSG00000162430	10.35298082	10.48423953	protein_coding	1	26126667	26144713	SEPN1
ENSG00000162433	10.50883489	10.49329404	protein_coding	1	65613232	65697828	AK4
ENSG00000162434	10.74046841	10.7118033	protein_coding	1	65298912	65432187	JAK1
ENSG00000162437	7.420203928	7.225413142	protein_coding	1	65210778	65298915	RAVER2
ENSG00000162438	1.616589159	0.390640832	protein_coding	1	15764935	15775737	CTRC
ENSG00000162441	7.37062428	7.402908012	protein_coding	1	9982171	10003465	LZIC
ENSG00000162444	1.16343121	0.950786998	protein_coding	1	10057264	10076078	RBP7
ENSG00000162458	8.371669553	6.668971382	protein_coding	1	16083102	16113089	FBLIM1
ENSG00000162461	3.274897671	3.133298822	protein_coding	1	16062900	16067891	SLC25A34
ENSG00000162482	4.261916566	4.448594745	protein_coding	1	19609052	19615744	AKR7A3

ENSG00000162490	1.16343121	2.788380093	protein_coding	1	11751786	11785914	C1orf187
ENSG00000162496	7.169937448	6.097646709	protein_coding	1	12627939	12677737	DHRS3
ENSG00000162510	2.10647801	2.420525079	protein_coding	1	31184124	31196434	MATN1
ENSG00000162511	5.785082014	3.368666104	protein_coding	1	31205316	31230667	LAPTM5
ENSG00000162512	10.30806544	9.974965329	protein_coding	1	31342314	31381608	SDC3
ENSG00000162517	9.211506993	9.322752514	protein_coding	1	32095463	32110497	PEF1
ENSG00000162520	6.032862302	5.832272499	protein_coding	1	33145507	33169197	SYNC
ENSG00000162521	8.883132512	8.76630664	protein_coding	1	33116743	33146258	RBBP4
ENSG00000162522	10.81517599	9.517741338	protein_coding	1	33207486	33240571	KIAA1522
ENSG00000162526	0	0.390640832	protein_coding	1	32827798	32829913	TSSK3
ENSG00000162542	7.262161623	6.959507185	protein_coding	1	20008706	20126438	TMCO4
ENSG00000162543	5.316509574	4.17820932	protein_coding	1	20512578	20522541	UBXN10
ENSG00000162545	7.214780754	6.930376707	protein_coding	1	20808884	20812713	CAMK2N1
ENSG00000162551	1.799000381	1.667587519	protein_coding	1	21835858	21904905	ALPL
ENSG00000162552	5.158541613	5.32851069	protein_coding	1	22446461	22470462	WNT4
ENSG00000162571	1.616589159	0	protein_coding	1	1109264	1133315	TTLL10
ENSG00000162572	5.995967334	6.450406542	protein_coding	1	1214447	1227409	SCNN1D
ENSG00000162576	5.858855738	6.349483465	protein_coding	1	1288069	1297157	MXRA8
ENSG00000162585	8.227927477	8.38394126	protein_coding	1	2115903	2144159	C1orf86
ENSG00000162591	9.385625588	10.22184491	protein_coding	1	3406484	3528059	MEGF6
ENSG00000162592	0.869158192	0	protein_coding	1	3668962	3688208	CCDC27
ENSG00000162595	0.869158192	0.697730409	protein_coding	1	68511645	68517314	DIRAS3
ENSG00000162599	7.720795447	6.45552765	protein_coding	1	61330931	61928465	NFIA
ENSG00000162600	6.129712275	6.771086752	protein_coding	1	58881056	59012474	OMA1
ENSG00000162601	8.259402007	8.560682282	protein_coding	1	59120411	59165764	MYSM1
ENSG00000162604	6.0776925	6.387491945	protein_coding	1	62146717	62191095	TM2D1
ENSG00000162607	9.360447332	9.587045627	protein_coding	1	62901968	62917475	USP1
ENSG00000162613	9.559268022	9.09886653	protein_coding	1	78409740	78444794	FUBP1
ENSG00000162614	6.17992145	7.167295693	protein_coding	1	78354198	78409580	NEXN
ENSG00000162616	7.23866569	7.992972811	protein_coding	1	78444859	78483648	DNAJB4
ENSG00000162620	3.077135474	4.021284656	protein_coding	1	74491699	74663871	LRRIQ3
ENSG00000162623	7.517784241	7.556178852	protein_coding	1	75198836	75232362	TYW3
ENSG00000162627	7.454602977	7.83885796	protein_coding	1	99127236	99226056	SNX7
ENSG00000162630	0	1.353254395	protein_coding	1	193148175	193155784	B3GALT2
ENSG00000162636	5.83815965	6.587342979	protein_coding	1	109102711	109187522	FAM102B
ENSG00000162639	5.741167503	5.6022688	protein_coding	1	109190912	109204148	HENMT1
ENSG00000162641	2.35979773	3.027231696	protein_coding	1	109358520	109506106	AKNAD1
ENSG00000162642	6.861834435	6.952279523	protein_coding	1	85715639	85725355	C1orf52
ENSG00000162643	0.499066092	0.390640832	protein_coding	1	85464830	85598821	WDR63
ENSG00000162645	8.759256101	9.728615271	protein_coding	1	89571815	89616139	GBP2
ENSG00000162650	6.386460534	7.022976646	protein_coding	1	110026101	110035426	ATXN7L2
ENSG00000162654	6.196276899	6.1803943	protein_coding	1	89646831	89664615	GBP4
ENSG00000162664	7.955082807	8.534302826	protein_coding	1	90457722	90494097	ZNF326
ENSG00000162676	1.407729925	2.721932731	protein_coding	1	92940319	92952433	GFI1
ENSG00000162687	0	0.390640832	protein_coding	1	196194909	196577541	KCNT2
ENSG00000162688	8.004679484	8.316585024	protein_coding	1	100315640	100389579	AGL
ENSG00000162692	2.471521042	0.697730409	protein_coding	1	101185305	101204601	VCAM1
ENSG00000162694	7.234712227	7.996490656	protein_coding	1	101337943	101361554	EXTL2
ENSG00000162695	8.597806729	8.612036819	protein_coding	1	101361632	101447309	SLC30A7
ENSG00000162702	8.312882337	8.330588949	protein_coding	1	200375827	200379184	ZNF281
ENSG00000162704	9.732576998	10.37361447	protein_coding	1	183592401	183604892	ARPC5
ENSG00000162706	0.499066092	0	protein_coding	1	159141399	159173103	CADM3
ENSG00000162711	0.869158192	0.697730409	protein_coding	1	247579458	247612410	NLRP3
ENSG00000162714	8.369868254	8.512355001	protein_coding	1	247460714	247495148	ZNF496
ENSG00000162722	0	0.390640832	protein_coding	1	248020501	248041507	TRIM58
ENSG00000162723	1.16343121	0.390640832	protein_coding	1	159921282	159924044	SLAMF9
ENSG00000162728	0.869158192	0.390640832	protein_coding	1	160051360	160060353	KCNJ9
ENSG00000162729	8.916832554	8.614328238	protein_coding	1	160061130	160068733	IGSF8
ENSG00000162733	3.211941663	3.133298822	protein_coding	1	162601163	162750237	DDR2
ENSG00000162734	11.27586285	11.84201006	protein_coding	1	160175127	160185166	PEA15
ENSG00000162735	9.154055005	9.302290571	protein_coding	1	160246602	160256138	PEX19
ENSG00000162736	10.54998355	11.03173896	protein_coding	1	160313062	160328742	NCSTN
ENSG00000162738	2.10647801	1.16600992	protein_coding	1	160370376	160398468	VANGL2

ENSG00000162745	5.472368541	4.17820932	protein_coding	1	161952982	161993644	OLFML2B
ENSG00000162746	2.928422289	2.420525079	protein_coding	1	161691353	161697933	FCRLB
ENSG00000162753	0.499066092	0	protein_coding	1	173469603	173572233	SLC9A11
ENSG00000162755	4.026095388	4.622587519	protein_coding	1	161068151	161070136	KLHDC9
ENSG00000162757	5.637181576	5.611474642	protein_coding	1	209955661	209957904	C1orf74
ENSG00000162769	7.759833327	7.907733332	protein_coding	1	213031597	213072705	FLVCR1
ENSG00000162772	9.989357796	8.166959047	protein_coding	1	212738676	212794119	ATF3
ENSG00000162775	7.811178326	7.444711851	protein_coding	1	110881128	110889299	RBM15
ENSG00000162777	5.60078458	4.7279204	protein_coding	1	111729796	111747157	DENND2D
ENSG00000162779	0.499066092	0.950786998	protein_coding	1	179334855	179523870	AXDND1
ENSG00000162783	9.531337629	9.274307066	protein_coding	1	181057638	181059977	IER5
ENSG00000162804	4.999881834	5.085909553	protein_coding	2	241938255	242034983	SNED1
ENSG00000162813	8.267164507	8.792849743	protein_coding	1	220230824	220263804	BPNT1
ENSG00000162814	2.928422289	3.748356452	protein_coding	1	217804666	218045038	SPATA17
ENSG00000162817	2.847891871	2.579085888	protein_coding	1	220863187	220872499	C1orf115
ENSG00000162819	9.305909516	9.264127823	protein_coding	1	222885895	222908538	BROX
ENSG00000162836	8.314756163	7.968104748	protein_coding	1	147119170	147142618	ACP6
ENSG00000162840	0.499066092	0.697730409	pseudogene	4	69242041	69242226	MT1P2
ENSG00000162843	0	0.390640832	protein_coding	1	241815580	241965435	WDR64
ENSG00000162849	3.78787645	2.652276565	protein_coding	1	245318287	245872733	KIF26B
ENSG00000162851	7.797842652	8.352716275	protein_coding	1	246703862	246729626	TFB2M
ENSG00000162852	8.207901394	8.136991056	protein_coding	1	246729746	246831886	CNST
ENSG00000162869	6.598204782	6.686500471	protein_coding	2	48667737	48742525	PPP1R21
ENSG00000162873	0	0.697730409	protein_coding	1	205305220	205326166	KLHDC8A
ENSG00000162877	0.869158192	0.950786998	protein_coding	1	205797150	205819260	PM20D1
ENSG00000162878	3.603959378	4.508954154	protein_coding	2	42275160	42285668	PKDCC
ENSG00000162881	0.499066092	0.390640832	protein_coding	2	42989642	42991401	OXER1
ENSG00000162882	0.499066092	0	protein_coding	2	42994229	43019733	HAAO
ENSG00000162885	9.432976748	9.244291255	protein_coding	1	235610533	235667781	B3GALNT2
ENSG00000162888	3.004694206	3.748356452	protein_coding	1	206664449	206671061	C1orf147
ENSG00000162889	10.09071393	10.04288547	protein_coding	1	206858289	206907628	MAPKAPK2
ENSG00000162891	1.960915222	2.334191469	protein_coding	1	207038699	207042568	IL20
ENSG00000162892	6.502803968	6.775188634	protein_coding	1	207070788	207077484	IL24
ENSG00000162894	5.175139844	5.792353798	protein_coding	1	207077731	207096592	FAIM3
ENSG00000162896	1.799000381	0.697730409	protein_coding	1	207101863	207119811	PIGR
ENSG00000162897	0	0.390640832	protein_coding	1	207131310	207143970	FCAMR
ENSG00000162909	12.14039258	11.79114049	protein_coding	1	223889295	223963720	CAPN2
ENSG00000162910	8.068262669	8.585420545	protein_coding	1	228294380	228297013	MRPL55
ENSG00000162913	4.491309013	3.845183986	protein_coding	1	228391207	228401365	C1orf145
ENSG00000162923	10.44053968	10.47291982	protein_coding	1	224572845	224624735	WDR26
ENSG00000162924	5.752272083	5.84793576	protein_coding	2	61108656	61150645	REL
ENSG00000162927	4.568725997	5.187895122	protein_coding	2	61169104	61245389	PUS10
ENSG00000162928	7.402691859	7.495318771	protein_coding	2	61244360	61276394	PEX13
ENSG00000162929	6.47626108	6.470782801	protein_coding	2	61293006	61391960	KIAA1841
ENSG00000162931	3.652182994	3.411459265	protein_coding	1	228595641	228604562	TRIM17
ENSG00000162944	1.407729925	1.667587519	protein_coding	2	198435524	198540769	RFTN2
ENSG00000162946	4.19837882	5.824376633	protein_coding	1	231762561	232177018	DISC1
ENSG00000162949	0.499066092	0.390640832	protein_coding	2	30945637	31043408	CAPN13
ENSG00000162959	3.335220907	3.64455972	protein_coding	2	32092878	32236299	MEMO1
ENSG00000162961	7.68357507	7.815148897	protein_coding	2	32092878	32264881	DPY30
ENSG00000162971	6.84118134	6.365895578	protein_coding	2	200794698	200820459	TYW5
ENSG00000162972	7.572060955	6.99862477	protein_coding	2	200820040	200873263	C2orf47
ENSG00000162976	6.535310252	6.24096575	protein_coding	2	11295324	11319000	PQLC3
ENSG00000162980	8.75650134	8.514810202	protein_coding	2	152645498	152685009	ARL5A
ENSG00000162994	4.062237333	4.990346085	protein_coding	2	55401927	55459699	C2orf63
ENSG00000162997	3.603959378	3.935919592	pseudogene	2	55509455	55511608	PRORSD1P
ENSG00000162998	0.499066092	0.390640832	protein_coding	2	183698002	183731890	FRZB
ENSG00000162999	4.437288965	4.640685763	protein_coding	2	183943287	183964733	DUSP19
ENSG00000163001	7.434062125	8.075120324	protein_coding	2	55746740	55773015	CCDC104
ENSG00000163002	7.819120959	7.397596322	protein_coding	2	183982241	184026408	NUP35
ENSG00000163006	6.112579918	6.155435249	protein_coding	2	109403213	109493034	CCDC138
ENSG00000163009	5.331395195	4.468996429	protein_coding	2	10281509	10351851	C2orf48
ENSG00000163013	7.770795701	6.690849666	protein_coding	2	73481810	73511559	FBXO41

ENSG00000163016	0.869158192	1.353254395	pseudogene	2	73872046	73912703	ALMS1P
ENSG00000163017	1.799000381	6.725179132	protein_coding	2	74119441	74146992	ACTG2
ENSG00000163026	7.420203928	7.222412001	protein_coding	2	24252210	24272445	C2orf44
ENSG00000163029	9.163444055	9.149514732	protein_coding	2	17845079	17981509	SMC6
ENSG00000163032	0	0.697730409	protein_coding	2	17720393	17838285	VSNL1
ENSG00000163040	5.346128795	5.200150955	protein_coding	2	132285248	132291239	CCDC74A
ENSG00000163041	5.938793061	6.535085617	protein_coding	1	226249552	226259702	H3F3A
ENSG00000163046	6.699208722	7.984140487	protein_coding	2	132905164	133015542	ANKRD30BL
ENSG00000163050	9.438143322	9.706769688	protein_coding	1	227085237	227175246	ADCK3
ENSG00000163053	4.800350936	1.16600992	protein_coding	2	230899698	230933715	SLC16A14
ENSG00000163060	1.16343121	1.16600992	protein_coding	2	95537178	95542574	TEKT4
ENSG00000163064	2.238690726	1.518964905	protein_coding	2	119599766	119605254	EN1
ENSG00000163067	6.328285199	6.016808232	protein_coding	2	95831177	95850065	ZNF2
ENSG00000163069	7.630400601	8.263555427	protein_coding	4	52886872	52904648	SGCB
ENSG00000163072	3.393122761	3.570966319	protein_coding	2	169643049	169722024	NOSTRIN
ENSG00000163075	0.499066092	2.501982735	protein_coding	2	120302008	120419827	
ENSG00000163082	5.124761037	5.112089758	protein_coding	2	223289236	223425667	SGPP2
ENSG00000163083	3.211941663	2.334191469	protein_coding	2	121103719	121109384	INHBB
ENSG00000163092	0	0.950786998	protein_coding	2	167744997	168116263	XIRP2
ENSG00000163093	4.618121645	4.604259349	protein_coding	2	170335688	170382432	BBS5
ENSG00000163104	8.502378699	8.846464976	protein_coding	4	95128762	95212443	SMARCAD1
ENSG00000163106	0.499066092	0	protein_coding	4	95219686	95264027	HPGDS
ENSG00000163110	10.01666786	10.01583991	protein_coding	4	95373037	95589377	PDLIM5
ENSG00000163113	9.051032802	9.289442519	protein_coding	1	149912231	149982686	OTUD7B
ENSG00000163116	0.499066092	0.390640832	protein_coding	4	98105244	99064391	C4orf37
ENSG00000163121	5.40360309	6.281901708	pseudogene	2	97163383	97173846	NEURL3
ENSG00000163125	9.990530519	9.8994674	protein_coding	1	150335567	150449042	RPRD2
ENSG00000163126	3.554067925	3.279072565	protein_coding	2	97490263	97523671	ANKRD23
ENSG00000163131	6.652556929	7.093659051	protein_coding	1	150702551	150738433	CTSS
ENSG00000163132	6.414691513	6.332882496	protein_coding	4	4861393	4865663	MSX1
ENSG00000163138	6.482942724	7.077141316	protein_coding	4	20697905	20754530	PACRGL
ENSG00000163141	2.928422289	3.133298822	protein_coding	1	151009046	151020076	BNIPL
ENSG00000163154	1.960915222	2.144285137	protein_coding	1	151129105	151132225	TNFAIP8L2
ENSG00000163155	6.851544845	6.988061092	protein_coding	1	151132224	151138424	LYSMD1
ENSG00000163156	8.465744027	8.9688072	protein_coding	1	151138498	151142773	SCNM1
ENSG00000163159	9.088754802	9.424191842	protein_coding	1	151142463	151167797	VPS72
ENSG00000163161	9.258993749	9.015499988	protein_coding	2	128014866	128051752	ERCC3
ENSG00000163162	9.038614381	8.501254594	protein_coding	2	101887681	101925163	RNF149
ENSG00000163166	9.665877245	9.254610923	protein_coding	2	128193783	128284462	IWS1
ENSG00000163170	6.155035276	6.963107483	protein_coding	2	74362525	74375121	BOLA3
ENSG00000163171	9.855452651	9.797285822	protein_coding	2	37869032	37965611	CDC42EP3
ENSG00000163191	12.38518684	12.72811878	protein_coding	1	152004982	152020383	S100A11
ENSG00000163207	1.16343121	0	protein_coding	1	152881021	152884362	IVL
ENSG00000163214	8.725846631	8.750768196	protein_coding	2	39024871	39103075	DHX57
ENSG00000163216	0.499066092	0	protein_coding	1	153012201	153014407	SPRR2D
ENSG00000163218	0	0.697730409	protein_coding	1	153302596	153321316	PGLYRP4
ENSG00000163220	6.26766507	4.547834947	protein_coding	1	153330330	153333503	S100A9
ENSG00000163221	0	0.390640832	protein_coding	1	153346184	153348125	S100A12
ENSG00000163235	8.274885464	5.629711952	protein_coding	2	70674412	70781325	TGFA
ENSG00000163249	9.320904342	8.308116953	protein_coding	2	208576264	208626563	CCNYL1
ENSG00000163251	7.546773135	6.835357159	protein_coding	2	208627310	208634287	FZD5
ENSG00000163257	8.631273684	8.985720419	protein_coding	4	17802278	17812381	DCAF16
ENSG00000163263	1.407729925	0	protein_coding	1	154171848	154178809	C1orf189
ENSG00000163281	7.08025234	7.201227369	protein_coding	4	44703885	44728612	GNPDA2
ENSG00000163283	0.499066092	0.950786998	protein_coding	2	233243244	233247599	ALPP
ENSG00000163291	7.943018447	8.200898549	protein_coding	4	79808281	79860592	PAQR3
ENSG00000163293	6.528867325	5.339632724	protein_coding	4	47916159	48042188	NIPAL1
ENSG00000163297	8.641761486	8.697730375	protein_coding	4	80822303	81046608	ANTXR2
ENSG00000163312	6.456028446	6.539915281	protein_coding	4	84328496	84377009	HELQ
ENSG00000163319	6.676071387	7.135740508	protein_coding	4	84377085	84390888	MRPS18C
ENSG00000163320	9.328343713	9.253876247	protein_coding	3	88101094	88199035	CGGBP1
ENSG00000163322	6.129712275	6.123616685	protein_coding	4	84382092	84444501	FAM175A
ENSG00000163328	3.911897206	4.250702764	protein_coding	2	175296966	175351822	GPR155

ENSG00000163344	8.858936753	8.927940468	protein_coding	1	154897210	154909467	PMVK
ENSG00000163346	8.53328596	9.042126408	protein_coding	1	154916552	154928599	PBXIP1
ENSG00000163347	10.36659374	10.83407108	protein_coding	3	190023490	190040264	CLDN1
ENSG00000163348	9.690621167	9.905093382	protein_coding	1	154929502	154936329	PYGO2
ENSG00000163349	9.2686931	9.113519788	protein_coding	1	114471814	114520426	HIPK1
ENSG00000163352	0.499066092	1.16600992	protein_coding	1	154966062	154966791	LENEP
ENSG00000163354	4.292665995	4.489113623	protein_coding	1	154990996	155006257	DCST2
ENSG00000163357	3.274897671	3.368666104	protein_coding	1	155006300	155023406	DCST1
ENSG00000163359	3.211941663	3.87606762	protein_coding	2	238232646	238323018	COL6A3
ENSG00000163362	8.42468852	8.431497548	protein_coding	1	200860176	200884863	C1orf106
ENSG00000163364	1.16343121	1.16600992	lincRNA	2	177494568	177502659	
ENSG00000163374	8.974866377	8.950786969	protein_coding	1	155629237	155658791	YY1AP1
ENSG00000163376	5.360713447	5.212303549	protein_coding	3	67048727	67061634	KBTBD8
ENSG00000163378	7.615275323	7.467704891	protein_coding	3	69024365	69063112	C3orf64
ENSG00000163380	0.499066092	1.518964905	protein_coding	3	69156023	69172183	LMOD3
ENSG00000163382	10.38944493	10.54345622	protein_coding	1	156561542	156564091	APOA1BP
ENSG00000163386	8.018542337	8.322202916	protein_coding	1	145289772	145370303	NBPF10
ENSG00000163389	7.356139979	7.668495823	protein_coding	3	119187785	119213555	POGLUT1
ENSG00000163393	4.77879189	5.393992	protein_coding	1	116519119	116612675	SLC22A15
ENSG00000163395	7.266040677	7.321103092	protein_coding	1	201159953	201198080	IGFN1
ENSG00000163399	12.24850143	11.94550623	protein_coding	1	116915290	116952883	ATP1A1
ENSG00000163406	3.146112541	3.781359661	protein_coding	3	121612936	121663034	SLC15A2
ENSG00000163412	3.989024711	3.993387124	protein_coding	3	71728440	71803924	EIF4E3
ENSG00000163428	9.547364003	9.314320673	protein_coding	3	120043356	120068186	LRRC58
ENSG00000163430	10.57208566	11.41228256	protein_coding	3	120111140	120170100	FSTL1
ENSG00000163431	2.471521042	0.950786998	protein_coding	1	201865580	201915716	LMOD1
ENSG00000163435	9.479658457	8.948972504	protein_coding	1	201977073	201986316	ELF3
ENSG00000163444	8.29020451	8.282410365	protein_coding	1	202976514	202993976	TMEM183A
ENSG00000163449	1.616589159	1.802319292	protein_coding	2	216946589	216967506	TMEM169
ENSG00000163453	5.301468762	7.666288907	protein_coding	4	57896939	57976551	IGFBP7
ENSG00000163462	4.6658819	5.35066967	protein_coding	1	155145873	155157447	TRIM46
ENSG00000163463	8.869173944	9.636922921	protein_coding	1	155141884	155159747	KRTCAP2
ENSG00000163464	0	0.390640832	protein_coding	2	219027568	219031725	CXCR1
ENSG00000163466	11.26814714	11.29612225	protein_coding	2	219081817	219119079	ARPC2
ENSG00000163467	3.146112541	4.342020395	protein_coding	1	156307105	156316786	C1orf182
ENSG00000163468	12.64005367	12.91688866	protein_coding	1	156278759	156337664	CCT3
ENSG00000163472	6.676071387	7.087074634	protein_coding	1	156252726	156262976	TMEM79
ENSG00000163479	11.33683831	11.63612717	protein_coding	1	155978839	155990750	SSR2
ENSG00000163481	7.615275323	7.652976045	protein_coding	2	219528587	219537134	RNF25
ENSG00000163482	8.369868254	9.005932523	protein_coding	2	219536749	219567439	STK36
ENSG00000163485	5.672682881	5.776071827	protein_coding	1	203059782	203136533	ADORA1
ENSG00000163486	9.856739382	9.753777222	protein_coding	1	206516198	206637783	SRGAP2
ENSG00000163491	3.274897671	4.048652948	protein_coding	3	27151576	27410951	NEK10
ENSG00000163492	2.238690726	2.501982735	protein_coding	2	179694484	179914813	CCDC141
ENSG00000163497	0	0.390640832	protein_coding	2	219845809	219850379	FEV
ENSG00000163499	2.57521082	3.279072565	protein_coding	2	219854911	219858143	CRYBA2
ENSG00000163501	0	0.950786998	protein_coding	2	219919146	219925189	IHH
ENSG00000163507	8.512210607	8.638170933	protein_coding	3	108268833	108308491	KIAA1524
ENSG00000163510	8.462367008	8.341695034	protein_coding	2	180809603	180871840	CWC22
ENSG00000163512	7.399163791	7.625976011	protein_coding	3	28356494	28390618	AZI2
ENSG00000163513	9.102921211	8.976843446	protein_coding	3	30647994	30735634	TGFBR2
ENSG00000163515	0.869158192	0.697730409	protein_coding	3	108462271	108476231	RETNLB
ENSG00000163516	8.996041004	9.57591961	protein_coding	2	220094479	220101391	ANKZF1
ENSG00000163517	7.308035767	6.877938671	protein_coding	3	13521224	13547916	HDAC11
ENSG00000163520	8.393111514	10.30894936	protein_coding	3	13573824	13679922	FBLN2
ENSG00000163521	5.91922012	5.863430791	protein_coding	2	220101328	220110200	GLB1L
ENSG00000163527	10.53197424	10.80978277	protein_coding	3	31574130	31679112	STT3B
ENSG00000163528	7.593829938	7.625976011	protein_coding	3	14153580	14166370	CHCHD4
ENSG00000163531	1.407729925	1.667587519	protein_coding	1	204797779	204991950	NFASC
ENSG00000163534	0	0.697730409	protein_coding	1	157764193	157789895	FCRL1
ENSG00000163535	8.479173607	8.524589435	protein_coding	2	201374731	201448505	SGOL2
ENSG00000163536	2.10647801	2.721932731	protein_coding	3	167453031	167543356	SERPINI1
ENSG00000163539	8.695964893	8.720244202	protein_coding	3	33537737	33759848	CLASP2

ENSG00000163541	9.579671796	9.945238564	protein_coding	2	84650647	84687169	SUCLG1
ENSG00000163545	8.839544001	8.913126522	protein_coding	1	205271187	205290883	NUAK2
ENSG00000163554	0.499066092	0.390640832	protein_coding	1	158580278	158656488	SPTA1
ENSG00000163558	9.319038489	8.932538804	protein_coding	3	169940153	170023769	PRKCI
ENSG00000163565	10.93178763	11.60059563	protein_coding	1	158969758	159024945	IFI16
ENSG00000163568	6.496213887	6.316088276	protein_coding	1	159032274	159116886	AIM2
ENSG00000163576	0.869158192	0.950786998	protein_coding	3	19920964	19988517	EFHB
ENSG00000163577	7.527512071	6.725179132	protein_coding	3	170606204	170626482	EIF5A2
ENSG00000163581	0	0.697730409	protein_coding	3	170714137	170744539	SLC2A2
ENSG00000163584	7.717966186	8.18248514	protein_coding	3	170582664	170588272	RPL22L1
ENSG00000163586	0	0.390640832	protein_coding	2	88422510	88427635	FABP1
ENSG00000163590	4.981125677	6.281901708	protein_coding	3	160473390	160796695	PPM1L
ENSG00000163596	4.491309013	5.32851069	protein_coding	2	203640690	203736708	ICA1L
ENSG00000163597	10.75054042	10.68261991	protein_coding	17	74553852	74561430	
ENSG00000163602	8.286389971	8.109639521	protein_coding	3	72423744	72496069	RYBP
ENSG00000163605	8.922987828	9.02758571	protein_coding	3	73045936	73118350	PPP4R2
ENSG00000163606	0.499066092	0.950786998	protein_coding	3	112640056	112693969	CD200R1
ENSG00000163607	6.364918751	6.695185789	protein_coding	3	112709765	112733907	GTPBP8
ENSG00000163608	8.70742151	8.658298522	protein_coding	3	112721287	112738708	C3orf17
ENSG00000163611	7.250461488	6.628734426	protein_coding	3	113161565	113234034	SPICE1
ENSG00000163612	0.869158192	0.950786998	pseudogene	4	9155022	9167177	
ENSG00000163617	6.252103507	6.3217081	protein_coding	3	113682984	113775460	KIAA1407
ENSG00000163623	1.616589159	1.925536307	protein_coding	4	85413061	85419603	NKX6-1
ENSG00000163624	5.938793061	3.183544561	protein_coding	4	85504132	85572491	CDS1
ENSG00000163625	8.616157604	8.929781562	protein_coding	4	85590704	85887544	WDFY3
ENSG00000163626	7.140657543	7.138927266	protein_coding	4	73921797	73935472	COX18
ENSG00000163629	7.381392865	7.23139676	protein_coding	4	87515468	87736324	PTPN13
ENSG00000163631	0.499066092	2.420525079	protein_coding	4	74262831	74287129	ALB
ENSG00000163632	1.16343121	1.16600992	protein_coding	3	63805038	63834312	C3orf49
ENSG00000163633	3.989024711	4.826083323	protein_coding	4	87797358	87857354	C4orf36
ENSG00000163634	8.582333986	8.89250574	protein_coding	3	63819546	63849579	THOC7
ENSG00000163635	8.120617457	8.253299769	protein_coding	3	63850233	63989138	ATXN7
ENSG00000163636	9.135091581	9.635231414	protein_coding	3	63996225	64009658	PSMD6
ENSG00000163637	7.464764762	8.098224647	protein_coding	3	64079543	64431152	PRICKLE2
ENSG00000163638	8.865343505	9.213637661	protein_coding	3	64501333	64673676	ADAMTS9
ENSG00000163644	6.469548347	6.601272494	protein_coding	4	89183315	89205921	PPM1K
ENSG00000163645	0	0.950786998	protein_coding	3	150377672	150421758	FAM194A
ENSG00000163655	10.37470026	10.22971271	protein_coding	3	155588325	155658457	GMPS
ENSG00000163659	9.25411951	8.891561392	protein_coding	3	156391024	156424559	TIPARP
ENSG00000163660	10.39344018	9.681859685	protein_coding	3	156864297	156878549	CCNL1
ENSG00000163661	9.796999249	10.65811888	protein_coding	3	157154578	157161417	PTX3
ENSG00000163666	3.004694206	2.788380093	protein_coding	3	57231944	57260549	HESX1
ENSG00000163681	9.842521827	9.596348809	protein_coding	3	57741177	57914895	SLMAP
ENSG00000163682	7.491518048	8.298174303	protein_coding	4	39455744	39460568	RPL9
ENSG00000163683	6.820228284	7.154756319	protein_coding	4	39552541	39640710	C4orf34
ENSG00000163684	2.928422289	2.721932731	protein_coding	3	58291974	58305816	RPP14
ENSG00000163686	6.693459083	6.365895578	protein_coding	3	58223233	58281420	ABHD6
ENSG00000163689	6.086493765	5.871116342	protein_coding	3	58703092	59035810	C3orf67
ENSG00000163694	8.99837467	7.817139602	protein_coding	4	40425272	40632892	RBM47
ENSG00000163697	8.937654214	8.768365845	protein_coding	4	40812044	41218731	APBB2
ENSG00000163701	4.19837882	2.242360793	protein_coding	3	9944296	9958086	IL17RE
ENSG00000163702	8.229914881	8.193254836	protein_coding	3	9958758	9975314	IL17RC
ENSG00000163703	7.178195136	7.182818183	protein_coding	3	9975506	9987097	CRELD1
ENSG00000163704	4.863159938	4.841813558	protein_coding	3	9987226	9994078	PRRT3
ENSG00000163705	1.16343121	0.950786998	protein_coding	3	10123001	10149915	C3orf24
ENSG00000163710	5.879259127	6.637773454	protein_coding	3	142534764	142608045	PCOLCE2
ENSG00000163714	10.53840334	10.79496056	protein_coding	3	142720010	142779567	U2SURP
ENSG00000163719	8.547698305	8.552341023	protein_coding	3	9691117	9744077	MTMR14
ENSG00000163728	7.494827594	8.061749803	protein_coding	3	180319918	180335616	TTC14
ENSG00000163734	6.275383355	6.403479675	protein_coding	4	74902306	74904524	CXCL3
ENSG00000163735	8.077121473	8.69988975	protein_coding	4	74861359	74864496	CXCL5
ENSG00000163738	6.350376764	6.99159092	protein_coding	4	74979891	75168816	MTHFD2L
ENSG00000163739	10.1242235	11.09771094	protein_coding	4	74735110	74736959	CXCL1

ENSG00000163743	7.04004079	7.106738413	protein_coding	4	76404247	76439974	RCHY1
ENSG00000163746	0.499066092	0	protein_coding	3	146109208	146213778	PLSCR2
ENSG00000163749	0.499066092	0	protein_coding	4	77234154	77343021	CCDC158
ENSG00000163754	8.515473076	8.49131535	protein_coding	3	148709128	148745419	GYG1
ENSG00000163755	9.517573819	9.008548131	protein_coding	3	148847371	148891519	HPS3
ENSG00000163762	4.842525946	1.16600992	protein_coding	3	149036285	149052201	TM4SF18
ENSG00000163781	9.673199027	9.574155001	protein_coding	3	133317019	133380737	TOPBP1
ENSG00000163785	9.40334477	9.317839931	protein_coding	3	133794023	133969689	RYK
ENSG00000163788	7.04004079	7.087074634	protein_coding	3	43328004	43466256	SNRK
ENSG00000163793	1.16343121	0.950786998	protein_coding	2	27498289	27504367	DNAJC5G
ENSG00000163794	3.78787645	3.813624741	protein_coding	2	27530268	27531313	UCN
ENSG00000163795	6.050961887	5.717592784	protein_coding	2	27600098	27603657	ZNF513
ENSG00000163798	8.555643385	8.615472585	protein_coding	2	27886338	27917840	SLC4A1AP
ENSG00000163803	3.274897671	2.788380093	protein_coding	2	28680012	28866654	PLB1
ENSG00000163806	3.004694206	3.87606762	protein_coding	2	29005383	29073477	SPDYA
ENSG00000163807	8.3053626	8.444434101	protein_coding	3	44779153	44803154	KIAA1143
ENSG00000163808	8.129161475	8.378559146	protein_coding	3	44803209	44914868	KIF15
ENSG00000163810	3.652182994	3.411459265	protein_coding	3	44916100	44956482	TGM4
ENSG00000163811	10.21280407	10.28793047	protein_coding	2	29117509	29171088	WDR43
ENSG00000163812	9.36407124	9.097229165	protein_coding	3	44956749	45017677	ZDHHC3
ENSG00000163814	9.582785382	10.02620285	protein_coding	3	45123770	45187914	CDCP1
ENSG00000163815	1.407729925	1.667587519	protein_coding	3	45043040	45077563	CLEC3B
ENSG00000163817	1.407729925	0.950786998	protein_coding	3	45796942	45838027	SLC6A20
ENSG00000163818	7.066972635	7.213370937	protein_coding	3	45864808	45957534	LZTFL1
ENSG00000163820	9.199386995	9.228672011	protein_coding	3	45959396	46037316	FYCO1
ENSG00000163823	1.16343121	1.518964905	protein_coding	3	46243200	46249887	CCR1
ENSG00000163825	1.616589159	3.232099092	protein_coding	3	46538981	46542439	RTP3
ENSG00000163827	0.499066092	0	protein_coding	3	46556913	46621589	LRRC2
ENSG00000163832	7.743231983	8.24888207	protein_coding	3	47537130	47555251	C3orf75
ENSG00000163833	0.499066092	0.390640832	protein_coding	3	121311966	121349139	FBXO40
ENSG00000163840	9.648894781	9.741251102	protein_coding	3	122283085	122294050	DTX3L
ENSG00000163848	8.888175056	8.674859633	protein_coding	3	124944405	125094198	ZNF148
ENSG00000163864	1.799000381	0.950786998	protein_coding	3	139279022	139396859	NMNAT3
ENSG00000163866	7.962273293	8.196317182	protein_coding	1	35178338	35325417	C1orf212
ENSG00000163867	7.568924112	7.797107849	protein_coding	1	35447134	35497569	ZMYM6
ENSG00000163870	9.49544012	9.705158168	protein_coding	3	127291912	127317094	TPRA1
ENSG00000163872	9.775389681	9.647589989	protein_coding	3	183415606	183530413	YEATS2
ENSG00000163873	0.499066092	0	protein_coding	1	37261128	37499730	GRIK3
ENSG00000163874	10.0326953	9.653729801	protein_coding	1	37940153	37949978	ZC3H12A
ENSG00000163875	8.131289597	8.188649093	protein_coding	1	37958176	37980375	MEAF6
ENSG00000163877	6.970425921	7.050313162	protein_coding	1	38002142	38019905	SNIP1
ENSG00000163879	0.499066092	1.518964905	protein_coding	1	38022520	38032458	DNALI1
ENSG00000163882	9.553725034	9.636359306	protein_coding	3	184079506	184086384	POLR2H
ENSG00000163884	1.616589159	2.579085888	protein_coding	3	126061478	126076285	KLF15
ENSG00000163885	1.16343121	2.144285137	protein_coding	3	126113782	126155399	CCDC37
ENSG00000163888	5.054730169	4.65855978	protein_coding	3	183977001	183979251	CAMK2N2
ENSG00000163898	7.097770581	7.016060809	protein_coding	3	185224050	185270401	LIPH
ENSG00000163900	8.169068555	7.869875863	protein_coding	3	185194284	185216845	TMEM41A
ENSG00000163902	11.56336037	11.50642848	protein_coding	3	128338817	128399918	RPN1
ENSG00000163904	8.958179637	8.455978641	protein_coding	3	185300284	185351339	SENP2
ENSG00000163909	5.072559152	4.961837817	protein_coding	1	40089825	40105617	HEYL
ENSG00000163913	8.515473076	8.289597152	protein_coding	3	129158968	129239198	IFT122
ENSG00000163914	0.499066092	0	protein_coding	3	129247483	129254012	RHO
ENSG00000163915	1.960915222	1.802319292	protein_coding	3	185430316	185447575	C3orf65
ENSG00000163918	9.024943975	9.109464345	protein_coding	3	186507669	186524847	RFC4
ENSG00000163923	8.261346552	8.334763756	protein_coding	3	186838736	186898696	RPL39L
ENSG00000163930	10.01896842	9.782588703	protein_coding	3	52435029	52444366	BAP1
ENSG00000163931	11.94373102	11.64568034	protein_coding	3	53258723	53290068	TKT
ENSG00000163932	8.133414584	8.026050991	protein_coding	3	53190025	53226733	PRKCD
ENSG00000163933	8.041355121	8.135396417	protein_coding	3	53122499	53164478	RFT1
ENSG00000163935	8.429884808	8.69123279	protein_coding	3	52937693	53080766	SFMBT1
ENSG00000163938	10.23424851	10.40902214	protein_coding	3	52715172	52728508	GNL3
ENSG00000163939	8.297803463	8.649387576	protein_coding	3	52579368	52719933	PBRM1

ENSG00000163945	7.488200893	7.343375265	protein_coding	4	1341054	1381837	KIAA1530
ENSG00000163946	10.2752422	9.99840118	protein_coding	3	56654161	56717265	FAM208A
ENSG00000163947	8.12703021	7.524852175	protein_coding	3	56761446	57113357	ARHGEF3
ENSG00000163950	9.201414078	9.23613084	protein_coding	4	1694527	1714282	SLBP
ENSG00000163956	9.630205981	10.24050771	protein_coding	4	3508103	3534286	LRPAP1
ENSG00000163958	0.869158192	0.390640832	protein_coding	3	195924320	195938308	ZDHHC19
ENSG00000163959	4.903563137	5.236305879	protein_coding	3	195938358	195970049	
ENSG00000163960	9.875903885	9.607318979	protein_coding	3	196074533	196159345	UBXN7
ENSG00000163961	9.207478296	8.668257984	protein_coding	3	196195654	196230639	RNF168
ENSG00000163964	8.213938461	7.799123587	protein_coding	3	196366646	196462878	PIGX
ENSG00000163975	10.42717699	10.15298033	protein_coding	3	196715492	196756687	MFI2
ENSG00000163993	3.698846687	2.039052734	protein_coding	4	6694796	6698897	S100P
ENSG00000163995	5.054730169	4.693656882	protein_coding	4	7967039	8160559	ABLIM2
ENSG00000164002	5.858855738	5.938503778	protein_coding	1	40974413	40982228	DEM1
ENSG00000164007	0.499066092	0	protein_coding	1	43198764	43205925	CLDN19
ENSG00000164008	4.491309013	5.236305879	protein_coding	1	43232940	43264127	C1orf50
ENSG00000164010	5.360713447	6.104183162	protein_coding	1	43282795	43310660	ERMAP
ENSG00000164011	5.741167503	6.450406542	protein_coding	1	43312280	43318148	ZNF691
ENSG00000164022	8.628263112	8.967911514	protein_coding	4	107236701	107270383	AIMP1
ENSG00000164023	8.70742151	8.471229048	protein_coding	4	108745719	108836203	SGMS2
ENSG00000164024	9.369490093	9.450720858	protein_coding	4	99916771	99983964	METAP1
ENSG00000164031	8.213938461	8.234057939	protein_coding	4	100817405	100867883	DNAJB14
ENSG00000164032	10.88115523	11.49792174	protein_coding	4	100869243	100871545	H2AFZ
ENSG00000164035	0	0.390640832	protein_coding	4	101316498	101801283	EMCN
ENSG00000164037	1.960915222	1.16600992	protein_coding	4	103806205	103940896	SLC9B1
ENSG00000164038	6.825495146	7.272601283	protein_coding	4	103941025	104006986	SLC9B2
ENSG00000164039	4.842525946	6.077857763	protein_coding	4	104000592	104021040	BDH2
ENSG00000164040	9.3522601	9.87244022	protein_coding	4	129190392	129209984	PGRMC2
ENSG00000164045	8.137655191	7.830998152	protein_coding	3	48198636	48229892	CDC25A
ENSG00000164048	7.15746161	7.635032283	protein_coding	3	48282590	48340743	ZNF589
ENSG00000164049	1.16343121	0.390640832	protein_coding	3	48413709	48442666	FBXW12
ENSG00000164050	8.37885234	7.749951106	protein_coding	3	48445261	48471594	PLXNB1
ENSG00000164051	7.712290964	7.696882678	protein_coding	3	48473574	48481866	CCDC51
ENSG00000164053	7.575190993	7.357122767	protein_coding	3	48488114	48507115	ATRIP
ENSG00000164054	10.54600086	10.58639925	protein_coding	3	48509197	48542259	SHISA5
ENSG00000164056	5.909333162	5.960284481	protein_coding	4	124317950	124324910	SPRY1
ENSG00000164061	5.498982484	5.683080141	protein_coding	3	49591922	49708978	BSN
ENSG00000164062	10.29862807	10.19377319	protein_coding	3	49711435	49721396	APEH
ENSG00000164066	5.806547963	6.204928885	protein_coding	4	128544426	128637930	INTU
ENSG00000164068	8.826469148	8.726612716	protein_coding	3	49726932	49758962	RNF123
ENSG00000164070	8.229914881	8.594734178	protein_coding	4	128702976	128755226	HSPA4L
ENSG00000164073	6.554468172	6.941369681	protein_coding	4	128838960	128887150	MFSD8
ENSG00000164074	6.320845526	6.440109466	protein_coding	4	128886435	128960866	C4orf29
ENSG00000164076	0.499066092	0	protein_coding	3	49895421	49907655	CAMKV
ENSG00000164077	7.587643674	7.793067905	protein_coding	3	49946302	49967606	MON1A
ENSG00000164078	8.794597401	7.760334499	protein_coding	3	49924435	49941299	MST1R
ENSG00000164080	7.058051099	6.843193319	protein_coding	3	51575596	51697610	RAD54L2
ENSG00000164081	7.748787017	8.177844832	protein_coding	3	51696709	51738339	TEX264
ENSG00000164082	1.960915222	0.950786998	protein_coding	3	51741086	51752629	GRM2
ENSG00000164086	9.061114416	8.289597152	protein_coding	3	52082935	52090566	DUSP7
ENSG00000164087	7.850461307	8.070120888	protein_coding	3	52109269	52188706	POC1A
ENSG00000164088	6.005279909	6.25865215	protein_coding	3	52279841	52284613	PPM1M
ENSG00000164089	0.499066092	0	protein_coding	4	109663196	109684210	AGXT2L1
ENSG00000164091	10.01839363	9.606743677	protein_coding	3	52288437	52322036	WDR82
ENSG00000164096	8.861502869	9.632407821	protein_coding	4	120218207	120225600	C4orf3
ENSG00000164099	3.393122761	0.697730409	protein_coding	4	119201193	119274158	PRSS12
ENSG00000164104	9.992287818	10.14071283	protein_coding	4	174252846	174256276	HMGB2
ENSG00000164105	7.049074049	8.188649093	protein_coding	4	174291120	174298683	SAP30
ENSG00000164106	0.499066092	1.667587519	protein_coding	4	174309299	174327531	SCRG1
ENSG00000164109	8.48251156	8.98660512	protein_coding	4	120980577	120988229	MAD2L1
ENSG00000164111	11.42405136	11.92742111	protein_coding	4	122589110	122618268	ANXA5
ENSG00000164114	7.514527001	8.613182984	protein_coding	4	156263810	156298122	MAP9
ENSG00000164116	0.499066092	3.232099092	protein_coding	4	156587863	156653501	GUCY1A3

ENSG00000164117	6.462804234	6.827478203	protein_coding	4	175157809	175205415	FBXO8
ENSG00000164118	6.882196036	7.292770473	protein_coding	4	175204828	175254531	CEP44
ENSG00000164120	2.238690726	0.697730409	protein_coding	4	175411328	175444305	HPGD
ENSG00000164123	0	0.390640832	protein_coding	4	159814286	159959912	C4orf45
ENSG00000164124	5.977159882	5.751299057	protein_coding	4	159122756	159176563	TMEM144
ENSG00000164125	6.068837212	4.448594745	protein_coding	4	159045626	159094470	FAM198B
ENSG00000164128	0	0.697730409	protein_coding	4	164245113	164265984	NPY1R
ENSG00000164134	10.11134422	9.853651825	protein_coding	4	140222609	140341187	NAA15
ENSG00000164136	5.207774174	6.016808232	protein_coding	4	142557752	142655140	IL15
ENSG00000164142	7.007971391	6.742042315	protein_coding	4	152330368	152584784	FAM160A1
ENSG00000164144	8.560389515	8.884933592	protein_coding	4	153701089	153839615	ARFIP1
ENSG00000164151	10.31276107	10.11626465	protein_coding	5	5420777	5490347	KIAA0947
ENSG00000164162	5.360713447	5.583678991	protein_coding	4	145888264	146019693	ANAPC10
ENSG00000164163	10.25976259	10.28073536	protein_coding	4	146019084	146050331	ABCE1
ENSG00000164164	9.565576897	9.889095769	protein_coding	4	146031990	146101313	OTUD4
ENSG00000164167	6.704935538	7.154756319	protein_coding	4	147096837	147121152	LSM6
ENSG00000164168	8.079327701	8.055017786	protein_coding	4	148538534	148593195	TMEM184C
ENSG00000164169	7.049074049	7.18590274	protein_coding	4	148558936	148605381	PRMT10
ENSG00000164171	10.07973621	8.997179425	protein_coding	5	52285156	52390609	ITGA2
ENSG00000164172	7.908691438	8.17939326	protein_coding	5	52391512	52405893	MOCS2
ENSG00000164176	9.91224858	6.775188634	protein_coding	5	83236373	83680611	EDIL3
ENSG00000164180	7.153278907	6.95589788	protein_coding	5	87485450	87565293	TMEM161B
ENSG00000164181	3.211941663	2.579085888	protein_coding	5	60047618	60140216	ELOVL7
ENSG00000164182	8.079327701	8.496293532	protein_coding	5	60240956	60448853	NDUFAF2
ENSG00000164185	0.499066092	0.697730409	protein_coding	5	121465208	121515312	ZNF474
ENSG00000164187	8.13977083	7.883239271	protein_coding	5	36098514	36152063	LMBRD2
ENSG00000164188	0.499066092	1.667587519	protein_coding	5	36248536	36302216	RANBP3L
ENSG00000164190	10.5444047	10.40505707	protein_coding	5	36876861	37066515	NIPBL
ENSG00000164197	0.499066092	0	protein_coding	5	63461671	63668696	RNF180
ENSG00000164199	5.774228067	5.57429348	protein_coding	5	89825161	90460038	GPR98
ENSG00000164209	9.096400149	9.166009895	protein_coding	5	110073837	110100857	SLC25A46
ENSG00000164211	8.826469148	8.301022059	protein_coding	5	110831731	110848288	STARD4
ENSG00000164219	7.46813618	6.870289474	protein_coding	5	114546527	114598569	PGGT1B
ENSG00000164220	1.16343121	0.950786998	protein_coding	5	75911328	75919259	F2RL2
ENSG00000164221	7.007971391	6.376734172	protein_coding	5	114602885	114632528	CCDC112
ENSG00000164236	5.124761037	3.96493948	protein_coding	5	10564442	10650308	ANKRD33B
ENSG00000164237	8.217949183	5.691786012	protein_coding	5	10275987	10308138	CMBL
ENSG00000164241	3.335220907	3.081239798	protein_coding	5	126378250	126409184	C5orf63
ENSG00000164244	9.560847829	9.376390077	protein_coding	5	126853301	126890782	PRRC1
ENSG00000164251	7.888702948	5.32851069	protein_coding	5	76114758	76131140	F2RL1
ENSG00000164252	8.518728184	8.351343215	protein_coding	5	76325076	76361059	AGGF1
ENSG00000164253	8.408986408	8.171634445	protein_coding	5	76721795	76916436	WDR41
ENSG00000164256	0.499066092	0	protein_coding	5	23507264	23528706	PRDM9
ENSG00000164258	7.67484765	8.122575449	protein_coding	5	52856463	52979168	NDUFS4
ENSG00000164265	0	0.697730409	protein_coding	5	147250245	147261754	SCGB3A2
ENSG00000164266	0.499066092	1.925536307	protein_coding	5	147204131	147211349	SPINK1
ENSG00000164284	7.689364157	7.743685008	protein_coding	5	148724993	148734146	GRPEL2
ENSG00000164287	0.499066092	0.390640832	protein_coding	5	54408799	54469005	CDC20B
ENSG00000164291	6.887241849	6.835357159	protein_coding	5	94890778	94940768	ARSK
ENSG00000164292	10.13221505	9.809336738	protein_coding	5	95049226	95160087	RHOBTB3
ENSG00000164294	8.992533411	9.268499153	protein_coding	5	54455946	54462899	GPX8
ENSG00000164296	5.417620896	5.620622115	protein_coding	5	149372681	149380730	TIGD6
ENSG00000164300	5.889353623	5.150490275	protein_coding	5	79407050	79551898	SERINC5
ENSG00000164303	2.928422289	4.101879561	protein_coding	4	185009859	185142383	ENPP6
ENSG00000164304	0.499066092	0.697730409	protein_coding	6	7326887	7389976	CAGE1
ENSG00000164305	8.109865782	8.758040301	protein_coding	4	185548850	185570663	CASP3
ENSG00000164306	6.35766608	6.877938671	protein_coding	4	185570767	185616117	CCDC111
ENSG00000164307	8.794597401	9.266315144	protein_coding	5	96096521	96143803	ERAP1
ENSG00000164308	9.882235894	9.430057217	protein_coding	5	96211643	96255420	ERAP2
ENSG00000164309	1.799000381	1.353254395	protein_coding	5	78985700	79096063	CMYA5
ENSG00000164323	8.944931872	9.179226353	protein_coding	4	186080819	186130658	KIAA1430
ENSG00000164327	9.902936511	9.705695541	protein_coding	5	38938021	39074510	RICTOR
ENSG00000164329	8.233881494	8.262094789	protein_coding	5	78907943	78982471	PAPD4

ENSG00000164330	0	0.950786998	protein_coding	5	158122924	158526788	EBF1
ENSG00000164331	5.977159882	6.403479675	protein_coding	5	72848160	72861511	ANKRA2
ENSG00000164332	7.943018447	7.969895325	protein_coding	5	158690089	158713044	UBLCP1
ENSG00000164338	8.193715877	7.960920138	protein_coding	5	72861268	72877794	UTP15
ENSG00000164342	6.35766608	6.535085617	protein_coding	4	186990306	187006255	TLR3
ENSG00000164344	0.499066092	2.501982735	protein_coding	4	187130133	187179625	KLKB1
ENSG00000164346	8.396654395	8.812940295	protein_coding	5	74062817	74072737	NSA2
ENSG00000164347	8.732871027	8.855210283	protein_coding	5	74017029	74063196	GFM2
ENSG00000164362	6.023726639	6.605885938	protein_coding	5	1253262	1295184	TERT
ENSG00000164363	0.499066092	0	protein_coding	5	1225470	1246304	SLC6A18
ENSG00000164366	7.827020104	8.379906558	protein_coding	5	204872	218330	CCDC127
ENSG00000164379	5.485736881	0	protein_coding	6	1312675	1314992	FOXQ1
ENSG00000164385	0.869158192	1.518964905	protein_coding	6	2622147	2634837	C6orf195
ENSG00000164393	1.799000381	0	protein_coding	6	47624172	47665533	GPR111
ENSG00000164398	0.869158192	0.950786998	protein_coding	5	131142683	131347936	ACSL6
ENSG00000164399	0	0.390640832	protein_coding	5	131396222	131398897	IL3
ENSG00000164400	7.169937448	8.487570409	protein_coding	5	131409483	131411859	CSF2
ENSG00000164402	9.216527094	9.240587696	protein_coding	5	132086509	132142933	8-sep
ENSG00000164403	7.945439403	8.103127799	protein_coding	5	132157833	132166590	SHROOM1
ENSG00000164404	2.238690726	1.925536307	protein_coding	5	132196873	132202576	GDF9
ENSG00000164405	8.724437638	9.237617989	protein_coding	5	132202252	132203723	UQCRQ
ENSG00000164406	3.554067925	3.411459265	protein_coding	5	132208014	132210738	LEAP2
ENSG00000164411	4.689181911	4.917990497	protein_coding	6	87992696	88038996	GJB7
ENSG00000164414	5.967663407	6.009862691	protein_coding	6	88180341	88222054	SLC35A1
ENSG00000164418	5.090170488	5.57429348	protein_coding	6	101846664	102517958	GRIK2
ENSG00000164430	7.868433623	7.950075687	protein_coding	6	74123238	74161999	MB21D1
ENSG00000164442	9.168633912	8.855210283	protein_coding	6	139693393	139695757	CITED2
ENSG00000164463	6.050961887	6.703819132	protein_coding	5	172483355	172566291	C5orf41
ENSG00000164465	7.888702948	7.915187059	protein_coding	6	117774980	117891021	DCBLD1
ENSG00000164466	9.608983025	9.266315144	protein_coding	5	174904065	174956745	SFXN1
ENSG00000164485	0.499066092	1.518964905	protein_coding	6	137464968	137494785	IL22RA2
ENSG00000164494	7.565780433	7.20731193	protein_coding	6	107473761	107780768	PDSS2
ENSG00000164506	8.08373006	8.122575449	protein_coding	6	147525508	147708707	STXBP5
ENSG00000164509	6.372135143	5.09905904	protein_coding	5	55147207	55218678	IL31RA
ENSG00000164512	1.799000381	1.802319292	protein_coding	5	55395507	55529186	ANKRD55
ENSG00000164520	3.744048221	2.971122874	protein_coding	6	150204511	150219238	RAET1E
ENSG00000164530	2.57521082	1.353254395	protein_coding	6	36555311	36932613	PI16
ENSG00000164535	8.89695732	8.695567763	protein_coding	7	6448757	6523821	DAGLB
ENSG00000164542	7.345180517	7.423961341	protein_coding	7	36363830	36429734	KIAA0895
ENSG00000164543	9.867630505	9.516516751	protein_coding	7	43622357	43666385	STK17A
ENSG00000164548	9.006512903	9.258278703	protein_coding	7	23544399	23571660	TRA2A
ENSG00000164556	1.407729925	1.353254395	protein_coding	7	38724946	38726637	FAM183B
ENSG00000164574	10.77558713	10.43226174	protein_coding	5	153570290	153800544	GALNT10
ENSG00000164576	6.032862302	6.293387477	protein_coding	5	153825517	153835891	SAP30L
ENSG00000164587	11.66105046	12.35689303	protein_coding	5	149822753	149829319	RPS14
ENSG00000164588	0.499066092	0	protein_coding	5	45259349	45696253	HCN1
ENSG00000164591	1.799000381	1.667587519	protein_coding	5	150040436	150058927	MYOZ3
ENSG00000164597	9.058880138	8.606292299	protein_coding	7	106842189	107204959	COG5
ENSG00000164603	6.442380648	6.505763096	protein_coding	7	112459202	112579971	C7orf60
ENSG00000164609	8.362640485	8.516036237	protein_coding	5	159828648	159848718	SLU7
ENSG00000164610	5.684325135	5.840125386	protein_coding	7	33134409	33149013	RP9
ENSG00000164611	8.450484798	8.916844293	protein_coding	5	159848829	159855748	PTTG1
ENSG00000164615	8.059349131	8.203944731	protein_coding	5	134074191	134087847	CAMLG
ENSG00000164619	6.541724534	3.96493948	protein_coding	7	33944523	34195484	BMPER
ENSG00000164620	5.538004898	4.826083323	protein_coding	5	141016517	141020644	RELL2
ENSG00000164621	0.499066092	0.950786998	antisense	5	135465196	135470579	SMAD5-AS1
ENSG00000164626	8.309127368	7.20731193	protein_coding	6	39156749	39197226	KCNK5
ENSG00000164627	1.616589159	0.950786998	protein_coding	6	39297766	39693181	KIF6
ENSG00000164631	8.135536446	7.996490656	protein_coding	7	6728064	6746620	ZNF12
ENSG00000164638	6.379315619	4.961837817	protein_coding	7	5314000	5343696	SLC29A4
ENSG00000164647	7.627388205	7.772696723	protein_coding	7	89783689	89794143	STEAP1
ENSG00000164649	9.465390183	9.153459292	protein_coding	7	21940518	21985702	CDCA7L
ENSG00000164651	3.698846687	2.721932731	protein_coding	7	20821903	20826505	SP8

ENSG00000164654	8.672775055	8.635917101	protein_coding	7	7606503	7648560	MIOS
ENSG00000164659	0	0.390640832	protein_coding	7	86506222	86689015	KIAA1324L
ENSG00000164663	6.138202758	6.637773454	protein_coding	6	41762107	41863099	USP49
ENSG00000164669	1.960915222	2.652276565	pseudogene	7	64601603	64694600	INTS4L1
ENSG00000164674	6.442380648	4.566889334	protein_coding	6	159071046	159185908	SYTL3
ENSG00000164675	1.16343121	0.950786998	protein_coding	7	123092454	123175131	IQUB
ENSG00000164683	3.077135474	4.075511708	protein_coding	8	80676245	80680098	HEY1
ENSG00000164684	4.437288965	6.941369681	protein_coding	8	81540686	81787016	ZNF704
ENSG00000164687	4.19837882	5.236305879	protein_coding	8	82192598	82197012	FABP5
ENSG00000164690	3.78787645	3.232099092	protein_coding	7	155592680	155604967	SHH
ENSG00000164691	1.407729925	1.518964905	protein_coding	6	159455500	159466184	TAGAP
ENSG00000164692	11.5729824	8.874456566	protein_coding	7	94023873	94060544	COL1A2
ENSG00000164694	0	0.390640832	protein_coding	6	159590429	159693141	FNDC1
ENSG00000164695	6.835971536	5.317302248	protein_coding	8	82644669	82671750	CHMP4C
ENSG00000164707	4.75690578	4.810179682	protein_coding	7	135365985	135412952	SLC13A4
ENSG00000164713	9.684112495	9.558175629	protein_coding	7	97881691	97922275	BRI3
ENSG00000164715	9.51676008	8.599368544	protein_coding	7	97736197	97838945	LMTK2
ENSG00000164729	0.499066092	1.353254395	protein_coding	17	33519539	33521412	SLC35G3
ENSG00000164733	11.80913707	12.50139115	protein_coding	8	11700033	11726957	CTSB
ENSG00000164741	9.186141067	8.522150834	protein_coding	8	12940870	13373167	DLC1
ENSG00000164742	0	0.697730409	protein_coding	7	45613739	45762715	ADCY1
ENSG00000164744	5.741167503	6.316088276	protein_coding	7	48026745	48068716	SUN3
ENSG00000164746	2.762599152	1.518964905	protein_coding	7	48075108	48100901	C7orf57
ENSG00000164749	1.16343121	2.242360793	protein_coding	8	76320149	76479078	HNF4G
ENSG00000164751	8.241782151	8.312357201	protein_coding	8	77892494	77913280	PEX2
ENSG00000164754	10.80120923	10.6252633	protein_coding	8	117858174	117887105	RAD21
ENSG00000164758	6.103936836	5.988823159	protein_coding	8	118532952	118552501	MED30
ENSG00000164761	4.923348194	5.306006045	protein_coding	8	119935796	119964439	TNFRSF11B
ENSG00000164764	3.274897671	2.851901313	protein_coding	8	73976775	74036323	C8orf84
ENSG00000164776	4.409500985	3.906303962	protein_coding	7	56148440	56160689	PHKG1
ENSG00000164778	6.146643566	5.974624386	protein_coding	7	155250824	155257526	EN2
ENSG00000164808	8.235860718	8.496293532	protein_coding	8	48173167	48648868	KIAA0146
ENSG00000164815	7.651313074	7.902117654	protein_coding	7	103766788	103848495	ORC5
ENSG00000164818	10.15488184	9.775951422	protein_coding	7	766338	829190	HEATR2
ENSG00000164821	0.499066092	0	protein_coding	8	6793344	6795860	DEFA4
ENSG00000164823	8.072698871	7.600774083	protein_coding	8	90914087	90940116	OSGIN2
ENSG00000164825	1.16343121	0	protein_coding	8	6728097	6735544	DEFB1
ENSG00000164828	10.90902113	10.61242459	protein_coding	7	855528	936072	SUN1
ENSG00000164830	8.016241092	7.951888765	protein_coding	8	107282473	107764922	OXR1
ENSG00000164845	2.35979773	2.144285137	pseudogene	12	8385108	8395544	FAM86FP
ENSG00000164849	2.671945279	3.532712221	protein_coding	7	1084212	1098897	GPR146
ENSG00000164850	7.053569556	7.073814954	protein_coding	7	1121844	1133451	GPER
ENSG00000164855	7.062518763	6.097646709	protein_coding	7	1585796	1600457	TMEM184A
ENSG00000164867	7.030950614	7.192052139	protein_coding	7	150688083	150711676	NOS3
ENSG00000164877	10.05584741	10.05727354	protein_coding	7	1468101	1499138	MICALL2
ENSG00000164879	0	1.518964905	protein_coding	8	86285665	86361269	CA3
ENSG00000164880	11.92973808	11.46115805	protein_coding	7	1509913	1545489	INTS1
ENSG00000164885	7.413224588	7.529716161	protein_coding	7	150750899	150755617	CDK5
ENSG00000164889	11.24524063	11.37661838	protein_coding	7	150754297	150773614	SLC4A2
ENSG00000164896	9.805686507	9.491804167	protein_coding	7	150773679	150777953	FASTK
ENSG00000164897	8.871721931	8.535512415	protein_coding	7	150778167	150780633	TMUB1
ENSG00000164898	4.131913373	4.7279204	protein_coding	7	139024203	139031065	C7orf55
ENSG00000164900	4.19837882	5.283144778	protein_coding	7	150845676	150871832	GBX1
ENSG00000164902	8.389559911	8.363653959	protein_coding	5	125935960	125962944	PHAX
ENSG00000164904	8.809292739	8.908465794	protein_coding	5	125877533	125931110	ALDH7A1
ENSG00000164916	11.24007313	10.22484723	protein_coding	7	4683388	4811074	FOXK1
ENSG00000164919	8.698837588	9.119178324	protein_coding	8	100885428	100906290	COX6C
ENSG00000164920	4.75690578	3.781359661	protein_coding	8	99956631	99964332	OSR2
ENSG00000164924	12.66331162	11.96942156	protein_coding	8	101928753	101965616	YWHAZ
ENSG00000164929	0	1.353254395	protein_coding	8	104152938	104242533	BAALC
ENSG00000164930	8.605481314	8.786767612	protein_coding	8	104310661	104345094	FZD6
ENSG00000164932	7.81647827	8.898158883	protein_coding	8	104383743	104395225	CTHRC1
ENSG00000164933	8.536501169	8.043160634	protein_coding	8	104410863	104427417	SLC25A32

ENSG00000164934	9.368588363	9.079094423	protein_coding	8	104426942	104455681	DCAF13
ENSG00000164938	5.223818625	5.893930351	protein_coding	8	95938200	95961639	TP53INP1
ENSG00000164941	8.641761486	8.402622096	protein_coding	8	95825539	95893974	INTS8
ENSG00000164944	9.541775001	9.381107599	protein_coding	8	95499921	95565757	KIAA1429
ENSG00000164946	4.543379137	7.100213554	protein_coding	9	14734664	14910993	FREM1
ENSG00000164949	5.637181576	4.319727133	protein_coding	8	95261481	95274578	GEM
ENSG00000164951	10.81616846	10.22709487	protein_coding	8	94870035	94938294	PDP1
ENSG00000164953	6.998676229	6.480864016	protein_coding	8	94767072	94831462	TMEM67
ENSG00000164961	9.072234198	9.056522014	protein_coding	8	126036502	126104082	KIAA0196
ENSG00000164967	6.704935538	7.326703453	protein_coding	9	34610483	34612101	C9orf23
ENSG00000164970	9.265790136	9.636922921	protein_coding	9	34398182	34458568	C9orf25
ENSG00000164972	1.799000381	1.16600992	protein_coding	9	34379017	34397830	C9orf24
ENSG00000164975	7.071412799	7.033288635	protein_coding	9	15422702	15465951	SNAPC3
ENSG00000164976	6.579622637	5.908941696	protein_coding	9	34366668	34376851	KIAA1161
ENSG00000164978	6.535310252	6.874119142	protein_coding	9	34329504	34343709	NUDT2
ENSG00000164983	7.737655476	7.836897019	protein_coding	8	125324231	125384933	TMEM65
ENSG00000164985	8.504022011	8.712778563	protein_coding	9	15464064	15511017	PSIP1
ENSG00000164989	3.335220907	4.385599336	protein_coding	9	15552895	16061661	C9orf93
ENSG00000165006	9.142495894	8.846464976	protein_coding	9	34179003	34252521	UBAP1
ENSG00000165025	6.050961887	0	protein_coding	9	93564069	93660831	SYK
ENSG00000165028	3.335220907	3.027231696	protein_coding	9	107526438	107539738	NIPSNAP3B
ENSG00000165029	9.680483837	9.574743444	protein_coding	9	107543283	107690518	ABCA1
ENSG00000165030	8.518728184	8.605140645	protein_coding	9	94171327	94186144	NFIL3
ENSG00000165046	6.435508026	6.136428444	protein_coding	8	38243725	38267045	LETM2
ENSG00000165055	8.619193517	8.130601896	protein_coding	7	128095894	128146656	METTL2B
ENSG00000165059	0.499066092	0	protein_coding	9	71627469	71629039	PRKACG
ENSG00000165060	7.878603882	8.32500368	protein_coding	9	71650175	71715094	FXN
ENSG00000165072	5.512107582	1.925536307	protein_coding	9	72658497	72841886	MAMDC2
ENSG00000165076	0	0.390640832	protein_coding	7	141536086	141541287	PRSS37
ENSG00000165078	0.499066092	0	protein_coding	8	68334360	68658620	CPA6
ENSG00000165084	0.499066092	0	protein_coding	8	69242957	69731257	C8orf34
ENSG00000165091	1.960915222	0.390640832	protein_coding	9	75136717	75455695	TMC1
ENSG00000165092	1.16343121	0	protein_coding	9	75515578	75695358	ALDH1A1
ENSG00000165097	8.583888749	8.262094789	protein_coding	6	18155560	18224084	KDM1B
ENSG00000165102	8.847332411	9.339469783	protein_coding	8	42995556	43057998	HGSNAT
ENSG00000165105	7.781675404	8.14810445	protein_coding	9	85594500	85678092	RASEF
ENSG00000165113	4.464551814	5.683080141	protein_coding	9	86354336	86444431	GKAP1
ENSG00000165115	4.981125677	5.824376633	protein_coding	9	86451613	86536342	KIF27
ENSG00000165118	8.183497283	7.73107057	protein_coding	9	86553226	86571901	C9orf64
ENSG00000165119	12.47317791	12.40135916	protein_coding	9	86582998	86595569	HNRNPK
ENSG00000165120	0	0.390640832	protein_coding	7	129847700	129856683	C7orf45
ENSG00000165121	3.004694206	4.319727133	pseudogene	9	88430881	88464426	
ENSG00000165124	4.464551814	4.101879561	protein_coding	9	113127531	113342160	SVEP1
ENSG00000165131	0	0.697730409	protein_coding	7	142636440	142637955	C7orf34
ENSG00000165138	9.875269153	9.547423533	protein_coding	9	101493611	101559247	ANKS6
ENSG00000165140	5.458875168	2.971122874	protein_coding	9	97365415	97402531	FBP1
ENSG00000165156	8.349904097	8.247406493	protein_coding	8	124260697	124287781	ZHX1
ENSG00000165169	7.813830732	8.159132889	protein_coding	X	37696010	37706890	DYNLT3
ENSG00000165171	3.698846687	4.319727133	protein_coding	7	73248920	73256865	WBSCR27
ENSG00000165175	9.979941537	10.09468113	protein_coding	X	38660685	38665790	MID1IP1
ENSG00000165178	0.499066092	0	pseudogene	7	74572445	74587848	NCF1C
ENSG00000165181	8.56354495	8.675956977	protein_coding	9	114448453	114557288	C9orf84
ENSG00000165182	1.16343121	0.390640832	protein_coding	X	23925918	23957624	CXorf58
ENSG00000165185	7.474855471	7.381542895	protein_coding	9	115249127	115431677	KIAA1958
ENSG00000165188	0.499066092	0	protein_coding	9	116059480	116065656	RNF183
ENSG00000165194	0.499066092	1.16600992	protein_coding	X	99546642	99665271	PCDH19
ENSG00000165195	7.395627075	7.701200733	protein_coding	X	15337573	15353676	PIGA
ENSG00000165197	0.869158192	1.518964905	protein_coding	X	15363713	15402498	FIGF
ENSG00000165209	8.681514984	8.480051236	protein_coding	9	125871779	126030855	STRBP
ENSG00000165215	5.270908378	4.127774132	protein_coding	7	73183328	73184600	CLDN3
ENSG00000165219	10.27042264	9.94751216	protein_coding	9	128024073	128129486	GAPVD1
ENSG00000165233	7.878603882	7.487839978	protein_coding	9	95858500	95875565	C9orf89
ENSG00000165238	6.681890601	4.202780776	protein_coding	9	95947198	96082854	WNK2

ENSG00000165240	6.721980839	7.434373903	protein_coding	X	77166194	77305892	ATP7A
ENSG00000165244	7.95748362	7.858322159	protein_coding	9	99148223	99180611	ZNF367
ENSG00000165259	4.75690578	5.901455548	protein_coding	X	83572882	83757487	HDX
ENSG00000165264	8.016241092	8.83570377	protein_coding	9	32552997	32573160	NDUFB6
ENSG00000165269	1.407729925	0.697730409	protein_coding	9	33384765	33402643	AQP7
ENSG00000165271	10.2558666	10.11464692	protein_coding	9	33461439	33473941	NOL6
ENSG00000165272	7.803191723	7.284161038	protein_coding	9	33441152	33447609	AQP3
ENSG00000165275	6.350376764	6.198834244	protein_coding	9	37753804	37778969	RG9MTD3
ENSG00000165280	12.04088917	11.93339824	protein_coding	9	35056061	35073246	VCP
ENSG00000165282	7.883662252	8.109639521	protein_coding	9	35088685	35096591	PIGO
ENSG00000165283	9.855452651	10.22372209	protein_coding	9	35099888	35103154	STOML2
ENSG00000165288	8.492479328	8.503728743	protein_coding	X	79926353	80065187	BRWD3
ENSG00000165300	1.16343121	4.075511708	protein_coding	13	88324870	88331868	SLITRK5
ENSG00000165304	9.132969064	9.275755399	protein_coding	9	36572859	36677678	MELK
ENSG00000165309	3.698846687	1.16600992	protein_coding	10	23216953	23326518	ARMC3
ENSG00000165312	8.571403474	7.598461017	protein_coding	10	23728198	23731308	OTUD1
ENSG00000165322	8.518728184	9.327648427	protein_coding	10	32094365	32217742	ARHGAP12
ENSG00000165325	0	0.390640832	protein_coding	11	93063137	93171653	CCDC67
ENSG00000165338	6.598204782	7.298481685	protein_coding	10	93170096	93274586	HECTD2
ENSG00000165355	7.471499737	7.619146302	protein_coding	14	39866873	39901704	FBXO33
ENSG00000165359	6.462804234	7.439552137	protein_coding	X	134654584	134716435	DDX26B
ENSG00000165376	3.335220907	2.420525079	protein_coding	X	106143394	106174091	CLDN2
ENSG00000165388	5.929039784	3.679994897	protein_coding	10	48355024	48373866	ZNF488
ENSG00000165389	8.438503912	9.014632836	protein_coding	14	34901995	34931562	SPTSSA
ENSG00000165392	8.032273249	8.076782959	protein_coding	8	30891317	31031285	WRN
ENSG00000165406	8.605481314	8.114504097	protein_coding	10	45950035	46090354	8-Mar
ENSG00000165409	0.499066092	1.16600992	protein_coding	14	81421333	81612646	TSHR
ENSG00000165410	9.003030709	9.370979666	protein_coding	14	35179593	35184029	CFL2
ENSG00000165416	8.116326397	7.568048738	protein_coding	13	53226844	53262433	SUGT1
ENSG00000165417	9.112647868	8.954409067	protein_coding	14	81641796	81687721	GTF2A1
ENSG00000165424	7.720795447	7.237355663	protein_coding	10	81142081	81205383	ZCCHC24
ENSG00000165434	8.730065371	9.001562612	protein_coding	11	74041363	74109518	PGM2L1
ENSG00000165443	0.499066092	0	protein_coding	10	60936350	61007534	PHYHIPL
ENSG00000165449	6.94645393	5.974624386	protein_coding	10	61410523	61495760	SLC16A9
ENSG00000165458	10.80421351	10.82816062	protein_coding	11	71934745	71950149	INPPL1
ENSG00000165474	6.482942724	4.406904905	protein_coding	13	20761609	20767037	GJB2
ENSG00000165475	6.585843317	7.00911166	protein_coding	13	20977806	21099996	CRYL1
ENSG00000165476	8.813274715	8.206984494	protein_coding	10	65281123	65384883	REEP3
ENSG00000165478	1.407729925	0.390640832	protein_coding	11	124789089	124806308	HEPACAM
ENSG00000165480	8.732871027	8.736113084	protein_coding	13	21727734	21750741	SKA3
ENSG00000165487	8.173205795	8.397309351	protein_coding	13	22066836	22178353	EFHA1
ENSG00000165490	8.508940744	8.65607594	protein_coding	11	82611017	82669319	C11orf82
ENSG00000165494	9.021506037	9.233151934	protein_coding	11	82868030	82898493	PCF11
ENSG00000165495	0	1.353254395	protein_coding	11	125034583	125303285	PKNOX2
ENSG00000165496	0.499066092	0.697730409	protein_coding	14	47120222	47121028	RPL10L
ENSG00000165501	7.587643674	7.933654619	protein_coding	14	50065415	50081390	LRR1
ENSG00000165502	9.752069226	10.38540204	protein_coding	14	50085237	50087403	RPL36AL
ENSG00000165506	7.345180517	7.825075067	protein_coding	14	50091892	50101948	DNAAF2
ENSG00000165507	8.391336806	8.926097022	protein_coding	10	45466429	45474258	C10orf10
ENSG00000165509	0	0.390640832	protein_coding	X	140926102	140985618	MAGEC3
ENSG00000165511	2.847891871	1.353254395	protein_coding	10	45493146	45496336	C10orf25
ENSG00000165512	5.858855738	6.16171561	protein_coding	10	45495923	45500774	ZNF22
ENSG00000165516	8.018542337	9.113519788	protein_coding	14	50234326	50249909	KLHDC2
ENSG00000165521	7.35977471	7.439552137	protein_coding	14	89078775	89259096	EML5
ENSG00000165525	8.959377969	9.351881826	protein_coding	14	50249997	50319921	NEMF
ENSG00000165526	8.377059993	8.667154767	protein_coding	11	126071993	126081587	RPUSD4
ENSG00000165527	10.39919165	10.21996528	protein_coding	14	50359810	50361490	ARF6
ENSG00000165533	8.727254249	8.52337065	protein_coding	14	89290497	89344335	TTC8
ENSG00000165548	4.097495944	3.183544561	protein_coding	14	77582911	77725838	TMEM63C
ENSG00000165555	4.062237333	4.917990497	protein_coding	14	77860364	77889860	NOXRED1
ENSG00000165568	6.965663283	6.174194902	protein_coding	10	4828820	4890254	AKR1E2
ENSG00000165572	6.979904289	6.365895578	protein_coding	13	41701705	41706882	KBTBD6
ENSG00000165591	3.274897671	2.039052734	protein_coding	X	57313139	57515629	FAAH2

ENSG00000165606	1.799000381	0	protein_coding	10	50572237	50603986	DRGX
ENSG00000165609	9.59671396	9.130429247	protein_coding	10	12207324	12238143	NUDT5
ENSG00000165617	0.499066092	1.353254395	protein_coding	14	59100685	59115039	DACT1
ENSG00000165626	5.827699195	4.990346085	protein_coding	10	13480484	13570974	BEND7
ENSG00000165629	10.19511088	10.26076174	protein_coding	10	7830092	7849778	ATP5C1
ENSG00000165630	7.169937448	6.775188634	protein_coding	10	13628927	13672868	PRPF18
ENSG00000165632	7.530740159	7.132546694	protein_coding	10	7860467	8058590	TAF3
ENSG00000165637	8.697401955	8.373156878	protein_coding	10	76969912	76991206	VDAC2
ENSG00000165644	7.692250012	7.260948155	protein_coding	10	76993414	76995788	COMTD1
ENSG00000165646	7.030950614	6.708116507	protein_coding	10	119000604	119038941	SLC18A2
ENSG00000165650	9.942255804	9.672543867	protein_coding	10	119040000	119134978	PDZD8
ENSG00000165655	4.322773689	6.130036786	protein_coding	10	77039484	77161664	ZNF503
ENSG00000165660	8.094677541	8.127396674	protein_coding	10	126490354	126525239	FAM175B
ENSG00000165661	9.950715896	9.924613326	protein_coding	9	139098179	139137687	QSOX2
ENSG00000165669	8.129161475	8.458531597	protein_coding	10	120065401	120101840	FAM204A
ENSG00000165671	9.977577838	9.797285822	protein_coding	5	176560026	176727216	NSD1
ENSG00000165672	10.74776895	11.03943311	protein_coding	10	120927215	120938345	PRDX3
ENSG00000165675	8.072698871	7.999999944	protein_coding	X	129751713	130037208	ENOX2
ENSG00000165678	11.03428274	11.36084034	protein_coding	10	85899196	85913311	GHITM
ENSG00000165684	9.909151231	9.741775201	protein_coding	9	139270029	139293249	SNAPC4
ENSG00000165685	8.027710784	7.572769481	protein_coding	12	10323141	10344400	C12orf59
ENSG00000165688	10.16166506	10.14824399	protein_coding	9	139305110	139318213	PMPCA
ENSG00000165689	10.31650661	9.921840881	protein_coding	9	139296377	139305061	SDCCAG3
ENSG00000165695	4.352265886	3.748356452	protein_coding	9	135600965	135754164	AK8
ENSG00000165698	5.707331524	5.734544355	protein_coding	9	135753414	135765588	C9orf9
ENSG00000165699	9.158235462	9.214393111	protein_coding	9	135766735	135820020	TSC1
ENSG00000165704	9.825536602	9.885305703	protein_coding	X	133594183	133654543	HPRT1
ENSG00000165714	5.752272083	6.198834244	protein_coding	12	12510013	12619840	LOH12CR1
ENSG00000165716	4.517578978	4.7279204	protein_coding	9	139607022	139618502	FAM69B
ENSG00000165724	8.549290825	8.69988975	protein_coding	9	140476531	140484942	ZMYND19
ENSG00000165730	4.097495944	4.226940739	protein_coding	10	70587298	70655188	STOX1
ENSG00000165732	11.89038555	11.47414795	protein_coding	10	70715884	70744829	DDX21
ENSG00000165733	10.19866692	9.748049581	protein_coding	10	43278249	43326856	BMS1
ENSG00000165752	9.108333003	9.176127473	protein_coding	10	134020994	134145353	STK32C
ENSG00000165757	10.71798852	9.623903733	protein_coding	10	30301729	30404423	KIAA1462
ENSG00000165775	8.602416377	9.423538659	protein_coding	X	154254255	154288578	FUNDC2
ENSG00000165782	8.235860718	8.570156468	protein_coding	14	20925878	20929771	TMEM55B
ENSG00000165792	8.561968095	9.274307066	protein_coding	14	21457929	21465189	METTL17
ENSG00000165795	5.223818625	4.640685763	protein_coding	14	21484922	21539031	NDRG2
ENSG00000165799	1.407729925	0.950786998	protein_coding	14	21510385	21512393	RNASE7
ENSG00000165801	8.974866377	9.224178134	protein_coding	14	21538429	21558399	ARHGEF40
ENSG00000165802	11.22371086	11.00688187	protein_coding	9	140342022	140353786	NELF
ENSG00000165804	6.846372398	7.289906366	protein_coding	14	21558205	21572881	ZNF219
ENSG00000165805	0.869158192	0.950786998	protein_coding	12	88373816	88427814	C12orf50
ENSG00000165806	8.164919416	8.599368544	protein_coding	10	115438942	115490662	CASP7
ENSG00000165807	1.16343121	2.420525079	protein_coding	14	65016620	65056098	PPP1R36
ENSG00000165813	7.627388205	7.737391576	protein_coding	10	115880621	115933979	C10orf118
ENSG00000165816	0.499066092	0	protein_coding	10	115999089	116051272	VWA2
ENSG00000165819	8.697401955	9.193089524	protein_coding	14	21966191	21979517	METTL3
ENSG00000165821	3.146112541	2.652276565	protein_coding	14	21989232	22005350	SALL2
ENSG00000165828	0.499066092	0.390640832	protein_coding	10	135160650	135166187	PRAP1
ENSG00000165832	7.945439403	8.490068116	protein_coding	10	116697952	116737430	TRUB1
ENSG00000165837	0	0.697730409	protein_coding	13	46108657	46189874	FAM194B
ENSG00000165861	8.557227164	9.188483253	protein_coding	14	73436159	73493920	ZFYVE1
ENSG00000165863	0.499066092	2.039052734	protein_coding	10	118423207	118429481	C10orf82
ENSG00000165868	7.30049066	8.222087975	protein_coding	10	118430703	118502085	HSPA12A
ENSG00000165874	0.869158192	0.697730409	protein_coding	10	46897641	46939148	FAM35B
ENSG00000165879	4.097495944	4.961837817	protein_coding	10	99079022	99081672	FRAT1
ENSG00000165886	7.494827594	7.246248121	protein_coding	10	99258625	99330966	UBTD1
ENSG00000165887	2.35979773	0.950786998	protein_coding	10	99332198	99343641	ANKRD2
ENSG00000165891	8.455589162	8.543951256	protein_coding	12	77415027	77459360	E2F7
ENSG00000165895	8.453889713	8.726612716	protein_coding	11	100558384	100861656	ARHGAP42
ENSG00000165898	7.457998197	7.357122767	protein_coding	14	74960423	74963809	ISCA2

ENSG00000165899	0	0.390640832	protein_coding	12	80603233	80772870	OTOGL
ENSG00000165905	7.868433623	4.794098771	protein_coding	11	45943172	45950647	GYLTL1B
ENSG00000165912	9.05999771	8.972384393	protein_coding	11	47199076	47207994	PACSIN3
ENSG00000165914	8.504022011	6.952279523	protein_coding	14	91006932	91282823	TTC7B
ENSG00000165915	9.336667406	9.960628829	protein_coding	11	47428683	47438047	SLC39A13
ENSG00000165916	10.77115846	11.04092444	protein_coding	11	47440320	47447993	PSMC3
ENSG00000165917	3.871730003	3.081239798	protein_coding	11	47459308	47470730	RAPSN
ENSG00000165923	4.19837882	3.781359661	protein_coding	11	47681143	47736941	AGBL2
ENSG00000165929	5.958104008	4.202780776	protein_coding	14	92246095	92333880	TC2N
ENSG00000165934	10.01839363	8.679244009	protein_coding	14	92588281	92630755	CPSF2
ENSG00000165935	3.393122761	2.971122874	protein_coding	12	27619743	27655118	C12orf70
ENSG00000165943	7.395627075	7.36802722	protein_coding	14	93648541	93651273	MOAP1
ENSG00000165948	6.482942724	6.398170093	protein_coding	14	94547628	94570192	IFI27L1
ENSG00000165949	10.39344018	10.82148229	protein_coding	14	94571182	94583033	IFI27
ENSG00000165959	7.955082807	6.064512706	protein_coding	14	95654735	95786243	CLMN
ENSG00000165972	1.407729925	1.353254395	protein_coding	12	96260826	96336752	CCDC38
ENSG00000165983	8.006999229	7.546612122	protein_coding	10	16478964	16555736	PTER
ENSG00000165985	2.35979773	2.420525079	protein_coding	10	16555742	16564004	C1QL3
ENSG00000165995	3.448790144	3.453019579	protein_coding	10	18429606	18830798	CACNB2
ENSG00000165996	8.45218826	8.914056863	protein_coding	10	17631958	17659376	PTPLA
ENSG00000165997	9.243337861	8.078443681	protein_coding	10	18948334	18970568	ARL5B
ENSG00000166002	5.986594255	6.392840898	protein_coding	11	93211638	93276674	C11orf75
ENSG00000166004	8.096857104	8.480051236	protein_coding	11	93394805	93463522	KIAA1731
ENSG00000166012	10.10702545	10.57438579	protein_coding	11	93463114	93517557	TAF1D
ENSG00000166016	8.937654214	9.098048079	protein_coding	11	34172535	34379555	ABTB2
ENSG00000166024	8.05038018	8.428896259	protein_coding	10	99894387	100004654	C10orf28
ENSG00000166025	7.891216707	5.236305879	protein_coding	11	94439597	94609918	AMOTL1
ENSG00000166033	7.254372086	8.581912372	protein_coding	10	124221041	124274424	HTRA1
ENSG00000166035	0.499066092	0.697730409	protein_coding	15	58702768	58861151	LIPC
ENSG00000166037	9.139327269	9.516516751	protein_coding	11	95523129	95565857	CEP57
ENSG00000166046	5.431503808	5.656642807	protein_coding	12	106695707	106740793	TCP11L2
ENSG00000166068	8.077121473	8.309531754	protein_coding	15	38544527	38649450	SPRED1
ENSG00000166073	9.916573728	10.11100039	protein_coding	15	40091233	40213093	GPR176
ENSG00000166086	4.352265886	4.585695337	protein_coding	11	133938820	134021896	JAM3
ENSG00000166104	0	0.390640832	pseudogene	15	62520095	62545434	
ENSG00000166106	6.716321419	6.376734172	protein_coding	11	130318869	130346532	ADAMTS15
ENSG00000166123	9.812996626	10.41987022	protein_coding	16	46918290	46965209	GPT2
ENSG00000166126	3.989024711	2.501982735	protein_coding	14	103388993	103399933	AMN
ENSG00000166128	8.344411036	9.162099548	protein_coding	15	63481668	63559981	RAB8B
ENSG00000166130	8.342575358	8.648269828	protein_coding	12	99007183	99038891	IKBIP
ENSG00000166133	7.409722215	7.243290054	protein_coding	15	40861499	40866659	RPUSD2
ENSG00000166135	8.857651982	9.069940847	protein_coding	10	102288829	102309763	HIF1AN
ENSG00000166136	6.567100227	7.053694091	protein_coding	10	102267203	102289757	NDUFB8
ENSG00000166140	8.778393539	8.622319665	protein_coding	15	41099284	41106767	ZFYVE19
ENSG00000166143	0.499066092	0	protein_coding	15	41107650	41120907	PPP1R14D
ENSG00000166145	9.304967144	3.906303962	protein_coding	15	41136216	41150405	SPINT1
ENSG00000166147	5.124761037	4.917990497	protein_coding	15	48700503	48938046	FBN1
ENSG00000166153	3.502389126	3.570966319	protein_coding	12	100597447	100660857	DEPDC4
ENSG00000166159	0.499066092	0.390640832	protein_coding	12	1929433	1945918	LRTM2
ENSG00000166164	8.436784206	8.91219558	protein_coding	16	50347938	50402845	BRD7
ENSG00000166165	9.897945387	7.304170377	protein_coding	14	103985996	103989448	CKB
ENSG00000166166	9.298353251	8.461080043	protein_coding	14	103995521	104003410	TRMT61A
ENSG00000166167	8.572970055	8.68797299	protein_coding	10	103113820	103317078	BTRC
ENSG00000166169	7.781675404	8.132201841	protein_coding	10	103338639	103348027	POLL
ENSG00000166170	9.381161578	8.578395649	protein_coding	14	104022881	104029168	BAG5
ENSG00000166171	7.478203418	7.992972811	protein_coding	10	103330317	103369425	DPCD
ENSG00000166173	6.244259325	7.022976646	protein_coding	15	71121469	71146498	LARP6
ENSG00000166181	10.56462462	10.9257172	protein_coding	11	43333513	43366079	API5
ENSG00000166183	4.999881834	4.17820932	protein_coding	14	104552016	104579098	ASPG
ENSG00000166188	7.737655476	8.117738058	protein_coding	16	58028572	58034357	ZNF319
ENSG00000166189	8.518728184	8.486319932	protein_coding	10	103825147	103827792	HPS6
ENSG00000166192	3.871730003	4.42790041	protein_coding	15	72406599	72433311	SENP8
ENSG00000166197	11.79762603	12.01728578	protein_coding	10	103911933	103923627	NOLC1

ENSG00000166199	8.131289597	8.177844832	protein_coding	11	43902361	43941816	ALKBH3
ENSG00000166200	9.139327269	9.400488931	protein_coding	15	49398268	49447858	COPS2
ENSG00000166206	6.902274257	7.326703453	protein_coding	15	26788693	27184686	GABRB3
ENSG00000166220	0.499066092	0	protein_coding	10	72530995	72545157	C10orf27
ENSG00000166224	9.978760171	9.407112231	protein_coding	10	72575717	72640930	SGPL1
ENSG00000166225	7.720795447	7.722599249	protein_coding	12	69864129	69973562	FRS2
ENSG00000166226	10.80187738	10.96866235	protein_coding	12	69979114	69995350	CCT2
ENSG00000166228	8.554057865	8.535512415	protein_coding	10	72642037	72648541	PCBD1
ENSG00000166233	9.182040809	9.140798547	protein_coding	15	72766667	72879692	ARIH1
ENSG00000166246	2.238690726	3.532712221	protein_coding	16	4784273	4799397	C16orf71
ENSG00000166250	6.941611359	5.832272499	protein_coding	11	122943035	123065989	CLMP
ENSG00000166260	8.221948786	8.193254836	protein_coding	17	53038568	53046064	COX11
ENSG00000166261	7.464764762	7.668495823	protein_coding	11	123594885	123612383	ZNF202
ENSG00000166262	4.165528823	4.17820932	protein_coding	15	49619159	49913128	C15orf33
ENSG00000166263	6.573375019	6.827478203	protein_coding	17	53046088	53241646	STXBP4
ENSG00000166266	8.762005612	9.00068704	protein_coding	11	107879459	107978503	CUL5
ENSG00000166268	0	1.518964905	protein_coding	12	70219084	70352877	C12orf28
ENSG00000166272	8.521975964	8.904726341	protein_coding	10	104503727	104576021	C10orf26
ENSG00000166275	5.498982484	6.440109466	protein_coding	10	104613980	104632990	C10orf32
ENSG00000166278	4.19837882	3.714580548	protein_coding	6	31865562	31913449	C2
ENSG00000166289	8.18144485	8.265014588	protein_coding	19	30156327	30166376	PLEKHF1
ENSG00000166292	1.616589159	0	protein_coding	17	53796990	53809482	TMEM100
ENSG00000166295	8.267164507	8.320800493	protein_coding	10	73975787	73995472	ANAPC16
ENSG00000166311	8.043616684	9.351195062	protein_coding	11	6411655	6416228	SMPD1
ENSG00000166313	7.624369505	8.101495266	protein_coding	11	6416354	6440644	APBB1
ENSG00000166317	1.16343121	0.390640832	protein_coding	10	75404639	75415830	SYNPO2L
ENSG00000166321	6.290697143	5.995870484	protein_coding	10	74870217	74891586	NUDT13
ENSG00000166323	3.274897671	2.579085888	protein_coding	11	108179246	108338258	C11orf65
ENSG00000166326	9.784207642	9.83559787	protein_coding	11	35684353	35829775	TRIM44
ENSG00000166333	4.165528823	4.693656882	protein_coding	11	6624961	6632102	ILK
ENSG00000166337	9.092036341	9.515904068	protein_coding	11	6627526	6633898	TAF10
ENSG00000166340	10.97152701	11.70170789	protein_coding	11	6634000	6640692	TPP1
ENSG00000166341	1.407729925	2.144285137	protein_coding	11	6642554	6677085	DCHS1
ENSG00000166343	3.871730003	3.232099092	protein_coding	10	75183339	75193319	ZMYND17
ENSG00000166347	7.484876093	8.289597152	protein_coding	18	71920528	71959251	CYB5A
ENSG00000166348	8.791909364	8.801925129	protein_coding	10	75257296	75385711	USP54
ENSG00000166349	2.671945279	3.493416095	protein_coding	11	36532259	36614706	RAG1
ENSG00000166352	6.407685389	6.900645892	protein_coding	11	36616051	36694823	C11orf74
ENSG00000166359	2.671945279	2.912743273	protein_coding	19	33622998	33666705	WDR88
ENSG00000166377	6.548110424	7.201227369	protein_coding	18	76829394	77138278	ATP9B
ENSG00000166387	7.19863491	7.519971735	protein_coding	11	7534529	7678358	PPFIBP2
ENSG00000166391	0.499066092	0	protein_coding	11	75428864	75444003	MOGAT2
ENSG00000166394	8.973680846	9.067434305	protein_coding	11	7686331	7698453	CYB5R2
ENSG00000166396	5.270908378	5.816437314	protein_coding	18	61420169	61472604	SERPINB7
ENSG00000166398	7.406211319	7.462626902	protein_coding	19	34745456	34846471	KIAA0355
ENSG00000166401	8.282565319	8.467431533	protein_coding	18	61637159	61672278	SERPINB8
ENSG00000166402	0	0.390640832	protein_coding	11	8040791	8127659	TUB
ENSG00000166407	1.616589159	1.925536307	protein_coding	11	8245851	8290263	LMO1
ENSG00000166411	7.642387679	7.688207601	protein_coding	15	78423840	78464291	IDH3A
ENSG00000166432	4.230497448	7.03670965	protein_coding	X	101137262	101187004	ZMAT1
ENSG00000166435	6.212449006	6.582669759	protein_coding	11	74518784	74660245	XRRA1
ENSG00000166436	7.868433623	7.612284109	protein_coding	11	8633584	8693413	TRIM66
ENSG00000166439	7.590740122	7.95731437	protein_coding	11	74459913	74553458	RNF169
ENSG00000166441	11.52967299	12.26772514	protein_coding	11	8703958	8736306	RPL27A
ENSG00000166444	10.19663597	10.42705723	protein_coding	11	8714898	8932498	ST5
ENSG00000166446	5.054730169	5.35066967	protein_coding	16	80637609	80838226	CDYL2
ENSG00000166448	0.499066092	0	protein_coding	7	98444111	98468394	TMEM130
ENSG00000166450	6.664362065	6.754561598	protein_coding	15	55903744	56035288	PRTG
ENSG00000166451	8.586993258	9.071609459	protein_coding	16	81040103	81066719	CENPN
ENSG00000166452	7.893726093	8.119352325	protein_coding	11	8932686	8941631	AKIP1
ENSG00000166454	9.226515195	9.304420832	protein_coding	16	81069452	81080963	ATMIN
ENSG00000166455	4.863159938	5.175534283	protein_coding	16	81087102	81110872	C16orf46
ENSG00000166471	8.693086466	7.955508102	protein_coding	11	9302201	9336327	TMEM41B

ENSG00000166473	4.491309013	4.640685763	polymorphic_pse	16	81134480	81253975	PKD1L2
ENSG00000166477	8.079327701	8.165397208	protein_coding	15	52230222	52264003	LEO1
ENSG00000166478	7.612231145	7.596144237	protein_coding	11	9481866	9550071	ZNF143
ENSG00000166479	8.711694395	9.186944555	protein_coding	18	66340925	66382535	TMX3
ENSG00000166482	3.502389126	4.448594745	protein_coding	17	19286755	19290553	MFAP4
ENSG00000166483	9.38473389	9.410412513	protein_coding	11	9595228	9615004	WEE1
ENSG00000166484	8.173205795	8.894392583	protein_coding	17	19281034	19286857	MAPK7
ENSG00000166492	1.799000381	1.16600992	pseudogene	11	3431582	3443726	FAM86GP
ENSG00000166503	8.211928911	8.149685112	protein_coding	15	83784320	83876770	
ENSG00000166507	7.746012174	7.926295939	protein_coding	10	75561669	75571589	NDST2
ENSG00000166508	12.30874036	12.01219011	protein_coding	7	99690351	99699563	MCM7
ENSG00000166510	5.879259127	5.709041716	protein_coding	18	52568740	52626739	CCDC68
ENSG00000166526	8.207901394	8.106387334	protein_coding	7	99661656	99680171	ZNF3
ENSG00000166529	5.124761037	4.810179682	protein_coding	7	99647390	99662661	ZSCAN21
ENSG00000166532	7.853042529	7.522414019	protein_coding	12	8834196	8935691	RIMKLB
ENSG00000166546	6.793601635	6.371325053	protein_coding	16	66461200	66527432	BEAN1
ENSG00000166548	8.032273249	8.360927302	protein_coding	16	66503715	66586447	TK2
ENSG00000166557	9.324628822	9.711058296	protein_coding	15	79603404	79704334	TMED3
ENSG00000166558	0.869158192	0.697730409	protein_coding	16	84043272	84076241	SLC38A8
ENSG00000166562	7.127924785	7.928139131	protein_coding	18	56807087	56826068	SEC11C
ENSG00000166575	8.366258896	7.650745242	protein_coding	11	86748886	87034756	TMEM135
ENSG00000166578	6.400645076	6.198834244	protein_coding	12	113633246	113658899	IQCD
ENSG00000166579	9.221529789	9.705158168	protein_coding	17	8339179	8383942	NDEL1
ENSG00000166582	8.687312327	9.309379244	protein_coding	17	16245848	16256813	CENPV
ENSG00000166589	1.407729925	0.697730409	protein_coding	16	66942025	66952887	CDH16
ENSG00000166592	10.15645003	10.60120454	protein_coding	16	66955582	66959547	RRAD
ENSG00000166595	9.198372384	9.678578614	protein_coding	16	66965959	66968326	FAM96B
ENSG00000166596	3.004694206	3.714580548	protein_coding	17	9479944	9546776	WDR16
ENSG00000166598	12.5744085	12.85924707	protein_coding	12	104323885	104347423	HSP90B1
ENSG00000166619	9.670274772	9.432656416	protein_coding	20	36130816	36156333	BLCAP
ENSG00000166664	1.16343121	1.518964905	protein_coding	15	30653443	30686052	CHRFAM7A
ENSG00000166667	3.744048221	3.32456471	pseudogene	7	101986192	101996889	
ENSG00000166669	7.618313091	7.918899532	protein_coding	16	10420291	10577495	ATF7IP2
ENSG00000166670	1.616589159	3.027231696	protein_coding	11	102641234	102651359	MMP10
ENSG00000166676	2.57521082	2.912743273	protein_coding	16	10855083	10912621	FAM18A
ENSG00000166682	3.077135474	3.532712221	protein_coding	11	113558272	113577095	TMPRSS5
ENSG00000166685	8.572970055	8.696649474	protein_coding	17	71188771	71204646	COG1
ENSG00000166689	5.977159882	5.466810074	protein_coding	11	16799842	17035990	PLEKHA7
ENSG00000166704	7.618313091	7.659647832	protein_coding	19	58488421	58514717	ZNF606
ENSG00000166707	0	0.390640832	processed_transc	X	103356822	103360533	
ENSG00000166710	12.65418289	13.69747082	protein_coding	15	45003675	45011075	B2M
ENSG00000166716	9.219530792	9.340162149	protein_coding	15	85291866	85349659	ZNF592
ENSG00000166734	9.442434712	9.087365757	protein_coding	15	44580927	44707956	CASC4
ENSG00000166736	1.799000381	1.353254395	protein_coding	11	113845603	113861035	HTR3A
ENSG00000166741	10.51128816	11.26364488	protein_coding	11	114128509	114184007	NNMT
ENSG00000166743	1.16343121	0	protein_coding	16	20634559	20710212	ACSM1
ENSG00000166747	10.31041517	10.09673117	protein_coding	16	71762913	71843104	AP1G1
ENSG00000166750	10.03383333	10.04288547	protein_coding	17	33570086	33594739	SLFN5
ENSG00000166762	6.121171528	5.886365696	protein_coding	15	43920701	43960316	CATSPER2
ENSG00000166763	1.616589159	1.802319292	pseudogene	15	43991686	44010382	STRCP1
ENSG00000166770	7.140657543	7.546612122	protein_coding	19	56989552	57006801	
ENSG00000166780	5.588445295	6.908136232	protein_coding	16	15528152	15718885	C16orf45
ENSG00000166783	9.409495425	9.491180897	protein_coding	16	15688243	15737023	KIAA0430
ENSG00000166788	8.221948786	8.202422444	protein_coding	11	18091482	18127638	SAAL1
ENSG00000166793	2.10647801	3.081239798	protein_coding	11	57412560	57417417	YPEL4
ENSG00000166794	10.02984629	10.51951035	protein_coding	15	64448011	64455404	PPIB
ENSG00000166796	5.613019223	5.808454063	protein_coding	11	18433854	18473605	LDHC
ENSG00000166797	8.101206377	8.502492199	protein_coding	15	64364758	64386217	FAM96A
ENSG00000166800	2.471521042	0.950786998	protein_coding	11	18477371	18501147	LDHAL6A
ENSG00000166801	9.304967144	9.681859685	protein_coding	11	58910221	58922512	FAM111A
ENSG00000166803	8.133414584	8.626866398	protein_coding	15	64657193	64679886	KIAA0101
ENSG00000166813	6.163378456	5.32851069	protein_coding	15	90152020	90198682	KIF7
ENSG00000166816	5.91922012	6.002883551	protein_coding	16	75145758	75150669	LDHD

ENSG00000166819	1.799000381	2.242360793	protein_coding	15	90207596	90222658	PLIN1
ENSG00000166821	5.806547963	5.691786012	protein_coding	15	90220995	90234014	PEX11A
ENSG00000166822	7.609180529	7.447284805	protein_coding	16	75476952	75550136	TMEM170A
ENSG00000166823	3.830412367	4.319727133	protein_coding	15	90291892	90294541	MESP1
ENSG00000166825	11.65643998	11.47083239	protein_coding	15	90328120	90358633	ANPEP
ENSG00000166828	0.499066092	0.390640832	protein_coding	16	23194036	23228204	SCNN1G
ENSG00000166831	0.499066092	0.950786998	protein_coding	15	65032091	65067786	RBPMS2
ENSG00000166833	9.689899429	10.46246481	protein_coding	11	19372271	20143144	NAV2
ENSG00000166839	5.525114348	5.893930351	protein_coding	15	65204101	65251042	ANKDD1A
ENSG00000166845	7.680671792	7.864110577	protein_coding	18	51884955	51908404	C18orf54
ENSG00000166847	9.51676008	9.442361917	protein_coding	16	23652713	23681195	DCTN5
ENSG00000166848	8.508940744	8.795881219	protein_coding	16	75681684	75795051	TERF2IP
ENSG00000166851	10.79752881	10.54705904	protein_coding	16	23688977	23701688	PLK1
ENSG00000166855	8.782461599	9.133627746	protein_coding	15	65440557	65477680	CLPX
ENSG00000166856	0	1.802319292	protein_coding	12	57388230	57390468	GPR182
ENSG00000166860	6.681890601	7.060432274	protein_coding	12	57392618	57400230	ZBTB39
ENSG00000166863	0.499066092	1.802319292	protein_coding	12	57403784	57422667	TAC3
ENSG00000166866	0	1.667587519	protein_coding	12	57422301	57444982	MYO1A
ENSG00000166881	9.240383373	9.409093306	protein_coding	12	57449426	57481846	TMEM194A
ENSG00000166886	8.396654395	8.015686953	protein_coding	12	57482677	57489259	NAB2
ENSG00000166887	9.446713374	9.171466637	protein_coding	15	42450899	42500514	VPS39
ENSG00000166888	10.91860221	11.03537742	protein_coding	12	57489191	57525922	STAT6
ENSG00000166889	10.5080162	10.54014571	protein_coding	11	59404189	59436471	PATL1
ENSG00000166896	6.023726639	6.371325053	protein_coding	12	58335324	58351052	XRCC6BP1
ENSG00000166897	9.047656547	6.941369681	protein_coding	22	37764000	37823505	ELFN2
ENSG00000166900	8.577659618	9.339469783	protein_coding	11	59480929	59573354	STX3
ENSG00000166902	9.012297947	9.336696989	protein_coding	11	59573608	59578345	MRPL16
ENSG00000166908	9.644431499	9.038718229	protein_coding	12	57984957	57997198	PIP4K2C
ENSG00000166912	7.0218028	7.405556541	protein_coding	15	31231144	31283810	MTMR10
ENSG00000166913	11.94977284	11.80186107	protein_coding	20	43514317	43537173	YWHAB
ENSG00000166920	9.918423401	10.11989792	protein_coding	15	45722727	45740959	C15orf48
ENSG00000166922	4.689181911	5.683080141	protein_coding	15	32933877	32989299	SCG5
ENSG00000166923	1.16343121	0	protein_coding	15	33010175	33026870	GREM1
ENSG00000166924	5.525114348	4.226940739	protein_coding	7	100081550	100092422	NYAP1
ENSG00000166925	9.845117281	9.260474903	protein_coding	7	100060982	100076902	TSC22D4
ENSG00000166927	0	0.390640832	protein_coding	11	60145955	60163424	MS4A7
ENSG00000166938	7.72361917	7.596144237	protein_coding	15	66585555	66626236	DIS3L
ENSG00000166946	7.978913023	8.266472275	protein_coding	15	43477316	43487396	CCNDBP1
ENSG00000166947	2.57521082	1.802319292	protein_coding	15	43398423	43513481	EPB42
ENSG00000166949	11.30018487	11.10179951	protein_coding	15	67356101	67487533	SMAD3
ENSG00000166963	4.642199401	5.35066967	protein_coding	15	43803156	43829571	MAP1A
ENSG00000166965	6.716321419	7.418726742	protein_coding	15	91498100	91506349	RCCD1
ENSG00000166971	6.350376764	6.835357159	protein_coding	16	53524146	53538323	AKTIP
ENSG00000166974	7.988335976	7.646273261	protein_coding	18	32556892	32725381	MAPRE2
ENSG00000166979	1.16343121	0.390640832	protein_coding	21	33784314	33887707	C21orf63
ENSG00000166984	0.499066092	0.697730409	protein_coding	6	167559902	167610401	TCP10L2
ENSG00000166986	11.61616959	11.80135835	protein_coding	12	57869228	57911352	MARS
ENSG00000166987	9.913485661	9.961978945	protein_coding	12	57914493	57923931	MBD6
ENSG00000166997	7.30049066	7.683850422	protein_coding	7	99717236	99723134	CNPY4
ENSG00000167004	11.358119	11.76275923	protein_coding	15	44038590	44065477	PDIA3
ENSG00000167005	10.0178186	10.42542696	protein_coding	16	56463045	56486111	NUDT21
ENSG00000167011	3.146112541	2.242360793	protein_coding	7	100813774	100823557	NAT16
ENSG00000167034	7.374222747	7.726841127	protein_coding	8	23536206	23540440	NKX3-1
ENSG00000167037	3.871730003	3.532712221	protein_coding	22	25202188	25323545	SGSM1
ENSG00000167046	2.57521082	2.420525079	protein_coding	20	61272071	61274656	
ENSG00000167065	6.414691513	6.750400563	protein_coding	22	31048038	31063877	DUSP18
ENSG00000167074	7.210761201	6.952279523	protein_coding	22	41763337	41795330	TEF
ENSG00000167077	3.448790144	4.385599336	protein_coding	22	42095503	42195460	mei-01
ENSG00000167080	0.499066092	0	protein_coding	17	47209822	47247351	B4GALNT2
ENSG00000167081	7.759833327	7.726841127	protein_coding	9	128509624	128729656	PBX3
ENSG00000167085	10.03497047	10.07900496	protein_coding	17	47481414	47492246	PHB
ENSG00000167088	8.805299741	9.102135695	protein_coding	18	19192260	19210204	SNRPD1
ENSG00000167094	1.16343121	1.925536307	protein_coding	9	130478345	130493879	TTC16

ENSG00000167100	1.407729925	1.353254395	protein_coding	17	48187404	48207246	SAMD14
ENSG00000167103	6.622614428	5.22435463	protein_coding	9	130683158	130693076	PIP5KL1
ENSG00000167105	6.364918751	5.759604001	protein_coding	17	48348767	48358844	TMEM92
ENSG00000167106	10.15121608	9.987835859	protein_coding	9	130702858	130742812	FAM102A
ENSG00000167107	7.058051099	7.444711851	protein_coding	17	48503519	48552206	ACSF2
ENSG00000167110	10.34199724	9.997084738	protein_coding	9	131018108	131038274	GOLGA2
ENSG00000167112	9.317170221	9.282254961	protein_coding	9	131071382	131085021	TRUB2
ENSG00000167113	9.139327269	9.197681135	protein_coding	9	131084815	131096351	COQ4
ENSG00000167114	10.11618743	9.730728943	protein_coding	9	131102925	131123502	SLC27A4
ENSG00000167117	0	0.390640832	processed_transc	17	48838390	48844918	LINC00483
ENSG00000167118	9.612034058	9.666483771	protein_coding	9	131133598	131153015	URM1
ENSG00000167123	9.442434712	9.730728943	protein_coding	9	131174030	131199627	CERCAM
ENSG00000167130	8.443650765	8.273738696	protein_coding	9	131843379	131852717	DOLPP1
ENSG00000167136	6.059927234	6.525377513	protein_coding	9	131580753	131584956	ENDOG
ENSG00000167157	2.762599152	2.039052734	protein_coding	9	132427920	132484875	PRRX2
ENSG00000167159	3.554067925	3.993387124	protein_coding	17	43717670	43719667	C17orf69
ENSG00000167173	9.615838797	8.93895195	protein_coding	15	75487984	75504510	C15orf39
ENSG00000167178	0.869158192	1.16600992	protein_coding	15	74392652	74430881	ISLR2
ENSG00000167182	8.436784206	7.827052132	protein_coding	17	45973516	46006323	SP2
ENSG00000167183	3.004694206	1.802319292	protein_coding	17	46029336	46035110	PRR15L
ENSG00000167186	6.965663283	7.289906366	protein_coding	16	19078921	19091762	COQ7
ENSG00000167191	10.08358792	9.402479116	protein_coding	16	19868616	19897489	GPRC5B
ENSG00000167193	10.22779886	10.32267696	protein_coding	17	1323983	1359552	CRK
ENSG00000167194	0	0.390640832	protein_coding	16	30034655	30039057	C16orf92
ENSG00000167196	8.247679379	8.235547225	protein_coding	15	76196200	76227609	FBXO22
ENSG00000167202	8.349904097	8.595894166	protein_coding	15	78276378	78370066	TBC1D2B
ENSG00000167207	4.131913373	3.411459265	protein_coding	16	50727514	50766988	NOD2
ENSG00000167208	0	0.390640832	protein_coding	16	50700211	50715264	SNX20
ENSG00000167216	4.77879189	5.212303549	protein_coding	18	44526787	44628613	KATNAL2
ENSG00000167220	7.182306324	7.384230902	protein_coding	18	44633782	44676871	HDHD2
ENSG00000167230	0.869158192	1.667587519	protein_coding	17	35732985	35749660	C17orf78
ENSG00000167232	8.199812472	8.492561507	protein_coding	19	23540501	23578269	ZNF91
ENSG00000167244	6.788216822	5.863430791	protein_coding	11	2150342	2182439	IGF2
ENSG00000167257	7.392081667	7.91704449	protein_coding	11	117103341	117157161	RNF214
ENSG00000167258	9.689899429	9.370979666	protein_coding	17	37617739	37721160	CDK12
ENSG00000167264	8.068262669	8.257703981	protein_coding	16	68050054	68113223	DUS2L
ENSG00000167272	7.392081667	7.584504199	protein_coding	12	121016567	121019201	POP5
ENSG00000167280	8.355376323	8.650504459	protein_coding	17	77071019	77084681	ENGASE
ENSG00000167281	3.698846687	3.87606762	protein_coding	17	77085427	77613550	RBFOX3
ENSG00000167283	8.919297816	9.478658596	protein_coding	11	118271869	118302211	ATP5L
ENSG00000167286	0	0.390640832	protein_coding	11	118209669	118213459	CD3D
ENSG00000167291	9.429522058	8.902852973	protein_coding	17	77911708	78009647	TBC1D16
ENSG00000167302	7.654275977	7.683850422	protein_coding	17	79202079	79212891	C17orf56
ENSG00000167306	7.60612345	5.486957566	protein_coding	18	47349156	47721451	MYO5B
ENSG00000167311	0.869158192	1.16600992	protein_coding	11	3659733	3663546	ART5
ENSG00000167315	9.024943975	9.177677745	protein_coding	18	47309869	47340273	ACAA2
ENSG00000167323	9.093128531	8.887777807	protein_coding	11	3875757	4114439	STIM1
ENSG00000167325	10.64688074	10.92525585	protein_coding	11	4115937	4160106	RRM1
ENSG00000167333	7.246540262	7.63953919	protein_coding	11	4619902	4629489	TRIM68
ENSG00000167355	1.16343121	0	processed_transc	11	5326206	5526882	
ENSG00000167363	6.328285199	5.175534283	protein_coding	17	80693452	80709073	FN3K
ENSG00000167371	4.409500985	5.125003832	protein_coding	16	29823177	29827795	PRRT2
ENSG00000167377	3.211941663	4.127774132	protein_coding	16	71481500	71496998	ZNF23
ENSG00000167378	9.179986301	9.108651887	protein_coding	19	44088521	44100287	IRGQ
ENSG00000167380	8.504022011	8.706348549	protein_coding	19	44645710	44681836	ZNF226
ENSG00000167383	8.46911316	8.475016594	protein_coding	19	44930426	44952665	ZNF229
ENSG00000167384	7.326727684	7.616862529	protein_coding	19	44979861	45004574	ZNF180
ENSG00000167390	0.499066092	0	pseudogene	20	23969929	23971084	POM121L3P
ENSG00000167393	8.259402007	8.434094156	protein_coding	X	294698	347690	PPP2R3B
ENSG00000167394	7.527512071	7.454976247	protein_coding	16	31072164	31085641	ZNF668
ENSG00000167395	8.749591335	8.742412084	protein_coding	16	31085743	31095517	ZNF646
ENSG00000167397	7.633406721	8.222087975	protein_coding	16	31102163	31107301	VKORC1
ENSG00000167414	0	0.390640832	protein_coding	19	47137333	47137939	GNG8

ENSG00000167434	0	0.390640832	protein_coding	17	58227302	58236902	CA4
ENSG00000167447	8.265227794	8.729786462	protein_coding	17	57287371	57292608	SMG8
ENSG00000167460	13.37211917	13.45451319	protein_coding	19	16178317	16213813	TPM4
ENSG00000167461	10.76568906	10.46055573	protein_coding	19	16222490	16244443	RAB8A
ENSG00000167468	12.01850017	12.44328782	protein_coding	19	1103936	1106786	GPX4
ENSG00000167470	11.08044692	10.06399506	protein_coding	19	1248552	1259139	MIDN
ENSG00000167476	8.46911316	8.187110572	protein_coding	19	2252250	2256422	JSRP1
ENSG00000167483	2.238690726	2.501982735	protein_coding	19	17634110	17664647	FAM129C
ENSG00000167487	7.847875458	7.405556541	protein_coding	19	18747838	18781302	KLHL26
ENSG00000167491	12.20862045	11.69806667	protein_coding	19	19496639	19619740	GATAD2A
ENSG00000167508	9.575770339	8.588920207	protein_coding	16	88718343	88729569	MVD
ENSG00000167513	9.796999249	9.3669085	protein_coding	16	88869621	88875666	CDT1
ENSG00000167515	8.53328596	8.717049372	protein_coding	16	88922628	88929094	TRAPPC2L
ENSG00000167522	11.20615173	11.11055089	protein_coding	16	89334038	89556969	ANKRD11
ENSG00000167523	7.277615649	7.844724842	protein_coding	16	89724210	89737680	C16orf55
ENSG00000167524	7.007971391	6.642271819	protein_coding	17	26907030	26941474	
ENSG00000167525	5.485736881	5.125003832	protein_coding	17	27030215	27038872	PROCA1
ENSG00000167526	12.55630746	12.9401949	protein_coding	16	89627065	89630950	RPL13
ENSG00000167528	5.575999561	5.486957566	protein_coding	12	48733791	48745197	ZNF641
ENSG00000167535	7.222786417	7.442134301	protein_coding	12	49207577	49222724	CACNB3
ENSG00000167536	6.598204782	6.787424764	protein_coding	17	27224799	27230089	DHRS13
ENSG00000167543	7.712290964	7.887034762	protein_coding	17	27895739	27900172	TP53I13
ENSG00000167548	11.0104901	11.01275647	protein_coding	12	49412758	49453557	MLL2
ENSG00000167549	6.496213887	6.246885341	protein_coding	17	27941774	27949917	CORO6
ENSG00000167550	5.889353623	6.737844962	protein_coding	12	49458468	49463808	RHEBL1
ENSG00000167552	9.30119149	10.32407756	protein_coding	12	49578579	49583107	TUBA1A
ENSG00000167553	10.24214716	10.46912676	protein_coding	12	49582519	49667116	TUBA1C
ENSG00000167554	6.727618145	6.99159092	protein_coding	19	52839498	52870375	ZNF610
ENSG00000167555	8.801295662	9.126421125	protein_coding	19	52901102	52921657	ZNF528
ENSG00000167562	7.413224588	7.582164879	protein_coding	19	53073526	53090427	ZNF701
ENSG00000167565	6.244259325	6.445267191	protein_coding	19	40946749	40950282	SERTAD3
ENSG00000167566	9.26675844	9.425497321	protein_coding	12	50184929	50222533	NCKAP5L
ENSG00000167578	5.649112675	5.259915406	protein_coding	19	41277553	41302847	RAB4B
ENSG00000167588	2.928422289	2.420525079	protein_coding	12	50497602	50505102	GPD1
ENSG00000167595	8.66984994	8.506198656	protein_coding	19	36249044	36260766	C19orf55
ENSG00000167600	4.437288965	4.342020395	protein_coding	19	41699115	41713443	CYP2S1
ENSG00000167601	9.597483841	9.315729406	protein_coding	19	41725108	41767670	AXL
ENSG00000167604	6.35766608	6.316088276	protein_coding	19	36379143	36393185	NFKBID
ENSG00000167608	6.535310252	4.202780776	protein_coding	19	54663846	54676944	TMC4
ENSG00000167612	3.211941663	3.532712221	protein_coding	12	52281744	52285448	ANKRD33
ENSG00000167614	4.883502971	3.906303962	protein_coding	19	54926373	54948080	TTYH1
ENSG00000167615	11.61901592	11.59146528	protein_coding	19	54960065	54973217	LENG8
ENSG00000167617	6.616550626	4.075511708	protein_coding	19	54976210	54984411	CDC42EP5
ENSG00000167619	4.568725997	4.342020395	protein_coding	19	42817477	42829214	TMEM145
ENSG00000167625	6.393570237	6.475832214	protein_coding	19	42724448	42732353	ZNF526
ENSG00000167632	7.615275323	7.65742733	protein_coding	8	140742586	141468678	TRAPPC9
ENSG00000167634	0.499066092	0.697730409	protein_coding	19	55434877	55477611	NLRP7
ENSG00000167635	10.051359	9.864280473	protein_coding	19	36705504	36729673	ZNF146
ENSG00000167637	6.567100227	6.787424764	protein_coding	19	44331444	44353307	ZNF283
ENSG00000167642	9.904181598	7.36802722	protein_coding	19	38755098	38783253	SPINT2
ENSG00000167645	8.552470601	8.754928172	protein_coding	19	38794200	38806606	YIF1B
ENSG00000167646	7.893726093	7.343375265	protein_coding	19	55670031	55678090	C19orf51
ENSG00000167653	2.847891871	2.039052734	protein_coding	8	143751726	143764142	PSCA
ENSG00000167654	1.799000381	2.144285137	protein_coding	19	3880618	3928076	ATCAY
ENSG00000167656	4.543379137	0	protein_coding	8	143866296	143868008	LY6D
ENSG00000167657	10.18899434	10.53319908	protein_coding	19	3958452	3971038	DAPK3
ENSG00000167658	15.94761114	15.75055344	protein_coding	19	3972316	3985461	EEF2
ENSG00000167664	0	0.390640832	protein_coding	19	4292237	4302428	TMIGD2
ENSG00000167670	10.18746114	10.57703147	protein_coding	19	4402659	4443394	CHAF1A
ENSG00000167671	10.068119	10.39009024	protein_coding	19	4445006	4457791	UBXN6
ENSG00000167674	10.17152198	10.22222054	protein_coding	19	4472255	4502213	
ENSG00000167676	8.460675529	5.47691899	protein_coding	19	4502204	4517716	PLIN4
ENSG00000167680	9.090943324	8.887777807	protein_coding	19	4542600	4558507	SEMA6B

ENSG00000167685	8.956980309	8.956216712	protein_coding	19	56652556	56672261	ZNF444
ENSG00000167693	10.21531405	9.974965329	protein_coding	17	702581	883010	NXN
ENSG00000167695	8.899456749	9.375039375	protein_coding	17	635847	646074	FAM57A
ENSG00000167699	9.212512411	9.890987074	protein_coding	17	662550	686505	GLOD4
ENSG00000167700	7.952677993	7.920752191	protein_coding	8	145734457	145736569	MFSD3
ENSG00000167701	2.57521082	3.133298822	protein_coding	8	145728356	145732557	GPT
ENSG00000167702	6.733233509	6.708116507	protein_coding	8	145691426	145699585	KIFC2
ENSG00000167703	9.225519491	8.591248608	protein_coding	17	1477666	1532180	SLC43A2
ENSG00000167705	6.92698535	7.246248121	protein_coding	17	1549444	1553352	RILP
ENSG00000167711	3.950976309	3.027231696	protein_coding	17	1646130	1658560	SERPINF2
ENSG00000167716	9.971057625	10.51920468	protein_coding	17	1619817	1641893	WDR81
ENSG00000167720	6.364918751	6.699508919	protein_coding	17	2207248	2228554	SRR
ENSG00000167721	10.37335233	10.96530032	protein_coding	17	2225992	2240678	TSR1
ENSG00000167723	5.331395195	5.908941696	protein_coding	17	3413796	3461289	TRPV3
ENSG00000167733	5.575999561	6.192713747	protein_coding	19	5681035	5688532	HSD11B1L
ENSG00000167740	7.695130106	8.214555999	protein_coding	17	4046462	4060989	CYB5D2
ENSG00000167741	0.499066092	0	protein_coding	17	4460224	4463876	GGT6
ENSG00000167744	3.448790144	1.353254395	protein_coding	19	49564397	49567124	NTF4
ENSG00000167747	9.600559263	9.700312778	protein_coding	19	51300962	51307974	C19orf48
ENSG00000167748	0.499066092	0	protein_coding	19	51322404	51327043	KLK1
ENSG00000167754	1.16343121	0	protein_coding	19	51446561	51456344	KLK5
ENSG00000167755	6.305850085	0.697730409	protein_coding	19	51461888	51472929	KLK6
ENSG00000167765	2.10647801	2.971122874	protein_coding	19	52095036	52097633	
ENSG00000167766	9.31529953	9.448796166	protein_coding	19	53115620	53193741	ZNF83
ENSG00000167767	10.65540882	10.49453849	protein_coding	12	52562780	52585784	KRT80
ENSG00000167770	10.0287051	10.17952981	protein_coding	11	63753325	63769283	OTUB1
ENSG00000167771	4.883502971	4.676215056	protein_coding	11	63678693	63684316	RCOR2
ENSG00000167772	8.2535526	8.094946597	protein_coding	19	8429011	8439257	ANGPTL4
ENSG00000167774	8.847332411	9.701390938	protein_coding	19	8376234	8386280	NDUFA7
ENSG00000167775	11.04137297	11.57832085	protein_coding	19	8367011	8373240	CD320
ENSG00000167778	9.545769354	9.288725373	protein_coding	12	53458388	53473204	SPRYD3
ENSG00000167779	9.052156467	7.716212954	protein_coding	12	53491220	53496129	IGFBP6
ENSG00000167780	1.407729925	0	protein_coding	12	53497302	53518322	SOAT2
ENSG00000167785	8.793254009	8.885882287	protein_coding	19	8920382	8933565	ZNF558
ENSG00000167791	1.16343121	0	protein_coding	11	67286409	67290899	CABP2
ENSG00000167792	12.32463616	12.52958821	protein_coding	11	67374323	67380006	NDUFV1
ENSG00000167797	10.60154986	10.953358	protein_coding	11	67273968	67276120	CDK2AP2
ENSG00000167799	7.464764762	8.127396674	protein_coding	11	67395409	67397401	NUDT8
ENSG00000167800	1.616589159	1.802319292	protein_coding	11	67398774	67407031	TBX10
ENSG00000167807	8.009315251	8.595894166	protein_coding	19	10416103	10426691	FDX1L
ENSG00000167815	11.14242766	11.5407157	protein_coding	19	12907634	12912694	PRDX2
ENSG00000167840	7.46813618	7.789016615	protein_coding	17	5008930	5026397	ZNF232
ENSG00000167842	8.56354495	9.351881826	protein_coding	17	5390247	5394134	MIS12
ENSG00000167850	0.499066092	1.16600992	protein_coding	17	72537247	72542282	CD300C
ENSG00000167858	0.499066092	1.16600992	protein_coding	17	6701312	6735080	TEKT1
ENSG00000167861	4.491309013	1.925536307	protein_coding	17	72946837	72969261	C17orf28
ENSG00000167862	7.96466218	8.099860884	protein_coding	17	73008780	73017355	ICT1
ENSG00000167863	9.225519491	9.726498497	protein_coding	17	73032145	73043074	ATP5H
ENSG00000167874	3.871730003	3.081239798	protein_coding	17	7758384	7759415	TMEM88
ENSG00000167880	9.046529371	7.780879746	protein_coding	17	74002927	74023507	EVPL
ENSG00000167881	9.931305133	9.754816173	protein_coding	17	74035187	74068607	SRP68
ENSG00000167889	6.892270075	7.263870273	protein_coding	17	74864538	74946475	MGAT5B
ENSG00000167895	2.928422289	1.802319292	protein_coding	17	76126859	76139049	TMC8
ENSG00000167900	10.3309294	10.23790941	protein_coding	17	76170160	76183314	TK1
ENSG00000167904	6.393570237	6.064512706	protein_coding	8	56608983	56685966	TMEM68
ENSG00000167910	0	0.950786998	protein_coding	8	59402737	59412795	CYP7A1
ENSG00000167912	0	3.813624741	processed_transc	8	60031599	60033905	
ENSG00000167914	0.499066092	0	protein_coding	17	38119226	38134019	GSDMA
ENSG00000167920	6.515894502	6.819555981	protein_coding	17	38975358	38992522	TMEM99
ENSG00000167925	8.282565319	8.242970684	protein_coding	17	40341106	40346550	GHDC
ENSG00000167930	10.30335447	10.83554492	protein_coding	16	284545	319942	ITFG3
ENSG00000167941	0.499066092	0	protein_coding	17	41831103	41836156	SOST
ENSG00000167945	1.407729925	1.353254395	protein_coding	16	855443	863861	PRR25

ENSG00000167962	10.23820324	10.25819969	protein_coding	16	2047655	2059824	ZNF598
ENSG00000167964	5.40360309	5.03207928	protein_coding	16	2190804	2204166	RAB26
ENSG00000167965	9.74513791	9.888622555	protein_coding	16	2254249	2259417	MLST8
ENSG00000167967	9.077762113	9.315025211	protein_coding	16	2273560	2285743	E4F1
ENSG00000167968	4.292665995	4.810179682	protein_coding	16	2285817	2288712	DNASE1L2
ENSG00000167969	9.232475031	9.417646668	protein_coding	16	2289396	2302301	ECI1
ENSG00000167971	1.16343121	2.242360793	protein_coding	16	2227184	2246526	CASKIN1
ENSG00000167972	11.46638963	11.64399908	protein_coding	16	2325882	2390747	ABCA3
ENSG00000167977	9.779466207	9.611339686	protein_coding	16	2732476	2759031	KCTD5
ENSG00000167978	13.64492996	13.62190384	protein_coding	16	2802330	2821411	SRRM2
ENSG00000167981	5.175139844	4.342020395	protein_coding	16	3486104	3493537	ZNF597
ENSG00000167984	4.464551814	4.794098771	protein_coding	16	3589038	3627401	NLRC3
ENSG00000167985	6.023726639	6.539915281	protein_coding	11	61197514	61215001	SDHAF2
ENSG00000167986	12.25266173	12.23179918	protein_coding	11	61066923	61110068	DDB1
ENSG00000167987	8.774313977	8.761145731	protein_coding	11	60897728	60929089	VPS37C
ENSG00000167992	3.871730003	4.127774132	protein_coding	11	61025762	61062896	VWCE
ENSG00000167994	7.527512071	7.664078611	protein_coding	11	61664773	61687741	RAB3IL1
ENSG00000167995	4.689181911	5.283144778	protein_coding	11	61717293	61732987	BEST1
ENSG00000167996	12.38752564	12.48121023	protein_coding	11	61727190	61735132	FTH1
ENSG00000168000	5.316509574	5.506827567	protein_coding	11	62457747	62477317	BSCL2
ENSG00000168002	8.655134734	9.281534232	protein_coding	11	62529016	62534182	POLR2G
ENSG00000168003	11.90507449	11.93316881	protein_coding	11	62623518	62656352	SLC3A2
ENSG00000168005	8.2535526	8.090015513	protein_coding	11	63580860	63595190	C11orf84
ENSG00000168010	8.135536446	9.133627746	protein_coding	11	72525353	72554719	ATG16L2
ENSG00000168014	8.593182305	8.680338024	protein_coding	11	73723763	73882255	C2CD3
ENSG00000168016	8.628263112	9.101319098	protein_coding	3	36868311	36986548	TRANK1
ENSG00000168026	4.593635215	4.990346085	protein_coding	3	39149152	39180394	TTC21A
ENSG00000168028	10.71621744	10.93491337	protein_coding	3	39448180	39453929	RPSA
ENSG00000168032	1.407729925	1.353254395	protein_coding	3	40428647	40470110	ENTPD3
ENSG00000168036	11.41665449	11.37864174	protein_coding	3	41236328	41301587	CTNNB1
ENSG00000168038	6.283060568	6.030599799	protein_coding	3	41288090	42003922	ULK4
ENSG00000168040	7.581430769	8.015686953	protein_coding	11	70049269	70053496	FADD
ENSG00000168056	9.615838797	10.46817693	protein_coding	11	65306031	65326401	LTBP3
ENSG00000168060	0.869158192	2.420525079	protein_coding	11	64812290	64826021	NAALADL1
ENSG00000168061	7.978913023	7.982367515	protein_coding	11	64808373	64812300	SAC3D1
ENSG00000168062	6.738827102	7.135740508	protein_coding	11	64755415	64764517	BATF2
ENSG00000168065	0.869158192	0.697730409	protein_coding	11	64323098	64340347	SLC22A11
ENSG00000168066	11.76493205	11.37931557	protein_coding	11	64532078	64546258	SF1
ENSG00000168067	8.320363082	7.999999944	protein_coding	11	64556290	64570713	MAP4K2
ENSG00000168070	3.652182994	3.570966319	protein_coding	11	64704989	64739557	C11orf85
ENSG00000168071	7.543580762	6.923001245	protein_coding	11	64107690	64125006	CCDC88B
ENSG00000168078	8.45218826	8.802929991	protein_coding	8	27667137	27695612	PBK
ENSG00000168079	0.869158192	0	protein_coding	8	27727399	27850244	SCARA5
ENSG00000168081	0	0.390640832	protein_coding	8	28174503	28200872	PNOC
ENSG00000168090	10.30004761	9.956570886	protein_coding	7	99686577	99689823	COPS6
ENSG00000168092	10.65281865	10.38741313	protein_coding	11	117014983	117047610	PAFAH1B2
ENSG00000168096	7.399163791	7.714077892	protein_coding	16	4746513	4784379	ANKS3
ENSG00000168101	6.917151646	7.56568256	protein_coding	16	4743695	4745860	NUDT16L1
ENSG00000168116	6.059927234	6.563824016	protein_coding	6	56911347	56920023	KIAA1586
ENSG00000168118	7.413224588	7.668495823	protein_coding	1	229406822	229441641	RAB4A
ENSG00000168122	0	0.390640832	pseudogene	21	14467626	14486111	ZNF355P
ENSG00000168124	2.471521042	2.420525079	protein_coding	16	3254247	3255188	OR1F1
ENSG00000168126	0	0.950786998	pseudogene	6	27905243	27906181	OR2W6P
ENSG00000168131	0	0.390640832	protein_coding	6	27878963	27880174	OR2B2
ENSG00000168137	10.79182236	10.42542696	protein_coding	3	9439299	9520924	SETD5
ENSG00000168140	9.636961872	10.0885135	protein_coding	16	4421849	4433529	VASN
ENSG00000168148	0.869158192	0.390640832	protein_coding	1	228612546	228613026	HIST3H3
ENSG00000168152	5.331395195	5.516660875	protein_coding	4	83821837	83841438	THAP9
ENSG00000168158	0	0.697730409	protein_coding	16	3405889	3406924	OR2C1
ENSG00000168159	10.36885012	10.58902301	protein_coding	1	228674762	228683467	RNF187
ENSG00000168172	6.749949642	7.309836726	protein_coding	8	42752075	42883255	HOOK3
ENSG00000168175	9.770619129	9.630144961	protein_coding	14	55518349	55532931	MAPK1IP1L
ENSG00000168209	11.0693905	10.9655247	protein_coding	10	74033678	74035794	DDIT4

ENSG00000168214	9.976986307	10.19300686	protein_coding	4	26165077	26436541	RBPJ
ENSG00000168216	7.165790811	6.919299326	protein_coding	6	70385694	70507003	LMBRD1
ENSG00000168228	6.573375019	6.414040573	protein_coding	4	25314407	25372005	ZCCHC4
ENSG00000168234	7.190493718	7.255086103	protein_coding	18	21572737	21715573	TTC39C
ENSG00000168237	4.261916566	5.045726316	protein_coding	3	52321105	52329272	GLYCTK
ENSG00000168242	1.16343121	1.518964905	protein_coding	6	26273144	26273622	HIST1H2BI
ENSG00000168243	0.499066092	0	protein_coding	1	235710987	235814054	GNG4
ENSG00000168246	8.239811039	8.557113361	protein_coding	5	171636644	171711075	UBTD2
ENSG00000168255	6.26766507	6.136428444	protein_coding	7	102178365	102232891	POLR2J3
ENSG00000168256	8.807962967	8.401295742	protein_coding	17	40169038	40177659	NKIRAS2
ENSG00000168259	9.475476535	9.423538659	protein_coding	17	40128439	40169715	DNAJC7
ENSG00000168260	0.869158192	1.353254395	protein_coding	14	50550369	50559361	C14orf183
ENSG00000168263	0.499066092	1.518964905	protein_coding	9	2717239	2730037	KCNV2
ENSG00000168264	9.792972033	9.643108201	protein_coding	1	234740015	234745271	IRF2BP2
ENSG00000168268	9.710684602	9.656512011	protein_coding	3	52558386	52569070	NT5DC2
ENSG00000168269	0.499066092	1.16600992	protein_coding	5	169532901	169536727	FOXI1
ENSG00000168273	6.212449006	6.966698819	protein_coding	3	52568029	52613253	C3orf78
ENSG00000168274	3.335220907	4.17820932	protein_coding	6	26217165	26217711	HIST1H2AE
ENSG00000168275	7.44778847	8.046558348	protein_coding	1	234509202	234519795	C1orf31
ENSG00000168280	0	0.697730409	protein_coding	2	149632819	149883273	KIF5C
ENSG00000168282	5.538004898	5.66550919	protein_coding	14	50087489	50090198	MGAT2
ENSG00000168283	9.423456371	9.269226422	protein_coding	10	22605381	22620413	BMI1
ENSG00000168286	8.13977083	8.401295742	protein_coding	16	67876213	67878097	THAP11
ENSG00000168288	9.758967401	9.690572902	protein_coding	2	150426148	150444330	MMADHC
ENSG00000168291	9.235445742	9.229419632	protein_coding	3	58413357	58419584	PDHB
ENSG00000168297	8.876804446	8.936206949	protein_coding	3	58318607	58411748	PXK
ENSG00000168298	4.517578978	5.306006045	protein_coding	6	26156559	26157343	HIST1H1E
ENSG00000168300	7.311793575	7.701200733	protein_coding	8	52730140	52811735	PCMTD1
ENSG00000168301	6.032862302	5.840125386	protein_coding	3	58477841	58488087	KCTD6
ENSG00000168303	8.805299741	8.738215808	protein_coding	7	40172342	40174258	C7orf11
ENSG00000168306	5.175139844	4.932754837	protein_coding	3	58490863	58523046	ACOX2
ENSG00000168309	1.407729925	0	protein_coding	3	58549844	58613337	FAM107A
ENSG00000168310	8.085926212	8.525807191	protein_coding	4	185308867	185395734	IRF2
ENSG00000168314	0	0.390640832	protein_coding	3	39508689	39567859	MOBP
ENSG00000168334	1.407729925	1.16600992	protein_coding	3	39224701	39234087	XIRP1
ENSG00000168350	3.211941663	2.144285137	protein_coding	14	100612756	100626500	DEGS2
ENSG00000168367	1.16343121	0	protein_coding	16	86369374	86379285	
ENSG00000168374	11.33129517	11.33015167	protein_coding	3	57557090	57583947	ARF4
ENSG00000168385	11.48793241	11.25522647	protein_coding	2	242254515	242293442	2-sep
ENSG00000168386	8.441937187	9.112709611	protein_coding	3	99548985	99833357	FILIP1L
ENSG00000168389	6.212449006	6.619638408	protein_coding	1	40420802	40435638	MFSD2A
ENSG00000168393	8.977234519	9.0649234	protein_coding	2	242615157	242626406	DTYMK
ENSG00000168394	9.809013881	10.32162561	protein_coding	6	32812986	32821755	TAP1
ENSG00000168395	8.143992822	7.877527247	protein_coding	2	242641450	242668893	ING5
ENSG00000168397	9.814983883	9.828213847	protein_coding	2	242576628	242613272	ATG4B
ENSG00000168398	7.420203928	6.835357159	protein_coding	14	96671016	96710666	BDKRB2
ENSG00000168404	8.403714162	7.782918269	protein_coding	16	74705755	74734789	MLKL
ENSG00000168405	2.238690726	2.788380093	protein_coding	6	25081295	25218698	CMAHP
ENSG00000168411	10.13274625	10.05895686	protein_coding	16	74655297	74700779	RFWD3
ENSG00000168412	0	0.390640832	protein_coding	4	187347700	187476721	MTNR1A
ENSG00000168427	2.238690726	1.16600992	protein_coding	2	239047363	239061588	KLHL30
ENSG00000168434	8.103376106	7.864110577	protein_coding	16	23399814	23464501	COG7
ENSG00000168438	7.834876234	7.426571535	protein_coding	6	110501344	110575478	CDC40
ENSG00000168439	11.68732373	11.44419103	protein_coding	11	63952744	63972015	STIP1
ENSG00000168447	0.869158192	0.697730409	protein_coding	16	23289552	23392620	SCNN1B
ENSG00000168453	6.469548347	4.202780776	protein_coding	8	21971928	21990897	HR
ENSG00000168454	0.499066092	1.16600992	protein_coding	18	9885763	9888377	TXNDC2
ENSG00000168461	10.17255565	9.33252778	protein_coding	18	9708228	9862553	RAB31
ENSG00000168476	9.138269512	8.870627795	protein_coding	8	21995533	21999464	REEP4
ENSG00000168477	7.149084042	7.03670965	protein_coding	6	32008931	32083111	TNXB
ENSG00000168481	0.869158192	0.697730409	protein_coding	8	22004338	22014597	LGI3
ENSG00000168487	9.895443335	10.38338814	protein_coding	8	22022249	22069839	BMP1
ENSG00000168488	11.56217774	11.64455972	protein_coding	16	28834356	28848558	ATXN2L

ENSG00000168490	3.911897206	3.64455972	protein_coding	8	22077222	22089854	PHYHIP
ENSG00000168491	2.238690726	2.501982735	protein_coding	4	186366336	186392913	CCDC110
ENSG00000168495	8.544507981	8.718115101	protein_coding	8	22102617	22109485	POLR3D
ENSG00000168496	9.479658457	9.831664428	protein_coding	11	61560109	61564716	FEN1
ENSG00000168497	4.230497448	2.334191469	protein_coding	2	192699028	192711981	SDPR
ENSG00000168502	8.927893208	8.785751426	protein_coding	18	8706513	8832776	CCDC165
ENSG00000168505	0.869158192	0.390640832	protein_coding	2	237073879	237077012	GBX2
ENSG00000168517	5.858855738	5.98174124	protein_coding	17	43238271	43247406	HEXIM2
ENSG00000168522	8.096857104	8.706348549	protein_coding	8	42889337	42940931	FNTA
ENSG00000168528	10.13963411	7.819127564	protein_coding	1	31882412	31907525	SERINC2
ENSG00000168538	7.751556534	7.862183683	protein_coding	4	184580420	184634745	TRAPPC11
ENSG00000168542	1.407729925	2.501982735	protein_coding	2	189839046	189877472	COL3A1
ENSG00000168556	6.228441838	6.619638408	protein_coding	4	184426147	184432249	ING2
ENSG00000168564	7.258272112	7.607691112	protein_coding	4	184365744	184369351	CDKN2AIP
ENSG00000168566	7.983632192	8.219079902	protein_coding	6	7590432	7612091	SNRNP48
ENSG00000168569	7.612231145	7.73107057	protein_coding	11	62539101	62559493	TMEM223
ENSG00000168575	8.680062001	8.617758557	protein_coding	8	42273993	42397069	SLC20A2
ENSG00000168589	0	0.950786998	protein_coding	16	80574631	80584657	DYNLRB2
ENSG00000168591	8.177331204	8.302443832	protein_coding	17	42264336	42269098	TMUB2
ENSG00000168594	0.499066092	0	protein_coding	4	175750819	175899331	ADAM29
ENSG00000168597	6.364918751	6.365895578	protein_coding	17	42253354	42264082	C17orf65
ENSG00000168610	10.73278039	10.51583798	protein_coding	17	40465342	40540449	STAT3
ENSG00000168612	7.768062907	7.705505903	protein_coding	20	44509866	44513905	ZSWIM1
ENSG00000168614	7.420203928	7.724721747	protein_coding	1	144811744	144830302	NBPF9
ENSG00000168615	11.36288118	11.43173959	protein_coding	8	38854388	38962663	ADAM9
ENSG00000168619	0	0.390640832	protein_coding	8	39442008	39587584	ADAM18
ENSG00000168621	0	0.390640832	protein_coding	5	37812779	37839788	GDNF
ENSG00000168634	1.16343121	3.183544561	protein_coding	20	44330655	44337456	WFDC13
ENSG00000168646	5.036678094	5.726093467	protein_coding	17	63524685	63557765	AXIN2
ENSG00000168653	9.740266097	10.46754337	protein_coding	1	39491990	39500308	NDUFS5
ENSG00000168658	1.16343121	0.390640832	protein_coding	2	98703579	98929762	VWA3B
ENSG00000168661	4.962122459	5.125003832	protein_coding	19	35417807	35436074	ZNF30
ENSG00000168672	9.886021803	8.17474298	protein_coding	8	127564687	127570638	FAM84B
ENSG00000168675	1.16343121	1.16600992	protein_coding	18	13218786	13652753	C18orf1
ENSG00000168676	4.261916566	3.368666104	protein_coding	16	67323331	67360666	KCTD19
ENSG00000168679	2.10647801	2.501982735	protein_coding	1	110905470	110933704	SLC16A4
ENSG00000168685	10.56028737	10.05769455	protein_coding	5	35852797	35879705	IL7R
ENSG00000168701	7.938164312	8.599368544	protein_coding	16	67261006	67263181	TMEM208
ENSG00000168702	1.16343121	0.390640832	protein_coding	2	140988992	142889270	LRP1B
ENSG00000168703	0	0.390640832	protein_coding	20	43752066	43753106	WFDC12
ENSG00000168710	10.78710593	10.68779701	protein_coding	1	110527308	110566357	AHCYL1
ENSG00000168724	9.862515511	9.488685117	protein_coding	5	34929698	34959069	DNAJC21
ENSG00000168734	8.259402007	9.494294562	protein_coding	20	43160426	43252888	PKIG
ENSG00000168743	0	0.697730409	protein_coding	4	106815932	106925184	NPNT
ENSG00000168748	1.799000381	0.390640832	protein_coding	16	66878282	66888056	CA7
ENSG00000168754	2.471521042	0	protein_coding	2	97541620	97684175	FAM178B
ENSG00000168758	9.265790136	8.154416734	protein_coding	2	97525453	97536494	SEMA4C
ENSG00000168763	8.325948295	8.214555999	protein_coding	2	97481982	97499648	CNNM3
ENSG00000168765	8.124895791	8.127396674	protein_coding	1	110198703	110208118	GSTM4
ENSG00000168769	7.250461488	7.666288907	protein_coding	4	106067032	106200973	TET2
ENSG00000168778	7.308035767	6.771086752	protein_coding	12	124155660	124192948	TCTN2
ENSG00000168779	3.603959378	5.150490275	protein_coding	3	157813743	157824292	SHOX2
ENSG00000168781	7.845284967	7.519971735	protein_coding	15	43825660	43982283	PPIP5K1
ENSG00000168785	8.783815073	8.664945797	protein_coding	4	99391518	99579780	TSPAN5
ENSG00000168787	0	0.697730409	protein_coding	6	29364416	29365448	OR12D2
ENSG00000168792	6.830742851	6.264499759	protein_coding	17	27887691	27894042	ABHD15
ENSG00000168795	7.507990374	8.044860491	protein_coding	9	37438111	37465396	ZBTB5
ENSG00000168802	10.07753061	9.770824962	protein_coding	16	69151913	69166487	CHTF8
ENSG00000168803	6.687686437	6.615068802	protein_coding	15	43622872	43646096	ADAL
ENSG00000168806	7.071412799	7.126137778	protein_coding	15	43619974	43622803	LCMT2
ENSG00000168807	9.066685021	8.891561392	protein_coding	16	69221032	69342955	SNTB2
ENSG00000168811	4.026095388	3.748356452	protein_coding	3	159706537	159713806	IL12A
ENSG00000168813	8.225937332	7.877527247	protein_coding	19	32836514	32878572	ZNF507

ENSG00000168818	7.609180529	8.032919217	protein_coding	4	4417469	4544073	STX18
ENSG00000168824	1.407729925	0	protein_coding	4	4349867	4420785	
ENSG00000168826	5.458875168	5.187895122	protein_coding	4	4291924	4323513	ZBTB49
ENSG00000168827	9.354083505	9.281534232	protein_coding	3	158362067	158410364	GFM1
ENSG00000168828	0	0.697730409	protein_coding	9	35869375	35870461	OR13J1
ENSG00000168843	0.499066092	0	protein_coding	4	162305049	163085187	FSTL5
ENSG00000168852	0	0.390640832	pseudogene	13	41396432	41495885	
ENSG00000168872	8.839544001	8.923327429	protein_coding	16	70380732	70407286	DDX19A
ENSG00000168874	5.4452544	5.700439663	protein_coding	2	85978467	86015189	ATOH8
ENSG00000168876	6.548110424	7.135740508	protein_coding	11	94226795	94232749	ANKRD49
ENSG00000168878	0.869158192	0.950786998	protein_coding	2	85884437	85895864	SFTPB
ENSG00000168883	10.00336794	9.994448246	protein_coding	2	85829979	85876406	USP39
ENSG00000168884	8.387780826	8.525807191	protein_coding	4	2743375	2758103	TNIP2
ENSG00000168887	8.233881494	8.099860884	protein_coding	2	85833777	85839189	C2orf68
ENSG00000168890	7.017207048	7.575124073	protein_coding	2	85825671	85830319	TMEM150A
ENSG00000168894	9.181013921	9.52932336	protein_coding	2	85822848	85824736	RNF181
ENSG00000168899	3.077135474	3.027231696	protein_coding	2	85811531	85820535	VAMP5
ENSG00000168904	6.393570237	6.398170093	protein_coding	15	99791567	99930934	LRRC28
ENSG00000168906	11.19729137	11.75018096	protein_coding	2	85766288	85772403	MAT2A
ENSG00000168907	2.471521042	0	protein_coding	15	42433332	42448839	PLA2G4F
ENSG00000168913	3.335220907	5.137803333	protein_coding	9	34521038	34523039	ENHO
ENSG00000168916	8.233881494	8.371803145	protein_coding	5	123972608	124084500	ZNF608
ENSG00000168917	5.346128795	4.7279204	protein_coding	3	136537489	136574734	TMEM22
ENSG00000168918	2.671945279	0.390640832	protein_coding	2	233924677	234116549	INPP5D
ENSG00000168924	10.57091017	10.16201509	protein_coding	4	1813206	1857974	LETM1
ENSG00000168925	1.16343121	0.697730409	protein_coding	16	75252898	75258822	CTRB1
ENSG00000168928	1.799000381	0.950786998	protein_coding	16	75237994	75241083	CTRB2
ENSG00000168936	7.873527714	8.171634445	protein_coding	4	1717679	1723085	TMEM129
ENSG00000168938	7.80585884	8.289597152	protein_coding	5	122358945	122372436	PPIC
ENSG00000168939	1.616589159	0.697730409	protein_coding	X	154997474	155012121	SPRY3
ENSG00000168944	7.420203928	7.408200217	protein_coding	5	122680579	122759286	CEP120
ENSG00000168952	0	0.390640832	protein_coding	14	25278862	25519503	STXBP6
ENSG00000168955	0	1.16600992	protein_coding	2	228226872	228246711	TM4SF20
ENSG00000168958	8.992533411	9.234642156	protein_coding	2	228189867	228222550	MFF
ENSG00000168961	4.230497448	4.42790041	protein_coding	17	25956824	25976586	LGALS9
ENSG00000168967	0	0.390640832	pseudogene	5	22142461	22152465	PMCHL1
ENSG00000168970	5.124761037	5.137803333	protein_coding	15	42120283	42140353	JMJD7-PLA2G4B
ENSG00000168992	1.16343121	0.950786998	pseudogene	2	96212279	96213315	
ENSG00000168993	7.947856304	7.534563804	protein_coding	4	778745	819986	CPLX1
ENSG00000168994	9.153007996	9.071609459	protein_coding	6	3722854	3752260	PXDC1
ENSG00000169016	7.654275977	7.113233895	protein_coding	2	11584501	11606297	E2F6
ENSG00000169018	9.035208904	8.976843446	protein_coding	15	68570141	68588203	FEM1B
ENSG00000169019	6.897280837	7.040122572	protein_coding	4	47452885	47465736	COMMD8
ENSG00000169020	7.136425764	7.944622739	protein_coding	4	666225	668127	ATP5I
ENSG00000169021	8.638772743	8.772475459	protein_coding	19	29698167	29704136	UQCRFS1
ENSG00000169026	5.207774174	4.202780776	protein_coding	4	675618	683230	MFSD7
ENSG00000169031	0.869158192	1.518964905	protein_coding	2	228029281	228179508	COL4A3
ENSG00000169032	9.381161578	9.602132979	protein_coding	15	66679155	66784754	MAP2K1
ENSG00000169035	3.652182994	0	protein_coding	19	51479729	51487320	KLK7
ENSG00000169045	11.84231745	11.98610646	protein_coding	5	179041179	179061785	HNRNPH1
ENSG00000169047	9.330197577	7.321103092	protein_coding	2	227599757	227664475	IRS1
ENSG00000169057	9.895443335	9.783098001	protein_coding	X	153287024	153402578	MECP2
ENSG00000169062	7.737655476	8.096586553	protein_coding	13	115047059	115071283	UPF3A
ENSG00000169067	3.004694206	3.232099092	protein_coding	5	56775846	56778636	ACTBL2
ENSG00000169071	0.869158192	0.697730409	protein_coding	9	94325373	94712444	ROR2
ENSG00000169084	6.482942724	6.695185789	protein_coding	X	2137557	2420846	DHRSX
ENSG00000169085	4.097495944	4.990346085	protein_coding	8	67372238	67430759	C8orf46
ENSG00000169087	6.664362065	6.970281236	protein_coding	3	122458846	122512671	HSPBAP1
ENSG00000169093	9.163444055	9.025004422	protein_coding	X	1522032	1572655	ASMTL
ENSG00000169100	12.1479012	12.08927076	protein_coding	X	1505045	1511626	SLC25A6
ENSG00000169105	7.60612345	7.577474828	protein_coding	15	40763160	40765353	CHST14
ENSG00000169116	3.554067925	2.912743273	protein_coding	4	75858305	75975325	PARM1
ENSG00000169118	8.099033379	7.612284109	protein_coding	15	64457716	64665968	CSNK1G1

ENSG00000169126	4.409500985	5.751299057	protein_coding	10	28064115	28287977	ARMC4
ENSG00000169129	7.537174746	4.622587519	protein_coding	10	116054583	116164515	AFAP1L2
ENSG00000169131	7.44778847	7.711939664	protein_coding	5	178138593	178157703	ZNF354A
ENSG00000169136	7.618313091	8.363653959	protein_coding	19	50431974	50437192	ATF5
ENSG00000169139	8.447071828	8.621180739	protein_coding	8	48920960	48976511	UBE2V2
ENSG00000169155	8.605481314	8.270836518	protein_coding	9	129567285	129600489	ZBTB43
ENSG00000169169	5.785082014	5.583678991	protein_coding	19	50194373	50216988	CPT1C
ENSG00000169174	1.799000381	0	protein_coding	1	55505221	55530525	PCSK9
ENSG00000169180	11.49331992	11.22761589	protein_coding	16	28109300	28223241	XPO6
ENSG00000169181	1.407729925	0.390640832	protein_coding	16	27798850	28074830	GSG1L
ENSG00000169184	8.458982065	8.658298522	protein_coding	22	28144265	28197486	MN1
ENSG00000169188	9.466233411	9.679673133	protein_coding	X	55026790	55035490	APEX2
ENSG00000169189	8.497437504	8.514810202	protein_coding	16	27236312	27280115	NSMCE1
ENSG00000169193	6.846372398	7.295628905	protein_coding	7	23636998	23684327	CCDC126
ENSG00000169194	0	0.950786998	protein_coding	5	131991955	131996802	IL13
ENSG00000169203	0.869158192	0.697730409	protein_coding	16	29495010	29517320	
ENSG00000169213	4.292665995	3.781359661	protein_coding	1	52373628	52456436	RAB3B
ENSG00000169217	9.796999249	9.913956561	protein_coding	16	30362087	30366682	CD2BP2
ENSG00000169218	0.499066092	0	protein_coding	1	38076951	38100595	RSPO1
ENSG00000169220	6.830742851	6.24096575	protein_coding	5	176784838	176799602	RGS14
ENSG00000169221	10.03383333	10.05516663	protein_coding	16	30368423	30381585	TBC1D10B
ENSG00000169223	10.05472661	10.16005582	protein_coding	5	176758563	176778853	LMAN2
ENSG00000169228	5.485736881	6.009862691	protein_coding	5	176728199	176730745	RAB24
ENSG00000169230	8.090308512	8.449576416	protein_coding	5	176730775	176733960	PRELID1
ENSG00000169231	8.972494341	10.57556225	protein_coding	1	155165379	155178842	THBS3
ENSG00000169239	6.393570237	6.210997888	protein_coding	X	15706953	15805747	CA5B
ENSG00000169241	8.658089807	8.620040914	protein_coding	1	155107820	155111329	SLC50A1
ENSG00000169242	10.46647804	11.81909835	protein_coding	1	155099936	155107333	EFNA1
ENSG00000169245	4.491309013	7.20731193	protein_coding	4	76942273	76944650	CXCL10
ENSG00000169246	4.517578978	3.96493948	protein_coding	16	21413548	21459888	NPIPL3
ENSG00000169247	4.821592551	3.411459265	protein_coding	5	148303202	148442726	SH3TC2
ENSG00000169248	4.19837882	6.210997888	protein_coding	4	76954835	76962568	CXCL11
ENSG00000169249	5.858855738	6.123616685	protein_coding	X	15808595	15841383	ZRSR2
ENSG00000169251	9.699971044	9.591123153	protein_coding	3	160822484	160971320	NMD3
ENSG00000169252	7.169937448	5.09905904	protein_coding	5	148206156	148208196	ADRB2
ENSG00000169253	0.499066092	1.353254395	pseudogene	9	111389113	111389430	
ENSG00000169255	7.066972635	6.59664425	protein_coding	3	160801671	160823172	B3GALNT1
ENSG00000169258	8.460675529	8.586588042	protein_coding	5	176022803	176037134	GPRIN1
ENSG00000169282	3.502389126	3.183544561	protein_coding	3	155755490	156256545	KCNAB1
ENSG00000169288	6.640654398	7.48283249	protein_coding	4	78783674	78882347	MRPL1
ENSG00000169291	0.869158192	0.390640832	protein_coding	1	154442248	154474589	SHE
ENSG00000169299	8.922987828	9.242810972	protein_coding	4	37828255	37864558	PGM2
ENSG00000169302	0.869158192	0	protein_coding	5	146614526	146767415	STK32A
ENSG00000169306	2.671945279	1.518964905	protein_coding	X	28605516	29974840	IL1RAPL1
ENSG00000169314	3.077135474	2.501982735	protein_coding	22	24105208	24108048	C22orf15
ENSG00000169330	5.538004898	5.923798455	protein_coding	15	79724858	79764632	KIAA1024
ENSG00000169359	8.666918882	9.059047571	protein_coding	3	155544305	155572218	SLC33A1
ENSG00000169371	6.400645076	7.016060809	protein_coding	15	75890424	75918810	SNUPN
ENSG00000169372	5.81716234	5.974624386	protein_coding	12	94071151	94288616	CRADD
ENSG00000169375	9.883498968	9.551016476	protein_coding	15	75661720	75748183	SIN3A
ENSG00000169379	7.853042529	7.84667518	protein_coding	3	93698983	93774512	ARL13B
ENSG00000169385	0	0.390640832	protein_coding	14	21423611	21424595	RNASE2
ENSG00000169397	0.869158192	0.697730409	protein_coding	14	21359558	21360507	RNASE3
ENSG00000169398	9.784207642	9.511607983	protein_coding	8	141667999	142012315	PTK2
ENSG00000169402	0	0.390640832	protein_coding	7	6793740	6838996	RSPH10B2
ENSG00000169403	5.718698586	4.226940739	protein_coding	1	28473677	28520447	PTAFR
ENSG00000169410	8.555643385	8.437980325	protein_coding	15	75759462	75871630	PTPN9
ENSG00000169418	6.585843317	5.988823159	protein_coding	1	153651113	153666468	NPR1
ENSG00000169429	10.8306468	11.36373741	protein_coding	4	74606223	74609433	IL8
ENSG00000169432	1.616589159	0.697730409	protein_coding	2	167051695	167232503	SCN9A
ENSG00000169435	3.335220907	2.144285137	protein_coding	4	74437267	74486348	RASSF6
ENSG00000169439	3.698846687	7.04692429	protein_coding	8	97505579	97624000	SDC2
ENSG00000169442	0.869158192	0	protein_coding	1	26644448	26647014	CD52

ENSG00000169446	8.925442603	8.923327429	protein_coding	X	135044229	135056222	MMGT1
ENSG00000169469	1.960915222	0	protein_coding	1	153003678	153005376	SPRR1B
ENSG00000169490	7.803191723	8.229580833	protein_coding	8	38846327	38854343	TM2D2
ENSG00000169495	0.869158192	0.950786998	protein_coding	8	38831683	38846181	HTRA4
ENSG00000169499	8.879339004	8.359562039	protein_coding	8	38758753	38831428	PLEKHA2
ENSG00000169504	11.47646902	11.73525669	protein_coding	1	25071848	25170815	CLIC4
ENSG00000169508	1.16343121	0.390640832	protein_coding	13	99946784	99959659	GPR183
ENSG00000169519	7.990682129	7.584504199	protein_coding	11	28129795	28355054	METTL15
ENSG00000169548	2.471521042	2.242360793	protein_coding	22	22868060	22874613	ZNF280A
ENSG00000169554	7.603059879	7.743685008	protein_coding	2	145121063	145282147	ZEB2
ENSG00000169564	11.05992546	11.0248651	protein_coding	2	70314585	70316332	PCBP1
ENSG00000169567	10.30900579	11.00688187	protein_coding	5	130494720	130507428	HINT1
ENSG00000169570	6.103936836	6.535085617	protein_coding	5	118173017	118324240	DTWD2
ENSG00000169583	7.847875458	7.858322159	protein_coding	9	139889087	139891255	CLIC3
ENSG00000169592	8.713115882	9.113519788	protein_coding	16	30006615	30017114	INO80E
ENSG00000169594	3.502389126	3.081239798	protein_coding	15	83924655	83953466	BNC1
ENSG00000169598	6.379315619	6.429738366	protein_coding	1	3773845	3801993	DFFB
ENSG00000169599	6.872051157	7.397596322	protein_coding	2	69622882	69664760	NFU1
ENSG00000169604	6.554468172	4.274079748	protein_coding	2	69240310	69476459	ANTXR1
ENSG00000169607	8.711694395	8.343077302	protein_coding	2	113493930	113522254	CKAP2L
ENSG00000169609	6.604346021	6.642271819	protein_coding	15	83657193	83680393	C15orf40
ENSG00000169612	6.335686705	6.803579823	protein_coding	15	83654959	83659809	FAM103A1
ENSG00000169618	0	0.697730409	protein_coding	2	68870721	68882708	PROKR1
ENSG00000169621	5.270908378	5.620622115	protein_coding	2	68694693	68858004	APLF
ENSG00000169627	3.274897671	3.714580548	protein_coding	16	30204255	30205627	BOLA2B
ENSG00000169629	3.146112541	3.906303962	protein_coding	2	113127669	113192065	RGPD8
ENSG00000169635	7.67484765	7.119700263	protein_coding	22	21771693	21805752	HIC2
ENSG00000169641	9.667344577	9.843432726	protein_coding	1	23410516	23504301	LUZP1
ENSG00000169660	6.74439909	7.179727017	protein_coding	17	80376252	80400515	HEXDC
ENSG00000169668	3.393122761	2.579085888	pseudogene	22	21457305	21476574	BCRP2
ENSG00000169679	9.925798469	9.626176395	protein_coding	2	111395275	111435691	BUB1
ENSG00000169682	5.054730169	5.22435463	protein_coding	16	28985542	28995869	SPNS1
ENSG00000169683	7.285281023	7.41347308	protein_coding	17	79981280	79989024	LRRC45
ENSG00000169684	5.331395195	5.404622322	protein_coding	15	78857862	78887611	CHRNA5
ENSG00000169689	7.95748362	8.114504097	protein_coding	17	79976579	79980794	STRA13
ENSG00000169692	9.340351466	8.75700367	protein_coding	9	139567595	139581875	AGPAT2
ENSG00000169696	7.789781675	7.73528765	protein_coding	17	79935426	79975280	ASPSCR1
ENSG00000169704	0	0.390640832	protein_coding	3	128779610	128781249	GP9
ENSG00000169710	13.75253186	12.42700451	protein_coding	17	80036215	80056106	FASN
ENSG00000169714	11.18760954	11.2182309	protein_coding	3	128888327	128902765	CNBP
ENSG00000169715	7.337827682	8.414504719	protein_coding	16	56659387	56661024	MT1E
ENSG00000169718	9.72977077	9.666483771	protein_coding	17	80015748	80023680	DUS1L
ENSG00000169727	9.9196552	9.92322777	protein_coding	17	80009763	80015346	GPS1
ENSG00000169733	7.776245808	7.747865429	protein_coding	17	80005778	80009719	RFNG
ENSG00000169738	7.978913023	8.435390709	protein_coding	17	79993757	79995573	DCXR
ENSG00000169740	7.04004079	7.500283181	protein_coding	10	44139307	44144304	ZNF32
ENSG00000169750	7.206730419	6.281901708	protein_coding	17	79989532	79992077	RAC3
ENSG00000169752	4.230497448	4.489113623	protein_coding	15	76228310	76352136	NRG4
ENSG00000169756	8.869173944	8.461080043	protein_coding	2	109150857	109303702	LIMS1
ENSG00000169758	3.335220907	3.570966319	protein_coding	15	76352178	76521462	C15orf27
ENSG00000169762	6.793601635	7.222412001	protein_coding	4	16162128	16229033	TAPT1
ENSG00000169764	9.099664364	9.418958081	protein_coding	2	64068074	64118696	UGP2
ENSG00000169777	0	0.390640832	protein_coding	5	9629109	9630463	TAS2R1
ENSG00000169783	0	0.390640832	protein_coding	15	77905369	78113242	LINGO1
ENSG00000169813	11.64884675	11.22405678	protein_coding	10	43881065	43904633	HNRNPF
ENSG00000169814	7.007971391	7.434373903	protein_coding	3	15642848	15687329	BTD
ENSG00000169826	8.085926212	7.830998152	protein_coding	10	43633934	43680756	CSGALNACT2
ENSG00000169851	7.546773135	7.82111279	protein_coding	4	30722037	31144728	PCDH7
ENSG00000169855	9.578892348	9.938396195	protein_coding	3	78646390	79816965	ROBO1
ENSG00000169856	2.471521042	3.935919592	protein_coding	15	53049186	53083273	ONECUT1
ENSG00000169857	6.835971536	6.881748114	protein_coding	15	34158428	34331377	AVEN
ENSG00000169862	0.499066092	0	protein_coding	5	10971952	11904155	CTNND2
ENSG00000169871	8.455589162	8.177844832	protein_coding	7	100728720	100735017	TRIM56

ENSG00000169876	0.499066092	0	protein_coding	7	100663353	100702020	MUC17
ENSG00000169884	5.191549282	5.545764946	protein_coding	12	49359123	49365546	WNT10B
ENSG00000169885	2.762599152	1.802319292	protein_coding	1	1846266	1848735	CALML6
ENSG00000169891	7.819120959	7.423961341	protein_coding	X	16964814	17171395	REPS2
ENSG00000169894	4.568725997	2.420525079	protein_coding	7	100547187	100611118	MUC3A
ENSG00000169895	9.621905585	9.69599206	protein_coding	X	16737755	16783459	SYAP1
ENSG00000169896	4.800350936	5.629711952	protein_coding	16	31271311	31344190	ITGAM
ENSG00000169902	8.775675113	8.317991549	protein_coding	7	65670186	65885530	TPST1
ENSG00000169903	0.499066092	0	protein_coding	3	149191761	149221068	TM4SF4
ENSG00000169905	9.955528053	9.822279296	protein_coding	1	179809102	179846934	TOR1AIP2
ENSG00000169908	10.11833477	9.162099548	protein_coding	3	149086809	149095652	TM4SF1
ENSG00000169914	7.553136766	7.614575135	protein_coding	1	20209006	20239438	OTUD3
ENSG00000169918	3.146112541	2.721932731	protein_coding	15	31775329	32162992	OTUD7A
ENSG00000169919	9.05552222	9.045526555	protein_coding	7	65425671	65447301	GUSB
ENSG00000169925	10.15383543	9.901345166	protein_coding	9	136894789	136933657	BRD3
ENSG00000169926	9.724141885	9.147142808	protein_coding	15	31619058	31727868	KLF13
ENSG00000169933	0.499066092	0	protein_coding	X	12156585	12742642	FRMPD4
ENSG00000169946	3.335220907	3.183544561	protein_coding	8	106330920	106816760	ZFPM2
ENSG00000169951	6.204385613	6.371325053	protein_coding	16	30565085	30569819	ZNF764
ENSG00000169955	5.986594255	5.871116342	protein_coding	16	30537244	30546668	ZNF747
ENSG00000169957	8.596266901	8.663840043	protein_coding	16	30535325	30538142	ZNF768
ENSG00000169962	3.830412367	3.453019579	protein_coding	1	1266694	1270686	TAS1R3
ENSG00000169964	6.604346021	6.624193585	protein_coding	3	44903361	44907162	TMEM42
ENSG00000169967	8.797280438	8.695567763	protein_coding	2	128056306	128146041	MAP3K2
ENSG00000169972	7.517784241	7.405556541	protein_coding	1	1243947	1247057	PUSL1
ENSG00000169976	9.157191483	9.695451059	protein_coding	6	144416018	144416754	SF3B5
ENSG00000169981	5.563445526	5.536128679	protein_coding	3	44690219	44702283	ZNF35
ENSG00000169989	1.960915222	2.242360793	protein_coding	4	153690506	153700916	TIGD4
ENSG00000169991	9.457778871	8.921478076	protein_coding	1	19230774	19283180	IFFO2
ENSG00000169992	10.78980292	11.27853085	protein_coding	17	7311502	7323179	NLGN2
ENSG00000169994	1.960915222	2.579085888	protein_coding	2	128293378	128395304	MYO7B
ENSG00000170004	11.8544452	12.37592509	protein_coding	17	7788124	7816078	CHD3
ENSG00000170006	7.337827682	5.004391715	protein_coding	4	153539784	153601317	TMEM154
ENSG00000170011	0	0.697730409	protein_coding	3	39850405	40301812	MYRIP
ENSG00000170017	10.41587333	10.69674488	protein_coding	3	105085753	105295744	ALCAM
ENSG00000170027	12.09136192	11.24565047	protein_coding	7	75956116	75988348	YWHAG
ENSG00000170035	9.102921211	8.426290271	protein_coding	2	181831975	181941312	UBE2E3
ENSG00000170037	10.36025702	10.5344096	protein_coding	17	7835473	7852896	CNTROB
ENSG00000170043	10.38007941	10.94200031	protein_coding	17	7833663	7835317	TRAPPC1
ENSG00000170044	0.869158192	0	protein_coding	3	101818088	102198685	ZPLD1
ENSG00000170049	5.81716234	6.535085617	protein_coding	17	7825177	7832753	KCNAB3
ENSG00000170075	3.274897671	4.127774132	protein_coding	1	202091972	202098640	GPR37L1
ENSG00000170085	7.734859118	7.522414019	protein_coding	5	175665365	175772994	C5orf25
ENSG00000170088	8.131289597	8.096586553	protein_coding	4	165997256	166129701	TMEM192
ENSG00000170089	5.538004898	5.901455548	pseudogene	5	177236408	177377733	
ENSG00000170091	0.499066092	0.390640832	protein_coding	5	173472607	173670504	
ENSG00000170092	2.928422289	2.851901313	pseudogene	7	75123401	75133628	
ENSG00000170099	1.16343121	1.667587519	protein_coding	14	94770585	94789731	SERPINA6
ENSG00000170100	5.774228067	5.709041716	protein_coding	16	89284118	89295363	ZNF778
ENSG00000170113	8.879339004	8.696649474	protein_coding	15	23043277	23100005	NIPA1
ENSG00000170122	0.499066092	0.390640832	protein_coding	9	116237	118417	FOXD4
ENSG00000170128	0	0.697730409	protein_coding	1	200842083	200843306	GPR25
ENSG00000170142	9.169669647	9.425497321	protein_coding	3	23847394	23932807	UBE2E1
ENSG00000170144	10.42370836	10.04670806	protein_coding	2	178077291	178086111	HNRNPA3
ENSG00000170145	9.244321347	9.478658596	protein_coding	11	111473115	111601577	SIK2
ENSG00000170160	5.83815965	6.775188634	protein_coding	17	16592851	16707767	CCDC144A
ENSG00000170161	3.077135474	3.081239798	protein_coding	9	66553273	66555928	
ENSG00000170166	5.718698586	5.317302248	protein_coding	2	177015950	177020802	HOXD4
ENSG00000170175	7.603059879	8.395978102	protein_coding	17	7348380	7361026	CHRNB1
ENSG00000170185	8.156585168	8.194786822	protein_coding	4	144106070	144144983	USP38
ENSG00000170190	8.207901394	7.683850422	protein_coding	17	73084055	73102246	SLC16A5
ENSG00000170191	8.241782151	8.292461872	protein_coding	20	25593571	25604811	NANP
ENSG00000170209	3.335220907	4.021284656	protein_coding	11	113258513	113271140	ANKK1

ENSG00000170214	3.448790144	3.232099092	protein_coding	5	159343790	159399551	ADRA1B
ENSG00000170222	5.458875168	6.708116507	protein_coding	17	10600931	10614550	C17orf48
ENSG00000170231	6.379315619	7.924450389	protein_coding	5	159614374	159665742	FABP6
ENSG00000170234	7.356139979	7.457530976	protein_coding	5	159488808	159546430	PWWP2A
ENSG00000170236	0	0.390640832	protein_coding	15	50792759	50838905	USP50
ENSG00000170242	9.905425612	10.14863928	protein_coding	11	11862970	11980870	USP47
ENSG00000170248	10.18285176	10.276401	protein_coding	3	33839844	33911194	PDCD6IP
ENSG00000170260	7.618313091	7.106738413	protein_coding	7	148936742	148952700	ZNF212
ENSG00000170262	0	1.16600992	protein_coding	21	33664124	33687095	MRAP
ENSG00000170264	6.400645076	6.708116507	protein_coding	2	62051989	62081278	FAM161A
ENSG00000170265	9.394512382	9.02758571	protein_coding	7	148892554	148923339	ZNF282
ENSG00000170266	8.693086466	9.266315144	protein_coding	3	33038112	33138722	GLB1
ENSG00000170270	6.103936836	6.077857763	protein_coding	14	93669239	93673439	C14orf142
ENSG00000170271	6.188122351	6.051043053	protein_coding	5	154198051	154238812	C5orf4
ENSG00000170275	10.52024797	10.82272135	protein_coding	3	33155471	33185153	CRTAP
ENSG00000170289	0.499066092	0	protein_coding	8	87566205	87755903	CNGB3
ENSG00000170290	0.499066092	0	protein_coding	11	107578104	107590419	SLN
ENSG00000170291	9.058880138	9.932898838	protein_coding	17	7155372	7163250	C17orf81
ENSG00000170293	5.695874189	4.794098771	protein_coding	3	32280171	32411817	CMTM8
ENSG00000170296	10.73767751	11.48075629	protein_coding	17	7143333	7145772	GABARAP
ENSG00000170310	7.402691859	8.315177126	protein_coding	17	9153789	9479275	STX8
ENSG00000170312	9.619633528	9.566483319	protein_coding	10	62538089	62554610	CDK1
ENSG00000170315	12.17239307	13.08027416	protein_coding	17	16284113	16286054	UBB
ENSG00000170322	9.007671769	9.093948849	protein_coding	11	129733671	129765485	NFRKB
ENSG00000170325	7.481543613	7.793067905	protein_coding	11	129769601	129872730	PRDM10
ENSG00000170340	7.186405829	7.170413581	protein_coding	2	62423248	62451866	B3GNT2
ENSG00000170345	10.56816361	4.761389014	protein_coding	14	75745477	75748933	FOS
ENSG00000170348	11.46280289	11.66984103	protein_coding	14	75598173	75643334	TMED10
ENSG00000170364	6.372135143	6.539915281	protein_coding	3	4344988	4359251	SETMAR
ENSG00000170365	7.136425764	7.292770473	protein_coding	4	146402346	146479231	SMAD1
ENSG00000170374	0.869158192	0.390640832	protein_coding	12	53720362	53739099	SP7
ENSG00000170379	5.158541613	5.486957566	protein_coding	7	143318043	143427502	FAM115C
ENSG00000170381	4.409500985	2.144285137	protein_coding	7	82993222	83278479	SEMA3E
ENSG00000170382	1.407729925	1.518964905	protein_coding	1	204586298	204654861	LRRN2
ENSG00000170385	8.485841807	8.141764426	protein_coding	1	211744910	211752084	SLC30A1
ENSG00000170390	4.863159938	5.611474642	protein_coding	4	150999426	151178609	DCLK2
ENSG00000170412	6.766474304	6.387491945	protein_coding	17	72427052	72446719	GPRC5C
ENSG00000170417	6.228441838	5.893930351	protein_coding	2	103353367	103460352	TMEM182
ENSG00000170421	12.69315009	12.63434894	protein_coding	12	53290971	53343738	KRT8
ENSG00000170423	0.869158192	0	protein_coding	12	53231588	53242876	KRT78
ENSG00000170425	8.571403474	8.75700367	protein_coding	17	15848231	15879060	ADORA2B
ENSG00000170426	0.499066092	1.518964905	protein_coding	12	57316938	57328189	SDR9C7
ENSG00000170430	4.903563137	6.544728831	protein_coding	10	131265448	131566271	MGMT
ENSG00000170439	4.381167248	3.679994897	protein_coding	12	56075330	56078395	METTL7B
ENSG00000170442	3.911897206	3.081239798	protein_coding	12	52643084	52702947	
ENSG00000170445	9.662202373	9.740202333	protein_coding	5	140052758	140071609	HARS
ENSG00000170448	7.599989788	7.632773539	protein_coding	4	47849257	47916653	NFXL1
ENSG00000170454	1.799000381	1.16600992	protein_coding	12	52817854	52828309	KRT75
ENSG00000170456	7.50471092	7.560938532	protein_coding	12	31535157	31744031	DENND5B
ENSG00000170458	6.095241663	5.683080141	protein_coding	5	140011313	140013286	CD14
ENSG00000170464	5.60078458	5.506827567	protein_coding	5	138744279	138780180	DNAJC18
ENSG00000170469	5.472368541	5.137803333	protein_coding	5	138732252	138739777	SPATA24
ENSG00000170471	10.54040657	10.67440754	protein_coding	20	37101459	37207504	RALGAPB
ENSG00000170473	8.109865782	8.329194658	protein_coding	12	56295197	56326402	WIBG
ENSG00000170476	0.499066092	0.697730409	protein_coding	5	138723169	138725770	
ENSG00000170482	2.762599152	2.242360793	protein_coding	5	138702885	138720242	SLC23A1
ENSG00000170485	8.090308512	7.260948155	protein_coding	2	101436614	101613291	NPAS2
ENSG00000170498	0.499066092	0	protein_coding	1	204159469	204165614	KISS1
ENSG00000170500	3.448790144	1.518964905	protein_coding	2	100889753	100939195	LONRF2
ENSG00000170502	7.757079669	8.226588358	protein_coding	4	88343734	88380606	NUDT9
ENSG00000170509	1.16343121	0.697730409	protein_coding	4	88224941	88244058	HSD17B13
ENSG00000170515	10.85917474	11.01297359	protein_coding	12	56498103	56509935	PA2G4
ENSG00000170522	9.936182363	9.859458974	protein_coding	4	110967002	111120355	ELOVL6

ENSG00000170523	0.869158192	0	protein_coding	12	52708085	52715182	KRT83
ENSG00000170525	10.13963411	9.607318979	protein_coding	10	6186881	6277495	PFKFB3
ENSG00000170537	7.097770581	6.057793599	protein_coding	16	18995256	19075264	TMC7
ENSG00000170540	10.71621744	10.64192502	protein_coding	16	18802991	18813000	ARL6IP1
ENSG00000170542	8.025424129	5.871116342	protein_coding	6	2887500	2903514	SERPINB9
ENSG00000170545	7.870982917	7.577474828	protein_coding	12	51639133	51664202	SMAGP
ENSG00000170558	0.499066092	1.353254395	protein_coding	18	25530930	25757410	CDH2
ENSG00000170561	2.238690726	0	protein_coding	5	2745959	2752969	IRX2
ENSG00000170571	7.627388205	7.760334499	protein_coding	5	49692026	49739082	EMB
ENSG00000170577	5.124761037	5.383282767	protein_coding	2	45232300	45236569	SIX2
ENSG00000170579	1.407729925	0.950786998	protein_coding	18	3496030	4151290	DLGAP1
ENSG00000170581	9.100750797	10.22821739	protein_coding	12	56735381	56753939	STAT2
ENSG00000170584	8.018542337	8.359562039	protein_coding	5	162880494	162887146	NUDCD2
ENSG00000170604	8.387780826	8.374509343	protein_coding	19	46386866	46389376	IRF2BP1
ENSG00000170606	11.16597419	10.94723595	protein_coding	5	132387654	132442141	HSPA4
ENSG00000170608	2.238690726	2.579085888	protein_coding	19	46367518	46377054	FOXA3
ENSG00000170615	1.16343121	1.16600992	protein_coding	7	102993177	103086624	SLC26A5
ENSG00000170616	0.869158192	0.390640832	protein_coding	8	145556724	145559943	SCRT1
ENSG00000170619	6.846372398	7.329495501	protein_coding	8	146066427	146079121	COMMD5
ENSG00000170629	3.911897206	3.411459265	pseudogene	7	102815580	102920857	DPY19L2P2
ENSG00000170631	5.649112675	5.988823159	protein_coding	8	146155744	146176274	ZNF16
ENSG00000170632	8.211928911	7.605389119	protein_coding	7	102715328	102740205	ARMC10
ENSG00000170633	7.853042529	8.386624806	protein_coding	12	121837844	121868389	RNF34
ENSG00000170634	4.642199401	5.271576845	protein_coding	2	54342540	54532437	ACYP2
ENSG00000170638	10.35115602	10.44132471	protein_coding	22	50624344	50638027	TRABD
ENSG00000170647	7.292905885	7.56568256	protein_coding	11	100862811	100864663	TMEM133
ENSG00000170653	8.49908645	8.478794222	protein_coding	12	53901640	54020199	ATF7
ENSG00000170667	1.960915222	1.925536307	protein_coding	7	102122892	102184211	RASA4B
ENSG00000170677	7.832262274	7.69255166	protein_coding	18	67956168	67997436	SOCS6
ENSG00000170681	4.842525946	5.150490275	protein_coding	9	103340361	103350188	MURC
ENSG00000170683	0.499066092	0	protein_coding	11	7960081	7961141	OR10A3
ENSG00000170684	6.283060568	5.840125386	protein_coding	19	45574759	45579845	ZNF296
ENSG00000170688	0	0.390640832	pseudogene	11	7870247	7871182	OR5E1P
ENSG00000170689	4.77879189	3.935919592	protein_coding	17	46698518	46703839	HOXB9
ENSG00000170727	8.221948786	7.793067905	protein_coding	8	145486055	145515082	BOP1
ENSG00000170734	7.615275323	8.149685112	protein_coding	6	43543887	43586701	POLH
ENSG00000170745	7.695130106	6.839280559	protein_coding	2	18059114	18542882	KCNS3
ENSG00000170759	10.92137194	11.95072919	protein_coding	10	32297938	32345359	KIF5B
ENSG00000170775	3.554067925	1.802319292	protein_coding	7	124386051	124405681	GPR37
ENSG00000170776	9.590540077	9.625608565	protein_coding	15	85777817	86292586	AKAP13
ENSG00000170779	9.559268022	8.806942451	protein_coding	14	105475910	105487485	CDCA4
ENSG00000170786	3.146112541	0	protein_coding	8	57212569	57233335	SDR16C5
ENSG00000170788	0.499066092	0	protein_coding	10	82095861	82116511	DYDC1
ENSG00000170791	7.717966186	7.902117654	protein_coding	8	57124245	57131357	CHCHD7
ENSG00000170801	1.16343121	0	protein_coding	4	8271492	8308838	HTRA3
ENSG00000170802	8.322227223	8.120964787	protein_coding	2	48541776	48606434	FOXN2
ENSG00000170807	0.499066092	0	protein_coding	7	123295861	123304344	LMOD2
ENSG00000170832	9.690621167	9.720661305	protein_coding	17	58254691	58469586	USP32
ENSG00000170835	4.903563137	4.508954154	protein_coding	9	135937365	135947248	CEL
ENSG00000170836	7.281453427	7.716212954	protein_coding	17	58677554	58742036	PPM1D
ENSG00000170846	7.759833327	7.630511253	processed_transc	4	6675178	6677774	
ENSG00000170848	2.35979773	2.971122874	protein_coding	19	43406231	43422162	PSG6
ENSG00000170852	8.990190269	8.868709593	protein_coding	7	32907784	32933743	KBTBD2
ENSG00000170854	8.782461599	8.657187659	protein_coding	3	97660661	97691301	MINA
ENSG00000170855	7.808521034	7.73528765	protein_coding	12	120881764	120884215	TRIAP1
ENSG00000170860	8.337054268	8.700968226	protein_coding	3	14219858	14242619	LSM3
ENSG00000170866	0.499066092	0.390640832	protein_coding	19	54799854	54809952	LILRA3
ENSG00000170871	7.990682129	8.295320915	protein_coding	4	6783102	6885897	KIAA0232
ENSG00000170873	3.274897671	3.411459265	protein_coding	8	125563031	125740730	MTSS1
ENSG00000170876	10.58766812	10.80050531	protein_coding	3	14166440	14185179	TMEM43
ENSG00000170881	7.990682129	7.922602475	protein_coding	8	125486979	125500155	RNF139
ENSG00000170889	11.94675509	12.24987398	protein_coding	19	54704610	54752862	RPS9
ENSG00000170890	0	0.697730409	protein_coding	12	120759914	120765592	PLA2G1B

ENSG00000170892	9.6309582	9.922765621	protein_coding	19	54693789	54697585	TSEN34
ENSG00000170899	5.036678094	5.923798455	protein_coding	6	52842751	52860176	GSTA4
ENSG00000170903	7.627388205	7.911465009	protein_coding	11	105866350	105893130	KIAA1826
ENSG00000170906	8.353554553	8.762179391	protein_coding	19	54606036	54614898	NDUFA3
ENSG00000170909	2.471521042	2.912743273	protein_coding	19	54597933	54606000	OSCAR
ENSG00000170915	5.869093502	6.044260771	protein_coding	6	52226219	52272575	PAQR8
ENSG00000170917	5.718698586	6.16171561	protein_coding	4	123813730	123844123	NUDT6
ENSG00000170919	5.40360309	4.976162367	processed_transc	13	45915480	45965872	
ENSG00000170921	9.622662143	9.539005037	protein_coding	17	61086898	61505067	TANC2
ENSG00000170927	0.869158192	0	protein_coding	6	51480098	51952423	PKHD1
ENSG00000170946	6.749949642	6.963107483	protein_coding	11	31391387	31453396	DNAJC24
ENSG00000170949	8.894453552	8.984835176	protein_coding	19	53569867	53606687	ZNF160
ENSG00000170954	7.119373418	6.89311646	protein_coding	19	53611133	53636171	ZNF415
ENSG00000170955	8.779750834	9.486810444	protein_coding	11	6340176	6341877	PRKCDBP
ENSG00000170961	5.498982484	5.792353798	protein_coding	8	122624356	122653680	HAS2
ENSG00000170962	2.928422289	3.32456471	protein_coding	11	103777914	104035107	PDGFD
ENSG00000170965	6.335686705	7.053694091	protein_coding	X	133699868	133898352	PLAC1
ENSG00000170989	1.616589159	1.16600992	protein_coding	1	101702444	101707074	S1PR1
ENSG00000171004	9.219530792	8.925174415	protein_coding	X	131760038	132095423	HS6ST2
ENSG00000171016	6.041940478	6.716673044	protein_coding	15	55831088	55881145	PYGO1
ENSG00000171017	4.800350936	4.508954154	protein_coding	19	7953390	7966906	LRRC8E
ENSG00000171044	5.054730169	5.564846512	protein_coding	8	10753555	11058875	XKR6
ENSG00000171045	6.994006093	7.278392738	protein_coding	8	143293441	143484601	TSNARE1
ENSG00000171049	0.499066092	0.697730409	protein_coding	19	52264183	52273760	FPR2
ENSG00000171051	2.471521042	2.501982735	protein_coding	19	52249027	52255150	FPR1
ENSG00000171053	0.499066092	1.353254395	protein_coding	11	125616188	125619743	PATE1
ENSG00000171054	0.499066092	1.518964905	protein_coding	X	130677950	130679030	OR13H1
ENSG00000171055	8.643253539	8.801925129	protein_coding	2	36778570	36873230	FEZ2
ENSG00000171056	1.960915222	1.802319292	protein_coding	8	10581278	10697357	SOX7
ENSG00000171060	0.499066092	0	protein_coding	8	10530147	10558103	C8orf74
ENSG00000171067	10.24853313	10.67193469	protein_coding	11	68028803	68039469	C11orf24
ENSG00000171084	5.124761037	4.42790041	pseudogene	3	125635463	125648867	
ENSG00000171094	2.35979773	2.788380093	protein_coding	2	29415640	30144432	ALK
ENSG00000171097	6.469548347	6.525377513	protein_coding	9	131595221	131644773	CCBL1
ENSG00000171100	7.474855471	7.668495823	protein_coding	X	149737069	149841795	MTM1
ENSG00000171103	7.093410923	7.332282157	protein_coding	2	29072687	29093167	TRMT61B
ENSG00000171105	9.220530637	9.57591961	protein_coding	19	7112266	7294011	INSR
ENSG00000171109	9.110492049	8.984835176	protein_coding	3	179065480	179112719	MFN1
ENSG00000171115	4.437288965	4.65855978	protein_coding	7	150147718	150176480	GIMAP8
ENSG00000171119	5.072559152	5.125003832	protein_coding	19	5823818	5828334	NRTN
ENSG00000171121	4.131913373	3.570966319	protein_coding	3	178957530	178984790	KCNMB3
ENSG00000171124	5.175139844	1.518964905	protein_coding	19	5842900	5851485	FUT3
ENSG00000171126	4.437288965	4.744751758	protein_coding	2	42669157	42721237	KCNG3
ENSG00000171129	1.407729925	0.390640832	protein_coding	X	148674172	148676974	HSFX2
ENSG00000171130	8.360827871	8.418443943	protein_coding	7	149570057	149577787	ATP6V0E2
ENSG00000171132	6.902274257	6.450406542	protein_coding	2	45878484	46415129	PRKCE
ENSG00000171133	0	0.390640832	protein_coding	9	114089763	114090800	OR2K2
ENSG00000171135	8.364450825	8.395978102	protein_coding	3	9932238	9936033	JAGN1
ENSG00000171136	0.499066092	0.390640832	protein_coding	19	14138960	14141854	RLN3
ENSG00000171148	9.734678093	9.824260193	protein_coding	3	9821544	9834695	TADA3
ENSG00000171150	8.103376106	7.733180651	protein_coding	2	46926091	46990268	SOCS5
ENSG00000171155	8.131289597	8.563056668	protein_coding	X	119759648	119764005	C1GALT1C1
ENSG00000171159	9.13084342	9.05820621	protein_coding	9	130922539	130926207	C9orf16
ENSG00000171160	6.727618145	6.827478203	protein_coding	10	99374310	99393852	MORN4
ENSG00000171161	8.42468852	8.784734524	protein_coding	1	249132409	249143716	ZNF672
ENSG00000171163	8.653654925	9.214393111	protein_coding	1	249144205	249153343	ZNF692
ENSG00000171169	6.598204782	6.896886088	protein_coding	9	130823512	130830485	NAIF1
ENSG00000171174	5.774228067	5.674321415	protein_coding	2	28004231	28113965	RBKS
ENSG00000171189	1.407729925	1.925536307	protein_coding	21	30909254	31312351	GRIK1
ENSG00000171195	0	0.950786998	protein_coding	4	71296209	71348714	MUC7
ENSG00000171202	6.956090577	7.515074729	protein_coding	11	85359011	85367591	TMEM126A
ENSG00000171204	8.450484798	9.025004422	protein_coding	11	85339629	85347580	TMEM126B
ENSG00000171206	10.86781572	10.95855266	protein_coding	10	104404252	104418164	TRIM8

ENSG00000171208	9.72977077	10.26696498	protein_coding	16	47111614	47177908	NETO2
ENSG00000171209	0	0.697730409	protein_coding	4	71108305	71117145	CSN3
ENSG00000171217	2.847891871	3.183544561	protein_coding	6	155585147	155597682	CLDN20
ENSG00000171219	7.46813618	4.021284656	protein_coding	11	64590859	64612041	CDC42BPG
ENSG00000171222	9.097489042	9.883881858	protein_coding	20	34541539	34547394	SCAND1
ENSG00000171223	12.54038067	11.93397166	protein_coding	19	12902310	12904124	JUNB
ENSG00000171224	6.252103507	5.953060722	protein_coding	10	71390007	71393352	C10orf35
ENSG00000171227	4.131913373	3.027231696	protein_coding	2	120187477	120196096	TMEM37
ENSG00000171236	7.501423995	6.25865215	protein_coding	19	4537227	4540048	LRG1
ENSG00000171241	8.964161368	9.28369534	protein_coding	16	46614466	46655538	SHCBP1
ENSG00000171246	0.499066092	0	protein_coding	17	78440633	78450404	NPTX1
ENSG00000171262	8.98077946	8.866788836	protein_coding	15	38746328	38779911	FAM98B
ENSG00000171282	9.256071182	8.635917101	protein_coding	17	79373540	79433357	BAHCC1
ENSG00000171291	7.102117104	7.664078611	protein_coding	19	11959576	11980306	ZNF439
ENSG00000171295	8.576098123	8.427593853	protein_coding	19	11925099	11946016	ZNF440
ENSG00000171298	10.26945679	10.73778314	protein_coding	17	78075355	78093678	GAA
ENSG00000171302	9.92763636	9.804829433	protein_coding	17	76987799	77005899	CANT1
ENSG00000171303	3.077135474	2.971122874	protein_coding	2	26915558	26956288	KCNK3
ENSG00000171307	9.225519491	9.647589989	protein_coding	10	99205927	99217127	ZDHHC16
ENSG00000171310	9.346776019	8.280968702	protein_coding	12	104849073	105155792	CHST11
ENSG00000171311	7.68357507	8.119352325	protein_coding	10	99190928	99205774	EXOSC1
ENSG00000171314	11.47688747	11.9266146	protein_coding	10	99185917	99193198	PGAM1
ENSG00000171316	8.960575307	8.565427152	protein_coding	8	61591337	61779465	CHD7
ENSG00000171320	7.501423995	7.628245414	protein_coding	8	27629466	27670157	ESCO2
ENSG00000171345	12.29622838	8.868709593	protein_coding	17	39679869	39684560	KRT19
ENSG00000171346	7.030950614	5.709041716	protein_coding	17	39669995	39678781	KRT15
ENSG00000171357	1.407729925	0.697730409	protein_coding	1	46669006	46686933	C1orf190
ENSG00000171365	8.011627561	8.541545191	protein_coding	X	49687225	49863892	CLCN5
ENSG00000171368	5.550781289	4.94736961	protein_coding	5	660883	693510	TPPP
ENSG00000171385	0.499066092	0	protein_coding	1	112313284	112531777	KCND3
ENSG00000171388	6.830742851	7.681666888	protein_coding	X	128779240	128788933	APLN
ENSG00000171401	0	0.390640832	protein_coding	17	39657233	39661849	KRT13
ENSG00000171403	0.499066092	0.697730409	protein_coding	17	39722096	39728310	KRT9
ENSG00000171405	0.499066092	0	protein_coding	X	52841228	52847325	XAGE5
ENSG00000171408	1.616589159	3.279072565	protein_coding	6	136172834	136516712	PDE7B
ENSG00000171421	8.241782151	8.468698482	protein_coding	5	1798500	1801480	MRPL36
ENSG00000171425	7.627388205	7.971683681	protein_coding	19	56154986	56156988	ZNF581
ENSG00000171428	5.036678094	5.466810074	protein_coding	8	18027986	18081198	NAT1
ENSG00000171431	0	0.390640832	protein_coding	17	39030852	39041479	KRT20
ENSG00000171433	0	0.390640832	protein_coding	X	48620154	48632064	GLOD5
ENSG00000171443	6.496213887	6.539915281	protein_coding	19	56111730	56114504	ZNF524
ENSG00000171444	8.337054268	5.840125386	protein_coding	5	112357796	112824527	MCC
ENSG00000171448	6.35766608	6.980975423	protein_coding	9	125677845	125693779	ZBTB26
ENSG00000171450	5.018397273	4.585695337	protein_coding	2	219824377	219826876	CDK5R2
ENSG00000171451	7.461385447	8.270836518	protein_coding	18	65173819	65184217	DSEL
ENSG00000171453	8.790563466	8.954409067	protein_coding	6	43477440	43497323	POLR1C
ENSG00000171456	10.82966424	10.96664607	protein_coding	20	30946147	31027122	ASXL1
ENSG00000171462	4.75690578	4.342020395	protein_coding	6	43418090	43424370	DLK2
ENSG00000171466	9.936790859	9.997084738	protein_coding	19	9759363	9785776	ZNF562
ENSG00000171467	9.000704568	9.515291124	protein_coding	6	43299513	43337181	ZNF318
ENSG00000171469	9.053279257	9.304420832	protein_coding	19	9715356	9732075	ZNF561
ENSG00000171471	4.689181911	4.448594745	protein_coding	12	116997186	117014425	MAP1LC3B2
ENSG00000171475	9.809013881	9.029304007	protein_coding	17	38375574	38440388	WIPF2
ENSG00000171483	0	0.697730409	protein_coding	X	47967365	47980073	SSX6
ENSG00000171488	6.188122351	1.802319292	protein_coding	1	90098631	90398809	LRRC8C
ENSG00000171490	11.114462	10.92225346	protein_coding	16	11931196	11945442	RSL1D1
ENSG00000171492	9.340351466	8.96970233	protein_coding	1	90286573	90402170	LRRC8D
ENSG00000171496	0.499066092	0.390640832	protein_coding	9	125329778	125330838	OR1L8
ENSG00000171497	8.025424129	8.643790155	protein_coding	4	159630286	159644548	PPID
ENSG00000171502	0	0.697730409	protein_coding	1	86194916	86622626	COL24A1
ENSG00000171503	7.266040677	7.675096386	protein_coding	4	159593277	159630775	ETFDH
ENSG00000171522	7.35977471	6.984522608	protein_coding	5	40679600	40693837	PTGER4
ENSG00000171530	8.944931872	9.242070261	protein_coding	5	76986991	77164604	TBCA

ENSG00000171532	0	0.390640832	protein_coding	17	37760021	37764196	NEUROD2
ENSG00000171533	4.322773689	5.200150955	protein_coding	11	75297963	75380165	MAP6
ENSG00000171551	0	0.697730409	protein_coding	2	233344537	233352538	ECEL1
ENSG00000171552	10.50555736	10.34077957	protein_coding	20	30252255	30311792	BCL2L1
ENSG00000171561	0	0.390640832	protein_coding	11	74799758	74800799	OR2AT4
ENSG00000171566	9.713528165	10.49422748	protein_coding	4	155456158	155471587	PLRG1
ENSG00000171570	7.853042529	7.614575135	protein_coding	19	41305048	41314336	EGLN2
ENSG00000171574	7.853042529	7.795089291	protein_coding	19	58920063	58956870	ZNF584
ENSG00000171587	0.499066092	0	protein_coding	21	41382926	42219065	DSCAM
ENSG00000171595	0	0.390640832	protein_coding	17	72270386	72311022	DNAI2
ENSG00000171596	0	0.390640832	protein_coding	2	232387871	232395206	NMUR1
ENSG00000171603	11.1589505	11.01470937	protein_coding	1	9789084	9884584	CLSTN1
ENSG00000171604	8.839544001	8.998057128	protein_coding	5	139026884	139063467	CXXC5
ENSG00000171606	8.930339658	9.512836742	protein_coding	19	58694396	58724927	ZNF274
ENSG00000171608	8.148202495	8.208501977	protein_coding	1	9711790	9789172	PIK3CD
ENSG00000171611	0.499066092	0	protein_coding	6	42883727	42893898	PTCRA
ENSG00000171612	5.498982484	5.953060722	protein_coding	1	9599541	9642831	SLC25A33
ENSG00000171617	9.809678436	9.442361917	protein_coding	5	73923234	73937249	ENC1
ENSG00000171621	8.294008989	7.9428005	protein_coding	1	9352939	9429591	SPSB1
ENSG00000171631	7.565780433	6.729413443	protein_coding	11	72975550	73009662	P2RY6
ENSG00000171634	9.614318105	9.686767343	protein_coding	17	65821637	66033571	BPTF
ENSG00000171649	8.036821332	7.644032061	protein_coding	19	58095508	58105253	ZIK1
ENSG00000171657	0.869158192	0.697730409	protein_coding	X	41583408	41589388	GPR82
ENSG00000171658	1.960915222	0.390640832	pseudogene	3	185677732	185698658	
ENSG00000171659	1.16343121	1.16600992	protein_coding	X	41548226	41556526	GPR34
ENSG00000171680	8.000028772	7.832967122	protein_coding	1	6526152	6580121	PLEKHG5
ENSG00000171681	10.45037457	10.02448085	protein_coding	12	14518610	14651697	ATF7IP
ENSG00000171695	0.499066092	0	protein_coding	20	62714733	62715712	C20orf201
ENSG00000171700	7.68357507	7.263870273	protein_coding	20	62704534	62711323	RGS19
ENSG00000171703	8.985492538	9.065760854	protein_coding	20	62681189	62703700	TCEA2
ENSG00000171720	9.47296155	9.138412238	protein_coding	5	141000443	141016437	HDAC3
ENSG00000171722	0	0.390640832	protein_coding	1	162343515	162346660	C1orf111
ENSG00000171723	9.206469362	9.479287299	protein_coding	14	66974125	67648520	GPHN
ENSG00000171729	7.416718478	7.856387513	protein_coding	1	15479028	15546974	TMEM51
ENSG00000171735	7.621344476	7.632773539	protein_coding	1	6845384	7827916	CAMTA1
ENSG00000171747	0	0.390640832	protein_coding	19	39292312	39303740	LGALS4
ENSG00000171757	6.979904289	6.316088276	protein_coding	3	169511216	169530774	LRRC34
ENSG00000171763	7.308035767	7.365308824	protein_coding	15	45694529	45713617	SPATA5L1
ENSG00000171766	4.77879189	4.961837817	protein_coding	15	45653322	45694416	GATM
ENSG00000171773	0	0.697730409	protein_coding	19	17566236	17571725	NXNL1
ENSG00000171777	1.16343121	1.16600992	protein_coding	19	38899698	38916945	RASGRP4
ENSG00000171786	4.19837882	4.127774132	protein_coding	1	160336857	160342638	NHLH1
ENSG00000171790	5.512107582	5.486957566	protein_coding	1	41481269	41488909	SLFNL1
ENSG00000171791	6.35766608	6.563824016	protein_coding	18	60790579	60987361	BCL2
ENSG00000171792	8.225937332	8.141764426	protein_coding	12	2985424	2998626	C12orf32
ENSG00000171793	9.40334477	9.629578691	protein_coding	1	41445007	41478235	CTPS
ENSG00000171798	6.861834435	6.874119142	protein_coding	10	134973951	135039916	KNDC1
ENSG00000171804	3.077135474	3.133298822	protein_coding	19	38375463	38397317	WDR87
ENSG00000171806	5.431503808	5.393992	protein_coding	1	169761670	169764107	METTL18
ENSG00000171811	3.502389126	2.912743273	protein_coding	10	134621896	134756327	TTC40
ENSG00000171812	3.274897671	2.144285137	protein_coding	1	36560837	36590821	COL8A2
ENSG00000171813	8.199812472	7.73107057	protein_coding	10	134210672	134231367	PWWP2B
ENSG00000171817	3.004694206	3.493416095	protein_coding	19	38085731	38105000	ZNF540
ENSG00000171819	0.869158192	0.950786998	protein_coding	1	11249398	11256038	ANGPTL7
ENSG00000171823	7.285281023	6.926693689	protein_coding	12	1675159	1703331	FBXL14
ENSG00000171824	9.526494794	9.705158168	protein_coding	1	11126675	11159938	EXOSC10
ENSG00000171827	5.223818625	5.072639111	protein_coding	19	37959982	37976260	ZNF570
ENSG00000171840	4.618121645	3.608232228	protein_coding	12	673462	772945	NINJ2
ENSG00000171843	5.827699195	6.544728831	protein_coding	9	20341663	20622542	MLLT3
ENSG00000171847	2.35979773	1.353254395	protein_coding	12	8373856	8380214	FAM90A1
ENSG00000171848	10.35115602	10.05052055	protein_coding	2	10262455	10271545	RRM2
ENSG00000171853	8.937654214	8.450859136	protein_coding	2	3383446	3488865	TRAPPC12
ENSG00000171855	0	0.390640832	protein_coding	9	21077104	21077943	IFNB1

ENSG00000171858	11.35857322	12.10289984	protein_coding	20	60962105	60963576	RPS21
ENSG00000171860	2.471521042	1.925536307	protein_coding	12	8210898	8219067	C3AR1
ENSG00000171861	8.13977083	8.749726325	protein_coding	17	685513	695749	RNMTL1
ENSG00000171862	9.507778518	9.434602747	protein_coding	10	89622870	89731687	PTEN
ENSG00000171863	9.891054276	9.967366806	protein_coding	2	3622795	3628509	RPS7
ENSG00000171864	0	0.390640832	protein_coding	20	4702556	4709106	PRND
ENSG00000171865	8.303476525	7.705505903	protein_coding	2	3592383	3606206	RNASEH1
ENSG00000171867	11.7580624	11.91388133	protein_coding	20	4666882	4682236	PRNP
ENSG00000171872	5.389447744	6.293387477	protein_coding	1	44584522	44600812	KLF17
ENSG00000171873	1.16343121	0.950786998	protein_coding	20	4201329	4229721	ADRA1D
ENSG00000171877	7.416718478	7.096940025	protein_coding	15	44162962	44487450	FRMD5
ENSG00000171889	3.077135474	2.501982735	processed_transc	9	21455641	21559668	MIR31HG
ENSG00000171914	7.695130106	8.301022059	protein_coding	15	62682725	63136830	TLN2
ENSG00000171928	7.334137164	7.871792517	protein_coding	17	18684519	18710027	FAM18B1
ENSG00000171931	2.671945279	1.802319292	protein_coding	17	18647326	18682662	FBXW10
ENSG00000171940	10.06366867	9.665379195	protein_coding	20	52183604	52226446	ZNF217
ENSG00000171943	5.158541613	5.03207928	pseudogene	1	121107124	121129949	SRGAP2P1
ENSG00000171951	2.762599152	3.714580548	protein_coding	2	224461658	224467221	SCG2
ENSG00000171953	7.759833327	8.367734306	protein_coding	17	17918373	17942523	ATPAF2
ENSG00000171954	0.499066092	0	protein_coding	19	15636144	15663127	CYP4F22
ENSG00000171956	1.16343121	1.353254395	protein_coding	15	60296421	60353929	FOXB1
ENSG00000171960	7.444369108	7.913327235	protein_coding	1	43124096	43142429	PPIH
ENSG00000171962	5.967663407	6.408769788	protein_coding	17	17876127	17920203	LRRC48
ENSG00000171970	6.693459083	6.874119142	protein_coding	19	2900896	2918474	ZNF57
ENSG00000171984	5.223818625	5.593003837	protein_coding	20	5731039	5845020	C20orf196
ENSG00000171988	9.47296155	9.230166865	protein_coding	10	64926981	65225722	JMJD1C
ENSG00000171989	0.869158192	1.518964905	protein_coding	15	59499042	59500705	LDHAL6B
ENSG00000171992	10.23276269	9.589377052	protein_coding	5	149980642	150038782	SYNPO
ENSG00000172000	6.809636522	6.343971006	protein_coding	19	2867333	2878501	ZNF556
ENSG00000172005	1.616589159	0.390640832	protein_coding	2	95691422	95719737	MAL
ENSG00000172006	6.788216822	7.100213554	protein_coding	19	2819872	2836733	ZNF554
ENSG00000172007	6.393570237	6.673373662	protein_coding	4	140374386	140397763	RAB33B
ENSG00000172009	11.15346388	11.33918459	protein_coding	19	2785506	2813588	THOP1
ENSG00000172014	0.499066092	0	protein_coding	9	69381812	69425560	ANKRD20A4
ENSG00000172020	0	0.390640832	protein_coding	3	115342171	115440337	GAP43
ENSG00000172031	1.407729925	1.16600992	protein_coding	1	92495539	92529093	EPHX4
ENSG00000172037	10.7766072	11.55241425	protein_coding	3	49158547	49170551	LAMB2
ENSG00000172046	9.364975797	9.331831739	protein_coding	3	49145479	49158371	USP19
ENSG00000172053	10.80554674	10.81601773	protein_coding	3	49133365	49142553	QARS
ENSG00000172057	8.5900911	8.587754596	protein_coding	17	38077294	38083854	ORMDL3
ENSG00000172058	0.499066092	0.950786998	protein_coding	5	70196492	70214357	SERF1A
ENSG00000172059	8.49908645	7.907733332	protein_coding	2	10182976	10194963	KLF11
ENSG00000172061	3.448790144	3.368666104	protein_coding	3	194075976	194090472	LRRC15
ENSG00000172062	4.863159938	4.585695337	protein_coding	5	70220768	70249769	SMN1
ENSG00000172070	8.682966505	8.64266806	protein_coding	20	627259	656444	SRXN1
ENSG00000172071	8.227927477	8.714915547	protein_coding	2	88856259	88927094	EIF2AK3
ENSG00000172073	0.499066092	1.518964905	protein_coding	2	88824169	88829102	C2orf51
ENSG00000172081	10.42110141	10.43031225	protein_coding	19	2071037	2096269	MOB3A
ENSG00000172086	7.474855471	8.061749803	protein_coding	2	88326724	88355248	KRCC1
ENSG00000172113	6.856698814	6.703819132	protein_coding	3	48334754	48343175	NME6
ENSG00000172115	10.88147134	10.74381326	protein_coding	7	25159710	25164980	CYCS
ENSG00000172116	0.499066092	0	protein_coding	2	87042462	87089047	CD8B
ENSG00000172123	6.579622637	6.703819132	protein_coding	17	33738079	33760218	SLFN12
ENSG00000172137	9.540174158	10.29830006	protein_coding	16	71392616	71424341	CALB2
ENSG00000172139	1.16343121	2.579085888	protein_coding	3	111859734	112013105	SLC9A10
ENSG00000172159	3.911897206	4.021284656	protein_coding	9	85857905	86153461	FRMD3
ENSG00000172164	2.671945279	3.133298822	protein_coding	8	121547985	121825513	SNTB1
ENSG00000172167	6.766474304	7.022976646	protein_coding	8	121457640	121554373	MTBP
ENSG00000172171	6.364918751	6.682138126	protein_coding	17	29226001	29233286	TEFM
ENSG00000172172	7.850461307	8.279525596	protein_coding	8	121393000	121457642	MRPL13
ENSG00000172175	8.611591722	8.945336714	protein_coding	18	56338618	56421130	MALT1
ENSG00000172183	6.086493765	6.699508919	protein_coding	15	89179384	89199714	ISG20
ENSG00000172197	7.210761201	5.893930351	protein_coding	6	20100935	20212670	MBOAT1

ENSG00000172201	4.6658819	4.810179682	protein_coding	6	19837617	19840915	ID4
ENSG00000172209	0.499066092	1.802319292	protein_coding	7	107110463	107116098	GPR22
ENSG00000172215	2.10647801	1.925536307	protein_coding	3	45982425	45989845	CXCR6
ENSG00000172216	9.981711773	9.791728663	protein_coding	20	48807376	48809212	CEBPB
ENSG00000172232	0.869158192	1.802319292	protein_coding	19	825097	832017	AZU1
ENSG00000172236	1.960915222	3.493416095	protein_coding	16	1290697	1293619	TPSAB1
ENSG00000172239	9.154055005	9.153459292	protein_coding	5	43526369	43557860	PAIP1
ENSG00000172243	2.57521082	0.390640832	protein_coding	12	10269376	10282857	CLEC7A
ENSG00000172244	7.269909329	7.434373903	protein_coding	5	43486803	43515247	C5orf34
ENSG00000172247	2.10647801	2.039052734	protein_coding	11	47611216	47616211	C1QTNF4
ENSG00000172250	2.928422289	1.667587519	processed_transc	22	42896585	42908566	SERHL
ENSG00000172260	0.869158192	4.202780776	protein_coding	1	71868625	72748417	NEGR1
ENSG00000172262	8.910650905	9.059888441	protein_coding	5	43065278	43192123	ZNF131
ENSG00000172264	3.603959378	4.250702764	protein_coding	20	13976015	16033842	MACROD2
ENSG00000172269	9.704980607	10.16982567	protein_coding	11	118967213	118979041	DPAGT1
ENSG00000172270	13.28996602	13.91694041	protein_coding	19	571310	583492	BSG
ENSG00000172273	7.642387679	8.231074746	protein_coding	11	118992297	119006752	HINFP
ENSG00000172289	0.499066092	0	protein_coding	11	59480296	59481337	OR10V1
ENSG00000172292	8.213938461	7.733180651	protein_coding	2	169312372	169631644	CERS6
ENSG00000172296	7.206730419	5.647721597	protein_coding	20	12989627	13147411	SPTLC3
ENSG00000172297	0.499066092	0	pseudogene	Y	27600708	27606719	GOLGA2P3Y
ENSG00000172301	8.422952257	8.525807191	protein_coding	17	30178883	30186310	C17orf79
ENSG00000172315	7.959880444	8.706348549	protein_coding	20	45313004	45318418	TP53RK
ENSG00000172331	7.292905885	7.307006333	protein_coding	7	134331560	134364565	BPGM
ENSG00000172336	9.118023364	8.9688072	protein_coding	7	100303676	100305118	POP7
ENSG00000172339	5.207774174	5.259915406	protein_coding	1	95448297	95538501	ALG14
ENSG00000172340	8.400188597	8.440565301	protein_coding	3	67410884	67705038	SUCLG2
ENSG00000172345	4.464551814	4.761389014	protein_coding	15	81601394	81616524	STARD5
ENSG00000172346	2.57521082	1.802319292	protein_coding	22	41956767	42069998	CSDC2
ENSG00000172348	0.499066092	0.697730409	protein_coding	6	46188475	46459709	RCAN2
ENSG00000172349	2.35979773	3.027231696	protein_coding	15	81451916	81605104	IL16
ENSG00000172350	3.554067925	3.411459265	protein_coding	11	119019722	119033360	ABCG4
ENSG00000172354	11.35675549	10.72113561	protein_coding	7	100271154	100276797	GNB2
ENSG00000172361	3.077135474	3.081239798	protein_coding	18	47753563	47792865	CCDC11
ENSG00000172366	7.974178365	8.063427911	protein_coding	16	691813	698474	FAM195A
ENSG00000172367	1.16343121	0.697730409	protein_coding	11	119056166	119060932	PDZD3
ENSG00000172375	7.698004462	7.975253757	protein_coding	11	118972908	118989252	C2CD2L
ENSG00000172379	5.858855738	5.611474642	protein_coding	15	80696692	80890278	ARNT2
ENSG00000172380	10.57833886	10.76922802	protein_coding	1	68167149	68299150	GNG12
ENSG00000172382	4.409500985	4.604259349	protein_coding	16	2762419	2770552	PRSS27
ENSG00000172399	1.407729925	0.697730409	protein_coding	4	120056939	120108944	MYOZ2
ENSG00000172403	1.407729925	2.242360793	protein_coding	4	119809996	119982402	SYNPO2
ENSG00000172404	1.407729925	1.353254395	protein_coding	22	41255553	41258130	DNAJB7
ENSG00000172409	7.935731106	8.094946597	protein_coding	11	57416465	57429340	CLP1
ENSG00000172421	2.238690726	2.144285137	protein_coding	17	60447579	60493837	EFCAB3
ENSG00000172426	2.847891871	2.788380093	protein_coding	6	43612783	43640337	RSPH9
ENSG00000172428	7.345180517	7.931818465	protein_coding	2	241065980	241076224	MYEOV2
ENSG00000172432	9.568721018	10.29187254	protein_coding	6	43573053	43596936	GTPBP2
ENSG00000172456	5.995967334	6.445267191	protein_coding	1	59762467	60233347	FGGY
ENSG00000172458	5.018397273	4.693656882	protein_coding	13	21276266	21297237	IL17D
ENSG00000172460	3.989024711	5.200150955	pseudogene	16	2889569	2892745	PRSS30P
ENSG00000172465	6.343050432	7.269696813	protein_coding	X	102883632	102885881	TCEAL1
ENSG00000172466	9.112647868	9.080752488	protein_coding	18	32912176	32924420	ZNF24
ENSG00000172469	7.457998197	7.701200733	protein_coding	6	96025419	96057333	MANEA
ENSG00000172476	2.671945279	2.912743273	protein_coding	X	102754678	102774417	RAB40A
ENSG00000172478	0.499066092	0	protein_coding	2	241825465	241836306	C2orf54
ENSG00000172482	0.499066092	0.390640832	protein_coding	2	241807896	241819919	AGXT
ENSG00000172493	8.968928959	9.069940847	protein_coding	4	87856154	88062206	AFF1
ENSG00000172500	9.707835423	10.09795979	protein_coding	11	65651212	65656010	FIBP
ENSG00000172508	5.054730169	5.200150955	protein_coding	11	67182439	67193078	CARNS1
ENSG00000172530	7.63640659	8.075120324	protein_coding	16	87982850	88152028	BANP
ENSG00000172531	12.17226388	11.95186212	protein_coding	11	67165654	67188654	PPP1CA
ENSG00000172534	11.74994854	11.26327988	protein_coding	X	153213004	153237258	HCFC1

ENSG00000172543	3.502389126	3.133298822	protein_coding	11	65647280	65651212	CTSW
ENSG00000172548	0	0.390640832	protein_coding	5	156887027	156901725	NIPAL4
ENSG00000172551	0.499066092	0	protein_coding	12	55224303	55252177	MUCL1
ENSG00000172575	4.165528823	5.150490275	protein_coding	15	38780304	38857776	RASGRP1
ENSG00000172578	0	0.390640832	protein_coding	3	183205319	183273477	KLHL6
ENSG00000172586	8.085926212	8.155990499	protein_coding	10	75541805	75543410	CHCHD1
ENSG00000172590	8.891945431	9.416334062	protein_coding	14	23299088	23304246	MRPL52
ENSG00000172594	2.57521082	4.101879561	protein_coding	6	123110315	123130865	SMPDL3A
ENSG00000172602	3.211941663	4.021284656	protein_coding	12	49250928	49259681	RND1
ENSG00000172613	9.064459359	8.958923939	protein_coding	11	67159176	67165881	RAD9A
ENSG00000172638	5.141750194	6.097646709	protein_coding	11	65633912	65641063	EFEMP2
ENSG00000172640	0.499066092	0.390640832	protein_coding	12	48596081	48597170	OR10AD1
ENSG00000172650	1.16343121	0.697730409	protein_coding	10	75434033	75457639	AGAP5
ENSG00000172653	1.960915222	1.353254395	protein_coding	17	34181955	34195895	C17orf66
ENSG00000172660	10.97774947	10.62696653	protein_coding	17	34136488	34174237	TAF15
ENSG00000172661	6.171673665	6.729413443	protein_coding	10	46222648	46288412	FAM21C
ENSG00000172663	8.770222845	9.140003549	protein_coding	11	67231824	67236743	TMEM134
ENSG00000172667	7.63640659	7.249200135	protein_coding	3	178735011	178790067	ZMAT3
ENSG00000172671	6.393570237	6.264499759	protein_coding	10	46111043	46168228	ZFAND4
ENSG00000172687	7.352496068	7.135740508	protein_coding	19	21541732	21562104	ZNF738
ENSG00000172716	2.10647801	0.950786998	protein_coding	17	33677324	33700642	SLFN11
ENSG00000172717	2.762599152	2.652276565	protein_coding	14	67656110	67695267	FAM71D
ENSG00000172725	11.34167114	11.33171911	protein_coding	11	67205519	67211292	CORO1B
ENSG00000172728	6.17992145	6.287656023	protein_coding	8	33228342	33330940	FUT10
ENSG00000172731	7.427149666	6.117167886	protein_coding	10	72058729	72142382	LRRC20
ENSG00000172732	8.443650765	9.157393097	protein_coding	11	65624597	65635124	MUS81
ENSG00000172733	0	2.039052734	protein_coding	8	30853321	30891231	PURG
ENSG00000172738	3.211941663	4.777836593	protein_coding	6	37179956	37225931	TMEM217
ENSG00000172748	4.097495944	5.486957566	protein_coding	8	182137	197342	ZNF596
ENSG00000172757	13.22923163	13.36664368	protein_coding	11	65590493	65629497	CFL1
ENSG00000172765	7.997697782	7.856387513	protein_coding	3	129366635	129612419	TMCC1
ENSG00000172766	7.311793575	6.779278886	protein_coding	13	41885341	41951166	NAA16
ENSG00000172771	2.471521042	2.788380093	protein_coding	3	129120164	129147494	C3orf25
ENSG00000172775	9.303080552	9.707843036	protein_coding	16	57186378	57220028	FAM192A
ENSG00000172780	5.018397273	4.319727133	protein_coding	3	128806412	128841644	RAB43
ENSG00000172785	7.003331296	6.889336956	protein_coding	9	121041	188979	CBWD1
ENSG00000172789	2.671945279	3.081239798	protein_coding	12	54379629	54429145	HOXC5
ENSG00000172794	2.10647801	0.950786998	protein_coding	17	72666717	72743474	RAB37
ENSG00000172795	8.364450825	8.272288337	protein_coding	5	112312399	112356667	DCP2
ENSG00000172803	3.393122761	3.813624741	protein_coding	11	65601112	65624367	SNX32
ENSG00000172809	11.13422738	11.85126812	protein_coding	17	72199721	72206676	RPL38
ENSG00000172818	4.800350936	0.390640832	protein_coding	11	65554493	65564690	OVOL1
ENSG00000172819	9.867630505	8.295320915	protein_coding	12	53604354	53626764	RARG
ENSG00000172824	3.603959378	4.489113623	protein_coding	16	67022492	67043661	CES4A
ENSG00000172828	2.10647801	3.027231696	protein_coding	16	66995140	67009051	CES3
ENSG00000172830	10.3061829	8.963424725	protein_coding	11	67070919	67080078	SSH3
ENSG00000172831	9.101836412	9.32205174	protein_coding	16	66968347	66997159	CES2
ENSG00000172840	7.384964606	7.593823731	protein_coding	16	66912492	66929657	PDP2
ENSG00000172845	9.749993326	9.406451268	protein_coding	2	174771187	174830430	SP3
ENSG00000172869	8.241782151	8.502492199	protein_coding	5	118373467	118584833	DMXL1
ENSG00000172878	6.75547892	6.877938671	protein_coding	2	172864490	172947158	METAP1D
ENSG00000172888	7.250461488	7.449853178	protein_coding	3	40566369	40616176	ZNF621
ENSG00000172889	1.960915222	1.802319292	protein_coding	9	139553308	139567130	EGFL7
ENSG00000172890	9.221529789	9.655399771	protein_coding	11	71164155	71239227	NADSYN1
ENSG00000172893	11.18171776	10.02663303	protein_coding	11	71139239	71163914	DHCR7
ENSG00000172900	0.499066092	0.390640832	pseudogene	11	71093647	71134469	
ENSG00000172901	8.16075831	2.912743273	protein_coding	5	115298151	115394680	
ENSG00000172915	5.331395195	6.167968749	protein_coding	13	35516424	36247159	NBEA
ENSG00000172922	8.249639777	9.124814753	protein_coding	11	65482367	65488418	RNASEH2C
ENSG00000172927	8.309127368	7.603083446	protein_coding	11	69061605	69182494	MYEOV
ENSG00000172932	9.976394534	9.673093524	protein_coding	11	67056018	67069956	ANKRD13D
ENSG00000172935	0.869158192	1.353254395	protein_coding	11	68771863	68780877	MRGPRF
ENSG00000172936	8.772951555	8.341695034	protein_coding	3	38179969	38184513	MYD88

ENSG00000172938	2.238690726	1.925536307	protein_coding	11	68747490	68748455	MRGPRD
ENSG00000172939	9.972245312	9.824754993	protein_coding	3	38206580	38296979	OXSR1
ENSG00000172940	2.471521042	1.667587519	protein_coding	3	38307303	38319806	SLC22A13
ENSG00000172943	8.916832554	8.837666333	protein_coding	X	53963109	54075391	PHF8
ENSG00000172954	8.749591335	8.165397208	protein_coding	2	30670092	30867091	LCLAT1
ENSG00000172955	1.616589159	2.039052734	protein_coding	4	100123795	100140694	ADH6
ENSG00000172965	7.813830732	7.913327235	processed_transc	2	111965353	112252677	
ENSG00000172967	1.407729925	1.353254395	protein_coding	22	17264302	17302589	XKR3
ENSG00000172971	0.499066092	0.697730409	pseudogene	3	75668657	75673000	
ENSG00000172974	2.847891871	1.925536307	pseudogene	2	65432137	65433396	
ENSG00000172977	8.16075831	8.093304774	protein_coding	11	65479467	65487075	KAT5
ENSG00000172985	0.499066092	0.390640832	protein_coding	2	109745804	110262207	SH3RF3
ENSG00000172986	8.484177644	8.454700467	protein_coding	3	72937224	73047289	GXYLT2
ENSG00000172992	7.985986001	7.67728988	protein_coding	17	43100710	43138473	DCAKD
ENSG00000173011	7.868433623	7.675096386	protein_coding	4	7043626	7058368	TADA2B
ENSG00000173013	3.830412367	4.127774132	protein_coding	4	7042579	7044728	CCDC96
ENSG00000173020	11.34602979	10.65895157	protein_coding	11	67033881	67054027	ADRBK1
ENSG00000173039	10.55077877	10.49484943	protein_coding	11	65421067	65430565	RELA
ENSG00000173040	1.616589159	0	protein_coding	4	5544499	5711275	EVC2
ENSG00000173041	7.481543613	6.99862477	protein_coding	7	63980262	64023484	ZNF680
ENSG00000173064	8.935220147	8.568975592	protein_coding	12	112597992	112819896	C12orf51
ENSG00000173065	8.547698305	7.864110577	protein_coding	17	27082996	27169841	C17orf63
ENSG00000173068	6.350376764	6.926693689	protein_coding	9	16416404	16870841	BNC2
ENSG00000173077	1.407729925	3.608232228	protein_coding	9	117904097	118164923	1-dec
ENSG00000173080	3.274897671	2.579085888	protein_coding	1	155911480	155912625	RXFP4
ENSG00000173083	6.882196036	6.117167886	protein_coding	4	84213614	84256306	HPSE
ENSG00000173085	7.873527714	7.737391576	protein_coding	4	84182689	84206067	COQ2
ENSG00000173088	0.499066092	1.353254395	protein_coding	10	97620305	97793507	C10orf131
ENSG00000173110	2.671945279	2.334191469	protein_coding	1	161494036	161496681	HSPA6
ENSG00000173113	10.29483576	10.51583798	protein_coding	11	64083932	64085556	TRMT112
ENSG00000173114	0	0.390640832	protein_coding	7	110731062	110765507	LRRN3
ENSG00000173120	12.18640502	11.93270983	protein_coding	11	66886740	67025141	KDM2A
ENSG00000173124	0.499066092	0.950786998	protein_coding	10	96953957	96988685	C10orf129
ENSG00000173137	6.129712275	6.605885938	protein_coding	8	145596790	145618457	ADCK5
ENSG00000173141	8.523597116	8.813937516	protein_coding	13	21750784	21753223	MRP63
ENSG00000173145	8.3053626	9.009418947	protein_coding	10	96075004	96122716	NOC3L
ENSG00000173153	9.346776019	8.958923939	protein_coding	11	64073044	64084215	ESRRA
ENSG00000173156	7.615275323	4.468996429	protein_coding	11	66824289	66839484	RHOD
ENSG00000173157	0	0.697730409	protein_coding	12	43747669	43945724	ADAMTS20
ENSG00000173163	6.138202758	7.126137778	protein_coding	2	62115859	62374382	COMMD1
ENSG00000173166	10.13698882	9.29944532	protein_coding	2	204259068	204400133	RAPH1
ENSG00000173171	8.602416377	8.632529738	protein_coding	1	155178490	155183614	MTX1
ENSG00000173175	1.799000381	2.851901313	protein_coding	3	123001143	123168605	ADCY5
ENSG00000173193	10.34291574	10.70321774	protein_coding	3	122399465	122449687	PARP14
ENSG00000173200	0.499066092	0.390640832	protein_coding	3	122296449	122357894	PARP15
ENSG00000173207	9.355904607	9.946148432	protein_coding	1	154947129	154951725	CKS1B
ENSG00000173208	0	0.390640832	protein_coding	12	39943835	40013553	ABCD2
ENSG00000173209	7.800519667	8.081759401	protein_coding	2	61404553	61414686	AHSA2
ENSG00000173210	10.23820324	9.938396195	protein_coding	5	148521046	148640105	ABLIM3
ENSG00000173212	0.499066092	0	protein_coding	1	116654376	116677861	MAB21L3
ENSG00000173213	1.616589159	0.390640832	protein_coding	18	47390	49557	
ENSG00000173214	5.707331524	5.931169853	protein_coding	6	111580551	111592370	KIAA1919
ENSG00000173218	8.710271506	8.633659743	protein_coding	1	116184574	116240845	VANGL1
ENSG00000173221	6.456028446	6.660126317	protein_coding	5	95087023	95158709	GLRX
ENSG00000173226	8.179389493	8.436686098	protein_coding	3	121488610	121553926	IQCB1
ENSG00000173227	8.360827871	6.746227492	protein_coding	11	66774249	66818334	SYT12
ENSG00000173230	9.984068715	10.35386622	protein_coding	3	121382046	121468602	GOLGB1
ENSG00000173237	4.800350936	4.42790041	protein_coding	11	66742748	66744475	C11orf86
ENSG00000173239	1.960915222	0	protein_coding	10	90562654	90580303	LIPM
ENSG00000173253	2.57521082	0	protein_coding	9	1050354	1057552	DMRT2
ENSG00000173258	4.026095388	2.912743273	protein_coding	9	114287439	114340124	ZNF483
ENSG00000173261	3.211941663	3.813624741	protein_coding	5	145463949	145483932	PLAC8L1
ENSG00000173262	0.869158192	0.697730409	protein_coding	12	7965108	8043744	SLC2A14

ENSG00000173264	8.490822808	9.250933796	protein_coding	11	64037534	64056972	GPR137
ENSG00000173267	6.228441838	4.363974406	protein_coding	10	88718375	88723017	SNCG
ENSG00000173269	4.097495944	3.87606762	protein_coding	10	88695297	88729238	MMRN2
ENSG00000173272	8.398422578	8.056703738	protein_coding	2	132222473	132250316	MZT2A
ENSG00000173273	8.840844993	8.874456566	protein_coding	8	9413424	9639856	TNKS
ENSG00000173275	6.320845526	6.877938671	protein_coding	X	134478721	134497077	ZNF449
ENSG00000173276	8.74542939	8.876367148	protein_coding	21	43406940	43430496	ZNF295
ENSG00000173281	8.687312327	8.514810202	protein_coding	8	8993765	9009084	PPP1R3B
ENSG00000173295	5.431503808	4.94736961	protein_coding	8	8097891	8102384	
ENSG00000173320	3.554067925	2.579085888	protein_coding	4	184774584	184944679	STOX2
ENSG00000173327	10.21932096	9.711058296	protein_coding	11	65365226	65382853	MAP3K11
ENSG00000173334	9.106170722	8.558303982	protein_coding	8	126442563	126450647	TRIB1
ENSG00000173338	2.471521042	2.242360793	protein_coding	11	65360326	65363467	KCNK7
ENSG00000173391	10.60193356	9.865242842	protein_coding	12	10310902	10324737	OLR1
ENSG00000173401	1.960915222	0.950786998	protein_coding	12	75728419	75764340	GLIPR1L1
ENSG00000173402	12.00492709	11.89173735	protein_coding	3	49506146	49573048	DAG1
ENSG00000173409	7.266040677	7.326703453	protein_coding	1	231114727	231136341	ARV1
ENSG00000173418	9.422587759	9.607894053	protein_coding	20	19997760	20014299	NAA20
ENSG00000173421	3.004694206	4.075511708	protein_coding	3	49235861	49295537	CCDC36
ENSG00000173431	0.869158192	0.390640832	protein_coding	14	21525981	21526614	RNASE8
ENSG00000173432	8.744039402	8.574870331	protein_coding	11	18287721	18291524	SAA1
ENSG00000173436	8.78921631	9.36009759	protein_coding	1	19923477	19956314	MINOS1
ENSG00000173442	10.39344018	10.20369859	protein_coding	11	65343509	65360121	EHBP1L1
ENSG00000173451	5.175139844	5.32851069	protein_coding	12	72056789	72074419	THAP2
ENSG00000173456	9.893563944	9.745961151	protein_coding	11	119205237	119208023	RNF26
ENSG00000173457	10.70232786	10.80377176	protein_coding	11	64011956	64014413	PPP1R14B
ENSG00000173465	8.474152153	9.08571528	protein_coding	11	65337901	65341413	SSSCA1
ENSG00000173473	10.73697893	10.72086981	protein_coding	3	47626762	47823596	SMARCC1
ENSG00000173480	7.017207048	7.343375265	protein_coding	19	58417142	58427978	ZNF417
ENSG00000173482	6.975172889	7.444711851	protein_coding	18	7567817	8406859	PTPRM
ENSG00000173486	8.694526397	9.144766978	protein_coding	11	64008475	64011604	FKBP2
ENSG00000173511	9.051032802	9.004186147	protein_coding	11	64002010	64006259	VEGFB
ENSG00000173517	8.544507981	8.693401904	protein_coding	15	77400471	77712486	
ENSG00000173530	7.549958459	7.532142019	protein_coding	8	22993101	23021543	TNFRSF10D
ENSG00000173531	4.593635215	5.496926774	protein_coding	3	49721380	49726934	MST1
ENSG00000173535	4.464551814	4.021284656	protein_coding	8	22941868	22990110	TNFRSF10C
ENSG00000173540	3.911897206	4.048652948	protein_coding	3	49754277	49761384	GMPPB
ENSG00000173542	8.325948295	8.213044874	protein_coding	4	71768043	71888166	MOB1B
ENSG00000173545	9.711396018	9.509762881	protein_coding	5	16451628	16465901	ZNF622
ENSG00000173546	6.528867325	5.784235782	protein_coding	15	75966663	76005189	CSPG4
ENSG00000173548	8.763378405	8.971490926	protein_coding	15	75940247	75954642	SNX33
ENSG00000173557	3.211941663	1.518964905	protein_coding	2	26785450	26802400	C2orf70
ENSG00000173559	8.875535495	9.381107599	protein_coding	2	192542794	192553251	OBFC2A
ENSG00000173566	6.228441838	6.424524724	protein_coding	8	21964383	21966932	NUDT18
ENSG00000173567	2.238690726	2.334191469	protein_coding	2	26531041	26569685	GPR113
ENSG00000173575	10.17874213	10.06357588	protein_coding	15	93425937	93571237	CHD2
ENSG00000173578	0.499066092	0.390640832	protein_coding	3	46058516	46069234	XCR1
ENSG00000173581	5.124761037	5.709041716	protein_coding	19	56158954	56164527	CCDC106
ENSG00000173588	6.94645393	7.106738413	protein_coding	12	94700225	94853764	CCDC41
ENSG00000173598	9.542574757	9.339469783	protein_coding	12	93771659	93797024	NUDT4
ENSG00000173599	11.17270558	10.6281009	protein_coding	11	66615704	66725847	PC
ENSG00000173611	5.967663407	5.656642807	protein_coding	9	127714380	127905785	SCAI
ENSG00000173614	6.522395495	6.668971382	protein_coding	1	10003486	10045559	NMNAT1
ENSG00000173621	9.882235894	9.856074312	protein_coding	11	66624118	66627946	LRFN4
ENSG00000173627	0	0.697730409	protein_coding	1	183615541	183622451	APOBEC4
ENSG00000173638	9.455232818	9.497401522	protein_coding	21	46913486	46964325	SLC19A1
ENSG00000173641	0	0.697730409	protein_coding	1	16340523	16346089	HSPB7
ENSG00000173653	9.265790136	8.872543451	protein_coding	11	66610306	66614017	RCE1
ENSG00000173660	9.328343713	9.630144961	protein_coding	1	46769303	46782448	UQCRH
ENSG00000173662	0.499066092	0.697730409	protein_coding	1	6615241	6639817	TAS1R1
ENSG00000173674	10.26655536	10.29294578	protein_coding	X	20142636	20159962	EIF1AX
ENSG00000173681	7.017207048	7.073814954	protein_coding	X	19930978	19988416	CXorf23
ENSG00000173692	10.86621944	10.819994	protein_coding	2	231921578	232037541	PSMD1

ENSG00000173698	1.407729925	2.144285137	protein_coding	X	19007427	19140755	GPR64
ENSG00000173702	0	0.697730409	protein_coding	3	124624289	124672663	MUC13
ENSG00000173705	2.847891871	2.652276565	protein_coding	3	33191537	33260707	SUSD5
ENSG00000173706	10.88273508	11.00666383	protein_coding	3	124684554	124774802	HEG1
ENSG00000173715	8.205883411	8.157562549	protein_coding	11	66511922	66610987	C11orf80
ENSG00000173726	10.96199326	11.08991059	protein_coding	1	235272651	235292251	TOMM20
ENSG00000173727	5.346128795	5.456629825	protein_coding	11	65222728	65234028	
ENSG00000173728	1.616589159	0.697730409	protein_coding	1	244515937	244552965	C1orf100
ENSG00000173744	10.00452934	9.53114363	protein_coding	2	228336868	228421384	AGFG1
ENSG00000173757	9.137210979	9.383796417	protein_coding	17	40351186	40428725	STAT5B
ENSG00000173762	1.407729925	0	protein_coding	17	80272746	80275480	CD7
ENSG00000173786	9.920270706	9.654286673	protein_coding	17	40118759	40127194	CNP
ENSG00000173801	10.61187421	9.176127473	protein_coding	17	39775692	39943183	JUP
ENSG00000173805	0.499066092	0.390640832	protein_coding	17	39873994	39890896	HAP1
ENSG00000173809	0	0.697730409	protein_coding	19	33210659	33320483	TDRD12
ENSG00000173811	0.499066092	0.697730409	antisense	3	42774067	42788260	CCDC13-AS1
ENSG00000173812	12.19406034	11.79253236	protein_coding	17	39845137	39848920	EIF1
ENSG00000173818	7.784382559	7.646273261	protein_coding	17	78388965	78411886	ENDOV
ENSG00000173821	11.14269141	11.02464977	protein_coding	17	78234665	78370078	RNF213
ENSG00000173825	3.077135474	3.183544561	protein_coding	11	65122238	65125084	TIGD3
ENSG00000173838	1.799000381	1.802319292	protein_coding	17	60778676	60885705	10-Mar
ENSG00000173846	6.646617938	6.911866869	protein_coding	1	45265897	45271662	PLK3
ENSG00000173848	9.796328827	8.605140645	protein_coding	10	5454514	5500426	NET1
ENSG00000173852	9.813659349	9.886254153	protein_coding	7	34961081	35077883	DPY19L1
ENSG00000173867	0.869158192	0.950786998	processed_transc	15	89002948	89054752	MRPL46
ENSG00000173868	3.146112541	3.232099092	protein_coding	17	47300724	47308128	PHOSPHO1
ENSG00000173875	8.565120083	8.431497548	protein_coding	19	12721732	12742735	ZNF791
ENSG00000173876	1.407729925	1.353254395	protein_coding	10	92828	96053	TUBB8
ENSG00000173889	8.072698871	7.928139131	protein_coding	3	169804520	169899537	PHC3
ENSG00000173890	3.393122761	3.453019579	protein_coding	3	169755717	169803191	GPR160
ENSG00000173894	8.18144485	7.924450389	protein_coding	17	77751977	77761449	CBX2
ENSG00000173898	9.784883719	8.630267069	protein_coding	11	66452719	66496697	SPTBN2
ENSG00000173905	9.585892264	9.474880612	protein_coding	3	167726465	167813763	GOLIM4
ENSG00000173914	7.720795447	7.992972811	protein_coding	11	66432469	66445392	RBM4B
ENSG00000173915	8.337054268	9.103767504	protein_coding	10	105148798	105156223	USMG5
ENSG00000173918	11.69597623	11.6385214	protein_coding	17	77020251	77045870	C1QTNF1
ENSG00000173926	7.813830732	8.327799018	protein_coding	5	126203406	126366500	3-Mar
ENSG00000173928	5.785082014	6.332882496	protein_coding	19	11485383	11487627	SWSAP1
ENSG00000173930	0.869158192	0	protein_coding	5	101569690	101632253	SLCO4C1
ENSG00000173933	6.462804234	6.408769788	protein_coding	11	66406088	66434153	RBM4
ENSG00000173947	1.407729925	3.532712221	protein_coding	1	111888910	111895635	C1orf88
ENSG00000173950	8.75235931	9.077434451	protein_coding	3	194789008	194991896	XXYLT1
ENSG00000173960	7.776245808	7.289906366	protein_coding	2	24150155	24227779	UBXN2A
ENSG00000173976	1.16343121	0.390640832	protein_coding	19	3769087	3772233	RAX2
ENSG00000173988	0.499066092	0.390640832	protein_coding	13	46786083	46844757	LRRC63
ENSG00000173991	4.19837882	3.368666104	protein_coding	17	37820440	37822808	TCAP
ENSG00000173992	9.153007996	9.759482214	protein_coding	11	66360292	66373490	CCS
ENSG00000174004	2.10647801	1.353254395	protein_coding	3	196366557	196388875	LRRC33
ENSG00000174007	5.375152132	5.085909553	protein_coding	3	196433148	196439164	CEP19
ENSG00000174010	7.33043718	7.429177016	protein_coding	X	24001837	24045303	KLHL15
ENSG00000174013	9.169669647	8.606292299	protein_coding	3	196295482	196315930	FBXO45
ENSG00000174021	8.864064429	9.223427792	protein_coding	1	84964008	84972248	GNG5
ENSG00000174028	0.869158192	1.353254395	pseudogene	X	23093875	23094884	FAM3C2
ENSG00000174032	6.400645076	5.045726316	protein_coding	13	45967451	45992590	SLC25A30
ENSG00000174038	2.35979773	1.802319292	protein_coding	9	35041092	35045988	C9orf131
ENSG00000174059	4.543379137	4.250702764	protein_coding	1	208057594	208084747	CD34
ENSG00000174080	7.218789138	8.963424725	protein_coding	11	66330934	66336312	CTSF
ENSG00000174083	0.499066092	1.353254395	processed_transc	17	8706041	8770994	PIK3R6
ENSG00000174093	5.774228067	5.620622115	protein_coding	17	36337717	36421858	
ENSG00000174099	7.80585884	8.385283657	protein_coding	12	65672423	65882024	MSRB3
ENSG00000174100	8.429884808	8.620040914	protein_coding	17	36452990	36479101	MRPL45
ENSG00000174106	7.824391858	7.937319932	protein_coding	12	65563351	65642107	LEMD3
ENSG00000174109	6.94645393	7.568048738	protein_coding	16	1469745	1479345	C16orf91

ENSG00000174111	5.637181576	5.212303549	protein_coding	17	36508005	36556015	SOCS7
ENSG00000174123	0.499066092	0	protein_coding	4	38773860	38784611	TLR10
ENSG00000174125	3.335220907	4.604259349	protein_coding	4	38792298	38858438	TLR1
ENSG00000174130	6.244259325	7.176629214	protein_coding	4	38825336	38858438	TLR6
ENSG00000174132	6.489593565	6.338437404	protein_coding	5	99871009	99922445	FAM174A
ENSG00000174136	8.183497283	8.394645624	protein_coding	5	98104354	98134347	RGMB
ENSG00000174137	4.230497448	5.09905904	protein_coding	4	1619642	1686029	FAM53A
ENSG00000174151	7.119373418	7.126137778	protein_coding	1	110036674	110045554	CYB561D1
ENSG00000174165	9.383841641	9.672543867	protein_coding	11	66288108	66313709	ZDHHC24
ENSG00000174171	0.499066092	0.390640832	processed_transc	15	42184991	42189585	
ENSG00000174173	8.3480754	8.650504459	protein_coding	3	101280706	101285290	RG9MTD1
ENSG00000174177	9.008829705	8.618900187	protein_coding	16	88772871	88781794	CTU2
ENSG00000174194	3.989024711	3.813624741	protein_coding	10	51224677	51252947	AGAP8
ENSG00000174197	10.06922944	10.00845419	protein_coding	15	41913422	42062141	MGA
ENSG00000174206	7.242608348	6.716673044	protein_coding	12	64580096	64616076	C12orf66
ENSG00000174225	1.16343121	1.353254395	protein_coding	X	100224697	100245818	ARL13A
ENSG00000174227	8.78516728	9.110276346	protein_coding	4	492989	533985	PIGG
ENSG00000174231	13.14642762	13.48801226	protein_coding	17	1553923	1588154	PRPF8
ENSG00000174233	7.666067113	8.211532164	protein_coding	12	49159975	49182820	ADCY6
ENSG00000174236	0.499066092	0.950786998	protein_coding	12	27849428	27850566	REP15
ENSG00000174238	10.24657122	10.64669035	protein_coding	17	1421287	1466110	PITPNA
ENSG00000174243	9.815645694	9.994887996	protein_coding	12	49223840	49246625	DDX23
ENSG00000174276	7.262161623	7.4317778	protein_coding	11	64883875	64885170	ZNHIT2
ENSG00000174282	10.08083775	10.38975587	protein_coding	17	7362685	7387517	ZBTB4
ENSG00000174292	8.052627651	7.76240224	protein_coding	17	7284365	7293092	TNK1
ENSG00000174306	9.105088364	9.212125573	protein_coding	20	39807088	39946312	ZHX3
ENSG00000174307	6.825495146	3.133298822	protein_coding	1	201434620	201438365	PHLDA3
ENSG00000174325	0	0.390640832	protein_coding	2	189598882	189654831	DIRC1
ENSG00000174326	2.671945279	2.788380093	protein_coding	17	6944949	6947242	SLC16A11
ENSG00000174327	8.75235931	8.89250574	protein_coding	17	6939477	6943438	SLC16A13
ENSG00000174332	0	0.697730409	protein_coding	1	53971910	54199877	GLIS1
ENSG00000174343	0	1.16600992	protein_coding	4	40337346	40357234	CHRNA9
ENSG00000174353	5.124761037	5.317302248	protein_coding	7	72440227	72476445	STAG3L3
ENSG00000174358	0.499066092	0.950786998	protein_coding	5	1201710	1225232	SLC6A19
ENSG00000174365	6.041940478	7.188980716	processed_transc	20	37075221	37079564	SNHG11
ENSG00000174368	0	0.697730409	pseudogene	7	74880905	74988262	PMS2P2
ENSG00000174370	6.155035276	6.327306117	protein_coding	11	128769460	128775930	C11orf45
ENSG00000174371	8.593182305	9.207579776	protein_coding	1	242011269	242058450	EXO1
ENSG00000174373	6.902274257	7.170413581	protein_coding	14	36007558	36278510	RALGAPA1
ENSG00000174374	8.977234519	8.672662438	protein_coding	7	74441226	74490064	WBSCR16
ENSG00000174403	0.499066092	0.390640832	antisense	20	61141550	61148733	C20orf166-AS1
ENSG00000174405	6.509364082	6.811590016	protein_coding	13	108859794	108870716	LIG4
ENSG00000174417	0	0.697730409	protein_coding	8	110098850	110131813	TRHR
ENSG00000174428	3.989024711	3.368666104	protein_coding	7	74508364	74767788	GTF2IRD2B
ENSG00000174429	0.499066092	0.697730409	protein_coding	8	107771711	107782473	ABRA
ENSG00000174437	11.65625525	11.23712609	protein_coding	12	110718561	110788898	ATP2A2
ENSG00000174442	8.568265199	8.922403049	protein_coding	15	66797297	66842115	ZWILCH
ENSG00000174444	12.64955038	12.95642923	protein_coding	15	66790355	66816870	RPL4
ENSG00000174446	7.0218028	7.295628905	protein_coding	15	66782644	66790151	SNAPC5
ENSG00000174448	0	0.390640832	protein_coding	18	51850964	51880943	STARD6
ENSG00000174450	1.407729925	0.950786998	protein_coding	15	23684645	23692381	GOLGA6L2
ENSG00000174456	5.270908378	5.555337277	protein_coding	12	110465521	110511491	C12orf76
ENSG00000174460	0.499066092	0	protein_coding	X	117957753	117960931	ZCCHC12
ENSG00000174469	0.499066092	2.501982735	protein_coding	7	145813453	148118090	CNTNAP2
ENSG00000174473	0.499066092	0.697730409	protein_coding	4	172733405	173962710	GALNTL6
ENSG00000174483	4.6658819	5.271576845	protein_coding	11	66278077	66301098	BBS1
ENSG00000174485	7.981274537	8.081759401	protein_coding	15	65950384	66084631	DENND4A
ENSG00000174498	0	0.390640832	protein_coding	15	65619465	65670378	IGDCC3
ENSG00000174500	4.842525946	4.676215056	protein_coding	3	111839688	111852152	GCET2
ENSG00000174501	4.942865586	4.857374129	protein_coding	2	96514587	96657541	ANKRD36C
ENSG00000174502	1.16343121	1.16600992	protein_coding	1	205882176	205912588	SLC26A9
ENSG00000174514	2.762599152	2.851901313	protein_coding	1	205538013	205572046	MFSD4
ENSG00000174516	7.921045056	8.157562549	protein_coding	11	66234216	66244808	PELI3

ENSG00000174521	1.16343121	0.697730409	protein_coding	19	40721965	40724306	TTC9B
ENSG00000174527	1.960915222	2.579085888	protein_coding	12	109826524	109893328	MYO1H
ENSG00000174529	4.491309013	3.570966319	protein_coding	1	205052258	205053645	TMEM81
ENSG00000174547	10.45165245	10.52408771	protein_coding	11	66202546	66234209	MRPL11
ENSG00000174564	6.835971536	4.903073497	protein_coding	3	136665072	136729927	IL20RB
ENSG00000174567	3.335220907	2.971122874	protein_coding	1	204167288	204183220	GOLT1A
ENSG00000174572	0.499066092	0.697730409	pseudogene	6	27179023	27179663	
ENSG00000174574	8.557227164	8.251828705	protein_coding	1	39456895	39471731	AKIRIN1
ENSG00000174579	8.807962967	8.157562549	protein_coding	3	135867764	135916083	MSL2
ENSG00000174586	7.787084643	7.70765368	protein_coding	19	58865725	58874120	ZNF497
ENSG00000174599	3.211941663	4.744751758	protein_coding	4	118004718	118006736	TRAM1L1
ENSG00000174600	0	0.697730409	protein_coding	12	108681821	108733118	CMKLR1
ENSG00000174606	8.05038018	8.410554709	protein_coding	1	213165524	213189168	ANGEL2
ENSG00000174607	5.270908378	0.950786998	protein_coding	4	115519611	115599380	UGT8
ENSG00000174611	0	0.390640832	protein_coding	3	134321980	134370478	KY
ENSG00000174628	7.273767634	7.161039629	protein_coding	16	19727778	19868907	IQCK
ENSG00000174640	3.146112541	1.518964905	protein_coding	3	133651540	133771028	SLCO2A1
ENSG00000174652	9.918423401	10.53016835	protein_coding	19	9523272	9546234	ZNF266
ENSG00000174669	8.678607554	7.57041104	protein_coding	11	66129992	66139685	SLC29A2
ENSG00000174672	7.853042529	7.480322213	protein_coding	11	1411129	1483919	BRSK2
ENSG00000174684	7.923503136	7.809160246	protein_coding	11	66112843	66115163	B3GNT1
ENSG00000174695	9.712107084	9.711058296	protein_coding	5	82348665	82373682	TMEM167A
ENSG00000174705	9.166560209	9.148724524	protein_coding	5	171752185	171881527	SH3PXD2B
ENSG00000174715	1.16343121	0.950786998	pseudogene	3	138362716	138363536	
ENSG00000174718	10.38454676	9.977637634	protein_coding	12	32112304	32146039	C12orf35
ENSG00000174720	7.981274537	8.529454305	protein_coding	4	113558120	113578748	LARP7
ENSG00000174721	3.077135474	4.29708397	protein_coding	10	93666346	93669240	FGFBP3
ENSG00000174738	8.112022537	8.441856054	protein_coding	3	23986751	24021237	NR1D2
ENSG00000174744	9.43383913	9.588211811	protein_coding	11	66104804	66112596	BRMS1
ENSG00000174748	10.99538144	11.4288133	protein_coding	3	23958036	23965183	RPL15
ENSG00000174749	7.789781675	7.879433769	protein_coding	4	113066553	113110237	C4orf32
ENSG00000174775	8.42468852	7.944622739	protein_coding	11	532242	537287	HRAS
ENSG00000174776	0.869158192	0	protein_coding	3	167196472	167371771	WDR49
ENSG00000174780	10.60691245	10.5994706	protein_coding	4	57333081	57369846	SRP72
ENSG00000174788	1.616589159	1.925536307	protein_coding	19	7696509	7698570	PCP2
ENSG00000174791	8.735671237	8.127396674	protein_coding	11	66097713	66104311	RIN1
ENSG00000174792	1.407729925	3.411459265	protein_coding	4	76481258	76491095	C4orf26
ENSG00000174796	6.068837212	6.485878328	protein_coding	4	76439156	76475683	THAP6
ENSG00000174799	6.917151646	7.63953919	protein_coding	4	56815037	56899270	CEP135
ENSG00000174804	6.112579918	5.80042639	protein_coding	11	86656721	86666433	FZD4
ENSG00000174807	0.499066092	0.390640832	protein_coding	11	66081958	66084515	CD248
ENSG00000174808	3.448790144	3.368666104	protein_coding	4	75669969	75719896	BTC
ENSG00000174827	3.004694206	2.144285137	protein_coding	1	145726918	145764074	PDZK1
ENSG00000174837	2.471521042	1.518964905	protein_coding	19	6887582	6952102	EMR1
ENSG00000174839	8.259402007	8.238521194	protein_coding	3	57611184	57678816	FAM116A
ENSG00000174840	8.783815073	8.482561984	protein_coding	3	57542003	57547684	PDE12
ENSG00000174842	6.496213887	6.95589788	protein_coding	1	92711959	92764544	GLMN
ENSG00000174844	1.799000381	1.353254395	protein_coding	3	57327727	57530071	DNAH12
ENSG00000174851	9.830784156	10.24310134	protein_coding	11	66052051	66056641	YIF1A
ENSG00000174871	3.146112541	3.279072565	protein_coding	11	66045661	66052772	CNIH2
ENSG00000174885	3.652182994	2.242360793	protein_coding	11	278365	285359	NLRP6
ENSG00000174886	7.356139979	8.129000175	protein_coding	19	5892928	5904025	NDUFA11
ENSG00000174891	8.148202495	8.056703738	protein_coding	3	157823644	158263519	RSRC1
ENSG00000174898	1.16343121	0.697730409	protein_coding	19	5720688	5778745	TMEM146
ENSG00000174899	3.448790144	4.29708397	protein_coding	3	157261035	157395538	C3orf55
ENSG00000174903	10.38811071	10.2684207	protein_coding	11	66036004	66044963	RAB1B
ENSG00000174912	0.499066092	0	pseudogene	3	156431673	156432717	METTL15P1
ENSG00000174915	8.541310587	8.598211347	protein_coding	11	448268	491393	PTDSS2
ENSG00000174917	9.46032044	10.14349205	protein_coding	19	5678433	5680911	C19orf70
ENSG00000174928	5.175139844	5.018301917	protein_coding	3	155480401	155524140	C3orf33
ENSG00000174938	9.250208226	9.828213847	protein_coding	16	29882480	29910868	SEZ6L2
ENSG00000174939	7.803191723	8.015686953	protein_coding	16	29911696	29931185	ASPHD1
ENSG00000174943	8.274885464	8.347216179	protein_coding	16	29916333	29938356	KCTD13

ENSG00000174945	3.554067925	2.912743273	protein_coding	7	2719156	2804759	AMZ1
ENSG00000174950	2.10647801	0.390640832	protein_coding	1	27705666	27709870	CD164L2
ENSG00000174951	6.386460534	5.112089758	protein_coding	19	49251268	49258647	FUT1
ENSG00000174953	9.660729799	9.378413761	protein_coding	3	153990335	154042286	DHX36
ENSG00000174977	0.499066092	1.667587519	pseudogene	17	18553508	18554855	
ENSG00000174989	8.282565319	8.402622096	protein_coding	12	117348761	117470180	FBXW8
ENSG00000174990	0	0.390640832	protein_coding	16	87921625	87970135	CA5A
ENSG00000174992	0	0.390640832	protein_coding	16	29789561	29793096	ZG16
ENSG00000174996	10.04403543	9.908831886	protein_coding	11	66024765	66035331	KLC2
ENSG00000175003	3.911897206	4.585695337	protein_coding	6	160542821	160579750	SLC22A1
ENSG00000175029	9.953123981	10.11586039	protein_coding	10	126676421	126849739	CTBP2
ENSG00000175048	7.381392865	7.348889996	protein_coding	6	157802165	158099178	ZDHHC14
ENSG00000175054	9.30119149	9.260474903	protein_coding	3	142168077	142297668	ATR
ENSG00000175061	12.03080722	12.11630032	non_coding	17	16342289	16367300	C17orf76-AS1
ENSG00000175063	10.20018826	10.26696498	protein_coding	20	44441215	44445596	UBE2C
ENSG00000175066	7.474855471	7.549009758	protein_coding	3	141882414	141944449	GK5
ENSG00000175073	8.962967004	8.301022059	protein_coding	8	67540722	67579452	VCPIP1
ENSG00000175084	4.77879189	4.319727133	protein_coding	2	220283099	220291461	DES
ENSG00000175087	6.155035276	6.434933235	protein_coding	1	26437664	26452034	PDIK1L
ENSG00000175097	0.499066092	0.390640832	protein_coding	11	36597124	36619829	RAG2
ENSG00000175104	6.386460534	6.866449614	protein_coding	11	36508577	36531822	TRAF6
ENSG00000175105	6.681890601	6.839280559	protein_coding	3	88188254	88193815	ZNF654
ENSG00000175106	5.588445295	5.855704078	protein_coding	17	15341205	15466945	FAM18B2
ENSG00000175110	8.337054268	8.620040914	protein_coding	3	138724648	139076065	MRPS22
ENSG00000175115	10.48490234	10.14547392	protein_coding	11	65837834	66012218	PACS1
ENSG00000175121	0	0.390640832	protein_coding	20	43738093	43743813	WFDC5
ENSG00000175130	9.679029815	9.859458974	protein_coding	1	32799433	32801980	MARCKSL1
ENSG00000175137	9.006512903	9.472987904	protein_coding	1	249104648	249120832	SH3BP5L
ENSG00000175147	1.960915222	0.950786998	processed_transc	1	15442448	15478960	C1orf126
ENSG00000175155	6.766474304	6.424524724	protein_coding	17	57409053	57479095	YPEL2
ENSG00000175161	0.869158192	0	protein_coding	3	85008132	86123579	CADM2
ENSG00000175164	0.869158192	0	processed_transc	9	136131053	136150617	ABO
ENSG00000175166	12.59950889	12.55590422	protein_coding	3	184016497	184026842	PSMD2
ENSG00000175170	5.60078458	6.465715653	protein_coding	20	25744102	25848786	FAM182B
ENSG00000175175	6.93675248	7.070480904	protein_coding	17	56833232	57062532	PPM1E
ENSG00000175182	7.850461307	7.780879746	protein_coding	3	184053714	184064063	FAM131A
ENSG00000175183	6.469548347	7.38691391	protein_coding	12	77252495	77272840	CSRP2
ENSG00000175189	1.960915222	1.802319292	protein_coding	12	57828527	57845842	INHBC
ENSG00000175193	8.455589162	8.289597152	protein_coding	3	183547173	183602721	PARL
ENSG00000175197	6.760987087	7.842771865	protein_coding	12	57910371	57914300	DDIT3
ENSG00000175198	7.285281023	7.225413142	protein_coding	13	100741269	101182686	PCCA
ENSG00000175203	10.42240548	10.74093244	protein_coding	12	57923885	57941114	DCTN2
ENSG00000175206	3.393122761	3.411459265	protein_coding	1	11905766	11908402	NPPA
ENSG00000175213	7.478203418	7.556178852	protein_coding	11	46722368	46727462	ZNF408
ENSG00000175215	11.26038994	11.02249463	protein_coding	12	58213710	58240522	CTDSP2
ENSG00000175216	11.49187142	11.47067432	protein_coding	11	46764598	46867847	CKAP5
ENSG00000175220	10.31650661	10.14745308	protein_coding	11	46698630	46722165	ARHGAP1
ENSG00000175221	9.825536602	9.823765224	protein_coding	19	867964	893218	MED16
ENSG00000175224	10.26413304	10.15219202	protein_coding	11	46638826	46696368	ATG13
ENSG00000175229	0.499066092	0.950786998	protein_coding	11	65809423	65816651	GAL3ST3
ENSG00000175262	0.499066092	0	protein_coding	1	11006528	11042094	C1orf127
ENSG00000175264	1.799000381	0	protein_coding	11	45670427	45687172	CHST1
ENSG00000175265	5.375152132	6.25865215	protein_coding	15	34671269	34880704	GOLGA8A
ENSG00000175267	0.499066092	0	protein_coding	16	22103859	22168287	VWA3A
ENSG00000175274	10.03724206	9.936566069	protein_coding	11	44907454	44972840	TP53I11
ENSG00000175279	5.929039784	6.577981353	protein_coding	1	10490159	10512210	APITD1
ENSG00000175283	8.094677541	8.199373043	protein_coding	9	131707809	131709898	DOLK
ENSG00000175287	3.78787645	2.501982735	protein_coding	9	131683174	131704320	PHYHD1
ENSG00000175294	4.409500985	4.585695337	protein_coding	11	65784223	65793988	CATSPER1
ENSG00000175305	5.550781289	6.016808232	protein_coding	8	95891998	95908906	CCNE2
ENSG00000175309	7.768062907	7.544210495	protein_coding	5	177635498	177659792	AGXT2L2
ENSG00000175311	1.16343121	1.16600992	protein_coding	16	21245016	21263750	ANKS4B
ENSG00000175315	4.464551814	1.518964905	protein_coding	11	65779312	65780976	CST6

ENSG00000175318	3.871730003	3.32456471	protein_coding	15	72452148	72490126	GRAMD2
ENSG00000175322	5.417620896	5.283144778	protein_coding	18	14099942	14132489	ZNF519
ENSG00000175324	6.298293508	6.815578497	protein_coding	8	38020839	38034248	LSM1
ENSG00000175329	1.407729925	2.039052734	protein_coding	22	35462129	35486030	ISX
ENSG00000175334	10.31369837	10.76845662	protein_coding	11	65769550	65771620	BANF1
ENSG00000175336	0.499066092	0	protein_coding	12	56754353	56756607	APOF
ENSG00000175344	2.238690726	2.420525079	protein_coding	15	30875128	32464722	CHRNA7
ENSG00000175348	8.280649185	8.467431533	protein_coding	11	8968841	8986558	TMEM9B
ENSG00000175352	7.210761201	6.952279523	protein_coding	11	9002123	9025596	NRIP3
ENSG00000175354	7.96466218	7.953699568	protein_coding	18	12785477	12884337	PTPN2
ENSG00000175356	0.499066092	2.144285137	protein_coding	11	9041071	9159661	SCUBE2
ENSG00000175376	8.523597116	8.319396705	protein_coding	11	65764016	65769647	EIF1AD
ENSG00000175387	9.356814297	9.424191842	protein_coding	18	45357922	45457515	SMAD2
ENSG00000175390	9.963910896	10.41363432	protein_coding	11	7991798	8023409	EIF3F
ENSG00000175395	5.660945913	7.246248121	protein_coding	10	38238500	38265561	ZNF25
ENSG00000175414	2.57521082	3.906303962	protein_coding	5	175792471	175828866	ARL10
ENSG00000175416	9.233465948	9.471092709	protein_coding	5	175819456	175843570	CLTB
ENSG00000175426	0.499066092	0.950786998	protein_coding	5	95726119	95769847	PCSK1
ENSG00000175449	3.335220907	3.748356452	protein_coding	5	94982458	94993786	RFESD
ENSG00000175455	9.072234198	9.801313989	protein_coding	3	123616152	123680564	CCDC14
ENSG00000175463	6.103936836	5.259915406	protein_coding	11	67171386	67177560	TBC1D10C
ENSG00000175467	10.49319987	10.39242863	protein_coding	11	65729160	65747299	SART1
ENSG00000175470	8.508940744	8.583082711	protein_coding	10	133747955	133773331	PPP2R2D
ENSG00000175471	6.146643566	5.886365696	protein_coding	5	94039446	94620279	MCTP1
ENSG00000175482	7.609180529	7.747865429	protein_coding	11	67118248	67124443	POLD4
ENSG00000175489	0	0.697730409	protein_coding	19	18501955	18508415	LRRC25
ENSG00000175505	9.049908262	8.633659743	protein_coding	11	67131639	67141648	CLCF1
ENSG00000175509	0.869158192	0	pseudogene	2	114425056	114426201	
ENSG00000175514	0	0.390640832	protein_coding	11	67218772	67220200	GPR152
ENSG00000175518	3.211941663	3.133298822	protein_coding	11	5535623	5537935	UBQLNL
ENSG00000175536	2.847891871	3.493416095	protein_coding	11	74202757	74204778	LIPT2
ENSG00000175544	5.795854912	5.163066621	protein_coding	11	67219877	67226699	CABP4
ENSG00000175548	5.223818625	6.3217081	protein_coding	12	38710380	38717784	ALG10B
ENSG00000175550	9.931305133	9.930602076	protein_coding	11	65686728	65689032	DRAP1
ENSG00000175556	7.266040677	7.219404604	protein_coding	X	118108581	118156888	LONRF3
ENSG00000175564	3.603959378	3.453019579	protein_coding	11	73711326	73720480	UCP3
ENSG00000175567	9.068907253	8.504964228	protein_coding	11	73685712	73694352	UCP2
ENSG00000175573	8.786518219	9.147142808	protein_coding	11	65684279	65686588	C11orf68
ENSG00000175575	7.206730419	7.436965343	protein_coding	11	73587744	73638790	PAAF1
ENSG00000175581	7.827020104	8.036341107	protein_coding	11	73498361	73576178	MRPL48
ENSG00000175582	9.832093067	9.746483542	protein_coding	11	73386938	73472182	RAB6A
ENSG00000175591	6.788216822	5.404622322	protein_coding	11	72929343	72947397	P2RY2
ENSG00000175592	10.8899802	10.10612397	protein_coding	11	65659490	65668044	FOSL1
ENSG00000175595	7.803191723	7.918899532	protein_coding	16	14014014	14046205	ERCC4
ENSG00000175600	3.744048221	4.101879561	protein_coding	7	40174575	40900362	C7orf10
ENSG00000175602	9.904181598	10.29580387	protein_coding	11	65657875	65659105	CCDC85B
ENSG00000175604	0.869158192	0.950786998	protein_coding	16	12180603	12184159	
ENSG00000175606	7.352496068	7.487839978	protein_coding	8	74884672	74895018	TMEM70
ENSG00000175611	4.903563137	5.564846512	processed_transc	9	98520892	98638259	LINC00476
ENSG00000175634	10.25293768	10.25085438	protein_coding	11	67195931	67202872	RPS6KB2
ENSG00000175643	8.137655191	8.116121984	protein_coding	16	11410636	11445619	RMI2
ENSG00000175662	8.29020451	8.833738534	protein_coding	17	17750139	17875784	TOM1L2
ENSG00000175691	6.095241663	6.009862691	protein_coding	19	2896754	2944969	ZNF77
ENSG00000175697	4.062237333	4.274079748	protein_coding	3	119883492	120003941	GPR156
ENSG00000175699	0.869158192	0.390640832	processed_transc	14	94463616	94478040	LINC00521
ENSG00000175701	6.509364082	6.624193585	lincRNA	2	110969106	111002997	LINC00116
ENSG00000175707	5.018397273	0.390640832	protein_coding	1	27276053	27286897	C1orf172
ENSG00000175711	7.514527001	7.799123587	protein_coding	17	80901672	81009686	B3GNTL1
ENSG00000175727	10.19968132	9.671994	protein_coding	12	122516628	122629266	MLXIP
ENSG00000175728	0.499066092	0.390640832	protein_coding	11	130542851	130587247	C11orf44
ENSG00000175730	4.230497448	4.342020395	pseudogene	20	31276723	31278816	BAK1P1
ENSG00000175741	0.499066092	2.039052734	pseudogene	7	39894093	39894655	RWDD4P2
ENSG00000175745	5.417620896	3.96493948	protein_coding	5	92919043	92930321	NR2F1

ENSG00000175749	0.869158192	0.390640832	pseudogene	5	102368080	102368735	
ENSG00000175756	9.636212778	9.982524041	protein_coding	1	1309110	1310875	AURKAIP1
ENSG00000175764	5.538004898	6.35497494	protein_coding	9	124584207	124855885	TTLL11
ENSG00000175768	6.462804234	6.592001111	protein_coding	9	37582643	37592639	TOMM5
ENSG00000175772	3.335220907	2.721932731	processed_transc	2	111132715	111142019	
ENSG00000175773	1.616589159	1.667587519	protein_coding	11	130184419	130273133	
ENSG00000175782	6.92698535	6.750400563	protein_coding	12	69139886	69187744	SLC35E3
ENSG00000175787	5.60078458	5.878761167	protein_coding	9	97021593	97063736	ZNF169
ENSG00000175792	10.36342886	10.10815782	protein_coding	3	127783625	127872757	RUVBL1
ENSG00000175793	11.782303	9.798293919	protein_coding	1	27189633	27190947	SFN
ENSG00000175806	6.220467583	6.544728831	protein_coding	8	9911778	10286401	MSRA
ENSG00000175820	0.499066092	1.518964905	protein_coding	13	103381801	103389159	CCDC168
ENSG00000175826	10.80554674	10.89235302	protein_coding	17	7146910	7155280	CTDNEP1
ENSG00000175832	9.123378905	8.497535398	protein_coding	17	41605212	41623762	ETV4
ENSG00000175841	1.16343121	0.390640832	pseudogene	3	101237711	101242731	FAM172BP
ENSG00000175854	7.842689815	8.066778281	protein_coding	9	131037658	131051269	SWI5
ENSG00000175866	8.889432943	8.647151213	protein_coding	17	79008947	79091232	BAIAP2
ENSG00000175873	5.331395195	5.583678991	lincRNA	7	2477398	2487485	
ENSG00000175874	1.960915222	0.697730409	protein_coding	2	101965209	102004057	CREG2
ENSG00000175877	1.616589159	1.353254395	protein_coding	7	73275489	73280223	WBSCR28
ENSG00000175879	1.16343121	1.667587519	protein_coding	2	176994422	176997423	HOXD8
ENSG00000175886	1.616589159	0.697730409	pseudogene	18	36914836	36915639	
ENSG00000175892	0	0.950786998	protein_coding	2	176992555	176994493	
ENSG00000175893	4.642199401	5.536128679	protein_coding	9	14615530	14693469	ZDHHC21
ENSG00000175894	5.124761037	4.566889334	protein_coding	21	45917775	46131495	TSPEAR
ENSG00000175895	6.469548347	6.338437404	protein_coding	8	96146032	96168912	PLEKHF2
ENSG00000175898	7.416718478	6.858739095	protein_coding	19	10332109	10341948	S1PR2
ENSG00000175899	0.499066092	0	protein_coding	12	9220260	9268825	A2M
ENSG00000175906	6.298293508	5.41517489	protein_coding	17	41476353	41478503	ARL4D
ENSG00000175911	4.322773689	2.579085888	protein_coding	17	78977135	78978932	
ENSG00000175920	4.593635215	2.144285137	protein_coding	4	3465033	3503200	DOK7
ENSG00000175928	0	0.390640832	protein_coding	3	3841121	3889387	LRRN1
ENSG00000175931	9.631710027	9.341545886	protein_coding	17	74385614	74449288	UBE2O
ENSG00000175938	7.384964606	7.281279771	protein_coding	16	30960387	30967782	ORAI3
ENSG00000175946	2.928422289	1.353254395	protein_coding	8	124657767	124665190	KLHL38
ENSG00000175970	9.001868107	8.741364159	protein_coding	12	121148238	121161443	UNC119B
ENSG00000175984	4.352265886	3.532712221	protein_coding	1	115125469	115213043	DENND2C
ENSG00000175985	1.16343121	1.667587519	protein_coding	14	69951409	69995215	PLEKHD1
ENSG00000176007	0.499066092	0.390640832	protein_coding	9	38526663	38528800	C9orf51
ENSG00000176009	1.407729925	1.802319292	protein_coding	11	8959119	8964580	ASCL3
ENSG00000176014	10.93239798	11.03345232	protein_coding	18	12308070	12326568	TUBB6
ENSG00000176018	1.16343121	1.353254395	protein_coding	5	89811428	89825401	LYSMD3
ENSG00000176022	8.727254249	8.672662438	protein_coding	1	1167629	1170421	B3GALT6
ENSG00000176024	6.509364082	6.750400563	protein_coding	19	52430688	52449045	ZNF613
ENSG00000176029	1.407729925	2.652276565	protein_coding	11	8941623	8954553	C11orf16
ENSG00000176040	0.499066092	0.697730409	protein_coding	3	111753690	111800116	TMPRSS7
ENSG00000176043	0.499066092	0	pseudogene	14	73396320	73396638	
ENSG00000176046	7.876068031	8.953504395	protein_coding	16	28548606	28550495	NUPR1
ENSG00000176054	1.960915222	2.242360793	pseudogene	21	30369934	30370354	RPL23P2
ENSG00000176055	1.616589159	1.518964905	protein_coding	5	89754020	89770585	MBLAC2
ENSG00000176058	7.776245808	7.994732806	protein_coding	9	140086069	140098645	TPRN
ENSG00000176076	0.869158192	1.353254395	protein_coding	X	108866929	108868393	KCNE1L
ENSG00000176087	10.14648923	9.633537922	protein_coding	5	139944041	139948688	SLC35A4
ENSG00000176092	4.097495944	2.721932731	protein_coding	1	26648350	26680621	AIM1L
ENSG00000176095	9.154055005	9.210611898	protein_coding	3	49761727	49823975	IP6K1
ENSG00000176101	9.064459359	9.317839931	protein_coding	9	140083099	140084822	SSNA1
ENSG00000176102	8.185546801	8.547552846	protein_coding	11	33098734	33183917	CSTF3
ENSG00000176105	9.953123981	9.460305991	protein_coding	18	721588	812327	YES1
ENSG00000176108	7.388527525	7.772696723	protein_coding	17	78965641	78973932	CHMP6
ENSG00000176124	6.121171528	5.878761167	protein_coding	13	50656307	51298199	DLEU1
ENSG00000176125	4.75690578	5.045726316	protein_coding	7	100486346	100487339	UFSP1
ENSG00000176134	0	0.390640832	protein_coding	9	69650263	69660358	
ENSG00000176142	8.366258896	8.579568843	protein_coding	3	119148347	119187677	TMEM39A

ENSG00000176148	7.834876234	8.235547225	protein_coding	11	33060963	33127489	TCP11L1
ENSG00000176153	4.942865586	3.027231696	protein_coding	14	65405870	65409531	GPX2
ENSG00000176155	9.065572619	8.979512278	protein_coding	17	80059346	80170689	CCDC57
ENSG00000176160	0	0.697730409	protein_coding	17	56497529	56565759	HSF5
ENSG00000176170	8.924215738	9.814826612	protein_coding	17	74372742	74383941	SPHK1
ENSG00000176171	8.472474444	9.740202333	protein_coding	10	133781578	133795435	BNIP3
ENSG00000176182	6.62865285	6.911866869	protein_coding	19	46393285	46405862	MYPOP
ENSG00000176183	0.499066092	1.353254395	pseudogene	5	97674448	97675536	
ENSG00000176208	7.746012174	7.405556541	protein_coding	17	29158988	29222295	ATAD5
ENSG00000176209	6.456028446	6.737844962	protein_coding	8	42396298	42408151	C8orf40
ENSG00000176222	4.883502971	5.466810074	protein_coding	19	44376519	44384291	ZNF404
ENSG00000176225	7.262161623	7.295628905	protein_coding	18	67671045	67872962	RTTN
ENSG00000176227	0.869158192	0	pseudogene	7	143268819	143271478	CTAGE15P
ENSG00000176236	1.407729925	1.667587519	protein_coding	10	15137384	15139318	C10orf111
ENSG00000176244	3.871730003	3.32456471	protein_coding	10	15119522	15130775	ACBD7
ENSG00000176248	9.614318105	9.379087693	protein_coding	9	140069236	140082989	ANAPC2
ENSG00000176261	7.345180517	7.507697912	protein_coding	1	33065773	33116504	ZBTB8OS
ENSG00000176268	0	0.390640832	pseudogene	13	41437735	41438038	CYCSP34
ENSG00000176273	7.323008624	7.616862529	protein_coding	10	95653730	95715819	SLC35G1
ENSG00000176274	4.352265886	4.17820932	protein_coding	X	103343898	103401708	MCART6
ENSG00000176289	0.499066092	0	pseudogene	X	148606540	148607582	IDSP1
ENSG00000176293	7.406211319	8.061749803	protein_coding	19	58570607	58597677	ZNF135
ENSG00000176302	0	0.697730409	protein_coding	11	118842417	118852001	FOXR1
ENSG00000176320	0.499066092	0	lincRNA	1	158167712	158173667	
ENSG00000176340	9.673929165	10.18300738	protein_coding	11	63742079	63744015	COX8A
ENSG00000176343	1.16343121	1.802319292	pseudogene	11	111759923	111760198	RPL37AP8
ENSG00000176349	1.799000381	2.912743273	protein_coding	7	1878222	1889567	
ENSG00000176354	3.698846687	3.570966319	pseudogene	3	37283303	37285686	
ENSG00000176358	3.077135474	2.788380093	protein_coding	17	47915671	47925379	TAC4
ENSG00000176371	6.35766608	6.223059835	protein_coding	15	85144217	85171027	ZSCAN2
ENSG00000176381	0.499066092	0.697730409	protein_coding	6	166719168	166721936	PRR18
ENSG00000176383	6.059927234	5.871116342	protein_coding	12	122688090	122693499	B3GNT4
ENSG00000176386	6.902274257	6.881748114	protein_coding	9	116018115	116037869	CDC26
ENSG00000176387	4.381167248	3.183544561	protein_coding	16	67464555	67471456	HSD11B2
ENSG00000176390	7.537174746	7.234379288	protein_coding	17	29109726	29151778	CRLF3
ENSG00000176393	10.32489868	10.00103046	protein_coding	1	201951500	201975275	RNPEP
ENSG00000176395	1.407729925	2.039052734	lincRNA	19	45005729	45033811	CEACAM20
ENSG00000176396	6.014532757	6.327306117	protein_coding	19	40028890	40030870	EID2
ENSG00000176399	3.871730003	3.87606762	protein_coding	9	22446840	22452472	DMRTA1
ENSG00000176401	3.830412367	4.17820932	protein_coding	19	40021630	40023494	EID2B
ENSG00000176402	0.499066092	1.16600992	protein_coding	7	99520892	99527243	GJC3
ENSG00000176406	0.869158192	1.16600992	protein_coding	8	104512976	105268322	RIMS2
ENSG00000176407	9.360447332	9.510993211	protein_coding	2	85198216	85282560	KCMF1
ENSG00000176410	6.733233509	6.795524906	protein_coding	7	73096601	73097783	DNAJC30
ENSG00000176422	7.427149666	7.803146634	protein_coding	12	56862301	56864763	SPRYD4
ENSG00000176428	3.211941663	2.501982735	protein_coding	7	73082155	73086442	VPS37D
ENSG00000176438	2.847891871	0.390640832	protein_coding	14	95883941	95942173	C14orf49
ENSG00000176444	9.14670993	9.602710123	protein_coding	1	155232659	155248282	CLK2
ENSG00000176454	9.332049062	8.639296529	protein_coding	15	34651106	34659479	LPCAT4
ENSG00000176463	8.278730502	8.065104068	protein_coding	15	92396925	92715665	SLCO3A1
ENSG00000176472	3.448790144	2.420525079	protein_coding	19	44029649	44040282	ZNF575
ENSG00000176473	7.123655438	6.95589788	protein_coding	14	100842755	100996640	WDR25
ENSG00000176476	7.169937448	7.632773539	protein_coding	16	28565236	28603111	CCDC101
ENSG00000176485	6.509364082	6.246885341	protein_coding	11	63340667	63384355	PLA2G16
ENSG00000176490	8.713115882	7.928139131	protein_coding	19	2714566	2721390	DIRAS1
ENSG00000176531	6.798966424	5.863430791	protein_coding	19	43979255	44009087	PHLDB3
ENSG00000176532	4.19837882	0	protein_coding	7	29603427	29606911	PRR15
ENSG00000176533	7.37062428	7.487839978	protein_coding	19	2511218	2702746	GNG7
ENSG00000176542	7.903720218	7.381542895	protein_coding	3	113367232	113415493	KIAA2018
ENSG00000176563	3.335220907	4.127774132	protein_coding	17	40950854	40963604	CNTD1
ENSG00000176593	7.1740722	7.029859489	protein_coding	19	58513429	58522600	
ENSG00000176595	4.19837882	5.863430791	protein_coding	8	1922044	1955102	KBTBD11
ENSG00000176597	9.047656547	8.637044457	protein_coding	3	182971032	183016292	B3GNT5

ENSG00000176601	0.499066092	0	protein_coding	2	135722061	135805038	YSK4
ENSG00000176619	12.60746111	12.37085179	protein_coding	19	2427636	2456994	LMNB2
ENSG00000176623	8.197783135	8.176294739	protein_coding	8	87480486	87526586	FAM82B
ENSG00000176624	8.994872754	8.883984273	protein_coding	18	48700920	48723690	MEX3C
ENSG00000176635	1.960915222	1.925536307	protein_coding	22	30476163	30606866	HORMAD2
ENSG00000176641	0	0.390640832	protein_coding	18	59475296	59560992	RNF152
ENSG00000176658	7.258272112	2.039052734	protein_coding	17	30819628	31203902	MYO1D
ENSG00000176678	5.485736881	4.961837817	protein_coding	16	86612115	86615303	FOXL1
ENSG00000176681	2.847891871	2.971122874	protein_coding	17	44370099	44415160	LRRC37A
ENSG00000176692	1.799000381	1.353254395	protein_coding	16	86600857	86602539	FOXC2
ENSG00000176697	5.977159882	5.988823159	protein_coding	11	27676440	27743605	BDNF
ENSG00000176700	6.835971536	6.775188634	pseudogene	15	85174682	85185695	SCAND2
ENSG00000176714	3.603959378	3.679994897	protein_coding	2	27848506	27851879	CCDC121
ENSG00000176715	8.674235392	8.925174415	protein_coding	16	89154783	89222254	ACSF3
ENSG00000176716	0	0.390640832	protein_coding	11	7749632	7750935	OR10AB1P
ENSG00000176720	8.977234519	9.117563863	protein_coding	2	242498136	242513546	BOK
ENSG00000176723	2.35979773	2.242360793	protein_coding	16	31445569	31454346	ZNF843
ENSG00000176731	6.887241849	6.642271819	protein_coding	8	86126311	86132650	C8orf59
ENSG00000176732	1.616589159	2.039052734	protein_coding	2	24338241	24346347	PFN4
ENSG00000176746	0	0.390640832	protein_coding	X	26210557	26213763	MAGEB6
ENSG00000176749	5.107569429	4.65855978	protein_coding	17	30813637	30818274	CDK5R1
ENSG00000176753	2.671945279	2.242360793	protein_coding	15	40542865	40545170	C15orf56
ENSG00000176761	0.869158192	1.353254395	pseudogene	19	44971858	44977444	
ENSG00000176783	8.023133844	8.256237404	protein_coding	5	178977559	179037027	RUFY1
ENSG00000176788	12.13946777	11.82145555	protein_coding	5	17217669	17276943	BASP1
ENSG00000176809	5.512107582	6.365895578	protein_coding	17	62850430	62895219	LRRC37A3
ENSG00000176824	0	0.390640832	pseudogene	2	145218679	145221645	
ENSG00000176826	3.448790144	3.133298822	pseudogene	7	55748767	55780945	FKBP9L
ENSG00000176834	7.757079669	6.465715653	protein_coding	12	118501398	118573831	VSIG10
ENSG00000176840	0.499066092	0.390640832	lincRNA	19	4769117	4772545	MIR7-3HG
ENSG00000176842	6.393570237	6.664555628	protein_coding	16	54964774	54968397	IRX5
ENSG00000176845	8.215945216	8.024328814	protein_coding	17	81037567	81052585	METRNL
ENSG00000176853	9.405983977	9.355310748	protein_coding	8	124780696	124827692	FAM91A1
ENSG00000176855	2.35979773	1.667587519	pseudogene	1	182928154	182929540	KRT18P28
ENSG00000176857	0.869158192	0.390640832	pseudogene	5	108387013	108388158	
ENSG00000176871	9.819610198	9.381107599	protein_coding	12	118470712	118500235	WSB2
ENSG00000176884	6.407685389	4.990346085	protein_coding	9	140032842	140063207	GRIN1
ENSG00000176890	9.58666794	9.742822827	protein_coding	18	657604	673578	TYMS
ENSG00000176894	4.75690578	4.888000647	protein_coding	12	133264192	133297276	PXMP2
ENSG00000176896	3.78787645	5.045726316	protein_coding	X	13671225	13700083	TCEANC
ENSG00000176903	9.334821842	9.516516751	protein_coding	14	74178494	74181128	PNMA1
ENSG00000176907	6.646617938	7.050313162	protein_coding	8	40010989	40012821	C8orf4
ENSG00000176909	4.131913373	4.794098771	protein_coding	19	49216255	49222978	MAMSTR
ENSG00000176912	3.146112541	3.679994897	protein_coding	18	649621	658340	C18orf56
ENSG00000176915	9.644431499	9.870045082	protein_coding	12	133302254	133338474	ANKLE2
ENSG00000176919	2.238690726	2.788380093	protein_coding	9	139839698	139841426	C8G
ENSG00000176920	5.207774174	3.748356452	protein_coding	19	49199228	49209207	FUT2
ENSG00000176927	2.238690726	2.579085888	protein_coding	17	28256218	28435470	EFCAB5
ENSG00000176928	3.554067925	4.075511708	protein_coding	5	74323289	74326724	GCNT4
ENSG00000176933	1.799000381	2.039052734	pseudogene	6	28185421	28186655	TOB2P1
ENSG00000176936	2.35979773	2.652276565	antisense	19	1385158	1389650	
ENSG00000176945	3.448790144	3.453019579	protein_coding	3	195343316	195467994	MUC20
ENSG00000176946	10.08083775	9.616492755	protein_coding	2	242523820	242576864	THAP4
ENSG00000176953	9.373990307	9.654843329	protein_coding	16	28962128	28978418	NFATC2IP
ENSG00000176956	0	0.390640832	protein_coding	8	144239331	144242128	LY6H
ENSG00000176971	0	0.390640832	protein_coding	11	27015628	27018630	FIBIN
ENSG00000176973	4.842525946	5.236305879	protein_coding	11	65339820	65341669	FAM89B
ENSG00000176974	9.548160668	9.729143979	protein_coding	17	18231187	18266832	SHMT1
ENSG00000176978	10.34704177	10.63742557	protein_coding	9	140004994	140009629	DPP7
ENSG00000176986	10.8899802	10.3739526	protein_coding	10	75504120	75531919	SEC24C
ENSG00000176994	8.094677541	8.520929986	protein_coding	17	18218716	18226517	SMCR8
ENSG00000176998	2.57521082	3.183544561	pseudogene	6	29758816	29760850	HCG4
ENSG00000177000	6.541724534	5.683080141	protein_coding	1	11845780	11866977	MTHFR

ENSG00000177025	5.124761037	5.259915406	protein_coding	19	58469807	58485904	C19orf18
ENSG00000177030	8.513842764	8.624594823	protein_coding	11	644233	706715	DEAF1
ENSG00000177034	7.07583934	6.750400563	protein_coding	5	79275584	79287082	MTX3
ENSG00000177042	5.806547963	6.246885341	protein_coding	11	695616	705028	TMEM80
ENSG00000177045	7.214780754	7.743685008	protein_coding	19	46268044	46272141	SIX5
ENSG00000177051	8.309127368	8.043160634	protein_coding	19	46213887	46234151	FBXO46
ENSG00000177054	8.13977083	8.280968702	protein_coding	11	19138646	19197969	ZDHHC13
ENSG00000177058	7.110771062	7.204272857	protein_coding	5	54921673	55069022	SLC38A9
ENSG00000177076	2.471521042	2.039052734	protein_coding	9	19408925	19452018	ACER2
ENSG00000177082	7.834876234	8.109639521	protein_coding	15	85185999	85197574	WDR73
ENSG00000177084	10.08688122	10.11302738	protein_coding	12	133200345	133413387	POLE
ENSG00000177096	7.206730419	6.952279523	protein_coding	22	42470255	42475445	FAM109B
ENSG00000177098	2.471521042	3.570966319	protein_coding	11	118004092	118023603	SCN4B
ENSG00000177103	0.499066092	0.697730409	protein_coding	11	117298489	117688240	DSCAML1
ENSG00000177105	9.462857538	9.454562557	protein_coding	11	3848208	3862213	RHOG
ENSG00000177106	9.702835781	8.679244009	protein_coding	11	694438	727727	EPS8L2
ENSG00000177108	0.499066092	0	protein_coding	14	77597613	77609077	ZDHHC22
ENSG00000177112	0.869158192	0.697730409	antisense	11	10562819	10621479	MRVI1-AS1
ENSG00000177119	10.17203891	10.51031183	protein_coding	12	45609770	45834187	ANO6
ENSG00000177125	7.202688343	7.070480904	protein_coding	9	129622944	129648157	ZBTB34
ENSG00000177133	2.238690726	1.353254395	processed_transc	1	2976179	2985001	
ENSG00000177138	2.471521042	1.925536307	protein_coding	X	8992273	9132680	FAM9B
ENSG00000177144	0	0.390640832	pseudogene	1	145139025	145139569	
ENSG00000177150	8.526833966	8.739266022	protein_coding	18	13663346	13726591	FAM210A
ENSG00000177156	10.36975169	10.22559683	protein_coding	11	747329	765024	TALDO1
ENSG00000177169	10.29198498	10.2534295	protein_coding	12	132379196	132407695	ULK1
ENSG00000177173	2.671945279	2.420525079	pseudogene	1	117075558	117076714	NAP1L4P1
ENSG00000177182	0.499066092	0	protein_coding	8	61969717	62414204	CLVS1
ENSG00000177189	10.714799	10.41297634	protein_coding	X	20168029	20285523	RPS6KA3
ENSG00000177191	2.10647801	1.925536307	protein_coding	19	41931265	41934635	B3GNT8
ENSG00000177192	9.287898339	8.832754911	protein_coding	12	132413745	132428406	PUS1
ENSG00000177197	0.499066092	0.390640832	pseudogene	13	48902220	48902700	PCNPP5
ENSG00000177200	8.768856552	9.356680039	protein_coding	16	53088945	53363062	CHD9
ENSG00000177202	0.499066092	0	protein_coding	19	49110000	49110970	SPACA4
ENSG00000177212	0	1.16600992	protein_coding	1	248436073	248437138	OR2T33
ENSG00000177225	8.861502869	9.049765501	protein_coding	11	767220	777501	PDDC1
ENSG00000177236	4.322773689	4.841813558	pseudogene	11	780117	780755	
ENSG00000177238	0.499066092	0	protein_coding	16	31225342	31236510	TRIM72
ENSG00000177239	10.65429932	10.95652219	protein_coding	9	139981379	140003635	MAN1B1
ENSG00000177261	0.499066092	0	pseudogene	2	215562451	215562788	ENSAP3
ENSG00000177272	0	0.950786998	protein_coding	1	111214310	111217655	KCNA3
ENSG00000177283	3.830412367	3.608232228	protein_coding	10	35927177	35930362	FZD8
ENSG00000177291	0	0.697730409	protein_coding	10	35894338	35897863	GJD4
ENSG00000177294	2.57521082	3.453019579	protein_coding	17	6679551	6690965	FBXO39
ENSG00000177302	9.571858303	9.989160763	protein_coding	17	18174742	18218288	TOP3A
ENSG00000177303	8.016241092	7.472765069	protein_coding	17	73496343	73511627	CASKIN2
ENSG00000177311	9.584339659	9.894290904	protein_coding	3	141043055	141168634	ZBTB38
ENSG00000177335	0.499066092	3.081239798	protein_coding	8	144120626	144141359	C8orf31
ENSG00000177337	6.041940478	6.520498852	protein_coding	18	3594112	3598350	
ENSG00000177340	2.35979773	2.144285137	protein_coding	12	31477250	31478879	
ENSG00000177350	2.471521042	3.027231696	pseudogene	14	56232895	56234435	RPL13AP3
ENSG00000177352	7.413224588	7.439552137	protein_coding	3	49199968	49203754	CCDC71
ENSG00000177359	3.274897671	3.027231696	pseudogene	12	31264586	31359088	
ENSG00000177363	1.799000381	2.788380093	protein_coding	11	62453874	62457371	LRRN4CL
ENSG00000177370	8.560389515	9.241329169	protein_coding	17	900357	905388	TIMM22
ENSG00000177374	6.917151646	6.941369681	protein_coding	17	1958393	1962980	HIC1
ENSG00000177380	8.074911866	7.164171052	protein_coding	19	49622663	49654278	PPFIA3
ENSG00000177383	8.357195795	8.49131535	protein_coding	3	184428156	184429836	MAGEF1
ENSG00000177398	1.799000381	1.667587519	protein_coding	21	43483068	43563105	UMODL1
ENSG00000177406	4.352265886	4.342020395	antisense	12	740057	772872	
ENSG00000177409	7.374222747	7.994732806	protein_coding	7	92759368	92777682	SAMD9L
ENSG00000177410	9.786910053	10.05853621	processed_transc	20	47894715	47905797	ZNFX1-AS1
ENSG00000177418	0.499066092	0.390640832	pseudogene	7	66021832	66022349	

ENSG00000177425	9.51676008	9.404466559	protein_coding	12	79968759	80084877	PAWR
ENSG00000177426	9.064459359	8.990138507	protein_coding	18	3411606	3459976	TGIF1
ENSG00000177427	7.67484765	8.231074746	protein_coding	17	18163869	18169095	SMCR7
ENSG00000177432	4.903563137	3.87606762	protein_coding	4	89617066	89619386	NAP1L5
ENSG00000177452	1.960915222	0.950786998	pseudogene	1	64254392	64254975	
ENSG00000177453	5.175139844	5.393992	protein_coding	5	43192173	43280952	
ENSG00000177455	4.062237333	4.226940739	protein_coding	16	28943260	28950667	CD19
ENSG00000177459	1.616589159	1.667587519	protein_coding	8	99076539	99105838	C8orf47
ENSG00000177462	0	1.16600992	protein_coding	1	248084320	248085258	OR2T8
ENSG00000177463	7.110771062	6.97385478	protein_coding	3	14989091	15095107	NR2C2
ENSG00000177464	0.499066092	0.390640832	protein_coding	19	46093025	46105466	GPR4
ENSG00000177465	5.986594255	5.66550919	protein_coding	14	74058410	74063200	ACOT4
ENSG00000177469	12.21088618	11.83133857	protein_coding	17	40554468	40575338	PTRF
ENSG00000177479	9.601327095	9.400488931	protein_coding	3	48956254	49023815	ARIH2
ENSG00000177483	2.928422289	2.851901313	protein_coding	2	238707032	238751451	RBM44
ENSG00000177485	9.016909338	9.209096633	protein_coding	X	119384607	119392253	ZBTB33
ENSG00000177508	7.269909329	7.848622884	protein_coding	16	54317216	54320675	IRX3
ENSG00000177542	9.757590402	9.713197829	protein_coding	11	790475	798316	SLC25A22
ENSG00000177548	7.63640659	8.458531597	protein_coding	16	28915742	28947847	RABEP2
ENSG00000177556	8.591637531	9.090661058	protein_coding	5	151121877	151152093	ATOX1
ENSG00000177565	10.52995925	9.999278141	protein_coding	3	176737143	176915261	TBL1XR1
ENSG00000177570	3.274897671	3.279072565	protein_coding	8	119201698	119634234	SAMD12
ENSG00000177575	1.16343121	0.697730409	protein_coding	12	7623409	7656489	CD163
ENSG00000177576	6.804311337	7.628245414	protein_coding	18	47008030	47013601	C18orf32
ENSG00000177595	8.048129203	7.772696723	protein_coding	11	799180	809753	PIDD
ENSG00000177599	4.19837882	4.508954154	protein_coding	19	11908482	11920437	ZNF491
ENSG00000177600	12.29028863	12.3421068	protein_coding	11	809647	812880	RPLP2
ENSG00000177602	8.327805238	8.614328238	protein_coding	17	3627211	3630067	GSG2
ENSG00000177606	11.15921124	10.09262819	protein_coding	1	59246465	59249785	JUN
ENSG00000177613	8.528449672	8.270836518	protein_coding	10	53455247	53459355	CSTF2T
ENSG00000177614	3.603959378	5.175534283	protein_coding	1	230457392	230561475	PGBD5
ENSG00000177627	1.960915222	2.334191469	protein_coding	12	48876286	48890295	C12orf54
ENSG00000177628	9.568721018	10.35557934	protein_coding	1	155204239	155214653	GBA
ENSG00000177640	4.689181911	4.202780776	non_coding	10	119805790	119969663	CASC2
ENSG00000177646	8.282565319	8.338926518	protein_coding	3	128598439	128634910	ACAD9
ENSG00000177663	7.845284967	7.827052132	protein_coding	22	17565844	17596583	IL17RA
ENSG00000177666	9.269659458	9.333918856	protein_coding	11	818902	825573	PNPLA2
ENSG00000177669	1.616589159	1.16600992	protein_coding	8	29989340	30002202	MBOAT4
ENSG00000177674	8.631273684	8.743459249	protein_coding	1	11796141	11814859	AGTRAP
ENSG00000177675	6.592037289	7.462626902	protein_coding	12	7499281	7632493	CD163L1
ENSG00000177679	4.689181911	3.081239798	protein_coding	7	75831216	75916605	SRRM3
ENSG00000177683	7.847875458	7.487839978	protein_coding	7	108194987	108210194	THAP5
ENSG00000177685	5.525114348	4.841813558	protein_coding	11	826144	831991	EFCAB4A
ENSG00000177688	0.499066092	0.390640832	protein_coding	6	149721495	149722177	SUMO4
ENSG00000177692	3.077135474	4.101879561	protein_coding	21	34860497	34864027	DNAJC28
ENSG00000177694	2.10647801	0.697730409	protein_coding	3	174156363	175523428	NAALADL2
ENSG00000177697	11.11634334	11.26546849	protein_coding	11	832843	839831	CD151
ENSG00000177699	0.869158192	0.390640832	antisense	15	79271231	79278268	
ENSG00000177700	8.779750834	9.351881826	protein_coding	11	837356	842545	POLR2L
ENSG00000177706	10.48615002	9.326251289	protein_coding	7	192969	300711	FAM20C
ENSG00000177707	6.592037289	7.605389119	protein_coding	3	110788918	110994410	PVRL3
ENSG00000177710	0.869158192	0.697730409	protein_coding	8	11188397	11189717	SLC35G5
ENSG00000177721	5.141750194	5.200150955	protein_coding	5	43039335	43043272	C5orf39
ENSG00000177725	0.499066092	2.242360793	pseudogene	8	22026851	22029223	
ENSG00000177728	9.5183871	9.464122284	protein_coding	17	73452664	73496530	KIAA0195
ENSG00000177731	12.27749479	12.66458408	protein_coding	17	18148150	18162056	FLII
ENSG00000177732	10.24214716	9.802319276	protein_coding	20	306207	310865	SOX12
ENSG00000177733	11.04645645	11.04305224	protein_coding	5	137087075	137090039	HNRNPA0
ENSG00000177736	0.499066092	0	pseudogene	9	127730576	127731223	
ENSG00000177738	6.335686705	6.002883551	lincRNA	5	43014516	43067521	
ENSG00000177757	1.16343121	0.950786998	lincRNA	1	752751	755214	FAM87B
ENSG00000177764	4.999881834	4.976162367	protein_coding	20	277737	280965	ZCCHC3
ENSG00000177776	0	0.390640832	pseudogene	14	74844521	74845121	RPS2P2

ENSG00000177788	3.554067925	4.075511708	processed_transc	1	229394028	229406775	
ENSG00000177791	0.499066092	0.697730409	protein_coding	10	75391412	75401515	MYOZ1
ENSG00000177800	0	0.697730409	protein_coding	1	229385383	229387557	TMEM78
ENSG00000177803	0.869158192	0.390640832	pseudogene	4	164853678	164854334	
ENSG00000177807	0.869158192	0.390640832	protein_coding	1	160007257	160040038	KCNJ10
ENSG00000177822	0	1.16600992	processed_transc	4	182795591	183066402	
ENSG00000177830	10.00916559	9.881981209	protein_coding	11	867357	915058	CHID1
ENSG00000177839	5.360713447	5.840125386	pseudogene	5	140566893	140571111	PCDHB9
ENSG00000177842	2.471521042	2.851901313	protein_coding	3	40547483	40560227	ZNF620
ENSG00000177853	7.743231983	8.001751392	processed_transc	10	97889472	97965044	ZNF518A
ENSG00000177854	7.524276744	7.307006333	protein_coding	X	153237778	153248646	TMEM187
ENSG00000177855	1.16343121	1.16600992	pseudogene	2	184472170	184472847	
ENSG00000177868	5.072559152	5.555337277	protein_coding	1	43272723	43282954	CCDC23
ENSG00000177873	4.026095388	4.406904905	protein_coding	3	40518604	40531727	ZNF619
ENSG00000177879	10.80354643	9.486185012	protein_coding	5	115177178	115249778	AP3S1
ENSG00000177885	9.812996626	9.439780161	protein_coding	17	73314157	73401790	GRB2
ENSG00000177888	5.613019223	5.824376633	protein_coding	1	197127572	197169672	ZBTB41
ENSG00000177889	9.370391259	9.442361917	protein_coding	12	93799449	93836038	UBE2N
ENSG00000177910	0	0.390640832	pseudogene	9	90744220	90749900	FAM75C2
ENSG00000177917	8.074911866	7.883239271	protein_coding	2	153574407	153617688	ARL6IP6
ENSG00000177932	6.86695184	7.073814954	protein_coding	5	178487416	178510538	ZNF354C
ENSG00000177943	6.897280837	7.080460027	protein_coding	9	139745395	139755249	MAMDC4
ENSG00000177946	6.204385613	5.742946029	protein_coding	16	90036206	90038942	CENPBD1
ENSG00000177947	0.869158192	1.16600992	protein_coding	11	196738	200261	ODF3
ENSG00000177951	8.340737341	8.381252713	protein_coding	11	167784	207428	BET1L
ENSG00000177954	9.286944125	10.01280331	protein_coding	1	153963235	153964626	RPS27
ENSG00000177963	10.24657122	10.45832528	protein_coding	11	207511	215113	RIC8A
ENSG00000177971	8.635777795	9.386480232	protein_coding	15	75931426	75941047	IMP3
ENSG00000177981	7.153278907	7.126137778	protein_coding	12	48541571	48574996	ASB8
ENSG00000177984	0	0.390640832	protein_coding	9	139654086	139660707	LCN15
ENSG00000177989	6.400645076	7.287036562	protein_coding	22	50968139	50971009	ODF3B
ENSG00000177990	4.261916566	3.081239798	protein_coding	12	63952693	64062719	DPY19L2
ENSG00000177993	1.407729925	2.501982735	processed_transc	22	29420987	29427464	ZNRF3-AS1
ENSG00000178026	4.999881834	3.935919592	protein_coding	22	24981588	24989175	FAM211B
ENSG00000178028	7.254372086	7.69255166	protein_coding	1	44679127	44686353	DMAP1
ENSG00000178031	1.16343121	0.390640832	protein_coding	9	18473892	18910948	ADAMTSL1
ENSG00000178033	1.407729925	3.532712221	protein_coding	6	116832809	116845955	FAM26E
ENSG00000178035	11.54352216	11.47871408	protein_coding	3	49061672	49066841	IMPDH2
ENSG00000178038	9.110492049	8.947155755	protein_coding	3	46710487	46735194	ALS2CL
ENSG00000178053	7.136425764	7.711939664	protein_coding	3	158288952	158325041	MLF1
ENSG00000178055	0	0.390640832	protein_coding	3	46871032	46875585	PRSS42
ENSG00000178057	8.239811039	8.44700755	protein_coding	3	49057892	49060905	NDUFAF3
ENSG00000178074	7.498129565	7.289906366	protein_coding	2	200775979	200820658	C2orf69
ENSG00000178075	4.981125677	5.393992	protein_coding	3	113547029	113666021	GRAMD1C
ENSG00000178078	8.510576602	8.848412964	protein_coding	19	4324041	4338847	STAP2
ENSG00000178081	1.16343121	0.950786998	pseudogene	15	30395923	30423960	ULK4P3
ENSG00000178082	1.16343121	0.950786998	pseudogene	17	27530441	27531492	
ENSG00000178093	5.718698586	6.104183162	protein_coding	19	19623230	19626838	TSSK6
ENSG00000178096	7.345180517	7.929979971	protein_coding	1	149858486	149872351	BOLA1
ENSG00000178104	9.560847829	10.22372209	protein_coding	1	144836157	145076186	PDE4DIP
ENSG00000178105	9.072234198	9.09886653	protein_coding	11	108535752	108811657	DDX10
ENSG00000178110	0	0.390640832	protein_coding	3	156544096	156763918	LEKR1
ENSG00000178115	0	0.390640832	protein_coding	15	30844183	30854704	
ENSG00000178125	0	0.697730409	protein_coding	8	67876334	67968839	PPP1R42
ENSG00000178127	9.139327269	9.270679862	protein_coding	18	9102628	9134343	NDUFV2
ENSG00000178149	7.575190993	7.454976247	protein_coding	3	49052921	49059726	DALRD3
ENSG00000178150	7.692250012	7.237355663	protein_coding	19	48774654	48790863	ZNF114
ENSG00000178162	0.499066092	0	pseudogene	2	131174328	131188936	
ENSG00000178163	7.478203418	4.976162367	protein_coding	4	10441498	10459034	ZNF518B
ENSG00000178171	0.869158192	0	protein_coding	2	131513008	131525707	FAM123C
ENSG00000178177	6.428602508	6.470782801	protein_coding	4	17844839	18023499	LCORL
ENSG00000178184	5.207774174	5.085909553	protein_coding	18	77915115	78005429	PARD6G
ENSG00000178187	3.950976309	4.127774132	protein_coding	5	178368192	178393434	ZNF454

ENSG00000178188	8.614637248	8.91219558	protein_coding	16	28857921	28885526	SH2B1
ENSG00000178199	3.004694206	2.420525079	protein_coding	6	149768794	149806197	ZC3H12D
ENSG00000178201	3.211941663	3.845183986	protein_coding	19	57966542	57967854	VN1R1
ENSG00000178202	8.398422578	8.280968702	protein_coding	11	108342832	108369159	KDELC2
ENSG00000178209	13.42631403	12.82682452	protein_coding	8	144989321	145050902	PLEC
ENSG00000178217	0.869158192	1.16600992	protein_coding	10	82297658	82406316	SH2D4B
ENSG00000178222	7.908691438	7.281279771	protein_coding	4	1050038	1107350	RNF212
ENSG00000178226	2.928422289	3.813624741	protein_coding	16	31150246	31161415	PRSS36
ENSG00000178229	6.676071387	6.563824016	protein_coding	19	57831865	57842144	ZNF543
ENSG00000178233	3.78787645	4.363974406	protein_coding	6	44238203	44346694	TMEM151B
ENSG00000178234	8.911889356	8.911264037	protein_coding	7	151722759	151819425	GALNT11
ENSG00000178235	0.499066092	0	protein_coding	13	84451344	84456528	SLITRK1
ENSG00000178252	10.52146546	10.60207072	protein_coding	3	49044495	49053386	WDR6
ENSG00000178287	0	0.390640832	protein_coding	8	7705398	7726389	SPAG11A
ENSG00000178295	8.150302734	8.005247922	protein_coding	2	17935125	17966632	GEN1
ENSG00000178297	6.522395495	6.633261019	protein_coding	19	2389769	2426086	TMPRSS9
ENSG00000178301	4.491309013	4.94736961	protein_coding	11	77300436	77321400	AQP11
ENSG00000178307	8.247679379	8.812940295	protein_coding	17	21101263	21117908	TMEM11
ENSG00000178338	6.456028446	6.338437404	protein_coding	5	178286954	178315123	ZNF354B
ENSG00000178342	0	1.353254395	protein_coding	18	77623668	77659816	KCNG2
ENSG00000178343	1.407729925	0	protein_coding	4	42399856	42404504	SHISA3
ENSG00000178381	7.234712227	7.362585296	protein_coding	7	1191707	1200395	ZFAND2A
ENSG00000178385	5.175139844	4.676215056	protein_coding	2	208693027	208890284	PLEKHM3
ENSG00000178386	5.795854912	5.938503778	protein_coding	19	44556164	44572142	ZNF223
ENSG00000178395	1.407729925	0.697730409	protein_coding	1	223566715	223568812	C1orf65
ENSG00000178397	8.173205795	8.133800013	protein_coding	7	6369040	6388612	C7orf70
ENSG00000178401	6.372135143	5.35066967	protein_coding	12	49740700	49751309	DNAJC22
ENSG00000178403	0.499066092	0	protein_coding	4	113434672	113437328	NEUROG2
ENSG00000178404	0.499066092	1.518964905	protein_coding	17	76866992	76899297	
ENSG00000178409	7.621344476	7.04352744	protein_coding	6	107386386	107436473	BEND3
ENSG00000178412	0.499066092	0.950786998	protein_coding	18	77398938	77439745	
ENSG00000178425	7.461385447	7.32390599	protein_coding	6	116421999	116566855	NT5DC1
ENSG00000178429	1.799000381	1.925536307	pseudogene	10	86320199	86321019	RPS3AP5
ENSG00000178430	3.830412367	2.851901313	pseudogene	16	3725411	3727060	
ENSG00000178440	2.238690726	1.353254395	processed_transc	10	51732523	51741805	
ENSG00000178445	1.407729925	0.697730409	protein_coding	9	6532464	6645650	GLDC
ENSG00000178449	7.153278907	7.960920138	protein_coding	12	50505762	50559203	C12orf62
ENSG00000178458	0.499066092	0	pseudogene	4	140619298	140619708	H3F3AP6
ENSG00000178460	3.448790144	3.411459265	protein_coding	8	67782984	67834283	C8orf45
ENSG00000178462	4.292665995	3.027231696	protein_coding	10	5435061	5446793	TUBAL3
ENSG00000178464	4.593635215	4.585695337	pseudogene	19	12754089	12754733	
ENSG00000178467	7.983632192	7.714077892	protein_coding	3	49027319	49044587	P4HTM
ENSG00000178498	7.434062125	7.060432274	protein_coding	12	57998405	58003587	DTX3
ENSG00000178502	5.346128795	5.691786012	protein_coding	17	40009797	40021684	KLHL11
ENSG00000178531	7.89873181	8.683615101	protein_coding	19	7989382	7991051	CTXN1
ENSG00000178537	6.652556929	6.847095496	protein_coding	3	48894369	48936426	SLC25A20
ENSG00000178556	0	0.390640832	pseudogene	X	30635571	30635810	CKS1BP6
ENSG00000178562	0	0.390640832	protein_coding	2	204571198	204602557	CD28
ENSG00000178567	7.165790811	7.852510423	protein_coding	3	37027357	37034795	EPM2AIP1
ENSG00000178568	0	0.390640832	protein_coding	2	212240446	213403565	ERBB4
ENSG00000178573	0.499066092	3.748356452	protein_coding	16	79619740	79634611	MAF
ENSG00000178585	7.746012174	6.754561598	protein_coding	1	9908334	9970394	CTNNBIP1
ENSG00000178597	1.799000381	1.925536307	protein_coding	4	7432022	7436700	PSAPL1
ENSG00000178605	8.650690744	9.065760854	protein_coding	X	220013	230886	GTPBP6
ENSG00000178607	6.979904289	6.668971382	protein_coding	17	62120390	62207502	ERN1
ENSG00000178623	4.491309013	4.794098771	protein_coding	2	241544848	241570676	GPR35
ENSG00000178631	1.799000381	2.242360793	pseudogene	3	139212651	139213779	ACTG1P1
ENSG00000178636	0.869158192	1.16600992	protein_coding	4	120113928	120133799	
ENSG00000178642	2.471521042	2.420525079	pseudogene	1	2316811	2319922	
ENSG00000178654	2.238690726	0.950786998	pseudogene	9	7597823	7598371	
ENSG00000178660	0	0.390640832	pseudogene	3	94225610	94226464	ARMC10P1
ENSG00000178665	4.923348194	4.794098771	protein_coding	7	55955169	56009918	ZNF713
ENSG00000178685	9.194306789	9.66482659	protein_coding	8	145051321	145086940	PARP10

ENSG00000178690	0	0.390640832	protein_coding	18	52258390	52266724	C18orf26
ENSG00000178691	9.43986141	9.296594447	protein_coding	17	30264056	30328064	SUZ12
ENSG00000178694	6.462804234	6.803579823	protein_coding	3	93781760	93847389	NSUN3
ENSG00000178695	9.304024157	8.729786462	protein_coding	13	77454312	77460540	KCTD12
ENSG00000178700	5.806547963	6.235021771	protein_coding	3	93766680	93782233	DHFRL1
ENSG00000178715	5.929039784	6.387491945	pseudogene	1	16154724	16155167	
ENSG00000178718	8.977234519	8.874456566	protein_coding	15	75246757	75249805	RPP25
ENSG00000178719	10.46816273	10.70321774	protein_coding	8	145064226	145067583	GRINA
ENSG00000178723	0	0.390640832	pseudogene	9	34917172	34918287	GLULP4
ENSG00000178726	4.437288965	4.622587519	protein_coding	20	23026270	23030378	THBD
ENSG00000178732	0.499066092	0.697730409	protein_coding	3	194115550	194119995	GP5
ENSG00000178741	9.832747078	9.844409099	protein_coding	15	75212132	75230509	COX5A
ENSG00000178750	0.499066092	1.16600992	protein_coding	3	93733213	93747454	STX19
ENSG00000178752	3.393122761	3.232099092	protein_coding	2	239067623	239077541	FAM132B
ENSG00000178761	7.581430769	8.075120324	protein_coding	15	75192328	75199462	C15orf17
ENSG00000178764	8.375265417	8.543951256	protein_coding	8	123793633	123986750	ZHX2
ENSG00000178772	0	0.390640832	protein_coding	3	194060494	194072057	CPN2
ENSG00000178773	9.550548025	9.767740319	protein_coding	16	89642176	89663654	CPNE7
ENSG00000178776	0	5.871116342	protein_coding	5	147260289	147286101	C5orf46
ENSG00000178795	0.499066092	0	protein_coding	11	76927603	77012732	GDPD4
ENSG00000178796	0.869158192	0.390640832	protein_coding	1	151682909	151702281	RIIAD1
ENSG00000178802	8.898207576	8.979512278	protein_coding	15	75182346	75191798	MPI
ENSG00000178803	4.292665995	4.021284656	protein_coding	22	24825178	24891042	C22orf45
ENSG00000178809	5.986594255	5.361622823	protein_coding	7	75024337	75040279	TRIM73
ENSG00000178814	8.666918882	9.213637661	processed_transc	8	145106167	145118735	OPLAH
ENSG00000178821	5.072559152	4.693656882	protein_coding	1	1849029	1850712	TMEM52
ENSG00000178826	1.616589159	1.925536307	protein_coding	7	142977050	142985141	TMEM139
ENSG00000178852	4.352265886	4.94736961	protein_coding	17	45400656	45518678	C17orf57
ENSG00000178860	3.146112541	3.081239798	protein_coding	8	72753784	72756703	MSC
ENSG00000178863	3.004694206	3.493416095	pseudogene	19	33793976	33795656	
ENSG00000178878	6.212449006	6.210997888	protein_coding	12	12878865	12944400	APOLD1
ENSG00000178882	3.393122761	2.579085888	protein_coding	12	124773710	124800570	FAM101A
ENSG00000178896	7.311793575	7.754113435	protein_coding	8	145133529	145135550	EXOSC4
ENSG00000178904	6.882196036	7.487839978	protein_coding	19	32896655	32976795	DPY19L3
ENSG00000178913	9.963313735	10.16005582	protein_coding	5	140698057	140700330	TAF7
ENSG00000178917	4.734682531	4.693656882	protein_coding	3	44540462	44552128	ZNF852
ENSG00000178919	5.684325135	4.94736961	protein_coding	9	100615536	100618986	FOXE1
ENSG00000178921	11.27490064	11.31667125	protein_coding	17	8152604	8173809	PFAS
ENSG00000178922	7.273767634	7.666288907	protein_coding	1	43916824	43919660	HYI
ENSG00000178927	8.249639777	8.303864205	protein_coding	17	80400520	80408694	C17orf62
ENSG00000178932	3.911897206	3.133298822	protein_coding	17	73814896	73818071	
ENSG00000178935	6.095241663	6.204928885	protein_coding	19	58318450	58326281	ZNF552
ENSG00000178947	1.616589159	1.925536307	non_coding	X	134555868	134559682	LINC00086
ENSG00000178950	10.14491017	9.942049496	protein_coding	4	843064	926161	GAK
ENSG00000178951	10.06478254	9.684588223	protein_coding	19	4045217	4066816	ZBTB7A
ENSG00000178952	12.06933229	12.16087443	protein_coding	16	28853732	28857729	TUFM
ENSG00000178965	0.499066092	0	protein_coding	1	75033795	75139422	C1orf173
ENSG00000178966	8.05038018	8.42106413	protein_coding	9	86595626	86618985	RMI1
ENSG00000178971	9.022652927	9.734420434	protein_coding	17	8128140	8151413	CTC1
ENSG00000178972	0.499066092	1.518964905	pseudogene	4	144256418	144258970	
ENSG00000178974	8.032273249	8.37044814	protein_coding	14	55738021	55828636	FBXO34
ENSG00000178977	2.57521082	3.935919592	antisense	17	8123960	8127361	LINC00324
ENSG00000178980	8.164919416	8.700968226	protein_coding	19	48281842	48287941	SEPW1
ENSG00000178982	9.858667329	10.15021936	protein_coding	19	39109722	39127595	EIF3K
ENSG00000178988	8.518728184	8.752849683	protein_coding	4	6709428	6711607	MRFAP1L1
ENSG00000178996	9.308732943	8.932538804	protein_coding	5	53813589	53842415	SNX18
ENSG00000178997	1.16343121	1.667587519	protein_coding	15	41474923	41522941	EXD1
ENSG00000178999	10.37649554	10.97268647	protein_coding	17	8108051	8113936	AURKB
ENSG00000179008	0	0.390640832	protein_coding	14	60863187	60982261	C14orf39
ENSG00000179010	11.11768565	11.16079758	protein_coding	4	6641818	6644472	MRFAP1
ENSG00000179021	7.136425764	7.204272857	protein_coding	3	88198893	88207118	C3orf38
ENSG00000179023	1.616589159	0	protein_coding	1	18807424	18812534	KLHDC7A
ENSG00000179029	6.236372259	7.022976646	protein_coding	17	8076555	8079717	TMEM107

ENSG00000179031	0	1.667587519	pseudogene	X	100165700	100166480	
ENSG00000179038	1.799000381	1.667587519	protein_coding	11	66963391	66964638	
ENSG00000179041	9.276405902	8.853271458	protein_coding	8	67341263	67342966	RRS1
ENSG00000179044	2.847891871	3.570966319	protein_coding	16	67218269	67224107	EXOC3L1
ENSG00000179046	6.998676229	6.742042315	protein_coding	4	189012427	189030757	TRIML2
ENSG00000179051	11.40130517	11.12146634	protein_coding	1	17733256	17766220	RCC2
ENSG00000179057	0.869158192	1.353254395	protein_coding	11	18725852	18747777	IGSF22
ENSG00000179058	1.16343121	0.697730409	protein_coding	9	132374504	132383055	C9orf50
ENSG00000179059	0.499066092	0.697730409	protein_coding	4	188916925	188926204	ZFP42
ENSG00000179061	0.499066092	0	pseudogene	2	25592818	25593185	
ENSG00000179066	0.499066092	1.925536307	protein_coding	19	35596873	35598141	
ENSG00000179071	1.16343121	0.697730409	protein_coding	11	85394893	85397320	CCDC89
ENSG00000179082	1.16343121	1.802319292	processed_transc	9	132083295	132087184	C9orf106
ENSG00000179085	6.835971536	7.711939664	protein_coding	1	155112367	155113071	DPM3
ENSG00000179091	10.25781591	10.4904901	protein_coding	8	145149930	145152428	CYC1
ENSG00000179094	9.226515195	8.191721221	protein_coding	17	8043809	8059678	PER1
ENSG00000179101	0	0.390640832	pseudogene	X	122648324	122648723	
ENSG00000179104	6.496213887	5.486957566	protein_coding	12	83080659	83528649	TMTC2
ENSG00000179111	6.0776925	4.622587519	protein_coding	17	8023908	8027410	HES7
ENSG00000179115	11.53530898	11.84762072	protein_coding	19	13033284	13044558	FARSA
ENSG00000179119	8.475827914	8.34031144	protein_coding	11	18627948	18656338	SPTY2D1
ENSG00000179131	1.407729925	0.390640832	pseudogene	7	66379568	66379930	
ENSG00000179133	3.077135474	1.802319292	protein_coding	10	23556124	23633774	C10orf67
ENSG00000179134	9.078865158	8.427593853	protein_coding	19	39833108	39875535	SAMD4B
ENSG00000179141	0.499066092	0.697730409	antisense	13	30051033	30061887	MTUS2-AS1
ENSG00000179148	3.950976309	3.570966319	protein_coding	17	7999218	8022234	ALOXE3
ENSG00000179151	8.992533411	8.76733661	protein_coding	15	74922899	74988633	EDC3
ENSG00000179152	7.31554162	7.764467022	protein_coding	3	44379611	44450943	C3orf23
ENSG00000179157	0	0.950786998	pseudogene	6	43331217	43331957	
ENSG00000179163	6.616550626	6.117167886	protein_coding	1	24171567	24194784	FUCA1
ENSG00000179165	0.499066092	0.697730409	protein_coding	6	36358328	36410666	PXT1
ENSG00000179168	3.554067925	2.721932731	protein_coding	19	38874995	38878668	GGN
ENSG00000179178	0.869158192	0	protein_coding	1	43735665	43739673	TMEM125
ENSG00000179195	10.19612778	10.0959115	protein_coding	12	124456392	124499974	ZNF664
ENSG00000179218	14.09874683	14.36031761	protein_coding	19	13049414	13055304	CALR
ENSG00000179219	1.16343121	0	lincRNA	16	85316564	85319569	LINC00311
ENSG00000179222	11.59164972	11.58913723	protein_coding	X	51546103	51645453	MAGED1
ENSG00000179240	4.292665995	5.236305879	protein_coding	11	76092357	76125663	
ENSG00000179241	8.14188337	8.010476879	protein_coding	11	35965531	36253686	LDLRAD3
ENSG00000179242	1.960915222	1.353254395	protein_coding	20	59827559	60512307	CDH4
ENSG00000179251	2.10647801	1.16600992	protein_coding	10	47151363	47151848	
ENSG00000179256	1.407729925	0.390640832	protein_coding	12	14957584	14967116	C12orf69
ENSG00000179262	11.87633872	11.86672759	protein_coding	19	13056654	13064448	RAD23A
ENSG00000179271	10.20777098	11.07999684	protein_coding	19	13064972	13068050	GADD45GIP1
ENSG00000179277	3.950976309	4.319727133	pseudogene	17	15690066	15690888	MEIS3P1
ENSG00000179284	2.671945279	3.183544561	protein_coding	19	13080432	13085567	DAND5
ENSG00000179292	0.499066092	0.390640832	protein_coding	11	66059341	66064135	TMEM151A
ENSG00000179294	8.154494062	8.315177126	protein_coding	17	36827961	36831187	C17orf96
ENSG00000179295	11.03371401	10.99087786	protein_coding	12	112856155	112947717	PTPN11
ENSG00000179296	1.616589159	2.579085888	pseudogene	10	49217898	49239743	CTGLF12P
ENSG00000179299	3.448790144	2.851901313	protein_coding	4	40751914	40812002	NSUN7
ENSG00000179300	3.211941663	1.925536307	protein_coding	X	77911566	77914825	ZCCHC5
ENSG00000179304	3.830412367	3.368666104	protein_coding	X	52920336	52937587	FAM156B
ENSG00000179314	1.616589159	0.950786998	protein_coding	17	5973934	6027745	WSCD1
ENSG00000179331	0.869158192	0	protein_coding	11	107799229	107834208	RAB39A
ENSG00000179335	8.225937332	8.266472275	protein_coding	15	74890841	74932057	CLK3
ENSG00000179342	0.499066092	0	pseudogene	7	65970142	65971210	
ENSG00000179344	0	0.390640832	protein_coding	6	32627244	32636160	HLA-DQB1
ENSG00000179348	8.622223055	8.419754632	protein_coding	3	128198270	128212028	GATA2
ENSG00000179361	6.664362065	5.404622322	protein_coding	15	74833518	74890472	ARID3B
ENSG00000179362	4.642199401	5.767861411	pseudogene	15	45803334	45878488	HMGN2P46
ENSG00000179363	0	0.950786998	protein_coding	X	102965837	102968956	TMEM31
ENSG00000179364	10.2307792	9.383796417	protein_coding	14	105766900	105864484	PACS2

ENSG00000179387	6.93675248	7.332282157	protein_coding	4	141445312	141474924	ELMOD2
ENSG00000179388	5.301468762	1.16600992	protein_coding	8	22545172	22550815	EGR3
ENSG00000179397	2.238690726	3.081239798	protein_coding	1	244617679	244804479	C1orf101
ENSG00000179403	9.083268927	9.29944532	protein_coding	1	1370241	1378262	VWA1
ENSG00000179406	6.560798025	6.155435249	lincRNA	7	65841031	65866325	LINC00174
ENSG00000179407	0.499066092	0	protein_coding	3	128181282	128186091	DNAJB8
ENSG00000179409	10.39698226	10.68043452	protein_coding	17	647661	655501	GEMIN4
ENSG00000179428	3.146112541	2.721932731	antisense	7	22765014	22767239	
ENSG00000179431	8.320363082	8.64266806	protein_coding	11	35639735	35642419	FJX1
ENSG00000179454	5.707331524	6.149127429	protein_coding	14	45397672	45511525	KLHL28
ENSG00000179455	3.554067925	4.021284656	protein_coding	15	23810454	23873064	MKRN3
ENSG00000179456	7.478203418	7.852510423	protein_coding	1	244214583	244220778	ZNF238
ENSG00000179467	0.499066092	0.697730409	pseudogene	20	55097413	55097855	
ENSG00000179476	4.381167248	5.339632724	protein_coding	14	45366498	45376460	C14orf28
ENSG00000179477	1.16343121	3.493416095	protein_coding	17	7975954	7991022	ALOX12B
ENSG00000179523	5.707331524	5.43605125	lincRNA	15	44819455	44829121	
ENSG00000179526	8.523597116	8.617758557	protein_coding	8	145153536	145163027	SHARPIN
ENSG00000179528	3.652182994	3.906303962	protein_coding	2	74724644	74732192	LBX2
ENSG00000179532	8.921758872	9.040423325	protein_coding	11	6518490	6593257	DNHD1
ENSG00000179546	5.124761037	3.368666104	protein_coding	1	23516993	23521222	HTR1D
ENSG00000179562	8.915598341	8.668257984	protein_coding	7	127220672	127233665	GCC1
ENSG00000179564	1.16343121	2.334191469	protein_coding	3	50316458	50325545	C3orf45
ENSG00000179580	0.499066092	1.16600992	protein_coding	16	2016824	2018976	RNF151
ENSG00000179583	5.255381949	3.714580548	protein_coding	16	10971039	11023624	CIITA
ENSG00000179588	7.781675404	7.467704891	protein_coding	16	88519725	88601572	ZFPM1
ENSG00000179598	6.809636522	6.475832214	protein_coding	17	17104309	17109646	PLD6
ENSG00000179604	10.04120874	9.53114363	protein_coding	17	71279764	71308143	CDC42EP4
ENSG00000179611	2.10647801	1.353254395	pseudogene	13	44542560	44545335	DGKZP1
ENSG00000179627	6.421663777	4.932754837	protein_coding	14	105266933	105271049	ZBTB42
ENSG00000179630	5.752272083	5.767861411	protein_coding	13	44453420	44468067	LACC1
ENSG00000179632	10.08852506	10.27784723	protein_coding	8	145159402	145162514	MAF1
ENSG00000179673	2.238690726	1.802319292	protein_coding	17	45055523	45056614	RPRML
ENSG00000179674	4.491309013	2.420525079	protein_coding	3	160394948	160396233	ARL14
ENSG00000179698	7.266040677	7.797107849	protein_coding	8	145162629	145173218	KIAA1875
ENSG00000179715	8.629769183	6.419292172	protein_coding	12	47473386	47630443	FAM113B
ENSG00000179743	3.989024711	4.990346085	processed_transc	1	16160560	16174642	
ENSG00000179750	7.967047117	8.222087975	protein_coding	22	39378352	39388809	APOBEC3B
ENSG00000179761	3.502389126	2.851901313	protein_coding	17	27369918	27384234	PIPOX
ENSG00000179766	1.16343121	1.667587519	pseudogene	9	35406752	35483026	ATP8B5P
ENSG00000179772	1.16343121	0.950786998	protein_coding	20	30432103	30433420	FOXS1
ENSG00000179774	0	0.390640832	protein_coding	10	69990386	69991871	ATOH7
ENSG00000179776	6.676071387	7.605389119	protein_coding	16	66400533	66438686	CDH5
ENSG00000179818	5.286269488	5.683080141	processed_transc	2	70189395	70315686	PCBP1-AS1
ENSG00000179820	11.43184222	10.79015471	protein_coding	19	54369477	54379691	MYADM
ENSG00000179826	0.499066092	2.851901313	protein_coding	11	18142502	18160027	MRGPRX3
ENSG00000179832	7.565780433	7.077141316	protein_coding	8	145202919	145316843	HEATR7A
ENSG00000179833	9.59671396	9.436546455	protein_coding	2	64858755	64977449	SERTAD2
ENSG00000179837	9.723436728	9.5085315	protein_coding	3	51428731	51435330	RBM15B
ENSG00000179841	3.004694206	3.232099092	protein_coding	14	64932217	64936425	AKAP5
ENSG00000179846	4.097495944	3.183544561	protein_coding	19	45653008	45663408	NKPD1
ENSG00000179855	0.869158192	0.390640832	protein_coding	19	3585551	3593539	GIPC3
ENSG00000179859	5.889353623	6.686500471	antisense	17	7816642	7819271	
ENSG00000179862	4.842525946	5.125003832	protein_coding	1	41326729	41328018	CITED4
ENSG00000179869	3.211941663	0.390640832	protein_coding	7	48211055	48687092	ABCA13
ENSG00000179873	0.499066092	1.16600992	protein_coding	19	56296770	56348128	NLRP11
ENSG00000179886	6.283060568	6.440109466	protein_coding	8	144680074	144682485	TIGD5
ENSG00000179889	10.15540476	10.20825661	protein_coding	16	15068448	15233196	PDXDC1
ENSG00000179899	2.847891871	2.579085888	pseudogene	12	55804263	55808633	PHC1P1
ENSG00000179902	0.499066092	0.390640832	protein_coding	1	109648573	109656479	C1orf194
ENSG00000179909	0	0.390640832	protein_coding	19	58208735	58220579	ZNF154
ENSG00000179912	8.871721931	8.512355001	protein_coding	12	57643392	57824788	R3HDM2
ENSG00000179913	8.013936171	3.993387124	protein_coding	19	17905919	17924385	B3GNT3
ENSG00000179918	4.062237333	4.777836593	protein_coding	16	30454952	30457502	SEPHS2

ENSG00000179921	2.10647801	2.144285137	protein_coding	2	219124219	219128582	GPBAR1
ENSG00000179922	6.462804234	6.515603638	protein_coding	19	56132107	56135941	ZNF784
ENSG00000179933	8.78516728	9.411730515	protein_coding	14	23563974	23569665	C14orf119
ENSG00000179934	0	0.390640832	protein_coding	3	39371197	39375171	CCR8
ENSG00000179935	0	0.950786998	processed_transc	20	18768295	18774979	
ENSG00000179938	0.869158192	1.16600992	protein_coding	15	30375158	30385702	GOLGA8J
ENSG00000179941	6.502803968	6.934050347	protein_coding	12	76738254	76742222	BBS10
ENSG00000179943	7.666067113	7.992972811	protein_coding	19	56102738	56110893	FIZ1
ENSG00000179950	10.37649554	10.47701775	protein_coding	8	144898514	144912029	PUF60
ENSG00000179954	6.112579918	6.371325053	protein_coding	19	55999870	56030466	SSC5D
ENSG00000179958	9.46791839	9.393168008	protein_coding	16	30434940	30441396	DCTPP1
ENSG00000179965	5.124761037	6.293387477	protein_coding	16	30418618	30430745	ZNF771
ENSG00000179967	3.652182994	4.021284656	pseudogene	4	140036084	140036528	PPP1R14BP3
ENSG00000179978	0.869158192	0.697730409	pseudogene	5	69390202	69424261	
ENSG00000179979	7.289098491	6.819555981	protein_coding	4	1385340	1389780	CRIPAK
ENSG00000179981	7.363400307	7.492830145	protein_coding	18	72922710	73001905	TSHZ1
ENSG00000179988	4.491309013	5.248158938	protein_coding	10	124713897	124757029	PSTK
ENSG00000179994	0.499066092	0.697730409	pseudogene	7	72333321	72341076	
ENSG00000180008	8.520352988	8.720244202	protein_coding	14	55493948	55516206	SOCS4
ENSG00000180011	8.37885234	8.461080043	protein_coding	18	72907063	72921303	ZADH2
ENSG00000180015	1.960915222	2.721932731	pseudogene	4	189659506	189660648	
ENSG00000180019	0.499066092	0	pseudogene	7	112086219	112086430	
ENSG00000180035	6.548110424	7.278392738	protein_coding	16	30389427	30411429	ZNF48
ENSG00000180043	0.499066092	0.390640832	protein_coding	19	55866277	55874620	FAM71E2
ENSG00000180061	0	0.390640832	protein_coding	19	55824169	55836708	TMEM150B
ENSG00000180066	4.543379137	5.150490275	protein_coding	10	134257831	134262912	C10orf91
ENSG00000180071	5.774228067	6.699508919	protein_coding	9	38540564	38620657	ANKRD18A
ENSG00000180083	0	0.950786998	protein_coding	20	44277202	44298909	WFDC11
ENSG00000180089	6.830742851	6.235021771	protein_coding	19	55738002	55740632	TMEM86B
ENSG00000180096	3.652182994	4.489113623	protein_coding	16	30389454	30407312	1-sep
ENSG00000180098	7.60612345	7.920752191	protein_coding	1	28879597	28905051	TRNAU1AP
ENSG00000180104	10.09344535	9.97005321	protein_coding	5	443273	472052	EXOC3
ENSG00000180113	3.335220907	4.528525526	protein_coding	6	46655612	46672056	TDRD6
ENSG00000180138	0.869158192	0.950786998	protein_coding	13	37677398	37679803	CSNK1A1L
ENSG00000180139	0.869158192	2.501982735	antisense	10	90692441	90699731	
ENSG00000180152	0.869158192	0	pseudogene	2	113610905	113611699	
ENSG00000180155	7.334137164	6.186567173	protein_coding	8	143845752	143859640	LYNX1
ENSG00000180176	5.316509574	2.144285137	protein_coding	11	2185159	2193107	TH
ENSG00000180178	0	0.697730409	pseudogene	2	130748029	130808704	
ENSG00000180182	11.11257821	10.84313585	protein_coding	X	40507558	40595110	MED14
ENSG00000180185	8.004679484	8.200898549	protein_coding	16	1876968	1890208	FAHD1
ENSG00000180189	1.960915222	3.608232228	pseudogene	14	58751491	58752116	HMGB1P14
ENSG00000180190	6.535310252	6.948652068	protein_coding	8	439803	495781	C8orf42
ENSG00000180198	10.4757196	10.40968193	protein_coding	1	28832455	28865708	RCC1
ENSG00000180209	0.869158192	1.353254395	protein_coding	16	30382255	30389312	MYLPF
ENSG00000180210	0.499066092	1.518964905	protein_coding	11	46740730	46761056	F2
ENSG00000180211	3.077135474	4.048652948	pseudogene	6	39926153	39926572	
ENSG00000180221	0	0.390640832	pseudogene	10	59972302	59972797	
ENSG00000180228	8.470794781	8.020878279	protein_coding	2	179296141	179316239	PRKRA
ENSG00000180229	6.94645393	8.253299769	pseudogene	15	20587869	20711433	HERC2P3
ENSG00000180233	7.50471092	7.103479673	protein_coding	7	30323923	30452118	ZNRF2
ENSG00000180245	0.499066092	2.501982735	protein_coding	4	110749150	110765760	RRH
ENSG00000180251	0	0.390640832	protein_coding	2	103089762	103150431	SLC9A4
ENSG00000180257	6.766474304	6.908136232	protein_coding	19	53430388	53466164	ZNF816
ENSG00000180263	8.613115289	8.580741083	protein_coding	12	95470525	95611258	FGD6
ENSG00000180279	0.499066092	0.950786998	protein_coding	19	51320937	51322131	
ENSG00000180284	0.499066092	0.950786998	pseudogene	X	102906661	102907034	
ENSG00000180304	8.429884808	8.542748725	pseudogene	15	64979773	64995417	
ENSG00000180305	0.499066092	0	protein_coding	20	44258165	44259835	WFDC10A
ENSG00000180316	4.352265886	3.081239798	protein_coding	6	36210980	36276372	PNPLA1
ENSG00000180329	7.413224588	7.38691391	protein_coding	17	42754805	42767165	CCDC43
ENSG00000180332	0	0.697730409	protein_coding	13	45766988	45775175	KCTD4
ENSG00000180336	3.652182994	3.781359661	protein_coding	17	42733762	42767676	C17orf104

ENSG00000180340	8.385999544	8.737164829	protein_coding	17	42634827	42636907	FZD2
ENSG00000180346	5.431503808	5.717592784	protein_coding	4	90033968	90036050	TIGD2
ENSG00000180353	2.57521082	0.697730409	protein_coding	3	121350246	121379774	HCLS1
ENSG00000180354	8.297803463	8.313767852	protein_coding	7	30174426	30202378	C7orf41
ENSG00000180357	9.487986111	9.193855808	protein_coding	15	64791491	64978264	ZNF609
ENSG00000180370	11.01741032	10.68861275	protein_coding	3	196466728	196559518	PAK2
ENSG00000180376	7.062518763	7.392265003	protein_coding	3	56591189	56655846	CCDC66
ENSG00000180385	6.393570237	6.408769788	pseudogene	3	10028577	10048674	
ENSG00000180389	2.671945279	3.608232228	protein_coding	13	28519343	28519727	ATP5EP2
ENSG00000180398	10.24608033	10.21958906	protein_coding	2	47129009	47168994	MCFD2
ENSG00000180422	2.10647801	2.788380093	lincRNA	16	89225554	89230653	LINC00304
ENSG00000180423	5.684325135	5.776071827	protein_coding	11	46624411	46639459	HARBI1
ENSG00000180425	4.962122459	5.784235782	protein_coding	11	114262165	114271233	C11orf71
ENSG00000180432	0.869158192	0	protein_coding	3	42897497	42917633	CYP8B1
ENSG00000180438	0.499066092	0.390640832	protein_coding	3	13978756	14124311	TPRXL
ENSG00000180447	1.16343121	1.667587519	protein_coding	9	89559279	89562104	GAS1
ENSG00000180448	10.63230386	10.18146283	protein_coding	19	1065930	1086627	HMHA1
ENSG00000180458	4.6658819	4.528525526	protein_coding	19	38036373	38040073	
ENSG00000180479	4.842525946	4.794098771	protein_coding	19	38053552	38085673	ZNF571
ENSG00000180481	1.616589159	1.802319292	protein_coding	12	75784850	75826468	GLIPR1L2
ENSG00000180488	6.994006093	7.057067116	protein_coding	1	78245309	78344106	FAM73A
ENSG00000180509	0.499066092	1.16600992	protein_coding	21	35818988	35884573	KCNE1
ENSG00000180525	1.407729925	2.501982735	protein_coding	10	695888	709947	C10orf108
ENSG00000180530	8.741255403	7.875618202	protein_coding	21	16333556	16437321	NRIP1
ENSG00000180532	2.762599152	2.039052734	protein_coding	19	58180303	58190519	ZSCAN4
ENSG00000180535	5.431503808	3.679994897	protein_coding	7	97841566	97842271	BHLHA15
ENSG00000180537	1.960915222	1.925536307	protein_coding	6	13924677	13980533	RNF182
ENSG00000180573	7.553136766	7.915187059	protein_coding	6	26124373	26139336	HIST1H2AC
ENSG00000180574	3.274897671	3.493416095	protein_coding	12	10658201	10675734	
ENSG00000180581	2.35979773	1.925536307	pseudogene	10	93565803	93567253	SRP9P1
ENSG00000180592	4.352265886	4.021284656	protein_coding	10	21802407	21814611	C10orf140
ENSG00000180596	4.19837882	4.250702764	protein_coding	6	26115129	26124154	HIST1H2BC
ENSG00000180610	1.960915222	1.802319292	pseudogene	4	39771701	39773000	ZBTB12B
ENSG00000180611	8.235860718	8.11126287	protein_coding	3	192514604	192635950	MB21D2
ENSG00000180616	1.799000381	2.579085888	protein_coding	17	71161160	71168060	SSTR2
ENSG00000180626	6.907250452	7.672899552	protein_coding	17	5082830	5095178	ZNF594
ENSG00000180628	8.994872754	9.416990515	protein_coding	10	92979908	93044088	PCGF5
ENSG00000180638	1.960915222	1.518964905	protein_coding	17	19581628	19622292	SLC47A2
ENSG00000180660	0	0.697730409	protein_coding	13	36047926	36050832	MAB21L1
ENSG00000180662	0.499066092	0.950786998	pseudogene	14	65934525	65935059	
ENSG00000180667	7.50471092	6.941369681	protein_coding	1	207217194	207226325	YOD1
ENSG00000180672	4.903563137	1.667587519	pseudogene	2	206642540	206644433	
ENSG00000180673	1.16343121	0.697730409	pseudogene	4	63682544	63684512	
ENSG00000180694	8.460675529	8.306700762	protein_coding	8	91634223	91803860	TMEM64
ENSG00000180697	0	1.16600992	protein_coding	3	126245842	126277808	C3orf22
ENSG00000180712	0.499066092	1.16600992	processed_transc	4	185261909	185275130	
ENSG00000180720	2.238690726	0.697730409	protein_coding	11	46406640	46408107	CHRM4
ENSG00000180739	3.950976309	2.144285137	protein_coding	19	10623419	10628668	S1PR5
ENSG00000180745	0	0.697730409	protein_coding	10	129676105	129691211	CLRN3
ENSG00000180747	4.062237333	4.319727133	pseudogene	16	21458004	21531765	
ENSG00000180758	7.888702948	7.392265003	protein_coding	1	9160364	9189250	GPR157
ENSG00000180764	4.062237333	3.993387124	pseudogene	10	95717948	95721297	PIPSL
ENSG00000180767	0	0.697730409	protein_coding	3	126243126	126262134	CHST13
ENSG00000180769	2.762599152	3.532712221	processed_transc	4	85887538	85932430	WDFY3-AS2
ENSG00000180771	8.292108003	8.608592853	pseudogene	11	94800056	94804388	SRSF8
ENSG00000180773	7.2307479	7.56568256	protein_coding	11	92877341	92931130	SLC36A4
ENSG00000180776	7.066972635	6.490875274	protein_coding	13	21950263	22033509	ZDHHC20
ENSG00000180777	0	0.697730409	protein_coding	18	14748239	14852737	ANKRD30B
ENSG00000180787	6.554468172	6.673373662	protein_coding	17	4981754	4999668	ZFP3
ENSG00000180801	7.842689815	7.829026491	protein_coding	4	114821440	114900883	ARSJ
ENSG00000180806	4.593635215	5.125003832	protein_coding	12	54388679	54397121	HOXC9
ENSG00000180812	0.869158192	1.518964905	pseudogene	10	43977224	43978457	
ENSG00000180815	1.616589159	0.950786998	protein_coding	X	19378174	19533379	MAP3K15

ENSG00000180817	9.984657349	9.986509737	protein_coding	10	71962587	71993667	PPA1
ENSG00000180818	3.652182994	4.468996429	protein_coding	12	54378849	54384063	HOXC10
ENSG00000180822	8.3053626	8.613182984	protein_coding	6	3231637	3303607	PSMG4
ENSG00000180834	5.331395195	5.294620694	protein_coding	3	183533664	183543382	MAP6D1
ENSG00000180846	2.847891871	3.411459265	protein_coding	19	1952530	1954548	CSNK1G2-AS1
ENSG00000180855	5.301468762	5.6022688	protein_coding	19	12540521	12551926	ZNF443
ENSG00000180861	0.499066092	0	protein_coding	12	13524023	13540101	C12orf36
ENSG00000180867	4.923348194	5.175534283	pseudogene	1	146649692	146651206	PDIA3P
ENSG00000180878	0.499066092	0	protein_coding	11	6226796	6232362	C11orf42
ENSG00000180879	10.26267769	10.62242012	protein_coding	X	153058971	153063960	SSR4
ENSG00000180881	4.999881834	4.888000647	protein_coding	12	75669759	75784708	CAPS2
ENSG00000180884	5.316509574	5.674321415	protein_coding	19	35447258	35454953	ZNF792
ENSG00000180891	8.223944437	7.968104748	protein_coding	17	55940337	56032684	CUEDC1
ENSG00000180900	10.91397415	10.49516031	protein_coding	8	144873090	144897549	SCRIB
ENSG00000180901	8.325948295	8.359562039	protein_coding	17	73043279	73061979	KCTD2
ENSG00000180902	7.572060955	7.176629214	protein_coding	2	242673994	242708231	D2HGDH
ENSG00000180914	5.286269488	5.085909553	protein_coding	3	8792094	8811314	OXTR
ENSG00000180917	8.137655191	8.315177126	protein_coding	16	71315292	71323618	FTSJD1
ENSG00000180919	0	0.390640832	protein_coding	11	6128914	6130065	OR56B4
ENSG00000180921	9.979941537	8.861975727	protein_coding	8	144806103	144815971	FAM83H
ENSG00000180929	1.616589159	1.16600992	protein_coding	3	51989330	51991509	GPR62
ENSG00000180938	3.830412367	4.29708397	protein_coding	8	125985540	125991631	ZNF572
ENSG00000180953	5.141750194	4.857374129	protein_coding	15	80191182	80216044	ST20
ENSG00000180957	10.94787535	10.94769033	protein_coding	22	28202413	28316122	PITPNB
ENSG00000180964	7.363400307	7.480322213	protein_coding	X	102507923	102510131	TCEAL8
ENSG00000180979	6.738827102	6.515603638	protein_coding	15	42834720	42841000	LRRC57
ENSG00000180988	0	0.390640832	protein_coding	11	5841544	5842578	OR52N2
ENSG00000180992	8.838241835	9.315025211	protein_coding	6	44081194	44095194	MRPL14
ENSG00000180998	1.799000381	4.15321211	protein_coding	14	53019866	53104431	GPR137C
ENSG00000181004	5.938793061	6.434933235	protein_coding	4	123653857	123666098	BBS12
ENSG00000181007	6.567100227	6.690849666	protein_coding	19	36874593	36909558	ZFP82
ENSG00000181013	2.10647801	2.242360793	protein_coding	17	56618948	56621683	C17orf47
ENSG00000181016	3.004694206	3.493416095	protein_coding	7	112120908	112130942	C7orf53
ENSG00000181019	10.83195583	10.5737972	protein_coding	16	69740899	69760854	NQO1
ENSG00000181026	8.875535495	8.439273392	protein_coding	15	89164527	89175513	AEN
ENSG00000181027	6.846372398	7.067139131	protein_coding	19	47249303	47261832	FKRP
ENSG00000181029	9.090943324	9.832648794	protein_coding	19	7745761	7747748	TRAPPC5
ENSG00000181031	7.484876093	6.35497494	protein_coding	17	62293	202888	RPH3AL
ENSG00000181035	6.760987087	6.775188634	protein_coding	19	19174803	19223704	SLC25A42
ENSG00000181038	7.423680977	7.747865429	protein_coding	17	74722940	74729961	METTL23
ENSG00000181039	4.292665995	3.935919592	protein_coding	1	145470508	145475646	ANKRD34A
ENSG00000181045	6.17992145	6.450406542	protein_coding	17	78194200	78227306	SLC26A11
ENSG00000181061	8.874265427	8.93712253	protein_coding	3	42798669	42846023	HIGD1A
ENSG00000181085	6.023726639	5.908941696	protein_coding	8	144798429	144804628	MAPK15
ENSG00000181090	9.802351442	9.621627484	protein_coding	9	140513444	140764468	EHMT1
ENSG00000181097	3.603959378	3.935919592	antisense	8	144778324	144780583	
ENSG00000181101	0.499066092	0.390640832	pseudogene	1	175013762	175014784	SDCCAG3P2
ENSG00000181104	7.437505974	7.093659051	protein_coding	5	76011868	76031606	F2R
ENSG00000181123	1.16343121	0.950786998	processed_transc	22	30887789	30888791	
ENSG00000181126	3.211941663	4.810179682	pseudogene	6	29758637	29765588	HLA-P
ENSG00000181135	6.548110424	6.787424764	protein_coding	8	144766622	144796068	ZNF707
ENSG00000181143	8.052627651	3.493416095	protein_coding	19	8959520	9092018	MUC16
ENSG00000181163	12.06154176	12.35774806	protein_coding	5	170814120	170838141	NPM1
ENSG00000181191	8.730065371	8.804937616	protein_coding	X	68380582	68385364	PJA1
ENSG00000181192	9.150911694	8.463623996	protein_coding	10	12110971	12165224	DHTKD1
ENSG00000181215	0	0.950786998	protein_coding	4	5958845	5991545	C4orf50
ENSG00000181218	4.352265886	5.018301917	protein_coding	1	228645065	228645560	HIST3H2A
ENSG00000181220	8.541310587	8.14810445	protein_coding	7	149169885	149194908	ZNF746
ENSG00000181222	11.90476351	11.94311554	protein_coding	17	7387866	7417933	POLR2A
ENSG00000181227	2.471521042	2.144285137	pseudogene	1	76209103	76210461	
ENSG00000181240	1.799000381	2.242360793	protein_coding	19	6426050	6433790	SLC25A41
ENSG00000181260	0.499066092	0	pseudogene	3	179182134	179183116	
ENSG00000181264	6.25990527	6.977419495	protein_coding	11	120195838	120204391	TMEM136

ENSG00000181274	7.04456449	7.359856617	protein_coding	10	99092255	99094458	FRAT2
ENSG00000181284	7.800519667	7.646273261	protein_coding	17	7338762	7340998	TMEM102
ENSG00000181315	4.689181911	5.259915406	protein_coding	6	26636518	26659980	ZNF322
ENSG00000181322	3.211941663	2.788380093	protein_coding	3	137980279	138048728	NME9
ENSG00000181323	0.869158192	2.334191469	protein_coding	17	7323643	7327553	SPEM1
ENSG00000181333	2.928422289	4.15321211	protein_coding	11	93754527	93846917	HEPHL1
ENSG00000181350	5.124761037	5.283144778	protein_coding	17	16344891	16395467	FAM211A
ENSG00000181355	2.471521042	2.420525079	protein_coding	6	9596343	10211841	OFCC1
ENSG00000181358	0.499066092	0	pseudogene	13	50464727	50467134	CTAGE10P
ENSG00000181359	1.960915222	0.697730409	pseudogene	4	171502621	171526601	HSP90AA6P
ENSG00000181378	0	0.950786998	protein_coding	2	219867568	219906249	CCDC108
ENSG00000181381	9.158235462	9.883881858	protein_coding	4	169277886	169458937	DDX60L
ENSG00000181392	5.512107582	2.501982735	protein_coding	19	36494209	36499695	C19orf46
ENSG00000181396	8.265227794	8.493806588	protein_coding	17	80347086	80376513	C17orf101
ENSG00000181404	4.800350936	5.072639111	pseudogene	9	14511	29739	
ENSG00000181408	0	0.390640832	protein_coding	17	80332153	80333462	UTS2R
ENSG00000181409	3.78787645	2.721932731	protein_coding	17	79091095	79139872	AATK
ENSG00000181418	5.239686603	3.279072565	protein_coding	12	49388932	49393092	DDN
ENSG00000181444	5.512107582	5.059245468	protein_coding	7	149461271	149470568	ZNF467
ENSG00000181449	3.448790144	0	protein_coding	3	181429714	181432221	SOX2
ENSG00000181450	5.417620896	5.840125386	protein_coding	1	227751236	227847594	ZNF678
ENSG00000181458	7.017207048	8.120964787	protein_coding	3	100211463	100296288	TMEM45A
ENSG00000181467	9.936182363	8.89344947	protein_coding	3	152880029	152886265	RAP2B
ENSG00000181472	8.085926212	7.813155441	protein_coding	6	151685252	151712683	ZBTB2
ENSG00000181481	8.036821332	7.86795666	protein_coding	17	29298041	29326929	RNF135
ENSG00000181511	0.869158192	0.390640832	pseudogene	X	119866948	119867250	
ENSG00000181513	6.604346021	6.877938671	protein_coding	17	43209967	43221539	ACBD4
ENSG00000181523	8.229914881	8.399968169	protein_coding	17	78183079	78194199	SGSH
ENSG00000181524	3.146112541	3.570966319	pseudogene	6	42924083	42924503	RPL24P4
ENSG00000181544	6.095241663	6.815578497	protein_coding	X	14861529	14891191	FANCB
ENSG00000181555	9.474638693	9.492427169	protein_coding	3	47057900	47205467	SETD2
ENSG00000181577	3.744048221	3.133298822	protein_coding	6	43968317	43972887	C6orf223
ENSG00000181585	3.448790144	3.081239798	protein_coding	3	46742823	46752376	TMIE
ENSG00000181588	9.787584866	9.452642986	protein_coding	19	1554669	1568057	MEX3D
ENSG00000181609	0.499066092	0.390640832	protein_coding	11	5509915	5510979	OR52D1
ENSG00000181610	8.81194861	9.163664959	protein_coding	17	55916842	55927399	MRPS23
ENSG00000181616	0.499066092	0	protein_coding	11	5565719	5566779	OR52H1
ENSG00000181617	0.869158192	3.679994897	protein_coding	4	71091788	71100969	FDCSP
ENSG00000181619	5.512107582	5.306006045	protein_coding	14	59895740	59932060	GPR135
ENSG00000181625	1.16343121	1.518964905	protein_coding	16	29465822	29469540	SLX1B
ENSG00000181634	1.16343121	2.420525079	protein_coding	9	117551600	117568406	TNFSF15
ENSG00000181638	7.792473675	7.694718794	protein_coding	8	144328991	144358573	ZFP41
ENSG00000181649	9.319971717	8.200898549	protein_coding	11	2949503	2950685	PHLDA2
ENSG00000181652	6.112579918	6.615068802	processed_transc	7	150709302	150721586	ATG9B
ENSG00000181656	0.499066092	0	protein_coding	1	101003693	101007574	GPR88
ENSG00000181666	8.112022537	8.316585024	protein_coding	19	37808813	37855355	HKR1
ENSG00000181690	6.155035276	6.577981353	protein_coding	8	57073463	57123883	PLAG1
ENSG00000181704	8.839544001	9.031020259	protein_coding	X	67718165	67757127	YIPF6
ENSG00000181722	3.78787645	3.96493948	protein_coding	3	114056941	114866118	ZBTB20
ENSG00000181741	0.869158192	0	pseudogene	20	33063729	33064279	FDX1P1
ENSG00000181744	7.222786417	7.442134301	protein_coding	3	143690640	143767561	C3orf58
ENSG00000181751	6.912209543	7.195117037	protein_coding	5	102594403	102614361	C5orf30
ENSG00000181754	2.928422289	3.081239798	protein_coding	1	110046797	110052360	AMIGO1
ENSG00000181773	4.842525946	5.059245468	protein_coding	1	27719148	27722318	GPR3
ENSG00000181781	5.538004898	6.110690132	protein_coding	19	463344	474983	ODF3L2
ENSG00000181788	8.480843549	8.273738696	protein_coding	3	150458914	150481264	SIAH2
ENSG00000181789	11.8136165	11.30004203	protein_coding	3	128968449	128996614	COPG
ENSG00000181790	4.165528823	4.810179682	protein_coding	8	143530791	143626370	BAI1
ENSG00000181798	3.652182994	2.971122874	processed_transc	2	232373137	232379050	LINC00471
ENSG00000181800	0.869158192	0.697730409	protein_coding	10	11358797	11361847	C10orf31
ENSG00000181804	6.155035276	5.953060722	protein_coding	3	142984064	143567373	SLC9A9
ENSG00000181817	7.72361917	8.034631177	protein_coding	1	36856839	36863493	LSM10
ENSG00000181826	5.986594255	5.656642807	protein_coding	4	37592422	37687998	RELL1

ENSG00000181827	8.958179637	8.69988975	protein_coding	15	56379478	56535483	RFX7
ENSG00000181830	9.066685021	8.859080135	protein_coding	11	45825623	45834566	SLC35C1
ENSG00000181847	0.869158192	0	protein_coding	3	113995760	114029135	TIGIT
ENSG00000181852	9.236434621	9.432007055	protein_coding	12	56598285	56615737	RNF41
ENSG00000181856	4.131913373	3.493416095	protein_coding	17	7185053	7191576	SLC2A4
ENSG00000181873	6.573375019	6.952279523	protein_coding	1	228353516	228369958	IBA57
ENSG00000181877	1.616589159	2.420525079	protein_coding	4	1189609	1195204	
ENSG00000181885	8.894453552	4.990346085	protein_coding	17	7163223	7166512	CLDN7
ENSG00000181894	8.314756163	8.135396417	protein_coding	19	58637697	58666477	ZNF329
ENSG00000181896	7.169937448	7.225413142	protein_coding	19	19779605	19791761	ZNF101
ENSG00000181903	0.499066092	0	protein_coding	11	55432614	55433671	OR4C6
ENSG00000181904	8.616157604	8.773501036	protein_coding	5	134181370	134195427	C5orf24
ENSG00000181908	1.407729925	0.950786998	protein_coding	11	64216546	64219126	
ENSG00000181915	8.508940744	8.273738696	protein_coding	10	64564516	64568238	ADO
ENSG00000181924	9.054401174	9.483053775	protein_coding	11	73583712	73588033	CHCHD8
ENSG00000181929	8.414239457	8.783716905	protein_coding	12	49396057	49412980	PRKAG1
ENSG00000181938	8.528449672	9.125618162	protein_coding	16	58328984	58440048	GINS3
ENSG00000181982	5.879259127	6.270323763	protein_coding	4	24807739	24981826	CCDC149
ENSG00000181991	8.009315251	8.610889744	protein_coding	15	89010684	89022222	MRPS11
ENSG00000181997	0.499066092	0	pseudogene	9	69633978	69650004	AQP7P2
ENSG00000182004	7.773523328	8.16070152	protein_coding	1	203830731	203839678	SNRPE
ENSG00000182010	3.78787645	4.903073497	protein_coding	10	63942794	64028466	RTKN2
ENSG00000182021	2.928422289	1.925536307	lincRNA	9	67017400	67032072	
ENSG00000182022	9.753451503	9.754816173	protein_coding	10	125767184	125853206	CHST15
ENSG00000182035	0.499066092	0.390640832	protein_coding	20	37209838	37217106	ADIG
ENSG00000182040	1.407729925	1.925536307	protein_coding	17	72912176	72919351	USH1G
ENSG00000182048	0.499066092	0	pseudogene	11	3631131	3658789	TRPC2
ENSG00000182050	0	1.16600992	protein_coding	12	86372516	86889092	MGAT4C
ENSG00000182054	9.719905763	10.5994706	protein_coding	15	90626277	90645736	IDH2
ENSG00000182057	4.097495944	4.29708397	lincRNA	22	42665759	42671202	
ENSG00000182087	11.27898561	11.39755719	protein_coding	19	1009650	1021141	C19orf6
ENSG00000182093	7.277615649	7.362585296	protein_coding	21	40752170	40800454	WRB
ENSG00000182095	11.73288208	11.05912178	protein_coding	7	5346421	5465045	TNRC18
ENSG00000182107	7.132181536	0.390640832	protein_coding	14	61744088	61748558	TMEM30B
ENSG00000182108	6.94645393	7.053694091	protein_coding	16	11022748	11036317	DEXI
ENSG00000182109	4.734682531	4.710890357	processed_transc	1	39987952	40011859	
ENSG00000182111	0.499066092	0.697730409	protein_coding	7	57509883	57533265	ZNF716
ENSG00000182117	9.291708897	9.782079226	protein_coding	15	34633917	34635378	NOP10
ENSG00000182118	6.095241663	5.404622322	protein_coding	1	231154704	231175992	FAM89A
ENSG00000182134	8.394884042	8.010476879	protein_coding	1	151742583	151763892	TDRKH
ENSG00000182141	7.115078652	7.603083446	protein_coding	19	21473963	21512212	ZNF708
ENSG00000182149	9.845117281	10.00278066	protein_coding	16	71919136	71962913	IST1
ENSG00000182150	8.072698871	8.295320915	protein_coding	9	98637900	98776842	C9orf102
ENSG00000182154	8.608539753	8.973277306	protein_coding	9	140445651	140447007	MRPL41
ENSG00000182158	10.46225768	9.70300667	protein_coding	7	137559725	137686813	CREB3L2
ENSG00000182162	1.16343121	0.697730409	protein_coding	X	1581465	1656000	P2RY8
ENSG00000182165	5.239686603	5.339632724	processed_transc	7	86954541	86974831	TP53TG1
ENSG00000182168	1.616589159	2.501982735	protein_coding	4	96083655	96470357	UNC5C
ENSG00000182173	8.085926212	7.887034762	protein_coding	17	73511788	73520820	TSEN54
ENSG00000182175	1.616589159	0.950786998	protein_coding	15	93586636	93632433	RGMA
ENSG00000182179	8.653654925	8.834721487	protein_coding	3	49842640	49851379	UBA7
ENSG00000182180	9.873363279	9.516516751	protein_coding	10	75006510	75012451	MRPS16
ENSG00000182183	0	0.697730409	protein_coding	1	53099016	53135355	FAM159A
ENSG00000182185	6.313367288	6.733635362	protein_coding	14	68286496	69196935	RAD51B
ENSG00000182195	9.92763636	10.01280331	protein_coding	X	140269934	140271310	LDOC1
ENSG00000182196	1.960915222	2.912743273	protein_coding	12	123464333	123467456	ARL6IP4
ENSG00000182197	10.78372758	10.32757313	protein_coding	8	118806729	119124092	EXT1
ENSG00000182199	11.94176197	12.32655617	protein_coding	12	57623110	57628718	SHMT2
ENSG00000182208	7.974178365	8.288162655	protein_coding	11	1490687	1522477	MOB2
ENSG00000182218	1.16343121	0.390640832	protein_coding	14	100111447	100146906	HHIPL1
ENSG00000182220	10.30241043	11.07833791	protein_coding	X	40440146	40465889	ATP6AP2
ENSG00000182223	1.616589159	1.353254395	protein_coding	4	48492269	48509084	ZAR1
ENSG00000182224	8.893200037	9.155034101	protein_coding	17	7761064	7780399	CYB5D1

ENSG00000182240	9.062230259	9.699773395	protein_coding	21	42539728	42648524	BACE2
ENSG00000182247	7.834876234	8.124184315	protein_coding	3	23244511	23633284	UBE2E2
ENSG00000182253	7.206730419	7.142107001	protein_coding	15	99638420	99675798	SYNM
ENSG00000182257	3.004694206	2.971122874	protein_coding	22	46445358	46450024	C22orf26
ENSG00000182261	2.10647801	0.697730409	protein_coding	11	7980971	7986973	NLRP10
ENSG00000182263	7.868433623	7.500283181	protein_coding	2	164449906	164592522	FIGN
ENSG00000182264	1.616589159	2.242360793	protein_coding	19	49244145	49250166	IZUMO1
ENSG00000182272	5.909333162	5.018301917	protein_coding	11	369796	382116	B4GALNT4
ENSG00000182287	8.414239457	9.084889332	protein_coding	X	15843929	15873054	AP1S2
ENSG00000182307	8.652173596	8.537928553	protein_coding	8	146277764	146281416	C8orf33
ENSG00000182308	3.989024711	4.385599336	protein_coding	4	41983713	41988476	DCAF4L1
ENSG00000182310	4.097495944	4.342020395	pseudogene	19	52196614	52197610	LINC00085
ENSG00000182318	7.845284967	7.672899552	protein_coding	19	58838385	58853712	ZSCAN22
ENSG00000182319	8.805299741	7.9428005	protein_coding	8	8175258	8244008	
ENSG00000182324	6.721980839	5.317302248	protein_coding	19	48958766	48969367	KCNJ14
ENSG00000182325	7.712290964	8.18248514	protein_coding	8	145579091	145583036	FBXL6
ENSG00000182326	9.978760171	11.01427562	protein_coding	12	7096351	7178336	C1S
ENSG00000182327	1.616589159	3.493416095	protein_coding	17	4692307	4693591	GLTPD2
ENSG00000182329	0.499066092	0	protein_coding	2	202937978	203062585	
ENSG00000182334	0	0.390640832	protein_coding	11	7846584	7847519	OR5P3
ENSG00000182347	0	0.697730409	pseudogene	9	5084999	5086112	
ENSG00000182359	4.026095388	5.22435463	protein_coding	11	105921825	105948492	KBTBD3
ENSG00000182362	5.785082014	6.35497494	protein_coding	21	47706251	47717665	YBEY
ENSG00000182366	0.869158192	0	pseudogene	8	325934	333174	FAM87A
ENSG00000182368	2.671945279	3.081239798	protein_coding	9	45727107	45728274	FAM27A
ENSG00000182372	7.840089987	7.905863867	protein_coding	8	1703944	1734738	CLN8
ENSG00000182376	2.10647801	3.232099092	antisense	16	88809175	88812156	
ENSG00000182378	5.360713447	4.903073497	protein_coding	X	192989	220023	PLCXD1
ENSG00000182379	6.592037289	6.338437404	protein_coding	12	57610578	57620232	NXPH4
ENSG00000182383	0.499066092	0	pseudogene	5	74286014	74286462	
ENSG00000182389	2.471521042	1.353254395	protein_coding	2	152689290	152955593	CACNB4
ENSG00000182393	0.869158192	0.697730409	protein_coding	19	39786965	39789312	IL29
ENSG00000182397	1.799000381	2.334191469	pseudogene	15	100330361	100347132	DNM1P46
ENSG00000182400	7.511262389	7.881337775	protein_coding	14	39617015	39639736	TRAPPC6B
ENSG00000182404	0	1.16600992	sense_intronic	6	128768789	128770446	
ENSG00000182405	4.903563137	4.7279204	protein_coding	15	34394274	34396591	PGBD4
ENSG00000182446	10.92382946	10.50661594	protein_coding	17	79523915	79616376	NPLOC4
ENSG00000182450	0	0.390640832	protein_coding	11	64058774	64072241	KCNK4
ENSG00000182459	5.072559152	6.480864016	protein_coding	17	80317123	80321652	TEX19
ENSG00000182463	5.729976786	6.009862691	protein_coding	20	51588946	52107617	TSHZ2
ENSG00000182472	3.274897671	1.802319292	protein_coding	19	39220832	39235114	CAPN12
ENSG00000182473	9.541775001	9.839520617	protein_coding	17	74077087	74099868	EXOC7
ENSG00000182477	0	0.390640832	pseudogene	6	28021006	28021943	OR2B8P
ENSG00000182481	11.25868748	11.45157859	protein_coding	17	66031848	66042969	KPNA2
ENSG00000182484	7.127924785	7.10998981	protein_coding	X	155249967	155255375	WASH6P
ENSG00000182489	2.57521082	2.144285137	protein_coding	X	100168431	100184422	XKRX
ENSG00000182492	2.471521042	1.925536307	protein_coding	X	152760397	152775012	BGN
ENSG00000182500	8.809292739	8.806942451	protein_coding	12	122064455	122080583	ORAI1
ENSG00000182502	0.499066092	0	pseudogene	22	22469236	22472374	FAM108A6P
ENSG00000182504	7.633406721	7.591499486	protein_coding	3	101442769	101489406	CEP97
ENSG00000182511	2.928422289	1.16600992	protein_coding	15	91426925	91439006	FES
ENSG00000182512	9.305909516	8.679244009	protein_coding	14	95999840	96011061	GLRX5
ENSG00000182518	6.196276899	7.067139131	protein_coding	X	55169535	55187743	FAM104B
ENSG00000182521	0.499066092	0.390640832	protein_coding	14	55880259	55923444	TBPL2
ENSG00000182533	0.499066092	0.950786998	protein_coding	3	8775486	8883492	CAV3
ENSG00000182534	10.13539932	10.85964836	protein_coding	17	74671809	74707087	MXRA7
ENSG00000182541	8.541310587	8.890616425	protein_coding	22	31608225	31676066	LIMK2
ENSG00000182544	8.665451116	8.966118473	protein_coding	12	53645035	53648189	MFSD5
ENSG00000182551	9.31342641	9.97005321	protein_coding	2	3501693	3523507	ADI1
ENSG00000182552	7.517784241	7.959118381	protein_coding	4	184560788	184580378	RWDD4
ENSG00000182557	3.393122761	4.761389014	protein_coding	17	4337219	4391498	SPNS3
ENSG00000182568	4.165528823	6.174194902	protein_coding	3	18386864	18487080	SATB1
ENSG00000182572	1.799000381	0.950786998	protein_coding	6	27839623	27840099	HIST1H3I

ENSG00000182574	0	0.950786998	pseudogene	X	100211594	100212528	
ENSG00000182575	1.960915222	0.950786998	protein_coding	17	47653220	47659652	NXPH3
ENSG00000182578	5.431503808	4.990346085	protein_coding	5	149432854	149492935	CSF1R
ENSG00000182580	4.409500985	2.851901313	protein_coding	3	184279572	184300197	EPHB3
ENSG00000182584	5.660945913	6.071200665	protein_coding	20	32254304	32256331	ACTL10
ENSG00000182585	1.799000381	0	protein_coding	4	75174190	75181024	EPGN
ENSG00000182586	0.499066092	0.950786998	antisense	21	46654267	46678645	LINC00334
ENSG00000182600	4.491309013	3.232099092	protein_coding	2	233721980	233743418	C2orf82
ENSG00000182606	9.396283191	8.870627795	protein_coding	3	42055294	42267381	TRAK1
ENSG00000182611	1.616589159	1.667587519	protein_coding	6	27782112	27782607	HIST1H2AJ
ENSG00000182612	4.842525946	5.486957566	protein_coding	17	79609349	79615779	TSPAN10
ENSG00000182621	3.950976309	1.518964905	protein_coding	20	8112824	8949003	PLCB1
ENSG00000182628	9.482995289	9.139208113	protein_coding	17	57187308	57232800	SKA2
ENSG00000182632	0.869158192	0.697730409	protein_coding	10	42903616	42970797	CCNYL2
ENSG00000182646	1.960915222	1.925536307	protein_coding	X	52976462	53024651	FAM156A
ENSG00000182648	1.799000381	3.279072565	processed_transc	7	156264890	156433286	
ENSG00000182670	10.60614758	10.94996007	protein_coding	21	38445526	38575413	TTC3
ENSG00000182676	0.499066092	0.390640832	protein_coding	17	79791368	79792926	PPP1R27
ENSG00000182685	5.036678094	4.65855978	protein_coding	16	2259254	2261951	C16orf79
ENSG00000182687	0.869158192	1.518964905	protein_coding	17	74070875	74073622	GALR2
ENSG00000182700	5.346128795	6.016808232	protein_coding	5	139505521	139508391	IGIP
ENSG00000182704	9.397167781	8.414504719	protein_coding	11	76493295	76509198	TSKU
ENSG00000182718	12.48903024	12.29931094	protein_coding	15	60639333	60695082	ANXA2
ENSG00000182722	1.616589159	1.353254395	pseudogene	7	64312775	64313732	SEPHS1P1
ENSG00000182732	3.989024711	2.334191469	protein_coding	14	72315323	73030654	RGS6
ENSG00000182742	0	0.950786998	protein_coding	17	46652875	46657473	HOXB4
ENSG00000182749	6.984620223	6.142791909	protein_coding	1	26187701	26197744	PAQR7
ENSG00000182752	6.573375019	6.535085617	protein_coding	9	118916083	119164601	PAPPA
ENSG00000182759	2.10647801	2.144285137	protein_coding	8	144501352	144512576	MAFA
ENSG00000182768	7.754320745	7.91704449	protein_coding	15	90808891	90816463	NGRN
ENSG00000182771	0.869158192	1.353254395	protein_coding	10	87359312	88126250	GRID1
ENSG00000182782	3.211941663	0.390640832	protein_coding	12	123185840	123187890	HCAR2
ENSG00000182791	3.335220907	3.081239798	protein_coding	11	66357640	66360554	CCDC87
ENSG00000182795	5.255381949	1.353254395	protein_coding	1	207191866	207206101	C1orf116
ENSG00000182796	7.178195136	7.029859489	pseudogene	12	56223529	56230030	TMEM198B
ENSG00000182798	0.869158192	0.390640832	protein_coding	X	16185604	16189587	MAGEB17
ENSG00000182809	8.844740944	8.704198827	protein_coding	14	105939299	105946499	CRIP2
ENSG00000182810	7.648344073	7.82111279	protein_coding	16	68055179	68057770	DDX28
ENSG00000182814	1.799000381	0.390640832	pseudogene	2	84517806	84519326	FUNDC2P2
ENSG00000182827	9.190229706	9.318542753	protein_coding	1	226332380	226374431	ACBD3
ENSG00000182831	9.223526019	8.888724634	protein_coding	16	9185537	9213536	C16orf72
ENSG00000182836	0.499066092	0	protein_coding	5	41307056	41510730	PLCXD3
ENSG00000182841	7.115078652	7.346135265	pseudogene	22	42951229	42978044	
ENSG00000182851	0.499066092	2.039052734	protein_coding	8	144295068	144299044	GPIHBP1
ENSG00000182853	2.238690726	1.518964905	protein_coding	17	4688581	4689729	VMO1
ENSG00000182858	8.433338631	8.319396705	protein_coding	22	50296867	50312106	ALG12
ENSG00000182866	2.238690726	0	protein_coding	1	32716840	32751766	LCK
ENSG00000182870	7.277615649	6.703819132	protein_coding	12	132680924	132905935	GALNT9
ENSG00000182871	9.62945337	9.905561226	protein_coding	21	46825052	46933634	COL18A1
ENSG00000182872	7.850461307	8.222087975	protein_coding	X	47004268	47046212	RBM10
ENSG00000182873	6.204385613	5.751299057	antisense	1	2113233	2115828	
ENSG00000182885	2.238690726	1.16600992	protein_coding	16	57702099	57723975	GPR97
ENSG00000182890	1.799000381	1.802319292	protein_coding	X	120181462	120183794	GLUD2
ENSG00000182896	0.499066092	0	protein_coding	17	7258442	7260538	TMEM95
ENSG00000182899	11.5649357	11.98034419	protein_coding	3	197676858	197683481	RPL35A
ENSG00000182901	0	1.802319292	protein_coding	1	240931554	241520530	RGS7
ENSG00000182902	4.618121645	3.279072565	protein_coding	22	18043139	18073647	SLC25A18
ENSG00000182903	7.053569556	6.95589788	protein_coding	4	419604	492945	ZNF721
ENSG00000182909	7.110771062	6.819555981	protein_coding	19	54972981	54974894	LENG9
ENSG00000182912	6.103936836	6.149127429	protein_coding	21	45937099	45945835	C21orf90
ENSG00000182919	7.409722215	7.850567963	protein_coding	11	93474757	93497915	C11orf54
ENSG00000182923	7.19863491	7.577474828	protein_coding	3	134204585	134293859	CEP63
ENSG00000182931	2.35979773	4.29708397	protein_coding	20	44313292	44333658	WFDC10B

ENSG00000182934	11.05516955	11.04432742	protein_coding	11	126132833	126139039	SRPR
ENSG00000182944	11.56729548	11.40502138	protein_coding	22	29663998	29696515	EWSR1
ENSG00000182950	1.407729925	0.697730409	protein_coding	15	76016318	76020029	ODF3L1
ENSG00000182952	9.402463961	9.917208266	protein_coding	6	26538572	26547160	HMGN4
ENSG00000182957	8.54291017	8.048254208	protein_coding	13	24734861	24896096	SPATA13
ENSG00000182963	8.600881464	8.490068116	protein_coding	17	42875816	42908179	GJC1
ENSG00000182965	0	0.697730409	pseudogene	7	112160543	112161725	NPM1P14
ENSG00000182973	8.036821332	8.197845922	protein_coding	3	32726637	32815367	CNOT10
ENSG00000182979	9.3522601	9.401152631	protein_coding	14	105886157	105937066	MTA1
ENSG00000182985	7.115078652	9.511607983	protein_coding	11	115039938	115375675	CADM1
ENSG00000182986	8.803966282	8.958022094	protein_coding	19	53379425	53393592	ZNF320
ENSG00000182993	4.77879189	4.857374129	protein_coding	12	14956506	15059520	C12orf60
ENSG00000183010	10.60116605	10.77230949	protein_coding	17	79890269	79895161	PYCR1
ENSG00000183011	8.678607554	9.855105805	protein_coding	17	7760005	7761172	LSMD1
ENSG00000183016	0.869158192	0.390640832	antisense	7	150887960	150902581	IQCA1P1
ENSG00000183018	5.270908378	7.754113435	protein_coding	17	4402129	4443228	SPNS2
ENSG00000183019	1.16343121	0.697730409	protein_coding	19	7741943	7744719	C19orf59
ENSG00000183020	9.633212506	9.448796166	protein_coding	11	924894	1012239	AP2A2
ENSG00000183022	0.499066092	0	pseudogene	2	231437882	231438524	TPM3P8
ENSG00000183023	0	0.950786998	protein_coding	2	40339286	40838193	SLC8A1
ENSG00000183032	0.869158192	0.950786998	protein_coding	14	37147636	37642071	SLC25A21
ENSG00000183044	4.883502971	5.486957566	protein_coding	16	8768422	8878432	ABAT
ENSG00000183048	10.22779886	10.15809388	protein_coding	17	79670404	79688041	MRPL12
ENSG00000183049	8.790563466	7.929979971	protein_coding	10	12391481	12877545	CAMK1D
ENSG00000183055	0.499066092	1.353254395	pseudogene	10	60475314	60476017	
ENSG00000183060	5.40360309	5.57429348	protein_coding	15	100255906	100273766	LYSMD4
ENSG00000183066	3.652182994	4.29708397	protein_coding	22	42394729	42454460	WBP2NL
ENSG00000183072	0	0.390640832	protein_coding	5	172659112	172662360	NKX2-5
ENSG00000183077	7.599989788	7.378849871	protein_coding	17	76183398	76203782	AFMID
ENSG00000183087	8.14188337	9.008548131	protein_coding	13	114523524	114567046	GAS6
ENSG00000183090	0	0.390640832	protein_coding	4	144498455	144621828	FREM3
ENSG00000183091	3.830412367	4.250702764	protein_coding	2	152341850	152591001	NEB
ENSG00000183092	5.909333162	4.406904905	protein_coding	14	101003486	101053750	BEGAIN
ENSG00000183098	5.498982484	6.50081754	protein_coding	13	93879095	95055812	GPC6
ENSG00000183111	4.062237333	3.608232228	protein_coding	5	148931510	149014531	ARHGEF37
ENSG00000183128	3.146112541	3.368666104	protein_coding	10	105232561	105238997	CALHM3
ENSG00000183134	3.077135474	2.912743273	protein_coding	11	60618413	60623444	PTGDR2
ENSG00000183137	6.414691513	6.525377513	protein_coding	6	109416313	109485135	CEP57L1
ENSG00000183150	4.883502971	5.163066621	protein_coding	12	12813825	12849141	GPR19
ENSG00000183153	4.292665995	3.679994897	protein_coding	17	38517235	38520067	GJD3
ENSG00000183154	0	0.390640832	protein_coding	8	37592279	37594944	
ENSG00000183155	6.856698814	7.258020107	protein_coding	1	202848085	202858263	RABIF
ENSG00000183160	2.238690726	2.242360793	protein_coding	12	108983622	108992096	TMEM119
ENSG00000183161	6.814942123	6.862599506	protein_coding	11	22644079	22647387	FANCF
ENSG00000183171	1.616589159	1.353254395	pseudogene	X	149282609	149284751	
ENSG00000183172	7.323008624	7.76240224	protein_coding	22	42475695	42532261	C22orf32
ENSG00000183186	3.698846687	4.226940739	protein_coding	19	405443	409170	C2CD4C
ENSG00000183196	6.598204782	4.777836593	protein_coding	16	75510949	75529282	CHST6
ENSG00000183199	4.026095388	3.027231696	pseudogene	4	88812995	88815167	HSP90AB3P
ENSG00000183206	0	1.16600992	protein_coding	18	14507338	14543599	POTEC
ENSG00000183207	10.81418283	10.92248464	protein_coding	19	49497156	49519182	RUVBL2
ENSG00000183208	4.642199401	4.528525526	protein_coding	15	90777040	90785315	C15orf58
ENSG00000183239	0.869158192	0.950786998	pseudogene	6	44056979	44057433	
ENSG00000183246	0.869158192	0.390640832	protein_coding	22	21899646	21905750	RIMBP3C
ENSG00000183248	4.062237333	2.242360793	protein_coding	19	7933605	7939326	
ENSG00000183250	4.6658819	3.570966319	protein_coding	21	46352729	46359828	C21orf67
ENSG00000183251	0.499066092	0.697730409	protein_coding	11	5322244	5323226	OR51B4
ENSG00000183255	11.29093363	11.25044641	protein_coding	21	46269500	46293752	PTTG1IP
ENSG00000183258	9.446713374	9.474880612	protein_coding	5	176938578	176944470	DDX41
ENSG00000183260	6.068837212	6.847095496	protein_coding	20	62492566	62494341	ABHD16B
ENSG00000183281	3.448790144	3.570966319	protein_coding	2	87229682	87248975	PLGLB1
ENSG00000183283	10.94938384	10.75059971	protein_coding	12	51632076	51640501	DAZAP2
ENSG00000183287	7.778963161	6.403479675	protein_coding	18	57102442	57364574	CCBE1

ENSG00000183291	9.56636357	9.864761738	protein_coding	1	87328132	87380107	
ENSG00000183298	2.238690726	2.144285137	pseudogene	1	102251892	102254059	
ENSG00000183307	3.004694206	3.133298822	protein_coding	22	17597189	17602257	CECR6
ENSG00000183308	0	0.390640832	antisense	2	201827986	201873825	
ENSG00000183309	7.751556534	7.758263791	protein_coding	8	144718183	144738588	ZNF623
ENSG00000183317	5.986594255	5.784235782	protein_coding	1	38179552	38230805	EPHA10
ENSG00000183323	6.820228284	6.720932357	protein_coding	5	68576002	68628636	CCDC125
ENSG00000183324	0.869158192	0.697730409	protein_coding	15	73735499	73852355	C15orf60
ENSG00000183337	9.522446643	9.333223486	protein_coding	X	39909068	40036582	BCOR
ENSG00000183340	7.04456449	7.593823731	protein_coding	11	96123153	96240738	JRKL
ENSG00000183346	0.499066092	1.518964905	protein_coding	10	63422719	63526524	C10orf107
ENSG00000183347	0	0.390640832	protein_coding	1	89829605	89853719	GBP6
ENSG00000183354	6.548110424	6.398170093	protein_coding	9	5881596	6007901	KIAA2026
ENSG00000183378	0	0.390640832	polymorphic_pse	11	7710669	7727968	OVCH2
ENSG00000183379	3.077135474	2.851901313	protein_coding	14	74872596	74892805	SYNDIG1L
ENSG00000183386	8.173205795	8.410554709	protein_coding	1	38462442	38471278	FHL3
ENSG00000183395	1.16343121	0.697730409	protein_coding	12	102590237	102591623	PMCH
ENSG00000183396	1.960915222	1.518964905	protein_coding	3	48658192	48659288	TMEM89
ENSG00000183397	7.19863491	7.281279771	protein_coding	19	3539155	3544028	C19orf71
ENSG00000183401	5.763291843	6.505763096	protein_coding	19	11457181	11465619	CCDC159
ENSG00000183405	4.131913373	4.15321211	pseudogene	17	26794814	26795460	RPS7P1
ENSG00000183421	9.614318105	8.517261232	protein_coding	21	43159529	43187266	RIPK4
ENSG00000183423	1.16343121	3.027231696	protein_coding	4	110772486	110793471	LRIT3
ENSG00000183426	4.923348194	5.57429348	protein_coding	16	14844670	15045931	NPIP
ENSG00000183431	9.988771077	10.1446815	protein_coding	1	38422647	38456593	SF3A3
ENSG00000183439	4.026095388	4.744751758	protein_coding	4	165875598	165898820	TRIM61
ENSG00000183444	4.842525946	5.059245468	pseudogene	7	97595402	97596335	
ENSG00000183454	3.950976309	5.709041716	protein_coding	16	9852376	10276611	GRIN2A
ENSG00000183458	5.158541613	5.564846512	pseudogene	16	15005408	15029565	
ENSG00000183461	0	1.802319292	protein_coding	X	52511761	52516905	XAGE1C
ENSG00000183473	0.499066092	1.16600992	protein_coding	22	37600278	37608362	SSTR3
ENSG00000183474	6.014532757	5.784235782	protein_coding	5	68856035	68890550	GTF2H2C
ENSG00000183475	6.965663283	6.866449614	protein_coding	15	101142739	101191910	ASB7
ENSG00000183476	0	0.390640832	protein_coding	15	78370150	78397251	SH2D7
ENSG00000183479	4.381167248	3.679994897	protein_coding	X	152710178	152736045	TREX2
ENSG00000183484	4.962122459	3.183544561	protein_coding	14	105515728	105531782	GPR132
ENSG00000183486	7.527512071	8.106387334	protein_coding	21	42733870	45746674	MX2
ENSG00000183495	10.46605656	10.33071191	protein_coding	12	132434505	132565005	EP400
ENSG00000183496	4.491309013	5.57429348	protein_coding	15	82334119	82338482	MEX3B
ENSG00000183506	6.393570237	6.539915281	pseudogene	22	21827289	21871822	PI4KAP2
ENSG00000183508	6.393570237	6.24096575	protein_coding	1	118148556	118170994	FAM46C
ENSG00000183513	7.311793575	7.373448694	protein_coding	2	99215773	99224978	COA5
ENSG00000183520	8.492479328	8.578395649	protein_coding	1	38474930	38490496	UTP11L
ENSG00000183527	8.568265199	8.853271458	protein_coding	21	40546695	40555777	PSMG1
ENSG00000183530	10.4388224	10.17060441	protein_coding	22	32072242	32146126	PRR14L
ENSG00000183535	1.16343121	2.334191469	processed_transc	21	46839631	46844985	COL18A1-AS1
ENSG00000183542	0	1.353254395	protein_coding	12	10559983	10562356	KLRC4
ENSG00000183558	2.57521082	3.748356452	protein_coding	1	149813505	149814478	HIST2H2AA3
ENSG00000183562	2.10647801	2.420525079	protein_coding	11	3011093	3012563	
ENSG00000183569	5.695874189	5.564846512	protein_coding	22	42949623	42970388	SERHL2
ENSG00000183570	5.563445526	6.077857763	protein_coding	21	47063608	47362368	PCBP3
ENSG00000183571	0	0.697730409	protein_coding	15	99511459	99551024	PGPEP1L
ENSG00000183576	9.775389681	8.862939634	protein_coding	14	99864083	99947216	SETD3
ENSG00000183578	4.437288965	4.363974406	protein_coding	15	51348795	51397473	TNFAIP8L3
ENSG00000183579	8.724437638	8.193254836	protein_coding	22	29279580	29453475	ZNRF3
ENSG00000183580	0	0.390640832	protein_coding	5	15500305	15939900	FBXL7
ENSG00000183597	8.116326397	8.099860884	protein_coding	22	20004537	20053449	C22orf25
ENSG00000183604	2.671945279	3.133298822	pseudogene	16	30234230	30346695	
ENSG00000183605	7.993024472	8.262094789	protein_coding	10	120900279	120925179	SFXN4
ENSG00000183615	3.989024711	5.059245468	protein_coding	1	32712834	32714457	FAM167B
ENSG00000183617	8.329659793	9.179226353	protein_coding	19	3762665	3767562	MRPL54
ENSG00000183621	6.496213887	7.370740503	protein_coding	10	31133563	31320866	ZNF438
ENSG00000183624	9.248248601	8.775550005	protein_coding	3	128997671	129024146	C3orf37

ENSG00000183625	1.960915222	0.697730409	protein_coding	3	46205096	46308197	CCR3
ENSG00000183628	5.239686603	5.974624386	protein_coding	22	18893541	18901751	DGCR6
ENSG00000183629	1.407729925	1.16600992	protein_coding	15	28764757	28778160	GOLGA8G
ENSG00000183638	3.830412367	3.232099092	protein_coding	8	10463859	10569697	RP1L1
ENSG00000183647	6.830742851	6.387491945	protein_coding	19	58111253	58119637	ZNF530
ENSG00000183648	7.30049066	6.980975423	protein_coding	14	92582466	92588261	NDUFB1
ENSG00000183655	6.462804234	6.419292172	protein_coding	15	86302554	86338261	KLHL25
ENSG00000183657	4.230497448	3.32456471	protein_coding	3	171509580	171527714	
ENSG00000183665	7.281453427	7.48283249	protein_coding	8	125463048	125474391	TRMT12
ENSG00000183666	5.60078458	5.47691899	pseudogene	5	21341942	21544886	
ENSG00000183668	2.762599152	2.579085888	protein_coding	19	43757434	43773682	PSG9
ENSG00000183671	3.830412367	4.489113623	protein_coding	2	207040040	207082771	GPR1
ENSG00000183674	1.616589159	2.242360793	processed_transc	6	10429488	10435107	LINC00518
ENSG00000183682	1.960915222	2.721932731	protein_coding	1	39957318	39991607	BMP8A
ENSG00000183684	9.776069902	9.695451059	protein_coding	17	79845713	79849462	ALYREF
ENSG00000183688	9.360447332	9.740202333	protein_coding	17	289769	295730	FAM101B
ENSG00000183690	4.942865586	4.528525526	protein_coding	X	44007128	44202923	EFHC2
ENSG00000183691	8.002356002	8.632529738	protein_coding	17	54671060	54672951	NOG
ENSG00000183696	10.75847887	10.11059465	protein_coding	7	48128225	48148330	UPP1
ENSG00000183718	5.588445295	6.057793599	protein_coding	5	180681417	180688119	TRIM52
ENSG00000183722	6.585843317	6.668971382	protein_coding	13	39917029	40177665	LHFP
ENSG00000183723	8.831713317	8.605140645	protein_coding	16	66648653	66730610	CMTM4
ENSG00000183726	9.16240384	9.519576271	protein_coding	1	25664408	25688852	TMEM50A
ENSG00000183729	1.799000381	1.16600992	protein_coding	8	53850991	53853677	NPBWR1
ENSG00000183735	8.193715877	8.320800493	protein_coding	12	64845660	64895888	TBK1
ENSG00000183741	11.3653694	11.01275647	protein_coding	22	39257455	39268319	CBX6
ENSG00000183742	2.35979773	0	protein_coding	7	20176020	20257027	MACC1
ENSG00000183751	10.37065269	10.36683522	protein_coding	16	2022038	2032934	TBL3
ENSG00000183760	3.652182994	3.279072565	protein_coding	19	39574945	39602080	
ENSG00000183762	8.759256101	7.641787375	protein_coding	22	29469066	29564321	KREMEN1
ENSG00000183763	6.979904289	7.243290054	protein_coding	3	49866034	49894007	TRAIP
ENSG00000183765	8.684416567	8.995422414	protein_coding	22	29083731	29138410	CHEK2
ENSG00000183770	5.588445295	5.404622322	protein_coding	3	138663066	138665982	FOXL2
ENSG00000183773	3.911897206	3.183544561	protein_coding	22	21319396	21335649	AIFM3
ENSG00000183775	1.407729925	1.518964905	protein_coding	5	143550396	143856944	KCTD16
ENSG00000183778	1.16343121	0	protein_coding	21	40928369	41034816	B3GALT5
ENSG00000183779	7.55947245	7.103479673	protein_coding	8	37553269	37557537	ZNF703
ENSG00000183785	1.616589159	1.16600992	protein_coding	22	18593097	18614498	TUBA8
ENSG00000183791	0	1.16600992	protein_coding	18	44554573	44556449	TCEB3C
ENSG00000183793	0.499066092	0.950786998	protein_coding	16	15457516	15474904	
ENSG00000183798	0.869158192	1.925536307	protein_coding	20	39988606	39995467	EMILIN3
ENSG00000183801	0	1.353254395	protein_coding	11	7506619	7532608	OLFML1
ENSG00000183808	7.81647827	7.862183683	protein_coding	8	94741584	94753245	RBM12B
ENSG00000183813	1.407729925	1.802319292	protein_coding	3	32993066	32997841	CCR4
ENSG00000183814	6.75547892	7.138927266	protein_coding	1	226418850	226497570	LIN9
ENSG00000183826	6.335686705	6.520498852	protein_coding	6	38136227	38607924	BTBD9
ENSG00000183828	6.979904289	6.480864016	protein_coding	14	105639275	105647660	NUDT14
ENSG00000183831	0.499066092	0	protein_coding	1	173578700	173639001	ANKRD45
ENSG00000183833	5.684325135	6.2290532	protein_coding	3	119421869	119485949	C3orf15
ENSG00000183840	5.938793061	4.841813558	protein_coding	2	133174147	133404132	GPR39
ENSG00000183844	0.499066092	1.518964905	protein_coding	21	42676139	42729358	FAM3B
ENSG00000183850	4.517578978	4.585695337	protein_coding	19	23299777	23332763	ZNF730
ENSG00000183853	11.56591941	11.75615959	protein_coding	1	157963063	158070052	KIRREL
ENSG00000183856	10.58572949	10.90994789	protein_coding	1	156495197	156542396	IQGAP3
ENSG00000183864	9.828162765	9.747527757	protein_coding	22	41829496	41843027	TOB2
ENSG00000183873	0	0.950786998	protein_coding	3	38589548	38691164	SCN5A
ENSG00000183876	5.763291843	5.611474642	protein_coding	5	149675906	149718870	ARSI
ENSG00000183889	1.407729925	1.802319292	protein_coding	16	16411301	16444447	
ENSG00000183891	5.417620896	5.236305879	protein_coding	2	20096404	20101747	TTC32
ENSG00000183900	0	1.925536307	pseudogene	5	72742083	72744352	
ENSG00000183908	0	0.697730409	protein_coding	11	56949221	56959191	LRRC55
ENSG00000183911	0.499066092	0.697730409	pseudogene	X	100594905	100595922	RPL21P132
ENSG00000183914	10.58378825	9.974965329	protein_coding	17	7623039	7737058	DNAH2

ENSG00000183929	6.068837212	6.563824016	pseudogene	1	228744885	228788150	DUSP5P
ENSG00000183935	3.554067925	3.133298822	pseudogene	12	13152812	13157764	HTR7P1
ENSG00000183943	8.023133844	7.760334499	protein_coding	X	3522411	3631649	PRKX
ENSG00000183955	8.577659618	8.500015927	protein_coding	12	123868320	123893905	SETD8
ENSG00000183963	10.59578202	10.42117962	protein_coding	22	31460091	31500610	SMTN
ENSG00000183971	2.35979773	1.16600992	protein_coding	16	2059927	2070756	NPW
ENSG00000183977	2.671945279	2.334191469	protein_coding	3	20021453	20053822	PP2D1
ENSG00000183978	7.413224588	8.170077662	protein_coding	17	40949653	40950704	CCDC56
ENSG00000183979	2.10647801	1.925536307	protein_coding	17	79860072	79860780	NPB
ENSG00000184007	11.19143908	10.87143099	protein_coding	1	32372022	32410457	PTP4A2
ENSG00000184009	15.11695939	14.776564	protein_coding	17	79476999	79479827	ACTG1
ENSG00000184012	6.103936836	5.840125386	protein_coding	21	42836478	42903043	TMPRSS2
ENSG00000184014	10.43796299	10.78431559	protein_coding	11	9160372	9286937	DENND5A
ENSG00000184047	5.774228067	5.908941696	protein_coding	12	122692210	122712081	DIABLO
ENSG00000184056	7.471499737	7.57041104	protein_coding	15	91541646	91565833	VPS33B
ENSG00000184058	2.847891871	1.802319292	protein_coding	22	19744226	19771116	TBX1
ENSG00000184060	0.869158192	1.925536307	protein_coding	17	29248698	29286340	ADAP2
ENSG00000184068	6.236372259	5.506827567	processed_transc	22	42227219	42230669	
ENSG00000184076	9.807683852	10.37022883	protein_coding	22	30163352	30166402	UQCR10
ENSG00000184083	8.464056506	9.103767504	protein_coding	X	54094757	54209714	FAM120C
ENSG00000184084	0	1.16600992	pseudogene	5	73099121	73099424	
ENSG00000184100	1.16343121	1.667587519	pseudogene	3	159818478	159820661	
ENSG00000184108	0	0.390640832	protein_coding	4	189060573	189068897	TRIML1
ENSG00000184110	6.407685389	6.926693689	protein_coding	16	28699879	28747051	EIF3C
ENSG00000184111	0.499066092	0.697730409	pseudogene	10	94356609	94357044	
ENSG00000184113	2.671945279	2.851901313	protein_coding	22	19510547	19515068	CLDN5
ENSG00000184117	10.64390259	10.54945594	protein_coding	22	29950797	29977326	NIPSNAP1
ENSG00000184144	0	0.697730409	protein_coding	1	205012325	205047627	CNTN2
ENSG00000184154	6.188122351	6.174194902	protein_coding	11	71791382	71821828	LRTOMT
ENSG00000184156	1.16343121	0	protein_coding	8	133139193	133493200	KCNQ3
ENSG00000184160	4.322773689	4.101879561	protein_coding	4	3768075	3770251	ADRA2C
ENSG00000184162	8.394884042	8.673761454	protein_coding	19	19312224	19314220	NR2C2AP
ENSG00000184163	4.409500985	5.339632724	protein_coding	1	1177826	1182102	FAM132A
ENSG00000184164	8.487504052	8.924251218	protein_coding	22	50311815	50321188	CRELD2
ENSG00000184178	7.811178326	7.637287497	protein_coding	4	53739149	54232242	SCFD2
ENSG00000184182	7.326727684	7.119700263	protein_coding	2	238875469	238951236	UBE2F
ENSG00000184185	6.598204782	7.8660349	protein_coding	17	21279699	21320404	KCNJ12
ENSG00000184188	0.499066092	0.390640832	pseudogene	5	79595497	79598666	
ENSG00000184194	5.141750194	6.167968749	protein_coding	X	53078273	53109797	GPR173
ENSG00000184203	8.396654395	8.206984494	protein_coding	3	195241221	195270209	PPP1R2
ENSG00000184205	8.599344916	8.410554709	protein_coding	X	53111549	53117722	TSPYL2
ENSG00000184206	1.799000381	1.925536307	protein_coding	15	84904525	84914120	GOLGA6L4
ENSG00000184207	8.717371959	8.741364159	protein_coding	16	2261998	2264808	PGP
ENSG00000184208	7.930852348	8.42106413	protein_coding	22	42084943	42094140	C22orf46
ENSG00000184209	6.640654398	6.544728831	protein_coding	12	123942188	123957701	SNRNP35
ENSG00000184210	0.499066092	0.697730409	protein_coding	X	69397333	69425395	DGAT2L6
ENSG00000184216	12.00056824	11.37931557	protein_coding	X	153275951	153285431	IRAK1
ENSG00000184220	8.814599602	8.849385973	protein_coding	3	99536678	99897447	C3orf26
ENSG00000184224	1.616589159	0.390640832	protein_coding	11	67370351	67374177	C11orf72
ENSG00000184226	1.407729925	1.16600992	protein_coding	13	66876967	67804468	PCDH9
ENSG00000184227	4.77879189	5.294620694	protein_coding	14	74003818	74010498	ACOT1
ENSG00000184232	10.82276754	10.42183387	protein_coding	11	120081475	120101001	OAF
ENSG00000184247	5.995967334	5.593003837	pseudogene	17	77910551	77910992	
ENSG00000184254	6.9512803	6.002883551	protein_coding	15	101402129	101456831	ALDH1A3
ENSG00000184258	1.16343121	0.950786998	protein_coding	X	139865425	139866723	CDR1
ENSG00000184260	2.238690726	3.64455972	protein_coding	1	149858525	149858961	HIST2H2AC
ENSG00000184261	1.407729925	1.353254395	protein_coding	2	47747910	47798078	KCNK12
ENSG00000184270	0.869158192	0.950786998	protein_coding	1	149859019	149859466	HIST2H2AB
ENSG00000184271	6.489593565	6.460630645	protein_coding	12	51580719	51611477	POU6F1
ENSG00000184274	1.407729925	2.144285137	lincRNA	21	46720160	46725172	LINC00315
ENSG00000184277	7.053569556	7.688207601	protein_coding	15	102161847	102192594	TM2D3
ENSG00000184281	8.856366065	8.971490926	protein_coding	11	2421718	2425106	TSSC4
ENSG00000184292	10.15958131	9.554600493	protein_coding	1	59041099	59043166	TACSTD2

ENSG00000184302	0.499066092	0	protein_coding	14	60975669	60979568	SIX6
ENSG00000184304	4.437288965	7.357122767	protein_coding	14	30045687	30661104	PRKD1
ENSG00000184305	1.799000381	0	protein_coding	4	91048686	92523064	FAM190A
ENSG00000184307	6.041940478	6.365895578	protein_coding	3	113666748	113684248	ZDHHC23
ENSG00000184313	2.238690726	1.802319292	protein_coding	1	55107459	55207981	HEATR8
ENSG00000184319	6.290697143	6.89311646	pseudogene	22	51195376	51239737	
ENSG00000184343	4.097495944	4.547834947	protein_coding	X	153041867	153051187	SRPK3
ENSG00000184347	0	0.390640832	protein_coding	5	168088745	168728133	SLIT3
ENSG00000184348	2.35979773	2.420525079	protein_coding	6	27805658	27806117	HIST1H2AK
ENSG00000184349	7.865879816	7.534563804	protein_coding	5	106712590	107006596	EFNA5
ENSG00000184357	3.146112541	3.781359661	protein_coding	6	27834570	27835359	HIST1H1B
ENSG00000184361	1.960915222	0.950786998	protein_coding	17	43331760	43339479	C17orf46
ENSG00000184363	10.10106595	9.035301979	protein_coding	11	392614	404908	PKP3
ENSG00000184368	2.928422289	1.353254395	protein_coding	X	20024831	20135114	MAP7D2
ENSG00000184371	11.05936676	11.12367977	protein_coding	1	110452864	110473614	CSF1
ENSG00000184374	0	0.697730409	protein_coding	8	120007691	120118821	COLEC10
ENSG00000184378	5.255381949	5.840125386	protein_coding	3	169484713	169487683	
ENSG00000184381	7.484876093	7.598461017	protein_coding	22	38507502	38588107	PLA2G6
ENSG00000184384	7.921045056	8.375860541	protein_coding	11	95709762	96076382	MAML2
ENSG00000184389	0.499066092	0.697730409	pseudogene	1	33772367	33778491	A3GALT2P
ENSG00000184402	7.985986001	8.251828705	protein_coding	20	60718822	60757540	SS18L1
ENSG00000184408	0	0.390640832	protein_coding	7	119913722	120390385	KCND2
ENSG00000184414	1.407729925	0.390640832	pseudogene	7	100167754	100168759	
ENSG00000184428	8.586993258	8.106387334	protein_coding	8	144386554	144442149	TOP1MT
ENSG00000184432	11.20008193	11.24620466	protein_coding	3	139074442	139108574	COPB2
ENSG00000184434	1.16343121	1.518964905	protein_coding	9	26993134	27005691	LRRC19
ENSG00000184436	7.8607586	8.351343215	protein_coding	22	21353393	21356485	THAP7
ENSG00000184441	5.223818625	5.446377228	processed_transc	21	45751117	45755734	
ENSG00000184445	8.922987828	8.98660512	protein_coding	12	123011793	123110926	KNTC1
ENSG00000184451	3.393122761	3.993387124	protein_coding	17	40830907	40833861	CCR10
ENSG00000184459	0	0.697730409	protein_coding	22	32809834	32860471	BPIFC
ENSG00000184465	8.116326397	8.301022059	protein_coding	6	169857307	170102159	WDR27
ENSG00000184470	8.546104025	9.007676788	protein_coding	22	19863040	19929359	TXNRD2
ENSG00000184471	0.499066092	0	protein_coding	16	1140005	1146244	C1QTNF8
ENSG00000184481	6.809636522	7.392265003	protein_coding	X	70316047	70323385	FOXO4
ENSG00000184486	0.869158192	1.16600992	protein_coding	6	99282580	99286660	POU3F2
ENSG00000184489	7.19863491	6.703819132	protein_coding	8	142402093	142441620	PTP4A3
ENSG00000184492	1.616589159	1.353254395	protein_coding	2	114256661	114258728	FOXD4L1
ENSG00000184497	5.858855738	6.155435249	protein_coding	13	114462216	114514926	FAM70B
ENSG00000184500	6.912209543	8.270836518	protein_coding	3	93591881	93692910	PROS1
ENSG00000184507	1.960915222	1.353254395	protein_coding	15	34635516	34649938	C15orf55
ENSG00000184508	3.335220907	4.961837817	protein_coding	15	91474148	91475799	HDDC3
ENSG00000184515	3.274897671	1.353254395	protein_coding	X	101408680	101411029	BEX5
ENSG00000184517	7.587643674	7.73107057	protein_coding	16	75182390	75206134	ZFP1
ENSG00000184524	1.407729925	2.912743273	protein_coding	11	787104	790123	CEND1
ENSG00000184530	3.211941663	4.872768657	protein_coding	6	127840600	127912962	C6orf58
ENSG00000184545	7.115078652	7.343375265	protein_coding	11	1575274	1593150	DUSP8
ENSG00000184551	6.522395495	5.816437314	pseudogene	17	80206943	80209703	
ENSG00000184557	8.124895791	6.915587884	protein_coding	17	76352864	76356158	SOCS3
ENSG00000184560	1.407729925	2.144285137	protein_coding	17	7328935	7330884	C17orf74
ENSG00000184564	6.907250452	4.021284656	protein_coding	13	86366925	86373623	SLITRK6
ENSG00000184574	3.004694206	1.802319292	protein_coding	12	6728001	6750426	LPAR5
ENSG00000184575	11.20589934	11.38503041	protein_coding	12	64798130	64844907	XPOT
ENSG00000184584	9.084367772	8.696649474	protein_coding	5	138855119	138862520	TMEM173
ENSG00000184588	0	0.697730409	protein_coding	1	66258197	66840259	PDE4B
ENSG00000184596	3.335220907	3.368666104	pseudogene	X	48797263	48798079	
ENSG00000184599	3.911897206	3.232099092	protein_coding	1	113263041	113269857	FAM19A3
ENSG00000184602	7.969428119	6.398170093	protein_coding	16	11762270	11773015	SNN
ENSG00000184608	0	0.390640832	protein_coding	8	11225911	11296167	C8orf12
ENSG00000184611	0	0.390640832	protein_coding	2	163227917	163695240	KCNH7
ENSG00000184612	1.616589159	0	pseudogene	6	17531125	17531867	
ENSG00000184616	2.928422289	3.027231696	pseudogene	7	74320406	74331163	
ENSG00000184617	0.869158192	0	pseudogene	20	45113100	45121279	ZNF840

ENSG00000184619	5.718698586	6.615068802	protein_coding	17	8271955	8280029	KRBA2
ENSG00000184634	9.070017088	9.512222494	protein_coding	X	70338406	70362303	MED12
ENSG00000184635	7.624369505	8.365015357	protein_coding	19	20011722	20046860	ZNF93
ENSG00000184640	11.8864536	11.16978367	protein_coding	17	75277492	75496674	9-sep
ENSG00000184661	8.359012976	8.554729165	protein_coding	8	25316513	25365436	CDCA2
ENSG00000184669	0.869158192	0.390640832	pseudogene	11	17035545	17074583	OR7E14P
ENSG00000184675	8.092494681	7.716212954	protein_coding	X	63404997	63425624	FAM123B
ENSG00000184677	9.000704568	8.995422414	protein_coding	1	22778344	22857650	ZBTB40
ENSG00000184678	7.484876093	6.525377513	protein_coding	1	149856010	149858232	HIST2H2BE
ENSG00000184682	2.10647801	2.420525079	protein_coding	11	1910375	1912084	C11orf89
ENSG00000184697	0.869158192	0.950786998	protein_coding	16	3064713	3070072	CLDN6
ENSG00000184702	4.026095388	3.133298822	protein_coding	22	19701987	19712295	5-sep
ENSG00000184708	8.596266901	8.709567138	protein_coding	22	31835349	31892094	EIF4ENIF1
ENSG00000184709	0.499066092	0	protein_coding	9	140063210	140064503	LRRC26
ENSG00000184719	6.138202758	6.117167886	protein_coding	10	90033621	90344287	RNLS
ENSG00000184730	2.471521042	2.039052734	protein_coding	16	28505970	28510291	APOBR
ENSG00000184743	10.25147099	10.35557934	protein_coding	11	63391559	63439393	ATL3
ENSG00000184752	7.773523328	8.119352325	protein_coding	12	95290831	95397546	NDUFA12
ENSG00000184774	0	0.390640832	protein_coding	1	202789394	202796353	
ENSG00000184779	1.16343121	0.390640832	protein_coding	15	82821158	82824972	RPS17
ENSG00000184785	6.212449006	6.424524724	protein_coding	X	134124968	134126501	CXorf69
ENSG00000184786	1.616589159	1.353254395	protein_coding	6	170140210	170151655	TCTE3
ENSG00000184787	9.944072862	9.754816173	protein_coding	21	46188955	46221934	UBE2G2
ENSG00000184788	5.018397273	5.163066621	protein_coding	X	84347293	84364054	SATL1
ENSG00000184792	8.724437638	8.395978102	protein_coding	22	31089769	31303811	OSBP2
ENSG00000184825	0.869158192	1.518964905	protein_coding	6	27114861	27115317	HIST1H2AH
ENSG00000184828	3.502389126	2.971122874	protein_coding	18	45553640	45935663	ZBTB7C
ENSG00000184831	7.440941622	8.019149911	protein_coding	X	23851470	23926057	APOO
ENSG00000184838	0.869158192	0.697730409	protein_coding	5	119799973	120023027	PRR16
ENSG00000184840	10.96587395	11.31684713	protein_coding	5	177019159	177023125	TMED9
ENSG00000184844	0.499066092	0.697730409	pseudogene	X	153106637	153107540	
ENSG00000184845	0	1.353254395	protein_coding	5	174867675	174871163	DRD1
ENSG00000184857	6.872051157	7.240325909	protein_coding	16	8874241	8891505	TMEM186
ENSG00000184863	9.736077124	9.20301961	protein_coding	7	155437145	155574179	RBM33
ENSG00000184867	4.999881834	5.317302248	protein_coding	X	100910267	100914876	ARMCX2
ENSG00000184886	7.484876093	7.335063441	protein_coding	17	34890847	34895150	PIGW
ENSG00000184887	8.894453552	8.083414408	protein_coding	14	105714827	105717430	BTBD6
ENSG00000184897	9.9349646	9.829200569	protein_coding	3	129033615	129035120	H1FX
ENSG00000184898	6.086493765	5.717592784	protein_coding	2	152104454	152118393	RBM43
ENSG00000184900	9.999878142	10.04882736	protein_coding	21	46191374	46238694	SUMO3
ENSG00000184903	5.346128795	5.41517489	protein_coding	7	110303110	111202573	IMMP2L
ENSG00000184905	0	0.390640832	protein_coding	X	101380660	101382683	TCEAL2
ENSG00000184906	0.499066092	0	pseudogene	9	42845768	42859085	
ENSG00000184908	2.671945279	1.667587519	protein_coding	1	16370247	16383803	CLCNKB
ENSG00000184916	9.312488937	6.889336956	protein_coding	14	105607318	105635161	JAG2
ENSG00000184922	6.912209543	5.638744877	protein_coding	17	43299292	43324681	FMNL1
ENSG00000184923	0.869158192	2.039052734	protein_coding	10	88985205	88994912	FAM22A
ENSG00000184924	5.625150983	6.2290532	protein_coding	2	25012855	25016251	PTRHD1
ENSG00000184925	1.960915222	2.652276565	protein_coding	9	139844003	139849949	LCN12
ENSG00000184937	3.744048221	3.453019579	protein_coding	11	32409321	32457176	WT1
ENSG00000184939	8.3053626	8.302443832	protein_coding	16	68563993	68601039	ZFP90
ENSG00000184949	6.522395495	6.945015469	protein_coding	22	38974125	39052634	
ENSG00000184956	3.652182994	3.570966319	protein_coding	11	1012821	1036706	MUC6
ENSG00000184967	8.991362316	8.65607594	protein_coding	12	132628993	132637013	NOC4L
ENSG00000184979	7.003331296	7.780879746	protein_coding	22	18632666	18672923	USP18
ENSG00000184983	9.200400892	9.871482642	protein_coding	22	42481529	42486959	NDUFA6
ENSG00000184984	0.869158192	1.353254395	protein_coding	15	34260921	34357291	CHRM5
ENSG00000184985	8.457286611	7.791043682	protein_coding	4	7194265	7744554	SORCS2
ENSG00000184986	4.261916566	5.361622823	protein_coding	14	105992940	105996539	TMEM121
ENSG00000184988	4.77879189	4.693656882	protein_coding	17	41363894	41371589	TMEM106A
ENSG00000184990	9.475476535	8.939865791	protein_coding	14	105219437	105234831	SIVA1
ENSG00000184992	8.678607554	8.391976969	protein_coding	12	125478246	125515684	BRI3BP
ENSG00000184995	2.10647801	1.353254395	protein_coding	9	21480841	21482312	IFNE

ENSG00000184999	0.499066092	0	protein_coding	11	62905339	63137190	SLC22A10
ENSG00000185000	7.423680977	7.953699568	protein_coding	8	145539954	145550573	DGAT1
ENSG00000185009	9.414746625	8.888724634	protein_coding	10	75881524	75910821	AP3M1
ENSG00000185010	6.252103507	7.026422172	protein_coding	X	154064063	154255215	F8
ENSG00000185013	1.16343121	0.697730409	protein_coding	2	18744138	18770838	NT5C1B
ENSG00000185015	3.911897206	4.693656882	protein_coding	8	86132816	86196302	CA13
ENSG00000185019	7.599989788	7.470237199	protein_coding	20	3088219	3140842	UBOX5
ENSG00000185020	0	0.390640832	pseudogene	9	40293025	40339528	
ENSG00000185022	9.479658457	8.531880603	protein_coding	22	38597889	38612518	MAFF
ENSG00000185024	8.878072281	8.17939326	protein_coding	14	105675623	105781926	BRF1
ENSG00000185028	0.499066092	0.950786998	protein_coding	5	191626	195468	LRRC14B
ENSG00000185031	0	0.697730409	pseudogene	1	65450289	65451772	
ENSG00000185033	11.26717977	10.51277051	protein_coding	15	90703836	90772911	SEMA4B
ENSG00000185038	0.499066092	0.950786998	protein_coding	2	234684370	234742069	HEATR7B1
ENSG00000185040	1.407729925	1.16600992	pseudogene	7	76163011	76170884	
ENSG00000185043	9.899819083	9.781059731	protein_coding	15	90773207	90777279	CIB1
ENSG00000185044	4.381167248	4.566889334	processed_transc	1	143354963	143467658	
ENSG00000185046	0.869158192	0.697730409	protein_coding	12	99120235	100378432	ANKS1B
ENSG00000185049	8.794597401	8.65940853	protein_coding	4	1984441	2043630	WHSC2
ENSG00000185052	0	0.697730409	protein_coding	20	19193290	19703545	SLC24A3
ENSG00000185055	1.799000381	2.039052734	protein_coding	7	105205567	105241322	EFCAB10
ENSG00000185056	0.499066092	0	protein_coding	5	173400782	173433143	C5orf47
ENSG00000185065	3.274897671	2.420525079	processed_transc	22	19435416	19437628	
ENSG00000185068	6.658471572	7.558560655	protein_coding	6	158589384	158615020	GTF2H5
ENSG00000185070	7.950269163	8.127396674	protein_coding	14	85996488	86095034	FLRT2
ENSG00000185078	0	0.390640832	pseudogene	2	208896107	208896677	
ENSG00000185085	9.16240384	9.259743207	protein_coding	11	62414320	62420774	INTS5
ENSG00000185088	7.556308086	8.788797839	protein_coding	15	63418071	63450220	RPS27L
ENSG00000185090	7.33043718	7.394933125	protein_coding	1	38259474	38266809	MANEAL
ENSG00000185100	5.938793061	4.342020395	protein_coding	14	105190523	105213662	ADSSL1
ENSG00000185101	8.637276046	6.252780742	protein_coding	11	417933	442011	ANO9
ENSG00000185104	9.166560209	9.237617989	protein_coding	1	50905150	51425935	FAF1
ENSG00000185105	1.407729925	1.16600992	protein_coding	17	79897521	79905109	MYADML2
ENSG00000185112	5.672682881	5.855704078	protein_coding	3	194406622	194409762	FAM43A
ENSG00000185115	7.23866569	7.370740503	protein_coding	15	29560353	29562033	NDNL2
ENSG00000185122	9.88413009	9.611339686	protein_coding	8	145515280	145538385	HSF1
ENSG00000185127	7.524276744	7.743685008	protein_coding	6	170102233	170106401	C6orf120
ENSG00000185128	3.004694206	3.411459265	protein_coding	17	36283971	36294915	TBC1D3F
ENSG00000185129	7.444369108	7.373448694	protein_coding	5	139487362	139496321	PURA
ENSG00000185130	1.799000381	2.144285137	protein_coding	6	27775257	27775709	HIST1H2BL
ENSG00000185133	7.127924785	7.340609974	protein_coding	22	31518717	31530682	INPP5J
ENSG00000185158	5.538004898	5.09905904	protein_coding	17	30335037	30380517	LRRC37B
ENSG00000185162	0.499066092	0.390640832	pseudogene	11	74409389	74409869	
ENSG00000185163	8.727254249	8.385283657	protein_coding	12	132621139	132628880	DDX51
ENSG00000185164	6.721980839	7.083771121	protein_coding	16	18511182	18573533	NOMO2
ENSG00000185168	3.502389126	3.714580548	protein_coding	17	79276624	79283048	LINC00482
ENSG00000185186	4.821592551	5.212303549	lincRNA	21	44881974	44899414	LINC00313
ENSG00000185187	7.258272112	6.930376707	protein_coding	11	405716	417455	SIGIRR
ENSG00000185189	7.82964357	8.14493792	protein_coding	8	144915764	144924200	NRBP2
ENSG00000185198	1.960915222	1.802319292	protein_coding	19	685548	695461	PRSS57
ENSG00000185201	9.540974801	10.00452874	protein_coding	11	307631	315272	IFITM2
ENSG00000185215	13.58364562	12.47570712	protein_coding	14	103589779	103603776	TNFAIP2
ENSG00000185219	7.660183591	7.873706627	protein_coding	3	44481262	44519162	ZNF445
ENSG00000185220	5.827699195	6.686500471	protein_coding	1	249200395	249214145	PGBD2
ENSG00000185221	0	0.390640832	pseudogene	1	215044077	215045059	GAPDHP24
ENSG00000185222	7.952677993	8.472492669	protein_coding	X	102611373	102613397	WBP5
ENSG00000185236	10.80120923	10.90994789	protein_coding	19	8455205	8469313	RAB11B
ENSG00000185238	9.003030709	9.151882762	protein_coding	11	20409076	20530840	PRMT3
ENSG00000185245	6.462804234	5.953060722	protein_coding	17	4835592	4838325	GP1BA
ENSG00000185246	8.209916558	8.985720419	protein_coding	14	45553302	45585485	PRPF39
ENSG00000185250	5.255381949	4.489113623	protein_coding	6	109711419	109762374	PPIL6
ENSG00000185252	8.006999229	7.883239271	protein_coding	22	20748405	20762745	ZNF74
ENSG00000185261	3.448790144	3.679994897	protein_coding	5	93488671	93954309	KIAA0825

ENSG00000185262	8.237837231	8.490068116	protein_coding	17	74261283	74267375	FAM100B
ENSG00000185267	3.950976309	3.608232228	protein_coding	10	14861249	14880574	CDNF
ENSG00000185269	2.847891871	1.518964905	protein_coding	17	79910388	79919716	NOTUM
ENSG00000185271	0.499066092	0.390640832	protein_coding	14	20896970	20903801	KLHL33
ENSG00000185272	1.407729925	0	protein_coding	21	15588451	15600693	RBM11
ENSG00000185275	6.670228606	5.03207928	pseudogene	Y	21154139	21154595	CD24P4
ENSG00000185278	3.830412367	4.744751758	protein_coding	1	173837220	173866494	ZBTB37
ENSG00000185291	4.131913373	2.721932731	protein_coding	X	1455509	1501578	IL3RA
ENSG00000185298	8.462367008	8.497535398	protein_coding	17	79633761	79640937	CCDC137
ENSG00000185304	0.499066092	0	protein_coding	2	88055474	88285309	RGPD2
ENSG00000185305	7.04004079	6.554308016	protein_coding	5	53179775	53606412	ARL15
ENSG00000185324	9.46032044	9.847821207	protein_coding	16	89747145	89762772	CDK10
ENSG00000185332	2.10647801	0.390640832	protein_coding	17	79285074	79304474	TMEM105
ENSG00000185338	5.938793061	6.030599799	protein_coding	16	11348262	11350036	SOCS1
ENSG00000185339	7.266040677	7.830998152	protein_coding	22	31002825	31023265	TCN2
ENSG00000185340	9.75276053	9.469827862	protein_coding	22	29702572	29708774	GAS2L1
ENSG00000185344	7.581430769	7.987679908	protein_coding	12	124196865	124245549	ATP6V0A2
ENSG00000185345	1.407729925	2.039052734	protein_coding	6	161768452	163148803	PARK2
ENSG00000185347	8.101206377	8.234057939	protein_coding	14	105956192	105965912	C14orf80
ENSG00000185359	10.74499215	10.65032385	protein_coding	17	79651020	79669145	HGS
ENSG00000185361	8.099033379	7.568048738	protein_coding	19	4639527	4655580	TNFAIP8L1
ENSG00000185379	7.2307479	7.20731193	protein_coding	17	33426811	33448541	RAD51D
ENSG00000185386	5.729976786	5.656642807	protein_coding	22	50702142	50709196	MAPK11
ENSG00000185404	7.993024472	8.14810445	protein_coding	2	231191899	231268447	SP140L
ENSG00000185414	0.869158192	0.697730409	protein_coding	2	99771461	99814089	MRPL30
ENSG00000185418	6.634666104	7.116470702	protein_coding	15	102193801	102264807	TARSL2
ENSG00000185420	6.652556929	6.97385478	protein_coding	1	245912642	246670614	SMYD3
ENSG00000185432	4.409500985	3.570966319	protein_coding	12	51317255	51326300	METTL7A
ENSG00000185433	0.869158192	0.950786998	non_coding	21	26758133	26804013	LINC00158
ENSG00000185436	4.292665995	2.334191469	protein_coding	1	24480647	24514449	IL28RA
ENSG00000185437	4.165528823	4.917990497	protein_coding	21	40817781	40887433	SH3BGR
ENSG00000185442	2.762599152	2.912743273	protein_coding	15	93160673	93353114	FAM174B
ENSG00000185453	6.252103507	5.923798455	protein_coding	19	48673949	48700877	C19orf68
ENSG00000185467	3.146112541	1.802319292	protein_coding	7	98771197	98805129	KPNA7
ENSG00000185475	7.206730419	7.83885796	protein_coding	11	62554887	62557718	TMEM179B
ENSG00000185479	0.869158192	0	protein_coding	12	52840435	52845971	KRT6B
ENSG00000185480	6.989320791	7.234379288	protein_coding	12	102513956	102591298	C12orf48
ENSG00000185482	5.375152132	5.43605125	protein_coding	12	57637236	57644976	STAC3
ENSG00000185483	7.950269163	7.487839978	protein_coding	1	64239693	64647181	ROR1
ENSG00000185485	6.212449006	6.563824016	pseudogene	3	195686619	195717189	SDHAP1
ENSG00000185495	4.734682531	4.622587519	processed_transc	1	224183659	224198309	
ENSG00000185499	9.248248601	9.648149235	protein_coding	1	155158300	155162707	MUC1
ENSG00000185504	8.833021386	8.791837833	protein_coding	17	79506911	79520987	C17orf70
ENSG00000185507	8.116326397	8.571336379	protein_coding	11	612553	615999	IRF7
ENSG00000185513	6.716321419	6.382123086	protein_coding	20	42136320	42179590	L3MBTL1
ENSG00000185515	8.629769183	8.76011133	protein_coding	X	154299695	154351349	BRCC3
ENSG00000185519	3.502389126	2.652276565	protein_coding	1	16384264	16400127	FAM131C
ENSG00000185522	3.950976309	3.608232228	protein_coding	11	554855	560779	C11orf35
ENSG00000185523	0.499066092	1.518964905	protein_coding	1	213003483	213020991	C1orf227
ENSG00000185527	2.57521082	2.420525079	protein_coding	17	79617489	79623607	PDE6G
ENSG00000185532	0.499066092	0.950786998	protein_coding	10	52750945	54058110	PRKG1
ENSG00000185551	8.98077946	8.899098923	protein_coding	15	96869167	96883492	NR2F2
ENSG00000185559	0.869158192	0.390640832	protein_coding	14	101192042	101201539	DLK1
ENSG00000185561	3.335220907	3.679994897	protein_coding	17	1611064	1613651	TLCD2
ENSG00000185567	10.82999183	10.74981828	protein_coding	14	105403581	105444694	AHNAK2
ENSG00000185585	8.886916071	7.442134301	protein_coding	9	127539437	127577164	OLFML2A
ENSG00000185591	10.45844877	10.16553509	protein_coding	12	53773960	53810230	SP1
ENSG00000185596	4.230497448	4.468996429	pseudogene	15	102501356	102516768	WASH3P
ENSG00000185607	2.10647801	1.925536307	pseudogene	15	44281259	44282382	ACTBP7
ENSG00000185608	8.124895791	8.564242397	protein_coding	22	19419425	19423598	MRPL40
ENSG00000185610	0	0.390640832	protein_coding	12	45408455	45444882	DBX2
ENSG00000185614	3.652182994	3.027231696	protein_coding	3	49840687	49842463	FAM212A
ENSG00000185615	1.407729925	1.667587519	protein_coding	16	333152	337215	PDIA2

ENSG00000185619	8.583888749	8.860045978	protein_coding	4	699537	764428	PCGF3
ENSG00000185621	7.501423995	7.329495501	protein_coding	3	197687071	197770591	LMLN
ENSG00000185624	13.0455922	13.30715736	protein_coding	17	79801037	79818545	P4HB
ENSG00000185627	10.19358417	10.24864348	protein_coding	11	236546	252983	PSMD13
ENSG00000185630	7.921045056	7.384230902	protein_coding	1	164524821	164868533	PBX1
ENSG00000185633	2.57521082	2.971122874	protein_coding	12	57628686	57634498	NDUFA4L2
ENSG00000185634	0.869158192	1.16600992	protein_coding	15	49115932	49255641	SHC4
ENSG00000185640	0	0.697730409	protein_coding	12	53215194	53228079	KRT79
ENSG00000185641	3.274897671	2.501982735	pseudogene	5	115387607	115387986	
ENSG00000185650	12.21377612	12.29761875	protein_coding	14	69254377	69263190	ZFP36L1
ENSG00000185651	10.16686131	10.38137143	protein_coding	22	21903736	21978323	UBE2L3
ENSG00000185658	8.389559911	8.853271458	protein_coding	21	40556102	40693485	BRWD1
ENSG00000185662	0	0.390640832	protein_coding	5	171212876	171221602	C5orf50
ENSG00000185664	4.230497448	3.781359661	protein_coding	12	56347889	56367099	PMEL
ENSG00000185666	2.671945279	3.781359661	protein_coding	22	32908539	33454358	SYN3
ENSG00000185668	0	0.390640832	protein_coding	1	38509523	38512450	POU3F1
ENSG00000185669	3.871730003	3.279072565	protein_coding	16	88744090	88752901	SNAI3
ENSG00000185670	6.320845526	6.577981353	protein_coding	11	62515795	62521660	ZBTB3
ENSG00000185674	0.499066092	1.353254395	protein_coding	2	99858709	99871745	LYG2
ENSG00000185681	0.499066092	1.16600992	protein_coding	9	124922190	124962367	MORN5
ENSG00000185684	7.210761201	6.923001245	protein_coding	12	132568828	132613029	EP400NL
ENSG00000185686	8.554057865	9.940224002	protein_coding	22	22890123	22901768	PRAME
ENSG00000185689	1.16343121	1.518964905	protein_coding	6	4079440	4131185	C6orf201
ENSG00000185697	8.680062001	8.988372895	protein_coding	8	67474414	67526482	MYBL1
ENSG00000185710	0.499066092	0	pseudogene	16	21890659	21930477	
ENSG00000185716	6.94645393	6.970281236	protein_coding	16	22018959	22098855	C16orf52
ENSG00000185721	9.492959727	10.15730836	protein_coding	22	31795509	31924726	DRG1
ENSG00000185722	10.32024245	10.83407108	protein_coding	17	4066665	4167274	ANKFY1
ENSG00000185728	9.510233586	9.595189186	processed_transc	8	64081112	64125346	YTHDF3
ENSG00000185730	5.83815965	5.767861411	protein_coding	8	144371846	144380231	ZNF696
ENSG00000185739	0.499066092	0.390640832	protein_coding	16	4239377	4292081	SRL
ENSG00000185745	8.193715877	9.952954219	protein_coding	10	91152303	91163745	IFIT1
ENSG00000185753	7.326727684	7.057067116	protein_coding	X	40488285	40506819	CXorf38
ENSG00000185758	1.407729925	0	protein_coding	4	184242917	184243579	CLDN24
ENSG00000185760	7.23866569	6.984522608	protein_coding	6	73331520	73908574	KCNQ5
ENSG00000185761	7.345180517	7.607691112	protein_coding	19	1505017	1513188	ADAMTSL5
ENSG00000185774	3.211941663	3.748356452	protein_coding	4	20730239	21950422	KCNIP4
ENSG00000185787	10.16634253	10.2839776	protein_coding	15	79102829	79190475	MORF4L1
ENSG00000185792	3.274897671	3.493416095	protein_coding	19	56219798	56249768	NLRP9
ENSG00000185798	6.941611359	6.758710666	protein_coding	3	196281056	196295545	WDR53
ENSG00000185800	8.375265417	8.278081046	protein_coding	19	46286205	46296060	DMWD
ENSG00000185803	9.205459721	9.125618162	protein_coding	8	145577795	145584932	GPR172A
ENSG00000185808	5.60078458	6.037446454	protein_coding	21	38435146	38445470	PIGP
ENSG00000185811	1.16343121	0	protein_coding	7	50343720	50472799	IKZF1
ENSG00000185813	7.990682129	7.373448694	protein_coding	17	79860777	79869340	PCYT2
ENSG00000185818	6.220467583	6.343971006	protein_coding	4	2061239	2070816	NAT8L
ENSG00000185825	10.95870143	11.09852958	protein_coding	X	152965947	152990152	BCAP31
ENSG00000185829	6.407685389	6.091080508	protein_coding	17	44594069	44657088	ARL17A
ENSG00000185837	2.238690726	1.667587519	antisense	22	17640274	17646335	CECR5-AS1
ENSG00000185838	8.105542576	8.006993014	protein_coding	22	19770747	19842462	GNB1L
ENSG00000185839	0.869158192	1.353254395	pseudogene	1	59096513	59097202	
ENSG00000185842	6.956090577	7.077141316	protein_coding	1	225083964	225586996	DNAH14
ENSG00000185860	4.6658819	5.953060722	protein_coding	1	162794248	162838605	C1orf110
ENSG00000185862	5.774228067	6.316088276	protein_coding	17	29630790	29641123	EVI2B
ENSG00000185864	5.072559152	4.777836593	protein_coding	16	21845890	21892148	
ENSG00000185869	7.153278907	7.370740503	protein_coding	19	37379026	37407193	ZNF829
ENSG00000185875	7.254372086	7.426571535	protein_coding	10	25305587	25315593	THNSL1
ENSG00000185880	7.102117104	7.951888765	protein_coding	15	45021186	45060027	TRIM69
ENSG00000185883	2.847891871	3.570966319	protein_coding	16	2563727	2570219	ATP6V0C
ENSG00000185885	7.840089987	8.527023921	protein_coding	11	313506	315272	IFITM1
ENSG00000185888	0	0.390640832	protein_coding	1	228003394	228034171	PRSS38
ENSG00000185896	11.58215179	11.59407985	protein_coding	13	113951556	113977987	LAMP1
ENSG00000185900	6.005279909	6.429738366	protein_coding	8	42948658	42978577	

ENSG00000185903	0.499066092	1.667587519	pseudogene	X	130458372	130459224	OR11N1P
ENSG00000185904	0.499066092	0	processed_transc	10	42970991	42990784	
ENSG00000185909	6.442380648	6.25865215	protein_coding	3	49209044	49213918	KLHDC8B
ENSG00000185917	7.395627075	7.805153958	protein_coding	21	37406839	37451687	SETD4
ENSG00000185920	8.753741308	8.608592853	protein_coding	9	98205262	98279339	PTCH1
ENSG00000185924	7.950269163	6.530239731	protein_coding	17	1837978	1928178	RTN4RL1
ENSG00000185928	7.876068031	8.020878279	protein_coding	16	29827285	29833215	C16orf53
ENSG00000185933	0.499066092	0.697730409	protein_coding	10	105213144	105218645	CALHM1
ENSG00000185946	5.563445526	6.117167886	protein_coding	1	104068313	104097854	RNPC3
ENSG00000185947	7.618313091	7.477807561	protein_coding	16	31885079	31929914	ZNF267
ENSG00000185950	8.179389493	8.548751381	protein_coding	13	110406184	110438915	IRS2
ENSG00000185955	3.077135474	1.925536307	protein_coding	7	100054238	100061894	C7orf61
ENSG00000185958	1.16343121	1.353254395	protein_coding	12	50720013	50790405	FAM186A
ENSG00000185963	9.87399885	9.587628836	protein_coding	9	95473645	95527094	BICD2
ENSG00000185972	0	0.390640832	protein_coding	9	36169389	36171329	CCIN
ENSG00000185973	0.499066092	0	protein_coding	X	154719776	154899605	TMLHE
ENSG00000185974	1.16343121	2.039052734	protein_coding	13	114321594	114438637	GRK1
ENSG00000185986	4.642199401	5.200150955	pseudogene	5	1568637	1594735	SDHAP3
ENSG00000185988	7.627388205	8.151264044	pseudogene	19	1524138	1535455	PLK5
ENSG00000185989	7.633406721	7.982367515	protein_coding	13	114747194	114898086	RASA3
ENSG00000186001	8.959377969	8.92701904	protein_coding	3	197518097	197615307	LRCH3
ENSG00000186007	3.274897671	3.993387124	protein_coding	1	205350506	205425082	LEMD1
ENSG00000186009	0	0.697730409	protein_coding	13	114303173	114312501	ATP4B
ENSG00000186010	8.340737341	9.261206227	protein_coding	19	19626596	19644285	NDUFA13
ENSG00000186017	6.407685389	6.699508919	protein_coding	19	36936021	36980804	ZNF566
ENSG00000186019	7.575190993	7.774746834	protein_coding	19	44598503	44612919	ZNF224
ENSG00000186020	7.007971391	7.18590274	protein_coding	19	37034518	37064197	ZNF529
ENSG00000186026	5.774228067	5.656642807	protein_coding	19	44576297	44591623	ZNF284
ENSG00000186047	1.407729925	0.697730409	protein_coding	13	51285144	51418075	DLEU7
ENSG00000186051	1.16343121	0.697730409	protein_coding	9	108424738	108425367	TAL2
ENSG00000186056	3.830412367	4.021284656	antisense	1	31190940	31199674	
ENSG00000186063	9.834707333	9.94978218	protein_coding	1	222841355	222886552	AIDA
ENSG00000186073	7.352496068	6.870289474	protein_coding	15	36871812	37102449	C15orf41
ENSG00000186076	0	0.950786998	pseudogene	12	94034598	94035362	
ENSG00000186081	0	0.697730409	protein_coding	12	52908359	52914471	KRT5
ENSG00000186082	0.869158192	0.950786998	pseudogene	11	35881799	35883048	
ENSG00000186088	6.204385613	6.414040573	protein_coding	7	76940068	77046115	PION
ENSG00000186104	6.681890601	7.495318771	protein_coding	11	14899553	14913798	CYP2R1
ENSG00000186105	0.499066092	0.950786998	protein_coding	5	61874562	61877275	LRRC70
ENSG00000186106	5.389447744	5.717592784	protein_coding	8	101521980	101572012	ANKRD46
ENSG00000186111	10.08633286	9.967814887	protein_coding	19	3630182	3700463	PIP5K1C
ENSG00000186118	0.499066092	0.697730409	protein_coding	1	47134527	47139266	ATPAF1-AS1
ENSG00000186130	7.474855471	7.502758994	protein_coding	9	125670335	125675609	ZBTB6
ENSG00000186132	4.568725997	4.528525526	protein_coding	2	120059801	120124404	C2orf76
ENSG00000186136	0.499066092	0.390640832	protein_coding	12	11338599	11339543	TAS2R42
ENSG00000186141	8.105542576	8.626866398	protein_coding	1	145592605	145611025	POLR3C
ENSG00000186150	2.928422289	2.851901313	protein_coding	1	110655062	110656569	UBL4B
ENSG00000186152	0	0.390640832	pseudogene	19	55208383	55212683	LILRP1
ENSG00000186153	6.75547892	6.835357159	protein_coding	16	78133310	79246564	WWOX
ENSG00000186160	0	0.950786998	protein_coding	1	47533160	47583991	CYP4Z1
ENSG00000186162	6.716321419	6.866449614	pseudogene	3	10055932	10068049	CIDECP
ENSG00000186163	1.16343121	0.390640832	pseudogene	7	141965650	141972054	
ENSG00000186166	8.090308512	8.245929405	protein_coding	11	118868852	118886501	CCDC84
ENSG00000186174	11.76253139	11.25338986	protein_coding	11	118764584	118796317	BCL9L
ENSG00000186184	8.594725428	7.994732806	protein_coding	13	28194903	28241548	POLR1D
ENSG00000186185	9.150911694	8.928861309	protein_coding	17	43003360	43025082	KIF18B
ENSG00000186187	8.566693498	8.543951256	protein_coding	16	75032928	75144892	ZNRF1
ENSG00000186188	1.407729925	0.950786998	protein_coding	10	95326422	95349829	O3FAR1
ENSG00000186190	0.499066092	0	protein_coding	20	31643230	31661434	BPIFB3
ENSG00000186193	9.137210979	8.483815721	protein_coding	9	139956581	139965040	C9orf140
ENSG00000186204	2.10647801	0.697730409	protein_coding	19	15783567	15807984	CYP4F12
ENSG00000186205	4.464551814	3.32456471	protein_coding	1	220960101	220987735	1-Mar
ENSG00000186207	0.869158192	0	protein_coding	1	152483320	152484653	LCE5A

ENSG00000186212	1.960915222	0.697730409	protein_coding	4	77816082	77819002	ANKRD56
ENSG00000186222	6.892270075	7.033288635	protein_coding	4	6717842	6719387	CNO
ENSG00000186230	6.877132514	6.815578497	protein_coding	19	57946697	57956853	ZNF749
ENSG00000186231	0.499066092	0.390640832	protein_coding	6	97372496	97588630	KLHL32
ENSG00000186235	0.499066092	0.390640832	protein_coding	2	239133193	239140318	
ENSG00000186260	8.687312327	8.397309351	protein_coding	16	14173145	14360629	MKL2
ENSG00000186265	0.499066092	0	protein_coding	3	112182815	112218408	BTLA
ENSG00000186272	6.462804234	6.327306117	protein_coding	19	57922529	57933307	ZNF17
ENSG00000186275	4.491309013	4.94736961	protein_coding	1	146373546	146467744	NBPF12
ENSG00000186280	5.331395195	6.037446454	protein_coding	11	94706845	94732682	KDM4D
ENSG00000186281	8.813274715	9.307964293	protein_coding	2	96687694	96705199	GPAT2
ENSG00000186283	8.280649185	8.450859136	protein_coding	1	179050512	179067158	TOR3A
ENSG00000186298	10.52065391	10.01713935	protein_coding	12	111157485	111180744	PPP1CC
ENSG00000186300	6.163378456	6.349483465	protein_coding	19	2841433	2860472	ZNF555
ENSG00000186301	2.928422289	3.679994897	pseudogene	1	16972069	16976914	
ENSG00000186312	7.136425764	7.287036562	pseudogene	X	15693055	15721472	CA5BP1
ENSG00000186314	5.929039784	5.726093467	protein_coding	5	144851362	145214932	PRELID2
ENSG00000186318	9.930083245	10.39142693	protein_coding	11	117156402	117186975	BACE1
ENSG00000186322	0.499066092	0	pseudogene	15	82804003	82810908	
ENSG00000186326	1.16343121	1.518964905	protein_coding	19	33166313	33169206	RGS9BP
ENSG00000186329	0	0.390640832	protein_coding	3	171561139	171656505	TMEM212
ENSG00000186334	0.869158192	0	protein_coding	5	150656323	150683327	SLC36A3
ENSG00000186335	0	0.390640832	protein_coding	5	150694539	150727151	SLC36A2
ENSG00000186340	4.165528823	4.841813558	protein_coding	6	169615875	169654139	THBS2
ENSG00000186350	10.37604692	9.60444017	protein_coding	9	137208944	137332431	RXRA
ENSG00000186352	4.230497448	4.17820932	protein_coding	4	186317175	186321782	ANKRD37
ENSG00000186354	2.471521042	2.912743273	protein_coding	9	91605778	91611055	C9orf47
ENSG00000186364	5.525114348	5.886365696	protein_coding	1	145586115	145590461	NUDT17
ENSG00000186369	0.869158192	0	lincRNA	14	62584197	62596352	
ENSG00000186376	6.804311337	7.505230566	protein_coding	X	134382867	134478012	ZNF75D
ENSG00000186377	5.072559152	5.647721597	protein_coding	1	47427036	47516423	CYP4X1
ENSG00000186395	7.933293789	7.770643695	protein_coding	17	38974369	38978847	KRT10
ENSG00000186399	2.671945279	3.279072565	protein_coding	15	30695943	30706463	
ENSG00000186409	1.616589159	3.453019579	protein_coding	1	42929001	43120335	CCDC30
ENSG00000186416	8.721615518	8.851330023	protein_coding	X	118722300	118739858	NKRF
ENSG00000186417	0.869158192	0.390640832	protein_coding	15	51633826	51700210	GLDN
ENSG00000186432	10.46605656	9.978082538	protein_coding	3	160212783	160283376	KPNA4
ENSG00000186446	5.613019223	5.751299057	protein_coding	3	44771088	44778575	ZNF501
ENSG00000186448	6.616550626	6.79148052	protein_coding	3	44626380	44689963	ZNF197
ENSG00000186451	2.471521042	3.027231696	protein_coding	3	57094469	57109460	SPATA12
ENSG00000186452	0	0.697730409	protein_coding	12	51236703	51281667	TMPRSS12
ENSG00000186466	1.16343121	0.697730409	pseudogene	9	67272038	67289492	AQP7P1
ENSG00000186468	11.19602115	11.52679437	protein_coding	5	81569177	81574396	RPS23
ENSG00000186469	2.35979773	3.183544561	protein_coding	14	52292913	52446060	GNG2
ENSG00000186470	9.102921211	9.569438796	protein_coding	6	26365387	26378546	BTN3A2
ENSG00000186471	0.499066092	1.667587519	protein_coding	X	119029800	119054679	AKAP14
ENSG00000186472	6.379315619	4.406904905	protein_coding	7	82383321	82792246	PCLO
ENSG00000186480	10.26073495	8.001751392	protein_coding	7	155089486	155101945	INSIG1
ENSG00000186481	3.393122761	3.748356452	pseudogene	18	14179096	14249455	ANKRD20A5P
ENSG00000186493	2.762599152	0.390640832	protein_coding	5	2752245	2755508	C5orf38
ENSG00000186496	2.35979773	3.368666104	protein_coding	18	32946661	32957301	ZNF396
ENSG00000186501	8.46911316	8.511125832	protein_coding	1	27648651	27662891	TMEM222
ENSG00000186510	0.499066092	0.390640832	protein_coding	1	16345330	16360545	CLCNKA
ENSG00000186517	4.292665995	3.714580548	protein_coding	1	161016736	161039760	ARHGAP30
ENSG00000186522	9.679757009	9.422885181	protein_coding	2	110300380	110371783	10-sep
ENSG00000186523	6.47626108	5.9163892	protein_coding	8	12035972	12052469	FAM86B1
ENSG00000186526	0.499066092	0	protein_coding	19	15726029	15740448	CYP4F8
ENSG00000186530	2.35979773	0.390640832	processed_transc	8	6666038	6693166	XKR5
ENSG00000186532	8.219950371	8.782698567	protein_coding	17	1682829	1733170	SMYD4
ENSG00000186543	0.869158192	0	pseudogene	1	21760811	21764051	
ENSG00000186564	4.821592551	4.693656882	protein_coding	1	47901689	47906363	FOXD2
ENSG00000186566	9.432976748	9.160532436	protein_coding	17	42472652	42580802	GPATCH8
ENSG00000186567	7.22677265	6.582669759	protein_coding	19	45174724	45187631	CEACAM19

ENSG00000186575	11.74439799	11.84701192	protein_coding	22	29999545	30094587	NF2
ENSG00000186577	7.04004079	7.966311947	protein_coding	6	34214157	34217247	C6orf1
ENSG00000186583	1.16343121	1.802319292	protein_coding	8	145086582	145101933	SPATC1
ENSG00000186591	11.26354634	10.86254281	protein_coding	7	129470572	129592789	UBE2H
ENSG00000186594	7.277615649	7.869875863	processed_transc	17	1614805	1619504	MIR22HG
ENSG00000186603	7.549958459	7.057067116	protein_coding	1	45792545	45794347	HPDL
ENSG00000186615	5.795854912	6.210997888	protein_coding	14	55965996	56046828	KTN1-AS1
ENSG00000186625	7.292905885	7.778838337	protein_coding	6	149916009	149970108	KATNA1
ENSG00000186628	4.292665995	4.468996429	protein_coding	15	83424114	83474822	FSD2
ENSG00000186635	10.16426552	10.30576281	protein_coding	11	72396114	72504644	ARAP1
ENSG00000186638	7.123655438	7.616862529	protein_coding	9	34252379	34329198	KIF24
ENSG00000186642	6.47626108	6.204928885	protein_coding	11	72287185	72385635	PDE2A
ENSG00000186645	0.499066092	0	pseudogene	7	76651635	76661674	SPDYE8P
ENSG00000186648	2.57521082	2.912743273	protein_coding	14	24521206	24538937	LRRC16B
ENSG00000186652	2.10647801	1.802319292	protein_coding	11	57154267	57158130	PRG2
ENSG00000186654	7.30049066	6.775188634	protein_coding	22	45064593	45133561	PRR5
ENSG00000186660	10.36839913	10.25636687	protein_coding	11	58346584	58388515	ZFP91
ENSG00000186665	6.96088487	7.628245414	protein_coding	17	65987218	65989765	C17orf58
ENSG00000186666	4.800350936	5.072639111	protein_coding	12	50231573	50236912	BCDIN3D
ENSG00000186676	6.023726639	6.198834244	pseudogene	7	124673507	124675355	
ENSG00000186684	7.190493718	5.901455548	protein_coding	2	127941696	127977654	CYP27C1
ENSG00000186687	7.67776266	7.41347308	protein_coding	5	130506503	130541119	LYRM7
ENSG00000186704	3.502389126	2.851901313	pseudogene	7	76607934	76673092	
ENSG00000186710	1.616589159	0.950786998	protein_coding	12	113587663	113597081	CCDC42B
ENSG00000186714	1.16343121	0.697730409	protein_coding	11	32623792	32816204	CCDC73
ENSG00000186715	2.10647801	2.039052734	protein_coding	1	17081405	17096732	MST1P9
ENSG00000186716	10.49485366	9.472356449	protein_coding	22	23521891	23660224	BCR
ENSG00000186743	0	0.390640832	pseudogene	6	116359919	116360658	TPI1P3
ENSG00000186765	3.554067925	3.183544561	protein_coding	17	79495422	79504156	FSCN2
ENSG00000186766	0.499066092	0	protein_coding	10	129535499	129539450	FOXI2
ENSG00000186767	7.845284967	7.646273261	protein_coding	X	62567107	62571223	SPIN4
ENSG00000186777	5.637181576	5.526427614	protein_coding	4	264464	299110	ZNF732
ENSG00000186787	3.950976309	5.283144778	protein_coding	X	57144984	57147980	SPIN2B
ENSG00000186790	1.16343121	0.697730409	protein_coding	1	47881744	47883723	FOXE3
ENSG00000186792	6.830742851	6.610484676	protein_coding	3	50330262	50336899	HYAL3
ENSG00000186806	5.375152132	5.620622115	protein_coding	19	51834790	51845378	VSIG10L
ENSG00000186807	5.270908378	3.133298822	protein_coding	10	47746936	47763041	ANXA8L2
ENSG00000186810	1.407729925	0.697730409	protein_coding	X	70835766	70838367	CXCR3
ENSG00000186812	6.372135143	6.664555628	protein_coding	18	32820994	32839191	ZNF397
ENSG00000186814	7.254372086	7.65742733	protein_coding	18	32831023	32870196	ZSCAN30
ENSG00000186815	11.29876545	11.09422652	protein_coding	12	113658855	113736390	TPCN1
ENSG00000186818	0	0.697730409	protein_coding	19	55155340	55181810	LILRB4
ENSG00000186827	1.960915222	2.144285137	protein_coding	1	1146706	1149518	TNFRSF4
ENSG00000186831	0.499066092	0	protein_coding	17	18330175	18335162	
ENSG00000186832	7.165790811	6.874119142	protein_coding	17	39766030	39769005	KRT16
ENSG00000186834	9.281205548	9.390496617	protein_coding	17	43224684	43229468	HEXIM1
ENSG00000186838	0	0.390640832	protein_coding	19	40005753	40011326	
ENSG00000186842	0	1.16600992	processed_transc	21	33944548	33948191	C21orf77
ENSG00000186847	0.499066092	0.697730409	protein_coding	17	39738531	39743173	KRT14
ENSG00000186854	9.05999771	8.729786462	protein_coding	2	85048774	85134132	C2orf89
ENSG00000186862	5.4452544	5.085909553	protein_coding	10	102767441	102790890	PDZD7
ENSG00000186866	8.987843316	8.980400792	protein_coding	21	46683843	46707813	POFUT2
ENSG00000186868	6.907250452	6.104183162	protein_coding	17	43971748	44105703	MAPT
ENSG00000186871	8.219950371	8.520929986	protein_coding	X	71424510	71458897	ERCC6L
ENSG00000186889	3.830412367	4.468996429	protein_coding	2	62727356	62739029	TMEM17
ENSG00000186891	4.642199401	3.714580548	protein_coding	1	1138888	1142071	TNFRSF18
ENSG00000186897	5.072559152	4.94736961	protein_coding	12	49726200	49730971	C1QL4
ENSG00000186907	5.741167503	5.593003837	protein_coding	11	57228022	57245007	RTN4RL2
ENSG00000186908	7.471499737	7.960920138	protein_coding	12	77157368	77247476	ZDHHC17
ENSG00000186912	2.35979773	0.697730409	protein_coding	X	69478016	69479654	P2RY4
ENSG00000186918	9.093128531	9.019827949	protein_coding	8	28203102	28260218	ZNF395
ENSG00000186919	1.16343121	0.950786998	protein_coding	17	74075263	74078885	ZACN
ENSG00000186940	0.869158192	0.697730409	pseudogene	9	82006219	82006674	CHCHD2P9

ENSG00000186951	7.842689815	7.462626902	protein_coding	22	46546424	46639653	PPARA
ENSG00000186952	2.238690726	1.925536307	protein_coding	5	109624934	110074657	TMEM232
ENSG00000186976	3.871730003	4.7279204	protein_coding	22	43924624	44208217	EFCAB6
ENSG00000186994	3.146112541	2.334191469	protein_coding	19	8387469	8408146	KANK3
ENSG00000186998	2.57521082	1.925536307	protein_coding	22	29601840	29655586	EMID1
ENSG00000187003	0.499066092	0	protein_coding	9	111624603	111626035	ACTL7A
ENSG00000187008	2.671945279	2.334191469	processed_transc	16	71963441	72033877	PKD1L3
ENSG00000187010	1.616589159	2.334191469	protein_coding	1	25598884	25656936	RHD
ENSG00000187013	2.10647801	3.608232228	protein_coding	17	59489112	59490641	C17orf82
ENSG00000187017	7.289098491	5.638744877	protein_coding	1	6484848	6521430	ESPN
ENSG00000187045	4.821592551	4.448594745	protein_coding	22	37461476	37505603	TMPRSS6
ENSG00000187049	6.604346021	6.577981353	protein_coding	11	61159159	61166335	TMEM216
ENSG00000187051	8.663981856	8.987489278	protein_coding	22	39925098	39928860	RPS19BP1
ENSG00000187054	0.499066092	0	protein_coding	4	68775103	68829858	TMPRSS11A
ENSG00000187066	2.671945279	3.679994897	protein_coding	11	64852451	64856847	
ENSG00000187068	0.499066092	0.697730409	protein_coding	3	184795838	184870802	C3orf70
ENSG00000187079	10.40931148	10.35180778	protein_coding	11	12695969	12966298	TEAD1
ENSG00000187091	4.962122459	4.841813558	protein_coding	3	38048987	38071253	PLCD1
ENSG00000187094	0	0.390640832	protein_coding	3	42299317	42307699	CCK
ENSG00000187097	8.295907473	8.542748725	protein_coding	14	74424713	74486102	ENTPD5
ENSG00000187098	7.423680977	7.41347308	protein_coding	3	69788586	70017488	MITF
ENSG00000187105	3.989024711	3.679994897	protein_coding	14	73945189	74025651	HEATR4
ENSG00000187109	12.31530691	12.52533275	protein_coding	12	76438670	76478813	NAP1L1
ENSG00000187118	6.693459083	7.154756319	protein_coding	3	28283075	28366633	CMC1
ENSG00000187123	2.57521082	3.411459265	protein_coding	2	150186499	150330662	LYPD6
ENSG00000187134	3.830412367	4.226940739	protein_coding	10	4934796	5025475	AKR1C1
ENSG00000187140	1.799000381	3.027231696	protein_coding	1	63788730	63790797	FOXD3
ENSG00000187144	1.616589159	0.697730409	protein_coding	1	16713612	16763919	SPATA21
ENSG00000187145	9.473800365	10.24753675	protein_coding	1	150266289	150281414	MRPS21
ENSG00000187147	8.635777795	8.722370165	protein_coding	1	44870866	45117396	RNF220
ENSG00000187151	0.499066092	0.390640832	protein_coding	11	101761405	101787253	ANGPTL5
ENSG00000187164	8.880604615	8.936206949	protein_coding	10	118643742	118886097	KIAA1598
ENSG00000187172	0.499066092	0	pseudogene	21	10996026	11098980	BAGE2
ENSG00000187185	1.407729925	0.950786998	protein_coding	16	57832098	57850850	
ENSG00000187186	1.407729925	1.353254395	protein_coding	9	34664160	34666109	
ENSG00000187187	4.062237333	4.585695337	protein_coding	19	40502943	40537972	ZNF546
ENSG00000187189	7.178195136	7.591499486	protein_coding	6	116571151	116575261	TSPYL4
ENSG00000187193	5.948480845	6.827478203	protein_coding	16	56716336	56718108	MT1X
ENSG00000187210	7.90122817	7.022976646	protein_coding	9	79034752	79122332	GCNT1
ENSG00000187229	0.869158192	1.16600992	processed_transc	8	18578415	18591915	
ENSG00000187231	7.827020104	7.701200733	protein_coding	2	179966483	180129517	SESTD1
ENSG00000187239	9.43986141	9.466026651	protein_coding	9	132649466	132805473	FNBP1
ENSG00000187240	8.74542939	9.197681135	protein_coding	11	102980160	103350591	DYNC2H1
ENSG00000187244	9.757590402	9.377739515	protein_coding	19	45312338	45324677	BCAM
ENSG00000187257	8.08373006	7.529716161	protein_coding	7	77325760	77409008	RSBN1L
ENSG00000187260	2.471521042	2.334191469	protein_coding	7	151072995	151107813	WDR86
ENSG00000187266	8.120617457	8.53672099	protein_coding	19	11487881	11495018	EPOR
ENSG00000187268	0	0.390640832	protein_coding	X	13053737	13062801	FAM9C
ENSG00000187288	0.499066092	0	protein_coding	3	9908398	9921938	CIDEC
ENSG00000187325	8.3480754	8.49131535	protein_coding	X	77385245	77395203	TAF9B
ENSG00000187372	6.814942123	6.803579823	protein_coding	5	140593509	140596993	PCDHB13
ENSG00000187391	3.393122761	3.532712221	protein_coding	7	77646393	79082890	MAGI2
ENSG00000187446	10.21781967	9.654843329	protein_coding	15	41523037	41574043	
ENSG00000187456	5.054730169	4.94736961	protein_coding	17	34245070	34257780	RDM1
ENSG00000187474	0	0.697730409	protein_coding	19	52298411	52329334	FPR3
ENSG00000187479	1.616589159	1.16600992	protein_coding	11	43946892	43965888	C11orf96
ENSG00000187486	2.10647801	1.802319292	protein_coding	11	17407406	17410878	KCNJ11
ENSG00000187492	1.799000381	1.667587519	protein_coding	3	49828165	49837268	CDHR4
ENSG00000187498	0.869158192	1.518964905	protein_coding	13	110801311	110959496	COL4A1
ENSG00000187504	0	0.390640832	pseudogene	17	49579849	49580594	
ENSG00000187510	0	0.697730409	protein_coding	12	93115281	93166231	PLEKHG7
ENSG00000187513	0	0.950786998	protein_coding	1	35258599	35261348	GJA4
ENSG00000187514	13.30730466	13.13446442	protein_coding	2	232571605	232578251	PTMA

ENSG00000187522	8.926668426	8.618900187	protein_coding	10	14880163	14913740	HSPA14
ENSG00000187531	7.457998197	7.400254612	protein_coding	17	79869815	79876052	SIRT7
ENSG00000187533	0	0.390640832	protein_coding	4	70999333	71042516	C4orf40
ENSG00000187534	1.16343121	1.16600992	pseudogene	19	40448563	40449634	
ENSG00000187535	8.653654925	8.796890297	protein_coding	16	1560428	1662111	IFT140
ENSG00000187536	0	0.950786998	pseudogene	2	26032794	26033525	TPM3P7
ENSG00000187546	0	0.950786998	protein_coding	7	15239943	15601640	AGMO
ENSG00000187550	0.869158192	1.667587519	protein_coding	19	56041100	56048456	SBK2
ENSG00000187554	6.244259325	5.767861411	protein_coding	1	223282748	223316624	TLR5
ENSG00000187555	11.0376904	10.87262791	protein_coding	16	8985951	9058371	USP7
ENSG00000187556	1.799000381	1.518964905	protein_coding	19	13987950	13991571	NANOS3
ENSG00000187566	5.175139844	5.072639111	protein_coding	6	18120718	18122851	NHLRC1
ENSG00000187581	0.499066092	0	protein_coding	14	93813537	93814702	COX8C
ENSG00000187583	6.146643566	6.2290532	protein_coding	1	901877	911245	PLEKHN1
ENSG00000187595	2.471521042	1.667587519	protein_coding	17	40177594	40215096	ZNF385C
ENSG00000187601	5.869093502	6.016808232	protein_coding	X	55478538	55479998	MAGEH1
ENSG00000187605	6.616550626	6.605885938	protein_coding	2	74229840	74329698	TET3
ENSG00000187607	6.771940731	7.188980716	protein_coding	17	15602891	15640874	ZNF286A
ENSG00000187608	9.506959233	10.64836849	protein_coding	1	948803	949920	ISG15
ENSG00000187609	7.003331296	6.568558581	protein_coding	9	140201348	140317714	EXD3
ENSG00000187624	6.912209543	7.619146302	protein_coding	17	260118	264367	C17orf97
ENSG00000187626	4.883502971	5.41517489	protein_coding	6	28212401	28227011	ZKSCAN4
ENSG00000187627	0	0.390640832	protein_coding	2	87135076	87241104	RGPD1
ENSG00000187630	6.204385613	6.57327766	protein_coding	14	24439148	24475617	DHRS4L2
ENSG00000187634	5.4452544	4.385599336	protein_coding	1	860260	879955	SAMD11
ENSG00000187642	4.230497448	4.226940739	protein_coding	1	910579	917497	C1orf170
ENSG00000187650	5.81716234	5.383282767	protein_coding	19	5904852	5910261	VMAC
ENSG00000187653	4.842525946	5.212303549	pseudogene	4	91759652	91760263	TMSL3
ENSG00000187664	1.799000381	2.144285137	protein_coding	19	19366456	19373596	HAPLN4
ENSG00000187667	5.575999561	5.995870484	pseudogene	15	23187728	23208737	WHAMMP3
ENSG00000187672	0.869158192	1.16600992	protein_coding	3	55542336	56502391	ERC2
ENSG00000187676	6.560798025	6.787424764	protein_coding	13	31774073	31906413	B3GALTL
ENSG00000187678	8.023133844	6.668971382	protein_coding	5	141689992	141706020	SPRY4
ENSG00000187682	0	1.16600992	protein_coding	X	48687283	48688548	ERAS
ENSG00000187686	0.499066092	1.16600992	pseudogene	11	124983057	124996677	
ENSG00000187688	8.207901394	6.419292172	protein_coding	17	16318888	16340317	TRPV2
ENSG00000187689	2.847891871	6.064512706	protein_coding	4	71384257	71398459	AMTN
ENSG00000187695	2.471521042	2.242360793	protein_coding	3	128628717	128690173	
ENSG00000187699	2.35979773	3.714580548	protein_coding	2	190744335	191068210	C2orf88
ENSG00000187713	8.763378405	8.823871996	protein_coding	9	140098534	140100090	TMEM203
ENSG00000187715	0	0.390640832	protein_coding	3	127634075	127706514	KBTBD12
ENSG00000187720	8.536501169	8.85617872	protein_coding	15	71389291	72075722	THSD4
ENSG00000187726	2.471521042	1.667587519	protein_coding	11	73661364	73681411	DNAJB13
ENSG00000187730	2.762599152	1.16600992	protein_coding	1	1950780	1962192	GABRD
ENSG00000187735	9.540174158	9.55340681	protein_coding	8	54879112	54935089	TCEA1
ENSG00000187736	2.10647801	2.579085888	protein_coding	2	219940039	220025587	NHEJ1
ENSG00000187741	9.288851923	9.522019224	protein_coding	16	89803957	89883065	FANCA
ENSG00000187742	8.53810609	8.616616024	protein_coding	9	91933421	91974557	SECISBP2
ENSG00000187747	0	0.390640832	protein_coding	11	5602107	5603114	OR52B6
ENSG00000187753	0.499066092	0.390640832	protein_coding	9	88836224	88874572	C9orf153
ENSG00000187762	1.16343121	0.697730409	pseudogene	6	34991297	34991592	
ENSG00000187764	6.96088487	6.419292172	protein_coding	9	91975702	92113045	SEMA4D
ENSG00000187775	5.827699195	6.746227492	protein_coding	17	76419778	76573476	DNAH17
ENSG00000187778	10.01147814	9.990484452	protein_coding	12	49950327	49961936	MCRS1
ENSG00000187783	0	0.390640832	protein_coding	10	45406648	45432450	TMEM72
ENSG00000187790	7.484876093	7.883239271	protein_coding	14	45605143	45670093	FANCM
ENSG00000187791	0	0.697730409	pseudogene	9	34889244	34895775	FAM205CP
ENSG00000187792	5.417620896	6.110690132	protein_coding	22	24083769	24093279	ZNF70
ENSG00000187796	6.014532757	5.555337277	protein_coding	9	139256355	139268133	CARD9
ENSG00000187800	4.863159938	2.721932731	protein_coding	1	156863490	156886226	PEAR1
ENSG00000187801	5.301468762	5.94580061	protein_coding	1	40915774	40929390	ZNF643
ENSG00000187808	3.274897671	3.279072565	protein_coding	X	118892576	118894164	ANKRD58
ENSG00000187811	3.146112541	3.081239798	protein_coding	11	71292956	71318487	

ENSG00000187815	5.763291843	6.077857763	protein_coding	1	40942887	40962015	ZNF642
ENSG00000187824	3.698846687	4.932754837	protein_coding	17	10616612	10633646	TMEM220
ENSG00000187833	0.869158192	0.950786998	protein_coding	2	74011316	74044274	C2orf78
ENSG00000187837	7.427149666	8.713847451	protein_coding	6	26055968	26056699	HIST1H1C
ENSG00000187838	9.348606364	9.915351046	protein_coding	17	7293053	7307416	PLSCR3
ENSG00000187840	10.19307491	10.43226174	protein_coding	8	37887859	37917883	EIF4EBP1
ENSG00000187848	1.799000381	1.353254395	protein_coding	12	133195366	133198972	P2RX2
ENSG00000187860	6.188122351	6.398170093	protein_coding	22	30752624	30774647	CCDC157
ENSG00000187866	7.348842929	7.568048738	protein_coding	9	71394964	71398609	FAM122A
ENSG00000187867	2.10647801	0	protein_coding	19	14164179	14169971	PALM3
ENSG00000187870	0.869158192	1.518964905	pseudogene	17	20646646	20657841	
ENSG00000187902	1.960915222	1.667587519	protein_coding	19	55940105	55954230	SHISA7
ENSG00000187904	0.499066092	0	processed_transc	4	6997068	7000685	
ENSG00000187905	2.238690726	1.16600992	protein_coding	22	21400249	21418457	
ENSG00000187908	0.499066092	0.697730409	protein_coding	10	124320181	124403252	DMBT1
ENSG00000187912	0	0.950786998	protein_coding	19	14693896	14721969	CLEC17A
ENSG00000187918	0	0.390640832	protein_coding	11	5474638	5475707	OR51I2
ENSG00000187922	0	0.390640832	protein_coding	9	139632619	139637808	LCN10
ENSG00000187939	3.077135474	2.242360793	protein_coding	17	6007	31427	DOC2B
ENSG00000187942	3.744048221	2.420525079	protein_coding	1	22138758	22151714	LDLRAD2
ENSG00000187944	1.407729925	2.242360793	protein_coding	2	197669726	197675000	C2orf66
ENSG00000187951	5.909333162	5.931169853	protein_coding	15	30916697	31060329	ARHGAP11B
ENSG00000187952	0.869158192	0.390640832	pseudogene	1	21754795	21756030	HS6ST1P1
ENSG00000187953	7.451199747	7.365308824	pseudogene	7	6749759	6793493	PMS2CL
ENSG00000187954	8.441937187	8.517261232	protein_coding	8	145674965	145691060	CYHR1
ENSG00000187955	0.499066092	1.802319292	protein_coding	8	121072019	121384275	COL14A1
ENSG00000187957	7.214780754	7.93548844	protein_coding	2	230222345	230579274	DNER
ENSG00000187959	0	0.697730409	protein_coding	17	71244588	71258491	CPSF4L
ENSG00000187961	8.171138658	8.289597152	protein_coding	1	895967	901095	KLHL17
ENSG00000187980	0.499066092	0.390640832	protein_coding	1	20487746	20503917	PLA2G2C
ENSG00000187984	5.255381949	4.508954154	pseudogene	9	95571670	95650975	ANKRD19P
ENSG00000187987	3.211941663	4.202780776	protein_coding	6	28399707	28411279	ZSCAN23
ENSG00000187988	0	0.390640832	pseudogene	9	37477146	37478314	
ENSG00000187990	4.352265886	3.993387124	protein_coding	6	26216428	26216872	HIST1H2BG
ENSG00000187994	1.799000381	2.242360793	protein_coding	19	39358470	39368894	RINL
ENSG00000187999	0	0.697730409	pseudogene	2	33861569	33862492	
ENSG00000188000	0.869158192	0	protein_coding	19	9296279	9299493	OR7D2
ENSG00000188001	2.847891871	2.652276565	protein_coding	3	188665003	189043093	TPRG1
ENSG00000188002	6.585843317	6.460630645	pseudogene	5	1599035	1634120	
ENSG00000188004	3.448790144	3.608232228	protein_coding	1	159804264	159825137	C1orf204
ENSG00000188010	6.25990527	6.712401119	protein_coding	2	39103103	39156213	MORN2
ENSG00000188013	4.6658819	4.976162367	pseudogene	17	20492371	20494013	MEIS3P2
ENSG00000188015	6.94645393	7.614575135	protein_coding	1	153519805	153521848	S100A3
ENSG00000188021	9.194306789	9.439133999	protein_coding	X	56590026	56593443	UBQLN2
ENSG00000188026	7.269909329	6.988061092	protein_coding	12	123955925	124018265	RILPL1
ENSG00000188032	1.16343121	0.390640832	protein_coding	19	14192431	14196687	C19orf67
ENSG00000188033	7.132181536	6.915587884	protein_coding	19	12688775	12750912	ZNF490
ENSG00000188038	0	0.697730409	protein_coding	16	67918781	67920275	NRN1L
ENSG00000188039	3.211941663	1.802319292	protein_coding	19	16830787	16928774	NWD1
ENSG00000188042	8.546104025	7.402908012	protein_coding	2	235401685	235405697	ARL4C
ENSG00000188050	0.869158192	0.390640832	protein_coding	7	122337766	122339210	RNF133
ENSG00000188051	2.762599152	2.912743273	protein_coding	19	17546318	17559376	TMEM221
ENSG00000188060	6.716321419	6.559073862	protein_coding	1	28918712	28921955	RAB42
ENSG00000188064	8.46911316	5.620622115	protein_coding	22	46316242	46373009	WNT7B
ENSG00000188070	7.123655438	7.527286218	processed_transc	11	63527364	63536113	C11orf95
ENSG00000188073	0	0.390640832	pseudogene	7	74916804	74928928	PMS2P10
ENSG00000188086	1.799000381	0.697730409	protein_coding	3	46783582	46854048	PRSS45
ENSG00000188089	0.869158192	1.802319292	protein_coding	15	42273780	42343388	PLA2G4E
ENSG00000188092	2.10647801	2.652276565	protein_coding	1	147400506	147465753	GPR89B
ENSG00000188095	3.744048221	3.748356452	protein_coding	15	90303822	90321982	MESP2
ENSG00000188107	2.10647801	1.925536307	protein_coding	6	64429876	66417118	EYS
ENSG00000188112	7.811178326	6.544728831	protein_coding	6	42068856	42110357	C6orf132
ENSG00000188126	5.090170488	4.961837817	protein_coding	17	73584139	73622923	MYO15B

ENSG00000188130	9.768569768	10.24679846	protein_coding	22	50683879	50700254	MAPK12
ENSG00000188133	0.499066092	0.390640832	protein_coding	9	32783497	32787397	TMEM215
ENSG00000188152	3.077135474	3.081239798	protein_coding	9	99690592	99704572	FAM22G
ENSG00000188153	8.020839917	7.803146634	protein_coding	X	107683074	107940775	COL4A5
ENSG00000188157	12.43986818	12.19390275	protein_coding	1	955503	991496	AGRN
ENSG00000188158	7.757079669	7.546612122	protein_coding	X	17393543	17754114	NHS
ENSG00000188162	0	0.390640832	protein_coding	11	17568920	17668697	OTOG
ENSG00000188163	1.616589159	1.16600992	protein_coding	9	140138036	140142222	FAM166A
ENSG00000188167	5.158541613	5.456629825	protein_coding	3	33131913	33138293	TMPPE
ENSG00000188171	8.000028772	8.168519197	protein_coding	19	20802745	20844406	ZNF626
ENSG00000188175	0.499066092	0	protein_coding	7	92817899	92855837	HEPACAM2
ENSG00000188176	0	0.697730409	protein_coding	17	4487294	4511614	SMTNL2
ENSG00000188177	6.598204782	6.338437404	protein_coding	2	113033171	113097640	ZC3H6
ENSG00000188185	6.912209543	7.157901395	processed_transc	7	39773231	39834216	LINC00265
ENSG00000188186	8.695964893	8.454700467	protein_coding	7	99746530	99753567	C7orf59
ENSG00000188191	9.373091386	9.519576271	protein_coding	7	588834	767287	PRKAR1B
ENSG00000188199	0.869158192	2.242360793	protein_coding	10	81462983	81474437	FAM22B
ENSG00000188206	5.360713447	6.535085617	processed_transc	1	245003940	245018799	HNRNPU-AS1
ENSG00000188211	8.114176072	8.436686098	protein_coding	11	17373273	17398888	
ENSG00000188215	7.72925009	7.287036562	protein_coding	16	20869396	20911706	DCUN1D3
ENSG00000188223	6.204385613	6.525377513	protein_coding	19	36239512	36245417	LIN37
ENSG00000188227	6.305850085	6.758710666	protein_coding	19	37997841	38034237	ZNF793
ENSG00000188229	12.52093205	12.27833076	protein_coding	9	140135665	140138159	TUBB4B
ENSG00000188234	3.211941663	3.453019579	protein_coding	10	46321042	46349323	AGAP4
ENSG00000188242	7.119373418	7.129345795	protein_coding	5	466239	473213	
ENSG00000188243	7.726437377	8.238521194	protein_coding	13	76099350	76123575	COMMD6
ENSG00000188263	2.35979773	1.518964905	protein_coding	22	50432942	50451088	IL17REL
ENSG00000188266	4.230497448	4.385599336	protein_coding	15	78799906	78829714	AGPHD1
ENSG00000188277	4.352265886	4.7279204	protein_coding	15	41062159	41064647	C15orf62
ENSG00000188279	1.799000381	0	protein_coding	10	49199649	49207835	FAM25C
ENSG00000188282	1.616589159	1.518964905	protein_coding	2	218899683	218955304	RUFY4
ENSG00000188283	5.018397273	5.22435463	protein_coding	19	37717366	37734566	ZNF383
ENSG00000188290	6.766474304	7.154756319	protein_coding	1	934342	935552	HES4
ENSG00000188295	6.068837212	6.009862691	protein_coding	1	247261406	247267674	ZNF669
ENSG00000188305	2.10647801	2.242360793	protein_coding	19	2274631	2282181	C19orf35
ENSG00000188306	1.616589159	1.16600992	protein_coding	3	169539710	169555563	LRRIQ4
ENSG00000188312	6.435508026	6.858739095	protein_coding	9	95087766	95382815	CENPP
ENSG00000188313	9.186141067	9.838540929	protein_coding	3	146232967	146262651	PLSCR1
ENSG00000188315	5.255381949	5.072639111	protein_coding	3	49306035	49315342	C3orf62
ENSG00000188316	4.165528823	4.101879561	protein_coding	10	118609023	118671297	ENO4
ENSG00000188321	7.757079669	8.135396417	protein_coding	19	9434873	9454519	ZNF559
ENSG00000188322	4.517578978	4.547834947	protein_coding	16	28303840	28335170	SBK1
ENSG00000188338	0	0.390640832	processed_transc	3	50242679	50258411	SLC38A3
ENSG00000188342	8.085926212	7.86795666	protein_coding	13	45694650	45858237	GTF2F2
ENSG00000188343	6.975172889	7.289906366	protein_coding	8	94710789	94743755	FAM92A1
ENSG00000188352	7.308035767	7.575124073	protein_coding	9	20658308	20995954	KIAA1797
ENSG00000188365	0.869158192	0.697730409	antisense	7	5459477	5462753	
ENSG00000188368	3.211941663	2.788380093	protein_coding	19	42806284	42814973	PRR19
ENSG00000188372	7.478203418	6.720932357	protein_coding	7	76026835	76071388	ZP3
ENSG00000188375	1.407729925	1.353254395	protein_coding	12	31944123	31945175	H3F3C
ENSG00000188383	1.799000381	0.697730409	pseudogene	2	97746835	97748986	
ENSG00000188385	4.131913373	3.714580548	protein_coding	10	133918175	133998313	JAKMIP3
ENSG00000188386	0.499066092	0.390640832	protein_coding	9	104353897	104357283	PPP3R2
ENSG00000188388	0.499066092	0	pseudogene	15	85783674	85790410	
ENSG00000188389	1.407729925	0.390640832	protein_coding	2	242792033	242801060	PDCD1
ENSG00000188394	0	0.390640832	protein_coding	9	125796806	125797975	GPR21
ENSG00000188396	2.57521082	1.925536307	protein_coding	1	45271585	45272957	TCTEX1D4
ENSG00000188404	1.799000381	0	protein_coding	1	169659808	169680839	SELL
ENSG00000188419	8.525216449	8.708495073	protein_coding	X	85116185	85302566	CHM
ENSG00000188428	6.835971536	7.040122572	protein_coding	6	8013800	8064647	MUTED
ENSG00000188451	1.407729925	2.144285137	pseudogene	6	158658368	158663829	SRP72P2
ENSG00000188452	0.869158192	0.390640832	protein_coding	2	182401403	182545392	CERKL
ENSG00000188459	0.499066092	0	pseudogene	X	47657325	47664188	WASF4P

ENSG00000188460	2.471521042	2.144285137	pseudogene	1	224051428	224052553	ACTBP11
ENSG00000188477	0.869158192	0.390640832	sense_intronic	16	19316203	19322269	CLEC19A
ENSG00000188483	8.734271811	8.351343215	protein_coding	9	131937835	131940540	IER5L
ENSG00000188486	10.69874479	11.16510226	protein_coding	11	118964564	118966177	H2AFX
ENSG00000188488	6.171673665	2.971122874	protein_coding	14	95027779	95059457	SERPINA5
ENSG00000188493	7.254372086	6.889336956	protein_coding	19	41246761	41256408	C19orf54
ENSG00000188501	1.799000381	2.242360793	protein_coding	15	66839517	66858317	LCTL
ENSG00000188505	0.869158192	0	protein_coding	19	39687604	39692522	NCCRP1
ENSG00000188508	0	1.518964905	protein_coding	19	35978226	35986460	KRTDAP
ENSG00000188511	0	0.390640832	protein_coding	22	49808176	50051190	C22orf34
ENSG00000188512	0	0.390640832	pseudogene	8	33827079	33827397	
ENSG00000188522	9.432113851	10.10163939	protein_coding	17	18872102	18908117	FAM83G
ENSG00000188523	0	0.950786998	protein_coding	9	135285430	135448704	C9orf171
ENSG00000188525	0	0.390640832	antisense	2	10143286	10146158	
ENSG00000188529	10.10215133	10.29008203	protein_coding	1	24291294	24307417	SRSF10
ENSG00000188542	4.923348194	4.932754837	protein_coding	2	241499471	241503431	DUSP28
ENSG00000188549	10.59192395	11.1941149	protein_coding	15	40623653	40633168	C15orf52
ENSG00000188554	10.35161244	10.55214769	protein_coding	17	41322511	41363706	NBR1
ENSG00000188559	8.340737341	8.628000846	protein_coding	20	20370196	20693266	RALGAPA2
ENSG00000188566	10.12368915	10.00932506	protein_coding	9	140100119	140113813	NDOR1
ENSG00000188573	0	0.390640832	pseudogene	5	167956510	167957508	FBLL1
ENSG00000188596	2.762599152	2.721932731	protein_coding	12	96883349	97269333	C12orf55
ENSG00000188599	6.244259325	6.530239731	protein_coding	16	15198178	15225458	NPIPP1
ENSG00000188603	5.899377978	6.3217081	protein_coding	16	28477983	28506896	CLN3
ENSG00000188610	5.054730169	4.903073497	protein_coding	1	120837272	120855681	FAM72B
ENSG00000188611	1.16343121	0.950786998	protein_coding	10	51884446	52039568	ASAH2
ENSG00000188612	9.305909516	9.619347638	protein_coding	17	73163825	73179098	SUMO2
ENSG00000188613	6.749949642	6.858739095	protein_coding	10	120789228	120793854	NANOS1
ENSG00000188626	0.499066092	1.16600992	protein_coding	15	28947100	28983530	
ENSG00000188629	6.155035276	7.073814954	protein_coding	19	9435021	9493291	ZNF559-ZNF177
ENSG00000188636	9.44329146	9.732839523	protein_coding	22	44888452	44894178	LDOC1L
ENSG00000188641	7.671926738	7.394933125	protein_coding	1	97543299	98386605	DPYD
ENSG00000188643	11.24499497	11.24601996	protein_coding	1	153579362	153585644	S100A16
ENSG00000188647	8.617676359	8.975952737	protein_coding	9	72324438	72374875	PTAR1
ENSG00000188649	0.499066092	0.950786998	protein_coding	10	97709744	97792441	CC2D2B
ENSG00000188659	2.762599152	3.570966319	protein_coding	15	82555151	82577271	FAM154B
ENSG00000188660	5.899377978	6.174194902	lincRNA	21	44866481	44873773	LINC00319
ENSG00000188662	0	0.390640832	pseudogene	17	48248789	48258539	HILS1
ENSG00000188672	2.847891871	2.788380093	protein_coding	1	25688741	25756683	RHCE
ENSG00000188676	2.35979773	2.420525079	protein_coding	8	39792133	39873910	IDO2
ENSG00000188677	6.984620223	6.945015469	protein_coding	22	44395091	44565106	PARVB
ENSG00000188681	5.83815965	6.610484676	pseudogene	21	9907190	9968585	
ENSG00000188682	0	1.16600992	protein_coding	11	30964749	31391357	DCDC1
ENSG00000188686	3.652182994	3.608232228	protein_coding	8	145490549	145492470	SCXA
ENSG00000188687	2.238690726	3.64455972	protein_coding	2	74443369	74570541	SLC4A5
ENSG00000188690	8.631273684	8.966118473	protein_coding	10	127477146	127511817	UROS
ENSG00000188693	1.407729925	0.390640832	processed_transc	7	91763918	91810039	
ENSG00000188706	9.834707333	9.80533094	protein_coding	X	128937264	128977885	ZDHHC9
ENSG00000188707	8.883132512	7.964516916	protein_coding	7	150026938	150029808	C7orf29
ENSG00000188710	2.762599152	2.652276565	protein_coding	9	133768815	133769225	QRFP
ENSG00000188712	0.499066092	0.390640832	pseudogene	9	107484616	107485552	
ENSG00000188716	0.499066092	0	protein_coding	10	76797594	76818272	DUPD1
ENSG00000188725	9.036344956	8.634788863	protein_coding	5	60453536	60458301	C5orf43
ENSG00000188732	4.734682531	6.167968749	protein_coding	7	23719749	23742868	C7orf46
ENSG00000188735	9.148812342	7.907733332	protein_coding	12	122150658	122220907	TMEM120B
ENSG00000188738	3.393122761	0.950786998	protein_coding	2	186603355	186698017	FSIP2
ENSG00000188739	3.393122761	2.721932731	protein_coding	1	235294498	235324772	RBM34
ENSG00000188747	4.999881834	4.976162367	protein_coding	9	140317802	140328858	NOXA1
ENSG00000188760	4.842525946	5.200150955	protein_coding	2	220408385	220415317	TMEM198
ENSG00000188761	1.616589159	1.667587519	protein_coding	1	114420790	114430169	BCL2L15
ENSG00000188763	6.171673665	5.878761167	protein_coding	7	72848109	72850450	FZD9
ENSG00000188765	0	0.390640832	pseudogene	2	3665138	3665347	TMSB4XP2
ENSG00000188766	3.274897671	3.368666104	protein_coding	19	38880840	38886868	SPRED3

ENSG00000188770	0.499066092	0.390640832	protein_coding	1	203463271	203477992	OPTC
ENSG00000188771	0.499066092	0.950786998	protein_coding	11	112118876	112131583	C11orf34
ENSG00000188779	1.799000381	1.802319292	protein_coding	15	68112042	68126899	SKOR1
ENSG00000188783	0.499066092	1.518964905	protein_coding	1	203444956	203460480	PRELP
ENSG00000188784	0	0.390640832	protein_coding	1	20246502	20250110	PLA2G2E
ENSG00000188785	7.23866569	7.138927266	protein_coding	19	57901218	57913917	ZNF548
ENSG00000188786	6.733233509	6.673373662	protein_coding	1	38279851	38325292	MTF1
ENSG00000188800	0	0.390640832	protein_coding	1	40711619	40717363	TMCO2
ENSG00000188801	1.16343121	1.353254395	pseudogene	9	99959537	99961910	ZNF322P1
ENSG00000188807	8.753741308	8.675956977	protein_coding	1	9648932	9674935	TMEM201
ENSG00000188811	6.693459083	6.349483465	protein_coding	13	39612443	39625205	NHLRC3
ENSG00000188818	8.002356002	8.157562549	protein_coding	5	710471	851101	ZDHHC11
ENSG00000188820	0	0.697730409	protein_coding	6	116782533	116784934	FAM26F
ENSG00000188822	0.499066092	0.390640832	protein_coding	1	24197016	24285549	CNR2
ENSG00000188825	5.346128795	5.175534283	protein_coding	17	41447213	41466266	
ENSG00000188827	8.502378699	8.519708104	protein_coding	16	3631182	3661599	SLX4
ENSG00000188828	0.499066092	0	protein_coding	X	102962152	102983583	GLRA4
ENSG00000188833	1.616589159	1.16600992	protein_coding	9	140328816	140336268	ENTPD8
ENSG00000188846	10.89875151	11.22742878	protein_coding	3	40498783	40506549	RPL14
ENSG00000188850	0	0.390640832	pseudogene	5	43336266	43348818	
ENSG00000188856	0.499066092	0.697730409	pseudogene	8	81471105	81471992	RPSAP47
ENSG00000188868	5.752272083	6.044260771	protein_coding	19	12428291	12444534	ZNF563
ENSG00000188869	2.10647801	1.518964905	protein_coding	15	81623558	81666554	TMC3
ENSG00000188873	1.616589159	2.652276565	pseudogene	8	48068741	48069394	RPL10AP2
ENSG00000188878	8.120617457	8.88967084	protein_coding	17	73905925	73937119	FBF1
ENSG00000188883	1.407729925	1.16600992	protein_coding	7	139137436	139168458	KLRG2
ENSG00000188886	2.671945279	0.950786998	protein_coding	2	96789589	96804175	ASTL
ENSG00000188888	1.960915222	2.971122874	protein_coding	17	36481493	36499730	GPR179
ENSG00000188895	9.263851576	9.167571071	protein_coding	17	38278790	38293040	MSL1
ENSG00000188897	4.981125677	4.903073497	protein_coding	16	11536047	11585611	
ENSG00000188910	8.027710784	2.334191469	protein_coding	1	35246790	35251970	GJB3
ENSG00000188916	1.960915222	2.851901313	protein_coding	10	128933694	128994422	FAM196A
ENSG00000188917	8.45218826	8.40394723	protein_coding	X	100264335	100307105	TRMT2B
ENSG00000188921	5.729976786	5.43605125	protein_coding	9	20995306	21031635	PTPLAD2
ENSG00000188931	5.672682881	5.656642807	protein_coding	1	161334521	161337664	C1orf192
ENSG00000188933	5.575999561	6.646756201	processed_transc	17	16689803	16719854	
ENSG00000188938	8.316627558	8.453421159	protein_coding	9	96208776	96215874	FAM120AOS
ENSG00000188958	0.499066092	1.925536307	protein_coding	3	190984957	191048325	UTS2D
ENSG00000188959	2.35979773	2.334191469	protein_coding	9	112952328	112970469	C9orf152
ENSG00000188971	7.341508784	7.541804862	protein_coding	2	69686414	69693664	
ENSG00000188976	10.46141213	10.30965652	protein_coding	1	879584	894689	NOC2L
ENSG00000188981	3.077135474	2.851901313	protein_coding	4	3246096	3273465	C4orf44
ENSG00000188985	1.407729925	1.667587519	pseudogene	18	23749724	23751313	
ENSG00000188986	10.89092254	10.38975587	protein_coding	9	140149625	140167998	COBRA1
ENSG00000188987	0.869158192	0.950786998	protein_coding	6	26188938	26189304	HIST1H4D
ENSG00000188992	0	0.950786998	protein_coding	21	15481134	15583166	LIPI
ENSG00000188993	1.16343121	2.039052734	protein_coding	4	52859868	52883786	LRRC66
ENSG00000188994	8.045874708	8.242970684	protein_coding	6	87862551	87973914	ZNF292
ENSG00000188996	2.671945279	1.802319292	protein_coding	6	655939	656963	HUS1B
ENSG00000188997	6.96088487	6.712401119	protein_coding	11	77882295	77899868	KCTD21
ENSG00000189001	3.335220907	3.133298822	protein_coding	19	36014269	36019253	SBSN
ENSG00000189007	7.654275977	7.756190105	protein_coding	6	143747078	143771810	ADAT2
ENSG00000189011	0	0.697730409	pseudogene	7	74702428	74714647	
ENSG00000189014	0	0.390640832	pseudogene	10	47379727	47420712	FAM35B2
ENSG00000189042	6.372135143	6.646756201	protein_coding	19	37178530	37214248	ZNF567
ENSG00000189043	9.293610409	9.654843329	protein_coding	7	10971578	10979883	NDUFA4
ENSG00000189045	0.499066092	1.802319292	protein_coding	5	74907301	74967671	ANKDD1B
ENSG00000189046	7.517784241	7.829026491	protein_coding	12	109525993	109531436	ALKBH2
ENSG00000189050	7.140657543	7.213370937	protein_coding	17	58029601	58042122	RNFT1
ENSG00000189051	1.407729925	1.667587519	protein_coding	17	8294022	8301144	RNF222
ENSG00000189052	0.499066092	0	protein_coding	19	49547063	49548566	CGB5
ENSG00000189056	1.616589159	0.390640832	protein_coding	7	103112231	103629963	RELN
ENSG00000189057	8.360827871	9.119178324	protein_coding	11	58874658	58894883	FAM111B

ENSG00000189060	11.08402209	10.46437136	protein_coding	22	38201114	38203442	H1F0
ENSG00000189067	10.79819868	9.943417103	protein_coding	16	11641582	11680806	LITAF
ENSG00000189068	0	0.390640832	protein_coding	19	54544079	54567207	VSTM1
ENSG00000189077	7.612231145	7.505230566	pseudogene	7	75616155	75623977	TMEM120A
ENSG00000189079	8.502378699	8.38394126	protein_coding	12	46123448	46301823	ARID2
ENSG00000189089	1.407729925	1.16600992	pseudogene	21	37422512	37423675	RIMKLBP1
ENSG00000189091	11.80447699	11.92765146	protein_coding	16	70557691	70608820	SF3B3
ENSG00000189108	2.238690726	1.925536307	protein_coding	X	103810996	105012102	IL1RAPL2
ENSG00000189114	7.976547636	7.307006333	protein_coding	19	45682003	45685057	BLOC1S3
ENSG00000189120	8.043616684	7.026422172	protein_coding	17	45922280	45933240	SP6
ENSG00000189127	2.35979773	0.697730409	protein_coding	5	79852574	79866307	ANKRD34B
ENSG00000189129	0.869158192	0.390640832	protein_coding	10	81891477	81905115	PLAC9
ENSG00000189136	3.871730003	4.17820932	pseudogene	15	85070012	85114447	UBE2Q2P1
ENSG00000189139	0.869158192	1.16600992	protein_coding	14	44973545	44976482	FSCB
ENSG00000189143	10.80554674	7.786986696	protein_coding	7	73213872	73247017	CLDN4
ENSG00000189144	4.903563137	5.294620694	protein_coding	19	38229203	38307940	ZNF573
ENSG00000189145	0.499066092	0	pseudogene	X	40767237	40767644	
ENSG00000189149	3.077135474	3.32456471	lincRNA	16	21312170	21329912	CRYM-AS1
ENSG00000189152	0	0.390640832	protein_coding	17	19030782	19062148	
ENSG00000189157	2.35979773	1.802319292	protein_coding	4	77135193	77232282	FAM47E
ENSG00000189159	10.33555133	10.10693785	protein_coding	17	73131343	73150778	HN1
ENSG00000189164	5.752272083	5.824376633	protein_coding	19	37862059	37883968	ZNF527
ENSG00000189166	0.499066092	0	pseudogene	7	57059595	57076856	TNRC18C
ENSG00000189167	0.499066092	0	protein_coding	13	32877837	32889481	ZAR1L
ENSG00000189171	8.655134734	9.066597822	protein_coding	1	153591263	153606873	S100A13
ENSG00000189180	8.429884808	9.539005037	protein_coding	10	38299578	38356282	ZNF33A
ENSG00000189190	7.720795447	7.472765069	protein_coding	19	53268747	53290034	ZNF600
ENSG00000189195	2.10647801	2.242360793	protein_coding	1	92545862	92613393	BTBD8
ENSG00000189212	2.238690726	2.144285137	pseudogene	7	35119601	35225934	DPY19L2P1
ENSG00000189221	9.556895063	10.51645069	protein_coding	X	43515467	43606068	MAOA
ENSG00000189223	8.817245731	9.356680039	processed_transc	2	113969099	114024587	
ENSG00000189227	5.316509574	5.674321415	protein_coding	15	67813406	67819628	C15orf61
ENSG00000189233	0.499066092	0	protein_coding	8	27879481	27941388	C8orf80
ENSG00000189238	3.077135474	3.906303962	lincRNA	12	127210816	127230798	
ENSG00000189241	9.713528165	9.506065582	protein_coding	6	116597741	116601066	TSPYL1
ENSG00000189266	8.557227164	9.002437654	protein_coding	1	24285599	24289952	PNRC2
ENSG00000189269	3.830412367	4.29708397	protein_coding	22	23950639	23974508	C22orf43
ENSG00000189280	4.689181911	1.667587519	protein_coding	1	35220648	35224113	GJB5
ENSG00000189283	3.004694206	3.493416095	protein_coding	3	59735036	61237133	FHIT
ENSG00000189292	0.499066092	0.390640832	protein_coding	2	279565	288851	FAM150B
ENSG00000189298	5.613019223	6.051043053	protein_coding	6	28317691	28335336	ZKSCAN3
ENSG00000189306	9.904803739	9.890041731	protein_coding	22	42905974	42915808	RRP7A
ENSG00000189308	7.471499737	7.410839058	protein_coding	4	83831126	83934079	LIN54
ENSG00000189316	0.499066092	0	antisense	7	64348905	64350478	
ENSG00000189319	8.826469148	8.567793748	protein_coding	10	126307861	126432838	FAM53B
ENSG00000189320	0	0.697730409	protein_coding	7	135413096	135433594	FAM180A
ENSG00000189332	3.911897206	3.64455972	protein_coding	11	18230685	18236109	
ENSG00000189334	5.672682881	1.353254395	protein_coding	1	153586731	153589462	S100A14
ENSG00000189337	7.066972635	6.668971382	protein_coding	1	14925200	15444539	KAZN
ENSG00000189339	8.874265427	9.237617989	protein_coding	1	1592939	1624167	SLC35E2B
ENSG00000189343	4.165528823	4.127774132	pseudogene	17	19349246	19350169	
ENSG00000189350	1.960915222	2.039052734	protein_coding	2	29179477	29275096	FAM179A
ENSG00000189362	6.541724534	6.434933235	protein_coding	2	191369068	191399448	TMEM194B
ENSG00000189366	3.652182994	2.721932731	protein_coding	3	125648118	125655882	ALG1L
ENSG00000189367	0.499066092	0.390640832	protein_coding	6	127761488	127780536	KIAA0408
ENSG00000189375	0.869158192	1.518964905	protein_coding	17	18538842	18547740	TBC1D28
ENSG00000189376	5.054730169	5.283144778	protein_coding	8	124232196	124253638	C8orf76
ENSG00000189377	0.869158192	0.390640832	protein_coding	19	42932696	42947136	CXCL17
ENSG00000189401	0.499066092	0.390640832	protein_coding	X	69282341	69284029	OTUD6A
ENSG00000189403	10.61605933	10.30576281	protein_coding	13	31032884	31191734	HMGB1
ENSG00000189409	2.57521082	3.532712221	protein_coding	1	1567474	1570639	MMP23B
ENSG00000189410	6.220467583	6.505763096	protein_coding	1	21046225	21059330	SH2D5
ENSG00000189419	0.499066092	1.353254395	processed_transc	15	100884662	100890438	

ENSG00000189423	1.616589159	3.232099092	pseudogene	17	20327905	20334324	USP32P3
ENSG00000189431	2.762599152	0.950786998	pseudogene	11	13030696	13032647	RASSF10
ENSG00000189433	1.799000381	0.697730409	protein_coding	1	35225342	35229325	GJB4
ENSG00000196072	7.933293789	8.194786822	protein_coding	10	102033713	102046439	BLOC1S2
ENSG00000196074	6.364918751	7.852510423	protein_coding	20	58438621	58508710	SYCP2
ENSG00000196081	7.481543613	7.454976247	protein_coding	19	23404401	23433162	ZNF724P
ENSG00000196083	9.223526019	9.111898978	protein_coding	3	190231840	190375843	IL1RAP
ENSG00000196092	0.869158192	0.390640832	protein_coding	9	36833272	37034476	PAX5
ENSG00000196104	1.16343121	0	protein_coding	4	167654535	168155947	SPOCK3
ENSG00000196109	5.707331524	5.886365696	protein_coding	19	22361903	22379753	ZNF676
ENSG00000196110	5.879259127	5.516660875	protein_coding	19	9404951	9415795	ZNF699
ENSG00000196114	1.407729925	0.697730409	pseudogene	6	34544035	34544433	
ENSG00000196116	7.740446424	7.485338407	protein_coding	9	100174232	100258407	TDRD7
ENSG00000196118	3.448790144	4.15321211	protein_coding	16	30768744	30774031	C16orf93
ENSG00000196123	7.615275323	7.999999944	protein_coding	16	67209505	67217943	KIAA0895L
ENSG00000196126	0.499066092	0	protein_coding	6	32546546	32557625	HLA-DRB1
ENSG00000196132	0	0.390640832	protein_coding	20	62783144	62873604	MYT1
ENSG00000196136	5.625150983	3.411459265	protein_coding	14	95058395	95090983	SERPINA3
ENSG00000196139	5.4452544	4.841813558	protein_coding	10	5077549	5149878	AKR1C3
ENSG00000196141	10.95028817	10.25600002	protein_coding	2	201170604	201343253	SPATS2L
ENSG00000196150	5.431503808	6.434933235	protein_coding	8	146076632	146127553	ZNF250
ENSG00000196151	6.421663777	6.414040573	protein_coding	2	160092304	160143310	WDSUB1
ENSG00000196152	6.335686705	6.287656023	protein_coding	9	130186661	130207651	ZNF79
ENSG00000196154	6.856698814	6.343971006	protein_coding	1	153516089	153522612	S100A4
ENSG00000196155	9.008829705	9.717467399	protein_coding	16	67311413	67323402	PLEKHG4
ENSG00000196159	5.389447744	6.223059835	protein_coding	4	126237554	126414087	FAT4
ENSG00000196167	0.499066092	0	protein_coding	11	111164114	111175770	C11orf92
ENSG00000196172	7.304268146	7.332282157	protein_coding	19	23921997	23941693	ZNF681
ENSG00000196176	0.869158192	0.697730409	protein_coding	6	26021907	26044555	HIST1H4A
ENSG00000196177	7.527512071	8.549948921	protein_coding	10	124768495	124817827	ACADSB
ENSG00000196182	9.032934112	8.721307575	protein_coding	1	36805225	36851497	STK40
ENSG00000196183	0.499066092	0.950786998	pseudogene	14	105302331	105303221	RPS2P4
ENSG00000196187	9.263851576	9.701929717	protein_coding	1	226033237	226070069	TMEM63A
ENSG00000196188	0.869158192	1.16600992	protein_coding	1	206317459	206332104	CTSE
ENSG00000196189	4.6658819	3.64455972	protein_coding	1	156117157	156147543	SEMA4A
ENSG00000196193	0.869158192	1.353254395	pseudogene	5	10201621	10201960	
ENSG00000196196	1.799000381	1.16600992	protein_coding	9	35906189	35907138	HRCT1
ENSG00000196199	8.622223055	8.879228285	protein_coding	13	20207788	20247596	MPHOSPH8
ENSG00000196204	7.671926738	7.879433769	pseudogene	7	5013619	5080306	RNF216P1
ENSG00000196205	4.491309013	4.710890357	pseudogene	9	135894816	135896553	EEF1A1P5
ENSG00000196208	5.107569429	5.294620694	protein_coding	2	11674242	11782914	GREB1
ENSG00000196209	1.16343121	1.16600992	protein_coding	20	1451386	1472233	SIRPB2
ENSG00000196214	8.243750574	8.101495266	protein_coding	19	52772824	52795977	ZNF766
ENSG00000196218	2.10647801	1.925536307	protein_coding	19	38924340	39078204	RYR1
ENSG00000196220	4.75690578	4.777836593	protein_coding	3	9022275	9404737	SRGAP3
ENSG00000196226	0.499066092	1.353254395	protein_coding	6	26043455	26043885	HIST1H2BB
ENSG00000196227	7.093410923	7.106738413	protein_coding	20	58508819	58523735	C20orf177
ENSG00000196230	14.14368213	14.31404127	protein_coding	6	30687978	30693203	TUBB
ENSG00000196233	7.940593421	7.946442679	protein_coding	10	98592017	98740800	LCOR
ENSG00000196235	9.482161805	9.549221123	protein_coding	19	39936186	39967306	SUPT5H
ENSG00000196236	8.835633972	8.846464976	protein_coding	22	41253081	41368585	XPNPEP3
ENSG00000196247	8.375265417	7.472765069	protein_coding	7	64126501	64171404	ZNF107
ENSG00000196260	0.499066092	0	protein_coding	6	30899130	30899952	SFTA2
ENSG00000196262	11.93340152	11.79959747	protein_coding	7	44836279	44864163	PPIA
ENSG00000196263	5.774228067	5.974624386	protein_coding	19	57019212	57040270	ZNF471
ENSG00000196267	6.0776925	6.136428444	protein_coding	19	52658125	52674896	ZNF836
ENSG00000196268	7.374222747	7.793067905	protein_coding	19	21579931	21610375	ZNF493
ENSG00000196275	2.762599152	3.027231696	protein_coding	7	74210483	74267847	GTF2IRD2
ENSG00000196284	6.112579918	5.938503778	protein_coding	6	44777054	45345690	SUPT3H
ENSG00000196289	0.869158192	0.697730409	pseudogene	1	242121039	242122364	BECN1P1
ENSG00000196290	8.267164507	8.432796436	protein_coding	2	201754050	201768655	NIF3L1
ENSG00000196295	6.766474304	6.839280559	processed_transc	7	30555925	30604233	
ENSG00000196296	5.485736881	5.32851069	protein_coding	16	28889726	28915830	ATP2A1

ENSG00000196305	12.56450073	12.59515222	protein_coding	9	94972489	95056038	IARS
ENSG00000196312	2.928422289	3.279072565	protein_coding	9	99711033	99775862	HIATL2
ENSG00000196313	9.171738888	8.114504097	protein_coding	7	72349936	72421979	POM121
ENSG00000196323	9.227510213	9.458394052	protein_coding	11	130096572	130184581	ZBTB44
ENSG00000196329	2.928422289	3.935919592	protein_coding	7	150419341	150447121	GIMAP5
ENSG00000196331	2.471521042	2.788380093	protein_coding	6	27861203	27861669	HIST1H2BO
ENSG00000196335	3.335220907	2.420525079	protein_coding	7	23749786	23872132	STK31
ENSG00000196337	4.464551814	3.279072565	protein_coding	19	49557531	49562117	CGB7
ENSG00000196338	4.999881834	5.09905904	protein_coding	X	70364681	70391051	NLGN3
ENSG00000196345	2.847891871	3.935919592	protein_coding	3	44596685	44635665	ZNF167
ENSG00000196352	8.440221571	8.793860943	protein_coding	1	207494853	207534311	CD55
ENSG00000196355	0.869158192	1.667587519	protein_coding	4	38628029	38666430	
ENSG00000196357	3.911897206	4.448594745	protein_coding	19	36673188	36737159	ZNF565
ENSG00000196358	3.652182994	3.232099092	protein_coding	9	135037334	135119921	NTNG2
ENSG00000196361	0.499066092	1.353254395	protein_coding	19	11562143	11591803	ELAVL3
ENSG00000196363	10.38499274	10.14745308	protein_coding	9	137001210	137025093	WDR5
ENSG00000196364	0	0.390640832	protein_coding	16	1310953	1313846	
ENSG00000196365	11.42860114	11.75888075	protein_coding	19	5691845	5720463	LONP1
ENSG00000196366	3.146112541	3.906303962	protein_coding	9	139377947	139380518	C9orf163
ENSG00000196367	10.75226989	10.14626591	protein_coding	7	98475556	98610866	TRRAP
ENSG00000196369	6.005279909	5.832272499	pseudogene	1	143913615	144094424	SRGAP2P2
ENSG00000196371	6.710639711	6.668971382	protein_coding	11	94277017	94283063	FUT4
ENSG00000196372	9.373990307	8.962525691	protein_coding	10	5680830	5708768	ASB13
ENSG00000196374	0	1.353254395	protein_coding	6	27782822	27783267	HIST1H2BM
ENSG00000196378	4.491309013	4.622587519	protein_coding	8	145997611	146012730	ZNF34
ENSG00000196381	4.19837882	4.810179682	protein_coding	19	38158650	38183216	ZNF781
ENSG00000196387	7.334137164	7.648510984	protein_coding	12	133656424	133684130	ZNF140
ENSG00000196388	4.842525946	5.611474642	protein_coding	17	4891426	4900905	INCA1
ENSG00000196390	0.869158192	0.697730409	pseudogene	5	74225705	74226391	
ENSG00000196391	4.942865586	4.917990497	protein_coding	15	90895477	90909324	ZNF774
ENSG00000196396	10.2684903	9.770824962	protein_coding	20	49126891	49201299	PTPN1
ENSG00000196400	0.869158192	1.667587519	protein_coding	9	69252845	69254032	
ENSG00000196405	9.190229706	8.553535588	protein_coding	14	100437786	100610573	EVL
ENSG00000196407	4.568725997	3.714580548	protein_coding	1	151818220	151826173	THEM5
ENSG00000196409	3.393122761	3.232099092	protein_coding	9	40760700	40836415	ZNF658
ENSG00000196411	11.13846561	9.621627484	protein_coding	7	100400187	100425121	EPHB4
ENSG00000196415	0	0.390640832	protein_coding	19	840985	848180	PRTN3
ENSG00000196417	7.921045056	7.646273261	protein_coding	19	53893046	53930574	ZNF765
ENSG00000196418	5.869093502	6.002883551	protein_coding	1	247285277	247335310	ZNF124
ENSG00000196419	12.17032476	12.27851138	protein_coding	22	42017123	42060044	XRCC6
ENSG00000196420	1.16343121	1.353254395	protein_coding	1	153509623	153514241	S100A5
ENSG00000196421	2.671945279	3.679994897	lincRNA	20	62665697	62671315	LINC00176
ENSG00000196422	9.264821181	8.52337065	protein_coding	9	138371648	138380739	PPP1R26
ENSG00000196427	0.499066092	0.390640832	protein_coding	1	108765087	108786703	NBPF4
ENSG00000196428	9.048782844	8.540340653	protein_coding	3	150126080	150177903	TSC22D2
ENSG00000196433	0	0.697730409	protein_coding	X	1733894	1761974	ASMT
ENSG00000196436	0	0.390640832	protein_coding	16	74411156	74425978	NPIPL2
ENSG00000196437	6.386460534	6.889336956	protein_coding	19	37902062	37958339	ZNF569
ENSG00000196440	6.298293508	6.874119142	protein_coding	X	100673275	100788446	ARMCX4
ENSG00000196449	7.575190993	7.340609974	protein_coding	1	38268616	38273857	YRDC
ENSG00000196453	8.426422697	8.275187599	protein_coding	7	149128454	149158214	ZNF777
ENSG00000196455	9.169669647	9.081580806	protein_coding	3	130397779	130465673	PIK3R4
ENSG00000196456	7.15746161	7.179727017	protein_coding	7	150065879	150109558	ZNF775
ENSG00000196458	8.061582684	8.313767852	protein_coding	12	133498047	133532892	ZNF605
ENSG00000196459	6.941611359	6.807590479	protein_coding	X	13730363	13752754	TRAPPC2
ENSG00000196460	0.869158192	0.390640832	protein_coding	2	101966891	102091165	RFX8
ENSG00000196465	8.415986232	8.933456715	protein_coding	12	56546040	56551771	MYL6B
ENSG00000196466	5.869093502	6.316088276	protein_coding	19	12490003	12512088	ZNF799
ENSG00000196470	6.693459083	6.889336956	protein_coding	16	48390275	48482313	SIAH1
ENSG00000196476	7.654275977	8.129000175	protein_coding	20	251504	271390	C20orf96
ENSG00000196482	0	0.390640832	protein_coding	1	216676588	217311097	ESRRG
ENSG00000196497	4.999881834	5.306006045	protein_coding	14	24649425	24658170	IPO4
ENSG00000196498	11.52421735	11.09422652	protein_coding	12	124808961	125052135	NCOR2

ENSG00000196502	0.869158192	0.697730409	protein_coding	16	28616903	28634946	SULT1A1
ENSG00000196503	2.10647801	3.183544561	protein_coding	4	57371375	57390058	ARL9
ENSG00000196504	10.55117621	10.49516031	protein_coding	2	153508107	153574511	PRPF40A
ENSG00000196505	6.856698814	6.847095496	protein_coding	1	118412037	118472253	GDAP2
ENSG00000196507	7.003331296	7.37615181	protein_coding	X	102862379	102884618	TCEAL3
ENSG00000196510	8.78921631	9.067434305	protein_coding	12	110810705	110841535	ANAPC7
ENSG00000196511	6.882196036	7.04692429	protein_coding	7	144149034	144533488	TPK1
ENSG00000196517	8.898207576	9.231660172	protein_coding	1	44457172	44497139	SLC6A9
ENSG00000196526	10.5335842	10.24421147	protein_coding	4	7760441	7941653	AFAP1
ENSG00000196531	11.8994666	12.24655409	protein_coding	12	57106212	57125412	NACA
ENSG00000196532	2.238690726	2.334191469	protein_coding	6	26045639	26046097	HIST1H3C
ENSG00000196533	0	0.390640832	protein_coding	1	206238872	206288647	C1orf186
ENSG00000196535	9.852875742	9.005932523	protein_coding	17	27400528	27507430	MYO18A
ENSG00000196542	1.16343121	0.390640832	protein_coding	3	161062580	161090668	SPTSSB
ENSG00000196544	7.543580762	8.005247922	protein_coding	17	8091652	8093564	C17orf59
ENSG00000196547	8.193715877	9.272857279	protein_coding	15	91445448	91465814	MAN2A2
ENSG00000196549	1.407729925	1.16600992	protein_coding	3	154741913	154901497	MME
ENSG00000196550	4.097495944	4.048652948	protein_coding	1	206136916	206155151	FAM72A
ENSG00000196553	0.869158192	1.353254395	processed_transc	14	66953072	66965271	LINC00238
ENSG00000196557	6.92698535	7.002128877	protein_coding	16	1203241	1271771	CACNA1H
ENSG00000196559	3.950976309	3.608232228	protein_coding	11	36290834	36294971	C11orf55
ENSG00000196562	3.603959378	1.925536307	protein_coding	20	46285092	46415360	SULF2
ENSG00000196566	0	0.697730409	antisense	10	89369920	89419760	
ENSG00000196569	3.393122761	2.851901313	protein_coding	6	129204286	129837714	LAMA2
ENSG00000196576	12.15719972	12.02311826	protein_coding	22	50713408	50746056	PLXNB2
ENSG00000196584	8.625246244	8.540340653	protein_coding	7	152341864	152373250	XRCC2
ENSG00000196586	7.784382559	5.404622322	protein_coding	6	76458909	76629254	MYO6
ENSG00000196588	9.700687762	9.736525625	protein_coding	22	40806285	41032706	MKL1
ENSG00000196591	10.28674387	10.4634184	protein_coding	6	114254192	114332472	HDAC2
ENSG00000196593	8.156585168	7.255086103	lincRNA	13	24513696	24520921	ANKRD20A19P
ENSG00000196597	5.417620896	5.629711952	protein_coding	9	99578754	99637905	ZNF782
ENSG00000196604	2.10647801	1.802319292	protein_coding	2	130831108	130886795	POTEF
ENSG00000196605	5.018397273	5.283144778	protein_coding	19	9868151	9879410	ZNF846
ENSG00000196611	4.942865586	4.990346085	protein_coding	11	102660651	102668891	MMP1
ENSG00000196622	1.16343121	1.16600992	protein_coding	22	20455682	20461786	RIMBP3
ENSG00000196628	0.499066092	2.144285137	protein_coding	18	52889562	53332018	TCF4
ENSG00000196632	0.499066092	0.950786998	protein_coding	X	54219256	54385075	WNK3
ENSG00000196636	7.627388205	7.632773539	protein_coding	7	96745902	96811075	ACN9
ENSG00000196639	7.808521034	7.329495501	protein_coding	3	11178779	11305243	HRH1
ENSG00000196642	11.09442207	10.81477289	protein_coding	9	139694818	139735639	C9orf86
ENSG00000196644	1.960915222	1.16600992	protein_coding	1	145883868	145924373	GPR89C
ENSG00000196646	7.720795447	7.864110577	protein_coding	19	12273879	12300064	ZNF136
ENSG00000196648	4.097495944	4.048652948	protein_coding	15	83098710	83108085	
ENSG00000196652	8.261346552	7.966311947	protein_coding	7	99101607	99132323	ZKSCAN5
ENSG00000196653	6.032862302	5.125003832	protein_coding	3	44754135	44765323	ZNF502
ENSG00000196655	8.470794781	8.967911514	protein_coding	11	118889142	118896164	TRAPPC4
ENSG00000196659	4.77879189	4.888000647	protein_coding	2	178414890	178417742	TTC30B
ENSG00000196663	8.552470601	7.856387513	protein_coding	14	102829300	102968818	TECPR2
ENSG00000196664	2.57521082	3.570966319	protein_coding	X	12885202	12908499	TLR7
ENSG00000196666	0.869158192	0.950786998	protein_coding	11	47608198	47610746	FAM180B
ENSG00000196668	3.830412367	2.721932731	processed_transc	12	116971227	116974323	LINC00173
ENSG00000196670	7.08903805	7.359856617	protein_coding	5	180274611	180288286	ZFP62
ENSG00000196674	1.16343121	1.16600992	pseudogene	16	653555	653904	
ENSG00000196678	7.304268146	7.635032283	protein_coding	16	20791515	20911671	ERI2
ENSG00000196683	10.19612778	10.41626325	protein_coding	7	22852251	22862470	TOMM7
ENSG00000196684	6.592037289	5.361622823	protein_coding	19	16244838	16269344	HSH2D
ENSG00000196689	7.876068031	8.49131535	protein_coding	17	3468743	3500392	TRPV1
ENSG00000196690	0.869158192	0	protein_coding	1	17196254	17198668	
ENSG00000196693	7.73205733	7.709798264	protein_coding	10	43069633	43134018	ZNF33B
ENSG00000196696	4.352265886	4.761389014	processed_transc	16	70010200	70099851	PDXDC2P
ENSG00000196700	9.805686507	9.98296744	protein_coding	20	62588055	62680113	ZNF512B
ENSG00000196704	9.27736711	9.636359306	protein_coding	17	66244145	66253305	AMZ2
ENSG00000196705	7.572060955	7.408200217	protein_coding	19	21324840	21368805	ZNF431

ENSG00000196712	10.30476938	9.565299431	protein_coding	17	29421945	29708905	NF1
ENSG00000196715	10.05248241	9.201496347	protein_coding	7	65338254	65424530	VKORC1L1
ENSG00000196724	6.548110424	7.03670965	protein_coding	19	58433252	58446755	ZNF418
ENSG00000196730	7.454602977	7.373448694	protein_coding	9	90112143	90323548	DAPK1
ENSG00000196737	3.146112541	3.493416095	pseudogene	17	666805	668559	
ENSG00000196739	9.477987143	9.888149185	protein_coding	9	116917840	117074791	COL27A1
ENSG00000196741	3.502389126	4.622587519	protein_coding	X	47342970	47344207	CXorf24
ENSG00000196743	8.819887015	8.263555427	protein_coding	5	150591711	150650001	GM2A
ENSG00000196747	0.869158192	2.721932731	protein_coding	6	27775899	27776429	HIST1H2AI
ENSG00000196748	0.869158192	1.518964905	protein_coding	6	35744371	35747329	C6orf126
ENSG00000196754	8.966547133	5.446377228	protein_coding	1	153533584	153540366	S100A2
ENSG00000196756	9.889169155	10.03606464	processed_transc	20	37049235	37063996	
ENSG00000196757	8.508940744	8.560682282	protein_coding	19	12035900	12061588	ZNF700
ENSG00000196776	10.4154368	10.17604383	protein_coding	3	107762145	107809872	CD47
ENSG00000196781	9.986421812	9.821783646	protein_coding	9	84198598	84304220	TLE1
ENSG00000196782	3.502389126	4.17820932	protein_coding	4	140640419	141075338	MAML3
ENSG00000196787	5.4452544	5.506827567	protein_coding	6	27100832	27103070	HIST1H2AG
ENSG00000196792	8.464056506	8.360927302	protein_coding	14	31363005	31495607	STRN3
ENSG00000196793	7.356139979	7.462626902	protein_coding	10	44051792	44070066	ZNF239
ENSG00000196810	5.672682881	5.96747225	protein_coding	4	1243088	1245224	C4orf42
ENSG00000196811	3.211941663	2.652276565	protein_coding	2	233404437	233412546	CHRNG
ENSG00000196812	4.464551814	4.810179682	protein_coding	6	28092338	28097860	ZSCAN16
ENSG00000196814	6.469548347	6.885547525	protein_coding	9	129089128	129269320	FAM125B
ENSG00000196821	10.53479049	10.33870236	protein_coding	6	34555065	34664636	C6orf106
ENSG00000196834	0.499066092	0.390640832	protein_coding	2	131217028	131267239	POTEI
ENSG00000196839	7.950269163	7.978815021	protein_coding	20	43248163	43280874	ADA
ENSG00000196843	7.67776266	7.799123587	protein_coding	2	97202480	97218375	ARID5A
ENSG00000196844	2.35979773	3.32456471	protein_coding	11	125646008	125648714	PATE2
ENSG00000196850	8.768856552	8.395978102	protein_coding	12	110969120	111021125	PPTC7
ENSG00000196860	1.16343121	1.16600992	protein_coding	14	58862634	58875419	TOMM20L
ENSG00000196865	8.183497283	8.46616347	protein_coding	10	115614420	115676953	NHLRC2
ENSG00000196867	7.2307479	7.436965343	protein_coding	19	57050317	57068169	ZFP28
ENSG00000196872	2.35979773	1.802319292	protein_coding	2	99410309	99552722	C2orf55
ENSG00000196873	3.274897671	3.232099092	protein_coding	9	70849767	70914931	CBWD3
ENSG00000196876	1.16343121	0.950786998	protein_coding	12	51984050	52202297	SCN8A
ENSG00000196878	12.46708279	12.24821499	protein_coding	1	209788220	209825811	LAMB3
ENSG00000196890	1.16343121	0.697730409	protein_coding	1	228645808	228646259	HIST3H2BB
ENSG00000196893	0.499066092	0	antisense	17	18854938	18857462	
ENSG00000196911	5.175139844	5.516660875	protein_coding	6	117002350	117063029	KPNA5
ENSG00000196912	4.842525946	4.15321211	pseudogene	2	98108978	98206428	ANKRD36B
ENSG00000196914	10.73593044	10.77025591	protein_coding	11	120207787	120360645	ARHGEF12
ENSG00000196917	6.509364082	1.925536307	protein_coding	12	123104824	123215390	HCAR1
ENSG00000196922	6.902274257	7.402908012	protein_coding	8	146198975	146228281	ZNF252
ENSG00000196923	10.67231523	11.35845013	protein_coding	5	176910395	176924607	PDLIM7
ENSG00000196924	15.74364514	15.74549093	protein_coding	X	153576892	153603006	FLNA
ENSG00000196933	0	0.697730409	pseudogene	X	71264395	71264742	RPS26P11
ENSG00000196935	9.365879787	8.782698567	protein_coding	12	64238073	64541613	SRGAP1
ENSG00000196937	9.493786999	9.035301979	protein_coding	7	120988905	121036418	FAM3C
ENSG00000196943	9.159278686	9.113519788	protein_coding	14	24769068	24778330	C14orf21
ENSG00000196950	10.78980292	9.697613846	protein_coding	2	196440701	196602426	SLC39A10
ENSG00000196951	2.10647801	2.242360793	processed_transc	4	141204880	141294514	
ENSG00000196954	10.01724335	10.36513542	protein_coding	11	104813593	104840163	CASP4
ENSG00000196960	2.671945279	4.604259349	protein_coding	10	31650356	31652937	
ENSG00000196961	10.70661584	10.54885709	protein_coding	19	50270180	50310369	AP2A1
ENSG00000196963	7.832262274	8.157562549	protein_coding	5	140560980	140565793	PCDHB16
ENSG00000196966	2.762599152	3.368666104	protein_coding	6	26225383	26225844	HIST1H3E
ENSG00000196967	5.525114348	5.901455548	protein_coding	19	37641004	37663615	ZNF585A
ENSG00000196968	7.671926738	7.84081624	protein_coding	10	75532049	75538821	FUT11
ENSG00000196972	0.499066092	0.697730409	processed_transc	X	134229015	134232654	LINC00087
ENSG00000196975	8.510576602	8.923327429	protein_coding	2	69947923	70053596	ANXA4
ENSG00000196976	8.703135933	9.565891497	protein_coding	X	153706028	153707596	LAGE3
ENSG00000196979	0.499066092	0.950786998	protein_coding	9	132902889	132906498	
ENSG00000196981	5.958104008	6.174194902	protein_coding	3	122130715	122134882	WDR5B

ENSG00000196987	0	2.721932731	protein_coding	X	153146127	153154444	
ENSG00000196993	0	0.697730409	protein_coding	16	28763108	28784128	
ENSG00000196998	7.834876234	9.127223641	protein_coding	X	48929385	48958108	WDR45
ENSG00000197006	9.328343713	9.542618981	protein_coding	16	21608539	21668794	METTL9
ENSG00000197008	6.766474304	6.117167886	protein_coding	7	64254766	64294054	ZNF138
ENSG00000197013	6.610461229	6.470782801	protein_coding	19	21688437	21721079	ZNF429
ENSG00000197016	7.308035767	7.686030656	protein_coding	19	57078890	57094261	ZNF470
ENSG00000197019	5.83815965	6.084484284	protein_coding	19	40927499	40931932	SERTAD1
ENSG00000197020	7.609180529	7.675096386	protein_coding	19	21905568	21950330	ZNF100
ENSG00000197021	7.680671792	7.497803111	protein_coding	X	149097745	149107029	CXorf40B
ENSG00000197024	7.811178326	7.51752531	protein_coding	7	148823508	148880116	ZNF398
ENSG00000197037	7.803191723	7.560938532	protein_coding	7	99214569	99230030	ZNF498
ENSG00000197043	9.546566899	8.89250574	protein_coding	5	150480273	150537443	ANXA6
ENSG00000197044	5.741167503	6.795524906	protein_coding	19	11877815	11894893	ZNF441
ENSG00000197045	9.53214319	10.04543499	protein_coding	14	54941202	54955914	GMFB
ENSG00000197046	1.616589159	2.334191469	protein_coding	18	43405545	43422521	SIGLEC15
ENSG00000197049	1.16343121	1.353254395	protein_coding	1	721320	722513	
ENSG00000197050	6.283060568	6.365895578	protein_coding	19	37569337	37621212	ZNF420
ENSG00000197054	4.381167248	4.976162367	protein_coding	19	12035890	12090390	ZNF763
ENSG00000197056	7.530740159	7.659647832	protein_coding	1	35525387	35581460	ZMYM1
ENSG00000197061	0.499066092	1.667587519	protein_coding	6	26104104	26104518	HIST1H4C
ENSG00000197062	5.707331524	6.619638408	pseudogene	6	28234788	28245974	
ENSG00000197063	9.304024157	9.187714109	protein_coding	17	79876149	79885588	MAFG
ENSG00000197067	0.499066092	1.518964905	pseudogene	1	248246941	248247898	
ENSG00000197070	9.14670993	8.293892101	protein_coding	9	140500106	140509812	ARRDC1
ENSG00000197071	0.499066092	0	pseudogene	5	87514708	87515080	
ENSG00000197077	10.21079294	9.914421539	protein_coding	22	25421600	25593415	KIAA1671
ENSG00000197081	12.02438058	12.05414925	protein_coding	6	160390131	160534539	IGF2R
ENSG00000197083	0.499066092	0.950786998	pseudogene	5	150310207	150325851	ZNF300P1
ENSG00000197085	1.16343121	0.390640832	processed_transc	7	34386124	34911194	
ENSG00000197093	4.77879189	3.748356452	protein_coding	7	99756867	99766373	GAL3ST4
ENSG00000197102	13.36309292	12.53315011	protein_coding	14	102430865	102517129	DYNC1H1
ENSG00000197106	0.499066092	0.697730409	protein_coding	1	110693108	110744824	SLC6A17
ENSG00000197111	11.02715748	10.77564033	protein_coding	12	53835525	53874946	PCBP2
ENSG00000197114	8.900704842	8.53672099	protein_coding	20	62338794	62370456	ZGPAT
ENSG00000197119	7.491518048	8.237034976	protein_coding	14	100757448	100772884	SLC25A29
ENSG00000197121	8.377059993	8.273738696	protein_coding	2	197697728	197792520	PGAP1
ENSG00000197122	9.874634141	10.55811145	protein_coding	20	35973088	36034453	SRC
ENSG00000197123	0.499066092	0.390640832	protein_coding	7	63688852	63727309	ZNF679
ENSG00000197124	6.616550626	7.022976646	protein_coding	19	20115227	20150277	ZNF682
ENSG00000197128	7.210761201	7.157901395	protein_coding	19	57980954	57988938	ZNF772
ENSG00000197134	6.095241663	5.960284481	protein_coding	19	22235266	22273901	ZNF257
ENSG00000197136	10.9164443	10.71847539	protein_coding	11	65383244	65404910	PCNXL3
ENSG00000197140	1.616589159	2.144285137	protein_coding	8	38964509	39142435	ADAM32
ENSG00000197142	9.555310919	8.887777807	protein_coding	10	114133776	114188138	ACSL5
ENSG00000197146	4.322773689	3.906303962	pseudogene	6	167357853	167360151	
ENSG00000197147	6.554468172	6.064512706	protein_coding	1	89990395	90063423	LRRC8B
ENSG00000197149	2.762599152	2.652276565	pseudogene	11	17249817	17250698	
ENSG00000197150	9.754142144	9.631842439	protein_coding	7	150725510	150744869	ABCB8
ENSG00000197153	0.869158192	1.353254395	protein_coding	6	27858093	27860884	HIST1H3J
ENSG00000197157	12.14263609	11.6521071	protein_coding	7	127292234	127732661	SND1
ENSG00000197161	0.499066092	0.390640832	pseudogene	11	48366900	48373999	
ENSG00000197162	5.763291843	5.683080141	protein_coding	16	30585061	30597092	ZNF785
ENSG00000197165	1.799000381	1.353254395	protein_coding	16	28603264	28608430	SULT1A2
ENSG00000197168	0.869158192	0.390640832	protein_coding	13	52611093	52703214	NEK5
ENSG00000197170	9.791627126	9.935650135	protein_coding	17	65334032	65362743	PSMD12
ENSG00000197180	2.238690726	2.420525079	pseudogene	X	153656537	153656795	
ENSG00000197181	1.799000381	1.802319292	protein_coding	8	22132810	22215076	PIWIL2
ENSG00000197182	6.014532757	6.35497494	protein_coding	22	46449749	46509808	
ENSG00000197183	7.603059879	6.510691757	protein_coding	20	31030862	31172876	C20orf112
ENSG00000197191	4.19837882	3.64455972	protein_coding	9	140119087	140120763	C9orf169
ENSG00000197208	6.975172889	7.65742733	protein_coding	5	131630136	131679899	SLC22A4
ENSG00000197210	0	1.16600992	processed_transc	22	21468896	21478740	

ENSG00000197213	0.499066092	2.039052734	protein_coding	19	56701058	56704421	ZSCAN5B
ENSG00000197217	8.78516728	8.954409067	protein_coding	8	23243296	23315208	ENTPD4
ENSG00000197223	6.138202758	6.419292172	protein_coding	2	68268262	68338080	C1D
ENSG00000197226	10.51496026	10.58289346	protein_coding	5	179289066	179334859	TBC1D9B
ENSG00000197238	2.10647801	2.652276565	protein_coding	6	27791884	27792257	HIST1H4J
ENSG00000197245	0.869158192	1.353254395	protein_coding	1	26485511	26489119	FAM110D
ENSG00000197249	10.98718	7.780879746	protein_coding	14	94843084	94857030	SERPINA1
ENSG00000197251	0.499066092	0	antisense	6	33553883	33561115	LINC00336
ENSG00000197253	2.928422289	3.906303962	polymorphic_pse	16	1277272	1280214	TPSB2
ENSG00000197256	10.30382626	10.47291982	protein_coding	19	11274944	11308243	KANK2
ENSG00000197258	1.407729925	1.925536307	pseudogene	7	104308196	104310023	EIF4BP6
ENSG00000197261	6.283060568	5.32851069	protein_coding	6	49518113	49529620	C6orf141
ENSG00000197265	8.66103884	8.529454305	protein_coding	8	30435835	30515768	GTF2E2
ENSG00000197275	7.062518763	7.381542895	protein_coding	8	95384188	95487337	RAD54B
ENSG00000197279	5.929039784	4.932754837	protein_coding	6	28048753	28057341	ZNF165
ENSG00000197283	8.074911866	8.487570409	protein_coding	6	33387847	33421466	SYNGAP1
ENSG00000197284	0	0.390640832	pseudogene	5	70070478	70074135	
ENSG00000197291	0	0.697730409	protein_coding	17	40905951	40913275	
ENSG00000197296	7.778963161	7.960920138	protein_coding	20	42931478	42939809	FITM2
ENSG00000197299	7.63640659	7.819127564	protein_coding	15	91260558	91358859	BLM
ENSG00000197301	4.409500985	3.532712221	protein_coding	12	66245120	66275947	
ENSG00000197302	7.097770581	7.167295693	protein_coding	16	31724550	31806190	ZNF720
ENSG00000197312	8.591637531	8.435390709	protein_coding	1	15943995	15995539	DDI2
ENSG00000197321	9.801015254	10.5228685	protein_coding	10	29746267	30025710	SVIL
ENSG00000197323	9.176899044	9.131229537	protein_coding	1	114935399	115053781	TRIM33
ENSG00000197324	11.42426834	11.69333284	protein_coding	14	23340822	23350789	LRP10
ENSG00000197329	7.071412799	7.312661576	protein_coding	2	64319786	64371588	PELI1
ENSG00000197332	6.640654398	7.148445524	pseudogene	19	11750591	11797382	ZNF833P
ENSG00000197343	9.371291863	8.644911378	protein_coding	7	99156029	99205433	ZNF655
ENSG00000197345	8.919297816	9.422885181	protein_coding	11	68658744	68671303	MRPL21
ENSG00000197355	7.90122817	8.667154767	protein_coding	9	139971953	139978991	UAP1L1
ENSG00000197358	5.91922012	7.439552137	pseudogene	14	28733596	28735180	BNIP3P1
ENSG00000197361	1.407729925	1.16600992	protein_coding	15	63889552	63894627	FBXL22
ENSG00000197362	7.304268146	6.885547525	protein_coding	7	148766735	148787874	ZNF786
ENSG00000197363	5.417620896	5.66550919	protein_coding	8	146024261	146036554	ZNF517
ENSG00000197372	7.935731106	7.621426466	protein_coding	19	23835708	23870017	ZNF675
ENSG00000197375	7.720795447	7.621426466	protein_coding	5	131705444	131731306	SLC22A5
ENSG00000197380	1.616589159	1.925536307	protein_coding	19	47150869	47164395	DACT3
ENSG00000197381	8.276809264	7.811159227	protein_coding	21	46493768	46646478	ADARB1
ENSG00000197385	4.19837882	4.075511708	protein_coding	3	32023263	32033120	ZNF860
ENSG00000197386	10.63755355	10.55095198	protein_coding	4	3076408	3245676	HTT
ENSG00000197405	2.928422289	2.501982735	protein_coding	19	47813104	47825327	C5AR1
ENSG00000197406	0.869158192	0	protein_coding	14	102027688	102029789	
ENSG00000197408	0	0.390640832	protein_coding	19	41497204	41524301	CYP2B6
ENSG00000197409	3.335220907	3.133298822	protein_coding	6	26197068	26199521	HIST1H3D
ENSG00000197410	0	1.802319292	protein_coding	4	155155527	155412930	DCHS2
ENSG00000197415	1.407729925	1.802319292	protein_coding	3	156977531	157251408	VEPH1
ENSG00000197417	8.787867896	8.871585941	protein_coding	17	3511893	3539616	SHPK
ENSG00000197429	6.646617938	6.568558581	protein_coding	1	46159996	46216322	IPP
ENSG00000197430	0.499066092	0	protein_coding	10	98102973	98119092	OPALIN
ENSG00000197442	7.976547636	7.58917149	protein_coding	6	136878185	137113656	MAP3K5
ENSG00000197444	2.10647801	0	protein_coding	10	50942689	50970425	OGDHL
ENSG00000197446	1.616589159	0	protein_coding	19	41620337	41634271	CYP2F1
ENSG00000197448	9.366783211	9.213637661	protein_coding	7	142941186	142967947	GSTK1
ENSG00000197451	10.79316709	10.80125976	protein_coding	5	177631508	177638164	HNRNPAB
ENSG00000197457	10.52349233	9.074940905	protein_coding	20	62271061	62284780	STMN3
ENSG00000197459	4.322773689	4.761389014	protein_coding	6	26251879	26252303	HIST1H2BH
ENSG00000197461	7.959880444	7.664078611	protein_coding	7	536895	559933	PDGFA
ENSG00000197462	0	0.390640832	lincRNA	7	125557925	125573886	
ENSG00000197467	7.64536895	7.652976045	protein_coding	10	71561644	71724031	COL13A1
ENSG00000197471	0	0.390640832	protein_coding	16	29674300	29682187	SPN
ENSG00000197472	4.593635215	4.710890357	protein_coding	1	247108849	247171395	ZNF695
ENSG00000197479	4.491309013	3.133298822	protein_coding	5	140579183	140582618	PCDHB11

ENSG00000197483	8.324088959	7.563312496	protein_coding	19	55987699	55995854	ZNF628
ENSG00000197497	6.086493765	6.097646709	protein_coding	19	53666552	53696619	ZNF665
ENSG00000197498	7.990682129	8.001751392	protein_coding	6	111303218	111347303	RPF2
ENSG00000197506	4.842525946	6.174194902	protein_coding	9	86890372	86955672	SLC28A3
ENSG00000197511	2.471521042	2.501982735	protein_coding	1	16862255	16864669	
ENSG00000197520	0.499066092	1.16600992	protein_coding	1	222910549	222924147	FAM177B
ENSG00000197530	8.103376106	8.825850709	protein_coding	1	1550795	1565990	MIB2
ENSG00000197535	8.836938493	9.050611798	protein_coding	15	52599480	52821247	MYO5A
ENSG00000197536	4.800350936	5.564846512	protein_coding	5	131746328	131811736	C5orf56
ENSG00000197540	1.407729925	2.501982735	protein_coding	19	544027	549919	GZMM
ENSG00000197548	9.330197577	8.961626095	protein_coding	3	11313995	11599139	ATG7
ENSG00000197550	1.616589159	1.16600992	pseudogene	9	69830288	69848759	
ENSG00000197555	10.1438565	10.26987495	protein_coding	14	71787166	72207946	SIPA1L1
ENSG00000197557	5.550781289	5.57429348	protein_coding	2	178479026	178483694	TTC30A
ENSG00000197558	7.08903805	6.310446475	processed_transc	7	149473131	149531068	SSPO
ENSG00000197561	0.869158192	0.950786998	protein_coding	19	852291	856242	ELANE
ENSG00000197562	8.311006074	8.208501977	protein_coding	16	639357	679272	RAB40C
ENSG00000197563	8.048129203	8.382597612	protein_coding	18	59711460	59854289	PIGN
ENSG00000197565	7.246540262	6.587342979	protein_coding	X	107386780	107682727	COL4A6
ENSG00000197566	5.431503808	6.3217081	protein_coding	17	16524051	16557158	ZNF624
ENSG00000197568	6.050961887	5.767861411	protein_coding	1	70820488	70851022	HHLA3
ENSG00000197575	0.499066092	1.16600992	pseudogene	5	116051929	116052387	RPS17P2
ENSG00000197576	0.499066092	0	protein_coding	7	27168126	27170418	HOXA4
ENSG00000197579	7.933293789	8.098224647	protein_coding	9	32540542	32552551	TOPORS
ENSG00000197580	2.762599152	2.851901313	protein_coding	11	112046190	112095422	BCO2
ENSG00000197582	1.960915222	2.039052734	pseudogene	X	13396854	13397459	GPX1P1
ENSG00000197586	10.42977299	10.62725021	protein_coding	20	25176329	25207365	ENTPD6
ENSG00000197587	4.734682531	3.411459265	protein_coding	1	46972668	46979898	DMBX1
ENSG00000197594	5.175139844	6.839280559	protein_coding	6	132129156	132216295	ENPP1
ENSG00000197599	5.191549282	5.41517489	protein_coding	16	1484384	1494557	CCDC154
ENSG00000197601	10.27042264	10.55721845	protein_coding	11	13690217	13753893	FAR1
ENSG00000197603	8.057112115	8.567793748	protein_coding	5	37106330	37249530	C5orf42
ENSG00000197604	2.57521082	2.039052734	protein_coding	10	72194585	72196314	
ENSG00000197608	9.934355333	10.47984797	protein_coding	19	52567719	52599018	ZNF841
ENSG00000197614	0	0.390640832	protein_coding	12	8789942	8815484	MFAP5
ENSG00000197617	0.499066092	0.390640832	pseudogene	1	247419374	247420447	VN1R5
ENSG00000197619	7.334137164	7.65742733	protein_coding	19	52494588	52511483	ZNF615
ENSG00000197620	7.003331296	6.664555628	protein_coding	X	148621900	148632055	CXorf40A
ENSG00000197622	10.15069164	10.65673	protein_coding	1	151023447	151032249	CDC42SE1
ENSG00000197632	8.853790788	4.274079748	protein_coding	18	61538926	61571124	SERPINB2
ENSG00000197635	8.092494681	6.110690132	protein_coding	2	162848751	162931052	DPP4
ENSG00000197641	0.499066092	0.697730409	protein_coding	18	61254223	61271873	SERPINB13
ENSG00000197646	5.458875168	6.515603638	protein_coding	9	5510545	5571282	PDCD1LG2
ENSG00000197647	5.899377978	6.117167886	protein_coding	19	12125547	12146556	ZNF433
ENSG00000197653	2.847891871	3.64455972	protein_coding	12	124247042	124420753	DNAH10
ENSG00000197670	3.698846687	3.87606762	processed_transc	20	52169309	52191847	
ENSG00000197681	0	0.390640832	protein_coding	17	36337711	36358166	TBC1D3
ENSG00000197689	0.499066092	0.950786998	protein_coding	17	28886584	28890507	TBC1D29
ENSG00000197694	11.98373622	12.03695669	protein_coding	9	131314866	131395941	SPTAN1
ENSG00000197696	7.409722215	7.724721747	protein_coding	15	85198360	85201794	NMB
ENSG00000197697	0.869158192	1.802319292	protein_coding	6	26183958	26184454	HIST1H2BE
ENSG00000197701	3.077135474	3.748356452	protein_coding	4	53179	88099	ZNF595
ENSG00000197702	9.174837196	9.665379195	protein_coding	11	12398732	12552348	PARVA
ENSG00000197712	9.217529022	9.115138778	protein_coding	4	38869298	38947360	FAM114A1
ENSG00000197713	8.410739551	8.162268448	protein_coding	2	210867289	210886300	RPE
ENSG00000197714	6.610461229	6.505763096	protein_coding	19	57791419	57805436	ZNF460
ENSG00000197721	0.499066092	0	protein_coding	1	207818458	207911761	CR1L
ENSG00000197723	0.499066092	1.16600992	protein_coding	17	40274756	40275371	HSPB9
ENSG00000197724	8.878072281	8.793860943	protein_coding	9	96338689	96441869	PHF2
ENSG00000197727	0	0.697730409	pseudogene	16	691039	691688	
ENSG00000197728	6.228441838	7.033288635	protein_coding	12	56435637	56438116	RPS26
ENSG00000197734	0.499066092	0	protein_coding	14	78227173	78236085	C14orf178
ENSG00000197744	1.799000381	1.16600992	pseudogene	5	118309491	118309817	PTMAP2

ENSG00000197746	12.95162415	13.39240736	protein_coding	10	73576055	73611126	PSAP
ENSG00000197747	11.35083198	10.75268144	protein_coding	1	151955391	151966866	S100A10
ENSG00000197748	3.335220907	3.781359661	protein_coding	10	105889646	105992120	WDR96
ENSG00000197753	1.407729925	2.242360793	protein_coding	6	35773070	35801651	LHFPL5
ENSG00000197756	11.23538177	11.68040503	protein_coding	2	217362912	217443903	RPL37A
ENSG00000197757	5.036678094	4.94736961	protein_coding	12	54384408	54424607	HOXC6
ENSG00000197763	6.738827102	6.819555981	protein_coding	3	126290622	126373998	TXNRD3
ENSG00000197766	6.171673665	6.235021771	protein_coding	19	859665	863606	CFD
ENSG00000197768	1.960915222	3.081239798	protein_coding	9	140145713	140147934	C9orf173
ENSG00000197769	1.616589159	1.925536307	protein_coding	1	242158792	242162375	MAP1LC3C
ENSG00000197771	10.06867432	10.57791229	protein_coding	10	121588972	121652068	MCMBP
ENSG00000197774	7.737655476	7.754113435	protein_coding	16	1823208	1831709	EME2
ENSG00000197776	2.238690726	2.652276565	protein_coding	14	50159823	50219870	KLHDC1
ENSG00000197779	6.305850085	6.771086752	protein_coding	X	47696301	47861960	ZNF81
ENSG00000197780	7.703736051	7.890820294	protein_coding	1	109605121	109618624	TAF13
ENSG00000197782	5.036678094	5.200150955	protein_coding	19	40570428	40596845	ZNF780A
ENSG00000197785	9.487155506	9.379761309	protein_coding	1	1447531	1470067	ATAD3A
ENSG00000197798	7.63640659	8.063427911	protein_coding	11	126081309	126132881	FAM118B
ENSG00000197808	5.4452544	5.446377228	protein_coding	19	37128283	37157743	ZNF461
ENSG00000197813	4.230497448	3.679994897	protein_coding	19	49831424	49843559	
ENSG00000197815	1.960915222	2.912743273	pseudogene	17	17761583	17762295	
ENSG00000197816	2.471521042	3.232099092	protein_coding	9	100000765	100140806	C9orf174
ENSG00000197818	8.227927477	8.275187599	protein_coding	20	48429250	48508779	SLC9A8
ENSG00000197822	6.050961887	5.486957566	protein_coding	5	68788119	68853931	OCLN
ENSG00000197825	0	0.390640832	pseudogene	17	26529824	26530831	
ENSG00000197830	2.471521042	1.925536307	pseudogene	5	137881629	137882992	
ENSG00000197837	5.512107582	4.385599336	protein_coding	12	14920933	14924065	HIST4H4
ENSG00000197841	5.207774174	5.792353798	protein_coding	19	35225061	35233777	ZNF181
ENSG00000197844	0.499066092	1.802319292	pseudogene	1	149689368	149689854	
ENSG00000197846	2.928422289	2.334191469	protein_coding	6	26199748	26200942	HIST1H2BF
ENSG00000197847	5.054730169	3.133298822	pseudogene	11	64981311	65010228	SLC22A20
ENSG00000197848	0	0.390640832	pseudogene	6	26864126	26865739	
ENSG00000197852	4.165528823	4.826083323	protein_coding	1	112223252	112298446	FAM212B
ENSG00000197857	7.423680977	7.512619979	protein_coding	19	12358092	12405702	ZNF44
ENSG00000197858	9.894817144	10.25636687	protein_coding	8	145137493	145141119	GPAA1
ENSG00000197859	2.35979773	2.144285137	protein_coding	9	136397286	136440641	ADAMTSL2
ENSG00000197860	6.851544845	6.592001111	protein_coding	5	64961755	65018862	SGTB
ENSG00000197863	5.60078458	5.742946029	protein_coding	19	37309224	37341215	ZNF790
ENSG00000197870	1.616589159	0.390640832	polymorphic_pse	12	11418857	11422739	PRB3
ENSG00000197879	11.87966549	12.06664909	protein_coding	17	1367480	1395995	MYO1C
ENSG00000197882	0	0.697730409	pseudogene	11	86543582	86544491	OR7E13P
ENSG00000197885	5.763291843	6.104183162	protein_coding	3	23933151	23988082	NKIRAS1
ENSG00000197889	1.616589159	0.697730409	protein_coding	10	15001438	15030049	MEIG1
ENSG00000197892	7.734859118	7.527286218	protein_coding	8	28924796	29120641	KIF13B
ENSG00000197893	0.499066092	0	protein_coding	10	115348475	115423805	NRAP
ENSG00000197894	9.187164314	9.580028658	protein_coding	4	99992132	100009952	ADH5
ENSG00000197903	8.640267888	8.953504395	protein_coding	6	27106073	27114619	HIST1H2BK
ENSG00000197905	8.738466022	8.146522054	protein_coding	12	3068496	3149839	TEAD4
ENSG00000197912	9.959725563	10.18184912	protein_coding	16	89557325	89624176	SPG7
ENSG00000197914	1.799000381	0.697730409	protein_coding	6	27798952	27799305	HIST1H4K
ENSG00000197915	3.077135474	3.32456471	protein_coding	1	152184558	152196669	HRNR
ENSG00000197927	5.431503808	5.742946029	protein_coding	2	132479948	132524973	C2orf27A
ENSG00000197928	6.535310252	6.601272494	protein_coding	19	53727087	53758126	ZNF677
ENSG00000197930	10.8858896	10.82791383	protein_coding	14	53106634	53162618	ERO1L
ENSG00000197932	5.625150983	5.526427614	protein_coding	X	154114635	154116335	F8A1
ENSG00000197933	6.738827102	6.926693689	protein_coding	19	11832081	11849824	ZNF823
ENSG00000197935	4.322773689	5.045726316	protein_coding	6	28962562	28973387	ZNF311
ENSG00000197937	7.768062907	8.138583935	protein_coding	19	53641958	53662322	ZNF347
ENSG00000197943	5.270908378	3.87606762	protein_coding	16	81772702	81991899	PLCG2
ENSG00000197948	7.990682129	7.551403417	protein_coding	5	141018869	141030986	FCHSD1
ENSG00000197951	7.784382559	7.928139131	protein_coding	19	57106664	57135540	ZNF71
ENSG00000197956	12.7820982	13.1210813	protein_coding	1	153507075	153508720	S100A6
ENSG00000197958	11.62808663	11.72628042	protein_coding	9	130209953	130213684	RPL12

ENSG00000197959	4.437288965	4.888000647	protein_coding	1	171810621	172387606	DNM3
ENSG00000197961	9.204449373	9.145559356	protein_coding	19	9672859	9695209	ZNF121
ENSG00000197965	10.32629262	10.50445563	protein_coding	1	167690429	167761156	MPZL1
ENSG00000197969	9.609746388	9.652057897	protein_coding	9	79792269	80036457	VPS13A
ENSG00000197971	8.890689734	7.933654619	protein_coding	18	74690783	74845639	MBP
ENSG00000197976	8.970118399	9.240587696	protein_coding	X	1710486	1721407	AKAP17A
ENSG00000197977	0.869158192	0.950786998	protein_coding	6	10980992	11044547	ELOVL2
ENSG00000197980	3.603959378	2.420525079	protein_coding	3	156543270	156697347	
ENSG00000197982	7.420203928	8.180940029	protein_coding	1	38272651	38275126	C1orf122
ENSG00000197989	7.906207969	8.748683701	processed_transc	1	28905050	28909495	SNHG12
ENSG00000197993	0.499066092	1.16600992	protein_coding	7	142638201	142659768	KEL
ENSG00000198000	9.582785382	9.772877733	protein_coding	9	95059640	95087918	NOL8
ENSG00000198001	7.214780754	7.148445524	protein_coding	12	44152747	44183346	IRAK4
ENSG00000198003	4.543379137	5.175534283	protein_coding	19	11531273	11546603	CCDC151
ENSG00000198010	0.499066092	0.390640832	protein_coding	8	1449531	1656642	DLGAP2
ENSG00000198015	8.329659793	8.496293532	protein_coding	12	93861264	93897545	MRPL42
ENSG00000198018	7.084651883	7.652976045	protein_coding	10	101419263	101465997	ENTPD7
ENSG00000198026	9.259966624	9.166009895	protein_coding	20	44577292	44600833	ZNF335
ENSG00000198028	7.218789138	7.527286218	protein_coding	19	9577031	9609279	ZNF560
ENSG00000198034	12.98282172	13.52033115	protein_coding	X	71475529	71497150	RPS4X
ENSG00000198039	6.658471572	5.96747225	protein_coding	7	64330550	64391344	ZNF273
ENSG00000198040	7.671926738	8.138583935	protein_coding	12	133613878	133639885	ZNF84
ENSG00000198042	8.156585168	8.365015357	protein_coding	8	33342268	33358687	MAK16
ENSG00000198046	6.798966424	6.95589788	protein_coding	19	56950696	56988770	ZNF667
ENSG00000198053	10.37963191	10.60955597	protein_coding	20	1875154	1920543	SIRPA
ENSG00000198055	8.955779984	9.315025211	protein_coding	5	176830205	176869902	GRK6
ENSG00000198056	8.197783135	8.66273344	protein_coding	12	57125372	57146157	PRIM1
ENSG00000198060	8.885655987	9.155034101	protein_coding	10	94050920	94113721	5-Mar
ENSG00000198064	5.637181576	5.294620694	protein_coding	16	30234258	30265831	
ENSG00000198074	3.077135474	1.353254395	protein_coding	7	134212344	134226160	AKR1B10
ENSG00000198077	0.499066092	0	protein_coding	19	41381344	41388657	CYP2A7
ENSG00000198081	6.469548347	6.559073862	protein_coding	18	5289018	5296039	ZFP161
ENSG00000198087	9.499564655	9.723848155	protein_coding	6	47445525	47594999	CD2AP
ENSG00000198088	6.171673665	5.824376633	protein_coding	X	106366657	106449670	NUP62CL
ENSG00000198089	9.482161805	9.930602076	protein_coding	22	31884674	32014572	SFI1
ENSG00000198090	1.16343121	0	protein_coding	17	39295685	39296739	KRTAP4-6
ENSG00000198093	7.596913151	7.811159227	protein_coding	19	52392489	52408305	ZNF649
ENSG00000198100	1.16343121	0	pseudogene	9	74576256	74576469	
ENSG00000198105	6.421663777	7.666288907	protein_coding	10	38091751	38147034	ZNF248
ENSG00000198106	7.363400307	7.384230902	protein_coding	16	29262829	29519817	SNX29P2
ENSG00000198108	5.417620896	3.608232228	protein_coding	5	129240165	129522327	CHSY3
ENSG00000198113	10.00626968	9.569438796	protein_coding	9	140172201	140177093	C9orf167
ENSG00000198121	6.804311337	4.342020395	protein_coding	9	113635543	113800981	LPAR1
ENSG00000198125	7.845284967	7.609989438	protein_coding	22	36002811	36033998	MB
ENSG00000198130	7.474855471	7.454976247	protein_coding	2	191054461	191208919	HIBCH
ENSG00000198131	9.394512382	9.29873313	protein_coding	19	58740070	58775010	ZNF544
ENSG00000198133	4.883502971	4.29708397	protein_coding	14	67913801	68000456	TMEM229B
ENSG00000198134	1.616589159	1.802319292	pseudogene	12	12264097	12264423	
ENSG00000198142	9.490475063	8.063427911	protein_coding	2	110371911	110376563	ANKRD57
ENSG00000198146	8.096857104	8.378559146	protein_coding	15	35270542	35280488	ZNF770
ENSG00000198150	4.437288965	4.857374129	protein_coding	17	8261731	8263538	
ENSG00000198153	0	0.390640832	protein_coding	19	22867969	22869468	
ENSG00000198155	5.431503808	5.486957566	pseudogene	4	206399	249774	ZNF876P
ENSG00000198156	0.499066092	0.950786998	protein_coding	16	28353876	28374829	
ENSG00000198157	6.163378456	6.097646709	protein_coding	X	80369200	80457441	HMGN5
ENSG00000198160	8.591637531	8.822881621	protein_coding	1	67390578	67454302	MIER1
ENSG00000198161	0.499066092	0	protein_coding	1	149553003	149553787	PPIAL4C
ENSG00000198162	8.704565874	8.92701904	protein_coding	1	117910071	118071494	MAN1A2
ENSG00000198168	7.102117104	7.586839732	protein_coding	11	22835345	22851845	SVIP
ENSG00000198169	6.9512803	7.510161044	protein_coding	8	145946298	145981802	ZNF251
ENSG00000198171	9.370391259	10.33627517	protein_coding	20	3170996	3185331	DDRGK1
ENSG00000198176	9.9233443	9.721192935	protein_coding	13	114239013	114295504	TFDP1
ENSG00000198182	7.218789138	6.919299326	protein_coding	19	38187264	38210691	ZNF607

ENSG00000198185	4.923348194	4.29708397	protein_coding	20	45129709	45142198	ZNF334
ENSG00000198189	7.96466218	8.254769334	protein_coding	4	88257762	88312538	HSD17B11
ENSG00000198198	8.662511098	8.776573399	protein_coding	1	43855553	43918321	SZT2
ENSG00000198203	0.499066092	0.390640832	protein_coding	2	108905095	108926371	SULT1C2
ENSG00000198205	5.286269488	5.674321415	protein_coding	X	57931864	57937067	ZXDA
ENSG00000198208	7.444369108	7.926295939	protein_coding	14	75370657	75390099	RPS6KL1
ENSG00000198211	2.671945279	1.16600992	protein_coding	16	89987800	90005169	TUBB3
ENSG00000198218	9.841872234	9.652615414	protein_coding	3	49067140	49131796	QRICH1
ENSG00000198221	3.146112541	3.453019579	processed_transc	6	168224556	168227466	C6orf124
ENSG00000198223	3.652182994	3.64455972	protein_coding	X	1387693	1429274	CSF2RA
ENSG00000198225	4.131913373	4.406904905	protein_coding	6	63921351	63922929	FKBP1C
ENSG00000198231	10.58844284	10.88240534	protein_coding	17	61851567	61896676	DDX42
ENSG00000198237	1.16343121	1.518964905	pseudogene	5	69171098	69274436	
ENSG00000198242	10.05696733	10.05811545	protein_coding	17	27046411	27051377	RPL23A
ENSG00000198246	6.676071387	6.403479675	protein_coding	10	73079015	73123142	SLC29A3
ENSG00000198250	0.499066092	0.390640832	protein_coding	10	47657581	47701443	ANTXRL
ENSG00000198252	8.371669553	8.25035614	protein_coding	14	53196898	53241716	STYX
ENSG00000198258	10.02241241	10.71740993	protein_coding	19	9938568	9940797	UBL5
ENSG00000198265	9.297405929	9.342237258	protein_coding	17	65066555	65241319	HELZ
ENSG00000198270	5.124761037	6.057793599	protein_coding	12	112369102	112450935	TMEM116
ENSG00000198276	9.003030709	9.078264676	protein_coding	20	62571186	62587769	UCKL1
ENSG00000198286	8.195750939	5.734544355	protein_coding	7	2945775	3083579	CARD11
ENSG00000198298	6.220467583	6.198834244	protein_coding	10	44101855	44113351	ZNF485
ENSG00000198300	5.40360309	5.974624386	protein_coding	19	57321445	57352096	ZIM2
ENSG00000198301	8.98077946	9.253141197	protein_coding	4	76862103	76912115	SDAD1
ENSG00000198315	7.367016815	7.586839732	protein_coding	6	28109716	28124089	ZNF192
ENSG00000198324	8.504022011	7.394933125	protein_coding	12	111798339	111806925	FAM109A
ENSG00000198326	0.869158192	1.16600992	protein_coding	20	2795614	2800930	TMEM239
ENSG00000198327	1.16343121	0.697730409	protein_coding	6	26240561	26240976	HIST1H4F
ENSG00000198331	7.511262389	8.093304774	protein_coding	11	125753509	125770543	HYLS1
ENSG00000198336	0	1.353254395	protein_coding	17	45286428	45301045	MYL4
ENSG00000198339	4.712111592	3.906303962	protein_coding	6	27107076	27108418	HIST1H4I
ENSG00000198342	4.322773689	4.872768657	protein_coding	19	12460185	12476719	ZNF442
ENSG00000198346	7.406211319	7.04352744	protein_coding	19	53970989	54006950	ZNF813
ENSG00000198353	5.588445295	6.505763096	protein_coding	12	54410715	54449814	HOXC4
ENSG00000198355	9.699971044	9.576507334	protein_coding	22	50354161	50357728	PIM3
ENSG00000198356	10.44311177	11.066044	protein_coding	19	12848306	12859137	ASNA1
ENSG00000198358	0	0.697730409	processed_transc	1	160902255	160919712	
ENSG00000198363	11.75478782	10.7532014	protein_coding	8	62413115	62627199	ASPH
ENSG00000198366	2.762599152	1.667587519	protein_coding	6	26020718	26021186	HIST1H3A
ENSG00000198369	8.346244381	8.038049013	protein_coding	2	65537985	65659771	SPRED2
ENSG00000198373	9.873363279	9.567666236	protein_coding	16	69796209	69975644	WWP2
ENSG00000198374	2.238690726	2.242360793	protein_coding	6	27833034	27833606	HIST1H2AL
ENSG00000198380	9.255095676	9.074940905	protein_coding	2	69546905	69614382	GFPT1
ENSG00000198382	8.002356002	8.326402026	protein_coding	11	75526212	75854239	UVRAG
ENSG00000198388	0	0.390640832	pseudogene	16	14548061	14548155	
ENSG00000198393	7.19863491	7.022976646	protein_coding	12	133562951	133589154	ZNF26
ENSG00000198399	8.798620088	8.443145654	protein_coding	2	24425733	24583583	ITSN2
ENSG00000198400	1.799000381	1.925536307	protein_coding	1	156785432	156851642	NTRK1
ENSG00000198406	2.471521042	2.420525079	pseudogene	3	116364592	116365574	BZW1P2
ENSG00000198408	11.16908489	11.21785423	protein_coding	10	103544200	103578696	MGEA5
ENSG00000198414	0.499066092	0.697730409	pseudogene	X	44143162	44145492	TATDN2P1
ENSG00000198417	3.989024711	3.532712221	protein_coding	16	56691606	56694610	MT1F
ENSG00000198420	9.31529953	9.498022112	protein_coding	7	143548468	143599291	FAM115A
ENSG00000198429	6.393570237	5.871116342	protein_coding	19	11998599	12025144	ZNF69
ENSG00000198431	12.4886153	11.76314651	protein_coding	12	104609557	104744061	TXNRD1
ENSG00000198435	7.337827682	7.694718794	protein_coding	9	140194083	140196703	NRARP
ENSG00000198440	6.379315619	6.937714655	protein_coding	19	56915383	56938733	ZNF583
ENSG00000198443	0.499066092	0	protein_coding	17	39340354	39341594	KRTAP4-1
ENSG00000198453	6.462804234	6.677762549	protein_coding	19	37407231	37488834	ZNF568
ENSG00000198455	7.654275977	7.752033772	protein_coding	X	57618269	57623906	ZXDB
ENSG00000198464	7.921045056	7.966311947	protein_coding	19	52800425	52827333	ZNF480
ENSG00000198466	9.212512411	9.253141197	protein_coding	19	58331094	58376485	ZNF587

ENSG00000198467	7.916116295	9.292307546	protein_coding	9	35681989	35691017	TPM2
ENSG00000198468	5.525114348	5.893930351	processed_transc	1	213025450	213031430	FLVCR1-AS1
ENSG00000198471	0	0.390640832	protein_coding	3	187416047	187420345	RTP2
ENSG00000198477	7.123655438	7.275499917	protein_coding	22	22838767	22863505	ZNF280B
ENSG00000198478	5.255381949	6.204928885	protein_coding	6	80341000	80413372	SH3BGRL2
ENSG00000198482	8.12703021	7.842771865	protein_coding	19	53030905	53077383	ZNF808
ENSG00000198488	0.499066092	0	protein_coding	11	76745385	76753096	B3GNT6
ENSG00000198492	9.849001727	9.664273773	protein_coding	1	29063133	29096287	YTHDF2
ENSG00000198496	5.967663407	5.960284481	protein_coding	17	41277609	41305688	NBR2
ENSG00000198498	7.988335976	8.096586553	protein_coding	4	164415594	164441691	C4orf43
ENSG00000198513	3.448790144	2.971122874	protein_coding	14	50999227	51099786	ATL1
ENSG00000198515	1.616589159	1.925536307	protein_coding	4	47937994	48018689	CNGA1
ENSG00000198517	9.407740769	8.741364159	protein_coding	7	1570350	1582679	MAFK
ENSG00000198518	3.871730003	2.579085888	protein_coding	6	26204858	26206266	HIST1H4E
ENSG00000198520	2.10647801	2.334191469	protein_coding	1	45140364	45191263	C1orf228
ENSG00000198521	7.717966186	7.681666888	protein_coding	19	21990085	22034830	ZNF43
ENSG00000198522	8.221948786	8.427593853	protein_coding	2	27851114	27874375	GPN1
ENSG00000198523	0.499066092	0.697730409	protein_coding	6	118869461	118881893	PLN
ENSG00000198526	0	0.390640832	pseudogene	2	147345012	147346878	PABPC1P2
ENSG00000198535	1.799000381	2.334191469	protein_coding	15	62359176	62361103	C2CD4A
ENSG00000198538	8.755121984	8.387964709	protein_coding	19	53300662	53324922	ZNF28
ENSG00000198542	2.671945279	2.579085888	protein_coding	13	102104966	102371145	ITGBL1
ENSG00000198546	7.663128351	8.122575449	protein_coding	10	135121979	135166033	ZNF511
ENSG00000198551	7.642387679	8.029489191	protein_coding	19	11708235	11729974	ZNF627
ENSG00000198553	1.16343121	0.390640832	protein_coding	13	50589390	50595058	KCNRG
ENSG00000198554	8.931561329	9.241329169	protein_coding	14	55405668	55493823	WDHD1
ENSG00000198555	0.499066092	1.16600992	pseudogene	16	33778506	33790097	
ENSG00000198556	7.119373418	7.38691391	protein_coding	7	99070464	99101273	ZNF789
ENSG00000198558	0	0.390640832	protein_coding	6	27840926	27841289	HIST1H4L
ENSG00000198561	11.70350483	11.42114433	protein_coding	11	57520715	57587018	CTNND1
ENSG00000198563	10.10160874	10.27567734	protein_coding	6	31497996	31510225	DDX39B
ENSG00000198569	3.448790144	3.279072565	protein_coding	9	140125209	140131006	SLC34A3
ENSG00000198573	0	0.390640832	protein_coding	X	140335596	140336629	SPANXC
ENSG00000198576	3.335220907	1.802319292	protein_coding	8	143692410	143695833	ARC
ENSG00000198580	3.989024711	4.075511708	protein_coding	7	6713376	6715991	
ENSG00000198585	8.653654925	8.69448524	protein_coding	3	131100515	131107674	NUDT16
ENSG00000198586	9.105088364	8.677053487	protein_coding	2	171847333	172087824	TLK1
ENSG00000198589	9.740963079	9.420268302	protein_coding	4	151185594	151936879	LRBA
ENSG00000198590	2.57521082	1.925536307	protein_coding	3	37427760	37476988	C3orf35
ENSG00000198598	8.295907473	7.760334499	protein_coding	12	132312938	132336328	MMP17
ENSG00000198604	10.11296043	10.38238014	protein_coding	14	35221937	35344853	BAZ1A
ENSG00000198610	0.499066092	0	protein_coding	10	5237426	5260912	AKR1C4
ENSG00000198612	8.970118399	8.916844293	protein_coding	2	237993955	238009109	COPS8
ENSG00000198618	3.652182994	2.579085888	pseudogene	21	20230097	20230594	PPIAP22
ENSG00000198624	4.517578978	3.608232228	protein_coding	5	150560613	150603706	CCDC69
ENSG00000198625	8.282565319	8.702045897	protein_coding	1	204485511	204542871	MDM4
ENSG00000198626	2.35979773	0	protein_coding	1	237205505	238129359	RYR2
ENSG00000198633	3.652182994	3.935919592	protein_coding	19	52932440	52955568	ZNF534
ENSG00000198642	6.830742851	7.167295693	protein_coding	9	21329670	21335379	KLHL9
ENSG00000198646	10.39122196	10.26295417	protein_coding	20	33302578	33413452	NCOA6
ENSG00000198648	8.797280438	8.288162655	protein_coding	2	168810530	169104651	STK39
ENSG00000198650	0.869158192	0	protein_coding	16	71599563	71611033	TAT
ENSG00000198663	8.658089807	9.089837938	protein_coding	6	36839646	36892331	C6orf89
ENSG00000198668	11.91019599	11.65127045	protein_coding	14	90862846	90874605	CALM1
ENSG00000198673	1.16343121	2.039052734	protein_coding	12	62102040	62672931	FAM19A2
ENSG00000198677	9.811670266	9.928301652	protein_coding	5	94799599	94890711	TTC37
ENSG00000198680	6.386460534	6.862599506	protein_coding	9	25676396	25678856	TUSC1
ENSG00000198682	10.15697238	10.30611722	protein_coding	10	89419370	89507462	PAPSS2
ENSG00000198685	0.499066092	0.950786998	protein_coding	3	128290843	128294929	C3orf27
ENSG00000198689	8.066039442	8.166959047	protein_coding	X	135067598	135129423	SLC9A6
ENSG00000198690	7.491518048	7.619146302	protein_coding	15	31196055	31235311	FAN1
ENSG00000198691	0.869158192	1.16600992	protein_coding	1	94458391	94586688	ABCA4
ENSG00000198695	13.93068312	12.97265035	protein_coding	MT	14149	14673	MT-ND6

ENSG00000198700	10.46352509	10.55870647	protein_coding	1	201798269	201853422	IPO9
ENSG00000198707	7.888702948	8.101495266	protein_coding	12	88442794	88535993	CEP290
ENSG00000198711	1.16343121	1.667587519	protein_coding	1	54703710	54704736	C1orf191
ENSG00000198712	16.50549691	16.0178658	protein_coding	MT	7586	8269	MT-CO2
ENSG00000198715	7.717966186	8.81990641	protein_coding	1	156259880	156265463	C1orf85
ENSG00000198718	7.071412799	7.470237199	protein_coding	14	45431393	45543634	FAM179B
ENSG00000198719	1.799000381	1.518964905	protein_coding	6	170591294	170599561	DLL1
ENSG00000198720	9.022652927	8.560682282	protein_coding	17	27916801	27941779	ANKRD13B
ENSG00000198721	8.3480754	8.334763756	protein_coding	6	4115923	4135831	ECI2
ENSG00000198722	8.505663452	8.797898671	protein_coding	9	35161999	35405335	UNC13B
ENSG00000198723	2.10647801	3.532712221	protein_coding	19	7557862	7573336	C19orf45
ENSG00000198727	16.0895307	15.52778963	protein_coding	MT	14747	15887	MT-CYB
ENSG00000198728	8.865343505	9.076603748	protein_coding	10	103867317	103880210	LDB1
ENSG00000198729	6.328285199	3.845183986	protein_coding	6	150464212	150571493	PPP1R14C
ENSG00000198730	9.27062517	9.307964293	protein_coding	11	10772534	10801290	CTR9
ENSG00000198732	0.499066092	1.518964905	protein_coding	14	70320848	70499083	SMOC1
ENSG00000198734	0.499066092	0	protein_coding	1	169483404	169555826	F5
ENSG00000198736	9.007671769	9.152671242	protein_coding	16	1988211	1993327	SEPX1
ENSG00000198739	0	0.390640832	protein_coding	10	68685764	68859588	LRRTM3
ENSG00000198740	8.133414584	7.809160246	protein_coding	17	47366568	47439835	ZNF652
ENSG00000198742	9.984657349	9.159748241	protein_coding	7	98625061	98741723	SMURF1
ENSG00000198743	8.560389515	9.190788227	protein_coding	21	35445870	35478559	SLC5A3
ENSG00000198744	13.22885902	12.21604336	pseudogene	1	569756	570302	
ENSG00000198746	7.017207048	7.067139131	protein_coding	1	27216979	27226962	GPATCH3
ENSG00000198750	1.616589159	1.353254395	protein_coding	7	74807499	74867509	GATSL2
ENSG00000198752	11.32200903	10.33974134	protein_coding	14	103398716	103523799	CDC42BPB
ENSG00000198753	8.284478912	8.531880603	protein_coding	X	153029651	153044801	PLXNB3
ENSG00000198754	1.407729925	1.16600992	protein_coding	1	40235195	40237020	OXCT2
ENSG00000198755	11.47751492	11.97712059	protein_coding	6	35436185	35438562	RPL10A
ENSG00000198756	0.499066092	1.667587519	protein_coding	1	183898796	184006863	GLT25D2
ENSG00000198759	2.671945279	3.279072565	protein_coding	X	13587724	13651694	EGFL6
ENSG00000198763	15.35466093	14.76967403	protein_coding	MT	4470	5511	MT-ND2
ENSG00000198768	7.689364157	7.426571535	protein_coding	20	57034157	57090187	APCDD1L
ENSG00000198771	1.960915222	3.32456471	protein_coding	1	167599330	167675486	RCSD1
ENSG00000198774	2.928422289	3.279072565	protein_coding	12	86198331	86230348	RASSF9
ENSG00000198780	4.903563137	3.96493948	protein_coding	5	74073399	74162776	FAM169A
ENSG00000198783	6.738827102	7.04352744	protein_coding	17	33288549	33290205	ZNF830
ENSG00000198785	4.568725997	3.906303962	protein_coding	9	104331635	104500862	GRIN3A
ENSG00000198786	15.66788949	14.93234384	protein_coding	MT	12337	14148	MT-ND5
ENSG00000198788	3.004694206	1.16600992	protein_coding	11	1074875	1104419	MUC2
ENSG00000198791	9.465390183	9.340162149	protein_coding	8	17086737	17104387	CNOT7
ENSG00000198792	10.18848346	9.974519463	protein_coding	22	38615298	38669040	TMEM184B
ENSG00000198793	10.24608033	10.35077746	protein_coding	1	11166592	11322608	MTOR
ENSG00000198794	7.119373418	7.699043321	protein_coding	15	75249560	75313837	SCAMP5
ENSG00000198795	1.407729925	0.950786998	protein_coding	18	22641888	22932214	ZNF521
ENSG00000198796	5.207774174	7.295628905	protein_coding	18	56148479	56296189	ALPK2
ENSG00000198798	0	0.390640832	protein_coding	X	30248553	30255607	MAGEB3
ENSG00000198799	7.04456449	6.934050347	protein_coding	1	113615831	113669822	LRIG2
ENSG00000198804	16.88630592	16.32941805	protein_coding	MT	5904	7445	MT-CO1
ENSG00000198805	9.358631958	9.498022112	protein_coding	14	20937113	20945253	PNP
ENSG00000198807	6.598204782	5.824376633	protein_coding	14	37126773	37148920	PAX9
ENSG00000198814	6.35766608	7.126137778	protein_coding	X	30671476	30748725	GK
ENSG00000198815	9.373990307	9.083236018	protein_coding	1	42642210	42801548	FOXJ3
ENSG00000198816	9.502033728	10.0132375	protein_coding	19	7581004	7585912	ZNF358
ENSG00000198818	8.120617457	8.253299769	protein_coding	6	166733216	166756079	SFT2D1
ENSG00000198821	1.16343121	0.390640832	protein_coding	1	167399877	167487847	CD247
ENSG00000198824	8.656613027	8.486319932	protein_coding	13	115079988	115092796	CHAMP1
ENSG00000198825	7.789781675	7.869875863	protein_coding	10	121485609	121588652	INPP5F
ENSG00000198826	9.579671796	9.505448443	protein_coding	15	32907345	32932150	ARHGAP11A
ENSG00000198829	0	0.390640832	protein_coding	3	151591431	151599665	SUCNR1
ENSG00000198830	10.46394731	10.62781739	protein_coding	1	26798941	26802463	HMGN2
ENSG00000198832	10.32350339	12.0544657	protein_coding	22	31500758	31516055	
ENSG00000198833	9.349520666	9.577682064	protein_coding	6	90036344	90062567	UBE2J1

ENSG00000198835	3.78787645	3.96493948	protein_coding	1	228337553	228347527	GJC2
ENSG00000198836	10.27476096	9.975411057	protein_coding	3	193310933	193415612	OPA1
ENSG00000198837	9.846413258	10.11707283	protein_coding	1	153901977	153919172	DENND4B
ENSG00000198838	3.871730003	3.608232228	protein_coding	15	33603163	34158303	RYR3
ENSG00000198839	7.35977471	7.100213554	protein_coding	7	111846643	111983151	ZNF277
ENSG00000198840	13.37560171	12.54993132	protein_coding	MT	10059	10404	MT-ND3
ENSG00000198841	6.766474304	6.915587884	protein_coding	1	52497775	52499488	KTI12
ENSG00000198842	3.502389126	3.679994897	protein_coding	1	167063282	167098402	DUSP27
ENSG00000198843	9.024943975	9.102135695	protein_coding	3	150320662	150348222	
ENSG00000198844	1.16343121	0	protein_coding	17	8213559	8225834	ARHGEF15
ENSG00000198846	1.616589159	2.420525079	protein_coding	8	59717977	60031767	TOX
ENSG00000198853	9.342189974	9.252405772	protein_coding	9	35490124	35561895	RUSC2
ENSG00000198855	5.986594255	6.064512706	protein_coding	12	108908962	108919614	FICD
ENSG00000198856	8.872994239	9.506682457	protein_coding	4	109571740	109588976	OSTC
ENSG00000198857	2.57521082	2.721932731	pseudogene	1	120143963	120151873	HSD3BP5
ENSG00000198858	9.866992122	10.02964069	protein_coding	19	896503	913225	R3HDM4
ENSG00000198860	7.651313074	8.006993014	protein_coding	1	184020811	184043346	TSEN15
ENSG00000198862	8.727254249	8.803934152	protein_coding	21	30300466	30365277	LTN1
ENSG00000198863	7.543580762	7.225413142	protein_coding	17	41132582	41145707	RUNDC1
ENSG00000198868	1.407729925	0.697730409	pseudogene	5	134263720	134264016	MIR4461
ENSG00000198870	4.568725997	3.845183986	protein_coding	9	136243117	136271220	C9orf96
ENSG00000198873	8.103376106	8.006993014	protein_coding	10	120967101	121215131	GRK5
ENSG00000198874	7.751556534	7.332282157	protein_coding	7	66460160	66704501	TYW1
ENSG00000198876	8.829093615	8.736113084	protein_coding	9	34086522	34127397	DCAF12
ENSG00000198883	0	0.390640832	protein_coding	X	152157368	152162671	PNMA5
ENSG00000198885	0.499066092	0	protein_coding	2	96991069	96994091	ITPRIPL1
ENSG00000198886	17.32265143	16.48337997	protein_coding	MT	10760	12137	MT-ND4
ENSG00000198887	8.787867896	8.877321491	protein_coding	9	72873937	72969804	SMC5
ENSG00000198888	15.64131492	14.90807786	protein_coding	MT	3307	4262	MT-ND1
ENSG00000198890	6.585843317	6.544728831	protein_coding	1	107599267	107601916	PRMT6
ENSG00000198892	6.364918751	5.931169853	protein_coding	1	201857808	201861434	SHISA4
ENSG00000198894	8.817245731	8.804937616	protein_coding	14	77564440	77583630	KIAA1737
ENSG00000198898	9.680483837	9.605016392	protein_coding	7	116451124	116559315	CAPZA2
ENSG00000198899	15.73281317	14.74001075	protein_coding	MT	8527	9207	MT-ATP6
ENSG00000198900	10.72856959	10.20255683	protein_coding	20	39657458	39753127	TOP1
ENSG00000198901	10.04685659	9.990043357	protein_coding	15	91509270	91538859	PRC1
ENSG00000198908	5.707331524	4.17820932	protein_coding	X	101975616	102008468	BHLHB9
ENSG00000198909	8.894453552	9.223427792	protein_coding	17	61699801	61773667	MAP3K3
ENSG00000198910	8.997208309	12.23301123	protein_coding	X	153126969	153174677	L1CAM
ENSG00000198911	11.88393152	11.19813051	protein_coding	22	42229109	42303312	SREBF2
ENSG00000198912	8.221948786	7.998246367	protein_coding	1	3805689	3816857	C1orf174
ENSG00000198915	7.654275977	7.070480904	protein_coding	10	43689983	43762367	RASGEF1A
ENSG00000198917	8.817245731	8.740315472	protein_coding	9	131581930	131592100	C9orf114
ENSG00000198918	11.37953801	12.12626962	protein_coding	X	118920467	118925606	RPL39
ENSG00000198919	7.717966186	8.080102494	protein_coding	3	108308337	108413693	DZIP3
ENSG00000198920	8.53006357	9.198444985	protein_coding	17	6481646	6544247	KIAA0753
ENSG00000198924	7.609180529	7.973469823	protein_coding	10	115594488	115614142	DCLRE1A
ENSG00000198925	10.27235239	10.62753383	protein_coding	2	220074494	220094439	ATG9A
ENSG00000198929	2.928422289	1.802319292	protein_coding	1	162039564	162353321	NOS1AP
ENSG00000198931	10.27860633	10.36343361	protein_coding	16	88875747	88878352	APRT
ENSG00000198932	3.448790144	2.652276565	protein_coding	X	101906294	101914008	GPRASP1
ENSG00000198933	6.994006093	6.264499759	protein_coding	17	45772630	45789427	TBKBP1
ENSG00000198934	5.316509574	6.057793599	protein_coding	X	75648046	75651744	MAGEE1
ENSG00000198937	6.931877181	7.457530976	protein_coding	6	37450696	37467700	CCDC167
ENSG00000198938	16.35565374	15.32639774	protein_coding	MT	9207	9990	MT-CO3
ENSG00000198939	4.165528823	4.640685763	protein_coding	5	178322895	178360213	ZFP2
ENSG00000198944	0.499066092	0.697730409	protein_coding	5	132149033	132152488	ANKRD43
ENSG00000198945	5.270908378	4.489113623	protein_coding	6	130334844	130462594	L3MBTL3
ENSG00000198947	4.19837882	4.342020395	protein_coding	X	31132808	33357558	DMD
ENSG00000198948	5.018397273	6.563824016	protein_coding	4	170907748	170954182	MFAP3L
ENSG00000198951	9.332049062	9.315729406	protein_coding	22	42428724	42466846	NAGA
ENSG00000198952	11.02028398	11.08434249	protein_coding	1	156219015	156252616	SMG5
ENSG00000198954	8.515473076	8.219079902	protein_coding	10	70748487	70776738	KIAA1279

ENSG00000198959	12.89200086	11.79770842	protein_coding	20	36756863	36794980	TGM2
ENSG00000198960	8.068262669	8.495050596	protein_coding	X	100870110	100872991	ARMCX6
ENSG00000198961	9.747220804	9.624472234	protein_coding	5	108670410	108745695	PJA2
ENSG00000198964	8.739861387	8.076782959	protein_coding	10	52065346	52384795	SGMS1
ENSG00000198972	0	0.697730409	miRNA	19	52196039	52196117	MIRLET7E
ENSG00000198975	1.407729925	0.950786998	miRNA	11	122017229	122017301	MIRLET7A2
ENSG00000198976	1.16343121	1.16600992	miRNA	1	1104385	1104467	MIR429
ENSG00000198981	0.499066092	0	miRNA	7	130561506	130561570	MIR29A
ENSG00000199004	2.35979773	1.16600992	miRNA	17	57918627	57918698	MIR21
ENSG00000199023	0	0.390640832	miRNA	7	1062569	1062662	MIR339
ENSG00000199024	0	0.697730409	miRNA	20	3898141	3898218	MIR103A2
ENSG00000199032	0	0.390640832	miRNA	3	49057581	49057667	MIR425
ENSG00000199036	0	0.697730409	miRNA	6	33175612	33175721	MIR219-1
ENSG00000199053	4.689181911	5.125003832	miRNA	17	7126616	7126698	MIR324
ENSG00000199058	0	0.390640832	miRNA	4	113569641	113569713	MIR302B
ENSG00000199071	0.499066092	0	miRNA	17	28444097	28444190	MIR423
ENSG00000199072	0.869158192	0.697730409	miRNA	9	96938629	96938715	MIRLET7F1
ENSG00000199090	0	0.390640832	miRNA	11	75046136	75046230	MIR326
ENSG00000199097	0.499066092	0	miRNA	X	133680644	133680741	MIR424
ENSG00000199102	0	0.390640832	miRNA	4	113569519	113569586	MIR302C
ENSG00000199130	0	0.390640832	miRNA	16	14403142	14403228	MIR365A
ENSG00000199133	2.847891871	1.353254395	miRNA	9	96941116	96941202	MIRLET7D
ENSG00000199165	0.499066092	0.390640832	miRNA	9	96938239	96938318	MIRLET7A1
ENSG00000199169	0.499066092	0	miRNA	4	113569030	113569097	MIR367
ENSG00000199172	0.499066092	0	miRNA	12	95702196	95702289	MIR331
ENSG00000199179	0	0.697730409	miRNA	12	62997466	62997550	MIRLET7I
ENSG00000199200	0	0.390640832	misc_RNA	10	34490908	34491002	
ENSG00000199217	0.499066092	0	snRNA	11	119527021	119527126	
ENSG00000199226	0.869158192	0.390640832	snRNA	X	46377201	46377303	RNU6-50
ENSG00000199237	0	0.697730409	snRNA	12	49986887	49986996	
ENSG00000199266	0.499066092	1.667587519	snoRNA	20	37078013	37078146	SNORA60
ENSG00000199273	0.499066092	1.16600992	misc_RNA	7	40167720	40167831	
ENSG00000199293	1.960915222	3.232099092	snoRNA	17	37009116	37009247	SNORA21
ENSG00000199298	0	0.390640832	snoRNA	17	19093343	19093558	SNORD3C
ENSG00000199301	0	0.390640832	snRNA	16	71699994	71700100	
ENSG00000199306	0	0.390640832	snRNA	3	197621378	197621484	
ENSG00000199313	1.16343121	0	snRNA	9	127649689	127649828	
ENSG00000199325	1.616589159	0	snRNA	11	66382435	66382561	
ENSG00000199327	0.499066092	0	snRNA	3	124837941	124838044	
ENSG00000199331	0.499066092	0.950786998	misc_RNA	9	111804666	111804767	
ENSG00000199349	0	0.697730409	misc_RNA	1	206921325	206921437	
ENSG00000199366	1.16343121	0	misc_RNA	18	21161980	21162085	
ENSG00000199377	0.499066092	0	snRNA	1	45187458	45187574	RNU5F-1
ENSG00000199426	0.499066092	0.390640832	snRNA	17	56743898	56744058	
ENSG00000199438	0.499066092	0	rRNA	14	68123822	68123928	
ENSG00000199444	0	0.390640832	misc_RNA	19	12199060	12199161	
ENSG00000199466	0.499066092	0.950786998	misc_RNA	10	126454765	126454866	
ENSG00000199470	0	0.390640832	snoRNA	7	12740383	12740514	
ENSG00000199471	0.499066092	0.390640832	misc_RNA	1	202884008	202884108	
ENSG00000199472	0.499066092	0	misc_RNA	14	74373019	74373130	
ENSG00000199476	0.499066092	1.518964905	misc_RNA	3	48330077	48330184	
ENSG00000199488	1.616589159	0	snRNA	3	170712659	170712822	
ENSG00000199509	0.499066092	0	rRNA	20	21119597	21119715	
ENSG00000199545	1.16343121	1.353254395	rRNA	5	138635541	138635657	RN5S195
ENSG00000199550	0.499066092	0.950786998	misc_RNA	11	2393868	2393980	
ENSG00000199568	1.407729925	1.518964905	snRNA	15	65588389	65588504	RNU5A-1
ENSG00000199591	0.499066092	0	misc_RNA	3	47973298	47973404	
ENSG00000199630	0	0.697730409	misc_RNA	6	90551048	90551143	
ENSG00000199638	0	0.390640832	rRNA	10	70221164	70221282	
ENSG00000199646	0.499066092	0	snRNA	7	138561105	138561211	
ENSG00000199649	1.799000381	1.667587519	snoRNA	12	57037464	57037534	SNORD59B
ENSG00000199667	0.499066092	0	misc_RNA	8	101197366	101197467	
ENSG00000199668	0	0.390640832	misc_RNA	16	11408924	11409035	

ENSG00000199676	0	0.390640832	misc_RNA	20	34441174	34441286	
ENSG00000199683	0.869158192	0.950786998	misc_RNA	20	36603558	36603883	
ENSG00000199691	1.16343121	1.353254395	misc_RNA	20	37390171	37390485	
ENSG00000199695	0.499066092	0	snRNA	22	31618731	31618839	
ENSG00000199705	0	0.390640832	misc_RNA	X	122990912	122991013	
ENSG00000199709	0.499066092	0.390640832	snRNA	11	122797742	122797881	
ENSG00000199715	0	0.390640832	misc_RNA	6	18273396	18273497	
ENSG00000199716	0	0.390640832	misc_RNA	14	23337027	23337126	
ENSG00000199719	0.499066092	0.697730409	misc_RNA	20	18275787	18276095	
ENSG00000199730	0.499066092	0	misc_RNA	15	58996051	58996353	
ENSG00000199731	0	0.697730409	snRNA	5	32234872	32234981	
ENSG00000199740	0	0.390640832	misc_RNA	12	51333710	51333809	
ENSG00000199751	0.499066092	0.390640832	misc_RNA	X	70710916	70711017	
ENSG00000199753	3.950976309	4.841813558	snoRNA	17	62223443	62223512	SNORD104
ENSG00000199787	0	0.697730409	snoRNA	16	30430946	30431080	
ENSG00000199789	0.499066092	0	snRNA	14	99910643	99910749	
ENSG00000199790	0.499066092	0	snRNA	4	41086624	41086730	
ENSG00000199804	1.16343121	1.667587519	rRNA	14	24627812	24627931	
ENSG00000199806	0	0.390640832	rRNA	21	38224211	38224328	
ENSG00000199824	0.499066092	0	snRNA	12	51556684	51556790	
ENSG00000199837	0.499066092	0	rRNA	1	182913500	182913625	RN5S71
ENSG00000199846	1.960915222	0.950786998	snRNA	7	129124345	129124497	
ENSG00000199866	0	0.697730409	misc_RNA	2	173422879	173422980	
ENSG00000199872	0	0.390640832	snRNA	2	26265544	26265650	
ENSG00000199874	0	0.390640832	rRNA	18	21750469	21750587	
ENSG00000199879	0.869158192	0	snRNA	1	147735751	147735907	
ENSG00000199890	0	0.697730409	misc_RNA	1	111989420	111989530	
ENSG00000199906	0	0.390640832	snRNA	3	40540382	40540494	RNU5B-2P
ENSG00000199916	5.072559152	5.125003832	misc_RNA	9	35657751	35658014	RMRP
ENSG00000199933	1.16343121	0.950786998	misc_RNA	12	102113586	102113696	
ENSG00000199961	0.869158192	1.518964905	snoRNA	17	74557191	74557274	SNORD1B
ENSG00000199975	0	0.390640832	misc_RNA	11	73543427	73543735	
ENSG00000199980	0	0.697730409	misc_RNA	14	35402650	35402749	
ENSG00000199990	0.499066092	0	misc_RNA	5	140090860	140090958	VTRNA1-1
ENSG00000200013	0.499066092	0	snRNA	17	58739694	58739798	
ENSG00000200033	0.869158192	0.390640832	snRNA	1	222010676	222010779	
ENSG00000200051	0.499066092	0	snoRNA	5	139906937	139907013	
ENSG00000200059	0.499066092	0	misc_RNA	16	2181852	2181951	
ENSG00000200063	0.499066092	0.390640832	snoRNA	17	76396016	76396144	
ENSG00000200066	0	0.697730409	misc_RNA	12	133664099	133664201	
ENSG00000200087	6.068837212	5.776071827	snoRNA	1	28835071	28835274	SNORA73B
ENSG00000200090	2.57521082	2.242360793	misc_RNA	11	47748446	47748544	
ENSG00000200095	0.869158192	0	snRNA	15	101100891	101100997	
ENSG00000200114	0.499066092	1.16600992	rRNA	3	12552594	12552712	RN5S123
ENSG00000200120	0	0.390640832	misc_RNA	15	49429959	49430060	
ENSG00000200127	1.407729925	0.390640832	misc_RNA	17	41149933	41150024	
ENSG00000200142	0	0.390640832	misc_RNA	15	91520438	91520535	
ENSG00000200152	0	0.697730409	snRNA	11	74679234	74679337	
ENSG00000200164	0.499066092	0.697730409	misc_RNA	16	69225026	69225126	
ENSG00000200169	1.407729925	0.697730409	snRNA	1	45196727	45196842	RNU5D-1
ENSG00000200170	0.499066092	0.697730409	misc_RNA	10	73980510	73980610	
ENSG00000200179	0	0.390640832	misc_RNA	19	36694249	36694361	
ENSG00000200181	0.499066092	0	snoRNA	1	31441011	31441083	SNORD85
ENSG00000200182	3.077135474	4.342020395	misc_RNA	3	169482398	169482835	TERC
ENSG00000200225	0	0.390640832	rRNA	14	20883146	20883257	
ENSG00000200237	0.499066092	0.390640832	snoRNA	19	9930630	9930770	
ENSG00000200241	0	0.697730409	misc_RNA	2	86387079	86387189	
ENSG00000200242	0.869158192	0.697730409	misc_RNA	11	118865270	118865365	RNY4P12
ENSG00000200253	0	0.390640832	snRNA	10	88800995	88801098	
ENSG00000200257	0.499066092	0	snRNA	17	74748994	74749097	
ENSG00000200261	0.499066092	0	misc_RNA	9	114931566	114931677	
ENSG00000200274	0.499066092	0.697730409	snRNA	12	110005780	110005920	
ENSG00000200285	0	0.390640832	snRNA	10	12158279	12158378	

ENSG00000200301	0.499066092	0	rRNA	20	33783109	33783226	
ENSG00000200309	0	0.390640832	misc_RNA	12	49526636	49526730	
ENSG00000200332	0	0.697730409	misc_RNA	22	42044093	42044194	
ENSG00000200344	0	1.16600992	misc_RNA	1	8042941	8043043	
ENSG00000200345	0.499066092	0.390640832	snRNA	12	7271381	7271487	
ENSG00000200350	0.499066092	0.950786998	snRNA	4	155465085	155465189	
ENSG00000200354	1.616589159	1.353254395	snoRNA	20	37062508	37062641	SNORA71D
ENSG00000200376	0.499066092	0.390640832	snRNA	11	47598023	47598140	RNU5E-10P
ENSG00000200394	0.499066092	0	snoRNA	17	65736785	65736915	SNORA38B
ENSG00000200397	0.499066092	0.390640832	misc_RNA	7	86847927	86848028	
ENSG00000200403	0.869158192	1.353254395	snRNA	1	19631570	19631676	
ENSG00000200448	0.499066092	0.697730409	misc_RNA	22	43158866	43158965	
ENSG00000200483	0	0.390640832	snRNA	12	132299808	132299913	
ENSG00000200502	1.960915222	2.039052734	misc_RNA	9	37160134	37160234	
ENSG00000200506	0.499066092	0	misc_RNA	14	100049354	100049455	
ENSG00000200508	0	0.697730409	misc_RNA	1	64532312	64532413	
ENSG00000200528	0.869158192	0.390640832	snRNA	8	54827279	54827384	
ENSG00000200530	2.847891871	3.64455972	snoRNA	19	50000977	50001063	SNORD35B
ENSG00000200534	3.077135474	4.489113623	snoRNA	6	133138358	133138487	SNORA33
ENSG00000200555	0	0.390640832	snRNA	5	179871509	179871612	
ENSG00000200560	0.869158192	0	snRNA	17	61650603	61650706	
ENSG00000200591	0	0.390640832	misc_RNA	1	33105552	33105653	
ENSG00000200594	0	0.390640832	snRNA	6	155146310	155146413	
ENSG00000200646	0.499066092	0	misc_RNA	19	56827893	56828005	
ENSG00000200651	0	0.950786998	misc_RNA	17	75438191	75438295	
ENSG00000200652	0.499066092	0	snoRNA	16	28190419	28190544	
ENSG00000200665	0	0.390640832	snRNA	12	116520375	116520481	
ENSG00000200677	0.499066092	1.16600992	snoRNA	15	91298473	91298542	
ENSG00000200685	0.499066092	0	misc_RNA	11	13708097	13708205	
ENSG00000200688	0.499066092	0	misc_RNA	12	112506908	112507013	
ENSG00000200693	1.407729925	3.608232228	snoRNA	14	64118015	64118217	
ENSG00000200714	0.499066092	0.697730409	misc_RNA	8	66504966	66505055	
ENSG00000200732	0.499066092	0.390640832	snRNA	6	119648446	119648550	
ENSG00000200737	0.869158192	0	misc_RNA	10	99180952	99181064	
ENSG00000200742	1.16343121	0.390640832	misc_RNA	14	56118252	56118352	
ENSG00000200750	0.499066092	0	misc_RNA	18	19304080	19304190	
ENSG00000200759	0	0.390640832	snRNA	1	150995222	150995328	
ENSG00000200769	0.499066092	0.950786998	misc_RNA	7	91831557	91831648	
ENSG00000200783	0.499066092	0	misc_RNA	17	70659962	70660291	
ENSG00000200788	0.869158192	0.697730409	misc_RNA	9	130233154	130233266	
ENSG00000200792	0	0.390640832	snoRNA	21	33749496	33749631	SNORA80
ENSG00000200795	1.407729925	0.950786998	snRNA	12	120730900	120731040	RNU4-1
ENSG00000200814	0.499066092	1.16600992	snRNA	12	62727872	62727978	
ENSG00000200815	0.869158192	0.697730409	snRNA	7	50503078	50503184	
ENSG00000200816	0.869158192	0.697730409	snoRNA	6	31590856	31590987	SNORA38
ENSG00000200837	1.16343121	1.518964905	misc_RNA	3	47542573	47542672	
ENSG00000200843	0.499066092	0.390640832	misc_RNA	3	136307051	136307153	RNY4P4
ENSG00000200847	0	0.390640832	misc_RNA	7	23531411	23531512	
ENSG00000200879	2.928422289	4.363974406	snoRNA	11	122928785	122928869	SNORD14E
ENSG00000200882	0	0.390640832	snRNA	3	176843805	176843908	
ENSG00000200906	0.499066092	0	snRNA	X	77092786	77092892	
ENSG00000200913	2.238690726	3.453019579	snoRNA	1	45242162	45242265	SNORD46
ENSG00000200914	0	0.697730409	rRNA	17	6504264	6504371	
ENSG00000200917	0	0.390640832	snRNA	4	106328154	106328249	
ENSG00000200922	0	0.390640832	misc_RNA	9	74517371	74517466	
ENSG00000200924	0.869158192	0.950786998	snRNA	2	44119829	44119935	
ENSG00000200959	2.238690726	2.334191469	snoRNA	5	138614470	138614667	SNORA74A
ENSG00000200966	0.499066092	0.390640832	misc_RNA	9	103343361	103343661	
ENSG00000200976	0	0.390640832	misc_RNA	22	30154747	30154848	
ENSG00000200997	1.16343121	0	snRNA	17	56756888	56757051	
ENSG00000201003	0.499066092	0.697730409	snoRNA	1	54236691	54236826	
ENSG00000201012	0	0.390640832	misc_RNA	6	135795642	135795747	
ENSG00000201028	0	0.390640832	snRNA	1	112193053	112193159	

ENSG00000201035	0.499066092	0	rRNA	19	21295934	21296042	
ENSG00000201041	0	0.390640832	rRNA	7	128337493	128337610	RN5S242
ENSG00000201044	0.499066092	0	snRNA	2	231616856	231616962	
ENSG00000201085	0	0.390640832	snRNA	4	189042685	189042788	
ENSG00000201109	0	0.390640832	rRNA	7	129667117	129667237	RN5S245
ENSG00000201118	0	0.390640832	misc_RNA	9	19089591	19089686	
ENSG00000201121	0.499066092	0.697730409	misc_RNA	3	101380904	101381008	
ENSG00000201133	0.499066092	0	snoRNA	7	51003047	51003190	
ENSG00000201134	1.960915222	0.950786998	misc_RNA	1	151814212	151814313	
ENSG00000201151	0.499066092	1.16600992	snoRNA	20	33704939	33705010	
ENSG00000201164	0	0.697730409	snRNA	16	68569418	68569561	
ENSG00000201207	0	0.390640832	misc_RNA	6	31631065	31631178	
ENSG00000201208	0	0.697730409	misc_RNA	12	113616228	113616324	
ENSG00000201209	0	0.390640832	snoRNA	22	28628744	28628811	
ENSG00000201217	0.869158192	0	misc_RNA	3	152167061	152167173	
ENSG00000201264	0	2.144285137	snoRNA	4	152023209	152023280	
ENSG00000201298	0.499066092	0	snRNA	5	134036862	134036968	
ENSG00000201302	2.847891871	3.748356452	snoRNA	9	130210780	130210909	SNORA65
ENSG00000201321	0.499066092	0	rRNA	1	228763895	228764013	RN5S9
ENSG00000201358	0.869158192	0	misc_RNA	14	50535286	50535591	
ENSG00000201367	0.499066092	0	snRNA	6	15315151	15315257	
ENSG00000201377	0.499066092	0	misc_RNA	X	69616129	69616225	RNY4P23
ENSG00000201388	1.407729925	0.390640832	snoRNA	19	33099243	33099375	
ENSG00000201390	0	0.390640832	snRNA	10	21950564	21950670	
ENSG00000201448	0	0.390640832	snoRNA	1	36884051	36884179	
ENSG00000201451	0.499066092	0	misc_RNA	9	136204565	136204661	
ENSG00000201457	2.671945279	1.925536307	snoRNA	1	40033046	40033180	SNORA55
ENSG00000201458	0	0.390640832	snRNA	3	172514219	172514348	RNU4-4P
ENSG00000201469	0	0.390640832	rRNA	12	133300531	133300643	
ENSG00000201492	1.16343121	0.950786998	rRNA	1	229685652	229685769	RN5S78
ENSG00000201499	0.499066092	0.697730409	snRNA	2	201746438	201746544	
ENSG00000201510	0	0.390640832	misc_RNA	15	77028982	77029321	
ENSG00000201512	3.652182994	3.532712221	snoRNA	20	37058313	37058446	SNORA71C
ENSG00000201524	0	0.390640832	snRNA	17	57924799	57924905	
ENSG00000201529	0	0.390640832	misc_RNA	14	68084019	68084132	
ENSG00000201532	0.499066092	0	rRNA	5	138348018	138348130	RN5S194
ENSG00000201533	0	0.697730409	misc_RNA	4	140606281	140606581	
ENSG00000201544	0	0.390640832	snoRNA	1	212526158	212526292	SNORA16B
ENSG00000201555	1.16343121	1.518964905	misc_RNA	6	31746594	31746682	
ENSG00000201558	2.10647801	1.667587519	snRNA	1	145382757	145382920	
ENSG00000201563	0.869158192	0	misc_RNA	12	32858337	32858440	
ENSG00000201564	0.499066092	0.390640832	misc_RNA	11	9702471	9702785	
ENSG00000201573	0	0.390640832	misc_RNA	14	50935115	50935216	
ENSG00000201579	0.499066092	0.390640832	snRNA	12	56982302	56982405	
ENSG00000201581	0.869158192	0.950786998	misc_RNA	10	6191586	6191903	
ENSG00000201586	0	0.697730409	snRNA	11	17137199	17137310	
ENSG00000201592	0.499066092	0	snoRNA	X	20154424	20154503	
ENSG00000201595	0	0.390640832	rRNA	3	51728481	51728598	RN5S132
ENSG00000201616	0.499066092	0.697730409	snRNA	11	3069589	3069751	
ENSG00000201619	0.499066092	0	snoRNA	1	179170622	179170762	
ENSG00000201641	0.499066092	0	snRNA	4	78071481	78071590	
ENSG00000201643	0.499066092	0.950786998	snoRNA	7	75573100	75573234	SNORA14A
ENSG00000201644	0.499066092	0.390640832	misc_RNA	4	76587305	76587397	
ENSG00000201659	1.616589159	0.390640832	snRNA	X	46992070	46992225	RNU12-2P
ENSG00000201678	2.928422289	1.16600992	misc_RNA	12	49321722	49321816	
ENSG00000201684	0.499066092	1.16600992	misc_RNA	11	67129885	67130179	
ENSG00000201699	1.616589159	0.697730409	snRNA	1	144534038	144534199	
ENSG00000201724	0	0.390640832	misc_RNA	15	100221991	100222082	
ENSG00000201733	0	0.697730409	snoRNA	11	66200234	66200373	
ENSG00000201736	0.499066092	0	rRNA	4	40992171	40992290	RN5S160
ENSG00000201749	0.499066092	0.390640832	misc_RNA	9	117377735	117377848	
ENSG00000201761	0.499066092	0	snRNA	8	22291904	22292010	
ENSG00000201770	0.869158192	0	snRNA	20	30168892	30168995	

ENSG00000201772	2.928422289	1.925536307	snoRNA	7	45144505	45144641	SNORA5C
ENSG00000201784	3.077135474	4.508954154	snoRNA	11	17096201	17096291	SNORD14A
ENSG00000201785	1.799000381	0.950786998	snoRNA	6	31504151	31504226	SNORD117
ENSG00000201793	0	0.390640832	misc_RNA	13	99857962	99858260	RN7SKP9
ENSG00000201801	0	0.390640832	snRNA	1	11969865	11969984	RNU5E-4P
ENSG00000201806	0	0.390640832	snRNA	2	98281054	98281194	RNU4-8P
ENSG00000201812	0	0.697730409	rRNA	21	15443192	15443307	
ENSG00000201813	0	0.390640832	snRNA	2	196535096	196535198	
ENSG00000201820	0	0.390640832	misc_RNA	14	51720651	51720741	
ENSG00000201821	0.499066092	0	snRNA	13	21612668	21612806	RNU4-9P
ENSG00000201823	1.616589159	1.802319292	snoRNA	6	31803040	31803103	SNORD48
ENSG00000201830	0.869158192	0	misc_RNA	9	100677972	100678078	
ENSG00000201838	2.10647801	1.353254395	snoRNA	17	74557716	74557787	SNORD1A
ENSG00000201846	0	0.390640832	rRNA	4	149479099	149479200	RN5S166
ENSG00000201850	0.499066092	0	misc_RNA	16	68123352	68123446	
ENSG00000201852	0.499066092	0.390640832	snRNA	18	19760073	19760179	
ENSG00000201861	0	0.390640832	rRNA	10	327955	328065	
ENSG00000201876	0.499066092	0	rRNA	2	74195250	74195333	RN5S97
ENSG00000201882	1.16343121	0.390640832	snoRNA	X	20154184	20154253	
ENSG00000201900	0.499066092	0.697730409	misc_RNA	1	115270341	115270445	
ENSG00000201916	0	0.950786998	misc_RNA	22	20835445	20835555	
ENSG00000201944	0	0.390640832	snoRNA	1	205700349	205700480	
ENSG00000201988	0.869158192	0.697730409	misc_RNA	6	30704081	30704191	
ENSG00000201998	0.869158192	1.667587519	snoRNA	11	9450320	9450501	SNORA23
ENSG00000202000	0.499066092	0.390640832	snRNA	4	88921389	88921553	
ENSG00000202019	0	0.390640832	misc_RNA	22	31626057	31626158	
ENSG00000202030	0.499066092	0	misc_RNA	5	135416186	135416286	VTRNA2-1
ENSG00000202031	2.10647801	2.788380093	snoRNA	1	45243515	45243584	SNORD38A
ENSG00000202058	3.004694206	1.667587519	misc_RNA	22	42961054	42961336	
ENSG00000202059	0.499066092	0	snoRNA	2	203917008	203917141	
ENSG00000202071	0.499066092	0	misc_RNA	3	65989514	65989615	
ENSG00000202077	2.10647801	1.667587519	snRNA	17	56736510	56736666	
ENSG00000202078	1.16343121	0.950786998	misc_RNA	1	160295894	160296006	
ENSG00000202079	0.499066092	0.390640832	misc_RNA	1	247458137	247458243	
ENSG00000202081	0	0.390640832	snRNA	15	86194753	86194859	
ENSG00000202089	0	0.390640832	snRNA	11	63650059	63650157	
ENSG00000202093	3.274897671	3.845183986	snoRNA	18	47015615	47015678	SNORD58C
ENSG00000202141	0.499066092	0	misc_RNA	2	183887587	183887699	
ENSG00000202150	0.499066092	0.697730409	snRNA	20	33618120	33618223	
ENSG00000202159	0	0.390640832	snRNA	18	55422626	55422732	
ENSG00000202181	0.499066092	0.390640832	snRNA	3	196474063	196474195	
ENSG00000202198	3.950976309	4.15321211	misc_RNA	6	52860418	52860748	RN7SK
ENSG00000202220	0	0.697730409	misc_RNA	8	74892019	74892112	
ENSG00000202227	0	0.390640832	snRNA	2	48729061	48729163	
ENSG00000202255	1.16343121	0.697730409	misc_RNA	20	35296962	35297073	
ENSG00000202259	0.869158192	0	snRNA	1	100466193	100466295	
ENSG00000202268	0	0.390640832	snoRNA	3	45296902	45297112	
ENSG00000202290	1.407729925	1.16600992	rRNA	13	98015226	98015355	RN5S37
ENSG00000202297	0.869158192	0	misc_RNA	10	94094220	94094321	
ENSG00000202314	1.799000381	3.232099092	snoRNA	11	93464668	93464740	SNORD6
ENSG00000202318	0	0.390640832	misc_RNA	12	6343511	6343621	
ENSG00000202337	0.499066092	1.518964905	snRNA	14	32672369	32672475	
ENSG00000202343	0.869158192	1.353254395	snoRNA	6	149915761	149915895	
ENSG00000202347	1.407729925	0.390640832	snRNA	13	114133230	114133393	RNU1-16P
ENSG00000202357	0	0.390640832	misc_RNA	19	11519448	11519556	
ENSG00000202358	0.499066092	0	snRNA	4	56751761	56751867	
ENSG00000202361	0	0.390640832	misc_RNA	17	59951445	59951543	
ENSG00000202364	3.274897671	2.721932731	snoRNA	17	19091329	19091544	SNORD3A
ENSG00000202379	1.16343121	1.16600992	snoRNA	3	108293412	108293545	
ENSG00000202382	0.869158192	0.697730409	misc_RNA	2	85594708	85594809	
ENSG00000202385	0	0.390640832	misc_RNA	1	89485929	89486048	
ENSG00000202392	0	0.697730409	misc_RNA	4	6998739	6999055	
ENSG00000202400	0.499066092	0.390640832	snoRNA	2	232325082	232325151	SNORD82

ENSG00000202408	0.499066092	0.697730409	snRNA	1	147806736	147806886	
ENSG00000202414	0	0.390640832	misc_RNA	20	25195334	25195434	
ENSG00000202415	1.616589159	2.039052734	misc_RNA	1	10007376	10007694	
ENSG00000202431	0	0.697730409	snRNA	7	34337108	34337210	
ENSG00000202434	0.499066092	0.390640832	snoRNA	2	198269442	198269578	
ENSG00000202441	1.799000381	1.802319292	misc_RNA	6	33167378	33167472	RNY4P10
ENSG00000202445	0.499066092	0	snRNA	9	97270020	97270126	
ENSG00000202461	1.16343121	0.697730409	misc_RNA	1	12084069	12084181	
ENSG00000202469	0	0.390640832	misc_RNA	X	10029262	10029363	
ENSG00000202470	0	0.390640832	misc_RNA	12	95669546	95669648	
ENSG00000202474	0.499066092	0	rRNA	9	66458144	66458262	
ENSG00000202487	0	0.390640832	misc_RNA	10	128609376	128609472	
ENSG00000202490	0	0.390640832	snRNA	14	64084761	64084862	
ENSG00000202502	0.869158192	0.390640832	rRNA	3	182976088	182976204	RN5S151
ENSG00000202512	0	0.390640832	misc_RNA	5	109035201	109035535	
ENSG00000202514	0	0.390640832	misc_RNA	8	42804531	42804638	
ENSG00000202515	1.407729925	0.950786998	misc_RNA	5	140105743	140105831	VTRNA1-3
ENSG00000202532	4.026095388	3.532712221	snRNA	2	128602810	128602915	
ENSG00000202533	0	1.667587519	misc_RNA	5	131803839	131803949	
ENSG00000202538	0.869158192	0	snRNA	12	120729566	120729706	RNU4-2
ENSG00000202566	0	0.390640832	miRNA	X	73438212	73438296	MIR421
ENSG00000202601	0.499066092	0	miRNA	5	58999432	58999529	MIR582
ENSG00000203258	0.499066092	0.390640832	antisense	11	12942733	12944472	
ENSG00000203276	0.499066092	1.667587519	pseudogene	1	54498816	54500553	
ENSG00000203279	1.616589159	1.518964905	lincRNA	9	100000046	100000960	
ENSG00000203280	1.960915222	1.16600992	antisense	22	25498407	25508659	
ENSG00000203288	2.671945279	2.788380093	antisense	1	151763280	151766878	
ENSG00000203301	2.10647801	2.242360793	protein_coding	1	2143360	2145620	
ENSG00000203305	2.671945279	2.579085888	pseudogene	19	56208088	56208594	
ENSG00000203306	5.331395195	4.528525526	pseudogene	11	124958377	124960411	
ENSG00000203314	0.499066092	0	pseudogene	17	75099519	75099654	
ENSG00000203315	1.16343121	1.353254395	pseudogene	17	75087901	75088024	
ENSG00000203321	1.407729925	2.039052734	processed_transc	9	77567884	77611209	
ENSG00000203325	0.499066092	0	antisense	1	32517892	32539075	
ENSG00000203326	8.150302734	7.70765368	protein_coding	19	53868946	53889846	ZNF525
ENSG00000203327	1.407729925	2.039052734	antisense	2	55441523	55443262	
ENSG00000203335	0	0.390640832	antisense	7	153721094	153758033	
ENSG00000203341	0	0.390640832	sense_intronic	13	99970408	99971209	UBAC2-IT1
ENSG00000203356	0.499066092	0.390640832	processed_transc	1	51660767	51700768	
ENSG00000203362	2.57521082	3.027231696	antisense	6	43555967	43559099	
ENSG00000203364	0	0.950786998	lincRNA	9	95909322	95910838	
ENSG00000203377	0.499066092	0	pseudogene	8	131085393	131085648	
ENSG00000203392	4.437288965	3.411459265	protein_coding	15	75970672	75973093	
ENSG00000203396	0	0.390640832	pseudogene	9	107090607	107091470	
ENSG00000203397	0.499066092	0	pseudogene	X	73285918	73286427	
ENSG00000203400	1.16343121	0	pseudogene	11	64657298	64657758	
ENSG00000203401	2.35979773	1.16600992	pseudogene	16	67466595	67468931	
ENSG00000203403	1.960915222	2.334191469	pseudogene	19	35628549	35630484	
ENSG00000203417	1.407729925	1.925536307	lincRNA	10	131984445	131985130	
ENSG00000203421	0.869158192	0	pseudogene	12	50711462	50711558	
ENSG00000203423	0	0.390640832	pseudogene	12	50710006	50710100	
ENSG00000203424	0.499066092	0	pseudogene	12	50707798	50707894	
ENSG00000203435	0	0.697730409	pseudogene	2	196858983	196860404	
ENSG00000203436	0.499066092	0	pseudogene	4	95371802	95372043	
ENSG00000203437	0.869158192	0.697730409	pseudogene	12	31617996	31618776	
ENSG00000203438	0.499066092	0	pseudogene	9	66473375	66473673	
ENSG00000203441	2.10647801	1.353254395	antisense	13	100151981	100153306	LINC00449
ENSG00000203446	0.869158192	1.667587519	antisense	7	40577726	40586527	
ENSG00000203457	0	0.390640832	pseudogene	10	126493333	126493557	
ENSG00000203472	3.004694206	1.353254395	processed_transc	16	75509794	75529305	
ENSG00000203482	0.499066092	0.390640832	pseudogene	17	26547062	26547215	
ENSG00000203485	11.87618012	10.2986563	protein_coding	14	105155943	105185942	INF2
ENSG00000203497	3.077135474	3.993387124	antisense	10	112629626	112631991	

ENSG00000203499	6.604346021	4.342020395	processed_transc	8	144816310	144828507	
ENSG00000203504	0.499066092	0.390640832	pseudogene	11	31705636	31707478	
ENSG00000203506	0.499066092	0.697730409	antisense	3	29657466	29684300	RBMS3-AS2
ENSG00000203512	0	0.390640832	pseudogene	9	34195642	34195883	
ENSG00000203513	0.499066092	0	pseudogene	9	34169687	34169934	
ENSG00000203542	0	0.390640832	pseudogene	9	32548215	32548487	
ENSG00000203546	0.869158192	2.579085888	protein_coding	14	31803518	31926647	
ENSG00000203562	0.499066092	1.16600992	pseudogene	17	4890302	4890972	
ENSG00000203563	1.16343121	1.518964905	pseudogene	5	21493692	21494062	
ENSG00000203564	3.004694206	2.971122874	antisense	9	114680537	114681204	
ENSG00000203581	1.616589159	1.16600992	pseudogene	16	3265562	3266546	OR1F2P
ENSG00000203588	0.499066092	0.697730409	antisense	X	69383692	69385056	IGBP1-AS1
ENSG00000203589	0	1.518964905	pseudogene	1	24032291	24033424	
ENSG00000203593	0	0.697730409	antisense	12	2113832	2120558	
ENSG00000203602	1.16343121	0.390640832	pseudogene	13	28008286	28008752	
ENSG00000203606	1.960915222	1.353254395	pseudogene	22	30831531	30832231	
ENSG00000203614	0.499066092	0.697730409	pseudogene	13	21286570	21288285	
ENSG00000203615	0.499066092	0	pseudogene	4	56277189	56277313	
ENSG00000203616	0.499066092	0	pseudogene	21	15309314	15309646	RHOT1P2
ENSG00000203618	1.16343121	0	protein_coding	22	19710468	19712294	GP1BB
ENSG00000203630	2.35979773	2.721932731	protein_coding	20	1316228	1317555	
ENSG00000203632	1.16343121	1.802319292	pseudogene	3	186650337	186652182	
ENSG00000203635	0.869158192	0	lincRNA	2	1624282	1629191	
ENSG00000203643	0.499066092	0	antisense	2	11861745	11864348	
ENSG00000203644	5.995967334	5.717592784	pseudogene	3	129565893	129566800	
ENSG00000203645	0.499066092	0	lincRNA	3	177012230	177041206	LINC00501
ENSG00000203647	0	0.390640832	pseudogene	3	65994445	65994875	
ENSG00000203650	0.869158192	2.334191469	processed_transc	X	117973519	117991849	
ENSG00000203652	0	0.390640832	pseudogene	3	30030355	30031951	
ENSG00000203666	5.958104008	6.712401119	protein_coding	1	245133007	245290466	EFCAB2
ENSG00000203667	4.062237333	4.622587519	protein_coding	1	244998624	245008359	FAM36A
ENSG00000203668	8.271030151	8.713847451	protein_coding	1	241792155	241799232	CHML
ENSG00000203684	1.960915222	2.242360793	protein_coding	1	228351787	228353213	C1orf148
ENSG00000203685	0.499066092	0	protein_coding	1	226736501	226796915	C1orf95
ENSG00000203688	0.869158192	0.950786998	lincRNA	6	168080306	168084467	
ENSG00000203691	2.35979773	2.912743273	protein_coding	1	225600404	225602040	
ENSG00000203697	1.616589159	1.16600992	protein_coding	1	223711349	223853436	CAPN8
ENSG00000203705	6.984620223	7.240325909	protein_coding	1	212965170	212989968	TATDN3
ENSG00000203706	4.491309013	5.361622823	protein_coding	1	210404801	210407392	C1orf133
ENSG00000203709	1.960915222	2.242360793	protein_coding	1	207986905	208042495	C1orf132
ENSG00000203711	2.847891871	2.420525079	protein_coding	6	159290954	159331385	C6orf99
ENSG00000203721	1.616589159	1.353254395	protein_coding	1	200311672	200343482	C1orf98
ENSG00000203722	2.762599152	2.039052734	protein_coding	6	150238014	150244257	RAET1G
ENSG00000203724	2.762599152	4.448594745	protein_coding	1	197871777	197876497	C1orf53
ENSG00000203727	1.407729925	0.697730409	protein_coding	6	147830063	148058683	SAMD5
ENSG00000203730	0	0.390640832	protein_coding	1	182367252	182369751	TEDDM1
ENSG00000203734	0.499066092	1.16600992	protein_coding	6	139117063	139225207	ECT2L
ENSG00000203737	1.407729925	1.667587519	protein_coding	1	174417212	174418683	GPR52
ENSG00000203739	2.35979773	2.971122874	antisense	1	173386932	173430501	
ENSG00000203760	7.289098491	7.858322159	protein_coding	6	126661320	126670021	CENPW
ENSG00000203761	3.146112541	4.127774132	pseudogene	1	155581011	155720105	
ENSG00000203772	4.821592551	5.200150955	protein_coding	10	135234170	135382916	SPRN
ENSG00000203778	5.879259127	6.97385478	protein_coding	6	112408802	112423993	C6orf225
ENSG00000203780	4.352265886	4.566889334	protein_coding	10	127585108	127698161	FANK1
ENSG00000203791	5.948480845	6.668971382	protein_coding	10	126436721	126480446	METTL10
ENSG00000203797	3.78787645	5.112089758	protein_coding	6	110712974	110740732	DDO
ENSG00000203799	5.525114348	5.372493443	pseudogene	6	109612231	109676266	CCDC162P
ENSG00000203801	1.16343121	1.16600992	lincRNA	6	109072857	109091145	LINC00222
ENSG00000203804	2.762599152	3.133298822	protein_coding	1	150533480	150533969	C1orf138
ENSG00000203805	0	0.950786998	protein_coding	10	122216466	122349367	PPAPDC1A
ENSG00000203808	0.499066092	0	protein_coding	6	105585562	105617820	C6orf112
ENSG00000203813	3.830412367	4.468996429	protein_coding	6	27777842	27778314	HIST1H3H
ENSG00000203814	5.207774174	4.841813558	protein_coding	1	149754245	149783928	HIST2H2BF

ENSG00000203815	5.660945913	5.832272499	protein_coding		1	149675978	149676609	
ENSG00000203817	3.004694206	2.851901313	protein_coding		1	149438750	149459549	FAM72C
ENSG00000203818	2.762599152	2.788380093	protein_coding		1	149398877	149400542	HIST2H3PS2
ENSG00000203832	6.032862302	6.490875274	protein_coding		1	148250249	148347506	NBPF20
ENSG00000203836	2.928422289	3.232099092	protein_coding		1	147574428	147624601	NBPF24
ENSG00000203837	0.499066092	0.950786998	protein_coding		10	118187379	118237469	PNLIPRP3
ENSG00000203843	0.499066092	0.697730409	pseudogene		1	144612266	144612683	PFN1P2
ENSG00000203849	2.671945279	3.32456471	processed_transc		1	142620645	142714605	
ENSG00000203855	0.869158192	1.353254395	pseudogene		1	120106689	120114734	HSD3BP4
ENSG00000203865	3.211941663	3.781359661	protein_coding		1	116941622	116961197	ATP1A1OS
ENSG00000203871	1.16343121	2.501982735	protein_coding		6	88106840	88109467	C6orf164
ENSG00000203872	1.616589159	3.133298822	protein_coding		6	88054571	88075181	C6orf163
ENSG00000203875	10.24362336	10.78609522	processed_transc		6	86386725	86388451	SNHG5
ENSG00000203876	0.499066092	0	protein_coding		10	111701029	111713649	
ENSG00000203879	10.57794882	10.52439235	protein_coding	X		153665266	153671814	GDI1
ENSG00000203880	9.016909338	9.263397979	protein_coding		20	62887048	62926855	PCMTD2
ENSG00000203883	2.10647801	1.925536307	protein_coding		20	62679076	62680994	SOX18
ENSG00000203886	0	0.390640832	antisense		10	104592478	104594273	CYP17A1-AS1
ENSG00000203896	1.616589159	1.518964905	protein_coding		20	62366815	62370456	LIME1
ENSG00000203897	0.499066092	0.697730409	antisense		1	109399839	109401146	
ENSG00000203907	0	0.950786998	protein_coding		6	74078278	74104856	OOEP
ENSG00000203914	0.869158192	1.802319292	pseudogene		1	92100568	92109633	HSP90B3P
ENSG00000203930	0.869158192	1.353254395	protein_coding	X		139791932	139854839	
ENSG00000203942	0	0.950786998	protein_coding		10	99349450	99350690	C10orf62
ENSG00000203943	2.10647801	2.334191469	protein_coding		1	84764049	84855640	SAMD13
ENSG00000203945	1.407729925	1.16600992	protein_coding	X		135056007	135057190	
ENSG00000203950	8.98077946	9.224178134	protein_coding	X		134184962	134186226	FAM127B
ENSG00000203951	0.499066092	0.390640832	protein_coding		20	60806978	60811355	
ENSG00000203952	0.499066092	0	protein_coding	X		133371077	133380237	CCDC160
ENSG00000203965	5.191549282	5.683080141	protein_coding		1	63989043	64038364	EFCAB7
ENSG00000203987	0.499066092	0	protein_coding		9	140762377	140787022	
ENSG00000203993	7.115078652	7.298481685	protein_coding		9	140509784	140513358	C9orf37
ENSG00000203995	7.144876945	7.357122767	protein_coding		1	53308183	53360670	ZYG11A
ENSG00000203999	4.381167248	3.64455972	processed_transc		20	48909257	48931459	
ENSG00000204003	0.499066092	0	protein_coding		9	139632619	139642980	LCN6
ENSG00000204010	0	0.390640832	protein_coding		10	91137813	91144962	IFIT1B
ENSG00000204011	2.847891871	2.039052734	protein_coding		9	137541273	137544656	C9orf104
ENSG00000204018	3.989024711	3.96493948	protein_coding		1	48231308	48462567	
ENSG00000204019	6.462804234	7.423961341	protein_coding	X		115592849	115594164	CXorf61
ENSG00000204022	0	0.697730409	protein_coding		10	90346510	90366733	LIPJ
ENSG00000204044	2.928422289	2.721932731	antisense		20	44642141	44650712	
ENSG00000204049	0.499066092	1.353254395	protein_coding		10	79626633	79629368	
ENSG00000204052	3.211941663	3.453019579	protein_coding		6	43474707	43478424	LRRC73
ENSG00000204054	8.162840364	8.273738696	processed_transc		9	132245730	132275947	
ENSG00000204055	2.57521082	2.652276565	antisense		9	131939050	131972827	
ENSG00000204060	4.131913373	4.17820932	protein_coding		1	41827594	41849262	FOXO6
ENSG00000204070	8.771587845	8.76011133	protein_coding		20	43990577	44005438	SYS1
ENSG00000204071	0	0.390640832	protein_coding	X		101395448	101397942	TCEAL6
ENSG00000204084	7.08025234	6.99159092	protein_coding		1	38326369	38412729	INPP5B
ENSG00000204086	1.616589159	1.353254395	protein_coding	X		96138907	96140446	RPA4
ENSG00000204091	0	0.390640832	antisense		6	40302693	40347624	TDRG1
ENSG00000204092	0	0.390640832	lincRNA		6	40312083	40313890	
ENSG00000204099	3.077135474	3.183544561	protein_coding		2	242749920	242758739	NEU4
ENSG00000204104	7.962273293	7.648510984	protein_coding		2	239229082	239309541	TRAF3IP1
ENSG00000204113	0.869158192	0.697730409	pseudogene	X		73405764	73406990	BMP2KL
ENSG00000204116	5.869093502	6.16171561	protein_coding	X		72783044	72906608	CHIC1
ENSG00000204118	0	0.390640832	protein_coding	X		72345876	72347919	NAP1L6
ENSG00000204120	9.887911038	9.538401832	protein_coding		2	233562009	233725285	GIGYF2
ENSG00000204128	0	0.697730409	protein_coding		2	231902205	231914434	C2orf72
ENSG00000204130	7.348842929	7.436965343	protein_coding		10	70100864	70167051	RUFY2
ENSG00000204131	1.616589159	1.353254395	protein_coding	X		71130938	71363424	NHSL2
ENSG00000204136	2.762599152	2.579085888	pseudogene		9	124207269	124262306	GGTA1P
ENSG00000204138	9.533752964	9.537798375	protein_coding		1	28696114	28826881	PHACTR4

ENSG00000204147	4.322773689	3.64455972	protein_coding	10	52499078	52576255	ASAH2B
ENSG00000204149	6.17992145	6.044260771	protein_coding	10	51748078	51770259	AGAP6
ENSG00000204150	1.16343121	0.697730409	pseudogene	10	47708307	47730235	CTGLF11P
ENSG00000204152	1.799000381	0.697730409	protein_coding	10	51371390	51732941	TIMM23B
ENSG00000204160	8.13977083	7.992972811	protein_coding	1	27153201	27183391	ZDHHC18
ENSG00000204164	2.57521082	1.802319292	processed_transc	10	48901102	48950972	
ENSG00000204165	0.499066092	0	protein_coding	X	70323837	70326561	CXorf65
ENSG00000204172	6.782811835	6.223059835	protein_coding	10	47191844	47239738	AGAP10
ENSG00000204173	0	1.667587519	protein_coding	9	114365111	114393569	C9orf29
ENSG00000204174	3.335220907	2.144285137	protein_coding	10	47083534	47088320	PPYR1
ENSG00000204175	4.712111592	0.697730409	protein_coding	10	46994087	47005643	GPRIN2
ENSG00000204176	6.793601635	5.938503778	protein_coding	10	46952762	46971400	SYT15
ENSG00000204177	7.222786417	6.885547525	pseudogene	10	46737612	46762776	BMS1P1
ENSG00000204178	7.813830732	7.887034762	protein_coding	1	25757388	25826700	TMEM57
ENSG00000204186	6.749949642	6.628734426	protein_coding	2	207139387	207179148	ZDBF2
ENSG00000204188	0.869158192	0.697730409	protein_coding	6	33551515	33556803	GGNBP1
ENSG00000204195	1.16343121	0	protein_coding	X	69454505	69460477	AWAT1
ENSG00000204209	9.820929282	10.21770649	protein_coding	6	33286335	33297046	DAXX
ENSG00000204217	9.382055486	9.18231859	protein_coding	2	203241659	203432474	BMPR2
ENSG00000204219	4.800350936	3.081239798	protein_coding	1	23707554	23751233	TCEA3
ENSG00000204220	8.441937187	8.581912372	protein_coding	6	33257079	33266178	PFDN6
ENSG00000204227	9.135091581	9.375714884	protein_coding	6	33176272	33180499	RING1
ENSG00000204228	5.929039784	6.130036786	protein_coding	6	33172419	33174608	HSD17B8
ENSG00000204231	9.099664364	9.636359306	protein_coding	6	33161365	33168630	RXRB
ENSG00000204237	6.727618145	7.182818183	protein_coding	17	79632067	79633618	C17orf90
ENSG00000204241	0.869158192	1.16600992	processed_transc	11	133902167	133916744	
ENSG00000204248	6.050961887	7.077141316	protein_coding	6	33130458	33160276	COL11A2
ENSG00000204253	2.10647801	1.802319292	pseudogene	2	190788062	190788942	
ENSG00000204256	11.54132321	11.33745188	protein_coding	6	32936437	32949282	BRD2
ENSG00000204257	6.798966424	6.703819132	protein_coding	6	32916390	32938493	HLA-DMA
ENSG00000204261	4.568725997	5.536128679	processed_transc	6	32811863	32814272	
ENSG00000204262	5.649112675	5.931169853	protein_coding	2	189896622	190044605	COL5A2
ENSG00000204264	9.572641559	10.27893096	protein_coding	6	32808494	32812480	PSMB8
ENSG00000204267	9.201414078	9.493672366	protein_coding	6	32781544	32806599	TAP2
ENSG00000204271	5.239686603	5.545764946	protein_coding	X	57002815	57021988	SPIN3
ENSG00000204272	8.096857104	8.299598884	processed_transc	X	56755692	56844813	
ENSG00000204282	2.928422289	0	protein_coding	17	76103480	76106416	
ENSG00000204287	2.471521042	0.697730409	protein_coding	6	32407619	32412823	HLA-DRA
ENSG00000204290	0.499066092	0.390640832	protein_coding	6	32359241	32379511	BTNL2
ENSG00000204291	4.863159938	5.32851069	protein_coding	9	101705461	101833069	COL15A1
ENSG00000204296	0	0.390640832	protein_coding	6	32256303	32352332	C6orf10
ENSG00000204301	2.471521042	3.027231696	protein_coding	6	32162620	32191844	NOTCH4
ENSG00000204304	8.839544001	9.021555505	protein_coding	6	32151966	32157963	PBX2
ENSG00000204305	5.207774174	5.32851069	protein_coding	6	32148745	32152101	AGER
ENSG00000204308	8.05038018	8.495050596	protein_coding	6	32146131	32151930	RNF5
ENSG00000204310	9.225519491	9.316433258	protein_coding	6	32135989	32145873	AGPAT1
ENSG00000204311	3.146112541	3.32456471	protein_coding	2	179316163	179326117	DFNB59
ENSG00000204314	5.485736881	5.683080141	protein_coding	6	32116136	32122150	PRRT1
ENSG00000204315	6.313367288	6.948652068	protein_coding	6	32096484	32098068	FKBPL
ENSG00000204316	9.57498878	9.919526433	protein_coding	17	73894724	73905899	MRPL38
ENSG00000204323	2.762599152	3.493416095	protein_coding	17	73629514	73637484	C17orf109
ENSG00000204334	2.238690726	3.081239798	protein_coding	2	171640296	171655481	
ENSG00000204335	4.75690578	5.072639111	protein_coding	2	171571861	171574588	SP5
ENSG00000204338	1.16343121	2.144285137	pseudogene	6	31973413	31976228	CYP21A1P
ENSG00000204343	1.16343121	0.390640832	protein_coding	9	97418353	97480105	C9orf118
ENSG00000204344	6.204385613	6.460630645	protein_coding	6	31938868	31949228	STK19
ENSG00000204345	0	0.390640832	protein_coding	17	72575504	72588422	CD300LD
ENSG00000204347	0	0.697730409	protein_coding	17	72352555	72357958	BTBD17
ENSG00000204348	7.72925009	8.220584723	protein_coding	6	31937587	31940069	DOM3Z
ENSG00000204351	10.61795765	10.81726149	protein_coding	6	31926857	31937532	SKIV2L
ENSG00000204356	9.511051014	9.905561226	protein_coding	6	31919864	31926887	RDBP
ENSG00000204361	0.499066092	1.802319292	protein_coding	11	114549108	114579362	FAM55B
ENSG00000204362	0.869158192	2.242360793	lincRNA	1	17516278	17524112	

ENSG00000204365	0	1.16600992	protein_coding	10	29135337	29170827	C10orf126
ENSG00000204366	6.825495146	6.559073862	protein_coding	6	31867384	31869769	ZBTB12
ENSG00000204370	8.727254249	8.595894166	protein_coding	11	111957497	111990353	SDHD
ENSG00000204371	9.892309656	9.857042169	protein_coding	6	31847536	31865464	EHMT2
ENSG00000204375	1.616589159	1.802319292	protein_coding	X	52541053	52546197	XAGE1E
ENSG00000204377	1.960915222	1.925536307	protein_coding	1	16555170	16556094	C1orf134
ENSG00000204379	1.407729925	1.667587519	protein_coding	X	52255219	52260363	XAGE1A
ENSG00000204380	1.960915222	2.242360793	antisense	2	159514849	159591514	
ENSG00000204382	0.499066092	2.501982735	protein_coding	X	52235228	52243954	XAGE1B
ENSG00000204385	0	1.16600992	protein_coding	6	31830969	31846823	SLC44A4
ENSG00000204386	9.225519491	10.18339326	protein_coding	6	31825436	31830683	NEU1
ENSG00000204387	9.829474056	10.41855964	protein_coding	6	31802385	31807541	C6orf48
ENSG00000204388	9.275444054	9.254610923	protein_coding	6	31795512	31798031	HSPA1B
ENSG00000204389	8.959377969	8.997179425	protein_coding	6	31783291	31785723	HSPA1A
ENSG00000204390	3.211941663	4.342020395	protein_coding	6	31777396	31783437	HSPA1L
ENSG00000204392	8.637276046	9.074940905	protein_coding	6	31765173	31774761	LSM2
ENSG00000204394	12.4757972	12.4054171	protein_coding	6	31745295	31763730	VARS
ENSG00000204396	4.165528823	3.133298822	protein_coding	6	31733367	31745108	VWA7
ENSG00000204397	4.543379137	5.536128679	protein_coding	11	104912053	104972158	CARD16
ENSG00000204406	7.049074049	6.827478203	protein_coding	2	148778580	149275805	MBD5
ENSG00000204410	6.372135143	6.174194902	protein_coding	6	31707725	31732622	MSH5
ENSG00000204420	1.16343121	0	protein_coding	6	31686371	31694491	C6orf25
ENSG00000204421	0.499066092	0.697730409	protein_coding	6	31686425	31689622	LY6G6C
ENSG00000204427	4.517578978	4.903073497	protein_coding	6	31654726	31671221	ABHD16A
ENSG00000204428	2.762599152	3.781359661	protein_coding	6	31644461	31651817	LY6G5C
ENSG00000204434	0.869158192	1.16600992	pseudogene	2	132349325	132384839	POTEKP
ENSG00000204435	9.946492051	10.36785414	protein_coding	6	31633013	31641323	CSNK2B
ENSG00000204438	7.381392865	8.055017786	protein_coding	6	31629006	31634060	GPANK1
ENSG00000204439	7.695130106	8.070120888	protein_coding	6	31626075	31628549	C6orf47
ENSG00000204444	3.871730003	3.279072565	protein_coding	6	31620193	31625987	APOM
ENSG00000204463	11.85170516	11.88582185	protein_coding	6	31606805	31620482	BAG6
ENSG00000204464	0	0.697730409	protein_coding	1	15490840	15497813	C1orf195
ENSG00000204469	12.94188174	12.59638432	protein_coding	6	31588450	31605548	PRRC2A
ENSG00000204472	0.869158192	0	protein_coding	6	31582961	31584798	AIF1
ENSG00000204475	0	0.390640832	protein_coding	6	31556672	31560762	NCR3
ENSG00000204482	4.568725997	4.319727133	protein_coding	6	31553901	31556686	LST1
ENSG00000204498	7.182306324	7.718344862	protein_coding	6	31514647	31527677	NFKBIL1
ENSG00000204511	0	1.353254395	protein_coding	6	31496494	31498009	MCCD1
ENSG00000204514	8.169068555	7.984140487	protein_coding	19	58380747	58400442	ZNF814
ENSG00000204516	8.579219424	8.17939326	protein_coding	6	31462658	31478901	MICB
ENSG00000204519	5.018397273	5.059245468	protein_coding	19	58193357	58201167	ZNF551
ENSG00000204520	8.112022537	8.292461872	protein_coding	6	31367561	31384016	MICA
ENSG00000204524	8.120617457	7.896480033	protein_coding	19	57751973	57774106	ZNF805
ENSG00000204525	11.33430035	12.4738155	protein_coding	6	31236526	31239907	HLA-C
ENSG00000204531	1.799000381	1.667587519	protein_coding	6	31130253	31148508	POU5F1
ENSG00000204532	0.499066092	0.390640832	protein_coding	19	56713670	56720821	ZSCAN5C
ENSG00000204533	3.652182994	3.493416095	protein_coding	19	56662314	56663250	
ENSG00000204536	8.614637248	8.899098923	protein_coding	6	31110216	31126015	CCHCR1
ENSG00000204538	1.616589159	2.652276565	protein_coding	6	31105313	31107127	PSORS1C2
ENSG00000204539	0	1.16600992	protein_coding	6	31082867	31088223	CDSN
ENSG00000204540	5.389447744	6.281901708	protein_coding	6	31082527	31107869	PSORS1C1
ENSG00000204544	0	0.390640832	protein_coding	6	30951485	30957680	MUC21
ENSG00000204556	0	1.353254395	pseudogene	20	25999468	26001320	
ENSG00000204560	9.707122248	9.627311386	protein_coding	6	30620896	30640814	DHX16
ENSG00000204564	7.451199747	7.621426466	protein_coding	6	30614816	30620987	C6orf136
ENSG00000204566	0.499066092	0	protein_coding	10	23492745	23539610	C10orf115
ENSG00000204568	9.658518116	9.598086496	protein_coding	6	30585486	30594172	MRPS18B
ENSG00000204569	10.17977063	9.798797703	protein_coding	6	30568177	30586389	PPP1R10
ENSG00000204572	0.869158192	2.501982735	protein_coding	11	71276609	71279980	KRTAP5-10
ENSG00000204574	11.15241645	11.429952	protein_coding	6	30539153	30564956	ABCF1
ENSG00000204576	8.448779323	8.295320915	protein_coding	6	30524663	30531500	PRR3
ENSG00000204577	1.960915222	2.420525079	protein_coding	19	54720147	54746602	LILRB3
ENSG00000204580	10.43710307	9.36078013	protein_coding	6	30844198	30867933	DDR1

ENSG00000204583	2.238690726	0.950786998	lincRNA	12	133179736	133187037	
ENSG00000204584	3.393122761	4.17820932	antisense	17	47923272	47926199	
ENSG00000204588	3.004694206	1.802319292	processed_transc	2	110744640	110753717	
ENSG00000204590	9.9349646	9.486810444	protein_coding	6	30509154	30525008	GNL1
ENSG00000204592	10.78169677	11.06017274	protein_coding	6	30457244	30461982	HLA-E
ENSG00000204595	0.499066092	0	protein_coding	19	54135310	54140263	DPRX
ENSG00000204599	7.04456449	7.216390925	protein_coding	6	30294256	30314587	TRIM39
ENSG00000204604	8.577659618	8.360927302	protein_coding	19	53341786	53360902	ZNF468
ENSG00000204610	1.407729925	0.950786998	protein_coding	6	30130983	30140473	TRIM15
ENSG00000204611	8.016241092	8.091661081	protein_coding	19	52617654	52643191	ZNF616
ENSG00000204613	0.499066092	1.353254395	protein_coding	6	30119722	30128711	TRIM10
ENSG00000204616	1.799000381	0.697730409	protein_coding	6	30070674	30080883	TRIM31
ENSG00000204618	4.491309013	4.29708397	protein_coding	6	30038043	30043664	RNF39
ENSG00000204619	9.454383134	9.497401522	protein_coding	6	30034486	30038110	PPP1R11
ENSG00000204620	4.437288965	3.608232228	protein_coding	X	48431482	48433275	
ENSG00000204622	2.35979773	2.501982735	pseudogene	6	29974360	29977733	HLA-J
ENSG00000204623	5.637181576	6.651226688	antisense	6	29968788	30029417	ZNRD1-AS1
ENSG00000204624	2.57521082	3.32456471	protein_coding	1	11539223	11597641	PTCHD2
ENSG00000204625	0	0.697730409	lincRNA	6	29942889	29946183	HCG9
ENSG00000204628	13.39753841	13.54572743	protein_coding	5	180663909	180675096	GNB2L1
ENSG00000204632	0.499066092	1.16600992	protein_coding	6	29794744	29798902	HLA-G
ENSG00000204634	8.269098624	7.534563804	protein_coding	2	101624079	101869328	TBC1D8
ENSG00000204637	0.499066092	0	pseudogene	2	101125739	101126077	
ENSG00000204642	7.565780433	9.073276144	protein_coding	6	29690552	29706305	HLA-F
ENSG00000204644	3.448790144	4.319727133	protein_coding	6	29640169	29648887	ZFP57
ENSG00000204650	6.456028446	6.327306117	pseudogene	17	43697976	43715325	
ENSG00000204653	3.744048221	3.411459265	protein_coding	19	51014858	51017947	ASPDH
ENSG00000204655	0	0.697730409	protein_coding	6	29624758	29640149	MOG
ENSG00000204657	2.10647801	3.279072565	protein_coding	6	29555683	29556745	OR2H2
ENSG00000204659	0.869158192	1.802319292	protein_coding	5	179105584	179107975	CBY3
ENSG00000204661	0.499066092	1.353254395	protein_coding	5	179068545	179072047	C5orf60
ENSG00000204673	10.22630637	10.08645176	protein_coding	19	50372295	50381716	AKT1S1
ENSG00000204681	8.620709081	8.771449152	protein_coding	6	29523406	29601753	GABBR1
ENSG00000204682	7.1740722	6.712401119	protein_coding	10	21781587	21786191	C10orf114
ENSG00000204683	0.499066092	0	protein_coding	10	21414692	21435488	C10orf113
ENSG00000204685	5.036678094	4.990346085	processed_transc	2	96874154	96908359	
ENSG00000204688	0.499066092	0.950786998	protein_coding	6	29424958	29432105	OR2H1
ENSG00000204706	0.869158192	0.950786998	antisense	9	72768050	72790804	
ENSG00000204709	1.16343121	0.950786998	protein_coding	6	28911654	28912314	C6orf100
ENSG00000204710	0	0.697730409	protein_coding	11	64937625	64940715	SPDYC
ENSG00000204711	0	0.390640832	protein_coding	9	72435709	72521148	C9orf135
ENSG00000204713	10.24362336	10.54945594	protein_coding	6	28870779	28891766	TRIM27
ENSG00000204718	0	0.697730409	pseudogene	13	19300846	19301554	CNN2P12
ENSG00000204745	4.464551814	3.845183986	pseudogene	2	87352513	87423770	
ENSG00000204758	3.989024711	4.250702764	lincRNA	5	172381789	172386395	
ENSG00000204764	6.236372259	6.690849666	protein_coding	5	170288874	170727019	RANBP17
ENSG00000204771	0.499066092	0.950786998	pseudogene	8	145462709	145485885	
ENSG00000204772	0.499066092	0	sense_intronic	3	41915024	41916531	ULK4-IT1
ENSG00000204778	2.10647801	2.242360793	pseudogene	9	70181520	70216621	
ENSG00000204779	0.499066092	0.390640832	protein_coding	9	70175707	70178815	FOXD4L5
ENSG00000204788	0	0.697730409	pseudogene	9	69377902	69380597	
ENSG00000204789	5.948480845	6.142791909	pseudogene	6	27325604	27339304	ZNF204P
ENSG00000204790	5.485736881	5.486957566	protein_coding	9	69204538	69272478	CBWD6
ENSG00000204791	2.10647801	2.039052734	antisense	8	145104030	145106423	
ENSG00000204792	1.960915222	3.748356452	lincRNA	2	75145275	75165545	
ENSG00000204794	2.57521082	1.925536307	pseudogene	9	68741234	68809906	PGM5P1
ENSG00000204802	2.928422289	2.144285137	processed_transc	9	46685429	46688137	
ENSG00000204804	0	0.950786998	pseudogene	9	45727513	45727852	FAM27D1
ENSG00000204805	1.407729925	0.697730409	pseudogene	9	44997777	44999352	FAM27E1
ENSG00000204807	1.407729925	1.667587519	protein_coding	9	45733559	45734896	FAM27E2
ENSG00000204813	0.499066092	0.950786998	pseudogene	9	46387012	46387222	
ENSG00000204814	0.499066092	0.950786998	protein_coding	9	44867571	44870528	
ENSG00000204815	3.871730003	3.368666104	protein_coding	17	40086898	40117648	

ENSG00000204818	0.499066092	0.697730409	pseudogene	9	44170447	44175727	
ENSG00000204822	3.744048221	4.250702764	protein_coding	2	74699085	74700449	MRPL53
ENSG00000204837	1.616589159	0.390640832	lincRNA	9	41961615	42019580	
ENSG00000204839	6.931877181	4.17820932	protein_coding	8	144648357	144655141	C8orf73
ENSG00000204842	9.158235462	9.117563863	protein_coding	12	111890018	112037480	ATXN2
ENSG00000204843	10.86109943	11.04751044	protein_coding	2	74588281	74619214	DCTN1
ENSG00000204851	0.499066092	0	protein_coding	19	46994453	46998722	PNMAL2
ENSG00000204852	7.858191159	8.622319665	protein_coding	12	111051832	111087235	TCTN1
ENSG00000204856	7.08903805	7.966311947	protein_coding	12	110906169	110928190	C12orf24
ENSG00000204859	7.194570056	7.182818183	protein_coding	1	6640061	6649340	ZBTB48
ENSG00000204860	4.491309013	4.94736961	protein_coding	9	38620471	38624987	FAM201A
ENSG00000204869	0.499066092	0.950786998	protein_coding	19	46543006	46544274	IGFL4
ENSG00000204876	2.57521082	3.570966319	protein_coding	7	155755326	155759037	
ENSG00000204880	1.16343121	0	protein_coding	17	39253233	39254393	KRTAP4-8
ENSG00000204894	2.471521042	2.788380093	pseudogene	7	152064256	152064345	
ENSG00000204899	7.850461307	7.122922611	protein_coding	13	73282495	73301825	MZT1
ENSG00000204904	0.869158192	1.667587519	protein_coding	X	46746852	46759138	CXorf31
ENSG00000204920	6.94645393	7.053694091	protein_coding	19	44488346	44502477	ZNF155
ENSG00000204922	6.328285199	7.057067116	protein_coding	11	62437745	62441159	C11orf83
ENSG00000204923	3.502389126	3.608232228	protein_coding	2	68689486	68694390	FBXO48
ENSG00000204929	0.869158192	1.518964905	processed_transc	2	65663864	66126649	
ENSG00000204930	0.869158192	0.950786998	protein_coding	9	35816388	35828744	C9orf128
ENSG00000204934	5.695874189	6.009862691	processed_transc	7	149564786	149577699	
ENSG00000204936	3.335220907	3.813624741	protein_coding	19	43857825	43867480	CD177
ENSG00000204941	3.78787645	3.608232228	protein_coding	19	43670408	43690688	PSG5
ENSG00000204946	6.749949642	6.699508919	protein_coding	7	148959262	148994393	ZNF783
ENSG00000204947	5.107569429	6.246885341	protein_coding	7	148799876	148823438	ZNF425
ENSG00000204949	4.689181911	3.679994897	antisense	8	124213412	124214983	
ENSG00000204950	1.799000381	1.802319292	protein_coding	11	61276272	61278482	LRRC10B
ENSG00000204952	1.16343121	0.950786998	protein_coding	17	37092685	37123655	FBXO47
ENSG00000204954	5.752272083	6.091080508	protein_coding	12	104343980	104359486	C12orf73
ENSG00000204956	3.911897206	3.081239798	protein_coding	5	140710252	140892546	PCDHGA1
ENSG00000204957	2.10647801	1.518964905	protein_coding	19	42746927	42749125	
ENSG00000204959	3.335220907	1.667587519	pseudogene	7	143969538	143991256	
ENSG00000204961	3.077135474	2.501982735	protein_coding	5	140227048	140391929	PCDHA9
ENSG00000204962	2.57521082	2.039052734	protein_coding	5	140220907	140391929	PCDHA8
ENSG00000204963	6.585843317	6.403479675	protein_coding	5	140213969	140391929	PCDHA7
ENSG00000204965	4.999881834	4.274079748	protein_coding	5	140201222	140391929	PCDHA5
ENSG00000204967	4.800350936	4.976162367	protein_coding	5	140186659	140391929	PCDHA4
ENSG00000204969	3.393122761	3.368666104	protein_coding	5	140174444	140391929	PCDHA2
ENSG00000204970	6.171673665	6.3217081	protein_coding	5	140165876	140391929	PCDHA1
ENSG00000204977	6.343050432	6.287656023	protein_coding	13	50570024	50594617	TRIM13
ENSG00000204983	0.869158192	0	protein_coding	7	142457319	142460923	PRSS1
ENSG00000204991	7.743231983	7.619146302	protein_coding	16	89884587	89937727	SPIRE2
ENSG00000205014	4.77879189	4.7279204	protein_coding	16	89284120	89302402	
ENSG00000205021	0	0.390640832	protein_coding	17	34623842	34625731	CCL3L1
ENSG00000205022	0.499066092	0	protein_coding	16	88928034	88933068	PABPN1L
ENSG00000205036	3.744048221	5.175534283	protein_coding	16	88620112	88636548	C16orf85
ENSG00000205037	1.616589159	1.802319292	processed_transc	16	88121647	88134591	
ENSG00000205041	1.960915222	3.027231696	protein_coding	19	40779396	40781386	
ENSG00000205045	0.499066092	0	protein_coding	17	33800708	33864880	SLFN12L
ENSG00000205047	6.509364082	6.037446454	protein_coding	16	87731749	87739290	
ENSG00000205056	1.799000381	1.667587519	processed_transc	12	92815307	92885703	CLLU1
ENSG00000205057	0.499066092	0.390640832	protein_coding	12	92813870	92821924	CLLU1OS
ENSG00000205060	8.857651982	8.453421159	protein_coding	7	133974084	134001803	SLC35B4
ENSG00000205076	0.499066092	0	protein_coding	19	39261608	39264157	LGALS7
ENSG00000205078	3.211941663	3.748356452	protein_coding	16	77233294	77247112	SYCE1L
ENSG00000205084	7.73205733	8.247406493	protein_coding	16	75572015	75590176	TMEM231
ENSG00000205085	3.004694206	1.925536307	protein_coding	7	128312342	128326929	FAM71F2
ENSG00000205086	0	0.390640832	protein_coding	2	42162508	42181406	
ENSG00000205089	2.35979773	1.802319292	protein_coding	5	132083137	132089856	CCNI2
ENSG00000205090	0.499066092	1.667587519	protein_coding	1	1470554	1475833	TMEM240
ENSG00000205100	1.616589159	0.950786998	pseudogene	4	190394119	190396346	HSP90AA4P

ENSG00000205105	0.499066092	0	pseudogene	13	47064997	47065397	COX17P1
ENSG00000205108	0.499066092	0.950786998	protein_coding	9	34723052	34729464	FAM205A
ENSG00000205116	1.407729925	1.353254395	protein_coding	1	1361508	1363167	TMEM88B
ENSG00000205129	2.847891871	4.841813558	protein_coding	4	186347394	186370980	C4orf47
ENSG00000205133	6.228441838	6.349483465	protein_coding	8	93895758	94029901	C8orf83
ENSG00000205138	6.298293508	6.651226688	protein_coding	19	36486090	36487220	SDHAF1
ENSG00000205143	1.616589159	1.16600992	protein_coding	9	34621376	34628104	ARID3C
ENSG00000205147	2.847891871	3.493416095	protein_coding	19	4041127	4043154	
ENSG00000205155	8.221948786	8.272288337	protein_coding	19	36236494	36237903	PSENEN
ENSG00000205177	1.960915222	1.353254395	protein_coding	11	33719807	33722347	C11orf91
ENSG00000205181	0	2.334191469	processed_transc	20	5480969	5485259	
ENSG00000205184	0	0.697730409	pseudogene	8	82546592	82547897	
ENSG00000205189	8.623735441	8.490068116	protein_coding	8	81397854	81438500	ZBTB10
ENSG00000205208	7.413224588	7.791043682	protein_coding	4	159587831	159593407	C4orf46
ENSG00000205209	1.407729925	1.925536307	protein_coding	19	35084347	35085490	SCGB2B2
ENSG00000205212	4.381167248	4.15321211	protein_coding	17	20739760	20799453	CCDC144NL
ENSG00000205213	9.712107084	9.726498497	protein_coding	11	27387508	27494322	LGR4
ENSG00000205217	1.616589159	1.925536307	protein_coding	17	20320741	20322363	FAM106B
ENSG00000205220	8.301587982	8.449576416	protein_coding	16	67968405	67970990	PSMB10
ENSG00000205221	0.499066092	1.518964905	protein_coding	2	36923833	37041935	VIT
ENSG00000205233	0	0.390640832	protein_coding	7	102312908	102319318	
ENSG00000205238	0.499066092	0.697730409	protein_coding	7	102191679	102202755	SPDYE2
ENSG00000205246	6.014532757	6.304782526	protein_coding	19	23945807	24010937	RPSAP58
ENSG00000205250	10.17771289	10.21619866	protein_coding	16	67226072	67232821	E2F4
ENSG00000205266	0.499066092	0	processed_transc	17	18319042	18328411	
ENSG00000205268	7.737655476	7.23139676	protein_coding	8	66629745	66754557	PDE7A
ENSG00000205269	4.437288965	5.339632724	protein_coding	6	11537938	11583757	TMEM170B
ENSG00000205277	1.799000381	1.353254395	protein_coding	7	100612904	100662230	MUC12
ENSG00000205293	0	1.353254395	lincRNA	8	58890917	58896685	
ENSG00000205300	3.393122761	3.279072565	protein_coding	20	4050799	4055812	
ENSG00000205302	8.903197794	9.005932523	protein_coding	5	122110691	122165803	SNX2
ENSG00000205309	6.681890601	6.97385478	protein_coding	17	17206649	17250977	NT5M
ENSG00000205318	0.869158192	1.16600992	protein_coding	6	10633993	10647501	GCNT6
ENSG00000205323	4.689181911	4.777836593	protein_coding	12	56146247	56211540	SARNP
ENSG00000205325	0.869158192	1.667587519	protein_coding	17	14670900	14683520	
ENSG00000205333	0	0.390640832	pseudogene	8	87669616	87672521	GOLGA2P1
ENSG00000205334	4.352265886	5.187895122	protein_coding	2	27928653	27938599	
ENSG00000205336	10.34795706	9.397831074	protein_coding	16	57644564	57698944	GPR56
ENSG00000205339	11.52987465	11.53225786	protein_coding	11	9406169	9469673	IPO7
ENSG00000205352	8.602416377	8.502492199	protein_coding	12	53835389	53840429	PRR13
ENSG00000205356	8.821205846	8.043160634	protein_coding	7	97843936	97881563	TECPR1
ENSG00000205358	0	0.390640832	protein_coding	16	56703726	56705041	MT1H
ENSG00000205361	0	0.697730409	pseudogene	16	56677617	56678698	MT1DP
ENSG00000205362	4.593635215	5.317302248	protein_coding	16	56672578	56673999	MT1A
ENSG00000205364	1.407729925	2.334191469	protein_coding	16	56666145	56667898	MT1M
ENSG00000205396	0.499066092	0	processed_transc	19	16126097	16138723	
ENSG00000205403	2.762599152	0.950786998	protein_coding	4	110661852	110723335	CFI
ENSG00000205412	0	0.950786998	pseudogene	3	147884131	147885085	HNRNPA1P20
ENSG00000205413	8.819887015	8.956216712	protein_coding	7	92728829	92747336	SAMD9
ENSG00000205414	0	0.390640832	antisense	16	50639987	50647595	
ENSG00000205423	6.984620223	7.176629214	protein_coding	16	50058321	50070999	TMEM188
ENSG00000205424	0	0.390640832	antisense	21	47247875	47256333	
ENSG00000205426	2.762599152	2.788380093	protein_coding	12	52679697	52685318	KRT81
ENSG00000205436	4.821592551	4.528525526	protein_coding	14	103566481	103576896	EXOC3L4
ENSG00000205464	3.989024711	5.125003832	protein_coding	5	81575281	81682796	ATP6AP1L
ENSG00000205476	9.333898174	8.032919217	protein_coding	14	99977603	100070363	CCDC85C
ENSG00000205482	1.16343121	0.697730409	pseudogene	7	76682095	76688757	
ENSG00000205485	5.785082014	5.516660875	pseudogene	7	76178677	76257299	
ENSG00000205488	0.499066092	0	antisense	10	5556207	5568209	
ENSG00000205500	2.35979773	0.697730409	processed_transc	2	27207644	27237480	
ENSG00000205502	1.407729925	2.788380093	protein_coding	15	62455734	62457482	C2CD4B
ENSG00000205517	7.423680977	6.445267191	protein_coding	19	11495017	11530018	RGL3
ENSG00000205531	10.43279577	10.40273907	protein_coding	11	2965661	3013607	NAP1L4

ENSG00000205534	2.57521082	2.788380093	pseudogene	16	29538889	29606395	
ENSG00000205537	0.499066092	1.353254395	antisense	12	48276432	48295308	
ENSG00000205542	12.10834152	12.9952996	protein_coding	X	12993227	12995346	TMSB4X
ENSG00000205544	6.710639711	7.766528853	protein_coding	17	7306294	7307456	C17orf61
ENSG00000205549	0	0.390640832	protein_coding	9	16203933	16276311	C9orf92
ENSG00000205559	2.671945279	3.64455972	antisense	22	51021455	51022306	
ENSG00000205560	4.437288965	5.112089758	protein_coding	22	51007290	51017899	CPT1B
ENSG00000205562	1.407729925	0.697730409	lincRNA	14	85991477	85996332	
ENSG00000205571	4.593635215	4.585695337	protein_coding	5	69345350	69374349	SMN2
ENSG00000205572	0.499066092	1.16600992	protein_coding	5	69321074	69338940	SERF1B
ENSG00000205578	1.407729925	0.697730409	pseudogene	7	72707497	72716496	POM121B
ENSG00000205579	0	0.950786998	pseudogene	14	81712638	81712907	DYNLL1P1
ENSG00000205581	10.09016702	10.29830006	protein_coding	21	40714241	40721573	HMGN1
ENSG00000205583	6.014532757	6.198834244	protein_coding	7	74988429	75024657	STAG3L1
ENSG00000205584	0	0.390640832	pseudogene	7	72440345	72442258	
ENSG00000205593	6.579622637	6.104183162	protein_coding	22	50750392	50765489	FAM116B
ENSG00000205609	0.499066092	0.950786998	protein_coding	16	28390900	28415200	EIF3CL
ENSG00000205629	8.103376106	8.360927302	protein_coding	16	25123047	25189552	LCMT1
ENSG00000205634	1.16343121	2.144285137	processed_transc	22	48016792	48027318	
ENSG00000205639	2.35979773	1.925536307	protein_coding	2	24232951	24286191	MFSD2B
ENSG00000205643	6.407685389	6.520498852	protein_coding	22	46639908	46646576	C22orf40
ENSG00000205644	0	1.16600992	pseudogene	5	64711180	64712049	
ENSG00000205659	7.066972635	7.057067116	protein_coding	14	74551499	74667936	LIN52
ENSG00000205664	6.482942724	6.866449614	protein_coding	X	3734598	3761934	
ENSG00000205669	1.16343121	0.697730409	protein_coding	14	74077649	74086592	ACOT6
ENSG00000205670	6.482942724	6.847095496	protein_coding	21	35747779	35780164	FAM165B
ENSG00000205673	0.869158192	0.697730409	lincRNA	21	35321230	35336260	
ENSG00000205678	0.499066092	0	protein_coding	4	65140975	65275186	TECRL
ENSG00000205683	5.331395195	5.726093467	protein_coding	14	73075742	73360809	DPF3
ENSG00000205702	3.274897671	3.87606762	polymorphic_pse	22	42536214	42540576	CYP2D7P1
ENSG00000205704	3.652182994	3.532712221	pseudogene	22	42348169	42354937	
ENSG00000205707	5.538004898	6.030599799	protein_coding	12	25348150	25362579	LYRM5
ENSG00000205710	3.554067925	3.368666104	protein_coding	17	4802713	4806227	C17orf107
ENSG00000205726	8.783815073	8.949880022	protein_coding	21	35014706	35272165	ITSN1
ENSG00000205730	10.67341097	10.63488845	protein_coding	16	19125254	19138618	ITPRIPL2
ENSG00000205740	0.869158192	1.16600992	protein_coding	10	1017097	1034281	
ENSG00000205744	6.428602508	4.15321211	protein_coding	19	6467219	6481798	DENND1C
ENSG00000205746	4.19837882	4.547834947	pseudogene	16	18428257	18488396	
ENSG00000205755	4.409500985	6.460630645	protein_coding	X	1314890	1331616	CRLF2
ENSG00000205758	6.704935538	7.087074634	protein_coding	21	34961647	35016232	CRYZL1
ENSG00000205763	5.331395195	5.42565083	pseudogene	7	32956427	32982788	RP9P
ENSG00000205765	10.10918645	10.08397376	protein_coding	5	41904290	41921738	C5orf51
ENSG00000205771	4.261916566	3.845183986	pseudogene	15	44019116	44039292	CATSPER2P1
ENSG00000205783	3.448790144	3.279072565	protein_coding	5	38819804	38821581	
ENSG00000205784	0.499066092	0	protein_coding	19	4890449	4902879	ARRDC5
ENSG00000205785	0.499066092	0.950786998	protein_coding	5	38820766	38823298	
ENSG00000205790	5.909333162	5.792353798	protein_coding	19	4679294	4685960	
ENSG00000205791	1.616589159	2.242360793	protein_coding	12	12508342	12510001	
ENSG00000205794	0	0.697730409	pseudogene	4	40044537	40058819	
ENSG00000205795	0	0.950786998	protein_coding	2	10196907	10221071	CYS1
ENSG00000205808	5.124761037	5.236305879	protein_coding	9	4662298	4665256	PPAPDC2
ENSG00000205809	0	0.390640832	protein_coding	12	10579453	10594899	KLRC2
ENSG00000205810	1.616589159	2.144285137	protein_coding	12	10564911	10573194	KLRC3
ENSG00000205821	3.274897671	3.081239798	protein_coding	17	2310279	2318731	
ENSG00000205832	0.499066092	1.353254395	protein_coding	16	4606491	4650715	C16orf96
ENSG00000205835	0.499066092	0	protein_coding	3	190570666	190610218	GMNC
ENSG00000205838	2.57521082	3.081239798	protein_coding	5	34838938	34899561	TTC23L
ENSG00000205847	0.499066092	0	pseudogene	2	71256158	71256736	
ENSG00000205853	1.960915222	2.334191469	protein_coding	22	32755893	32767063	RFPL3-AS1
ENSG00000205856	2.238690726	2.334191469	protein_coding	22	32544993	32555309	C22orf42
ENSG00000205861	0.869158192	0	antisense	13	24463028	24471402	C1QTNF9B-AS1
ENSG00000205863	3.004694206	3.133298822	protein_coding	13	24462240	24476794	C1QTNF9B
ENSG00000205869	1.616589159	1.16600992	protein_coding	11	1605572	1606513	KRTAP5-1

ENSG00000205871	1.407729925	1.802319292	pseudogene	15	43407897	43408691	RPS3AP47
ENSG00000205873	5.175139844	5.059245468	protein_coding	8	12294626	12424423	
ENSG00000205879	0.499066092	0.950786998	pseudogene	8	12029711	12036084	FAM90A2P
ENSG00000205885	5.795854912	5.446377228	antisense	12	7260648	7275097	
ENSG00000205890	1.616589159	0	protein_coding	16	3082482	3089131	
ENSG00000205898	0.499066092	1.802319292	pseudogene	7	124800028	124800601	
ENSG00000205903	8.280649185	7.830998152	pseudogene	7	6676953	6694242	
ENSG00000205913	3.698846687	4.17820932	protein_coding	16	2787104	2790805	
ENSG00000205918	1.16343121	2.144285137	pseudogene	16	2666122	2693297	
ENSG00000205922	0	0.390640832	protein_coding	19	1752372	1780987	ONECUT3
ENSG00000205929	0	0.697730409	protein_coding	21	34162985	34186053	C21orf62
ENSG00000205930	1.16343121	2.788380093	protein_coding	21	34144411	34266043	C21orf49
ENSG00000205937	10.44225492	10.85626414	protein_coding	16	2303117	2318413	RNPS1
ENSG00000205940	1.799000381	1.667587519	pseudogene	4	13338103	13339926	HSP90AB2P
ENSG00000205955	1.16343121	0.950786998	pseudogene	3	183833140	183835686	HSP90AA5P
ENSG00000205959	2.238690726	3.183544561	processed_transc	4	8483997	8514337	
ENSG00000205978	4.261916566	2.144285137	protein_coding	14	24867992	24888494	NYNRIN
ENSG00000205981	6.965663283	7.219404604	protein_coding	3	180701497	180707562	DNAJC19
ENSG00000206013	0	0.697730409	protein_coding	11	298200	299526	IFITM5
ENSG00000206028	0	0.390640832	processed_transc	22	27063656	27068617	
ENSG00000206043	0	0.390640832	protein_coding	18	71983110	72026422	C18orf63
ENSG00000206044	2.762599152	2.144285137	protein_coding	12	2958397	2966213	
ENSG00000206053	11.34831854	11.37948398	protein_coding	16	1728257	1752281	HN1L
ENSG00000206066	1.16343121	2.420525079	pseudogene	22	25714223	25716047	IGLL3P
ENSG00000206073	6.155035276	3.845183986	protein_coding	18	61304493	61311532	SERPINB4
ENSG00000206075	8.415986232	2.039052734	protein_coding	18	61143994	61172318	SERPINB5
ENSG00000206077	1.616589159	2.334191469	protein_coding	5	710475	767067	ZDHHC11B
ENSG00000206082	5.124761037	4.693656882	protein_coding	19	200112	202173	
ENSG00000206113	0	0.390640832	antisense	4	2420701	2430972	
ENSG00000206127	0	0.390640832	protein_coding	15	32737307	32747835	
ENSG00000206140	2.671945279	2.501982735	processed_transc	22	21820712	21825558	TMEM191C
ENSG00000206144	0.499066092	0	pseudogene	8	54449620	54452860	
ENSG00000206145	2.762599152	2.334191469	pseudogene	22	21389532	21399306	P2RX6P
ENSG00000206147	2.471521042	1.667587519	pseudogene	9	6639139	6639604	
ENSG00000206149	5.588445295	6.016808232	pseudogene	15	28834638	28930410	HERC2P9
ENSG00000206150	0	1.353254395	protein_coding	14	21500979	21502944	RNASE13
ENSG00000206168	0.499066092	0	pseudogene	16	281133	281471	
ENSG00000206176	0.499066092	0.390640832	lincRNA	22	20378814	20380440	
ENSG00000206181	1.16343121	0.390640832	protein_coding	18	44558943	44561988	TCEB3B
ENSG00000206190	2.471521042	0.390640832	protein_coding	15	25922420	26110317	ATP10A
ENSG00000206192	0	0.950786998	pseudogene	13	19390858	19446107	ANKRD20A9P
ENSG00000206195	7.093410923	8.254769334	pseudogene	22	16147979	16193004	
ENSG00000206199	0.499066092	0	protein_coding	3	149478892	149686172	ANKUB1
ENSG00000206228	0.869158192	0	pseudogene	8	83203859	83204614	HNRNPA1P4
ENSG00000206252	6.502803968	8.206984494	protein_coding	22	16157306	16157938	
ENSG00000206260	0	0.390640832	protein_coding	3	138722804	138725110	PRR23A
ENSG00000206262	4.19837882	4.794098771	protein_coding	3	138666076	138672293	C3orf72
ENSG00000206337	7.345180517	8.117738058	processed_transc	6	31368479	31445283	HCP5
ENSG00000206341	6.882196036	8.005247922	pseudogene	6	29855350	29858259	HLA-H
ENSG00000206344	2.35979773	3.279072565	protein_coding	6	31165537	31171745	HCG27
ENSG00000206384	1.960915222	2.144285137	protein_coding	3	130279178	130396999	COL6A6
ENSG00000206417	3.871730003	4.810179682	antisense	3	129034235	129045068	H1FX-AS1
ENSG00000206418	8.585441838	8.423679567	protein_coding	18	8609443	8639380	RAB12
ENSG00000206448	1.16343121	0.697730409	pseudogene	10	15196801	15197319	
ENSG00000206503	11.66160272	12.75789552	protein_coding	6	29909037	29913661	HLA-A
ENSG00000206527	9.648151859	9.871961511	protein_coding	3	123209667	123304032	PTPLB
ENSG00000206530	5.741167503	7.00911166	protein_coding	3	113005777	113160457	WDR52
ENSG00000206532	4.097495944	7.138927266	protein_coding	3	110607231	110612323	
ENSG00000206535	5.637181576	5.41517489	protein_coding	3	100120037	100175163	LNP1
ENSG00000206549	0.869158192	0.390640832	protein_coding	3	46753605	46854064	PRSS50
ENSG00000206557	0.499066092	0	protein_coding	3	32859510	32933771	TRIM71
ENSG00000206559	1.407729925	1.802319292	protein_coding	3	28390637	28579613	ZCWPW2
ENSG00000206560	9.529725156	9.323452948	protein_coding	3	15708743	15901278	ANKRD28

ENSG00000206561	2.35979773	3.906303962	protein_coding	3	15491640	15563258	COLQ
ENSG00000206562	6.652556929	6.568558581	protein_coding	3	15422782	15469047	METTL6
ENSG00000206567	4.19837882	4.640685763	processed_transc	3	10048102	10052893	
ENSG00000206573	6.496213887	6.716673044	processed_transc	3	9391373	9440263	
ENSG00000206582	0.869158192	0	misc_RNA	20	33114315	33114411	
ENSG00000206595	0	0.390640832	snRNA	1	51847980	51848086	
ENSG00000206597	3.744048221	4.990346085	snoRNA	11	62432894	62433042	SNORA57
ENSG00000206601	0.499066092	0.390640832	snRNA	4	109573306	109573412	
ENSG00000206602	1.960915222	2.652276565	snoRNA	18	47017653	47017717	SNORD58A
ENSG00000206603	1.16343121	1.667587519	snoRNA	7	56123058	56123195	
ENSG00000206604	0	0.390640832	snRNA	3	141864296	141864397	
ENSG00000206613	0	0.390640832	snRNA	4	169607759	169607867	
ENSG00000206622	0.869158192	1.925536307	snoRNA	X	118921316	118921447	SNORA69
ENSG00000206630	2.57521082	4.508954154	snoRNA	16	2205024	2205106	SNORD60
ENSG00000206633	2.671945279	0.390640832	snoRNA	2	10586840	10586975	SNORA80B
ENSG00000206634	1.16343121	0.390640832	snoRNA	7	65220513	65220646	SNORA22
ENSG00000206635	0	0.390640832	snRNA	1	151601800	151601906	
ENSG00000206645	0	0.390640832	misc_RNA	3	37134624	37134725	
ENSG00000206647	1.407729925	0.697730409	snoRNA	2	10295199	10295333	
ENSG00000206650	0	0.950786998	snoRNA	12	69021014	69021155	SNORA70G
ENSG00000206651	0	0.390640832	misc_RNA	1	156453890	156454002	
ENSG00000206652	0.499066092	0	snRNA	1	16840617	16840780	RNU1-1
ENSG00000206660	0.499066092	0	misc_RNA	10	32345674	32345767	
ENSG00000206663	0.499066092	0	misc_RNA	X	20003176	20003277	
ENSG00000206669	0	0.390640832	misc_RNA	19	37349129	37349231	
ENSG00000206675	0	0.390640832	snRNA	4	39299225	39299331	
ENSG00000206682	0.869158192	0.390640832	misc_RNA	5	31505791	31505895	
ENSG00000206688	0.499066092	0	snoRNA	15	25330532	25330623	SNORD116-18
ENSG00000206698	0	0.697730409	snRNA	5	96511654	96511817	
ENSG00000206712	0	0.950786998	snRNA	3	98523822	98523928	
ENSG00000206713	0	0.390640832	misc_RNA	17	73085957	73086069	
ENSG00000206715	0	0.390640832	snRNA	6	88198163	88198270	
ENSG00000206728	0.499066092	0.950786998	misc_RNA	3	23315298	23315401	
ENSG00000206731	0.869158192	0.390640832	snoRNA	2	27864910	27865036	
ENSG00000206739	0	0.390640832	misc_RNA	22	19032769	19032880	
ENSG00000206747	0.499066092	0	snRNA	X	67759285	67759391	
ENSG00000206751	0.499066092	0	misc_RNA	14	73319932	73320039	
ENSG00000206754	2.671945279	3.570966319	snoRNA	6	133136446	133136518	SNORD101
ENSG00000206755	0	0.950786998	snoRNA	16	30721858	30721986	SNORA30
ENSG00000206762	0.499066092	1.16600992	snRNA	1	205564169	205564275	
ENSG00000206763	0.499066092	0	snRNA	7	92331022	92331128	
ENSG00000206768	0	0.390640832	misc_RNA	14	69610569	69610670	
ENSG00000206775	1.616589159	2.334191469	snoRNA	19	3982505	3982570	SNORD37
ENSG00000206781	0.499066092	0	misc_RNA	15	64737329	64737442	
ENSG00000206790	0.499066092	0.390640832	misc_RNA	12	100651466	100651568	
ENSG00000206813	0	0.390640832	misc_RNA	22	42143316	42143417	
ENSG00000206815	0	0.390640832	snRNA	3	57534565	57534672	
ENSG00000206816	0.869158192	0.697730409	misc_RNA	11	77402695	77402796	
ENSG00000206820	0	0.390640832	snRNA	4	114341479	114341642	
ENSG00000206822	0.869158192	0.390640832	misc_RNA	14	64088478	64088589	
ENSG00000206823	2.471521042	3.845183986	snoRNA	4	119200345	119200475	SNORA24
ENSG00000206824	0	0.390640832	misc_RNA	17	48463506	48463607	
ENSG00000206830	0.499066092	0.390640832	snRNA	12	51407917	51408024	
ENSG00000206836	0	0.390640832	snRNA	2	198312469	198312575	
ENSG00000206838	2.238690726	2.420525079	snoRNA	7	45143948	45144081	SNORA5A
ENSG00000206846	0.499066092	0	misc_RNA	15	30965953	30966046	
ENSG00000206847	0	0.390640832	misc_RNA	11	128900354	128900467	
ENSG00000206848	0.499066092	0	snRNA	6	42631900	42632006	
ENSG00000206859	0	0.390640832	snRNA	17	17073397	17073503	
ENSG00000206878	1.799000381	2.851901313	snoRNA	1	228788183	228788307	
ENSG00000206881	1.16343121	0	snRNA	6	17619764	17619865	
ENSG00000206885	1.960915222	2.039052734	snoRNA	2	232320511	232320647	SNORA75
ENSG00000206892	0	0.390640832	snRNA	3	196511851	196511957	RNU6-42

ENSG00000206895	0.869158192	0	misc_RNA	7	4850819	4850920	
ENSG00000206897	2.10647801	0.697730409	snoRNA	12	124101255	124101387	
ENSG00000206913	1.407729925	0.950786998	snoRNA	11	73963459	73963597	
ENSG00000206914	0	0.390640832	misc_RNA	12	123252646	123252747	
ENSG00000206921	0	0.697730409	snRNA	1	161371079	161371185	
ENSG00000206924	0.499066092	0.390640832	snRNA	14	75487464	75487570	
ENSG00000206926	0	0.390640832	snRNA	3	15474617	15474723	
ENSG00000206931	0.499066092	0.697730409	snRNA	1	150674342	150674448	
ENSG00000206936	0.499066092	0.950786998	snRNA	X	48939686	48939823	
ENSG00000206938	0.499066092	0	snRNA	7	156664772	156664912	
ENSG00000206941	0	0.390640832	snoRNA	11	75111435	75111582	SNORD15A
ENSG00000206942	3.274897671	3.781359661	snoRNA	7	45024977	45025109	SNORA9
ENSG00000206952	0.499066092	0	snoRNA	16	58593700	58593833	SNORA50
ENSG00000206967	0	0.390640832	misc_RNA	11	108100230	108100331	
ENSG00000206989	0.499066092	0.697730409	snoRNA	5	137896732	137896799	SNORD63
ENSG00000206991	0.869158192	0	snRNA	15	43929830	43929936	
ENSG00000206992	0	0.950786998	snRNA	12	52381441	52381547	
ENSG00000207001	0	0.697730409	snoRNA	15	25299357	25299451	SNORD116-2
ENSG00000207003	0.499066092	1.667587519	snRNA	19	45550716	45550819	
ENSG00000207004	0.499066092	0.697730409	snRNA	14	52190821	52190927	
ENSG00000207008	0.869158192	0.390640832	snoRNA	11	2985001	2985123	SNORA54
ENSG00000207009	0.499066092	0	misc_RNA	4	1685147	1685256	
ENSG00000207011	0	0.390640832	misc_RNA	17	28386070	28386165	RNY4P13
ENSG00000207014	0	0.390640832	snoRNA	15	25302007	25302101	SNORD116-3
ENSG00000207021	0	0.390640832	misc_RNA	17	79540516	79540628	
ENSG00000207031	1.16343121	2.652276565	snoRNA	12	57038811	57038885	SNORD59A
ENSG00000207033	0.499066092	0	snRNA	X	115532008	115532113	
ENSG00000207034	0	0.390640832	misc_RNA	18	2778939	2779055	
ENSG00000207037	0.499066092	0	snRNA	15	85481647	85481753	
ENSG00000207047	1.799000381	2.242360793	snoRNA	2	207026605	207026678	SNORD51
ENSG00000207063	0.499066092	0	snoRNA	15	25296624	25296718	SNORD116-1
ENSG00000207081	0.499066092	0	snRNA	X	134052340	134052446	
ENSG00000207083	0	0.390640832	snRNA	16	69209574	69209680	
ENSG00000207086	0	0.390640832	misc_RNA	2	9755396	9755502	
ENSG00000207087	0	0.390640832	snRNA	2	43318526	43318632	
ENSG00000207088	0.869158192	0.390640832	snoRNA	3	129116053	129116191	SNORA7B
ENSG00000207093	0.499066092	0	snoRNA	15	25315579	25315673	SNORD116-8
ENSG00000207109	0	0.390640832	snoRNA	3	53367087	53367155	
ENSG00000207110	5.707331524	5.42565083	snRNA	8	129011377	129011540	
ENSG00000207129	0.499066092	0.697730409	rRNA	5	87570075	87570193	RN5S187
ENSG00000207133	0	0.390640832	snoRNA	15	25312935	25313029	SNORD116-7
ENSG00000207137	0.499066092	0	snoRNA	15	25324205	25324296	SNORD116-13
ENSG00000207146	0.869158192	0.390640832	misc_RNA	X	107920181	107920287	
ENSG00000207151	0	0.697730409	misc_RNA	X	11134212	11134313	
ENSG00000207158	0.499066092	0	snRNA	20	55361037	55361143	
ENSG00000207166	3.274897671	3.781359661	snoRNA	19	17973397	17973529	SNORA68
ENSG00000207168	0.869158192	0.950786998	snoRNA	7	56128163	56128295	SNORA15
ENSG00000207171	0	0.950786998	snoRNA	4	169392560	169392690	
ENSG00000207175	0.499066092	0.390640832	snRNA	X	118557705	118557868	
ENSG00000207181	1.616589159	1.925536307	snoRNA	1	235291118	235291252	SNORA14B
ENSG00000207186	0	0.390640832	rRNA	2	242524383	242524490	RN5S122
ENSG00000207189	0	0.390640832	misc_RNA	12	123833546	123833647	
ENSG00000207190	0	0.390640832	snRNA	1	64494566	64494672	
ENSG00000207191	0.499066092	0	snoRNA	15	25307480	25307574	SNORD116-5
ENSG00000207200	1.799000381	1.16600992	snRNA	11	63737942	63738048	
ENSG00000207203	0	0.390640832	snRNA	13	36841305	36841411	RNU6-71
ENSG00000207205	0.499066092	0.390640832	snRNA	1	148604908	148605072	
ENSG00000207209	0	0.390640832	misc_RNA	1	146891926	146892027	
ENSG00000207217	0.499066092	0	snoRNA	7	6056508	6056642	
ENSG00000207221	0.499066092	0	snoRNA	11	82752506	82752640	SNORA70E
ENSG00000207233	0.499066092	0.697730409	snoRNA	18	51748654	51748782	SNORA37
ENSG00000207234	0	0.697730409	snRNA	1	89282462	89282568	
ENSG00000207237	0.869158192	0	snRNA	1	26290688	26290791	

ENSG00000207243	0.499066092	0	misc_RNA	16	228553	228655	
ENSG00000207245	0.499066092	0.390640832	snoRNA	15	25351668	25351750	SNORD116-29
ENSG00000207256	0	0.390640832	snRNA	1	43457109	43457215	
ENSG00000207266	0	0.390640832	snRNA	16	28009616	28009722	
ENSG00000207277	0.499066092	0.390640832	rRNA	9	68408924	68409041	
ENSG00000207279	0.499066092	0	snoRNA	15	25339184	25339275	SNORD116-24
ENSG00000207280	2.238690726	1.518964905	snoRNA	2	232321155	232321234	SNORD20
ENSG00000207292	0.499066092	0	misc_RNA	6	149952573	149952685	
ENSG00000207293	0.869158192	0	misc_RNA	5	56464935	56465036	
ENSG00000207297	0.499066092	0.697730409	snoRNA	17	33900676	33900772	SNORD7
ENSG00000207313	1.16343121	0.390640832	snoRNA	12	49061240	49061376	SNORA2B
ENSG00000207315	0.499066092	1.353254395	snoRNA	14	103804186	103804311	SNORA28
ENSG00000207327	0	0.697730409	snRNA	10	75288809	75288915	
ENSG00000207331	0	0.697730409	snRNA	3	98627303	98627408	
ENSG00000207336	0.499066092	0.390640832	snRNA	5	74020924	74021029	
ENSG00000207342	1.616589159	0.697730409	misc_RNA	14	89306509	89306607	
ENSG00000207344	0.869158192	0	snoRNA	7	64526377	64526510	
ENSG00000207357	0.869158192	0.390640832	snRNA	19	1021521	1021627	
ENSG00000207359	2.57521082	0.390640832	snRNA	8	90912949	90913052	
ENSG00000207363	0	0.390640832	misc_RNA	X	107144375	107144476	
ENSG00000207375	0.499066092	0	snoRNA	15	25336933	25337024	SNORD116-23
ENSG00000207380	0	0.390640832	misc_RNA	20	5945184	5945296	
ENSG00000207382	0.499066092	0	misc_RNA	17	60738982	60739086	
ENSG00000207383	1.616589159	1.802319292	misc_RNA	2	113337061	113337161	
ENSG00000207392	0	1.667587519	snoRNA	6	160201282	160201413	SNORA20
ENSG00000207399	0.499066092	1.16600992	snRNA	8	104397189	104397295	
ENSG00000207424	2.762599152	3.935919592	snoRNA	11	62621135	62621204	SNORD30
ENSG00000207425	2.762599152	2.039052734	misc_RNA	16	15009314	15009413	
ENSG00000207426	0.499066092	0	misc_RNA	X	95956506	95956618	
ENSG00000207427	0.869158192	1.667587519	snoRNA	20	2635713	2635844	SNORA51
ENSG00000207442	0	0.697730409	snoRNA	15	25310173	25310268	SNORD116-6
ENSG00000207445	3.744048221	3.368666104	snoRNA	11	75115465	75115610	SNORD15B
ENSG00000207447	0.499066092	0	snRNA	10	13259269	13259375	RNU6-2
ENSG00000207450	0	0.390640832	misc_RNA	8	48717640	48717740	
ENSG00000207451	0.499066092	0	snRNA	1	11286311	11286417	
ENSG00000207457	1.799000381	1.16600992	snRNA	22	42075715	42075821	
ENSG00000207460	0	0.390640832	snoRNA	15	25331674	25331765	SNORD116-19
ENSG00000207464	1.16343121	0	snoRNA	15	25304685	25304780	SNORD116-4
ENSG00000207475	1.799000381	1.353254395	snoRNA	1	155889700	155889836	SNORA42
ENSG00000207493	0	0.697730409	snoRNA	16	58582403	58582537	SNORA46
ENSG00000207494	0	0.390640832	misc_RNA	10	126503527	126503631	
ENSG00000207498	0	0.390640832	snRNA	15	50639620	50639726	
ENSG00000207501	1.799000381	0.950786998	snRNA	1	148241465	148241628	
ENSG00000207514	0	0.390640832	snRNA	16	72410603	72410659	
ENSG00000207523	1.960915222	2.912743273	snoRNA	1	93306276	93306408	SNORA66
ENSG00000207525	1.616589159	0.697730409	misc_RNA	16	74497786	74497886	
ENSG00000207546	0	0.390640832	miRNA	12	65016289	65016385	MIR548C
ENSG00000207547	3.335220907	3.411459265	miRNA	7	99691183	99691266	MIR25
ENSG00000207550	0	0.390640832	miRNA	19	52195865	52195934	MIR99B
ENSG00000207554	3.211941663	2.971122874	miRNA	20	62573984	62574079	MIR647
ENSG00000207556	0.869158192	2.242360793	miRNA	17	74732532	74732630	MIR636
ENSG00000207561	1.960915222	2.334191469	miRNA	17	66420592	66420689	MIR635
ENSG00000207563	0.499066092	0	miRNA	9	97847490	97847586	MIR23B
ENSG00000207574	0	0.697730409	miRNA	8	145019359	145019447	MIR661
ENSG00000207588	0	0.390640832	miRNA	7	127721913	127722012	MIR593
ENSG00000207590	0.499066092	0.390640832	miRNA	1	220291195	220291304	MIR215
ENSG00000207606	0.499066092	0	miRNA	1	151518272	151518367	MIR554
ENSG00000207607	1.16343121	0.390640832	miRNA	1	1103243	1103332	MIR200A
ENSG00000207611	0	0.390640832	miRNA	2	241395418	241395506	MIR149
ENSG00000207612	0	0.390640832	miRNA	10	29833933	29834026	MIR604
ENSG00000207614	1.407729925	0.697730409	miRNA	17	29887015	29887102	MIR193A
ENSG00000207618	0.499066092	0	miRNA	17	27188551	27188636	MIR144
ENSG00000207622	0.499066092	0.390640832	miRNA	12	109230684	109230782	MIR619

ENSG00000207623	1.16343121	0	miRNA	1	209605478	209605587	MIR205
ENSG00000207624	0.499066092	0	miRNA	1	220291499	220291583	MIR194-1
ENSG00000207626	0	0.390640832	miRNA	2	233037363	233037457	MIR562
ENSG00000207627	0	0.390640832	miRNA	5	53247334	53247429	MIR581
ENSG00000207631	1.16343121	1.518964905	miRNA	19	40788450	40788548	MIR641
ENSG00000207635	0.499066092	1.353254395	miRNA	20	33578179	33578300	MIR499A
ENSG00000207643	0	0.950786998	miRNA	4	65017	65125	
ENSG00000207648	0.499066092	0.390640832	miRNA	11	64658609	64658718	MIR192
ENSG00000207650	2.847891871	2.652276565	miRNA	3	195426272	195426368	MIR570
ENSG00000207652	3.652182994	2.912743273	miRNA	13	41384902	41384997	MIR621
ENSG00000207653	0.499066092	0	miRNA	2	32757220	32757313	MIR558
ENSG00000207693	0	0.390640832	miRNA	9	140732871	140732968	MIR602
ENSG00000207696	0.499066092	0.390640832	miRNA	22	38243685	38243781	MIR659
ENSG00000207697	1.407729925	0.950786998	miRNA	4	24521815	24521913	MIR573
ENSG00000207698	0	0.390640832	miRNA	9	111808509	111808578	MIR32
ENSG00000207704	0.869158192	1.16600992	miRNA	8	124360274	124360370	MIR548D1
ENSG00000207709	1.16343121	0.950786998	miRNA	1	110141513	110141593	MIR197
ENSG00000207719	0.499066092	0	miRNA	13	100008385	100008482	MIR623
ENSG00000207721	1.407729925	2.039052734	miRNA	1	71533314	71533399	MIR186
ENSG00000207725	0.869158192	0	miRNA	X	45606421	45606530	MIR222
ENSG00000207730	1.16343121	0	miRNA	1	1102484	1102578	MIR200B
ENSG00000207733	3.871730003	3.368666104	miRNA	19	3961412	3961510	MIR637
ENSG00000207737	0.499066092	0.390640832	miRNA	9	127455989	127456077	MIR181B2
ENSG00000207741	1.407729925	2.501982735	miRNA	7	73605528	73605624	MIR590
ENSG00000207750	0	0.697730409	miRNA	1	100746797	100746864	MIR553
ENSG00000207752	0	0.950786998	miRNA	19	10928102	10928172	MIR199A1
ENSG00000207755	0	0.390640832	miRNA	X	133674538	133674637	MIR450A2
ENSG00000207757	2.35979773	1.802319292	miRNA	7	99691391	99691470	MIR93
ENSG00000207770	1.16343121	1.667587519	miRNA	3	114035322	114035416	MIR568
ENSG00000207773	0	0.697730409	miRNA	19	46178186	46178282	MIR642A
ENSG00000207780	0.499066092	0	miRNA	22	18463634	18463727	MIR648
ENSG00000207787	0.499066092	0.697730409	miRNA	X	53583184	53583302	MIR98
ENSG00000207789	0	0.697730409	miRNA	12	58218392	58218475	MIR26A2
ENSG00000207808	2.35979773	1.667587519	miRNA	19	13947254	13947331	MIR27A
ENSG00000207814	0.499066092	0	miRNA	9	123007257	123007328	MIR147A
ENSG00000207820	0.499066092	0	miRNA	X	73506939	73507044	MIR545
ENSG00000207839	0.499066092	1.16600992	miRNA	17	17717150	17717245	MIR33B
ENSG00000207864	0	0.390640832	miRNA	9	97847727	97847823	MIR27B
ENSG00000207865	0.869158192	0.390640832	miRNA	1	9211727	9211836	MIR34A
ENSG00000207870	2.35979773	1.353254395	miRNA	X	45605585	45605694	MIR221
ENSG00000207874	0	0.390640832	miRNA	11	28078362	28078457	MIR610
ENSG00000207929	0.499066092	0	miRNA	17	6920934	6921020	MIR195
ENSG00000207932	0.869158192	0	miRNA	22	42296948	42297016	MIR33A
ENSG00000207933	0.499066092	0.390640832	miRNA	1	156390133	156390221	MIR9-1
ENSG00000207944	0.499066092	0.390640832	miRNA	4	38869653	38869748	MIR574
ENSG00000207948	0	0.390640832	miRNA	16	67236224	67236298	MIR328
ENSG00000207949	0	0.390640832	miRNA	1	172107938	172108047	MIR214
ENSG00000207956	0	0.950786998	miRNA	5	32394484	32394581	MIR579
ENSG00000207964	0	0.697730409	miRNA	15	55665138	55665232	MIR628
ENSG00000207966	0.499066092	1.16600992	miRNA	1	207975197	207975284	MIR29C
ENSG00000207973	1.407729925	1.16600992	miRNA	7	5535450	5535548	MIR589
ENSG00000207975	0	0.390640832	miRNA	1	198828002	198828111	MIR181B1
ENSG00000207980	2.471521042	2.652276565	miRNA	19	13947401	13947473	MIR23A
ENSG00000207983	0	0.697730409	miRNA	12	12917583	12917677	MIR613
ENSG00000207985	6.693459083	8.302443832	miRNA	20	26188822	26188914	MIR663A
ENSG00000207991	0	0.390640832	miRNA	9	126164804	126164882	MIR601
ENSG00000207997	0	1.16600992	miRNA	20	33054130	33054223	MIR644A
ENSG00000208002	0	0.390640832	miRNA	19	52785050	52785146	MIR643
ENSG00000208005	1.16343121	0.950786998	miRNA	X	133680358	133680428	MIR503
ENSG00000208008	0	0.390640832	miRNA	19	52196507	52196592	MIR125A
ENSG00000208011	0	0.950786998	miRNA	1	155164968	155165063	MIR92B
ENSG00000208018	0	0.390640832	miRNA	20	49202323	49202416	MIR645
ENSG00000208028	2.35979773	2.579085888	miRNA	12	57912946	57913042	MIR616

ENSG00000208036	1.407729925	1.802319292	miRNA	7	99691616	99691697	MIR106B
ENSG00000208308	0	0.390640832	snoRNA	2	135894198	135894325	
ENSG00000208310	1.16343121	2.652276565	snoRNA	1	173836017	173836076	SNORD75
ENSG00000208317	3.448790144	3.748356452	snoRNA	1	173834760	173834824	SNORD78
ENSG00000208506	0.869158192	0.697730409	snRNA	2	122288457	122288583	RNU4ATAC
ENSG00000208772	2.238690726	1.925536307	snoRNA	2	86362993	86363129	SNORD94
ENSG00000208883	0	0.390640832	snoRNA	X	109468213	109468291	SNORD96B
ENSG00000208892	0.499066092	0.697730409	snoRNA	12	132515769	132515904	SNORA49
ENSG00000209042	3.744048221	2.579085888	snoRNA	20	47895477	47895565	SNORD12C
ENSG00000209082	7.834876234	7.040122572	Mt_tRNA	MT	3230	3304	
ENSG00000209418	2.10647801	1.518964905	snoRNA	15	25230247	25230313	SNORD64
ENSG00000209482	4.261916566	5.09905904	snoRNA	22	39711218	39711312	SNORD83A
ENSG00000209582	5.255381949	4.65855978	snoRNA	17	7478031	7478165	SNORA48
ENSG00000209645	1.616589159	1.16600992	snoRNA	19	10218327	10218411	SNORD105
ENSG00000209702	0.499066092	0.697730409	snoRNA	19	12817263	12817332	SNORD41
ENSG00000209707	2.57521082	1.802319292	miRNA	19	13947101	13947173	MIR24-2
ENSG00000209804	1.407729925	0.697730409	snRNA	1	28975112	28975245	RNU11
ENSG00000210049	7.04004079	6.235021771	Mt_tRNA	MT	577	647	
ENSG00000210077	6.041940478	5.372493443	Mt_tRNA	MT	1602	1670	
ENSG00000210082	16.9430781	18.24466944	Mt_rRNA	MT	1671	3229	MIR4485
ENSG00000210100	4.842525946	3.96493948	Mt_tRNA	MT	4263	4331	
ENSG00000210112	2.847891871	2.652276565	Mt_tRNA	MT	4402	4469	
ENSG00000210117	3.274897671	2.501982735	Mt_tRNA	MT	5512	5579	
ENSG00000210140	9.061114416	8.936206949	Mt_tRNA	MT	5761	5826	
ENSG00000210144	9.026088136	8.791837833	Mt_tRNA	MT	5826	5891	
ENSG00000210154	0	0.950786998	Mt_tRNA	MT	7518	7585	
ENSG00000210156	0.499066092	0.390640832	Mt_tRNA	MT	8295	8364	
ENSG00000210164	4.883502971	4.826083323	Mt_tRNA	MT	9991	10058	
ENSG00000210174	4.689181911	4.29708397	Mt_tRNA	MT	10405	10469	
ENSG00000210176	5.239686603	4.94736961	Mt_tRNA	MT	12138	12206	
ENSG00000210184	4.689181911	4.640685763	Mt_tRNA	MT	12207	12265	
ENSG00000210191	5.538004898	5.271576845	Mt_tRNA	MT	12266	12336	
ENSG00000210194	3.146112541	3.845183986	Mt_tRNA	MT	14674	14742	
ENSG00000210195	4.593635215	3.714580548	Mt_tRNA	MT	15888	15953	
ENSG00000210196	8.299696963	8.512355001	Mt_tRNA	MT	15956	16023	
ENSG00000210841	0	0.390640832	snRNA	3	57605174	57605299	
ENSG00000211445	6.515894502	5.583678991	protein_coding	5	150400124	150408554	GPX3
ENSG00000211448	7.218789138	6.25865215	protein_coding	14	80663873	80854100	DIO2
ENSG00000211450	7.726437377	8.302443832	protein_coding	11	57508825	57510529	C11orf31
ENSG00000211451	3.004694206	2.912743273	protein_coding	1	145509752	145516076	GNRHR2
ENSG00000211452	0.499066092	1.518964905	protein_coding	1	54356912	54376759	DIO1
ENSG00000211454	3.871730003	3.845183986	polymorphic_pse	1	19592478	19600688	AKR7L
ENSG00000211455	8.652173596	8.851330023	protein_coding	12	27396901	27478892	STK38L
ENSG00000211456	8.146099194	8.548751381	protein_coding	3	45730548	45786901	SACM1L
ENSG00000211459	13.55172566	15.27886679	Mt_rRNA	MT	648	1601	
ENSG00000211460	9.789607412	9.190788227	protein_coding	2	122494679	122525429	TSN
ENSG00000211482	0	1.518964905	miRNA	4	403598	403704	
ENSG00000211513	1.407729925	0.390640832	miRNA	19	47212541	47212607	MIR320E
ENSG00000211553	0.869158192	1.802319292	miRNA	4	130242	130349	
ENSG00000211571	0	0.390640832	miRNA	19	56196138	56196282	
ENSG00000211581	0	0.697730409	miRNA	1	156905923	156906036	MIR765
ENSG00000211584	6.305850085	5.776071827	protein_coding	12	48147699	48176536	SLC48A1
ENSG00000211643	0.499066092	0	IG_V_gene	22	22673082	22673581	IGLV5-52
ENSG00000211644	2.238690726	2.039052734	IG_V_gene	22	22676828	22677336	IGLV1-51
ENSG00000211683	2.10647801	1.925536307	processed_transc	22	23974071	23981128	
ENSG00000211714	0	0.390640832	TR_V_gene	7	142247109	142247615	TRBV7-3
ENSG00000211772	0	0.390640832	TR_C_gene	7	142498725	142500432	TRBC2
ENSG00000211843	0	0.390640832	TR_J_gene	14	22962389	22962451	TRAJ46
ENSG00000211890	0.499066092	0	IG_C_gene	14	106053226	106054732	IGHA2
ENSG00000212024	0	0.390640832	miRNA	7	29720349	29720445	MIR550A3
ENSG00000212027	0	0.697730409	miRNA	X	73438382	73438453	MIR374B
ENSG00000212045	0	0.390640832	miRNA	4	29751818	29751932	
ENSG00000212089	0	0.390640832	miRNA	11	71789376	71789466	

ENSG00000212093	0	0.390640832	miRNA	11	68273551	68273634	
ENSG00000212123	6.887241849	7.720473624	protein_coding	19	5782971	5784776	PRR22
ENSG00000212124	0.869158192	1.353254395	protein_coding	12	11174218	11175219	TAS2R19
ENSG00000212125	1.16343121	0.950786998	pseudogene	12	11117024	11117951	TAS2R15
ENSG00000212126	0.499066092	0.390640832	protein_coding	12	11138512	11139511	TAS2R50
ENSG00000212127	4.230497448	3.96493948	protein_coding	12	11090005	11324172	TAS2R14
ENSG00000212128	0.499066092	0.390640832	protein_coding	12	11060525	11062161	TAS2R13
ENSG00000212135	0.499066092	1.353254395	snoRNA	11	46783939	46784049	SNORD67
ENSG00000212138	0	0.697730409	rRNA	12	109497791	109497905	
ENSG00000212144	0	0.390640832	snoRNA	1	234729021	234729148	
ENSG00000212158	0	0.390640832	snoRNA	3	184043484	184043559	SNORD66
ENSG00000212163	0	0.950786998	snoRNA	17	2233570	2233664	SNORD91A
ENSG00000212175	0.499066092	0	snoRNA	2	55792839	55792986	
ENSG00000212195	0.869158192	1.353254395	snoRNA	17	56709003	56709197	
ENSG00000212232	3.274897671	3.453019579	snoRNA	20	17943353	17943589	SNORD17
ENSG00000212237	2.671945279	3.32456471	rRNA	1	228783659	228783779	RN5S18
ENSG00000212240	0	1.16600992	snRNA	6	28883422	28883521	
ENSG00000212242	0.499066092	0.697730409	rRNA	6	136951381	136951493	RN5S219
ENSG00000212257	0.499066092	0	snRNA	1	65488651	65488757	
ENSG00000212264	0	0.390640832	snoRNA	7	23436065	23436135	
ENSG00000212283	2.10647801	1.16600992	snoRNA	2	101889398	101889511	SNORD89
ENSG00000212296	1.407729925	2.334191469	snoRNA	5	40832758	40832837	SNORD72
ENSG00000212300	1.16343121	1.353254395	snoRNA	4	159621843	159622060	
ENSG00000212302	0	0.390640832	snoRNA	14	23225970	23226038	
ENSG00000212304	3.744048221	3.64455972	snoRNA	20	47897220	47897309	SNORD12
ENSG00000212309	1.16343121	0.390640832	snoRNA	2	203142831	203142915	
ENSG00000212327	0	0.390640832	snRNA	3	9960735	9960837	
ENSG00000212332	0.499066092	0	snRNA	10	88248203	88248306	
ENSG00000212335	0.869158192	0.390640832	misc_RNA	14	23321825	23321937	
ENSG00000212342	0	0.697730409	snoRNA	8	131175218	131175373	
ENSG00000212371	1.407729925	2.652276565	snoRNA	14	77932627	77932779	
ENSG00000212385	0.499066092	0.697730409	snRNA	1	116956388	116956491	
ENSG00000212391	0	0.390640832	snoRNA	2	227833705	227833838	
ENSG00000212396	0.499066092	1.802319292	rRNA	10	95270305	95270437	
ENSG00000212402	0	0.950786998	snoRNA	5	172447731	172447931	SNORA74B
ENSG00000212411	0	0.390640832	snoRNA	10	29864235	29864310	
ENSG00000212418	0	0.697730409	misc_RNA	17	74699714	74699809	
ENSG00000212432	0.499066092	0	snoRNA	12	9597654	9597801	
ENSG00000212443	1.616589159	1.667587519	snoRNA	12	98993413	98993661	SNORA53
ENSG00000212445	0.499066092	0.390640832	snoRNA	16	68223190	68223324	
ENSG00000212452	2.762599152	2.334191469	snoRNA	3	52726752	52726828	SNORD69
ENSG00000212456	0	0.390640832	snRNA	1	147994146	147994313	
ENSG00000212458	0.499066092	1.16600992	snoRNA	4	1112672	1112801	
ENSG00000212461	0	0.390640832	snoRNA	12	72033135	72033263	
ENSG00000212464	0.869158192	0.697730409	snoRNA	10	101996913	101997059	SNORA12
ENSG00000212469	0	0.390640832	snRNA	17	54948531	54948637	
ENSG00000212493	0.499066092	1.802319292	snoRNA	3	52723264	52723331	SNORD19
ENSG00000212497	1.407729925	0.390640832	rRNA	19	12138728	12138836	
ENSG00000212517	0.499066092	0.390640832	snoRNA	20	5102063	5102185	
ENSG00000212533	1.16343121	0.697730409	snoRNA	12	31229801	31229950	
ENSG00000212534	1.616589159	0.390640832	snoRNA	2	203141154	203141241	SNORD70
ENSG00000212536	0.869158192	0.697730409	rRNA	20	5079033	5079122	
ENSG00000212541	0	1.16600992	snRNA	1	38457134	38457241	
ENSG00000212544	0.499066092	0	snRNA	1	149514090	149514256	
ENSG00000212549	0	0.390640832	rRNA	12	26526531	26526666	
ENSG00000212552	2.10647801	0.697730409	snoRNA	17	2232413	2232507	SNORD91B
ENSG00000212559	0.499066092	0	rRNA	13	94677724	94677852	RN5S35
ENSG00000212569	0.499066092	0	misc_RNA	22	51108232	51108329	
ENSG00000212576	0.869158192	0.697730409	rRNA	19	12217312	12217429	
ENSG00000212579	0	0.390640832	snoRNA	6	35619595	35619722	
ENSG00000212588	2.35979773	2.912743273	snoRNA	4	53579416	53579537	SNORA26
ENSG00000212593	0	0.390640832	snoRNA	16	23453162	23453242	
ENSG00000212607	2.35979773	3.608232228	snoRNA	11	8706986	8707116	SNORA45

ENSG00000212615	0	0.697730409	snoRNA	14	45557446	45557511	
ENSG00000212624	0	0.390640832	snoRNA	1	52190447	52190568	
ENSG00000212664	1.407729925	1.925536307	pseudogene	15	71633466	71634086	
ENSG00000212670	2.928422289	3.232099092	protein_coding	1	54569968	54571750	
ENSG00000212673	1.16343121	2.652276565	protein_coding	1	32379174	32380745	
ENSG00000212694	6.103936836	6.064512706	lincRNA	12	122233173	122241812	
ENSG00000212695	2.10647801	2.420525079	pseudogene	9	8713311	8713841	
ENSG00000212719	9.706408721	10.42379484	protein_coding	17	21428051	21477781	C17orf51
ENSG00000212724	3.393122761	0.390640832	protein_coding	17	39215495	39216344	KRTAP2-3
ENSG00000212743	1.960915222	1.16600992	protein_coding	10	6392278	6394723	
ENSG00000212747	8.215945216	8.283850589	protein_coding	X	134154543	134156559	FAM127C
ENSG00000212766	1.407729925	1.16600992	processed_transc	15	69365266	69388148	LINC00277
ENSG00000212769	0.499066092	0.390640832	pseudogene	10	85841185	85842572	HMGN2P8
ENSG00000212789	1.799000381	0.950786998	pseudogene	11	18283529	18284638	ST13P5
ENSG00000212802	3.077135474	3.279072565	pseudogene	6	12514342	12515006	RPL15P3
ENSG00000212829	3.004694206	3.133298822	pseudogene	9	9090874	9091323	RPS26P3
ENSG00000212864	4.381167248	4.888000647	protein_coding	9	140114707	140116033	RNF208
ENSG00000212907	14.31421674	13.50597938	protein_coding	MT	10470	10766	MT-ND4L
ENSG00000212916	5.054730169	4.777836593	protein_coding	1	232940643	232946092	KIAA1383
ENSG00000212951	0.869158192	0.950786998	pseudogene	9	46842616	46844326	
ENSG00000212961	0	1.16600992	pseudogene	11	74065488	74066765	
ENSG00000212978	7.119373418	7.145279744	processed_transc	2	61368732	61372104	
ENSG00000212993	2.35979773	0.697730409	protein_coding	8	128426535	128432311	POU5F1B
ENSG00000212994	0.499066092	0.390640832	pseudogene	8	101907974	101908387	RPS26P6
ENSG00000212997	1.407729925	0.697730409	protein_coding	8	95558771	95560630	
ENSG00000212998	2.762599152	2.971122874	protein_coding	8	94752349	94753001	C8orf39
ENSG00000213005	1.16343121	0.390640832	pseudogene	8	67679632	67680240	PTTG3P
ENSG00000213013	0.499066092	0	pseudogene	19	58727546	58727927	
ENSG00000213014	0.869158192	0.390640832	pseudogene	19	56828527	56830849	VN2R17P
ENSG00000213015	8.485841807	8.796890297	protein_coding	19	56152392	56154835	ZNF580
ENSG00000213018	2.10647801	1.802319292	pseudogene	X	70639933	70640364	
ENSG00000213020	7.947856304	7.546612122	protein_coding	19	53206066	53238307	ZNF611
ENSG00000213022	1.407729925	0	protein_coding	19	51503806	51512890	KLK9
ENSG00000213023	4.097495944	2.912743273	protein_coding	19	51125236	51171651	SYT3
ENSG00000213024	10.09671625	10.05516663	protein_coding	19	50410086	50432786	NUP62
ENSG00000213025	3.448790144	3.993387124	pseudogene	10	70392128	70392484	
ENSG00000213028	1.960915222	1.353254395	pseudogene	1	229824746	229825651	
ENSG00000213030	2.238690726	2.420525079	protein_coding	19	49538826	49552400	CGB8
ENSG00000213033	0.499066092	0.390640832	pseudogene	1	220440053	220441259	AURKAPS1
ENSG00000213036	0	0.950786998	pseudogene	1	214656156	214656730	
ENSG00000213041	0.499066092	0.390640832	pseudogene	1	205171319	205171871	
ENSG00000213046	0	0.950786998	pseudogene	1	197657890	197659765	EEF1A1P32
ENSG00000213047	6.050961887	6.440109466	protein_coding	1	197473878	197744826	DENND1B
ENSG00000213050	0.869158192	1.353254395	pseudogene	7	116612219	116612965	TPM3P1
ENSG00000213051	1.16343121	1.16600992	pseudogene	1	185195937	185196832	
ENSG00000213055	0.869158192	0.697730409	pseudogene	2	233594233	233594908	
ENSG00000213057	0.499066092	1.518964905	protein_coding	1	178511887	178518024	C1orf220
ENSG00000213060	0	0.697730409	pseudogene	1	171772657	171773099	
ENSG00000213061	0	0.390640832	pseudogene	10	104597768	104605230	PFN1P11
ENSG00000213062	3.077135474	1.802319292	pseudogene	1	169455380	169456846	
ENSG00000213063	0.499066092	0.950786998	pseudogene	1	168907705	168908161	
ENSG00000213064	5.774228067	5.751299057	protein_coding	1	168195176	168212378	SFT2D2
ENSG00000213066	7.593829938	8.001751392	protein_coding	6	167412670	167455906	FGFR1OP
ENSG00000213069	0	0.390640832	pseudogene	2	178062713	178063262	KRT8P40
ENSG00000213071	1.799000381	0.697730409	pseudogene	6	160874460	160932156	LPAL2
ENSG00000213073	4.292665995	4.468996429	pseudogene	6	160514114	160517244	
ENSG00000213075	1.616589159	0	pseudogene	1	161653485	161655042	RPL31P11
ENSG00000213077	2.471521042	2.788380093	protein_coding	17	18427880	18430160	FAM106A
ENSG00000213078	0	0.390640832	pseudogene	6	157787022	157787955	
ENSG00000213079	8.311006074	8.315177126	protein_coding	6	155054459	155155192	SCAF8
ENSG00000213080	2.238690726	2.144285137	pseudogene	1	160864770	160865866	
ENSG00000213082	1.960915222	1.353254395	pseudogene	2	208546314	208546757	PPP1R14BP2
ENSG00000213085	5.83815965	5.871116342	protein_coding	1	159842154	159869953	CCDC19

ENSG00000213087	0.499066092	0.697730409	pseudogene	6	151546685	151547082	
ENSG00000213090	0.499066092	0.390640832	pseudogene	2	202275267	202278031	
ENSG00000213096	7.700873103	7.429177016	protein_coding	19	24216276	24312643	ZNF254
ENSG00000213100	1.16343121	0	pseudogene	X	45842800	45843799	
ENSG00000213104	1.16343121	0.390640832	pseudogene	2	198244425	198245616	
ENSG00000213109	0	0.390640832	pseudogene	6	137221521	137222289	
ENSG00000213111	1.407729925	3.027231696	pseudogene	6	136355655	136356026	COX5BP2
ENSG00000213117	0	0.390640832	pseudogene	6	135219012	135219889	
ENSG00000213121	0.499066092	0	protein_coding	6	153552455	153668623	
ENSG00000213123	5.948480845	6.246885341	protein_coding	3	195975101	196045170	TCTEX1D2
ENSG00000213126	0.499066092	0	pseudogene	2	180678978	180679402	
ENSG00000213131	0.869158192	1.353254395	pseudogene	6	127676901	127677934	YWHAZP4
ENSG00000213132	1.960915222	0.697730409	protein_coding	3	187896331	187898598	
ENSG00000213135	1.407729925	1.667587519	pseudogene	17	75085986	75087063	
ENSG00000213137	0	0.390640832	pseudogene	12	121282913	121283450	ARF1P2
ENSG00000213139	3.146112541	3.714580548	protein_coding	3	186256230	186264491	CRYGS
ENSG00000213140	0	0.390640832	pseudogene	14	106138648	106139651	ELK2AP
ENSG00000213144	1.960915222	2.334191469	pseudogene	12	119632655	119633199	
ENSG00000213145	2.10647801	1.667587519	protein_coding	14	105952654	105955284	CRIP1
ENSG00000213149	0.869158192	0.950786998	pseudogene	6	111179442	111180350	CNN2P9
ENSG00000213152	1.16343121	1.353254395	pseudogene	12	112739677	112740467	RPL7AP60
ENSG00000213160	7.55947245	7.157901395	protein_coding	2	170550998	170633499	KLHL23
ENSG00000213169	0.499066092	0.697730409	pseudogene	3	174095034	174095795	
ENSG00000213171	0	0.390640832	protein_coding	1	151772740	151778630	LINGO4
ENSG00000213174	0.499066092	0.390640832	pseudogene	3	170371635	170372048	
ENSG00000213176	0	0.950786998	pseudogene	14	103507806	103508431	RPL13P6
ENSG00000213177	0.869158192	1.802319292	pseudogene	6	100583940	100584539	
ENSG00000213178	1.16343121	1.802319292	pseudogene	3	169201459	169201868	
ENSG00000213179	0	0.390640832	pseudogene	17	65780869	65781409	
ENSG00000213180	0	0.390640832	pseudogene	17	65220844	65221157	
ENSG00000213184	0	0.390640832	pseudogene	11	122888346	122889369	
ENSG00000213185	0.499066092	1.518964905	protein_coding	10	124608594	124639157	FAM24B
ENSG00000213186	8.316627558	8.076782959	protein_coding	3	160150233	160203561	TRIM59
ENSG00000213188	0.499066092	0.950786998	pseudogene	7	151825964	151826771	YBX1P4
ENSG00000213189	0.499066092	0.697730409	pseudogene	2	159860287	159861065	BTF3L4P2
ENSG00000213190	5.995967334	6.89311646	protein_coding	1	151030234	151040970	MLLT11
ENSG00000213194	0.869158192	0.697730409	pseudogene	2	158067359	158068051	
ENSG00000213197	0.499066092	1.518964905	pseudogene	2	153246451	153247144	
ENSG00000213199	7.768062907	7.23139676	protein_coding	7	150745379	150749843	ACCN3
ENSG00000213203	0.499066092	0	protein_coding	7	150413645	150421372	GIMAP1
ENSG00000213204	3.004694206	2.721932731	protein_coding	6	88117701	88221352	C6orf165
ENSG00000213209	0.499066092	0.390640832	pseudogene	7	148334271	148334679	
ENSG00000213212	2.762599152	2.039052734	pseudogene	9	139707240	139709988	NCLP1
ENSG00000213213	4.292665995	4.777836593	protein_coding	9	139690790	139704494	KIAA1984
ENSG00000213214	2.35979773	0	protein_coding	7	143883176	143991230	ARHGEF35
ENSG00000213216	0	0.697730409	pseudogene	9	125791652	125792032	
ENSG00000213218	2.928422289	2.652276565	protein_coding	17	61949372	61951126	CSH2
ENSG00000213221	2.35979773	3.027231696	protein_coding	9	139253932	139258241	DNLZ
ENSG00000213222	4.322773689	5.045726316	pseudogene	2	132480826	132481852	
ENSG00000213225	1.16343121	1.16600992	pseudogene	2	130986949	130989679	
ENSG00000213226	0	0.390640832	pseudogene	1	146790828	146791941	
ENSG00000213228	0.499066092	0.390640832	pseudogene	17	58511084	58511573	
ENSG00000213234	0	1.16600992	pseudogene	11	112138013	112139094	ST13P10
ENSG00000213235	2.10647801	2.721932731	pseudogene	12	17143604	17145017	EEF1A1P33
ENSG00000213236	0.499066092	0	pseudogene	2	127315012	127315739	YWHAZP2
ENSG00000213237	1.616589159	2.144285137	pseudogene	7	138088940	138089268	
ENSG00000213239	0.499066092	0.390640832	pseudogene	2	122466088	122466691	
ENSG00000213240	5.649112675	6.25865215	protein_coding	1	145209119	145291954	NOTCH2NL
ENSG00000213244	2.10647801	2.334191469	pseudogene	1	120904675	120905090	
ENSG00000213246	8.640267888	9.038718229	protein_coding	17	56422539	56429563	SUPT4H1
ENSG00000213250	0.869158192	0.390640832	pseudogene	12	94817520	94818745	RBMS2P1
ENSG00000213252	0.499066092	0	pseudogene	11	106697118	106698616	
ENSG00000213253	1.960915222	0.697730409	pseudogene	19	13268773	13269602	

ENSG00000213260	0	0.390640832	pseudogene	10	107446080	107446809	YWHAZP5
ENSG00000213261	0.499066092	1.518964905	pseudogene	7	131346711	131347383	
ENSG00000213269	0	0.697730409	pseudogene	X	73595960	73596720	
ENSG00000213270	1.16343121	0.697730409	pseudogene	12	83545110	83545966	RPL6P25
ENSG00000213272	0.499066092	0.390640832	pseudogene	12	76993451	76994247	
ENSG00000213275	1.616589159	1.518964905	pseudogene	11	69070879	69071274	
ENSG00000213277	1.616589159	1.16600992	pseudogene	10	104935311	104935851	
ENSG00000213280	0.499066092	0.950786998	pseudogene	7	128210295	128210742	
ENSG00000213281	9.645920794	9.448154031	protein_coding	1	115247090	115259515	NRAS
ENSG00000213283	5.458875168	5.125003832	pseudogene	2	96191093	96192968	
ENSG00000213287	0	0.390640832	pseudogene	11	86969893	86970301	
ENSG00000213290	2.35979773	2.144285137	pseudogene	19	12670385	12671919	
ENSG00000213291	0.499066092	0.390640832	pseudogene	7	124940558	124940932	
ENSG00000213293	0.499066092	0.950786998	pseudogene	19	12370941	12371352	
ENSG00000213296	0.499066092	0.390640832	pseudogene	7	124120578	124121449	
ENSG00000213300	2.671945279	3.027231696	pseudogene	3	75263782	75264787	
ENSG00000213303	2.10647801	1.802319292	pseudogene	19	11634251	11634646	
ENSG00000213304	2.847891871	2.579085888	pseudogene	19	11632783	11633313	
ENSG00000213305	0	0.950786998	pseudogene	11	84731829	84732699	
ENSG00000213309	0.499066092	0.697730409	pseudogene	6	64325732	64326266	
ENSG00000213310	0.499066092	0	pseudogene	7	121038506	121038821	
ENSG00000213312	0	1.667587519	pseudogene	6	64258613	64259413	
ENSG00000213315	3.78787645	4.29708397	pseudogene	14	91232875	91233333	
ENSG00000213316	0.499066092	0.390640832	protein_coding	5	179220981	179223648	LTC4S
ENSG00000213326	1.616589159	1.16600992	pseudogene	17	44798948	44799533	RPS7P11
ENSG00000213328	0.499066092	0.390640832	pseudogene	5	178336711	178337610	
ENSG00000213331	0	0.697730409	pseudogene	4	188891427	188892438	
ENSG00000213332	0.499066092	0.390640832	pseudogene	4	187249870	187250755	SLC25A5P6
ENSG00000213333	0.499066092	0.697730409	pseudogene	11	74790310	74791176	
ENSG00000213337	5.301468762	5.175534283	protein_coding	2	97502348	97523832	ANKRD39
ENSG00000213339	9.89419068	10.66807958	protein_coding	19	10812112	10824113	QTRT1
ENSG00000213341	8.775675113	9.052302903	protein_coding	10	101948055	101989376	CHUK
ENSG00000213347	6.573375019	7.170413581	protein_coding	5	176728462	176739758	MXD3
ENSG00000213352	1.616589159	2.144285137	pseudogene	12	63149007	63150013	GAPDHP44
ENSG00000213361	0.499066092	0.390640832	pseudogene	7	104885099	104885417	
ENSG00000213362	2.762599152	3.532712221	pseudogene	9	15527131	15527681	FTH1P12
ENSG00000213363	2.57521082	2.501982735	pseudogene	12	62415351	62415955	RPS3P6
ENSG00000213365	0	0.390640832	pseudogene	11	71991195	71992222	
ENSG00000213366	5.588445295	6.434933235	protein_coding	1	110210644	110252171	GSTM2
ENSG00000213370	1.799000381	0.697730409	pseudogene	4	174554879	174555522	RANP6
ENSG00000213371	3.274897671	2.579085888	pseudogene	3	120524713	120525592	NAP1L1P3
ENSG00000213373	0.499066092	0	processed_transc	17	41026687	41050751	
ENSG00000213376	1.960915222	2.039052734	pseudogene	5	173940308	173941310	GAPDHP71
ENSG00000213380	7.30049066	7.195117037	protein_coding	16	69354043	69373570	COG8
ENSG00000213383	0.499066092	0	pseudogene	3	17913221	17914090	
ENSG00000213385	2.471521042	2.242360793	pseudogene	7	102395593	102396386	
ENSG00000213386	2.35979773	1.16600992	pseudogene	5	172189524	172190261	
ENSG00000213390	7.81647827	7.756190105	protein_coding	10	98757795	99052413	ARHGAP19
ENSG00000213394	0	0.390640832	pseudogene	7	100846895	100847776	
ENSG00000213397	5.498982484	5.248158938	protein_coding	X	152713124	152760978	HAUS7
ENSG00000213398	6.573375019	6.470782801	protein_coding	16	67973653	67978414	LCAT
ENSG00000213399	0.869158192	0.697730409	pseudogene	2	85142910	85143789	
ENSG00000213400	0	0.950786998	pseudogene	2	85101820	85102144	RPL12P18
ENSG00000213402	5.124761037	5.94580061	protein_coding	11	67202981	67205538	PTPRCAP
ENSG00000213403	0	0.390640832	pseudogene	2	20026010	20026336	CISD1P1
ENSG00000213406	2.10647801	1.802319292	pseudogene	4	154228944	154229869	ANXA2P1
ENSG00000213409	1.616589159	1.16600992	pseudogene	11	66529046	66529870	
ENSG00000213411	0	0.390640832	pseudogene	13	31113734	31117598	RBM22P2
ENSG00000213412	6.469548347	4.932754837	pseudogene	10	47133338	47133898	
ENSG00000213413	0.869158192	1.16600992	protein_coding	7	99815864	99819113	PVRIG
ENSG00000213416	0.869158192	0	protein_coding	17	39279343	39280419	KRTAP4-12
ENSG00000213420	5.684325135	5.09905904	protein_coding	7	99767229	99774995	GPC2
ENSG00000213423	0.499066092	0.390640832	pseudogene	X	140357479	140358349	RBMX2P2

ENSG00000213424	0.499066092	0	protein_coding	17	38810917	38821408	KRT222
ENSG00000213430	6.749949642	6.24096575	pseudogene	5	21882694	21884421	HSPD1P1
ENSG00000213432	0.869158192	2.144285137	pseudogene	10	95043761	95044315	
ENSG00000213433	2.762599152	2.851901313	pseudogene	5	151145420	151145939	
ENSG00000213434	0.499066092	0.697730409	pseudogene	4	184404643	184405338	
ENSG00000213438	1.16343121	0.950786998	pseudogene	10	125751426	125752185	
ENSG00000213440	0	0.390640832	pseudogene	21	45466228	45466922	H2AFZP1
ENSG00000213442	5.729976786	6.35497494	pseudogene	12	104659056	104659669	RPL18AP3
ENSG00000213443	2.57521082	2.420525079	pseudogene	12	9847980	9848823	NPM1P5
ENSG00000213445	9.604394344	9.499262492	protein_coding	11	65405568	65418401	SIPA1
ENSG00000213448	0.499066092	0.390640832	pseudogene	4	138369241	138369655	
ENSG00000213450	0	0.390640832	pseudogene	3	77365903	77366749	
ENSG00000213452	0.499066092	0.697730409	pseudogene	3	74185110	74186043	AKR1B1P2
ENSG00000213453	2.762599152	3.679994897	pseudogene	2	27615651	27616234	FTH1P3
ENSG00000213461	0	0.697730409	pseudogene	6	41275904	41276303	
ENSG00000213462	6.320845526	6.204928885	protein_coding	7	64451187	64467121	ERV3-1
ENSG00000213463	7.639400234	7.797107849	protein_coding	14	70838148	70883778	SYNJ2BP
ENSG00000213465	8.825155121	9.592867142	protein_coding	11	64781585	64789656	ARL2
ENSG00000213467	0	0.390640832	pseudogene	9	124282101	124282730	HMGB1P37
ENSG00000213468	4.026095388	3.87606762	processed_transc	X	130836678	130964671	
ENSG00000213471	0.499066092	0	protein_coding	15	90792762	90808199	TTLL13
ENSG00000213478	2.10647801	1.667587519	pseudogene	1	51623460	51624143	CFL1P2
ENSG00000213480	0.499066092	0	pseudogene	4	122290588	122291500	
ENSG00000213483	0	0.697730409	pseudogene	7	93474101	93474643	
ENSG00000213492	2.35979773	2.420525079	pseudogene	4	118495667	118497329	
ENSG00000213493	1.407729925	0.950786998	pseudogene	4	117519317	117520767	
ENSG00000213498	0.869158192	0.390640832	pseudogene	X	120997551	120998049	
ENSG00000213500	0	0.390640832	pseudogene	6	36641594	36643047	LAP3P2
ENSG00000213509	1.616589159	1.16600992	pseudogene	3	57927545	57928031	PPIAP16
ENSG00000213513	2.471521042	2.144285137	pseudogene	10	79540095	79541634	
ENSG00000213514	1.407729925	0.697730409	pseudogene	10	79490524	79494527	
ENSG00000213516	7.709444959	7.56568256	protein_coding	1	89445139	89458643	RBMXL1
ENSG00000213519	0.499066092	1.16600992	pseudogene	3	197307215	197307500	
ENSG00000213523	8.663981856	8.607443034	protein_coding	5	139929752	139937895	SRA1
ENSG00000213526	0.499066092	1.16600992	pseudogene	X	116097017	116097644	SETP8
ENSG00000213529	0.499066092	1.16600992	pseudogene	9	115124994	115125396	
ENSG00000213533	3.744048221	3.845183986	protein_coding	3	52870235	52931612	TMEM110
ENSG00000213538	1.616589159	1.518964905	pseudogene	11	9115910	9117737	KRT8P41
ENSG00000213539	1.407729925	2.242360793	pseudogene	9	112295156	112296531	YBX1P6
ENSG00000213540	0.499066092	0.390640832	pseudogene	1	84551424	84551916	
ENSG00000213542	2.10647801	1.925536307	pseudogene	7	76744716	76745509	
ENSG00000213549	0	0.950786998	pseudogene	7	75742344	75742718	
ENSG00000213551	6.305850085	6.343971006	protein_coding	10	74943120	75008620	DNAJC9
ENSG00000213553	3.830412367	3.845183986	pseudogene	2	38708920	38710012	RPLP0P6
ENSG00000213558	0	0.390640832	pseudogene	9	110220319	110220587	HMGN2P32
ENSG00000213559	0	0.390640832	pseudogene	1	80916767	80917690	
ENSG00000213563	7.527512071	7.758263791	protein_coding	8	145751117	145754516	C8orf82
ENSG00000213569	0.499066092	0	pseudogene	X	106374887	106375486	
ENSG00000213574	2.762599152	3.133298822	pseudogene	10	120692186	120692807	LDHAP5
ENSG00000213579	0.869158192	0	pseudogene	1	76047784	76048240	
ENSG00000213585	11.35174487	11.11318624	protein_coding	5	133307606	133340824	VDAC1
ENSG00000213587	0	0.390640832	pseudogene	3	50756913	50757382	
ENSG00000213588	7.881135284	7.905863867	protein_coding	6	33422356	33425325	ZBTB9
ENSG00000213590	1.407729925	1.925536307	pseudogene	9	139830292	139831613	
ENSG00000213592	1.16343121	1.802319292	pseudogene	11	57485516	57486002	
ENSG00000213593	9.252165193	9.541415338	protein_coding	11	57480072	57508445	TMX2
ENSG00000213594	0	0.390640832	pseudogene	2	38512552	38513552	GAPDHP25
ENSG00000213598	0.869158192	1.518964905	pseudogene	14	59261372	59261747	
ENSG00000213599	1.616589159	0.697730409	processed_transc	16	30205213	30215631	SULT1A3
ENSG00000213600	1.16343121	0.950786998	pseudogene	3	50277054	50277416	
ENSG00000213601	0.499066092	0.697730409	pseudogene	2	190175989	190176693	KRT18P19
ENSG00000213608	1.16343121	0	pseudogene	4	84398674	84399568	
ENSG00000213609	1.799000381	1.667587519	pseudogene	10	65662211	65663005	

ENSG00000213612	2.10647801	2.420525079	pseudogene	9	99883941	99884713	
ENSG00000213613	2.671945279	2.851901313	pseudogene	10	89705259	89705781	
ENSG00000213614	7.337827682	7.575124073	protein_coding	15	72635775	72668817	HEXA
ENSG00000213619	8.829093615	9.483053775	protein_coding	11	47586888	47606114	NDUFS3
ENSG00000213621	1.616589159	2.501982735	pseudogene	13	21535449	21536334	RPSAP54
ENSG00000213625	8.734271811	9.084889332	protein_coding	1	65886270	65901690	LEPROT
ENSG00000213626	4.689181911	3.081239798	protein_coding	2	30454397	30546596	LBH
ENSG00000213638	6.421663777	7.00911166	protein_coding	19	1905417	1913444	ADAT3
ENSG00000213639	11.10772284	10.84606359	protein_coding	2	28974506	29025806	PPP1CB
ENSG00000213640	0	1.667587519	pseudogene	7	64323377	64324748	
ENSG00000213641	0.869158192	1.16600992	pseudogene	14	56010119	56010887	
ENSG00000213642	1.407729925	2.242360793	pseudogene	7	64029806	64030815	
ENSG00000213643	0	0.390640832	pseudogene	7	63610063	63610810	VN1R37P
ENSG00000213644	0.499066092	0	pseudogene	7	63642088	63643271	
ENSG00000213645	0	0.390640832	pseudogene	7	63391519	63392346	
ENSG00000213650	1.407729925	0	pseudogene	7	56635599	56636551	
ENSG00000213652	0.499066092	0	pseudogene	X	111933143	111933748	HMGB3P30
ENSG00000213654	7.190493718	7.778838337	protein_coding	6	32158543	32163300	GPSM3
ENSG00000213658	1.16343121	1.16600992	protein_coding	16	28996147	29002104	LAT
ENSG00000213661	0	0.390640832	pseudogene	5	120951942	120952502	
ENSG00000213669	0	0.390640832	pseudogene	9	95439225	95439689	
ENSG00000213671	0	0.697730409	pseudogene	17	20678923	20680213	
ENSG00000213672	8.732871027	9.28800787	protein_coding	3	48701364	48723797	NCKIPSD
ENSG00000213673	0	0.390640832	pseudogene	7	54487137	54488021	
ENSG00000213676	9.319971717	9.388489822	protein_coding	6	32065907	32096030	ATF6B
ENSG00000213683	0	1.16600992	pseudogene	22	51193096	51193862	
ENSG00000213684	0.869158192	0.697730409	pseudogene	X	75555240	75556244	LDHBP2
ENSG00000213688	2.10647801	2.851901313	processed_transc	17	18566485	18585572	FOXO3B
ENSG00000213689	6.509364082	6.587342979	protein_coding	3	48506445	48509044	TREX1
ENSG00000213693	3.603959378	3.279072565	pseudogene	11	43919006	43921186	
ENSG00000213694	7.913645586	7.577474828	protein_coding	9	91606362	91619925	S1PR3
ENSG00000213695	2.10647801	1.925536307	pseudogene	X	73629353	73629935	RPS7P14
ENSG00000213697	0.499066092	0	pseudogene	11	43543586	43544814	
ENSG00000213698	0	0.390640832	pseudogene	X	71947802	71948646	
ENSG00000213699	10.24214716	9.992687904	protein_coding	2	26987152	27004099	C2orf18
ENSG00000213700	2.10647801	2.144285137	pseudogene	10	74765591	74766353	
ENSG00000213701	2.57521082	2.144285137	pseudogene	5	96392106	96393062	SETP22
ENSG00000213703	0	0.390640832	pseudogene	1	63106793	63107338	
ENSG00000213704	0	0.697730409	pseudogene	X	97644508	97645918	EEF1A1P15
ENSG00000213706	0	0.950786998	pseudogene	X	70572268	70572838	
ENSG00000213707	0.869158192	0.697730409	pseudogene	22	26956492	26957054	HMGB1P10
ENSG00000213711	0.499066092	0.390640832	pseudogene	9	88513818	88514631	PHBP7
ENSG00000213713	5.806547963	5.583678991	pseudogene	11	33097112	33098006	PIGCP1
ENSG00000213714	2.10647801	2.420525079	protein_coding	20	55108302	55111576	FAM209B
ENSG00000213717	0.499066092	0	pseudogene	7	43313758	43314181	
ENSG00000213719	11.89289638	12.23887067	protein_coding	6	31698358	31707540	CLIC1
ENSG00000213721	0.499066092	0	pseudogene	7	42152964	42153219	HMGN2P30
ENSG00000213722	8.066039442	8.64266806	protein_coding	6	31694815	31698394	DDAH2
ENSG00000213726	0.869158192	0.390640832	pseudogene	19	690381	691175	
ENSG00000213731	0.499066092	0.697730409	pseudogene	10	76183193	76183772	
ENSG00000213736	0	0.390640832	pseudogene	12	25120061	25120438	
ENSG00000213740	2.10647801	0.697730409	pseudogene	X	68003315	68004909	
ENSG00000213741	10.71728035	11.53422571	protein_coding	14	50043390	50065408	RPS29
ENSG00000213742	6.121171528	6.59664425	antisense	20	25604681	25658710	
ENSG00000213744	0	0.390640832	pseudogene	7	37415125	37415626	RPS10P14
ENSG00000213750	0.869158192	0.697730409	pseudogene	8	98636431	98637307	
ENSG00000213753	9.399818301	9.32205174	pseudogene	19	59084870	59111168	
ENSG00000213755	0.499066092	0.390640832	pseudogene	5	78806257	78806742	
ENSG00000213757	2.35979773	2.334191469	pseudogene	5	78579955	78580741	
ENSG00000213760	2.10647801	3.493416095	protein_coding	6	31512239	31516204	ATP6V1G2
ENSG00000213761	1.407729925	1.16600992	pseudogene	9	98175401	98175799	MT1P1
ENSG00000213762	8.217949183	7.858322159	protein_coding	19	58125619	58134721	ZNF134
ENSG00000213763	4.131913373	3.232099092	pseudogene	5	77080705	77081827	

ENSG00000213770	0	0.697730409	pseudogene	10	27638646	27639219	
ENSG00000213772	0.869158192	1.353254395	pseudogene	2	9411421	9411757	
ENSG00000213777	1.799000381	2.039052734	pseudogene	19	54006646	54007532	
ENSG00000213779	1.799000381	1.16600992	pseudogene	11	17215036	17215855	
ENSG00000213780	2.847891871	3.87606762	protein_coding	6	30875961	30881883	GTF2H4
ENSG00000213782	5.785082014	4.932754837	protein_coding	12	12966250	12982915	DDX47
ENSG00000213785	0	1.518964905	pseudogene	11	16505631	16506218	
ENSG00000213786	0	0.390640832	pseudogene	7	27087788	27088247	NHP2P2
ENSG00000213787	0	0.697730409	pseudogene	7	26961729	26962531	RPL7AP38
ENSG00000213790	1.407729925	0.390640832	pseudogene	22	42503769	42504957	
ENSG00000213791	0.499066092	0.390640832	pseudogene	8	81213104	81213716	
ENSG00000213793	0.499066092	0.697730409	protein_coding	19	53418450	53421126	
ENSG00000213798	0.499066092	0.390640832	pseudogene	7	25251789	25252584	
ENSG00000213799	8.094677541	7.694718794	protein_coding	19	53837002	53858122	ZNF845
ENSG00000213809	0	1.353254395	protein_coding	12	10524952	10544473	KLRK1
ENSG00000213820	1.799000381	2.242360793	pseudogene	20	44728157	44728815	RPL13P2
ENSG00000213828	0.499066092	0	protein_coding	2	240083493	240084053	
ENSG00000213830	1.16343121	0	pseudogene	5	68609198	68609699	CFL1P5
ENSG00000213839	5.255381949	5.248158938	pseudogene	9	37885680	37886610	
ENSG00000213842	0.869158192	1.353254395	pseudogene	3	32794402	32795393	SUGT1P2
ENSG00000213846	3.652182994	3.411459265	pseudogene	3	27674467	27675211	
ENSG00000213849	0	0.697730409	pseudogene	3	32549447	32549817	
ENSG00000213851	2.10647801	1.518964905	pseudogene	4	43412058	43412954	
ENSG00000213853	9.787584866	9.140798547	protein_coding	16	10622279	10674555	EMP2
ENSG00000213857	1.799000381	1.16600992	pseudogene	22	41470184	41471243	
ENSG00000213859	8.658089807	9.71586779	protein_coding	17	7255208	7258258	KCTD11
ENSG00000213860	0	0.697730409	pseudogene	7	20042346	20042909	RPL21P28
ENSG00000213862	1.960915222	2.788380093	pseudogene	15	48022341	48023132	
ENSG00000213863	0	1.353254395	lincRNA	6	171034285	171037576	
ENSG00000213864	1.407729925	0.950786998	pseudogene	5	67455046	67455721	EEF1B2P2
ENSG00000213865	4.409500985	4.101879561	protein_coding	8	67579850	67597799	C8orf44
ENSG00000213866	2.35979773	3.232099092	pseudogene	9	35971341	35972315	YBX1P10
ENSG00000213867	1.407729925	0.950786998	pseudogene	14	31290625	31291049	
ENSG00000213871	0.499066092	0	pseudogene	3	25796416	25797161	TAF9BP1
ENSG00000213872	1.16343121	0.697730409	pseudogene	3	25790979	25791387	
ENSG00000213875	1.407729925	1.518964905	pseudogene	7	13911481	13912138	
ENSG00000213880	0	0.697730409	pseudogene	6	29770972	29771768	RPL7AP7
ENSG00000213881	2.847891871	3.453019579	pseudogene	8	62114909	62115779	NPM1P6
ENSG00000213885	2.10647801	2.144285137	pseudogene	21	26734135	26734745	RPL13AP7
ENSG00000213886	0.499066092	0.390640832	protein_coding	6	29523292	29527702	UBD
ENSG00000213888	4.026095388	4.448594745	protein_coding	22	31742875	31744670	
ENSG00000213889	3.393122761	3.714580548	protein_coding	19	45992035	46005768	PPM1N
ENSG00000213891	1.616589159	1.925536307	pseudogene	5	60686495	60687698	RPL3P6
ENSG00000213892	1.16343121	1.16600992	protein_coding	19	45202492	45213986	CEACAM16
ENSG00000213901	5.360713447	5.901455548	protein_coding	2	219940051	220035549	SLC23A3
ENSG00000213903	8.185546801	8.682523569	protein_coding	14	24780656	24787242	LTB4R
ENSG00000213904	3.004694206	2.721932731	protein_coding	19	42928421	43030020	
ENSG00000213906	3.989024711	4.676215056	protein_coding	14	24774940	24781259	LTB4R2
ENSG00000213917	2.762599152	3.993387124	pseudogene	2	215145362	215146176	
ENSG00000213918	6.554468172	6.771086752	protein_coding	16	3692939	3713727	DNASE1
ENSG00000213920	2.762599152	3.133298822	protein_coding	14	24681945	24685276	MDP1
ENSG00000213923	11.16778958	10.70267945	protein_coding	22	38686697	38794527	CSNK1E
ENSG00000213925	0.869158192	1.353254395	pseudogene	2	204637354	204638225	
ENSG00000213928	5.346128795	6.130036786	protein_coding	14	24630262	24635774	IRF9
ENSG00000213930	6.442380648	6.99862477	protein_coding	9	34638130	34651032	GALT
ENSG00000213931	2.471521042	1.353254395	protein_coding	11	5289582	5526847	HBE1
ENSG00000213937	4.642199401	4.075511708	protein_coding	16	3062457	3064506	CLDN9
ENSG00000213938	0.869158192	0.390640832	pseudogene	2	200524274	200525452	SEPHS1P6
ENSG00000213939	1.960915222	2.039052734	pseudogene	17	4608111	4608512	
ENSG00000213940	0	0.390640832	pseudogene	5	52502391	52503006	
ENSG00000213942	0	1.518964905	pseudogene	12	6518803	6519179	
ENSG00000213943	0.499066092	0.697730409	pseudogene	3	12828892	12830170	KRT18P17
ENSG00000213946	0	0.390640832	pseudogene	2	192745908	192746931	DNAJB1P1

ENSG00000213949	6.851544845	7.909600377	protein_coding	5	52083730	52252327	ITGA1
ENSG00000213950	0.499066092	1.353254395	pseudogene	20	14738209	14738702	RPS10P2
ENSG00000213953	0.499066092	0.390640832	protein_coding	2	187361840	187365393	
ENSG00000213958	0	0.390640832	pseudogene	2	182825939	182827205	KRT18P29
ENSG00000213959	1.16343121	0.950786998	pseudogene	20	37243556	37244119	
ENSG00000213962	0	0.697730409	pseudogene	2	178862000	178863497	API5P2
ENSG00000213963	3.78787645	4.319727133	processed_transc	2	178148236	178257419	
ENSG00000213965	7.549958459	7.301328834	protein_coding	19	33182867	33204701	NUDT19
ENSG00000213967	6.676071387	6.628734426	protein_coding	19	24097678	24127961	ZNF726
ENSG00000213970	1.16343121	0.390640832	pseudogene	12	4433538	4433977	
ENSG00000213971	5.785082014	5.6022688	protein_coding	19	23442763	23445436	
ENSG00000213972	0.499066092	0	pseudogene	6	25272429	25273976	
ENSG00000213976	1.16343121	2.788380093	pseudogene	19	21566730	21566877	
ENSG00000213977	6.449220685	7.249200135	protein_coding	17	3566196	3571976	TAX1BP3
ENSG00000213979	0	0.390640832	pseudogene	20	36073135	36073936	RPL7AP14
ENSG00000213983	8.708847212	8.886830358	protein_coding	14	24028774	24037279	AP1G2
ENSG00000213985	0.869158192	1.667587519	pseudogene	19	20368529	20370227	
ENSG00000213987	0	0.390640832	pseudogene	1	41730220	41730534	
ENSG00000213988	7.983632192	7.959118381	protein_coding	19	20188803	20237885	ZNF90
ENSG00000213993	1.407729925	1.16600992	protein_coding	19	19550811	19553580	
ENSG00000213994	0.499066092	0.950786998	antisense	10	6239575	6244656	
ENSG00000213995	7.759833327	7.63953919	protein_coding	13	111267881	111292340	CARKD
ENSG00000213996	0	0.390640832	protein_coding	19	19375174	19384074	TM6SF2
ENSG00000213997	1.407729925	1.353254395	pseudogene	X	46506029	46506765	PGAM1P7
ENSG00000213999	4.026095388	4.101879561	protein_coding	19	19256377	19303400	MEF2B
ENSG00000214006	0.499066092	1.353254395	pseudogene	9	32556904	32557921	
ENSG00000214009	0.499066092	0.390640832	pseudogene	X	45918186	45918963	
ENSG00000214012	0.499066092	0	pseudogene	6	19612986	19614267	KRT18P38
ENSG00000214013	6.428602508	6.57327766	protein_coding	15	42565431	42645864	GANC
ENSG00000214014	4.800350936	5.784235782	protein_coding	17	1945277	1946479	OVCA2
ENSG00000214016	1.16343121	1.802319292	pseudogene	X	44600333	44601210	
ENSG00000214018	1.799000381	0.697730409	pseudogene	X	44168560	44169722	RRM2P3
ENSG00000214019	2.847891871	3.183544561	pseudogene	X	43888578	43890167	
ENSG00000214021	6.050961887	6.149127429	protein_coding	3	9849770	9896822	TTLL3
ENSG00000214022	10.84854275	10.97201656	protein_coding	7	150065278	150071133	REPIN1
ENSG00000214026	8.207901394	8.509895614	protein_coding	11	1968508	2005752	MRPL23
ENSG00000214027	0.499066092	0	pseudogene	6	15935013	15935546	ARPC3P5
ENSG00000214029	6.086493765	6.037446454	protein_coding	12	133694740	133707059	ZNF891
ENSG00000214041	0.499066092	0.697730409	pseudogene	3	9390071	9390952	PGAM1P4
ENSG00000214046	9.520012288	9.373687408	protein_coding	19	16741575	16770955	C19orf42
ENSG00000214049	9.132969064	4.385599336	non_coding	19	15939757	15947130	UCA1
ENSG00000214050	4.352265886	4.65855978	protein_coding	8	28205804	28347835	FBXO16
ENSG00000214051	0.499066092	0	pseudogene	13	101408805	101409313	ARF4P3
ENSG00000214062	0.499066092	0.390640832	pseudogene	4	78003556	78004279	
ENSG00000214063	9.519199923	9.488685117	protein_coding	11	842808	867116	TSPAN4
ENSG00000214067	0.499066092	0.390640832	pseudogene	10	112016822	112017601	
ENSG00000214070	0	0.390640832	pseudogene	2	136655525	136656548	
ENSG00000214076	6.032862302	5.886365696	pseudogene	22	32665368	32669097	CPSF1P1
ENSG00000214077	0	0.390640832	pseudogene	2	132181370	132182440	GNAQP1
ENSG00000214078	11.10474761	11.53966118	protein_coding	20	34213953	34252878	CPNE1
ENSG00000214081	0.499066092	0	pseudogene	2	131437623	131449264	CYP4F30P
ENSG00000214087	6.788216822	6.485878328	protein_coding	17	79648250	79650954	ARL16
ENSG00000214089	0.869158192	0.697730409	pseudogene	10	44910097	44910601	
ENSG00000214093	1.799000381	2.242360793	pseudogene	22	31955469	31955925	
ENSG00000214097	0.499066092	0	protein_coding	3	196233748	196242237	C3orf43
ENSG00000214102	0	0.390640832	protein_coding	7	141408153	141431071	WEE2
ENSG00000214106	5.729976786	5.638744877	protein_coding	7	154720208	154741618	
ENSG00000214108	1.407729925	1.16600992	pseudogene	5	31908467	31909292	
ENSG00000214109	0	1.16600992	protein_coding	19	13890884	13900972	
ENSG00000214110	3.78787645	4.342020395	pseudogene	9	14921335	14922332	LDHAP4
ENSG00000214111	0.869158192	1.16600992	pseudogene	X	25047662	25048352	
ENSG00000214112	4.261916566	4.7279204	protein_coding	17	77015291	77023683	
ENSG00000214113	8.112022537	8.455978641	protein_coding	6	5108653	5261172	LYRM4

ENSG00000214114	7.363400307	7.222412001	protein_coding	1	39328636	39347289	MYCBP
ENSG00000214117	0.869158192	0.950786998	protein_coding	7	139073926	139077714	
ENSG00000214121	0.869158192	0.390640832	pseudogene	9	12972847	12973438	
ENSG00000214125	0	0.390640832	pseudogene	22	31551952	31552633	
ENSG00000214128	0.499066092	0	protein_coding	7	138482695	138522846	TMEM213
ENSG00000214132	0	0.390640832	pseudogene	5	20304154	20305557	
ENSG00000214135	6.462804234	6.515603638	pseudogene	3	197305084	197354777	
ENSG00000214140	1.16343121	1.667587519	protein_coding	17	74523871	74541458	PRCD
ENSG00000214142	0	0.390640832	pseudogene	7	99737252	99738305	
ENSG00000214144	0.499066092	0.390640832	pseudogene	1	247393242	247395180	
ENSG00000214145	4.842525946	4.385599336	lincRNA	3	194014254	194030592	
ENSG00000214146	0.869158192	1.353254395	processed_transc	3	193675161	193721448	
ENSG00000214160	10.08742937	10.25489893	protein_coding	3	183960089	183967336	ALG3
ENSG00000214161	0	0.390640832	pseudogene	22	30877298	30877898	SDC4P
ENSG00000214174	5.498982484	6.204928885	pseudogene	17	62962668	62971694	AMZ2P1
ENSG00000214176	4.800350936	5.03207928	protein_coding	17	62796901	62833243	
ENSG00000214182	3.146112541	2.788380093	pseudogene	13	82264203	82264535	PTMAP5
ENSG00000214184	2.57521082	2.721932731	antisense	2	109123971	109150652	
ENSG00000214185	2.671945279	2.334191469	pseudogene	20	32801301	32803698	XPOTP1
ENSG00000214188	1.407729925	1.667587519	protein_coding	7	116593953	116738860	ST7-OT4
ENSG00000214189	1.616589159	3.081239798	protein_coding	19	12203078	12225499	ZNF788
ENSG00000214190	0.499066092	1.518964905	pseudogene	9	5418373	5418976	
ENSG00000214192	1.407729925	0.390640832	pseudogene	3	175436019	175436458	UBE2V1P2
ENSG00000214193	6.917151646	7.646273261	protein_coding	1	36771988	36790484	SH3D21
ENSG00000214194	7.853042529	8.086718736	protein_coding	7	112756773	112758668	
ENSG00000214195	0.499066092	0.697730409	pseudogene	9	5311444	5311711	HMGN2P31
ENSG00000214198	2.35979773	3.232099092	pseudogene	12	104237527	104323989	
ENSG00000214199	3.502389126	3.679994897	pseudogene	2	107313787	107315132	EEF1A1P12
ENSG00000214200	0.499066092	0	pseudogene	20	32500129	32501142	TPM3P2
ENSG00000214203	0.869158192	0.950786998	pseudogene	12	100402830	100403616	
ENSG00000214207	1.616589159	0.390640832	pseudogene	Y	5441186	5442472	KRT18P10
ENSG00000214212	3.652182994	3.845183986	protein_coding	19	10959106	10980360	C19orf38
ENSG00000214222	1.16343121	0.390640832	pseudogene	13	41958909	41959319	TUBBP2
ENSG00000214223	3.393122761	3.493416095	pseudogene	19	11776884	11777845	HNRNPA1P10
ENSG00000214226	5.472368541	5.98174124	protein_coding	17	54869274	54916134	C17orf67
ENSG00000214239	1.16343121	0.950786998	protein_coding	6	159047471	159049322	
ENSG00000214243	0.499066092	1.16600992	pseudogene	7	76279718	76280214	
ENSG00000214248	1.616589159	0.950786998	protein_coding	19	7963689	7968427	
ENSG00000214249	2.928422289	3.027231696	pseudogene	13	75812080	75814432	CTAGE11P
ENSG00000214253	9.408618364	9.534777296	protein_coding	7	100882739	100895597	FIS1
ENSG00000214255	0.499066092	0	pseudogene	18	68096396	68098361	
ENSG00000214262	6.26766507	5.863430791	protein_coding	1	168214803	168216581	ANKRD36BP1
ENSG00000214264	0.869158192	0	pseudogene	11	112051496	112053914	
ENSG00000214265	3.004694206	3.532712221	protein_coding	15	25200135	25245423	SNURF
ENSG00000214269	4.097495944	3.453019579	pseudogene	13	65532229	65533528	LGMNP1
ENSG00000214273	0.869158192	2.039052734	pseudogene	4	190962497	190964569	AGGF1P1
ENSG00000214274	4.062237333	4.640685763	protein_coding	14	21152336	21167130	ANG
ENSG00000214278	0	0.390640832	pseudogene	5	403846	404661	
ENSG00000214279	7.834876234	8.593573257	protein_coding	10	135267432	135337062	
ENSG00000214280	0.499066092	1.518964905	pseudogene	3	139301770	139302435	
ENSG00000214281	0	0.390640832	pseudogene	13	59584508	59584740	HMGN2P39
ENSG00000214282	0.869158192	0.950786998	pseudogene	X	45491537	45492978	KRT8P14
ENSG00000214283	0	0.697730409	pseudogene	3	136617918	136618918	
ENSG00000214289	1.407729925	0.697730409	pseudogene	3	134070694	134075403	RPL39P5
ENSG00000214290	1.407729925	0	protein_coding	11	111169565	111179460	C11orf93
ENSG00000214293	6.681890601	6.223059835	processed_transc	7	77286977	77325582	
ENSG00000214294	0.499066092	0.950786998	protein_coding	10	127445352	127445665	
ENSG00000214297	2.10647801	1.518964905	pseudogene	10	127355444	127356519	ALDOAP2
ENSG00000214298	0	0.390640832	pseudogene	10	126855246	126855506	
ENSG00000214300	5.83815965	5.200150955	protein_coding	7	99905325	99919819	SPDYE3
ENSG00000214309	3.698846687	4.468996429	protein_coding	7	99724317	99726118	MBLAC1
ENSG00000214313	0.499066092	0.950786998	pseudogene	7	99578385	99585158	AZGP1P1
ENSG00000214318	1.799000381	0	pseudogene	18	61164222	61164633	ATP5G1P6

ENSG00000214321	0.499066092	0	pseudogene	X	17300760	17301216	CBX1P4
ENSG00000214331	4.292665995	4.528525526	pseudogene	16	74366300	74402138	
ENSG00000214335	0	0.390640832	pseudogene	13	59102749	59105160	CTAGE16P
ENSG00000214336	1.616589159	0.950786998	pseudogene	2	88747726	88752131	
ENSG00000214338	0.499066092	1.518964905	protein_coding	6	127759551	127840500	C6orf174
ENSG00000214347	0.499066092	0	pseudogene	19	6393820	6412349	
ENSG00000214353	3.911897206	3.906303962	antisense	16	70789001	70807154	
ENSG00000214354	0.499066092	0	pseudogene	2	87865348	87866283	
ENSG00000214357	4.842525946	4.841813558	protein_coding	5	172068269	172118543	NEURL1B
ENSG00000214359	0	0.697730409	pseudogene	13	50673467	50674004	RPL18P10
ENSG00000214360	0	1.353254395	protein_coding	5	171621176	171630456	EFCAB9
ENSG00000214362	1.799000381	2.912743273	pseudogene	10	97354355	97355129	
ENSG00000214367	7.102117104	7.405556541	protein_coding	4	2229191	2243891	HAUS3
ENSG00000214374	0	0.390640832	pseudogene	6	27932953	27933292	RPLP2P1
ENSG00000214376	1.407729925	0.950786998	protein_coding	11	93551398	93583697	VSTM5
ENSG00000214380	0	0.390640832	pseudogene	3	109634823	109635787	
ENSG00000214389	1.799000381	1.667587519	pseudogene	7	98015113	98015896	RPS3AP26
ENSG00000214391	3.448790144	3.411459265	pseudogene	11	90015728	90017340	
ENSG00000214401	1.407729925	2.039052734	protein_coding	17	44270939	44274037	
ENSG00000214402	2.847891871	1.353254395	protein_coding	9	139876356	139880862	LCNL1
ENSG00000214407	0.499066092	0.390640832	lincRNA	3	101659703	101716770	
ENSG00000214413	6.400645076	6.896886088	protein_coding	10	112658488	112679032	BBIP1
ENSG00000214424	0	0.390640832	pseudogene	15	97300217	97301823	FAM149B2
ENSG00000214425	2.57521082	2.788380093	protein_coding	17	43578685	43595264	
ENSG00000214429	1.407729925	0.390640832	pseudogene	2	78639919	78640220	
ENSG00000214432	0.869158192	1.802319292	antisense	15	91565849	91579841	
ENSG00000214433	0.869158192	0	pseudogene	15	90835194	90838915	
ENSG00000214435	5.4452544	6.077857763	protein_coding	10	104629273	104661656	AS3MT
ENSG00000214439	2.847891871	2.334191469	pseudogene	7	76713203	76751433	FAM185BP
ENSG00000214455	6.851544845	7.106738413	pseudogene	13	45964486	45965402	RCN1P2
ENSG00000214456	3.652182994	2.912743273	protein_coding	19	4522546	4535208	PLIN5
ENSG00000214460	0.869158192	0.950786998	pseudogene	6	86137171	86137684	
ENSG00000214465	0.499066092	0	pseudogene	2	26372073	26373600	SMARCE1P6
ENSG00000214479	0	0.390640832	protein_coding	6	83782692	83782854	
ENSG00000214485	1.616589159	0	pseudogene	5	149473860	149474619	
ENSG00000214491	1.407729925	1.518964905	protein_coding	22	30920465	30942669	SEC14L6
ENSG00000214511	1.16343121	0.390640832	protein_coding	12	51347705	51364289	HIGD1C
ENSG00000214513	0.869158192	0.697730409	protein_coding	2	73429386	73439641	NOTO
ENSG00000214514	0.869158192	0	pseudogene	17	39782579	39796451	KRT42P
ENSG00000214517	9.458626558	9.54382162	protein_coding	11	73882144	73965748	PPME1
ENSG00000214525	0.499066092	1.353254395	pseudogene	2	68755373	68756584	
ENSG00000214526	0	0.950786998	pseudogene	22	23664730	23665114	
ENSG00000214530	8.307246212	8.398639372	protein_coding	11	72465774	72505213	STARD10
ENSG00000214533	0.499066092	0.390640832	pseudogene	2	65893829	65894871	
ENSG00000214534	2.238690726	1.925536307	pseudogene	11	71518637	71532594	ZNF705E
ENSG00000214535	2.928422289	3.748356452	pseudogene	20	21146846	21147236	RPS15AP1
ENSG00000214541	0.499066092	0	pseudogene	20	55176757	55177169	
ENSG00000214546	0.869158192	0.950786998	protein_coding	17	37775866	37778766	
ENSG00000214548	0	0.390640832	processed_transc	14	101245747	101327368	MEG3
ENSG00000214552	0.499066092	0.697730409	pseudogene	3	68194154	68194780	
ENSG00000214553	1.407729925	0.390640832	antisense	17	37186159	37201194	
ENSG00000214556	0	0.390640832	protein_coding	17	36991339	36997664	C17orf98
ENSG00000214559	0.499066092	0.390640832	antisense	4	99172839	99182781	
ENSG00000214560	0.499066092	0	pseudogene	3	66687573	66687836	
ENSG00000214561	0	0.390640832	pseudogene	6	58446019	58447307	RBBP4P4
ENSG00000214562	3.335220907	4.585695337	protein_coding	10	89117425	89130452	FAM22D
ENSG00000214575	0.499066092	0.950786998	protein_coding	15	83211951	83317612	CPEB1
ENSG00000214593	0	1.16600992	pseudogene	9	128026124	128026369	
ENSG00000214595	4.261916566	4.15321211	protein_coding	2	54950636	55199157	EML6
ENSG00000214597	2.471521042	2.334191469	protein_coding	8	145575878	145579269	
ENSG00000214612	0.869158192	0.950786998	pseudogene	20	18485277	18485710	RPS19P1
ENSG00000214617	0	0.390640832	pseudogene	16	32888797	32896822	SLC6A10P
ENSG00000214626	0.499066092	0.697730409	pseudogene	10	76414714	76415889	

ENSG00000214628	0.499066092	0.390640832	pseudogene	X	96208638	96209194	
ENSG00000214629	0	0.390640832	pseudogene	10	76131293	76132174	
ENSG00000214650	1.616589159	1.667587519	lincRNA	12	124997768	124999359	
ENSG00000214651	0.499066092	0	pseudogene	9	124000954	124002290	
ENSG00000214652	2.35979773	2.039052734	pseudogene	7	63505821	63539697	
ENSG00000214653	0	0.390640832	pseudogene	X	139114503	139115665	HNRNPA3P3
ENSG00000214654	5.286269488	5.072639111	pseudogene	9	123555829	123561848	
ENSG00000214655	9.379372099	9.594609026	protein_coding	10	75545383	75561551	KIAA0913
ENSG00000214659	2.10647801	1.518964905	pseudogene	11	65494410	65495685	KRT8P26
ENSG00000214671	0.499066092	0.950786998	pseudogene	4	170087423	170088268	RPL6P12
ENSG00000214676	0.869158192	2.039052734	pseudogene	4	169676967	169677533	RPL9P16
ENSG00000214686	0	0.390640832	protein_coding	3	51812580	51813009	IQCF6
ENSG00000214694	3.393122761	3.714580548	protein_coding	2	39117021	39202590	ARHGEF33
ENSG00000214700	0.869158192	0.950786998	protein_coding	12	27233990	27235447	C12orf71
ENSG00000214706	9.794315688	9.97407346	protein_coding	3	50325163	50330349	IFRD2
ENSG00000214708	1.799000381	2.721932731	protein_coding	17	30468245	30470137	
ENSG00000214717	7.983632192	7.834933409	protein_coding	X	2404455	2419008	ZBED1
ENSG00000214719	7.657232808	7.188980716	processed_transc	17	28903526	28964482	LRRC37BP1
ENSG00000214725	3.393122761	3.532712221	processed_transc	16	29874914	29879371	
ENSG00000214733	1.799000381	1.16600992	antisense	8	144871090	144872629	
ENSG00000214748	5.346128795	5.404622322	pseudogene	X	136407131	136407208	
ENSG00000214753	9.282163564	9.166009895	protein_coding	11	62480102	62494821	HNRNPUL2
ENSG00000214756	5.239686603	6.217041467	protein_coding	11	62432781	62435968	METTL12
ENSG00000214761	1.16343121	0.697730409	pseudogene	9	128357948	128358907	HNRNPA1P15
ENSG00000214765	6.75547892	6.729413443	pseudogene	7	45763379	45808617	SEPT7P2
ENSG00000214770	2.847891871	3.781359661	antisense	14	64796149	64805317	
ENSG00000214773	1.799000381	0.697730409	protein_coding	3	47643205	47646167	
ENSG00000214776	4.026095388	4.274079748	pseudogene	12	9620148	9728864	
ENSG00000214780	0.869158192	1.16600992	protein_coding	11	61532328	61535285	
ENSG00000214783	5.889353623	5.545764946	pseudogene	7	43980494	44058774	POLR2J4
ENSG00000214784	1.16343121	1.667587519	pseudogene	5	110527924	110528740	
ENSG00000214796	3.211941663	2.039052734	pseudogene	1	202820956	202830748	
ENSG00000214803	0.499066092	0.390640832	lincRNA	8	125204912	125259639	
ENSG00000214807	1.407729925	0.390640832	pseudogene	10	126556079	126556895	
ENSG00000214810	0.499066092	0.697730409	pseudogene	6	34187216	34187843	CYCSP55
ENSG00000214812	0	0.390640832	pseudogene	1	28446958	28447832	
ENSG00000214814	1.16343121	3.32456471	protein_coding	8	124864227	125132302	FER1L6
ENSG00000214815	3.393122761	2.652276565	pseudogene	7	138125435	138126983	
ENSG00000214820	1.16343121	1.667587519	pseudogene	3	44621430	44622518	
ENSG00000214822	0.499066092	0	pseudogene	17	20404982	20407811	KRT16P3
ENSG00000214823	0	0.390640832	pseudogene	13	39743773	39744088	NXT1P1
ENSG00000214826	6.155035276	6.310446475	pseudogene	12	9570309	9600825	DDX12P
ENSG00000214827	4.352265886	4.42790041	protein_coding	X	154289897	154376212	MTCP1
ENSG00000214832	3.274897671	0.950786998	pseudogene	17	20279020	20280146	
ENSG00000214837	6.228441838	5.953060722	processed_transc	1	243219131	243265046	
ENSG00000214846	0	0.697730409	pseudogene	4	15732585	15733250	
ENSG00000214851	0.869158192	0	processed_transc	12	9208182	9217672	C12orf33
ENSG00000214855	0.499066092	0	protein_coding	19	45430060	45434643	
ENSG00000214860	1.16343121	0.390640832	protein_coding	17	18281079	18292961	EVPLL
ENSG00000214867	1.16343121	0.697730409	pseudogene	21	37667471	37668000	SRSF9P1
ENSG00000214870	2.10647801	1.925536307	processed_transc	7	26438213	26538594	
ENSG00000214872	4.230497448	4.101879561	protein_coding	11	57308979	57318108	SMTNL1
ENSG00000214875	0	0.950786998	pseudogene	10	70750893	70751463	
ENSG00000214881	0.499066092	0	pseudogene	10	70304246	70304590	TMEM14D
ENSG00000214883	1.16343121	0	pseudogene	11	50368215	50381479	
ENSG00000214888	0	0.390640832	antisense	9	90471930	90474314	
ENSG00000214894	1.407729925	2.652276565	processed_transc	6	30766431	30798436	LINC00243
ENSG00000214896	0	0.950786998	pseudogene	15	53177792	53178667	RPSAP55
ENSG00000214897	0	0.390640832	pseudogene	X	152662421	152664307	
ENSG00000214899	2.238690726	2.501982735	protein_coding	17	17072078	17088887	C17orf84
ENSG00000214900	5.752272083	5.09905904	protein_coding	14	50448430	50474238	C14orf182
ENSG00000214917	0	0.697730409	pseudogene	18	29655848	29656235	
ENSG00000214919	0	0.390640832	protein_coding	3	155459933	155461515	

ENSG00000214922	3.211941663	4.274079748	processed_transc	6	29694378	29716826	HLA-F-AS1
ENSG00000214940	0.499066092	0	protein_coding	16	18411799	18441131	
ENSG00000214941	7.066972635	7.591499486	protein_coding	17	15879874	15903031	ZSWIM7
ENSG00000214944	8.913126744	9.363507064	protein_coding	5	72921983	73237818	
ENSG00000214946	2.10647801	2.334191469	protein_coding	17	15635570	15647766	TBC1D26
ENSG00000214954	2.928422289	2.334191469	protein_coding	8	92114060	92231464	LRRC69
ENSG00000214955	1.16343121	0.697730409	processed_transc	21	35445892	35732332	
ENSG00000214960	3.004694206	3.608232228	protein_coding	7	16130817	16460947	ISPD
ENSG00000214967	0.499066092	0	protein_coding	16	16472912	16487811	
ENSG00000214973	0.499066092	0.390640832	pseudogene	1	27527325	27527964	
ENSG00000214975	0.499066092	0	pseudogene	6	24976647	24977210	PPIAP29
ENSG00000214982	3.077135474	3.368666104	pseudogene	10	51623417	51732824	
ENSG00000214988	0	0.390640832	pseudogene	4	83411976	83412788	RPL7AP26
ENSG00000214992	0.499066092	0.950786998	pseudogene	X	118383309	118391633	AKAP16BP
ENSG00000214999	2.35979773	3.608232228	protein_coding	17	7982800	7984883	
ENSG00000215002	0.499066092	0	pseudogene	10	108309864	108310879	
ENSG00000215003	1.407729925	0.697730409	pseudogene	16	16025560	16026153	
ENSG00000215005	0	0.390640832	pseudogene	21	30259602	30261594	HSPD1P7
ENSG00000215006	0	0.390640832	pseudogene	5	68629756	68630076	CHCHD2P2
ENSG00000215007	0	0.697730409	pseudogene	X	106594885	106596073	DNAJA1P3
ENSG00000215009	3.830412367	3.935919592	protein_coding	12	7456880	7481320	ACSM4
ENSG00000215012	8.219950371	8.565427152	protein_coding	22	19833661	19842419	C22orf29
ENSG00000215014	2.10647801	0.697730409	protein_coding	1	1510355	1511373	
ENSG00000215018	3.989024711	3.64455972	protein_coding	7	7395834	7575484	COL28A1
ENSG00000215021	11.1505816	11.10953601	protein_coding	12	7074490	7079988	PHB2
ENSG00000215023	1.407729925	1.667587519	antisense	2	242740331	242741322	
ENSG00000215030	5.879259127	5.863430791	pseudogene	17	17286691	17287326	
ENSG00000215032	1.616589159	0	pseudogene	5	60187762	60189404	GNL3LP1
ENSG00000215033	1.960915222	0.390640832	protein_coding	10	47746962	47770871	
ENSG00000215034	1.960915222	0.390640832	pseudogene	3	39255684	39256163	
ENSG00000215035	0	0.950786998	pseudogene	X	44335273	44336668	FDPSP5
ENSG00000215039	6.749949642	6.815578497	processed_transc	12	6548167	6560733	
ENSG00000215041	9.029515185	9.549819822	protein_coding	17	7218951	7232638	NEURL4
ENSG00000215045	0	0.950786998	protein_coding	7	6537093	6591067	GRID2IP
ENSG00000215049	0	0.390640832	pseudogene	13	30870284	30870872	PRDX2P1
ENSG00000215051	0	0.390640832	pseudogene	18	21289784	21290251	
ENSG00000215057	0.499066092	1.16600992	pseudogene	6	5972645	5974245	
ENSG00000215066	0.499066092	1.802319292	protein_coding	9	6467618	6470375	C9orf38
ENSG00000215067	5.107569429	6.210997888	protein_coding	17	6888442	6915653	
ENSG00000215068	4.568725997	4.448594745	lincRNA	5	43041677	43045492	
ENSG00000215070	0.869158192	0.697730409	pseudogene	X	98716600	98976047	
ENSG00000215086	0	0.697730409	pseudogene	10	74677399	74678292	
ENSG00000215093	2.57521082	2.420525079	pseudogene	X	85415268	85416363	EEF1A1P29
ENSG00000215099	5.672682881	6.387491945	protein_coding	17	5328469	5336196	
ENSG00000215102	0.499066092	0	pseudogene	X	83003833	83004947	TERF1P4
ENSG00000215105	3.393122761	3.411459265	pseudogene	X	74960541	74966749	TTC3P1
ENSG00000215114	8.362640485	8.402622096	protein_coding	8	59323823	59364060	UBXN2B
ENSG00000215120	0	0.697730409	pseudogene	X	70747664	70750075	
ENSG00000215124	0.499066092	0	pseudogene	6	11861859	11863203	
ENSG00000215126	3.004694206	3.183544561	protein_coding	9	42668608	42714962	CBWD7
ENSG00000215131	0.499066092	0.950786998	protein_coding	16	3543484	3545480	C16orf90
ENSG00000215142	0.869158192	0	pseudogene	9	40491622	40633699	
ENSG00000215146	3.211941663	1.353254395	pseudogene	10	42832238	42833689	
ENSG00000215148	0	0.390640832	pseudogene	16	2848397	2855133	
ENSG00000215154	7.04456449	7.309836726	pseudogene	16	2653351	2680495	
ENSG00000215156	1.16343121	1.16600992	pseudogene	5	34190138	34193758	
ENSG00000215158	6.095241663	6.223059835	pseudogene	5	34164803	34244901	
ENSG00000215179	0.869158192	0	pseudogene	8	47884104	47886240	MAPK6PS4
ENSG00000215182	0	0.697730409	protein_coding	11	1151580	1222364	MUC5AC
ENSG00000215184	0.869158192	1.16600992	pseudogene	10	34964563	34964969	
ENSG00000215187	2.35979773	2.721932731	protein_coding	9	35561828	35563896	FAM166B
ENSG00000215190	5.498982484	5.709041716	processed_transc	6	58272352	58287779	GUSBP4
ENSG00000215193	9.599022371	9.418958081	protein_coding	22	18560689	18613905	PEX26

ENSG00000215196	3.004694206	3.368666104	lincRNA	5	17130137	17217508	
ENSG00000215198	0	0.390640832	pseudogene	9	35147407	35149181	
ENSG00000215199	3.744048221	2.420525079	pseudogene	9	34922186	34922918	YWHAZP6
ENSG00000215201	0.869158192	0.390640832	pseudogene	21	33826002	33830768	
ENSG00000215204	0.499066092	0.390640832	protein_coding	9	34830901	34838494	FAM205B
ENSG00000215208	0	1.353254395	pseudogene	12	65812511	65813807	
ENSG00000215210	0.869158192	1.518964905	pseudogene	9	30689103	30690270	RBMXP2
ENSG00000215217	0	0.950786998	protein_coding	5	7830491	7851603	C5orf49
ENSG00000215221	1.407729925	1.16600992	pseudogene	9	22012154	22012535	UBA52P6
ENSG00000215236	0.869158192	0	pseudogene	9	15361391	15362132	
ENSG00000215241	3.211941663	3.993387124	lincRNA	12	8388011	8395160	
ENSG00000215244	2.35979773	1.802319292	protein_coding	10	6319650	6377938	
ENSG00000215246	1.616589159	0.950786998	antisense	5	987295	997423	
ENSG00000215251	9.026088136	9.145559356	protein_coding	20	3127165	3140543	FASTKD5
ENSG00000215252	7.657232808	8.275187599	protein_coding	15	34817408	34880704	GOLGA8B
ENSG00000215256	5.207774174	5.564846512	processed_transc	14	24407940	24458048	C14orf167
ENSG00000215262	0	0.390640832	protein_coding	8	36641842	36793646	KCNU1
ENSG00000215263	0.499066092	0	pseudogene	2	42759906	42760582	
ENSG00000215264	0.869158192	0	pseudogene	8	34180510	34181160	
ENSG00000215267	2.238690726	1.353254395	pseudogene	10	5317136	5330433	
ENSG00000215268	0.499066092	0	pseudogene	22	16413687	16415946	
ENSG00000215270	2.762599152	3.845183986	pseudogene	22	16122720	16123768	
ENSG00000215271	5.91922012	6.1803943	protein_coding	14	23743250	23755326	HOMEZ
ENSG00000215274	0.869158192	0.697730409	protein_coding	X	49160148	49176323	GAGE10
ENSG00000215277	3.004694206	3.027231696	protein_coding	14	23654525	23742686	C14orf164
ENSG00000215283	1.616589159	1.518964905	pseudogene	9	36303496	36304921	HMGB3P24
ENSG00000215284	0	0.697730409	pseudogene	X	46584599	46585253	
ENSG00000215296	0.499066092	0	pseudogene	15	33527753	33539822	TMCO5B
ENSG00000215301	11.8458855	11.83539567	protein_coding	X	41192651	41223725	DDX3X
ENSG00000215302	2.238690726	3.133298822	pseudogene	15	30762982	30799826	
ENSG00000215304	0	0.950786998	processed_transc	15	32691157	32727434	ULK4P1
ENSG00000215305	9.423456371	9.723317502	protein_coding	20	2821349	2847378	VPS16
ENSG00000215306	0.869158192	0.390640832	protein_coding	14	23286463	23291750	
ENSG00000215313	0.869158192	0.950786998	pseudogene	X	16216551	16217412	RPL6P30
ENSG00000215319	1.16343121	1.16600992	pseudogene	X	9786279	9787509	
ENSG00000215325	0	0.390640832	pseudogene	5	144608374	144609617	ASS1P10
ENSG00000215326	0.869158192	1.16600992	pseudogene	21	28515663	28516268	GPX1P2
ENSG00000215333	0.499066092	0.390640832	pseudogene	22	44962845	44964127	KRT18P23
ENSG00000215346	1.16343121	1.518964905	protein_coding	8	10983980	10987745	
ENSG00000215347	1.407729925	2.242360793	pseudogene	22	42397073	42397970	SLC25A5P1
ENSG00000215349	1.16343121	0.390640832	pseudogene	13	19878733	19879765	MRPL3P1
ENSG00000215367	1.616589159	2.652276565	pseudogene	4	1108985	1147514	TMED11P
ENSG00000215374	0	0.390640832	processed_transc	8	7159133	7212876	FAM66B
ENSG00000215375	6.228441838	5.923798455	protein_coding	4	667369	675822	MYL5
ENSG00000215381	1.16343121	0	pseudogene	1	23571258	23571717	
ENSG00000215386	0.499066092	0	processed_transc	21	17442842	17982094	LINC00478
ENSG00000215388	3.502389126	3.183544561	pseudogene	20	1141228	1142341	ACTG1P3
ENSG00000215394	1.960915222	3.87606762	lincRNA	14	19889386	19904890	LINC00516
ENSG00000215409	0.499066092	0	pseudogene	10	27620286	27630181	
ENSG00000215414	1.799000381	1.518964905	pseudogene	Y	15398518	15399258	PSMA6P1
ENSG00000215417	4.437288965	4.15321211	non_coding	13	92000074	92006833	MIR17HG
ENSG00000215421	7.149084042	7.234379288	protein_coding	18	72342919	72777628	ZNF407
ENSG00000215424	5.909333162	5.526427614	processed_transc	21	47649145	47671615	MCM3AP-AS1
ENSG00000215431	0	0.390640832	protein_coding	18	60491423	60492724	
ENSG00000215440	7.035502862	7.210344614	protein_coding	20	57264187	57294294	NPEPL1
ENSG00000215441	0	0.390640832	pseudogene	9	27608385	27610743	CTAGE12P
ENSG00000215447	5.072559152	4.932754837	processed_transc	21	46707967	46711653	
ENSG00000215450	0.499066092	0	pseudogene	20	45789477	45790339	
ENSG00000215452	0.499066092	0.697730409	pseudogene	20	45042867	45087915	
ENSG00000215458	4.689181911	4.468996429	antisense	21	45225639	45232448	
ENSG00000215464	0	0.390640832	pseudogene	22	24665316	24666102	
ENSG00000215467	0	0.390640832	pseudogene	20	42281163	42281931	RPL27AP
ENSG00000215472	9.597483841	10.32827122	protein_coding	18	47014858	47018906	RPL17

ENSG00000215478	0.499066092	0.390640832	pseudogene	22	23701790	23729918	CES5AP1
ENSG00000215481	0.499066092	0.950786998	pseudogene	22	25028882	25046799	BCRP3
ENSG00000215482	0.499066092	0.697730409	pseudogene	13	41744838	41745284	CALM2P3
ENSG00000215483	0	0.697730409	lincRNA	13	41025131	41055143	
ENSG00000215492	3.077135474	2.420525079	pseudogene	18	29991471	29993200	HNRNPA1P7
ENSG00000215493	2.847891871	3.906303962	pseudogene	22	20804409	20806111	
ENSG00000215494	3.950976309	3.232099092	protein_coding	21	43313829	43315499	
ENSG00000215504	2.35979773	1.802319292	protein_coding	11	85563600	85565986	
ENSG00000215513	6.112579918	5.995870484	pseudogene	22	20383524	20427835	PI4KAP1
ENSG00000215515	0.499066092	0.697730409	pseudogene	13	32958797	32960245	IFIT1P1
ENSG00000215527	2.35979773	2.501982735	protein_coding	18	12658205	12658712	
ENSG00000215529	2.762599152	3.453019579	protein_coding	20	31446729	31549006	EFCAB8
ENSG00000215533	0	0.390640832	processed_transc	21	30565801	30660526	LINC00189
ENSG00000215548	3.871730003	4.468996429	pseudogene	9	68427861	68455375	
ENSG00000215559	1.616589159	1.518964905	pseudogene	21	15281895	15352758	ANKRD20A11P
ENSG00000215565	0	0.390640832	pseudogene	11	129952103	129952367	
ENSG00000215568	1.799000381	2.420525079	protein_coding	22	17442826	17489112	GAB4
ENSG00000215570	7.581430769	7.281279771	protein_coding	13	21946717	21949350	
ENSG00000215571	1.799000381	1.802319292	pseudogene	13	21893231	21895005	GRK6P1
ENSG00000215586	1.16343121	1.518964905	pseudogene	20	7812445	7812969	
ENSG00000215595	0.499066092	1.353254395	protein_coding	20	1184098	1188918	C20orf202
ENSG00000215604	0	0.390640832	pseudogene	13	19041312	19059588	ZNF962P
ENSG00000215606	0.499066092	0	pseudogene	3	141189476	141190744	
ENSG00000215621	4.923348194	5.41517489	protein_coding	17	79780293	79784533	
ENSG00000215630	2.471521042	2.788380093	pseudogene	5	70435716	70585611	GUSBP9
ENSG00000215644	3.78787645	3.133298822	protein_coding	17	79762010	79771889	GCGR
ENSG00000215704	0.499066092	0.697730409	protein_coding	1	15792404	15817895	CELA2B
ENSG00000215712	6.298293508	6.766973175	protein_coding	6	157712662	157744633	TMEM242
ENSG00000215717	7.751556534	7.768587741	protein_coding	1	109633357	109639556	TMEM167B
ENSG00000215720	0.499066092	0.697730409	pseudogene	1	15518324	15519341	
ENSG00000215749	0.499066092	0	protein_coding	15	82930438	82939159	
ENSG00000215760	0.499066092	0	pseudogene	10	116569134	116570929	TAF9BP2
ENSG00000215769	5.431503808	6.281901708	protein_coding	17	62745781	62778117	
ENSG00000215771	0.499066092	0.390640832	pseudogene	22	41585731	41586192	
ENSG00000215784	4.568725997	4.448594745	protein_coding	1	143896452	143913143	FAM72D
ENSG00000215785	0	0.390640832	pseudogene	1	11051035	11051854	CFL1P6
ENSG00000215788	7.144876945	6.862599506	protein_coding	1	6521211	6526255	TNFRSF25
ENSG00000215790	4.517578978	4.841813558	protein_coding	1	1656277	1677431	SLC35E2
ENSG00000215795	1.407729925	2.039052734	pseudogene	1	247347115	247348784	
ENSG00000215796	1.407729925	1.518964905	pseudogene	1	247206093	247207323	
ENSG00000215808	0	0.390640832	processed_transc	1	238643684	238649323	
ENSG00000215811	0	0.697730409	protein_coding	1	228698126	228700004	
ENSG00000215812	1.16343121	1.16600992	pseudogene	1	227884501	227894412	ZNF847P
ENSG00000215835	1.407729925	0.950786998	pseudogene	1	166244866	166246834	
ENSG00000215837	6.693459083	7.096940025	pseudogene	3	195384933	195415733	SDHAP2
ENSG00000215838	0	0.390640832	processed_transc	1	165667987	165679205	
ENSG00000215840	1.799000381	1.353254395	pseudogene	1	161375858	161376872	
ENSG00000215841	0.869158192	0	antisense	11	72926043	72926958	
ENSG00000215845	5.879259127	6.304782526	protein_coding	1	161007421	161008780	TSTD1
ENSG00000215857	0.499066092	1.16600992	protein_coding	1	150273648	150277452	
ENSG00000215861	0.499066092	0	pseudogene	1	144676874	144679969	
ENSG00000215863	0	1.667587519	processed_transc	1	147835127	147931933	
ENSG00000215864	1.407729925	1.353254395	pseudogene	1	120377388	120387779	NBPF7
ENSG00000215867	0	1.16600992	pseudogene	1	112190916	112192166	
ENSG00000215873	0	0.697730409	pseudogene	1	91793926	91795070	FEN1P1
ENSG00000215878	0.499066092	0	pseudogene	6	138723726	138724698	
ENSG00000215883	4.491309013	4.932754837	protein_coding	1	54638009	54665709	CYB5RL
ENSG00000215889	0	0.697730409	lincRNA	1	48567387	48627543	SKINTL
ENSG00000215893	0	1.16600992	pseudogene	1	41564308	41564800	
ENSG00000215895	1.616589159	1.802319292	pseudogene	1	39174626	39176564	
ENSG00000215897	0.499066092	0.390640832	protein_coding	1	32930670	32953461	ZBTB8B
ENSG00000215899	3.077135474	3.133298822	pseudogene	1	31960438	31961608	
ENSG00000215900	2.10647801	2.334191469	pseudogene	1	31567839	31568405	SEPW1P

ENSG00000215902	1.407729925	1.16600992	protein_coding	1	28527013	28527152	
ENSG00000215908	8.599344916	8.936206949	processed_transc	1	16944751	16971178	CROCCP2
ENSG00000215910	0.499066092	0.950786998	protein_coding	1	11821844	11849642	C1orf167
ENSG00000215912	1.16343121	1.518964905	protein_coding	1	2567415	2706280	TTC34
ENSG00000215915	4.543379137	4.841813558	protein_coding	1	1385069	1405538	ATAD3C
ENSG00000215946	0	0.697730409	miRNA	20	62551376	62551466	MIR941-1
ENSG00000215972	0	0.697730409	miRNA	10	127531513	127531598	
ENSG00000215973	0	0.950786998	miRNA	2	176032361	176032437	MIR933
ENSG00000215979	0	0.697730409	miRNA	6	26365704	26365780	
ENSG00000216001	0	0.390640832	miRNA	X	133674215	133674292	MIR450B
ENSG00000216047	0	0.390640832	miRNA	X	130538484	130538576	
ENSG00000216101	0	0.697730409	miRNA	6	30552109	30552194	MIR877
ENSG00000216102	0.499066092	0	miRNA	19	9931204	9931281	
ENSG00000216109	0.869158192	0.950786998	miRNA	1	155206540	155206628	
ENSG00000216164	0.869158192	0	miRNA	20	62550733	62550823	
ENSG00000216168	0	0.697730409	miRNA	17	57066229	57066315	
ENSG00000216182	0	0.390640832	miRNA	9	119155868	119155955	
ENSG00000216195	0	0.390640832	miRNA	20	62551096	62551186	MIR941-2
ENSG00000216285	0	0.950786998	pseudogene	12	104424557	104425321	
ENSG00000216307	0	0.390640832	pseudogene	6	4189160	4190040	
ENSG00000216316	0.499066092	1.16600992	pseudogene	6	119590298	119590879	
ENSG00000216331	2.35979773	1.518964905	pseudogene	6	26195794	26195999	HIST1H1PS1
ENSG00000216360	0.499066092	0.950786998	pseudogene	6	5817654	5818599	
ENSG00000216368	1.407729925	0.390640832	pseudogene	6	10118685	10119485	
ENSG00000216378	0	0.390640832	pseudogene	6	100625085	100626068	
ENSG00000216475	0	0.390640832	pseudogene	6	144578170	144578760	
ENSG00000216490	10.06422571	10.56434697	protein_coding	19	18284579	18288927	IFI30
ENSG00000216588	3.274897671	3.993387124	protein_coding	19	45116940	45140081	IGSF23
ENSG00000216616	0.499066092	0	pseudogene	6	47720564	47720976	
ENSG00000216624	1.799000381	1.518964905	pseudogene	6	166477779	166478788	GAPDHP72
ENSG00000216636	0.499066092	1.353254395	pseudogene	6	34584315	34585035	
ENSG00000216639	0.499066092	1.353254395	pseudogene	6	105746024	105746770	
ENSG00000216657	1.16343121	0.697730409	pseudogene	6	3978296	3979333	
ENSG00000216721	0	0.390640832	pseudogene	2	216669454	216669704	
ENSG00000216740	0	0.697730409	pseudogene	10	66585329	66586336	ANXA2P3
ENSG00000216775	3.211941663	3.906303962	pseudogene	6	52530072	52531676	
ENSG00000216802	0	0.390640832	pseudogene	6	138971363	138972110	
ENSG00000216809	0.499066092	0.950786998	pseudogene	6	118773632	118776155	
ENSG00000216813	0	0.950786998	pseudogene	6	47530358	47530754	
ENSG00000216819	0.499066092	0.697730409	pseudogene	6	3177278	3179998	
ENSG00000216854	0.869158192	2.144285137	pseudogene	6	56735989	56736543	
ENSG00000216866	3.004694206	2.579085888	pseudogene	X	40794198	40794969	RPS2P55
ENSG00000216867	0.499066092	1.16600992	pseudogene	2	111391524	111391910	
ENSG00000216895	4.6658819	4.202780776	protein_coding	7	155403925	155437899	
ENSG00000216901	1.799000381	1.802319292	pseudogene	6	28143966	28144452	
ENSG00000216906	1.16343121	0.950786998	pseudogene	6	150225379	150227554	
ENSG00000216917	0.499066092	0	pseudogene	6	132101861	132102452	
ENSG00000216921	0.869158192	0.950786998	protein_coding	2	242836136	242844702	
ENSG00000216937	3.502389126	4.566889334	protein_coding	10	32735041	32863501	CCDC7
ENSG00000216977	0.499066092	0	pseudogene	6	107090338	107090803	
ENSG00000216990	0.869158192	1.353254395	pseudogene	6	88008490	88010165	HSPD1P10
ENSG00000217026	0	1.353254395	pseudogene	21	28792699	28793342	RPL10P1
ENSG00000217027	1.799000381	1.16600992	pseudogene	6	144521584	144522102	
ENSG00000217041	0	0.390640832	pseudogene	6	111021765	111022103	
ENSG00000217060	0	0.390640832	pseudogene	6	85995794	86000567	
ENSG00000217075	1.616589159	0.697730409	protein_coding	2	36758948	36779411	
ENSG00000217078	0	1.518964905	pseudogene	6	16163608	16163993	
ENSG00000217094	1.16343121	0	pseudogene	10	32477251	32477745	
ENSG00000217128	8.12275821	8.327799018	protein_coding	5	130761584	131132756	FNIP1
ENSG00000217130	1.799000381	2.144285137	pseudogene	6	34711953	34712450	
ENSG00000217159	0	0.950786998	pseudogene	6	26164265	26164551	
ENSG00000217165	5.175139844	5.446377228	pseudogene	6	39078097	39080728	ANKRD18EP
ENSG00000217181	0.499066092	0.390640832	pseudogene	6	10574982	10575460	

ENSG00000217227	0.869158192	0.390640832	pseudogene	6	38555099	38556305	
ENSG00000217239	1.16343121	1.518964905	pseudogene	6	5788579	5788932	
ENSG00000217241	1.407729925	0.950786998	pseudogene	6	116774177	116774728	CBX3P9
ENSG00000217258	1.16343121	1.353254395	processed_transc	2	10592445	10595678	
ENSG00000217275	2.471521042	1.518964905	pseudogene	6	26202384	26202881	RPS10P1
ENSG00000217315	0	0.390640832	pseudogene	6	28001725	28002633	OR2W2P
ENSG00000217325	2.847891871	2.971122874	pseudogene	6	126964634	126965536	
ENSG00000217327	0.499066092	0	pseudogene	1	240175946	240176526	RPS7P5
ENSG00000217334	0	0.697730409	pseudogene	6	86441089	86441729	
ENSG00000217340	0	0.950786998	protein_coding	6	17878685	17882609	
ENSG00000217372	0.499066092	0	pseudogene	6	153219182	153220415	
ENSG00000217385	0.499066092	0	pseudogene	6	4702697	4704013	PSMC1P11
ENSG00000217442	3.146112541	3.279072565	protein_coding	22	50989541	51001334	SYCE3
ENSG00000217447	0.499066092	1.353254395	pseudogene	6	167760960	167761541	
ENSG00000217488	0.499066092	0.950786998	pseudogene	6	76320674	76321661	
ENSG00000217495	0	0.390640832	pseudogene	6	143619907	143620639	
ENSG00000217512	0	0.697730409	pseudogene	6	79777409	79777855	
ENSG00000217527	0.499066092	0.390640832	pseudogene	6	53201741	53202144	
ENSG00000217555	6.421663777	7.337839373	protein_coding	16	66586466	66601967	CKLF
ENSG00000217576	3.989024711	3.714580548	processed_transc	13	52741844	52908412	THSD1P1
ENSG00000217612	0	0.390640832	pseudogene	6	145810766	145813228	
ENSG00000217624	0.499066092	0	pseudogene	X	41535013	41535747	
ENSG00000217643	0	0.697730409	pseudogene	2	26045338	26045819	
ENSG00000217644	1.16343121	1.667587519	pseudogene	1	33445543	33446073	
ENSG00000217646	0.499066092	0.390640832	pseudogene	6	27831840	27832179	HIST1H2BPS2
ENSG00000217648	3.211941663	3.081239798	pseudogene	6	143663383	143664520	
ENSG00000217653	0.869158192	0.390640832	pseudogene	6	90524428	90525182	
ENSG00000217684	0	0.390640832	pseudogene	6	141082666	141083396	
ENSG00000217702	0.499066092	0.697730409	protein_coding	2	74361599	74362521	
ENSG00000217716	1.960915222	1.518964905	pseudogene	9	90631215	90631708	RPS10P3
ENSG00000217733	0.499066092	0	pseudogene	6	150201098	150202708	CCT7P1
ENSG00000217746	0	0.390640832	pseudogene	6	7938663	7939555	
ENSG00000217783	0.499066092	0.950786998	pseudogene	6	157720077	157721158	
ENSG00000217791	0.499066092	0	pseudogene	5	53154996	53156231	ASS1P9
ENSG00000217801	6.604346021	7.32390599	pseudogene	1	995114	1004735	
ENSG00000217825	1.407729925	1.353254395	protein_coding	7	154988273	154990165	
ENSG00000217835	0.499066092	0	pseudogene	X	65176131	65177038	
ENSG00000217862	0.499066092	1.16600992	pseudogene	6	27774836	27775114	HIST1H4PS1
ENSG00000217889	0	0.390640832	pseudogene	X	152869952	152870803	
ENSG00000217897	0.499066092	0.390640832	pseudogene	1	39769966	39770570	
ENSG00000217929	0.499066092	0.390640832	pseudogene	6	142705	145059	
ENSG00000217930	7.491518048	7.862183683	protein_coding	16	4390252	4401373	PAM16
ENSG00000217950	0.869158192	1.518964905	pseudogene	2	132200167	132202467	
ENSG00000218014	1.407729925	0.697730409	pseudogene	6	72294424	72295609	
ENSG00000218018	1.616589159	0.697730409	antisense	20	55959216	55968118	
ENSG00000218052	0	1.353254395	pseudogene	15	85798600	85873565	
ENSG00000218069	0	0.390640832	pseudogene	6	27748399	27748890	RSL24D1P1
ENSG00000218073	1.616589159	1.16600992	pseudogene	6	16161157	16161642	
ENSG00000218175	4.568725997	5.306006045	pseudogene	2	177065636	177065980	
ENSG00000218180	0	0.390640832	pseudogene	6	121974941	121975836	
ENSG00000218208	2.10647801	2.652276565	pseudogene	6	113902703	113903149	
ENSG00000218226	5.346128795	5.059245468	pseudogene	6	159030738	159042668	TATDN2P2
ENSG00000218227	2.928422289	2.788380093	pseudogene	5	177482605	177483195	
ENSG00000218233	1.16343121	1.16600992	pseudogene	6	117954869	117966250	NEPNP
ENSG00000218265	0	0.950786998	pseudogene	6	13521498	13522277	
ENSG00000218281	1.16343121	0.950786998	pseudogene	6	26233350	26233483	HIST1H2APS3
ENSG00000218283	2.57521082	2.579085888	pseudogene	1	220426912	220427878	MORF4L1P1
ENSG00000218313	0	1.16600992	pseudogene	6	87883086	87884021	
ENSG00000218336	8.883132512	9.315729406	protein_coding	4	183065140	183724177	ODZ3
ENSG00000218347	0.499066092	1.16600992	pseudogene	6	27490855	27492118	HNRNPA1P1
ENSG00000218350	0.499066092	0	pseudogene	6	71874779	71875473	
ENSG00000218358	2.35979773	2.420525079	pseudogene	6	150319155	150326293	RAET1K
ENSG00000218359	0	0.390640832	pseudogene	6	17582265	17582536	

ENSG00000218416	2.928422289	1.802319292	protein_coding	2	241388732	241396131	
ENSG00000218418	0.499066092	2.242360793	pseudogene	6	80774002	80777854	
ENSG00000218422	4.19837882	4.872768657	pseudogene	4	1741078	1743830	
ENSG00000218426	4.6658819	4.961837817	pseudogene	6	153603422	153603868	
ENSG00000218428	0	0.390640832	pseudogene	6	116458221	116458735	
ENSG00000218454	0	0.390640832	pseudogene	17	20674379	20674736	HNRNPA1P19
ENSG00000218475	0.499066092	1.667587519	pseudogene	6	101878287	101879429	
ENSG00000218502	1.16343121	0.950786998	pseudogene	13	99867626	99868012	H2AFZP3
ENSG00000218510	5.613019223	6.091080508	lincRNA	1	22351681	22357716	LINC00339
ENSG00000218521	0.499066092	0.697730409	pseudogene	6	37970608	37971089	
ENSG00000218537	1.16343121	1.518964905	protein_coding	22	24236613	24241117	
ENSG00000218565	0	0.390640832	pseudogene	6	139659184	139660717	
ENSG00000218574	0	0.390640832	pseudogene	6	5609460	5610428	
ENSG00000218582	2.762599152	2.788380093	pseudogene	6	80662722	80663728	GAPDHP63
ENSG00000218586	0.869158192	0.390640832	pseudogene	7	55001392	55005877	
ENSG00000218596	0.499066092	0.697730409	pseudogene	6	157297428	157298203	
ENSG00000218631	0.499066092	0.697730409	pseudogene	6	157793588	157794203	
ENSG00000218632	0.499066092	0.697730409	pseudogene	6	109648378	109649117	
ENSG00000218676	0	0.390640832	pseudogene	6	111751463	111752984	BRD7P4
ENSG00000218682	0	0.950786998	pseudogene	2	26079330	26079835	
ENSG00000218690	1.799000381	2.242360793	pseudogene	6	26272421	26272768	HIST1H2APS4
ENSG00000218713	0.869158192	0.390640832	pseudogene	6	53071396	53073495	
ENSG00000218739	7.488200893	7.41347308	protein_coding	2	37423631	37440868	
ENSG00000218823	0.499066092	0	protein_coding	7	4897364	4901625	PAPOLB
ENSG00000218857	0	0.390640832	pseudogene	6	131505465	131506626	
ENSG00000218890	0	0.390640832	pseudogene	6	66803324	66804802	
ENSG00000218891	7.981274537	7.966311947	protein_coding	19	56088893	56092211	ZNF579
ENSG00000218896	0	1.667587519	pseudogene	6	143757353	143757847	
ENSG00000218902	0.499066092	1.16600992	pseudogene	20	17992599	17992928	PTMAP3
ENSG00000218976	0.499066092	0	pseudogene	6	18366967	18368875	
ENSG00000218980	5.563445526	5.259915406	pseudogene	6	56869330	56869598	FTH1P15
ENSG00000218996	0.869158192	0	pseudogene	6	151256104	151256702	
ENSG00000219023	1.407729925	0.390640832	pseudogene	6	35523650	35524041	
ENSG00000219027	0.499066092	0.950786998	pseudogene	16	1527829	1528584	RPS3AP2
ENSG00000219039	0	0.390640832	pseudogene	7	75464981	75465459	
ENSG00000219085	0	1.353254395	pseudogene	6	70415179	70416052	
ENSG00000219102	3.077135474	2.579085888	pseudogene	1	54440648	54441772	
ENSG00000219135	1.407729925	1.518964905	pseudogene	6	122001118	122001605	
ENSG00000219146	0.869158192	1.16600992	pseudogene	6	154897443	154898213	
ENSG00000219149	0	0.390640832	pseudogene	9	79164198	79164684	
ENSG00000219159	0	0.390640832	protein_coding	2	241625751	241629831	
ENSG00000219163	1.407729925	0	pseudogene	6	53100570	53101095	HMGB1P20
ENSG00000219200	5.512107582	6.495854971	protein_coding	17	6915736	6918199	RNASEK
ENSG00000219201	0.499066092	0.950786998	pseudogene	1	78276507	78277621	
ENSG00000219222	0.499066092	0	pseudogene	6	43277969	43278095	
ENSG00000219249	0.499066092	0	pseudogene	6	159146779	159147835	AMZ2P2
ENSG00000219257	0	0.390640832	pseudogene	6	100498259	100499141	
ENSG00000219274	1.16343121	1.16600992	pseudogene	16	1379079	1379778	RPS20P2
ENSG00000219294	0.869158192	0	pseudogene	6	7986730	7988425	PIP5K1P1
ENSG00000219298	0.499066092	0	pseudogene	6	150298607	150300241	
ENSG00000219329	1.16343121	1.16600992	pseudogene	6	111244769	111245310	
ENSG00000219355	1.407729925	1.353254395	pseudogene	12	120888240	120888606	RPL31P52
ENSG00000219361	0.499066092	0.950786998	pseudogene	6	81179788	81180145	
ENSG00000219375	0.499066092	0	pseudogene	6	7331519	7331909	
ENSG00000219392	1.407729925	0.950786998	pseudogene	6	28083406	28084329	
ENSG00000219395	1.616589159	3.027231696	pseudogene	6	151732394	151735080	
ENSG00000219409	0	0.390640832	pseudogene	6	144719095	144719888	
ENSG00000219410	2.57521082	0.950786998	protein_coding	12	6772426	6781235	
ENSG00000219433	1.16343121	0	pseudogene	6	150115899	150116882	
ENSG00000219445	0.499066092	0	processed_transc	7	124869633	124904345	
ENSG00000219448	0.499066092	0.390640832	pseudogene	6	157998119	158003309	
ENSG00000219451	1.616589159	1.16600992	pseudogene	7	20866945	20867372	RPL23P8
ENSG00000219470	1.616589159	1.353254395	pseudogene	6	43506559	43507440	

ENSG00000219481	7.461385447	8.049948078	processed_transc	1	16888818	16940083	NBPF1
ENSG00000219487	0.869158192	0.697730409	pseudogene	6	149433145	149433808	
ENSG00000219507	4.77879189	4.640685763	pseudogene	X	147133753	147134266	FTH1P8
ENSG00000219529	0	0.390640832	pseudogene	11	77524365	77524722	
ENSG00000219532	0.499066092	0.697730409	pseudogene	6	132140943	132141231	
ENSG00000219545	7.119373418	6.870289474	protein_coding	7	7680342	8043689	
ENSG00000219553	0.499066092	0	pseudogene	6	149812703	149813425	
ENSG00000219565	0	0.390640832	pseudogene	6	109106892	109108256	ZNF259P1
ENSG00000219607	3.004694206	2.851901313	protein_coding	6	5085720	5087455	PPP1R3G
ENSG00000219608	0.499066092	0.697730409	pseudogene	20	34466398	34466655	HIGD1AP16
ENSG00000219622	0	0.390640832	pseudogene	6	150825947	150831436	
ENSG00000219626	3.652182994	4.342020395	protein_coding	2	24299396	24392509	
ENSG00000219665	6.25990527	6.332882496	protein_coding	19	12098432	12157090	
ENSG00000219700	1.16343121	0.390640832	pseudogene	6	109609774	109611706	
ENSG00000219712	0	0.390640832	pseudogene	6	669081	670307	
ENSG00000219747	0.499066092	1.518964905	pseudogene	6	151420665	151421069	
ENSG00000219755	1.616589159	1.925536307	pseudogene	6	100023588	100024332	
ENSG00000219773	0.499066092	0.390640832	pseudogene	6	112677043	112677916	
ENSG00000219790	0	0.390640832	pseudogene	6	56840402	56840902	
ENSG00000219797	2.10647801	1.802319292	pseudogene	6	31487257	31488068	PPIAP9
ENSG00000219806	0.499066092	0.390640832	pseudogene	6	139935575	139936341	ATP5F1P6
ENSG00000219863	0	0.390640832	pseudogene	6	53219513	53219863	
ENSG00000219881	0	0.697730409	pseudogene	6	70455763	70456743	GAPDHP42
ENSG00000219891	3.603959378	2.579085888	pseudogene	6	28058932	28061442	ZSCAN12P1
ENSG00000219902	0	0.390640832	pseudogene	6	105750328	105750698	RPL35P3
ENSG00000219928	1.407729925	1.518964905	pseudogene	9	90140217	90140390	
ENSG00000219932	0.869158192	0.390640832	pseudogene	10	69634090	69634669	RPL12P8
ENSG00000219951	0.869158192	0.950786998	pseudogene	6	84102135	84103128	
ENSG00000219986	1.16343121	1.667587519	pseudogene	6	6795081	6795635	BTF3P7
ENSG00000219992	0.499066092	1.518964905	pseudogene	6	3754333	3755068	
ENSG00000219993	0	0.390640832	pseudogene	6	7506436	7506782	
ENSG00000220008	0.499066092	0.390640832	protein_coding	19	2289774	2308156	LINGO3
ENSG00000220032	2.762599152	2.242360793	protein_coding	18	74207477	74210045	
ENSG00000220105	0	0.697730409	pseudogene	9	4781475	4782078	
ENSG00000220130	0	0.390640832	pseudogene	6	89711267	89711533	
ENSG00000220157	1.616589159	0.390640832	pseudogene	5	79654899	79655859	HNRNPA1P12
ENSG00000220161	2.471521042	3.532712221	protein_coding	17	18314473	18317694	
ENSG00000220201	3.744048221	4.406904905	protein_coding	19	10415479	10420556	ZGLP1
ENSG00000220205	8.421213901	8.203944731	protein_coding	17	8054134	8066864	VAMP2
ENSG00000220248	0	0.390640832	pseudogene	22	17338509	17341608	ZNF402P
ENSG00000220267	0.869158192	0	pseudogene	6	88985601	88986708	ACTBP8
ENSG00000220305	1.799000381	1.353254395	pseudogene	6	160133833	160135017	
ENSG00000220370	4.491309013	4.7279204	pseudogene	6	95054455	95054680	
ENSG00000220378	1.960915222	0.697730409	pseudogene	6	134618134	134619834	KRT8P42
ENSG00000220392	0	0.390640832	pseudogene	6	111674905	111675386	
ENSG00000220412	0	2.039052734	pseudogene	6	138026560	138028338	
ENSG00000220446	0	0.390640832	pseudogene	6	5291291	5291628	
ENSG00000220472	2.238690726	2.144285137	pseudogene	6	7339072	7339801	
ENSG00000220506	0.499066092	1.16600992	pseudogene	6	111816194	111816916	
ENSG00000220515	0	0.390640832	pseudogene	6	73764820	73765580	PGAM1P10
ENSG00000220517	1.407729925	1.16600992	pseudogene	6	25023475	25024711	ASS1P1
ENSG00000220541	0	0.697730409	pseudogene	X	112765753	112766265	
ENSG00000220563	1.16343121	0.950786998	pseudogene	6	86369610	86370324	
ENSG00000220583	0.499066092	1.667587519	pseudogene	6	34231088	34231450	RPL35P2
ENSG00000220614	0	0.390640832	pseudogene	6	43295872	43296214	
ENSG00000220666	0.499066092	0	pseudogene	6	56296756	56297979	RCC2P7
ENSG00000220685	1.799000381	1.802319292	pseudogene	6	5066029	5067216	
ENSG00000220693	0.499066092	1.16600992	pseudogene	19	4477048	4479576	
ENSG00000220694	0.499066092	0	pseudogene	6	127756781	127757334	
ENSG00000220695	0	0.950786998	pseudogene	6	100978152	100978928	
ENSG00000220702	0.869158192	0.950786998	protein_coding	22	44761431	44762164	
ENSG00000220744	0	0.390640832	pseudogene	6	127683658	127684548	
ENSG00000220745	0.499066092	0	pseudogene	6	153280494	153280740	

ENSG00000220749	0.499066092	0.950786998	pseudogene	1	212224829	212225371	
ENSG00000220771	0	1.16600992	pseudogene	6	21602359	21602614	
ENSG00000220785	6.482942724	7.057067116	pseudogene	1	32697259	32707221	MTMR9LP
ENSG00000220793	0.869158192	0.950786998	pseudogene	16	9250219	9250761	RPL21P119
ENSG00000220804	3.698846687	3.570966319	pseudogene	2	243030784	243102304	
ENSG00000220842	2.57521082	2.912743273	pseudogene	10	122114177	122114718	
ENSG00000220848	2.671945279	3.027231696	pseudogene	6	149915220	149915679	RPS18P9
ENSG00000220867	0	0.390640832	pseudogene	6	158345434	158345708	
ENSG00000220875	0.499066092	1.353254395	pseudogene	6	26322104	26322520	H3F3AP1
ENSG00000220891	0	0.697730409	protein_coding	22	22901750	22909007	
ENSG00000220920	0	0.697730409	pseudogene	6	17953803	17954161	
ENSG00000220924	0	0.390640832	pseudogene	4	113606211	113606659	OSTCP4
ENSG00000220925	0.499066092	0.390640832	antisense	X	69368432	69369504	IGBP1-AS2
ENSG00000220937	0	0.950786998	pseudogene	9	4944673	4945902	
ENSG00000220987	0	0.390640832	miRNA	7	95936072	95936172	
ENSG00000220988	1.960915222	1.518964905	snoRNA	19	51305585	51305675	SNORD88C
ENSG00000220996	1.407729925	0	miRNA	5	115378868	115378958	
ENSG00000220998	0	0.390640832	miRNA	12	105479133	105479223	
ENSG00000221020	0	0.390640832	miRNA	17	40551027	40551109	
ENSG00000221027	0	0.390640832	miRNA	8	7996350	7996452	
ENSG00000221038	1.616589159	0.390640832	snRNA	17	29890787	29890913	
ENSG00000221044	1.799000381	0.950786998	snoRNA	17	42102389	42102591	
ENSG00000221046	0	0.697730409	snRNA	20	44407337	44407462	
ENSG00000221051	0	0.390640832	miRNA	19	40710019	40710106	
ENSG00000221070	0	0.950786998	miRNA	5	153719592	153719686	
ENSG00000221079	0	0.390640832	miRNA	4	89415047	89415167	
ENSG00000221102	0.869158192	0	snoRNA	14	91592769	91592897	SNORA11B
ENSG00000221111	0.499066092	0	miRNA	9	30771095	30771197	
ENSG00000221122	0	0.390640832	miRNA	4	40465733	40465821	
ENSG00000221164	2.57521082	2.039052734	snoRNA	10	74885838	74885965	
ENSG00000221174	0	0.697730409	miRNA	20	57180140	57180283	
ENSG00000221176	0	1.518964905	miRNA	8	129061398	129061484	MIR1207
ENSG00000221180	0.499066092	0	miRNA	14	106663421	106663529	
ENSG00000221181	0	0.390640832	miRNA	19	20473327	20473431	
ENSG00000221184	1.616589159	1.518964905	miRNA	10	70519075	70519171	MIR1254-1
ENSG00000221203	0.869158192	0	miRNA	1	68649201	68649293	MIR1262
ENSG00000221214	0	0.390640832	miRNA	10	112748684	112748771	MIR548E
ENSG00000221216	0.499066092	0	snRNA	1	28807874	28807992	
ENSG00000221220	0.869158192	0	miRNA	20	17823227	17823372	
ENSG00000221230	0.869158192	0.390640832	miRNA	11	94199661	94199746	MIR548L
ENSG00000221241	2.762599152	1.518964905	snoRNA	19	51302699	51302789	SNORD88A
ENSG00000221258	0	0.390640832	miRNA	19	38544518	38544605	
ENSG00000221261	0.869158192	0	miRNA	8	129162362	129162434	MIR1208
ENSG00000221288	3.335220907	4.961837817	miRNA	2	133014539	133014653	MIR663B
ENSG00000221297	0.499066092	0.697730409	miRNA	9	131019986	131020068	
ENSG00000221299	0.869158192	0.390640832	miRNA	X	134029784	134029872	
ENSG00000221300	0	0.390640832	snoRNA	2	42667517	42667576	
ENSG00000221309	0	0.390640832	miRNA	15	90838273	90838355	
ENSG00000221319	0.869158192	0.390640832	miRNA	20	29637162	29637300	
ENSG00000221322	0.869158192	0	miRNA	11	17095499	17095582	
ENSG00000221333	0.499066092	0.390640832	miRNA	11	70130061	70130176	MIR548K
ENSG00000221337	0	0.390640832	miRNA	3	41690747	41690828	
ENSG00000221338	0.499066092	0.697730409	miRNA	11	31457017	31457110	
ENSG00000221355	0	0.390640832	miRNA	17	16185328	16185402	MIR1288
ENSG00000221381	3.502389126	3.714580548	snoRNA	19	51302289	51302379	SNORD88B
ENSG00000221394	0.499066092	0.950786998	miRNA	5	179225278	179225346	MIR1229
ENSG00000221406	0	0.390640832	miRNA	1	224444706	224444843	MIR320B2
ENSG00000221410	0	0.697730409	miRNA	19	10662798	10662880	MIR1238
ENSG00000221411	0.499066092	1.353254395	miRNA	19	2234061	2234148	MIR1227
ENSG00000221413	0.499066092	0	miRNA	3	127383547	127383653	
ENSG00000221423	0.499066092	0	miRNA	1	33391895	33392000	
ENSG00000221429	0.869158192	0	miRNA	7	127930057	127930144	
ENSG00000221439	0.499066092	0.390640832	snRNA	12	890299	890424	

ENSG00000221462	2.471521042	2.579085888	snoRNA	17	62223697	62223831	SNORA76
ENSG00000221487	0.499066092	0	miRNA	7	99310660	99310775	
ENSG00000221491	2.471521042	0	snoRNA	12	49048165	49048301	SNORA34
ENSG00000221496	1.16343121	0.390640832	snoRNA	17	42381382	42381570	
ENSG00000221500	2.928422289	3.993387124	snoRNA	6	133137941	133138016	SNORD100
ENSG00000221520	1.16343121	0.950786998	miRNA	7	91833329	91833412	MIR1285-1
ENSG00000221526	0.499066092	0.390640832	miRNA	16	67911560	67911643	
ENSG00000221539	3.004694206	4.127774132	snoRNA	1	28905261	28905334	SNORD99
ENSG00000221540	0.869158192	0.950786998	miRNA	17	19247819	19247887	MIR1180
ENSG00000221579	0.499066092	0	miRNA	2	85526157	85526241	
ENSG00000221585	0.499066092	0.697730409	miRNA	3	47891045	47891119	MIR1226
ENSG00000221595	0.499066092	0	miRNA	19	48554681	48554766	
ENSG00000221598	0	0.390640832	miRNA	22	45596835	45596900	MIR1249
ENSG00000221611	0	0.697730409	snoRNA	12	13124987	13125077	
ENSG00000221613	0	0.390640832	miRNA	9	138420457	138420546	
ENSG00000221625	0.499066092	0.950786998	miRNA	12	98999652	98999738	
ENSG00000221634	0	0.390640832	miRNA	15	86313727	86313809	MIR1276
ENSG00000221639	0.499066092	0	snoRNA	4	74129677	74129801	
ENSG00000221643	0.499066092	0	snoRNA	1	203698709	203698833	SNORA77
ENSG00000221656	0.499066092	0.390640832	miRNA	16	2140196	2140285	MIR1225
ENSG00000221662	0.499066092	0.390640832	miRNA	1	19223565	19223642	MIR1290
ENSG00000221671	0	0.390640832	miRNA	8	64121666	64121754	
ENSG00000221676	0	0.950786998	snRNA	9	137029561	137029686	RNU6ATAC
ENSG00000221716	0.499066092	0.697730409	snoRNA	X	54840803	54840930	SNORA11
ENSG00000221725	0	0.390640832	snRNA	16	70886601	70886717	
ENSG00000221740	3.502389126	2.851901313	snoRNA	7	22896232	22896305	SNORD93
ENSG00000221743	2.471521042	1.16600992	miRNA	6	36108275	36108365	
ENSG00000221763	0	1.16600992	miRNA	20	34041776	34041919	MIR1289-1
ENSG00000221771	2.762599152	2.652276565	miRNA	8	128972879	128972941	MIR1205
ENSG00000221803	2.238690726	2.971122874	snoRNA	19	48259110	48259219	SNORD23
ENSG00000221806	0	0.390640832	snRNA	20	18591691	18591811	
ENSG00000221817	3.830412367	3.453019579	antisense	10	75255283	75264940	
ENSG00000221818	0.869158192	0.950786998	protein_coding	8	25699246	25902913	EBF2
ENSG00000221819	3.448790144	2.579085888	protein_coding	16	90095316	90096309	C16orf3
ENSG00000221821	4.165528823	4.976162367	protein_coding	6	42858005	42858554	C6orf226
ENSG00000221823	9.369490093	9.1807733	protein_coding	2	68358370	68488362	PPP3R1
ENSG00000221826	1.407729925	2.242360793	protein_coding	19	43225794	43244721	PSG3
ENSG00000221829	8.247679379	8.574870331	protein_coding	9	35073832	35080013	FANCG
ENSG00000221838	7.292905885	7.632773539	protein_coding	7	99699172	99707968	AP4M1
ENSG00000221843	2.847891871	3.608232228	protein_coding	2	27799389	27805588	C2orf16
ENSG00000221844	0.499066092	0	pseudogene	9	76089294	76090989	
ENSG00000221845	2.762599152	1.667587519	protein_coding	7	47694842	47701246	C7orf65
ENSG00000221857	2.57521082	2.039052734	protein_coding	19	35629728	35634013	FXYD1
ENSG00000221866	1.960915222	1.353254395	protein_coding	7	131808091	132333447	PLXNA4
ENSG00000221869	8.941297632	9.11108789	protein_coding	8	48649471	48651648	CEBPD
ENSG00000221878	0.499066092	1.667587519	polymorphic_pse	19	43428292	43441330	PSG7
ENSG00000221883	1.616589159	2.971122874	protein_coding	3	48955221	48956818	C3orf71
ENSG00000221886	5.4452544	6.45552765	protein_coding	5	159820155	159827104	C5orf54
ENSG00000221887	4.131913373	4.744751758	protein_coding	18	61616535	61649008	HMSD
ENSG00000221890	8.066039442	8.319396705	protein_coding	22	39214457	39239987	NPTXR
ENSG00000221909	6.244259325	5.938503778	protein_coding	7	99143931	99156159	FAM200A
ENSG00000221914	9.575770339	9.42680162	protein_coding	8	26149007	26230196	PPP2R2A
ENSG00000221916	2.671945279	3.368666104	protein_coding	19	49621655	49622397	C19orf73
ENSG00000221923	8.869173944	8.64603173	protein_coding	19	52873170	52889048	ZNF880
ENSG00000221926	8.316627558	8.180940029	protein_coding	17	15531274	15587613	TRIM16
ENSG00000221930	0.499066092	0.697730409	pseudogene	X	129629133	129630206	FAM45B
ENSG00000221944	5.637181576	5.80042639	protein_coding	2	233412779	233415226	TIGD1
ENSG00000221946	0.869158192	0.950786998	protein_coding	19	35634154	35645205	FXYD7
ENSG00000221947	5.958104008	5.726093467	protein_coding	8	71581600	71702606	XKR9
ENSG00000221949	2.238690726	2.420525079	protein_coding	12	62995531	62997214	C12orf61
ENSG00000221953	1.616589159	2.144285137	protein_coding	1	247273462	247275719	C1orf229
ENSG00000221954	0	0.390640832	protein_coding	11	50003009	50004071	OR4C12
ENSG00000221955	7.165790811	6.097646709	protein_coding	3	124801480	124998021	SLC12A8

ENSG00000221957	0.499066092	0	polymorphic_pse	19	55344131	55360024	KIR2DS4
ENSG00000221962	0.869158192	0.950786998	protein_coding	3	152057484	152058779	TMEM14E
ENSG00000221963	8.528449672	8.618900187	protein_coding	22	36044442	36057404	APOL6
ENSG00000221968	10.20322613	10.92686993	protein_coding	11	61640991	61659523	FADS3
ENSG00000221970	0	0.697730409	protein_coding	7	144015218	144016150	OR2A1
ENSG00000221971	0.499066092	0	pseudogene	7	46039220	46040377	TTC4P1
ENSG00000221978	9.967488686	10.37327626	protein_coding	1	1321091	1334708	CCNL2
ENSG00000221983	12.29989878	13.04599158	protein_coding	19	18682614	18688269	UBA52
ENSG00000221988	7.680671792	8.043160634	protein_coding	6	32121218	32134011	PPT2
ENSG00000221989	0.499066092	0	protein_coding	7	143806607	143807657	OR2A2
ENSG00000221990	5.60078458	6.281901708	protein_coding	5	441645	443258	C5orf55
ENSG00000221994	4.842525946	4.810179682	protein_coding	X	47842756	47931025	ZNF630
ENSG00000222000	0	0.390640832	protein_coding	2	98947852	98972468	
ENSG00000222004	0	1.16600992	protein_coding	7	26677490	26686889	C7orf71
ENSG00000222005	0.499066092	0.697730409	protein_coding	2	47043807	47049796	
ENSG00000222007	0.499066092	0	protein_coding	2	236682499	236692031	
ENSG00000222009	5.158541613	5.496926774	protein_coding	1	45274154	45281257	BTBD19
ENSG00000222011	5.958104008	5.855704078	protein_coding	7	102389418	102467706	FAM185A
ENSG00000222018	1.407729925	2.652276565	protein_coding	21	35772615	35773370	
ENSG00000222019	3.393122761	3.453019579	protein_coding	16	90106169	90114181	
ENSG00000222020	1.407729925	1.518964905	protein_coding	2	240323130	240324058	
ENSG00000222028	0	0.390640832	protein_coding	14	23511376	23513269	PSMB11
ENSG00000222031	0	0.390640832	protein_coding	2	151857734	151905288	
ENSG00000222032	0	0.390640832	lincRNA	2	238337563	238343465	
ENSG00000222033	3.911897206	4.101879561	protein_coding	2	171568961	171571077	
ENSG00000222035	0.499066092	0.390640832	antisense	2	202978349	202981781	
ENSG00000222036	0	0.697730409	protein_coding	14	19553365	19590078	POTEG
ENSG00000222040	2.35979773	0.697730409	protein_coding	2	96778707	96781984	ADRA2B
ENSG00000222041	7.444369108	7.819127564	processed_transc	2	87754887	87906324	LINC00152
ENSG00000222043	2.471521042	1.518964905	antisense	2	178129087	178130243	
ENSG00000222044	0.869158192	0.390640832	antisense	22	38428172	38430235	
ENSG00000222046	2.471521042	2.334191469	protein_coding	1	32674695	32681797	DCDC2B
ENSG00000222047	4.322773689	3.081239798	protein_coding	10	75669727	75682535	C10orf55
ENSG00000222071	0	0.697730409	miRNA	10	21785491	21785570	MIR1915
ENSG00000222072	0	0.390640832	misc_RNA	10	102666931	102667037	
ENSG00000222076	0.499066092	0.950786998	snRNA	15	96289033	96289223	RNU2-3P
ENSG00000222078	0	0.390640832	misc_RNA	6	90710941	90711225	
ENSG00000222108	1.16343121	0.697730409	rRNA	10	51739425	51739540	
ENSG00000222112	1.407729925	2.721932731	misc_RNA	1	33802167	33802465	
ENSG00000222145	0	0.390640832	snoRNA	6	89423961	89424149	
ENSG00000222152	0.499066092	0	snRNA	20	34357707	34357789	
ENSG00000222162	0.499066092	0.390640832	misc_RNA	11	13374755	13375070	
ENSG00000222164	0	0.390640832	misc_RNA	7	18847616	18847902	
ENSG00000222179	0.499066092	0.390640832	misc_RNA	18	43569619	43569911	
ENSG00000222197	0	0.390640832	miRNA	10	119117867	119117967	
ENSG00000222222	0.499066092	0.390640832	snRNA	1	150209315	150209504	
ENSG00000222224	0	0.390640832	misc_RNA	2	29150109	29150206	
ENSG00000222248	0.869158192	0.697730409	rRNA	6	3088920	3089043	RN5S201
ENSG00000222266	1.16343121	0.390640832	snRNA	5	134051173	134051272	
ENSG00000222267	0	0.697730409	snRNA	8	20087290	20087388	
ENSG00000222282	0.499066092	0	snRNA	1	38350695	38350789	
ENSG00000222287	0	0.697730409	snRNA	16	30712658	30712756	
ENSG00000222296	1.16343121	0.390640832	misc_RNA	9	130596230	130596321	
ENSG00000222301	1.16343121	1.353254395	misc_RNA	13	103472343	103472419	RNY5P8
ENSG00000222306	1.799000381	0.950786998	misc_RNA	4	113195228	113195309	
ENSG00000222344	0	0.390640832	snRNA	2	230759062	230759169	
ENSG00000222345	1.16343121	2.579085888	snoRNA	3	52725394	52725469	
ENSG00000222346	0	0.390640832	rRNA	7	111593708	111593820	RN5S237
ENSG00000222348	1.407729925	1.16600992	snRNA	3	10258896	10259003	
ENSG00000222351	0	0.390640832	misc_RNA	9	125708267	125708373	
ENSG00000222365	4.097495944	3.368666104	snoRNA	20	47896856	47896946	SNORD12B
ENSG00000222378	0	0.390640832	rRNA	1	39619836	39619968	RN5S44
ENSG00000222383	1.960915222	1.802319292	rRNA	6	10753024	10753159	RN5S203

ENSG00000222389	0.499066092	0	snRNA	3	81558627	81558815	
ENSG00000222395	0.499066092	0	misc_RNA	18	29843098	29843201	
ENSG00000222414	3.989024711	2.912743273	snRNA	10	103124602	103124792	
ENSG00000222432	0.499066092	0	misc_RNA	7	77525197	77525309	
ENSG00000222448	0.869158192	0	misc_RNA	5	179272832	179273152	
ENSG00000222460	1.16343121	1.518964905	misc_RNA	20	34231065	34231384	
ENSG00000222482	0	0.390640832	miRNA	7	99817650	99817743	
ENSG00000222489	1.799000381	2.242360793	snoRNA	14	20791338	20791485	
ENSG00000222493	1.799000381	1.802319292	misc_RNA	12	2969115	2969199	
ENSG00000222494	0.499066092	0.390640832	miRNA	17	37237318	37237440	
ENSG00000222501	3.004694206	2.652276565	snRNA	8	129022628	129022857	
ENSG00000222503	0.499066092	0	misc_RNA	10	105432916	105433023	
ENSG00000222509	0.499066092	0	misc_RNA	2	171639677	171639781	
ENSG00000222533	0	0.390640832	snRNA	5	180728968	180729071	
ENSG00000222545	0.869158192	0.390640832	miRNA	17	70710522	70710610	
ENSG00000222561	0	0.697730409	snRNA	11	948421	948524	
ENSG00000222578	0	0.390640832	rRNA	11	95573181	95573288	
ENSG00000222588	0.499066092	0.390640832	snoRNA	10	120820495	120820623	
ENSG00000222614	0	0.390640832	misc_RNA	19	47239569	47239676	
ENSG00000222627	0.499066092	0.390640832	snRNA	3	127793916	127794092	
ENSG00000222629	0.869158192	0	snRNA	10	127579135	127579247	
ENSG00000222640	0.869158192	0	snRNA	14	70827609	70827805	
ENSG00000222650	0	0.390640832	snRNA	1	236431080	236431258	
ENSG00000222690	0	0.390640832	miRNA	18	47013743	47013792	MIR1539
ENSG00000222724	1.16343121	0.950786998	snRNA	2	88315873	88316066	
ENSG00000222726	0.869158192	0.390640832	snRNA	13	21186300	21186477	RNU2-7P
ENSG00000222730	4.464551814	3.411459265	miRNA	19	36821351	36821449	
ENSG00000222732	0	0.390640832	miRNA	5	171706206	171706319	
ENSG00000222747	0	0.390640832	rRNA	13	21943929	21944038	RN5S25
ENSG00000222764	0	0.390640832	misc_RNA	6	141807278	141807549	
ENSG00000222808	1.407729925	0.950786998	snRNA	17	75148643	75148756	
ENSG00000222872	0.499066092	0	snRNA	3	42263332	42263457	
ENSG00000222881	2.238690726	2.242360793	misc_RNA	17	38547484	38547590	
ENSG00000222894	0.869158192	0.390640832	miRNA	6	30584006	30584082	
ENSG00000222898	2.471521042	0.390640832	misc_RNA	8	62544239	62544571	
ENSG00000222915	1.16343121	1.16600992	snRNA	22	30817034	30817137	
ENSG00000222937	3.146112541	2.039052734	snoRNA	5	137894659	137894728	
ENSG00000222942	0	0.390640832	misc_RNA	3	41808107	41808411	
ENSG00000222954	0.499066092	0	miRNA	4	56796148	56796239	
ENSG00000222969	1.16343121	0.697730409	misc_RNA	13	98856089	98856446	RN7SKP8
ENSG00000223001	0	0.390640832	snRNA	6	89773219	89773409	
ENSG00000223023	0	0.390640832	misc_RNA	18	20604559	20604666	
ENSG00000223044	0	0.697730409	snRNA	6	82920055	82920157	
ENSG00000223060	1.16343121	0.697730409	misc_RNA	19	56616785	56616879	
ENSG00000223086	0.869158192	0	rRNA	4	10117380	10117508	RN5S155
ENSG00000223117	2.10647801	1.802319292	misc_RNA	3	190359952	190360292	
ENSG00000223120	0	0.390640832	misc_RNA	15	97385299	97385614	
ENSG00000223138	0	0.697730409	rRNA	18	9923917	9924018	
ENSG00000223174	0.499066092	0.950786998	misc_RNA	14	77356606	77356956	
ENSG00000223198	0	0.697730409	snRNA	2	232366701	232366912	
ENSG00000223214	0.499066092	0	miRNA	8	15379393	15379475	
ENSG00000223220	0.499066092	0	misc_RNA	8	71693149	71693243	
ENSG00000223224	0	0.390640832	snoRNA	16	71792305	71792390	SNORD71
ENSG00000223225	0	0.390640832	snRNA	4	119527605	119527708	
ENSG00000223244	1.616589159	1.667587519	miRNA	19	1816158	1816237	MIR1909
ENSG00000223247	0.499066092	0.697730409	snRNA	3	73160143	73160333	
ENSG00000223254	2.10647801	0	misc_RNA	1	85729979	85730085	
ENSG00000223263	0	0.950786998	snRNA	1	67882897	67883001	
ENSG00000223271	0	0.390640832	misc_RNA	15	66640614	66640713	
ENSG00000223279	0	0.697730409	snRNA	10	111629616	111629750	RNU4-5P
ENSG00000223284	0.499066092	0	snRNA	19	41213960	41214068	
ENSG00000223298	0.499066092	0.697730409	misc_RNA	13	95963084	95963209	RNY3P8
ENSG00000223300	0	0.390640832	misc_RNA	14	74324764	74324868	

ENSG00000223305	0.869158192	0	misc_RNA	4	56406669	56407002	
ENSG00000223306	0.499066092	0.390640832	snRNA	1	225928977	225929084	
ENSG00000223309	1.16343121	0	snRNA	X	48816440	48816543	
ENSG00000223313	1.16343121	0.390640832	snRNA	15	40821769	40821872	
ENSG00000223336	2.471521042	0.950786998	snRNA	13	46948536	46948725	RNU2-6P
ENSG00000223343	1.16343121	0	antisense	3	49022482	49027421	
ENSG00000223345	1.407729925	0.697730409	processed_transc	1	120906028	120915073	HIST2H2BA
ENSG00000223346	2.35979773	2.501982735	lincRNA	20	29638246	29641792	
ENSG00000223356	1.960915222	1.667587519	antisense	1	156682004	156682966	
ENSG00000223361	0	0.950786998	pseudogene	5	17353804	17354733	FTH1P10
ENSG00000223374	3.004694206	2.501982735	antisense	2	242290755	242292519	
ENSG00000223379	0.499066092	0	lincRNA	9	45004879	45023236	
ENSG00000223380	9.398051829	9.951595627	processed_transc	1	145096220	145116922	SEC22B
ENSG00000223385	6.328285199	6.926693689	processed_transc	17	13932609	13972775	COX10-AS1
ENSG00000223387	0.499066092	0.950786998	lincRNA	3	172278678	172313397	
ENSG00000223390	1.407729925	0.697730409	antisense	1	52499063	52509951	
ENSG00000223393	0.499066092	0	antisense	1	231010592	231014761	
ENSG00000223396	2.238690726	1.518964905	processed_transc	1	201487831	201499602	RPS10P7
ENSG00000223401	1.799000381	2.144285137	lincRNA	3	187461474	187463208	
ENSG00000223403	0	0.390640832	lincRNA	14	101536248	101539274	
ENSG00000223409	0	1.353254395	pseudogene	3	65829565	65830121	RPL17P17
ENSG00000223416	0	0.390640832	pseudogene	1	58521805	58522152	
ENSG00000223424	0	0.390640832	antisense	4	156275368	156281613	
ENSG00000223427	0.499066092	0	pseudogene	2	80389554	80390261	
ENSG00000223429	0	0.390640832	pseudogene	1	52627858	52628698	
ENSG00000223431	0.869158192	1.16600992	pseudogene	21	45892778	45893622	MTND6P21
ENSG00000223440	0.499066092	0	antisense	9	32633452	32648683	
ENSG00000223442	1.16343121	1.16600992	antisense	5	131966281	131999964	
ENSG00000223445	0	0.390640832	pseudogene	7	14110160	14111005	
ENSG00000223459	2.847891871	3.64455972	pseudogene	7	143295133	143305111	FAM115B
ENSG00000223460	0.499066092	0.390640832	pseudogene	13	29880915	29881922	GAPDHP69
ENSG00000223461	1.16343121	2.039052734	antisense	22	19109042	19112016	
ENSG00000223466	0.499066092	0.390640832	lincRNA	2	196397759	196403405	
ENSG00000223473	4.261916566	3.368666104	lincRNA	7	65956083	65958553	
ENSG00000223474	2.10647801	1.667587519	protein_coding	1	26145212	26147288	
ENSG00000223476	1.799000381	0.697730409	pseudogene	7	64393651	64393994	VN1R42P
ENSG00000223477	4.642199401	2.334191469	lincRNA	10	47096454	47099716	
ENSG00000223478	5.331395195	5.98174124	antisense	9	131486724	131495473	
ENSG00000223482	7.392081667	7.768587741	antisense	10	88963610	89102369	
ENSG00000223485	4.593635215	5.004391715	lincRNA	6	169558187	169563019	
ENSG00000223486	0.869158192	0.697730409	processed_transc	X	39868529	39871435	
ENSG00000223488	0.499066092	0	pseudogene	21	23808610	23810669	MAPK6PS2
ENSG00000223491	0.499066092	0	pseudogene	1	148000213	148000494	
ENSG00000223492	0.499066092	0	antisense	20	51785182	51796089	
ENSG00000223496	7.893726093	7.780879746	protein_coding	16	70284134	70285833	EXOSC6
ENSG00000223500	0.499066092	0.390640832	pseudogene	7	134813196	134814437	
ENSG00000223501	9.05999771	9.319245234	protein_coding	6	33218049	33239824	VPS52
ENSG00000223503	0	1.802319292	pseudogene	1	155560392	155561053	
ENSG00000223505	0.499066092	0.390640832	pseudogene	1	203804708	203805040	
ENSG00000223508	2.57521082	2.721932731	pseudogene	8	163251	163707	RPL23AP53
ENSG00000223509	4.097495944	4.528525526	pseudogene	15	32814894	32829127	
ENSG00000223510	1.616589159	1.353254395	protein_coding	17	14138990	14140179	CDRT15
ENSG00000223511	5.4452544	6.434933235	lincRNA	X	1851477	1874878	
ENSG00000223518	0.869158192	0.390640832	pseudogene	15	37090797	37110660	CSNK1A1P1
ENSG00000223519	0.869158192	2.334191469	protein_coding	1	246939531	246952963	
ENSG00000223522	3.274897671	2.971122874	antisense	2	28530558	28533326	
ENSG00000223529	2.928422289	3.411459265	pseudogene	3	183744157	183745544	EEF1A1P8
ENSG00000223540	0.499066092	0	pseudogene	10	123473121	123473508	RPS15AP5
ENSG00000223546	5.301468762	5.824376633	processed_transc	X	102024089	102140334	
ENSG00000223547	7.985986001	7.960920138	protein_coding	19	12175514	12192380	ZNF844
ENSG00000223549	0.869158192	1.16600992	pseudogene	2	120972757	120974855	MTND5P28
ENSG00000223551	3.989024711	4.385599336	protein_coding	9	131104432	131105049	TMSL4
ENSG00000223552	4.026095388	2.039052734	antisense	3	46406446	46448550	

ENSG00000223553	0.499066092	0.390640832	pseudogene	22	20956882	21011201	SMPD4P1
ENSG00000223558	2.671945279	2.242360793	pseudogene	7	63545938	63546954	TRIM60P17
ENSG00000223559	1.960915222	1.925536307	pseudogene	7	56355923	56359118	
ENSG00000223561	0.499066092	0.390640832	processed_transc	7	25632971	25790614	
ENSG00000223566	1.16343121	0.697730409	pseudogene	7	63028121	63045453	
ENSG00000223571	0.499066092	0.390640832	sense_intronic	X	2252336	2254451	DHRSX-IT1
ENSG00000223572	4.097495944	0.390640832	protein_coding	15	43886225	43991420	CKMT1A
ENSG00000223573	5.207774174	5.372493443	processed_transc	19	5558178	5568045	PLAC2
ENSG00000223575	0	0.390640832	pseudogene	1	119627531	119628577	RBMX2P3
ENSG00000223576	0.869158192	1.16600992	antisense	13	20417276	20417812	ZMYM5-AS1
ENSG00000223579	0.869158192	0	lincRNA	22	20632132	20661407	
ENSG00000223583	1.407729925	1.353254395	pseudogene	1	26781144	26781678	
ENSG00000223584	0	0.390640832	pseudogene	7	90261220	90261882	
ENSG00000223586	0.499066092	0	processed_transc	6	134142285	134172938	
ENSG00000223588	0	0.390640832	pseudogene	2	132534140	132534601	
ENSG00000223598	0.869158192	0	processed_transc	6	151409239	151549582	
ENSG00000223599	0.499066092	1.16600992	pseudogene	1	153824582	153825890	
ENSG00000223624	1.16343121	0.390640832	pseudogene	1	26215461	26215600	
ENSG00000223634	0.499066092	0	lincRNA	2	23580387	23583247	
ENSG00000223635	0	0.390640832	antisense	1	229644248	229650019	
ENSG00000223638	0.499066092	0	protein_coding	19	56270380	56274541	RFPL4A
ENSG00000223650	0.869158192	1.518964905	pseudogene	X	73325407	73327793	UHRF2P1
ENSG00000223652	0	0.390640832	antisense	5	122422943	122425994	
ENSG00000223653	0.499066092	1.518964905	processed_transc	1	85742398	85913807	
ENSG00000223658	0	0.390640832	pseudogene	2	43902292	43903461	
ENSG00000223662	0.499066092	0	antisense	21	15954523	15970624	SAMSN1-AS1
ENSG00000223668	1.16343121	0.697730409	pseudogene	3	39400036	39401413	EEF1A1P24
ENSG00000223685	0	1.353254395	lincRNA	13	38624954	38717369	
ENSG00000223692	1.407729925	1.16600992	sense_intronic	21	47882384	47889219	DIP2A-IT1
ENSG00000223694	0	0.390640832	pseudogene	1	240333455	240334591	ADH5P3
ENSG00000223695	0.499066092	2.334191469	lincRNA	22	36784671	36792562	
ENSG00000223697	1.616589159	1.16600992	antisense	8	133850375	133856543	
ENSG00000223700	0	0.390640832	pseudogene	2	84104851	84105677	
ENSG00000223701	0	0.390640832	processed_transc	6	150211239	150240644	
ENSG00000223703	0	0.697730409	pseudogene	2	91924752	91955465	
ENSG00000223704	2.762599152	3.368666104	lincRNA	22	27254597	27261749	
ENSG00000223705	7.686472517	7.705505903	pseudogene	7	75039605	75046066	NSUN5P1
ENSG00000223711	0.499066092	1.353254395	antisense	3	195270871	195277400	
ENSG00000223715	0	0.390640832	lincRNA	3	176321931	176353320	
ENSG00000223718	0.869158192	0	pseudogene	7	135344787	135345401	
ENSG00000223722	1.407729925	1.353254395	pseudogene	12	31907651	31908055	
ENSG00000223723	0.499066092	0.697730409	pseudogene	X	36980269	36981498	
ENSG00000223724	0.499066092	0	pseudogene	13	49618215	49620930	RAD17P2
ENSG00000223725	1.616589159	2.501982735	antisense	2	208104588	208394519	
ENSG00000223732	0	0.697730409	lincRNA	13	45620725	45657260	
ENSG00000223739	0	0.390640832	pseudogene	2	65738905	65739210	
ENSG00000223740	0.499066092	0.697730409	pseudogene	7	56593121	56593806	
ENSG00000223741	2.847891871	3.679994897	pseudogene	21	37858281	37859709	PSMD4P1
ENSG00000223745	6.275383355	6.549526374	processed_transc	1	93727743	93811582	
ENSG00000223749	6.604346021	7.053694091	processed_transc	X	133677367	133680662	
ENSG00000223750	0	0.697730409	pseudogene	20	1645929	1688846	
ENSG00000223751	1.799000381	1.16600992	lincRNA	2	742488	747767	
ENSG00000223756	4.230497448	3.813624741	pseudogene	11	3402191	3430208	TSSC2
ENSG00000223759	3.603959378	3.493416095	processed_transc	1	149672997	149677287	
ENSG00000223764	3.335220907	2.652276565	processed_transc	1	852250	855072	
ENSG00000223768	4.75690578	4.961837817	non_coding	21	46713200	46717269	LINC00205
ENSG00000223770	0.499066092	0	antisense	7	81638493	81659271	MIR1255B1
ENSG00000223773	4.642199401	4.744751758	pseudogene	X	2527389	2575270	CD99P1
ENSG00000223774	0.499066092	0	antisense	1	201862970	201869106	
ENSG00000223776	0.499066092	1.518964905	antisense	1	236686369	236687808	LGALS8-AS1
ENSG00000223779	1.799000381	2.039052734	processed_transc	1	149239551	149265510	
ENSG00000223784	4.437288965	2.039052734	lincRNA	10	6779344	6780988	
ENSG00000223787	0	1.16600992	pseudogene	1	93046038	93046404	

ENSG00000223791	0	0.697730409	antisense	3	23845515	23848396	UBE2E1-AS1
ENSG00000223795	0	0.390640832	lincRNA	9	137397318	137413288	
ENSG00000223797	3.603959378	3.935919592	processed_transc	3	40355293	40494820	ENTPD3-AS1
ENSG00000223799	1.407729925	2.144285137	processed_transc	21	34636178	34638565	
ENSG00000223802	0	1.667587519	protein_coding	19	18979361	19007488	CERS1
ENSG00000223803	0.869158192	0.390640832	pseudogene	3	186617987	186618346	
ENSG00000223804	1.407729925	2.334191469	processed_transc	1	143647638	143745417	
ENSG00000223807	0	0.390640832	pseudogene	9	32926049	32926448	
ENSG00000223811	0	0.390640832	lincRNA	6	113678205	113689146	
ENSG00000223812	5.538004898	6.434933235	lincRNA	3	191275113	191308145	
ENSG00000223813	1.407729925	1.518964905	processed_transc	7	29554385	29603286	
ENSG00000223814	0.869158192	1.353254395	lincRNA	1	48226804	48231219	
ENSG00000223819	0	0.390640832	pseudogene	X	100055291	100055773	
ENSG00000223820	2.238690726	2.334191469	pseudogene	10	89577685	89605369	CFL1P1
ENSG00000223822	2.238690726	2.501982735	pseudogene	21	24762579	24763317	EEF1A1P1
ENSG00000223825	3.077135474	2.039052734	pseudogene	2	203066103	203066609	
ENSG00000223829	1.16343121	0.390640832	processed_transc	7	47009300	47067079	
ENSG00000223831	3.554067925	3.748356452	sense_intronic	22	30994296	31002674	
ENSG00000223837	1.960915222	2.144285137	sense_intronic	6	32938080	32938594	BRD2-IT1
ENSG00000223839	2.57521082	1.16600992	processed_transc	9	42466317	42474236	FAM95B1
ENSG00000223842	1.16343121	1.518964905	lincRNA	1	219395590	219398839	
ENSG00000223849	1.799000381	0.950786998	pseudogene	9	97885803	97886150	
ENSG00000223855	2.238690726	3.32456471	processed_transc	7	560028	564869	
ENSG00000223861	2.928422289	3.081239798	pseudogene	1	151529922	151530416	
ENSG00000223864	0.869158192	0.390640832	pseudogene	20	37606354	37607065	NPM1P19
ENSG00000223865	0.869158192	0	protein_coding	6	33043703	33054978	HLA-DPB1
ENSG00000223872	0	1.16600992	lincRNA	7	157294016	157296271	
ENSG00000223877	1.16343121	2.144285137	pseudogene	15	22440227	22440848	
ENSG00000223878	3.146112541	3.993387124	pseudogene	17	15410180	15410668	
ENSG00000223882	1.16343121	0.390640832	antisense	3	183724126	183729207	ABCC5-AS1
ENSG00000223886	1.616589159	2.652276565	pseudogene	7	105170656	105171118	
ENSG00000223891	4.883502971	5.751299057	processed_transc	20	42839600	42854667	
ENSG00000223896	0.499066092	0	pseudogene	1	93221366	93222509	CCNJP2
ENSG00000223901	0.869158192	1.667587519	antisense	21	47640183	47641992	
ENSG00000223903	0.869158192	0.390640832	pseudogene	2	220562521	220562936	
ENSG00000223907	0	0.390640832	lincRNA	1	31984036	31989846	
ENSG00000223910	0.499066092	0.390640832	processed_transc	10	44124265	44170151	ZNF32-AS3
ENSG00000223916	0.499066092	0.950786998	pseudogene	3	43526464	43527372	
ENSG00000223917	0	0.390640832	pseudogene	2	96152228	96154375	
ENSG00000223922	1.799000381	1.353254395	pseudogene	2	39037574	39038796	ASS1P2
ENSG00000223923	0	0.390640832	antisense	2	218843430	218857338	
ENSG00000223935	1.16343121	0.390640832	antisense	2	64622354	64680932	
ENSG00000223940	0	0.390640832	pseudogene	X	151648049	151649496	KRT8P8
ENSG00000223945	1.407729925	1.353254395	pseudogene	1	150025846	150027016	
ENSG00000223947	2.10647801	1.667587519	antisense	2	101610138	101618706	
ENSG00000223949	3.146112541	1.925536307	antisense	1	64560125	64636980	
ENSG00000223956	1.16343121	0.390640832	lincRNA	1	56880635	56881638	
ENSG00000223959	8.120617457	8.712778563	pseudogene	16	90038994	90068569	AFG3L1P
ENSG00000223960	6.005279909	6.293387477	processed_transc	2	179278666	179303866	
ENSG00000223963	0.499066092	0	pseudogene	1	240932721	240934834	PRKRIRP8
ENSG00000223968	0	0.390640832	pseudogene	3	27665693	27665947	
ENSG00000223969	0	1.925536307	antisense	7	90219933	90226667	
ENSG00000223973	1.16343121	1.353254395	pseudogene	2	112141329	112142569	
ENSG00000223974	1.960915222	1.16600992	pseudogene	7	64011876	64012444	
ENSG00000223975	1.407729925	2.144285137	lincRNA	21	44885189	44887178	
ENSG00000223979	0.499066092	0.390640832	lincRNA	17	17577340	17581002	SMCR2
ENSG00000223981	0.499066092	0	pseudogene	7	74619641	74620426	PHBP6
ENSG00000223984	1.16343121	0.950786998	pseudogene	10	21913521	21915215	
ENSG00000223991	0.499066092	0	antisense	2	241920932	241925489	
ENSG00000223998	1.16343121	0.390640832	sense_intronic	6	86270033	86270928	
ENSG00000224001	0	0.390640832	pseudogene	9	100997010	100997818	
ENSG00000224003	0.869158192	0	pseudogene	22	26043228	26045199	
ENSG00000224004	0.869158192	0.950786998	pseudogene	14	54457818	54458713	ATP5C1P1

ENSG00000224005	1.616589159	1.16600992	pseudogene	10	75473354	75473609	
ENSG00000224007	0.499066092	0.697730409	pseudogene	2	149053225	149054248	
ENSG00000224011	0.499066092	0.697730409	pseudogene	17	58804526	58804843	
ENSG00000224014	0	0.390640832	pseudogene	1	247338558	247338827	
ENSG00000224016	0	0.390640832	pseudogene	7	149588280	149606793	
ENSG00000224017	0.499066092	0	lincRNA	7	41141202	41173105	
ENSG00000224019	0	0.697730409	pseudogene	2	186821314	186821790	
ENSG00000224020	2.671945279	3.532712221	antisense	9	127420746	127460910	MIR181A2HG
ENSG00000224021	0	0.390640832	antisense	1	32817122	32819143	
ENSG00000224023	1.407729925	2.144285137	processed_transc	10	127389005	127408135	
ENSG00000224025	0.499066092	0.697730409	pseudogene	9	71224041	71226893	
ENSG00000224031	0	0.697730409	antisense	X	102881004	102881420	TCEAL3-AS1
ENSG00000224032	6.907250452	7.026422172	non_coding	5	111496223	111499973	EPB41L4A-AS1
ENSG00000224034	0.499066092	0.697730409	lincRNA	10	5307996	5313199	
ENSG00000224037	1.616589159	1.518964905	processed_transc	1	234845005	234855723	
ENSG00000224040	0	1.16600992	pseudogene	1	182911250	182911539	HMGN1P4
ENSG00000224043	2.238690726	1.802319292	antisense	2	135493034	135676280	
ENSG00000224046	3.448790144	3.279072565	processed_transc	7	86780739	86781736	
ENSG00000224050	3.004694206	2.039052734	lincRNA	22	32669407	32673173	
ENSG00000224051	8.223944437	7.582164879	protein_coding	1	1260136	1264277	GLTPD1
ENSG00000224055	0.869158192	1.353254395	pseudogene	15	44355709	44356511	GAPDHP55
ENSG00000224057	0.499066092	1.518964905	antisense	7	55247443	55256627	
ENSG00000224063	1.616589159	2.420525079	processed_transc	2	187867947	188419390	
ENSG00000224066	1.407729925	1.353254395	antisense	1	32670370	32672415	
ENSG00000224067	2.238690726	1.802319292	pseudogene	9	114565260	114565710	
ENSG00000224072	1.799000381	1.667587519	pseudogene	X	46313532	46316634	
ENSG00000224073	0	0.950786998	lincRNA	6	160060339	160061133	
ENSG00000224074	0.499066092	0.390640832	processed_transc	3	24137760	24144738	
ENSG00000224077	0.869158192	0.390640832	antisense	11	116969703	116978886	
ENSG00000224078	9.422587759	9.145559356	processed_transc	15	25224704	25664609	SNHG14
ENSG00000224079	2.10647801	1.518964905	antisense	7	1114086	1117672	
ENSG00000224080	1.960915222	1.925536307	pseudogene	3	37185003	37185449	
ENSG00000224081	1.960915222	0.950786998	processed_transc	1	95104017	95285837	
ENSG00000224083	0.499066092	0	pseudogene	9	5096666	5098193	
ENSG00000224086	3.989024711	5.187895122	antisense	22	22292609	22297799	
ENSG00000224093	1.407729925	2.039052734	antisense	1	94057756	94071130	
ENSG00000224094	2.238690726	2.788380093	pseudogene	3	45201266	45201667	
ENSG00000224097	1.799000381	1.353254395	pseudogene	4	39481875	39483525	
ENSG00000224110	0.499066092	0.390640832	pseudogene	8	130740990	130742691	
ENSG00000224113	4.437288965	3.279072565	protein_coding	17	33332275	33333756	
ENSG00000224114	0.499066092	0.697730409	pseudogene	1	206869182	206869614	
ENSG00000224116	2.238690726	2.579085888	antisense	7	41733514	41818986	
ENSG00000224117	0	0.390640832	pseudogene	13	31127807	31128830	PTPN2P2
ENSG00000224119	0.499066092	0	sense_intronic	11	70412492	70417428	
ENSG00000224121	0	0.390640832	pseudogene	2	223769358	223769773	ATG12P2
ENSG00000224124	2.471521042	2.039052734	pseudogene	22	25041133	25055114	POM121L10P
ENSG00000224126	2.57521082	2.242360793	pseudogene	17	18580574	18581072	
ENSG00000224132	0	0.390640832	protein_coding	2	238165734	238166319	
ENSG00000224137	1.616589159	0.950786998	lincRNA	2	208527099	208531748	
ENSG00000224138	3.146112541	2.851901313	antisense	7	126990182	126991577	
ENSG00000224142	0	0.390640832	sense_intronic	X	109548648	109550449	AMMECR1-IT1
ENSG00000224152	2.762599152	2.334191469	antisense	2	160471807	160473593	
ENSG00000224155	0.499066092	0	pseudogene	7	56372371	56373219	
ENSG00000224157	0.869158192	0.950786998	antisense	6	28864307	28865099	HCG14
ENSG00000224165	3.211941663	3.608232228	processed_transc	2	25194259	25262563	DNAJC27-AS1
ENSG00000224172	1.16343121	0.950786998	pseudogene	7	64196311	64197121	
ENSG00000224173	0.499066092	0	lincRNA	2	67789199	67801176	
ENSG00000224177	1.799000381	1.802319292	lincRNA	2	11534107	11543203	
ENSG00000224183	0	0.390640832	pseudogene	1	25620655	25621134	SDHDP6
ENSG00000224184	1.16343121	0	lincRNA	2	11988748	12718474	
ENSG00000224186	3.448790144	3.411459265	protein_coding	5	134368970	134691744	
ENSG00000224189	2.762599152	3.027231696	processed_transc	2	177037923	177053686	
ENSG00000224194	0.499066092	0	antisense	2	15808808	15820608	

ENSG00000224195	2.671945279	1.667587519	antisense	10	75573276	75574495	
ENSG00000224207	0	0.697730409	pseudogene	4	103655515	103655772	
ENSG00000224208	1.616589159	1.518964905	pseudogene	X	70534762	70535729	
ENSG00000224209	0.869158192	0	processed_transc	1	63624707	63782902	LINC00466
ENSG00000224215	0.499066092	0.697730409	antisense	10	23632886	23634110	
ENSG00000224216	0	0.390640832	antisense	X	154564167	154581271	
ENSG00000224221	0	0.390640832	pseudogene	6	74100761	74101490	
ENSG00000224222	0.869158192	0.697730409	lincRNA	10	71754382	71767592	
ENSG00000224224	0	0.697730409	pseudogene	X	18883889	18884672	
ENSG00000224232	1.799000381	1.16600992	pseudogene	2	242417731	242418408	
ENSG00000224238	1.16343121	0.697730409	sense_intronic	1	119590028	119607408	WARS2-IT1
ENSG00000224245	0.869158192	1.16600992	pseudogene	9	97258557	97275161	
ENSG00000224251	0.869158192	0.390640832	antisense	10	5038045	5039572	
ENSG00000224255	0.499066092	0	pseudogene	1	52645518	52646221	
ENSG00000224259	2.471521042	2.652276565	lincRNA	1	159931008	159948851	
ENSG00000224260	0.499066092	0	antisense	1	209701800	209741018	
ENSG00000224261	1.960915222	2.242360793	pseudogene	1	160236129	160237016	
ENSG00000224263	0	0.390640832	pseudogene	3	11920304	11920603	
ENSG00000224271	2.57521082	2.912743273	processed_transc	22	48027423	48251349	
ENSG00000224272	2.671945279	2.788380093	processed_transc	2	242749407	242753111	
ENSG00000224273	0.499066092	0.697730409	pseudogene	7	75747043	75761505	
ENSG00000224274	0	0.390640832	pseudogene	20	15600139	15600464	ENSAP1
ENSG00000224276	0	0.390640832	antisense	1	155961181	155971578	
ENSG00000224280	0.499066092	0.950786998	pseudogene	7	16623407	16626382	
ENSG00000224281	3.077135474	3.813624741	processed_transc	X	118599997	118603061	SLC25A5-AS1
ENSG00000224282	0.499066092	0.697730409	pseudogene	5	31822668	31823243	
ENSG00000224287	5.741167503	5.792353798	pseudogene	2	234774083	234777090	MSL3P1
ENSG00000224289	0.499066092	0	pseudogene	10	91122051	91123244	IFIT6P
ENSG00000224292	3.211941663	1.802319292	antisense	X	48364911	48367226	
ENSG00000224294	0.499066092	0.390640832	lincRNA	X	43036243	43085847	
ENSG00000224295	1.407729925	0.950786998	pseudogene	11	5539671	5546185	
ENSG00000224299	1.407729925	0.697730409	pseudogene	2	202415247	202415772	
ENSG00000224301	0	0.390640832	antisense	10	64883698	64885297	
ENSG00000224303	0	0.390640832	lincRNA	9	43144980	43160008	
ENSG00000224304	0.499066092	0.390640832	lincRNA	9	36332947	36334969	
ENSG00000224308	0.869158192	1.802319292	antisense	1	152902516	152921686	
ENSG00000224312	1.407729925	1.802319292	pseudogene	6	29962214	29963091	MCCD1P2
ENSG00000224315	0.499066092	0.697730409	pseudogene	1	8846262	8846994	
ENSG00000224316	2.238690726	2.334191469	pseudogene	7	65238607	65267054	
ENSG00000224321	2.238690726	2.652276565	pseudogene	1	16119291	16119780	
ENSG00000224324	0.869158192	0.697730409	pseudogene	7	158415880	158416141	
ENSG00000224328	0.869158192	0.390640832	antisense	6	30670844	30680961	MDC1-AS1
ENSG00000224331	0	0.390640832	pseudogene	2	165543797	165544106	
ENSG00000224333	0.499066092	0.390640832	pseudogene	1	32867710	32868716	GAPDHP20
ENSG00000224340	0.869158192	0	pseudogene	1	10114503	10114839	
ENSG00000224341	0	0.390640832	pseudogene	X	118011849	118013148	
ENSG00000224342	0.499066092	0.390640832	lincRNA	2	208038358	208087514	
ENSG00000224345	3.146112541	2.579085888	antisense	6	88410228	88411243	AKIRIN2-AS1
ENSG00000224356	0.499066092	0.390640832	processed_transc	13	101189577	101232371	
ENSG00000224358	1.799000381	2.242360793	processed_transc	1	165738166	165744413	
ENSG00000224361	0	0.390640832	protein_coding	2	23729913	23747214	
ENSG00000224366	0.499066092	0	pseudogene	19	45073550	45073810	
ENSG00000224370	1.799000381	0.950786998	pseudogene	7	56448669	56449005	
ENSG00000224376	3.78787645	3.96493948	processed_transc	2	232253687	232259702	
ENSG00000224381	0.499066092	0	antisense	22	29867930	29874059	
ENSG00000224383	2.847891871	1.925536307	protein_coding	17	62075711	62081664	C17orf72
ENSG00000224387	0	0.390640832	antisense	1	2423739	2424697	
ENSG00000224388	0	0.390640832	antisense	21	42552024	42552553	BACE2-IT1
ENSG00000224389	2.671945279	3.411459265	protein_coding	6	31982539	32003195	C4B
ENSG00000224397	2.928422289	1.667587519	lincRNA	20	48884023	48896332	
ENSG00000224401	1.16343121	1.802319292	pseudogene	X	47700362	47701101	RPL7P57
ENSG00000224403	0	0.390640832	pseudogene	3	193711120	193711774	
ENSG00000224409	0.499066092	0	antisense	1	33183335	33190838	

ENSG00000224411	4.464551814	4.202780776	pseudogene	11	27910385	27912580	
ENSG00000224413	0.869158192	2.579085888	lincRNA	21	47456966	47459721	
ENSG00000224414	0.499066092	0	pseudogene	2	218558618	218561486	
ENSG00000224415	1.799000381	2.039052734	pseudogene	7	102781796	102782386	RPL19P12
ENSG00000224416	0	0.390640832	pseudogene	9	21278054	21278618	IFNA22P
ENSG00000224418	0.869158192	0.697730409	antisense	13	99229498	99231084	STK24-AS1
ENSG00000224419	0	0.390640832	pseudogene	13	90882638	90883936	KRT18P27
ENSG00000224420	5.485736881	5.072639111	protein_coding	19	50193095	50193750	C19orf76
ENSG00000224424	3.502389126	4.127774132	antisense	3	48885005	48889414	PRKAR2A-AS1
ENSG00000224427	1.960915222	1.925536307	pseudogene	21	34213806	34214121	
ENSG00000224428	5.695874189	6.515603638	processed_transc	10	81586459	81601328	
ENSG00000224429	2.10647801	2.242360793	lincRNA	13	21877651	21922860	LINC00422
ENSG00000224430	1.16343121	0.950786998	pseudogene	X	73380895	73382363	
ENSG00000224431	2.35979773	3.232099092	pseudogene	5	131535149	131539180	
ENSG00000224437	0	0.390640832	pseudogene	9	73531862	73532426	
ENSG00000224438	0.499066092	0	pseudogene	12	2891070	2891537	
ENSG00000224439	0.869158192	0	pseudogene	10	28725130	28726007	
ENSG00000224447	0.499066092	0	pseudogene	6	26016607	26016829	HIST1H1PS2
ENSG00000224448	0.499066092	0.390640832	pseudogene	7	99235865	99236390	
ENSG00000224450	0.499066092	0	pseudogene	3	197907620	197917237	
ENSG00000224451	0.499066092	0.697730409	pseudogene	13	52172542	52173930	ATP5F1P1
ENSG00000224452	1.799000381	1.802319292	pseudogene	20	30758193	30758593	RSL24D1P6
ENSG00000224458	1.616589159	0.390640832	pseudogene	7	63560683	63580922	GUSBP6
ENSG00000224460	1.407729925	0	lincRNA	6	143267184	143277835	
ENSG00000224462	0	0.697730409	pseudogene	1	207338841	207357255	C4BPAP1
ENSG00000224463	0	1.353254395	pseudogene	2	203096419	203096572	
ENSG00000224464	1.16343121	0	pseudogene	2	24095138	24095884	PGAM1P6
ENSG00000224467	0	0.390640832	antisense	2	161952742	162016735	
ENSG00000224468	2.238690726	2.788380093	processed_transc	1	183107514	183110439	
ENSG00000224470	9.407740769	9.336696989	protein_coding	16	71879894	71891231	ATXN1L
ENSG00000224471	0	0.390640832	pseudogene	X	102054491	102054814	
ENSG00000224473	0.499066092	0	pseudogene	10	30692126	30692705	
ENSG00000224474	0.869158192	0.390640832	protein_coding	10	99179228	99185831	
ENSG00000224476	0	0.390640832	pseudogene	19	41675766	41676071	
ENSG00000224479	1.407729925	0.390640832	pseudogene	3	63727969	63813121	
ENSG00000224481	0.499066092	0.697730409	lincRNA	1	147801124	147816764	
ENSG00000224489	3.004694206	2.334191469	processed_transc	10	121430230	121484200	
ENSG00000224490	1.16343121	1.518964905	processed_transc	2	166790367	166806401	
ENSG00000224502	1.16343121	1.16600992	sense_intronic	10	6221120	6222152	
ENSG00000224503	0	0.390640832	pseudogene	3	65573177	65573725	
ENSG00000224511	0	0.390640832	lincRNA	13	30677315	30683012	LINC00365
ENSG00000224514	0.499066092	0.697730409	antisense	3	13692196	13788132	
ENSG00000224520	2.35979773	1.518964905	pseudogene	1	157043049	157044494	KRT8P45
ENSG00000224531	8.546104025	9.001562612	protein_coding	6	11094039	11138964	C6orf228
ENSG00000224535	0.499066092	0	lincRNA	1	212640158	212641085	
ENSG00000224536	0.499066092	0.390640832	processed_transc	1	201476369	201502736	
ENSG00000224541	0	0.950786998	antisense	21	27530409	27548142	
ENSG00000224543	3.698846687	4.274079748	protein_coding	19	14599758	14600691	
ENSG00000224545	0.499066092	0	pseudogene	7	131204896	131205262	
ENSG00000224546	0.869158192	0.697730409	pseudogene	9	98908307	98910141	EIF4BP3
ENSG00000224549	0	0.697730409	lincRNA	9	22767174	22768315	
ENSG00000224550	0	0.950786998	pseudogene	1	108047825	108048070	
ENSG00000224551	0	0.390640832	pseudogene	7	96764327	96764873	HMGB3P21
ENSG00000224555	1.407729925	0	pseudogene	11	4010387	4010693	
ENSG00000224557	0	0.390640832	pseudogene	6	33080228	33102442	HLA-DPB2
ENSG00000224563	0.499066092	0.390640832	antisense	3	188280026	188286454	LPP-AS1
ENSG00000224565	0	0.390640832	lincRNA	20	46020673	46041071	
ENSG00000224566	0.869158192	0.390640832	pseudogene	1	228302703	228303174	
ENSG00000224574	0	0.390640832	antisense	21	46827301	46829980	COL18A1-AS2
ENSG00000224578	5.107569429	5.072639111	pseudogene	16	51679667	51681043	
ENSG00000224579	1.16343121	0.950786998	pseudogene	19	54712960	54713286	
ENSG00000224584	0	0.390640832	pseudogene	1	214786304	214786741	
ENSG00000224594	0	0.390640832	pseudogene	8	49297150	49297609	RPL29P19

ENSG00000224596	5.512107582	6.403479675	processed_transc	10	80703085	80828817	
ENSG00000224597	8.209916558	9.255345226	antisense	10	29698331	29776674	
ENSG00000224598	1.16343121	1.16600992	pseudogene	21	35507732	35508371	RPS5P2
ENSG00000224599	0.499066092	0	pseudogene	9	70083601	70087216	
ENSG00000224606	0.499066092	0	sense_intronic	2	70694517	70695627	TGFA-IT1
ENSG00000224609	0.499066092	0	processed_transc	1	59597608	59664293	
ENSG00000224613	0	0.390640832	antisense	1	103960701	104068115	
ENSG00000224614	2.10647801	2.334191469	processed_transc	3	195634947	195638128	
ENSG00000224616	0.869158192	1.667587519	antisense	1	100729565	100731676	
ENSG00000224621	2.35979773	2.242360793	antisense	1	16485761	16488378	
ENSG00000224628	0	0.390640832	pseudogene	20	29873120	29874638	
ENSG00000224629	1.407729925	1.667587519	pseudogene	7	157055716	157055923	
ENSG00000224631	4.352265886	4.961837817	pseudogene	16	61089349	61089818	
ENSG00000224635	4.962122459	5.175534283	lincRNA	20	37034658	37045444	
ENSG00000224638	0.499066092	0.390640832	lincRNA	2	174745593	174764156	
ENSG00000224644	0.869158192	0.697730409	antisense	9	114336472	114337707	
ENSG00000224647	2.10647801	1.16600992	pseudogene	17	7233771	7238732	
ENSG00000224649	0.499066092	0.697730409	antisense	21	30554348	30560116	
ENSG00000224652	1.960915222	0.390640832	processed_transc	3	195869507	195887761	
ENSG00000224655	0.499066092	1.16600992	lincRNA	2	122548020	122554278	
ENSG00000224660	5.107569429	5.372493443	processed_transc	3	15295691	15306000	
ENSG00000224666	0	0.950786998	antisense	6	36354608	36359771	
ENSG00000224668	1.16343121	1.925536307	pseudogene	1	211032519	211035627	IPO8P1
ENSG00000224669	0.499066092	0.390640832	pseudogene	7	64043111	64043585	
ENSG00000224671	0.869158192	0	antisense	1	203113092	203122028	
ENSG00000224672	0.869158192	0	pseudogene	2	201966105	201967859	
ENSG00000224677	0.499066092	0.390640832	pseudogene	15	43941321	43941478	
ENSG00000224678	0.869158192	0	pseudogene	1	92580359	92581362	GAPDHP46
ENSG00000224680	1.16343121	1.518964905	pseudogene	1	52834347	52834911	PLA2G12AP1
ENSG00000224682	1.799000381	1.925536307	pseudogene	9	138339292	138342000	
ENSG00000224683	0	0.697730409	pseudogene	7	16248570	16248890	
ENSG00000224684	0.499066092	0	antisense	9	118969286	118970488	
ENSG00000224687	2.238690726	3.32456471	processed_transc	1	178060643	178063119	
ENSG00000224699	1.16343121	1.518964905	antisense	1	110950147	110961033	
ENSG00000224707	0	0.390640832	sense_intronic	6	20438052	20440409	E2F3-IT1
ENSG00000224712	0	0.390640832	protein_coding	16	14802801	15045901	
ENSG00000224713	2.35979773	3.368666104	antisense	12	58005901	58011848	
ENSG00000224715	2.762599152	3.279072565	lincRNA	22	47741319	47769291	
ENSG00000224722	0.869158192	0.390640832	pseudogene	8	128098508	128099755	
ENSG00000224727	1.407729925	1.353254395	pseudogene	1	243430559	243431421	
ENSG00000224728	1.616589159	2.420525079	pseudogene	3	15919554	15921078	
ENSG00000224729	1.960915222	0	antisense	7	100187025	100201829	
ENSG00000224733	0	0.390640832	antisense	6	128821672	128822321	
ENSG00000224738	4.062237333	3.813624741	antisense	17	57183959	57195628	
ENSG00000224745	1.407729925	2.579085888	sense_intronic	10	89638449	89640184	
ENSG00000224746	0	1.16600992	processed_transc	7	135611203	135617629	
ENSG00000224747	2.238690726	1.518964905	pseudogene	21	45889812	45892399	
ENSG00000224750	0.499066092	0	lincRNA	10	92213927	92290873	
ENSG00000224751	0.499066092	1.518964905	pseudogene	1	44315974	44317405	SHMT1P1
ENSG00000224763	1.616589159	0.950786998	pseudogene	1	211833531	211834619	FDPSP8
ENSG00000224769	0.499066092	0	pseudogene	3	195341818	195347104	
ENSG00000224773	0	1.16600992	pseudogene	X	114009490	114011334	
ENSG00000224776	0	0.390640832	pseudogene	11	109852918	109853769	
ENSG00000224781	0	0.390640832	pseudogene	X	52861754	52862949	
ENSG00000224785	2.35979773	0	pseudogene	7	23602078	23603880	
ENSG00000224786	0.869158192	1.353254395	pseudogene	4	123651703	123653348	CETN4P
ENSG00000224789	0.499066092	0.390640832	antisense	2	120932461	120974120	
ENSG00000224790	1.407729925	1.925536307	lincRNA	21	38338812	38344128	
ENSG00000224791	0.869158192	0.950786998	pseudogene	2	204629018	204630316	
ENSG00000224792	0.499066092	0	pseudogene	3	51851619	51853651	
ENSG00000224794	0.499066092	1.353254395	antisense	22	39688993	39691568	
ENSG00000224798	0.499066092	0.390640832	sense_intronic	13	50351804	50354767	KPNA3-IT1
ENSG00000224799	1.407729925	1.802319292	antisense	X	56999943	57001229	

ENSG00000224800	0	0.697730409	pseudogene	1	150853712	150854159	
ENSG00000224802	0	0.950786998	pseudogene	9	34109199	34109966	
ENSG00000224803	0	0.390640832	pseudogene	9	66836847	66837138	
ENSG00000224804	0	0.697730409	antisense	7	74855312	74864233	
ENSG00000224805	0.869158192	1.518964905	antisense	1	47644922	47646011	
ENSG00000224809	0.869158192	0	pseudogene	9	94933974	94935776	
ENSG00000224812	0	0.697730409	processed_transc	10	45306472	45455137	TMEM72-AS1
ENSG00000224813	0	1.353254395	pseudogene	1	329784	453948	
ENSG00000224818	3.077135474	0.390640832	lincRNA	1	201433511	201434274	
ENSG00000224820	0	0.390640832	pseudogene	9	74722735	74723228	BTF3P4
ENSG00000224822	0	0.697730409	sense_intronic	3	24496627	24500925	THRB-IT1
ENSG00000224827	2.10647801	2.242360793	pseudogene	Y	26422392	26423173	LINC00265-2P
ENSG00000224831	2.471521042	1.16600992	pseudogene	3	149699965	149701095	
ENSG00000224834	0.499066092	0	pseudogene	1	227621765	227622047	BTF3P9
ENSG00000224837	0.499066092	1.16600992	pseudogene	1	168025139	168025660	
ENSG00000224839	0	0.697730409	pseudogene	2	208921972	208922469	
ENSG00000224843	2.762599152	4.406904905	processed_transc	6	26988232	26991703	LINC00240
ENSG00000224844	0	0.390640832	lincRNA	2	238031553	238032740	
ENSG00000224846	0	0.390640832	antisense	6	2989960	2999368	
ENSG00000224848	4.981125677	4.710890357	lincRNA	9	99449338	99483403	
ENSG00000224855	0	0.697730409	antisense	3	193336398	193345126	OPA1-AS1
ENSG00000224856	0.499066092	0.697730409	pseudogene	22	44352907	44353222	
ENSG00000224857	0.499066092	0.697730409	lincRNA	1	121315744	121322322	
ENSG00000224858	0	0.950786998	pseudogene	3	37058014	37058505	
ENSG00000224861	1.960915222	1.925536307	pseudogene	14	66479548	66480507	YBX1P1
ENSG00000224863	1.799000381	1.518964905	processed_transc	1	46912345	46915376	
ENSG00000224864	0	0.697730409	pseudogene	19	20235248	20236293	
ENSG00000224870	6.068837212	6.3217081	processed_transc	1	1334902	1337426	
ENSG00000224877	7.326727684	7.764467022	protein_coding	17	79213111	79215098	C17orf89
ENSG00000224881	2.57521082	3.133298822	pseudogene	2	87607003	87607890	
ENSG00000224885	0.869158192	1.353254395	sense_intronic	2	3302112	3305236	TSSC1-IT1
ENSG00000224886	3.146112541	3.411459265	pseudogene	10	81444248	81444739	
ENSG00000224888	5.054730169	5.361622823	antisense	16	88797588	88807826	
ENSG00000224890	0	0.390640832	pseudogene	9	123838730	123839154	
ENSG00000224891	0.499066092	1.667587519	pseudogene	2	37375673	37376447	
ENSG00000224892	1.799000381	0.697730409	pseudogene	13	52034661	52035532	RPS4XP16
ENSG00000224897	3.448790144	3.714580548	processed_transc	7	124569927	124819369	
ENSG00000224899	0.499066092	0	lincRNA	7	124791380	124793665	
ENSG00000224900	1.799000381	1.802319292	sense_intronic	1	206576565	206577294	
ENSG00000224903	2.471521042	1.16600992	antisense	7	156446879	156450387	
ENSG00000224905	2.35979773	3.279072565	processed_transc	21	15399742	15516789	
ENSG00000224906	0	0.390640832	pseudogene	12	6547664	6548018	
ENSG00000224908	0.499066092	0	pseudogene	X	133300712	133300962	TIMM8BP2
ENSG00000224914	3.211941663	4.202780776	lincRNA	10	89102681	89115187	
ENSG00000224916	0	0.390640832	protein_coding	19	45445495	45452820	APOC4
ENSG00000224927	0	0.390640832	pseudogene	1	110353266	110353620	
ENSG00000224928	0.869158192	0.950786998	pseudogene	2	219818123	219818772	KRT8P30
ENSG00000224930	1.407729925	0	lincRNA	21	46419122	46424642	LINC00162
ENSG00000224932	0.869158192	1.16600992	lincRNA	4	118235753	118281795	
ENSG00000224934	2.10647801	1.802319292	antisense	10	101190948	101195542	
ENSG00000224939	1.16343121	1.518964905	lincRNA	1	234765057	234770526	LINC00184
ENSG00000224940	1.16343121	0.390640832	protein_coding	7	127990379	128001739	PRRT4
ENSG00000224945	3.871730003	2.144285137	sense_intronic	9	91703519	91724849	
ENSG00000224949	0	0.390640832	pseudogene	X	54173805	54174062	
ENSG00000224950	3.335220907	3.781359661	processed_transc	1	117035645	117041834	
ENSG00000224956	6.75547892	6.252780742	pseudogene	1	661611	663527	
ENSG00000224959	4.437288965	4.528525526	processed_transc	2	112249520	112252388	
ENSG00000224961	1.16343121	0.697730409	lincRNA	20	10733738	10734614	
ENSG00000224962	1.16343121	0.950786998	pseudogene	3	166408975	166409503	
ENSG00000224965	0	0.390640832	antisense	1	110751456	110752609	KCNC4-AS1
ENSG00000224967	1.16343121	0	pseudogene	2	118691601	118692365	
ENSG00000224969	2.471521042	1.667587519	antisense	1	947377	948573	
ENSG00000224972	0	0.697730409	processed_transc	9	6902670	6978859	

ENSG00000224973	0	0.390640832	processed_transc	22	34121000	34146830	
ENSG00000224975	4.75690578	4.841813558	protein_coding	X	47064320	47065264	INE1
ENSG00000224976	0.499066092	0.697730409	pseudogene	13	19923276	19982102	PARP4P2
ENSG00000224977	0	0.390640832	lincRNA	1	173991647	173992123	
ENSG00000224981	0.499066092	0	pseudogene	7	126508821	126510586	
ENSG00000224986	0.499066092	0.697730409	pseudogene	1	48790762	48791795	PPP1R8P1
ENSG00000224988	0.499066092	0	pseudogene	9	132645972	132646449	
ENSG00000224992	2.238690726	1.925536307	sense_intronic	9	135644840	135645599	
ENSG00000224993	0.869158192	0.950786998	pseudogene	5	43666867	43667334	
ENSG00000224997	4.437288965	3.532712221	protein_coding	14	104177607	104179149	
ENSG00000224999	0	0.390640832	pseudogene	6	169696566	169698054	
ENSG00000225014	0.499066092	0	pseudogene	17	20015137	20016303	KCTD9P1
ENSG00000225015	0.499066092	0.697730409	pseudogene	1	142660017	142660749	SNX18P15
ENSG00000225016	0.869158192	0	pseudogene	13	19383970	19384188	LINC00328-2P
ENSG00000225017	0	0.390640832	pseudogene	8	100358280	100360484	
ENSG00000225022	1.407729925	0.697730409	pseudogene	20	5273034	5273477	UBE2D3P1
ENSG00000225026	0.499066092	0	pseudogene	3	12369502	12369773	
ENSG00000225028	0	0.390640832	lincRNA	1	48283738	48285909	
ENSG00000225030	1.16343121	0.390640832	sense_intronic	1	53757463	53757810	
ENSG00000225031	4.352265886	4.250702764	pseudogene	X	110862905	110864717	EIF4BP7
ENSG00000225032	5.375152132	5.137803333	antisense	9	130578345	130584799	
ENSG00000225037	1.16343121	1.16600992	antisense	X	20158086	20158562	EIF1AX-AS1
ENSG00000225039	0.499066092	0.390640832	lincRNA	13	30993656	30996374	
ENSG00000225043	1.16343121	1.16600992	pseudogene	21	47840467	47840948	RPL18AP2
ENSG00000225051	1.616589159	1.518964905	pseudogene	5	179106033	179121782	HMGB3P22
ENSG00000225057	0.499066092	0	processed_transc	2	239140325	239164274	
ENSG00000225059	1.16343121	0.697730409	pseudogene	6	31351824	31352193	
ENSG00000225062	0	0.950786998	processed_transc	2	219231388	219232558	
ENSG00000225065	0	0.697730409	antisense	20	33284722	33302432	
ENSG00000225067	0.869158192	1.16600992	pseudogene	19	15722467	15722934	
ENSG00000225071	0	0.390640832	pseudogene	X	24447690	24448037	
ENSG00000225075	0.869158192	2.501982735	antisense	1	113236310	113239243	
ENSG00000225076	0.499066092	0.390640832	antisense	X	9992882	10006694	WWC3-AS1
ENSG00000225077	3.448790144	3.532712221	antisense	1	6296300	6299504	LINC00337
ENSG00000225082	0.869158192	0.697730409	pseudogene	1	155556435	155571988	DAP3P1
ENSG00000225083	0.499066092	0	antisense	13	114005988	114016183	GRTP1-AS1
ENSG00000225084	0.869158192	0	antisense	17	2135974	2136904	
ENSG00000225087	0.499066092	0	lincRNA	1	73214604	73364823	
ENSG00000225092	1.16343121	0.697730409	processed_transc	6	7183316	7185520	
ENSG00000225093	3.004694206	2.971122874	pseudogene	6	108325561	108326772	
ENSG00000225094	1.16343121	1.16600992	pseudogene	4	110474478	110475250	SETP20
ENSG00000225099	0.499066092	0	pseudogene	1	43368903	43369583	ATP6V1E1P1
ENSG00000225100	0.499066092	0	pseudogene	10	81742671	81742996	
ENSG00000225111	0.499066092	0	sense_intronic	2	208733306	208734639	
ENSG00000225113	1.799000381	1.518964905	antisense	1	110138847	110140403	
ENSG00000225118	0.499066092	0	pseudogene	10	94637849	94638046	
ENSG00000225119	5.752272083	5.564846512	processed_transc	10	38692117	38737700	
ENSG00000225124	0.499066092	0	pseudogene	2	203939408	203939865	
ENSG00000225125	2.238690726	3.081239798	pseudogene	X	135889424	135890036	RANP4
ENSG00000225131	1.616589159	1.16600992	pseudogene	13	49345264	49345965	PSME2P2
ENSG00000225135	4.712111592	4.872768657	lincRNA	6	147708800	147711601	
ENSG00000225137	0.869158192	1.16600992	pseudogene	10	52024738	52026575	
ENSG00000225138	7.102117104	8.501254594	antisense	5	478238	480999	
ENSG00000225140	0.869158192	1.353254395	antisense	10	674578	677195	
ENSG00000225146	1.16343121	1.16600992	antisense	7	1068661	1083527	
ENSG00000225151	7.715131365	7.862183683	pseudogene	15	84867600	84898890	
ENSG00000225156	0.499066092	0	antisense	2	45181803	45195901	
ENSG00000225157	0	0.697730409	pseudogene	19	45687584	45688190	
ENSG00000225159	2.10647801	1.925536307	pseudogene	1	27533421	27534287	
ENSG00000225160	1.960915222	1.16600992	lincRNA	20	25982172	25982935	
ENSG00000225163	1.407729925	1.353254395	lincRNA	14	97409916	97411731	C14orf63
ENSG00000225165	0.869158192	0.390640832	pseudogene	5	94775748	94776391	
ENSG00000225169	0.499066092	0.390640832	pseudogene	1	100678849	100679226	BRI3P1

ENSG00000225170	1.16343121	0	pseudogene	20	22252690	22254092	
ENSG00000225171	2.35979773	1.518964905	pseudogene	1	166837985	166838447	DUTP6
ENSG00000225173	1.960915222	2.039052734	lincRNA	6	28805646	28806783	
ENSG00000225177	3.335220907	3.453019579	antisense	6	139013685	139018425	
ENSG00000225178	0	0.697730409	pseudogene	16	73974538	73975423	RPSAP56
ENSG00000225180	1.16343121	0	antisense	17	79139307	79156964	AATK-AS1
ENSG00000225185	0.499066092	0.390640832	pseudogene	12	98984501	98984992	
ENSG00000225187	0.869158192	0.390640832	antisense	2	47294961	47298343	
ENSG00000225190	8.646233023	8.755966294	protein_coding	17	43513267	43568146	PLEKHM1
ENSG00000225192	0.499066092	1.667587519	pseudogene	10	38083204	38085037	
ENSG00000225193	2.471521042	1.925536307	pseudogene	15	90749804	90750195	RPS12P26
ENSG00000225200	2.928422289	2.579085888	pseudogene	14	106444607	106445233	
ENSG00000225205	3.698846687	3.64455972	antisense	2	173345568	173421324	
ENSG00000225206	0.499066092	1.353254395	pseudogene	1	98508913	98511014	MIR2682
ENSG00000225210	7.127924785	8.721307575	lincRNA	14	19650018	19718563	
ENSG00000225213	0	0.390640832	antisense	10	16763436	16765207	
ENSG00000225215	0	0.697730409	pseudogene	11	107274130	107275315	SMARCE1P1
ENSG00000225218	2.847891871	2.721932731	lincRNA	21	44251150	44256587	
ENSG00000225222	0	0.697730409	pseudogene	1	246342080	246342507	
ENSG00000225224	1.616589159	1.353254395	pseudogene	7	156985062	156985526	
ENSG00000225225	2.10647801	1.667587519	pseudogene	22	18585223	18586415	ARL2BPP10
ENSG00000225230	0.869158192	1.16600992	antisense	5	56195868	56206541	
ENSG00000225234	1.960915222	2.579085888	antisense	2	3485013	3486180	TRAPPC12-AS1
ENSG00000225235	0.499066092	0	antisense	X	134654008	134654599	DDX26B-AS1
ENSG00000225241	8.088119026	8.486319932	lincRNA	1	144593363	144621656	NBPF8
ENSG00000225251	0	0.390640832	pseudogene	10	13099902	13100676	
ENSG00000225254	0	0.390640832	pseudogene	9	38452786	38454067	
ENSG00000225259	0.869158192	0.697730409	pseudogene	8	134420249	134421471	ST13P6
ENSG00000225261	2.57521082	0.950786998	antisense	X	148629755	148631380	
ENSG00000225264	3.989024711	3.232099092	processed_transc	7	29638411	29724871	
ENSG00000225265	2.671945279	3.232099092	antisense	1	222763167	222766374	
ENSG00000225267	0	0.390640832	pseudogene	21	31635698	31636199	RPL8P2
ENSG00000225269	2.847891871	0.950786998	lincRNA	10	4697619	4704611	
ENSG00000225272	0	0.697730409	pseudogene	1	165645547	165646015	
ENSG00000225278	1.960915222	2.144285137	processed_transc	1	143211528	143275032	
ENSG00000225285	3.871730003	4.622587519	lincRNA	1	1365919	1369953	
ENSG00000225286	0	1.353254395	pseudogene	7	28654714	28655505	
ENSG00000225297	0.499066092	0.390640832	pseudogene	1	93665312	93665496	
ENSG00000225300	1.960915222	2.144285137	sense_intronic	1	246769175	246771404	
ENSG00000225302	2.847891871	3.368666104	antisense	10	118592512	118609716	
ENSG00000225306	1.407729925	0.697730409	antisense	1	36057977	36059256	
ENSG00000225308	1.16343121	1.353254395	pseudogene	7	21259832	21261047	ASS1P11
ENSG00000225311	0	0.390640832	antisense	6	144632835	144654437	
ENSG00000225313	1.16343121	2.144285137	antisense	1	33780296	33791644	
ENSG00000225331	2.762599152	2.971122874	lincRNA	21	45578623	45579959	
ENSG00000225333	0	0.390640832	pseudogene	1	40183700	40184268	
ENSG00000225335	1.16343121	2.144285137	antisense	22	18559293	18560563	
ENSG00000225336	0	0.390640832	pseudogene	20	33421378	33422265	HMGB3P1
ENSG00000225338	1.616589159	1.353254395	pseudogene	1	212482393	212482860	
ENSG00000225339	10.0120557	9.327648427	processed_transc	6	34247482	34252266	
ENSG00000225345	3.554067925	4.585695337	pseudogene	9	38566257	38568208	SNX18P3
ENSG00000225347	0.869158192	0.390640832	pseudogene	9	32333455	32334352	
ENSG00000225353	0.499066092	0.697730409	lincRNA	9	46270786	46274928	
ENSG00000225356	0.499066092	0	pseudogene	4	182443813	182444154	
ENSG00000225357	0.499066092	0.390640832	pseudogene	20	34340736	34341514	
ENSG00000225358	0.499066092	0	pseudogene	7	112375221	112378748	MIPEPP1
ENSG00000225361	3.211941663	3.232099092	antisense	9	138359934	138364095	
ENSG00000225362	0.869158192	0.697730409	protein_coding	15	71402583	71407833	CT62
ENSG00000225364	0.499066092	0.390640832	pseudogene	5	132284185	132284410	ATP6V0E1P1
ENSG00000225371	1.799000381	0	pseudogene	7	56430151	56432963	CICP8
ENSG00000225373	4.883502971	5.072639111	protein_coding	19	65917	70966	
ENSG00000225374	1.407729925	1.802319292	antisense	X	13785519	13785984	
ENSG00000225377	4.800350936	5.187895122	antisense	20	300957	328868	

ENSG00000225383	6.17992145	5.816437314	lincRNA	10	10826402	10836943	SFTA1P
ENSG00000225385	1.799000381	1.353254395	pseudogene	9	90681448	90682936	
ENSG00000225393	0.499066092	0	antisense	X	154697947	154716707	
ENSG00000225399	0.869158192	2.039052734	protein_coding	3	49297518	49298749	
ENSG00000225400	1.799000381	2.039052734	pseudogene	X	135929543	135930193	
ENSG00000225401	0.499066092	0.390640832	pseudogene	1	244558278	244558962	TGIF2P1
ENSG00000225402	0.499066092	0.390640832	pseudogene	2	38044058	38045104	
ENSG00000225405	2.57521082	2.420525079	pseudogene	4	62971378	62971767	
ENSG00000225406	0.499066092	0.390640832	pseudogene	2	186411862	186412545	
ENSG00000225407	0.499066092	0	lincRNA	5	75987264	76012040	
ENSG00000225411	1.407729925	1.667587519	processed_transc	9	68397878	68401759	
ENSG00000225416	0.499066092	1.16600992	pseudogene	7	152299298	152299977	
ENSG00000225419	0	1.353254395	pseudogene	20	18465909	18466696	RPL21P3
ENSG00000225420	0.499066092	0	antisense	2	88838238	88875128	
ENSG00000225422	0.499066092	0	pseudogene	12	66627862	66629014	RBMS1P1
ENSG00000225423	0.869158192	0.390640832	pseudogene	10	86150664	86151801	
ENSG00000225425	0.499066092	0.950786998	antisense	9	132677198	132677715	
ENSG00000225433	0.499066092	0.390640832	pseudogene	9	42779796	42779998	MTND1P12
ENSG00000225434	2.57521082	2.788380093	processed_transc	9	74920345	74958126	
ENSG00000225436	4.261916566	5.085909553	antisense	17	16229799	16250988	
ENSG00000225439	3.146112541	3.679994897	processed_transc	2	74375136	74377188	BOLA3-AS1
ENSG00000225442	0.869158192	0	antisense	17	16979352	16980672	
ENSG00000225443	0.499066092	0	pseudogene	7	44550624	44550931	
ENSG00000225445	0.869158192	0.390640832	processed_transc	17	42384668	42392683	
ENSG00000225447	0.499066092	1.353254395	pseudogene	1	46111488	46111869	RPS15AP10
ENSG00000225450	0.869158192	1.518964905	antisense	22	39135008	39145046	
ENSG00000225451	0	0.390640832	pseudogene	7	63336719	63337728	
ENSG00000225460	0	0.697730409	pseudogene	9	92087152	92087607	
ENSG00000225461	1.16343121	0.390640832	antisense	10	112257757	112258300	
ENSG00000225462	0	0.697730409	pseudogene	1	187532193	187533263	FDPSP1
ENSG00000225465	0.869158192	0.390640832	antisense	22	29833006	29838118	RFPL1-AS1
ENSG00000225470	7.1740722	7.18590274	lincRNA	X	73164177	73220326	JPX
ENSG00000225471	3.911897206	3.532712221	pseudogene	X	71364034	71381600	
ENSG00000225472	1.407729925	0.697730409	antisense	9	14317084	14357907	
ENSG00000225473	0	0.390640832	antisense	3	193271002	193272877	ATP13A4-AS1
ENSG00000225474	1.16343121	2.334191469	antisense	13	35621967	35623401	NBEA-AS1
ENSG00000225475	0.499066092	0	pseudogene	1	57085082	57085222	
ENSG00000225483	0	0.390640832	pseudogene	1	206180054	206180443	
ENSG00000225484	5.458875168	5.726093467	processed_transc	10	81563813	81586350	
ENSG00000225485	9.625684414	8.099860884	protein_coding	17	36584720	36668628	ARHGAP23
ENSG00000225486	0.869158192	0.390640832	pseudogene	1	233971826	233972178	
ENSG00000225492	1.960915222	1.518964905	pseudogene	1	89873238	89891802	GBP1P1
ENSG00000225493	1.16343121	2.501982735	lincRNA	2	239419331	239463695	
ENSG00000225495	0.499066092	0.390640832	processed_transc	1	75018088	75218059	
ENSG00000225496	0.869158192	0	pseudogene	2	112697879	112698613	
ENSG00000225498	0.499066092	0.697730409	lincRNA	7	89941810	89951906	
ENSG00000225505	0.499066092	0.950786998	pseudogene	1	93197557	93198814	
ENSG00000225506	0.499066092	0	lincRNA	1	47562325	47644943	
ENSG00000225507	4.689181911	4.127774132	pseudogene	7	47996390	47996915	
ENSG00000225511	1.407729925	0	processed_transc	9	94903580	94921890	LINC00475
ENSG00000225513	0	0.697730409	pseudogene	9	114935802	114936251	
ENSG00000225515	0.869158192	0.950786998	antisense	6	47495926	47496397	
ENSG00000225518	2.471521042	2.144285137	processed_transc	1	226274582	226277993	
ENSG00000225521	0.499066092	0	antisense	2	241955016	242003533	
ENSG00000225526	2.762599152	2.420525079	protein_coding	3	12556433	12602558	MKRN2-AS1
ENSG00000225527	0.499066092	0.697730409	antisense	10	18820778	18822265	
ENSG00000225531	0.869158192	0	pseudogene	9	109879110	109879838	
ENSG00000225532	0.499066092	0	lincRNA	6	171045261	171046865	
ENSG00000225533	0	0.390640832	pseudogene	10	96943410	96943974	
ENSG00000225535	1.407729925	0.950786998	lincRNA	7	114719012	114766368	
ENSG00000225536	0.499066092	0.697730409	pseudogene	X	85339719	85341344	
ENSG00000225541	2.928422289	2.652276565	processed_transc	7	22611226	22701411	
ENSG00000225544	1.616589159	1.353254395	pseudogene	22	22239621	22240012	

ENSG00000225549	0.499066092	0	pseudogene	6	119078681	119104583	
ENSG00000225551	0	0.390640832	pseudogene	9	95301335	95301466	
ENSG00000225554	0	0.390640832	lincRNA	1	241587592	241596792	
ENSG00000225555	0	0.390640832	antisense	21	35743101	35747647	
ENSG00000225556	2.35979773	2.652276565	protein_coding	1	151810295	151813033	C2CD4D
ENSG00000225558	0.499066092	0.950786998	pseudogene	3	37176149	37176593	UBE2D3P2
ENSG00000225568	0.499066092	0	pseudogene	1	87511558	87512383	
ENSG00000225572	1.16343121	1.667587519	antisense	7	111448572	111461829	
ENSG00000225573	1.16343121	1.353254395	pseudogene	7	66071725	66072094	RPL35P5
ENSG00000225578	2.10647801	2.420525079	antisense	3	196666748	196669405	NCBP2-AS1
ENSG00000225580	1.16343121	0.950786998	pseudogene	6	112937905	112939082	
ENSG00000225591	0.499066092	0.390640832	pseudogene	1	173710813	173711062	
ENSG00000225594	0.499066092	0	pseudogene	2	132844862	132860137	
ENSG00000225595	0	0.697730409	processed_transc	6	28827402	28831127	
ENSG00000225602	0.499066092	0.390640832	antisense	1	11203955	11209594	MTOR-AS1
ENSG00000225603	1.16343121	0.390640832	pseudogene	1	146522379	146524043	
ENSG00000225606	0.499066092	0	antisense	7	12609751	12611017	
ENSG00000225611	0	1.16600992	antisense	3	42812104	42815127	
ENSG00000225614	3.211941663	2.971122874	protein_coding	16	88493879	88507165	ZNF469
ENSG00000225616	4.062237333	4.385599336	pseudogene	1	29308796	29309122	
ENSG00000225620	0	0.390640832	lincRNA	1	202601556	202602039	
ENSG00000225627	0.869158192	0.390640832	antisense	1	40747986	40749177	
ENSG00000225630	3.830412367	3.081239798	pseudogene	1	565020	566063	MTND2P28
ENSG00000225636	0	0.390640832	pseudogene	2	197879714	197880739	
ENSG00000225643	1.960915222	2.579085888	lincRNA	1	25907969	25916847	
ENSG00000225647	0.499066092	0	lincRNA	7	108238819	108254711	
ENSG00000225648	7.947856304	7.86795666	pseudogene	7	72300004	72307909	SBDSP1
ENSG00000225650	0	0.390640832	pseudogene	1	115010937	115011900	EIF2S2P5
ENSG00000225656	0	0.390640832	lincRNA	1	230959387	230960453	
ENSG00000225663	5.431503808	5.931169853	protein_coding	17	79780293	79791167	FAM195B
ENSG00000225665	0.499066092	0	pseudogene	4	121263211	121263802	
ENSG00000225666	3.744048221	3.570966319	antisense	7	156461646	156469257	
ENSG00000225667	3.004694206	3.532712221	lincRNA	1	46921495	46948147	LINC00505
ENSG00000225670	0.499066092	0	antisense	1	159165775	159172177	
ENSG00000225674	0.869158192	1.16600992	pseudogene	13	24696204	24697953	IPO7P2
ENSG00000225675	0.869158192	1.667587519	processed_transc	1	53793921	53802890	
ENSG00000225676	0	0.950786998	antisense	22	30580633	30603098	
ENSG00000225678	0	0.390640832	pseudogene	11	102621801	102625674	
ENSG00000225684	3.911897206	3.845183986	processed_transc	9	115865618	115873780	LINC00256B
ENSG00000225693	1.16343121	2.501982735	pseudogene	9	33019680	33020163	
ENSG00000225695	0.869158192	0.950786998	pseudogene	2	201927812	201928774	
ENSG00000225697	9.362260424	9.582956544	protein_coding	3	48663156	48672926	SLC26A6
ENSG00000225704	0.499066092	0	pseudogene	1	171314228	171314459	
ENSG00000225711	0	0.390640832	pseudogene	1	179190073	179191260	
ENSG00000225715	0.499066092	0.390640832	pseudogene	X	79965427	79966204	
ENSG00000225720	1.16343121	0	pseudogene	22	39487591	39488860	
ENSG00000225721	1.960915222	1.518964905	antisense	1	45224709	45241482	
ENSG00000225724	0	0.697730409	pseudogene	22	22693926	22695567	
ENSG00000225725	1.16343121	1.16600992	processed_transc	8	7812536	7848219	FAM66E
ENSG00000225726	1.16343121	2.242360793	pseudogene	7	76701068	76701554	
ENSG00000225733	9.064459359	9.206061322	antisense	3	14961854	14989931	FGD5-AS1
ENSG00000225742	0.869158192	0	lincRNA	3	193920805	193967942	
ENSG00000225744	0	0.697730409	lincRNA	2	112365418	112370095	
ENSG00000225745	0.499066092	0.390640832	processed_transc	21	42548249	42558715	
ENSG00000225750	0	0.390640832	lincRNA	1	29471006	29473506	
ENSG00000225751	0	0.950786998	antisense	17	9364561	9373331	
ENSG00000225755	0	0.697730409	pseudogene	1	163207004	163209345	
ENSG00000225756	6.386460534	6.030599799	antisense	9	136519708	136522435	DBH-AS1
ENSG00000225760	3.393122761	1.925536307	lincRNA	13	111635654	111642911	
ENSG00000225761	2.35979773	0.950786998	sense_intronic	10	75764895	75787673	
ENSG00000225762	3.744048221	4.075511708	antisense	1	47846600	47874149	
ENSG00000225764	2.35979773	1.802319292	antisense	3	189838753	189862635	LEPREL1-AS1
ENSG00000225766	2.10647801	1.353254395	pseudogene	14	24505662	24520237	

ENSG00000225767	0	0.390640832	antisense	1	50927141	50936822	
ENSG00000225770	0.499066092	0.950786998	pseudogene	3	196263403	196263573	
ENSG00000225771	0.869158192	0.390640832	antisense	1	62580420	62584681	
ENSG00000225774	0.499066092	0	pseudogene	22	30938523	30940292	SIRPAP1
ENSG00000225778	3.603959378	3.232099092	antisense	10	11891612	11936699	
ENSG00000225779	0.869158192	0	pseudogene	1	46836258	46836627	
ENSG00000225783	3.744048221	4.021284656	processed_transc	22	27042392	27072438	MIAT
ENSG00000225790	2.10647801	3.570966319	lincRNA	3	177652611	177655450	
ENSG00000225791	2.671945279	2.721932731	processed_transc	6	52442283	52444325	
ENSG00000225792	0.869158192	1.353254395	antisense	7	26411764	26416321	
ENSG00000225793	0	0.950786998	processed_transc	6	75994730	76006283	
ENSG00000225796	0.869158192	0.950786998	pseudogene	2	202078021	202079383	MTND4P23
ENSG00000225798	0.499066092	1.353254395	antisense	15	59182166	59186281	
ENSG00000225805	0	0.697730409	pseudogene	11	71589499	71595607	
ENSG00000225806	0	0.697730409	antisense	20	57438583	57463881	
ENSG00000225807	0	0.390640832	lincRNA	7	100107340	100117549	
ENSG00000225808	3.146112541	2.039052734	pseudogene	2	178093919	178094234	
ENSG00000225810	0.869158192	0.950786998	processed_transc	2	42592810	42652228	
ENSG00000225813	2.35979773	3.87606762	pseudogene	2	162138413	162139129	
ENSG00000225820	1.799000381	1.518964905	sense_intronic	1	8066074	8066784	ERRFI1-IT1
ENSG00000225822	0	0.390640832	antisense	3	196158256	196159401	UBXN7-AS1
ENSG00000225823	0	0.390640832	pseudogene	13	105466678	105467420	RPL7P45
ENSG00000225826	0.869158192	0.390640832	lincRNA	1	168756177	168762124	
ENSG00000225828	4.097495944	3.813624741	protein_coding	1	32826871	32829879	
ENSG00000225830	8.116326397	8.529454305	protein_coding	10	50663414	50747584	ERCC6
ENSG00000225832	0	0.390640832	pseudogene	6	20722559	20723886	
ENSG00000225836	0.499066092	0	lincRNA	10	91406046	91410579	
ENSG00000225839	1.16343121	0.390640832	antisense	X	100297573	100298694	TRMT2B-AS1
ENSG00000225840	4.568725997	4.528525526	pseudogene	Y	10036113	10036711	RN18S1
ENSG00000225849	0.499066092	0	pseudogene	20	45092541	45093763	MKRN7P
ENSG00000225850	0	1.353254395	antisense	10	99094321	99102960	
ENSG00000225854	0.869158192	0	pseudogene	1	26653178	26653530	
ENSG00000225855	6.721980839	6.376734172	antisense	1	155286654	155293967	RUSC1-AS1
ENSG00000225857	0	0.697730409	lincRNA	1	180875787	180878302	
ENSG00000225864	0.499066092	0.390640832	pseudogene	6	29690758	29691748	HCG4P11
ENSG00000225865	0.869158192	1.802319292	lincRNA	20	42418584	42455002	
ENSG00000225867	1.16343121	0	antisense	22	37946033	37947742	
ENSG00000225868	1.407729925	0.950786998	lincRNA	19	38315798	38345836	
ENSG00000225869	0.869158192	0	pseudogene	5	143131663	143132269	
ENSG00000225871	0	1.667587519	pseudogene	1	147907215	147909056	
ENSG00000225872	0.499066092	0	processed_transc	19	36279778	36288787	
ENSG00000225874	0	0.697730409	processed_transc	6	38141449	38145069	
ENSG00000225877	0.499066092	2.039052734	lincRNA	19	43326015	43331030	
ENSG00000225880	3.077135474	3.32456471	lincRNA	1	761586	762902	LINC00115
ENSG00000225885	0	0.390640832	protein_coding	8	119294481	119412844	
ENSG00000225886	3.211941663	4.127774132	antisense	1	27995979	27996787	
ENSG00000225889	0.499066092	1.353254395	processed_transc	2	64370373	64479993	
ENSG00000225891	0	0.390640832	antisense	1	26789247	26793773	
ENSG00000225892	4.77879189	4.676215056	pseudogene	4	119553429	119555914	
ENSG00000225899	0	0.390640832	protein_coding	10	135437399	135440299	FRG2B
ENSG00000225905	0.869158192	0	antisense	1	1355625	1358071	
ENSG00000225911	0	0.390640832	pseudogene	2	220913543	220913993	
ENSG00000225912	0.499066092	0	pseudogene	X	91931518	91931953	
ENSG00000225913	1.407729925	2.144285137	processed_transc	10	89367742	89419036	
ENSG00000225914	1.16343121	1.667587519	antisense	6	32223488	32233615	
ENSG00000225915	0.869158192	1.16600992	antisense	20	39809096	39825427	
ENSG00000225916	0.499066092	0.950786998	lincRNA	2	208091673	208093032	
ENSG00000225918	0.499066092	0.697730409	pseudogene	7	144737092	144738034	
ENSG00000225920	2.35979773	2.788380093	pseudogene	1	219373258	219373909	RIMKLBP2
ENSG00000225921	9.320904342	9.731784619	protein_coding	6	13615559	13632971	NOL7
ENSG00000225928	0	0.390640832	pseudogene	10	86214441	86215812	
ENSG00000225929	0.499066092	0.697730409	antisense	22	51174253	51176567	
ENSG00000225931	0	0.950786998	antisense	1	2497849	2508811	

ENSG00000225933	0	0.390640832	pseudogene	2	87954409	87954762	
ENSG00000225934	0	0.390640832	pseudogene	1	227786128	227786795	
ENSG00000225938	0	0.390640832	antisense	1	101701239	101702084	
ENSG00000225940	0.499066092	0.390640832	protein_coding	5	55807394	55902059	
ENSG00000225946	2.238690726	0.950786998	processed_transc	7	100606878	100611410	
ENSG00000225950	0.499066092	0	protein_coding	19	49559050	49568333	
ENSG00000225951	0.499066092	1.16600992	antisense	9	131231236	131235291	
ENSG00000225953	1.616589159	2.039052734	processed_transc	2	200322423	200341658	
ENSG00000225963	0	1.802319292	antisense	2	231036839	231038822	
ENSG00000225964	1.960915222	1.353254395	antisense	2	6968645	6980595	
ENSG00000225965	0.499066092	0	pseudogene	10	38450498	38450677	
ENSG00000225968	2.57521082	3.027231696	protein_coding	7	1727755	1787590	ELFN1
ENSG00000225969	1.799000381	2.144285137	antisense	7	73149368	73150384	ABHD11-AS1
ENSG00000225971	0.499066092	0.950786998	pseudogene	22	30532662	30533670	
ENSG00000225972	4.903563137	4.17820932	pseudogene	1	564442	564813	MTND1P23
ENSG00000225973	5.239686603	4.826083323	antisense	15	55609382	55611359	
ENSG00000225975	2.10647801	1.16600992	antisense	19	37176216	37178351	
ENSG00000225978	3.78787645	4.048652948	processed_transc	20	61733557	61735738	HAR1A
ENSG00000225981	0.869158192	1.518964905	antisense	7	1499573	1503644	
ENSG00000225984	0.499066092	0	pseudogene	1	229840981	229841608	HMGB1P26
ENSG00000225985	1.16343121	1.667587519	antisense	13	100143537	100144751	
ENSG00000225986	1.407729925	1.16600992	antisense	1	20510735	20512979	
ENSG00000226002	2.471521042	2.971122874	pseudogene	7	64544481	64560610	
ENSG00000226009	1.407729925	2.420525079	antisense	10	103578835	103588536	
ENSG00000226015	2.928422289	2.652276565	pseudogene	1	146674871	146676513	CCT8P1
ENSG00000226017	0.869158192	0.697730409	antisense	3	64173220	64186641	PRICKLE2-AS3
ENSG00000226025	0.499066092	0.390640832	lincRNA	19	40170014	40185217	
ENSG00000226026	0.869158192	0.390640832	antisense	1	95628775	95699538	
ENSG00000226028	0.499066092	0.390640832	pseudogene	1	149114655	149115188	PFN1P12
ENSG00000226029	3.393122761	3.32456471	lincRNA	1	16787443	16794976	
ENSG00000226036	0	0.390640832	pseudogene	1	212820638	212821097	
ENSG00000226040	0	0.390640832	pseudogene	5	141275733	141276254	
ENSG00000226044	2.10647801	1.16600992	lincRNA	10	48324788	48332197	
ENSG00000226049	1.16343121	2.039052734	pseudogene	17	32363700	32366891	TLK2P1
ENSG00000226053	0	0.390640832	processed_transc	1	98676303	98738214	
ENSG00000226054	1.616589159	1.518964905	pseudogene	21	37502669	37504208	MEMO1P1
ENSG00000226055	1.799000381	1.925536307	pseudogene	9	37878106	37879490	PAICSP1
ENSG00000226056	0.499066092	0.390640832	pseudogene	X	102054870	102056327	MTND4P32
ENSG00000226065	3.146112541	2.652276565	pseudogene	2	111140689	111142219	
ENSG00000226067	5.223818625	5.41517489	processed_transc	1	149284645	149287106	
ENSG00000226070	0	0.390640832	lincRNA	6	40326483	40337213	
ENSG00000226080	0.499066092	0	pseudogene	2	11242268	11243123	
ENSG00000226082	0.869158192	0.950786998	pseudogene	3	62912046	62912557	
ENSG00000226084	0	1.16600992	pseudogene	1	77594768	77595384	
ENSG00000226085	0	1.16600992	pseudogene	22	40271293	40272119	UQCRFS1P1
ENSG00000226086	0.869158192	0.390640832	pseudogene	10	38368984	38369805	
ENSG00000226087	0.869158192	0	lincRNA	2	47452933	47466364	
ENSG00000226088	1.16343121	0.950786998	antisense	1	70825245	70826120	
ENSG00000226091	2.762599152	3.453019579	processed_transc	12	8448582	8549399	
ENSG00000226094	0.869158192	0.697730409	pseudogene	20	61169878	61170591	RPL7P3
ENSG00000226101	0	0.697730409	lincRNA	17	70068474	70089622	
ENSG00000226102	0.499066092	0	pseudogene	7	35985904	36013282	SEPT7P3
ENSG00000226104	1.16343121	0.697730409	protein_coding	19	10751715	10752093	
ENSG00000226107	1.960915222	2.579085888	pseudogene	X	133656732	133657020	
ENSG00000226115	3.146112541	2.851901313	lincRNA	21	47476430	47477481	
ENSG00000226120	0.869158192	2.039052734	lincRNA	19	36198272	36199158	
ENSG00000226121	3.950976309	3.679994897	pseudogene	2	162357396	162364216	
ENSG00000226124	3.335220907	3.87606762	protein_coding	2	200625267	200715896	
ENSG00000226125	0.499066092	0.950786998	lincRNA	2	231555636	231565244	
ENSG00000226126	1.16343121	0.697730409	pseudogene	1	119156955	119158269	PSMC1P12
ENSG00000226128	0	0.390640832	pseudogene	1	98051418	98051850	RPL26P9
ENSG00000226135	2.10647801	2.144285137	sense_intronic	1	235989882	235991238	LYST-IT1
ENSG00000226137	5.141750194	4.448594745	protein_coding	17	79003502	79005315	

ENSG00000226138	0	0.697730409	pseudogene	12	48478833	48479204	
ENSG00000226144	0.499066092	0.390640832	pseudogene	20	35677657	35678102	RPS27AP3
ENSG00000226148	0.499066092	1.667587519	pseudogene	1	202765158	202766222	
ENSG00000226150	0.869158192	1.16600992	processed_transc	7	6833765	6836573	
ENSG00000226155	0.499066092	1.518964905	antisense	3	195638920	195640857	
ENSG00000226158	0	0.390640832	pseudogene	13	115072121	115072541	CLCP2
ENSG00000226161	0.869158192	1.925536307	pseudogene	13	37512537	37513631	EIF4A1P5
ENSG00000226167	1.960915222	2.420525079	processed_transc	1	114399257	114443859	
ENSG00000226169	0	0.390640832	antisense	22	28112468	28117665	
ENSG00000226174	3.448790144	3.027231696	protein_coding	14	105864916	105916443	TEX22
ENSG00000226179	3.077135474	2.788380093	antisense	X	281725	282586	PPP2R3B-AS1
ENSG00000226180	3.698846687	4.7279204	protein_coding	16	87727144	87729753	
ENSG00000226186	0.499066092	1.16600992	pseudogene	2	71183029	71183364	TCEB1P21
ENSG00000226188	0.499066092	1.802319292	pseudogene	20	44374376	44374926	HNRNPA1P3
ENSG00000226194	0	0.390640832	lincRNA	6	169770093	169788480	
ENSG00000226200	0.869158192	0	antisense	10	52388910	52391396	
ENSG00000226203	1.799000381	3.133298822	lincRNA	20	25936448	25949924	
ENSG00000226205	0	0.697730409	pseudogene	7	133417246	133418348	
ENSG00000226210	6.414691513	6.490875274	pseudogene	12	73725	91223	
ENSG00000226216	0	0.390640832	pseudogene	3	29432339	29432709	RPS12P5
ENSG00000226220	1.16343121	1.16600992	pseudogene	7	39836192	39837510	
ENSG00000226221	2.471521042	2.912743273	pseudogene	5	55800462	55800899	
ENSG00000226232	0.869158192	0.697730409	protein_coding	16	70010291	70099851	
ENSG00000226233	0.499066092	0	pseudogene	22	37611778	37611931	
ENSG00000226234	0	0.950786998	pseudogene	9	81651329	81652484	
ENSG00000226235	0.499066092	0	antisense	1	205342380	205356568	
ENSG00000226239	0	0.390640832	antisense	20	30615556	30619378	
ENSG00000226241	0	0.390640832	pseudogene	X	102342124	102344204	
ENSG00000226243	1.16343121	1.667587519	pseudogene	20	43095204	43095482	RPL37AP1
ENSG00000226245	1.799000381	1.802319292	antisense	10	44139320	44140495	ZNF32-AS1
ENSG00000226253	0.499066092	0	pseudogene	10	76287342	76287705	MRPL35P3
ENSG00000226254	0.499066092	0	pseudogene	7	90736689	90737108	PTP4A1P3
ENSG00000226259	2.10647801	2.652276565	pseudogene	5	69711179	69743885	GTF2H2B
ENSG00000226261	0	0.697730409	pseudogene	2	203200747	203201450	
ENSG00000226263	1.16343121	1.353254395	antisense	20	13218448	13220321	
ENSG00000226266	1.407729925	2.334191469	processed_transc	2	160527219	160568941	
ENSG00000226268	0.499066092	0.390640832	pseudogene	11	49455025	49455990	
ENSG00000226272	0	0.697730409	antisense	5	142239169	142248487	ARHGAP26-AS1
ENSG00000226278	3.554067925	4.250702764	pseudogene	7	55832490	55840981	PSPHP1
ENSG00000226279	1.407729925	1.925536307	pseudogene	7	22921512	22922009	
ENSG00000226284	1.799000381	1.518964905	pseudogene	20	47751017	47751549	ARPC3P1
ENSG00000226286	0	0.390640832	antisense	1	3049491	3050273	
ENSG00000226287	2.238690726	1.353254395	processed_transc	22	21055220	21058894	TMEM191A
ENSG00000226288	0	0.697730409	protein_coding	11	4608021	4609135	OR52I2
ENSG00000226291	0.869158192	0.390640832	antisense	7	1120499	1121814	
ENSG00000226301	0	0.390640832	pseudogene	8	61299821	61300554	
ENSG00000226302	0	0.390640832	lincRNA	3	40644616	40646918	
ENSG00000226306	0	1.16600992	pseudogene	5	137136882	137146440	NPY6R
ENSG00000226310	0.499066092	2.334191469	antisense	X	57148095	57153676	
ENSG00000226312	2.471521042	1.16600992	antisense	2	202005007	202022515	CFLAR-AS1
ENSG00000226314	4.593635215	4.622587519	pseudogene	6	28129559	28137372	ZNF192P1
ENSG00000226318	1.799000381	2.420525079	pseudogene	10	69720459	69721221	
ENSG00000226327	0.499066092	0.950786998	pseudogene	9	90939405	90940255	
ENSG00000226328	7.140657543	7.799123587	processed_transc	22	45529710	45559662	
ENSG00000226329	4.6658819	4.604259349	pseudogene	7	22903493	22920969	
ENSG00000226330	1.16343121	0.697730409	processed_transc	1	204346978	204404974	
ENSG00000226332	4.77879189	4.226940739	antisense	20	60880488	60881452	
ENSG00000226334	1.799000381	0.950786998	processed_transc	9	107689834	107691173	
ENSG00000226338	0	0.697730409	pseudogene	2	154064600	154064677	
ENSG00000226342	1.960915222	0.950786998	pseudogene	7	63369344	63370831	NMD3P1
ENSG00000226345	0	0.390640832	pseudogene	3	196150193	196150393	
ENSG00000226348	0	0.697730409	pseudogene	16	77254303	77255155	VN2R10P
ENSG00000226349	0	0.697730409	sense_intronic	1	225898670	225923976	

ENSG00000226352	2.762599152	2.420525079	processed_transc	13	20248764	20250024	PSPC1-OT1
ENSG00000226359	1.407729925	1.802319292	pseudogene	17	17145324	17146200	
ENSG00000226360	4.437288965	4.810179682	pseudogene	3	61728129	61728780	RPL10AP6
ENSG00000226361	0.499066092	0.697730409	pseudogene	13	19254634	19255846	TERF1P5
ENSG00000226367	0	0.950786998	antisense	7	116712126	116786534	ST7-AS2
ENSG00000226377	0.869158192	0	antisense	17	31203777	31205655	
ENSG00000226380	4.863159938	4.021284656	processed_transc	7	130565751	130598069	
ENSG00000226381	3.448790144	3.993387124	processed_transc	10	81524486	81546199	
ENSG00000226383	1.960915222	1.353254395	lincRNA	2	156868129	156881125	
ENSG00000226386	0.499066092	0.697730409	antisense	10	35104695	35105314	
ENSG00000226390	0.499066092	1.16600992	antisense	20	62079404	62080682	
ENSG00000226393	0	0.950786998	pseudogene	9	21321299	21321826	IFNA20P
ENSG00000226396	1.616589159	2.420525079	pseudogene	1	19934608	19935062	
ENSG00000226406	0	0.390640832	pseudogene	21	16201475	16202310	RBMX2P1
ENSG00000226410	0.499066092	0.697730409	pseudogene	2	187027676	187027972	
ENSG00000226413	0	0.950786998	pseudogene	12	48835813	48836730	OR8T1P
ENSG00000226415	3.698846687	3.748356452	pseudogene	1	77165474	77166223	TPI1P1
ENSG00000226416	4.618121645	5.085909553	antisense	11	2004467	2011150	MRPL23-AS1
ENSG00000226419	5.090170488	5.163066621	processed_transc	1	113499037	113544813	
ENSG00000226421	0.499066092	0	pseudogene	7	32511125	32512021	
ENSG00000226423	0.499066092	1.353254395	lincRNA	2	242967334	242968327	
ENSG00000226425	0	0.390640832	antisense	10	94819420	94820183	
ENSG00000226427	0	0.390640832	pseudogene	3	15416880	15417152	HMGN2P7
ENSG00000226432	0.499066092	0	pseudogene	5	24170479	24171355	
ENSG00000226434	1.16343121	0	lincRNA	X	13395307	13421138	
ENSG00000226435	0	0.697730409	pseudogene	3	197780393	197807591	ANKRD18DP
ENSG00000226436	0	0.950786998	pseudogene	X	28677182	28677586	
ENSG00000226438	0	0.390640832	antisense	1	39715510	39723321	
ENSG00000226439	0	0.390640832	pseudogene	4	62641167	62641568	
ENSG00000226443	0.869158192	1.667587519	pseudogene	1	120076372	120077369	GAPDHP32
ENSG00000226445	3.698846687	4.693656882	antisense	6	169613349	169639660	
ENSG00000226450	2.35979773	3.027231696	pseudogene	22	42545877	42551007	CYP2D8P1
ENSG00000226453	0	0.390640832	lincRNA	6	82554902	82643197	
ENSG00000226457	1.16343121	1.518964905	pseudogene	1	16695645	16696025	
ENSG00000226458	3.211941663	1.16600992	sense_intronic	10	75788171	75789962	
ENSG00000226465	2.238690726	2.501982735	antisense	20	25731854	25733397	
ENSG00000226466	0	0.697730409	pseudogene	10	82896296	82897091	
ENSG00000226469	0.869158192	1.802319292	pseudogene	12	112364822	112366821	
ENSG00000226471	3.274897671	3.133298822	antisense	22	29196671	29244547	
ENSG00000226472	1.407729925	1.802319292	lincRNA	12	31149973	31158938	
ENSG00000226476	3.603959378	3.845183986	lincRNA	1	61005921	61106163	
ENSG00000226478	0	0.390640832	pseudogene	17	16648945	16650075	UPF3AP1
ENSG00000226479	8.848626402	8.310945169	protein_coding	2	120978854	120980984	TMEM185B
ENSG00000226484	0	0.390640832	processed_transc	X	36383741	36458375	
ENSG00000226491	0	0.697730409	pseudogene	2	43024365	43025852	
ENSG00000226493	1.799000381	1.667587519	pseudogene	10	70259289	70259725	
ENSG00000226496	0.499066092	1.518964905	antisense	21	42513427	42520060	LINC00323
ENSG00000226498	0	0.390640832	pseudogene	1	236982934	236983813	
ENSG00000226501	0	0.390640832	pseudogene	21	34706877	34707402	USF1P1
ENSG00000226510	0.499066092	1.353254395	antisense	19	36158850	36164193	UPK1A-AS1
ENSG00000226520	2.762599152	2.501982735	sense_intronic	1	157995340	158000956	KIRREL-IT1
ENSG00000226521	0	0.950786998	pseudogene	17	20619796	20640440	
ENSG00000226524	0	1.518964905	lincRNA	6	88081311	88102519	
ENSG00000226525	0.499066092	0.950786998	pseudogene	13	22202552	22203154	RPS7P10
ENSG00000226533	0.499066092	0	antisense	6	38449468	38450307	BTBD9-AS1
ENSG00000226535	0	0.390640832	pseudogene	9	109931850	109933429	
ENSG00000226539	0	0.697730409	pseudogene	2	224569338	224570055	
ENSG00000226540	1.16343121	1.518964905	pseudogene	6	135940303	135941309	GAPDHP73
ENSG00000226544	0	0.697730409	pseudogene	5	72021246	72021978	RPL7P22
ENSG00000226545	0.499066092	0	pseudogene	1	8858534	8859565	
ENSG00000226549	1.16343121	1.353254395	pseudogene	17	20687958	20689040	SCDP1
ENSG00000226556	0.499066092	0.390640832	pseudogene	X	47298135	47299173	
ENSG00000226561	0.499066092	0	sense_intronic	10	52348409	52349592	

ENSG00000226562	1.16343121	1.925536307	lincRNA	9	33602499	33605291	
ENSG00000226564	4.165528823	3.781359661	pseudogene	2	181737594	181738141	FTH1P20
ENSG00000226573	0.499066092	0	pseudogene	22	30501083	30501397	
ENSG00000226574	0	0.697730409	processed_transc	X	140590843	140738057	SPANXA2-OT1
ENSG00000226578	2.238690726	3.781359661	lincRNA	10	38064299	38073059	
ENSG00000226580	1.616589159	2.242360793	pseudogene	21	18872997	18873142	RPL39P40
ENSG00000226581	1.616589159	1.353254395	lincRNA	7	63361201	63378372	
ENSG00000226585	0.869158192	1.353254395	antisense	1	178326311	178333230	
ENSG00000226602	1.16343121	1.518964905	sense_intronic	9	131448620	131449914	
ENSG00000226604	0	0.697730409	antisense	9	119048108	119051048	
ENSG00000226608	3.335220907	3.96493948	pseudogene	20	4004552	4005091	FTLP3
ENSG00000226609	0	1.16600992	antisense	9	115511182	115512736	
ENSG00000226621	0.499066092	0.950786998	pseudogene	3	11332092	11332349	
ENSG00000226624	0.869158192	0.950786998	pseudogene	7	105459938	105460547	
ENSG00000226628	3.78787645	3.570966319	pseudogene	1	1667256	1668690	
ENSG00000226631	0.499066092	0.390640832	pseudogene	10	51784895	51787123	SLC9A3P3
ENSG00000226632	0.869158192	0.390640832	pseudogene	20	3342527	3342971	UBE2V1P1
ENSG00000226636	0	0.390640832	pseudogene	7	121799354	121802297	
ENSG00000226637	0	0.390640832	processed_transc	1	143719663	143721171	
ENSG00000226641	0	0.390640832	pseudogene	X	41181854	41182364	
ENSG00000226642	0.499066092	0	pseudogene	3	12111695	12112860	
ENSG00000226644	1.799000381	2.652276565	lincRNA	20	2187863	2193797	
ENSG00000226645	1.616589159	1.16600992	antisense	11	116644105	116644921	
ENSG00000226647	0.499066092	1.16600992	antisense	10	5754177	5786030	
ENSG00000226648	0.499066092	0.390640832	antisense	20	39726969	39766643	
ENSG00000226649	1.407729925	1.16600992	lincRNA	2	3160527	3161568	
ENSG00000226650	1.960915222	1.518964905	protein_coding	5	154393315	154397692	KIF4B
ENSG00000226652	0.499066092	0	pseudogene	3	186478893	186479564	PSMD10P2
ENSG00000226653	0	0.390640832	pseudogene	1	146890786	146891588	OR13Z1P
ENSG00000226659	2.762599152	1.802319292	antisense	10	82289353	82295698	
ENSG00000226661	0.869158192	0	antisense	X	118490264	118527558	
ENSG00000226665	0.499066092	0	pseudogene	13	75710291	75711077	SSR1P2
ENSG00000226666	2.671945279	2.334191469	pseudogene	2	222826456	222828484	HSPA9P1
ENSG00000226667	0	0.697730409	lincRNA	10	47570513	47573855	
ENSG00000226668	0	0.697730409	pseudogene	9	95644849	95661890	
ENSG00000226673	0.869158192	1.16600992	lincRNA	6	14280358	14285376	
ENSG00000226674	1.16343121	1.16600992	lincRNA	2	145425534	145940216	
ENSG00000226675	0.499066092	0	pseudogene	1	148598464	148598976	
ENSG00000226677	0.499066092	0.697730409	pseudogene	14	35408530	35409693	IGBP1P1
ENSG00000226686	3.077135474	3.493416095	processed_transc	19	37742828	37747421	
ENSG00000226688	3.871730003	4.585695337	processed_transc	10	97512963	97849995	
ENSG00000226695	0.869158192	0	pseudogene	13	25324795	25326309	ANKRD20A10P
ENSG00000226696	4.942865586	4.961837817	antisense	19	54955991	54960223	LENG8-AS1
ENSG00000226698	2.928422289	3.027231696	antisense	1	27202624	27204736	
ENSG00000226699	1.16343121	1.518964905	antisense	10	135159258	135164230	
ENSG00000226701	0.499066092	1.16600992	pseudogene	10	73949699	73950334	
ENSG00000226702	0	0.390640832	lincRNA	2	56190326	56274461	
ENSG00000226706	1.407729925	2.652276565	antisense	9	138395119	138398293	
ENSG00000226708	0	0.950786998	lincRNA	2	124200842	124202021	
ENSG00000226711	3.393122761	4.640685763	antisense	12	8332805	8368747	FAM66C
ENSG00000226723	0	1.16600992	pseudogene	1	192215849	192216617	
ENSG00000226738	5.575999561	4.363974406	antisense	22	50984844	50986081	
ENSG00000226741	0.499066092	0	lincRNA	22	27706612	27713417	
ENSG00000226742	6.196276899	5.647721597	protein_coding	18	77724582	77730820	HSBP1L1
ENSG00000226745	0	0.950786998	pseudogene	1	40598434	40599121	
ENSG00000226746	1.960915222	2.721932731	protein_coding	17	17680000	17682843	SMCR5
ENSG00000226752	7.1740722	7.799123587	processed_transc	9	123605378	123616651	
ENSG00000226754	4.261916566	4.101879561	processed_transc	1	53704282	53708455	
ENSG00000226756	1.16343121	0	lincRNA	2	64871746	64873832	
ENSG00000226758	0.869158192	0.390640832	sense_intronic	1	232061580	232080979	DISC1-IT1
ENSG00000226761	0.499066092	0	protein_coding	12	11213964	11214893	TAS2R46
ENSG00000226763	3.78787645	3.748356452	protein_coding	19	44100769	44118649	SRRM5
ENSG00000226766	0.499066092	0	pseudogene	1	243787968	243788358	FABP7P1

ENSG00000226772	0.499066092	0	antisense	22	29495029	29507671	
ENSG00000226779	0	0.390640832	antisense	3	174952651	174988885	NAALADL2-AS2
ENSG00000226783	0	0.390640832	pseudogene	9	125448212	125450651	TLK1P1
ENSG00000226784	0	1.925536307	protein_coding	X	77223458	77225135	PGAM4
ENSG00000226786	0	0.390640832	processed_transc	6	19535175	19640814	
ENSG00000226789	0.499066092	0.390640832	pseudogene	2	128004834	128007658	
ENSG00000226790	0.869158192	0.390640832	pseudogene	10	44282860	44285865	HNRNPA3P1
ENSG00000226793	0	0.390640832	pseudogene	9	67348382	67348907	
ENSG00000226797	0.869158192	1.353254395	antisense	17	61458592	61491673	
ENSG00000226798	1.16343121	1.518964905	lincRNA	9	83484673	83486210	
ENSG00000226800	0.499066092	0	protein_coding	19	3611566	3619189	C19orf29-AS1
ENSG00000226801	1.407729925	1.353254395	pseudogene	9	33968075	33968516	
ENSG00000226803	1.616589159	0.950786998	processed_transc	6	56979709	57037194	
ENSG00000226806	0.869158192	0	antisense	2	136577761	136580657	
ENSG00000226807	1.16343121	0	polymorphic_pse	8	142443929	142517330	
ENSG00000226819	0.869158192	0.390640832	antisense	2	66653867	66660602	
ENSG00000226823	2.10647801	2.144285137	pseudogene	9	33503653	33510709	
ENSG00000226824	3.335220907	2.971122874	sense_intronic	7	66119525	66134842	
ENSG00000226828	0.499066092	0	processed_transc	1	244080704	244210619	
ENSG00000226833	1.616589159	2.334191469	antisense	2	28931819	28959071	
ENSG00000226835	1.16343121	1.802319292	lincRNA	1	94798823	94800404	
ENSG00000226842	0.499066092	0.390640832	antisense	10	32570614	32571757	
ENSG00000226845	0.499066092	0	pseudogene	3	40637698	40638985	
ENSG00000226849	1.799000381	0.697730409	antisense	1	11128528	11133154	
ENSG00000226853	2.471521042	2.242360793	lincRNA	2	175352117	175353028	
ENSG00000226855	1.616589159	0.950786998	pseudogene	1	154350683	154351565	
ENSG00000226856	1.407729925	2.039052734	lincRNA	2	118889704	118943962	
ENSG00000226862	0.869158192	0.697730409	antisense	1	202573396	202574421	
ENSG00000226864	2.238690726	3.081239798	antisense	10	123687827	123711480	
ENSG00000226872	0.499066092	0.697730409	antisense	22	21385646	21398258	
ENSG00000226874	1.16343121	1.16600992	pseudogene	7	30583669	30584047	
ENSG00000226876	0	0.390640832	antisense	1	246271928	246277168	
ENSG00000226877	1.616589159	1.925536307	antisense	9	86444571	86452779	
ENSG00000226878	0.499066092	0.390640832	pseudogene	2	159912606	159913371	GSTM3P2
ENSG00000226881	0	0.390640832	pseudogene	X	50648438	50648866	H3F3AP5
ENSG00000226883	0	0.390640832	antisense	1	60220419	60254854	
ENSG00000226887	4.689181911	4.021284656	lincRNA	4	53588785	53617807	ERVMER34-1
ENSG00000226889	0.869158192	1.353254395	antisense	1	161732646	161736000	
ENSG00000226890	0	0.390640832	antisense	16	1675629	1676161	
ENSG00000226891	1.616589159	0.697730409	processed_transc	1	65437908	65468159	
ENSG00000226899	0	0.390640832	antisense	10	126633773	126635001	
ENSG00000226907	0.869158192	0.390640832	protein_coding	X	134961634	134971244	CT45A6
ENSG00000226908	0	0.390640832	pseudogene	13	22057973	22058313	HIST1H2BPS3
ENSG00000226912	0.869158192	0.950786998	pseudogene	22	27040853	27041300	ISCA2P1
ENSG00000226913	1.16343121	1.353254395	processed_transc	3	49586739	49591799	BSN-AS2
ENSG00000226915	0	0.390640832	pseudogene	2	131051395	131051978	
ENSG00000226917	0.499066092	0.697730409	lincRNA	6	41470182	41487590	
ENSG00000226920	2.238690726	2.788380093	lincRNA	1	229576031	229576767	
ENSG00000226925	0.499066092	0	antisense	2	102789081	102798568	
ENSG00000226933	0	0.697730409	pseudogene	3	46064788	46065648	
ENSG00000226937	0.499066092	0.390640832	processed_transc	10	45497679	45567036	
ENSG00000226942	3.78787645	3.411459265	pseudogene	16	79336	88321	IL9RP3
ENSG00000226943	0.499066092	0.390640832	pseudogene	7	6963435	6970466	
ENSG00000226944	1.407729925	1.667587519	antisense	1	6264900	6265840	
ENSG00000226945	2.928422289	2.039052734	pseudogene	1	207080964	207081574	
ENSG00000226948	0.869158192	0.697730409	pseudogene	20	4610073	4610865	
ENSG00000226950	8.366258896	9.076603748	processed_transc	4	53578597	53586518	SNHG13
ENSG00000226952	0	0.950786998	pseudogene	1	100564795	100565046	
ENSG00000226954	0	0.390640832	processed_transc	22	50111701	50118154	
ENSG00000226957	0.499066092	0.390640832	antisense	1	46512490	46514040	
ENSG00000226958	15.13978265	15.21036828	pseudogene	X	108297361	108297792	RN28S1
ENSG00000226961	0.499066092	0.697730409	pseudogene	2	11491753	11492250	
ENSG00000226964	0	0.390640832	pseudogene	10	47396223	47396822	

ENSG00000226966	0.499066092	0.950786998	sense_intronic	17	19174706	19177701	EPN2-IT1
ENSG00000226967	0	0.390640832	pseudogene	1	170338364	170339508	
ENSG00000226969	0	0.390640832	antisense	1	1980642	1981509	
ENSG00000226971	0.869158192	0	pseudogene	X	48305273	48307251	
ENSG00000226972	0.869158192	0.390640832	pseudogene	2	44497760	44498247	
ENSG00000226973	0.499066092	2.144285137	pseudogene	1	116014562	116018648	
ENSG00000226976	2.57521082	3.081239798	protein_coding	6	37012607	37013177	COX6A1P2
ENSG00000226977	0.499066092	0.697730409	pseudogene	13	96461564	96461861	HMGN1P24
ENSG00000226979	3.448790144	1.925536307	protein_coding	6	31539831	31542101	LTA
ENSG00000226981	1.16343121	0.390640832	pseudogene	17	20744360	20747570	FAM108A7P
ENSG00000226982	0.499066092	1.16600992	pseudogene	12	89893870	89896447	CENPC1P1
ENSG00000226984	0	0.950786998	pseudogene	1	115002556	115002982	
ENSG00000226985	0	0.390640832	lincRNA	X	13353360	13391753	
ENSG00000226986	1.799000381	1.925536307	pseudogene	1	211380389	211381286	
ENSG00000226989	0.499066092	1.16600992	pseudogene	22	43249409	43249805	
ENSG00000227001	1.799000381	2.144285137	pseudogene	1	21751118	21754460	NBPF2P
ENSG00000227002	0	0.390640832	pseudogene	1	223831811	223832085	SNRPEP10
ENSG00000227006	2.238690726	2.579085888	antisense	1	230394440	230404229	
ENSG00000227008	1.616589159	2.242360793	pseudogene	X	132804999	132805414	
ENSG00000227009	0.499066092	0	pseudogene	22	39551530	39551950	FUNDC2P4
ENSG00000227010	0.499066092	0	processed_transc	1	155966114	155972355	
ENSG00000227014	2.671945279	1.667587519	antisense	7	30028216	30065276	
ENSG00000227017	0.869158192	0.390640832	lincRNA	7	30216003	30218630	
ENSG00000227035	0	0.390640832	pseudogene	7	63567200	63567950	
ENSG00000227036	6.749949642	6.811590016	processed_transc	17	70380443	70588943	
ENSG00000227038	2.847891871	1.667587519	pseudogene	7	75719749	75738097	
ENSG00000227039	3.989024711	3.781359661	processed_transc	21	46340966	46349593	
ENSG00000227045	0	0.390640832	antisense	1	151673502	151680862	
ENSG00000227047	0.499066092	0	lincRNA	2	20699331	20701071	
ENSG00000227051	0	0.697730409	protein_coding	14	96505661	96560417	C14orf132
ENSG00000227053	1.16343121	0.697730409	antisense	7	100657601	100660889	
ENSG00000227056	0	0.697730409	pseudogene	1	119761932	119762719	
ENSG00000227057	10.07366267	10.253797	protein_coding	6	33246885	33257304	WDR46
ENSG00000227060	2.762599152	2.501982735	processed_transc	X	133684003	133693953	
ENSG00000227066	4.734682531	1.518964905	antisense	1	20480831	20487061	
ENSG00000227068	0	0.390640832	antisense	9	128329858	128335302	
ENSG00000227070	1.960915222	1.518964905	processed_transc	1	51983981	52027301	
ENSG00000227071	0.869158192	1.16600992	lincRNA	9	20683303	20684687	
ENSG00000227073	0	0.390640832	pseudogene	7	135129209	135129673	SDHDP2
ENSG00000227076	3.603959378	3.64455972	sense_intronic	10	129732962	129734431	
ENSG00000227077	2.238690726	1.802319292	pseudogene	17	18476045	18476415	
ENSG00000227078	0.499066092	1.518964905	lincRNA	17	19322348	19327670	
ENSG00000227080	0	0.390640832	pseudogene	7	51456127	51456811	
ENSG00000227081	2.10647801	2.144285137	pseudogene	12	3320775	3321096	
ENSG00000227082	1.16343121	1.802319292	processed_transc	1	121138614	121204983	
ENSG00000227083	1.16343121	0.950786998	antisense	X	146981696	146982977	
ENSG00000227087	0.499066092	0	pseudogene	5	79627392	79628360	RBMX2P5
ENSG00000227096	0	0.390640832	pseudogene	10	118198980	118199556	HMGB3P8
ENSG00000227097	10.09453648	10.50507319	pseudogene	11	82400571	82400944	
ENSG00000227105	0.869158192	0.390640832	pseudogene	13	111589106	111592131	PARP1P1
ENSG00000227107	0	0.390640832	lincRNA	2	238521620	238535168	
ENSG00000227110	1.616589159	1.667587519	processed_transc	3	7994492	8653610	
ENSG00000227113	1.616589159	1.16600992	pseudogene	7	64535401	64539158	
ENSG00000227117	3.146112541	2.721932731	processed_transc	22	30404731	30476469	
ENSG00000227118	0.499066092	0.950786998	pseudogene	4	99661893	99662382	BTF3P13
ENSG00000227123	0	0.390640832	pseudogene	3	44979165	44979974	
ENSG00000227124	6.887241849	6.819555981	protein_coding	3	75758794	75834734	ZNF717
ENSG00000227136	0	0.390640832	lincRNA	10	80027085	80090352	C10orf101
ENSG00000227154	0.869158192	0	pseudogene	6	35410821	35417643	
ENSG00000227158	0	0.390640832	pseudogene	17	17325995	17327018	
ENSG00000227160	0.499066092	0	pseudogene	11	32133595	32364955	
ENSG00000227161	0	0.390640832	processed_transc	15	59936497	59964501	
ENSG00000227163	1.407729925	0.950786998	pseudogene	1	44553633	44553765	

ENSG00000227165	0	0.390640832	processed_transc	10	122521324	122551437	
ENSG00000227173	0	0.390640832	pseudogene	10	66929196	66929627	MYL6P3
ENSG00000227176	1.960915222	0.697730409	pseudogene	2	170933734	170934804	
ENSG00000227182	0.499066092	0.950786998	pseudogene	7	57482534	57483429	VN1R28P
ENSG00000227183	6.830742851	6.729413443	pseudogene	X	130780647	130780899	HDGFP1
ENSG00000227184	8.512210607	8.454700467	protein_coding	8	144939497	144952632	EPPK1
ENSG00000227186	0	0.390640832	antisense	10	76783233	76784009	
ENSG00000227187	0.499066092	0	pseudogene	2	175898506	175898920	
ENSG00000227189	3.393122761	3.183544561	antisense	4	1145160	1147489	
ENSG00000227192	1.16343121	1.353254395	antisense	6	143360562	143363461	
ENSG00000227193	0.869158192	0.697730409	lincRNA	1	120876263	120905159	
ENSG00000227195	5.255381949	7.234379288	processed_transc	20	26167655	26189869	
ENSG00000227197	0	0.390640832	antisense	7	132515453	132534353	
ENSG00000227199	2.928422289	2.851901313	antisense	7	116592500	116594388	ST7-AS1
ENSG00000227200	1.407729925	1.667587519	sense_intronic	9	127025155	127028088	
ENSG00000227201	3.077135474	2.144285137	pseudogene	22	30442265	30443182	CNN2P1
ENSG00000227203	1.16343121	0.390640832	pseudogene	8	11647514	11647897	SUB1P1
ENSG00000227207	0	0.390640832	pseudogene	1	72767155	72767506	
ENSG00000227208	0.869158192	0.390640832	pseudogene	13	114451484	114454062	
ENSG00000227210	0.869158192	0.950786998	antisense	2	20189978	20203971	
ENSG00000227212	0.869158192	0.390640832	pseudogene	1	148574653	148575051	PFN1P7
ENSG00000227213	0.499066092	0	antisense	13	24826887	24828577	SPATA13-AS1
ENSG00000227214	1.960915222	3.081239798	antisense	6	28953980	28955261	HCG15
ENSG00000227215	0.499066092	0	antisense	6	83642320	83648396	
ENSG00000227216	0.499066092	1.667587519	pseudogene	1	146059926	146060344	PFN1P5
ENSG00000227218	0.869158192	1.518964905	antisense	9	130702574	130703231	
ENSG00000227220	0.869158192	0.390640832	antisense	6	132272086	132398533	
ENSG00000227225	0	0.390640832	pseudogene	17	19505572	19506521	MTND1P14
ENSG00000227227	0.499066092	0	antisense	2	187506066	187560014	
ENSG00000227230	1.799000381	0.950786998	antisense	1	243299200	243303890	
ENSG00000227232	2.35979773	3.027231696	pseudogene	1	14363	29806	WASH7P
ENSG00000227233	2.238690726	1.667587519	pseudogene	7	51449981	51452357	
ENSG00000227237	0.499066092	0.390640832	antisense	1	249153363	249158995	
ENSG00000227242	1.799000381	1.667587519	pseudogene	1	146490895	146596107	
ENSG00000227245	0	0.950786998	lincRNA	3	43128971	43129560	
ENSG00000227252	3.077135474	2.788380093	processed_transc	2	237968077	237994460	
ENSG00000227253	0	0.390640832	antisense	10	32555217	32558039	
ENSG00000227256	1.16343121	1.802319292	antisense	21	33650174	33653299	MIS18A-AS1
ENSG00000227258	0	1.518964905	antisense	13	44716682	44813010	C13orf44-AS1
ENSG00000227262	1.407729925	2.420525079	pseudogene	6	29892500	29894992	
ENSG00000227267	0	0.390640832	pseudogene	3	135774026	135774606	
ENSG00000227268	0.499066092	0	protein_coding	10	89621708	89622244	KLLN
ENSG00000227279	0.869158192	1.667587519	antisense	18	25534483	25543651	
ENSG00000227280	1.16343121	0.697730409	antisense	1	145373054	145375554	
ENSG00000227285	2.238690726	2.912743273	antisense	X	119055224	119056803	
ENSG00000227288	0.499066092	0	pseudogene	1	81967455	81969147	
ENSG00000227295	0.499066092	0.697730409	pseudogene	1	158145640	158147545	ELL2P1
ENSG00000227297	0.499066092	0.390640832	lincRNA	20	56174751	56176256	
ENSG00000227300	2.671945279	0.697730409	pseudogene	17	16733313	16736147	KRT16P2
ENSG00000227301	0.499066092	0.390640832	lincRNA	9	33719688	33722553	
ENSG00000227307	0	0.390640832	lincRNA	10	122685059	122740213	
ENSG00000227308	0.869158192	0.390640832	processed_transc	2	220551709	220579408	
ENSG00000227313	0	0.697730409	lincRNA	10	36185802	36187891	
ENSG00000227321	1.799000381	1.16600992	pseudogene	9	94873147	94874179	MTND4P15
ENSG00000227329	1.799000381	2.334191469	pseudogene	X	53193574	53193739	
ENSG00000227331	1.407729925	0.697730409	pseudogene	2	159404937	159405736	
ENSG00000227332	0	0.697730409	lincRNA	13	19433967	19434882	LINC00419
ENSG00000227337	0.499066092	1.353254395	pseudogene	3	32827138	32827608	
ENSG00000227339	0.499066092	0.390640832	pseudogene	3	31494324	31497386	THRAP3P1
ENSG00000227344	0.499066092	0	pseudogene	7	53255081	53256631	
ENSG00000227345	7.127924785	7.029859489	protein_coding	10	51026325	51130715	PARG
ENSG00000227347	1.799000381	0.697730409	pseudogene	2	136956684	136958073	HNRNPKP2
ENSG00000227348	1.16343121	0	pseudogene	2	202077006	202077804	MTND5P25

ENSG00000227354	3.603959378	3.532712221	processed_transc	13	79980444	79997090	RBM26-AS1
ENSG00000227355	1.616589159	1.16600992	antisense	9	124137519	124214455	
ENSG00000227356	0.499066092	0	antisense	10	99588235	99609535	
ENSG00000227359	3.146112541	2.912743273	lincRNA	2	114435279	114461655	
ENSG00000227361	0.499066092	0	pseudogene	2	62030278	62030523	
ENSG00000227363	0	1.16600992	lincRNA	2	3891774	3895001	
ENSG00000227370	2.35979773	2.721932731	pseudogene	22	42528550	42529005	
ENSG00000227372	7.409722215	7.88892877	processed_transc	1	3652548	3663900	TP73-AS1
ENSG00000227373	0.499066092	0.697730409	lincRNA	1	174084438	174129142	
ENSG00000227374	0	1.16600992	lincRNA	10	131567108	131567699	
ENSG00000227375	3.393122761	1.518964905	processed_transc	3	197025123	197030618	
ENSG00000227376	3.393122761	3.368666104	pseudogene	11	77445520	77446071	FTH1P16
ENSG00000227379	0	1.353254395	pseudogene	20	25348911	25349402	PPIAP2
ENSG00000227382	0.869158192	0.697730409	pseudogene	10	74959584	74960983	
ENSG00000227383	0	0.390640832	pseudogene	9	34191553	34191879	
ENSG00000227388	0	0.697730409	antisense	9	35786643	35790429	
ENSG00000227392	0	0.697730409	protein_coding	19	35549963	35597208	
ENSG00000227395	0.499066092	0	pseudogene	10	45301891	45303671	EIF2AP4
ENSG00000227397	1.407729925	0	pseudogene	7	63250287	63250476	
ENSG00000227398	2.847891871	4.075511708	processed_transc	3	47205987	47285546	
ENSG00000227401	1.960915222	0.950786998	pseudogene	20	34176246	34176529	RPL37P1
ENSG00000227403	5.239686603	5.317302248	processed_transc	2	162101250	162105561	
ENSG00000227404	0	0.697730409	pseudogene	7	38694805	38696213	KRT8P20
ENSG00000227407	1.16343121	1.16600992	antisense	19	54941832	54945878	
ENSG00000227409	0	0.390640832	antisense	1	35824423	35831678	ZMYM4-AS1
ENSG00000227410	1.616589159	1.518964905	processed_transc	9	71736209	71737148	
ENSG00000227413	1.799000381	0.950786998	pseudogene	22	40829140	40829645	
ENSG00000227415	0	0.390640832	processed_transc	1	152346430	152417932	
ENSG00000227417	1.799000381	1.667587519	pseudogene	1	203774746	203775391	
ENSG00000227431	0	0.950786998	antisense	20	47657000	47662581	
ENSG00000227432	0	0.950786998	antisense	2	220346795	220381599	
ENSG00000227436	0.869158192	1.16600992	pseudogene	7	23658952	23662445	
ENSG00000227440	2.238690726	1.925536307	pseudogene	3	172546526	172546966	ATP5G1P4
ENSG00000227449	0	1.16600992	pseudogene	9	46687462	46746500	
ENSG00000227450	2.10647801	2.721932731	protein_coding	17	36905613	36906969	
ENSG00000227452	0	0.390640832	sense_intronic	9	128047877	128048998	
ENSG00000227453	0	0.390640832	pseudogene	1	55002469	55003431	
ENSG00000227456	1.616589159	2.039052734	lincRNA	21	35552978	35562220	LINC00310
ENSG00000227467	0	0.390640832	processed_transc	11	72281704	72284273	
ENSG00000227470	0.869158192	0.950786998	pseudogene	2	109494764	109494916	
ENSG00000227471	3.911897206	2.144285137	protein_coding	7	134233888	134264592	AKR1B15
ENSG00000227473	2.57521082	2.501982735	pseudogene	8	145196117	145198567	TSSK5P1
ENSG00000227474	0	0.697730409	pseudogene	10	13633460	13634323	
ENSG00000227477	1.799000381	1.925536307	processed_transc	20	43592435	43595043	
ENSG00000227479	0	0.390640832	lincRNA	2	241166977	241195452	
ENSG00000227482	1.407729925	1.353254395	lincRNA	9	116413236	116445135	
ENSG00000227484	0.499066092	0	antisense	22	50753060	50754437	
ENSG00000227486	1.407729925	0.950786998	processed_transc	X	55934556	56041606	
ENSG00000227490	0	0.390640832	pseudogene	9	46169973	46170336	
ENSG00000227492	1.16343121	1.353254395	antisense	10	101989424	101993757	
ENSG00000227495	2.35979773	2.851901313	antisense	17	4922509	4923388	
ENSG00000227496	4.464551814	4.693656882	antisense	1	225888306	225939945	
ENSG00000227499	0.499066092	0	pseudogene	7	55945605	55945857	
ENSG00000227500	10.39432652	10.44035639	protein_coding	19	1905371	1926012	SCAMP4
ENSG00000227502	0	0.697730409	processed_transc	6	114191162	114191899	
ENSG00000227505	0.499066092	0	pseudogene	X	146882523	146882820	
ENSG00000227507	8.735671237	8.677053487	protein_coding	6	31548302	31550299	LTB
ENSG00000227512	2.10647801	2.039052734	antisense	9	139437333	139438287	
ENSG00000227516	0.499066092	0	lincRNA	6	41836360	41837469	
ENSG00000227518	4.689181911	4.888000647	antisense	9	27657	30891	MIR1302-10
ENSG00000227523	0.869158192	0.697730409	pseudogene	3	27503997	27504307	
ENSG00000227525	1.616589159	0.390640832	pseudogene	12	19241974	19242825	
ENSG00000227527	0.499066092	1.16600992	processed_transc	1	42801057	42804047	

ENSG00000227533	3.077135474	3.493416095	processed_transc		1	43424720	43449029	
ENSG00000227536	2.57521082	2.721932731	pseudogene	X		70263064	70265320	
ENSG00000227539	0.499066092	0.697730409	sense_intronic		10	76185995	76186461	
ENSG00000227540	3.744048221	3.748356452	processed_transc		10	75012549	75014107	
ENSG00000227541	0.499066092	0.390640832	pseudogene	X		119335910	119336597	
ENSG00000227542	1.799000381	1.925536307	lincRNA		2	192093891	192110898	
ENSG00000227543	4.409500985	3.453019579	processed_transc		17	26925808	26944393	SPAG5-AS1
ENSG00000227544	0.499066092	0.697730409	lincRNA		7	35756055	35774497	
ENSG00000227554	0	0.390640832	antisense		1	183723553	183724072	
ENSG00000227558	1.799000381	0.390640832	pseudogene		9	69112841	69147854	PGM5P2
ENSG00000227560	0.499066092	0	pseudogene		10	114747235	114747624	
ENSG00000227568	0.869158192	0	pseudogene		13	19381419	19382158	SNX18P26
ENSG00000227582	1.16343121	2.144285137	pseudogene		9	66516697	66517831	
ENSG00000227583	0.869158192	0.390640832	pseudogene		10	70434113	70434857	
ENSG00000227586	0.869158192	1.802319292	pseudogene		10	124931048	124931395	
ENSG00000227589	0.499066092	0.390640832	antisense		1	3575502	3585336	
ENSG00000227591	1.960915222	1.353254395	antisense		1	209834709	209897470	
ENSG00000227593	0.499066092	0	pseudogene		11	93788189	93789138	
ENSG00000227598	1.616589159	1.802319292	antisense		6	167383114	167412553	
ENSG00000227603	0.499066092	0.950786998	antisense		9	96197614	96199403	
ENSG00000227606	0.499066092	1.518964905	antisense		19	43657431	43665014	
ENSG00000227608	0.499066092	0.697730409	antisense		13	114110944	114116670	DCUN1D2-AS1
ENSG00000227609	0	0.950786998	pseudogene		6	29545236	29545525	TMEM183AP1
ENSG00000227612	0.499066092	0.390640832	antisense		1	14104760	14105625	
ENSG00000227615	4.903563137	5.536128679	pseudogene		11	74456761	74457159	
ENSG00000227619	1.799000381	2.039052734	antisense		9	132337688	132346835	
ENSG00000227620	1.960915222	0	pseudogene		11	67552733	67559809	
ENSG00000227621	1.16343121	0.390640832	pseudogene		1	224044281	224045089	PHBP11
ENSG00000227627	0.499066092	0.390640832	antisense		6	153304885	153310358	
ENSG00000227630	1.960915222	2.420525079	processed_transc		1	234861148	234867390	
ENSG00000227632	1.799000381	0	pseudogene		2	130959885	130970201	
ENSG00000227637	0.499066092	0.950786998	antisense		1	29479001	29479503	
ENSG00000227638	1.407729925	1.16600992	pseudogene		9	91025798	91026363	HNRNPA1P14
ENSG00000227640	0.869158192	0	processed_transc		13	95364970	95366749	SOX21-AS1
ENSG00000227645	0	0.390640832	pseudogene	X		78018723	78019382	RPL7P54
ENSG00000227646	0	0.390640832	processed_transc		7	89511667	89840949	STEAP2-AS1
ENSG00000227649	0.499066092	0	pseudogene	X		102061092	102061574	
ENSG00000227651	0	0.697730409	pseudogene		9	33574356	33574633	
ENSG00000227666	0.499066092	1.16600992	pseudogene		9	96800312	96800627	
ENSG00000227671	7.44778847	8.046558348	pseudogene		1	247353153	247372795	
ENSG00000227673	0.869158192	0	antisense		1	155679889	155680354	
ENSG00000227674	1.16343121	0.390640832	processed_transc		13	64560504	64650144	LINC00355
ENSG00000227682	0.499066092	0.697730409	pseudogene		2	156126145	156126399	ATP5A1P2
ENSG00000227684	0	1.16600992	pseudogene		1	17076728	17077459	
ENSG00000227688	0	0.390640832	pseudogene		20	35248806	35249826	HNRNPA3P2
ENSG00000227692	0	0.950786998	pseudogene		2	187229325	187229855	
ENSG00000227693	1.407729925	0.950786998	pseudogene		20	21482033	21482606	GSTM3P1
ENSG00000227694	2.471521042	2.242360793	pseudogene		17	59147232	59147702	
ENSG00000227695	0	0.697730409	antisense		10	101686767	101718138	DNMBP-AS1
ENSG00000227698	1.616589159	1.802319292	sense_intronic		21	43294865	43297722	
ENSG00000227700	0.499066092	0.390640832	pseudogene		1	147718440	147718956	
ENSG00000227702	0.869158192	0	lincRNA		21	43099341	43117496	LINC00111
ENSG00000227705	0	0.390640832	antisense		20	51812768	51825244	
ENSG00000227707	0.499066092	0	pseudogene		3	33813455	33813787	SDAD1P3
ENSG00000227709	0.499066092	0	pseudogene		3	155875691	155894700	
ENSG00000227711	0	0.697730409	lincRNA		1	227698297	227708178	
ENSG00000227714	0	0.697730409	pseudogene		13	96346237	96346749	MTND6P18
ENSG00000227719	1.16343121	0.697730409	antisense		7	8153655	8156191	
ENSG00000227725	0	0.390640832	pseudogene		4	68904262	68905368	GCOM2
ENSG00000227729	1.407729925	0.697730409	protein_coding		14	104406763	104408645	TDRD9-AS1
ENSG00000227733	3.004694206	0.950786998	sense_intronic		1	146093234	146159102	
ENSG00000227736	0.499066092	0	pseudogene		2	202417582	202418335	
ENSG00000227740	0	0.390640832	lincRNA		1	175873898	175889649	

ENSG00000227741	3.554067925	3.532712221	processed_transc	1	160171989	160178659	
ENSG00000227742	1.16343121	0.950786998	pseudogene	1	52027538	52060569	CALR4P
ENSG00000227744	0	0.390640832	lincRNA	2	239840998	239847965	
ENSG00000227745	0.869158192	0	pseudogene	2	132890429	132897002	
ENSG00000227747	0.499066092	0	pseudogene	1	198918145	198918934	
ENSG00000227758	1.16343121	0.390640832	pseudogene	6	29716066	29716290	HCG9P5
ENSG00000227762	1.16343121	0.697730409	pseudogene	5	98868324	98870346	GUSBP8
ENSG00000227764	1.616589159	0.950786998	processed_transc	1	211813037	211827923	
ENSG00000227765	0.499066092	0.697730409	pseudogene	4	185220282	185220800	
ENSG00000227766	0.869158192	1.518964905	pseudogene	6	29909852	29910844	HCG4P5
ENSG00000227770	0.499066092	0	pseudogene	2	38458710	38459459	
ENSG00000227775	4.75690578	4.990346085	processed_transc	1	1656054	1663343	
ENSG00000227779	0	0.390640832	pseudogene	X	140474987	140475271	
ENSG00000227781	0.499066092	0	processed_transc	10	48952569	48980342	
ENSG00000227782	1.407729925	0.950786998	protein_coding	17	15943505	15943994	
ENSG00000227788	1.16343121	0.950786998	pseudogene	2	120969303	120970438	
ENSG00000227791	2.847891871	3.279072565	pseudogene	2	64892741	64893199	
ENSG00000227799	2.847891871	2.721932731	pseudogene	2	55451416	55453044	
ENSG00000227811	0	0.390640832	processed_transc	1	112282463	112290420	
ENSG00000227813	0	0.390640832	pseudogene	22	32286330	32287420	
ENSG00000227815	0.869158192	1.16600992	pseudogene	1	176241619	176242564	
ENSG00000227821	0.499066092	0.697730409	pseudogene	10	6867825	6868186	
ENSG00000227825	1.407729925	1.802319292	pseudogene	12	98847613	98850923	SLC9A7P1
ENSG00000227827	2.35979773	2.579085888	pseudogene	16	16450081	16471364	
ENSG00000227834	0.499066092	0	pseudogene	11	67419047	67420875	
ENSG00000227844	3.077135474	5.638744877	antisense	6	136364990	136393965	
ENSG00000227845	0.499066092	0.697730409	pseudogene	9	128134613	128134971	
ENSG00000227848	1.407729925	2.039052734	antisense	13	48575525	48576688	SUCLA2-AS1
ENSG00000227850	0	0.390640832	pseudogene	1	106240661	106240840	SEPT2P1
ENSG00000227852	0.499066092	0.390640832	pseudogene	9	35592783	35592948	
ENSG00000227854	0	0.390640832	pseudogene	1	239216048	239216203	
ENSG00000227855	5.389447744	4.65855978	pseudogene	7	29689843	29782210	
ENSG00000227857	0.869158192	0.950786998	antisense	1	46599043	46604753	
ENSG00000227864	0	0.390640832	pseudogene	X	118320405	118320792	
ENSG00000227868	1.16343121	0.390640832	protein_coding	1	23337327	23342343	
ENSG00000227874	0.869158192	0.697730409	pseudogene	21	15038189	15039867	GRAMD4P1
ENSG00000227877	0	0.390640832	lincRNA	10	61496753	61513203	
ENSG00000227879	0	0.390640832	pseudogene	13	19775777	19801572	PSPC1P1
ENSG00000227881	0	0.697730409	pseudogene	X	114899215	114900453	ASS1P5
ENSG00000227885	1.16343121	1.925536307	lincRNA	6	53605547	53611820	
ENSG00000227887	0	0.390640832	pseudogene	1	208870714	208871043	
ENSG00000227895	3.502389126	3.133298822	antisense	22	35318599	35468882	
ENSG00000227896	1.616589159	2.851901313	antisense	10	88281702	88282443	
ENSG00000227906	0	0.390640832	processed_transc	20	10006264	10349451	
ENSG00000227908	3.448790144	2.851901313	protein_coding	5	55290995	55299477	
ENSG00000227910	2.238690726	1.802319292	pseudogene	7	63385165	63386684	
ENSG00000227921	1.960915222	1.667587519	protein_coding	9	40028620	40032417	
ENSG00000227925	0	0.390640832	lincRNA	1	222001008	222014008	
ENSG00000227934	0.869158192	0	pseudogene	1	231157357	231157929	
ENSG00000227938	1.16343121	1.518964905	lincRNA	2	28671034	28673051	
ENSG00000227939	1.960915222	1.802319292	pseudogene	6	31248094	31249296	RPL3P2
ENSG00000227941	0.499066092	0.950786998	pseudogene	1	162511122	162511418	UQCRBP2
ENSG00000227942	0.499066092	0	pseudogene	X	64770778	64772301	FRMD8P1
ENSG00000227945	0	0.390640832	antisense	6	128349031	128406393	
ENSG00000227946	2.57521082	2.039052734	lincRNA	2	206949329	206951288	
ENSG00000227948	0	0.390640832	pseudogene	7	111051375	111051939	
ENSG00000227950	0	0.950786998	pseudogene	1	6894393	6894678	
ENSG00000227953	3.830412367	4.961837817	processed_transc	1	246939315	246955687	
ENSG00000227954	0	0.390640832	processed_transc	6	133823390	133832866	
ENSG00000227959	3.554067925	3.027231696	antisense	1	16481706	16483824	
ENSG00000227962	0.499066092	1.518964905	pseudogene	1	235500121	235500402	
ENSG00000227963	1.799000381	1.667587519	processed_transc	1	110828997	110881793	
ENSG00000227968	0.499066092	0	pseudogene	7	108634088	108635086	

ENSG00000227971	0.499066092	0	pseudogene	3	189363116	189363255	
ENSG00000227972	0.499066092	0.697730409	pseudogene	10	54170895	54173252	PRKRIRP3
ENSG00000227973	0.499066092	1.353254395	pseudogene	15	44168047	44168442	
ENSG00000227986	3.603959378	3.411459265	pseudogene	7	63815456	63816577	TRIM60P18
ENSG00000227990	0.499066092	0.697730409	antisense	7	1881252	1881812	
ENSG00000227992	3.211941663	2.788380093	pseudogene	2	111961541	111963792	
ENSG00000227999	2.762599152	2.501982735	pseudogene	21	45893606	45894980	MTND5P1
ENSG00000228000	0	2.579085888	pseudogene	17	18215218	18215965	
ENSG00000228002	1.960915222	0.950786998	pseudogene	13	77559003	77564724	DHX9P1
ENSG00000228005	0.499066092	0	antisense	7	50181136	50182419	
ENSG00000228006	5.40360309	5.271576845	protein_coding	19	58198294	58202072	
ENSG00000228007	1.407729925	1.667587519	pseudogene	17	73334908	73335228	
ENSG00000228008	1.799000381	0.950786998	antisense	3	49941278	49954370	
ENSG00000228010	1.799000381	2.039052734	processed_transc	7	6703605	6748532	
ENSG00000228011	0.499066092	1.518964905	lincRNA	13	78236635	78237275	LINC00413
ENSG00000228013	0.499066092	0	processed_transc	1	154374804	154379040	
ENSG00000228014	0	0.390640832	pseudogene	7	63929563	63931031	
ENSG00000228016	0	1.353254395	processed_transc	2	173541869	173600934	
ENSG00000228019	1.407729925	0.950786998	pseudogene	7	66772592	66773343	
ENSG00000228021	0	0.697730409	antisense	10	127371812	127398246	
ENSG00000228022	1.407729925	2.242360793	lincRNA	6	30734602	30760027	HCG20
ENSG00000228028	1.960915222	1.802319292	antisense	3	195977420	195978525	
ENSG00000228032	0	0.697730409	protein_coding	14	104710541	104712473	C14orf144
ENSG00000228033	0.499066092	1.353254395	lincRNA	2	52949815	53137158	
ENSG00000228037	0.499066092	0.390640832	antisense	1	2512999	2515972	
ENSG00000228038	1.616589159	1.353254395	pseudogene	9	99855976	99856920	VN1R51P
ENSG00000228040	0	0.390640832	pseudogene	1	113433389	113433544	
ENSG00000228043	2.57521082	2.334191469	processed_transc	2	139175854	139259268	
ENSG00000228044	1.407729925	0	processed_transc	1	234782035	234818922	
ENSG00000228046	0.869158192	0	pseudogene	9	85042166	85042940	
ENSG00000228049	0.499066092	0	protein_coding	7	102178366	102312182	POLR2J2
ENSG00000228056	0.869158192	0.697730409	pseudogene	1	64308862	64309356	CFL1P3
ENSG00000228057	1.16343121	1.518964905	pseudogene	1	98011257	98012514	SEC63P1
ENSG00000228060	1.16343121	1.925536307	antisense	1	40030832	40038875	
ENSG00000228061	0	0.390640832	processed_transc	11	31658875	31789501	
ENSG00000228063	1.616589159	0	processed_transc	1	219259944	219347303	
ENSG00000228065	2.57521082	3.64455972	lincRNA	10	67345446	67441220	
ENSG00000228069	1.616589159	1.353254395	pseudogene	9	94871295	94872377	
ENSG00000228071	1.407729925	1.353254395	pseudogene	16	18026577	18027132	RPL7P47
ENSG00000228072	0	0.390640832	antisense	9	33512957	33514771	
ENSG00000228076	1.16343121	0.390640832	pseudogene	1	109309468	109310206	
ENSG00000228079	0	0.390640832	lincRNA	2	64313484	64315380	
ENSG00000228084	1.16343121	1.802319292	antisense	1	100433939	100435420	
ENSG00000228086	0	0.390640832	antisense	1	100927955	100951553	
ENSG00000228091	0.499066092	0	antisense	10	103880410	103881139	
ENSG00000228106	4.352265886	5.466810074	processed_transc	1	222988364	223010726	
ENSG00000228107	0.869158192	0.697730409	processed_transc	21	37732928	37734338	
ENSG00000228108	0	0.390640832	antisense	2	54743185	54767834	
ENSG00000228109	5.417620896	5.271576845	antisense	3	196726331	196731615	MFI2-AS1
ENSG00000228110	1.16343121	0.390640832	pseudogene	1	210438984	210440587	ST13P19
ENSG00000228112	0	0.390640832	pseudogene	3	33768742	33769096	
ENSG00000228120	1.799000381	3.133298822	protein_coding	21	44579176	44582387	
ENSG00000228121	1.16343121	0.697730409	pseudogene	1	59465348	59466166	PHBP3
ENSG00000228124	0	0.390640832	antisense	6	90383188	90385502	
ENSG00000228125	0	0.390640832	pseudogene	X	67352710	67353278	
ENSG00000228126	1.16343121	1.802319292	lincRNA	1	150488233	150490508	
ENSG00000228133	1.407729925	0.950786998	antisense	17	1921177	1924000	
ENSG00000228135	0.869158192	1.353254395	antisense	2	219765533	219795358	
ENSG00000228137	1.16343121	1.16600992	antisense	21	47666804	47667596	
ENSG00000228140	2.928422289	1.802319292	processed_transc	1	15729475	15735929	
ENSG00000228141	0.869158192	0.697730409	lincRNA	15	83379223	83382778	
ENSG00000228146	2.238690726	2.652276565	protein_coding	16	3194244	3199964	
ENSG00000228149	0.869158192	0.950786998	pseudogene	21	37541268	37542478	RPL3P1

ENSG00000228150	1.16343121	1.353254395	processed_transc	1	10002981	10010032	
ENSG00000228157	0	0.390640832	protein_coding	17	18996287	18999674	
ENSG00000228158	3.652182994	3.183544561	pseudogene	X	64627964	64628565	TLE1P1
ENSG00000228168	0.499066092	0.390640832	pseudogene	3	39376470	39377430	HNRNPA1P21
ENSG00000228169	0	0.390640832	pseudogene	10	116449902	116450393	PPIAP19
ENSG00000228172	1.16343121	0.390640832	processed_transc	1	26143240	26146263	
ENSG00000228175	3.603959378	4.075511708	pseudogene	1	90459152	90459880	GEMIN8P4
ENSG00000228176	0.499066092	0	lincRNA	1	30181698	30182394	
ENSG00000228184	1.16343121	0	pseudogene	21	14897920	14900900	SNX19P1
ENSG00000228192	0.499066092	1.353254395	antisense	1	43307569	43312058	
ENSG00000228195	0	0.390640832	pseudogene	10	118509416	118510772	
ENSG00000228196	0	0.390640832	pseudogene	1	178715818	178716889	PTPN2P1
ENSG00000228201	1.16343121	2.144285137	antisense	16	698473	699200	
ENSG00000228203	2.928422289	2.144285137	processed_transc	2	7052408	7058813	
ENSG00000228205	3.393122761	3.935919592	pseudogene	3	183867492	183868223	
ENSG00000228208	0	1.518964905	protein_coding	1	218683438	218699320	C1orf143
ENSG00000228212	1.799000381	1.16600992	pseudogene	5	37209466	37212483	OFD1P17
ENSG00000228214	0	0.390640832	antisense	3	28616282	28799296	
ENSG00000228218	0.869158192	0.950786998	pseudogene	17	74221832	74222887	ATF4P3
ENSG00000228223	7.149084042	7.260948155	lincRNA	6	26522076	26526807	HCG11
ENSG00000228224	3.335220907	3.453019579	pseudogene	8	102374022	102384935	NACAP1
ENSG00000228232	4.962122459	4.250702764	pseudogene	X	39646386	39647390	GAPDHP1
ENSG00000228234	0	0.390640832	pseudogene	20	36231254	36231587	
ENSG00000228235	5.054730169	5.339632724	lincRNA	21	47472510	47473019	
ENSG00000228236	0	0.697730409	pseudogene	2	149925146	149925424	
ENSG00000228237	0.869158192	0.697730409	antisense	1	47139708	47157770	
ENSG00000228242	1.960915222	1.667587519	antisense	3	14186320	14189784	
ENSG00000228246	5.431503808	3.813624741	processed_transc	7	2757467	2762622	
ENSG00000228247	0.499066092	0.390640832	pseudogene	1	218023745	218023975	UBBP2
ENSG00000228251	1.616589159	1.16600992	lincRNA	2	113348373	113349516	
ENSG00000228252	0.499066092	1.667587519	pseudogene	3	129931666	129992649	COL6A4P2
ENSG00000228253	13.37689149	12.10218572	protein_coding	MT	8366	8572	
ENSG00000228261	1.16343121	1.353254395	lincRNA	10	106083127	106086762	
ENSG00000228262	0	0.390640832	lincRNA	2	34902646	34963298	
ENSG00000228264	1.16343121	1.16600992	pseudogene	1	154386845	154387613	
ENSG00000228265	3.698846687	3.32456471	lincRNA	20	32570858	32582163	
ENSG00000228271	2.10647801	2.242360793	lincRNA	3	193994471	194005157	
ENSG00000228274	4.464551814	4.693656882	antisense	22	39063835	39077825	
ENSG00000228275	0.499066092	0	antisense	X	100877973	100879154	ARMCX3-AS1
ENSG00000228283	1.407729925	0	pseudogene	6	147122809	147124960	
ENSG00000228285	1.16343121	0.390640832	pseudogene	6	33333325	33334020	LYPLA2P1
ENSG00000228286	0	0.390640832	pseudogene	11	71960900	71962332	
ENSG00000228288	3.335220907	4.17820932	antisense	1	202780082	202781284	
ENSG00000228292	0	0.697730409	pseudogene	9	37205109	37205365	
ENSG00000228294	3.077135474	4.547834947	lincRNA	14	19670480	19686002	
ENSG00000228300	5.741167503	6.155435249	protein_coding	19	1275438	1279227	C19orf24
ENSG00000228302	4.593635215	3.279072565	antisense	10	12287627	12289844	
ENSG00000228305	1.960915222	2.420525079	pseudogene	2	63849309	63849965	
ENSG00000228313	1.799000381	1.667587519	protein_coding	14	23064715	23067855	
ENSG00000228314	0.869158192	2.242360793	pseudogene	21	15215454	15220685	CYP4F29P
ENSG00000228315	7.524276744	7.786986696	pseudogene	22	23980674	24059543	GUSBP11
ENSG00000228316	0.499066092	0.390640832	pseudogene	X	102053324	102054488	
ENSG00000228317	0.499066092	0.390640832	antisense	9	108316316	108320310	
ENSG00000228318	0.499066092	0.950786998	antisense	21	42813321	42814669	
ENSG00000228322	0.499066092	0.697730409	processed_transc	9	4299390	4306046	
ENSG00000228323	2.762599152	1.667587519	antisense	19	54357835	54372361	
ENSG00000228327	2.671945279	3.279072565	processed_transc	1	700237	714006	
ENSG00000228328	1.16343121	1.802319292	pseudogene	X	67290362	67291609	
ENSG00000228330	0	0.697730409	pseudogene	10	13184185	13184977	
ENSG00000228331	1.16343121	0.390640832	pseudogene	17	19400256	19400842	
ENSG00000228335	1.407729925	1.353254395	pseudogene	7	99040513	99041119	
ENSG00000228341	0	0.390640832	pseudogene	7	107090528	107090807	
ENSG00000228342	0	1.353254395	lincRNA	6	41107594	41108570	

ENSG00000228343	5.848544806	5.593003837	antisense	X	48438162	48439545	
ENSG00000228344	3.603959378	3.935919592	protein_coding	18	5232875	5238332	
ENSG00000228347	0.499066092	0	pseudogene	1	104695928	104696452	FTLP17
ENSG00000228348	0.499066092	0	pseudogene	1	35587623	35588085	
ENSG00000228349	0	0.390640832	pseudogene	21	40863470	40863824	RPS26P4
ENSG00000228350	0.499066092	0.950786998	antisense	3	48701209	48706607	
ENSG00000228352	1.960915222	0.950786998	antisense	9	34084330	34096676	
ENSG00000228354	0	0.390640832	pseudogene	X	52684257	52686304	
ENSG00000228358	1.616589159	2.144285137	lincRNA	4	118281781	118294008	
ENSG00000228360	0.499066092	0.390640832	pseudogene	7	138912283	138912574	
ENSG00000228363	2.35979773	1.802319292	antisense	2	86789198	86845889	
ENSG00000228364	0	0.697730409	pseudogene	6	29044350	29044945	
ENSG00000228366	0.499066092	1.16600992	pseudogene	9	108472571	108473062	
ENSG00000228368	0.869158192	0.697730409	protein_coding	7	116605900	116607526	
ENSG00000228369	0.499066092	0.390640832	antisense	1	52516590	52518355	
ENSG00000228376	0	0.390640832	pseudogene	9	99838528	99840128	
ENSG00000228380	0	0.390640832	pseudogene	2	209134067	209134681	
ENSG00000228382	0	0.390640832	sense_intronic	1	226844341	226862768	ITPKB-IT1
ENSG00000228386	0.499066092	1.16600992	lincRNA	20	32602775	32605555	
ENSG00000228389	2.238690726	2.912743273	antisense	2	172629992	172632354	
ENSG00000228393	2.471521042	2.652276565	antisense	7	104590762	104653491	
ENSG00000228395	1.960915222	2.652276565	antisense	9	131291180	131314689	
ENSG00000228397	0.869158192	0.390640832	lincRNA	1	22350487	22351461	
ENSG00000228398	0	0.697730409	pseudogene	3	141583849	141584121	HMGN2P25
ENSG00000228399	1.16343121	1.667587519	pseudogene	1	101721830	101722172	
ENSG00000228401	0.499066092	0	antisense	9	139543062	139554873	
ENSG00000228404	0.869158192	0.390640832	antisense	21	47604993	47608855	
ENSG00000228407	1.16343121	0.950786998	pseudogene	1	52625942	52626274	
ENSG00000228409	5.60078458	5.726093467	pseudogene	7	65216129	65228341	CCT6P1
ENSG00000228412	0.499066092	1.802319292	processed_transc	6	19803602	19804973	
ENSG00000228413	1.616589159	0.950786998	pseudogene	3	195771566	195772125	
ENSG00000228414	0	0.390640832	processed_transc	2	61052267	61108449	
ENSG00000228415	1.16343121	2.144285137	pseudogene	6	30601409	30601675	PTMAP1
ENSG00000228417	0.499066092	0	antisense	10	111967608	111968349	
ENSG00000228420	0	0.697730409	lincRNA	1	110370977	110414058	
ENSG00000228422	0	0.390640832	lincRNA	20	11781111	11851363	C20orf61
ENSG00000228423	0	0.390640832	lincRNA	1	8865919	8867110	
ENSG00000228432	0.499066092	0	pseudogene	6	31334129	31334675	DHFRP2
ENSG00000228434	6.059927234	5.593003837	processed_transc	7	44043645	44047465	
ENSG00000228436	1.407729925	1.925536307	antisense	1	39325672	39385068	
ENSG00000228437	6.146643566	5.466810074	lincRNA	1	222139683	222158306	
ENSG00000228439	3.146112541	3.027231696	antisense	6	99968569	99979794	
ENSG00000228444	2.762599152	2.501982735	antisense	13	76203370	76206271	LMO7-AS1
ENSG00000228446	1.16343121	1.16600992	pseudogene	2	225364103	225364817	
ENSG00000228450	0	0.390640832	pseudogene	X	130626257	130626860	
ENSG00000228451	3.393122761	4.406904905	pseudogene	8	26236775	26240469	SDAD1P1
ENSG00000228452	1.960915222	0.950786998	antisense	1	43241484	43242461	
ENSG00000228456	0.499066092	0.950786998	pseudogene	9	45442004	45442713	
ENSG00000228459	0.869158192	0	protein_coding	X	3189861	3202694	CXorf28
ENSG00000228463	5.175139844	5.84793576	processed_transc	1	227615	267253	
ENSG00000228466	0	0.390640832	pseudogene	X	122695493	122696817	
ENSG00000228470	1.799000381	2.242360793	processed_transc	1	214517515	214530958	
ENSG00000228474	9.020358234	9.66813905	protein_coding	2	27293340	27294641	OST4
ENSG00000228477	2.10647801	2.242360793	pseudogene	1	40428352	40429076	
ENSG00000228481	0.869158192	0	antisense	2	45901840	45902615	
ENSG00000228484	0.869158192	1.16600992	lincRNA	10	116524547	116539662	
ENSG00000228485	0.499066092	1.16600992	processed_transc	10	120968043	120971272	
ENSG00000228486	3.146112541	4.274079748	processed_transc	2	98280680	98319519	
ENSG00000228487	0.869158192	0.390640832	antisense	9	129976314	129984186	
ENSG00000228488	0	0.950786998	lincRNA	2	101338683	101339344	
ENSG00000228492	6.220467583	6.3217081	pseudogene	X	73422669	73424477	RAB11FIP1P1
ENSG00000228499	1.16343121	1.667587519	pseudogene	2	236791006	236791137	TMSB10P1
ENSG00000228501	0.869158192	1.16600992	pseudogene	13	100802771	100803346	RPL15P18

ENSG00000228502	4.19837882	4.528525526	pseudogene	1	96912486	96913874	EEF1A1P11
ENSG00000228506	4.381167248	4.794098771	antisense	6	99872798	99879249	
ENSG00000228507	0.499066092	1.16600992	pseudogene	2	172347932	172348441	MRPS29P2
ENSG00000228508	0.869158192	0.390640832	processed_transc	10	24536208	24540350	
ENSG00000228509	2.671945279	3.608232228	antisense	2	191541670	191573442	
ENSG00000228510	2.35979773	0.697730409	pseudogene	9	131207280	131208907	
ENSG00000228513	0	0.697730409	pseudogene	2	203926037	203927126	
ENSG00000228519	0.869158192	0.390640832	pseudogene	X	25663839	25664444	
ENSG00000228521	0.499066092	0	lincRNA	7	154995857	154996899	
ENSG00000228522	0.869158192	0.697730409	pseudogene	9	67285298	67298632	
ENSG00000228525	0	1.353254395	pseudogene	1	227145770	227145968	
ENSG00000228526	0.499066092	1.16600992	lincRNA	1	9228327	9262678	
ENSG00000228532	5.175139844	5.6022688	protein_coding	X	114952650	114953669	
ENSG00000228536	0	0.950786998	lincRNA	1	219583023	219585283	
ENSG00000228540	0.869158192	0.390640832	sense_intronic	7	110364215	110365881	IMMP2L-IT1
ENSG00000228541	0	0.697730409	lincRNA	2	62690262	62691205	
ENSG00000228543	0	0.390640832	lincRNA	X	9217960	9243246	
ENSG00000228544	3.603959378	5.072639111	antisense	9	139698379	139703300	
ENSG00000228546	3.335220907	2.971122874	pseudogene	7	101977727	101979559	
ENSG00000228547	0.499066092	0	pseudogene	10	15041102	15042077	
ENSG00000228549	0.869158192	1.16600992	antisense	1	17197440	17200587	
ENSG00000228554	2.35979773	1.518964905	antisense	7	39739940	39742895	
ENSG00000228568	0	0.390640832	pseudogene	2	72515914	72516239	
ENSG00000228569	0	0.697730409	lincRNA	7	156225217	156228527	
ENSG00000228589	0.499066092	0.950786998	pseudogene	1	28421582	28422933	
ENSG00000228594	5.879259127	6.281901708	protein_coding	1	1533392	1535476	
ENSG00000228596	0.499066092	0	antisense	7	44824649	44826939	
ENSG00000228599	0.869158192	0.950786998	pseudogene	22	40502329	40503070	
ENSG00000228601	0	0.390640832	pseudogene	20	55034484	55034639	RPL39P
ENSG00000228604	0.869158192	0.697730409	lincRNA	20	21129382	21143531	
ENSG00000228606	2.10647801	2.501982735	antisense	1	160231534	160232568	
ENSG00000228609	5.018397273	4.547834947	lincRNA	X	100258821	100262962	
ENSG00000228612	0	0.697730409	pseudogene	X	79827370	79830106	HK2P1
ENSG00000228614	0	0.390640832	antisense	6	53655673	53659072	
ENSG00000228622	0	0.390640832	lincRNA	22	33501369	33502045	
ENSG00000228623	7.242608348	7.225413142	lincRNA	9	115759495	115774507	ZNF883
ENSG00000228624	1.960915222	3.411459265	processed_transc	6	114290865	114792869	
ENSG00000228626	0.499066092	0	pseudogene	1	147760107	147761057	
ENSG00000228630	1.16343121	3.411459265	antisense	12	54356092	54368740	HOTAIR
ENSG00000228634	1.616589159	1.667587519	lincRNA	1	32398621	32399576	
ENSG00000228638	4.642199401	4.101879561	pseudogene	3	48332283	48332865	
ENSG00000228643	0	0.697730409	processed_transc	2	286419	301515	
ENSG00000228649	7.740446424	6.651226688	processed_transc	7	22895705	22900458	
ENSG00000228653	2.57521082	2.242360793	pseudogene	7	63961203	63962107	
ENSG00000228657	0	0.390640832	pseudogene	10	104332514	104332982	
ENSG00000228660	0	0.390640832	pseudogene	7	63423809	63424501	VN1R35P
ENSG00000228661	1.16343121	1.667587519	antisense	11	3875548	3876739	
ENSG00000228663	0.869158192	0.950786998	pseudogene	20	49530268	49530948	PSMD10P1
ENSG00000228665	0.499066092	1.353254395	pseudogene	1	109572688	109572941	
ENSG00000228666	0	0.390640832	pseudogene	6	28936907	28938237	KRT18P1
ENSG00000228667	0.869158192	0	pseudogene	10	25156574	25157119	
ENSG00000228668	0.499066092	0.390640832	TR_V_pseudogen	7	38384631	38385100	TRGV5P
ENSG00000228671	0.499066092	1.667587519	lincRNA	10	131581117	131584811	
ENSG00000228672	6.548110424	6.264499759	protein_coding	5	138727635	138730885	C5orf65
ENSG00000228673	0.499066092	0.390640832	sense_intronic	10	74461207	74463097	
ENSG00000228677	0.499066092	0	antisense	21	38559967	38566227	TTC3-AS1
ENSG00000228678	1.407729925	0.390640832	lincRNA	9	33500946	33502751	
ENSG00000228679	0.499066092	0.390640832	lincRNA	6	84693447	84717042	
ENSG00000228681	0.499066092	0	pseudogene	2	225159647	225160470	
ENSG00000228686	0.869158192	0.390640832	antisense	1	175986413	175987896	
ENSG00000228692	0.499066092	0	lincRNA	6	164007615	164009197	
ENSG00000228695	0.499066092	0	pseudogene	16	55794460	55828070	CES1P1
ENSG00000228696	1.407729925	0.697730409	protein_coding	17	44376501	44439163	ARL17B

ENSG00000228701	1.16343121	1.667587519	antisense	10	93542596	93558048	
ENSG00000228702	0	0.390640832	pseudogene	10	46221142	46221486	
ENSG00000228705	1.960915222	0.950786998	antisense	20	61405480	61406764	
ENSG00000228707	2.238690726	3.748356452	lincRNA	9	118774640	118775244	
ENSG00000228708	0	2.039052734	processed_transc	21	19849676	19858917	
ENSG00000228709	5.637181576	5.66550919	processed_transc	21	45905460	45910171	
ENSG00000228716	8.810621286	8.965221116	protein_coding	5	79922047	79950802	DHFR
ENSG00000228717	0.499066092	0.390640832	pseudogene	X	150394701	150396187	
ENSG00000228718	0.869158192	0	lincRNA	6	2637170	2640235	
ENSG00000228719	1.799000381	1.16600992	lincRNA	22	36841445	36850991	
ENSG00000228723	0.499066092	0	antisense	3	9234177	9236137	SRGAP3-AS2
ENSG00000228726	1.16343121	0.697730409	antisense	10	75855924	75862185	
ENSG00000228727	5.072559152	5.516660875	protein_coding	6	31730576	31732628	SAPCD1
ENSG00000228728	0	1.16600992	pseudogene	X	149987226	149987719	
ENSG00000228737	0	0.950786998	antisense	5	140997981	141006048	
ENSG00000228741	2.10647801	2.144285137	processed_transc	13	24553949	24608145	
ENSG00000228742	5.175139844	5.09905904	lincRNA	7	106415457	106436010	
ENSG00000228747	0.499066092	0	antisense	7	87858974	87861089	
ENSG00000228748	1.16343121	0.950786998	antisense	10	79542624	79551665	
ENSG00000228754	0	0.390640832	pseudogene	10	49953590	49954173	
ENSG00000228759	0	0.390640832	pseudogene	10	93525656	93526953	
ENSG00000228763	0	0.697730409	antisense	2	109293251	109295057	
ENSG00000228767	0.869158192	1.16600992	pseudogene	3	35257197	35258641	KRT8P18
ENSG00000228768	1.616589159	1.16600992	protein_coding	17	29898161	29902678	
ENSG00000228772	0	0.390640832	lincRNA	6	22589366	22594062	
ENSG00000228775	1.407729925	2.912743273	processed_transc	7	141404138	141438146	
ENSG00000228779	2.10647801	1.802319292	antisense	16	175736	177218	
ENSG00000228781	0.499066092	0	pseudogene	9	80795130	80795609	
ENSG00000228782	2.671945279	3.493416095	protein_coding	17	45527593	45569849	
ENSG00000228784	0.869158192	1.518964905	processed_transc	2	20068621	20084808	
ENSG00000228791	0.869158192	0.697730409	lincRNA	3	24535578	24541502	
ENSG00000228793	0.499066092	0.950786998	lincRNA	6	3594481	3624981	
ENSG00000228794	7.058051099	7.20731193	processed_transc	1	762988	794826	
ENSG00000228797	0.869158192	0.390640832	pseudogene	13	19271143	19271647	FAM207BP
ENSG00000228800	0	0.950786998	lincRNA	10	30842783	30843412	
ENSG00000228801	4.999881834	4.917990497	antisense	8	52811885	52859733	
ENSG00000228802	0	0.697730409	antisense	2	224830418	224832423	
ENSG00000228803	0	0.390640832	pseudogene	7	22787294	22788423	
ENSG00000228804	0.869158192	0	protein_coding	3	187420101	187451637	
ENSG00000228807	0	0.390640832	pseudogene	22	44879375	44879597	
ENSG00000228808	0.869158192	0.390640832	pseudogene	13	100024427	100024940	HMGB3P4
ENSG00000228809	1.16343121	0.697730409	antisense	20	16695489	16711593	
ENSG00000228812	1.407729925	1.353254395	antisense	20	60928066	60931536	
ENSG00000228816	3.077135474	3.493416095	pseudogene	10	33233409	33233927	AK3P5
ENSG00000228817	0.499066092	0.697730409	sense_intronic	21	30744821	30745886	BACH1-IT2
ENSG00000228818	2.57521082	2.501982735	pseudogene	1	240305970	240308689	
ENSG00000228819	0	0.697730409	pseudogene	X	131264119	131265380	
ENSG00000228820	0	0.390640832	pseudogene	20	49747325	49748204	RPSAP1
ENSG00000228824	0	0.390640832	processed_transc	13	88096247	88323514	MIR4500HG
ENSG00000228826	0.869158192	0	lincRNA	1	121236177	121252295	
ENSG00000228828	1.407729925	2.501982735	pseudogene	10	38107534	38109910	
ENSG00000228829	0	0.390640832	pseudogene	7	75803051	75806523	
ENSG00000228832	0.869158192	1.16600992	antisense	10	33608520	33609590	
ENSG00000228838	0	1.16600992	antisense	1	53753696	53755378	
ENSG00000228839	0.869158192	2.039052734	antisense	22	31688485	31734007	
ENSG00000228844	0	0.390640832	pseudogene	1	240799899	240800352	
ENSG00000228847	0.499066092	1.667587519	pseudogene	X	41958007	41958426	ATP5G2P4
ENSG00000228852	0	1.16600992	antisense	1	95708781	95729610	
ENSG00000228857	2.238690726	2.971122874	lincRNA	2	114588626	114600933	
ENSG00000228865	0.499066092	0	antisense	10	48357926	48358600	
ENSG00000228866	0.869158192	1.518964905	antisense	9	139932430	139933342	
ENSG00000228868	0.499066092	0	pseudogene	3	75377700	75388222	MYLKP1
ENSG00000228872	0	0.390640832	pseudogene	2	63944256	63945033	

ENSG00000228873	0.869158192	0.390640832	lincRNA		2	96811341	96812751	
ENSG00000228878	1.960915222	2.579085888	processed_transc		7	35794574	35840226	
ENSG00000228879	0.499066092	0.390640832	pseudogene		1	246918813	246919134	
ENSG00000228882	0.499066092	0	pseudogene		10	38741915	38744293	CICP9
ENSG00000228885	0	0.697730409	antisense	X		102753723	102754210	
ENSG00000228886	0.869158192	0	lincRNA		13	45924458	45925485	
ENSG00000228887	1.16343121	1.518964905	pseudogene		19	14181210	14182052	
ENSG00000228889	4.322773689	3.781359661	processed_transc		13	99848696	99852964	UBAC2-AS1
ENSG00000228897	1.16343121	1.353254395	pseudogene		7	51454060	51454798	
ENSG00000228903	3.502389126	3.453019579	pseudogene		7	44066550	44081491	
ENSG00000228906	1.616589159	1.667587519	lincRNA	X		73168808	73169393	
ENSG00000228909	0.499066092	0	lincRNA		2	220768772	220769610	
ENSG00000228915	1.616589159	0.950786998	pseudogene		11	71604456	71605479	
ENSG00000228919	0	1.802319292	protein_coding		4	7940728	7942023	
ENSG00000228925	3.448790144	3.232099092	processed_transc		2	47126414	47135817	
ENSG00000228929	4.097495944	4.319727133	pseudogene		1	53237866	53238320	
ENSG00000228930	0.499066092	0	pseudogene		21	46796106	46796297	MTCO1P3
ENSG00000228932	0.499066092	0	processed_transc		9	132891870	132895515	
ENSG00000228933	2.238690726	2.501982735	processed_transc	X		27193037	27417114	
ENSG00000228952	3.077135474	1.802319292	lincRNA		3	187166633	187167238	
ENSG00000228955	1.616589159	0.950786998	pseudogene		1	246795558	246795780	
ENSG00000228958	0.499066092	0	pseudogene		20	32552968	32553382	
ENSG00000228960	0.499066092	0	pseudogene		7	143991573	143998027	OR2A9P
ENSG00000228962	0	0.950786998	antisense		6	32358287	32361463	HCG23
ENSG00000228972	0	0.390640832	sense_intronic		6	128752086	128753913	
ENSG00000228974	0	0.697730409	pseudogene		7	5600374	5600686	
ENSG00000228976	0	0.390640832	pseudogene		6	108939203	108939488	SUMO2P8
ENSG00000228979	0.869158192	0.950786998	pseudogene		17	61548965	61549456	
ENSG00000228981	2.762599152	2.788380093	pseudogene		4	144270428	144271270	
ENSG00000228986	0	0.390640832	pseudogene	X		154521460	154522731	
ENSG00000228989	4.292665995	4.566889334	antisense		2	242629829	242633704	
ENSG00000228991	5.525114348	3.64455972	pseudogene		9	66693277	66721073	
ENSG00000228992	0.869158192	0	pseudogene		15	28140850	28141728	
ENSG00000228995	0.869158192	0.950786998	pseudogene		20	55932482	55933425	MTND1P9
ENSG00000228997	1.616589159	1.925536307	pseudogene		10	10818186	10818624	
ENSG00000228998	6.023726639	6.091080508	protein_coding		15	90818266	90820841	
ENSG00000229000	0	0.390640832	pseudogene		19	54275760	54276534	SEPT7P8
ENSG00000229001	0	0.390640832	pseudogene		10	70782534	70783622	
ENSG00000229002	0.869158192	0	pseudogene		1	148542873	148545128	
ENSG00000229007	2.847891871	2.912743273	pseudogene		21	33869122	33869621	EXOSC3P1
ENSG00000229010	0.499066092	0.950786998	pseudogene		1	23466818	23467635	
ENSG00000229016	0.499066092	0.390640832	pseudogene		1	218474604	218475314	
ENSG00000229017	0	1.925536307	antisense		6	143287561	143358719	
ENSG00000229018	1.407729925	1.16600992	pseudogene		7	72476064	72555749	
ENSG00000229020	3.004694206	2.144285137	pseudogene		1	113466045	113466966	AKR7A2P1
ENSG00000229021	0.869158192	1.353254395	processed_transc		1	151967007	152015250	
ENSG00000229022	0.869158192	1.16600992	pseudogene		1	241995237	241996089	
ENSG00000229023	0	0.390640832	pseudogene		2	191857365	191858293	
ENSG00000229029	0	0.697730409	pseudogene		9	14593575	14594181	
ENSG00000229033	1.616589159	1.802319292	processed_transc	X		122867024	122868144	
ENSG00000229036	2.238690726	2.242360793	antisense		6	143824531	143827475	
ENSG00000229037	0.499066092	0.390640832	pseudogene	X		53834274	53835116	
ENSG00000229043	6.386460534	6.392840898	antisense		7	1199662	1204903	
ENSG00000229046	0	0.697730409	pseudogene		21	33078868	33079177	HMGN1P2
ENSG00000229052	0	0.390640832	pseudogene		1	93396902	93397643	
ENSG00000229054	1.407729925	0.390640832	pseudogene		7	33075824	33075994	
ENSG00000229056	3.393122761	2.721932731	processed_transc		2	197124748	197128928	
ENSG00000229065	1.407729925	0.697730409	antisense		9	97875990	97876743	
ENSG00000229068	0	0.390640832	pseudogene		6	30434229	30435771	TMPOP1
ENSG00000229070	2.671945279	2.851901313	protein_coding		17	75440786	75443249	
ENSG00000229072	0	0.390640832	pseudogene		3	40740557	40740829	HMGN2P24
ENSG00000229081	1.799000381	1.925536307	antisense		10	134334331	134335953	
ENSG00000229082	0.869158192	0.950786998	pseudogene	X		130475421	130476426	

ENSG00000229083	0.499066092	0.390640832	pseudogene	X	12843959	12844952	PSMA6P2
ENSG00000229091	1.616589159	1.353254395	pseudogene	7	10490938	10492153	
ENSG00000229097	3.211941663	2.971122874	pseudogene	10	71923441	71923880	CALM2P2
ENSG00000229105	0.499066092	0.697730409	antisense	9	119266562	119324572	
ENSG00000229106	0	0.390640832	pseudogene	1	24227961	24229227	
ENSG00000229107	0	0.390640832	pseudogene	22	21022124	21025272	FAM108A5P
ENSG00000229109	0	0.390640832	antisense	9	85834246	85849157	
ENSG00000229111	0.869158192	0	antisense	13	48651273	48654127	MED4-AS1
ENSG00000229112	0.499066092	0	pseudogene	1	246691864	246692143	
ENSG00000229115	0	0.950786998	pseudogene	9	126521499	126522054	
ENSG00000229117	10.42847557	11.05785962	protein_coding	12	56510370	56511727	RPL41
ENSG00000229118	1.616589159	1.667587519	lincRNA	2	112022860	112037457	
ENSG00000229119	1.616589159	2.144285137	pseudogene	5	165809310	165809604	
ENSG00000229122	1.960915222	1.802319292	sense_intronic	2	27283906	27284683	AGBL5-IT1
ENSG00000229124	3.950976309	4.127774132	antisense	10	17256238	17268897	
ENSG00000229127	3.077135474	3.570966319	antisense	2	210895457	210929080	
ENSG00000229132	3.502389126	3.64455972	pseudogene	X	91368244	91369460	EIF4A1P10
ENSG00000229133	0.869158192	1.16600992	pseudogene	1	68708157	68708738	RPS7P4
ENSG00000229137	0.869158192	1.353254395	lincRNA	13	21861545	21868997	
ENSG00000229145	0.499066092	0.697730409	pseudogene	X	46147451	46148563	ACTBP1
ENSG00000229152	5.158541613	4.990346085	sense_intronic	13	111547406	111551519	ANKRD10-IT1
ENSG00000229153	0.499066092	0.950786998	processed_transc	7	143104906	143220542	
ENSG00000229154	0.869158192	0	antisense	6	73844526	73853237	KCNQ5-AS1
ENSG00000229160	0	0.950786998	lincRNA	2	38359779	38366088	
ENSG00000229161	1.616589159	1.353254395	pseudogene	7	42834505	42842093	TCP1P1
ENSG00000229162	1.407729925	1.518964905	antisense	1	25287836	25289588	
ENSG00000229165	0.499066092	0	pseudogene	7	48942152	48943487	GDI2P1
ENSG00000229167	1.960915222	0.950786998	antisense	1	32037186	32041174	
ENSG00000229168	0	0.697730409	pseudogene	X	44508375	44508965	RPL19P20
ENSG00000229178	3.146112541	3.96493948	processed_transc	3	195382436	195384798	
ENSG00000229180	6.856698814	6.877938671	lincRNA	7	65993425	66007536	
ENSG00000229186	4.381167248	4.508954154	pseudogene	12	112337067	112339195	ADAM1
ENSG00000229190	0.869158192	0.950786998	antisense	10	17737708	17742231	
ENSG00000229191	0.869158192	1.925536307	processed_transc	1	200993077	200997920	
ENSG00000229203	2.471521042	1.925536307	lincRNA	2	132587374	132589604	
ENSG00000229204	0.869158192	0.697730409	pseudogene	4	170712371	170712853	
ENSG00000229205	0	0.390640832	non_coding	10	1205708	1210612	LINC00200
ENSG00000229207	1.407729925	1.353254395	pseudogene	9	34318405	34319653	
ENSG00000229211	0.499066092	0.950786998	pseudogene	8	31077410	31078500	
ENSG00000229212	3.652182994	3.679994897	pseudogene	15	85747141	85778026	
ENSG00000229214	1.616589159	1.925536307	processed_transc	6	170188886	170198921	LINC00242
ENSG00000229217	0.499066092	0	pseudogene	3	10099657	10099971	
ENSG00000229222	1.799000381	2.579085888	pseudogene	20	48573282	48574569	KRT18P4
ENSG00000229227	1.616589159	1.667587519	antisense	10	46937463	46951699	
ENSG00000229228	0.499066092	0.390640832	antisense	1	231727038	231747836	
ENSG00000229230	0	0.697730409	pseudogene	20	33805812	33805988	
ENSG00000229233	1.16343121	0	lincRNA	7	12693804	12694610	
ENSG00000229235	0	0.390640832	pseudogene	10	34952787	34953029	
ENSG00000229237	0.499066092	0	pseudogene	X	154045374	154045676	HMGN1P26
ENSG00000229241	0	0.697730409	pseudogene	3	4023665	4025979	
ENSG00000229251	0.499066092	0	pseudogene	7	84612863	84613825	HNRNPA1P8
ENSG00000229256	0.499066092	0.390640832	pseudogene	10	104975466	104976557	ST13P13
ENSG00000229259	0	0.390640832	pseudogene	1	32888815	32892390	
ENSG00000229261	1.407729925	1.925536307	processed_transc	10	70975089	70992246	
ENSG00000229267	0	0.390640832	processed_transc	2	215825116	215826786	
ENSG00000229268	0.499066092	0	pseudogene	9	13986174	13987908	
ENSG00000229272	0.869158192	0	antisense	10	120763048	120763396	
ENSG00000229273	2.238690726	1.802319292	pseudogene	9	41954580	41955037	
ENSG00000229276	0.499066092	0	sense_intronic	6	111681844	111682810	REV3L-IT1
ENSG00000229278	0.499066092	1.802319292	antisense	10	101411746	101413662	
ENSG00000229282	0.499066092	0	lincRNA	6	3056124	3056995	
ENSG00000229286	0	0.697730409	pseudogene	22	16076052	16076172	
ENSG00000229291	0	0.950786998	lincRNA	1	236121179	236135125	

ENSG00000229292	0.499066092	0	protein_coding	19	56280507	56284545	
ENSG00000229298	1.16343121	2.039052734	pseudogene	9	21811620	21812346	
ENSG00000229304	0.499066092	0.697730409	sense_intronic	10	121672907	121673914	
ENSG00000229314	0.869158192	0	protein_coding	9	117085336	117088755	ORM1
ENSG00000229320	5.995967334	6.445267191	pseudogene	3	160283235	160287036	KRT8P12
ENSG00000229323	0.499066092	0	lincRNA	13	51095069	51101585	
ENSG00000229324	0.869158192	0.697730409	pseudogene	X	70236381	70236755	
ENSG00000229325	2.762599152	1.353254395	sense_intronic	3	195001452	195003470	ACAP2-IT1
ENSG00000229326	1.16343121	0.390640832	pseudogene	2	120456199	120457733	
ENSG00000229330	1.16343121	0.390640832	sense_intronic	17	64672475	64673522	
ENSG00000229334	3.830412367	3.714580548	antisense	3	194353652	194366130	
ENSG00000229336	0.869158192	0.390640832	pseudogene	21	21112023	21112464	
ENSG00000229339	0	0.390640832	pseudogene	3	42092673	42093186	
ENSG00000229344	7.254372086	6.919299326	pseudogene	1	568137	568818	
ENSG00000229348	1.960915222	1.667587519	antisense	1	43919598	43922666	HYI-AS1
ENSG00000229349	1.616589159	0.950786998	pseudogene	6	46172466	46175123	ACTG1P9
ENSG00000229356	0.499066092	0.390640832	antisense	21	45870869	45875167	LRRC3-AS1
ENSG00000229358	5.207774174	4.888000647	pseudogene	7	32660019	32801399	
ENSG00000229367	0	0.390640832	pseudogene	1	229706279	229706543	HMGN2P19
ENSG00000229368	1.407729925	1.518964905	processed_transc	11	3875842	3876629	
ENSG00000229372	1.16343121	1.353254395	antisense	1	43913447	43914315	SZT2-AS1
ENSG00000229373	0.869158192	1.353254395	lincRNA	13	114598076	114626485	LINC00452
ENSG00000229376	0.499066092	0.697730409	pseudogene	1	657465	660287	CICP3
ENSG00000229380	0.869158192	1.353254395	antisense	7	601595	605256	
ENSG00000229382	1.407729925	0	lincRNA	21	46756622	46758580	
ENSG00000229388	1.799000381	1.518964905	lincRNA	1	28969740	28975093	
ENSG00000229390	0	0.390640832	pseudogene	6	29938578	29940241	MICD
ENSG00000229391	0	0.390640832	pseudogene	6	32520490	32527799	HLA-DRB6
ENSG00000229393	0.499066092	0.950786998	antisense	1	2424876	2425918	
ENSG00000229401	1.616589159	1.353254395	lincRNA	6	10434549	10457014	
ENSG00000229413	4.19837882	3.368666104	pseudogene	7	128293740	128293989	
ENSG00000229414	0.869158192	0.390640832	processed_transc	11	2861365	2882798	KCNQ1-AS1
ENSG00000229415	2.471521042	0.390640832	protein_coding	14	36942493	36983034	SFTA3
ENSG00000229417	0.869158192	0.697730409	pseudogene	10	97949122	97949975	
ENSG00000229419	1.960915222	1.802319292	pseudogene	9	108282409	108288640	RALGAPA1P
ENSG00000229422	0.499066092	0.697730409	pseudogene	9	66493572	66494196	
ENSG00000229431	3.004694206	3.714580548	antisense	1	43850784	43854826	
ENSG00000229435	0	0.950786998	pseudogene	7	159024125	159026067	PIP5K1P2
ENSG00000229444	0	0.390640832	antisense	1	44175063	44193014	
ENSG00000229447	1.616589159	1.353254395	pseudogene	1	31736331	31736529	
ENSG00000229450	1.799000381	3.027231696	protein_coding	4	120299287	120326749	
ENSG00000229455	0	1.518964905	pseudogene	10	126178392	126179147	RPS10P18
ENSG00000229456	0.499066092	0.390640832	pseudogene	13	41192875	41195485	RLIMP1
ENSG00000229463	0.499066092	0	sense_intronic	1	236002783	236003482	LYST-IT2
ENSG00000229468	0	0.390640832	pseudogene	3	20053944	20054098	
ENSG00000229472	3.603959378	3.279072565	antisense	20	32668955	32669754	
ENSG00000229473	0	0.390640832	pseudogene	13	41566915	41567467	RGS17P1
ENSG00000229474	2.35979773	5.018301917	protein_coding	15	44957930	45003514	PATL2
ENSG00000229481	5.375152132	4.622587519	lincRNA	19	38320191	38322797	
ENSG00000229485	0	0.390640832	pseudogene	10	42644758	42644997	
ENSG00000229487	1.616589159	2.039052734	antisense	X	110949877	110954329	ALG13-AS1
ENSG00000229489	0	0.390640832	pseudogene	9	43399987	43401310	ATP5A1P5
ENSG00000229492	0.869158192	1.16600992	pseudogene	22	17897744	17898875	
ENSG00000229497	0	0.390640832	pseudogene	7	43806889	43807198	
ENSG00000229498	0.869158192	0.697730409	antisense	2	86042253	86052514	
ENSG00000229507	1.16343121	1.353254395	sense_intronic	17	57707431	57709331	CLTC-IT1
ENSG00000229512	3.274897671	3.027231696	sense_intronic	11	1784239	1784979	
ENSG00000229515	0	0.390640832	pseudogene	3	46183850	46185169	
ENSG00000229519	1.16343121	1.667587519	pseudogene	1	6607966	6608683	
ENSG00000229521	0.499066092	0	antisense	13	77654646	77655325	MYCBP2-AS2
ENSG00000229523	0	0.390640832	pseudogene	9	45729709	45730417	
ENSG00000229525	0.869158192	1.16600992	processed_transc	2	220253218	220268355	
ENSG00000229530	0.869158192	0.390640832	pseudogene	9	95101940	95103945	

ENSG00000229534	0	0.390640832	pseudogene	11	5592477	5593422	
ENSG00000229539	2.238690726	2.971122874	antisense	20	3868886	3869515	
ENSG00000229543	0	0.390640832	lincRNA	10	80053599	80060760	
ENSG00000229544	0.499066092	0.697730409	protein_coding	10	126135592	126138753	NKX1-2
ENSG00000229558	0.499066092	1.353254395	lincRNA	13	23993110	24002818	SACS-AS1
ENSG00000229563	0	0.390640832	processed_transc	X	45364633	45489447	
ENSG00000229567	0	0.950786998	pseudogene	1	93744518	93744686	
ENSG00000229585	0.499066092	0.390640832	pseudogene	4	54852751	54853225	RPL21P44
ENSG00000229587	2.35979773	1.925536307	sense_intronic	9	77624744	77630953	
ENSG00000229589	2.471521042	2.912743273	processed_transc	3	38492518	38496311	
ENSG00000229590	0	0.390640832	pseudogene	17	56234809	56234926	MSX2P1
ENSG00000229591	1.799000381	1.16600992	antisense	7	151817086	151818802	
ENSG00000229595	0	0.390640832	pseudogene	1	231253577	231254071	
ENSG00000229598	1.799000381	1.353254395	pseudogene	22	39118748	39119510	PRDX3P1
ENSG00000229601	0.499066092	0.390640832	pseudogene	X	70633684	70633936	
ENSG00000229604	1.16343121	0.697730409	pseudogene	2	88124461	88124664	MT-ATP8
ENSG00000229605	0.499066092	0	pseudogene	10	29187925	29188422	
ENSG00000229608	0.499066092	1.518964905	pseudogene	22	43180468	43183106	GOLGA2P4
ENSG00000229621	0	0.390640832	lincRNA	2	21910306	21933495	
ENSG00000229622	0	0.697730409	pseudogene	13	96344792	96346226	MTND5P2
ENSG00000229623	0.499066092	0.390640832	pseudogene	21	40607312	40607946	METTL21AP1
ENSG00000229626	0.499066092	0	pseudogene	7	107449179	107450013	
ENSG00000229628	0.499066092	0	lincRNA	7	46030504	46040497	
ENSG00000229635	0	0.390640832	pseudogene	1	93850044	93850555	
ENSG00000229638	3.78787645	3.608232228	pseudogene	3	185135283	185136566	
ENSG00000229644	8.009315251	7.980592361	protein_coding	10	36810649	36813162	NAMPTL
ENSG00000229645	1.616589159	0	lincRNA	14	95873606	95876427	LINC00341
ENSG00000229647	2.10647801	1.518964905	lincRNA	2	208104374	208110611	
ENSG00000229656	4.322773689	5.840125386	lincRNA	10	33247773	33371030	
ENSG00000229659	5.191549282	6.287656023	pseudogene	10	75182017	75182454	
ENSG00000229660	0.499066092	0	lincRNA	7	157074230	157075380	
ENSG00000229666	2.10647801	2.039052734	antisense	5	66297211	66299781	MAST4-AS1
ENSG00000229670	0	0.390640832	pseudogene	2	210043690	210045847	PKP4P1
ENSG00000229672	1.799000381	0.390640832	antisense	10	3793259	3805418	
ENSG00000229677	1.960915222	1.518964905	pseudogene	7	138734202	138734752	
ENSG00000229688	0.499066092	0	processed_transc	7	16250113	16310229	
ENSG00000229689	4.464551814	4.42790041	protein_coding	2	96194603	96198143	
ENSG00000229691	1.799000381	1.518964905	antisense	1	155279829	155280381	
ENSG00000229692	1.960915222	1.925536307	sense_intronic	2	39220117	39220998	SOS1-IT1
ENSG00000229694	0.499066092	0	lincRNA	9	93881344	93945044	
ENSG00000229696	0	0.697730409	pseudogene	13	42499734	42501485	KARSP1
ENSG00000229697	0	0.950786998	lincRNA	9	45034957	45047145	
ENSG00000229700	0	0.390640832	pseudogene	6	20042686	20043171	
ENSG00000229702	0	0.390640832	processed_transc	X	129611043	129658231	
ENSG00000229704	0.869158192	0.697730409	pseudogene	3	184963248	184964229	EIF2S2P2
ENSG00000229715	0.869158192	0	pseudogene	13	32420978	32527609	EEF1DP3
ENSG00000229716	0.499066092	0	pseudogene	1	8891173	8891431	
ENSG00000229719	1.407729925	1.16600992	lincRNA	11	64658898	64660921	
ENSG00000229721	0.869158192	0.950786998	pseudogene	5	179653378	179654000	
ENSG00000229728	3.335220907	3.279072565	antisense	20	1342266	1343229	
ENSG00000229729	4.962122459	5.137803333	protein_coding	3	88108424	88188123	
ENSG00000229742	0.869158192	0	pseudogene	1	224485348	224486302	
ENSG00000229750	0	0.390640832	antisense	2	176032632	176033250	
ENSG00000229751	0	0.390640832	antisense	10	14033815	14049525	
ENSG00000229758	0.869158192	0	pseudogene	2	128957475	128957767	DYNLT3P2
ENSG00000229759	1.16343121	0.950786998	pseudogene	3	48297840	48298428	MRPS18AP1
ENSG00000229763	1.16343121	1.16600992	antisense	10	11495945	11497386	
ENSG00000229765	0.869158192	0.390640832	pseudogene	6	10214083	10214595	
ENSG00000229766	1.616589159	0	processed_transc	20	8000549	8008603	
ENSG00000229780	0.869158192	2.144285137	antisense	1	154526085	154527493	UBE2Q1-AS1
ENSG00000229782	0.499066092	1.353254395	antisense	17	4400689	4402969	
ENSG00000229786	0.869158192	0.950786998	pseudogene	1	26213851	26214101	SNRPFP2
ENSG00000229789	2.671945279	2.912743273	pseudogene	9	68726629	68745997	

ENSG00000229793	3.274897671	3.453019579	antisense	10	103570752	103571371	
ENSG00000229794	0	0.950786998	pseudogene	X	102061669	102062755	
ENSG00000229795	0.499066092	0	pseudogene	1	235596300	235596539	
ENSG00000229800	0.499066092	0	pseudogene	13	33051411	33052978	ATP8A2P2
ENSG00000229801	0.499066092	2.144285137	lincRNA	1	149590752	149591762	
ENSG00000229806	1.960915222	2.501982735	pseudogene	3	54660242	54660673	RPS15P5
ENSG00000229808	3.274897671	2.501982735	pseudogene	1	161860623	161861986	
ENSG00000229809	6.710639711	6.948652068	protein_coding	16	30580667	30584055	ZNF688
ENSG00000229813	1.16343121	0.697730409	lincRNA	1	2357419	2362089	
ENSG00000229816	1.960915222	2.652276565	pseudogene	2	32426669	32429068	DDX50P1
ENSG00000229820	0	0.697730409	pseudogene	1	28780031	28780444	
ENSG00000229821	0.869158192	0	antisense	1	201191241	201192251	
ENSG00000229827	0.499066092	0.697730409	pseudogene	2	170433881	170434256	
ENSG00000229828	0.499066092	0.697730409	pseudogene	1	146011091	146025308	
ENSG00000229832	0	0.697730409	antisense	1	212530760	212531695	
ENSG00000229833	7.04456449	8.0397549	protein_coding	19	7694671	7696509	C19orf79
ENSG00000229835	0.869158192	1.353254395	pseudogene	9	21695175	21696948	KHSRPP1
ENSG00000229839	0	1.16600992	antisense	2	62296582	62374016	
ENSG00000229841	2.35979773	3.32456471	antisense	1	217665317	217667625	
ENSG00000229848	1.407729925	0.950786998	antisense	17	79481073	79494802	
ENSG00000229850	0	0.950786998	sense_intronic	9	127431666	127432491	
ENSG00000229852	4.381167248	4.17820932	processed_transc	6	73972938	74011124	
ENSG00000229853	1.960915222	1.353254395	pseudogene	20	46259010	46260182	
ENSG00000229862	0	0.697730409	antisense	6	74233341	74280319	
ENSG00000229863	0	0.390640832	pseudogene	3	57168170	57168751	
ENSG00000229867	2.238690726	2.242360793	antisense	2	120001998	120006647	STEAP3-AS1
ENSG00000229869	1.799000381	1.518964905	antisense	10	978966	988683	
ENSG00000229870	0.869158192	0	pseudogene	10	51023142	51023608	
ENSG00000229871	0	0.390640832	pseudogene	1	56673239	56674128	
ENSG00000229873	2.671945279	3.027231696	antisense	20	61431979	61436939	
ENSG00000229874	1.407729925	2.144285137	antisense	1	206135293	206137828	
ENSG00000229878	0	0.390640832	pseudogene	10	33324397	33324592	
ENSG00000229880	0	0.390640832	pseudogene	21	46095785	46098003	IMMTP1
ENSG00000229886	0.499066092	0	pseudogene	7	65490113	65496531	
ENSG00000229887	0.499066092	0.390640832	pseudogene	1	58513847	58514963	HNRNPA1P6
ENSG00000229891	5.848544806	6.2290532	lincRNA	22	42760406	42765242	
ENSG00000229893	0	0.390640832	antisense	7	27773014	27780014	
ENSG00000229894	0	0.950786998	pseudogene	4	166198944	166201175	GK3P
ENSG00000229895	0.869158192	1.353254395	antisense	1	116916755	116917283	
ENSG00000229896	1.616589159	1.353254395	processed_transc	6	12007903	12009089	
ENSG00000229897	0.499066092	0.950786998	pseudogene	9	101369275	101370411	SEPT7P7
ENSG00000229901	0	1.667587519	antisense	1	41840676	41841341	
ENSG00000229905	0	0.950786998	lincRNA	1	696291	697369	
ENSG00000229910	0.499066092	0	pseudogene	13	68875737	68877434	HNRNPA1P18
ENSG00000229912	0.869158192	1.518964905	lincRNA	3	197177198	197183525	
ENSG00000229914	0.499066092	0.390640832	pseudogene	1	158165423	158165921	
ENSG00000229915	2.238690726	0.390640832	antisense	2	239335718	239336370	
ENSG00000229917	0	0.390640832	pseudogene	16	11120881	11121618	
ENSG00000229918	0.499066092	0.697730409	antisense	13	99484338	99486883	DOCK9-AS1
ENSG00000229920	0.499066092	0.697730409	pseudogene	2	63869589	63871172	
ENSG00000229921	3.004694206	0.697730409	lincRNA	6	168393502	168397757	KIF25-AS1
ENSG00000229926	1.16343121	0.950786998	pseudogene	9	141031547	141038316	
ENSG00000229927	1.799000381	1.518964905	pseudogene	10	46914088	46914795	
ENSG00000229931	3.989024711	3.845183986	antisense	6	16761369	16762883	
ENSG00000229932	1.407729925	0.950786998	pseudogene	10	23425852	23426590	YWHAZP3
ENSG00000229939	3.393122761	4.17820932	pseudogene	X	110862036	110862322	
ENSG00000229941	0	0.390640832	antisense	2	178563157	178588017	
ENSG00000229944	1.407729925	1.925536307	pseudogene	17	47500970	47502298	
ENSG00000229947	0.499066092	0.390640832	pseudogene	17	79480089	79480210	
ENSG00000229948	0	0.390640832	pseudogene	2	69824485	69826081	
ENSG00000229950	2.671945279	3.368666104	antisense	6	10409573	10416679	
ENSG00000229951	2.57521082	2.652276565	processed_transc	2	28607276	28617539	
ENSG00000229953	4.800350936	5.47691899	antisense	1	156616299	156631216	

ENSG00000229954	1.16343121	0.697730409	pseudogene	X	102041741	102043315	MTND2P2
ENSG00000229955	0.499066092	0.390640832	processed_transc	22	38783923	38784862	
ENSG00000229956	0.499066092	0.697730409	processed_transc	1	71547007	71733913	ZRANB2-AS2
ENSG00000229964	2.10647801	1.518964905	lincRNA	3	193809350	193826963	
ENSG00000229965	0	0.390640832	pseudogene	1	226626265	226627045	
ENSG00000229970	3.830412367	2.501982735	processed_transc	7	8301894	8384146	
ENSG00000229972	0	0.390640832	protein_coding	3	51851620	51864876	IQCF3
ENSG00000229980	1.616589159	1.16600992	processed_transc	17	48944040	48987098	
ENSG00000229983	0.499066092	0.390640832	lincRNA	1	212341549	212363601	
ENSG00000229986	0	0.390640832	lincRNA	21	40218171	40220568	
ENSG00000229989	0.499066092	0.697730409	processed_transc	1	198776622	198906558	
ENSG00000229990	0.869158192	0.390640832	antisense	10	75601591	75606370	
ENSG00000229991	0.869158192	0.697730409	pseudogene	1	224762136	224763091	AKR1B1P1
ENSG00000229992	0.869158192	1.16600992	pseudogene	1	93112605	93113187	HMGB3P9
ENSG00000229994	0.499066092	0.950786998	pseudogene	1	35816323	35817208	
ENSG00000229996	1.16343121	0	antisense	2	241949462	241950161	
ENSG00000229999	1.16343121	0.390640832	antisense	22	40768668	40784294	
ENSG00000230001	0.499066092	0.390640832	sense_intronic	9	4070906	4071884	
ENSG00000230002	2.762599152	2.579085888	sense_intronic	2	73684230	73686609	ALMS1-IT1
ENSG00000230005	0.499066092	0.697730409	sense_intronic	1	227931532	227934892	SNAP47-IT1
ENSG00000230006	1.407729925	1.667587519	processed_transc	2	89065324	89106126	ANKRD36BP2
ENSG00000230010	0.499066092	0.697730409	lincRNA	20	18303196	18339944	
ENSG00000230013	0.499066092	0.390640832	lincRNA	9	107753077	107754062	
ENSG00000230015	0	0.390640832	lincRNA	1	240902729	240906911	
ENSG00000230021	0.869158192	1.353254395	processed_transc	1	536816	659930	
ENSG00000230022	0.869158192	0.390640832	pseudogene	13	22104829	22105462	FNTAP2
ENSG00000230024	0	0.950786998	lincRNA	1	219608494	219615984	
ENSG00000230026	0.499066092	0.390640832	pseudogene	1	235524468	235525575	
ENSG00000230037	1.16343121	2.242360793	pseudogene	2	137087011	137087483	UBBP1
ENSG00000230042	1.799000381	1.16600992	pseudogene	7	23168797	23169460	AK3P3
ENSG00000230046	0.499066092	1.16600992	antisense	2	32602699	32604667	
ENSG00000230047	0.499066092	0	pseudogene	2	232301812	232302132	
ENSG00000230051	0	0.697730409	lincRNA	22	27727748	27775226	
ENSG00000230052	0	0.390640832	pseudogene	7	63566032	63566373	MTND3P2
ENSG00000230054	1.16343121	1.16600992	lincRNA	9	117418584	117424654	
ENSG00000230055	8.528449672	8.38930337	protein_coding	17	36886510	36891858	CISD3
ENSG00000230061	0.499066092	2.242360793	processed_transc	21	45834473	45845155	
ENSG00000230067	1.407729925	0.390640832	pseudogene	3	36808608	36810290	HSPD1P6
ENSG00000230068	0	1.667587519	sense_intronic	1	22385690	22390692	CDC42-IT1
ENSG00000230069	3.211941663	2.851901313	pseudogene	4	103648431	103651878	
ENSG00000230071	3.146112541	3.493416095	pseudogene	22	40982710	40984007	
ENSG00000230074	3.744048221	3.87606762	antisense	9	34665662	34681295	
ENSG00000230076	0.499066092	1.925536307	pseudogene	2	215711852	215712169	
ENSG00000230077	1.799000381	0.697730409	pseudogene	3	189686819	189687650	MTAPP2
ENSG00000230081	0	0.390640832	pseudogene	9	115043258	115043692	
ENSG00000230082	3.830412367	3.570966319	antisense	3	9989088	9996471	PRRT3-AS1
ENSG00000230084	1.799000381	1.802319292	antisense	3	42643455	42695880	
ENSG00000230090	0	0.390640832	antisense	2	5758459	5831250	
ENSG00000230091	2.57521082	3.368666104	processed_transc	10	81806616	81838668	
ENSG00000230092	0.499066092	1.518964905	lincRNA	1	736259	745541	
ENSG00000230097	0	0.390640832	pseudogene	9	29824756	29826709	
ENSG00000230098	0.499066092	0	antisense	10	132893481	132894040	
ENSG00000230102	0.869158192	0.950786998	processed_transc	3	193723245	193788759	
ENSG00000230105	0	0.697730409	lincRNA	X	56644824	56650891	
ENSG00000230107	2.35979773	3.493416095	lincRNA	22	42834029	42842201	
ENSG00000230109	1.799000381	2.851901313	lincRNA	10	21629162	21661879	
ENSG00000230110	0	0.697730409	pseudogene	7	101804400	101804617	
ENSG00000230124	4.689181911	5.259915406	antisense	1	180238788	180245359	
ENSG00000230125	0	0.697730409	pseudogene	10	30590752	30591001	EEF1A1P39
ENSG00000230131	1.16343121	1.518964905	lincRNA	10	125115966	125277224	
ENSG00000230133	0	0.390640832	lincRNA	20	24180403	24205224	
ENSG00000230140	0.499066092	0.390640832	antisense	2	101589110	101593623	
ENSG00000230146	1.616589159	0.390640832	pseudogene	X	72989083	72990258	SEPHS1P4

ENSG00000230147	0.499066092	0	pseudogene	9	93931863	93932327	
ENSG00000230149	2.57521082	2.851901313	processed_transc	22	39130735	39134995	
ENSG00000230154	0	0.390640832	pseudogene	2	11448151	11449067	
ENSG00000230155	1.16343121	0.950786998	antisense	20	34064028	34078808	
ENSG00000230162	0	0.950786998	pseudogene	X	135024637	135029298	
ENSG00000230163	3.871730003	3.935919592	antisense	1	35316295	35317156	
ENSG00000230166	0	0.390640832	pseudogene	10	51117659	51117988	
ENSG00000230170	0.499066092	0	pseudogene	X	118354745	118355705	HNRNPA1P28
ENSG00000230174	0.499066092	0	lincRNA	6	31409444	31414750	
ENSG00000230175	0	0.697730409	pseudogene	1	165640493	165640962	
ENSG00000230176	2.238690726	1.925536307	antisense	20	4173714	4176375	
ENSG00000230177	2.471521042	2.788380093	antisense	6	111599135	111599945	
ENSG00000230183	0.869158192	1.16600992	pseudogene	15	56297911	56299374	
ENSG00000230184	0	0.697730409	sense_intronic	1	246484965	246485740	SMYD3-IT1
ENSG00000230185	2.10647801	1.667587519	protein_coding	9	115246722	115249484	C9orf147
ENSG00000230186	1.16343121	0.697730409	antisense	1	149400063	149429580	
ENSG00000230188	0.499066092	1.16600992	pseudogene	9	37490418	37490890	
ENSG00000230189	4.19837882	3.32456471	pseudogene	7	65874130	65955046	
ENSG00000230191	1.799000381	0.697730409	pseudogene	7	56230838	56231226	
ENSG00000230194	0	0.390640832	pseudogene	22	19225966	19226262	
ENSG00000230196	0	1.16600992	pseudogene	7	81240200	81240642	
ENSG00000230200	1.407729925	1.802319292	processed_transc	9	138356344	138356726	
ENSG00000230201	0.869158192	1.353254395	pseudogene	17	4760052	4760669	ATP6V0CP1
ENSG00000230202	5.899377978	6.733635362	pseudogene	6	118320138	118320611	
ENSG00000230204	1.16343121	1.353254395	pseudogene	6	50880425	50880969	FTH1P5
ENSG00000230207	2.671945279	2.579085888	pseudogene	9	7477045	7478320	
ENSG00000230212	0.869158192	0.697730409	processed_transc	21	37441940	37498938	
ENSG00000230216	0.869158192	1.518964905	pseudogene	X	49090415	49090960	HSPB1P2
ENSG00000230221	0	0.390640832	pseudogene	9	98055086	98057088	
ENSG00000230224	3.652182994	4.15321211	pseudogene	10	102008028	102008852	PHBP9
ENSG00000230243	0	0.390640832	pseudogene	13	47079253	47079571	FKBP1AP3
ENSG00000230251	0	0.390640832	pseudogene	2	155143102	155143938	PHBP4
ENSG00000230252	0	0.390640832	pseudogene	7	63570925	63571928	MTND2P4
ENSG00000230253	0.499066092	0.390640832	pseudogene	9	68410687	68412235	
ENSG00000230255	0	0.697730409	pseudogene	1	142791488	142792556	
ENSG00000230257	7.427149666	6.862599506	processed_transc	7	102613969	102629303	
ENSG00000230262	4.618121645	4.250702764	lincRNA	9	96938884	96966818	MIRLET7DHG
ENSG00000230266	0	0.390640832	antisense	3	194868600	194873519	
ENSG00000230267	0	1.16600992	pseudogene	16	32163040	32199434	HERC2P4
ENSG00000230272	0	1.518964905	antisense	1	51731525	51732716	
ENSG00000230274	0.499066092	0	pseudogene	3	40364206	40364770	PGAM1P3
ENSG00000230280	0.869158192	2.334191469	pseudogene	1	202880940	202881859	
ENSG00000230285	0	0.950786998	lincRNA	1	86064814	86066417	
ENSG00000230288	0.499066092	0.390640832	lincRNA	15	45671395	45694525	
ENSG00000230289	1.616589159	0.950786998	antisense	9	134391945	134397616	
ENSG00000230290	0	0.390640832	antisense	6	109244179	109245306	
ENSG00000230291	1.799000381	2.579085888	pseudogene	12	80496679	80497113	
ENSG00000230295	2.57521082	2.039052734	pseudogene	7	66345695	66347968	
ENSG00000230299	0	0.390640832	pseudogene	2	85489267	85489390	
ENSG00000230300	0.499066092	0	sense_intronic	13	33732724	33738546	STARD13-IT1
ENSG00000230303	0	0.390640832	lincRNA	9	88400637	88408395	
ENSG00000230305	0	0.697730409	pseudogene	7	76153832	76162009	
ENSG00000230306	2.847891871	2.334191469	pseudogene	10	135158147	135158410	
ENSG00000230309	0	0.950786998	lincRNA	6	79314184	79315753	
ENSG00000230310	0	1.16600992	antisense	19	12663411	12664458	
ENSG00000230311	0	0.390640832	pseudogene	X	72442960	72443397	
ENSG00000230318	0.499066092	0.390640832	pseudogene	1	220487324	220489045	
ENSG00000230319	0.499066092	0.950786998	antisense	22	43434591	43448372	
ENSG00000230321	1.960915222	1.802319292	pseudogene	19	12615719	12616790	
ENSG00000230325	0.869158192	1.16600992	processed_transc	1	236703394	236713580	
ENSG00000230326	0	0.390640832	pseudogene	X	9833133	9833583	HMGN1P33
ENSG00000230330	0	0.390640832	pseudogene	16	26043860	26044132	HMGN2P3
ENSG00000230337	2.10647801	3.081239798	antisense	1	11159732	11162157	

ENSG00000230338	0	0.390640832	pseudogene	10	96533913	96534390	MTND4P19
ENSG00000230345	0	0.390640832	lincRNA	22	48256420	48257811	
ENSG00000230355	0.499066092	0.390640832	pseudogene	2	67108219	67108354	
ENSG00000230358	0.869158192	1.16600992	pseudogene	7	66744765	66751606	
ENSG00000230359	1.616589159	2.144285137	pseudogene	7	128696073	128696824	TPI1P2
ENSG00000230360	2.471521042	2.039052734	pseudogene	9	84701070	84703683	
ENSG00000230362	0.499066092	1.16600992	pseudogene	3	175695285	175696358	
ENSG00000230364	1.799000381	1.802319292	pseudogene	1	171652268	171653578	
ENSG00000230366	1.407729925	2.144285137	processed_transc	21	38580804	38594041	DSCR9
ENSG00000230368	1.16343121	2.039052734	lincRNA	1	803451	812283	FAM41C
ENSG00000230370	0	0.697730409	pseudogene	7	11046026	11046503	
ENSG00000230373	3.211941663	2.851901313	pseudogene	15	85051116	85060045	GOLGA6L5
ENSG00000230381	0.499066092	0.697730409	processed_transc	1	116971673	117105789	
ENSG00000230383	1.407729925	0.697730409	pseudogene	7	137406731	137407592	
ENSG00000230392	0.499066092	0	lincRNA	X	118425492	118469573	
ENSG00000230394	0	0.390640832	lincRNA	X	7975419	7986201	
ENSG00000230396	1.16343121	0.390640832	sense_intronic	9	127885377	127886583	
ENSG00000230397	0	1.353254395	pseudogene	10	31649884	31650144	SPTLC1P1
ENSG00000230399	0	0.390640832	pseudogene	X	117766338	117768181	RBBP8P1
ENSG00000230400	0	0.390640832	lincRNA	20	22656710	22665982	
ENSG00000230402	0	0.697730409	lincRNA	1	101092605	101094500	
ENSG00000230406	0	0.390640832	pseudogene	2	222457161	222457581	
ENSG00000230408	0.869158192	0.390640832	antisense	2	201645218	201676893	
ENSG00000230409	1.16343121	1.353254395	pseudogene	3	37317087	37318089	TCEA1P2
ENSG00000230415	0	1.16600992	processed_transc	1	1210603	1215800	
ENSG00000230417	0	0.390640832	processed_transc	10	80008497	80434724	
ENSG00000230424	0.499066092	0.950786998	antisense	1	19536995	19567198	
ENSG00000230426	0.499066092	0	lincRNA	1	187610358	187612992	ERVMER61-1
ENSG00000230427	0	0.390640832	antisense	1	95527152	95533101	
ENSG00000230428	0	0.390640832	pseudogene	16	501404	503461	
ENSG00000230432	0	0.390640832	antisense	2	220163724	220168852	
ENSG00000230433	0.869158192	0.950786998	antisense	6	524171	525581	
ENSG00000230438	2.10647801	1.16600992	lincRNA	6	2854891	2876744	
ENSG00000230439	4.517578978	3.232099092	processed_transc	1	94218480	94241000	
ENSG00000230444	1.16343121	0.697730409	pseudogene	7	1654226	1654961	TFAMP1
ENSG00000230445	3.335220907	3.748356452	pseudogene	10	27535279	27540722	
ENSG00000230446	0.499066092	0	pseudogene	1	142790233	142790520	
ENSG00000230447	0	0.697730409	pseudogene	16	30915669	30921529	CTF2P
ENSG00000230449	0.499066092	1.518964905	pseudogene	6	30664583	30665312	RPL7P4
ENSG00000230450	0.869158192	1.16600992	pseudogene	2	131935191	131936519	NEK2P4
ENSG00000230453	3.950976309	3.64455972	protein_coding	9	33524392	33573007	ANKRD18B
ENSG00000230454	2.35979773	2.652276565	processed_transc	3	50297735	50300790	
ENSG00000230457	3.502389126	3.232099092	pseudogene	3	156527340	156528521	PA2G4P4
ENSG00000230479	3.211941663	4.585695337	antisense	21	37802658	37853368	
ENSG00000230480	0	0.697730409	pseudogene	3	28489216	28489564	
ENSG00000230482	0	0.390640832	pseudogene	2	198127835	198128259	ATP5G2P3
ENSG00000230487	6.658471572	6.192713747	processed_transc	7	1609709	1629262	
ENSG00000230494	0.499066092	0.390640832	sense_intronic	9	112202342	112203532	
ENSG00000230495	0.499066092	0	pseudogene	20	17579900	17580452	
ENSG00000230499	2.35979773	2.039052734	lincRNA	2	111953540	111964071	
ENSG00000230508	1.16343121	1.667587519	pseudogene	X	150161974	150162564	RPL19P21
ENSG00000230510	1.960915222	2.912743273	protein_coding	19	47021936	47104457	
ENSG00000230513	4.734682531	4.826083323	antisense	22	21356175	21364631	THAP7-AS1
ENSG00000230519	0.499066092	0.390640832	pseudogene	12	27855271	27855839	
ENSG00000230521	2.471521042	2.912743273	pseudogene	6	29855071	29856045	HCG4P7
ENSG00000230523	0	0.390640832	processed_transc	1	29656132	29676626	
ENSG00000230524	2.35979773	2.039052734	pseudogene	3	15193340	15247466	COL6A4P1
ENSG00000230526	0	0.950786998	lincRNA	10	73638114	73638865	
ENSG00000230528	0	0.950786998	processed_transc	17	20340137	20350557	
ENSG00000230530	1.16343121	1.16600992	processed_transc	3	45720535	45730626	
ENSG00000230534	0.869158192	1.353254395	antisense	10	35386934	35415948	
ENSG00000230536	0.499066092	0.950786998	antisense	9	130853248	130856736	
ENSG00000230537	1.616589159	1.667587519	lincRNA	9	93867239	93869586	

ENSG00000230539	1.16343121	0	sense_intronic	7	36637440	36639726	AOAH-IT1
ENSG00000230542	0.499066092	0.390640832	lincRNA	X	2531029	2533388	LINC00102
ENSG00000230547	0	0.390640832	pseudogene	1	171240093	171240729	HMGB1P11
ENSG00000230550	0.869158192	0	antisense	1	204110536	204112137	
ENSG00000230551	7.19863491	6.889336956	processed_transc	5	148872949	148884233	
ENSG00000230554	0	0.390640832	sense_intronic	6	45227825	45228305	
ENSG00000230555	1.960915222	2.912743273	lincRNA	10	43916047	43917254	
ENSG00000230558	0.869158192	0.390640832	pseudogene	19	43057636	43065909	CEACAMP2
ENSG00000230561	1.16343121	1.353254395	lincRNA	5	127039082	127277326	
ENSG00000230565	2.238690726	1.802319292	antisense	10	44141390	44143467	ZNF32-AS2
ENSG00000230568	0.869158192	1.667587519	pseudogene	20	3470176	3471652	SF3A3P1
ENSG00000230569	0.869158192	0.390640832	lincRNA	2	139228226	139231502	
ENSG00000230573	0	0.390640832	lincRNA	10	3876142	3876920	
ENSG00000230580	1.16343121	1.16600992	pseudogene	2	219218798	219219295	
ENSG00000230581	2.10647801	2.579085888	pseudogene	9	6834386	6835577	
ENSG00000230583	0.869158192	0.390640832	pseudogene	7	66274980	66309813	
ENSG00000230584	4.437288965	3.532712221	pseudogene	13	79483054	79484677	CCT5P2
ENSG00000230585	0.869158192	0.697730409	pseudogene	1	51246011	51246818	PHBP12
ENSG00000230590	5.431503808	5.466810074	processed_transc	X	73467699	73513362	FTX
ENSG00000230592	1.16343121	1.353254395	pseudogene	X	99410881	99411772	RPSAP8
ENSG00000230601	0.869158192	2.144285137	lincRNA	9	117428716	117434029	
ENSG00000230604	0.499066092	0	pseudogene	1	52325449	52325804	
ENSG00000230606	6.687686437	7.228408053	processed_transc	2	98081683	98091049	
ENSG00000230609	0	0.390640832	pseudogene	10	74320022	74320214	
ENSG00000230612	1.799000381	1.353254395	antisense	5	132024373	132057275	
ENSG00000230613	1.407729925	1.353254395	antisense	20	30155510	30161066	HM13-AS1
ENSG00000230615	6.535310252	7.148445524	processed_transc	1	44509600	44570647	
ENSG00000230620	1.16343121	0.697730409	antisense	10	71968555	71969331	
ENSG00000230623	0	0.390640832	lincRNA	1	200443048	200447421	
ENSG00000230625	0.869158192	0.697730409	sense_intronic	13	20651665	20653696	ZMYM2-IT1
ENSG00000230626	3.502389126	3.279072565	pseudogene	7	128766572	128770847	
ENSG00000230629	2.238690726	2.334191469	pseudogene	X	70182814	70183140	RPS23P8
ENSG00000230634	0.499066092	0	pseudogene	22	46877226	46877657	
ENSG00000230635	0.499066092	0	pseudogene	9	44019596	44020591	CYP4F60P
ENSG00000230637	1.16343121	0.950786998	pseudogene	22	25723219	25735942	
ENSG00000230638	3.077135474	1.925536307	pseudogene	1	42007739	42010258	
ENSG00000230641	0.869158192	0	antisense	13	27746396	27757135	USP12-AS2
ENSG00000230647	2.35979773	2.420525079	lincRNA	17	14277456	14324343	
ENSG00000230648	0	0.390640832	antisense	6	4019077	4021449	
ENSG00000230650	4.981125677	4.274079748	pseudogene	2	111118185	111121314	
ENSG00000230655	0.499066092	0	pseudogene	3	75419599	75420493	
ENSG00000230657	0.869158192	0.390640832	protein_coding	12	11460017	11463369	PRB4
ENSG00000230658	3.502389126	3.133298822	processed_transc	7	23140847	23145322	KLHL7-AS1
ENSG00000230659	0	1.353254395	pseudogene	1	165788590	165789024	
ENSG00000230665	0	0.697730409	pseudogene	X	73616704	73619385	PRKRIRP1
ENSG00000230666	4.261916566	2.334191469	pseudogene	19	45041045	45124119	CEACAM22P
ENSG00000230667	0	1.16600992	pseudogene	1	92540077	92540968	SETP18
ENSG00000230668	0	0.390640832	antisense	X	139763271	139769870	
ENSG00000230673	3.502389126	3.081239798	pseudogene	X	73802923	73803381	PABPC1P3
ENSG00000230677	0	0.390640832	antisense	10	65035906	65041994	
ENSG00000230679	0.869158192	0.950786998	antisense	1	8938894	8939953	ENO1-AS1
ENSG00000230683	0.499066092	0.697730409	pseudogene	3	38556939	38557321	
ENSG00000230684	0.499066092	0	antisense	9	132695815	132698820	
ENSG00000230693	1.16343121	0.697730409	pseudogene	X	72744797	72745991	
ENSG00000230695	0	0.697730409	antisense	2	216300976	216301715	
ENSG00000230696	0.499066092	0	processed_transc	2	110352270	110363275	
ENSG00000230697	0.499066092	0.697730409	pseudogene	3	20049344	20049739	
ENSG00000230699	3.502389126	3.64455972	antisense	1	846815	850328	
ENSG00000230701	2.671945279	2.652276565	pseudogene	22	23605323	23606541	FBXW4P1
ENSG00000230702	0	0.697730409	pseudogene	2	62083830	62084190	
ENSG00000230709	0.499066092	0	processed_transc	17	16879313	16884667	
ENSG00000230715	6.228441838	5.824376633	pseudogene	7	128292895	128293719	
ENSG00000230716	1.16343121	1.518964905	pseudogene	11	119473587	119475018	KRT8P7

ENSG00000230721	1.407729925	2.420525079	pseudogene	1	87250596	87251150	
ENSG00000230724	5.018397273	4.761389014	processed_transc	11	127115	139117	
ENSG00000230732	1.407729925	0.950786998	antisense	3	196640757	196641313	
ENSG00000230733	8.541310587	7.896480033	antisense	7	5515435	5519442	
ENSG00000230734	8.504022011	9.096409785	pseudogene	9	119942907	119943374	RPL10P3
ENSG00000230736	3.146112541	2.721932731	processed_transc	22	32772651	32780330	
ENSG00000230739	0.869158192	0.697730409	pseudogene	1	162794585	162819257	
ENSG00000230746	0.499066092	0	lincRNA	7	145372975	145378462	
ENSG00000230750	0.499066092	0.697730409	pseudogene	20	10366427	10368454	SDAD1P2
ENSG00000230751	1.616589159	0.390640832	sense_intronic	7	30180486	30181033	
ENSG00000230753	0.499066092	0.697730409	antisense	20	32375179	32398903	
ENSG00000230756	0	0.697730409	pseudogene	2	130970443	130971063	RHOQP3
ENSG00000230758	0.869158192	0	pseudogene	20	47654894	47655139	SNAP23P
ENSG00000230768	0	0.390640832	lincRNA	1	106132316	106161557	
ENSG00000230769	0.869158192	0.950786998	pseudogene	22	41184781	41185271	
ENSG00000230772	2.35979773	2.721932731	pseudogene	20	25714900	25715729	VN1R108P
ENSG00000230777	0	1.518964905	pseudogene	1	175891111	175891281	
ENSG00000230778	0.499066092	0	protein_coding	15	40573645	40574787	ANKRD63
ENSG00000230782	1.16343121	1.16600992	processed_transc	9	109737114	109865269	
ENSG00000230783	0.499066092	0	pseudogene	2	160545728	160546802	
ENSG00000230786	1.16343121	2.039052734	protein_coding	10	135380204	135383462	
ENSG00000230787	0	0.697730409	pseudogene	1	79520630	79521740	
ENSG00000230789	1.16343121	0.697730409	sense_intronic	5	142572065	142573731	ARHGAP26-IT1
ENSG00000230790	0	0.697730409	antisense	2	11881123	11885427	
ENSG00000230793	0.499066092	0.390640832	pseudogene	13	45168994	45170218	SMARCE1P5
ENSG00000230795	1.616589159	1.16600992	pseudogene	6	29894236	29897009	HLA-K
ENSG00000230797	4.77879189	5.361622823	protein_coding	X	21874105	21876845	YY2
ENSG00000230798	2.238690726	3.935919592	antisense	1	63786555	63790112	
ENSG00000230799	1.960915222	1.518964905	pseudogene	2	202627460	202628296	
ENSG00000230801	0.869158192	0.697730409	pseudogene	20	30396306	30396552	
ENSG00000230812	0.869158192	1.802319292	lincRNA	1	59486059	59510286	
ENSG00000230813	0	0.390640832	pseudogene	1	246690332	246691457	
ENSG00000230815	0.499066092	0.390640832	sense_intronic	9	97586554	97587055	
ENSG00000230817	0.499066092	0.390640832	lincRNA	1	83368866	83632498	
ENSG00000230823	1.407729925	1.16600992	pseudogene	X	62519126	62519552	CBX1P1
ENSG00000230825	0.869158192	0.390640832	processed_transc	7	7294785	7317410	
ENSG00000230826	0.499066092	0.390640832	antisense	9	126498716	126514220	
ENSG00000230829	0.499066092	0	pseudogene	5	32222038	32222453	
ENSG00000230832	0.869158192	1.16600992	pseudogene	1	146553887	146555127	
ENSG00000230834	1.16343121	0.390640832	lincRNA	11	1798160	1799618	
ENSG00000230836	2.847891871	4.528525526	lincRNA	2	75167385	75169797	
ENSG00000230837	1.16343121	0.950786998	pseudogene	20	32339973	32340349	RPL31P2
ENSG00000230839	0.499066092	1.16600992	lincRNA	20	1784662	1798252	
ENSG00000230844	5.806547963	6.50081754	processed_transc	X	46404928	46407671	
ENSG00000230849	3.871730003	4.342020395	pseudogene	1	173110239	173111489	GOT2P2
ENSG00000230850	2.35979773	1.925536307	processed_transc	1	143119061	143233417	
ENSG00000230860	0	0.390640832	pseudogene	20	44196629	44197446	CCNB1IP1P2
ENSG00000230869	0	0.390640832	pseudogene	10	46174140	46195980	CTGLF10P
ENSG00000230870	1.616589159	0.950786998	pseudogene	21	32999440	33000913	FBXW11P1
ENSG00000230872	2.10647801	2.039052734	pseudogene	16	22393757	22418749	
ENSG00000230873	0	0.697730409	protein_coding	6	17102489	17131603	
ENSG00000230876	0	0.390640832	lincRNA	2	33050510	33151760	LINC00486
ENSG00000230880	1.799000381	2.788380093	processed_transc	1	142618766	142729975	
ENSG00000230881	0	0.390640832	antisense	1	42001114	42001539	
ENSG00000230882	5.485736881	3.714580548	pseudogene	7	75700787	75704281	
ENSG00000230894	0.499066092	0	antisense	9	119330728	119348607	
ENSG00000230896	3.004694206	2.579085888	processed_transc	1	46160356	46162747	
ENSG00000230897	1.616589159	1.16600992	pseudogene	17	14608393	14608851	
ENSG00000230899	1.407729925	2.144285137	antisense	X	149007636	149009870	
ENSG00000230905	0.869158192	1.16600992	sense_intronic	13	33066436	33072629	N4BP2L2-IT1
ENSG00000230911	0.499066092	1.16600992	pseudogene	11	111900582	111901091	PPIHP1
ENSG00000230912	1.799000381	1.16600992	antisense	22	39062513	39064755	
ENSG00000230913	2.847891871	3.027231696	pseudogene	6	41634644	41635387	

ENSG00000230914	0	0.390640832	lincRNA	7	2423756	2428232	
ENSG00000230916	1.407729925	1.518964905	pseudogene	X	125605688	125606437	
ENSG00000230918	0.499066092	0	antisense	2	162929766	162931679	
ENSG00000230925	0.499066092	0.390640832	antisense	9	33909913	33911120	
ENSG00000230928	0.499066092	0	antisense	10	100011780	100016332	
ENSG00000230929	0.869158192	0.390640832	pseudogene	5	125963711	125964011	
ENSG00000230936	0.499066092	0	pseudogene	7	55410009	55412651	
ENSG00000230937	7.743231983	0	processed_transc	1	209602165	209606183	MIR205HG
ENSG00000230939	0	1.16600992	lincRNA	6	8784411	8785678	
ENSG00000230943	0.869158192	1.518964905	lincRNA	6	113944737	113971276	
ENSG00000230946	1.616589159	1.353254395	pseudogene	1	101406568	101407550	
ENSG00000230947	0	0.950786998	sense_intronic	22	24994021	24995365	
ENSG00000230950	0.869158192	0.390640832	pseudogene	2	117782653	117783694	MTND2P21
ENSG00000230952	0.499066092	0	lincRNA	2	11497641	11505762	
ENSG00000230953	1.16343121	1.16600992	pseudogene	1	53386077	53386269	
ENSG00000230956	0.499066092	0	pseudogene	X	87351397	87352226	
ENSG00000230958	0	0.390640832	pseudogene	2	112623375	112623982	
ENSG00000230964	0	0.390640832	pseudogene	2	91766537	91766632	
ENSG00000230970	0.499066092	0	antisense	3	42744145	42746120	HHATL-AS1
ENSG00000230978	0	0.390640832	lincRNA	21	36096105	36109478	LINC00160
ENSG00000230979	3.004694206	2.721932731	pseudogene	2	47917855	47918385	
ENSG00000230980	1.16343121	0.697730409	pseudogene	11	1813783	1814100	
ENSG00000230982	2.471521042	2.652276565	pseudogene	21	48073470	48073934	DSTNP1
ENSG00000230989	9.470442173	9.901814226	protein_coding	16	83841448	83853342	HSBP1
ENSG00000230992	1.616589159	1.518964905	pseudogene	2	133110240	133112412	FAM201B
[truncated: 696,728 more chars]
